# Attributable deaths and disability-adjusted life-years caused by infections with antibiotic-resistant bacteria in the EU and the European Economic Area in 2015: a population-level modelling analysis

**DOI:** 10.1016/S1473-3099(18)30605-4

**Published:** 2019-01

**Authors:** Alessandro Cassini, Liselotte Diaz Högberg, Diamantis Plachouras, Annalisa Quattrocchi, Ana Hoxha, Gunnar Skov Simonsen, Mélanie Colomb-Cotinat, Mirjam E Kretzschmar, Brecht Devleesschauwer, Michele Cecchini, Driss Ait Ouakrim, Tiago Cravo Oliveira, Marc J Struelens, Carl Suetens, Dominique L Monnet, Burden of AMR Collaborative Group, Reinhild Strauss, Karl Mertens, Thomas Struyf, Boudewijn Catry, Katrien Latour, Ivan N Ivanov, Elina G Dobreva, Arjana Tambic Andraševic, Silvija Soprek, Ana Budimir, Niki Paphitou, Helena Žemlicková, Stefan Schytte Olsen, Ute Wolff Sönksen, Pille Märtin, Marina Ivanova, Outi Lyytikäinen, Jari Jalava, Bruno Coignard, Tim Eckmanns, Muna Abu Sin, Sebastian Haller, George L Daikos, Achilleas Gikas, Sotirios Tsiodras, Flora Kontopidou, Ákos Tóth, Ágnes Hajdu, Ólafur Guólaugsson, Karl G Kristinsson, Stephen Murchan, Karen Burns, Patrizio Pezzotti, Carlo Gagliotti, Uga Dumpis, Agne Liuimiene, Monique Perrin, Michael A Borg, Sabine C de Greeff, Jos CM Monen, Mayke BG Koek, Petter Elstrøm, Dorota Zabicka, Aleksander Deptula, Waleria Hryniewicz, Manuela Caniça, Paulo Jorge Nogueira, Paulo André Fernandes, Vera Manageiro, Gabriel A Popescu, Roxana I Serban, Eva Schréterová, Slavka Litvová, Mária Štefkovicová, Jana Kolman, Irena Klavs, Aleš Korošec, Belén Aracil, Angel Asensio, María Pérez-Vázquez, Hanna Billström, Sofie Larsson, Jacqui S Reilly, Alan Johnson, Susan Hopkins

**Affiliations:** a European Centre for Disease Prevention and Control, Solna, Sweden; b Julius Center for Health Sciences and Primary Care, University Medical Center Utrecht, Utrecht, Netherlands; c University Hospital of North Norway, Tromsø, Norway; d Research Group for Host-Microbe Interaction, Faculty of Health Sciences, UiT The Arctic University of Norway, Tromsø, Norway; e Santé publique France, Saint-Maurice, France; f Centre for Infectious Disease Control, National Institute for Public Health and the Environment (RIVM), Bilthoven, Netherlands; g Department of Epidemiology and Public Health, Sciensano, Brussels, Belgium; h Department of Veterinary Public Health and Food Safety, Faculty of Veterinary Medicine, Ghent University, Merelbeke, Belgium; i Organisation for Economic Co-operation and Development, Paris, France

## Abstract

**Background:**

Infections due to antibiotic-resistant bacteria are threatening modern health care. However, estimating their incidence, complications, and attributable mortality is challenging. We aimed to estimate the burden of infections caused by antibiotic-resistant bacteria of public health concern in countries of the EU and European Economic Area (EEA) in 2015, measured in number of cases, attributable deaths, and disability-adjusted life-years (DALYs).

**Methods:**

We estimated the incidence of infections with 16 antibiotic resistance–bacterium combinations from European Antimicrobial Resistance Surveillance Network (EARS-Net) 2015 data that was country-corrected for population coverage. We multiplied the number of bloodstream infections (BSIs) by a conversion factor derived from the European Centre for Disease Prevention and Control point prevalence survey of health-care-associated infections in European acute care hospitals in 2011–12 to estimate the number of non-BSIs. We developed disease outcome models for five types of infection on the basis of systematic reviews of the literature.

**Findings:**

From EARS-Net data collected between Jan 1, 2015, and Dec 31, 2015, we estimated 671 689 (95% uncertainty interval [UI] 583 148–763 966) infections with antibiotic-resistant bacteria, of which 63·5% (426 277 of 671 689) were associated with health care. These infections accounted for an estimated 33 110 (28 480–38 430) attributable deaths and 874 541 (768 837–989 068) DALYs. The burden for the EU and EEA was highest in infants (aged <1 year) and people aged 65 years or older, had increased since 2007, and was highest in Italy and Greece.

**Interpretation:**

Our results present the health burden of five types of infection with antibiotic-resistant bacteria expressed, for the first time, in DALYs. The estimated burden of infections with antibiotic-resistant bacteria in the EU and EEA is substantial compared with that of other infectious diseases, and has increased since 2007. Our burden estimates provide useful information for public health decision-makers prioritising interventions for infectious diseases.

**Funding:**

European Centre for Disease Prevention and Control.

## Introduction

Infections due to antibiotic-resistant bacteria are a threat to modern health care and have triggered the development of coordinated and comprehensive national, European, and global actions plans.^1^, ^2^ As outlined in these action plans, monitoring and evaluating interventions requires robust information on the incidence of infections with antibiotic-resistant bacteria and their effect on the health of populations; however, such information is scarce.^3^ This information would also be useful to set priorities, across and within countries, and model future scenarios.^4^

Data from the European Antimicrobial Resistance Surveillance Network (EARS-Net) are relevant when monitoring trends in the EU and European Economic Area (EEA), but do not give the full epidemiological picture, in particular for monitoring the effect of the European action plan.

There are several challenges when estimating the burden of disease associated with infections due to antibiotic-resistant bacteria. For example, sampling and microbiological procedures for testing of the isolates, data collection processes, and the structures of surveillance systems might vary between and within countries. Furthermore, knowledge of the clinical and public health consequences of infections with antibiotic-resistant bacteria in humans is still scarce. In particular, scientific debate is ongoing on the appropriate epidemiological study design and statistical inference methods to measure reliable estimates of untoward clinical outcomes attributable to infections with antibiotic-resistant bacteria.^3^, ^4^

**Research in context**

**Evidence before this study**

Estimates of the incidence, attributable mortality, attributable length of stay, and attributable disability-adjusted life-years (DALYs) of all infection types with antibiotic-resistant bacteria are scarce. We searched PubMed for articles published between inception and Aug 1, 2018, using the terms “burden” and “antimicrobial resistance”, with no language restrictions. We found 25 relevant peer-reviewed publications. Previous studies estimating the burden of infections with antibiotic-resistant bacteria were either restricted in their geographical scope, number of infections, or types of infections. Reports were published by the European Centre for Disease Prevention and Control in 2009, the US Centers for Disease Control and Prevention in 2013, and the UK Review on Antimicrobial Resistance in 2014, but these were not peer-reviewed. Challenges when estimating the incidence of diseases include poor availability of data, heterogeneous sampling and microbiological procedures for testing of the isolates, data collection processes, and differences on how surveillance systems are structured. Moreover, quantifying the risk of death or other clinical outcomes following an infection with an antibiotic-resistant bacteria, which generally occurs in patients affected by other diseases, is challenging and its attribution is debateable. To our knowledge, data on the burden of infections with antibiotic-resistant bacteria expressed by a composite health measure to compare with the effect of other diseases, have not been published to date.

**Added value of this study**

EU and European Economic Area (EEA) countries report data to the European Antimicrobial Resistance Surveillance Network (EARS-Net) on bloodstream infections with antibiotic-resistant bacteria in a coherent and standardised manner. On the basis of EARS-Net data, we developed a step-wise approach, involving other data sources (such as the European Centre for Disease Prevention and Control point prevalence survey of health-care-associated infections and antimicrobial use in European acute care hospitals in 2011–12), to determine the country-specific incidence of infections with antibiotic-resistant bacteria of public health importance in 2015. We did systematic reviews of the literature, including a standardised approach to the selection and extraction of the evidence, to inform the attributable mortality and attributable length of stay of each antibiotic resistance–bacterium combination. Finally, Monte Carlo simulations on 2400 disease models provided estimates of DALYs, which could be placed within the general context of the burden of other diseases.

**Implications of all the available evidence**

The estimated burden of infections with antibiotic-resistant bacteria in the EU and EEA was similar to the cumulative burden of influenza, tuberculosis, and HIV, was notably diverse across countries, and has increased between 2007 and 2015. Strategies to prevent and control antibiotic-resistant bacteria require coordination at EU and EEA and global levels, and interventions that are tailored to national and local challenges. Our finding that most of the estimated burden was in hospitals and other health-care settings suggests the urgent need to address antimicrobial resistance as a patient safety issue and the need for alternative treatment options for patients with such infections who have comorbidities or are otherwise vulnerable (eg, because of their poor immune system or age).

Previous studies estimating the burden of infections with antibiotic-resistant bacteria were restricted by the number of included bacteria or type of infections.^5^ In this study, we aimed to estimate the burden of infections due to selected antibiotic-resistant bacteria of public health importance in the EU and EEA in 2015, based on a country-specific evaluation of available surveillance data and on scientific evidence on attributable clinical outcomes (deaths, length of stay, risk of developing sequelae and their duration, attributable to the infections [yes or no]). We measured burden as the number of cases of all types of infections with antibiotic-resistant bacteria, the number of deaths attributable to these infections and, for the first time, the resulting number of disability-adjusted life-years (DALYs).

## Methods

### Overview

The study focused on the eight bacterial species frequently isolated from blood or cerebrospinal fluid (invasive isolates) in the EU and EEA in 2015 and reported to the EARS-Net. Other criteria considered were inclusion in the European Centre for Disease Prevention and Control (ECDC) point prevalence survey of health-care-associated infections and antimicrobial use (2011–12) and inclusion in the list of EU antibiotic resistance policy indicators in the ECDC, European Food Safety Authority, and European Medicines Agency Joint Scientific Opinion, and consideration of emerging threats (eg, colistin resistance).^6^ The included antibiotic resistance-bacterium combinations were colistin-resistant, carbapenem-resistant, or multidrug-resistant *Acinetobacter* spp; vancomycin-resistant *Enterococcus faecalis* and *Enterococcus faecium*; colistin-resistant, carbapenem-resistant, or third-generation cephalosporin-resistant *Escherichia coli*; colistin-resistant, carbapenem-resistant, or third-generation cephalosporin-resistant *Klebsiella pneumoniae*; colistin-resistant, carbapenem-resistant, or multidrug-resistant *Pseudomonas aeruginosa*; meticillin-resistant *Staphylococcus aureus* (MRSA); and penicillin-resistant and macrolide-resistant *Streptococcus pneumoniae*. Full details are provided in the appendix (p 197). We included all five types of infection: bloodstream infections (BSIs), urinary tract infections, respiratory tract infections, surgical site infections, and other infections.

### Study design and population

We adapted this study from the Burden of Communicable Diseases in Europe (BCoDE) project,^7^ which aimed to estimate DALYs and was specific to bacterium, type of antibiotic resistance, type of infection, and was based on incidence. We used the Global Burden of Disease 2010 (GBD 2010) standard life expectancy table.^8^ Years lived with disabilities (YLD) include the length of time lived with disabilities (duration) multiplied by disability weights reflecting the ill health incurred; the latter were derived from the European disability weight project.^9^

We downloaded data in aggregate format by specific age and sex strata, without any personal identifiers, and did not require informed consent from participants. The checklist from the Guidelines for Accurate and Transparent Health Estimates Reporting is shown in the appendix (pp 219–21).

We developed a four-step approach to estimate the incidence of infections with antibiotic-resistant bacteria for five types of infection and in each of the 30 countries in the EU and EEA (appendix pp 191–211). Greece did not report data on *S pneumoniae* isolates to EARS-Net.

### Disease models and attributable mortality

To account for all notable disabilities related to infections with the selected antibiotic-resistant bacteria, we developed disease models (or outcome trees) on the basis of published evidence. The baseline models for the five types of infection were expounded from a previous study that aimed to estimate the burden of health-care-associated infections,^10^ with improvements such as the inclusion of the effect of comorbidities on long-term sequelae (appendix, pp 168–90).

We did a systematic review of peer-reviewed publications on the attributable case fatality and attributable length of stay of infections with antibiotic-resistant bacteria for each selected antibiotic resistance-bacterium combination and each type of infection. The literature search focused on the effects attributable to these infections compared with a matched non-infected population or to a population infected with susceptible isolates of the same bacteria. Full details on the methodology, search strategy, results, and extraction tables are given in the appendix (pp 3–141).

The results of the literature review were critically reviewed by five authors (AC, DP, CS, LDH, and AH) who scored the publications according to a defined set of applicability criteria (appendix pp 142–67). Two authors (AC and DP) discussed and agreed on the best summary estimate for each outcome parameter (mortality and length of stay; appendix pp 3–141). The final health outcome parameter values of each of the disease models are summarised in the appendix (p 176).

### Estimation of incidence

First, using data reported to EARS-Net, we extracted the age-specific and sex-specific annual number of infections with antibiotic-resistant bacteria in 2015 in each EU and EEA country. For each antibiotic resistance-bacterium combination, unknown age and sex data were re-distributed by imputation.

Second, the ECDC National Focal Points for antimicrobial resistance and for health-care-associated infections were asked to report on the estimated country population coverage (including its uncertainties [appendix pp 198–99]) for each bacterium, reflecting the estimated national population coverage. We applied these country coverage correction factors to the number of cases reported to EARS-Net to estimate the total number of BSIs due to each selected combination of antibiotic resistance and bacterium.

Third, we adjusted the country coverage-corrected number of BSIs from EARS-Net with a multiplier reflecting the ratio of BSIs to non-BSIs for each antibiotic resistance–bacterium combination and derived from the ECDC point prevalence survey 2011–12.^11^ For each antibiotic-resistant bacterium, we applied the BSIs to non-BSIs ratio to the numbers from step two to estimate the number of urinary tract infections, respiratory tract infections, surgical site infections, and other infections. Finally, we deducted the percentage of secondary BSIs from each of the non-BSIs.

The same method used for the present study was also applied to the European Antimicrobial Resistance Surveillance System data for 2007. Information on 2007 self-reported country coverage was retrieved from the authors of the ECDC–EMEA 2009 report^12^ on the burden of multidrug-resistant bacteria in the EU and EEA. For comparison, the 2015 results were adjusted to include the same antibiotic resistance–bacterium combinations and countries, and by standardising the populations according to the Eurostat revised standard population.^13^

### Computational analysis and uncertainty

We inserted the final designs of the outcome trees into a custom version of the BCoDE modelling toolkit.^14^ For each antibiotic-resistant bacterium, five models (one for each type of infection) were made, resulting in 80 disease models that repeated for each EU and EEA country, totalling 2400 models. We entered the sex-specific and age group-specific annual number of cases of infection with the selected antibiotic-resistant bacteria in each model.

Disease model parameters are given with 95% uncertainty intervals (UIs), which were included in the calculations as uniform distribution (two parameters; minimum and maximum) or PERT distribution (three parameters; minimum, maximum, and most likely).^15^ To calculate 95% uncertainty intervals, each model was run at 10 000 iterations of Monte Carlo simulations. We did not use time discounting and age weighting.

Modelling outputs included the annual number of cases and incidence rate, the number of attributable deaths and attributable mortality rate, the number of DALYs (including years of life lost [YLLs] and YLDs), and DALY rate. We calculated values per 100 000 population. For each output, we calculated the median estimate and 95% UI on the basis of the input uncertainties. We standardised country-specific results by age group according to the Eurostat revised standard population.^13^

### Attribution to health care and analysis of MRSA

We estimated the proportion of infections with health-care-associated antibiotic-resistant bacteria on the basis of various assumptions and epidemiological data (appendix pp 216–17). We further analysed results for MRSA infections to explore the apparent contradiction between the declining proportions of MRSA among *S aureus* infections as reported to the European Antimicrobial Resistance Surveillance System and EARS-Net between 2007 and 2015, and the results of this study (appendix pp 218–19).

### Role of the funding source

No specific funding was allocated for this study, which was done as part of routine work of ECDC and participating institutions. The decision to submit for publication was taken by AC (employed by ECDC). The corresponding author had full access to all the data in the study and had final responsibility for the decision to submit for publication.

## Results

From EARS-Net data collected between Jan 1, 2015, and Dec 31, 2015, we estimated that 671 689 (95% UI 583 148–763 966) cases of infections with selected antibiotic-resistant bacteria occurred in 2015 in the EU and EEA (table 1). These infections accounted for 33 110 (28 480–38 430) attributable deaths and 874 541 (768 837–989 068) DALYs. These estimates corresponded to an incidence of 131 (113–149) infections per 100 000 population and an attributable mortality of 6·44 (5·54–7·48) deaths per 100 000 population, causing 170 (150–192) DALYs per 100 000 population. YLLs accounted for 85·3% (145 of 170) and BSIs for 71·7% (122 of 170) of total DALYs, suggesting that the attributable mortality estimates affect the final results the most, in particular for BSIs.

**Table 1:** Estimated annual burden of infection with antibiotic-resistant bacteria of public health importance, by decreasing number of DALYs per 100 0000 population, EU and European Economic Area, 2015

	**Median number of infections**	**Median number of attributable deaths**	**Median number of DALYs per 100 000 population**	**Median percentage of total DALYs**	**Median percentage of DALYs in women**	**Median percentage of DALYs per 100 000 population due to BSI**
Third-generation cephalosporin-resistant Escherichia coli*†	297 416 (255 377–341 064)	9066 (7787–10 607)	37·2 (32·8–41·8)	21·9% (37·2/170)	46·0% (87 937/191 127)	80·5% (29·9/37·2)
Meticillin-resistant *Staphylococcus aureus*	148 727 (131 757–166 361)	7049 (6308–7863)	32·6 (29·8–35·6)	19·2% (32·6/170)	38·0% (63 715/167 767)	63·9% (20·9/32·6)
Carbapenem-resistant *Pseudomonas aeruginosa*‡	61 892 (53 210–70 984)	4155 (3398–5087)	27·2 (23·0–32·0)	16·0% (27·2/170)	37·2% (52 007/139 832)	44·1% (12·0/27·2)
Third-generation cephalosporin-resistant Klebsiella pneumoniae*†	68 588 (61 459–76 068)	3687 (3370–4031)	22·5 (20·8–24·3)	13·2% (22·5/170)	35·3% (40 820/115 546)	78·0% (17·5/22·5)
Carbapenem-resistant *Acinetobacter* spp‡	27 343 (24 064–30 794)	2363 (1947–2810)	14·0 (12·0–16·2)	8·24% (14·0/170)	35·6% (25 687/72 062)	77·9% (10·9/14·0)
Carbapenem-resistant *K pneumoniae*‡	15 947 (13 473–18 478)	2118 (1795–2473)	11·5 (9·87–13·2)	6·75% (11·5/170)	34·8% (20 518/58 992)	92·9% (10·7/11·5)
Colistin-resistant *K pneumoniae*	7450 (6223–8715)	1635 (1362–1922)	8·57 (7·19–10·0)	5·04% (8·57/170)	31·7% (13 947/44 035)	95·5% (8·19/8·57)
Vancomycin-resistant *Enterococcus faecalis* and *Enterococcus faecium*	16 146 (13 206–19 334)	1081 (891–1292)	5·49 (4·68–6·47)	3·23% (5·49/170)	37·3% (10 538/28 223)	91·1% (5·00/5·49)
Multidrug-resistant P aeruginosa*§	9028 (7736–10 425)	572 (456–703)	3·14 (2·60–3·76)	1·85% (3·14/170)	41·4% (6681/16 142)	43·1% (1·35/3·14)
Colistin-resistant *E coli*	7156 (6107–8241)	621 (518–751)	2·57 (2·22–2·95)	1·51% (2·57/170)	54·4% (7182/13 209)	92·2% (2·37/2·57)
Penicillin-resistant *Streptococcus pneumoniae*¶	2836 (2581–3119)	172 (160–185)	1·54 (1·42–1·68)	0·91% (1·54/170)	30·1% (2387/7919)	49·1% (0·76/1·54)
Penicillin-resistant and macrolide-resistant *S pneumoniae*‖	2013 (1776–2252)	172 (141–206)	0·91 (0·76–1·06)	0·53% (0·91/170)	41·2% (1922/4664)	77·4% (0·70/0·91)
Multidrug-resistant *Acinetobacter* spp**	2181·5 (1942·8–2449)	100 (89·5–113)	0·90 (0·79–1·05)	0·53% (0·90/170)	56·4% (2595/4601)	30·6% (0·27/0·90)
Carbapenem-resistant *E coli*‡	2619·0 (2269·0–2961)	141 (119–165)	0·80 (0·68–0·92)	0·47% (0·80/170)	33·9% (1390/4101)	89·0% (0·71/0·80)
Colistin-resistant *Acinetobacter* spp	1084·7 (926·0–1246)	94·5 (73·9–114)	0·64 (0·53–0·77)	0·38% (0·64/170)	27·7% (918/3314)	78·1% (0·50/0·64)
Colistin-resistant *P aeruginosa*	1261·9 (1043·4–1476)	84·5 (65·5–108)	0·59 (0·46–0·72)	0·34% (0·59/170)	42·0% (1264/3007)	44·0% (0·26/0·59)
Overall	671 689 (583 148–763 966)	33 110 (28 480–38 430)	170 (150–192)	100%	38·8% (339 510/874 541)	71·7% (122/170)

Data are median number (95% uncertainty interval) or % (n/N). Data are not age-standardised. DALYs=disability-adjusted life-years. BSI=bloodstream infection.

* Excluding isolates also resistant to colistin or carbapenem.

† In 2015, most of the third-generation cephalosporin-resistant *E coli* (88·6%) and *K pneumoniae* (85·3%) isolates reported to EARS-Net produced an extended-spectrum β-lactamase.^9^

‡ Excluding isolates also resistant to colistin.

§ Resistance to three or more antibiotic groups as marker of multidrug resistance.

¶ Excluding isolates also resistant to macrolides.

‖ Excluding isolates only resistant to penicillins.

** Aminoglycoside-resistant and fluoroquinolone-resistant as marker of multidrug resistance.

67·9% (115 of 170) of the total DALYs per 100 000 were caused by infections with four antibiotic-resistant bacteria with the largest effect on health in our study: third-generation cephalosporin-resistant *E coli*, MRSA, carbapenem-resistant *P aeruginosa*, and third-generation cephalosporin-resistant *K pneumoniae* (table 1). Infections with colistin-resistant or carbapenem-resistant bacteria accounted for 38·7% (65·9 of 170) of the total DALYs per 100 000. A greater proportion of the estimated total number of DALYs occurred in men (535 032 [61·2%] of 874 541) than in women (table 1), ranging from 43·6% (2006 of 874 541) for colistin-resistant *E coli* to 72·3% (2396 of 874 541) for colistin-resistant *Acinetobacter* spp in men.

**Figure 1:**
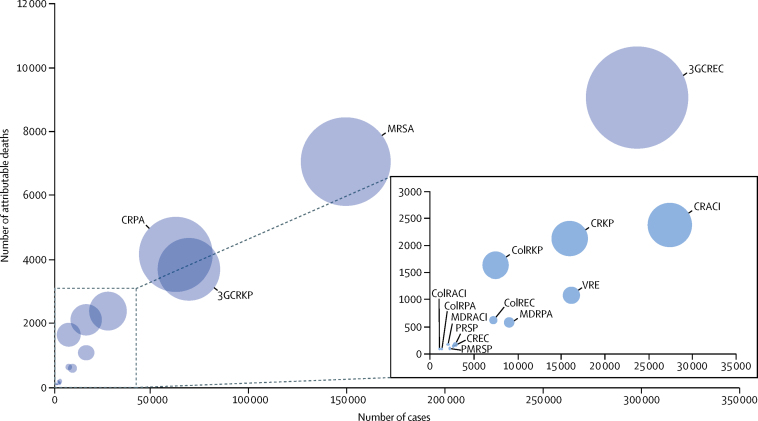
Infections with antibiotic-resistant bacteria, EU and European Economic Area, 2015 Diameter of bubbles represents the number of disability-adjusted life-years. ColRACI=colistin-resistant *Acinetobacter* spp. CRACI=carbapenem-resistant *Acinetobacter* spp. MDRACI=multidrug-resistant *Acinetobacter* spp. VRE=vancomycin-resistant *Enterococcus faecalis* and *Enterococcus faecium*. ColREC=colistin-resistant *Escherichia coli*. CREC=carbapenem-resistant *E coli*. 3GCREC=third-generation cephalosporin-resistant *E coli*. ColRKP=colistin-resistant *Klebsiella pneumoniae*. CRKP=carbapenem-resistant *K pneumoniae*. 3GCRKP=third-generation cephalosporin-resistant *K pneumoniae*. ColRPA=colistin-resistant *Pseudomonas aeruginosa*. CRPA=carbapenem-resistant *P aeruginosa*. MDRPA=multidrug-resistant *P aeruginosa*. MRSA=meticillin-resistant *Staphylococcus aureus*. PRSP=penicillin-resistant *Streptococcus pneumoniae*. PMRSP=penicillin-resistant and macrolide-resistant *S pneumoniae*.

Figure 1 shows the association between the number of cases, the number of attributable deaths, and the DALYs for each antibiotic resistance combination. The ranking of infections with antibiotic-resistant bacteria might differ depending on which indicator is used for measuring their health burden. Despite its relatively low incidence, carbapenem-resistant *K pneumoniae* had a high burden of disease because of its high attributable mortality, whereas vancomycin-resistant *E faecalis* and *E faecium* (which had a similar incidence to carbapenem-resistant *K pneumoniae*) was associated with a low burden of disease.

**Figure 2:**
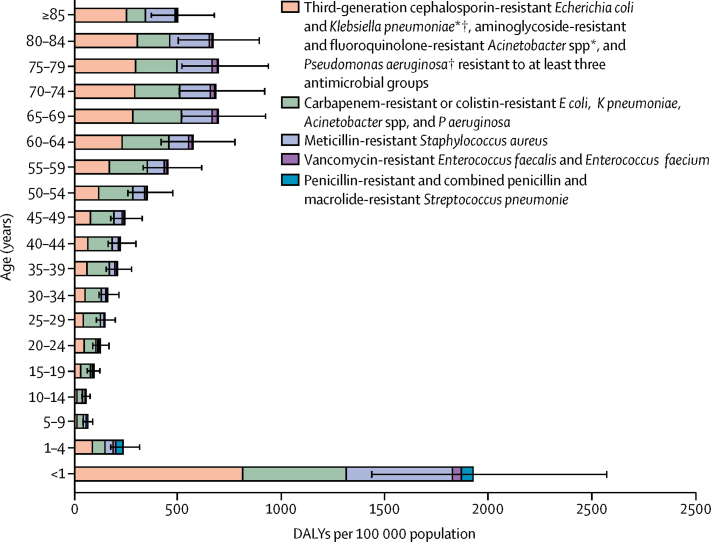
Model estimates of the burden of infections with antibiotic-resistant bacteria of public health importance in DALYs, by age group, EU and European Economic Area, 2015 Error bars are 95% uncertainty intervals. DALYs=disability-adjusted life-years. *Excludes those resistant to carbapenem or colistin. †In 2015, most of the third-generation cephalosporin-resistant *E coli* (88·6%) and *K pneumoniae* (85·3%) isolates reported to the European Antimicrobial Resistance Surveillance Network produced an extended-spectrum β-lactamase.^9^

The total burden of infections with selected antibiotic-resistant bacteria was highest in infants (aged <1 year), followed by those aged 65 years or older (figure 2).

We estimated that 63·5% (426 277 of 671 689) of cases of infections with antibiotic-resistant bacteria were associated with health care, resulting in 72·4% (23 976 of 33 110) of attributable deaths and 74·9% (127 of 180) of DALYs per 100 000 population. This finding suggests that the health effects of infections with antibiotic-resistant bacteria predominantly occur in hospitals and other health-care settings.

**Figure 3:**
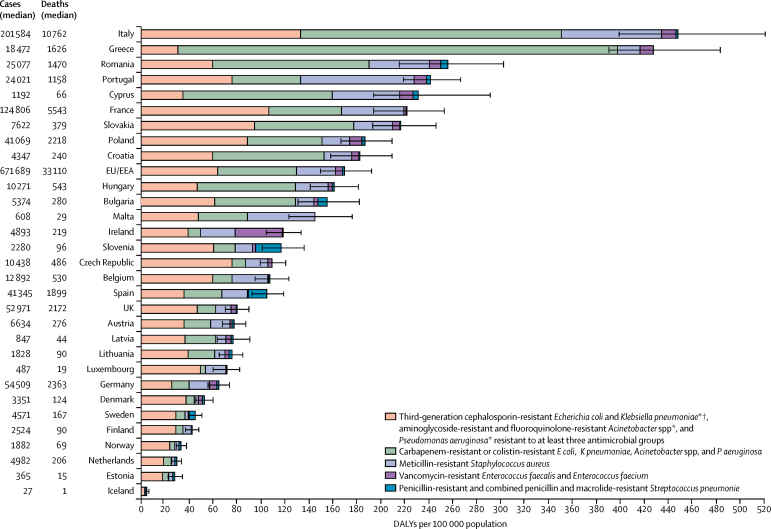
Burden of infections with antibiotic-resistant bacteria in DALYs, EU and European Economic Area, 2015 Error bars are 95% uncertainty intervals. Greece did not report data on *S pneumoniae* isolates to the European Antimicrobial Resistance Surveillance Network in 2015. DALY rates are age-standardised to limit the effect of demographic differences across countries; numbers of cases and deaths are not age-standardised. DALYs=disability-adjusted life-years. *Excludes those resistant to carbapenem or colistin. †In 2015, most of the third-generation cephalosporin-resistant *E coli* (88·6%) and *K pneumoniae* (85·3%) isolates reported to the European Antimicrobial Resistance Surveillance Network produced an extended-spectrum β-lactamase.^9^

Italy and Greece had a substantially higher estimated burden of antibiotic-resistant bacteria than other EU and EEA countries, with carbapenem-resistant or colistin-resistant bacteria causing a larger proportion of the total burden in Greece than it did in Italy (figure 3). In 2015, in addition to a substantial burden due to infections with carbapenem-resistant or colistin-resistant bacteria, Portugal and Malta had a substantial burden due to MRSA infections. In Ireland, vancomycin-resistant *E faecalis* and *E faecium* caused a higher proportion of the total burden than in other countries. In Spain and Slovenia, a higher proportion of their burden estimates were due to antibiotic-resistant *S pneumoniae* infections than in other countries.

**Figure 4:**
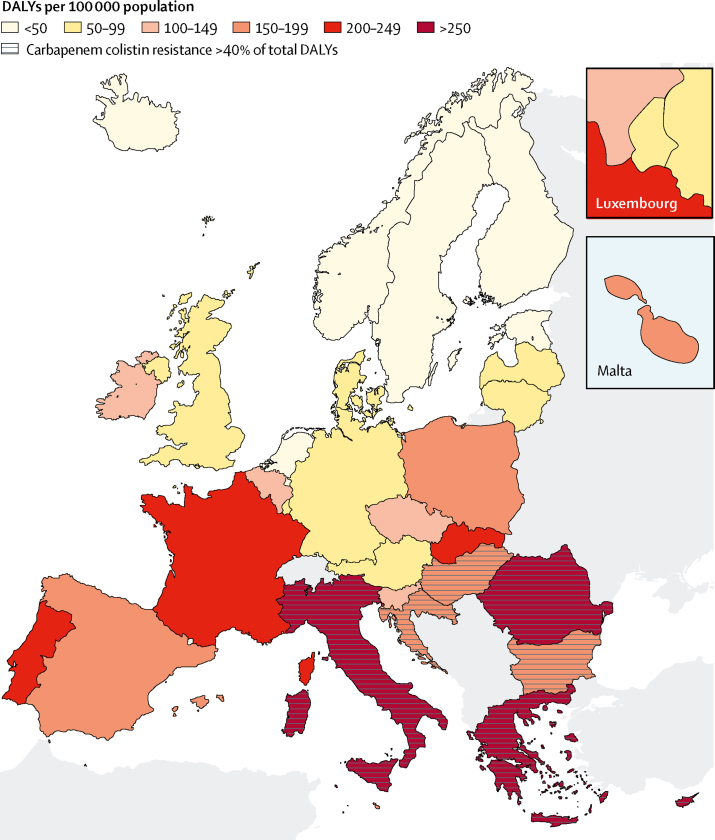
Model estimates of the burden of infections with selected antibiotic-resistant bacteria of public health importance in DALYs per 100 000 population, EU and European Economic Area, 2015 Greece did not report data on *S pneumoniae* isolates to the European Antimicrobial Resistance Surveillance Network in 2015. DALYs=disability-adjusted life-years.

The burden of infections with antibiotic-resistant bacteria was focused in the southern and eastern part of the EU and EEA (figure 4). In Croatia, Bulgaria, and Hungary more than 40% of the burden was due to infections with carbapenem-resistant or colistin-resistant bacteria, but the total burden in these countries was similar to the EU and EEA average. More detailed information on results per country is shown in the appendix (pp 222–55).

The estimated age-standardised number of cases of infections with antibiotic-resistant bacteria was 239 238 (95% UI 215 544–262 951) in 2007, which increased to 602 609 (524 237–686 497) in 2015 (table 2). The median number of attributable deaths increased from 11 144 (9999–12 407) in 2007 to 27 249 (23 544–31 471) in 2015. The burden of carbapenem-resistant *K pneumoniae* increased the most (by 6·16 times) in terms of number of infections and number of deaths, followed by carbapenem-resistant *E coli*, third-generation cephalosporin-resistant *E coli*, and third-generation cephalosporin-resistant *K pneumoniae*. The number of deaths attributable to third-generation cephalosporin-resistant *E coli* infections increased by 4·12 times during 2007–15, increasing to 8750 (7505–10 262).

**Table 2:** Estimated annual burden of infections with selected antibiotic-resistant bacteria of public health importance, age-group standardised, EU and European Economic Area, 2007–15

	**Median number of infections**		**Median number of attributable deaths**		**Factor increase in attributable deaths between 2007 and 2015**
	2007	2015	2007	2015	
Third-generation cephalosporin-resistant Escherichia coli*†	70 276 (63 113–77 778)	285 758 (246 318–328 828)	2139 (1901–2420)	8750 (7505–10 262)	4·12 (3·29–5·13)
Meticillin-resistant *Staphylococcus aureus*	112 782 (103 186–122 006)	143 947 (127 592–161 158)	5340 (4952–5723)	6810 (6096–7559)	1·28 (1·11–1·47)
Carbapenem-resistant *Pseudomonas aeruginosa*	17 972 (15 685–20 170)	59 529 (51 237–68 238)	1216 (1000–1469)	4008 (3235–4898)	3·29 (2·41–4·46)
Third-generation cephalosporin–resistant Klebsiella pneumoniae*†	16 474 (15 097–17 825)	64 980 (58 360–72 048)	891 (830–950)	3508 (3197–3824)	3·95 (3·51–4·43)
Carbapenem-resistant *K pneumoniae*	2535 (2125–2952)	15 910 (13 352–18 377)	341 (288–404)	2094 (1779–2460)	6·16 (4·78–8·04)
Vancomycin-resistant *Enterococcus faecalis* and *Enterococcus faecium*	8277 (6699–9950)	15 917 (12 900–19 092)	538 (452–652)	1065 (874–1283)	1·95 (1·47–2·58)
Multidrug-resistant *P aeruginosa*‡	5603 (4796–6430)	8749 (7470–10 044)	357 (281–439)	556 (447–681)	1·55 (1·11–2·17)
Penicillin-resistant *Streptococcus pneumoniae*§	2183 (2033–2355)	2817 (2552–3104)	134 (126–143)	171 (159–184)	1·28 (1·15–1·42)
Penicillin-resistant and macrolide-resistant *S pneumoniae*¶	1916 (1782–2075)	2386 (2173–2648)	118 (110–126)	145 (135–158)	1·25 (1·12–1·40)
Carbapenem-resistant *E coli*	543 (442–647)	2616 (2283–2960)	29·2 (22·2–37·6)	141 (118–163)	4·76 (3·51–6·90)
Overall	239 238 (215 544–262 951)	602 609 (524 237–686 497)	11 144 (9999–12 407)	27 249 (23 544–31 471)	2·46 (1·01–3·00)

Data are median (95% uncertainty interval) and are age-standardised. Note that only bacteria under surveillance in both 2007 and 2015 are included in this analysis.

* Excluding isolates resistant to colistin or carbapenems.

† In 2015, most of the third-generation cephalosporin-resistant *E coli* (88·6%) and *K pneumoniae* (85·3%) isolates reported to EARS-Net produced an extended-spectrum β-lactamase.^9^

‡ Resistance to three or more antibiotic groups as marker of multidrug resistance.

§ Excluding isolates resistant to macrolides.

¶ Excluding isolates resistant to penicillins, but not to macrolides.

Although the EU and EEA population-weighted proportion of MRSA among *S aureus* isolates reported to EARS-Net decreased from 26·6% in 2007 (Diaz Högberg L, European Centre for Disease Prevention and Control, personal communication) to 16·8% in 2015, our study found that the estimated incidence of MRSA infections increased by 1·28 times (95% UI 1·11–1·47). The estimated age-specific incidence of MRSA in 2007 and 2015 showed that incidence mainly increased in infants and in people aged 55 years or older; appendix p 217). In adults, the estimated incidence decreased during 2007–15, although this decrease was not significant.

## Discussion

To our knowledge, this study is the first to estimate the burden of all types of infections with antibiotic-resistant bacteria expressed in DALYs. We aimed to provide reliable data for population health indicators, through a comprehensive and evidence-based approach, for planning, prioritisation, and to inform policy for control and prevention of this increasing public health threat. Moreover, DALYs allow for comparisons with the burden of other diseases and our incidence-based approach helps to assess the effect of future prevention and control interventions.^16^

Our findings show that all age groups are affected by infections with antibiotic-resistant bacteria, although their burden is significantly higher among infants than in any other age group. Among adults, the burden increases with age, suggesting that the ageing EU and EEA population could result in an increasing burden. In adults and young adults, a higher proportion of the burden was caused by infections with carbapenem-resistant and colistin-resistant bacteria. This finding might be due to a lower risk of complications after an infection in this age group in general, except for patients who are often admitted to hospital and have difficult-to-treat infections because of carbapenem or colistin resistance.

Our finding of 170 DALYs per 100 000 population is similar to the combined burden of three major infectious diseases (influenza, tuberculosis, and HIV), which was 183 DALYs per 100 000 population.^17^ We estimated that about 75% of the total burden of infections with antibiotic-resistant bacteria in EU and EEA countries (ie, 127 DALYs per 100 000 population) were associated with health care. This estimation would mean, if compared with a previous study on the burden of health-care-associated infections in the EU and EEA,^10^ that 25% (127 of 501 DALYs per 100 000) of the burden of health-care-associated infections is due to such infections with antibiotic-resistant bacteria selected for our study. However, given the differences in the data sources and methods used to estimate the incidence of infections, this comparison should be considered with caution.

In 2013, the US Centers for Disease Control and Prevention published the first estimates of the burden of infections with antibiotic-resistant bacteria in the USA, based on 2011 national surveillance data.^18^ Our study estimated a 2·6 times higher incidence of infections with antibiotic-resistant bacteria (131 cases per 100 000 population), although attributable mortality was only 1·22 times higher in our study. This increase is due to our conservative approach when defining case fatality of infections with antibiotic-resistant bacteria. The structures and resources that are available for the prevention and control of infections with antibiotic-resistant bacteria might have also caused differences, particularly in health care.

In 2016, a study^19^ estimated the morbidity and mortality associated with antibiotic-resistant bacteria in France based on 2012 EARS-Net data. We used a similar methodology and found a similar incidence for France. Nevertheless, we estimated fewer MRSA infections, which can partly be explained by the decreasing trends in MRSA infections in France between 2012 and 2015, and much fewer carbapenem-resistant *P aeruginosa* infections (fewer than 50% of the number in the French study). We also estimated half the number of attributable deaths as in the French study, because of the reduced case fatality proportion stemming from our literature review.

Between 2007 and 2015, the burden increased for all antibiotic-resistant bacteria. The proportion of the DALYs due to all carbapenem-resistant bacteria combined increased from 18% (56 150 of 311 715) in 2007 to 28% (185 421 of 678 845) in 2015, and the proportion of the DALYs due to carbapenem-resistant *K pneumoniae* and carbapenem-resistant *E coli* combined doubled from 4·3% (13 515 of 311 715) in 2007 to 8·79% (57 536 of 678 845) in 2015, reflecting the emergence and rapid increase of carbapenem-resistant *K pneumoniae* infections in the EU and EEA during this period.

We were initially surprised to find that the incidence of MRSA infections increased between 2007 and 2015, given that the proportion of MRSA over meticillin-susceptible *S aureus* had decreased. This increase could be because of the increased reporting of *S aureus* BSI overall from 30 027 cases in 2007 to 45 364 cases in 2015. Further analysis of the age group-specific incidence of MRSA infections in 2007 and 2015 showed that the increase was mainly seen in infants and people aged 55 years or older (appendix p 217). The elderly population is more vulnerable to MRSA infections^20^ and this population has grown since 2007; the improvement of neonatal services, leading to an increased survival of at-risk infants, might have also contributed to further increasing the size of the population at risk for MRSA infection. Studies in Sweden,^21^ Finland,^22^ and Norway,^23^ have also found that incidence of MRSA did not decrease in these countries.

Italy and Greece have the greatest burden of infections due to antibiotic-resistant bacteria or a combined 21·3% (171 899 of 874 541) of the EU and EEA total DALYs per 100 000 population and 36·2% (319 019 of 874 541) of EU and EEA DALYs per 100 000 population due to carbapenem-resistant or colistin-resistant bacteria. Even if one considers its large and ageing population, it is notable that about a third of the deaths due to infections with antibiotic-resistant bacteria in the EU and EEA were in Italy. Italy has published its National Action Plan on Antimicrobial Resistance 2017–20,^24^ which includes targets for the reduction of antibiotic use and the control of health-care-associated infections. Greece published its National Action Plan (known as Procrustes) in 2010, which outlined the best practices for monitoring and preventing infections due to carbapenem-resistant Gram-negative bacteria.^25^ Given that, in 2015, most of the burden in Greece was due to infections with carbapenem-resistant or colistin-resistant bacteria (overall case fatality proportion of 8·80), there is an urgent need to expand the measures to contain carbapenem-resistant bacteria in this country.

Our results are consistent with the European survey on carbapenemase-producing Enterobacteriaceae, which highlighted the geographical heterogeneity of carbapenemase-producing Enterobacteriaceae in the EU and EEA, and the endemic situation in Italy and Greece,^26^ where the incidence of such infections per 100 000 patient-days was the highest of all EU and EEA countries.^27^ Grundmann and colleagues^27^ reported a ratio of 11 to one for *K pneumoniae* to *E coli* carbapenemase-producing bacteria; our study also found a higher number of carbapenem-resistant *K pneumoniae* than carbapenem-resistant *E coli* (ratio six to one).

Considering that, in our study, a large proportion of the burden was due to health-care-associated bloodstream, respiratory tract, or surgical site infections, and that more than half of health-care-associated infections are considered preventable,^28^ reducing the burden of antibiotic-resistant bacteria in the EU and EEA through enhanced infection prevention and control measures could be an achievable goal. ECDC recently published guidance on infection prevention and control measures and tools for the prevention of the spread of carbapenem-resistant Enterobacteriaceae in hospitals or other health-care settings.^29^ Stewardship interventions can be successful at safely reducing unnecessary use of antibiotics in hospitals.^30^

A substantial proportion of the burden of infections with antibiotic-resistant bacteria in the EU and EEA in 2015 was estimated to have been due to community-associated infections. This finding suggests that antimicrobial stewardship targeting prescribers and infection prevention and control interventions in primary care would also be necessary to reduce the burden of these infections in the EU and EEA.

Our study has several limitations. The disease models were based on the data retrieved from systematic literature reviews, which varied in availability, quality, and representativeness of evidence. We did not grade the strength of evidence supporting each parameter estimate on the basis of the statistical analysis methods used in the clinical outcome studies. Moreover, death from an infection with antibiotic-resistant bacteria is the result of many factors that are related to the pathogen, patient, and therapy, in particular regarding the delay in the administration of appropriate antibiotic therapy.^4^ We did not adjust our models for age-specific risks, co-infections, appropriateness of antibiotic therapy, or for type of care, assuming common transition probabilities for all subgroups. However, to cover uncertainties related to the different patient case-mix, we considered studies focusing on specific populations for inclusion in the parameters of the disease models (appendix pp 168–90), and included when pertinent.

In the appendix (p 203), we list the limitations related to the method of estimating the incidence of infections from EARS-Net data, including frequency of susceptibility testing and representativeness of participating laboratories (geographical, type of hospital, and case-mix of patients). ECDC is currently working with countries in the evaluation of all factors affecting the EARS-Net country coverage. We also list limitations related to the factors used for converting the number of BSIs to other types of infection (appendix p 203), including the different time span of the ECDC point prevalence survey (2011–12) and EARS-Net, application of data from the point prevalence survey to community-associated infections, dependence of health-care-associated infections on the day of measurement, and estimation of non-BSIs (might have been affected by the case-mix of patients and could differ between hospitals). Finally, we defined multidrug-resistant isolates (appendix p 196) on the basis of antibiotic groups frequently used for empirical treatment of BSI as included in EARS-Net. Nevertheless, our definition might not reflect the available options for treatment in each individual case.

The strength of this study is the high quality of the surveillance data sources. EARS-Net and ECDC point prevalence survey 2011–12 are the most comprehensive, standardised, multi-country surveillance initiatives to date for antibiotic-resistant bacteria and health-care-associated infections. Another strength was the use of systematic literature reviews to determine the best available estimates of attributable mortality, attributable length of stay, and attributable short-term and long-term complications of infections.

To our knowledge, this study estimated for the first time the burden of five types of infection with antibiotic-resistant bacteria in the EU and EEA expressed in DALYs and provided reliable EU and EEA and country-specific profiles for 2015 data.

The estimated burden of infections with antibiotic-resistant bacteria in the EU and EEA is substantial compared with that of other infectious diseases, and has increased since 2007. Strategies to prevent and control antibiotic-resistant bacteria require coordination at EU and EEA and global level. However, our study showed that the contribution of various antibiotic-resistant bacteria to the overall burden varies greatly between countries, thus highlighting the need for prevention and control strategies that are tailored to the needs of each country in the EU and EEA. Our study also showed that most of the burden of infections with antibiotic-resistant bacteria in the EU and EEA was health care associated, thus emphasising the need to urgently address antimicrobial resistance as a patient safety issue and the need for alternative treatment options for patients with such infections who have co-morbidities or are otherwise vulnerable (eg, because of their poor immune system or age).

Future studies should include estimates of the burden of infections due to other antibiotic-resistant bacteria of public health importance, such as drug-resistant *Mycobacterium tuberculosis*, drug-resistant *Salmonella* spp, and drug-resistant *Neisseria gonorrhoeae*, to give a more comprehensive estimate of the burden of antimicrobial resistance. In the long term, research should be done to better understand the factors underlying the estimations of EARS-Net country coverage, such as catchment population, patient case-mix, laboratory capacity, and the appropriateness and frequency of collection of blood cultures.
