# Supplementary material for: Attributable deaths and disability-adjusted life-years caused by infections with antibiotic-resistant bacteria in the EU and the European Economic Area in 2015: a population-level modelling analysis
Source: Lancet Infect Dis. 2019 Jan;19(1):56–66. doi: 10.1016/S1473-3099(18)30605-4 (PMC6300481; doi:10.1016/S1473-3099(18)30605-4)
Supplement: Supplementary appendix [file mmc1.pdf]

THE LANCET
Infectious Diseases

Supplementary appendix

This appendix formed part of the original submission and has been peer reviewed. We post it as supplied by the authors.

Supplement to: Cassini A, Högberg LD, Plachouras D, et al. Attributable deaths and disability-adjusted life-years caused by infections with antibiotic-resistant bacteria in the EU and the European Economic Area in 2015: a population-level modelling analysis. *Lancet Infect Dis* 2018; published online Nov 5. http://dx.doi.org/10.1016/S1473-3099(18)30605-4.

Burden of antimicrobial resistance

Appendix to

“Attributable deaths and disability-adjusted life-years caused by infections with antibiotic-resistant bacteria in the European Union and the European Economic Area in 2015: a population-level health estimate”

Contents

Literature review report 3

Literature selection grids... 142

Final disease outcome trees... 168

Methodology protocol to estimate incidence.. 191

GATHER checklist and further analysis on MRSA and attribution as healthcare-associated infections.. 213

Country specific results... 223

Burden of antimicrobial resistance

Literature review report

Literature search undergone by Ana Hoxha and Victoria Simpkin

Report written by Ana Hoxha and Alessandro Cassini with contributions from Carl Suetens, Liselotte Diaz Högberg, Diamantis Plachouras and Dominique Monnet

Contents

List of abbreviations 5
Introduction 6
Methodology 6
PICO Questions 7
PRISMA Checklist 9
Results 14
1. Carbapenem resistant *Acinetobacter baumannii* 14
2. Colistin resistant Acinetobacter baumannii 21
3. Multidrug resistant Acinetobacter baumannii 24
4. Carbapenem resistant *Escherichia coli* 31
5. Colistin resistant *Escherichia coli* 35
6. Third-generation Cephalosporin resistant *Escherichia coli* 38
7. Carbapenem resistant *Klebsiella pneumoniae* 47
8. Colistin resistant Klebsiella pneumoniae 55
9. Third-generation Cephalosporin resistant *Klebsiella pneumoniae* 58
10. Methicillin resistant Staphylococcus aureus 66
11. Carbapenem resistant Pseudomonas aeruginosa 86
12. Colistin resistant Pseudomonas aeruginosa 92
13. Multidrug resistant Pseudomonas aeruginosa 94
14. Penicillin resistant *Streptococcus pneumoniae* 101
15. Penicillin and macrolide resistant *Streptococcus pneumoniae* 106
16. Vancomycin resistant *Enterococcus faecalis* and faecium 107
Conclusions 116
References 118

List of abbreviations

3GCREC	third-generation cephalosporin-resistant *E. coli*
3GCRKP	third-generation cephalosporin-resistant *K. pneumoniae*
AMR	antimicrobial resistance
BSI	bloodstream infection
CAI	Community-associated infections
CI	confidence interval
ColRACI	colistin-resistant *Acinetobacter* spp.
ColREC	colistin-resistant *E. coli*
ColRKP	colistin-resistant *K. pneumoniae*
ColRPA	colistin-resistant *P. aeruginosa*
CRACI	carbapenem-resistant *Acinetobacter* spp.
CREC	carbapenem-resistant *E. coli*
CRKP	carbapenem-resistant *K. pneumoniae*
CRPA	carbapenem-resistant *P. aeruginosa*
CSF	cerebral spine fluid
DALY	disability-adjusted life years
EARS-Net	European Antimicrobial Resistance Surveillance Network
EEA	European Economic Area
EU	European Union
HAI	healthcare-associated infection
LOS	length of stay
MDR	multidrug-resistant
MDRACI	multidrug-resistant *Acinetobacter* spp
MDRPA	multidrug-resistant *P. aeruginosa*
MRSA	meticillin-resistant *Staphylococcus aureus*
MS	Member State
NI	Non infected
OECD	Organisation for Economic Co-operation and Development
OTH	other infection site including digestive tract infections, skin and soft tissue infections (SSTI), eye, ear, nose or mouth infections, bone and joint infections, cardiovascular infections, reproductive tract infections and other less frequent infections
PMRSP	penicillin- and macrolide-resistant S. pneumoniae
PPS	point prevalence survey of healthcare-associated infections and antimicrobial use in acute care hospitals
PRSP	penicillin-resistant *S. pneumoniae*;
R	Resistant
RESP	respiratory infections (including pneumonia, and low respiratory tract infection)
S	Susceptible
S-BSI	secondary BSI
SP	specified pathogens
SPDAR	specified pathogens with defined antimicrobial resistance
SSI	surgical site infection
UTI	urinary tract infection
VRE	vancomycin-resistant enterococci

Introduction

This document is part of the burden of antibiotic resistance in Europe project.
The objective of this research was to retrieve available evidence from peer-reviewed publications on the attributable case fatality and attributable length of stay (LOS) of resistant bacteria under surveillance by the European Antimicrobial Resistance Surveillance Network (EARS-Net) and as defined in the Supplementary appendix 4. The aim of the review is to provide information for relevant disease progression models.

Methodology

The bacteria included in the review were the followings; *Acinetobacter baumanni, Escherichia coli, Klebsiella pneumoniae, Staphylococcus aureus, Pseudomonas aeruginosa, Streptococcus pneumoniae, Enterococcus faecalis* and *Enterococcus faecium*. For each of these bacteria we performed separate reviews to study the resistance to specific antibiotics or specific antibiotic class. The following literature reviews were performed:

1. Colistin Resistant *Acinetobacter baumannii*
2. Carbapenem Resistant *Acinetobacter baumannii*
3. Multidrug resistant *Acinetobacter baumannii*
4. Colistin Resistant *Escherichia coli*
5. Carbapenem Resistant *Escherichia coli*
6. Third-generation Cephalosporin Resistant *Escherichia coli*
7. Colistin Resistant *Klebsiella pneumoniae*
8. Carbapenem Resistant *Klebsiella pneumoniae*
9. Third-generation Cephalosporin Resistant *Klebsiella pneumoniae*
10. Meticillin-resistant *Staphylococcus aureus*
11. Colistin Resistant *Pseudomonas aeruginosa*
12. Carbapenem Resistant *Pseudomonas aeruginosa*
13. Multidrug resistant *Pseudomonas aeruginosa*
14. Penicillin resistant *Streptococcus pneumoniae*
15. Penicillin and macrolide resistant *Streptococcus pneumoniae*
16. Vancomycin resistant *Enterococcus faecalis* and *faecium*

The databases used for the literature review were PubMed, EMBASE and Cochrane Database of Systematic Reviews. Each review was performed using specific key words; research filters were applied where specified below. No time filter was applied, except for meticillin-resistant *Staphylococcus aureus* (MRSA), see below. Searches were undergone between April 2016 and December 2016, articles published after these dates might not be included.

Most studies identified were based on hospitalized populations. Therefore, we define as "attributable" the quantitative information below on mortality and/or case fatality (CF) and/or length of stay (LOS) as being attributable to the infection, factoring out the risk resulting from the underlying co-morbidity. This attributable risk (risk difference) is the comparison of the absolute risks between patients with the respective resistant (R) infection

and patients (with similar characteristics) without the infection (non-infected, NI) or infected with a susceptible (S) infection.

Primary studies reporting original results were considered eligible. Existing systematic reviews and meta-analysis were also included

To be eligible, a study had to fulfil the following inclusion criteria defined a priori:

1. Original study reporting mortality rates, attributable mortality data, case fatality rates, attributable case fatality data.
2. Original study reporting length of stay in hospital or/and attributable length of stay as compared to controls.
3. Include a control group defined as affected by a susceptible infection or/and a control group of not infected patients.

Relevant data were extracted, collected in excel spread sheets and presented in the relevant tables below.

Matched case-control or cohort studies were considered having a higher value of evidence, though we did not apply any evaluation criteria to the included studies.

Studies focusing on specific sites of infection (e.g. blood stream infections) were prioritized, although studies not specifying the infection site were also included and the data extracted.

Case report studies and articles with less than 5 case patients were excluded.

Most eligible studies reported an infected case group and a control group. The latter could be represented by non-infected (NI) patients or patients reporting an infection susceptible (S) to the considered antibiotic/class of antibiotics.

PICO Questions

Research question: What is the attributable mortality and the attributable length of stay of patients with infections with antibiotic-resistant bacteria when compared with non-infected or susceptible infection?

**Population**	**Intervention (exposure)**
Patients with defined infections with antibiotic-resistant bacteria, stratified for age group and infection type/site.	Infection due to:<br>– Colistin Resistant *Acinetobacter baumannii*<br>– Carbapenem Resistant *Acinetobacter baumannii*<br>– Multidrug resistant *Acinetobacter baumannii*<br>– Colistin Resistant *Escherichia coli*<br>– Carbapenem Resistant *Escherichia coli*<br>– Third-generation Cephalosporin Resistant *Escherichia coli*<br>– Colistin Resistant *Kl*ebsiella pneumoniae<br>– Carbapenem Resistant *Klebsiella pneumoniae*

	– Third-generation Cephalosporin Resistant *Klebsiella pneumoniae*<br>– Methicillin-resistant *Staphylococcus aureus*<br>– Colistin Resistant *Pseudomonas aeruginosa*<br>– Carbapenem Resistant *Pseudomonas aeruginosa*<br>– Multidrug resistant *Pseudomonas aeruginosa*<br>– Penicillin resistant *Streptococcus pneumoniae*<br>– Penicillin and macrolide resistant *Streptococcus pneumoniae*<br>– Vancomycin resistant *Enterococcus faecalis* and *Enterococcus faecium*
**Comparison**<br>Non-infected patients or patients infected with susceptible strain of the same species.	**Outcome**<br>Attributable case fatality and attributable length of stay.

PRISMA Checklist

Section/topic	#	Checklist item
**TITLE**		
Title	1	Burden of infections with antibiotic resistant bacteria: a systematic literature review of case fatality and length of stay
**ABSTRACT**		
Structured summary	2	Background and Objectives: in order to estimate the burden infections with antibiotic-resistant we built disease progression models. We performed several systematic reviews of the literature to extract the best available evidence on the attributable case fatality proportion and attributable length of stay (LOS) in order to inform the disease progression models. Separate systematic literature reviews were performed for each bacteria under surveillance by the European Antimicrobial Resistance Surveillance Network (EARS-Net), each type of resistance and each infection type/site.<br><br>Data source: The articles were searched on PubMed, Embase and the Cochrane Database of Systematic Reviews.<br>Study Eligibility criteria: The review included primary studies reporting case fatality rate and LOS of patients with the resistant (R) infection and patients (with similar characteristics) without the infection (non-infected, NI) or infected with a susceptible (S) strain, for each resistant bacteria and each infection type/site.<br>Participants: The cases were infected patients with a resistant infection while the controls had a susceptible or infection or were non-infected. Both groups were followed up for a period of 14-30 days measuring their mortality and LOS rates.<br>Results: The number of studies varied between the different types of resistance considered, ranging from the 0 for colistin resistant E. coli to 44 for meticillin resistant S. aureus. The information was extracted for all studies included in the review and outcomes are available in Table form.<br>Limitations: The major limitation of the review includes the heterogeneity of the studies, in terms of settings as well as type of patients and outcomes.<br>Conclusions: We found a wide difference between the studies identified in our search. Many are limited to specific healthcare services (e.g. ICUs) and/or performed on particular groups of patients (e.g. only children, only elderly) and/or with specific underlying conditions (e.g. immunodeficiency, organ transplant). Therefore, extracting valid case attributable fatality proportions and attributable LOS will be particularly challenging. Based on the heterogeneity of the studies detected, we advise to avoid pooling and averaging the results based on our categorizing criteria. We suggest an alternative method for selecting and extracting the data for the disease progression models.

**INTRODUCTION**		
Rationale	3	In order to estimate the burden infections with antibiotic-resistant we built disease progression models. We performed several systematic reviews of the literature to extract the best available evidence on the attributable case fatality proportion and attributable length of stay (LOS) in order to inform the disease progression models. Separate systematic literature reviews were performed for each bacteria under surveillance by the European Antimicrobial Resistance Surveillance Network (EARS-Net), each type of resistance and each infection type/site.
Objectives	4	To identify evidence from published scientific literature on the attributable case fatality and attributable length of stay (LOS) of infections due to antibiotic-resistant bacteria under surveillance by the European Antimicrobial Resistance Surveillance Network (EARS-Net).
**METHODS**		
Protocol and registration	5	NA
Eligibility criteria	6	To be eligible, a study had to fulfil the following inclusion criteria defined a priori:<br>– Original study reporting mortality rates, attributable mortality data, case fatality rates, attributable case fatality data.<br>– Original study reporting length of stay in hospital or/and attributable length of stay as compared to controls.<br>– Include a control group defined as affected by a susceptible infection or/and a control group of not infected patients.<br>The bacteria included in the review were the following; *Acinetobacter baumanni*, *Escherichia coli*, *Klebsiella pneumoniae*, *Staphylococcus aureus*, *Pseudomonas aeruginosa*, *Streptococcus pneumoniae*, *Enterococcus faecalis* and *Enterococcus faecium*. For each of these bacteria we performed separate reviews to study the resistance to specific antibiotics or specific antibiotic class. The following literature reviews were performed:<br>1. Colistin Resistant *Acinetobacter baumannii*<br>2. Carbapenem Resistant *Acinetobacter baumannii*<br>3. Multidrug resistant *Acinetobacter baumannii*<br>4. Colistin Resistant *Escherichia coli*<br>5. Carbapenem Resistant *Escherichia coli*<br>6. Third-generation Cephalosporin Resistant *Escherichia coli*<br>7. Colistin Resistant *Kl*ebsiella pneumoniae<br>8. Carbapenem Resistant *Klebsiella pneumoniae*<br>9. Third-generation Cephalosporin Resistant *Klebsiella pneumoniae*<br>10. Methicillin-resistant *Staphylococcus aureus*

		11. Colistin Resistant *Pseudomonas aeruginosa*<br>12. Carbapenem Resistant *Pseudomonas aeruginosa*<br>13. Multidrug resistant *Pseudomonas aeruginosa*<br>14. Penicillin resistant *Streptococcus pneumoniae*<br>*15.* Penicillin and macrolide resistant *Streptococcus pneumoniae*<br>16. Vancomycin resistant *Enterococcus faecalis* and *Enterococcus faecium*
Information sources	7	PubMed, Embase, Cochrane Database of Systematic Reviews
Search	8	In the different information sources, we used the following string types:<br>Pubmed: we used once the mesh term "*bacteria name*" AND all fields "*antibiotic name* resistant", and the second time the mesh term "*bacteria name*" AND all fields "*antibiotic name* resistance". Both times a filter for human studies was applied.<br>Embase: we searched for '*bacteria name*' AND '*antibiotic name* resistant', and '*bacteria name* AND '*antibiotic name* resistance'. We applied a filter to include only human studies.<br>Cochrane Library: we performed an advanced search using terms "*bacteria name*" AND "*antibiotic name*".
Study selection	9	All results from the three different data sources were exported in a reference manager and duplicates were removed. The first screening was performed by the articles titles, then by the abstract and finally by the evaluation of the full text.
Data collection process	10	The data of the included studies were extracted in an excel file containing a list of indicators of interest and are displayed in Tables in the report of the systematic review and in excel files for scoring (Appendix 2).
Data items	11	The indicators reported in the Literature review report tables (Appendix 1) include:<br>• Author<br>• Study period<br>• Outcome<br>• Location<br>• Period of Outcome<br>• Population<br>• Matching criteria<br>• LOS (days)<br>• Notes/comments<br><br>The indicators collected during the study evaluation (Appendix 2) were:

		• First author, year<br>• Study type<br>• Sample size: Number cases and controls, Calculation a priori of the sample size<br>• Representativeness: Geographical, Demographical, Clinical<br>• Matching or controlling for confounders: Underlying disease, Infection site, Hospital and unit/ward, Follow-up<br>• Risk difference: Case fatality, LOS, Statistically significant outcome results
Risk of bias in individual studies	12	Every indicator was scored using the following scoring scale:<br>++ : Matches completely/is completely fulfilled<br>+ : Matches incompletely but sufficiently/is only partly but sufficiently fulfilled<br>- : Does not match or matches insufficiently/is insufficiently<br>c.b.e : Cannot be evaluated
Summary measures	13	Attributable case fatality and attributable LOS
Synthesis of results	14	The results were evaluated from ECDC experts.
Risk of bias across studies	15	Publication bias was not assessed, although some bias was expected due to the lower propensity to publish negative studies.<br>Studies were conducted in different countries and different settings, making their direct comparison challenging.<br>We scored each individual study for the criteria/indicators listed above (Appendix 2)
Additional analyses	16	NA
**RESULTS**		
Study selection	17	The study selection process for each antibiotic resistance-bacterium combination is shown in specific Figures in Appendix 1.
Study characteristics	18	These are presented in Appendix 1 (Tables) and Appendix 2 (excel form).
Risk of bias within studies	19	These were scored according to a set criteria/indicators and the results are presented in Appendix: 2
Results of individual studies	20	Tables in Appendix 1 and excel in Appendix 2.
Synthesis of results	21	The articles included in the review were further analysed and evaluated by ECDC experts and those considered of higher quality were used as a source for the estimate of the attributable case fatality and attributable LOS, see

		Appendix 3.<br>Appendix 3 provides an overview of the final decisions for the disease progression models.
Risk of bias across studies	22	Very often the studies were conducted in different countries and different settings, making their direct comparison challenging. To mitigate this heterogeneity, we used the scoring system of the indicators in order to better summarise the information. Majority of studies identified were observational and carry inherent biases related to their design.
Additional analysis	23	NA
**DISCUSSION**		
Summary of evidence	24	The synthetized results showed an increased mortality and LOS in infected patients. For some antibiotic resistance-bacterium combinations, the resistant infection showed increased mortality and LOS compared to the susceptible infections. There was a large variation across different types of infection, bacterial species and resistant phenotypes.
Limitations	25	The major limitation of the review includes the heterogeneity of the studies, in terms of study design, settings as well as type of patients and outcomes.
Conclusions	26	We found a wide difference between the studies identified in our search. Many are limited to specific healthcare services (e.g. ICUs) and/or performed on particular groups of patients (e.g. only children, only elderly) and/or with specific underlying conditions (e.g. immunodeficiency, organ transplant). Therefore, extracting valid case attributable fatality proportions and attributable LOS will be particularly challenging. Based on the heterogeneity of the studies detected, we advise to avoid pooling and averaging the results based on our categorizing criteria.<br>Therefore, we chose to score the evidence identified by a self-designed evaluation grid, see Appendix 2.
**FUNDING**		
Funding	27	Funding provided by ECDC

Results

The results of the reviews are presented separately for every bacteria and are antibiotic or antibiotic class specific.
Each chapter includes:

- a short description of the key words researched in every database;
- a scheme of the number of articles included and excluded during each step of the reviewing process;
- the articles selected as eligible for the review listed in separate tables based on the infection site and the comparison control group;
- some final considerations regarding the specific review.

1. Carbapenem resistant *Acinetobacter baumannii*

To research the attributable case fatality and the attributable LOS to carbapenem resistant *Acinetobacter baumannii* (CRAB) we performed 2 advanced searches on Pubmed using once the mesh term "Acinetobacter baumannii" AND all fields "carbapenem resistant", and the second time the mesh term "Acinetobacter baumannii" AND all fields "carbapenem resistance". Both times a filter for human studies was applied. Embase was searched for 'Acinetobacter baumannii' AND 'carbapenem resistant', and 'Acinetobacter baumannii AND 'carbapenem resistance'. We applied a filter to include only human studies. An advanced search on the Cochrane Library was performed using terms "Acinetobacter baumannii" AND "carbapenem". No language restrictions were made, and no further filters were applied. The flow diagram, presented in Figure 1 describes the steps in the literature review of carbapenem resistant *Acinetobacter baumannii* infections. The results obtained using the two different strings for PubMed and Embase were aggregated.
Once identified the studies reporting relevant data, these were grouped based on the site of infection.
All eligible studies reported a CRAB infected group and a control group. The latter could be represented by non-infected (NI) or carbapenem susceptible *Acinetobacter baumannii* (CSAB) infected patients.

Figure 1: Search for studies on carbapenem resistant *Acinetobacter baumannii*. Inclusion and exclusion diagram.

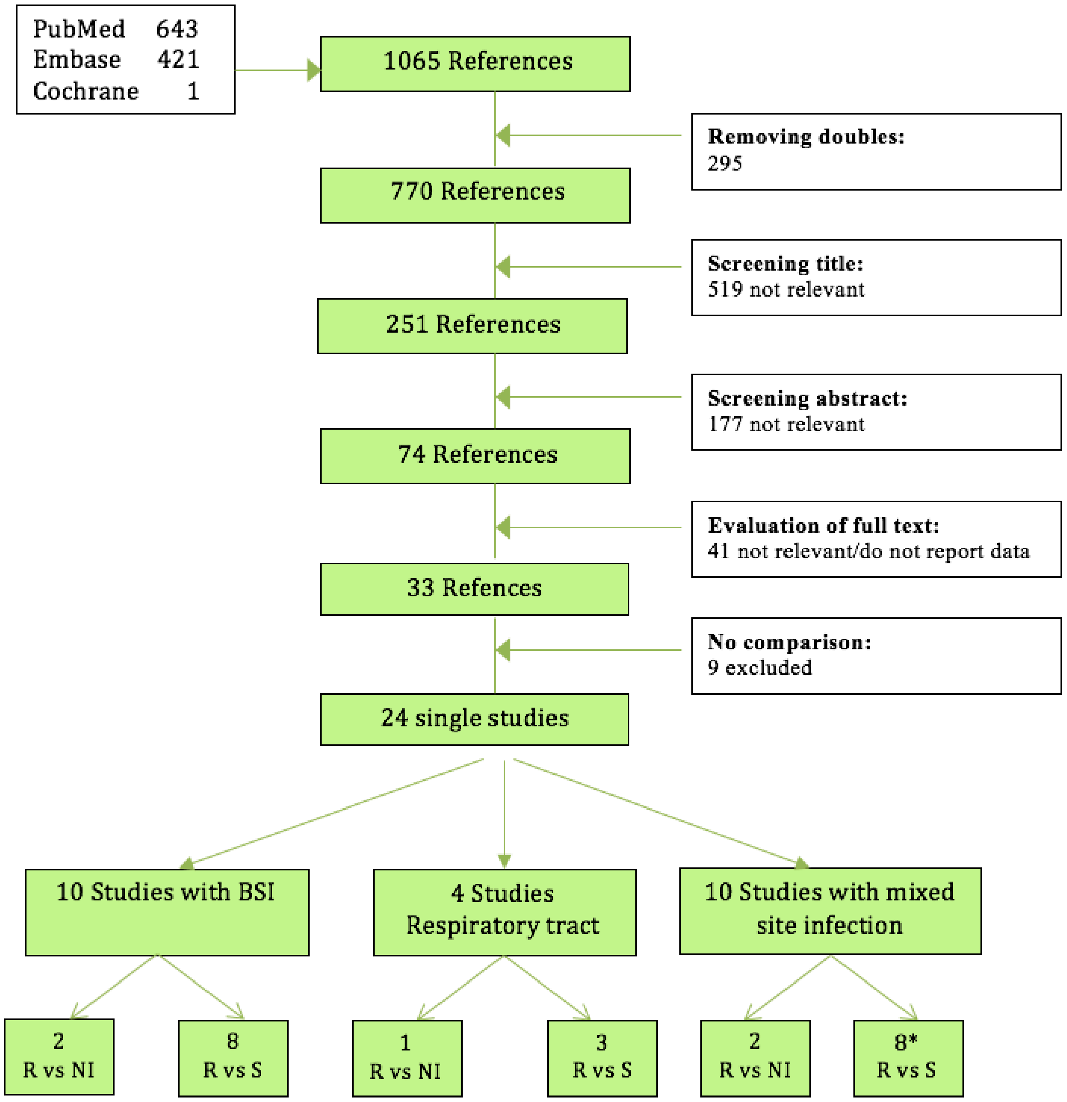

* One of these studies was a systematic review and meta-analysis.

The following tables include studies reporting results for BSI, respiratory tract and for mixed site of infection due to CRAB, based on the comparison group they used. All information is reported as was presented in the original papers.
Studies that include both a susceptible and a non-infected control group, were categorized in the latter group since we prioritized the non-infected comparator.

Table 1: Short extraction table of original articles on BSI mortality of CRAB infected patients vs non infected patients

Nr	Author	Study Period	Study type	Outcome	Location	Period of outcome	Population	Matching criteria	LOS (days)	Notes
1	Kim 2014[1]	01/05-12/11	Retrospective case-control study	Mort. rate cases=94.7%<br>Mort. rate controls=8.3%	Korea	hospitaliz	19 cases<br>38 controls	undelying condition	median (range)<br>cases=35(30–47)<br>controls=29(26.75–34)	all patients were hematopoietic stem cell transplantation recipients
2	Thatrimontrichai 2013[2]	01/91-12/10	Case-case-control study	Mort. rate resist=42.9%<br>Mort. rate sensit=13.2%<br>Mort. rate controls=6.8%<br>OR RvsS=5; 95%CI(1.2-20-4)<br>OR RvsNI=10.2; 95%CI(2.1-50)	Thailand	hospitaliz	14 resistant<br>38 sensitive<br>44 controls	age, ICU	median(range)<br>resist=34(2-206)<br>sensitive=53.5(3-188)<br>controls=24.5(1-102)	patients were neonates in ICU

Table 2: Short extraction table of original articles on BSI mortality of CRAB infected patients vs CSAB infected patients

Nr	Author	Study Period	Study type	Outcome	Location	Period of outcome	Population	Matching criteria	LOS (days)	Notes
1	Deris 2011[3]	no info	Cross sectional case-control study	Mort. rate cases=64.3%<br>Mort. rate controls=40.5%<br>OR=0.38; 95%CI(0.11-1.36)<br>*Mort. rate cases=42.9%<br>*Mort. rate controls=24.3%<br>OR=2.33; 95%CI(0.64-8.65)	Malaysia	hospitaliz	15 cases<br>41 controls	none	median (IQR)<br>cases=32.3(16.4)<br>controls=32.8(25.0)	*attributable mortality; within 72h from isolation
2	Esterly 2011[4]	01/05-12/08	Retrospective cohort study	Mort. rate cases=56.8%<br>Mort. rate controls=23.8%<br>Model1 OR=0.58; 95%CI (0.037–9.22)<br>Model2 OR=0.73; 95%CI(0.14–3.84)	Chicago USA	hospitaliz	37 cases<br>42 controls	none	Prior isolation median (IQR)<br>cases=16(1-30)<br>controls=0(0-9)	

3	Huang 2012[5]	06/02-12/07	Retrospective cohort study	Mort. rate cases=35.5%<br>Mort. rate controls=20.7%<br>OR= 1.03 ; 95%CI(0.48-2.20)	Taiwan	14 days	62 cases<br>164 controls	none	Prior isolation mean±SD cases=35.9±33.6 controls=28.2±44	
4	Jamulitrat 2009[6]		Cohort study	Mort. rate cases=52.2%<br>Mort. rate controls=19.9%<br>aHR=1.7; 95%CI(0.9-2.9)	Thailand	hospitaliz	67 cases<br>131 controls	none	median ±IQR cases=37±32 controls=27±37	
5	Kim 2012 [7]	06/07-06/10	Retrospective study	Mort. rate cases=49.1%<br>Mort. rate controls=9.5%<br>OR=7.29; 95%CI(1.57-33.8)	Korea	14 days	53 cases<br>42 controls	none	Prior isolation mean±SD cases=30.0±33.3 controls=30.4±68.3	the study differentiates those who received appropriate therapy from those who did not
6	Kumar 2014[8]	01/10-12/12	Retrospective chart review	Mort. rate cases=27.3%<br>Mort. rate controls=9.4%	India	hospitaliz	33 cases<br>32 controls	age, ICU	no data	patients were neonates in ICU
7	Routsi 2010[9]	09/04-01/06	Prospective observational study	Mort. rate cases=43.3%<br>Mort. rate controls=46.9%	Greece	hospitaliz	30 cases<br>66 controls	none	median (IQR) cases=33(18.8-55.8) controls=28.5(14.5-51.0)	
8	Tal-Jasper 2016[10]	01/07-12/12	Retrospective cohort study	a) Mort. rate cases=70.5%<br>Mort. rate controls=45.1%<br>b) Mort. rate cases=66.2%<br>Mort. rate controls=44.4%<br>c)Mort. rate cases=73.6%<br>Mort. rate controls=53.3%<br>OR 90days=0.6, 95%CI (0.1–2.3)	Israel	a) hospitaliz<br>b)30 days<br>c) 90 days	149 cases<br>91 controls	none	median (IQR) cases=21(10.8-39.5) controls=10(7-20.5)	

Table 3: Short extraction table of original articles on respiratory tract infection mortality of CRAB infected patients vs sensitive and vs non infected patients

Nr	Author	Study Period	Study type	Outcome	Location	Period of outcome	Population	Matching criteria	LOS (days)	Notes
1	Thatrimontrichai 2016[11]	01/09-12/14	Case-case-control study	Mort. rate resist=15.9%<br>Mort. sensit=7.7%<br>Mort. control=0%	Thailand	hospitaliz	63 resistant<br>13 sensitive<br>25 controls	age, ICU	median(range)<br>resist=29(8-93)<br>senstiv=20(8-31)<br>controls=22(8-68)	patients were neonates in ICU

Table 4: Short extraction table of original articles on respiratory tract infection mortality of CRAB infected patients vs sensitive infected patients

Nr	Author	Study Period	Study type	Outcome	Location	Period of outcome	Population	Matching criteria	LOS (days)	Notes
1	Chang 2011[12]	01/05-12/07	Retrospective observational study	Mort. rate cases=61.3%<br>Mort. rate controls=46.0%	Taiwan	hospitaliz	93 cases<br>87 controls	none	after VAP onset<br>median±SD<br>cases=23.1±24.3<br>controls=26.7±24.3	all patients were critically ill
2	Garnacho-Montero 2003[13]	01/97-06/01	Prospective cohort study	Mort. rate cases=61.9%<br>Mort. rate controls=64.2%<br>*VAP related mortality<br>Mort. rate cases=38%<br>Mort. rate controls=35.7%	Spain	hospitaliz	21 cases<br>14 controls	underlying condition	mean±SD<br>cases=45.2±30.7<br>controls=53.9±50	cases treated with colistin, controls with imipenem-cilastatin<br>*occurring during treatment, when signs of pneumonia remained and due to septic shock
3	Zheng 2013[14]	01/06-12/11	Retrospective study	Mort. rate cases=45.6%<br>Mort. rate controls=29.9%	China	28 days	97 cases<br>145 controls	underlying condition	before pneu<br>mean±SD<br>cases=17.7±6.6<br>controls=18±6.2	

Table 5 and Table 6 present the results of studies that include patients with different sites of infection. Almost all the articles specify the number of cases for the different infection sites but they do not report different mortalities based on the infection site. When it was possible the results are presented differentiating the mortality for each site of infection.

Table 5: Short extraction table of original articles on mortality of CRAB infected patients vs non infected patients for different infection sites

Nr	Author	Study Period	Study type	Outcome	Location	Period of outcome	Population	Matching criteria	LOS (days)	Notes
1	Henig 2015[15]	01/07-12/12	Matched case-control study	Mort. rate cases=73%<br>Mort. rate controls=55%<br>aHR=2.33; 95%CI(2.08–2.6)	Israel	hospitaliz	1190 cases<br>1190 controls	age, ward, season, Charlson score, LOS	matching criteria<br>median (range)<br>cases=18 (0–97)<br>controls=17 (0–96)	the study considers also 241 cases and controls with BSI but gives not info on their mortality
2	Nazer 2015[16]	01/10-12/13	Matched case-control study	Mort. rate cases=73%<br>Mort. rate controls=61.2%	Jordan	in ICU stay	142 cases<br>232 controls	age, gender, APACHE II, type of malignancy, mechanical ventilation	median (IQR)<br>cases=12(6-13)<br>controls=3(1.7)	all patients were critically ill with cancer and in ICU

Table 6: Short extraction table of original articles on mortality of CRAB infected patients vs CSAB infected patients for different infection sites

Nr	Author	Study Period	Study type	Outcome	Location	Period of outcome	Population	Matching criteria	LOS (days)	Notes
1	Aydemir 2012[17]	01/05-12/06	Retrospective case-control study	Mort. rate cases=61.8%<br>Mort. rate controls=52.7%	Turkey	hospitaliz	110 cases<br>55 controls	none	after isolation<br>mean±SD<br>cases=18.1±14.0<br>controls=15.7±12.3	
2	de Gouvea 2012[18]	01/02-01/09	Retrospective study	Mort. rate cases=44.4%<br>Mort. rate controls=29.0%<br>Inf. related. OR=0.73; 95%CI(0.12 – 4.47)	Brasil	30 days	18 cases<br>31 controls	undelying condition	no data	all patients had solid organ transplant

				Overall mort. OR=1,93; 95%CI(0.48 – 7.85)						
3	del Mar 2005[19]	10/01-08/02	Case-control study	Mort. rate cases=43.3% Mort. rate controls=19% *Related mortality=30%	Spain	30 days	30 cases 31 controls	same period of isolation	attributable LOS=14 days	cases were multi resistant * when infection was established, by clinical criteria, as the primary cause of death
4	Lautenbach 2009[20]	01/89-12/04	Prospective cohort study	Mort. rate cases=18% Mort. rate controls=21.2% RR=0.85; 95% CI(0.52–1.39)	New Jersey, USA	30 days	89 cases 297 controls	none	median(range) cases=21(14-29) controls=16(12-18)	
5	Lemos 2014 a[21]	04/06-04/10	Prospective, multicentre cohort study	Mort. rate cases=40% Mort. rate controls=12.2% aHR=1.45; 95%CI(0.74-2.87)	New York, USA	30 days	104 cases 61 controls	ICU	adjusted mean cases=19.3 controls=16.2	patients were in ICU
6	Lemos 2014 b[22]	up to 05/13	Systematic review and meta-analysis	crude OR=2.22; 95% CI=1.66-2.98	World					
7	Sheng 2010[23]	05/04-11/06	Multicentre retrospective study	a) Mort. rate cases=13.3% Mort. rate controls=20% b) Mort. rate cases=33% Mort. rate controls=17.5%	Taiwan	hospitaliz	30 cases 30 controls	none	Median (range) a)cases=41 (5–282) controls=29 (2–227) b) cases=37 (1–218) controls=23 (1–151)	a) colonized patients b) infected patients
8	The Brooklyn Antibiotic Resistance Task Force, 2002[24]	3 months 1999	Matched case-control study	a) Mort. rate cases=34% Mort. rate controls=27% b) Mort. rate cases=20% Mort. rate controls=30%	USA	hospitaliz	a) 44 cases 33 controls b) 10 cases 10 controls	age, site of infect, underlying diseases, operative procedure, type of infection	after infection median cases=31.5 controls=13	a) all cases and controls b) 10 matched pairs

Some of the detected studies reported pooled results for gram negative bacteria, or for *Acinetobacter baumannii* and *Pseudomonas aeruginosa*. These were not included.
We detected different studies that reported treatment options for the resistant infections, comparing two or more therapeutic strategies. All these studies included mortality data of CRAB infected patients, but since they used just cases infected with CRAB and no control group, as defined by our eligibility criteria, these studies were not included in the results of this review.
All studies were performed in healthcare settings and considered HAI, though some of them report cases of infection that originated in the community or that had the onset in the community, but none of them reported only CAI cases.

2. Colistin resistant Acinetobacter baumannii

To research the attributable case fatality and the attributable LOS to colistin resistant *Acinetobacter baumannii* (CoRAB) we performed 2 advanced searches on Pubmed using once the mesh term "Acinetobacter baumannii" AND all fields "colistin resistant", and the second time the mesh term "Acinetobacter baumannii" AND all fields "colistin resistance". Embase was searched for 'Acinetobacter baumannii' AND 'colistin resistance', and for 'Acinetobacter baumannii' AND 'colistin resistant'. An advanced search on the Cochrane Library was performed using terms "Acinetobacter baumannii" AND "colistin". No language restrictions were made, and no filters were applied.

The flow diagram, presented in Figure 2 describes the steps in the literature review of colistin resistant *Acinetobacter baumannii* infections. The results obtained using the two different key words in PubMed and Embase were aggregated.

Figure 2: Search for studies on colistin resistant *Acinetobacter baumannii*. Inclusion and exclusion diagram.

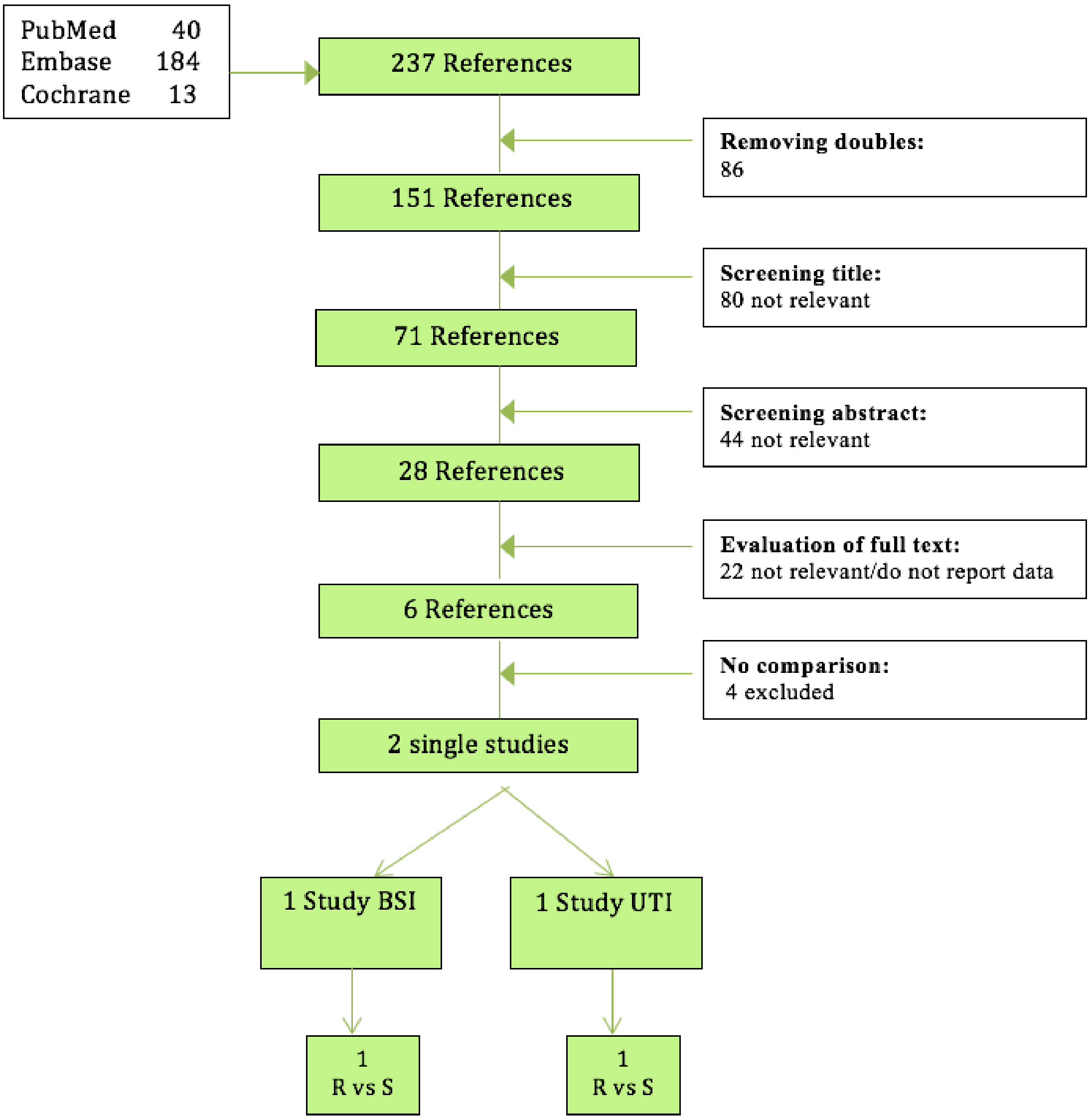

The following table 7 includes studies reporting mortality data for CoRAB. All information is reported as was presented in the original papers.

Table 7: Short extraction table of original articles on BSI mortality of CoRAN* infected patients vs CoSAB infected patients

Nr	Author	Study Period	Study type	Outcome	Location	Period of outcome	Population	LOS (days)	Matching	Notes
1	Wang 2015[25]	1998-2008	Retrospective study	a) Mort. rate cases=10.3%<br>Mort. rate controls=10.3%<br>b) Mort. rate cases=15.5%<br>Mort. rate controls=15%	Taiwan	a) 14 days<br>b) 28 days	58 cases<br>213 controls	prior infection<br>median (IQR)<br>cases=56 (32-101)<br>controls=37 (22-65)	none	

*The study in table 7 deals with *A. nosocomialis* instead of *A. baumanni* but we decided to include it in the review as eligible for the purposes of the project.

Table 8: Short extraction table of original articles on UTI mortality of CoRAB infected patients vs CoSAB infected patients

Nr	Author	Study Period	Study type	Outcome	Location	Period of outcome	Population	LOS (days)	Matching	Notes
1	Taneja 2011[26]	02/07-06/08	Prospective cohort study	Mort. rate both=0%	India	hospitaliz	8 cases<br>42 controls	No data	all bacteria were MDR	patients with complicated urinary tract infection

We identified 4 additional studies[27-30] investigating the mortality due to CoRAB, but they did not use a comparison group. In these studies the mortality for the cases ranged from 0%-70%.

We did not apply any specific filter to the literature research because we wanted to be as broad as possible in including all the papers that had as subjects *Acinetobacter baumannii* and colistin resistance. The research produced numerous results, but not all of them were eligible for the purposes of this review.
Many detected papers reported studies on the efficacy of colistin on multi-drug resistant Acinetobacte baumannii strains, where multidrug resistance was defined as resistance to all antibiotics except for colistin.
The colistin resistant *Acinetobacter baumannii* infections are a relative new research topic, and it seems that no structured studies were conducted yet to investigate the burden of these infections in terms of attributable mortality or attributable length of stay in hospital.

3. Multidrug resistant Acinetobacter baumannii

For the purposes of this project a multidrug resistant isolate is defined as resistant to at least three classes of antimicrobial agents, all penicillins and cephalosporins (including inhibitor combinations), fluroquinolones, and aminoglycosides. Here are excluded those resistant to carbapenem and/or to colistin. If an isolate is multidrug resistant and also resistant to carbapenem and/or colistin, it will be classified as resistant to the latter antibiotic.

To research the attributable case fatality and the attributable LOS to multidrug resistant *Acinetobacter baumannii* (MDRAB) we performed 3 advanced searches on Pubmed using once the mesh term "Acinetobacter baumannii" AND all fields "multidrug resistant" AND all fields "mortality", the second time the mesh term "Acinetobacter baumannii" AND all fields "multidrug resistance" AND all fields "mortality", and the third time the mesh term "Acinetobacter baumannii" AND all fields "MDR" AND all fields "mortality". All times a filter for human studies was applied. Embase was searched for 'Acinetobacter baumannii' AND 'multidrug resistant' AND 'mortality', and 'Acinetobacter baumannii AND 'multidrug resistance' AND 'mortality'. We applied a filter to include only human studies. Two advanced searches were also performed on the Cochrane Library, using once the terms "Acinetobacter baumannii" AND "multidrug resistant", and the second time the terms "Acinetobacter baumannii" AND "multidrug resistance". No language restrictions were made, and no further filters were applied.

The flow diagram, presented in Figure 3 describes the steps in the literature review of Multidrug resistant *Acinetobacter baumannii* infections. The results obtained using the different strings for PubMed, Embase and the Cochrane Library were aggregated.

Once identified the studies reporting relevant data, these were grouped based on the site of infection.
All eligible studies reported a MDRAB infected group and a control group. The latter could represent patients non infected (NI) or infected with susceptible (S) *A. baumannii* infections.

Figure 3: Search for studies on multidrug resistant *Acinetobacter baumannii*. Inclusion and exclusion diagram.

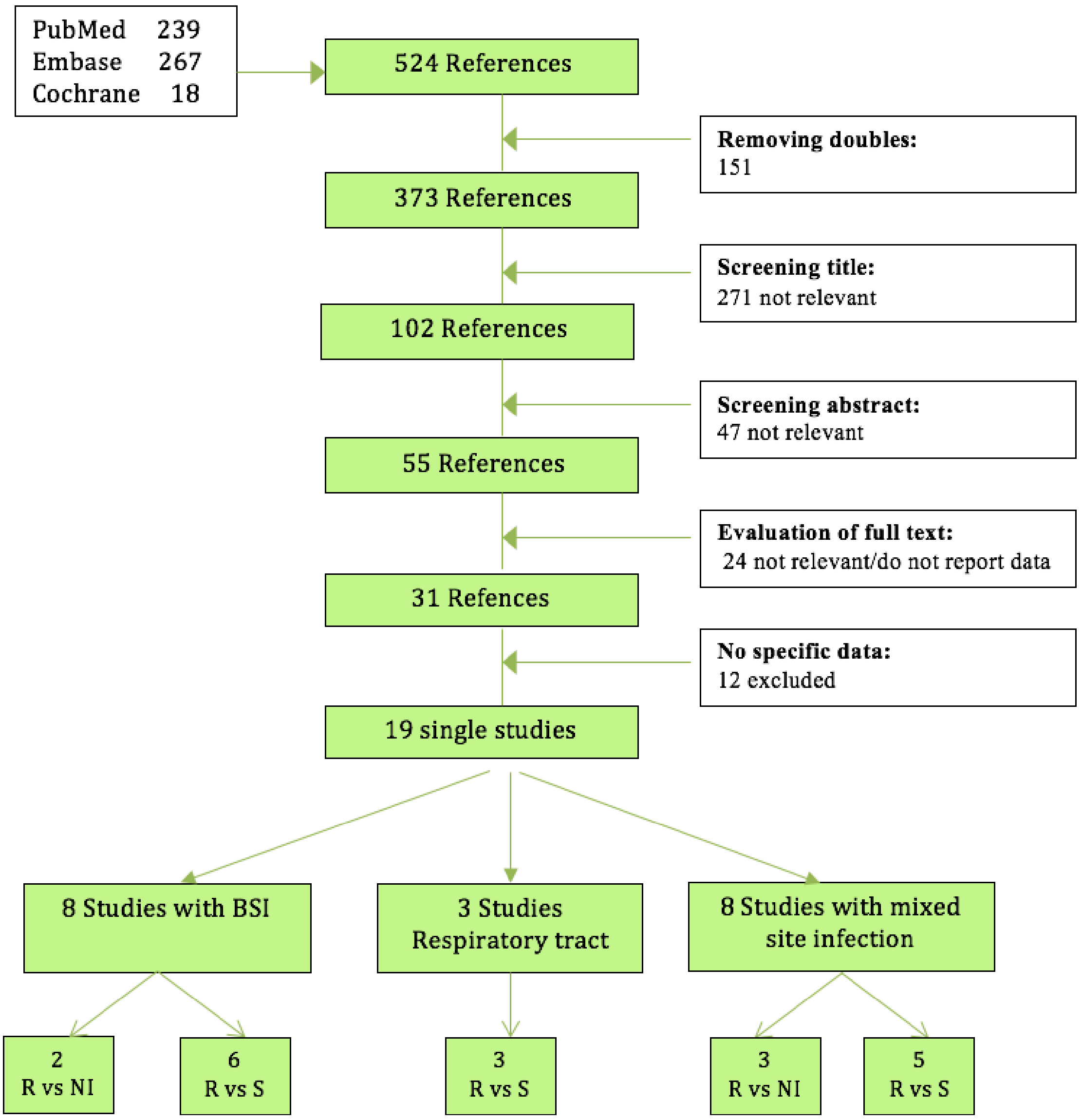

The following tables include studies reporting results for BSI, respiratory tract and for mixed site of infection due to MDRAB, based on the comparison group they used. All information is reported as was presented in the original articles.

Table 9: Short extraction table of original articles on BSI mortality of MDRAB infected patients vs non infected patients

Nr	Author	Study Period	Study type	Outcome	Location	Period of outcome	Population	Matching criteria	LOS (days)	Notes
1	Al Jarousha 2009[31]	02/04-01/05	Prospective case-control study	Mort. cases=37.5%<br>Mort. controls=12%	Palestine	hospitaliz	40 cases<br>100 controls	age, birth weight	no specific data	patients were in neonatal ICU
2	Gulen 2015[32]	01/07-12/10	Retrospective cohort study	a) Mort. cases=30%<br>b) Mort. cases=52.4%<br>c)Mort. Cases=53.7%<br>Attribut. mort.=24.4%	Turkey	a) 14 days<br>b) 28 days<br>c) hospitaliz	41 cases<br>45 controls*	BSI	before BSI<br>mean±SD<br>cases=25.49±21.47<br>controls=22.80±19.28	*patients had other gram-negative infections

Table 10: Short extraction table of original articles on BSI mortality of MDRAB infected patients vs MDSAB infected patients

Nr	Author	Study Period	Study type	Outcome	Location	Period of outcome	Population	Matching criteria	LOS (days)	Notes
1	Anunnatsiri 2011[33]	01/05-12/07	Retrospective study	Mort. cases=91.7%<br>Mort. controls=48%<br>OR=11.92; 95%CI(2.30-61.83)	Thailand	hospitaliz	24 cases<br>25 controls	none	before BSI<br>median (range)<br>cases=21.5(4-161)<br>controls=14(2-86)	
2	Fitzpatrick 2015[34]	06/05-10/12	Retrospective cohort study	a) Mort. cases=36%<br>Mort. controls=8%<br>b) Mort. cases=44%<br>Mort. controls=8%	Chicago, USA	a) 14 days<br>b) 30 days	92 cases<br>24 controls	none	Post culture<br>median(range)<br>cases=11.5(2-69)<br>controls=6(2-38)	
3	Guo 2016[35]	06/12-06/15	Retrospective study	Mort. cases=44.8%<br>Mort. controls=4.3%<br>OR=26.3; 95%CI(3.0-231.4)	China	30 days	64 cases<br>23 controls	none	in ICU<br>mean<br>cases=51<br>controls=5	
4	Lee 2007[36]	04/96-08/01	Retrospective matched cohort study	a) Mort. cases=47.8%<br>Mort. controls=39.1%<br>b) Mort. cases=34.8%	Taiwan	hospitaliz	46 cases<br>46 controls	sex, age, severity of underlying	mean±SD<br>cases=54.2±42.8<br>controls=34.1±30.5	a) crude mortality<br>b) sepsis related mortality

				Mort. controls=13.0%				and acute illness		
5	Lee 2010[37]	01/00-12/05	Retrospective study	Mort. cases=32%<br>Mort. controls=8,3%<br>OR=7.46;<br>95%CI(3.78-14.70)	Taiwan	30 days	147 cases<br>144 controls	none	no specific data	the study investigated the difference between gen. sp.2 vs gen. sp. 3<br>data were extrapolated
6	Smolyako 2003[38]	01/05-12/05	Prospective study	Mort. cases=29.4%<br>Mort. controls=11.6%<br>OR=4	Israel	hospitaliz	51 cases<br>43 controls	none	mean±SD<br>cases=37.7±30<br>controls=24.3±18.3	

Table 11: Short extraction table of original articles on respiratory tract infection mortality of MDRAB infected patients vs MDSAB infected patients

Nr	Author	Study Period	Study type	Outcome	Location	Period of outcome	Population	Matching criteria	LOS (days)	Notes
1	Cai 2012[39]	01/09-08/11	Prospective study	Mort. cases=18.26%<br>Mort. controls=4.44%	China	hospitaliz	115 cases<br>45 controls	ward	in ICU<br>mean±SD<br>cases=17.39±7.05<br>controls=14.43±3.92	patients were children with pneumonia
2	Inchai 2015[40]	01/05-12/11	Retrospective cohort study	a)Mort. cases=31.9%<br>Mort. controls=21.2%<br>b)Mort. cases=44.4%<br>Mort. controls=27.3%<br>HR=1.03;<br>95%CI(0.44–2.45)	Thailand	a) 30 days<br>b) hospitaliz	72 cases<br>33 controls	ICU	no data	patients were in ICU
3	Park 2006[41]	01/02-11/04	Retrospective study	Mort. cases=58.8%<br>Mort. controls=40%	Korea	hospitaliz	17 cases<br>30 controls	no data	mean±SD<br>cases=42.8±24.0<br>controls=66.2±56.1	

Table 12 and Table 13 present results of studies that include patients with different sites of infection. Almost all the articles specify the number of cases for the different infection sites but they do not report different mortalities based on the infection site. When it was possible the results are presented differentiating the mortality for each site of infection.

Table 12: Short extraction table of original articles on mortality of MDRAB infected patients vs non infected patients for different infection sites

Nr	Author	Study Period	Study type	Outcome	Location	Period of outcome	Population	Matching criteria	LOS (days)	Notes
1	Abbo 2007[42]	01/01-06/01	Retrospective matched cohort study	Mort. cases=36%<br>Mort. controls=21%<br>OR=2.21, 95%CI(1.17–4.16)	Israel	hospitaliz	118 cases<br>118 controls	ward, month of hosp, LOS before culture	median<br>cases=17<br>controls=11	
2	Fukuta 2013[43]	01/08-12/11	Case-control study	Mort. cases=41.9%<br>Mort. controls=29%	Pittsburgh, USA	90 days	31 cases<br>62 controls	underlying condition	median(IQR)<br>cases=28 (9-59)<br>controls=10(5-18)	patients had cancer
3	Lee 2016[44]	01/12-12/14	Retrospective propensity-matched cohort study	Mort. cases=32%<br>Mort. controls=36.8%<br>OR=3.64; 95%CI(1.80-7.37)	Korea	hospitaliz	122 cases<br>122 controls	propensity score	mean±SD<br>cases=40.3±40.6<br>controls=24.6±28	presence of MDRAB isolate without signs or symptoms

Table 13: Short extraction table of original articles on mortality of MDRAB infected patients vs MDSAB infected patients for different infection sites

Nr	Author	Study Period	Study type	Outcome	Location	Period of outcome	Population	Matching criteria	LOS (days)	Notes
1	Brahmi 2007[45]	01/04-12/05	Prospective study	Mort. cases=67.5%<br>Mort. controls=46.7%	Tunisia	hospitaliz	29 cases<br>34 controls	ward	in ICU<br>mean±SD<br>cases=27±17.8<br>controls=32.3±23.9	patients were in ICU
2	Daniels 2008[46]	06/03-06/06	Retrospective propensity-matched cohort study	a)Mort. cases=15.8%<br>Mort. controls=9.5%<br>b) Mort. cases=14.3%<br>Mort. controls=9.5%	Tennessee, USA	28 days	a)146 cases<br>42 controls)<br>b) 42 cases<br>42 controls	propensity score	median (IQR)<br>cases=32.5(22-51)<br>controls=26.5(16-43)	patients were in ICU<br>a) all patients<br>b) matched pairs

3	Lemos 2011[47]	04/06-04/10	Prospective, observational multicentre study	a) Mort. cases=31%<br>Mort. controls=9%<br>b) Mort. cases=41%<br>Mort. controls=9%<br>b) RR=1,219; 95%CI(0,923–1,611)	Colombia	14 days	103 cases<br>62 controls	ICU	median(IQR)<br>pre ICU<br>cases=4(1.2-18)<br>controls=5(1.5-22.3)<br>in ICU<br>cases=15(13-60)<br>controls=13(8-44)	patients were in ICU
4	Pierri 2015[48]	01/09-12/11	Retrospective matched cohort study	Mort. cases=57%<br>Mort. controls=46%	Italy	hospitaliz	14 cases<br>26 controls	propensity score	mean±SD<br>cases=36±20<br>controls=25±18	patients underwent cardiac surgery
5	Zilberberg 2016[49]	01/09-12/13	Retrospective cohort study	Mort. cases=23.7%<br>Mort. controls=12.7%	USA	hospitaliz	1171 cases<br>252 controls	underlying condition	no data	patients had pneumonia or sepsis

The vast majority of papers containing information on mortality did not report a comparison group.
Many studies reported treatment options for the resistant infections, but since they used just cases infected with MRDAB and no control group, as defined by our eligibility criteria, these studies were not included.
All studies were performed in healthcare settings though some of them report cases of infection that originated in the community, but none of them treated only patients with infection onset in the community.
The studies that included patients with MDRAB which were also resistant to carbapenems were also not included.

We did not perform a literature review for third-generation cephalosporin resistant *Acinetobacter baumannii* (3GCRAB), but we decided to report here the results of a multicentre European study[50] that investigated the mortality of patients infected with 3GCRAB. The authors compared patients with resistant and susceptible *A. baumannii* to cefetizime (3GCRAB vs 3GCSAB), for BSI and respiratory tract infections. Table 14 reports the characteristics and results of this study.

Table 14: Short extraction table of the original article on mortality of 3GCRAB infected patients vs 3GCSAB infected patients for BSI and respiratory tract infections

Nr	Author	Study Period	Study type	Outcome	Location	Period of outcome	Population	Matching criteria	LOS (days)	Notes
1	Lambert 2011[50]	01/05-12/08	Prospective cohort study	a) Mort. cases=43%<br>Mort. controls=55%<br>aHR=0.8; 95%CI(0.3-2.1)<br>b) Mort. cases=38%<br>Mort. controls=40%<br>aHR=1.0; 95%CI(0.6-1.8)	Europe	ICU hospitalization	a)31 cases<br>11 controls<br>b)130 cases<br>43 controls	ICU	From infection to discharge<br>median (IQR)<br>a)cases= 20 (7–38)<br>controls=9 (2-13)<br>b)cases= 14 (11–26)<br>controls= 25(14-40)	patients were in ICU<br>a)patients with BSI<br>b)patients with pneumonia

4. Carbapenem resistant *Escherichia coli*

To research the attributable case fatality and the attributable LOS to carbapenem resistant *Escherichia coli* (CREC) we performed an advanced search on Pubmed using “Escherichia coli” [MeSH Terms] AND “carbapenem resistance” all fields, together with the string “Escherichia coli” [MeSH Terms] AND “carbapenem resistant” all fields. No filters were applied. Embase was searched for ‘Escherichia coli’ AND ‘carbapenem resistance’, and for ‘Escherichia coli’ AND ‘carbapenem resistant’. No filters were applied. An advanced search on the Cochrane Library was performed using terms “Escherichia coli” AND “carbapenem”. Studies on animals, cells and tissues, as well as case report studies were excluded from the results. No language restrictions were made to any database.

The following flow diagram describes the steps in the literature review of carbapenem resistant *Escherichia coli* infections.

Figure 4: Search for studies on carbapenem resistant *Escherichia coli*. Inclusion and exclusion diagram.

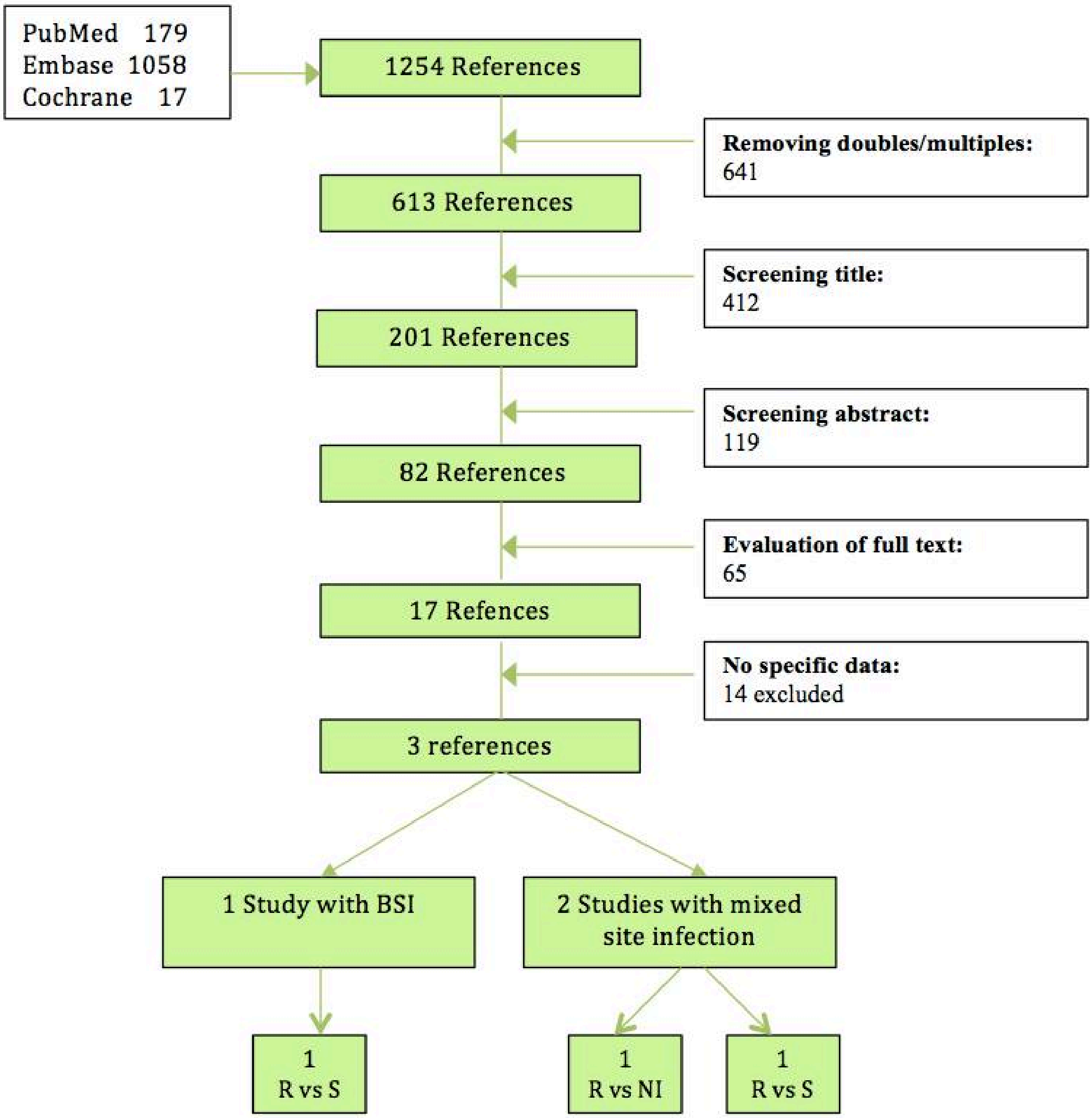

The following tables include studies reporting results for BSI and mixed site infection, based on the comparison group they used, carbapenem susceptible *Escherichia coli* (CSEC) infected patients or non-infected (NI). All information is reported as was presented in the original papers

Table 15: Short extraction table of original articles on BSI mortality of CREC infected patients vs CSEC infected patients

Nr	Author	Study Period	Study type	Outcome	Location	Period of outcome	Population	Matching criteria	LOS (days)	Notes
1	Chang 2011[51]	01/06-12/08	Matched case-control study	a)Mort. rate cases=47.06% Mort. rate controls=38.24% b)Mort. rate cases=70.59% Mort. rate controls=47.06% c)Mort. rate cases=94.12% Mort. rate controls=50%	Taiwan	a) ≤14 days b) ≤ 28 days c) hospitaliz	17 cases 34 controls	gender, age, year of hosp, length stay before isolation	mean±SD (min-max) cases=68.4± 56.4 (7-161) controls=35.8±34.5 (4-141)	Cases are non-susceptible, MIC non reported

Table 16 and Table 17 present the results of studies that include patients with different sites of infection. Almost all the articles specify the number of cases for the different infection sites but they do not report different mortalities based on the infection site.

Table 16: Short extraction table of original articles on mortality of CREC infected patients vs non infected patients for different infection sites

Nr	Author	Study Period	Study type	Outcome	Location	Period of outcome	Population	LOS (days)	Matching	Notes
1	Epstein 2014[52]	01/13-12/13	Case-control study	Mort. rate cases=25%* Mort. rate controls=0%	Illinois, USA	hospitaliz	8 cases 27 controls	median cases=8.5 controls=25.0 mean(SD)[range] cases=13.3 (12.0) [1-37]	patients from the same ward	patients fro the same ward * death not related to

								controls=30.2 (13.2) [14-65]		CREC

* deaths not related to CREC (author's statement)

Table 17: Short extraction table of original articles on mortality of CREC infected patients vs CSEC infected patients for different infection sites

Nr	Author	Study Period	Study type	Outcome	Location	Period of outcome	Population	Matching	LOS (days)	Notes
1	Ahn 2014[53]	10/06 - 12/10	Matched case-controls study	Mort. rate cases=14.8%<br>Mort. rate controls=10.2%	Korea	28 days	57 cases<br>114 controls	Site of infection	mean±SD<br>cases=26.6±37.9<br>controls=13.1±41.3	

Table 18: Short extraction table of original articles on mortality of CREC infected patients without a control group

Nr	Study	Study Period	Study type	Outcome	Location	Period of outcome	Population	Matchin g	LOS (days)	Notes
1	Balkan 2014[54]	02/11-03/13	Retrospective nested case–control study	Mort. rate cases=50%	Turkey	hospitaliz	8 cases	none	No data	comp. with klebsiella and enteorbacter
2	Poirel 2014[55]	03/12-09/12	Observational study	Mort. rate cases=33.3%	Bulgaria	hospitaliz	12 cases	none	No data	study on Enterobacteriaceae
3	Porwal 2014[56]	05/11-05/12	Retrospective study	Mort. rate cases=38.5%	Chenmai, India	30 days	13 cases	none	No data	study on Gram-
4	Shukla 2013[57]		Retrospective study	Mort. rate cases= 6%	Mumbai, India	hospitaliz	17 cases	none	No data	study on Gram-

The studies included in table 18 are not eligible studies since they do not have a control group. They are part of this report as further possible source of information on the mortality of carbapenem resistant *Escherichia coli* infections, since the number of eligible studies detected with this literature review was limited. These studies included investigated the mortality of Gram negative or Enterobacteriacea, only comparing the different bacteria on each group.

5. Colistin resistant *Escherichia coli*

Escherichia coli resistance to colistin was attributed to chromosomal mutations until November 2015, when Liu et al.[58] published a research, from China, showing that the resistance, in animals and humans, was due to the mcr-1 gene, which can be horizontally transmitted through plasmids. After the publication the gene was detected in other countries, both in animals and humans.

To research the attributable case fatality and the attributable LOS to colistin resistant *Escherichia coli* (CoREC) we performed 2 advanced searches on Pubmed using once the mesh term “Escherichia coli” AND all fields “colistin resistant”, and the second time the mesh term “Escherichia coli” AND all fields “colistin resistance”. Embase was searched for ‘Escherichia coli’ AND ‘colistin resistance’, and for ‘Escherichia coli’ AND ‘colistin resistant’. An advanced search on the Cochrane Library was performed using terms “Escherichia coli” AND “colistin”. No language restrictions were made, and no filters were applied.

The flow diagram, presented in Figure 5 describes the steps in the literature review of colistin resistant *Escherichia coli* infections. The results obtained using the two different key words in PubMed and Embase were aggregated.

Figure 5: Search for studies on colistin resistant *Escherichia coli*. Inclusion and exclusion diagram.

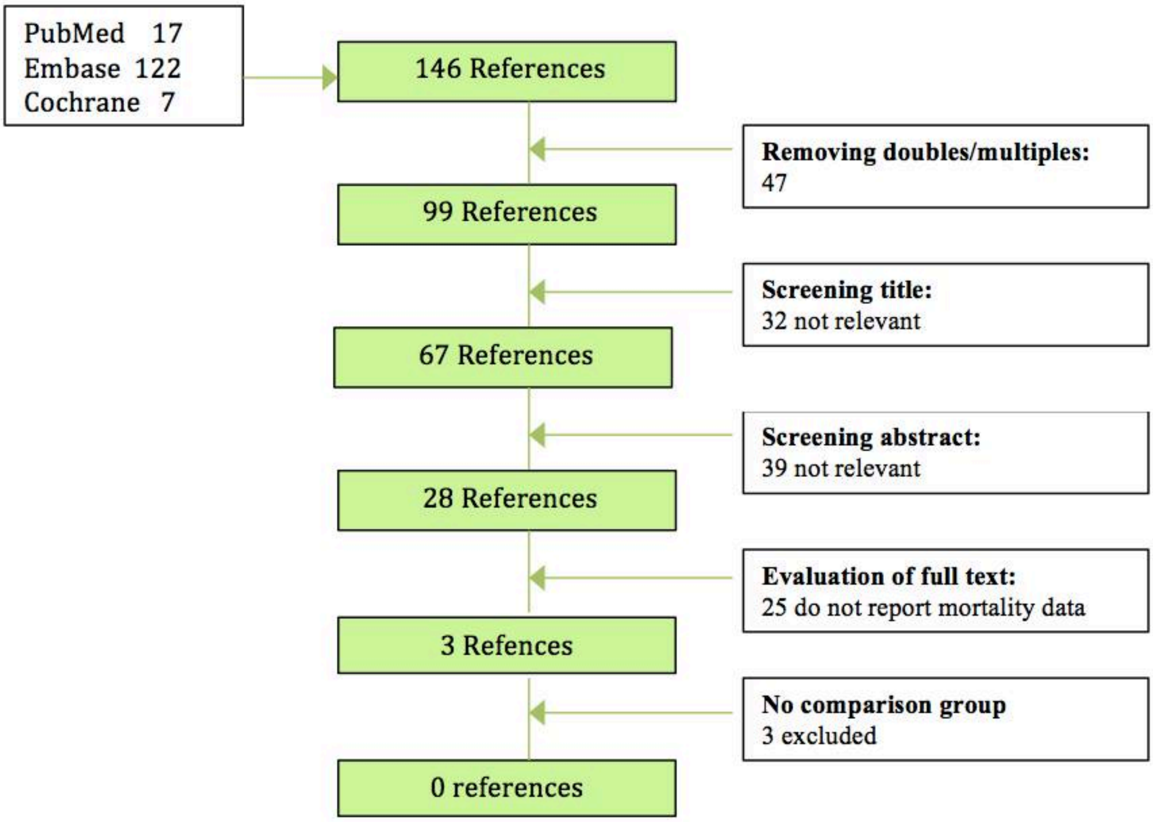

The following table includes 3 studies reporting mortality data for CoREC infected patients without a comparison group. Though these studies do not fulfil our eligibility criteria, they were reported since no other evidence on the mortality due CoREC was available. All information is reported as was presented in the original papers.

Table 19: Short extraction table of original articles on CoREC infected patients

Nr	Author	Study Period	Infection site	Study type	Outcome	Location	Period of outcome	Population	Matching criteria	LOS (days)	Notes
1	Kontopidou 2011[28]	11/03-12/06	not defined	Retrospective cohort study	Mort. rate=0%	Athens, Greece	hospitaliz	2 cases	none	no data	the study analysed Gram- bacteria
2	Nordmann 2016[59]	2015	BSI	Prospective cohort study	Mort. rate=0%	Switzerland	hospitaliz	2 cases	none	no data	the first 2 cases in a row in the hosp
3	Oostdijk 2011[60]	01/08-10/10	different	Retrospective cohort study	no data	Utrecht, Netherlands	hospitaliz	11 cases	none	*median(range, IQR) cases=29 (8–96, 29.8)	*info based on 18 patients (not just the EC cases) with Gram- inf.

We did not apply any filter to the literature research because we wanted to be as broad as possible in including all the papers that had *Escherichia coli* and colistin resistance as subjects. The first search yielded numerous studies; however, after analysing them the vast majority was excluded because not relevant for our study question and none of them fulfilled our eligibility criteria.
Many papers reported CoREC strains in animal samples or referred to molecular characterization of resistant bacteria. Resistance in humans was analysed in studies which included different Gram negative bacteria or all Enterobacteriaceae. When it was possible, the results of mortality due to CoREC infections were extrapolated from these studies, otherwise they were excluded as unspecific for our research purpose.
The colistin resistant Escherichia coli infections are a relative new research topic, and it seems that no structured study was yet conducted to investigate the burden of these infections in terms of attributable mortality or attributable length of stay in hospital

6. Third-generation Cephalosporin resistant *Escherichia coli*

The resistance to the 3rd generation of cephalosporin is due to the production of extended-spectrum beta-lactamase enzymes (ESBL). These enzymes can deactivate extended spectrum (3rd generation) cephalosporins (e.g., ceftazidime, cefotaxime, and ceftriaxone) and monobactams (e.g., aztreonam).
The guidelines internationally accepted for the detection of ESBL-producing bacteria, including *Escherichia coli*, have been defined in 1999 by the US-based National Committee for Clinical Laboratory Standards (now the Clinical and Laboratory Standards Institute).
For the purpose of these review, 3rd generation cephalosporin resistant *Escherichia coli* (3GCREC) and ESBL producing *Escherichia coli* (ESBL-EC) will be considered equivalent.

To research the attributable case fatality and the attributable LOS to third-generation cephalosporin resistant *Escherichia coli* (3GCREC) we performed 2 advanced searches on Pubmed using once the mesh term "Escherichia coli" AND all fields "third generation cephalosporin", and the second time the mesh term "Escherichia" AND all fields "ESBL OR extended-spectrum beta lactamase". Both times a filter for human studies was applied. Embase was searched for 'Escherichia AND third generation cephalosporin', and 'Escherichia coli AND (ESBL OR extended-spectrum beta-lactamase'. We applied a filter to include only human studies and to exclude abstracts that did not have a correspondent article. An advanced search on the Cochrane Library was performed using terms "Escherichia coli" AND "third generation cephalosporin", plus a second search using "Escherichia coli" AND "ESBL OR extended-spectrum beta-lactamase". No language restrictions were made, and filter for research in humans was applied.

The flow diagram, presented in Figure 6 describes the steps in the literature review of 3rd generation cephalosporin resistant *Escherichia coli* infections. The results obtained using ESBL and 3rd generation cephalosporin as key words were aggregated.

Once identified the studies reporting relevant data, these were grouped based on the site of infection. The majority of them reported BSI infections, even though not all of them had a control group.

Figure 6: Search for studies on 3rd generation cephalosporin resistant *Escherichia coli*. Inclusion and exclusion diagram.

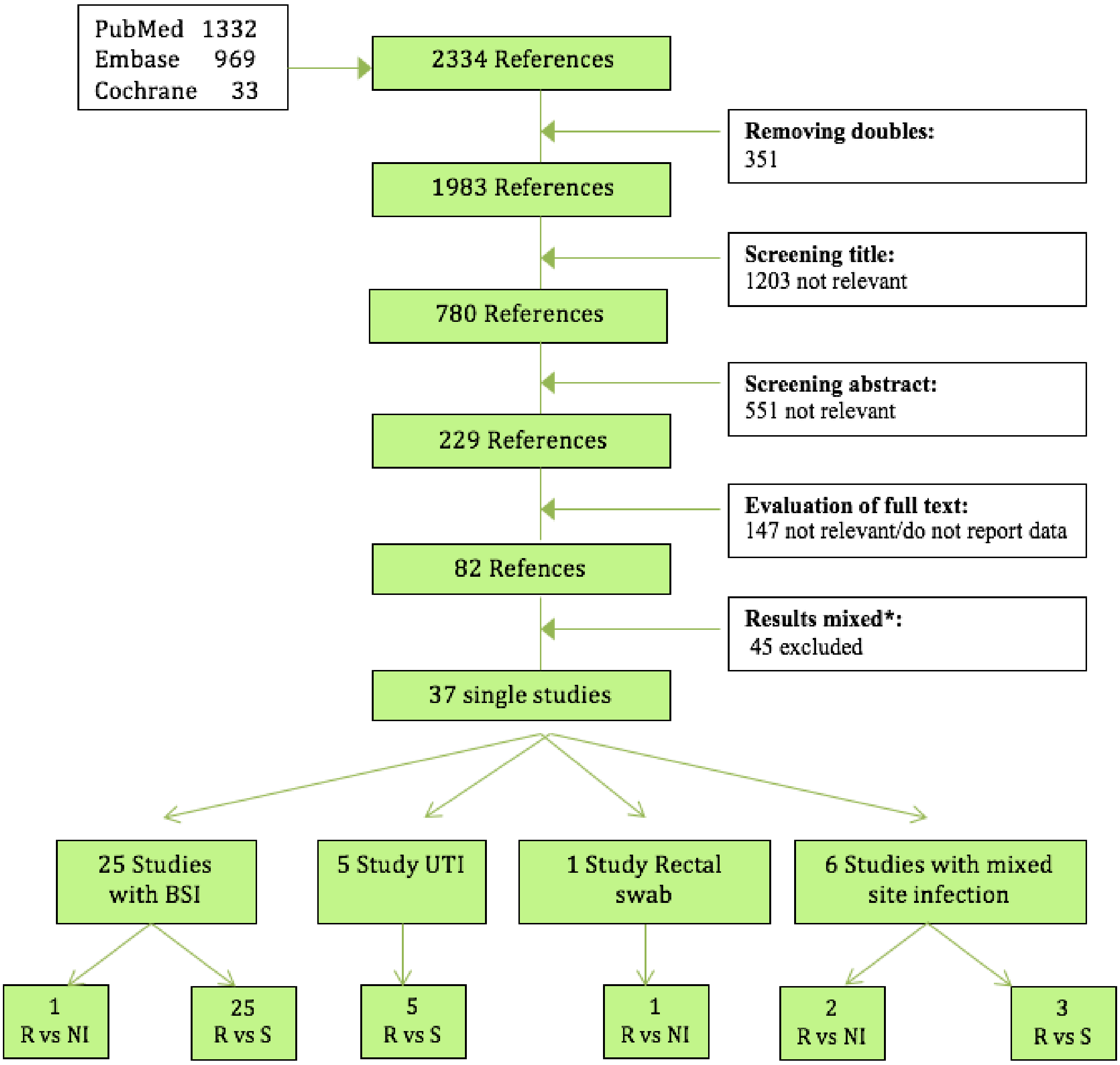

*These studies reported results for ESBL-*Enterobacteriaceae* or together for *E.coli* and *Klebsiella pneumoniae.*

The following tables include studies reporting results for BSI, UTI, rectal swab and for mixed site of infection due to 3GCREC, based on the comparison group they used. All information is reported as was presented in the original papers.

Table 20: Short extraction table of original articles on BSI mortality of 3GCREC infected patients vs 3GCSEC infected patients and vs non infected patients

Nr	Author	Study Period	Study type	Outcome	Location	Period of outcome	Population	Matching criteria	LOS (days)	Notes
1	de Kraker 2011[61]	07/07-06/08	Prospective parallel matched double cohort	a)C1: Mort. resist=32%<br>Mort. control=6%<br>OR=4.6, 95%CI(1.7–12.3)<br>C2: Mort. sensit=17%<br>Mort. control=7%<br>OR=1.9; 95%CI(1.4–2.5)<br>OR R vs S= 2.5; 95%CI(0.9–6.8)<br>b)C1: Mort. resist=36%<br>Mort. control=5%<br>HR=5.7; 95%CI(2.5–13.0)<br>C2:Mort. sensit=17%<br>Mort. control=7%<br>HR=2.0; 95%CI(1.5–2.5)<br>HR R vs S=2.9; 95%CI(1.2–6.9)	13 EU countries	a)30 days<br>b) hospitaliz	C1) 111 resistant<br>204 NI controls for resistant<br>C2) 1110 sensitive<br>2084 controls for sensitive	LOS before enrolment	median (IQR)<br>resist=12(6-25)<br>sensit=10(6-17)<br>control=6(3-16)	

Table 21: Short extraction table of original articles on BSI mortality of 3GCREC infected patients vs 3GCSEC infected patients

Nr	Author	Study Period	Study type	Outcome	Location	Period of outcome	Matching	Population	LOS (days)	Notes
1	Anunnatsiri 2012[62]	2005-2006	Retrospective case-control study	Mort. cases=29%<br>Mort. controls= 11.5%	Thailand	hospitaliz	none	32 cases<br>113 controls	prior BSI<br>median (range)<br>cases=4 (0-66)<br>controls=0 (0-82)	
2	Apisarnthanarak 2008[63]	07/03-12/07	Retrospective case-control study	Mort. cases=8%<br>Mort. controls= 15%*	Thailand	hospitaliz	microorganism, site of infection, unit, date of admission	24 cases<br>108 controls *	median (range)<br>cases=8 (1–43)<br>controls=6 (3–15)	*controls have EC or KP
3	Cornejo-Juarez 2012[64]	2004-2009	Retrospective case-control study	a)Mort. cases=38.6%<br>Mort. controls= 21.6%<br>b)Mort. cases=19.3%<br>Mort. controls= 36.4%	Mexico	60 days	unit	88 cases<br>88 controls	difficult to interpret	patients with haematological malignancies<br>a) death related to infection<br>b) death not related to infection
4	Courpon-Claudinon 2011[65]	2005	Prospective multi-centre study	Mort. cases=30.8%<br>Mort. controls= 12.3%	France	hospitaliz	none	39 cases<br>1012 controls	no data	surveillance study
5	Ortega 2009[66]	01/91-12/07	Prospective study	Mort. cases=15.6%<br>Mort. controls= 7.6%	Spain	a) 7days<br>b) 30days	none	211 cases<br>3247 controls	no data	
6	Trecarichi 2009[67]	01/00-12/07	Retrospective cohort	Mort. cases=42.3%<br>Mort. controls= 5.6%<br>OR=8.84; 95%CI(1.48-52.91)	Italy	30 days	none	26 cases<br>36 controls	no data	patients with haematological malignancies

7	Hsieh 2010[68]	01/05-12/06	Retrospective cohort	Mort. cases=21.1% Mort. controls= 12.2%	Taiwan, China	30 days	emergency room patients	19 cases 385 controls	mean (±SD) cases=15.5(±8.5) controls=6.1(±6.9)	patients with community onset infection
8	Tumbarello 2010[69]	01/06-12/06	Retrospective cohort	Mort. cases=29.7% Mort. controls= 6.1% OR=4.43; 95%CI(2.46-7.95)	Italy	21 days	none	37 cases 97 controls	mean (±SD) cases=20(±17) controls=13(±9)	
9	Gudiol 2010[70]	01/06-10/08	Prospective cohort	a)Mort. cases=12% Mort. controls= 8% b)Mort. cases=35% Mort. controls= 19%	Spain	a) 7days b) 30days	underlying condition	17 cases 118 controls	no data	patients with cancer/stem cell transplant
10	Ho 2002[71]	01/97-12/98	Retrospective case-control study	Mort. cases=18% Mort. controls= 7%	China	30 days	specialty, sex, age(±10), date of isolation	50 cases 100 controls	no data	
11	Rodriguez-Bano 2010[72]	10/04-01/06	Prospective cohort	Mort. cases=17% Mort. controls= 8%	Spain	14 days	hosp, time period	95 cases 188 controls	no data	patients with community onset infection
12	Kang 2010[73]	09/08-04/09	Retrospective cohort	Mort. cases=15% Mort. controls= 7.6% OR=2.99; 95%CI(1.01–8.84)	Republic of Korea	30 days	none	82 case 783 controls	no data	
13	Garcia-Hernandez[74]	01/06-07/07	Retrospective and prospective cohort study	a)Mort. cases=35.5% Mort. controls= 16.8% b)Mort. cases=26.5% Mort. controls= 9.2%	Spain	a)within 7 days b)within 30 days	none	34 cases 119 controls	mean (±SD) cases=24.3(±22) controls=20.9(±2 6.7)	
14	Denis 2015[75]	01/05-12/09	Retrospective study	Mort. cases=30% Mort. controls= 27% OR=1.23; 95%CI(0.36–4.23)	France	30 days	date of isolation	41 cases 41 controls	median (range) cases=15 (10-21) controls=11 (7-17)	

15	Ha 2013[76]	01/10-05/12	Retrospective cohort study	Mort. cases=22.1%<br>Mort. controls= 12.2%<br>OR = 3.01; 95%CI(1.45–6.28)	Republic of Korea	30 days	underlying condition	95 cases<br>255 controls	no data	all patients had cancer
16	Kaya 2013[77]	01/07-10/11	Retrospective case-control study	Mort. cases=36.4%<br>Mort. controls= 27.5%	Turkey	hospitaliz	none	44 cases<br>69 controls	no data	
17	Kim 2013[78]	01/07-12/08	Retrospective study	Mort. cases=6.7%<br>Mort. controls= 5.5%	South Korea	30 days	underlying condition	15 cases<br>72 controls	no data	neutropenic patients
18	Lambert 2011[50]	01/05-12/08	Retrospective cohort study	a)Mort. cases=50%<br>Mort. controls= 40%<br>aHR=1.3; 95%CI(0.8–2.2)<br>b)Mort. cases=47%<br>Mort. controls= 29%<br>aHR=1.4; 95%CI (0.9–2.3)	Eu countries	hospitaliz	ICU	42 cases<br>217 controls	*median (IQR)<br>cases=18(11-29)<br>controls=17(9-29)	a)BSI<br>b)pneumonia<br>patients admitted to ICU<br>*from infection to discharge
19	Leistner 2014 a[79]	01/08-12/10	Retrospective cohort study	Mort. cases=26%<br>Mort. controls=18%	Germany	hospitaliz	none	115 cases<br>983 controls	median (range)<br>cases=27 (12-53)<br>controls=15 (8-32)	
20	Leistner 2014 b[80]	01/08-12/11	Retrospective cohort study	Mort. cases=25.3%<br>Mort. controls=17.6%	Germany	hospitaliz	none	178 cases<br>1322 controls	Multiplicative effect<br>cases=1.07<br>controls=0.83	
21	Martelius 2016[81]	01/99-12/13	Retrospective study	a)Mort. cases=7.7%<br>Mort. controls= 5.9%<br>b)Mort. cases=14.3%<br>Mort. controls= 11.9%	Finland	a) 7 days<br>b) 28 days	Unit, specimen	182 cases<br>2035 controls	no data	
22	Park 2011[82]	01/05-12/10	Retrospective cohort study	a) Mort. cases=8%<br>Mort. controls= 6%<br>b)Mort. cases=18%<br>Mort. controls= 8%	Republic of Korea	a) 7days<br>b) 30days	community-onset infection, time period	50 cases<br>100 controls	no data	patients with community -onset infection

				OR=6.4; 95%CI(0.3–145.5)						
23	Yip 2006[83]	10/94-08/03	Retrospective study	Mort. cases=27.3%<br>Mort. controls= 3.9%	Hong Kong	hospitaliz	underlying condition	11 cases<br>77 controls	no data	patients were in continuous ambulatory peritoneal dialysis
24	Van Aken 2014[84]	01/11-09/12	Retrospective case-control study	Mort. cases=9%<br>Mort. controls= 7%		14 days	time period, study location	70 cases<br>140 controls	median±SD<br>cases=11±12<br>controls=9±10	
25	Melzer 2007[85]	06/03-11/05	Retrospective cohort	Mort. cases=60.9%<br>Mort. controls= 23.7%<br>OR=3.57; 95%CI(1.48-8.60)	United Kingdom	30 days	none	46 cases<br>308 controls	median<br>cases=9<br>controls=12	

Table 22: Short extraction table of original articles on UTI mortality of 3GCREC infected patients vs 3GCSEC infected patients

Nr	Author	Study Period	Study type	Outcome	Location	Period of outcome	Matching	Population	LOS (days)	Notes
1	Al-Otaibi 2013[86]	06/09-06/11`	Retrospective case-control study	Mort. cases=8.9%<br>Mort. controls= 0.9%	Saudi Arabia	hospitaliz	none	113 cases<br>226 controls	no data	hospitalised and outpatients
2	Ena 2006[87]	01/99-12/04	Retrospective cohort	Mort. cases=8%<br>Mort. controls= 8%*	Spain	hospitaliz	temporal occurrence	61 cases<br>61 controls	no data	*author reports that death was not attributable
3	Suankratay 2008[88]	01/04-12/06	Prospective cohort	Mort. cases=2.9%<br>Mort. controls= 0%*	Thailand	b) 14days	underlying condition	35 cases<br>71 controls*	no data	female patient with acute pyelonephritis<br>*controls were EC, KP, PM
4	Esteve-Palau 2015[89]	08/10-07/13	Matched cohort study	Mort. cases=10%<br>Mort. controls= 7%<br>OR=8.011; 95%CI(2.48-25.92)*	Barcelona, Spain	30 days	sex, age, date of admission	60 cases<br>60 controls	mean (±SD)<br>cases=11.6(±1.5)<br>controls=7.5(±0.8)	*Risk of failure within 7 days (R vs S)

5	Park 2015[90]	01/07-12/13	Retrospective case-control study	Mort. cases=1.3%<br>Mort. controls= 1.8%	Republic of Korea	7 days	time of treatment	75 cases<br>225 controls	no data	patients with community-acquired infection, and acute pyelonephritis

Table 23: Short extraction table of original articles on rectal swab infections mortality of 3GCREC infected patients vs non infected patients

Nr	Author	Study Period	Study type	Outcome	Location	Period of outcome	Matching	Population	LOS (days)	Notes
1	Arnan 2011[91]	05/06-12/07	Prospective multicentre cohort study	a)Mort. cases=1.6%<br>Mort. controls= 0%<br>b)Mort. cases=10%<br>Mort. controls= 11%	Barcelona, Spain	a) 7days<br>b)hospitaliz	undelying condition	63 cases<br>154controls	mean (±SD) cases=30.15 (±13.70) controls=29.05 (±12.11)	patients with acute leukaemia undergoing haematopoietic stem cell transplantation, and who received chemotherapy and developed grade IV neutropenia

Table 24 and Table 25 present the results of studies that include patients with different sites of infection. Almost all the articles specify the number of cases for the different infection sites but they do not report different mortalities based on the infection site. When it was possible, the results are presented differentiating the mortality for each site of infection.

Table 24: Short extraction table of original articles on mortality of CRKP infected patients vs non infected patients for different infection sites

Nr	Author	Study Period	Study type	Outcome	Location	Period of outcome	Matching	Population	LOS (days)	Notes
1	Nicolas-Chanoine 2012[92]	11/08-06/09	Prospective case-control-control study	Mort. resist=12%<br>Mort. sensit=7%<br>Mort. control=5%	France	hospitaliz	none	152 resist<br>152 sensit<br>152 controls	no data	

2	Hayakawa 2013[93]	02/10-07/11	Retrospective case-case-control study	a)Mort. resist=5.7%<br>Mort. sensit=3.5%<br>Mort. NI=3.8%<br>OR R vs S= 1.66; 95%CI(0.37–7.34)<br>OR R vs NI=2.33; 95%CI(0.9–6.07)<br>b)Mort. resist=13.7%<br>Mort. sensit=10.2%<br>Mort. NI=7.6%<br>OR R vs S=1.4; 95%CI(0.52–3.75)<br>OR R vs NI=2.36; 95%CI(1.17–4.78)	USA	a) hospitaliz<br>b) 3 months	none	319 resist<br>58 sensit<br>319 controls	median (IQR)<br>resist=7(4-12)<br>sensit=7(3-20)<br>NIl=4(2-6)	

Table 25: Short extraction table of original articles on mortality of 3GCREC infected patients vs 3GCREC infected patients for different infection sites

Nr	Author	Study Period	Study type	Outcome	Location	Period of outcome	Matching	Population	LOS (days)	Notes
1	Pena 2008[94]	01/96-12/03	Retrospective matched cohort study	a)Mort. cases=16%<br>Mort. controls= 6%<br>b)Mort. cases=25%<br>Mort. controls= 11%	Spain	a)7 days<br>b)30 days	site of infection, date of admission	100 cases<br>100 controls	no data	
2	Kang 2011[95]	09/10-05/11	Retrospective case-control study	Mort. cases=7.4%<br>Mort. controls= 2%	Republic of Korea	30 days	none	108 cases<br>100 controls	no data	patients with community onset infection
3	Maslikowska 2016[96]	06/10-04/13	Retrospective case-control study	a)Mort. cases=15%<br>Mort. controls= 2%<br>b)Mort. cases=5%<br>Mort. controls= 2%	Canada	14 days	sex, age, type of inf, bed allocation	61 cases<br>49 controls	not specific for EC	a) all cause mortality<br>b) attributable mortality: defined as chart-documented indication of infection contributing to the cause of death

This literature research produced a large amount of results, but not all of them fulfilled our selection criteria. Information on mortality is often published, but this was not specific for Escherichia coli: they were pooled for Enterobacteriaceae, or for Escherichia coli and Klebsiella pneumoniae together.
Many studies were excluded because they did not report a comparison group. Several studies referred to genetic analyses and were not eligible for the purposes of this review. All studies were performed in healthcare settings, though some of them report cases of infection that originated in the community or that had onset in the community.

7. Carbapenem resistant *Klebsiella pneumoniae*

To research the attributable case fatality and the attributable LOS to carbapenem resistant *Klebsiella pneumoniae* (CRKP) we performed an advanced search on Pubmed using mesh term “Klebsiella pneumoniae” and all fields “carbapenem resistant”. Embase was searched for “carbapenem resistant klebsiella pneumoniae”. An advanced search on the Cochrane Library was performed using terms “klebsiella pneumonia” and “carbapenem”. No language restrictions were made, and a filter for research in humans was applied.

Once identified the studies reporting relevant data, these were grouped based on the site of infection. The majority of them reported mixed samples, as for example blood and urine, blood and bronco-specimens, etc.

Figure 7: Search for studies on carbapenem-resistant *Klebsiella pneumoniae*. Inclusion and exclusion diagram.

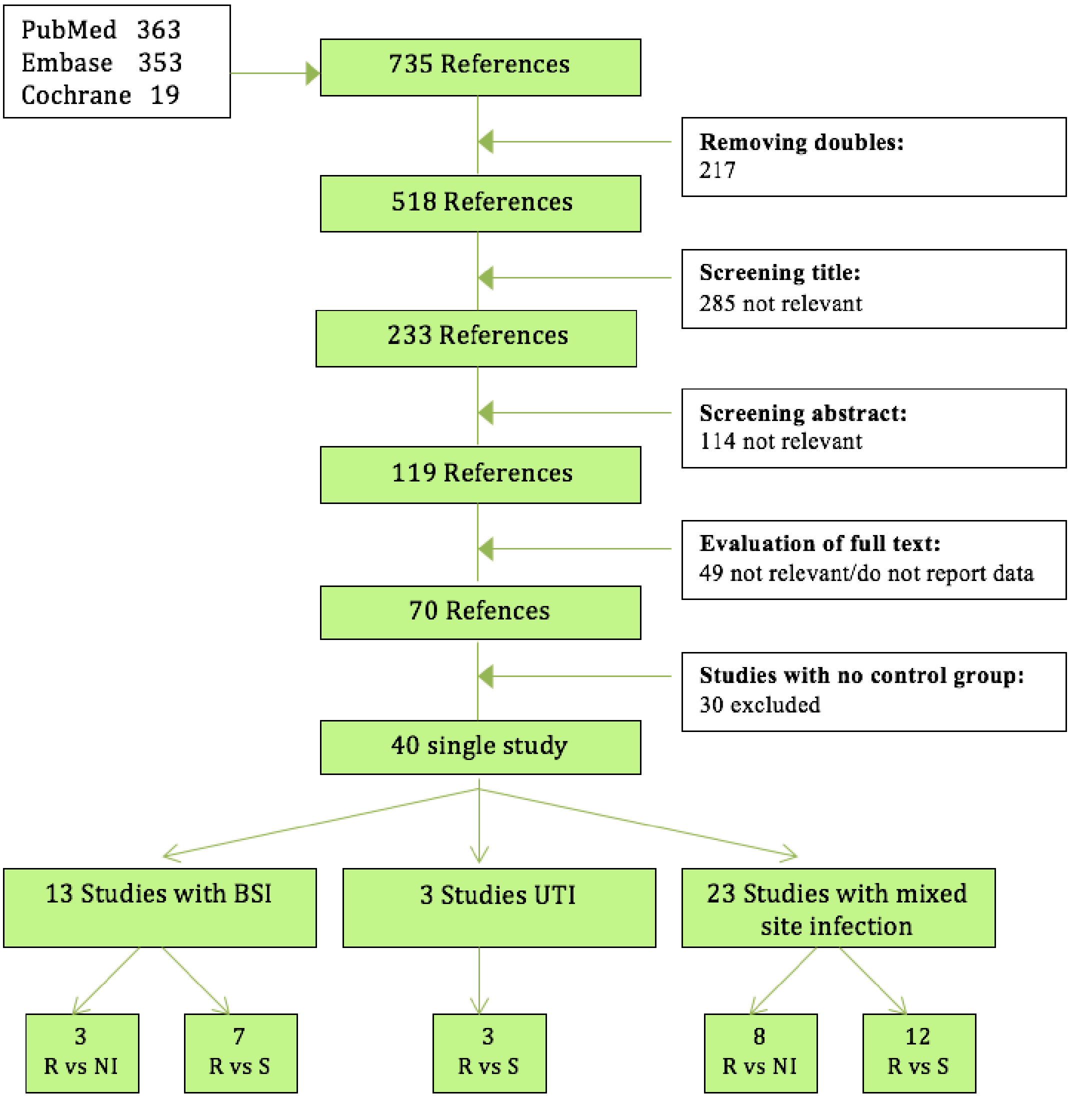

From the 13 studies focusing on BSI, 10 were eligible for our purposes. 3 studies were discarded because the comparison was different from those in our inclusion criteria. The same was applied to the studies with mixed site of infection.

In the results were also included 3 systematic review of the literature studies[97-99] of which;

- 1 reported a meta-analysis of data[97]
- 2 reported results for bacteraemia[97,98],
- 1 does not specify the infection site[99]

All 3 reviews considered CSKP infected patients as a comparison group. Those considering bacteraemia were included in the results for BSI in the R vs S group. References included in the systematic reviews were examined in order to include primary studies if they were missed by our initial search. The published results of 2 systematic reviews are also reported in the tables below.

The following tables include studies reporting results for BSI and UTI from CRKP, based on the comparison group they used. All information is reported as was presented in the original papers.

Table 26: Short extraction table of original articles on BSI mortality of CRKP infected patients vs non infected patients

Nr	Author	Study Period	Study type	Outcome	Location	Period of outcome	Population	Matching criteria	LOS (days)	Notes
1	Borer 2009[100]	10/05-10/08	Matched retrospective study	Mort. cases=71.9%<br>Mort. controls=21.9%<br>RR=3.3; 95%CI(2.9-28.5)	Beer-Sheva Israel	hospitaliz	32 cases<br>32 controls	*	median:<br>cases=21<br>contr=18	
2	Gallagher 2014[101]	06/05-10/10	Case-case-control study	Mort. cases= 45%<br>Mort. contr= 18%<br>OR=3.8; 95%CI(1.4-9.9)	Philadelphia USA	hospitaliz	43 cases<br>154 controls	**<br>controls are hospitalized for >= 72h	mean:<br>cases=54<br>contr=19.5	-controls are patients hospitalized for >= 72h
3	Mouloudi 2014[102]	01/08-12/11	Observational matched cohort study	Mort. cases=82.7%<br>Mort. contr=32%	Thessaloniki Greece	hospitaliz	17 cases<br>34 controls	Liver transplant	median (range):<br>cases=28(10-64)<br>in ICU	-patients are liver transplant recipients<br>-controls had also live transplant but no KP infection

* Hospitalization period, LOS, Charlson comorbidity index, McCabe score, underlying disease, age and sex
** same month/year of occurrence and same medical unit

Table 27: Short extraction table of original articles on BSI mortality of CRKP infected patients vs CSKP infected patients

Nr	Author	Study Period	Study type	Outcome	Location	Period of outcome	Population	LOS (days)	Notes
1	Falagas 2014[97]	until 04/12	Review Meta-analysis	Attribut. mort.= 26-44% in 7 studies<br>Attribut. mort. = (-3)-(-4)% in 2 studies<br>RR=2.05; 95%CI(1.56–2.69)*	World	different based on the study	985 patients	no data	* referred to all 9 studies<br>** only 6 BSI studies<br>*** remaining 3 (not BSI/mixed site of infection) studies

				RR 2.19; 95%CI(1.82–2.63)**<br>2RR=1.46; 95%CI(0.47–43.49)***					
2	Mouloudi 2010[103]	01/07-12/08	Nested case-control study	a) Mort.cases= 56.8%<br>Mort. contr= 41%<br>b) Mort. cases= 67.6%<br>Mort. contr= 41%<br>Mort. cases= 27%<br>Mort. contr= 14%	Thessaloniki Greece	a) mortality in ICU<br>b) mortality in hospital<br>c) attributable mortality*	37 cases<br>22 controls	median(range):<br>total=26 (8-90) in ICU	*within 24h from infection or with infection symptoms despite appropriate therapy
3	Simkins 2014[104]	06-10	Case-control study	Mort. cases= 46%<br>Mort. contr= 0%	New York, USA	6.5 months	13 cases<br>39 controls	no data	-patients are kidney transplant recipients
4	Viale et 2013[98]	1977-2012	Review	Crude mortality rates =20-70%<br>Attribut. mort. patients:<br>-hematologic=75%<br>-ICU=11%<br>-transplant=7%<br>-LTC=5%<br>-medicine=2%<br>-surgery=2%	Italy			median time in developing CRKP infections in hospital (range): cases=14(0-152)	-it is a review not a meta-analysis<br>-good source of references.
5	Hussein 2013[105]	01/06-12/08	Retrospective case-control study	Mort.cases= 43.7%<br>Mort.contr= 29%	Haifa, Israel	30 days	103 cases<br>214 controls	median (range):<br>cases=17(1-125)<br>controls=12(1-202)	
6	Ben-David 2012[106]	01/06-12/06	Retrospective cohort study	a)Mort. cases=69%<br>Mort. controls=24%<br>b)Mort. cases=48%<br>Mort. controls=17%	Sheba, Israel	a)hospitaliz<br>b)infection-related mortality	42 cases<br>85 controls	mean LOS before infection detection ±SD: survivors=25±81<br>deceased=27±37	
7	Daikos. 2009[107]	02/04-03/06	Prospective observational study	Mort.cases= 42.8%<br>Mort.contr= 16.9%<br>OR=3.95; 95%CI(0.94–16.6)	Athens, Greece	14 days	14 cases<br>148 controls	mean LOS before bacteraemia ±SD: both groups=25.85 ± 37.87	-all patients were infected with VIM-1-producing KP

Table 28: Short extraction table of original articles on UTI mortality of CRKP infected patients vs CSKP infected patients

Nr	Author	Study Period	Study type	Outcome	Location	Period of outcome	Population	Matching	LOS (days)	Notes
1	Brizendine 2015[108]	2006-2012	Retrospective cohort study	Mort. cases=18%<br>Mort. contr.=2%	Cleveland, USA	hospitaliz	22 cases<br>64 controls	Patients had solid organ transpalantation	median:<br>cases=7<br>contr=1	4/22 cases died<br>1/64 controls died<br>-controls are susceptible
2	Pouch 2015[109]	01/07-12/10	Case-control study	Mort. cases=30%<br>Mort. contr.=10%<br>HR=3.0;<br>95%CI(1.0–9.0)	New York, USA	not defined	20 cases<br>252 controls	patients had kidney transplantation	median(IQR):<br>cases=17(9-43)<br>controls=7(5-10)	-patients are kidney transplant recipients
3	Shilo 2013[110]	01/06-04/09	Case-control study	Mort. cases=29%<br>Mort. contr.=25%	Jerusalem, Isreael	hospitaliz	135 cases<br>127 controls		mean±SD<br>cases=28±33<br>controls=22±28	-controls are CSKP producing ESBL

In the study of Brizendine et al.[108], 7 case patients (32%) and 8 control patients (13%) had also bacteraemia of CRKP. Pouch et al.[109] reported in their study that 15% of cases and 3% of controls developed bacteraemia within 14 day of bacteriuria onset, while the study of Shilo et al.[110], 3 patients (4%) in both groups had also bacteraemia. These cases were not excluded from the results displayed in table 28. In Pouch et al. 2 deaths in the cases group followed CRKP sepsis, while none of the deaths in the control group was related to CSKP sepsis.

Table 29 and Table 30 present the results of studies that include patients with different sites of infection. Almost all the articles specify the number of cases for the different infection sites but they do not report different mortalities based on the infection site. When it was possible the results are presented differentiating the mortality for each site of infection.

Table 29: Short extraction table of original articles on mortality of CRKP infected patients vs non infected patients for different infection sites

Nr	Study	Study Period	Study type	Outcome	Location	Period of outcome	Population	Matching	LOS (days)	Notes

1	Giannella 2014[111]	06/11-06/13	Matched case-control study	a)Mort cases=0%<br>Mort. contr.=5%<br>b)Mort. cases=0%<br>Mort. contr.=15%	Bologna, Italy	a)30 days<br>b)60 days	10 cases<br>20 controls	liver transplant	median (range):<br>cases=16(14-27)<br>controls=20(15-62)	-all patients had liver transplant
2	Luebbert 2014[112]	09/10-09/11	Case-control study	Mort. cases=78%<br>Mort. contr.=11%	Leipzig, Germany	3 months after last case	9 cases<br>18 controls	liver transplant	cases=60<br>controls=32	-patients are liver transplant recipients<br>-controls had also live transplant but not KP infection
3	Nouvenne 2014[113]	08/11-05/12	Cross-sectional study	Mort. cases =21.8%<br>(Mort. Infected=47.5%<br>Mort. Colonized=10.7%)<br>Mort.contr. =15%	Parma, Italy	hospitaliz	133 cases<br>(93 colonized, 40infected)<br>400 controls		mean±SD<br>cases=35±24<br>controls=18±12	
4	Bleumin 2012[114]	01/06-06/09	Nested case-control study	Mort. cases= 88%<br>Mort. contr.= 52%<br>aHR=5.9; 95%CI(3.2-11.0)	Jerusalem, Isreael	study period	43 cases<br>150 controls	-patients had hemodialysis for at least 14 days	no data	-patients had hemodialysis for at least 14 days
5	Debby 2012[115]	05/07-04/ 08	Prospective cohort study	Mort. cases=45.8%<br>Mort. contr.=38.6%	Tel Hashomer, Israel	hospitaliz	48 cases<br>132 controls		mean±SD<br>cases=6.06±1.2<br>3 controls=8.39 ±1.49	-the cases acquired the infection in the hospital
6	Schwaber 2008[116]	2003-2006	Case-case-control study	Mort. rate cases=44%<br>Mort. rate controls=2%<br>OR=6.7; 95%CI(2.4–18.8)	Tel-Aviv, Israel	hospitaliz	48 CRKP<br>59 controls		median (IQR):<br>cases=19 (9-38)<br>controls=2 (1-4)	
7	Pereira 2015[117]	01/10-01/13	Retrospective cohort study	Mort. rate cases=45%<br>Mort. rate contr.=7%<br>R vs NI: HR=6.92; 95%CI(3.24–14.79)	New York, USA	1 year from transplant	20 CRKP<br>248 controls	-patients had liver transplant	Median (range):<br>cases=40(23-82)<br>controls=12 (9-21)	-patients are liver transplant recipients
8	Kofteridis 2014[118]	01/09-12/11	Case-case-control study	Mort. rate cases=27%<br>Mort. rate contr=4%	Heraklion, Greece	not defined	83 cases<br>161 controls	no data	none	

Table 30: Short extraction table of original articles on mortality of CRKP infected patients vs CSKP infected patients for different infection sites

Nr	Study	Study Period	Study type	Outcome	Location	Period of outcome	Population	LOS (days)	Matching	Notes
1	Candevir Ulu 2015[119]	01/12-12/12	Retrospective cohort study	Mortality cases=44,7%<br>Mortality contr.=51%	Adana, Turkey	hospitaliz	47 cases<br>51 controls	mean:<br>cases=37.3<br>contr.=29.94<br>median:<br>cases=19<br>contr.=11	none	
2	Dizbay 2014[120]	2004-2010	Retrospective study	Mortality cases=57.1%<br>Mortality contr.=27.4%	Turkey	not defined	42 cases<br>798 controls	mean<br>cases=38.27<br>contr.=28.17	none	-cases had nosocomial infections<br>- K spp
3	Hoxha 2016[121]	12/12-07/13	Matched cohort study	a)Attribut. mort.= 16%<br>b)Attribut. mort.= 41%<br>mIRR=3.0; 95%CI(1.3-7.1)	Italy	a)6 days<br>b)30 days	49 cases<br>49 controls	no data	Age, infection site hospital	
4	Jiao 2015[122]	01/10-12/11	Retrospective case-control study	Mort. cases=33.6%<br>Mort. contr.=16.6%	Shangai, China	hospitaliz	30 cases<br>30 controls	mean:<br>cases=33.8<br>controls=18	none	
5	Kofteridis 2014[118]	01/09-12/11	Case-case-control study	Mort. rate cases=27%<br>Mort. Rate contr.=15%	Heraklion, Greece	not defined	83 cases<br>79 controls	no data	none	
6	Patel 2008[123]	07/04-06/06	Matched case-case study	Mort. cases=48%<br>Mort. contr.=26%<br>OR=4.69; 95%CI(1.9–11.58	New York, USA	hospitaliz	99 cases<br>99 controls	mean±SD<br>cases=25.19±24.9<br>controls=6.44±10.1 median:<br>cases=21<br>controls=1	Inf. site	
7	Correa 2013[124]	01/06-08/08	Matched case-control study	Mort. cases=50%<br>Mort. contr.=27.5%	Sao Paulo, Brazil	hospitaliz	20 cases<br>40 controls	mean:<br>cases=45.5<br>controls=27	Inf. date, inf. site, unit where acquired	

8	CDC Atalanta 2011[125]	04/09-02/11	Matched case-case study	Mortality cases=5%<br>Mortality controls=8%	West Virginia, USA	hospitaliz	19 cases<br>38 controls	mean:<br>cases=11.4<br>controls=7.4	Age, data of sample collection	-cases likely arrived at the hospital with the infection
9	Schwaber 2008[116]	2003-2006	Case-case-control study	Mort. rate cases=44%<br>Mort. rate contr.=12.5%<br>OR=5.4; 95%CI(1.7–17.1)	Tel-Aviv, Israel	hospitaliz	48 CRKP<br>56 controls	median (IQR):<br>cases=19(9-38)<br>controls= 1 (1-8)	none	
10	Falagas 2007[126]	10/00-04/06 *<br>10/03-04/06 **	Matched case-control study	Mortality cases=30.1%<br>Mortality controls=33.9%	Athens and Crete, Greece	hospitaliz	53 cases<br>53 controls	mean±SD<br>cases=21.4±23.9<br>contr.=15.3±19.9	Age, LOS, inf. site, year of admission	*Athens<br>**Crete
11	Pereira 2015[117]	01/10-01/13	Retrospective cohort study	Mort. rate cases=45%<br>Mort. rate contr.=28%	New York, USA	1 year from transplant	20 CRKP<br>36 controls	Median (range)<br>CRKP=40(23-82)<br>CSKP=12 (9-21)	none	-patients are liver transplant recipients
12	Hauck 2016[127]	12/11-09/14	Prospective multicenter observational study	Mort.rate BSI=38%<br>aHR=2.59; 95%CI(1.52–4.50)<br>Mort.rate Pneu=34%<br>aHR=3.44; 95%CI(1.80–6.48)<br>Mort.rate UTI=7%<br>aHR=0.68; 95%CI(0.30–1.45)<br>Mort.rate contr.=9%	San Diego, USA	hospitalization	260 cases:<br>(90 BSI, 49 pneumonia, 121 UTI)<br>223 controls*	median (IQR)<br>BSI=14 (9-24)<br>Pneu=19 (10-30)<br>UTI=10 (6-17)<br>contr=9 (5-16)	none	*R vs CRKP urinary colonization

* controls had CRKP urinary colonization

All studies were performed in healthcare settings, though some of them report cases of infection that might have been originated in other settings.

Comparing our review with the systemic review of the literature published by the World Health Organisation (WHO) in 2014[128], we detected more recent publications (2014-2016). The WHO report includes studies comparing with CSKP, while we also included NI as a comparator.
We excluded two studies reported in the WHO review as they did not agree with our eligibility criteria. Daikos et al. 2007[129] defined cases as VIM-1-producing *K. pneumoniae* but did not test the samples for carbapenem resistance, while Lee et al. 2012[130] defined cases as non-susceptible (intermediately susceptible or resistant) to carbapenem.

8. Colistin resistant Klebsiella pneumoniae

To research the attributable case fatality and the attributable LOS to colistin resistant *Klebsiella pneumoniae* (CoRKP) we performed 2 advanced searches on Pubmed using once the mesh term “Klebsiella pneumoniae” AND all fields “colistin resistant”, and the second time the mesh term “Klebsiella pneumoniae” AND all fields “colistin resistance”. Embase was searched for ‘Klebsiella pneumoniae’ AND ‘colistin resistance’, and for ‘Klebsiella pneumoniae’ AND ‘colistin resistant’. An advanced search on the Cochrane Library was performed using terms “Klebsiella pneumoniae” AND “colistin”. No language restrictions were made, and no filters were applied.

The flow diagram, presented in Figure 8 describes the steps in the literature review of colistin resistant *Klebsiella pneumoniae* infections. The results obtained using the two different key words in PubMed and Embase were aggregated.

Figure 8: Search for studies on colistin resistant *Klebsiella pneumoniae*. Inclusion and exclusion diagram.

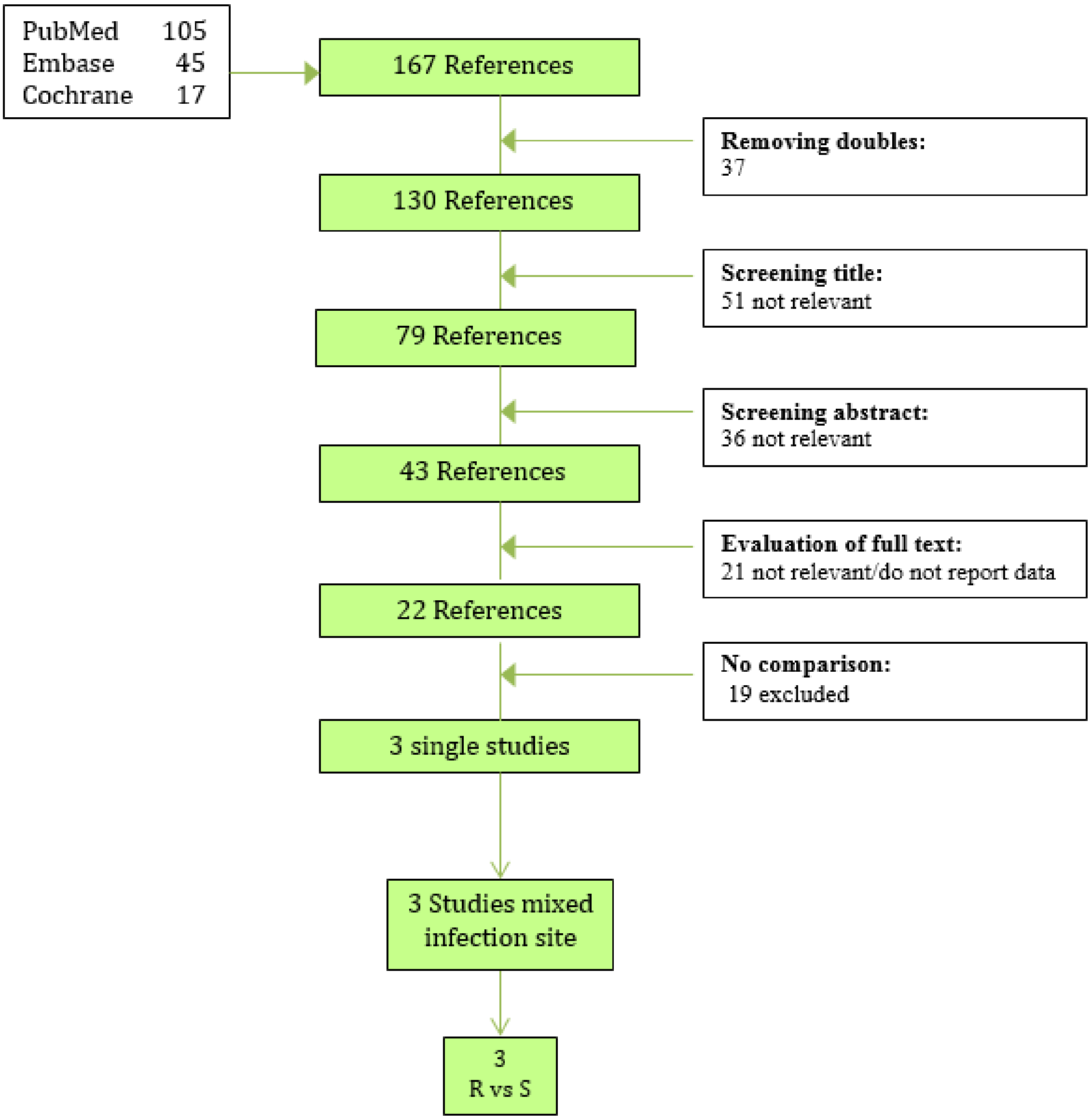

The following table includes studies reporting mortality data for CoRKP. All information is reported as was presented in the original papers.

Table 31: Short extraction table of original articles on mortality of CoRKP infected patients vs CoSKP infected patients for mixed site of infection

Nr	Author	Study Period	Study type	Outcome	Location	Period of outcome	Population	LOS (days)	Matching	Notes
1	Marchaim 2010[131]	09/08-08/09	Case-cohort Study	Mort. rate cases=40%<br>Mort. rate controls=26%	Detroit, USA	hospitaliz	5 cases<br>60 controls	Mean±SD<br>Cases=33±23<br>Controls=30±23	none	Outbreak patients, controls were CRKP
2	Zarkotou 2010[132]	04/08-06/09	Matched Case-Control Study	Mort. rate cases=69.2%<br>Mort. Rate controls=35.9%<br>Mort. Attributed to KPC-KP* =37.5% in both groups	Piraeus, Greece	hospitaliz	13 cases<br>39 controls	Mean±SD<br>Cases= 20.5±20.8<br>Controls= 16.4±12.3	Infection site	*not explained how was defined
3	Capone 2013[133]	12/10-05/11	Multicentre Prospective Study	Mort. rate cases=40.6%<br>Mort. rate controls=20.3%	Rome, Italy	hospitaliz	35 cases<br>62 controls	Not specific	none	all patients were CRKP

The following table reports an article identified after the review was concluded, but we report it since it fulfils our eligibility criteria. The article from Rojas et al.[134], studied patients with CoRKP and CoSKP infection in different site of infection.

Table 32: Short extraction table of original articles on mortality of CoRKP infected patients vs CoSKP infected patients for mixed site of infection

Nr	Author	Study Period	Study type	Outcome	Location	Period of outcome	Population	LOS (days)	Matching	Notes
1	Rojas[134]	12/11-10/14	Prospective nested cohort study	Mort. rate cases=51%<br>Mort. rate controls=39%<br>aHR(95%CI)=3.48 (1.77-6.57) p<0.001	USA	30 days	31 cases<br>215 controls	Median(IQR)<br>Cases=8(5-12)<br>Controls=13(7-26)	none	All samples were resistant to carbapenems too

Between the non-eligible articles, many reported mortality data for CoRKP infections in a group of selected patients without a comparison group. In these studies[28,135-139] the overall case fatality for the CoRKP infected patients ranged between 20 and 100%. Similar results were reported also in the review of Ah et al. in 2014[140], while the infection related deaths for CoRKP infected patients in range between 25-71%.

Many papers reported outbreak studies or characterization of the CoRKP baceria, and few of them included data on mortality. We excluded the majority of studies reporting mortality data because they did not report a comparison group.

The colistin resistant Klebsiella pneumoniae infections are a relative new research topic, and there is a lot of interest on studying them. Many studies on the risk factor and the mortality rate of the infections are published, but few of them try to estimate the attributable mortality or the attributable length of stay of this resistance.

9. Third-generation Cephalosporin resistant *Klebsiella pneumoniae*

The resistance to the 3rd generation of cephalosporin is due to the production from the bacteria of extended-spectrum beta-lactamase enzymes (ESBL). These enzymes can deactivate extended spectrum (3rd generation) cephalosporins (e.g., ceftazidime, cefotaxime, and ceftriaxone) and monobactams (e.g., aztreonam).
The guidelines internationally accepted for the detection of ESBL-producing bacteria, including *K. pneumoniae*, have been defined in 1999 by the US-based National Committee for Clinical Laboratory Standards (now the Clinical and Laboratory Standards Institute).
For the purpose of this review, 3rd generation cephalosporin resistant *Klebsiella pneumoniae* (3GCRKP) and ESBL producing *Klebsiella pneumoniae* (ESBL-KP) will be used to define the same phenomenon.

To research the attributable case fatality and the attributable LOS to third-generation cephalosporin resistant *Klebsiella pneumoniae* (3GCRKP) we performed 2 advanced searches on Pubmed using once the mesh term “Klebsiella pneumoniae” AND all fields “third generation cephalosporin”, and the second time the mesh term “Klebsiella pneumoniae” AND all fields “ESBL OR extende-spectrum beta lactamase”. Embase was searched for ‘klebsiella pneumoniae AND third generation cephalosporin’, and ‘Klebsiella pneumoniae AND (ESBL OR extended-spectrum beta-lactamase’). An advanced search on the Cochrane Library was performed using terms “klebsiella pneumonia” AND “third generation cephalosporin”, plus a second search using “klebsiella pneumoniae” AND “ESBL OR extended-spectrum beta-lactamase”. No language restrictions were made, and a filter for research in humans was applied.

The flow diagram, presented in Figure 9 describes the steps in the literature review of 3rd generation cephalorporin resistant *Klebsiella pneumoniae* infections. The results obtained using ESBL and 3rd generation cephalosporin as key words were aggregated.
Though we did not apply any language filter, 2 studies were excluded from the final results since they were in Chinese language.

Once identified the studies reporting relevant data, these were grouped based on the site of infection. The majority of them reported BSI infections, even though not all of them had a control group.

Figure 9: Search for studies on 3rd generation cephalosporin resistant *Klebsiella pneumoniae*. Inclusion and exclusion diagram.

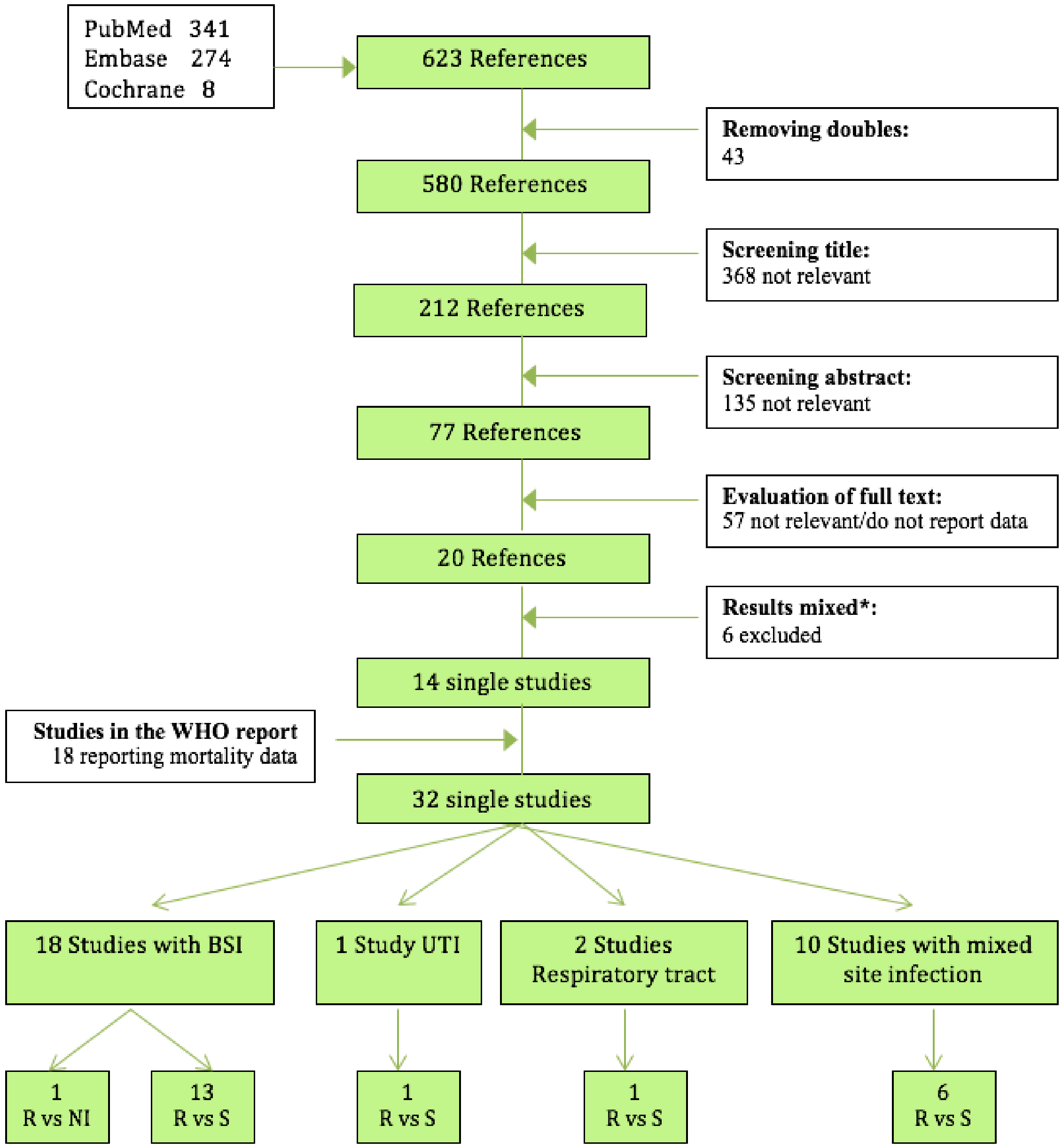

*These studies reported results for ESBL-*Enterobacteriaceae* or together for *K. pneumoniae* and *Escherichia coli*

From the 18 studies focusing on BSI, 13 were eligible for our purposes. 5 studies were discarded because the comparison was different from those in our inclusion criteria. The same was applied to the studies for respiratory tract and with mixed site of infection.

We also identified a systematic review of the literature on the crude mortality of ESBL-KP infections (Viale et al[98]), which did not consider a comparison group. The crude mortality rate for a BSI infections of ESBL-KP in this review ranged between 14.1% and 43.3%.
The following tables include studies reporting results for BSI, UTI and respiratory tract from 3GCRKP, based on the comparison group they used. All information is reported as was presented in the original papers

Table 33: Short extraction table of original articles on BSI mortality of 3GCRKP infected patients vs non infected patients

Nr	Author	Study Period	Study type	Outcome	Location	Period of outcome	Population	Matching criteria	LOS (days)	Notes
1	Gallagher 2014[141]	06/05-10/10	Case-case-control study	Mort. rate cases=32%<br>Mort. rate contr.=13%<br>OR=3.6; 95%CI(1.2 to 10.5)	Pennsylva nia, USA	hospitaliz	111 cases<br>111 controls	Month and medical unit, min 72h hospitaliz	mean cases=63 controls= 20	

Table 34: Short extraction table of original articles on BSI mortality of 3GCRKP infected patients vs 3GCSKP infected patients

Nr	Author	Study Period	Study type	Outcome	Location	Period of outcome	Population	LOS (days)	Notes
1	Lee 2014[53]	01/03-12/09	Retrospective case-control study	a)Mort. rate cases=33.3%<br>Mort. rate controls=24.2%<br>b)Mort. rate cases=33.3%<br>Mort. rate controls=16.3%	Daegu, Korea	a)30 day mortality<br>b)BSI-related mortality	9 cases<br>153 controls	not reported	community acquired infection, study included also EC results
2	Panhotra 2004[142]	07/01-07/03	Retrospective case-control study	Mort. rate cases=60%<br>Mort. controls=6.2%	Saudi Arabia	hospitaliz	10 cases<br>16 controls	cases=44.2<br>controls-24.1	hospital acquired infection
3	Tumbarello 2006[143]	01/99-12/03	Case-case-control study	a) Mort. rate cases= 25%<br>Mort. rate controls=11%<br>b) Mort. rate cases=52%<br>Mort. rate controls=29%	Italy	a) 7 days<br>b) 21 days	48 cases<br>99 controls	mean ± SD<br>cases=34±20<br>controls=31±17	
4	Marra 2006[144]	01/96-05/01	Retrospective cohort study	Mort. rate cases=32%<br>Mort. rate controls=15%	Brazil	15 days	56 cases<br>52 controls		
5	Kang 2004[145]	01/98-12/02	Retrospective cohort study	Mort. rate cases=60%<br>Mort. rate controls=30%	South-Korea	30 days	10 cases<br>20 controls		patients had spontaneous bacterial peritonitis with advanced liver cirrhosis

6	Kim BN 2002[146]	07/96-06/00	Retrospective cohort study	Mort. rate cases=23%<br>Mort. rate controls=20%	South-Korea	hospitaliz	44 cases<br>118 controls	Mean<br>cases=39.6<br>controls=23.9	
7	Ariffin 2000[147]	01/96-12/97	Retrospective cohort study	Mort. rate cases=50%<br>Mort. rate controls=13%<br>OR=6.5; 95%CI(1.1-38.6)	Malaysia	Hospitaliz	16 cases<br>15 controls		-patients are children with febrile neutropia
8	Mosqueda-Gomez 2008[148]	01/93-12/02	Retrospective case-control study	Mort. rate cases=35%<br>Mort. rate controls=26.9%	Mexico	Hospitaliz	17 cases<br>104 controls	mean<br>cases=20.9<br>controls=15.9	
9	Szilagyi 2009[149]	01/05-12/08	Retrospective cohort study	a)Mort. rate cases=36%<br>Mort. rate controls=23%<br>OR=2.5; 95%CI(1.0–5.4)<br>b)Mort. rate cases=18%<br>Mort. rate controls=18%<br>OR=5; 95%CI(1.5–16.2)	Hungary	Hospitaliz	100 cases<br>100 controls	median<br>cases=10.5<br>controls=10	a) all cause mort.<br>b)inf. related mort.
10	Pena 2001[150]	05/93-06/95	Prospective cohort study	a)Mort. rate cases=32%<br>Mort. rate controls=24%<br>b)Mort. rate cases=16%<br>Mort. rate controls=14%	Barcelona, Spain	Hospitaliz	49 cases<br>43 controls		-hospital acquires infection<br>a)overall mortality<br>b)attributable mortality
11	Paterson 2004[151]	01/96-12/97	International prospective study	Mort. rate cases=27%<br>Mort. rate controls=23%	South Africa, Taiwan, Australia, Argentina, the United States, Belgium, and Turkey	14 days	78 cases<br>175 controls		
12	Tuon 2010[152]	01/06-01/09	Cohort study	Mort. rate cases=49.2%<br>Mort. rate controls=41.9%	Brazil	30 days	63 cases<br>41 controls	Mean ± SD<br>cases= 52.2 ± 39.6<br>controls=36.0 ±29.4	patients are older than 12
13	Lee 2011[153]	01/02-09/09	Case-control study	Mort. rate cases=12.1%<br>Mort. rate controls=16%	South Korea	30 days	33 cases<br>219 controls	Mean ± SD<br>cases=26.5 ± 39.0<br>controls=19.9 ± 42.4	the infection had community onset

Table 35: Short extraction table of original articles on UTI mortality of 3GCRKP infected patients vs 3GCSKP infected patients

Nr	Author	Study Period	Study type	Outcome	Location	Period of outcome	Population	Matching	LOS (days)	Notes
1	Brizendine 2015[154]	2006-2012	Retrospective cohort study	Mort. cases=18%<br>Mort. contr.=2%	Cleveland, USA	hospitaliz	22 cases<br>64 controls	Patients had solid organ transplantation	Prior to UTI<br>Median (IQR)<br>cases=8(1-14)<br>controls=1(1-4)	patients had solid organ transplant

Brizendine et al.[108] (Table 35) reports that 4 cases (18%) and 8 controls (13%) had also bacteraemia of ESBL-KP, but these cases were not excluded from the results displayed in table 35.

Table 36: Short extraction table of original articles on respiratory tract infections mortality of 3GCRKP infected patients vs 3GCSKP infected patients

Nr	Study	Study Period	Study type	Outcome	Location	Period of outcome	Population	Matching	LOS (days)	Notes
1	Loh 2006[155]	01/04-12/04	Retrospective cohort study	Mort. rate cases=21.3%<br>Mort. rate controls=12.4%	Malaysia	hospitaliz	47 cases<br>394 controls	None	Median (IQR)<br>cases=14(8-32)<br>controls=5(3-10)	-community and hospital acquired infections

Table 37 presents the results of studies that include patients with different sites of infection. Almost all the articles specify the number of cases for the different infection sites but they do not report different mortalities based on the infection site. When possible, results are presented differentiating the mortality for each site of infection.

Table 37: Short extraction table of original articles on mortality of 3GCRKP infected patients vs 3GCSKP infected patients for different infection sites

Nr	Study	Study Period	Study type	Outcome	Location	Period of outcome	Population	LOS (days)	Matching	Notes
1	Gomes 2006[156]	01/98-12/98	Retrospective cohort study	Mort. rate cases=20.6%<br>Mort. rate controls=21.3%	Brazil	21 days	68 cases<br>75 controls	no data	none	
2	Lin 2003[157]	05/01-09/01	Case-control study	Mort. rate cases=27.9%<br>Mort. rate controls=20.9%	Taiwan	hospitaliz	43 cases<br>86 controls	mean(range)<br>cases=45 (0-143)<br>controls=18 0-82)	type of speciment, date of isolation	
3	Kuo 2007[158]	01/00-10/05	Case-control study	Mort. rate cases=15%<br>Mort. rate controls=13%	Taiwan	30 days	54 cases<br>54 controls	Mean ± SD<br>cases=34.4 ± 5.2<br>controls=14.3± 2.3	type of speciment, date of isolation	
4	Chiu 2005[159]	01/01-12/01	Case-control study	Mort. rate cases=20%<br>Mort. rate controls=12.5%	Taiwan	hospitaliz	15 cases<br>16 controls	Not reported	none	patients are in NICU
5	Huang 2007[160]	01/00-12/02	Prospective cohort study	Mort. rate cases=10.5%<br>Mort. rate controls=0%	China	30 days	19 cases<br>12 controls	Not reported	none	
6	BARTF* 2002[24]	1999	Survey	Mort. rate cases=44%<br>Mort. rate controls=33%	Brooklyn, USA	hospitaliz	9 cases<br>9 controls	median:<br>cases=20<br>controls=33.5	Site of infection	

* Brooklyn Antibiotic Resistance Task Force

The following table reports a study which considers the mortality of BSI infections caused by third-generation cephalosporine resistant (3GCRE) and susceptible (3GCSE) *Enterobacteriaceae* . The bacteria included were *Escherichia coli*, *Klebsiella* spp. and *Proteus* spp. But the results are not separated for each strain. Even though the study is not fully eligible for our purposes we decided to include it in this report because of its methodological value.

Table 38: Short extraction table of original articles on BSI mortality of 3GCRE infected patients vs 3GCSE infected patients vs non-infected (NI)

Nr	Study	Study Period	Study type	Outcome	Location	Period of outcome	Population	LOS (days)	Matching	Notes
1	Stewardson 2016[161]	01/10-12/11	Multicentre retrospective cohort study	Mort. rate cases=16.1%<br>Mort. rate controls=10.1%<br>Mort. rate NI=1.7%<br>HR=1.39/1.43/1.63*	Europe	hospitaliz	360 cases<br>2100 controls<br>604797 NI	no specific data, see full-text for modelling values*	none	* the study models the excess in LOS and mortality between groups proposing 3 models for each

The terms ESBL and extended-spectrum beta-lactamase are widely used in the literature. Our very broad searching strategy produced 580 unique studies but not all the studies were eligible for our study question. Many of the detected studies were abstracts proposed in congresses and never finalised with a peer reviewed paper. These were not included in the results.
All studies were performed in healthcare settings, though some of them report cases of infection that originated in the community.

10. Methicillin resistant Staphylococcus aureus

Information on the attributable case fatality and the attributable LOS to meticillin resistant *Staphylococcus aureus* (MRSA), was initially retrieved from the WHO Antimicrobial Resistance Global report on surveillance[162]. We identified the 140 articles included in their literature review, accessed the full text of these papers and extracted the useful data for our study.

The WHO report identifies 46 studies that report the bacterium attributable mortality. The criteria used to classify the deaths due to the infection may vary from study to study, but all the 46 studies considered them as attributable. Some of the studies reported also the all-cause mortality rates for the cases and controls.

The 46 studies are actually 45 unique articles, since one article, Kuint et al. 2007[163], is reported twice, considering once MRSA cases (which was considered in our review) and the second time multi-drug resistant MRSA. We classified the 45 studies based on the site, Figure 10.1 shows their distributions.

All the studies included a case group of MRSA infected patients and a comparison group of methicillin susceptible *Staphylococcus aureus* (MSSA).

Figure 10.1. Classification of 46 studies that analyse the attributable mortality to MRSA, reported in the WHO Antimicrobial resistance report 2014[162], based on the site of infection

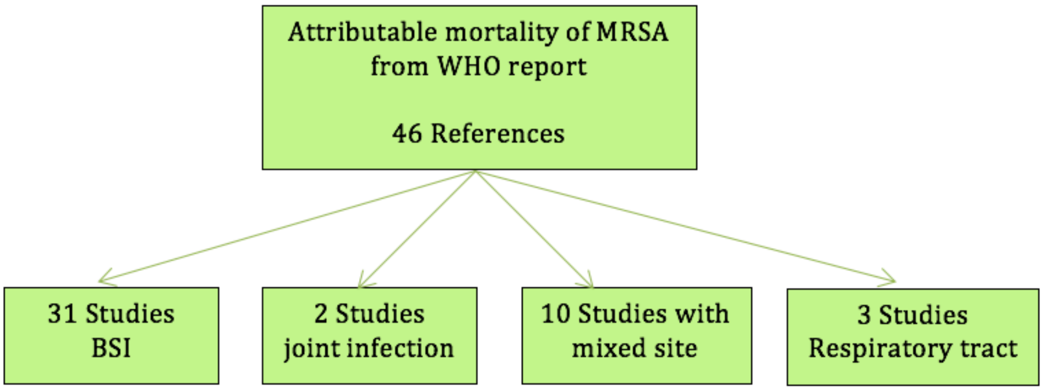

The WHO report considers that the data of these studies were sufficiently similar and of sufficient quality to include them in meta-analyses. The conclusion of this meta-analysis was the following; "There was a significant increase in bacterium-attributable mortality for patients with MRSA infections (RR 1.64, 95% CI: 1.43 to 1.87, P < 0.00001)."[162] The absolute effect of MRSA compared to MSSA is 108 more deaths per 1,000 cases (from 73 to 147).

The following tables report the results of the 46 papers based on the site of infection.

Table 39: Short extraction table of original articles on BSI mortality of MRSA infected patients vs MSSA infected patients

Nr	Author	Study Period	Study type	Outcome	Location	Period of outcome	Population	Matching criteria	LOS (days)	Notes
1	Guilarde 2006[164]	01/00-12/01	Retrospective cohort study	Mort. rate cases=23.5%<br>Mort. rate controls=21.6%	Brazil	30 days	61 cases<br>50 controls	none		
2	Castillo 2012[165]	01/05-12/08	Multicentre cohort study	Mort. rate cases=57%<br>Mort. rate controls=46.2%<br>Attribut. mort. cases=71.7%<br>Attribut. mort. controls=57%	Colombia	30 days	186 cases<br>186 controls	hospital, year	median(IQR)<br>(before BSI)<br>cases=12(2-15)<br>controls=5(1-9)	
3	Lawes 2012[166]	01/06-12/10	Retrospective cohort study	a)Mort. rate cases=20%<br>Mort. rate controls=17%<br>b)Mort. rate cases=25%<br>Mort. rate controls=21%	Scotland	a)30 days<br>b)hospitaliz	659 cases<br>208 controls	none		
4	Blot 2002[167]	01/92-12/98	Retrospective cohort study	a)Mort. rate cases=43%<br>Mort. rate controls=16%<br>b)Mort. rate cases=53%<br>Mort. rate controls=18%<br>c)Mort. rate cases=64%<br>Mort. rate controls=24%	Belgium	a)15days<br>b)30 days<br>c)hospitaliz	47 cases<br>38 controls	APACHE II score, diagnostic category	(in ICU) mean±SD<br>cases=37±28.7<br>controls=18±16.2<br>median (IQR)<br>cases=28(12-54)<br>controls=14(3-24)	
5	Talon 2002[168]	97-98	Prospective cohort study	Mort. rate cases=43.3%<br>Mort. rate controls=20.3%	France	hospitaliz	30 cases<br>69 controls	none	no data	
6	Soriano 2000[169]	01/91-12/98	Cohort study	Mort. rate cases=22%<br>Mort. rate controls=9%	Spain	hospitaliz	225 cases<br>683 controls	pre-existing comorbidities, underlying disease, LOS, and age	mean<br>cases=18<br>controls=8	Patients with nosocomial SA bacteraemia
7	Selvey 2000[170]	92-97	Retrospective cohort study	Attribut. mort. rate cases=13.8<br>Attribut. mort. rate controls=8.2%	Australia	hospitaliz	188 cases<br>316 controls	none	(prior BSI) median<br>cases=16<br>controls=6	Inpatients with nosocomial SA bacteraemia
8	Rubio-Terres 2009[171]	01/05-12/05	Retrospective multi	a)Mort. rate cases=61.7%<br>Mort.rate controls=56.2%<br>b)Mort. rate cases=29.8%	Spain	hospitaliz	121 cases<br>245 controls	none	mean (95%CI)<br>cases=24.88<br>(19.9–29.9)	SA bacteraemia patients<br>a) related to

			centre cohort study	Mort. rate controls=34.4%<br>c)Mort. rate cases=8.5%<br>Mort. rate controls=9.4%					controls=22.66 (18.8–26.5)	infection;<br>b)underlying conditions;<br>c)other causes
9	Romero-Vivas 1995[172]	01/90-09/93	Prospective cohort study	Mort. rate cases=58.3%<br>Mort. rate controls=32%	Spain	hospitaliz	84 cases<br>100 controls	none	mean<br>cases=32<br>controls=14	N SA bacteraemia patients
10	Reshad 1994[173]	01/83-12/91	Cohort study	Mort. rate cases=73.9%<br>Mort. rate controls=47.9%	Japan	no data*	46 cases<br>48 controls	no data*	no data*	Patients with SA septicaemia<br>* not possible to extract the data because the full-text is in Japanese
11	Quilty 2009[174]	07/04-06/07	Retrospective audit	Mort. rate cases=60%<br>Mort. rate controls=40%	Australia	no data	5 cases<br>5 controls	Underlying disease	no data	Chemotherapy-induced febrile neutropenia patients with SA sepsis
12	Hakim 2007[175]	01/00-12/04	Retrospective cohort study	Mort. rate cases=12%<br>Mort. rate controls=0%	New York, USA	hospitaliz	14 cases<br>22 controls	none	no data	Bacteraemia in children
13	Nickerson 2006[176]	06/03-12/04	Retrospective cohort study	Mort. rate cases=69%<br>Mort. rate controls=44%<br>*Attribut. mort.=48%	Thailand	hospitaliz	36 cases	none	no data	*it is not defined how it calculated
14	Kim 2003[177]	01/98-11/01	Retrospective cohort study	Mort.rate cases=40%<br>Mort.rate controls=33.3%	Seul, Korea	hospitaliz	127 cases<br>111 controls	none	(before inf.)<br>mean±SD<br>cases=33.9±34.6<br>controls=17.7±34.3	
15	Melzer 2003[178]	01/95-12/00	Cohort study	a)Mort. rate cases=11.8%<br>Mort. rate controls=5.1%<br>b)Mort. rate cases=17.8%<br>Mort. rate controls=8.5%	England, UK	hospitaliz	433 cases<br>382 controls	none	no data	nosocomial BSI,<br>a)mort. due to inf.<br>b)mort. due to other causes

16	Kuint 2007[163]	01/93-12/03	Retrospective cohort study	a)Mort.rate cases=9%<br>Mort.rate controls=8%<br>b)Mort. rate cases=27%<br>Mort. rate controls=25%	Tel Aviv, Israel	hospitaliz	11 cases<br>12 controls	none	no data	patients in neonatal ICU<br>a)sepsis related mort.<br>b)all cause mort.
17	Khatib 2006[179]	01/02-12/02	Prospective observational study	Attribut. mort=12.2%*	Detroit, USA	hospitaliz	174 cases<br>168 controls	none	no data	*Attributable mortality was defined as death with positive blood culture or persistent sepsis
18	Lodise 2005[180]	01/99-01/01	Retrospective cohort study	Mort. rate cases=30.6%<br>Mort. rate controls=15.3%	Michigan, USA	30 days	170 cases<br>183 controls	none	mean<br>cases=20.1<br>controls=13.7	
19	Nickerson 2009[181]	11/06-11/07	Prospective observational study	Mort. rate cases=68%<br>Mort. rate controls=46%	Thailand	12 weeks	27 cases<br>71 controls	none	no data	
20	Harbarth 1998[182]	01/89-12/95	Case-control study	a)Mort. rate cases=18%<br>Mort. rate controls=24%<br>b)For both groups=34%	Geneva, Switzerland	hospitaliz	38 cases<br>38 controls	age, LOS, nr. comorbidities, McCabe score, Jackson score	(after BSI)<br>median (range)<br>cases=30(1-141)<br>controls=23(1-178)	a) mort. caused by infection<br>b)all cause mort.
21	Cunney 1994[183]	12/91-05/93	Retrospective cohort study	Mort. rate cases=22%<br>Mort. rate controls=3%	Dublin, Ireland	hospitaliz	18 cases<br>60 controls	none	no data	hospital acquired inf.
22	Das 2006[184]	11/01-12/02	Prospective cohort study	a)Mort. rate cases=33%<br>Mort. rate controls=16%<br>b)Mort. rate cases=56%<br>Mort. rate controls=39%	Birmingham, UK	3 months	84 cases<br>56 controls	none	(before BSI)<br>median<br>cases=14<br>controls=8	a)attribut. mort<br>b)all cause mort.
23	Austin 2003[185]	01/94-01/99	Matched case-control study	a)Mort. rate cases=36%<br>Mort. rate controls=20%<br>b)Mort. rate cases=50%<br>Mort. rate controls=69%	Ontario, Canada	hospitaliz	50 cases<br>50 controls	sex, age, primary diagnoses	mean<br>cases=44.1<br>controls=33.9	Inpatient bacteraemia<br>a)attribut. mort<br>b)all cause mort.
24	Conterno 1999[186]	01/91-09/92	Nested case-control study	*Mort. rate cases=10-90%<br>Mort. rate controls=4-76%	Brazil	14 days	90 cases<br>46 controls	none	no data	*the mortality is estimated with predictive models

25	Hill 2008[187]	06/00-12/06	Cohort study	Mort. rate cases=56%<br>Mort. rate controls=30%<br>a)Mort. rate cases=25%<br>Mort. rate controls=67%<br>b)Mort. rate cases=86%<br>Mort. rate controls=100%	Belgium	6 months	16 cases<br>56 controls	Underlying disease	no data	Infective endocarditis<br>a)CA inf.<br>b)antimicrobial therapy due to a contraindication to surgery
26	Ganga 2009[188]	11/05-12/06	Prospective observationa l study	a)Mort. rate cases=23.9%<br>Mort. rate controls=8.9%<br>b)Mort. rate cases=7.4%<br>Mort. rate controls=2.2%	Michigan, USA	hospitaliz	163 cases<br>90 controls	none		inpatients with SA bacteraemia<br>a) all cause mort.<br>b) attributable mort.
27	Burke 2009[189]	2001-2006	Retrospctive study	Mort. rate cases=10%<br>Mort. rate controls=4%	California, USA	hospitaliz	29 cases<br>121 controls	age	mean<br>cases=36<br>controls=16.3<br>median(IQR)<br>cases=38(16-73)<br>controls=16(7-42)	all patients were children
28	de Oliveira 2002[190]	90-91/95-96	Nested case-control study	Mort. rate cases=45.9%<br>Mort. rate controls=20.7%	Brazil	14 days	159 cases<br>92 controls	none	no data	patients with <14 years
29	Lesens 2003[191]	05/01-05/02	Prospective cohort study	Mort. rate cases=34%<br>Mort. rate controls=20.4%	France	hospitaliz	53 cases<br>113 controls	none	no data	
30	O'kane 1998[192]	01/93-12/93	Retrospectiv e study	Mort. rate cases=9% *<br>Mort. rate controls=6%*	Australia	hospitaliz	32 cases<br>73 controls	none	post infection mean<br>cases=24<br>controls=19	*directly attributable mortality
31	De Kraker 2011[193]	07/07-06/08	Prospective parallel matched-cohort study	R vs NI (Cohort I)<br>a) Mort. rate resist= 31%<br>Mort. rate NI=8%<br>aOR=4.4<br>b) Mort. rate resist= 36%<br>Mort. rate NI=9%<br>aHR=3.1<br>S vs NI (Cohort II)<br>a) Mort. rate sensit=22%	Europe	a)30 days<br>b)hospitaliz	I) 248 resistant<br>453 NI<br>II) 618 sensitive<br>1170 NI	LOS	median (IQR)<br>Cohort I<br>resistant=16 (6-32)*<br>NI=7(3-18)*<br>Cohort II<br>sensitive=15(7-26)*<br>NI=8(4-14)*	

				Mort. rate NI=8%<br>aOR=2.4<br>b) Mort. rate sensit=23%<br>Mort. rate NI=7%<br>aHR=3.1<br>R vs S<br>a) OR=1.8<br>b) HR= 1.1					excess LOS<br>R vs NI= 9.2*<br>S vs NI= 8.6*<br>R vs S= 0.6	

Table 40: Short extraction table of original articles on joint infections mortality of MRSA infected patients vs MSSA infected patients

Nr	Author	Study Period	Study type	Outcome	Location	Period of outcome	Population	Matching criteria	LOS (days)	Notes
1	Salgado 2007[194]	01/95-12/04	Retrospective cohort study	Mort. rate both=0%	South Carolina, USA	hospitaliz	31 cases<br>12 controls	none	Median cases=15<br>controls=10	Patients with prosthetic joint infection from SA
2	Al-Nammari 2007[195]	06/00-06/05	Retrospective study	a)Mort. rate cases=13%<br>Mort. rate controls=5%<br>b)Mort. rate cases=26%<br>Mort. rate controls=7%	England, UK	a) sepsis related<br>b)6 months	15 cases<br>43 controls	septic arthritis	no data	

Table 41: Short extraction table of original articles on mortality of MRSA infected patients vs MSSA infected patients for different infection sites

Nr	Author	Study Period	Study type	Outcome	Location	Period of outcome	Population	Matching criteria	LOS (days)	Notes
1	Rello 2013[196]		Retrospective cohort study	a)Mort. rate cases=60%<br>Mort. rate controls=50%<br>b)Mort. rate cases=33.3%<br>Mort. rate controls=10%	a)Latin America<br>b)Europe	hospitaliz	a)5 cases<br>6 controls<br>b)15 cases<br>30 controls	VAP	no data	patients in ICU

2	Spindel 1995[197]	01/87-12/91	Observational analitical study	Mort. rate cases=10.7%<br>Mort. rate controls=10%	Oregon, USA	hospitaliz	20 cases<br>48 controls		mean<br>cases=15<br>controls=12	Veterans' nursing home, elderly men with severe underlying diseases
3	Davis 2007[198]	10/03-12/05	Prospective cohort study	No death related to infections	USA		102 cases<br>102 controls	none		Patients with community associated SA infections
4	Graffunder 2002[199]	97-99	Case-control study	a)Attibut. mort. rate cases=8.3%<br>Attribut. mort. rate controls=5.7%<br>b)Mort. rate cases=28.8%<br>Mort. rate controls=19.5%	USA	hospitaliz	121 cases<br>123 controls	none	(before culture)<br>mean(range)<br>cases=18.8(2-98)<br>controls=8.4(2-45)	nosocomial infection<br>a) to the infection<br>b) all cause
5	Priest 2005[200]	94-00	Retrospective case-control study	a)Mort. rate cases=55%<br>Mort. rate controls=4%<br>b)Mort. rate cases=27%<br>Mort. rate controls=0%	North Carolina, USA	hospitaliz	11 cases<br>24 controls	vertebral osteomy elitis	no data	Inpatients with SA haematogenous vertebral osteomyelitis<br>a)all cause mort.<br>b)inf. related mort.
6	Hershow 1992[201]	01/89-12/89	Retrospective cohort study	Mort. rate cases=1%<br>Mort. rate controls=0%	Illinois, USA	hospitaliz	22 cases<br>22 controls	none	median<br>cases=14<br>controls=10	Adults with nosocomial SA infection
7	Hulten 2010[202]	08/01-07/07	Retrospective cohort study	Mort. rate cases=17.2%<br>Mort. rate controls=7.0%*	Texas, USA	hospitaliz	29 cases<br>71 controls	none	mean<br>cases=57.6<br>controls=68.2<br>median(range)<br>cases=29 (3–339)<br>controls=56 (0–158)	patients were children<br>*mortality was calculated just for bacteraemia and catheter associated inf., not all cases and controls of the study
8	Joo 2012[203]	2007-2009	Matched case-control study	a)Mort. rate cases=9.5%<br>Mort. rate controls=4.8%<br>b)Mort. rate cases=9.5%<br>Mort. rate controls=3.6%	Seul, Korea	hospitaliz	84 cases<br>84 controls	admissio n date	median(range)<br>cases=20 (2–202)<br>controls=22 (2–158)	Community-onset SA infection,<br>a) all cause mort.<br>b)inf. Related mort.

9	Capitano 2003[204]	01/96-08/00	Retrospective cohort study	Mort. rate cases=10% * Mort. rate controls=10%*	Connecticut, USA	hospitaliz	41 cases 49 controls	long-term care facility	no data	all patients were in long term care facility * infection related mortality
10	Clancy 2005[205]	01/02-06/04	Retrospective cohort study	Mort. rate cases=0% Mort. rate controls=1.5%	Colorado, USA	30 days	57 cases 136 controls	CA-SA	no data	infections were community-acquired

Table 42: Short extraction table of original articles on mortality of MRSA infected patients vs MSSA infected patients with respiratory tract infection

**Nr**	**Author**	**Study Period**	**Study type**	**Outcome**	**Location**	**Period of outcome**	**Population**	**Matching criteria**	**LOS (days)**	**Notes**
1	Rello 1994[206]	01/91-06/93	Retrospective cohort study	a)Mort. rate cases=61.7% Mort. rate controls=56.2% b)Mort. rate cases=18.1% Mort. rate controls=26.3%	Spain	hospitaliz	11 cases 38 controls	none	no data	patients with VAP a)mort. related to infection; b)mort. non related to infection
2	Gonzalez 1999[207]	01/90-12/95	Retrospective cohort study	a)Mort. rate cases=25% Mort. rate controls=14.8% b)Mort. rate cases=56.3% Mort. rate controls=40.7%	Spain	a) first 48h b) due to infection	32 cases 54 controls	bacteremic pneumoniae	mean±SD cases=44.4±27.7 controls=30.3±21.7	
3	Pujol 1998[208]	01/90-12/94	Prospective cohort study	Mort. rate cases=56.1% Mort. rate controls=37.8%	Spain	hospitaliz	41 cases 98 controls	Underlying disease	prior inf. mean±SD cases=26.2±17.7 controls=5±6.4*; 15.8±13.3**	Mechanically ventilated ICU patients who developed SA pneumonia, *early onset; **late onset

We also performed a separate literature review using time filters from April 2013 up to the 12th of December 2016 to detect new articles.

We performed an advanced search on Pubmed using the mesh term “MRSA” AND all fields “mortality”. On Embase was searched for ‘MRSA’ AND ‘mortality’ and an advanced search was performed on the Cochrane Library was performed using terms “MRSA” AND “mortality”. We applied a filter to include only human studies, and included only published articles. No language restrictions were made, and no further filters were applied.

The flow diagram presented in Figure 10.2 describes the steps in the literature review of MRSA infections.

Once identified the studies reporting relevant data, these were grouped based on the site of infection. The majority reported BSI infections, even though not all of them had a control group.

Figure 10.2. Search for studies on MRSA infections. Inclusion and exclusion diagram.

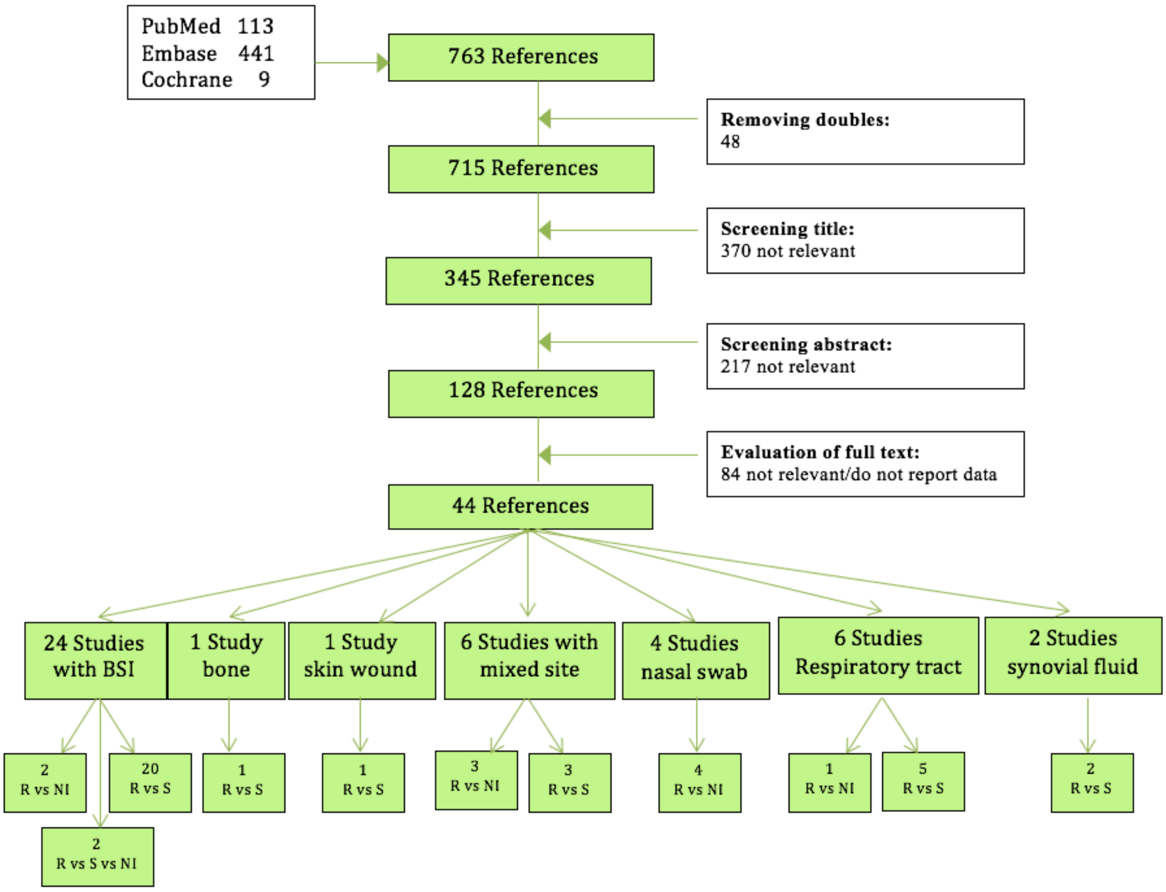

As shown in Figures 10.2., even though we applied a time filter and looked for papers containing the term “mortality”, which makes the research very specific, more than 700 unique papers were detected. After the different exclusion steps the vast majority of them were not eligible for the purposes of our study, but we still could include 44 articles. This is the highest number of eligible articles among all the reviews performed.

The following tables include studies reporting results for the different site of infection based on the comparison group they used. All information is reported as was presented in the original papers.

Table 43: Short extraction table of original articles on mortality of patients with MRSA BSI infections vs non-infected patients

Nr	Author	Study Period	Study type	Outcome	Location	Period of outcome	Population	Matching criteria	LOS (days)	Notes
1	Atmaca 2014[128]	01/04-12/11	Nested case-control study	Mort. rate cases= 47.5%<br>Mort. rate controls=29.3%	Turkey	hospitaliz	99 cases<br>99 controls	BSI, ward, period of time	median<br>cases=59<br>controls=8	data extracted from original paper in Turkish with help of google translate
2	McMaster 2015[209]	04/07-11/13	Prospective study	a) Mort. rate cases= 19%<br>Mort. rate controls=21.2%<br>b) Mort. rate cases= 32.8%<br>Mort. rate controls=34.5%	Glasgow, UK	a) hospitaliz<br>b) 180 days	58 cases<br>174 controls	age, APACHE II, LOS in ICU, surgical/non-surgical status	in ICU<br>median (range)<br>cases=16.6 (1.5-124.1)<br>controls=16.9 (1.9-92.1)	all patients were in ICU

Table 44: Short extraction table of original articles on BSI mortality of MRSA infected patients vs MSSA infected patients and vs non infected patients

Nr	Author	Study Period	Study type	Outcome	Location	Period of outcome	Population	Matching criteria	LOS (days)	Notes
1	Stewardson 2016[161]	01/20-12/12	Multicentre retrospective cohort study	Mort. rate resistant=22.1%<br>Mort.rate sensitive=16.8%<br>Mort.rate NI=1.7%<br>R vs NI:<br>HR*=3.81/2.61/2.42<br>R vs S: HR*=1.19/1.20/1.26	Europe	hospitaliz	163 resistant<br>885 sensitive<br>604797 NI	none	no specific data, see full-text for modelling values*	* the study models the excess in LOS between groups proposing 3 models for each
2	Yasmin 2016[210]	01/12-10/14	Case-case-control study	a)Mort. rate resist= 22%<br>Mort. rate sensit=19.5%<br>Mort. rate NI=1.7%<br>b)Mort. rate resist=19.5%<br>Mort. rate sensit=10.3%	New York, USA	hospitaliz	118 resistant<br>145 sensitive<br>118 NI	date of hosp.	no specific data	a) mortality during hospitalisation<br>b) attributable mortality

Table 45: Short extraction table of original articles on BSI mortality of MRSA infected patients vs MSSA infected patients

Nr	Author	Study Period	Study type	Outcome	Location	Period of outcome	Population	Matching criteria	LOS (days)	Notes
1	De la Calle 2016[211]	01/00-12/14	Retrospective study	a) Mort. rate cases= 40.5%<br>Mort. rate controls=37.5%<br>b) Mort. rate cases= 50%<br>Mort. rate controls=44.6%	Spain	a) 30 days<br>b) infection related	42 cases<br>56 controls	pneumonia	no data	all patients had bacteremic pneumonia
2	De Rosa 2016[212]	2010-2011	Retrospective study	Mort. rate cases= 17.9%<br>Mort. rate controls=14.3%	Italy	21 days	106 cases<br>83 controls	BSI	no data	
3	Deodhar 2015[213]	05/11-06/12	Prospective study	Mort. rate cases= 21%<br>Mort. rate controls=21.3%	India	hospitaliz	40 cases<br>61 controls	none	no data	
4	Dolapo 2014[214]	2000-2009	Retrospective study	a) Mort. rate cases= 0%<br>Mort. rate controls=9.8%<br>b) Mort. rate cases= 15.7%<br>Mort. rate controls=5.2%	Tennessee, USA	14 days	a) 10 cases<br>31 controls<br>b) 64 cases<br>51 controls	NICU	mean±SD<br>a) cases=80.6±42.4<br>controls=87.4±40.6<br>b) cases=53.6±36.6<br>controls=55.7±30.2	patients were in NICU<br>a) period A (2000-2003)<br>b) period B (2004-2009
5	Fortuin-de Smidt 2015[215]	09/12-09/13	Cross-sectional study	Mort. rate cases= 29.1%<br>Mort. rate controls=14.3%	South Africa	hospitaliz	86 cases<br>154 controls	none	median (IQR)<br>cases=38 (14–64)<br>controls=19 (7–33)	
6	Kim D.H 2014[216]	06/06-02/11	Retrospective study	Mort. rate cases= 62%<br>Mort. rate controls=0%<br>OR=0.158; 95%CI(0.047–0.534)	Tennessee, USA	hospitaliz	29 cases<br>8 controls	cardiac implant	no specific data	all patients had Cardiac Implanted Electronic Device-Related Infective Endocarditis
7	Kobayashi 2014[217]	04/04-03/11	Retrospective cohort study	Mort. rate cases= 39.7%<br>Mort. rate controls=30.7%	Japan	90 days	151 cases<br>189 controls	none	no data	
8	Lee J.Y 2014[218]	01/04-12/12		1-a)Mort. rate cases= 3.2%<br>Mort. rate controls=3.6%<br>1-b) Mort. rate cases= 6.5%<br>Mort. rate controls=13.8%<br>1-c) Mort. rate cases= 16.1%	South Korea	a) 1 week<br>b) 30 days<br>c) 12 weeks	31 cases<br>138 controls		median (IQR)<br>cases=47 (19–87)<br>controls=24 (14–44.5)	patients had CA-infections<br>1-a/b/c) all-cause mortality<br>2-a/b/c) infection related mortality

				Mort. rate controls=19.6%<br>2-a) Mort. rate cases= 0%<br>Mort. rate controls=3.6%<br>2-b) Mort. rate cases= 3.2%<br>Mort. rate controls=9.4%<br>2-c) Mort. rate cases= 6.5%<br>Mort. rate controls=13%						
9	Manandhar 2016[219]	01/10-06/13	Retrospective study	a) Mort. rate cases= 26.7%<br>Mort. rate controls=38.1%<br>OR=0.62; 95%CI(0.15–2.61)<br>b) Mort. rate cases= 26.7%<br>Mort. rate controls=42.9%<br>OR=0.51; 95%CI(0.12–2.14)	Pittsburgh, USA	a) 30 days<br>b) 90 days	15 cases<br>21 controls	bacteruria	no specific data	all patients had S. aureus bacteruria also
10	McMullan 2016[220]	01/07-12/12	Prospective cohort study	a) Mort. rate cases= 3.5%<br>Mort. rate controls=2.5%<br>b) Mort. rate cases= 8.5%<br>Mort. rate controls=4.1%	Australia & New Zeeland	a) 7 days<br>b) 30 days	142 cases<br>931 controls	age	median (IQR)<br>cases=17 (8-38)<br>controls=14 (7-33)	patients were children
11	Nagao 2016[221]	2006-2014	Retrospective cohort study	Mort. rate cases= 9%<br>Mort. rate controls=5.8%	Japan	30 days	199 cases<br>278 controls	BSI	no data	
12	Naidoo 2013[222]	01/07-12/11	Retrospective study	MRSA OR (95%CI)<br>univariate=3.71 (1.77-7.76)*<br>multivariate=3.76 (1.12-12.67)*<br>*(p<0.05)	South Africa	30 days	95 cases<br>270 controls	BSI	no specific data	the mortality was not available in %
13	Ong 2013[223]	01/98-12/07	Retrospective cohort study	Mort. rate cases= 63%<br>Mort. rate controls=39%<br>HR=1.37; 95%CI(0.95–1.97)	Australia	1 year	76 cases<br>363 controls	none	no data	the study aimed the log-term survival rate
14	Park 2013[224]	1998-2009	Review and Meta-analysis	OR fixed= 2.33; 95%CI(1.42-3.82)	World	hospitaliz	191 cases<br>476 controls	age	no data	all patients were neonates and children
15	Simsek 2014[225]	01/04-06/12	Retrospective study	Mort. rate cases= 49%<br>Mort. rate controls=15%<br>OR=12.117; 95%CI(3.159 46.475)	Turkey	hospitaliz	13 cases<br>47 controls	Poststernotomy Mediastinitis	no specific data	all patients had Poststernotomy Mediastinitis

16	Thaden 2015[226]	1997-2012	Retrospective study	1-a)Mort. rate cases= 3%<br>Mort. rate controls=4%<br>1-b) Mort. rate cases= 8%<br>Mort. rate controls=8%<br>1-c) Mort. rate cases= 11%<br>Mort. rate controls=10%<br>2-a) Mort. rate cases= 4%<br>Mort. rate controls=2%<br>2-b) Mort. rate cases= 9%<br>Mort. rate controls=3%<br>2-c) Mort. rate cases= 12%<br>Mort. rate controls=4%	North Carolina ,USA	a) 7 days<br>b) 30 days<br>c) hospitaliz	1) 567 cases<br>2047 controls<br>2) 280 cases<br>445 controls	age, ICU	no data	all patients were infants in ICU<br>1) with adequate therapy<br>2) with inadequate therapy
17	Theodorou 2013[227]	01/89-12/09	Retrospective cohort study	Mort. rate cases= 33.3%<br>Mort. rate controls=24.4%<br>OR=1.55, 95%CI(0.56–4.28)	Germany	hospitaliz	33 cases<br>41 controls	thermal injuries	median cases=28.5<br>controls21	all patients had thermal injuries
18	Wang 2015[228]	01/11-12/13	Retrospective cohort study	a) Mort. rate cases= 47.5%<br>OR=2.249; 95%CI(1.188-4.259)<br>b) Mort. rate cases= 30.5%<br>OR=0.998; 95%CI(0.453-2.200)<br>c) Mort. rate cases=35.3%<br>OR=1.223; 95%CI(0.466-3.210)<br>Mort. rate controls=25.3%<br>Total MRSA OR=1.707	Taiwan	hospitaliz	a) 101 cases<br>b) 59 cases<br>159 controls	none	no data	a) HA-MRSA<br>b) CA-MRSA<br>c) unclassified-MRSA
19	Yaw 2014[229]	07/97-06/07	Observational cohort study	a) Mort. rate cases= 15%<br>Mort. rate controls=12%<br>b) Mort. rate cases= 28%<br>Mort. rate controls=19%<br>c) Mort. rate cases= 75%<br>Mort. rate controls=62%	Australia	a) 8 days<br>b) 30 days<br>c) follow-up*	185 cases<br>397 controls	none	no data	* 01/03/2013
20	Yilmaz 2016[230]	2010-2012	Prospective multicentre study	Mort. rate cases= 22%<br>Mort. rate controls=11.7%	Turkey	28 days	100 cases<br>145 controls	none	no specific data	

Table 46: Short extraction table of original articles on the mortality of MRSA bone infected patients vs MSSA skin wound infected patients

Nr	Author	Study Period	Study type	Outcome	Location	Period of outcome	Population	Matching criteria	LOS (days)	Notes
1	Shoji 2016[231]	01/07-12/11	Retrospective study	Mort. rate cases= 25%<br>Mort. rate controls=5.5%	Japan	hospitaliz	16 cases<br>55 controls	vertebral osteomyelitis	no data	all patients had vertebral osteomyelitis infected by S. aureus

Table 47: Short extraction table of original articles on the mortality of MRSA skin wound infected patients vs MSSA skin wound infected patients

Nr	Author	Study Period	Study type	Outcome	Location	Period of outcome	Population	Matching criteria	LOS (days)	Notes
1	Alizadeh 2014[232]	2008-2010	Cross-sectional study	No deaths reported in any group	Iran	hospitaliz	82 cases<br>32 controls	none	no data	

Table 48: Short extraction table of original articles on mortality of patients with MRSA infections with mixed infection site vs non-infected patients

Nr	Author	Study Period	Study type	Outcome	Location	Period of outcome	Population	Matching criteria	LOS (days)	Notes
1	Balm 2013[233]	06/07-06/11	Retrospective cohort study	a) Mort. rate cases= 16.5%<br>Mort. rate controls=4.6%<br>OR=5.49; 95%CI(2.75-10.95)<br>b) Mort. rate cases= 28.9%<br>Mort. rate controls=12.5%	Singapore	a) 30 days<br>b) 6 months	121 cases<br>716 controls	none	no data	* controls had positive nasal swab for MRSA

				OR=2.94; 95%CI(1.78 – 4.85)						
2	Nelson 2015[234]	10/07-09/10	Retrospective cohort study	a) MRSA aHR (95%CI)=1.420(1.322-1.525)*<br>b) MRSA aHR (95%CI)=1.370(1.231-1.524)*<br>*(p<0.05)	Utah, USA	1 year	a) 3599 cases 366144 controls<br>b) 3592 cases 3592 controls	propensity score (b)	mean<br>a) cases=22.4 controls=9.3<br>b) cases=20.1 controls=23.2	a) full cohort<br>b) propensity score matched
3	Tran 2016[235]	01/10-12/12	Retrospective cohort study	a)Mort. rate cases= 8.2%<br>Mort. rate controls=5.7%<br>b)Mort. rate cases= 8%<br>Mort. rate controls=7%	Canada	hospitaliz	745 cases<br>17649 cosntrols	none	mean<br>a)cases=12.8<br>controls=7.6<br>b) cases=11.9<br>controls=9.1	a) pre-match cohort<br>b) post-match cohort

Table 49: Short extraction table of original articles on mortality of patients with MRSA infections with mixed infection site vs patients with MSSA infections

Nr	Author	Study Period	Study type	Outcome	Location	Period of outcome	Population	Matching criteria	LOS (days)	Notes
1	Ericson 2015[236]	1997-2012	Multicentre retrospective study	a)Mort. rate cases= 11.9%<br>Mort. rate controls=9.6%<br>RR=1.19; 95%CI(0.96-1.49)<br>b)Mort. rate cases= 3.8%<br>Mort. rate controls=3.8%<br>RR=0.90; 95%CI(0.65-1.24)<br>c)Mort. rate cases= 8.7%<br>Mort. rate controls=7.2%<br>RR=1.15; 95%CI(0.90-1.46)	USA	a) before discharge<br>b) 7days<br>c) 30 days	1063 cases<br>2825 controls	NICU	Both groups=64	Patients were in NICU
2	Gimenes 2016[237]	2006-2011	Retrospective study	Mort. rate cases= 33.8%<br>Mort. rate controls=52.17%	Brazil	hospitaliz	71 cases<br>23 controls	ICU	median (IQR)<br>cases=20 (20)<br>controls=19 (17)	all patients were in ICU

3	Kim E.S 2014[238]	01/12-12/12	Prospective study	Mort. rate cases= 16.8%<br>Mort. rate controls=14%	South Korea	30 days	303 cases<br>357 controls	none	no data	the infection had community onset

Table 50: Short extraction table of original articles on mortality of patients colonised with MRSA in the nasal mucosa vs no-colonised patients

Nr	Author	Study Period	Study type	Outcome	Location	Period of outcome	Population	Matching criteria	LOS (days)	Notes
1	Altinbas 2013[239]	04/06-04/07	Prospective study	Mort. rate cases= 18.1%<br>Mort. rate controls=37.3%	Turkey	1 year	11 cases<br>257 controls	ICU	mean<br>cases=18<br>controls=8	the difference in mortality is not statistically significant
2	Chan 2015[240]	11/08-12/09	Prospective cohort study	a) Mort. rate cases= 64%<br>Mort. rate controls=44.6%<br>b) Mort. rate cases= 30.7%<br>Mort. rate controls=18.8%<br>HR=1.96, 95%CI(1.01-3.78)	Hong Kong	hospitaliz	75 cases<br>117 controls	none	all patients were in a nursering home	a) all-cause mortality<br>b) infection related mortality*<br>* according to International Classification of Diseases, 9th Revision, Clinical Modification
3	Chen 2013[241]	07/09-06/10	Retrospective cohort study	Mort. rate cases= 12.2%<br>Mort. rate controls=15.6%	Texas, USA	hospitaliz	90 cases<br>90 controls	none	mean±SD<br>cases=5.2±7.4<br>controls=5.9±8.8	
4	Moore 2014[242]	01/07-12/12	Retrospective study	a) Mort. rate cases= 17%<br>Mort. rate controls=7%<br>b) Mort. rate cases= 3.6%<br>Mort. rate controls=0%	Ireland	5 years	28 cases<br>56 controls	age & gender recipient & donor, CMI, cause of renal failure pre-operatively, date of transpl.	no data	all patients were renal transplant recipients<br>a) all-cause mortality<br>b) infection related mortality

Table 51: Short extraction table of original articles on respiratory tract infections mortality of MRSA infected patients vs non-infected patients

Nr	Author	Study Period	Study type	Outcome	Location	Period of outcome	Population	Matching criteria	LOS (days)	Notes
1	Minejima 2014[243]	01/05-10-11	Retrospective study	Mort. rate cases= 22%<br>Mort. rate controls=3%	California, USA	28 days	134 cases<br>134 controls	pneumonia	mean<br>cases=10<br>controls=5	* patients had non-MRSA pneumonia

Table 52: Short extraction table of original articles on respiratory tract infections mortality of MRSA infected patients vs MSSA infected patients

Nr	Author	Study Period	Study type	Outcome	Location	Period of outcome	Population	Matching criteria	LOS (days)	Notes
1	Hill 2013[244]	01/04-12/09	Retrospective study	Mort. rate cases= 17.5%<br>Mort. rate controls=25.4%	Tennessee, USA	hospitaliz	no exact data*<br>~80 cases<br>~60 controls	VAP	in ICU<br>mean<br>cases=34<br>controls=35	patients had Ventilator-associated pneumonia (VAP)<br>*population estimated from graph
2	Tosh[245] 2013	09/08-08/10	Retrospective study	Mort. rate cases= 12%<br>Mort. rate controls=2%	USA	hospitaliz	42 cases<br>52 controls	LRI	mean<br>cases=9<br>controls=6	all patients had lower respiratory tract infection (LRI)
3	Jung 2013[246]	01/08-12/11	Retrospective study	Mort. rate cases= 33.3%<br>Mort. rate controls=21.5%	South Korea	hospitaliz	78 cases<br>865 controls*	pneumonia	median (IQR)<br>cases=16.5 (10–30)<br>controls=11 (6–20)	* controls were patients with non-nosocomial pneumonia due to other bacteria (non-MRSA)
4	Rello 2013[196]	not clear	Retrospective comparison of 2 cohorts	a) Mort. rate cases= 33.3%<br>Mort. rate controls=10%<br>b) Mort. rate cases= 60%<br>Mort. rate controls=50%	Europe & Latin America *	in ICU	a) 15 cases<br>30 controls<br>b) 5 cases<br>6 controls	none	no data	all patients had pneumonia and were in ICU<br>*the study aimed to compare 2 cohorts from different continents<br>a) Europe<br>b) Latin America
5	Self 2016[247]	2,5 years	Prospective study	Mort. rate cases= 13.3%<br>Mort. rate controls=9.1%	Australia	hospitaliz	15 cases<br>22 controls	CA-pneumonia	median (IQR)<br>cases=9 (8-	all patients had CA-pneumonia

									13)<br>controls=6<br>(4–10)	

Table 53: Short extraction table of original articles on joint infections mortality of MRSA infected patients vs MSSA infected patients

Nr	Author	Study Period	Study type	Outcome	Location	Period of outcome	Population	Matching criteria	LOS (days)	Notes
1	Lin 2015[248]	01/08-12/11	Clinical case-series	Mort. rate cases= 5.3%<br>Mort. rate controls=5.5%	Taiwan	hospitaliz	38 cases<br>55 controls	joint infection	no data	all patients had septic arthritis caused by S. aureus
2	Minguez 2015[249]	1984-2011	Retrospective study	Mort. rate cases= 57.1%<br>Mort. rate controls=17.6%	Mexico	hospitaliz	7 cases<br>17 controls	joint infection	mean cases=33 controls=20	all patients had septic arthritis due to S. aureus

During the review we have identified an article of 2011(Lambert et al.[50]) which was not included in WHO review and which reports mortality data for MRSA and MSSA patients with BSI and pneumonia. We decided to report the results of this article given its high quality.

Table 54: Short extraction table of the original article reporting BSI and pneumonia infections due to MRSA vs MSSA organisms.

Nr	Author	Study Period	Study type	Outcome	Location	Period of outcome	Population	Matching criteria	LOS (days)	Notes
1	Lambert 2011[50]	01/05-12/08	Prospective cohort study	a) Mort. cases=38%<br>Mort. controls=26%<br>aHR=1.6; 95%CI(1.1-2.3)<br>b) Mort. cases=33%<br>Mort. controls=23%<br>aHR=1.3; 95%CI(1.0-1.6)	Europe	ICU hospitalization	a)171 cases<br>284 controls<br>b)524 cases<br>1014 controls	ICU	From infection to discharge<br>median (IQR)<br>a)cases=14(6-24)<br>controls=11 (5-24)<br>b)cases= 15(8-27)<br>controls=14 (8–25)	patients were in ICU<br>a)patients with BSI<br>b)patients with pneumonia

Additionally to the primary studies included in the WHO AMR report and in our literature review, we would like to report the analytical study from de Kraker et al of 2011[250]. In this study the authors estimate the excess number of deaths, bed-days and hospital costs associated with BSI caused by MRSA and G3CREC in 31 European countries that participated in the EARSS for 2007. Official data from ECDC, WHO and OECD together with previously published mortality data [61,193] and model equations[251] are used to model the indicators for each country individually and for all of them pooled. The results resumed are: "In 2007, 27,711 episodes of MRSA BSIs were associated with 5,503 excess deaths and 255,683 excess hospital days in the participating countries, whereas 15,183 episodes of G3CREC BSIs were associated with 2,712 excess deaths and 120,065 extra hospital days. The total costs attributable to excess hospital stay were 44.0 and 18.1 million euros (63.1 and 29.7 million international dollars), respectively." For further details on the methodology and country specific results we invite the reader to access the full text article.

11. Carbapenem resistant Pseudomonas aeruginosa

To research the attributable case fatality and the attributable LOS to carbapenem resistant *Pseudomonas aeruginosa* (CRPA) we performed 2 advanced searches on Pubmed using once the mesh term "Pseudomonas aeruginosa" AND all fields "carbapenem resistant" AND "mortality", and the second time the mesh term "Pseudomonas aeruginosa" AND all fields "carbapenem resistance" AND "mortality". Embase was searched for 'Pseudomonas aeruginosa' AND 'carbapenem resistant' AND 'mortality', and 'Pseudomonas aeruginosa AND 'carbapenem resistance' AND 'mortality'. We applied a filter to include only human studies. An advanced search on the Cochrane Library was performed using terms "Pseudomonas aeruginosa" AND "carbapenem". No language restrictions were made, and no further filters were applied.

The flow diagram, presented in Figure 11 describes the steps in the literature review of carbapenem resistant *Pseudomonas aeruginosa* infections. The results obtained using the two different strings for PubMed and Embase were aggregated.

Once identified the studies reporting relevant data, these were grouped based on the site of infection.

Figure 11: Search for studies on carbapenem resistant *Pseudomonas aeruginosa*. Inclusion and exclusion diagram.

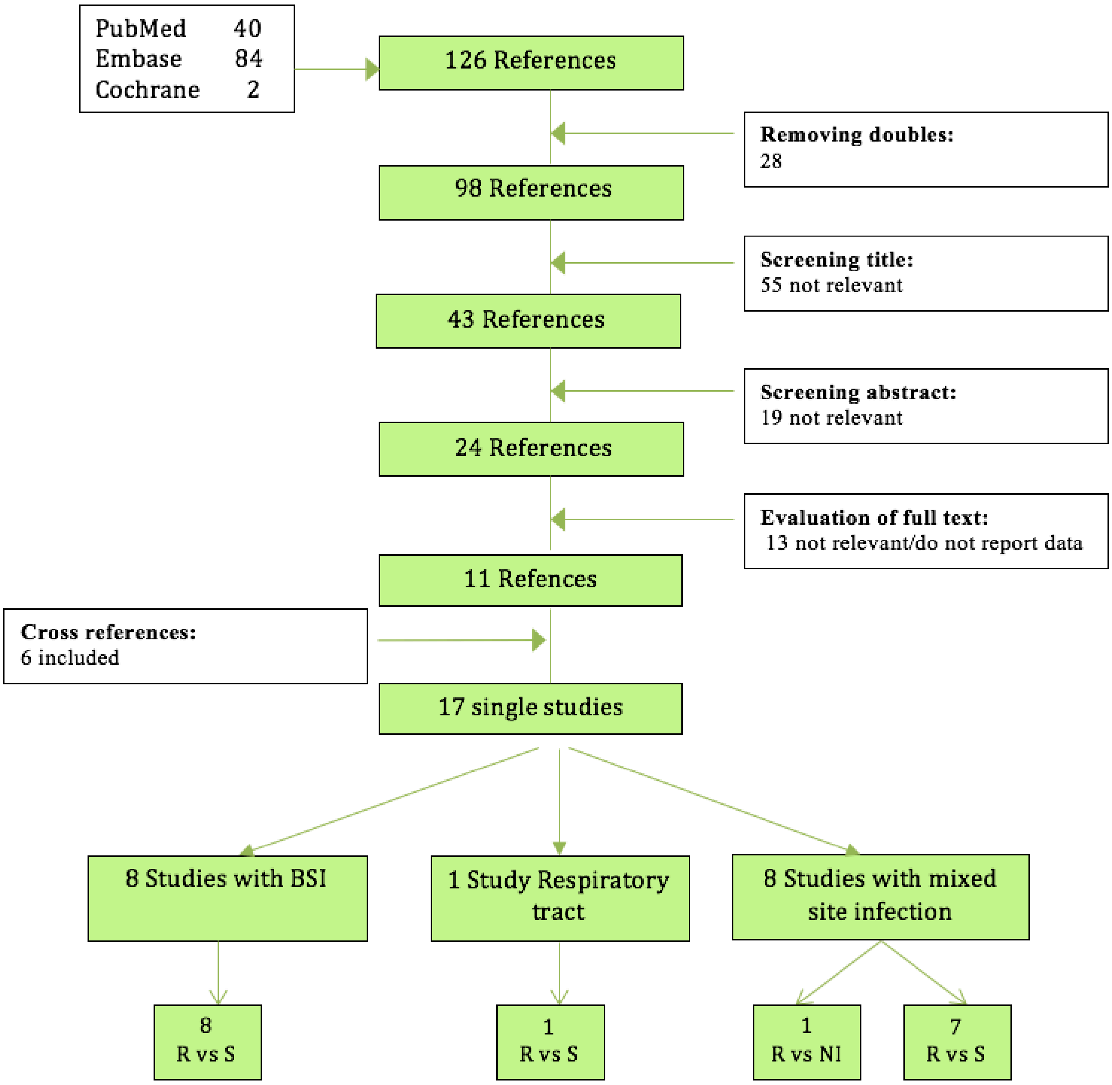

As shown in Figure 11, 6 studies were not detected by our searching strategy (because they used imipenem in their key words instead of the generic term carbapenem) and included in the review because were found in a meta-analysis from Liu et al, 2015[252] which was part of our results.

The following tables include studies reporting results for BSI, respiratory tract and for mixed site of infection due to CRPA, based on the comparison group they used. All information is reported as was presented in the original papers.

Studies that include both a sensitive and a non-infected control group, were categorised in the latter group.

Table 55: Short extraction table of original articles on BSI mortality of CRPA infected patients vs CSPA infected patients

Nr	Author	Study Period	Study type	Outcome	Location	Period of outcome	Population	Matching criteria	LOS (days)	Notes
1	Dantas 2014[253]	05/09-08/11	Retrospective study	Mort. cases= 47.3%<br>Mort. controls= 36.9%<br>OR=1.53	Brazil	30 days	55 cases<br>65 controls	BSI	mean (range)<br>cases=48(4-205)<br>controls=41(3-378)	
2	Hatteme r 2013[254]	08/99-01/10	Retrospective study	Mort. cases= 54%<br>Mort. controls= 32%	New York, USA	30 days	13 cases<br>137 controls	BSI	no data	patients had HAI or CAI
3	Joo 2011[255]	10/07-03/09	Retrospective cohort study	Mort. cases= 39.1%<br>Mort. controls= 21.2%OR=2.40; 95%CI(1.18–4.86)<br>aOR=2.74: 95%(1.02–7.37)*	Korea	30 days	46 cases<br>156 controls	BSI	median (IQR)<br>cases=16(14-42)<br>controls=16(9-29)	*adjusted for corticosteroid use, nosocomial acquisition, polymicrobial infection, Charlson's weighted index of co-morbidity, and admission to ICUs
4	Kang 2005[256]	01/98-12/02	Retrospective study	Mort. cases= 53.6%<br>Mort. controls= 35.2%<br>OR=2.216; 95%CI(0.946–4.776)	Korea	30 days	28 cases<br>158 controls	none	no data	
5	Kim 2014[257]	01/10-12/12	Retrospective study	Mort. cases= 22.0%<br>Mort. controls= 22.4%<br>OR=3.65; 95%CI(0.72-18.67)	Korea	14 days	118 cases<br>116 controls	BSI	no data	the study was focused on survivors and non-surivors
6	Pena 2012[258]	01/08-12/09	Prospective multicentre study	a) Mort. cases= 13%<br>Mort. controls= 13%<br>b)Mort. cases= 35%<br>Mort. controls= 27%	Spain	a) 48h<br>b) 30 days	145 cases<br>487 controls	BSI	no data	
7	Suarez 2010[259]	01/05-12/05	Retrospective cohort study	Mort. cases= 46%<br>Mort. controls= 36%<br>*Attribut. mort. cases=33%<br>Attribut. mort. controls=30%	Spain	30 days	33 cases<br>88 controls	BSI	mean (range)<br>cases=19(2-98)<br>controls=9(2-56)	*within 7 days from BSI and excluding other causes

				OR=1.8; 95%CI(0.5–6.8)						
8	Tuon 2012[260]	01/06-01/09	Case-control study	Mort. cases= 54.2%<br>Mort. controls= 44.8%<br>OR=0.68; 95%CI(0.27–1.73)	Brazil	30 days	29 cases<br>48 controls	BSI	mean±SD<br>cases=43±31.7<br>controls=43.1±31.2	

Table 56: Short extraction table of original articles on respiratory tract infection mortality of CRPA infected patients vs CSPA infected patients

Nr	Author	Study Period	Study type	Outcome	Location	Period of outcome	Population	Matching criteria	LOS (days)	Notes
1	Luyt 2014[261]	18 months	Prospective observational study	Mort. cases= 37%<br>Mort. controls= 31%	France	In ICU stay	68* cases<br>101 controls	none	median (IQR)<br>cases=37(23-57)<br>controls=29(19-46)	* cases included resistant and intermediate susceptible PA, was not possible to separate them

Table 57 and Table 58 present the results of studies that include patients with different sites of infection. Almost all the articles specify the number of cases for the different infection sites but they do not report different mortalities based on the infection site. When it was possible the results are presented differentiating the mortality for each site of infection. The study in Table 57 includes a not infected and a susceptible infected control group.

Table 57: Short extraction table of original articles on mortality of CRKP infected vs non infected vs CSPA infected patients, for different infection sites

Nr	Author	Study Period	Study type	Outcome	Location	Period of outcome	Population	Matching criteria	LOS (days)	Notes
1	Eagye 2009[262]	02/06-10/06	Case-control-control study	Mort. resit= 31%<br>Mort. sensit= 15%<br>Mort. control=9%	Connecticut, USA	hospitaliz	58 resistant<br>125 sensitive<br>57 non infected	none	median(IQR)<br>resist=30(15-70)<br>sensit=16(6-33)<br>control=10(6-17)	

Table 58: Short extraction table of original articles on mortality of CRPA infected patients vs CSPA infected patients for different infection sites

Nr	Author	Study Period	Study type	Outcome	Location	Period of outcome	Population	Matching criteria	LOS (days)	Notes
1	Judd 2016[263]	01/11-12/13	Retrospective observational cohort study	Mort. cases= 28.1%<br>Mort. controls= 8.9%<br>OR=2.89; 95%CI(1.15-7.28)	Kentucky, USA	hospitaliz	32 cases<br>350 controls	none	median (IQR)<br>cases=14(8-29)<br>controls=9(5-15)	
2	Lautenbach 2006[264]	01/91-12/00	Case-control study	Mort. cases= 31.1%<br>Mort. controls= 16.7%<br>RR=1.86; 95%CI(1.38-2.51)	Pennsylvania USA	hospitaliz	142 cases<br>737 controls	none	median (IQR)<br>cases=11(1-41)<br>controls=4(1-15)	study considers all the cases recovered with PA
3	Lautenbach 2010[265]	01/01-12/06	Case-control study	Mort. cases= 17.4%<br>Mort. controls= 13.4%<br>RR=1.39; 95%CI(1.09–1.76)	Pennsylvania USA	hospitaliz	253 cases<br>2289 controls	none	prior isolation median (IQR)<br>cases=8(4-12)<br>controls=4(4-5)	study considers all the cases recovered with PA
4	Lin 2014[266]	01/00-12/10	Retrospective study	Mort. cases= 22.0%<br>Mort. controls= 19.5%	Taiwan	hospitaliz	82 cases<br>82 controls	none	no data	matched randomly by computer
5	Liu 2015[252]	1994-2014	Meta-analyses	Crude OR=1.64 (95%CI=1.40, 1.93)<br>Adjusted OR=2.38 (95%CI=1.53, 3.69)	World		16 studies			
6	Meradji 2015[267]	01/12-12/13	Prospective observational study	Mort. cases= 13.34%<br>Mort. controls= 1.53%	Morocco	hospitaliz	15 cases<br>65 controls	none	no data	
7	Brooklyn Antibiotic Resistance Task Force 2002[268]	1999	Matched case-control study	Mort. cases= 20%<br>Mort. controls= 10%	USA	hospitaliz	10 cases<br>10 controls	age, site of infection, major diagnosis, operative procedures, type of infection	median preinfection<br>cases=12<br>controls=15<br>postinfection<br>cases=33.5<br>controls=20	

Many studies were excluded because they did not report a comparison group. Other studies identified were genetic analyses and were not eligible for the purposes of this review.
We also identified a meta-analysis that calculated the mortality due to carbapenem resistant *Pseudomonas aeruginosa* compared to a susceptible group. The published studies included in this meta-analysis were also identified in our literature search, except for six publications which we subsequently included since they fulfilled the eligibility criteria.

We decided to report in this chapter the study of Lambert et al[50] since it included information on 3rd generation cephalosporin resistant Pseudomonas aeruginosa, which might be useful in the evaluation of the resistances of this bacterium. This article studied the mortality rates of patients with BSI or pneumonia due to 3rd generation cephalosporin resistant *P. aeruginosa* (3GCRPA) and 3rd generation cephalosporin susceptible *P. aeruginosa* (3GCSPA)

Table 59: Short extraction table of the original article reporting BSI and pneumonia infections due to 3GCRPA vs 3GCSPA organisms

Nr	Author	Study Period	Study type	Outcome	Location	Period of outcome	Population	Matching criteria	LOS (days)	Notes
1	Lambert 2011[50]	01/05-12/08	Prospective cohort study	a) Mort. cases=41%<br>Mort. controls=39%<br>aHR=1.2; 95%CI(0.8-1.9)<br>b) Mort. cases=43%<br>Mort. controls=37%<br>aHR=1.2; 95%CI(1.0-1.5)	Europe	ICU hospitalization	a)82 cases<br>282 controls<br>b)366 cases<br>1266 controls	ICU	From infection to discharge<br>median (IQR)<br>a)cases=16(7-33)<br>controls=14 (6-26)<br>b)cases= 18(11-33)<br>controls=16 (9–29)	patients were in ICU<br>a)patients with BSI<br>b)patients with pneumonia

12. Colistin resistant Pseudomonas aeruginosa

To research the attributable case fatality and the attributable LOS to colistin resistant *Psudomonas aeruginosa* (CoRPA) we performed 2 advanced searches on Pubmed using once the mesh term “Pseudomonas aeruginosa” AND all fields “colistin resistant”, and the second time the mesh term “Pseudomonas aeruginosa” AND all fields “colistin resistance”. Embase was searched for ‘Pseudomonas aeruginosa’ AND ‘colistin resistance’, and for ‘Pseudomonas aeruginosa’ AND ‘colistin resistant’. In Embase studies was filtered, including just those conducted in humans. An advanced search on the Cochrane Library was performed using terms “Pseudomonas aeruginosa” AND “colistin”. No language restrictions were made, and no other filters were applied.

The flow diagram, presented in Figure 12 describes the steps in the literature review of colistin resistant *Pseudomonas aeruginosa* infections. The results obtained using the two different key words in PubMed and Embase were aggregated.

Figure 12: Search for studies on colistin resistant *Pseudomonas aeruginosa*. Inclusion and exclusion diagram.

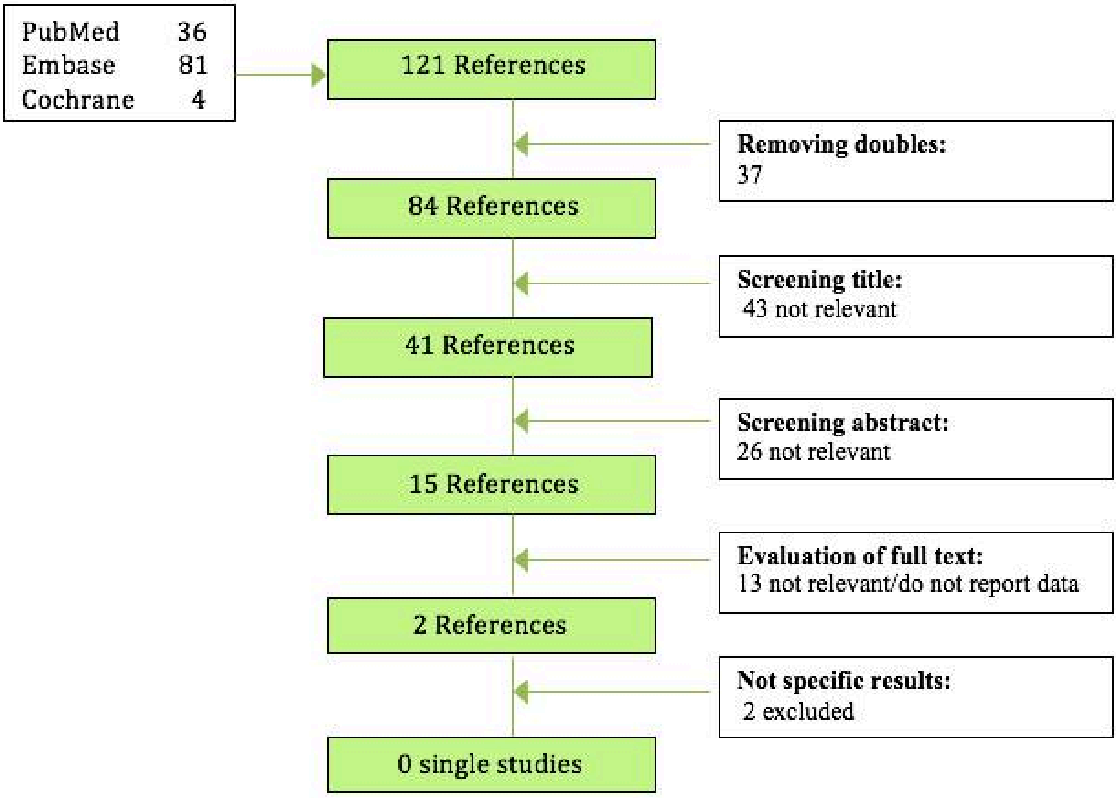

The review did not identify any published study eligible with the selection criteria. However, two articles reported studies on colistin resistance in patients infected with *Pseudomonas aeruginosa*.

Matthaiou et al. in 2008[269] studied the risk factors associated with the isolation of colistin-resistant Gram-negative bacteria in a matched case-control study. Amongst 40 patients, two reported a CoRPA infection but the sample could not be considered representative. Yilmaz et al 2016[270], also studied the risk factors of colistin-resistant Gram-negative bacteria in a multicentre study and included in their sample of 56 resistant patients, 18 infected with CoRPA. The sample is more representative but the results of the study are presented pooled together for all patients infected with Gram-negative bacteria. They reported a mortality rate of cases (resistant to colistin) of 53.6%, compared to the mortality rate of controls (sensitive to colistin) of 45%.

The colistin resistant Pseudomonas aeruginosa infections are a relatively new research topic, and it seems that no structured studies were conducted yet to investigate the burden of these infections in terms of attributable mortality or attributable length of stay in hospital.

13. Multidrug resistant Pseudomonas aeruginosa

For the purposes of these project a multidrug resistant isolate is defined as resistant to at least three classes of antimicrobial agents, all penicillins and cephalosporins (including inhibitor combinations), fluroquinolones, and aminoglycosides. Here are excluded those resistant to carbapenem and/or to colistin. If an isolate is multidrug resistant and also resistant to carbapenem and/or colistin, it will be classified as resistant to the latter antibiotic.

To research the attributable case fatality and the attributable LOS to multidrug resistant *Pseudomonas aeruginosa* (MDRPA) we performed 3 advanced searches on Pubmed using once the mesh term “Pseudomonas aeruginosa” AND all fields “multidrug resistant” AND all fields “mortality”, the second time the mesh term “Pseudomonas aeruginosa” AND all fields “multidrug resistance” AND all fields “mortality”, and the third time the mesh term “Pseudomonas aeruginosa” AND all fields “MDR” AND all fields “mortality”. A filter for human studies was applied. Embase was also searched for 3 different strings; once ‘Pseudomonas aeruginosa’ AND ‘multidrug resistant’ AND ‘mortality’, the second time ‘Pseudomonas aeruginosa AND ‘multidrug resistance’ AND ‘mortality’, and the third time ‘Pseudomonas aeruginosa’ AND ‘MDR AND ‘mortality’. We applied a filter to include only human studies. Two advanced searches were also performed on the Cochrane Library using once the terms “Pseudomonas aeruginosa” AND “multidrug resistant”, and the second time the terms “Pseudomonas aeruginosa” AND “multidrug resistance”. No language restrictions were made, and no further filters were applied.

The flow diagram, presented in Figure 13 describes the steps in the literature review of multidrug resistant *Pseudomonas aeruginosa* infections. The results obtained using the different strings for PubMed, Embase and the Cochrane Library were summed up together.

Figure 13: Search for studies on multidrug resistant *Pseudomonas aeruginosa*. Inclusion and exclusion diagram.

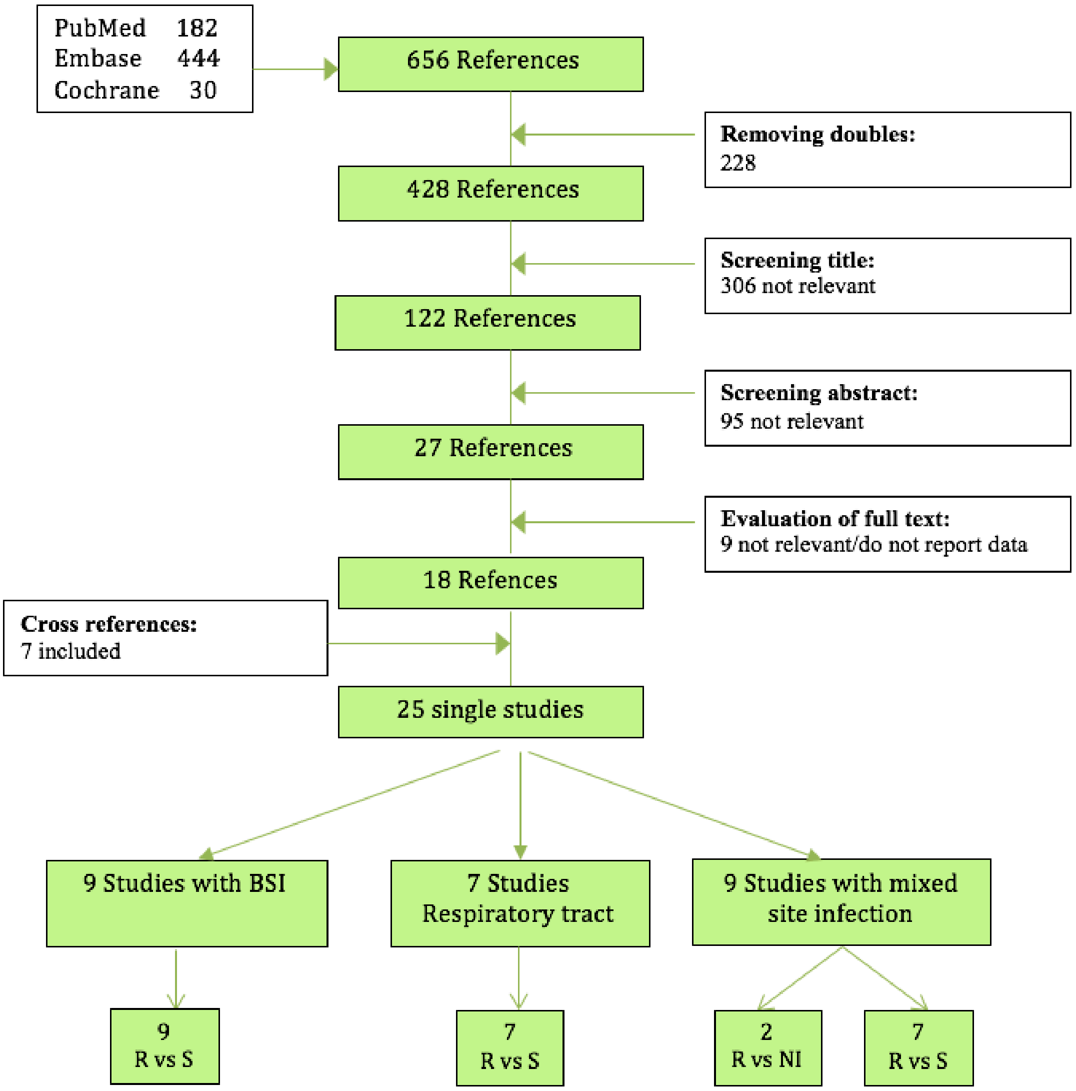

The following tables include studies reporting results for BSI, respiratory tract and for mixed site of infection due to MDRPA, based on the comparison group they used. All information is reported as was presented in the original papers.

Table 60: Short extraction table of original articles on BSI mortality of MDRPA infected patients vs MDSPA infected patients

Nr	Author	Study Period	Study type	Outcome	Location	Period of outcome	Population	Matching criteria	LOS (days)	Notes
1	Caselli 2010[271]	01/00-12/08	Retrospective study	Mort. rate cases=35%.8<br>Mort. rate controls=12.5%<br>aOR=4.3; 95%CI(1.67-11.07)	Italy	30 days	39 cases<br>88 controls	underlying condition	no data	patients were children under chemotherapy and HSC transplant
2	Johnson 2009[272]	01/96-12/05	Retrospective cohort study	Mort. rate cases=36.6%<br>Mort. rate controls=46%<br>OR=0.68; 95%CI(0.27-1.71)	Boston, USA	28 days	41 cases<br>50 controls	transplant	no specific data	patients were trasplant recipients
3	Morata 2012[273]	01/00-12/08	Prospective study	Mort. rate cases=17.2%<br>Mort. rate controls=32.3%	Spain	30 days	127 cases<br>582 controls	BSI	mean±SD<br>cases=31.83±30<br>controls=16.38±18	
4	Tam 2010[274]	01/05-12/08	Retrospective cohort study	I) Mort. rate cases=40%<br>Mort. rate controls=11.9%<br>OR=6.829; 95%CI(1.945–23.984)<br>II) Mort. rate cases=52%<br>Mort. rate controls=9.5%<br>III) Mort. rate cases=56%<br>Mort. rate controls=16.7%<br>IV) c) Mort. rate cases=61.9%<br>Mort. rate controls=9.5%	Houston, USA	I) 30 days<br>II) infection related<br>III) overall mortality<br>IV) matched pairs	25 cases<br>84 controls	BSI	prior isolation<br>mean±SD<br>cases=32.6±37.3<br>controls=14.4±43.6	
5	Theodorou 2013[275]	01/89-12(09	Retrospective cohort study	Mort. rate cases=42.2%<br>Mort. rate controls=45.2%<br>OR=1.076; 95%CI(0.356–3.254)	Germany	hospitaliz	45 cases<br>42 controls	underlying condition	prior isolation<br>mean±SD<br>cases=28.2±23.8<br>controls=26.6±22.3	patients were in a burn ICU
6	Trecarichi 2011[276]	01/09-?	Prospective study	Mort. rate cases=40.7%<br>Mort. rate controls=9.1%	Italy	21 days	27 cases<br>11 controls	underlying condition		patients had haematological malignancies

7	Tumbarello 2011[277]	01/06-12/07	Case-case-control study	Mort. rate cases=50%<br>Mort. rate controls=24.2%	Italy	21 days	40 cases<br>66 controls	BSI	mean±SD<br>cases=27±14<br>controls=17±13	
8	Dantas 2014[253]	05/09-08/11	Retrospective study	Mort. rate cases=42.1%<br>Mort. rate controls=41.2%	Brazil	30 days	57 cases<br>63 controls	BSI	mean (range)<br>cases=59 (4–205)<br>controls=62 (3–378)	
9	Joo 2011[255]	10/07-03/09	Retrospective cohort study	Mort. rate cases=38.1%<br>Mort. rate controls=21.9%<br>OR=2.20; 95%CI(1.06–4.55)<br>aOR=2.24; 95%CI (0.80–6.29)*	Korea	30 days	42 cases<br>160 controls	BSI	median (IQR)<br>cases=25 (13–38)<br>controls=16 (10–31)	*adjusted for corticosteroid use, nosocomial acquisition, polymicrobial infection, Charlson's weighted index of co-morbidity, and admission to ICUs

Table 61: Short extraction table of original articles on respiratory tract infection mortality of MDRPA infected patients vs MDSPA infected patients

Nr	Author	Study Period	Study type	Outcome	Location	Period of outcome	Population	Matching criteria	LOS (days)	Notes
1	Cilloniz 2016[278]	01/99-12/14	Prospective observational study	Mort. Rate cases=22.7%<br>Mort. rate controls=17.4%	Spain	30 days	22 cases<br>46 controls	pneumonia	median (IQR)<br>cases=14(12-21)<br>controls=11(7-16)	patients had all CAI
2	Montero 2009[279]	01/00-12/05	Case-control study	Mort. rate cases=60%<br>Mort. rate controls=28%<br>OR=6.2; 95%CI(1.7–22.1)	Spain	2 years	50 cases<br>50 controls	age, sex, date of adm, degree of air obstruction	no data	Patients had COPD<br>the isolation sample was sputum
3	Micek 2015[280]	no data	Multicentre retrospective study	Mort. rate cases=44.7%<br>Mort. rate controls=31.7%	World*	hospitaliz	226 cases<br>514 controls	pneumonia	median (IQR)<br>cases=27(14-56.3)<br>controls=25(13-46)	*USA, Europe (4 countries)

				HR=1.39; 95%CI(1.05-1.83)						
4	Pena 2013[281]	01/06-12/11	Retrospective study	a) Mort. rate cases=15%<br>Mort. rate controls=29%<br>b) Mort. rate cases=50%<br>Mort. rate controls=55%	Spain	a) 7 days<br>b) hospitaliz	60 cases<br>31 controls	pneumonia	no data	
5	Tumbarello 2013[282]	01/08-12/10	Retrospective study	Mort. rate cases=59.5%<br>Mort. rate controls=35.2%<br>OR=2.69; 95%CI(1.14–6.43)	Italy	in ICU	42 cases<br>68 controls	ICU	no data	patients were in ICU
6	Yang 2009[283]	10/02-04/06	Prospective observational study	Mort. rate cases=35%<br>Mort. rate controls=29%	San Francisco, USA	28 days	20 cases<br>55 controls	VAP	median (IQR)<br>cases=37(28.5-72)<br>controls=34(21-47)	
7	Yayan 2015[284]	01/04-08/14	Retrospective study	a) Mort. rate cases=28.6%<br>Mort. rate controls=23.8%<br>b) Mort. rate cases=7.4%<br>Mort. rate controls=18.5%	Germany	hospitaliz	a) HAI<br>14 cases<br>63 controls<br>b) CAI<br>27 cases<br>64 controls	VAP	no specific data	data were extracted since presented from different prospective

Table 62 and Table 63 present the results of studies that include patients with different sites of infection. Almost all the articles specify the number of cases for the different infection sites but do not report different mortalities based on the infection site. When it was possible the results are presented differentiating the mortality for each site of infection.

Table 62: Short extraction table of original articles on mortality of MDRPA infected patients vs non infected patients for different infection sites

Nr	Author	Study Period	Study type	Outcome	Location	Period of outcome	Population	Matching criteria	LOS (days)	Notes
1	Aloush 2016[285]	no data	Matched cohort study	Mort. rate cases=21%<br>Mort. rate controls=12%<br>OR=4.4	Israel	hospitaliz	82 cases<br>82 controls	ward, LOS, calendar time	matching criteria	
2	Paramythiotou 2004[286]	01/99-12/00	Matched case-control study	a) Mort. rate cases=44%<br>Mort. rate controls=47%	Greece	a) in ICU<br>b) hospitaliz	34 cases<br>34 controls	severity of illness, LOS in ICU	in ICU<br>mean±SD	patients were in ICU

				b) Mort. rate cases=47%<br>Mort. rate controls=50%					cases=11.1±18.6<br>controls=5.8±10.4	

Table 63: Short extraction table of original articles on mortality of MDRPA infected patients vs MDSPA infected patients for different infection sites

Nr	Author	Study Period	Study type	Outcome	Location	Period of outcome	Population	Matching criteria	LOS (days)	Notes
1	Cao 2004[287]	01/99-12/02	Retrospective cohort study	Mort. rate cases=54.5%<br>Mort. rate controls=16.2%	China	hospitaliz	44 cases<br>68 controls	none	in ICU<br>mean(SD)<br>cases=21(47.7)<br>11(16.2)	
2	da Matos 2016[288]	01/10-02/12	Retrospective cohort study	Mort. rate cases=70%<br>Mort. rate controls=58.9%<br>OR=1.6333;<br>95%CI(0.5043–5.2901)<br>Adults:<br>cases=81.3%<br>controls=71.4%<br>paediatric:<br>cases=25.0%<br>controls=55.0%	Brazil	hospitaliz	20 cases<br>34 controls	ICU	no specific data	adults, children and neonates patients in ICU
3	Morales 2012[289]	01/05-12/06	Retrospective study	Mort. rate cases=24.6%<br>Mort. rate controls=12.8%<br>OR=1.77; 95%CI(1.41-2.22)	Spain	hospitaliz	134 cases<br>149 controls	none	mean(SD), median<br>cases=25.1(16.1), 20<br>controls=39.0(30.3), 30	
4	Nathwani 2014[290]	01/00-02/13	Review and meta-analysis	Mort. rate cases=25-60%<br>Mort. rate controls=7-50%<br>RR=2.34, 95%CI(1.53 – 3.57)	World	hospitaliz	559 cases<br>7881 controls			
5	Ortega 2004[291]	01/01-12/01	Prospective study	Mort. rate cases=22%<br>Mort. rate controls=23%	Netherlands	in ICU	18 cases*<br>35 controls**	underlying condition	In ICU<br>median (range)<br>cases=18(0-40)<br>controls=10(0-44)	all patients were critically ill<br>*resistant of at least 2 antibiotics<br>**susceptible or

										resistant to only 1 antibiotic
6	Peng 2014[292]	07/08-12/12	Case-control study	Mort. rate cases=26.1%<br>Mort. rate controls=12.5%	China	hospitaliz	188 cases<br>168 controls	none	median (range)<br>cases=35(5-697)<br>controls=24(1-262)	it was a surveillance study
7	Su 2016[293]	01/03-06/15	Retrospective double centre study	a) Mort. rate cases=36%<br>Mort. rate controls=31.0%<br>b) Mort. rate cases=47.6%<br>Mort. rate controls=37.9%	China	30 days	25 cases<br>29 controls	none	no specific data	a) related mortality<br>b) overall mortality

This literature research was specific in the searching strings and we did not detect many unspecific articles. The introduction of the term mortality excluded many ineligible studies. The vast majority of papers contained related information to mortality but very often they did not report a comparison group.
Many studies reported treatment options for the resistant infections, but since they used just cases infected with MRDPA and no control group, as defined by our eligibility criteria, these were excluded.
All studies were performed in healthcare settings, although some report cases of infection that originated in the community; only one study treated only patients with infection onset in the community.
The studies that included patients with MDRPA which were all also resistant to carbapenems were also not included in this review since the mortality reported in these cases is not only attributable to the multidrug resistance but also to the carbapenem resistance.
The only exception is the meta-analysis reported in Table 63 from Nathwani et al 2014[290] which included patients resistant to carbapenems.

14. Penicillin resistant *Streptococcus pneumoniae*

To research the attributable case fatality and the attributable LOS to penicillin resistant *Streptococcus pneumoniae* (PRSP) we searched the following databases: PubMed, EMBASE and Cochrane Database of Systematic Reviews. We performed an advanced search on Pubmed using MeSH term “Streptococcus pneumoniae” and all fields “penicillin resistant”. Embase was searched for “penicillin resistant’’ AND streptococcus pneumoniae”. An advanced search on the Cochrane Library was performed using terms “streptococcus pneumoniae” and “penicillin”. No language restrictions were made, and filter for research in humans was applied.

Relevant data were extracted and retrospective extraction tables are shown in the results section.

The flow diagram shown in Figure 14 describes the steps in the literature review of penicillin resistant *Streptococcus pneumoniae*.

Once identified, the studies reporting relevant data were grouped based on the site of infection. All studies compared penicillin-resistant *Streptococcus pneumoniae* (PRSP) with penicillin-susceptible *Streptococcus pneumoniae* (PSSP). No studies were found with non-infected patients as comparator.

Figure 14: Search for studies on Penicillin resistant *Streptococcus pneumoniae*. Inclusion and exclusion diagram.

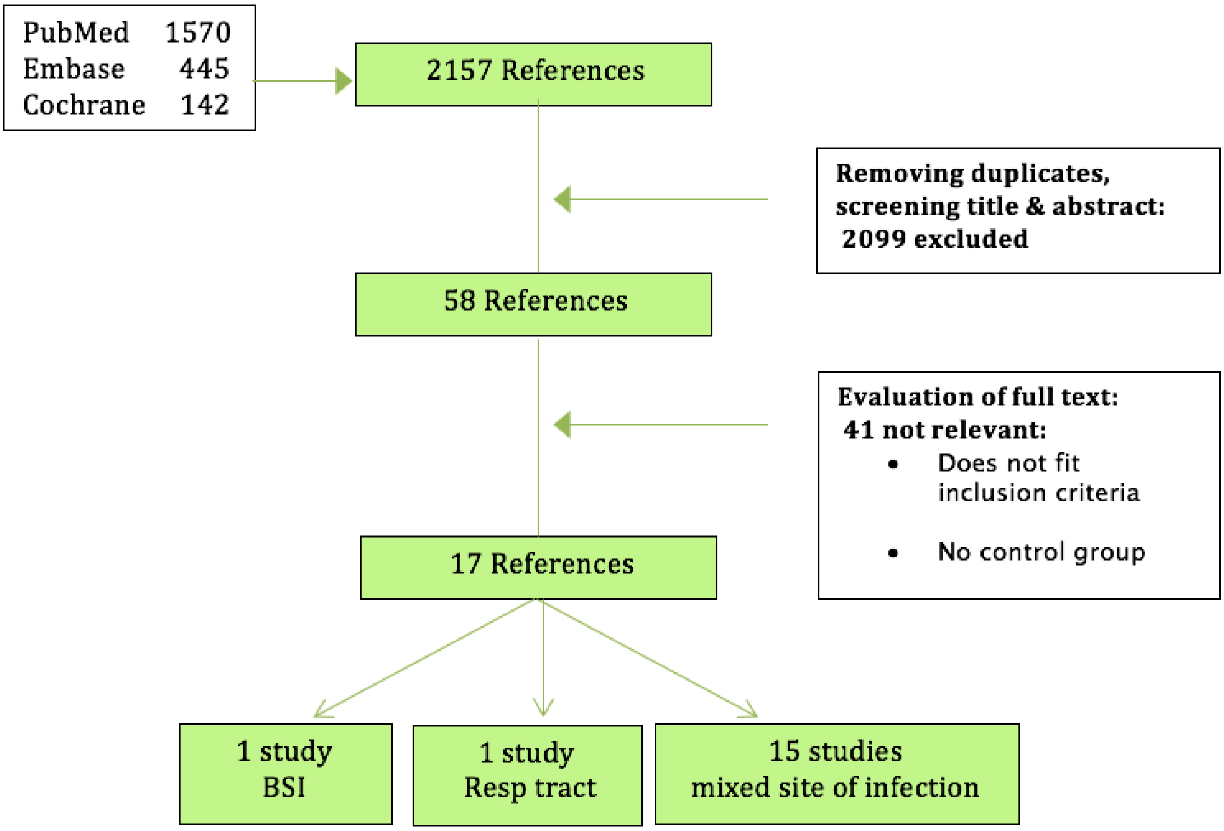

One systematic review and meta-analysis was found[294].

The following tables include studies reporting results for BSI, respiratory tract and mixed site of infection, based on the comparison group they used. All information is reported as was presented in the original papers.

Table 64. Short extraction table of original articles on meningitis mortality of PRSP infected patients vs PSSP patients.

Nr	Author	Study Period	Study type	Outcome	Location	Period of outcome	Population	Matching criteria	LOS (days)
1	Yu 2003[295]	12/98-01/01	Prospective, international, observational study	Mort. rate case=23.7% Mort. rate controls=15.1%	Global	>14 days after onset of BSI	76 cases 598 controls	N/A	N/A

Table 65. Short extraction table of original articles on BSI mortality of PRSP infected patients vs PSSP patients.

Nr	Author	Study Period	Study type	Outcome	Location	Period of outcome	Population	Matching criteria	LOS (days)	Notes
1	Watanabe 2000[296]	12/92-05/97	Retrospective comparative study	a)Mort. rate case=0% Mort. rate controls=8% b) Mort. rate case=12.5% Mort. rate controls=20%	Japan	7 days after start of treatment	24 cases 25 controls	N/A	N/A	a) infection related mortality b) mortality due to complications

Table 66. Short extraction table of original articles on pneumonia mortality of PRSP infected patients vs PSSP patients.

Nr	Author	Study Period	Study type	Outcome	Location	Period of outcome	Population	Matching criteria	LOS (days)	Cost (US dollars)
1	Gouveia 2011[297]	1996 - 2006	Observational study	Mortality: adjusted hazard ratio*: 1.62 (1.08-2.43)	Salvador, Brazil	hospitaliz	93 cases 548 Total population	N/A	N/A	
2	Reechaipichitkul 2006[298]	01/95-12/04	Cross-sectional study	a)Mort. rate case=9.1% Mort. rate controls=11.9% b)Mort. rate case=9.1% Mort. rate controls=9.5%	Khon Kaen, Thailand	Hospitalization a) all-cause mortality b) pneumonia related mortality	22 cases 42 controls	N/A	Mean(SD) R: 12 (9) S: 15.5 (17.7) p=0.43	
3	Sun 2006[299]	1999 – 2003	Retrospective cohort study	Mort. rate case=9% Mort. rate controls=14%	Connecticut, USA	Hospitalization	44 cases 168 controls	N/A	N/A	Median R: $10,809 (6,118-22,209)

							(cases are non-susceptible)			S: ($8,503) (5,452-15,862)
4	Song 2004[300]	01/00-06/01	Retrospective study	Mort. rate case=16% Mort. rate controls=12.4% OR=1.3; 95%CI(0.5–3.4)	Asia	30 days after diagnosis	69 cases 105 controls	N/A	N/A	N/A
5	Einarsson 1998[301]	1988 – 1994	Retrospective case-control study	Mort. rate case=11% Mort. rate controls=5.5%	Iceland	not defined but most probably mortality in hospital	36 cases 36 controls	Age, gender, hospitalised at the same time	Mean Case: 26.8 Control 11.5 (p=0.001)	Average pharmacy costs $736 vs. $213 (p<0.01)
6	Sangthawan 2003[302]	1998-2001	Prospective study	Mort. rate case=36.8% Mort. rate controls=18.5%	Thailand	not defined but most probably mortality in hospital	19 cases 27 controls	N/A	N/A	N/A
7	Falco 2004[303]	1997-2001	Retrospective and prospective	a)Mort. rate case=26.7% Mort. rate controls=12.7% b)Mort. rate case=13.3% Mort. rate controls=9.9%	Barcelona, Spain	Hospitalization a) all-cause mortality b) pneumonia related mortality	15 cases 181 controls	N/A	N/A	N/A
8	Pallares 1995 [304]	1984 – 1993	Prospective study	Mort. rate case=27.6% Mort. rate controls=21.8% aOR=1.0; 95%CI(0.5-1.9)	Barcelona, Spain	Within one month of discharge	47 cases 440 controls	N/A	N/A	N/A
9	Ewig 1999 [305]	1996 – 1998	Prospective study	Mort. rate case=18% Mort. rate controls=5.8%	Barcelona, Spain	Hospitalization	33 cases 52 controls	N/A	N/A	
10	Aspa 2014[306]	01/99-04/00	Prospective multi-centre study	Mort. rate case=18.3% Mort. rate controls=12.2%	Spain	30 days	64 cases 409 controls	N/A	No data	N/A
11	Feikin 2000[307]	1995 – 1997	Population-based	a) OR= 1.3; 95%CI(0.5-3.7)	USA	30 days a) resistant	a) 37 cases b) 20 cases 383 controls	N/A	No data	N/A

			surveillance study	b) OR=2.3 ; 95%CI(0.7-7.4)		b) highly resistant, MIC>4				
12	Tleyjeh 2006[294]	Up to 01/05	Review and meta-analyses	a) Mort. rate non susceptible=19.4%<br>Mort. rate controls=15.7%<br>b)OR non-suscptible=1.3; 95%CI(1.08–1.59)<br>OR intermediate=1.34; 95% CI(1.13–1.60)<br>OR resistant=1.29; 95% CI(1.01–1.66)<br>c) aRR non susceptible=1.29; 95%CI(1.04–1.59)	World	different based on the included studies<br>a) pooled mortality rate<br>b) all-cause mortality<br>c) combined adjusted (6 studies)	10 studies (some included also non susceptible)	N/A	No data	N/A
13	Yigla 1995[308]	11/89-04/90	Prospective study	Mort. rate case=43%<br>Mort. rate controls=33%	Israel	Hospitalization	7 cases<br>15 controls	N/A	No data	N/A
14	Pallares 2002[309]	01/94-10/00	Prospective study	Mort. rate case=43%<br>Mort. rate controls=20.3%	Barcelona, Spain	30 days	77 cases<br>300 controls	N/A	No data	N/A
15	Jehl 2002[310]	09/98-04/00	Prospective survey	a)Mort. rate case=8.3%<br>Mort. rate controls=3.5%<br>b)Mort. rate case=4.2%<br>Mort. rate controls=10.4%<br>c)Mort. rate case=12.5%<br>Mort. rate controls=13.9%	France	a) 3 days<br>b) 30 days<br>c) total	49 cases<br>246 controls	N/A	No data	N/A

*After adjusting for age group, PR pneumococcal meningitis cases had higher case fatality than cases caused by penicillin-susceptible organisms

There was a limited number of case-control studies reporting outcomes of interest. Many studies reviewed penicillin non-susceptible *Streptococcus pneumoniae* (including intermediately resistant and resistant) rather than solely penicillin-resistant *Streptococcus pneumoniae*. The majority of infections in the non-susceptible group fall into the intermediate categories. Most evidence indicates that standard treatment with a beta-lactam antimicrobial is effective against pneumococcal pneumonia caused by strains with penicillin MIC <2, thus including these studies may skew the results and they were excluded.

In January 2008 the Clinical and Laboratory Standards Institute revised the penicillin susceptibility breakpoints for non-meningeal pneumococcal infections from <0.06 micrograms/ml to <2 micrograms/ml[311]. Most studies found, however, predate this change but were not excluded and may represent a limitation when applied to incidence data from EARS-Net.

15. Penicillin and macrolide resistant *Streptococcus pneumoniae*

To research the attributable case fatality and the attributable LOS to penicillin and macrolide resistant *Streptococcus pneumoniae* (PMRSP) the following databases were searched: PubMed, EMBASE and Cochrane Database of Systematic Reviews. We performed an advanced search on Pubmed using MeSH term “Streptococcus pneumoniae” and all fields “penicillin and macrolide resistant”. Embase was searched for “penicillin and macrolide resistant’’ AND streptococcus pneumoniae”. An advanced search on the Cochrane Library was performed using terms “streptococcus pneumoniae” and “penicillin and macrolide”. No language restrictions were made and filter for research in humans was applied.

The following flow diagram in Figure 15 describes the steps in the literature review of penicillin resistant Streptococcus pneumoniae.

Figure 15: Search for studies on Penicillin- and macrolide-resistant *Streptococcus pneumoniae*. Inclusion and exclusion diagram.

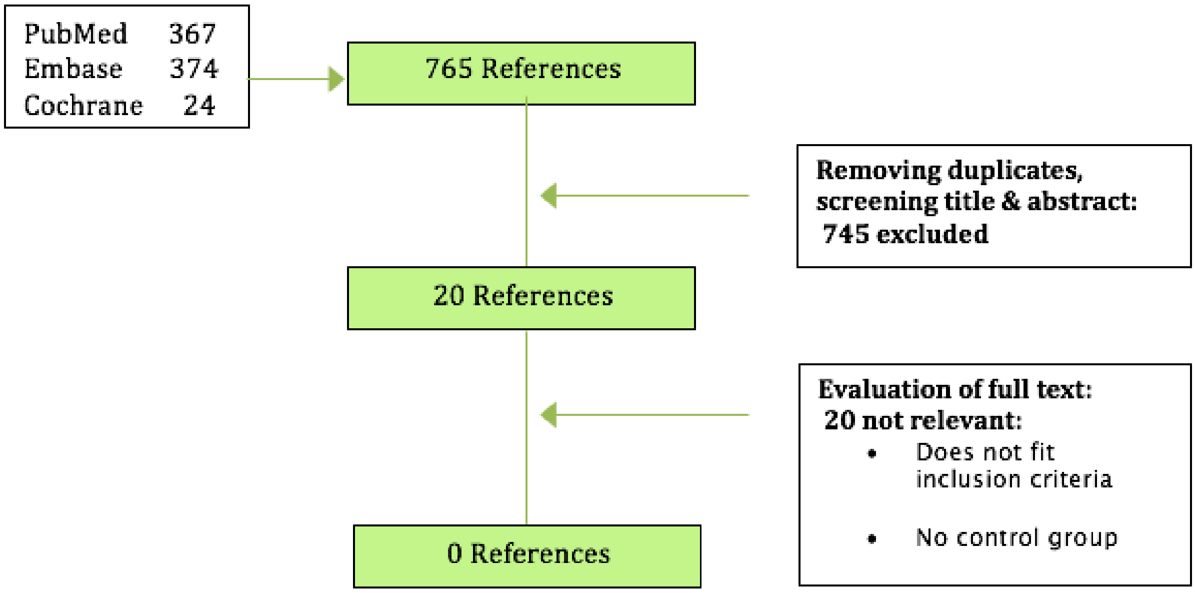

Unfortunately, no studies were found which focused solely on penicillin and macrolide resistant *Streptococcus pneumoniae*. Studies were available for penicillin resistant *streptococcus pneumoniae* and macrolide resistant *streptococcus pneumoniae* individually.

16. Vancomycin resistant *Enterococcus faecalis* and faecium

To research the attributable case fatality and the attributable LOS to vancomycin resistant *Enterococcus faecalis* and *faecium* (VRE)*,* we searched the following databases: PubMed, EMBASE and Cochrane Database of Systematic Reviews. We performed an advanced search on Pubmed using MeSH term “Enterococcus faec*” and all fields “vancomycin-resistant”. Embase was searched for “vancomycin resistant’’ AND “enterococcus faec*”. An advanced search on the Cochrane Library was performed using terms “enterococcus faec*” and “vancomycin’’.
No language restrictions were made, and filter for research in humans was applied.

Relevant data were extracted from relevant studies identified during the literature search using a standardised data extraction form.

The following flow diagram describes the steps in the literature review of vancomycin-resistant Enterococcus.

Once identified the studies reporting relevant data were grouped based on the site of infection. All eligible studies reported a VRE infected group and a control group. The latter could represent NI or S infections.

Figure 16: Search for studies on Vancomycin resistant *Enterococcus faecalis or faecium*. Inclusion and exclusion diagram.

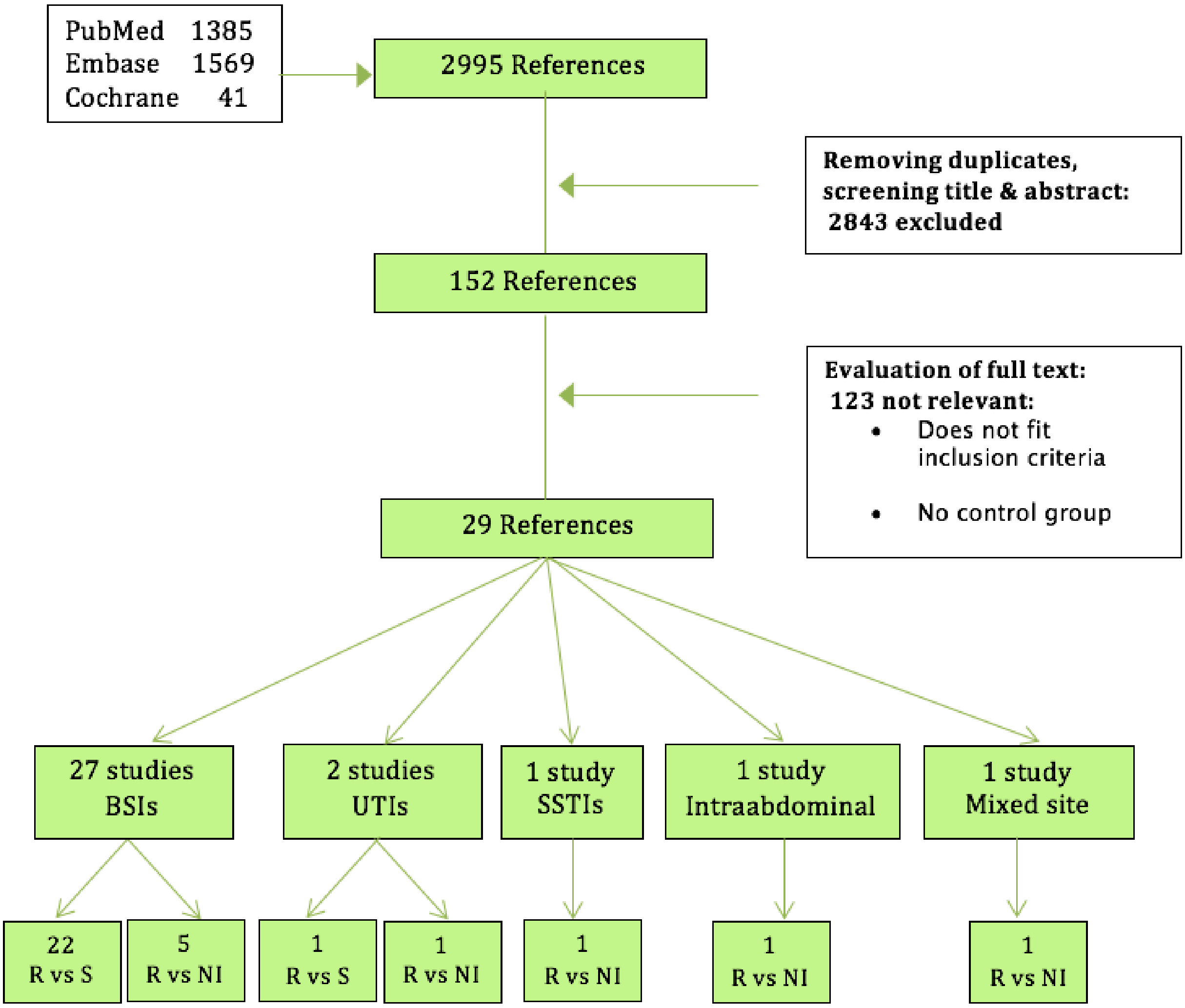

The sum of the studies categorised for infection site is higher than the total of 29 eligible studies because one study, Carmeli et al. 2002[312], reported specific data for different infection sites, therefore was categorised three times.
In addition, two meta-analyses reviewing bacteraemia were found and the references included in the systematic reviews were hand-searched for further primary studies missed in the literature search[313,314].

The following tables include studies reporting results for BSIs, UTIs, SSTIs and other infections from VRE, based on the comparison group they used. All information is reported as was presented in the original papers. All the included studies reported at least one health or economic outcome of interest.

Table 67. Short extraction table of original articles on BSI mortality of VRE infected patients vs. VSE patients.

Nr	Author	Study Period	Study type	Outcome	Location	Period of outcome	Population	Matching criteria	LOS (days)	Cost per admission
1	Da Silva 2014[315]	1998 - 2008	Retrospective cohort study	OR 2.73; 95% CI: 1.09-7.78	Brazil	In-hospital	30 cases<br>284 controls	same unit and time interval	N/A	N/A
2	Yoo 2005[316]	2000 – 2001	Retrospective cohort study	Mort. rate cases=78.9%<br>Mort. rate controls=62.5%	Seoul	Post-discharge	19 cases<br>8 controls	same time period	Mean<br>Case 56.2 (28.2)<br>Control 22.9 (13.3)	N/A
3	Lodise 2002[317]	1996 – 2000	Retrospective matched case-control	a)Mort. rate cases=52.8%<br>Mort. rate controls=26.4%<br>aOR=4.0; 95%CI(1.2–13.3)<br>a)Mort. rate cases=37.7%<br>Mort. rate controls=20.8%<br>aOR=52; 95%CI(1.4-2.0)	Michigan, USA	7 days<br>a) all-cause mortality<br>b) infection related mortality	53 cases<br>53 controls	age, APACHE score at onset, admitting service, LOS prior to onset	Mean<br>Case 22.7 (1.88)<br>Control 15.9 (1.7)<br>(after diagnosis)	N/A
4	Peset 2000[318]	1994 – 1996	Prospective case-control	Mort. rate cases=23.5%<br>Mort. rate controls=12.4%<br>OR=2.7; 95%CI(0.47–7.89)	Valencia, Spain	In-hospital	17 cases<br>169 controls	Controls selected consecutively	Mean<br>Case: 74.75 (46.27)<br>Control 47.26 (37.59)	N/A
5	Garbutt 2000[319]	1995 – 1997	Retrospective cohort	a)Mort. rate cases=48%<br>Mort. rate controls=30%<br>OR=1.74; 95%CI(0.50–6.12)<br>b)Mort. rate cases=43%<br>Mort. rate controls=36%<br>OR=1.28; 95%CI(0.47–3.51)	Missouri, USA	In-hospital	46 cases<br>23 controls		Mean<br>Case: 39.7<br>Control: 20.9	N/A
6	Bhavnani 2000[320]	1995 – 1997	Multicentre, case-control	a)Mort. rate cases=52%<br>Mort. rate controls=27%<br>aOR=3.34; 95%CI(1.61-6.91)<br>b)Mort. rate cases=32%	USA	Within 30-days of BSI	150 cases<br>150 controls	Time of occurrence and	N/A	N/A

				Mort. rate controls=17%				enterococcal species		
7	Lucas 1998[321]	1991 – 1996	Case-control	Mort. rate cases=45%<br>Mort. rate controls=27%<br>aOR=2.07; 95%CI(0.96-4.42)	Baltimore, USA	In-hospital	93 cases<br>101 controls	Timing	Mean<br>Case: 49.1 (48.3)<br>Control: 38.6 (31.2)	N/A
8	Stosor 1998[322]	1992 – 1995	Retrospective comparison	a) Mort. rate cases=76%<br>Mort. rate controls=41%<br>b)Mort. rate cases=38%<br>Mort. rate controls=9%<br>c)Mort. rate cases=24%<br>Mort. rate controls=19%<br>d)Mort. rate cases=14%<br>Mort. rate controls=13%	Chicago, USA	In-hospital<br>a) overall mortality<br>b) infection directly related mortality<br>c) infection indirectly related mortality<br>d) unrelated mortality	21 cases<br>32 controls		Mean<br>Case: 34.8 (23.3)<br>Control 16.7 (16.1)	Case $83,897<br>Control $56,707<br>Excess cost: $27,190
9	Linden 1996[323]	1991 – 1994	Retrospective cohort	a)Mort. rate cases=57%<br>Mort. rate controls=35%<br>aRR=3.47(1.47-8.19)<br>b)Mort. rate cases=46%<br>Mort. rate controls=25%	Pittsburgh, USA	In-hospital a) all-cause mortality<br>b) infection related mortality	54 cases<br>48 controls	Liver transplant patients with VREF or VSEF	Median: Case 46<br>Control 19<br>After diagnosis	N/A
10	Krcmery 2001[324]		Retrospective review	Mort. rate cases=22%<br>Mort. rate controls=41.3%	Slovak Republic		9 cases<br>92 controls		N/A	N/A
11	Vergis, 2000 [325]	1995-1997	Prospective multicentre observational	Mort. rate cases=33%<br>Mort. rate controls=11%<br>aOR=2.1(1.14-3.88)	USA	14 days from 1st positive BC	147 cases<br>251 controls		N/A	N/A
12	Shay 1995 [326]	1991 – 1993	Retrospective case- control	Mort. rate cases=33%<br>Mort. rate controls=6.5%<br>aOR=3.3(0.7-15)	NY, USA		46 cases<br>46 controls	Randomly selected control pts during study period	N/A	N/A
13	Wells 1995 [327]	1994	Retrospective case-control	Mort. rate cases=17%<br>Mort. rate controls=27%	USA	In-hospital	6 cases<br>37 controls	Same time period	N/A	N/A
14	Cheah 2013 [328]	2002 – 2010	Retrospective matched cohort	Mort. rate cases=36%<br>Mort. rate controls=26%<br>aOR=1.21; 95%CI(0.52-2.79)	Australia	In-hospital	116 cases<br>116 controls	Date and unit of admission	Median excess 4.89 (0.56 – 11.52)	Excess cost: Australian $ 28,872

15	DiazGranados 2005 [329]	1994 – 2001	Retrospective cohort	Mort. rate cases=63.6%<br>Mort. rate controls=41%<br>aHR=4.97; 95%CI(1.21-20.44)	Atlanta, USA	60 days from BSI	22 cases<br>61 controls	Patients with neutropenia	Median Case:<br>42 (24-75)<br>Control<br>31 (2-72)	N/A
16	Peel 2012 [330]	2000-2009	Case-case-control	Mort. rate resistant=37.5%<br>Mort. rate sensitive=23.8%<br>Mort. rate NI=2.5%	Australia		80 resistant<br>80 sensitive<br>80 NI	Time	N/A	N/A
17	Mainous 1997 [331]	1992 – 1994	Retrospective cohort	Mort. rate cases=40%<br>Mort. rate controls=39%	Baltimore, USA	In-hospital	10 cases<br>31 controls	Stay in SICU	mean (SD)<br>cases=28 (18)<br>controls=12 (10)	N/A
18	Salgado 2003[314]	01/86-04/02	Review and Meta-analyses	a)Mort. rate cases=41.5(0-66.7)%<br>Mort. rate controls=47.8(17-100)%<br>b)Mort. rate cases=45.2%<br>Mort. rate controls=19.0%<br>RR=2.38; 95%CI(2.13-2.66)<br>c)Mort. rate cases=48.9%<br>Mort. rate controls=19%<br>RR=2.57; 95%CI(2.27-2.91)<br>d)Mort. rate cases=28.7%<br>Mort. rate controls=10.9%<br>RR=2.62; 95%CI(1.84-3.73)<br>e)Mort. rate cases=39.1%<br>Mort. rate controls=21.8%<br>RR=1.79; 95%CI(1.28-1-5)<br>f) Attributable mortality =17-46%	World	a)crude mortality rates of pooled cases (14 studies) and controls (26 studies)<br>b) pooled cases and controls from 13 studies<br>c) pooled cases and controls from 9 studies with higher sample size<br>d) pooled cases and controls from 5 studies with infection related mortality<br>e) studies of point d) excluded one that was producing heterogeneity<br>f) range of values from 4 studies which matched for severity of illness	856 cases<br>1180 controls		Excess of total stay of VRE vs VSE=10-46 days	N/A
19	DiazGranados 2005(b)[313]	Up to 03/03	Review and Meta-analyses	OR=2.52; 95%CI(1.9–3.4)	World	N/A	683 cases<br>891 controls (pooled cases and controls	Severity of illness	N/A	N/A

							from 9 studies)			
20	CDC 1993[332]	01/89-03/93	Surveillance study	Mort. rate cases=36.6%<br>Mort. rate controls=16.4%	USA	not specified, but most probably mortality in hospital	71 cases<br>1810 controls		N/A	N/A
21	Lautenbach 1999[333]	01/93-12/95	Historical cohort study	Mort. rate cases=45.8%<br>Mort. rate controls=33.5%	USA	not specified, but most probably mortality in hospital	72 cases<br>188 controls	Unit	median (range)<br>cases= 23 (19.18-27.82)<br>controls= 12 (9.0-15.0)	N/A
22	Stroud 1996[334]	01/89-06/93	Retrospective cohort study	Mort. rate cases=69%<br>Mort. rate controls=76%	USA	not specified, but most probably mortality in hospital	26 cases<br>119 controls		overall cases+controls<br>mean(range)=8<br>1(9-583)<br>median=60	N/A

Table 68. Short extraction table of original articles on BSI mortality of VRE infected patients versus NI

Nr	Author	Study Period	Study type	Outcome	Location	Period of outcome	Population	Matching criteria	LOS (days)	Cost per admission
1	Song 2003 [335]	1993 - 2000	Population-based matched cohort study	Mort. rate cases=50.3%<br>Mort. rate controls=27.7%<br>aOR=3.04; 95%CI(1.66-5.53)	Baltimore, USA	In-hospital	159 cases<br>159 controls	*	Median<br>Case 53<br>Control 28	Excess cost $81,208
2	Carmeli 2002 [312]	1993 - 1997	Matched cohort study	RR= 12.3<br>Attributable mortality=25%	USA	In-hospital	233 cases<br>647 controls	**	Multiplicative effect: 2.06<br>Attributable days 15	Attributable $13,537

3	Edmond 1996 [336]	1993 – 1995	Matched retrospective cohort study	Mort. rate cases=67%<br>Mort. rate controls=30%<br>OR=2.3; 95%CI(1.2-4.1)	USA	In-hospital	27 cases<br>27 controls	***	N/A	N/A
4	Edmond 1995 [337]	1993	Pairwise matched case-control	Mort. rate cases=73%<br>Mort. rate controls=30%	USA	In-hospital	11 cases<br>22 controls	****	Mean Case: 74.75± 46.27<br>Control: 47.26± 37.59	N/A
5	Butler 2010 [338]	2002-2003	Retrospective cohort	Mort. rate resistant= 32%<br>Mort. rate sensitive=17%<br>Mort. rate controls=4%	Missouri, USA	In-hospital	94 resistant<br>182 sensitive<br>20150 NI	Non-surgical patients	Attributable LOS VRE: 2.2 (1.7-2.7)<br>Attributable LOS VSE: 1.1 (0.9-1.4)	Attributable costs<br>VRE: $4,036 (3,170-5,140)<br>VSE: $2,023 (1,588-2,575)

*matched based on age, year of admission, exposure time, same principle diagnosis at admission, same primary procedure, same APR-DRGs, subgroup of matched case-patients and control patients who had the same APR-DRG complexity level.
** hospital ward, calendar date (within 7 days), duration of hospital stay at time of matching, up to three controls for each case.
***control hospitalised during same period and at least as long as case patient; matched for underlying disease, matched for age within 10 years, matched for gender, matched for surgical procedure.
****hospitalised on oncology unit during study period. Underlying haematological disorder, hospitalised for at least as long as matched case patient had been up until 1st BC yielded VREF.
Five studies reported mortality for patients with VRE BSI compared with no infection. Two of these studies undertook a multivariate analysis adjusting for severity of illness.

One study was found for UTI mortality for patients with VRE versus VSE. This study only reported mortality and no other outcome of interest.

Table 69: Short extraction table of original articles on UTI mortality of VRE infected patients versus VSE patients.

**Author**	**Study Period**	**Study type**	**Outcome**	**Location**	**Period of outcome**	**Population**	**Matching criteria**	**LOS (days)**
Khair 2013 [339]	2011	Prospective cohort study	Mort. rate cases=14%<br>Mort. rate controls=7%	Missouri, USA	In-hospital	74 cases<br>180 controls	N/A	after bacteruria<br>mean (range)<br>cases= 6.8(0.1-150.8)<br>controls= 5(0.2-78.2)

One study was found for reporting mortality, LOS and cost for patients with VRE UTIs, SSTIs and VRE intraabdominal infection versus non-infected patients (NI).

Table 70. Short extraction table of original articles on UTI mortality of VRE infected patients versus NI patients.

Author	Study Period	Study type	Outcome*	Location	Period of outcome	Population	Matching criteria	LOS (days)**	Cost per admission
Carmeli 2002 [312]	1993 – 1997	Matched cohort study	RR: 4.28<br>Attributable to VRE: 9%	USA	In-hospital	233 cases<br>647 controls	***	ME:1.75<br>Attributable 5.4 days	Attributable: $6,665

*Adjusted mortality reported as RR and attributable percentage
**Study reported LOS as ME (multiplicative effect) and attributable days
***hospital ward, calendar date (within 7 days), duration of hospital stay at time of matching, up to three controls for each case.

Table 71. Short extraction table of original articles on SST mortality of VRE infected patients versus NI patients.

Author	Study Period	Study type	Outcome*	Location	Period of outcome	Population	Matching criteria	LOS (days)**	Cost per admission
Carmeli 2002 [312]	1993 – 1997	Matched cohort study	RR: 2.00<br>Attributable to VRE: 6%	USA	In-hospital	233 cases<br>647 controls	***	ME: 1.78 Attributable 6.2 days	Attributable: $13,884

*Adjusted mortality reported as RR and attributable percentage
**Study reported LOS as ME (multiplicative effect) and attributable days
*** hospital ward, calendar date (within 7 days), duration of hospital stay at time of matching, up to three controls for each case.

Table 72. Short extraction table of original articles on intraabdominal infection of VRE infected patients versus NI patients.

*Adjusted mortality reported as RR and attributable percentage

**Author**	**Study Period**	**Study type**	**Outcome***	**Location**	**Period of outcome**	**Population**	**Matching criteria**	**LOS (days)****	**Cost per admission**
Carmeli 2002 [312]	1993 – 1997	Matched cohort study	RR: 3.75<br>Attributable to VRE: 3%	USA	In-hospital	233 cases<br>647 controls		ME: 1.42 Attributable 2.6 days	Attributable: $22,896

***Study reported LOS as ME (multiplicative effect) and attributable days

Table 73. Short extraction table of original articles of VRE infected patients versus NI patients for mixed site of infection

**Author**	**Study Period**	**Study type**	**Outcome**	**Location**	**Period of outcome**	**Population**	**Matching criteria**	**LOS (days)**	**Cost per admission**
Tornieporth, 1996 [340]	1990-1992	Case control study	a)Mort. rate cases=37%<br>Mort. rate controls=19%<br>b)Mort. rate cases=58%<br>Mort. rate controls=43%	NY, USA	In-hospital<br>a) overall mortality<br>b) ICU admitted patients mortality	24 cases<br>23 controls	VSEF isolates within 1 month of VREF isolates	mean (range)<br>cases=64.5 (2-290)<br>controls=40.8 (2-248)<br>median<br>cases= 48<br>controls=26	N/A

The most recent published eligible study found was in 2014 with studies found dating back to 1995. A limited number of studies focused solely on Enterococcus faecium and faecalis but rather more generally on enterococcal species. These two species do however constitute the majority of VRE infections.

Conclusions

The literature reviews conducted to determine the attributable case fatality and the attributable length of stay due to the selected resistant bacterial infections have detected 281 original articles eligible for the purposes of the project. These peer reviewed publications vary based, amongst other, for the underlying study design, type of patients, time of outcome, control for adjusting and confounding variables, unit or set of study.

As described in the objectives of our literature review, for each pathogen/resistance combination we categorized the detected studies based on the site of infection and on the control group they used to compare the cases with the resistant infection.
We noticed a wide difference between the studies identified in our search. Many are limited to specific healthcare services (e.g. ICUs) and/or performed on particular groups of patients (e.g. only children, only elderly) and/or with specific underlying conditions (e.g. immunodeficiency, organ transplant). Therefore, extracting valid case fatality proportions from published risk differences for the burden of AMR project will be particularly challenging.

Based on the heterogeneity of the studies detected, we advise to avoid pooling and averaging the results based on our categorizing criteria. We suggest below a list of methods for the selection of the studies identified. This list is not mutually exclusive and we recommend using a combination of the methods below to identify the published data to include in the disease models for the burden of AMR project.

Different Approaches

a) Grading of Recommendations, Assessment, Development and Evaluations (GRADE); it is a widely accepted systematic and explicit approach to grading the quality of evidence and strength of recommendations. This approach was used in the WHO 2014 Antimicrobial Resistance Global Report on Surveillance. The quality of evidence was collected into four categories: very low, low, moderate and high. Most studies identified in the WHO report were observational and were graded as low and very low. Our systematic review also found a predominance of observational studies and we can easily deduce that the evidence offered by our detected studies might be similar.

b) Sample size criteria; in every statistical and inferential approach the sample size effect is fundamental to determine if an observed value or characteristic is just a case or an evidence of a reliable and repeatable phenomenon that describes the studied population. Very often studies on antibiotic resistant infections are not based on a large population and they do not include sample size calculations. A small sample size

weakens the evidence produced by their results; the approach, therefore, would consist of including only studies that have a “large” sample size. The definition of large can be described as consistent with a sample size calculation that we can perform based on the data available in literature.

c) Control for confounders and biases; the control for confounders and biases is another fundamental criterion in the evaluation of the evidence. The best quality studies we identified were matched case control studies with matching for the main variables of the two groups that might interfere in the disease outcome (e.g. age, underlying health conditions, therapy etc.). The final selection would include studies that control the cases and control groups by matching them for specific and defined variables or those performing statistical analysis that controls for those variables (e.g. multivariate logistic regression model). If a study seems of good quality but lacks one or two essential quality criteria (e.g. control for confounders and biases), one could contact the author/s of the study and ask for raw data. However, this approach might be very time-consuming and the outcome is uncertain.

d) Meta-analysis; Meta-analyses of the results of all detected studies as they are reported in the articles could be possible. Another approach would entail extracting information on each single patient and their characteristics and insert them in a self-build database. However, this would require contacting authors in order to fill in the information gaps of the published studies.

e) Self-designed evaluation grid; the previously described approaches (b-d) give us useful information on the quality and reliability of the evidence produced by a study. this information could be pooled together in a grid showing the selected criteria to be assigned to every study (we suggest those listed in b, c and d). The criteria would be qualitative ordinal variables with pre-assigned values. The grid would lead to an approach similar to the GRADE but could be tailored for studies on antibiotic resistance. The aspects to consider and their ranking should be agreed with antibiotic resistance experts.

The purpose of this summary is to list some aspects that should be considered in the evaluation of the detected studies.
Further expert’s opinions and a specific literature research can help considering the best approach and include additional aspects.

References

1. Kim SB, Min YH, Cheong JW, et al. Incidence and risk factors for carbapenem- and multidrug-resistant Acinetobacter baumannii bacteremia in hematopoietic stem cell transplantation recipients. *Scandinavian journal of infectious diseases.* Feb 2014;46(2):81-88.
2. Thatrimontrichai A, Apisarnthanarak A, Chanvitan P, Janjindamai W, Dissaneevate S, Maneenil G. Risk factors and outcomes of carbapenem-resistant Acinetobacter baumannii bacteremia in neonatal intensive care unit: a case-case-control study. *The Pediatric infectious disease journal.* Feb 2013;32(2):140-145.
3. Deris ZZ, Shafei MN, Harun A. Risk factors and outcomes of imipenem-resistant Acinetobacter bloodstream infection in North-Eastern Malaysia. *Asian Pacific journal of tropical biomedicine.* Aug 2011;1(4):313-315.
4. Esterly JS, Griffith M, Qi C, Malczynski M, Postelnick MJ, Scheetz MH. Impact of carbapenem resistance and receipt of active antimicrobial therapy on clinical outcomes of Acinetobacter baumannii bloodstream infections. *Antimicrobial agents and chemotherapy.* Oct 2011;55(10):4844-4849.
5. Huang ST, Chiang MC, Kuo SC, et al. Risk factors and clinical outcomes of patients with carbapenem-resistant Acinetobacter baumannii bacteremia. *Journal of microbiology, immunology, and infection = Wei mian yu gan ran za zhi.* Oct 2012;45(5):356-362.
6. Jamulitrat S, Arunpan P, Phainuphong P. Attributable mortality of imipenem-resistant nosocomial Acinetobacter baumannii bloodstream infection. *Journal of the Medical Association of Thailand = Chotmaihet thangphaet.* Mar 2009;92(3):413-419.
7. Kim YJ, Kim SI, Hong KW, Kim YR, Park YJ, Kang MW. Risk factors for mortality in patients with carbapenem-resistant Acinetobacter baumannii bacteremia: impact of appropriate antimicrobial therapy. *Journal of Korean medical science.* May 2012;27(5):471-475.
8. Kumar A, Randhawa VS, Nirupam N, Rai Y, Saili A. Risk factors for carbapenem-resistant Acinetobacter baumanii blood stream infections in a neonatal intensive care unit, Delhi, India. *Journal of infection in developing countries.* Aug 13 2014;8(8):1049-1054.
9. Routsi C, Pratikaki M, Platsouka E, et al. Carbapenem-resistant versus carbapenem-susceptible Acinetobacter baumannii bacteremia in a Greek intensive care unit: risk factors, clinical features and outcomes. *Infection.* Jun 2010;38(3):173-180.
10. Tal-Jasper R, Katz DE, Amrami N, et al. Clinical and epidemiological significance of carbapenem resistance in Acinetobacter baumannii infections. *Antimicrobial agents and chemotherapy.* 2016;60(5):3127-3131.
11. Thatrimontrichai A, Techato C, Dissaneevate S, et al. Risk factors and outcomes of carbapenem-resistant Acinetobacter baumannii ventilator-associated pneumonia in the neonate: A case-case-control study. *Journal of Infection and Chemotherapy.* 2016;22(7):444-449.
12. Chang HC, Chen YC, Lin MC, et al. Mortality risk factors in patients with Acinetobacter baumannii ventilator: associated pneumonia. *Journal of the Formosan Medical Association = Taiwan yi zhi.* Sep 2011;110(9):564-571.
13. Garnacho-Montero J, Ortiz-Leyba C, Jimenez-Jimenez FJ, et al. Treatment of multidrug-resistant Acinetobacter baumannii ventilator-associated pneumonia (VAP) with intravenous colistin: a comparison with imipenem-susceptible VAP. *Clinical infectious diseases : an official publication of the Infectious Diseases Society of America.* May 1 2003;36(9):1111-1118.

14. Zheng YL, Wan YF, Zhou LY, et al. Risk factors and mortality of patients with nosocomial carbapenem-resistant Acinetobacter baumannii pneumonia. *American journal of infection control.* Jul 2013;41(7):e59-63.
15. Henig O, Weber G, Hoshen MB, et al. Risk factors for and impact of carbapenem-resistant Acinetobacter baumannii colonization and infection: matched case-control study. *European journal of clinical microbiology & infectious diseases : official publication of the European Society of Clinical Microbiology.* Oct 2015;34(10):2063-2068.
16. Nazer LH, Kharabsheh A, Rimawi D, Mubarak S, Hawari F. Characteristics and Outcomes of Acinetobacter baumannii Infections in Critically Ill Patients with Cancer: A Matched Case-Control Study. *Microbial drug resistance (Larchmont, N.Y.).* Oct 2015;21(5):556-561.
17. Aydemir H, Celebi G, Piskin N, et al. Mortality attributable to carbapenem-resistant nosocomial Acinetobacter baumannii infections in a Turkish university hospital. *Japanese journal of infectious diseases.* 2012;65(1):66-71.
18. de Gouvea EF, Martins IS, Halpern M, et al. The influence of carbapenem resistance on mortality in solid organ transplant recipients with Acinetobacter baumannii infection. *BMC infectious diseases.* Dec 13 2012;12:351.
19. del Mar Tomas M, Cartelle M, Pertega S, et al. Hospital outbreak caused by a carbapenem-resistant strain of Acinetobacter baumannii: patient prognosis and risk-factors for colonisation and infection. *Clinical microbiology and infection : the official publication of the European Society of Clinical Microbiology and Infectious Diseases.* Jul 2005;11(7):540-546.
20. Lautenbach E, Synnestvedt M, Weiner MG, et al. Epidemiology and impact of imipenem resistance in Acinetobacter baumannii. *Infection control and hospital epidemiology.* Dec 2009;30(12):1186-1192.
21. Lemos EV, de la Hoz FP, Alvis N, et al. Impact of carbapenem resistance on clinical and economic outcomes among patients with Acinetobacter baumannii infection in Colombia. *Clinical microbiology and infection : the official publication of the European Society of Clinical Microbiology and Infectious Diseases.* Feb 2014;20(2):174-180.
22. Lemos EV, de la Hoz FP, Einarson TR, et al. Carbapenem resistance and mortality in patients with Acinetobacter baumannii infection: systematic review and meta-analysis. *Clinical microbiology and infection : the official publication of the European Society of Clinical Microbiology and Infectious Diseases.* May 2014;20(5):416-423.
23. Sheng WH, Liao CH, Lauderdale TL, et al. A multicenter study of risk factors and outcome of hospitalized patients with infections due to carbapenem-resistant Acinetobacter baumannii. *International journal of infectious diseases : IJID : official publication of the International Society for Infectious Diseases.* Sep 2010;14(9):e764-769.
24. Brooklyn Antibiotic Resistance Task Force. The cost of antibiotic resistance: effect of resistance among Staphylococcus aureus, Klebsiella pneumoniae, Acinetobacter baumannii, and Pseudmonas aeruginosa on length of hospital stay. *Infection control and hospital epidemiology.* Feb 2002;23(2):106-108.
25. Wang YC, Lee YT, Yang YS, et al. Risk factors and outcome for colistin-resistant Acinetobacter nosocomialis bacteraemia in patients without previous colistin exposure. *Clinical microbiology and infection : the official publication of the European Society of Clinical Microbiology and Infectious Diseases.* Aug 2015;21(8):758-764.
26. Taneja N, Singh G, Singh M, Sharma M. Emergence of tigecycline & colistin resistant Acinetobacter baumanii in patients with complicated urinary tract infections in north India. *Indian J Med Res.* Jun 2011;133:681-684.
27. Baran I, Mumcuoglu I, Aksu N, et al. Evaluation of colistin-resistant Acinetobacter strains. *Clinical Microbiology and Infection.* 2012;18:321.

28. Kontopidou F, Plachouras D, Papadomichelakis E, et al. Colonization and infection by colistin-resistant Gram-negative bacteria in a cohort of critically ill patients. *Clinical Microbiology and Infection.* 2011;17(11):E9-E11.
29. Lee SY, Shin JH, Park KH, et al. Identification, genotypic relation, and clinical features of colistin-resistant isolates of Acinetobacter genomic species 13BJ/14TU from bloodstreams of patients in a university hospital. *Journal of Clinical Microbiology.* 2014;52(3):931-939.
30. Qureshi ZA, Hittle LE, O'Hara JA, et al. Colistin-resistant acinetobacter baumannii: Beyond carbapenem resistance. *Clinical Infectious Diseases.* 2015;60(9):1295-1303.
31. Al Jarousha AM, El Jadba AH, Al Afifi AS, El Qouqa IA. Nosocomial multidrug-resistant Acinetobacter baumannii in the neonatal intensive care unit in Gaza City, Palestine. *International journal of infectious diseases : IJID : official publication of the International Society for Infectious Diseases.* Sep 2009;13(5):623-628.
32. Gulen TA, Guner R, Celikbilek N, Keske S, Tasyaran M. Clinical importance and cost of bacteremia caused by nosocomial multi drug resistant acinetobacter baumannii. *International journal of infectious diseases : IJID : official publication of the International Society for Infectious Diseases.* Sep 2015;38:32-35.
33. Anunnatsiri S, Tonsawan P. Risk factors and clinical outcomes of multidrug-resistant Acinetobacter baumannii bacteremia at a university hospital in Thailand. *The Southeast Asian journal of tropical medicine and public health.* May 2011;42(3):693-703.
34. Fitzpatrick MA, Ozer E, Bolon MK, Hauser AR. Influence of ACB complex genospecies on clinical outcomes in a U.S. hospital with high rates of multidrug resistance. *The Journal of infection.* Feb 2015;70(2):144-152.
35. Guo N, Xue W, Tang D, Ding J, Zhao B. Risk factors and outcomes of hospitalized patients with blood infections caused by multidrug-resistant Acinetobacter baumannii complex in a hospital of Northern China. *American journal of infection control.* 2016;44(4):e37-e39.
36. Lee NY, Lee HC, Ko NY, et al. Clinical and economic impact of multidrug resistance in nosocomial Acinetobacter baumannii bacteremia. *Infection control and hospital epidemiology.* Jun 2007;28(6):713-719.
37. Lee NY, Chang TC, Wu CJ, et al. Clinical manifestations, antimicrobial therapy, and prognostic factors of monomicrobial Acinetobacter baumannii complex bacteremia. *The Journal of infection.* Sep 2010;61(3):219-227.
38. Smolyakov R, Borer A, Riesenberg K, et al. Nosocomial multi-drug resistant Acinetobacter baumannii bloodstream infection: risk factors and outcome with ampicillin-sulbactam treatment. *The Journal of hospital infection.* May 2003;54(1):32-38.
39. Cai XF, Sun JM, Bao LS, Li WB. Risk factors and antibiotic resistance of pneumonia caused by multidrug resistant Acinetobacter baumannii in pediatric intensive care unit. *World Journal of Emergency Medicine.* 2012;3(3):202-207.
40. Inchai J, Pothirat C, Bumroongkit C, Limsukon A, Khositsakulchai W, Liwsrisakun C. Prognostic factors associated with mortality of drug-resistant Acinetobacter baumannii ventilator-associated pneumonia. *Journal of Intensive Care.* 2015;3(1).
41. Park II, Kim IK, Koo HC, et al. Clinical characteristics and prognosis of Acinetobacter nosocomial pneumonia between MDR and non-MDR. *Tuberculosis and Respiratory Diseases.* 2006;61(1):13-19.
42. Abbo A, Carmeli Y, Navon-Venezia S, Siegman-Igra Y, Schwaber MJ. Impact of multi-drug-resistant Acinetobacter baumannii on clinical outcomes. *European journal of clinical microbiology & infectious diseases : official publication of the European Society of Clinical Microbiology.* Nov 2007;26(11):793-800.

43. Fukuta Y, Doi Y, Muder RR, et al. Risk factors for acquisition of multi-drug resistant Acinetobacter baumannii among cancer patients. *International Journal of Antimicrobial Agents.* 2013;42:S140-S141.
44. Lee H, Lee H. Clinical and economic evaluation of multidrug-resistant Acinetobacter baumannii colonization in the intensive care unit. *Infection and Chemotherapy.* 2016;48(3):174-180.
45. Brahmi N, Beji O, Abidi N, et al. Epidemiology and risk factors for colonization and infection by Acinetobacter baumannii in an ICU in Tunisia, where this pathogen is endemic. *Journal of infection and chemotherapy : official journal of the Japan Society of Chemotherapy.* Dec 2007;13(6):400-404.
46. Daniels TL, Deppen S, Arbogast PG, Griffin MR, Schaffner W, Talbot TR. Mortality rates associated with multidrug-resistant Acinetobacter baumannii infection in surgical intensive care units. *Infection control and hospital epidemiology.* Nov 2008;29(11):1080-1083.
47. Lemos EV, De la Hoz Restrepo F, Alvis N, Quevedo E, Canon O, Leon Y. [Acinetobacter baumannii - related mortality in intensive care units in Colombia]. *Revista panamericana de salud publica = Pan American journal of public health.* Oct 2011;30(4):287-294.
48. Pierri MD, Crescenzi G, Capestro F, et al. Risk Factors and Impact on Clinical Outcome of Multidrug-Resistant Acinetobacter Baumannii Acquisition in Cardiac Surgery Patients. *Journal of Cardiothoracic and Vascular Anesthesia.* 2016;30(3):680-686.
49. Zilberberg MD, Nathanson BH, Sulham K, Fan W, Shorr AF. Multidrug resistance, inappropriate empiric therapy, and hospital mortality in Acinetobacter baumannii pneumonia and sepsis. *Critical Care.* 2016;20(1).
50. Lambert ML, Suetens C, Savey A, et al. Clinical outcomes of health-care-associated infections and antimicrobial resistance in patients admitted to European intensive-care units: A cohort study. *The Lancet Infectious Diseases.* 2011;11(1):30-38.
51. Chang HJ, Hsu PC, Yang CC, et al. Risk factors and outcomes of carbapenem-nonsusceptible Escherichia coli bacteremia: A matched case-control study. *Journal of Microbiology, Immunology and Infection.* 2011;44(2):125-130.
52. Epstein L, Hunter JC, Arwady MA, et al. New Delhi metallo-β-lactamase-producing carbapenem-resistant escherichia coli associated with exposure to duodenoscopes. *JAMA - Journal of the American Medical Association.* 2014;312(14):1447-1455.
53. Ahn JY, Song JE, Kim MH, et al. Risk factors for the acquisition of carbapenem-resistant Escherichia coli at a tertiary care center in South Korea: A matched case-control study. *American journal of infection control.* 2014;42(6):621-625.
54. Balkan, II, Aygun G, Aydin S, et al. Blood stream infections due to OXA-48-like carbapenemase-producing Enterobacteriaceae: treatment and survival. *International journal of infectious diseases : IJID : official publication of the International Society for Infectious Diseases.* Sep 2014;26:51-56.
55. Poirel L, Savov E, Nazli A, et al. Outbreak caused by NDM-1- and RmtB-producing Escherichia coli in Bulgaria. *Antimicrobial agents and chemotherapy.* 2014;58(4):2472-2474.
56. Porwal R, Gopalakrishnan R, Rajesh NJ, Ramasubramanian V. Carbapenem resistant Gram-negative bacteremia in an Indian intensive care unit: A review of the clinical profile and treatment outcome of 50 patients. *Indian Journal of Critical Care Medicine.* 2014;18(11):750-753.
57. Shukla N, Tulasigeri C, Kate A, et al. High carbapenem resistance in gram negative bacterial isolates: An outcome study. *American Journal of Respiratory and Critical Care Medicine.* 2013;187.

58. Liu YY, Wang Y, Walsh TR, et al. Emergence of plasmid-mediated colistin resistance mechanism MCR-1 in animals and human beings in China: a microbiological and molecular biological study. *The Lancet. Infectious diseases.* Feb 2016;16(2):161-168.
59. Nordmann P, Lienhard R, Kieffer N, Clerc O, Poirel L. Plasmid-mediated colistin-resistant Escherichia coli in bacteremia in Switzerland. *Clinical Infectious Diseases.* 2016;62(10):1322-1323.
60. Oostdijk EA, Leverstein-van Hall M, Muilwijk J, Kesecioglu J, Bonten MJ. Colistin resistance in Gram-negative bacteria during prophylactic colistin use in intensive care units. *Clinical Microbiology and Infection.* 2011;17:S294.
61. de Kraker MEA, Wolkewitz M, Davey PG, et al. Burden of antimicrobial resistance in European hospitals: Excess mortality and length of hospital stay associated with bloodstream infections due to Escherichia coli resistant to third-generation cephalosporins. *Journal of Antimicrobial Chemotherapy.* 2011;66(2):398-407.
62. Anunnatsiri S, Towiwat P, Chaimanee P. Risk factors and clinical outcomes of extended spectrum beta-lactamase (ESBL)-producing Escherichia coli septicemia at Srinagarind University Hospital, Thailand. *The Southeast Asian journal of tropical medicine and public health.* Sep 2012;43(5):1169-1177.
63. Apisarnthanarak A, Kiratisin P, Mundy LM. Predictors of mortality from community-onset bloodstream infections due to extended-spectrum beta-lactamase-producing Escherichia coli and Klebsiella pneumoniae. *Infection control and hospital epidemiology.* Jul 2008;29(7):671-674.
64. Cornejo-Juarez P, Perez-Jimenez C, Silva-Sanchez J, et al. Molecular analysis and risk factors for Escherichia coli producing extended-spectrum beta-lactamase bloodstream infection in hematological malignancies. *PloS one.* 2012;7(4):e35780.
65. Courpon-Claudinon A, Lefort A, Panhard X, et al. Bacteraemia caused by third-generation cephalosporin-resistant Escherichia coli in France: Prevalence, molecular epidemiology and clinical features. *Clinical Microbiology and Infection.* 2011;17(4):557-565.
66. Ortega M, Marco F, Soriano A, et al. Analysis of 4758 Escherichia coli bacteraemia episodes: predictive factors for isolation of an antibiotic-resistant strain and their impact on the outcome. *The Journal of antimicrobial chemotherapy.* Mar 2009;63(3):568-574.
67. Trecarichi EM, Tumbarello M, Spanu T, et al. Incidence and clinical impact of extended-spectrum-beta-lactamase (ESBL) production and fluoroquinolone resistance in bloodstream infections caused by Escherichia coli in patients with hematological malignancies. *The Journal of infection.* Apr 2009;58(4):299-307.
68. Hsieh CJ, Shen YH, Hwang KP. Clinical implications, risk factors and mortality following community-onset bacteremia caused by extended-spectrum beta-lactamase (ESBL) and non-ESBL producing Escherichia coli. *Journal of microbiology, immunology, and infection = Wei mian yu gan ran za zhi.* Jun 2010;43(3):240-248.
69. Tumbarello M, Spanu T, Di Bidino R, et al. Costs of bloodstream infections caused by Escherichia coli and influence of extended-spectrum-beta-lactamase production and inadequate initial antibiotic therapy. *Antimicrobial agents and chemotherapy.* Oct 2010;54(10):4085-4091.
70. Gudiol C, Calatayud L, Garcia-Vidal C, et al. Bacteraemia due to extended-spectrum beta-lactamase-producing Escherichia coli (ESBL-EC) in cancer patients: clinical features, risk factors, molecular epidemiology and outcome. *The Journal of antimicrobial chemotherapy.* Feb 2010;65(2):333-341.
71. Ho PL, Chan WM, Tsang KW, Wong SS, Young K. Bacteremia caused by Escherichia coli producing extended-spectrum beta-lactamase: a case-control study of risk factors and outcomes. *Scandinavian journal of infectious diseases.* 2002;34(8):567-573.

72. Rodriguez-Bano J, Picon E, Gijon P, et al. Community-onset bacteremia due to extended-spectrum beta-lactamase-producing Escherichia coli: risk factors and prognosis. *Clinical infectious diseases : an official publication of the Infectious Diseases Society of America.* Jan 1 2010;50(1):40-48.
73. Kang CI, Song JH, Chung DR, et al. Risk factors and treatment outcomes of community-onset bacteraemia caused by extended-spectrum beta-lactamase-producing Escherichia coli. *Int J Antimicrob Agents.* Sep 2010;36(3):284-287.
74. Garcia Hernandez A, Garcia-Vazquez E, Gomez Gomez J, Canteras M, Hernandez-Torres A, Ruiz Gomez J. [Predictive factors of ESBL versus non-ESBL Escherichia coli bacteraemia and influence of resistance on the mortality of the patients]. *Medicina clinica.* Jan 29 2011;136(2):56-60.
75. Denis B, Lafaurie M, Donay JL, et al. Prevalence, risk factors, and impact on clinical outcome of extended-spectrum beta-lactamase-producing Escherichia coli bacteraemia: a five-year study. *International journal of infectious diseases : IJID : official publication of the International Society for Infectious Diseases.* Oct 2015;39:1-6.
76. Ha YE, Kang CI, Cha MK, et al. Epidemiology and clinical outcomes of bloodstream infections caused by extended-spectrum beta-lactamase-producing Escherichia coli in patients with cancer. *Int J Antimicrob Agents.* Nov 2013;42(5):403-409.
77. Kaya O, Akcam FZ, Gonen I, Unal O, Ceylan T. Risk factors for bacteremia due to extended-spectrum beta-lactamase-producing Escherichia coli in a Turkish hospital. *Journal of infection in developing countries.* Jul 15 2013;7(7):507-512.
78. Kim SH, Kwon JC, Choi SM, et al. Escherichia coli and Klebsiella pneumoniae bacteremia in patients with neutropenic fever: factors associated with extended-spectrum beta-lactamase production and its impact on outcome. *Annals of hematology.* Apr 2013;92(4):533-541.
79. Leistner R, Gurntke S, Sakellariou C, et al. Bloodstream infection due to extended-spectrum beta-lactamase (ESBL)-positive K. pneumoniae and E. coli: an analysis of the disease burden in a large cohort. *Infection.* Dec 2014;42(6):991-997.
80. Leistner R, Sakellariou C, Gürntke S, et al. Mortality and molecular epidemiology associated with extended-spectrum β-lactamase production in Escherichia coli from bloodstream infection. *Infection and Drug Resistance.* 2014;7:57-62.
81. Martelius T, Jalava J, Karki T, Mottonen T, Ollgren J, Lyytikainen O. Nosocomial bloodstream infections caused by Escherichia coli and Klebsiella pneumoniae resistant to third-generation cephalosporins, Finland, 1999-2013: Trends, patient characteristics and mortality. *Infectious diseases (London, England).* 2016;48(3):229-234.
82. Park SH, Choi SM, Lee DG, et al. Emergence of extended-spectrum beta-lactamase-producing escherichia coli as a cause of community-onset bacteremia in South Korea: risk factors and clinical outcomes. *Microbial drug resistance (Larchmont, N.Y.).* Dec 2011;17(4):537-544.
83. Yip T, Tse KC, Lam MF, et al. Risk factors and outcomes of extended-spectrum beta-lactamase-producing E. coli peritonitis in CAPD patients. *Peritoneal dialysis international : journal of the International Society for Peritoneal Dialysis.* Mar-Apr 2006;26(2):191-197.
84. Van Aken S, Lund N, Ahl J, Odenholt I, Tham J. Risk factors, outcome and impact of empirical antimicrobial treatment in extended-spectrum beta-lactamase-producing Escherichia coli bacteraemia. *Scandinavian journal of infectious diseases.* Nov 2014;46(11):753-762.
85. Melzer M, Petersen I. Mortality following bacteraemic infection caused by extended spectrum beta-lactamase (ESBL) producing E. coli compared to non-ESBL producing E. coli. *The Journal of infection.* Sep 2007;55(3):254-259.

86. Al-Otaibi FE, Bukhari EE. Clinical and laboratory profiles of urinary tract infections caused by extended-spectrum beta-lactamase-producing Escherichia coli in a tertiary care center in central Saudi Arabia. *Saudi medical journal.* Feb 2013;34(2):171-176.
87. Ena J, Arjona F, Martinez-Peinado C, Lopez-Perezagua Mdel M, Amador C. Epidemiology of urinary tract infections caused by extended-spectrum beta-lactamase-producing Escherichia coli. *Urology.* Dec 2006;68(6):1169-1174.
88. Suankratay C, Jutivorakool K, Jirajariyavej S. A prospective study of ceftriaxone treatment in acute pyelonephritis caused by extended-spectrum beta-lactamase-producing bacteria. *Journal of the Medical Association of Thailand = Chotmaihet thangphaet.* Aug 2008;91(8):1172-1181.
89. Esteve-Palau E, Solande G, Sanchez F, et al. Clinical and economic impact of urinary tract infections caused by ESBL-producing Escherichia coli requiring hospitalization: A matched cohort study. *The Journal of infection.* Dec 2015;71(6):667-674.
90. Park SH, Choi SM, Lee DG, et al. Impact of extended-spectrum beta-lactamase production on treatment outcomes of acute pyelonephritis caused by escherichia coli in patients without health care-associated risk factors. *Antimicrobial agents and chemotherapy.* Apr 2015;59(4):1962-1968.
91. Arnan M, Gudiol C, Calatayud L, et al. Risk factors for, and clinical relevance of, faecal extended-spectrum beta-lactamase producing Escherichia coli (ESBL-EC) carriage in neutropenic patients with haematological malignancies. *European journal of clinical microbiology & infectious diseases : official publication of the European Society of Clinical Microbiology.* Mar 2011;30(3):355-360.
92. Nicolas-Chanoine MH, Jarlier V, Robert J, et al. Patient's origin and lifestyle associated with CTX-M-producing Escherichia coli: a case-control-control study. *PloS one.* 2012;7(1):e30498.
93. Hayakawa K, Gattu S, Marchaim D, et al. Epidemiology and risk factors for isolation of Escherichia coli producing CTX-M-type extended-spectrum beta-lactamase in a large U.S. Medical Center. *Antimicrobial agents and chemotherapy.* Aug 2013;57(8):4010-4018.
94. Pena C, Gudiol C, Calatayud L, et al. Infections due to Escherichia coli producing extended-spectrum beta-lactamase among hospitalised patients: factors influencing mortality. *The Journal of hospital infection.* Feb 2008;68(2):116-122.
95. Kang CI, Wi YM, Lee MY, et al. Epidemiology and risk factors of community onset infections caused by extended-spectrum beta-lactamase-producing Escherichia coli strains. *J Clin Microbiol.* Feb 2012;50(2):312-317.
96. Maslikowska JA, Walker SAN, Elligsen M, et al. Impact of infection with extended-spectrum β-lactamase-producing Escherichia coli or Klebsiella species on outcome and hospitalization costs. *Journal of Hospital Infection.* 2016;92(1):33-41.
97. Falagas ME, Tansarli GS, Karageorgopoulos DE, Vardakas KZ. Deaths attributable to carbapenem-resistant Enterobacteriaceae infections. *Emerging infectious diseases.* Jul 2014;20(7):1170-1175.
98. Viale P, Giannella M, Lewis R, Trecarichi EM, Petrosillo N, Tumbarello M. Predictors of mortality in multidrug-resistant Klebsiella pneumoniae bloodstream infections. *Expert Review of Anti-Infective Therapy.* 2013;11(10):1053-1063.
99. Boyle DP, Zembower TR. Epidemiology and Management of Emerging Drug-Resistant Gram-Negative Bacteria: Extended-Spectrum beta-Lactamases and Beyond. *The Urologic clinics of North America.* Nov 2015;42(4):493-505.
100. Borer A, Saidel-Odes L, Riesenberg K, et al. Attributable mortality rate for carbapenem-resistant Klebsiella pneumoniae bacteremia. *Infection control and hospital epidemiology.* 2009;30(10):972-976.

101. Gallagher JC, Kuriakose S, Haynes K, Axelrod P. Case-case-control study of patients with carbapenem-resistant and third-generation-cephalosporin-resistant Klebsiella pneumoniae bloodstream infections. *Antimicrobial agents and chemotherapy.* Oct 2014;58(10):5732-5735.
102. Mouloudi E, Massa E, Papadopoulos S, et al. Bloodstream infections caused by carbapenemase-producing klebsiella pneumoniae among intensive care unit patients after orthotopic liver transplantation: Risk factors for infection and impact of resistance on outcomes. *Transplantation Proceedings.* 2014;46(9):3216-3218.
103. Mouloudi E, Protonotariou E, Zagorianou A, et al. Bloodstream infections caused by metallo-beta-lactamase/Klebsiella pneumoniae carbapenemase-producing K. pneumoniae among intensive care unit patients in Greece: risk factors for infection and impact of type of resistance on outcomes. *Infection control and hospital epidemiology.* Dec 2010;31(12):1250-1256.
104. Simkins J, Muggia V, Cohen HW, Minamoto GY. Carbapenem-resistant Klebsiella pneumoniae infections in kidney transplant recipients: A case-control study. *Transplant Infectious Disease.* 2014;16(5):775-782.
105. Hussein K, Raz-Pasteur A, Finkelstein R, et al. Impact of carbapenem resistance on the outcome of patients' hospital-acquired bacteraemia caused by Klebsiella pneumoniae. *Journal of Hospital Infection.* 2013;83(4):307-313.
106. Ben-David D, Kordevani R, Keller N, et al. Outcome of carbapenem resistant Klebsiella pneumoniae bloodstream infections. *Clinical Microbiology and Infection.* 2012;18(1):54-60.
107. Daikos GL, Petrikkos P, Psichogiou M, et al. Prospective observational study of the impact of VIM-1 metallo-beta-lactamase on the outcome of patients with Klebsiella pneumoniae bloodstream infections. *Antimicrobial agents and chemotherapy.* May 2009;53(5):1868-1873.
108. Brizendine KD, Richter SS, Cober ED, Van Duin D. Carbapenem-resistant Klebsiella pneumoniae urinary tract infection following solid organ transplantation. *Antimicrobial agents and chemotherapy.* 2015;59(1):553-557.
109. Pouch SM, Kubin CJ, Satlin MJ, et al. Epidemiology and outcomes of carbapenem-resistant Klebsiella pneumoniae bacteriuria in kidney transplant recipients. *Transplant Infectious Disease.* 2015;17(6):800-809.
110. Shilo S, Assous MV, Lachish T, et al. Risk factors for bacteriuria with carbapenem-resistant Klebsiella pneumoniae and its impact on mortality: A case-control study. *Infection.* 2013;41(2):503-509.
111. Giannella M, Morelli MC, Cristini F, et al. Carbapenem-resistant Klebsiella pneumoniae colonization at liver transplantation: a management challenge. *Liver transplantation : official publication of the American Association for the Study of Liver Diseases and the International Liver Transplantation Society.* May 2014;20(5):631-633.
112. Lubbert C, Becker-Rux D, Rodloff AC, et al. Colonization of liver transplant recipients with KPC-producing Klebsiella pneumoniae is associated with high infection rates and excess mortality: a case-control analysis. *Infection.* Apr 2014;42(2):309-316.
113. Nouvenne A, Ticinesi A, Lauretani F, et al. Comorbidities and disease severity as risk factors for carbapenem-resistant Klebsiella pneumoniae colonization: report of an experience in an internal medicine unit. *PloS one.* 2014;9(10):e110001.
114. Bleumin D, Cohen MJ, Moranne O, et al. Carbapenem-resistant Klebsiella pneumoniae is associated with poor outcome in hemodialysis patients. *The Journal of infection.* Oct 2012;65(4):318-325.

115. Debby BD, Ganor O, Yasmin M, et al. Epidemiology of carbapenem resistant Klebsiella pneumoniae colonization in an intensive care unit. *European journal of clinical microbiology & infectious diseases : official publication of the European Society of Clinical Microbiology.* Aug 2012;31(8):1811-1817.
116. Schwaber MJ, Klarfeld-Lidji S, Navon-Venezia S, Schwartz D, Leavitt A, Carmeli Y. Predictors of carbapenem-resistant Klebsiella pneumoniae acquisition among hospitalized adults and effect of acquisition on mortality. *Antimicrobial agents and chemotherapy.* Mar 2008;52(3):1028-1033.
117. Pereira MR, Scully BF, Pouch SM, et al. Risk factors and outcomes of carbapenem-resistant Klebsiella pneumoniae infections in liver transplant recipients. *Liver transplantation : official publication of the American Association for the Study of Liver Diseases and the International Liver Transplantation Society.* Dec 2015;21(12):1511-1519.
118. Kofteridis DP, Valachis A, Dimopoulou D, et al. Risk factors for carbapenem-resistant Klebsiella pneumoniae infection/colonization: a case-case-control study. *Journal of infection and chemotherapy : official journal of the Japan Society of Chemotherapy.* May 2014;20(5):293-297.
119. Candevir Ulu A, Kurtaran B, Inal AS, et al. Risk factors of carbapenem-resistant Klebsiella pneumoniae infection: a serious threat in ICUs. *Medical science monitor : international medical journal of experimental and clinical research.* 2015;21:219-224.
120. Dizbay M, Guzel Tunccan O, Karasahin O, Aktas F. Emergence of carbapenem-resistant Klebsiella spp. infections in a Turkish university hospital: epidemiology and risk factors. *Journal of infection in developing countries.* Jan 2014;8(1):44-49.
121. Hoxha A, Karki T, Giambi C, et al. Attributable mortality of carbapenem-resistant Klebsiella pneumoniae infections in a prospective matched cohort study in Italy, 2012-2013. *The Journal of hospital infection.* Jan 2016;92(1):61-66.
122. Jiao Y, Qin Y, Liu J, et al. Risk factors for carbapenem-resistant Klebsiella pneumoniae infection/colonization and predictors of mortality: a retrospective study. *Pathogens and global health.* Mar 2015;109(2):68-74.
123. Patel G, Huprikar S, Factor SH, Jenkins SG, Calfee DP. Outcomes of carbapenem-resistant Klebsiella pneumoniae infection and the impact of antimicrobial and adjunctive therapies. *Infection control and hospital epidemiology.* Dec 2008;29(12):1099-1106.
124. Correa L, Martino MD, Siqueira I, et al. A hospital-based matched case-control study to identify clinical outcome and risk factors associated with carbapenem-resistant Klebsiella pneumoniae infection. *BMC infectious diseases.* 2013;13:80.
125. Centers for Disease Control and Prevention C. *Carbapenem-Resistant Klebsiella pneumoniae Associated with a Long-Term--Care Facility--West Virginia, 2009-2011* October 21 2011.
126. Falagas ME, Rafailidis PI, Kofteridis D, et al. Risk factors of carbapenem-resistant Klebsiella pneumoniae infections: a matched case control study. *The Journal of antimicrobial chemotherapy.* Nov 2007;60(5):1124-1130.
127. Hauck C, Cober E, Richter SS, et al. Spectrum of excess mortality due to carbapenem-resistant Klebsiella pneumoniae infections. *Clinical microbiology and infection : the official publication of the European Society of Clinical Microbiology and Infectious Diseases.* Jun 2016;22(6):513-519.
128. Atmaca O, Zarakolu P, Karahan C, Cakir B, Unal S. [Risk factors and antibiotic use in methicillin-resistant Staphylococcus aureus bacteremia in hospitalized patients at Hacettepe University Adult and Oncology Hospitals (2004-2011) and antimicrobial susceptibilities of the isolates: a nested case-control study]. *Mikrobiyoloji bulteni.* Oct 2014;48(4):523-537.

129. Daikos GL, Karabinis A, Paramythiotou E, et al. VIM-1-producing Klebsiella pneumoniae bloodstream infections: analysis of 28 cases. *Int J Antimicrob Agents.* Apr 2007;29(4):471-473.
130. Lee NY, Wu JJ, Lin SH, Ko WC, Tsai LH, Yan JJ. Characterization of carbapenem-nonsusceptible Klebsiella pneumoniae bloodstream isolates at a Taiwanese hospital: clinical impacts of lowered breakpoints for carbapenems. *European journal of clinical microbiology & infectious diseases : official publication of the European Society of Clinical Microbiology.* Aug 2012;31(8):1941-1950.
131. Marchaim D, Chopra T, Pogue JM, et al. Outbreak of colistin-resistant, carbapenem-resistant Klebsiella pneumoniae in metropolitan Detroit, Michigan. *Antimicrobial agents and chemotherapy.* Feb 2011;55(2):593-599.
132. Zarkotou O, Pournaras S, Voulgari E, et al. Risk factors and outcomes associated with acquisition of colistin-resistant KPC-producing Klebsiella pneumoniae: a matched case-control study. *J Clin Microbiol.* Jun 2010;48(6):2271-2274.
133. Capone A, Giannella M, Fortini D, et al. High rate of colistin resistance among patients with carbapenem-resistant Klebsiella pneumoniae infection accounts for an excess of mortality. *Clinical microbiology and infection : the official publication of the European Society of Clinical Microbiology and Infectious Diseases.* Jan 2013;19(1):E23-30.
134. Rojas LJ, Salim M, Cober E, et al. Colistin Resistance in Carbapenem-Resistant Klebsiella pneumoniae: Laboratory Detection and Impact on Mortality. *Clinical infectious diseases : an official publication of the Infectious Diseases Society of America.* Dec 10 2016.
135. Falagas ME, Rafailidis PI, Matthaiou DK, Virtzili S, Nikita D, Michalopoulos A. Pandrug-resistant Klebsiella pneumoniae, Pseudomonas aeruginosa and Acinetobacter baumannii infections: characteristics and outcome in a series of 28 patients. *Int J Antimicrob Agents.* Nov 2008;32(5):450-454.
136. Falagas ME, Bliziotis IA, Kasiakou SK, Samonis G, Athanassopoulou P, Michalopoulos A. Outcome of infections due to pandrug-resistant (PDR) Gram-negative bacteria. *BMC infectious diseases.* Apr 08 2005;5:24.
137. Antoniadou A, Kontopidou F, Poulakou G, et al. Colistin-resistant isolates of Klebsiella pneumoniae emerging in intensive care unit patients: first report of a multiclonal cluster. *The Journal of antimicrobial chemotherapy.* Apr 2007;59(4):786-790.
138. Toth A, Damjanova I, Puskas E, et al. Emergence of a colistin-resistant KPC-2-producing Klebsiella pneumoniae ST258 clone in Hungary. *European journal of clinical microbiology & infectious diseases : official publication of the European Society of Clinical Microbiology.* Jul 2010;29(7):765-769.
139. Kontopoulou K, Protonotariou E, Vasilakos K, et al. Hospital outbreak caused by Klebsiella pneumoniae producing KPC-2 beta-lactamase resistant to colistin. *The Journal of hospital infection.* Sep 2010;76(1):70-73.
140. Ah YM, Kim AJ, Lee JY. Colistin resistance in Klebsiella pneumoniae. *Int J Antimicrob Agents.* Jul 2014;44(1):8-15.
141. Gallagher JC, Kuriakose S, Haynes K, Axelrod P. Case-case-control study of patients with carbapenem-resistant and third-generation-cephalosporin-resistant Klebsiella pneumoniae bloodstream infections. *Antimicrob. Agents Chemother.* 2014;58(10):5732-5735.
142. Panhotra BR, Saxena AK, Al-Ghamdi AM. Extended-spectrum beta-lactamase-producing Klebsiella pneumoniae hospital acquired bacteremia. Risk factors and clinical outcome. *Saudi medical journal.* Dec 2004;25(12):1871-1876.
143. Tumbarello M, Spanu T, Sanguinetti M, et al. Bloodstream infections caused by extended-spectrum-beta-lactamase-producing Klebsiella pneumoniae: risk factors, molecular

epidemiology, and clinical outcome. *Antimicrobial agents and chemotherapy.* Feb 2006;50(2):498-504.
144. Marra AR, Wey SB, Castelo A, et al. Nosocomial bloodstream infections caused by Klebsiella pneumoniae: impact of extended-spectrum beta-lactamase (ESBL) production on clinical outcome in a hospital with high ESBL prevalence. *BMC infectious diseases.* 2006;6:24.
145. Kang CI, Kim SH, Park WB, et al. Clinical outcome of bacteremic spontaneous bacterial peritonitis due to extended-spectrum beta-lactamase-producing Escherichia coli and Klebsiella pneumoniae. *The Korean journal of internal medicine.* Sep 2004;19(3):160-164.
146. Kim BN, Woo JH, Kim MN, Ryu J, Kim YS. Clinical implications of extended-spectrum beta-lactamase-producing Klebsiella pneumoniae bacteraemia. *The Journal of hospital infection.* Oct 2002;52(2):99-106.
147. Ariffin H, Navaratnam P, Mohamed M, et al. Ceftazidime-resistant Klebsiella pneumoniae bloodstream infection in children with febrile neutropenia. *International journal of infectious diseases : IJID : official publication of the International Society for Infectious Diseases.* 2000;4(1):21-25.
148. Mosqueda-Gomez JL, Montano-Loza A, Rolon AL, et al. Molecular epidemiology and risk factors of bloodstream infections caused by extended-spectrum beta-lactamase-producing Klebsiella pneumoniae A case-control study. *International journal of infectious diseases : IJID : official publication of the International Society for Infectious Diseases.* Nov 2008;12(6):653-659.
149. Szilagyi E, Fuzi M, Borocz K, Kurcz A, Toth A, Nagy K. Risk factors and outcomes for bloodstream infections with extended-spectrum beta -lactamase-producing Klebsiella pneumoniae ; Findings of the nosocomial surveillance system in Hungary. *Acta microbiologica et immunologica Hungarica.* Sep 2009;56(3):251-262.
150. Pena C, Pujol M, Ardanuy C, et al. An outbreak of hospital-acquired Klebsiella pneumoniae bacteraemia, including strains producing extended-spectrum beta-lactamase. *The Journal of hospital infection.* Jan 2001;47(1):53-59.
151. Paterson DL, Ko WC, Von Gottberg A, et al. International prospective study of Klebsiella pneumoniae bacteremia: implications of extended-spectrum beta-lactamase production in nosocomial Infections. *Annals of internal medicine.* Jan 6 2004;140(1):26-32.
152. Tuon FF, Kruger M, Terreri M, Penteado-Filho SR, Gortz L. Klebsiella ESBL bacteremia-mortality and risk factors. *The Brazilian journal of infectious diseases : an official publication of the Brazilian Society of Infectious Diseases.* Nov-Dec 2011;15(6):594-598.
153. Lee JA, Kang CI, Joo EJ, et al. Epidemiology and clinical features of community-onset bacteremia caused by extended-spectrum beta-lactamase-producing Klebsiella pneumoniae. *Microbial drug resistance (Larchmont, N.Y.).* Jun 2011;17(2):267-273.
154. Brizendine KD, Richter SS, Cober ED, van Duin D. Carbapenem-resistant Klebsiella pneumoniae urinary tract infection following solid organ transplantation. *Antimicrobial agents and chemotherapy.* Jan 2015;59(1):553-557.
155. Loh LC, Nor Izran Hanim Bt Abdul S, Rosdara Masayuni Bt Mohd S, Raman S, Thayaparan T, Kumar S. Hospital Outcomes of Adult Respiratory Tract Infections with Extended-Spectrum B-Lactamase (ESBL) Producing Klebsiella Pneumoniae. *The Malaysian journal of medical sciences : MJMS.* Jul 2007;14(2):36-40.
156. Gomes CC, Vormittag E, Santos CR, Levin AS. Nosocomial infection with cephalosporin-resistant Klebsiella pneumoniae is not associated with increased mortality. *Infection control and hospital epidemiology.* Sep 2006;27(9):907-912.

157. Lin MF, Huang ML, Lai SH. Risk factors in the acquisition of extended-spectrum beta-lactamase Klebsiella pneumoniae: a case-control study in a district teaching hospital in Taiwan. *The Journal of hospital infection.* Jan 2003;53(1):39-45.
158. Kuo KC, Shen YH, Hwang KP. Clinical implications and risk factors of extended-spectrum beta-lactamase-producing Klebsiella pneumoniae infection in children: a case-control retrospective study in a medical center in southern Taiwan. *Journal of microbiology, immunology, and infection = Wei mian yu gan ran za zhi.* Jun 2007;40(3):248-254.
159. Chiu S, Huang YC, Lien RI, Chou YH, Lin TY. Clinical features of nosocomial infections by extended-spectrum beta-lactamase-producing Enterobacteriaceae in neonatal intensive care units. *Acta paediatrica.* Nov 2005;94(11):1644-1649.
160. Huang Y, Zhuang S, Du M. Risk factors of nosocomial infection with extended-spectrum beta-lactamase-producing bacteria in a neonatal intensive care unit in China. *Infection.* Oct 2007;35(5):339-345.
161. Stewardson AJ, Allignol A, Beyersmann J, et al. The health and economic burden of bloodstream infections caused by antimicrobial-susceptible and non-susceptible Enterobacteriaceae and Staphylococcus aureus in European hospitals, 2010 and 2011: A multicentre retrospective cohort study. *Eurosurveillance.* 2016;21(33).
162. WHO. *Antimicrobial resistance: global report on surveillance.* France: WHO;2014.
163. Kuint J, Barzilai A, Regev-Yochay G, Rubinstein E, Keller N, Maayan-Metzger A. Comparison of community-acquired methicillin-resistant Staphylococcus aureus bacteremia to other staphylococcal species in a neonatal intensive care unit. *Eur J Pediatr.* Apr 2007;166(4):319-325.
164. Guilarde AO, Turchi MD, Martelli CM, Primo MG. Staphylococcus aureus bacteraemia: incidence, risk factors and predictors for death in a Brazilian teaching hospital. *The Journal of hospital infection.* Jul 2006;63(3):330-336.
165. Castillo JS, Leal AL, Cortes JA, et al. Mortality among critically ill patients with methicillin-resistant Staphylococcus aureus bacteremia: a multicenter cohort study in Colombia. *Revista panamericana de salud publica = Pan American journal of public health.* Nov 2012;32(5):343-350.
166. Lawes T, Edwards B, Lopez-Lozano JM, Gould I. Trends in Staphylococcus aureus bacteraemia and impacts of infection control practices including universal MRSA admission screening in a hospital in Scotland, 2006-2010: retrospective cohort study and time-series intervention analysis. *BMJ Open.* 2012;2(3).
167. Blot SI, Vandewoude KH, Hoste EA, Colardyn FA. Outcome and attributable mortality in critically Ill patients with bacteremia involving methicillin-susceptible and methicillin-resistant Staphylococcus aureus. *Arch Intern Med.* Oct 28 2002;162(19):2229-2235.
168. Talon D, Woronoff-Lemsi MC, Limat S, et al. The impact of resistance to methicillin in Staphylococcus aureus bacteremia on mortality. *Eur J Intern Med.* Feb 2002;13(1):31-36.
169. Soriano A, Martinez JA, Mensa J, et al. Pathogenic significance of methicillin resistance for patients with Staphylococcus aureus bacteremia. *Clinical infectious diseases : an official publication of the Infectious Diseases Society of America.* Feb 2000;30(2):368-373.
170. Selvey LA, Whitby M, Johnson B. Nosocomial methicillin-resistant Staphylococcus aureus bacteremia: is it any worse than nosocomial methicillin-sensitive Staphylococcus aureus bacteremia? *Infection control and hospital epidemiology.* Oct 2000;21(10):645-648.
171. Rubio-Terres C, Garau J, Grau S, Martinez-Martinez L, Cast of Resistance Study g. Cost of bacteraemia caused by methicillin-resistant vs. methicillin-susceptible Staphylococcus aureus in Spain: a retrospective cohort study. *Clinical microbiology and infection : the official publication of the European Society of Clinical Microbiology and Infectious Diseases.* Jun 2010;16(6):722-728.

172. Romero-Vivas J, Rubio M, Fernandez C, Picazo JJ. Mortality associated with nosocomial bacteremia due to methicillin-resistant Staphylococcus aureus. *Clinical infectious diseases : an official publication of the Infectious Diseases Society of America.* Dec 1995;21(6):1417-1423.
173. Reshad K, Tanaka F, Sekine T, et al. [A prospective study of septic episodes due to Staphylococcus aureus and the background of the patients]. *Kansenshogaku Zasshi.* Feb 1994;68(2):171-176.
174. Quilty S, Kwok G, Hajkowicz K, Currie B. High incidence of methicillin-resistant Staphylococcus aureus sepsis and death in patients with febrile neutropenia at Royal Darwin Hospital. *Intern Med J.* Aug 2009;39(8):557-559.
175. Hakim H, Mylotte JM, Faden H. Morbidity and mortality of Staphylococcal bacteremia in children. *American journal of infection control.* Mar 2007;35(2):102-105.
176. Nickerson EK, Wuthiekanun V, Day NP, Chaowagul W, Peacock SJ. Meticillin-resistant Staphylococcus aureus in rural Asia. *The Lancet. Infectious diseases.* Feb 2006;6(2):70-71.
177. Kim SH, Park WB, Lee KD, et al. Outcome of Staphylococcus aureus bacteremia in patients with eradicable foci versus noneradicable foci. *Clinical infectious diseases : an official publication of the Infectious Diseases Society of America.* Sep 15 2003;37(6):794-799.
178. Melzer M, Eykyn SJ, Gransden WR, Chinn S. Is methicillin-resistant Staphylococcus aureus more virulent than methicillin-susceptible S. aureus? A comparative cohort study of British patients with nosocomial infection and bacteremia. *Clinical infectious diseases : an official publication of the Infectious Diseases Society of America.* Dec 01 2003;37(11):1453-1460.
179. Khatib R, Johnson LB, Fakih MG, et al. Persistence in Staphylococcus aureus bacteremia: incidence, characteristics of patients and outcome. *Scandinavian journal of infectious diseases.* 2006;38(1):7-14.
180. Lodise TP, McKinnon PS. Clinical and economic impact of methicillin resistance in patients with Staphylococcus aureus bacteremia. *Diagn Microbiol Infect Dis.* Jun 2005;52(2):113-122.
181. Nickerson EK, Hongsuwan M, Limmathurotsakul D, et al. Staphylococcus aureus bacteraemia in a tropical setting: patient outcome and impact of antibiotic resistance. *PloS one.* 2009;4(1):e4308.
182. Harbarth S, Rutschmann O, Sudre P, Pittet D. Impact of methicillin resistance on the outcome of patients with bacteremia caused by Staphylococcus aureus. *Arch Intern Med.* Jan 26 1998;158(2):182-189.
183. Cunney RJ, McNamara EB, alAnsari N, Smyth EG. Community and hospital acquired Staphylococcus aureus septicaemia: 115 cases from a Dublin teaching hospital. *The Journal of infection.* Jul 1996;33(1):11-13.
184. Das I, O'Connell N, Lambert P. Epidemiology, clinical and laboratory characteristics of Staphylococcus aureus bacteraemia in a university hospital in UK. *The Journal of hospital infection.* Feb 2007;65(2):117-123.
185. Austin TW, Austin MA, Coleman B. Methicillin-resistant/methicillin-sensitive Staphylococcus aureus bacteremia. *Saudi medical journal.* Mar 2003;24(3):256-260.
186. Conterno LO, Wey SB, Castelo A. Risk factors for mortality in Staphylococcus aureus bacteremia. *Infection control and hospital epidemiology.* Jan 1998;19(1):32-37.
187. Hill EE, Peetermans WE, Vanderschueren S, Claus P, Herregods MC, Herijgers P. Methicillin-resistant versus methicillin-sensitive Staphylococcus aureus infective endocarditis. *European journal of clinical microbiology & infectious diseases : official publication of the European Society of Clinical Microbiology.* Jun 2008;27(6):445-450.
188. Ganga R, Riederer K, Sharma M, et al. Role of SCCmec type in outcome of Staphylococcus aureus bacteremia in a single medical center. *J Clin Microbiol.* Mar 2009;47(3):590-595.

189. Burke RE, Halpern MS, Baron EJ, Gutierrez K. Pediatric and neonatal Staphylococcus aureus bacteremia: epidemiology, risk factors, and outcome. *Infection control and hospital epidemiology.* Jul 2009;30(7):636-644.
190. de Oliveira Conterno L, Wey SB, Castelo A. Staphylococcus aureus bacteremia: comparison of two periods and a predictive model of mortality. *The Brazilian journal of infectious diseases : an official publication of the Brazilian Society of Infectious Diseases.* Dec 2002;6(6):288-297.
191. Lesens O, Methlin C, Hansmann Y, et al. Role of comorbidity in mortality related to Staphylococcus aureus bacteremia: a prospective study using the Charlson weighted index of comorbidity. *Infection control and hospital epidemiology.* Dec 2003;24(12):890-896.
192. O'Kane GM, Gottlieb T, Bradbury R. Staphylococcal bacteraemia: the hospital or the home? A review of Staphylococcus aureus bacteraemia at Concord Hospital in 1993. *Aust N Z J Med.* Feb 1998;28(1):23-27.
193. de Kraker ME, Wolkewitz M, Davey PG, et al. Clinical impact of antimicrobial resistance in European hospitals: excess mortality and length of hospital stay related to methicillin-resistant Staphylococcus aureus bloodstream infections. *Antimicrobial agents and chemotherapy.* Apr 2011;55(4):1598-1605.
194. Salgado CD, Dash S, Cantey JR, Marculescu CE. Higher risk of failure of methicillin-resistant Staphylococcus aureus prosthetic joint infections. *Clin Orthop Relat Res.* Aug 2007;461:48-53.
195. Al-Nammari SS, Bobak P, Venkatesh R. Methicillin resistant Staphylococcus aureus versus methicillin sensitive Staphylococcus aureus adult haematogenous septic arthritis. *Arch Orthop Trauma Surg.* Sep 2007;127(7):537-542.
196. Rello J, Molano D, Villabon M, et al. Differences in hospital- and ventilator-associated pneumonia due to Staphylococcus aureus (methicillin-susceptible and methicillin-resistant) between Europe and Latin America: a comparison of the EUVAP and LATINVAP study cohorts. *Med Intensiva.* May 2013;37(4):241-247.
197. Spindel SJ, Strausbaugh LJ, Jacobson C. Infections caused by Staphylococcus aureus in a Veterans' Affairs nursing home care unit: a 5-year experience. *Infection control and hospital epidemiology.* Apr 1995;16(4):217-223.
198. Davis SL, Perri MB, Donabedian SM, et al. Epidemiology and outcomes of community-associated methicillin-resistant Staphylococcus aureus infection. *J Clin Microbiol.* Jun 2007;45(6):1705-1711.
199. Graffunder EM, Venezia RA. Risk factors associated with nosocomial methicillin-resistant Staphylococcus aureus (MRSA) infection including previous use of antimicrobials. *The Journal of antimicrobial chemotherapy.* Jun 2002;49(6):999-1005.
200. Priest DH, Peacock JE, Jr. Hematogenous vertebral osteomyelitis due to Staphylococcus aureus in the adult: clinical features and therapeutic outcomes. *South Med J.* Sep 2005;98(9):854-862.
201. Hershow RC, Khayr WF, Smith NL. A comparison of clinical virulence of nosocomially acquired methicillin-resistant and methicillin-sensitive Staphylococcus aureus infections in a university hospital. *Infection control and hospital epidemiology.* Oct 1992;13(10):587-593.
202. Hulten KG, Kaplan SL, Lamberth LB, et al. Hospital-acquired Staphylococcus aureus infections at Texas Children's Hospital, 2001-2007. *Infection control and hospital epidemiology.* Feb 2010;31(2):183-190.
203. Joo EJ, Chung DR, Ha YE, et al. Clinical predictors of community-genotype ST72-methicillin-resistant Staphylococcus aureus-SCCmec type IV in patients with community-onset S. aureus infection. *The Journal of antimicrobial chemotherapy.* Jul 2012;67(7):1755-1759.

204. Capitano B, Leshem OA, Nightingale CH, Nicolau DP. Cost effect of managing methicillin-resistant Staphylococcus aureus in a long-term care facility. *J Am Geriatr Soc.* Jan 2003;51(1):10-16.
205. Clancy MJ, Graepler A, Breese PE, Price CS, Burman WJ. Widespread emergence of methicillin resistance in community-acquired Staphylococcus aureus infections in Denver. *South Med J.* Nov 2005;98(11):1069-1075.
206. Rello J, Torres A, Ricart M, et al. Ventilator-associated pneumonia by Staphylococcus aureus. Comparison of methicillin-resistant and methicillin-sensitive episodes. *Am J Respir Crit Care Med.* Dec 1994;150(6 Pt 1):1545-1549.
207. Gonzalez C, Rubio M, Romero-Vivas J, Gonzalez M, Picazo JJ. Bacteremic pneumonia due to Staphylococcus aureus: A comparison of disease caused by methicillin-resistant and methicillin-susceptible organisms. *Clinical infectious diseases : an official publication of the Infectious Diseases Society of America.* Nov 1999;29(5):1171-1177.
208. Pujol M, Pena C, Pallares R, et al. Nosocomial Staphylococcus aureus bacteremia among nasal carriers of methicillin-resistant and methicillin-susceptible strains. *Am J Med.* May 1996;100(5):509-516.
209. McMaster J, Booth MG, Smith A, Hamilton K. Meticillin-resistant Staphylococcus aureus in the intensive care unit: its effect on outcome and risk factors for acquisition. *The Journal of hospital infection.* Aug 2015;90(4):327-332.
210. Yasmin M, El Hage H, Obeid R, El Haddad H, Zaarour M, Khalil A. Epidemiology of bloodstream infections caused by methicillin-resistant Staphylococcus aureus at a tertiary care hospital in New York. *American journal of infection control.* 2016;44(1):41-46.
211. De la Calle C, Morata L, Cobos-Trigueros N, et al. Staphylococcus aureus bacteremic pneumonia. *European Journal of Clinical Microbiology and Infectious Diseases.* 2016;35(3):497-502.
212. De Rosa FG, Corcione S, Motta I, et al. Risk factors for mortality in patients with Staphylococcus aureus bloodstream infection. *Journal of Chemotherapy.* 2016;28(3):187-190.
213. Deodhar D, Varghese G, Balaji V, et al. Prevalence of toxin genes among the clinical isolates of Staphylococcus aureus and its clinical impact. *Journal of Global Infectious Diseases.* 2015;7(3):97-102.
214. Dolapo O, Dhanireddy R, Talati AJ. Trends of Staphylococcus aureus bloodstream infections in a neonatal intensive care unit from 2000-2009. *BMC pediatrics.* May 09 2014;14:121.
215. Fortuin-de Smidt MC, Singh-Moodley A, Badat R, et al. Staphylococcus aureus bacteraemia in Gauteng academic hospitals, South Africa. *International journal of infectious diseases : IJID : official publication of the International Society for Infectious Diseases.* Jan 2015;30:41-48.
216. Kim DH, Tate J, Dresen WF, et al. Cardiac implanted electronic device-related infective endocarditis: clinical features, management, and outcomes of 80 consecutive patients. *Pacing and clinical electrophysiology : PACE.* Aug 2014;37(8):978-985.
217. Kobayashi D, Yokota K, Takahashi O, Arioka H, Fukui T. A predictive rule for mortality of inpatients with Staphylococcus aureus bacteraemia: A classification and regression tree analysis. *European journal of internal medicine.* Dec 2014;25(10):914-918.
218. Lee JY, Chong YP, Kim T, et al. Bone and joint infection as a predictor of community-acquired methicillin-resistant Staphylococcus aureus bacteraemia: a comparative cohort study. *The Journal of antimicrobial chemotherapy.* Jul 2014;69(7):1966-1971.
219. Manandhar S, Pai G, Gidwani H, et al. Does staphylococcus aureus bacteriuria predict clinical outcomes in patients with bacteremia?: Analysis of 274 patients with

staphylococcus aureus blood stream infection. *Infectious Diseases in Clinical Practice.* 2016;24(3):151-154.
220. McMullan BJ, Bowen A, Blyth CC, et al. Epidemiology and mortality of staphylococcus aureus Bacteremia in Australian and New Zealand children. *JAMA pediatrics.* 2016;170(10):979-986.
221. Nagao M, Yamamoto M, Matsumura Y, et al. Complete adherence to evidence-based quality-of-care indicators for Staphylococcus aureus bacteremia resulted in better prognosis. *Infection.* 2016:1-9.
222. Naidoo R, Nuttall J, Whitelaw A, Eley B. Epidemiology of Staphylococcus aureus bacteraemia at a tertiary children's hospital in Cape Town, South Africa. *PloS one.* 2013;8(10):e78396.
223. Ong CW, Roberts JL, Collignon PJ. Long-term survival outcome following staphylococcus aureus bacteraemia. *Healthcare Infection.* 2013;18(3):102-109.
224. Park DA, Lee SM, Peck KR, Joo EJ, Oh EG. Impact of methicillin-resistance on mortality in children and neonates with staphylococcus aureus bacteremia: A meta-analysis. *Infection and Chemotherapy.* 2013;45(2):202-210.
225. Simsek Yavuz S, Sensoy A, Ceken S, Deniz D, Yekeler I. Methicillin-resistant Staphylococcus aureus infection: an independent risk factor for mortality in patients with poststernotomy mediastinitis. *Medical principles and practice : international journal of the Kuwait University, Health Science Centre.* 2014;23(6):517-523.
226. Thaden JT, Ericson JE, Cross H, et al. Survival benefit of empirical therapy for staphylococcus aureus bloodstream infections in infants. *Pediatric Infectious Disease Journal.* 2015;34(11):1175-1179.
227. Theodorou P, Lefering R, Perbix W, et al. Staphylococcus aureus bacteremia after thermal injury: the clinical impact of methicillin resistance. *Burns : journal of the International Society for Burn Injuries.* May 2013;39(3):404-412.
228. Wang JT, Hsu LY, Lauderdale TL, Fan WC, Wang FD. Comparison of Outcomes among Adult Patients with Nosocomial Bacteremia Caused by Methicillin-Susceptible and Methicillin-Resistant Staphylococcus aureus: A Retrospective Cohort Study. *PloS one.* 2015;10(12).
229. Yaw LK, Robinson JO, Ho KM. A comparison of long-term outcomes after meticillin-resistant and meticillin-sensitive Staphylococcus aureus bacteraemia: an observational cohort study. *The Lancet Infectious Diseases.* 2014.
230. Yilmaz M, Elaldi N, Balkan, et al. Mortality predictors of Staphylococcus aureus bacteremia: A prospective multicenter study. *Annals of Clinical Microbiology and Antimicrobials.* 2016;15(1).
231. Shoji H, Urakawa T, Watanabe K, et al. Clinical features, outcomes, and survival factor in patients with vertebral osteomyelitis infected by methicillin-resistant staphylococci. *Journal of Orthopaedic Science.* 2016;21(3):282-286.
232. Alizadeh N, Oskuee ABE, Golchai J, et al. Methicillin-resistant Staphylococcus aureus (MRSA) an important microorganism: Determination of its prevalence and evaluation of its associated factors in hospitalized dermatologic patients. *Iranian Journal of Dermatology.* 2014;17(68):54-58.
233. Balm MN, Lover AA, Salmon S, Tambyah PA, Fisher DA. Progression from new methicillin-resistant Staphylococcus aureus colonisation to infection: an observational study in a hospital cohort. *BMC infectious diseases.* Oct 22 2013;13:491.
234. Nelson RE, Stevens VW, Jones M, Samore MH, Rubin MA. Health care-associated methicillin-resistant Staphylococcus aureus infections increases the risk of postdischarge mortality. *American journal of infection control.* Jan 2015;43(1):38-43.

235. Tran K, Bell C, Stall N, et al. The Effect of Hospital Isolation Precautions on Patient Outcomes and Cost of Care: A Multi-Site, Retrospective, Propensity Score-Matched Cohort Study. *Journal of General Internal Medicine.* 2016:1-7.
236. Ericson JE, Popoola VO, Smith PB, et al. Burden of Invasive Staphylococcus aureus Infections in Hospitalized Infants. *JAMA pediatrics.* Dec 2015;169(12):1105-1111.
237. Gimenes M, Salci TP, Tognim MCB, Siqueira VLD, Caparroz-Assef SM. Treating Staphylococcus aureus infections in an intensive care unit at a University Hospital in Brazil. *International Journal of Clinical Pharmacy.* 2016;38(2):228-232.
238. Kim ES, Kim HB, Kim G, et al. Clinical and epidemiological factors associated with methicillin resistance in community-onset invasive Staphylococcus aureus infections: prospective multicenter cross-sectional study in Korea. *PloS one.* 2014;9(12):e114127.
239. Altinbas A, Shorbagi A, Ascioglu S, Zarakolu P, Cetinkaya-Sardan Y. Risk factors for intensive care unit acquired nasal colonization of MRSA and its impact on MRSA infection. *Journal of clinical laboratory analysis.* Sep 2013;27(5):412-417.
240. Chan TC, Cheng VC, Hung IF, Chan FH, Ng WC, Yuen KY. The association between methicillin resistant staphylococcus aureus colonization and mortality in Chinese nursing home older adults: a 2-year prospective cohort. *Journal of the American Medical Directors Association.* Sep 01 2015;16(9):796-797.
241. Chen CC, Pass SE. Risk factors for and impact of methicillin-resistant Staphylococcus aureus nasal colonization in patients in a medical intensive care unit. *American journal of infection control.* Nov 2013;41(11):1100-1101.
242. Moore C, Davis NF, Burke JP, et al. Colonisation with methicillin-resistant Staphylococcus aureus prior to renal transplantation is associated with long-term renal allograft failure. *Transplant international : official journal of the European Society for Organ Transplantation.* Sep 2014;27(9):926-930.
243. Minejima E, Lou M, Nieberg P, Wong-Beringer A. Patients presenting to the hospital with MRSA pneumonia: differentiating characteristics and outcomes with empiric treatment. *BMC infectious diseases.* May 10 2014;14:252.
244. Hill DM, Schroeppel TJ, Magnotti LJ, et al. Methicillin-resistant Staphylococcus aureus in early ventilator-associated pneumonia: cause for concern? *Surgical infections.* Dec 2013;14(6):520-524.
245. Tosh PK, Bulens SN, Nadle J, et al. Characterization of hospitalized community-onset staphylococcus aureus lower respiratory tract infections among generally healthy persons 50 years of age or younger. *Infectious Diseases in Clinical Practice.* 2013;21(6):359-365.
246. Jung WJ, Kang YA, Park MS, et al. Prediction of methicillin-resistant Staphylococcus aureus in patients with non-nosocomial pneumonia. *BMC infectious diseases.* Aug 09 2013;13:370.
247. Self WH, Wunderink RG, Williams DJ, et al. Staphylococcus aureus Community-acquired Pneumonia: Prevalence, Clinical Characteristics, and Outcomes. *Clinical Infectious Diseases.* 2016;63(3):300-309.
248. Lin WT, Wu CD, Cheng SC, et al. High Prevalence of Methicillin-Resistant Staphylococcus aureus among Patients with Septic Arthritis Caused by Staphylococcus aureus. *PloS one.* 2015;10(5):e0127150.
249. Mínguez S, Molinos S, Mateo L, et al. Septic arthritis due to methylcyllin-resistant Staphylococcus aureus in adults. *Reumatologia Clinica.* 2015;11(6):381-386.
250. de Kraker ME, Davey PG, Grundmann H, group Bs. Mortality and hospital stay associated with resistant Staphylococcus aureus and Escherichia coli bacteremia: estimating the burden of antibiotic resistance in Europe. *PLoS Med.* Oct 2011;8(10):e1001104.

251. Bender R, Blettner M. Calculating the "number needed to be exposed" with adjustment for confounding variables in epidemiological studies. *J Clin Epidemiol.* May 2002;55(5):525-530.
252. Liu Q, Li X, Li W, et al. Influence of carbapenem resistance on mortality of patients with Pseudomonas aeruginosa infection: a meta-analysis. *Scientific reports.* Jun 25 2015;5:11715.
253. Dantas RC, Ferreira ML, Gontijo-Filho PP, Ribas RM. Pseudomonas aeruginosa bacteraemia: independent risk factors for mortality and impact of resistance on outcome. *Journal of medical microbiology.* Dec 2014;63(Pt 12):1679-1687.
254. Hattemer A, Hauser A, Diaz M, et al. Bacterial and clinical characteristics of health care- and community-acquired bloodstream infections due to Pseudomonas aeruginosa. *Antimicrobial agents and chemotherapy.* Aug 2013;57(8):3969-3975.
255. Joo EJ, Kang CI, Ha YE, et al. Risk factors for mortality in patients with Pseudomonas aeruginosa bacteremia: clinical impact of antimicrobial resistance on outcome. *Microbial drug resistance (Larchmont, N.Y.).* Jun 2011;17(2):305-312.
256. Kang CI, Kim SH, Park WB, et al. Risk factors for antimicrobial resistance and influence of resistance on mortality in patients with bloodstream infection caused by Pseudomonas aeruginosa. *Microbial drug resistance (Larchmont, N.Y.).* Spring 2005;11(1):68-74.
257. Kim YJ, Jun YH, Kim YR, et al. Risk factors for mortality in patients with Pseudomonas aeruginosa bacteremia; retrospective study of impact of combination antimicrobial therapy. *BMC infectious diseases.* Mar 24 2014;14:161.
258. Pena C, Suarez C, Gozalo M, et al. Prospective multicenter study of the impact of carbapenem resistance on mortality in Pseudomonas aeruginosa bloodstream infections. *Antimicrobial agents and chemotherapy.* Mar 2012;56(3):1265-1272.
259. Suarez C, Pena C, Gavalda L, et al. Influence of carbapenem resistance on mortality and the dynamics of mortality in Pseudomonas aeruginosa bloodstream infection. *International journal of infectious diseases : IJID : official publication of the International Society for Infectious Diseases.* Sep 2010;14 Suppl 3:e73-78.
260. Tuon FF, Gortz LW, Rocha JL. Risk factors for pan-resistant Pseudomonas aeruginosa bacteremia and the adequacy of antibiotic therapy. *The Brazilian journal of infectious diseases : an official publication of the Brazilian Society of Infectious Diseases.* Jul-Aug 2012;16(4):351-356.
261. Luyt CE, Aubry A, Lu Q, et al. Imipenem, meropenem or doripenem to treat patients with pseudomonas aeruginosa ventilator-associated pneumonia. *American Journal of Respiratory and Critical Care Medicine.* 2014;189.
262. Eagye KJ, Kuti JL, Nicolau DP. Risk factors and outcomes associated with isolation of meropenem high-level-resistant Pseudomonas aeruginosa. *Infection control and hospital epidemiology.* Aug 2009;30(8):746-752.
263. Judd WR, Ratliff PD, Hickson RP, Stephens DM, Kennedy CA. Clinical and economic impact of meropenem resistance in Pseudomonas aeruginosa–infected patients. *American journal of infection control.* 2016;44(11):1275-1279.
264. Lautenbach E, Weiner MG, Nachamkin I, Bilker WB, Sheridan A, Fishman NO. Imipenem resistance among pseudomonas aeruginosa isolates: risk factors for infection and impact of resistance on clinical and economic outcomes. *Infection control and hospital epidemiology.* Sep 2006;27(9):893-900.
265. Lautenbach E, Synnestvedt M, Weiner MG, et al. Imipenem resistance in Pseudomonas aeruginosa: emergence, epidemiology, and impact on clinical and economic outcomes. *Infection control and hospital epidemiology.* Jan 2010;31(1):47-53.

266. Lin KY, Lauderdale TL, Wang JT, Chang SC. Carbapenem-resistant Pseudomonas aeruginosa in Taiwan: Prevalence, risk factors, and impact on outcome of infections. *Journal of microbiology, immunology, and infection = Wei mian yu gan ran za zhi.* Feb 2016;49(1):52-59.
267. Meradji S, Barguigua A, Zerouali K, et al. Epidemiology of carbapenem non-susceptible Pseudomonas aeruginosa isolates in Eastern Algeria. *Antimicrobial Resistance and Infection Control.* 2015;4(1).
268. The cost of antibiotic resistance: effect of resistance among Staphylococcus aureus, Klebsiella pneumoniae, Acinetobacter baumannii, and Pseudmonas aeruginosa on length of hospital stay. *Infection control and hospital epidemiology.* Feb 2002;23(2):106-108.
269. Matthaiou DK, Michalopoulos A, Rafailidis PI, et al. Risk factors associated with the isolation of colistin-resistant Gram-negative bacteria: A matched case-control study. *Critical Care Medicine.* 2008;36(3):807-811.
270. Yilmaz GR, Dizbay M, Guven T, et al. Risk factors for infection with colistin-resistant gram-negative microorganisms: A multicenter study. *Annals of Saudi Medicine.* 2016;36(3):216-222.
271. Caselli D, Cesaro S, Ziino O, et al. Multidrug resistant Pseudomonas aeruginosa infection in children undergoing chemotherapy and hematopoietic stem cell transplantation. *Haematologica.* Sep 2010;95(9):1612-1615.
272. Johnson LE, D'Agata EM, Paterson DL, et al. Pseudomonas aeruginosa bacteremia over a 10-year period: multidrug resistance and outcomes in transplant recipients. *Transplant infectious disease : an official journal of the Transplantation Society.* Jun 2009;11(3):227-234.
273. Morata L, Cobos-Trigueros N, Martinez JA, et al. Influence of multidrug resistance and appropriate empirical therapy on the 30-day mortality rate of Pseudomonas aeruginosa bacteremia. *Antimicrobial agents and chemotherapy.* Sep 2012;56(9):4833-4837.
274. Tam VH, Rogers CA, Chang KT, Weston JS, Caeiro JP, Garey KW. Impact of multidrug-resistant Pseudomonas aeruginosa bacteremia on patient outcomes. *Antimicrobial agents and chemotherapy.* Sep 2010;54(9):3717-3722.
275. Theodorou P, Thamm OC, Perbix W, Phan VT. Pseudomonas aeruginosa bacteremia after burn injury: the impact of multiple-drug resistance. *Journal of burn care & research : official publication of the American Burn Association.* Nov-Dec 2013;34(6):649-658.
276. Trecarichi EM, Tumbarello M, Caira M, et al. Multidrug resistant Pseudomonas aeruginosa bloodstream infection in adult patients with hematologic malignancies. *Haematologica.* Jan 2011;96(1):e1-3; author reply e4.
277. Tumbarello M, Repetto E, Trecarichi EM, et al. Multidrug-resistant Pseudomonas aeruginosa bloodstream infections: risk factors and mortality. *Epidemiology and infection.* Nov 2011;139(11):1740-1749.
278. Cillóniz C, Gabarrús A, Ferrer M, et al. Community-Acquired Pneumonia Due to Multidrug- and Non–Multidrug-Resistant Pseudomonas aeruginosa. *Chest.* 2016;150(2):415-425.
279. Montero M, Dominguez M, Orozco-Levi M, Salvado M, Knobel H. Mortality of COPD patients infected with multi-resistant Pseudomonas aeruginosa: a case and control study. *Infection.* Feb 2009;37(1):16-19.
280. Micek ST, Wunderink RG, Kollef MH, et al. An international multicenter retrospective study of Pseudomonas aeruginosa nosocomial pneumonia: impact of multidrug resistance. *Critical care (London, England).* May 06 2015;19:219.
281. Pena C, Gomez-Zorrilla S, Oriol I, et al. Impact of multidrug resistance on Pseudomonas aeruginosa ventilator-associated pneumonia outcome: predictors of early and crude

mortality. *European journal of clinical microbiology & infectious diseases : official publication of the European Society of Clinical Microbiology.* Mar 2013;32(3):413-420.
282. Tumbarello M, De Pascale G, Trecarichi EM, et al. Clinical outcomes of Pseudomonas aeruginosa pneumonia in intensive care unit patients. *Intensive care medicine.* Apr 2013;39(4):682-692.
283. Yang K, Zhuo H, Guglielmo BJ, Wiener-Kronish J. Multidrug-resistant Pseudomonas aeruginosa ventilator-associated pneumonia: the role of endotracheal aspirate surveillance cultures. *The Annals of pharmacotherapy.* Jan 2009;43(1):28-35.
284. Yayan J, Ghebremedhin B, Rasche K. Antibiotic resistance of pseudomonas aeruginosa in pneumonia at a single university hospital center in Germany over a 10-Year Period. *PloS one.* 2015;10(10).
285. Aloush V, Navon-Venezia S, Seigman-Igra Y, Cabili S, Carmeli Y. Multidrug-resistant Pseudomonas aeruginosa: risk factors and clinical impact. *Antimicrobial agents and chemotherapy.* Jan 2006;50(1):43-48.
286. Paramythiotou E, Lucet JC, Timsit JF, et al. Acquisition of multidrug-resistant Pseudomonas aeruginosa in patients in intensive care units: role of antibiotics with antipseudomonal activity. *Clinical infectious diseases : an official publication of the Infectious Diseases Society of America.* Mar 1 2004;38(5):670-677.
287. Cao B, Wang H, Sun H, Zhu Y, Chen M. Risk factors and clinical outcomes of nosocomial multi-drug resistant Pseudomonas aeruginosa infections. *The Journal of hospital infection.* Jun 2004;57(2):112-118.
288. de Matos ECO, De Matos HJ, Conceição ML, Rodrigues YC, Carneiro ICRS, Lima KVB. Clinical and microbiological features of infections caused by pseudomonas aeruginosa in patients hospitalized in intensive care units. *Revista da Sociedade Brasileira de Medicina Tropical.* 2016;49(3):305-311.
289. Morales E, Cots F, Sala M, et al. Hospital costs of nosocomial multi-drug resistant Pseudomonas aeruginosa acquisition. *BMC health services research.* May 23 2012;12:122.
290. Nathwani D, Raman G, Sulham K, Gavaghan M, Menon V. Clinical and economic consequences of hospital-acquired resistant and multidrug-resistant Pseudomonas aeruginosa infections: A systematic review and meta-analysis. *Antimicrobial Resistance and Infection Control.* 2014;3(1).
291. Ortega B, Groeneveld AB, Schultsz C. Endemic multidrug-resistant Pseudomonas aeruginosa in critically ill patients. *Infection control and hospital epidemiology.* Oct 2004;25(10):825-831.
292. Peng Y, Bi J, Shi J, et al. Multidrug-resistant Pseudomonas aeruginosa infections pose growing threat to health careeassociated infection control in the hospitals of Southern China: A case-control surveillance study. *American journal of infection control.* 2014;42(12):1308-1311.
293. Su H, Ye Q, Wan Q, Zhou J. Predictors of Mortality in Abdominal Organ Transplant Recipients with Pseudomonas aeruginosa Infections. *Annals of transplantation.* Feb 09 2016;21:86-93.
294. Tleyjeh IM, Tlaygeh HM, Hejal R, Montori VM, Baddour LM. The impact of penicillin resistance on short-term mortality in hospitalized adults with pneumococcal pneumonia: a systematic review and meta-analysis. *Clinical infectious diseases : an official publication of the Infectious Diseases Society of America.* Mar 15 2006;42(6):788-797.
295. Yu VL, Chiou CC, Feldman C, et al. An international prospective study of pneumococcal bacteremia: correlation with in vitro resistance, antibiotics administered, and clinical outcome. *Clinical infectious diseases : an official publication of the Infectious Diseases Society of America.* Jul 15 2003;37(2):230-237.

296. Watanabe H, Sato S, Kawakami K, et al. A comparative clinical study of pneumonia by penicillin-resistant and -sensitive Streptococcus pneumoniae in a community hospital. *Respirology.* Mar 2000;5(1):59-64.
297. Gouveia EL, Reis JN, Flannery B, et al. Clinical outcome of pneumococcal meningitis during the emergence of pencillin-resistant Streptococcus pneumoniae: an observational study. *BMC infectious diseases.* 2011;11:323.
298. Reechaipichitkul W, Assawasanti K, Chaimanee P. Risk factors and clinical outcomes of penicillin resistant S. pneumoniae community-acquired pneumonia in Khon Kaen, Thailand. *The Southeast Asian journal of tropical medicine and public health.* Mar 2006;37(2):320-326.
299. Sun HK, Nicolau DP, Kuti JL. Resource utilization of adults admitted to a large urban hospital with community-acquired pneumonia caused by Streptococcus pneumoniae. *Chest.* Sep 2006;130(3):807-814.
300. Song JH, Jung SI, Ki HK, et al. Clinical outcomes of pneumococcal pneumonia caused by antibiotic-resistant strains in asian countries: a study by the Asian Network for Surveillance of Resistant Pathogens. *Clinical infectious diseases : an official publication of the Infectious Diseases Society of America.* Jun 1 2004;38(11):1570-1578.
301. Einarsson S, Kristjansson M, Kristinsson KG, Kjartansson G, Jonsson S. Pneumonia caused by penicillin-non-susceptible and penicillin-susceptible pneumococci in adults: a case-control study. *Scandinavian journal of infectious diseases.* 1998;30(3):253-256.
302. Sangthawan P, Chantaratchada S, Chanthadisai N, Wattanathum A. Prevalence and clinical significance of community-acquired penicillin-resistant pneumococcal pneumonia in Thailand. *Respirology.* Jun 2003;8(2):208-212.
303. Falco V, Almirante B, Jordano Q, et al. Influence of penicillin resistance on outcome in adult patients with invasive pneumococcal pneumonia: is penicillin useful against intermediately resistant strains? *The Journal of antimicrobial chemotherapy.* Aug 2004;54(2):481-488.
304. Pallares R, Linares J, Vadillo M, et al. Resistance to penicillin and cephalosporin and mortality from severe pneumococcal pneumonia in Barcelona, Spain. *N Engl J Med.* Aug 24 1995;333(8):474-480.
305. Ewig S, Ruiz M, Torres A, et al. Pneumonia acquired in the community through drug-resistant Streptococcus pneumoniae. *Am J Respir Crit Care Med.* Jun 1999;159(6):1835-1842.
306. Aspa J, Rajas O, Rodriguez de Castro F, et al. Drug-resistant pneumococcal pneumonia: clinical relevance and related factors. *Clinical infectious diseases : an official publication of the Infectious Diseases Society of America.* Mar 15 2004;38(6):787-798.
307. Feikin DR, Schuchat A, Kolczak M, et al. Mortality from invasive pneumococcal pneumonia in the era of antibiotic resistance, 1995-1997. *Am J Public Health.* Feb 2000;90(2):223-229.
308. Yigla M, Finkelstein R, Hashman N, Green P, Cohn L, Merzbach D. Epidemiology and clinical spectrum of pneumococcal infections: an Israeli viewpoint. *The Journal of hospital infection.* Jan 1995;29(1):57-64.
309. Pallares R, Capdevila O, Linares J, et al. The effect of cephalosporin resistance on mortality in adult patients with nonmeningeal systemic pneumococcal infections. *Am J Med.* Aug 01 2002;113(2):120-126.
310. Jehl FB, JP.; Poirer, R.; Leophonte, P.; Sirot, J.; Chardon H. Enquête nationale sur les pneumonies communautaires à pneumocoque chez des malades adultes hospitalisés. *Médecine et maladies infectieuses.* 2002;+

32(6):267-283.
311. Weinstein MP, Klugman KP, Jones RN. Rationale for revised penicillin susceptibility breakpoints versus Streptococcus pneumoniae: coping with antimicrobial susceptibility in an era of resistance. *Clinical infectious diseases : an official publication of the Infectious Diseases Society of America.* Jun 1 2009;48(11):1596-1600.
312. Carmeli Y, Eliopoulos G, Mozaffari E, Samore M. Health and economic outcomes of vancomycin-resistant enterococci. *Arch Intern Med.* Oct 28 2002;162(19):2223-2228.
313. DiazGranados CA, Zimmer SM, Klein M, Jernigan JA. Comparison of mortality associated with vancomycin-resistant and vancomycin-susceptible enterococcal bloodstream infections: a meta-analysis. *Clinical infectious diseases : an official publication of the Infectious Diseases Society of America.* Aug 1 2005;41(3):327-333.
314. Salgado CD, Farr BM. Outcomes associated with vancomycin-resistant enterococci: a meta-analysis. *Infection control and hospital epidemiology.* Sep 2003;24(9):690-698.
315. da Silva NS, Muniz VD, Estofolete CF, Furtado GH, Rubio FG. Identification of temporal clusters and risk factors of bacteremia by nosocomial vancomycin-resistant enterococci. *American journal of infection control.* Apr 2014;42(4):389-392.
316. Yoo J-H, Lee, D-G., Choi, SM., Choi, J-H., Shin, W-S., Kim, M., Yong, D., Lee, K., Min, W-S., Kim, C-C. Vancomycin-resistant Enterococcal Bacteremia in a Hematology Unit: Molecular Epidemioogy and Analysis of Clinical Course. *Journal of Korean medical science.* 2005;20:169-176.
317. Lodise TP, McKinnon PS, Tam VH, Rybak MJ. Clinical outcomes for patients with bacteremia caused by vancomycin-resistant enterococcus in a level 1 trauma center. *Clinical infectious diseases : an official publication of the Infectious Diseases Society of America.* Apr 1 2002;34(7):922-929.
318. Peset V, Tallon P, Sola C, et al. Epidemiological, microbiological, clinical, and prognostic factors of bacteremia caused by high-level vancomycin-resistant Enterococcus species. *European journal of clinical microbiology & infectious diseases : official publication of the European Society of Clinical Microbiology.* Oct 2000;19(10):742-749.
319. Garbutt JM, Ventrapragada M, Littenberg B, Mundy LM. Association between resistance to vancomycin and death in cases of Enterococcus faecium bacteremia. *Clinical infectious diseases : an official publication of the Infectious Diseases Society of America.* Mar 2000;30(3):466-472.
320. Bhavnani S, Drake, JA., Forrest, A., Deinhart, JA., Jones, RN. Biedenbach, DJ., Ballow, CH. Nationwide, multicenter, case-control study comparing risk factors, treatment, and outcome for vancomycin-resistant and -susceptible enterococcal bacteremia. *Diagn Microbiol Infect Dis.* 2000;36:145-158.
321. Lucas GM, Lechtzin N, Puryear DW, Yau LL, Flexner CW, Moore RD. Vancomycin-resistant and vancomycin-susceptible enterococcal bacteremia: comparison of clinical features and outcomes. *Clinical infectious diseases : an official publication of the Infectious Diseases Society of America.* May 1998;26(5):1127-1133.
322. Stosor V, Peterson LR, Postelnick M, Noskin GA. Enterococcus faecium bacteremia: does vancomycin resistance make a difference? *Arch Intern Med.* Mar 9 1998;158(5):522-527.
323. Linden PK, Pasculle AW, Manez R, et al. Differences in outcomes for patients with bacteremia due to vancomycin-resistant Enterococcus faecium or vancomycin-susceptible E. faecium. *Clinical infectious diseases : an official publication of the Infectious Diseases Society of America.* Apr 1996;22(4):663-670.
324. Krcmery V, Bilikova E, Svetlansky I, Kovacicova G. Is vancomycin resistance in enterococci predictive of inferior outcome of enterococcal bacteremia? *Clinical infectious diseases : an*

official publication of the Infectious Diseases Society of America. Apr 1 2001;32(7):1110-1112.
325. Vergis EN, Hayden MK, Chow JW, et al. Determinants of vancomycin resistance and mortality rates in enterococcal bacteremia. a prospective multicenter study. *Annals of internal medicine.* Oct 2 2001;135(7):484-492.
326. Shay DK, Maloney SA, Montecalvo M, et al. Epidemiology and mortality risk of vancomycin-resistant enterococcal bloodstream infections. *J Infect Dis.* Oct 1995;172(4):993-1000.
327. Wells CL, Juni BA, Cameron SB, et al. Stool carriage, clinical isolation, and mortality during an outbreak of vancomycin-resistant enterococci in hospitalized medical and/or surgical patients. *Clinical infectious diseases : an official publication of the Infectious Diseases Society of America.* Jul 1995;21(1):45-50.
328. Cheah AL, Spelman T, Liew D, et al. Enterococcal bacteraemia: factors influencing mortality, length of stay and costs of hospitalization. *Clinical microbiology and infection : the official publication of the European Society of Clinical Microbiology and Infectious Diseases.* Apr 2013;19(4):E181-189.
329. DiazGranados CA, Jernigan JA. Impact of vancomycin resistance on mortality among patients with neutropenia and enterococcal bloodstream infection. *J Infect Dis.* Feb 15 2005;191(4):588-595.
330. Peel T, Cheng AC, Spelman T, Huysmans M, Spelman D. Differing risk factors for vancomycin-resistant and vancomycin-sensitive enterococcal bacteraemia. *Clinical microbiology and infection : the official publication of the European Society of Clinical Microbiology and Infectious Diseases.* Apr 2012;18(4):388-394.
331. Mainous MR, Lipsett PA, O'Brien M. Enterococcal bacteremia in the surgical intensive care unit. Does vancomycin resistance affect mortality? The Johns Hopkins SICU Study Group. *Arch Surg.* Jan 1997;132(1):76-81.
332. Centers for Disease C, Prevention. Nosocomial enterococci resistant to vancomycin--United States, 1989-1993. *MMWR Morb Mortal Wkly Rep.* Aug 06 1993;42(30):597-599.
333. Lautenbach E, Bilker WB, Brennan PJ. Enterococcal bacteremia: risk factors for vancomycin resistance and predictors of mortality. *Infection control and hospital epidemiology.* May 1999;20(5):318-323.
334. Stroud L, Edwards J, Danzing L, Culver D, Gaynes R. Risk factors for mortality associated with enterococcal bloodstream infections. *Infection control and hospital epidemiology.* Sep 1996;17(9):576-580.
335. Song X, Srinivasan A, Plaut D, Perl TM. Effect of nosocomial vancomycin-resistant enterococcal bacteremia on mortality, length of stay, and costs. *Infection control and hospital epidemiology.* Apr 2003;24(4):251-256.
336. Edmond MB, Ober JF, Dawson JD, Weinbaum DL, Wenzel RP. Vancomycin-resistant enterococcal bacteremia: natural history and attributable mortality. *Clinical infectious diseases : an official publication of the Infectious Diseases Society of America.* Dec 1996;23(6):1234-1239.
337. Edmond MB, Ober JF, Weinbaum DL, et al. Vancomycin-resistant Enterococcus faecium bacteremia: risk factors for infection. *Clinical infectious diseases : an official publication of the Infectious Diseases Society of America.* May 1995;20(5):1126-1133.
338. Butler AM, Olsen MA, Merz LR, et al. Attributable costs of enterococcal bloodstream infections in a nonsurgical hospital cohort. *Infection control and hospital epidemiology.* Jan 2010;31(1):28-35.

339. Khair HN, VanTassell P, Henderson JP, Warren DK, Marschall J, Program CDCPE. Vancomycin resistance has no influence on outcomes of enterococcal bacteriuria. *The Journal of hospital infection.* Nov 2013;85(3):183-188.
340. Tornieporth NG, Roberts RB, John J, Hafner A, Riley LW. Risk factors associated with vancomycin-resistant Enterococcus faecium infection or colonization in 145 matched case patients and control patients. *Clinical infectious diseases : an official publication of the Infectious Diseases Society of America.* Oct 1996;23(4):767-772.

Literature selection grids

Alessandro Cassini and Diamantis Plachouras with contributions from Ana Hoxha, Liselotte Diaz Högberg and Carl Suetens

**Sample size**	**N** refers to total number of cases and controls **(no score)**
	**Calculation *a priori:*** ++ if performed (e.g. based on expected prevalence or on litterature data) or the study refers to large national surveys; - if not performed
**Study type**	**Study type:** ++ matched cohort or case-control studies; + prospective cohort or case-control studies; - restrospective database studies
**Infection type**	**HCA:** health-care associated; **CA:** community associated; **Mix:** health-care and community associated together
**Representativeness**	**Geographical:** ++ if the study was conducted in an EU/EEA setting; + if the study was conducted in high-income country(ref. World bank); - if the study was not conducted in an EU/EEA or high-i
	**Demographical:** ++ if the study population represents all age groups; + if the study population represents selected age-specific groups (e.g. elderly, infants); - if the study population represents a
	**Clinical:** ++ if the study population represents the overall hospital population; + if the study population partially represents the hospital population; - if the study population represents a disease-sp
**Matching or controlling for confounders**	**Exlusion/inclusion criteria**: ++ if the same exclusion/inclusion criteria were adopted for both cases and controls; + if some (but not all) exclusion/inclusion criteria were adopted for both cases an
	**Demographics:** ++ if performed for age and sex; + if performed only for age or only for sex; - if not performed at all
	**Underlying disease:** ++ if performed for two or more criteria (e.g. comorbidity score, McCabe score, severity index, n. comorbidities, allergy, immunosuppression status, prior hospitalisation etc..)
	**Infection site**: ++ if performed ; - if not performed
	**Hospital and unit/ward:** ++ if same hospital and same unit/ward; + if same hospital but different unit/ward; - if not same hospital and/or not same unit/ward
	**Follow-up**: ++ if ≥ 28 days; - if < 28 days
**Risk difference**	**Statistically significant outcome results:** ++ if results and 95%CI are statistically significant; - if results and 95%CI are not statistically significant
	**Case fatality and LOS** refer to risk difference estimates in cases and controls **(no score)**

Scoring table

++	Matches completely/is completely fulfilled
+	Matches incompletely but sufficiently/is only partly but sufficiently fulfilled
-	Does not match or matches insufficiently/is insufficiently fulfilled
c.b.e.	Cannot be evaluated

etting
tegory and age-specific groups
(e.g. liver transplanted patients, cancer patients, etc..) xclusion/inclusion
criteria were different in cases and controls

for only one criteria; - if not performed at all

3GCREC	third-generation cephalosporin-resistant *E. coli*
3GCRKP	third-generation cephalosporin-resistant *K. pneumoniae*
AMR	antimicrobial resistance
BSI	bloodstream infection
CAI	Community-associated infections
CI	confidence interval
ColRACI	colistin-resistant *Acinetobacter* spp.
ColREC	colistin-resistant *E. coli*
ColRKP	colistin-resistant *K. pneumoniae*
ColRPA	colistin-resistant *P. aeruginosa*
CRACI	carbapenem-resistant *Acinetobacter* spp.
CREC	carbapenem-resistant *E. coli*
CRKP	carbapenem-resistant *K. pneumoniae*
CRPA	carbapenem-resistant *P. aeruginosa*
CSF	cerebral spine fluid
DALY	disability-adjusted life years
EARS-Net	European Antimicrobial Resistance Surveillance Network
EEA	European Economic Area
EU	European Union
HAI	healthcare-associated infection
LOS	length of stay
MDR	multidrug-resistant
MDRACI	multidrug-resistant *Acinetobacter* spp
MDRPA	multidrug-resistant *P. aeruginosa*
MRSA	meticillin-resistant *Staphylococcus aureus*
MS	Member State
OECD	Organisation for Economic Co-operation and Development
OTH	other infection site including digestive tract infections, skin and soft tissue infections (SSTI), eye, ear, nose or mouth infections, bone and joint infections, cardiovascular infections, reproductive tract infections and other less frequent infections
PMRSP	penicillin- and macrolide-resistant S. pneumoniae
PPS	point prevalence survey of healthcare-associated infections and antimicrobial use in acute care hospitals
PRSP	penicillin-resistant *S. pneumoniae* ;
RESP	respiratory infections (including pneumonia, and low respiratory tract infection)
S-BSI	secondary BSI
SP	specified pathogens
SPDAR	specified pathogens with defined antimicrobial resistance
SSI	surgical site infection
UTI	urinary tract infection
VRE	vancomycin-resistant enterococci

3GCRKP C[illegible]list for the evaluation of the most relevant studies for building antimicrobial resistance (AMR) outcome trees, healthcare-associated infection (HAI) attributable case fatality and attributable length-of-stay (LOS)

	Study			Study type	Infection type	Sample size		Representativeness			Matching or controlling for confounders						Risk difference		
						N (cases;controls)	calculation *a priori*	Geographical	Demographical	Clinical	Exclusion/inclusion criteria	Demographics	Underlying disease	Infection site	Hospital and unit/ward	Follow-up	Statistically significat outcome results	Case fatality	LOS
	Nr.	First author, year			HCA; CA; Mix; c.b.e		□ ++; □ -; □ c.b.e	□ ++; +; -; □ c.b.e	□ ++; □ +; □ -; □ c.b.e	□ ++; -; □ c.b.e	□ ++; □ +; □ -; □ c.b.e	□ ++; □ +; □ -; □ c.b.e	□ ++; □ +; □ -; □ c.b.e	□ ++; □ -; □ c.b.e	□ ++; □ +; □ -; □ c.b.e	□ ++; -; □ c.b.e	□ ++; □ -; □ c.b.e	%; RR; OR; aHR; etc	(days)
BSI R vs NI																			
	1	Gallagher, 2014	Score	++	c.b.e	111 cases 111 controls	-	+	++	++	++	-	-	-	++	c.b.e	++	Mort. rate cases=32%<br>Mort.rate contr=13%<br>OR=3.6; 95%CI(1.2 to 10.5)	mean<br>cases=63<br>controls=20
			Comments	Matched Case-control study (matched only by month/year and unit)	no information in the article							no univariate analyses	no univariate analyses	R vs NI		mortality in hospital			
	2	Stewardson, 2016	Score	-	Mix	360 cases 2100 controls 604797 NI	++	++	++	+	++	-	-	-	-	c.b.e	++	Mort. rate resistant=16.1%<br>Mort.rate sensitive=10.1%<br>Mort.rate NI=1.7%<br>R vs NI: HR*=2.88/2.25/1.80<br>R vs S: HR*=1.39/1.43/1.63	no specific data, see full-text for modelling values*
			Comments	Multicentre retrospective cohort study	HC onset: 59.4% cases, 37.1% controls community onset: 40.6% cases, 62.9% controls	the study analysed Enterobacteriaceae (E. coli, K. spp. and Proteus spp), but included because of its methodological value				patients with acute-care episodes lasting more than one day		no univariate analyses matched in model 2	no univariate analyses	all BSI or NI		mortality in hospital	all HR	* the study models the excess in LOS and mortality between groups proposing 3 models for each	* the study models the excess in LOS and mortality between groups proposing 3 models for each
BSI R vs S																			
	1	Lee, 2014	Score	-	CA	9 cases 153 controls	-	+	++	++	++	-	-	-	+	++	-	a)Mort.rate cases=33.3%<br>Mort.rate controls=24.2%<br>b)Mort.rate cases=33.3%<br>Mort.rate controls=16.3%	no data
			Comments	Retrospective case-control study								no univariate analyses	no univariate analyses	all BSI		a)30 day mortality b)bacteremia-related mortality		a)30 day mortality<br>b)bacteremia-related mortality	
	2	Panhotra, 2014	Score	-	HCA	10 cases 16 controls	-	+	++	++	++	-	-	-	+	c.b.e	++	Mort.rate cases=60%<br>Mort.controls=6.2%	cases=44.2<br>controls-24.1
			Comments	Retrospective case-control study										all BSI		mortality in hospital			
	3	Tumbarello, 2006	Score	-	Mix	48 cases 99 controls	-	++	++	++	++	+	++	-	+	-	++	a) Mort.rate cases= 25%<br>Mort.rate controls=11%<br>b) Mort. rate cases=52%<br>Mort.rate controls=29%<br>OR=2.62; 95%CI(1.28-5.35)	mean ± SD<br>cases=34±20<br>controls=31±17
			Comments	Retrospective case-case-control study	HCA: 96% cases, 82% controls							not matched but controlled in univariate analyses for gender, age was higher in	not matched but controlled in univariate analyses for Charlson index, APACHE II score and main	all BSI		a)7 days b)21 days		a)7 days<br>b)21 days	
	4	Marra, 2006	Score	-	HCA	56 cases 52 controls	-	-	++	++	++	++	++	-	+	-	++	Mort. rate cases=32%<br>Mort.rate controls=15%	no specific data
			Comments	Retrospective cohort study					included adults and infants			not matched but controlled in univariate analyses	not matched but controlled in univariate analyses for McCabe score and nr. Of comorbidities	all BSI		15 days			
	5	Kang, 2004	Score	-	Mix	10 cases 20 controls	-	+	++	-	++	++	++	-	+	++	c.b.e	Mort. rate cases=60%<br>Mort.rate controls=30%	
			Comments	Retrospective cohort study	HCA: 93.3% cases, 93.3% controls					all patients had liver chirrosis and spontaneous bacterial peritonitis			matched for Child-Pugh score (severity of chirrosis) and APACHE II score	all BSI		30 days	no test for KP since the study researched KP and EC together, mortality data were available only for KP		
	6	Kim BN, 2002	Score	-	Mix	44 cases 118 controls	-	+	++	++	++	++	++	-	+	c.b.e	-	Mort. rate cases=23%<br>Mort.rate controls=20%	Mean<br>cases=39.6<br>controls=23.9
			Comments	Retrospective cohort study	HCA: 86.4% cases, 42.4% controls							not matched but controlled in univariate analyses	not matched but controlled in univariate analyses for McCabe score	all BSI		mortality in hospital			
	7	Ariffin, 2000	Score	-	c.b.e	16 cases 15 controls	-	-	+	-	++	c.b.e	+	-	++	c.b.e	++	Mort. rate cases=50%<br>Mort.rate controls=13%<br>OR=6.5; 95%CI(1.1-38.6)	
			Comments	Retrospective cohort study	no information in the article				all patients are pediatric patients <12 years	all patients were in pediatric oncology unit and had febrile neutropenia		no information	not matched but controlled in univariate analyses for hematological malignacies	all BSI		mortality in hospital		the article defines the outcome as sepsis related mortality	
	8	Mosqueda-Gomez, 2008	Score	-	Mix	17 cases 104 controls	-	-	++	++	++	+	-	-	+	c.b.e	-	Mort. rate cases=35%<br>Mort.rate controls=26.9%	mean<br>cases=20.9<br>controls=15.9
			Comments	Retrospective case-control study	HCA: 100% cases, 45.2% controls							not matched but controlled for gender in univariate analyses, age was higher in controls		all BSI		mortality in hospital			
	9	Szilagyi, 2009	Score	-	HCA	100 cases 100 controls	-	++	++	++	++	++	-	-	-	c.b.e	++	a) Mort. rate cases=36%<br>Mort.rate controls=23%<br>OR=2.5; 95%CI(1.0–5.4)<br>b) Mort. rate cases=18%<br>Mort.rate controls=18%<br>OR=5; 95%CI(1.5–16.2)	median<br>cases=10.5<br>controls=10
			Comments	Retrospective cohort study								not matched but controlled in univariate analyses		all BSI		mortality in hospital		a)all cause mort.<br>b)inf. related mort.	
	10	Pena, 2001	Score	+	HCA	49 cases 43 controls	-	++	++	+	++	++	-	-	+	c.b.e	-	a)Mort. rate cases=32%<br>Mort.rate controls=24%<br>b)Mort. rate cases=16%<br>Mort.rate controls=14%	
			Comments	Prospective cohort study						patients were part of an outbreak of ESBL-KP		not matched but controlled in univariate analyses		all BSI		mortality in hospital		a)overall mortality<br>b)attributable mortality	
	11	Paterson, 2004	Score	+	HCA	78 cases 175 controls	-	c.b.e	++	++	++	++	-	-	-	-	-	Mort. rate cases=27%<br>Mort.rate controls=23%	before isolation<br>mean±SD<br>cases=36.3±5.4<br>controls=19.9±1.9
			Comments	International prospective study				different countries; South Africa, Taiwan, Australia, Argentina, the United States, Belgium, and Turkey	>16 years			not matched but controlled in univariate analyses		all BSI		14 days			
	12	Tuon, 2010	Score	-	c.b.e	63 cases 41 controls	-	-	++	++	++	+	+	-	+	++	-	Mort. rate cases=49.2%<br>Mort.rate controls=41.9%	Mean ± SD<br>cases= 52.2 ± 39.6<br>controls=36.0 ±29.4
			Comments	Retrospective cohort study	no information in the article				>12 years			not matched but controlled in univariate analyses for age, gender was not equally distributed	not matched but controlled in univariate analyses for main diseases, only diabetes was higher in controls	all BSI		30 days			
	13	Lee, 2011	Score	-	CA	33 cases 219 controls	-	+	++	+	++	++	++	-	+	c.b.e	-	Mort. rate cases=12.1%<br>Mort.rate controls=16%	Mean ± SD<br>cases=26.5 ± 39.0<br>controls=19.9 ± 42.4
			Comments	Retrospective case-control study	community onset infection HCA: 75.8% cases, 56.2% controls	community-onset bacteremia				patients from Emergency department, with community onset infection		not matched but controlled in univariate analyses	not matched but controlled in univariate analyses for McCabe score and main diseases	all BSI		mortality in hospital			
UTI R vs S																			
	1	Brizendine, 2015	Score	-	HCA	22 cases 64 controls	-	+	++	-	++	+	+	-	+	c.b.e	-	Mort. cases=18%<br>Mort. Contr.=2%	Prior to UTI<br>Median (IQR)<br>cases=8(1-14)<br>controls=1(1-4)
			Comments	Retrospective cohort study	not expicitly said in the article, but very specific patients					all patients had solid organ transplant		not matched but controlled in univariate analyses for age, gender was not equally distributed	all patients had solid organ transplant	all UTIs		mortality in hospital			
Resp tract R vs S																			
	1	Loh, 2006	Score	-	Mix	47 cases 394 controls	-	-	++	++	++	++	++	-	+	c.b.e	-	Mort. rate cases=21.3%<br>Mort.rate controls=12.4%	Median (IQR)<br>cases=14(8-32)<br>controls=5(3-10)
			Comments	Retrospective cohort study	no data on proportion HCA/CA				>12 years			not matched but controlled in univariate analyses	not matched but controlled in univariate analyses for comorbidity scoring	all Resp tract		mortality in hospital			
Mixed inf. site R vs S																			
	1	Gomes, 2006	Score	-	HCA	68 cases 75 controls	++	-	++	++	++	-	-	-	+	-	c.b.e	Mort. rate cases=20.6%<br>Mort.rate controls=21.3%	no data
			Comments	Retrospective cohort study								univariate not available because compared died vs survived patients				21 days	univariate not available because compared died vs survived patients		
	2	Lin, 2003	Score	+	Mix	43 cases 86 controls	-	+	++	++	++	+	++	++	+	c.b.e	-	Mort. rate cases=27.9%<br>Mort.rate controls=20.9%	mean(range)<br>cases=45 (0-143)<br>controls=18 0-82)
			Comments	Prospective case-control study	HCA: 44.2% cases, 14% controls							not matched but controlled in univariate analyses for gender, age was higher in cases	not matched but controlled in univariate analyses for main diseases			mortality in hospital			
	3	Kuo, 2007	Score	-	Mix	54 cases 54 controls	-	+	+	+	++	++	-	++	++	++	-	Mort. rate cases=15%<br>Mort.rate controls=13%	mean(range)<br>cases=45 (0-143)<br>controls=18 0-82)
			Comments	Retrospective case-control study	HCA: 79.6% cases, 48.1% controls				all patients were children	patients were in ICU		not matched but controlled in univariate analyses				30 days			
	4	Chiu, 2005	Score	-	HCA	15 cases 16 controls	-	+	+	+	++	+	c.b.e	c.be	++	c.b.e	c.b.e	Mort. rate cases=20%<br>Mort.rate controls=12.5%	no data
			Comments	Retrospective case-control study		nosocomial infections			all patients were neonatals	patients were in NICU		not matched but controlled in univariate analyses for age	the study was on Enterobacteriaceae and despite mortality data no else could be extrapolated	the study was on Enterobacteriaceae and despite mortality data no else could be extrapolated		mortality in hospital	the study was on Enterobacteriaceae and despite mortality data no else could be extrapolated		
	5	Huang, 2007	Score	+	HCA	19 cases 12 controls	-	-	+	+	++	c.b.e	-	-	++	++	c.b.e	Mort. rate cases=10.5%<br>Mort.rate controls=0%	no data
			Comments	Prospective cohort study		nosocomial infections			all patients were neonatals	patients were in NICU		study was on KP and EC and data could not be separated				30 days		study was on KP and EC and data could not be separated	
	6	BARF, 2002	Score	-	Mix	9 cases 9 controls	-	+	++	++	++	+	++	++	-	c.b.e	-	Mort. rate cases=44%<br>Mort.rate controls=33%	median:<br>cases=20<br>controls=33.5
			Comments	Survey	HCA: 95% cases, 39% controls							matched for age	matched for major diseases			mortality in hospital			

Sample size — **N** refers to total number of cases and controls **(no score)**
Calculation *a priori*: ++ if performed (e.g. based on expected prevalence or on litterature data) or the study refers to large national surveys; - if not performed

Study type — **Study type:** ++ matched cohort or case-control studies; + prospective cohort or case-control studies; - restrospective database studies

Infection type — **HCA:** health-care associated; **CA:** community associated; **Mix:** health-care and community associated together

Representativeness
Geographical: ++ if the study was conducted in an EU/EEA setting; + if the study was conducted in high-income country(ref. World bank); - if the study was not conducted in an EU/EEA or high-income country setting
Demographical: ++ if the study population represents all age groups; + if the study population represents selected age-specific groups (e.g. elderly, infants); - if the study population represents a selected sex category and age-specific groups
Clinical: ++ if the study population represents the overall hospital population; + if the study population partially represents the hospital population; - if the study population represents a disease-specific population (e.g. liver transplanted patients, cancer patients, etc..)

Matching or controlling for confounders
Exlusion/inclusion criteria: ++ if the same exclusion/inclusion criteria were adopted for both cases and controls; + if some (but not all) exclusion/inclusion criteria were adopted for both cases and controls; - if exclusion/inclusion criteria were different in cases and controls
Demographics: ++ if performed for age and sex; + if performed only for age or only for sex; - if not performed at all
Underlying disease: ++ if performed for two or more criteria (e.g. comorbidity score, McCabe score, severity index, n. comorbidities, allergy, immunosuppression status, prior hospitalisation etc..); + if performed for only one criteria; - if not performed at all
Infection site: ++ if performed ; - if not performed
Hospital and unit/ward: ++ if same hospital and same unit/ward; + if same hospital but different unit/ward; - if not same hospital and/or not same unit/ward
Follow-up: ++ if ≥ 28 days; - if < 28 days

Risk difference — **Statistically significant outcome results:** ++ if results and 95%CI are statistically significant; - if results and 95%CI are not statistically significant
Case fatality and LOS refer to risk difference estimates in cases and controls **(no score)**

Scoring table

++	Matches completely/is completely fulfilled
+	Matches incompletely but sufficiently/is only partlybut sufficiently fulfilled
-	Does not match or matches insufficiently/is insufficiently fulfilled
c.b.e.	Cannot be evaluated

CRKP **Checklist for the evaluation of the most relevant studies for building antimicrobial resistance (AMR) outcome trees, healthcare-associated infection (HAI) attributable case fatality and attributable length-of-stay (LOS)**

	Study			Study type	Infection type	Sample size		Representativeness			Matching or controlling for confounders						Risk difference		
						N (cases;controls)	calculation *a priori*	Geographical	Demographical	Clinical	Exclusion/inclusion criteria	Demographics	Underlying disease	Infection site	Hospital and unit/ward	Follow-up	Statistically significat outcome results	Case fatality	LOS
	Nr.	First author, year			HCA; CA; Mix; c.b.e		□ ++; □ -; □ c.b.e	□ ++; +; -; □ c.b.e	□ ++; □ +; □ -; □ c.b.e	□ ++; -; □ c.b.e	□ ++; □ +; □ -; □ c.b.e	□ ++; □ +; □ -; □ c.b.e	□ ++; □ +; □ -; □ c.b.e	□ ++; □ -; □ c.b.e	□ ++; □ +; □ -; □ c.b.e	□ ++; -; □ c.b.e	□ ++; □ -; □ c.b.e	%; RR; OR; aHR; etc	(days)
BSI<br>R vs NI	1	Borer, 2009	Score	++	c.b.e	32 cases<br>32 controls	-	+	++	++	++	++	++	-	+	c.b.e	++	Mort. rate cases=71.9%<br>Mort. rate controls=21.9%<br>RR=3.3; 95%CI(2.9-28.5)	median:<br>cases=21<br>controls=18
			Comments	Matched retrospective study	no information in the article	controls had an infectious disease but not BSI								BSI vs NI (without BSI)		mortality in hospital			
	2	Gallagher, 2014	Score	++	c.b.e	43 cases<br>154 controls	-	+	++	++	++	-	-	-	++	c.b.e	++	Mort.cases= 45%<br>Mort.contr= 18%<br>OR=3.8; 95%CI(1.4-9.9)	mean:<br>cases=54<br>controls=19
			Comments	Matched Case-control study (matched only by month/year and unit)	no information in the article							no univariate analyses	no univariate analyses	BSI vs NI		mortality in hospital			
	3	Mouloudi, 2014	Score	++	HCA	17 cases<br>34 controls	-	++	++	-	++	++	++	-	++	c.b.e	c.b.e	Mort.cases=82.7%<br>Mort. contr=32%<br>□	in ICU<br>median (range):<br>cases=28(10-64)<br>controls=no info
			Comments	Observational matched cohort study						patients with liver transplant in ICU	patients with liver transplant in ICU	not matched but controlled in univariate analyses	not matched but controlled in univariate analyses for main indicators	BSI vs NI	ICU	mortality in hospital	no infrmation		
BSI<br>R vs S	1	Falagas, 2014	Score	-	c.b.e	985 patients	-	c.b.e	++	++	++	c.b.e	c.b.e		-	c.b.e	++	Attribut. mort.= 26-44%<br>in 7 studies<br>(-3) and (-4)% in 2 remaining studies<br>RR=2.05; 95%CI(1.56–2.69)*<br>RR 2.19; 95%CI(1.82–2.63)**<br>RR=1.46; 95%CI(0.47–4.49)***	no data
			Comments	Review Metanalysis (on Enterobacteriaceae, but 8/9 studies	no information in the article			Articles from different countries			the same refered to cases and controls of each study	different in the different studies	different in the different studies	6 studies were on BSI, the other 3 were on mixed site of infection		different in different studies	only RR* and RR**	* referred to all 9 studies<br>** only 6 BSI studies<br>*** remaining 3 (not BSI/mixed site of infection) studies	
	2	Mouloudi, 2010	Score	+	HCA	37 cases<br>22 controls	-	++	++	+	++	++	++	-	++	c.b.e	c.b.e	a) Mort.cases= 56.8%<br>Mort.contr= 41%<br>b) Mort.cases=<br>65%Mort=41%<br>c)Mort.cases= 27%<br>Mort.contr= 14%	median(range):<br>total=26 (8-90) in ICU
			Comments	Prospective nested case-control study		cases were all CRKP, 18 metallo-β-lactamase KP and 17 carbapenemase-producing KP. The mortality for data were summed up together				ICU patientd		not matched but controlled in univariate analyses	not matched but controlled in univariate analyses for main diseases	all BSI		mortality in hospital	no information	a) mortality in ICU<br>b) mortality in hospital<br>c) attributable mortality (within 24h from infection or with infection symptoms despite appropriate therapy)	
	3	Simkins, 2014	Score		c.b.e	13 cases<br>39 controls		+								++		Mort.cases= 46%<br>Mort.contr= 0%	no data
			Comments	Case-control study	no information in the article											6.5 months			
	4	Viale, 2013	Score	-	Mix	c.b.e	-	c.b.e	++	++	NA	c.b.e	c.b.e	-	NA	c.b.e	c.b.e	Crude mortality rates =20-70%<br>Attribut. mort. patients:<br>-hematologic=75%<br>-ICU=11%<br>-transplant=7%<br>-LTC=5%<br>-medicine=2%<br>-surgery=2%	median time in developing CRKP infections in hospital (range): cases=14(0-152)
			Comments	Review		the review does not pool the patients together, but considers every study separately		Articles from different countries				articled with different results	articled with different results	all BSI		different in different studies			
	5	Hussein, 2013	Score	-	HCA	103 cases<br>214 controls	-	+	+	++	++	++	-	-	+	++	++	Mort.cases= 43.7%<br>Mort.contr= 29%	median (range):<br>cases=17(1-125)<br>controls=12(1-202)
			Comments	Retrospective case-control study		all patients had health-care associated infection						not matched but controlled in univariate analyses		all BSI		30 days			
	6	Ben-David, 2012	Score	-	Mix	42 cases<br>85 controls	-	+	++	++	++	++	++	-	+	c.b.e	++	a)Mort. cases=69%<br>Mort. controls=24%<br>b)Mort. cases=48%<br>Mort. controls=17%	mean LOS before infection detection ±SD: survivors=25±81<br>deceased=27±37
			Comments	Retrospective cohort study	HCA: 100% cases, 79% controls							not matched but controlled in univariate analyses	not matched but controlled in univariate analyses for Charlson's score, not other diseases	all BSI		mortality in hospital		a)in hospital mortality<br>b)infection related mortality	
	7	Daikos, 2009	Score	+	c.b.e	14 cases<br>148 controls	-	++	++	++	++	+	-	-	-	-	-	Mort.cases= 42.8%<br>Mort.contr= 16.9%<br>OR=3.95; 95%CI(0.94–16.6)	mean LOS before bacteraemia ±SD:<br>both groups=25.85 ± 37.87
			Comments	Prospective observational study	no information in the article							not matched but controlled in univariate analyses for gender, age higher in controls		all BSI	2 hospitals	14 days			
UTI<br>R vs S	1	Brizendine, 2015	Score	-	c.b.e	22 cases<br>64 controls	-	+	++	-	++	++	+	-	+	c.b.e	c.b.e	Mort. cases=18%<br>Mort. contr.=2%	median:<br>cases=7<br>controls=1
			Comments	Retrospective cohort study	no information in the article					all patients had solid organ transplant		not matched but controlled in univariate analyses	all patients had solid organ transplant, no other comorbidieties considered	all UTI		mortality in hospital	no information		
	2	Pouch, 2015	Score	-	HCA	20 cases<br>80 controls	-	+	++	-	++	++	-	-	+	c.b.e	++	Mort. cases=30%<br>Mort. contr.=10%<br>HR=3.0; 95%CI(1.0–9.0)	median(IQR):<br>cases=17(9-43)<br>controls=7(5-10)
			Comments	Retrospective case-control study	not expicitly said in the article, but very specific patients					all patients had solid kideny transplant		not matched but controlled in univariate analyses		all UTI		not defined			
	3	Shilo, 2013	Score	-	c.b.e	135 cases<br>127 controls	++	+	++	++	++	++	++	-	+	c.b.e	-	Mort. cases=29%<br>Mort. contr.=25%	mean±SD<br>cases=28±33<br>controls=22±28
			Comments	Retrospective case-control study	no information in the article	cases defined as bacteruria, since UTI relates with symptomps while bacteruria not always						not matched but controlled in univariate analyses	not matched but controlled in univariate analyses for main diseases	all bacteruria		mortality in hospital			
Mixed inf. site<br>R vs NI	1	Giannella, 2014	Score	++	HCA	10 cases<br>20 controls	-	++	++	-	++	++	++	-	+	++	-	a)Mort cases=0%<br>Mort. Contr.=5%<br>b)Mort. cases=0%<br>Mort. contr.=15%	median (range):<br>cases=16(14-27)<br>controls=20(15-62)
			Comments	Matched case-control study	not expicitly said in the article, but very specific patients	the cases were selected as colonized, though all of them after developed BSI				all patients had liver transplant		not matched but controlled in univariate analyses	not matched but controlled in univariate analyses	all cases had positive rectal swabs initially and BSIs after		a)30 days<br>b)60 days		a)30 days<br>b)60 days	
	2	Luebbert, 2014	Score	-	HCA	9 cases<br>18 controls	-	++	++	-	++	++	-	-	+	++	++	Mort. cases=78%<br>Mort. contr.=11%	cases=60<br>controls=32
			Comments	Retrospective case-control study	not expicitly said in the article, but very specific patients					all patients had liver transplant				R vs NI		3 months from last case			
	3	Nouvenne, 2014	Score	-	c.b.e	133 cases<br>(93 colonized, 40infected)<br>400 controls	-	++	++	++	+	++	-	-	++	c.b.e	-	Mort. cases =21.8%<br>(Mort. Infected=47.5%<br>Mort. Colonized=10.7%)<br>Mort.contr. =15%	mean±SD<br>cases=35±24<br>controls=18±12
			Comments	Retrospective cross-sectional study	no information in the article						cases were part of an outbreak, while controls were randomly selcted	not matched but controlled in univariate analyses		R vs NI		mortality in hospital	referes only yo the comparison of all cases (infected+colonized) with controls		
	4	Bleumin, 2012	Score	+	HCA	43 cases<br>150 controls	-	+	++	-	++	+	++	-	++	c.b.e	++	Mort.cases= 88%<br>Mort.contr.= 52%<br>aHR=5.9; 95%CI(3.2-11.0)	no data
			Comments	Prospective nested case control study						all patients were cronic hemodialyses patients		not matched but controlled in univariate analyses for age, cases had more female patients	not matched but controlled in univariate analyses for main diseases, differences in central catheter presence	R vs NI		study period			
	5	Debby, 2012	Score	+	HCA	48 cases<br>132 controls	-	+	++	+	++	++	++	-	++	c.b.e	-	Mort.cases=45.8%<br>Mort.contr.=38.6%	mean±SD<br>cases=6.06±1.23<br>controls=8.39 ±1.49
			Comments	Prospective cohort study		all cases were detected with rectal swab analyses, but developed BSI, pneumonia, etc				ICU patients		not matched but controlled in univariate analyses	not matched but controlled in univariate analyses for main diseases	R vs NI		mortality in hospital			
	6	Schwaber, 2008	Score	-	HCA	48 CRKP<br>56 CSKP<br>59 controls	-	+	++	++	++	++	-	-	+	c.b.e	++	Mort. rate resistant=44%<br>Mort. rate sensitive=12.5%<br>Mort. rate NI=2%<br>R vs S: OR=5.4;<br>95%CI(1.7–17.1)<br>R vs NI: OR=6.7;<br>95%CI(2.4–18.8)	median (IQR):<br>cases=19 (9-38)<br>controls=2 (1-4)
			Comments	Retrospective case-case-control study								not matched but controlled in univariate analyses				mortality in hospital			
	7	Pereira, 2015	Score	-	HCA	20 CRKP<br>36 CSKP<br>248 controls	-	+	++	-	++	++	-	-	+	++	++	Mort. rate resistant=45%<br>Mort. rate sensitive=28%<br>Mort.rate NI=7%<br>R vs NI: HR=6.92;<br>95%CI(3.24–14.79)	Median (range):<br>cases=40(23-82)<br>controls=12 (9-21)
			Comments	Retrospective cohort study	not expicitly said in the article, but very specific patients	the results are presented for R vs (S+NI)				all patients had liver transplant		not matched but controlled in univariate analyses				1 year from transplant	HR, no data on mortality rates	Mortality rates extraxcted from survival analyses	
	8	Kofteridis, 2014	Score		c.b.e	83 resistant<br>78 sensitive<br>161 NI	-	++	++	++	++	+	-	-	+	c.b.e	++	Mort. rate resistant=27%<br>Mort. rate sensitive=15%<br>Mort.rate NI=4%	no data
			Comments	Case-case-control study	no information in the article							not matched but controlled in univariate analyses for age				not defined	significative R vs NI, not significant R vs S		
Mixed inf. site<br>R vs S	1	Candevir Ulu, 2015	Score	-	HCA	47 cases<br>51 controls	-	-	++	++	++	+	-	-	+	c.b.e	-	Mortality cases=44,7%<br>Mortality contr.=51%	mean:<br>cases=37.3<br>controls=29.94<br>median:<br>cases=19<br>controls=11
			Comments	Retrospective cohort study								not matched but controlled in univariate analyses for age				mortality in hospital			
	2	Dizbay, 2014	Score	-	HCA	42 cases<br>798 controls	-	-	++	++	++	++	-	-	+	c.b.e	++	Mortality cases=57.1%<br>Mortality contr.=27.4%	mean<br>cases=38.27<br>controls=28.17
			Comments	Retrospective study								not matched but controlled in univariate analyses				not defined			

3	Hoxha 2016	Score	++	c.b.e	49 cases<br>49 controls	++	++	++	++	++	++	++	++	-	++	++	a) Attribut. mort.= 16%<br>b) Attribut. mort.= 41%<br>mIRR=3.0; 95%CI(1.3-7.1)	no data
		Comments	Matched cohort study	no information in the article							Matched for age and controlled in univariate analyese for gender	not matched but controlled in univariate analyses for Charlson index and SAPS 2 score			a)6 days<br>b)30 days		a)6 days<br>b)30 days	
4	Jiao, 2015	Score	-	HCA	30 cases<br>30 controls	-	-	++	++	++	++	-	-	+	c.b.e	c.b.e	Mort. cases=33.6%<br>Mort. contr.=16.6%	mean:<br>cases=33.8<br>controls=18
		Comments	Retrospective case-control study								Matched for age and controlled in univariate analyese for gender				mortality in hospital	no information		
5	Patel, 2008	Score	++	c.b.e	99 cases<br>99 controls	-	+	++	++	++	++	++	++	+	c.b.e	++	Mort. cases=48%<br>Mort. contr.=26%<br>OR=4.69; 95%CI(1.9–11.58)	mean±SD<br>cases=25.19±24.9<br>controls=6.44±10.1<br>median:<br>cases=21<br>controls=1
		Comments	Matched case-case study	no information in the article							not matched but controlled in univariate analyses	not matched but controlled in univariate analyses for main diseases			mortality in hospital			
6	Correa, 2013	Score	++	HCA	20 cases<br>40 controls	-	-	++	++	++	++	+	++	++	c.b.e	-	Mort. cases=50%<br>Mort. contr.=27.5%	mean:<br>cases=45.5<br>controls=27
		Comments	Matched case-control study		all HCA infections						not matched but controlled in univariate analyses	not matched but controlled in univariate analyses for MC Cabe score, APACHE II score at admission was different			mortality in hospital			
7	CDC Atlanta, 2011	Score	++	c.b.e	19 cases<br>38 controls	-	+	++	++	++	+	++	-	-	c.b.e	c.b.e	Mortality cases=5%<br>Mortality controls=8%	mean:<br>cases=11.4<br>controls=7.4
		Comments	Matched case-case study	no information in the article	outbreak patients						matched for age	not matched but controlled in univariate analyses for Charlson index			mortality in hospital	no information		
8	Falagas, 2007	Score	++	HCA	53 cases<br>53 controls	-	++	++	++	++	++	-	++	-	c.b.e	-	Mortality cases=30.1%<br>Mortality controls=33.9%	mean±SD<br>cases=21.4±23.9<br>contr.=15.3±19.9
		Comments	Matched case-control study								matched for age, and controlled for gender in univariate analyses				mortality in hospital			
9	Hauck, 2016	Score	-	c.b.e	260 cases:<br>(90 BSI, 49 pneumonia, 121 UTI)<br>223 controls*	-	+	++	++	++	++	++	NA	-	c.b.e	++	Mort.rate BSI=38%<br>aHR=2.59; 95%CI(1.52–4.50)<br>Mort.rate Pneu=34%<br>aHR=3.44; 95%CI(1.80–6.48)<br>Mort.rate UTI=7%<br>aHR=0.68; 95%CI(0.30–1.45)<br>Mort.rate contr.=9%	median (IQR)<br>BSI=14 (9-24)<br>Pneu=19 (10-30)<br>UTI=10 (6-17)<br>controls=9 (5-16)
		Comments	Prospective multicenter observational study	no information in the article	*R vs CRKP urinary colonization						not matched but controlled in univariate analyses for age and gender (Pneu & UTI). For BSI were both different	not matched but controlled in univariate analyses for Charlson index and main diseases			mortality in hospital	aHR BSI and aHR Pneu		

Sample size
N refers to total number of cases and controls **(no score)**
Calculation *a priori:* ++ if performed (e.g. based on expected prevalence or on litterature data) or the study refers to large national surveys; - if not performed

Study type — **Study type:** ++ matched cohort or case-control studies; + prospective cohort or case-control studies; - restrospective database studies
Infection type — **HCA:** health-care associated; **CA:** community associated; **Mix:** health-care and community associated together

Representativeness
Geographical: ++ if the study was conducted in an EU/EEA setting; + if the study was conducted in high-income country(ref. World bank); - if the study was not conducted in an EU/EEA or high-income country setting
Demographical: ++ if the study population represents all age groups; + if the study population represents selected age-specific groups (e.g. elderly, infants); - if the study population represents a selected sex category and age-specific groups
Clinical: ++ if the study population represents the overall hospital population; + if the study population partially represents the hospital population; - if the study population represents a disease-specific population (e.g. liver transplanted patients, cancer patients, etc..)
Exlusion/inclusion criteria: ++ if the same exclusion/inclusion criteria were adopted for both cases and controls; + if some (but not all) exclusion/inclusion criteria were adopted for both cases and controls; - if exclusion/inclusion criteria were different in cases and controls

confounders
Demographics: ++ if performed for age and sex; + if performed only for age or only for sex; - if not performed at all
Matching or controlling for Underlying disease: ++ if performed for two or more criteria (e.g. comorbidity score, McCabe score, severity index, n. comorbidities, allergy, immunosuppression status, prior hospitalisation etc..); + if performed for only one criteria; - if not performed at all
Infection site: ++ if performed ; - if not performed
Hospital and unit/ward: ++ if same hospital and same unit/ward; + if same hospital but different unit/ward; - if not same hospital and/or not same unit/ward
Follow-up: ++ if ≥ 28 days; - if < 28 days

Risk difference
Statistically significant outcome results: ++ if results and 95%CI are statistically significant; - if results and 95%CI are not statistically significant
Case fatality and LOS refer to risk difference estimates in cases and controls **(no score)**

Scoring table

++	Matches completely/is completely fulfilled
+	Matches incompletely but sufficiently/is only partlybut sufficiently fulfilled
-	Does not match or matches insufficiently/is insufficiently fulfilled
c.b.e.	Cannot be evaluated

ColRKP

Checklist for the evaluation of the most relevant studies for building antimicrobial resistance (AMR) outcome trees, healthcare-associated infection (HAI) attributable case fatality and attributable length-of-stay (LOS)

	Study			Study type	Infection type	Sample size		Representativeness			Matching or controlling for confounders						Risk difference		
						N (cases;controls)	calculation *a priori*	Geographical	Demographical	Clinical	Exclusion/inclusion criteria	Demographics	Underlying disease	Infection site	Hospital and unit/ward	Follow-up	Statistically significat outcome results	Case fatality	LOS
	Nr.	First author, year			HCA; CA; Mix; c.b.e		□ ++; □ -; □ c.b.e	□ ++; +; -; □ c.b.e	□ ++; □ +; □ -; □ c.b.e	□ ++; -; □ c.b.e	□ ++; □ +; □ -; □ c.b.e	□ ++; □ +; □ -; □ c.b.e	□ ++; □ +; □ -; □ c.b.e	□ ++; □ -; □ c.b.e	□ ++; □ +; □ -; □ c.b.e	□ ++; -; □ c.b.e	□ ++; □ -; □ c.b.e	%; RR; OR; aHR; etc	(days)
Mixed inf. site R vs S	1	Marchaim, 2010	Score	+	HCA	5 cases 60 controls	-	+	++	++	++	-	-	-	-	c.b.e.	-	Mort. rate cases=40% Mort. rate controls=26%	Mean±SD Cases=33±23 Controls=30±23
			Comments	Prospective case-cohort Study		all patients were also CRKP										mortality in hospital			
	2	Zarkotou, 2010	Score	++	HCA	13 cases 39 controls	-	++	++	++	++	+	++	++	++	c.b.e.	++	Mort. rate cases=69.2% Mort. Rate controls=35.9% Attribut. mort.*=37.5% in both groups	Mean±SD Cases= 20.5±20.8 Controls= 16.4±12.3
			Comments	Matched Case-Control Study								not matched but controlled for age in univariate analyese. Gender is different	not matched but controlled for main comorbidities and there was no difference			mortality in hospital	difference in all cause mortality	*not defined in the paper	
	3	Capone, 2013	Score	+	HCA	35 cases 62 controls	-	++	++	++	++	c.b.e	c.b.e	c.b.e	c.b.e	c.b.e.	++	Mort. rate cases=40.6% Mort. rate controls=20.3%	not specific data
			Comments	Multicentre Prospective Study								study not specific for colistin resistance, only mortality data availble for the two groups	study not specific for colistin resistance, only mortality data availble for the two groups	study not specific for colistin resistance, only mortality data availble for the two groups	study not specific for colistin resistance, only mortality data availble for the two groups	mortality in hospital			
	4	Rojas, 2016	Score		Mix	31 cases 215 controls	-	+	++	++	++	++	++	-	-	++	++	Mort. rate cases=51% Mort. rate controls=39% aHR=3.48; 95%CI(1.77-6.57)	Median(IQR) Cases=8(5-12) Controls=13(7-26)
			Comments	Prospective nested cohort study	HCA: 32% cases, 49% controls	all patients were also CRKP						not matched but controlled in univariate analyese	not matched but controlled for main comorbidities and Charlson's index			30 days			

Sample size
N refers to total number of cases and controls **(no score)**
Calculation *a priori:* ++ if performed (e.g. based on expected prevalence or on litterature data) or the study refers to large national surveys; - if not performed

Study type
Study type: ++ matched cohort or case-control studies; + prospective cohort or case-control studies; - restrospective database studies

Infection type
HCA: health-care associated; **CA:** community associated; **Mix:** health-care and community associated together

Representativeness
Geographical: ++ if the study was conducted in an EU/EEA setting; + if the study was conducted in high-income country(ref. World bank); - if the study was not conducted in an EU/EEA or high-income country setting
Demographical: ++ if the study population represents all age groups; + if the study population represents selected age-specific groups (e.g. elderly, infants); - if the study population represents a selected sex category and age-specific groups
Clinical: ++ if the study population represents the overall hospital population; + if the study population partially represents the hospital population; - if the study population represents a disease-specific population (e.g. liver transplanted patients, cancer patients, etc..)
Exlusion/inclusion criteria: ++ if the same exclusion/inclusion criteria were adopted for both cases and controls; + if some (but not all) exclusion/inclusion criteria were adopted for both cases and controls; - if exclusion/inclusion criteria were different in cases and controls

Matching or controlling for confounders
Demographics: ++ if performed for age and sex; + if performed only for age or only for sex; - if not performed at all
Underlying disease: ++ if performed for two or more criteria (e.g. comorbidity score, McCabe score, severity index, n. comorbidities, allergy, immunosuppression status, prior hospitalisation etc..); + if performed for only one criteria; - if not performed at all
Infection site: ++ if performed ; - if not performed
Hospital and unit/ward: ++ if same hospital and same unit/ward; + if same hospital but different unit/ward; - if not same hospital and/or not same unit/ward
Follow-up: ++ if ≥ 28 days; - if < 28 days

Risk difference
Statistically significant outcome results: ++ if results and 95%CI are statistically significant; - if results and 95%CI are not statistically significant
Case fatality and LOS refer to risk difference estimates in cases and controls **(no score)**

Scoring table

++	Matches completely/is completely fulfilled
+	Matches incompletely but sufficiently/is only partly but sufficiently fulfilled
-	Does not match or matches insufficiently/is insufficiently fulfilled
c.b.e.	Cannot be evaluated

[illegible]

Checklist for the evaluation of the most relevant studies for building antimicrobial resistance (AMR) outcome trees, healthcare-associated infection (HAI) attributable case fatality and attributable length-of-stay (LOS)

	Study			Study type	Infection type	Sample size		Representativeness			Matching or controlling for confounders							Risk difference	
						N (cases;controls)	calculation *a priori*	Geographical	Demographical	Clinical	Exclusion/inclusion criteria	Demographics	Underlying disease	Infection site	Hospital and unit/ward	Follow-up	Statistically significat outcome results	Case fatality	LOS
	Nr.	First author, year			HCA; CA; Mix; c.b.e		□ ++; □ -; □ c.b.e	□ ++; +; -; □ c.b.e	□ ++; □ +; □ -; □ c.b.e	□ ++; -; □ c.b.e	□ ++; □ +; □ -; □ c.b.e	□ ++; □ +; □ -; □ c.b.e	□ ++; □ +; □ -; □ c.b.e	□ ++; □ -; □ c.b.e	□ ++; □ +; □ -; □ c.b.e	□ ++; -; □ c.b.e	□ ++; □ -; □ c.b.e	%; RR; OR; aHR; etc	(days)
BSI<br>R vs NI	1	de Kraker, 2011	Score	++	Mix	C1) 111 resistant<br>204 NI controls for resistant<br>C2) 1110 sensitive<br>2084 NI controls for sensitive	-	++	++	++	++	++	-	-	-	++	++	a) C1: Mort. resist=32%<br>Mort. control=6%<br>OR=4.6, 95%CI(1.7–12.3)<br>C2: Mort. sensit=17%<br>Mort. control=7%<br>OR=1.9; 95%CI(1.4–2.5)<br>OR R vs S= 2.5; 95%CI(0.9–6.8)<br>b) C1: Mort. resist=36%<br>Mort. control=5%<br>HR=5.7; 95%CI(2.5–13.0)<br>C2:Mort. sensit=17%<br>Mort. control=7%<br>HR=2.0; 95%CI(1.5–2.5)<br>HR R vs S=2.9; 95%CI(1.2–6.9)	median (IQR)<br>C1: resist=12(6-25)<br>control=6(3-16)<br>C2: sensit=10(6-17)<br>control=7 (4–14)
			Comments	Prospective parallel matched double cohort	hospital onset of BSI:<br>57% resistant<br>28% sensitive	the study considered the patients as two cohorts, C1=R vs NI and C2=S vs NI						not matched but controlled in the univariate analyses.<br>In the sensitive-control groups the age was higher in the cases		all BSI		a)30 days<br>b)hospitaliz		a)30 days<br>b)hospitaliz	
BSI<br>R vs S	1	Anunatsiri, 2012	Score	-	Mix	32 cases<br>113 controls	-	-	++	++	++	++	++	-	+	c.b.e	++	Mort. cases=29%<br>Mort. controls= 11.5%	prior bacteremia<br>median (range)<br>cases=4 (0-66)<br>controls=0 (0-82)
			Comments	Retrospective case-control study	HCA: 75% cases, 26.5% controls<br>CA: 25% cases, 73.5% controls				>15 years			not matched but controlled in univariate analyses	not matched but controlled in univariate analyses for main diseases and APACHE II score	all BSI		mortality in hospital			
	2	Apisarnthanarak, 2008	Score	-	CA	24 cases<br>108 controls*	-	-	++	++	++	c.b.e	c.b.e	++	++	c.b.e	c.b.e	Mort. cases=8%<br>Mort. controls= 15%*	median (range)<br>cases=8 [illegible]<br>[illegible]6(3–15)
			Comments	Retrospective case-control study	all cases had community onset BSI	*controls had EC or KP the study included EC and KP but it was possible to have the mortality only for EC cases						the study results included EC and KP	the study results included EC and KP			mortality in hospital	the study results included EC and KP	*controls had EC or KP	for cases and controls with EC or KP
	3	Cornejo-Juarez, 2012	Score	-	HCA	88 cases<br>88 controls	-	-	-	-	++	++	-	-	++	++	c.b.e	a)Mort. cases=38.6%<br>Mort. controls= 21.6%<br>b)Mort. cases=19.3%<br>Mort. controls= 25%	difficult to interpret
			Comments	Retrospective case-control study		the study started with 100 patients for each group, but 12 from each group were lost at follow-up				all patients had hematological malignacies		not matched but controlled in univariate analyses		all BSI		60 days	the results were presented separately for appropriate and unappropriate therapy. Here we pooled them.	a) death related to infection<br>b) death not related to infection	
	4	Courpon-Claudinon, 2011	Score	+	Mix	39 cases<br>1012 controls	-	++	++	++	++	+	-	-	-	c.b.e	++	Mort. cases=30.8%<br>Mort. controls= 12.3%	no data
			Comments	Prospective multi-centre study	HCA: 23.1% cases, 19.1% controls<br>CA: 76.9% cases, 80.9% controls							not matched but controlled and age is not signficantly different, gender does		all BSI		mortality in hospital			
	5	Ortega, 2009	Score	+	Mix	211 cases<br>3247 controls	-	++	++	++	++	c.b.e	c.b.e	-	+	++	c.b.e	Mort. cases=15.6%<br>Mort. controls= 7.6%	no data
			Comments	Prospective study	cases: 61% HCA, 39% CA<br>controls: no specific info							data on the ESBL-EC available only on supplementary tables which are inaccessable	data on the ESBL-EC available only on supplementary tables which are inaccessable	all BSI		a) 7days<br>b) 30days	data on the ESBL-EC available only on supplementary tables which are inaccessable		
	6	Trecarichi, 2009	Score	-	Mix	26 cases<br>36 controls	-	++	++	-	++	+	-			++	++	Mort. cases=42.3%<br>Mort. controls= 5.6%<br>OR=8.84; 95%CI(1.48-52.91)	no data
			Comments	Retrospective cohort	HCA: 96.1% cases, 75% controls<br>CA: 3.8% cases, 25% controls					all patients had hematological malignacies		not matched but controlled and gende is not signficantly different, age is higher in cases	different type of hematological melignacies	all BSI		30 days	OR		
	7	Hsieh, 2010	Score	-	CA	19 cases<br>385 controls	-	+	++	++	++	++	++	-	+	++	-	Mort. cases=21.1%<br>Mort. controls= 12.2%	mean (±SD)<br>cases=15.5(±8.5)<br>controls=6.1(±6.9)
			Comments	Retrospective cohort	community onset BSI							not matched but controlled in univariate analyses	not matched but controlled in univariate analyses for main diseases	all BSI		30 days			
	8	Tumbarello, 2010	Score	-	Mix	37 cases<br>97 controls	-	++	++	++	++	++	-	-	+	-	++	Mort. cases=29.7%<br>Mort. controls= 6.1%<br>OR=4.43; 95%CI(2.46-7.95)	mean (±SD)<br>cases=20(±17)<br>controls=13(±9)
			Comments	Retrospective cohort	HCA: 91.8% cases, 69% controls<br>CA: 8.2% cases, 30.9% controls							not matched but controlled in univariate analyses	not matched but controlled in univariate analyses for APACHE score. There are differences in solid tumors, more in controls	all BSI		21 days	OR		
	9	Gudiol, 2010	Score	+	Mix	17 cases<br>118 controls	-	++	++	-	++	+	++	-	+	++*	-	a)Mort. cases=12%<br>Mort. controls= 8%<br>b)Mort. cases=35%<br>Mort. controls=19%	no data
			Comments	Prospective cohort	HCA: 94% cases, 88% controls					hospitalized patients with cancer or hematological malignacies		not matched but controlled and age is not signficantly different, gender does	not matched but controlled for additional comorbidities	all BSI		a) within 7days<br>b) within 30days		a) within 7days<br>b) within 30days	
	10	Ho, 2002	Score	-	Mix	50 cases<br>100 controls	-	+	++	++	++	++	-	-	+	++	++	Mort. cases=18%<br>Mort. controls= 7%	no data
			Comments	Retrospective case-control study	HCA: 80% cases, 22.2% controls									all BSI		within 30 days			
	11	Rodriguez-Bano, 2010	Score	+	CA	95 cases<br>188 controls	-	++	++	++	++	++	-	-	-	-	++	Mort. cases=17%<br>Mort. controls= 8%	no data
			Comments	Prospective cohort	community onset BSI<br>HCA: 76% cases. 53% controls	patients with community onset BSI				patients with community onset BSI		not matched but controlled in univariate analyses		all BSI	patients in different hospitals but matched for hospital	14 days			
	12	Kang, 2010	Score	-	CA	82 case<br>783 controls	-	+	++	++	++	+	-	-	-	++	++	Mort. cases=15%<br>Mort. controls= 7.6%<br>OR=2.99; 95%CI(1.01–8.84)	no data
			Comments	Retrospective cohort	community onset BSI<br>HCA: 36.6% cases. 15.7% controls	patients with community onset BSI				patients with community onset BSI		not matched but controlled and age is not signficantly different, gender does		all BSI		30 days	OR		
	13	Garcia-Hernandez, 2010	Score	c.b.e	Mix	34 cases<br>119 controls	-	++	++	++	++	++	-	-	+	-	++	a)Mort. cases=35.5%<br>Mort. controls= 16.8%<br>b)Mort. cases=[illegible] Mort controls= 9.2%	mean (±SD)<br>cases=24.3(±22)<br>controls=20.9(±26.7)
			Comments	Retrospective and prospective cohort study	HCA: 47.1% cases, 44.5% controls<br>CA: 52.9% cases, 55.5% controls							not matched but controlled in univariate analyses		all BSI		7 days		a) within 72h<br>b)within 7days	
	14	Denis, 2015	Score	-	Mix	41 cases<br>41 controls	-	++	++	++	++	++	-	-	+	++	-	Mort. cases=30%<br>Mort. controls= 27%<br>OR=1.23; 95%CI(0.36–4.23)	median (range)<br>cases=15 (10-21)<br>controls=11 (7-17)
			Comments	Retrospective study	HCA: 83% cases, 71% controls							not matched but controlled in univariate analyses	not matched but controlled in univariate analyses for APACHE II score. There are differences in hematological malignacies, more in cases	all BSI		30 days			
	15	Ha, 2013	Score	-	Mix	95 cases<br>255 controls	-	+	++	-	++	+	-	-	+	++	++	Mort. cases=22.1%<br>Mort. controls= 12.2%<br>OR = 3.01; 95%CI(1.45–6.28)	no data
			Comments	Retrospective cohort study	HCA: 85.3% cases, 66.3% controls<br>CA: 85.3% cases, 33.7% controls					patients with cancer		not matched but controlled and gende is not signficantly different, age is higher in cases		all BSI		30 days			
	16	Kaya, 2013	Score	-	Mix	44 cases<br>69 controls	-	-	++	-	++	++	-	-	+	c.b.e	-	Mort. cases=36.4%<br>Mort. controls= 27.5%	no data
			Comments	Retrospective case-control study	Cases: 51.4% hospital onset, 38.6% community onset					patients with systemic inflammatory response syndrome		not matched but controlled in univariate analyses		all BSI		mortality in hospital			
	17	Kim, 2013	Score	-	HCA	15 cases<br>72 controls	-	+	++	-	++	c.b.e	c.b.e	-	++	++	c.b.e	Mort. cases=6.7%<br>Mort. controls= 5.5%	no data
			Comments	Retrospective study						patients who had hematological diseases and neutropenic fever		data for EC not available because pooled with KP cases	data for EC not available because pooled with KP cases	all BSI		30 days	data for EC not available because pooled with KP cases	mortality data extracted for EC	
	18	Lambert, 2011	Score	-	Mix	a)42 cases<br>218 controls<br>b)58 cases<br>464 controls	-	++	++	+	++	c.b.e	-	++	++	c.b.e	-	a) Mort. cases=50%<br>Mort. controls=40%<br>aHR=1.3; 95%CI(0.8–2.2)<br>b) Mort. cases=47%<br>Mort. controls=29%<br>aHR=1.4; 95%CI (0.9–2.3)	from infection to discharge*<br>median (IQR)<br>a)cases=16(4-24)<br>controls=9(5-20) b)cases=18(11-29)<br>controls=17(9-29)
			Comments	Retrospective cohort study	no specific information available	a)BSI<br>b)pneumonia				ICU patients					all ICU patients	mortality in hospital		a)BSI<br>b)pneumonia	*for those disacherged alive<br>a)BSI<br>b)pneumonia
	19	Leistner, 2014 a	Score	-	Mix	115 cases<br>983 controls	-	++	++	++	++	-	-	-	+	c.b.e	-	Mort. cases=26%<br>Mort. controls=18%	median (range)<br>cases=27 (12-53)<br>controls=15 (8-32)
			Comments	Retrospective cohort study	HCA: 58% cases, 38% controls									all BSI		mortality in hospital			
	20	Leistner, 2014 b	Score	-	Mix	178 cases<br>1322 controls	-	++	++	++	++	c.b.e	c.b.e	-	+	c.b.e	c.b.e	Mort. cases=25.3%<br>Mort. controls=17.6%	no specific data
			Comments	Retrospective cohort study	no specific information available							no information becasue data are pooled with KP infections	no information becasue data are pooled with KP infections	all BSI		mortality in hospital	no information becasue data are pooled with KP infections		
	21	Martellius, 2016	Score	-	HCA	182 cases<br>2035 controls	-	++	++	++	++	++	-	-	-	++	-	a)Mort. cases=7.7%<br>Mort. controls= 5.9%<br>b)Mort. cases=14.3%<br>Mort. controls=11.9%	no data
			Comments	Retrospective study								not matched but controlled in univariate analyses		all BSI		a) 7days<br>b) 28days		a) 7 days<br>b) 28 days	
	22	Park, 2011	Score	-	Mix	50 cases<br>100 controls	-	+	++	++	++	++	-	-	+	++	-	a)Mort. cases=8%<br>Mort. controls= 6%<br>b)Mort. cases=18%<br>Mort. controls=8%<br>OR=6.4; 95%CI(0.3–145.5)	no data
			Comments	Retrospective cohort study	HCA	community onset BSI						not matched but controlled in univariate analyses		all BSI		a) 7days<br>b) 30days		a) 7days<br>b) 30days	
			Score	-	HCA	11 cases<br>77 controls	-	+	++	-	++	++	-	-	+	c.b.e	++	Mort. cases=27.3%<br>Mort. controls= 3.9%	no data

	23	Yip, 2006	Comments	Retrospective study	not expicitly said in the article, but very specific patients					patients undergoing continuous ambulatory peritoneal dialysis		not matched but controlled in univariate analyses	continuous ambulatory peritoneal dialysis	all BSI		mortality in hospital			
	24	Van Aken, 2014	Score	-	Mix	70 cases<br>140 controls	-	++	++	++	++	+	++	-	-	-	-	Mort. cases=9%<br>Mort. controls= 7%	median±SD<br>cases=11±12<br>controls=9±10
			Comments	Retrospective case-control study	HCA: 31.4% cases, 31.4% controls<br>CA: 68.8% cases, 68.6% controls							not matched but controlled and gende is not signficantly different, age is higher in controls	not matched but controlled for Charlson's comorbidity index	all BSI		14 days			
	25	Melzer, 2007	Score	-	Mix	46 cases<br>308 controls	-	++	++	++	++	-	-	-	+	++	++	Mort. cases=60.9%<br>Mort. controls= 23.7%<br>OR=3.57; 95%CI(1.48-8.60)	median<br>cases=9<br>controls=12
			Comments	Retrospective cohort	HCA: 65.2% cases, 26.6% controls<br>CA: 34.8% cases, 73.4% controls									all BSI		30 days			
UTI<br>R vs S	1	Al-Otaibi, 2013	Score	-	Mix	113 cases<br>226 controls	-	+	++	+	++	-	-	-	++	c.b.e	++	Mort. cases=8.9%<br>Mort. controls= 0.9%	no data
			Comments	Retrospective case-control study	Cases: 43% HCA, 57% CA				children and aduls together	patients from bacteriology unit				all UTI		mortality in hospital			
	2	Ena, 2006	Score	-	Mix	61 cases<br>61 controls	-	++	++	++	++	+	++	-	+	c.b.e	-	Mort. cases=8%<br>Mort. controls= 8%*	no data
			Comments	Retrospective cohort	HCA: 54% cases, 42% controls<br>CA: 46% cases, 59% controls							not matched but controlled in univariate analyses for gender. Age was different, cases were older	not matched but controlled in univariate analyses for main disease and Charlson's morbidity score.	all UTI		mortality in hospital		*deaths in both groups were not attributable, not occurred during the phase of active infection or while the patient was receiving antibiotic therapy	
	3	Suankratay, 2008	Score	+	Mix	35 cases<br>71 controls*	-	-	+	-	++	c.b.e	-	-	+	-	-	Mort. cases=2.9%<br>Mort. controls= 0%*	no data
			Comments	Prospective cohort	no info on proportion HCA/CA	*controls were EC, KP, PM and was not possible to separate them			patients were only women >15 years	patients with acute pyelonephritis		cases and controls pooled with patients with other infections, KP, PM		all UTI		14 days		*controls were EC, KP, PM and was not possible to separate them	
	4	Esteve-Palau, 2015	Score	++	Mix	60 cases<br>60 controls	++	++	++	-	++	++	-	-	+	++	++	Mort. cases=10%<br>Mort. controls= 7%<br>OR=8.011; 95%CI(2.48-25.92)*	mean (±SD)<br>cases=11.6(±1.5)<br>controls=7.5(±0.8)
			Comments	Matched cohort study	HCA: 60% cases, 33% controls<br>CA: 40% cases, 67% controls					patients with symtomatic UTI				all UTI		within 30 days	OR	*Risk of failure within 7 days (R vs S)	
	5	Park, 2015	Score	-	CA	75 cases<br>225 controls	-	+	++	-	++	+	-	-	+	-	c.b.e	Mort. cases=1.3%<br>Mort. controls= 1.8%	no data
			Comments	Retrospective case-control study					>15 years	patients with acute pyelonephritis		not matched but controlled in univariate analyses for gender. Age was different, cases were older		all UTI		7 days	no information on mortality because the outcome as aclinical failure included relapses.		
Rectal swab<br>R vs NI	1	Arnan, 2011	Score	+	Mix	63 cases<br>154controls	-	++	++	-	++	++	-	-	-	c.b.e	-	a)Mort. cases=1.6%<br>Mort. controls= 0%<br>b)Mort. cases=10%<br>Mort. controls= 11%	mean (±SD)<br>cases=30.15 (±13.70)<br>controls=29.05 (±12.11)
			Comments	Prospective multicentre cohort study	HCA: 75% cases, 81% controls					patients with hematological malignacies		not matched but controlled in univariate analyses	not matched but all patients had hematological malignacies	all rectal swabs		a) 7days<br>b) hospitaliz		a) 7days<br>b) hospitaliz	
Mixed inf. site<br>R vs NI	1	Nicolas-Chanoine, 2012	Score	+	Mix	152 resistant<br>152 sensitive<br>152 NI	++	++	++	++	++	++	-	-	-	c.b.e	++	Mort. resist=12%<br>Mort. sensit=7%<br>Mort. NI=5%	no data
			Comments	Prospective case-control-control study	no info on proportion HCA/CA							not matched but controlled in univariate analyses				mortality in hospital	only mortality of resistant vs NI		
	2	Hayakawa, 2013	Score	-	Mix	319 resistant<br>58 sensitive<br>319 NI	-	+	++	++	++	-	-	-	++	++	++*	a) Mort. resist=5.7%<br>Mort. sensit=3.5%<br>Mort. NI=3.8%<br>OR R vs S= 1.66; 95%CI(0.37–7.34)<br>OR R vs NI=2.33; 95%CI(0.9–6.07)<br>b) Mort.resist=13.7%<br>Mort. sensit=10.2%<br>Mort NI=7 6%	median (IQR)<br>resist=7(4-12)<br>sensit=7(3-20)<br>NI=4(2-6)
			Comments	Retrospective case-case-control study	no info on proportion HCA/CA										different hospitals but cases were matched for hospital unit	a) hospitaliz<br>b) 3 months	*only 3 months OR R vs NI	a) hospitaliz<br>b) 3 months	
Mixed inf. site<br>R vs S	1	Pena, 2008	Score	++	c.b.e	100 cases<br>100 controls	-	++	++	++	++	c.b.e.	c.b.e.	++	+	++	++	a)Mort. cases=16%<br>Mort. controls= 6%<br>b)Mort. cases=25%<br>Mort. controls= 11%	no data
			Comments	Retrospective matched cohort study	no information							information not specific for 2 groups, but for died and survived	information not specific for 2 groups, but for died and survived			a) 7 days<br>b) 30 days	both mortalites are significant	a) 7 days<br>b) 30 days	information not specific for 2 groups, but for died and survived
	2	Kang, 2011	Score	-	CA	108 cases<br>100 controls	-	+	++	++	++	++	-	++	++	++	-	Mort. cases=7.4%<br>Mort. controls= 2%	no data
			Comments	Retrospective case-control study	community onset infection<br>HCA: 51.9% cases, 19.4% controls	community onset infection			>15 years			not matched but controlled and age is not signficantly different, gender does				30 days			
	3	Maslikowska, 2016	Score	-	Mix	61 cases<br>49 controls	-	+	++	++	++	++*	++*	++*	++*	-	++	a)Mort. cases=15%<br>Mort. controls= 2%<br>b)Mort. cases=5%<br>Mort. controls= 2%	not specific for EC
			Comments	Retrospective case-control study	no info on proportion HCA/CA							*original study reporting EC or KP infections	not matched but controlled for Charlson comorbidity index and APACHE II score<br>*original study reporting EC or KP infections	*original study reporting EC or KP infections	*original study reporting EC or KP infections	14 days	only all cause mortality	data extracted only for EC<br>a) all cause mortality<br>b) attributable mortality: infection cause of death	

Sample size
N refers to total number of cases and controls **(no score)**
Calculation *a priori*: ++ if performed (e.g. based on expected prevalence or on litterature data) or the study refers to large national surveys; - if not performed

Study type — **Study type:** ++ matched cohort or case-control studies; + prospective cohort or case-control studies; - restrospective database studies
Infection type — **HCA:** health-care associated; **CA:** community associated; **Mix:** health-care and community associated together

Representativeness
Geographical: ++ if the study was conducted in an EU/EEA setting; + if the study was conducted in high-income country(ref. World bank); - if the study was not conducted in an EU/EEA or high-income country setting
Demographical: ++ if the study population represents all age groups; + if the study population represents selected age-specific groups (e.g. elderly, infants); - if the study population represents a selected sex category and age-specific groups
Clinical: ++ if the study population represents the overall hospital population; + if the study population partially represents the hospital population; - if the study population represents a disease-specific population (e.g. liver transplanted patients, cancer patients, etc..)
Exlusion/inclusion criteria: ++ if the same exclusion/inclusion criteria were adopted for both cases and controls; + if some (but not all) exclusion/inclusion criteria were adopted for both cases and controls; - if exclusion/inclusion criteria were different in cases and controls

confounders
Demographics: ++ if performed for age and sex; + if performed only for age or only for sex; - if not performed at all
Matching or controlling for Underlying disease: ++ if performed for two or more criteria (e.g. comorbidity score, McCabe score, severity index, n. comorbidities, allergy, immunosuppression status, prior hospitalisation etc..); + if performed for only one criteria; - if not performed at all
Infection site: ++ if performed ; - if not performed
Hospital and unit/ward: ++ if same hospital and same unit/ward; + if same hospital but different unit/ward; - if not same hospital and/or not same unit/ward
Follow-up: ++ if ≥ 28 days; - if < 28 days

Risk difference
Statistically significant outcome results: ++ if results and 95%CI are statistically significant; - if results and 95%CI are not statistically significant
Case fatality and LOS refer to risk difference estimates in cases and controls **(no score)**

Scoring table

++	Matches completely/is completely fulfilled
+	Matches incompletely but sufficiently/is only partly but sufficiently fulfilled
-	Does not match or matches insufficiently/is insufficiently fulfilled
c.b.e.	Cannot be evaluated

CREC **Checklist for the evaluation of the most relevant studies for building antimicrobial resistance (AMR) outcome trees, healthcare-associated infection (HAI) attributable case fatality and attributable length-of-stay (LOS)**

	Study			Study type	Infection type	Sample size		Representativeness			Matching or controlling for confounders						Risk difference		
						N (cases;controls)	calculation *a priori*	Geographical	Demographical	Clinical	Exclusion/inclusion criteria	Demographics	Underlying disease	Infection site	Hospital and unit/ward	Follow-up	Statistically significat outcome results	Case fatality	LOS
	Nr.	First author, year			HCA; CA; Mix; c.b.e		□ ++; □ -; □ c.b.e	□ ++; +; -; □ c.b.e	□ ++; □ +; □ -; □ c.b.e	□ ++; -; □ c.b.e	□ ++; □ +; □ -; □ c.b.e	□ ++; □ +; □ -; □ c.b.e	□ ++; □ +; □ -; □ c.b.e	□ ++; □ -; □ c.b.e	□ ++; □ +; □ -; □ c.b.e	□ ++; -; □ c.b.e	□ ++; □ -; □ c.b.e	%; RR; OR; aHR; etc	(days)
BSI<br>R vs S	1	Chang, 2011	Score	++	Mix	17 cases<br>34 controls	-	+	++	++	++	++	-	-	+	++	++*	a)Mort.cases=47.06%<br>Mort.controls=38.24%<br>b)Mort.cases=70.59%<br>Mort.controls=47.06%<br>c)Mort.cases=94.12%<br>Mort.controls=50%	mean±SD (min-max)<br>cases=68.4± 56.4 (7-161) controls=35.8±34.5 (4-141)
			Comments	Matched case-control study	"mostly HCA" but not specified	Cases are non-susceptible (i.e. resistant and intermediate MIC values) but was not possible to differentiate them								all BSI		a) ≤14 days<br>b) ≤ 28 days<br>c) hospitaliz	*only mortality during hospitalization © was significantly higher in cases compared to controls	a) ≤14 days<br>b) ≤ 28 days<br>c) hospitaliz	
Mixed inf. site<br>R vs NI	1	Epstein, 2014	Score	+	HCA	8 cases<br>27 controls	-	+	++	+	++	++	-	-	++	c.b.e.	c.b.e	Mort.cases=25%*<br>Mort.controls=0%	median<br>cases=8.5<br>controls=25.0<br>mean(SD)[range]<br>cases=13.3 (12.0) [1-37]<br>controls=30.2 (13.2) [14-65]
			Comments	Prospective case-control study	cases exposed to duodenoscopes, considered the source of infection					cases are exposed to duodenoscopes and controls are from rehabilitation ward		not matched but controlled in the univariate analyses				hospitalization	no information	* death not related to CREC	
Mixed inf. site<br>R vs S	1	Ahn, 2014	Score	++	Mix	57 cases<br>114 controls	-	+	++	++	++	++	-	++	+	++	-	Mort.cases=14.8%<br>Mort.controls=10.2%	mean±SD<br>cases=26.6±37.9<br>controls=13.1±41.3
			Comments	Matched case-controls study	HCA: 84.2% cases, 71.9% controls<br>CA: 15.8% cases, 28.1% controls							not matched but controlled in the univariate analyses				28 days			

Sample size
N refers to total number of cases and controls **(no score)**
Calculation *a priori:* ++ if performed (e.g. based on expected prevalence or on litterature data) or the study refers to large national surveys; - if not performed

Study type
Study type: ++ matched cohort or case-control studies; + prospective cohort or case-control studies; - restrospective database studies

Infection type
HCA: health-care associated; **CA:** community associated; **Mix:** health-care and community associated together

Representativeness
Geographical: ++ if the study was conducted in an EU/EEA setting; + if the study was conducted in high-income country(ref. World bank); - if the study was not conducted in an EU/EEA or high-income country setting
Demographical: ++ if the study population represents all age groups; + if the study population represents selected age-specific groups (e.g. elderly, infants); - if the study population represents a selected sex category and age-specific groups
Clinical: ++ if the study population represents the overall hospital population; + if the study population partially represents the hospital population; - if the study population represents a disease-specific population (e.g. liver transplanted patients, cancer patients, etc..) **Exlusion/inclusion criteria**: ++ if the same exclusion/inclusion criteria were adopted for both cases and controls; + if some (but not all) exclusion/inclusion criteria were adopted for both cases and controls; - if exclusion/inclusion criteria were different in cases and controls **Demographics:** ++ if performed for age and sex; + if performed only for age or only for sex; - if not performed at all

Matching or controlling for confounders
Underlying disease: ++ if performed for two or more criteria (e.g. comorbidity score, McCabe score, severity index, n. comorbidities, allergy, immunosuppression status, prior hospitalisation etc..); + if performed for only one criteria; - if not performed at all
Infection site: ++ if performed ; - if not performed
Hospital and unit/ward: ++ if same hospital and same unit/ward; + if same hospital but different unit/ward; - if not same hospital and/or not same unit/ward
Follow-up: ++ if ≥ 28 days; - if < 28 days

Risk difference
Statistically significant outcome results: ++ if results and 95%CI are statistically significant; - if results and 95%CI are not statistically significant
Case fatality and LOS refer to risk difference estimates in cases and controls **(no score)**

Scoring table

++	Matches completely/is completely fulfilled
+	Matches incompletely but sufficiently/is only partly but sufficiently fulfilled
-	Does not match or matches insufficiently/is insufficiently fulfilled
c.b.e.	Cannot be evaluated

MDRACI

C[illegible]list for the evaluation of the most relevant studies for building antimicrobial resistance (AMR) outcome trees, healthcare-associated infection (HAI) attributable case fatality and attributable length-of-stay (LOS)

	Study			Study type	Infection type	Sample size		Representativeness			Matching or controlling for confounders						Risk difference		
						N (cases;controls)	calculation *a priori*	Geographical	Demographical	Clinical	Exclusion/inclusion criteria	Demographics	Underlying disease	Infection site	Hospital and unit/ward	Follow-up	Statistically significat outcome results	Case fatality	LOS
	Nr.	First author, year			HCA; CA; Mix; c.b.e		□ ++; □ -; □ c.b.e	□ ++; +; -; □ c.b.e	□ ++; □ +; □ -; □ c.b.e	□ ++; -; □ c.b.e	□ ++; □ +; □ -; □ c.b.e	□ ++; □ +; □ -; □ c.b.e	□ ++; □ +; □ -; □ c.b.e	□ ++; □ -; □ c.b.e	□ ++; □ +; □ -; □ c.b.e	□ ++; -; □ c.b.e	□ ++; □ -; □ c.b.e	%; RR; OR; aHR; etc	(days)
BSI<br>R vs NI	1	Al Jarousha, 2009	Score	+	HCA	40 cases<br>100 controls	-	-	+	-	++	++	-	-	-	c.b.e	++	Mort. cases=37.5%<br>Mort. controls=12%	no specific data
			Comments	Prospective case-control study					neonates in ICU	NICU patients		matched for age and controlled in the univariate analyses for gender		all BSI	2 hospitals	mortality in hospital	test on the mortality rates		
	2	Gulen, 2015	Score	-	HCA	41 cases<br>45 controls*	-	-	++	+	++	++	-	-	++	++	c.b.e	a) Mort. cases=30%<br>b) Mort. cases=52.4%<br>c)Mort. Cases=53.7%<br>Attribut. mort.=24.4%	before BSI<br>mean±SD<br>cases=25.49±21.47<br>controls=22.80±19.28
			Comments	Retrospective cohort study		*patients had other gram-negative infections				ICU patients		not matched but controlled in the univariate analyses		all BSI	ICU	3 mortalities measured		a) 14 days<br>b) 28 days<br>c)hospitaliz	
BSI<br>R vs S	1	Anunnatsiri, 2011	Score	-	Mix	24 cases<br>25 controls	-	-	++	++	++	++	-	-	+	c.b.e	++	Mort. cases=91.7%<br>Mort. controls=48%<br>OR=11.92; 95%CI(2.30-61.83)	before BSI<br>median (range)<br>cases=21.5(4-161)<br>cotnrols=14(2-86)
			Comments	Retrospective study	cases were 100% HCA, controls were 76% HCA and 24% CA							not matched but controlled in the univariate analyses		all BSI		mortality in hospital	both, the mortality rates and the OR		
	2	Fitzpatrick, 2015	Score	-	Mix	92 cases<br>24 controls	-	+	++	++	++	++	-	-	+	++	++	a) Mort. cases=36%<br>Mort. controls=8%<br>b) Mort. cases=44%<br>Mort. controls=8%	postculture<br>median(range)<br>cases=11.5(2-69)<br>controls=6(2-38)
			Comments	Retrospective cohort study	63 % cases and 39% controls were HCA, no information about the rest							not matched but controlled in the univariate analyses		all BSI		a) 14 days<br>b) 30 days	mortality signficantly higher for resistant both at 14 and 30 days	a) 14 days<br>b) 30 days	
	3	Guo, 2016	Score	-	c.b.e	64 cases<br>23 controls	-	-	++	++	++	-	-	-	+	++	++	Mort. cases=44.8%<br>Mort. controls=4.3%<br>OR=26.3; 95%CI(3.0-231.4)	in ICU<br>mean<br>cases=51<br>controls=5
			Comments	Retrospective study	no information available in the article							no info on eventual univariate analyses		all BSI		30 days	significant OR, no test on mortality rates		
	4	Lee, 2007	Score	++	HCA	46 cases<br>46 controls	-	+	++	++	++	++	++	-	+	c.b.e	++	a) Mort. cases=47.8%<br>Mort. controls=39.1%<br>b) Mort. cases=34.8%<br>Mort. controls=13.0%	mean±SD<br>cases=54.2±42.8<br>controls=34.1±30.5
			Comments	Retrospective matched cohort study										all BSI		hospitalization	only the difference in sepsis related mortality was significant	a) crude mortality<br>b) sepsis related mortality	
	5	Lee, 2010	Score	-	MIx	147 cases<br>144 controls	-	+	++	++	++	c.b.e	c.b.e	-	+	++	++	Mort. cases=32%<br>Mort. controls=8,3%<br>OR=7.46; 95%CI(3.78-14.70)	no specific data
			Comments	Retrospective study	not possible to extract data									all BSI		30 days	OR, no test on mortality		
	6	Smolyako, 2003	Score	+	HCA	51 cases<br>43 controls	-	+	++	++	++	+	-		+	c.b.e	++	Mort. cases=29.4%<br>Mort. controls=11.6%<br>OR=4	mean±SD<br>cases=37.7±30<br>controls=24.3±18.3
			Comments	Prospective study								controlled for gen, while age is significantly higher in the control group		all BSI		hospitalization			
Resp. Tract<br>R vs S	1	Cai, 2012	Score	+	HCA	115 cases<br>45 controls	-	-	+	+	++	+	+	-	+	c.b.e	++	Mort. cases=18.26%<br>Mort. controls=4.44%	in ICU<br>mean±SD<br>cases=17.39±7.05<br>controls=14.43±3.92
			Comments	Prospective study					all patients were children	children with pneumonia		controlled in the univariate analyses only for age	univariate analyses for main basic diseases in not significant for any condition	all repsiratory tract infections		hospitalization			
	2	Inchai, 2015	Score	-	HCA	72 cases<br>33 controls	-	-	++	+	++	++	-	-	+	++	-	a)Mort. cases=31.9%<br>Mort. controls=21.2%<br>b)Mort. cases=44.4%<br>Mort. controls=27.3%<br>HR=1.03; 95%CI(0.44–2.45)	no data
			Comments	Retrospective cohort study						patienst in ICU		not matched but controlled in the univariate analyses		all repsiratory tract infections		a) 30 days<br>b) hospitalization		a) 30 days<br>b) hospitalization	
	3	Park, 2006	Score	-	HCA	17 cases<br>30 controls	c.b.e	+	c.b.e	-	c.b.e	-	++	-	+	c.b.e	-	Mort. cases=58.8%<br>Mort. controls=40%	mean±SD<br>cases=42.8±24.0<br>controls=66.2±56.1
			Comments	Retrospective study			full text in korean language, data extracted from abstract and tables in english		full text in korean language, data extracted from abstract and tables in english	patients with pneumonia	full text in korean language, data extracted from abstract and tables in english		not matched, but APACHE II score is not significantly different	all repsiratory tract infections		full text in korean language, data extracted from abstract and tables in english			
Mixed inf. site<br>R vs NI	1	Abbo, 2007	Score	++	c.b.e	118 cases<br>118 controls	-	+	++	++	++	++	-	-	+	c.b.e	++	Mort. cases=36%<br>Mort. controls=21%<br>OR=2.21, 95%CI(1.17–4.16)	median<br>cases=17<br>controls=11
			Comments	Retrospective matched cohort study	no information available in the article							not matched but controlled in the univariate analyses	matching criteria were ward, calendar month of hospit., and duration of hospit. before culture			hospitalization			
	2	Fukuta, 2013	Score		c.b.e	31 cases<br>62 controls	-	+	++	+	++	++	+	-	+	++	-	Mort. cases=41.9%<br>Mort. controls=29%	median(IQR)<br>cases=28 (9-59)<br>controls=10(5-18)
			Comments	Case-control study	no information available in the article					all patients had cancer		not matched but controlled in the univariate analyses	the type of cancer was not the same in all patients			90 days			
	3	Lee, 2016	Score	++	HCA	122 cases<br>122 controls	-	+	++	+	++	++	++	-	++	c.b.e	++	Mort. cases=49.2%<br>Mort. controls=32%<br>OR=3.64; 95%CI(1.80-7.37)	mean±SD<br>cases=40.3±40.6<br>controls=24.6±28
			Comments	Retrospective propensity-matched cohort study	not expicitly said in the article, but very specific patients	all patients were only colonized with AB, not infected				patients in ICU		not matched but controlled in the univariate analyses	not matched but controlled in the univariate analyses			hospitalization			
Mixed inf. site<br>R vs S	1	Brahmi, 2007	Score	+	HCA	29 cases<br>34 controls	-	-	++	+	++	+	++	-	++	c.b.e	++	Mort. cases=67.5%<br>Mort. controls=46.7%	in ICU<br>mean±SD<br>cases=27±17.8<br>controls=32.3±23.9
			Comments	Prospective study						patients in ICU		not matched but controlled in the univariate analyses for age only	not matched but controlled in the univariate analyses			hospitalization			
	2	Daniels, 2008	Score	-	HCA	a)146 cases<br>42 controls<br>b)42 cases<br>42 controls	-	+	++	++	++	++	++	++	++	++	-	a) Mort. cases=15.8%<br>Mort. controls=9.5%<br>b) Mort. cases=14.3%<br>Mort. controls=9.5%	median (IQR)<br>cases=32.5(22-51)<br>controls=26.5(16-43)
			Comments	Retrospective propensity-matched cohort study		a) all patients<br>b) matched pairs				patients in ICU		not matched but controlled in the univariate analyses, both groups	42 pairs matched for propensity score, the whole grops had also no difference in the univariate analyses	not matched but controlled in the univariate analyses, both groups		28 days	none of the mortality rates	a) all patients<br>b) matched pairs	
	3	Lemos, 2011	Score	+	HCA	103 cases<br>62 controls	-	-	++	+	++	++	-	-	-	++	++	a) Mort. cases=31%<br>Mort. controls=9%<br>b) Mort. cases=41%<br>Mort. controls=9%<br>b) RR=1,219;<br>95%CI(0,923–1,611)	median(IQR)<br>pre ICU<br>cases=4(1.2-18)<br>controls=5(1.5-22.3)<br>in ICU<br>cases=15(13-60)<br>controls=13(8-44)
			Comments	Prospective, observational multicentre study						patients in ICU		not matched but controlled in the univariate analyses			ICU of different hospitals	a)14 days<br>b) 30 days	30 days		
	4	Pierri, 2015	Score	++	HCA	14 cases<br>26 controls	-	++	++	+	++	++	++	-	++	c.b.e	-	Mort. cases=57%<br>Mort. controls=46%	mean±SD<br>cases=36±20<br>controls=25±18
			Comments	Retrospective matched cohort study	not expicitly said in the article, but very specific patients					patients in cardiac surgery ICU		not matched but controlled in the univariate analyses	matched for propensity score			hospitalization			
	5	Zilberberg, 2016	Score	-	Mix	1171 cases<br>252 controls	-	+	++	++	++	+	-	++	-	c.b.e	++	Mort. cases=23.7%<br>Mort. controls=12.7%	no data
			Comments	Retrospective cohort study	HCA: 45.9% cases; 42.5% controls<br>CA: 54.1% cases; 57.5% controls							controlled in the univariate analyses only for gender, age older in cases		not matched but controlled in the univariate analyses, were only BSI and resp tract infections		hospitalization			

f

Sample size
N refers to total number of cases and controls **(no score)**
Calculation *a priori*: ++ if performed (e.g. based on expected prevalence or on litterature data) or the study refers to large national surveys; - if not performed

Study type — **Study type:** ++ matched cohort or case-control studies; + prospective cohort or case-control studies; - restrospective database studies
Infection type — **HCA:** health-care associated; **CA:** community associated; **Mix:** health-care and community associated together

Representativeness
Geographical: ++ if the study was conducted in an EU/EEA setting; + if the study was conducted in high-income country(ref. World bank); - if the study was not conducted in an EU/EEA or high-income country setting
Demographical: ++ if the study population represents all age groups; + if the study population represents selected age-specific groups (e.g. elderly, infants); - if the study population represents a selected sex category and age-specific groups
Clinical: ++ if the study population represents the overall hospital population; + if the study population partially represents the hospital population; - if the study population represents a disease-specific population (e.g. liver transplanted patients, cancer patients, etc..)
Exlusion/inclusion criteria: ++ if the same exclusion/inclusion criteria were adopted for both cases and controls; + if some (but not all) exclusion/inclusion criteria were adopted for both cases and controls; - if exclusion/inclusion criteria were different in cases and controls
Demographics: ++ if performed for age and sex; + if performed only for age or only for sex; - if not performed at all

Matching or controlling for confounders
Underlying disease: ++ if performed for two or more criteria (e.g. comorbidity score, McCabe score, severity index, n. comorbidities, allergy, immunosuppression status, prior hospitalisation etc..); + if performed for only one criteria; - if not performed at all
Infection site: ++ if performed ; - if not performed
Hospital and unit/ward: ++ if same hospital and same unit/ward; + if same hospital but different unit/ward; - if not same hospital and/or not same unit/ward
Follow-up: ++ if ≥ 28 days; - if < 28 days

Risk difference
Statistically significant outcome results: ++ if results and 95%CI are statistically significant; - if results and 95%CI are not statistically significant
Case fatality and LOS refer to risk difference estimates in cases and controls **(no score)**

Scoring table

++	Matches completely/is completely fulfilled
+	Matches incompletely but sufficiently/is only partly but sufficiently fulfilled
-	Does not match or matches insufficiently/is insufficiently fulfilled
c.b.e.	Cannot be evaluated

CRACI

Checklist for the evaluation of the most relevant studies for building antimicrobial resistance (AMR) outcome trees, healthcare-associated infection (HAI) attributable case fatality and attributable length-of-stay (LOS)

	Study			Study type	Infection type	Sample size		Representativeness			Matching or controlling for confounders						Statistically significant outcome results	Risk difference	
						N (cases;controls)	calculation *a priori*	Geographical	Demographical	Clinical	Exclusion/inclusion criteria	Demographics	Underlying disease	Infection site	Hospital and unit/ward	Follow-up		Case fatality	LOS
	Nr.	First author, year			HCA; CA; Mix; c.b.e		□ ++; □ -; □ c.b.e	□ ++; +; -; □ c.b.e	□ ++; □ +; □ -; □ c.b.e	□ ++; -; □ c.b.e	□ ++; □ +; □ -; □ c.b.e	□ ++; □ +; □ -; □ c.b.e	□ ++; □ +; □ -; □ c.b.e	□ ++; □ -; □ c.b.e	□ ++; □ +; □ -; □ c.b.e	□ ++; -; □ c.b.e	□ ++; □ -; □ c.b.e	%; RR; OR; aHR; etc	(days)
BSI R vs NI	1	Kim, 2014	Score	-	HCA	19 cases<br>38 controls	-	+	++	-	++	++	+	-	++	c.b.e	++	Mort. rate cases=94.7%<br>Mort.rate controls=8.3%	median (range)<br>cases=35(30–47)<br>controls=29(26.75–34)
			Comments	Retrospective case-control study	not expicitly said in the article, but very specific patients					hemopoietic stem-cell transplantation recipients	hemopoietic stem-cell transplantation recipients	not matched but differences were not dignificant	hemopoietic stem-cell transplantation recipients	all BSI		mortality in hospital	Fisher's exact test on the fatality rates		total admission duration including the trasplantation
	2	Thatrimontrichai, 2013	Score	-	HCA	14 resistant<br>38 sensitive<br>44 controls	-	-	+	+	++	-	+	-	++	-	++	Mort. rate resist=42.9%<br>Mort. rate sensit=13.2%<br>Mort. rate controls=6.8%<br>OR RvsS=5; 95%CI(1.2-20-4)<br>OR RvsNI=10.2; 95%CI(2.1-50)	median(range)<br>resist=34(2-206)<br>sensit=53.5(3-188)<br>controls=24.5(1-102)
			Comments	Case-case-control study	not expicitly said in the article, but very specific patients				neonates	neonates in ICU		not matched but all were neonates	presence in ICU	all BSI	all in NICU	7 days mortlaity	OR for mortality		
BSI R vs S	1	Deris, 2011	Score	-	HCA	15 cases<br>41 controls	-	-	++	++	++	++	-	-	+	c.b.e	_	Mort. rate cases=64.3%<br>Mort.rate controls=40.5%<br>OR (95%CI)=0.38 (0.11-1.36)<br>*Attrib.mort. rate cases=42.9%<br>Attib. mort.rate controls=24.3%<br>OR (95%CI)=2.33(0.64-8.65)	median (IQR)<br>cases=32.3(16.4)<br>controls=32.8(25.0)
			Comments	Cross sectional case-control study								not matched but differences were not dignificant		all BSI		mortality in hospital	neither the all-cause nor the attributable mortality	* within 72h from isolation	
	2	Esterly, 2011	Score	-	c.b.e	37 cases<br>42 controls	-	+	++	++	++	++	+	-	+	c.b.e	a) ++<br>b) -	Mort. rate cases=56.8%<br>Mort.rate controls=23.8%<br>Model1 OR=0.58;95%CI (0.037–9.22)	Prior isolation<br>median (IQR)<br>cases=16(1-30)<br>controls=0(0-9)
			Comments	Retrospective cohort study	no information available in the article							not matched but controlled in the multivariate models	multivariate analysis controlled for underlying conditions	all BSI		mortality in hospital	a) comparison of mortality rates<br>b) OR multivariate analyses(none is significant)		
	3	Huang, 2012	Score	-	c.b.e	62 cases<br>164 controls	-	+	++	++	++	++	++	-	+	-	++	Mort. rate cases=35.5%<br>Mort.rate controls=20.7%<br>OR= 1.03 ; 95%CI(0.48-2.20)	Prior isolation<br>mean±SD<br>cases=35.9±33.6<br>controls=28.2±44
			Comments	Retrospective cohort study	no information available in the article							not matched but differences were not dignificant	not matched, but controlled for Apache II score	all BSI		14 days	Resistance was significant for mortality in a univariate analyses, but not in the multivariate		
	4	Jumalitrat, 2009	Score	-	HCA	67 cases<br>131 controls	-	-	++	++	++	++	++	-	+	-	++	Mort. rate cases=52.2%<br>Mort.rate controls=19.9%<br>aHR=1.7; 95%CI(0.9-2.9)	median ±IQR<br>cases=37±32<br>controls=27±37
			Comments	Retrospective cohort study					>15 years			not matched but controlled in the HR model	controlled for ASA score in aHR	all BSI		mortality in hospital	significant only the difference between the mortality rates		
	5	Kim 2012	Score	-	c.b.e	53 cases<br>42 controls	-	+	++	++	++	++	-	-	+	-	++	Mort. rate cases=49.1%<br>Mort.rate controls=9.5%<br>OR=7.29; 95%CI(1.57-33.8)	Prior isolation<br>mean±SD<br>cases=30.0±33.3<br>controls=30.4±68.3
			Comments	Retrospective study	no information available in the article				>16 years			not matched but controlled in the univariate analyses		all BSI		14 days	significant OR		
	6	Kumar 2014	Score	-	HCA	33 cases<br>32 controls	-	-	+	+	++	-	-	-	++	c.b.e	-	Mort. rate cases=27.3%<br>Mort.rate controls=9.4%	no data
			Comments	Retrospective chart review					neonates	neonates in ICU			neonates in ICU	all BSI	all in NICU	mortality in hospital			
	7	Routsi, 2010	Score	+	HCA	30 cases<br>66 controls	-	++	++	+	++	++	-	-	++	c.b.e	-	Mort. rate cases=43.3%<br>Mort.rate controls=46.9%	median (IQR)<br>cases=33(18.8-55.8)<br>controls=28.5(14.5-51.0)
			Comments	Prospective observational study	not expicitly said in the article, but very specific patients					only ICU patients		not matched but controlled in the univariate analyses		all BSI	all in ICU	mortality in hospital			
	8	Tal-Jasper 2016	Score	-	c.b.e	149 cases<br>91 controls	-	+	++	++	++	+	-	-	+	++	++	a) Mort. rate cases=70.5%<br>Mort.rate controls=45.1%<br>b) Mort. rate cases=62% Mort.rate controls=44.4% c)Mort. rate cases=73.6% Mort.rate controls=53.3%<br>OR 90days=0.6, 95%CI (0.1–2.3)	median (IQR)<br>cases=21(10.8-39.5)<br>controls=10(7-20.5)
			Comments	Retrospective cohort study	no information available in the article							not matched but controlled in the univariate analyses for age		all BSI		a) in hospital<br>b) 30 days<br>c)90 days	differences between mortality rates were always significant, OR was significant only for 90 days	a) in hospital<br>b) 30 days<br>c)90 days	
Resp. Tract R vs NI	1	Thatrimontrichai, 2016	Score	-	HCA	63 resistant<br>13 sensitive<br>25 non-infected	-	-	+	+	++	++	+	-	++	c.b.e	++	Mort. rate resit=15.9%<br>Mort. sensit=7.7%<br>Mort. NI=0%	median(range)<br>resist=29(8-93)<br>senstiv=20(8-31)<br>NI=22(8-68)
			Comments	Retrospective Case-case-control study	not expicitly said in the article, but very specific patients	resistants include also intermediate MIC values (numbers not specified)			all neonates	all neonates in ICU with VAP		not matched but controlled in the univariate analyses	VAP	all resp. Tract infections	all in NICU	mortality in hospital	significant difference between the mortality rates of R vs NI		
Resp. Tract R vs S	1	Chang, 2011	Score	-	HCA	93 cases<br>87 controls	-	+	++	++	++	++	-	-	+	c.b.e	++	Mort. rate cases=61.3%<br>Mort.rate controls=46.0%	after VAP onset<br>median±SD<br>cases=23.1±24.3<br>controls=26.7±24.3
			Comments	Retrospective observational study								not matched but controlled in the univariate analyses		all resp. Tract infections		mortality in hospital	comparison of mortality rates		
	2	Garnacho-Montero, 2003	Score	+	HCA	21 cases<br>14 controls	-	++	++	+	++	-	++	-	++	c.b.e	-	Mort. rate cases=61.9%<br>Mort.rate controls=64.2%<br>*VAP related mortality<br>Mort. rate cases=38%<br>Mort.rate controls=35.7%	mean±SD<br>cases=45.2±30.7<br>controls=53.9±50
			Comments	Prospective cohort study	not expicitly said in the article, but very specific patients					ICU patients			not matched but controlled for ApacheII and SOFA	all resp. Tract infections	all in ICU	mortality in hospital	no difference in any mortality	*occurred during the treatment period, when the signs of pneumonia remained, an	
	3	Zheng, 2013	Score	-	HCA	97 cases<br>145 controls	-	-	++	++	++	++	-	-	+	++	++	Mort. rate cases=45.6%<br>Mort.rate controls=29.9%	before pneum<br>mean±SD<br>cases=17.7±6.6<br>controls=18±6.2
			Comments	Retrospective study								not matched but controlled in the univariate analyses		all resp. Tract infections			comparison of mortality rates		
Mixed inf. site R vs NI	1	Henig, 2015	Score	++	Mix	1190 cases<br>1190 controls	-	+	++	++	++	+	++	-	++	c.b.e	++	Mort. rate cases=73%<br>Mort.rate controls=55%<br>aHR=2.33; 95%CI(2.08–2.6)	median (range)<br>cases=18 (0–97)<br>controls=17 (0–96)
			Comments	Matched case-control study	no information on the proportion HCA/CA							matched for age	Charlson index is a matching criteria			not defined, most probably hospitalization	significant aHR, no info on the comparison of mortality rates		
	2	Nazer, 2015	Score	++	HCA	142 cases<br>232 controls	-	-	++	-	++	++	++	-	++	c.b.e	++	Mort. rate cases=73%<br>Mort.rate controls=61.2%	median (IQR)<br>cases=12(6-13)<br>controls=3(1.7)
			Comments	Matched case-control study	not expicitly said in the article, but very specific patients					critically ill patients with cancer in ICU					all in ICU	mortality in ICU	comparison of mortality rates		
Mixed inf. site R vs S	1	Aydemir, 2012	Score	-	HCA	110 cases<br>55 controls	-	-	++	++	++	++	-	-	+	c.b.e	-	Mort. rate cases=61.8%<br>Mort.rate controls=52.7%	after isolation<br>mean±SD<br>cases=18.1±14.0<br>controls=15.7±12.3
			Comments	Retrospective case-control study					>16 years			not matched but controlled in the univariate analyses				mortality in ICU			
	2	de Gouvea, 2012	Score	-	HCA	18 cases<br>31 controls	-	-	++	-	+	++	-	-	+	++	-	Mort. rate cases=44.4%<br>Mort.rate controls=29.0%<br>Inf. related. OR=0.73; 95%CI(0.12 – 4.47)<br>Overall mort. OR=1,93; 95%CI(0.48 – 7.85)	no data
			Comments	Retrospective study						liver and kidney transpant patient		not matched but controlled in the univariate analyses				30 days	none of the data on mortality is significant		
	3	del Mar, 2005	Score	c.b.e	HCA	30 cases<br>31 controls	-	++	++	+	++	++	-	-	++	++	c.b.e	Mort. rate cases=43.3%<br>Mort.rate controls=19%<br>*Related mortality=30%	attributable LOS=14 days
			Comments	Case-control study (Not specified if prospective or retrospective)	it was an hospital outbreak					RU and ICU patients		not matched but controlled in the univariate analyses			RU and ICU	30 days		* when infection was established, by clinical criteria, as theprimary cause of death	
	4	Lautenbach, 2009	Score	+	c.b.e	89 cases<br>297 controls	-	+	++	++	++	-	-	-	-	++	-	Mort. rate cases=18%<br>Mort.rate controls=21.2%<br>RR=0.85; 95% CI(0.52–1.39)	median(range)<br>cases=21(14-29)<br>controls=16(12-18)
			Comments	Prospective cohort study	no information available in the article				significant difference in age						2 hospitals	30 days			
	5	Lemos, 2014 a	Score	+	c.b.e	104 cases<br>61 controls	-	-	++	+	++	+	-	-	++	++	-	Mort. rate cases=40%<br>Mort.rate controls=12.2%<br>aHR=1.45; 95%CI(0.74-2.87)	adjusted mean(range)<br>cases=19.3(16-22.5)<br>controls=16.2(11.5-19.9)
			Comments	Prospetive, multicentre cohort study	no information available in the article					ICU patients		not matched but controlled, age is significantly different				30 days			
	6	Lemos, 2014 b	Score	-	NA	1013 cases<br>1553 controls	-	c.b.e	++	++	c.b.e	-	-	-	-	c.b.e	++	crude OR=2.22<br>95% CI=1.66-2.98	
			Comments	Systematic review and meta-analysis		sum of cases and controls of all studies		studies from all the world			the criteria refer to the included studies					study specific			
	7	Sheng, 2010	Score	-	HCA	a)30 cases<br>30 controls<br>b) 91 cases<br>97 controls	-	+	++	++	++	++	++	++	-	++	++	a)Mort. rate cases=13.3%<br>Mort.rate controls=20%<br>b)Mort. rate cases=33%<br>Mort.rate controls=17.5%	after isolation<br>median(range)<br>a)cases=41 (5–282)<br>controls=29 (2–227)<br>b) cases=37 (1–218)<br>controls=23 (1–151)
			Comments	Multicentre retrospective study		a) colonized patients<br>b) infected patients						not matched but no significant differencein test distribution	not matched but no significant differencein test distribution	not matched but controlled in the univariate analyses		30 days	significant only for infection (b)	a) colonized patients<br>b) infected patients	a) colonized patients<br>b) infected patients
	8	**The Brooklyn Antibiotic Resistance Task Force**	Score	++	Mix	a)44 cases<br>33 controls<br>b) 10 cases<br>10 controls	-	+	++	++	++	+	++	++	-	c.b.e	-	a)Mort. ratecases=34%<br>Mort.rate controls=27%<br>b)Mort. ratecases=20%<br>Mort.rate controls=30%	after isolation<br>median<br>b) cases=31.5<br>controls=13

		Comments	Matched case-control study	100% of cases and 85% of controlswere HCA	a) all cases and control<br>b) matched pairs						matched for age the 10 pairs	only the 10 matched pairs	only the 10 matched pairs		mortality in hospital	none of the data on mortality is significant		data not available for group a)

Sample size
N refers to total number of cases and controls **(no score)**
Calculation *a priori:* ++ if performed (e.g. based on expected prevalence or on litterature data) or the study refers to large national surveys; - if not performed

Study type
Study type: ++ matched cohort or case-control studies; + prospective cohort or case-control studies; - restrospective database studies

Infection type
HCA: health-care associated; **CA:** community associated; **Mix:** health-care and community associated together

Representativeness
Geographical: ++ if the study was conducted in an EU/EEA setting; + if the study was conducted in high-income country(ref. World bank); - if the study was not conducted in an EU/EEA or high-income country setting
Demographical: ++ if the study population represents all age groups; + if the study population represents selected age-specific groups (e.g. elderly, infants); - if the study population represents a selected sex category and age-specific groups
Clinical: ++ if the study population represents the overall hospital population; + if the study population partially represents the hospital population; - if the study population represents a disease-specific population (e.g. liver transplanted patients, cancer patients, etc..)
Exlusion/inclusion criteria: ++ if the same exclusion/inclusion criteria were adopted for both cases and controls; + if some (but not all) exclusion/inclusion criteria were adopted for both cases and controls; - if exclusion/inclusion criteria were different in cases and controls

Matching or controlling for confounders
Demographics: ++ if performed for age and sex; + if performed only for age or only for sex; - if not performed at all
Underlying disease: ++ if performed for two or more criteria (e.g. comorbidity score, McCabe score, severity index, n. comorbidities, allergy, immunosuppression status, prior hospitalisation etc..); + if performed for only one criteria; - if not performed at all
Infection site: ++ if performed ; - if not performed
Hospital and unit/ward: ++ if same hospital and same unit/ward; + if same hospital but different unit/ward; - if not same hospital and/or not same unit/ward
Follow-up: ++ if ≥ 28 days; - if < 28 days

Risk difference
Statistically significant outcome results: ++ if results and 95%CI are statistically significant; - if results and 95%CI are not statistically significant
Case fatality and LOS refer to risk difference estimates in cases and controls **(no score)**

Scoring table

++	Matches completely/is completely fulfilled
+	Matches incompletely but sufficiently/is only partlybut sufficiently fulfilled
-	Does not match or matches insufficiently/isinsufficiently fulfilled
c.b.e.	Cannot be evaluated

ColRACI **Checklist for the evaluation of the most relevant studies for building antimicrobial resistance (AMR) outcome trees, healthcare-associated infection (HAI) attributable case fatality and attributable length-of-stay (LOS)**

	Study				Study type	Infection type	Sample size		Representativeness			Matching or controlling for confounders						Risk difference		
							N (cases;controls)	calculation *a priori*	Geographical	Demographical	Clinical	Exclusion/inclusion criteria	Demographics	Underlying disease	Infection site	Hospital and unit/ward	Follow-up	Statistically significat outcome results	Case fatality	LOS
	Nr.	First author, year				HCA; CA; Mix; c.b.e		□ ++; □ -; □ c.b.e	□ ++; +; -; □ c.b.e	□ ++; □ +; □ -; □ c.b.e	□ ++; -; □ c.b.e	□ ++; □ +; □ -; □ c.b.e	□ ++; □ +; □ -; □ c.b.e	□ ++; □ +; □ -; □ c.b.e	□ ++; □ -; □ c.b.e	□ ++; □ +; □ -; □ c.b.e	□ ++; -; □ c.b.e	□ ++; □ -; □ c.b.e	%; RR; OR; aHR; etc	(days)
BSI R vs S	1	Wang, 2015	Score		-	c.b.e	58 cases 213 controls	-	+	++	++	++	++	-	-	+	-	-	a) Mort. ratecases=10.3% Mort.rate controls=10.3% b) Mort. ratecases=15.5% Mort.rate controls=15%	prior infection median (IQR) cases=56 (32-101) controls=37 (22-65)
			Comments		Retrospective study	no information available in the article	all patient had *A.nosomialis* BSI but all they had also experienced at least one *A. calcoaceticus-baumannii* complex BSI						not matched but controlled in the univariate analyses		all BSI infections		a) 14 days b) 18 days	none of the follow up mortalities	a) 14 days b) 18 days	
UTI R vs S	1	Taneja, 2011	Score		-	c.b.e	8 cases 42 controls	-	-	++	-	++	-	-	-	-	c.b.e	-	Mort. Rate both groups=0%	no data
			Comments		Prospective cohort study	no information available in the article					all patients had complicated UTI infections				all UTI infections					

Sample size — **N** refers to total number of cases and controls **(no score)**
Calculation *a priori*: ++ if performed (e.g. based on expected prevalence or on litterature data) or the study refers to large national surveys; - if not performed

Study type — **Study type:** ++ matched cohort or case-control studies; + prospective cohort or case-control studies; - restrospective database studies

Infection type — **HCA:** health-care associated; **CA:** community associated; **Mix:** health-care and community associated together

Representativeness
Geographical: ++ if the study was conducted in an EU/EEA setting; + if the study was conducted in high-income country(ref. World bank); - if the study was not conducted in an EU/EEA or high-income country setting
Demographical: ++ if the study population represents all age groups; + if the study population represents selected age-specific groups (e.g. elderly, infants); - if the study population represents a selected sex category and age-specific groups
Clinical: ++ if the study population represents the overall hospital population; + if the study population partially represents the hospital population; - if the study population represents a disease-specific population (e.g. liver transplanted patients, cancer patients, etc..)

Matching or controlling for confounders
Exlusion/inclusion criteria: ++ if the same exclusion/inclusion criteria were adopted for both cases and controls; + if some (but not all) exclusion/inclusion criteria were adopted for both cases and controls; - if exclusion/inclusion criteria were different in cases and controls
Demographics: ++ if performed for age and sex; + if performed only for age or only for sex; - if not performed at all
Underlying disease: ++ if performed for two or more criteria (e.g. comorbidity score, McCabe score, severity index, n. comorbidities, allergy, immunosuppression status, prior hospitalisation etc..); + if performed for only one criteria; - if not performed at all
Infection site: ++ if performed ; - if not performed
Hospital and unit/ward: ++ if same hospital and same unit/ward; + if same hospital but different unit/ward; - if not same hospital and/or not same unit/ward
Follow-up: ++ if ≥ 28 days; - if < 28 days

Risk difference
Statistically significant outcome results: ++ if results and 95%CI are statistically significant; - if results and 95%CI are not statistically significant
Case fatality and LOS refer to risk difference estimates in cases and controls **(no score)**

Scoring table

++	Matches completely/is completely fulfilled
+	Matches incompletely but sufficiently/is only partly but sufficiently fulfilled
-	Does not match or matches insufficiently/is insufficiently fulfilled
c.b.e.	Cannot be evaluated

MDRPA

Checklist for the evaluation of the most relevant studies for building antimicrobial resistance (AMR) outcome trees, healthcare-associated infection (HAI) attributable case fatality and attributable length-of-stay (LOS)

	Study			Study type	Sample size		Representativeness			Matching or controlling for confounders							Risk difference	
					N (cases;controls)	calculation *a priori*	Geographical	Demographical	Clinical	Exclusion/inclusion criteria	Demographics	Underlying disease	Infection site	Hospital and unit/ward	Follow-up	Statistically significat outcome results	Case fatality	LOS
	Nr.	First author, year				□ ++; □ -; □ c.b.e	□ ++; +; -; □ c.b.e	□ ++; □ +; □ -; □ c.b.e	□ ++; -; □ c.b.e	□ ++; □ +; □ -; □ c.b.e	□ ++; □ +; □ -; □ c.b.e	□ ++; □ +; □ -; □ c.b.e	□ ++; □ -; □ c.b.e	□ ++; □ +; □ -; □ c.b.e	□ ++; -; □ c.b.e	□ ++; □ -; □ c.b.e	%; RR; OR; aHR; etc	(days)
BSI<br>R vs S	1	Caselli, 2010	Score	-	39 cases<br>88 controls	-	++	+	-	++	-	-	-	-	++	++	Mort. rate cases=35%.8<br>Mort. rate controls=12.5%<br>aOR=4.3; 95%CI(1.67-11.07)	no data
			Comments	Retrospective study				children patients	children under chemotherapy and HSC transplant				all BSI		30 days		OR adjusted for gender, type of cancer and neutopenia	
	2	Johnson, 2009	Score	-	41 cases<br>50 controls	-	+	++	-	++	c.b.e	c.b.e	-	-	++	-	Mort. rate cases=36.6%<br>Mort. rate controls=46%<br>OR=0.68; 95%CI(0.27-1.71)	no specific data
			Comments	Retrospective cohort study					transplanted patients		no information	no information	all BSI		28 days			
	3	Morata, 2012	Score	+	127 cases<br>582	-	++	++	++	++	++	++	-	+	++	++	Mort. rate cases=17.2%<br>Mort. rate controls=32.3%	mean±SD<br>cases=31.83±30<br>controls=16.38±18
			Comments	Prospective study							not matched but controlled in univariate	not matched but controlled in univariate	all BSI		30 days			
	4	Tam, 2010	Score	-	a) 25 cases<br>84 controls<br>b) 21 cases<br>21 controls	-	+	++	++	++	a) ++<br>b) ++	a) -<br>b) ++	-	+	++	++	I) Mort. rate cases=40%<br>Mort. rate controls=11.9%<br>OR=6.829; 95%CI(1.945–23.984)<br>II) Mort. rate cases=52%<br>Mort. rate controls=9.5%<br>III) Mort. rate cases=56%<br>Mort. rate controls=16.7%<br>IV) c) Mort. rate cases=61.9%<br>Mort. rate controls=9.5%	mean±SD<br>cases=26.4±28.3<br>controls=16.5 ±23.6
			Comments	Retrospective cohort study	a) whole sample<br>b) matched pairs						a) whole sample<br>b) matched pairs<br>group a) was controlled in univariate analyses	a) whole sample<br>b) matched pairs	all BSI		I)30 days<br>II)infection related<br>III) overall mortality<br>IV) matched pairs	all results are significant	I) 30 days<br>II) infection related<br>III) overall mortality<br>IV) matched pairs	
	5	Theodorou, 2013	Score	-	45 cases<br>42 controls	-	++	++	-	++	-	-	-	++	-	-	Mort. rate cases=42.2%<br>Mort. rate controls=45.2%<br>OR=1.076; 95%CI(0.356–3.254)	In ICU<br>mean±SD<br>cases=49.3±34.6<br>controls=46.8±32.3
			Comments	Retrospective cohort study					burned patients in ICU				all BSI		mortality in hospital			
	6	Trecarichi, 2011	Score	+	27 cases<br>11 controls	-	++	++	-	++	++	++	-	-	-	-	Mort. rate cases=40.7% Mort. rate controls=9.1%	no data
			Comments	Prospective study					patients with hematological malignancies		not matched but controlled in univariate analyses	not matched but controlled in univariate analyses	all BSI		21 days			
	7	Tumbarello, 2011	Score	-	40 cases<br>66 controls	-	++	++	-	++	-	-	-	-	-	++	Mort. rate cases=50%<br>Mort. rate controls=24.2%<br>OR=3.31; 95%CI(1.27–8.59)	mean±SD<br>cases=27±14<br>controls=17±13
			Comments	Retrospective case-control study					patients with BSI and systemic inflammatory response syndrome				all BSI		21 days			
	8	Dantas, 2014	Score	-	57 cases<br>63 controls	-	-	++	++	++	c.b.e	c.b.e	-	+	++	-	Mort. rate cases=42.1%<br>Mort. rate controls=41.2%	mean (range)<br>cases=59 (4–205)<br>controls=62 (3–378)
			Comments	Retrospective study							the study considered more resistances and no information was available for the matching	the study considered more resistance and no information was available for the CRAB matching	all BSI		30 days			
	9	Joo 2011	Score	-	42 cases<br>160 controls	-	+	++	++	++	c.b.e	c.b.e	-	+	++	++	Mort. rate cases=38.1%<br>Mort. rate controls=21.9%<br>OR=2.20; 95%CI(1.06–4.55)<br>aOR=2.24; 95%CI (0.80–6.29)*	median (IQR)<br>cases=25 (13–38)<br>controls=16 (10–31)
			Comments	Retrospective study							the article did not compare cases with controls, instead suvivors with non survivors	the article did not compare cases with controls, instead suvivors with non survivors	all BSI		30 days		*adjusted for corticosteroid use, nosocomial acquisition, polymicrobial infection, Charlson's weighted index of co-morbidity, and admission to ICUs	
Resp tract<br>R vs S	1	Cilloniz, 2016	Score	+	22 cases<br>46 controls	-	++	++	++	++	++	++	-	+	++	-	Mort. Rate cases=22.7%<br>Mort. rate controls=17.4%	median (IQR)<br>cases=14(12-21)<br>controls=11(7-16)
			Comments	Prospective observational study					there are some exclusione criteria for immunosoppressed patients (look fulltext)		not matched but controlled in univariate analyses	not matched but controlled in univariate analyses	all pneumonia		30 days			
	2	Montero, 2009	Score	c.b.e	50 cases<br>50 controls	-	++	++	-	++	++	+	-	+	++	++	Mort. rate cases=60%<br>Mort. rate controls=28%<br>OR=6.2; 95%CI(1.7–22.1)	no data
			Comments	Case-control study	patients with COPD, isolation sample was sputum				patients with COPD acute exacerbation		matched for age, controlle for gender	mathced for degree of airway obstruction	all resp tract		2 years			
	3	Micek, 2015	Score	-	226 cases<br>31 controls	-	++	++	++	++	-	++	-	-	-	++	Mort. rate cases=44.7%<br>Mort. rate controls=31.7%<br>HR=1.39; 95%CI(1.05-1.83)	median (IQR)<br>cases=27(14-56.3)<br>controls=25(13-46)
			Comments	Multicentre restrospective study			EU coutnries + USA					not matched but controlled in univariate analyses for Charlson score	all resp tract		mortality in hospital			
	4	Pena, 2013	Score	-	60 cases<br>31 controls	-	++	++	++	++	+	+	-	+	-	-	a) Mort. rate cases=15%<br>Mort. rate controls=29%<br>b) Mort. rate cases=50% Mort. rate controls=55%	no data
			Comments	Retrospective study							not matched but controlled in univariate analyses for gender, age is higher in cases	not matched but controlled in univariate analyses for Charlson score	all resp tract		a) 7 days<br>b) mortality in hospital		a) 7 days<br>b) mortality in hospital	
	5	Tumbarello, 2013	Score	-	42 cases<br>68 controls	-	++	++	+	++	c.b.e	c.b.e	-	+	-	++	Mort. rate cases=59.5%<br>Mort. rate controls=35.2%<br>OR=2.69; 95%CI(1.14–6.43)	no data
			Comments	Retrospective study					ICU patients		no matching and no comparison between cases and controls	no matching and no comparison between cases and controls	all resp tract		mortality in ICU			
	6	Yang, 2009	Score	+	20 cases<br>55 controls	-	+	++	++	++	++	++	-	+	++	-	Mort. rate cases=35%<br>Mort. rate controls=29%	median (IQR)<br>cases=37(28.5-72)<br>controls=34(21-47)
			Comments	Prospective observational study					excluded patients with known risk factors (loot at full text)		not matched but controlled in univariate analyses	not matched but controlled in univariate analyses	all resp tract		28 days			
	7	Yayan, 2015	Score	-	a) 14 cases<br>63 controls<br>b) 27 cases<br>64 controls	-	++	++	++	++	c.b.e	c.b.e	-	+	-	-	a) Mort. rate cases=28.6%<br>Mort. rate controls=23.8%<br>b) Mort. rate cases=7.4%<br>Mort. rate controls=18.5%	Mean ± SD<br>a) cases= 25.8 ± 23.6<br>b) cases=17.5 ± 20.8
			Comments	Retrospective study	a) HAI<br>b) CAI						cases and controls are not compared, the comparison is between HAI and CAI	cases and controls are not compared, the comparison is between HAI and CAI	all resp tract		mortality in hospital		a) HAI<br>b) CAI	a) HAI<br>b) CAI
Mixed inf. site<br>R vs NI	1	Aloush, 2006	Score	++	82 cases<br>82 controls	-	++	++	++	++	++	+	NA	++	-	++	Mort. rate cases=21%<br>Mort. rate controls=12%<br>OR=4.4	median<br>cases=20<br>controls=10
			Comments	Matched cohort study							not matched but controlled in univariate analyses	not matched but controlled in univariate analyses for McCabe score			mortality in hospital	only OR		
	2	Paramythiotou, 2004	Score	++	34 cases<br>34 control	-	++	++	+	++	++	++	NA	++	-	-	a) Mort. rate cases=44%<br>Mort. rate controls=47%<br>b) Mort. rate cases=47%<br>Mort. rate controls=50%	mean SD<br>cases=57.0±37.3<br>controls=46.9±22.5
			Comments	Matched case-control study					patients in ICU		not matched but controlled in univariate analyses				a) mortality in ICU<br>b) mortality in hospital		a) mortality in ICU<br>b) mortality in hospital	
Mixed inf. site<br>R vs S	1	Cao, 2004	Score	-	44 cases<br>68 controls	-	-	++	++	++	++	-	-	+	-	++	Mort. rate cases=54.5%<br>Mort. rate controls=16.2%	in ICU<br>mean(SD)<br>cases=21(47.7)<br>control=11(16.2)
			Comments	Retrospective cohort study	cases are resistant and intermediate						not matched but controlled in univariate analyses for gender, age is higher in cases				mortality in hospital			
	2	de Matos, 2016	Score	-	20 cases<br>34 controls	-	-	++	+	++	-	-	-	++	-	-	Mort. rate cases=70%<br>Mort. rate controls=58.9%<br>OR=1.6333;<br>95%CI(0.5043–5.2901)<br>Adults:<br>cases=81.3%<br>controls=71.4%<br>paediatric:<br>cases=25.0%	no specific data
			Comments	Retrospective cohort study				adluts, children and neonates	patients in ICU						mortality in hospital			
	3	Morales, 2012	Score	-	134 cases<br>149 controls	-	++	++	++	++	+	+	++	+	-	++	Mort. rate cases=24.6%<br>Mort. rate controls=12.8%<br>OR=1.77; 95%CI(1.41-2.22)	mean(SD), median<br>cases=25.1(16.1), 20<br>controls=39.0(30.3), 30
			Comments	Retrospective study							not matched but controlled in univariate analyses for age, gender is different	not matched but controlled in univariate analyses for main diseases, only renal disease is higher in cases	not matched but controlled in univariate analyses		mortality in hospital			
	4	Nathawani, 2014	Score	-	559 cases<br>7881 controls	-	NA	++	++	++	NA	NA	NA	-	-	++	Mort. rate cases=25-60%<br>Mort. rate controls=7-50%<br>RR=2.34, 95%CI(1.53 – 3.57)	
			Comments	Review and Meta-analyses			different countries								mortality in hospital	significant RR		
	5	Ortega, 2004	Score	+	18 cases<br>35 controls	-	++	++	+	++	++	++	++	++	-	-	Mort. rate cases=22%<br>Mort. rate controls=23%	in ICU<br>median [illegible]<br>controls=10(0-44)
			Comments	Prospective study					patients in ICU		not mathced but controlled in univariate analyses	not mathced but controlled in univariate analyses	not mathced but controlled in univariate analyses		mortality in ICU			
	6	Peng 2014	Score	-	188 cases<br>168 controls	-	-	++	++	++	c.b.e	c.b.e	-	-	-	++	Mort. rate cases=26.1%<br>Mort. rate controls=12.5%	median (range)<br>cases=39(5-697)<br>controls=24(1-262)

		Comments	Retrospective case-control study							no information available	no information available			mortality in hospital			
7	Su, 2016	Score	-	25 cases<br>29 controls	-	-	++	-	++	c.b.e	c.b.e	-	-	++	-	a) Mort. rate cases=36%<br>Mort. rate controls=31.0%<br>b) Mort. rate cases=47.6%<br>Mort. rate controls=37.9%	no specific data
		Comments	Retrospective double centre study					patients with abdominal organ transplantation		no comparison between cases and controls, but between dead patients and not	no comparison between cases and controls, but between dead patients and not			30 days		a) related mortality<br>b) overall mortality	no comparison between cases and controls, but between dead patients and not

Sample size

N refers to total number of cases and controls **(no score)**
Calculation *a priori:* ++ if performed (e.g. based on expected prevalence or on litterature data) or the study refers to large national surveys; - if not performed

Study type

Study type: ++ matched cohort or case-control studies; + prospective cohort or case-control studies; - restrospective database studies

Representativeness

Geographical: ++ if the study was conducted in an EU/EEA setting; + if the study was conducted in high-income country(ref. World bank); - if the study was not conducted in an EU/EEA or high-income country setting
Demographical: ++ if the study population represents all age groups; + if the study population represents selected age-specific groups (e.g. elderly, infants); - if the study population represents a selected sex category and age-specific groups
Clinical: ++ if the study population represents the overall hospital population; + if the study population partially represents the hospital population; - if the study population represents a disease-specific population (e.g. liver transplanted patients, cancer patients, etc..)
Exlusion/inclusion criteria: ++ if the same exclusion/inclusion criteria were adopted for both cases and controls; + if some (but not all) exclusion/inclusion criteria were adopted for both cases and controls; - if exclusion/inclusion criteria were different in cases and controls

Matching or controlling for confounders

Demographics: ++ if performed for age and sex; + if performed only for age or only for sex; - if not performed at all
Underlying disease: ++ if performed for two or more criteria (e.g. comorbidity score, McCabe score, severity index, n. comorbidities, allergy, immunosuppression status, prior hospitalisation etc..); + if performed for only one criteria; - if not performed at all
Infection site: ++ if performed ; - if not performed
Hospital and unit/ward: ++ if same hospital and same unit/ward; + if same hospital but different unit/ward; - if not same hospital and/or not same unit/ward
Follow-up: ++ if ≥ 28 days; - if < 28 days

Risk difference

Statistically significant outcome results: ++ if results and 95%CI are statistically significant; - if results and 95%CI are not statistically significant
Case fatality and LOS refer to risk difference estimates in cases and controls **(no score)**

Scoring table

++	Matches completely/is completely fulfilled
+	Matches incompletely but sufficiently/is only partly but sufficiently fulfilled
-	Does not match or matches insufficiently/is insufficiently fulfilled
c.b.e.	Cannot be evaluated

CRPA **Checklist for the evaluation of the most relevant studies for building antimicrobial resistance (AMR) outcome trees, healthcare-associated infection (HAI) attributable case fatality and attributable length-of-stay (LOS)**

	Study			Study type	Sample size		Representativeness			Matching or controlling for confounders						Risk difference		
					N (cases;controls)	calculation *a priori*	Geographical	Demographical	Clinical	Exclusion/inclusion criteria	Demographics	Underlying disease	Infection site	Hospital and unit/ward	Follow-up	Statistically significat outcome results	Case fatality	LOS
	Nr.	First author, year				□ ++; □ -; □ c.b.e	□ ++; +; -; □ c.b.e	□ ++; □ +; □ -; □ c.b.e	□ ++; -; □ c.b.e	□ ++; □ +; □ -; □ c.b.e	□ ++; □ +; □ -; □ c.b.e	□ ++; □ +; □ -; □ c.b.e	□ ++; □ -; □ c.b.e	□ ++; □ +; □ -; □ c.b.e	□ ++; -; □ c.b.e	□ ++; □ -; □ c.b.e	%; RR; OR; aHR; etc	(days)
BSI<br>R vs S	1	Dantas, 2014	Score	-	55 cases<br>65 controls	-	-	++	++	++	c.b.e	c.b.e	-	+	++	-	Mort. cases= 47.3%<br>Mort. controls= 36.9%<br>OR=1.53	mean (range) cases=48(4-205) controls=41(3-378)
			Comments	Retrospective study							the study considered more resistance and no information was available for the CRAB matching	the study considered more resistance and no information was available for the CRAB matching	all BSI		30 days			
	2	Hattemer, 2013	Score	-	13 cases<br>137 controls	-	+	++	++	++	++	++	-	-	++	-	Mort. cases= 54%<br>Mort. controls= 32%	no specific data
				Retrospective study							not matched but contolled in univariate analyses	not matched but contolled in univariate analyses	all BSI	2 hospitals	30 days			
	3	Joo, 2011	Score	-	44 cases<br>156 controls	-	+	++	++	++	c.b.e	c.b.e	-	+	++	++	Mort. cases= 39.1%<br>Mort. controls= 21.2%<br>OR=2.40; 95%CI(1.18–4.86)<br>aOR=2.74: 95%(1.02–7.37)*	median (IQR)<br>cases=16(14-42)<br>controls=16(9-29)
			Comments	Retrospective cohort study							the article did not compare cases with controls, instead suvivors with non	the article did not compare cases with controls, instead suvivors with non	all BSI		30 days		*adjusted for corticosteroid use, nosocomial acquisition, polymicrobial infection, Charlson's weighted index of co-morbidity, and admission to	
	4	Kang, 2005	Score	-	28 cases<br>158 controls	-	+	++	++	++	c.b.e	c.b.e	-	+	++	-	Mort. cases= 53.6%<br>Mort. controls= 35.2%<br>OR=2.216; 95%CI(0.946–4.776)	no data
			Comments	Retrospective study							the information was not specific	the information was not specific	all BSI		30 days			
	5	Kim, 2014	Score	-	118 cases<br>116 controls	-	+	++	++	++	c.b.e	c.b.e	-	+	-	-	Mort. cases= 22.0%<br>Mort. controls= 22.4%<br>OR=3.65; 95%CI(0.72-18.67)	no data
			Comments	Retrospective study							the article did not compare cases with controls, instead suvivors with non survivors	the article did not compare cases with controls, instead suvivors with non survivors	all BSI		14 days			the article did not compare cases with controls, instead suvivors with non survivors
	6	Pena, 2012	Score	+	145 cases<br>487 controls	++	++	++	++	++	+	++	-	-	++	-	a) Mort. cases= 13%<br>Mort. controls= 13%<br>b)Mort. cases= 35%<br>Mort. controls= 27%	no data
			Comments	Prospective multicentre study							not matched but contolled in univariate analyses for age, gender is different	not matched but contolled in univariate analyses for SAPS score, Charlson index and main diseases. Only immunosuppresive therapy is higher in cases	all BSI	10 hospitals	a) 48h<br>b) 30 days		a) 48h<br>b) 30 days	
	7	Suarez, 2010	Score	-	33 cases<br>88 controls	-	++	++	++	++	c.b.e	++	-	+	++	-	a)Mort. cases= 46%<br>Mort. controls= 36%<br>b)Attribut. mort. cases=33%<br>Attribut. mort. controls=30%<br>OR=1.8; 95%CI(0.5–6.8)	mean (range)<br>cases=19(2-98)<br>controls=9(2-56)
			Comments	Retrospective cohort study							the article did not compare cases with controls, instead suvivors with non survivors	not matched but controlled in univariate analyses	all BSI		a) 30 days<br>b) 7 days		a) 30 days<br>b) 7 days (defined as attributable mortality)	
	8	Tuon, 2012	Score	c.b.e	29 cases<br>48 controls	-	-	++	++	++	++	++	-	+	++	-	Mort. cases= 54.2%<br>Mort. controls= 44.8%<br>OR=0.68; 95%CI(0.27–1.73)	mean±SD<br>cases=43±31.7<br>controls=43.1±31.2
			Comments	Case-control study							not matched but controlled in univariate analyses	not matched but contolled in univariate analyses for main comorbidities	all BSI		30 days			
Recsp tract<br>R vs S	1	Luyt, 2014	Score	+	68 cases*<br>101 controls	-	++	++	+	++	++	++	-	++	-	-	Mort. cases= 37%<br>Mort. controls= 31%	median (IQR) cases=[illegible]<br>controls=29(19-46)
			Comments	Prospective observational study	* cases included resistant and intermediate susceptible PA, was not possible to separate them				ICU patients		not matched but controlled in univariate analyses	not matched but controlled in univariate analyses	all Resp tract	ICU	mortality during ICU stay			
Mixed inf. site<br>R vs NI	1	Eagye, 2009	Score	-	58 resistant<br>125 sensitive<br>57 non infected	-	+	++	++	++	a) ++<br>b) +	a) ++<br>b) ++	-	++	-	-	Mort. resit= 31%<br>Mort. sensit= 15%<br>Mort. control=9%	median(IQR)<br>resist=30(15-70)<br>sensit=16(6-33)<br>control=10(6-17)
			Comments	Retrospective case-control-control study							not matched but controlled in univariate analyses<br>a) R vs S<br>b) R vs NI (only gender)	not matched but controlled in univariate analyses<br>a) R vs S, except congestive heart failure<br>b) R vs NI, except COPD			mortality in hospital			
Mixed inf. site<br>R vs S	1	Judd, 2016	Score	-	32 cases<br>350 controls	-	+	++	++	++	++	-	-	+	-	++	Mort. cases= [illegible] Mort. controls= 8.9%<br>OR=2.89; 95%CI(1.15-7.28)	median (IQR)<br>cases=14(8-29)<br>controls=9(5-15)
			Comments	Retrospective observational cohort							not matched but controlled in univariate analyses				mortality in hospital			
	2	Lautenbach, 2006	Score	-	135 cases<br>719 controls	-	+	++	++	++	-	-	-	+	-	++	Mort. cases= 31.1%<br>Mort. controls= 16.7%<br>RR=1.86; 95%CI(1.38-2.51)	median (IQR)<br>cases=11(1-41)<br>controls=4(1-15)
			Comments	Retrospective cohort study											mortality in hospital			
	3	Lautenbach, 2010	Score	-	253 cases<br>2289 controls	-	+	++	++	++	-	-	-	-	-	++	Mort. cases= 17.4%<br>Mort. controls= 13.4%<br>RR=1.39; 95%CI(1.09–1.76)	prior isolation<br>median (IQR)<br>cases=8(4-12)<br>controls=4(4-5)
			Comments	Retrospective cohort study											mortality in hospital			
	4	Lin, 2014	Score	-	82 cases<br>82 controls	-	+	++	++	++	++	++	-	-	-	-	Mort. cases= 22.0%<br>Mort. controls= 19.5%	no data
			Comments	Retrospective study							not matched but controlled in univariate analyses	not matched but controlled in univariate analyses			mortality in hospital			
	5	Liu, 2015	Score	-	16 studies	-	NA	++	++	++	NA	NA	-	-	c.b.e	++	Crude OR=1.64; 95%CI(1.40, 1.93)<br>Adjusted OR=2.38; 95%CI(1.53,	no data
			Comments	Meta-analyses			articles from different countries								different in the different articles			
	6	Meradji, 2015	Score	+	15 cases<br>65 controls	-	-	++	++	++	++	++	-	-	-	-	Mort. cases= 13.34%<br>Mort. controls= 1.53%	no data
			Comments	Prospective observational study							not matched but controlled in univariate analyses	not matched but controlled in univariate analyses			mortality in hospital			
	7	Brooklyn Antibiotic Resistance Task Force	Score	++	10 cases<br>10 controls	-	+	++	++	++	+	++	++	-	-	-	Mort. cases= 20%<br>Mort. controls= 10%	median<br>preinfection<br>cases=12<br>controls=15<br>postinfection<br>cases=33.5<br>controls=20
			Comments	Matched case-control study							matched for age				mortality in hospital			

Sample size — **N** refers to total number of cases and controls **(no score)**
Calculation *a priori:* ++ if performed (e.g. based on expected prevalence or on litterature data) or the study refers to large national surveys; - if not performed

Study type — **Study type:** ++ matched cohort or case-control studies; + prospective cohort or case-control studies; - restrospective database studies

Representativeness
Geographical: ++ if the study was conducted in an EU/EEA setting; + if the study was conducted in high-income country(ref. World bank); - if the study was not conducted in an EU/EEA or high-income country setting
Demographical: ++ if the study population represents all age groups; + if the study population represents selected age-specific groups (e.g. elderly, infants); - if the study population represents a selected sex category and age-specific groups
Clinical: ++ if the study population represents the overall hospital population; + if the study population partially represents the hospital population; - if the study population represents a disease-specific population (e.g. liver transplanted patients, cancer patients, etc..)

Matching or controlling for confounders
Exlusion/inclusion criteria: ++ if the same exclusion/inclusion criteria were adopted for both cases and controls; + if some (but not all) exclusion/inclusion criteria were adopted for both cases and controls; - if exclusion/inclusion criteria were different in cases and controls
Demographics: ++ if performed for age and sex; + if performed only for age or only for sex; - if not performed at all
Underlying disease: ++ if performed for two or more criteria (e.g. comorbidity score, McCabe score, severity index, n. comorbidities, allergy, immunosuppression status, prior hospitalisation etc..); + if performed for only one criteria; - if not performed at all
Infection site: ++ if performed ; - if not performed
Hospital and unit/ward: ++ if same hospital and same unit/ward; + if same hospital but different unit/ward; - if not same hospital and/or not same unit/ward
Follow-up: ++ if ≥ 28 days; - if < 28 days

Risk difference
Statistically significant outcome results: ++ if results and 95%CI are statistically significant; - if results and 95%CI are not statistically significant
Case fatality and LOS refer to risk difference estimates in cases and controls **(no score)**

Scoring table

++	Matches completely/is completely fulfilled
+	Matches incompletely but sufficiently/is only partly but sufficiently fulfilled
-	Does not match or matches insufficiently/is insufficiently fulfilled
c.b.e.	Cannot be evaluated

MRSA

Checklist for the evaluation of the most relevant studies for building antimicrobial resistance (AMR) outcome trees, healthcare-associated infection (HAI) attributable case fatality and attributable length-of-stay (LOS)

	Study			Study type	Infection type	Sample size		Representativeness			Matching or controlling for confounders							Risk difference	
						N (cases;controls)	calculation *a priori*	Geographical	Demographical	Clinical	Exclusion/inclusion criteria	Demographics	Underlying disease	Infection site	Hospital and unit/ward	Follow-up	Statistically significant outcome results	Case fatality	LOS
	Nr.	First author, year			HCA; CA; Mix; c.b.e		□ ++; □ -; □ c.b.e	□ ++; +; -; □ c.b.e	□ ++; □ +; □ -; □ c.b.e	□ ++; -; □ c.b.e	□ ++; □ +; □ -; □ c.b.e	□ ++; □ +; □ -; □ c.b.e	□ ++; □ +; □ -; □ c.b.e	□ ++; □ -; □ c.b.e	□ ++; □ +; □ -; □ c.b.e	□ ++; -; □ c.b.e	□ ++; □ -; □ c.b.e	%; RR; OR; aHR; etc	(days)
BSI R vs NI	1	Atmaca, 2014	Score	c.b.e	c.b.e	99 cases<br>99 controls	+	-	++	+	++	++	++	-	++	c.b.e	++	Mort. rate cases= 47.5%<br>Mort. rate controls=29.3%	median<br>cases=59<br>controls=8
			Comments	Nested case control study	full-text in turkish, data extracted from abstracted in english					patients in infectious disease unit		not matched but controlled in univariate analyses	not matched but controlled in univariate analyses	all BSI		mortality in hospital		data extracted from original paper in Turkish with help of google translate	data extracted from original paper in Turkish with help of google translate
	2	McMaster, 2015	Score	+	HCA*	58 cases*<br>174 controls<br>(231 cases**)	-	++	++	+	++	++	++	-	++	++	-	a) Mort. rate cases= 19%<br>Mort. rate controls=21.2%<br>b) Mort. rate cases*= [illegible]% Mort rate controls=34.5%<br>(Mort. rate cases**= 42.9%)	in ICU<br>median (range)<br>cases*=16.6 (1.5-124.1)<br>controls=16.9 (1.9-92.1)<br>(cases**=2.9 (0.4-497.2))
			Comments	Prospective study	not esplicit in the paper but most probably allHCA	* infection aquired in ICU<br>** infection acquired before ICU admission				all ICU patients		cases acquires in ICU (*) were matched with controls	cases acquires in ICU (*) were matched with controls (APACHE II)	all BSI		a) hospitaliz<br>b) 180 days		a) hospitaliz<br>b) 180 days<br>* infection aquired in ICU<br>** infection acquired before ICU admission	* infection aquired in ICU<br>** infection acquired before ICU admission
BSI R vs NI vs R	1	Stewardson, 2016	Score	-	Mix	163 resistant<br>885 sensitive<br>604797 NI	++	++	++	+		-	-	-	-	c.b.e	++	Mort. rate resistant=22.1%<br>Mort.rate sensitive=16.8%<br>Mort.rate NI=1.7%<br>R vs NI: HR*=3.81/2.61/2.42<br>R vs S: HR*=1.19/1.20/1.26	no specific data, see full-text for modelling values*
			Comments	Multicentre retrospective cohort study	HC onset: 38% cases, 51% controls community onset: 62% cases, 49% controls					patients with acute-care episodes lasting more than one day		no univariate analyses, matched in model 2	no univariate analyses	all BSI or NI		mortality in hospital	only R vs NI	* the study models the excess in LOS and mortality between groups proposing 3 models for each	* the study models the excess in LOS and mortality between groups proposing 3 models for each
	2	Yasmin, 2016	Score	-	Mix	118 resistant<br>145 sensitive<br>118 NI	-	+	++	++	++	++	-	-	+	c.b.e	-	a)Mort. rate resist= 22%<br>Mort. rate sensit=19.5%<br>Mort. rate NI=1.7%<br>b)Mort. rate resist=19.5%<br>Mort. rate sensit=10.3%	no specific data
			Comments	Retrospective case-case-cohort study	HC onset: 14% cases, 10% controls community onset: 75.4% cases, 63.4% controls							not matched but controlled in univariate analyses		all BSI		mortality in hospital		a) mortality during hospitalisation<br>b) attributable mortality	
	3	De Kraker 2011	Score	++	c.b.e	I) 248 resistant<br>453 NI<br>II) 618 sensitive<br>1170 NI	-	++	++	++	++	+	-	-	-	++	++	**R vs NI (Cohort I)**<br>a) Mort. rate resist= 31%*<br>Mort. rate NI=8%*<br>aOR=4.4*<br>b) Mort. rate resist= 36%*<br>Mort. rate NI=9%*<br>aHR=3.1*<br>**S vs NI (Cohort II)**<br>a) Mort. rate sensit=22%*<br>Mort. rate NI=8%*<br>aOR=2.4*<br>b) Mort. rate sensit=23%*<br>Mort. rate NI=7%*<br>aHR=3.1*<br>**R vs S**<br>a) OR=1.8*	median (IQR)<br>Cohort I<br>resistant=16 (6-32)*<br>NI=7(3-18)*<br>Cohort II<br>sensitive=15(7-26)*<br>NI=8(4-14)*<br>excess LOS<br>R vs NI= 9.2*<br>S vs NI= 8.6*<br>R vs S= 0.6
			Comments	Prospective parallel matched-cohort study	no information available	I) cohort I<br>II) cohort II		13 EU countries				not matched but controlled in univariate analyses for age, gender is different		all BSI		a) 30 days<br>b) mortality in hospital	all the outcomes with *	a) 30 days mortality<br>b) mortality in hospital	
BSI R vs S	1	De la Calle, 2016	Score	-	Mix	42 cases<br>56 controls	-	++	++	++	++	+	+	-	+	++	-	a) Mort. rate cases= 40.5%<br>Mort. rate controls=37.5%	no data
			Comments	Retrospective study	similar HCA/CA between cases and control, but no specific info	all patients had bacteremic pneumonia with a positive BSI						not matched but controlled in univariate analyses fot gender, while age was higher in the cases	not matched but controlled in univariate analyses for different diseases, except for cardiovascular diseases that were higher in cases	all BSI		a) 30 days<br>b) related mortality		a) 30 days<br>b) related mortality (patient died without a definitive infection control)	
	2	De Rosa, 2016	Score	-	c.b.e	106 cases<br>83 controls	-	++	++	++	++	-	-	-	+	-	c.b.e	Mort. rate cases= 17.9%<br>Mort. rate controls=14.3%	no data
			Comments	Retrospective study	no information available									all BSI		21 days	no information available		
	3	Deodhars, 2015	Score	+	c.b.e	40 cases<br>61 controls	-	-	++	++	++	c.b.e	c.b.e	-	+	c.b.e	-	Mort. rate cases= 21%<br>Mort. rate controls=21.3%	no data
			Comments	Prospective study	most probably all HCA				patients >12 years			no matching and the control is for died vs survived	no matching and the control is for died vs survived	all BSI		mortality in hospital			
	4	Dolapo, 2014	Score	-	HCA	a) 10 cases<br>31 controls<br>b) 64 cases 51 controls	-	+	+	+	++	c.b.e	c.b.e	-	++	-	c.b.e	a) Mort. rate cases= 0%<br>Mort. rate controls=9.8%<br>b) Mort. rate cases= 15.7%<br>Mort. rate controls=5.2%	mean±SD<br>a) cases=80.6±42.4<br>controls=87.4±40.6<br>b) cases=53.6±36.6<br>controls=55.7±30.2
			Comments	Retrospective study	not specified in the article but patients are neonates in ICU	a) period A (2000-2003)<br>b) period B (2004-2009)			neonates patients	patients in NICU		no matching and no control for cases vs controls	no matching and no control for cases vs controls	all BSI		14 days	no information available	a) period A (2000-2003)<br>b) period B (2004-2009)	a) period A (2000-2003)<br>b) period B (2004-2009)
	5	Fortuin de Smidt, 2015	Score	+	Mix	86 cases<br>154 controls	-	-	++	++	++	++	+	-	-	c.b.e	++	Mort. rate cases= 29.1%<br>Mort. rate controls=14.3%	median (IQR)<br>cases=38 (14–64)<br>controls=19 (7–33)
			Comments	Prospective cross-sectional study	HCA: 76% cases, 50% controls CA: 24% cases, 49% controls							not matched but controlled in univariate analyses	not matched but controlled for Pitt bacteremia score	all BSI		mortality in hospital			
	6	Kim D.H, 2014	Score	-	c.b.e	29 cases<br>8 controls	-	+	++	-	++	c.b.e	c.b.e	-	+	c.b.e	++	Mort. rate cases= 62%<br>Mort. rate controls=0%<br>OR=0.158; 95%CI(0.047–0.534)	no specific data
			Comments	Retrospective study	no information available					patients had Cardiac Implanted Electronic Device-Related Infective Endocarditis (CIED-related IE)		no matching and no control for cases vs controls	no matching and no control for cases vs controls	all BSI		mortality in hospital			
	7	Kobayashi, 2014	Score	-	c.b.e	151 cases<br>189 controls	-	+	++	++	++	c.b.e	c.b.e	-	+	++	c.b.e	Mort. rate cases= 39.7%<br>Mort. rate controls=30.7%	no data
			Comments	Retrospective cohort study	no information available							no matching and no control for cases vs controls	no matching and no control for cases vs controls	all BSI		90 days	no information available		
	8	Lee J.Y, 2014	Score	c.b.e	CA	31 cases<br>138 controls	-	+	++	++	++	++	++	-	+	++	-	1-a)Mort. rate cases= 3.2%<br>Mort. rate controls=3.6%<br>1-b) Mort. rate cases= 6.5%<br>Mort. rate controls=13.8%<br>1-c) Mort. rate cases= 16.1%<br>Mort. rate controls=19.6%<br>2-a) Mort. rate cases= 0%<br>Mort. rate controls=3.6%<br>2-b) Mort. rate cases= 3.2%<br>Mort. rate controls=9.4%<br>2-c) Mort. rate cases= 6.5%<br>Mort. rate controls=13%	median (IQR)<br>cases=47 (19–87)<br>controls=24(14–44.5)
			Comments	Cohort study (with prospective and retrospective studies)								not matched but controlled in univariate analyses	not matched but controlled in univariate analyses	all BSI		a) 1 week<br>b) 30 days<br>c) 12 weeks	none of the results is significant	1- a/b/c) all-cause mortality<br>2- a/b/c) infection related mortality	
	9	Manandhar, 2016	Score	-	c.b.e	15 cases<br>21 controls	-	+	++	++	++	+	++	-	+	++	-	a) Mort. rate cases= 26.7%<br>Mort. rate controls=38.1%<br>OR=0.62;95%CI(0.15–2.61)<br>b) Mort. rate cases= 26.7%<br>Mort. rate controls=42.9%<br>OR=0.51;95%CI(0.12–2.14)	no specific data
			Comments	Retrospective study	no information available					all patients had BSI and UTI		not matched but controlled in univariate analyses for gender	not matched but controlled in univariate analyses	all BSI		a) 30 days<br>b) 90 days		a) 30 days<br>b) 90 days	
	10	McMullan, 2016	Score	+	Mix	142 cases<br>931 controls	-	+	+	++	++	+	-	-	-	++	-	a) Mort. rate cases= 3.5%<br>Mort. rate controls=2.5%<br>b) Mort. rate cases= [illegible]% Mort rate controls=4.1%	median (IQR)<br>cases=17 (8-38)<br>controls=14 (7-33)
			Comments	Prospective study	HCA: 48.6% cases, 51.7% controls CA: 51.4% cases, 48.3% controls				pediatric patients <18 years			not matched but controlled in univariate analyses for gender		all BSI		a) 7 days<br>b) 30 days		a) 7 days<br>b) 30 days	
	11	Nagao, 2016	Score	-	Mix	199 cases<br>278 controls	-	+	++	++	++	c.b.e	c.b.e	-	+	++	-	Mort. rate cases= 9%<br>Mort. rate controls=5.8%	no data
			Comments	Retrospective cohort study	HCA: total=70.2% CA: total=29.8%							no matching and no control for cases vs controls	no matching and no control for cases vs controls	all BSI		30 days			
	12	Naidoo, 2013	Score	-	Mix	95 cases<br>270 controls	-	-	+	++	++	+	-	-	+	c.b.e	++	MRSA OR (95%CI)<br>univariate=3.71 (1.77-7.76)<br>multivariate=3.76 (1.12-12.67)	no specific data
			Comments	Retrospective study	HCA: total=49% CA: total=51%				pediatric patients <13 years			not matched but controlled in univariate analyses for gender, age (in months) was different		all BSI		mortality in hospital		the mortality was not available in %	
	13	Ong, 2013	Score	-	Mix	76 cases<br>363 controls	-	+	++	++	++	-	-	-	+	++	-	Mort. rate cases= 63%<br>Mort. rate controls=39%<br>HR=1.37; 95%CI(0.95–1.97)	no data
			Comments	Retrospective cohort study	HCA: total=57% CA: total=42%									all BSI		1 year		the study aimed the log-term survival rate	
	14	Park, 2013	Score	-	c.b.e	191 cases<br>476 controls	-	c.b.e	+	-	++	NA	NA	-	-	c.b.e	++	OR fixed= 2.33; 95%CI(1.42-3.82)	no data
			Comments	Review and meta-analyses	no information available			papers from different countries	patients are children and neonates	endocarditis was in serching criteria				all BSI		mortality in hospital			
	15	Simsek, 2014	Score	-	HCA	13 cases<br>47 controls	-	-	++	-	++	c.b.e	c.b.e	-	+	c.b.e	++	Mort. rate cases= 49%<br>Mort. rate controls=15%<br>OR=12.117; 95%CI(3.159 46.475)	no specific data
			Comments	Retrospective study	not specified in the article but very specific patients					patients with with poststernotomy mediastinitis		no matching and no control for cases vs controls	no matching and no control for cases vs controls	all BSI		mortality in hospital			

	16	Thaden, 2015	Score	-	HCA	1) 567 cases<br>2047 controls<br>2) 280 cases<br>445 controls	-	+	+	+	++	c.b.e	c.b.e	-	-	++	c.b.e	1-a)Mort. rate cases= 3%<br>Mort. rate controls=4%<br>1-b) Mort. rate cases= 8% Mort rate controls=8%<br>1-c) Mort. rate cases= 11% Mort rate controls=10%<br>2-a) Mort. rate cases= 4% Mort rate controls=2%<br>2-b) Mort. rate cases= 9% Mort rate controls=3%<br>2-c) Mort. rate cases= 12% Mort rate controls=4%	no data
			Comments	Retrospective study	not specified in the article but very specific patients	1) with adequate therapy<br>2) with inadequate therapy			patients are infants in NICU	patients in NICU		no matching, and comparison between adequate and inadequate therapy	no matching, and comparison between adequate and inadequate therapy	all BSI		a) 7 days<br>b) 30 days<br>c) hospitaliz	comparison between adequate and inadequate therapy	1) with adequate therapy<br>2) with inadequate therapy<br>a) 7 days<br>b) 30 days<br>c) hospitaliz	
	17	Theodorou, 2013	Score	-	Mix	33 cases<br>41 controls	-	++	++	-	++	++	-	-		c.b.e	-	Mort. rate cases= 33.3%<br>Mort. rate controls=24.4%<br>OR=1.55, 95%CI(0.56–4.28)	median<br>cases=28.5<br>controls=21
			Comments	Retrospective cohort study	HCA: 89.2%<br>no other infos					burned patients in ICU		not matched but controlled in univariate analyses		all BSI		mortality in hospital			
	18	Wang, 2015	Score	-	Mix	a) 101 cases<br>b) 59 cases<br>c) 35 cases<br>159 controls	-	-	++	++	++	c.b.e	c.b.e	-	-	c.b.e	++	a) Mort. rate cases= 47.5%<br>OR=2.249; 95%CI(1.188-4.259)<br>b) Mort. rate cases= 30.5%<br>OR=0.998; 95%CI(0.453-2.200)<br>c) Mort. rate cases=35.3%<br>OR=1.223; 95%CI(0.466-3.210)<br>Mort. rate controls=25.3%<br>Total MRSA OR=1.707	no data
			Comments	Retrospective cohort study	separated by infection type	a) HA-MRSA<br>b) CA-MRSA<br>c) unclassified-MRSA						not matched and the comparison resulta are not undestandable	not matched and the comparison resulta are not undestandable	all BSI		mortality in hospital	only OR HA-MRSA and Total MRSA OR	a) HA-MRSA<br>b) CA-MRSA<br>c) unclassified-MRSA	
	19	Yaw, 2014	Score	-	Mix	185 cases<br>397 controls	-	+	++	++	++	+	+	-	+	++	++	a) Mort. rate cases=15%<br>Mort. rate controls=12%<br>b) Mort. rate cases=28%<br>Mort. rate controls=19%<br>c) Mort. rate cases=75%<br>Mort. rate controls=62%	no data
			Comments	Retrospective observational cohort study	HCA: 90% cases, 75% controls<br>CA: 10% cases, 25% controls							not matched but controlled in univariate analyses for gender	not matched but controlled in univariate analyses for Charlson index, SAPS score is different	all BSI		a) 8 days<br>b) 30 days<br>c) follow up at 01/03/2013	significative only b) and c)	a) 8 days<br>b) 30 days<br>c) follow up at 01/03/2013	
	20	Yilmaz, 2016	Score	+	Mix	100 cases<br>145 controls	-	-	++	++	++	c.b.e	c.b.e	-	-	++	-	Mort. rate cases= 22%<br>Mort. rate controls=11.7%<br>HR=0.99; 95%CI(0.33–3.0)	no data
			Comments	Prospective multicentre study	HCA: 95% cases, 30% controls<br>CA: 5% cases, 70% controls							no matching and no control for cases vs controls	no matching and no control for cases vs controls	all BSI		28 days			
Bone<br>R vs S	1	Shoji, 2016	Score	-	Mix	16 cases<br>55 controls	-	+	++	-	++	++	+	-	-	c.b.e	++	Mort. rate cases= 25%<br>Mort. rate controls=5.5%	no data
			Comments	Retrospective study	Hosp onset:<br>37.5% cases,<br>27.3% controls<br>no further info					patients with vertebral osteomyelitis		not matched but controlled in univariate analyses. Differences in age≥65years	not matched but controlled for main diseases. Differences only in diabetes mellitus	all vertebrae		mortality in hospital			
Skin Wound<br>R vs S	1	Alizadeh, 2014	Score	c.b.e	c.b.e	82 cases<br>32 controls	-	-	++	-	++	+	-	-	++	c.b.e	NA	no deaths reported in any group	no data
			Comments	Cross-sectional study	no information available					patients with cutaneous lesions		not matched but controlled in univariate analyses for gender, age distribution was different	underlying conditions were different in the two groups	all skin wound infections		mortality in hospital			
Mixed inf. site<br>R vs NI	1	Balm, 2013	Score	-	HCA	121 cases<br>716 controls	-	+	++	++	++	-	-	-	+	++	++	a) Mort. rate cases= 16.5%<br>Mort. rate controls=4.6%<br>OR=5.49; 95%CI(2.75-10.95)<br>b) Mort. rate cases= 28.9%<br>Mort. rate controls=12.5%<br>OR=2.94; 95%CI(1.78 – 4.85)	no data
			Comments	Retrospective cohort study	All patients were negative to infection when admitted to the hosp	cases had clinical MRSA infection<br>controls were only colonized with MRSA (nasal swab)						not matched and not controlled	not matched and not controlled			a) 30 days<br>b) 6 months	Both ORs, no information on mortality rates comparison	a) 30 days<br>b) 6 months	
	2	Nelson, 2015	Score	++	HCA	a) 3599 cases<br>366144 controls<br>b) 3592 cases<br>3592 controls	-	+	++	++	++	++	++	-	+	++	++	a) aHR=1.420; 95%CI(1.322-1.525)<br>b) aHR=1.370; 95%CI(1.231-1.524)	mean<br>a) cases=22.4<br>controls=9.3<br>b) cases=20.1<br>controls=23.2
			Comments	Retrospective matched cohort study		a) full cohort<br>b) propensity score matched<br>Paper studies the mortality post hosp discharge							propensity score matching			1 year after discharge		a) full cohort<br>b) propensity score matched<br>Data for all-cause mortality 1 year after hosp discharge	
	3	Tran, 2016	Score	++	HCA	a)745 cases<br>17649 controls<br>b)737 cases<br>737 controls	-	+	++	++	++	++	++	-	-	c.b.e	++	a)Mort. rate cases= 8.2%<br>Mort. rate controls=5.7%<br>b)Mort. rate cases= 8% Mort rate controls=7%	mean<br>a)cases=12.8<br>controls=7.6<br>b) cases=11.9<br>controls=9.1
			Comments	Retrospective matched cohort study		a) pre-match cohort<br>b) post-match cohort							propensity score matching			mortality in hospital	only a) was significant	a) pre-match cohort<br>b) post-match cohort	a) pre-match cohort<br>b) post-match cohort
Mixed inf. site<br>R vs S	1	Ericson, 2015	Score	-	c.b.e	1063 cases<br>2825 controls	-	+	+	+	++	++	-	-	-	++	-	a) Mort. rate cases= 11.9%<br>Mort. rate controls=9.6%<br>RR=1.19; 95%CI(0.96-1.49)<br>b) Mort. rate cases= 3.8%<br>Mort. rate controls=3.8%<br>RR=0.90; 95%CI(0.65-1.24)<br>c) Mort. rate cases= 8.7%<br>Mort. rate controls=7.2%<br>RR=1.15; 95%CI(0.90-1.46)	both groups=64
			Comments	Multicentre retrospective study	no information available	invasive infection; included blood, cerebrospinal fluid, abscess, and sterile sites			patients were infants	patients in NICU		not matched but controlled in univariate analyses				a) before discharge<br>b) 7days<br>c) 30 days		a) before discharge<br>b) 7days<br>c) 30 days	
	2	Gimenes, 2016	Score	-	c.b.e	71 cases<br>23 controls	-	-	++	+	++	+	-	-	++	c.b.e	c.b.e	Mort. rate cases= 33.8%<br>Mort. rate controls=52.17%	median (IQR)<br>cases=20 (20)<br>controls=19 (17)
			Comments	Retrospective study	no information available					patients in ICU		not matched but controlled in univariate analyses for age, gender was different				mortality in hospital	no comparison between rates Kaplan Meier survival curve was not significantly different		
	3	Kim E.S, 2014	Score	+	Mix	303 cases<br>357 controls	-	+	++	++	++	c.b.e	c.b.e	-	-	++	-	Mort. rate cases= 16.8%<br>Mort. rate controls=14%<br>OR=1.243; 95%CI(0.813–1.899)	no data
			Comments	Prospective study	difficult to have the right % of remaing patients because 126 were excluded because without a follow-up	infection had comunity onset						no matching and difficult to evaluate the control since 126 patients were escluded because without a follow-up	no matching and difficult to evaluate the control since 126 patients were escluded because without a follow-up			30 days			
Nasalal swab<br>R vs NI	1	Altinbas, 2013	Score	+	HCA	11 cases<br>257 controls	-	-	++	+	++	++	++	-	+	++	-	Mort. rate cases= 18.1%<br>Mort. rate controls=37.3%	mean<br>cases=18<br>controls=8
			Comments	Prospective study						patients in ICU		not matched but controlled in univariate analyses	not matched but controlled in univariate analyses for main diseases, difference only in Gastrostomy and Femoral catheter	all nasal swabs		1 year			
	2	Chan, 2015	Score	+	c.b.e	75 cases<br>117 controls	-	+	+	+	++	-	-	-	++	++	++	a) Mort. rate cases= 64%<br>Mort. rate controls=44.6%<br>b) Mort. rate cases= [illegible] Mort rate controls=18.8%<br>HR=1.96, 95%CI(1.01-3.78)	no data
			Comments	Prospective chort study	no information available				elderly patients	patients were nursing home residents				all nasal swabs		2 years		a) all cause mortality<br>b) infection related mortality	all patients were in nursering home
	3	Chen, 2013	Score	-	c.b.e	90 cases<br>90 controls	-	+	++	+	++	-	-	-	++	c.b.e	-	Mort. rate cases= 12.2%<br>Mort. rate controls=15.6%	mean±SD<br>cases=5.2±7.4<br>controls=5.9±8.8
			Comments	Retrospective cohort study	no information available					patients in ICU				all nasal swabs		mortality in hospital			
	4	Moore, 2014	Score	++	c.b.e	28 cases<br>56 controls	-	++	++	-	++	++	+	-	++	++	-	a) Mort. rate cases=17%<br>Mort. rate controls=7%<br>b) Mort. rate cases= 3.6%<br>Mort. rate controls=0%	no data
			Comments	Matched case-control study	no information available					patients prior renal transplant			not matched but controlled in univariate analyses for Charlson index, some other disease were different	all nasal swabs		5 years		a) all-cause mortality<br>b) infection related mortality	
Resp. Tract<br>R vs NI	1	Minejima, 2014	Score	-	CA	134 cases<br>134 controls	-	+	++	++	++	+	-	-	+	++	++	Mort. rate cases= 22%<br>Mort. rate controls=3%	mean<br>cases=10<br>controls=5
			Comments	Retrospective study	community onset, not specified if 100% CA	patients had non-MRSA pneumonia				patients with penuomonia		not matched but controlled in univariate analyses for gender, age was different				28 days			
Resp. Tract<br>R vs S	1	Hill, 2013	Score	-	c.b.e	no exact data*<br>80 casess<br>60 controls	-	+	++	-	++	++	-	-	++	c.b.e	-	Mort. rate cases= 17.5%<br>Mort. rate controls=25.4%	in ICU<br>mean<br>cases=34<br>controls=35
			Comments	Retrospective study	no information available	*population estimated from graph				trauma patients with VAP		not matched but controlled in univariate analyses		all Resp tract		mortality in hospital			
	2	Tosh, 2013	Score	-	CA	42 cases<br>52 controls	-	+	+	++	++	++	+	-	-	c.b.e	-	Mort. rate cases= 12%<br>Mort. rate controls=2%	mean<br>cases=9<br>controls=6
			Comments	Retrospective study	community onset, not specified if 100% CA				patients ≤50 years	patients with penuomonia		not matched but controlled in univariate analyses	not matched but controlled in univariate analyses for Charlson index			mortality in hospital			
			Score	-	CA	78 cases<br>865 controls*	-	+	++	++	++	+	-	-	+	c.b.e	++	Mort. rate cases= 33.3%<br>Mort. rate controls=21.5%	median (IQR)<br>cases=16.5 ([illegible])<br>controls=11(6–20)

	3	Jung, 2013	Comments	Retrospective study	defined as "non-nosocomial pneumonia"	* controls were patients with non-nosocomial pneumonia due to other bacteria (non-MRSA)				patients with penuomonia		not matched but controlled in univariate analyses for gender, age was differet				mortality in hospital			
	4	Rello, 2013	Score	-	HCA	a) 15 cases<br>30 controls<br>b) 5 cases<br>6 controls	-	++*	++	+	++	-	-	-	-	c.b.e	c.b.e.	a) Mort. rate cases= 33.3%<br>Mort. rate controls=10%<br>b) Mort. rate cases= 60%<br>Mort. rate controls=50%	no data
			Comments	Retrospective comparison of 2 cohorts	patient with HCA and ventilator associated penumonia	a) Europe<br>b) Latin America		*Europe and South America		patients in ICU, with pneumonia						mortality in ICU	no information available		
	5	Self, 2016	Score	+	CA	15 cases<br>22 controls	-	+	++	++	++	-	-	-	-	c.b.e	c.b.e	Mort. rate cases= 13.3%<br>Mort. rate controls=9.1%	median (IQR)<br>cases=9 (8-13)<br>controls=6 (4–10)
			Comments	Prospective study						patients with pneumonia						mortality in hospital	no information available		
Sinuvial fluid<br>R vs S	1	Lin, 2015	Score	-	Mix	38 cases<br>55 controls	-	-	++	-	++	+	++	-	-	c.b.e	-	Mort. rate cases= 5.3%<br>Mort. rate controls=5.5%	no data
			Comments	Retrospective clinical case-series	HCA: 0% cases, 18.4% controls<br>CA: 100% cases, 81.6% controls	all patients had septic arthritis caused by S. aureus				patients with septic arthritis		not matched but controlled in univariate analyses for gender, age was differet		all sinuvial fluid		mortality in hospital			
	2	Minguez, 2015	Score	-	c.b.e	7 cases<br>17 controls	-	-	++	-	++	+	-	-	+	c.b.e	-	Mort. rate cases= 57.1%<br>Mort. rate controls=17.6%	mean<br>cases=33<br>controls=20
			Comments	Retrospective study	no information available	all patients had septic arthritis caused by S. aureus				patients with septic arthritis		not matched but controlled in univariate analyses for gender, age was differet		all sinuvial fluid		mortality in hospital			

Sample size
N refers to total number of cases and controls **(no score)**
Calculation *a priori:* ++ if performed (e.g. based on expected prevalence or on litterature data) or the study refers to large national surveys; - if not performed

Study type — **Study type:** ++ matched cohort or case-control studies; + prospective cohort or case-control studies; - restrospective database studies
Infection type — **HCA:** health-care associated; **CA:** community associated; **Mix:** health-care and community associated together

Representativeness
Geographical: ++ if the study was conducted in an EU/EEA setting; + if the study was conducted in high-income country(ref. World bank); - if the study was not conducted in an EU/EEA or high-income country setting
Demographical: ++ if the study population represents all age groups; + if the study population represents selected age-specific groups (e.g. elderly, infants); - if the study population represents a selected sex category and age-specific groups
Clinical: ++ if the study population represents the overall hospital population; + if the study population partially represents the hospital population; - if the study population represents a disease-specific population (e.g. liver transplanted patients, cancer patients, etc..)
Exlusion/inclusion criteria: ++ if the same exclusion/inclusion criteria were adopted for both cases and controls; + if some (but not all) exclusion/inclusion criteria were adopted for both cases and controls; - if exclusion/inclusion criteria were different in cases and controls

confounders
Demographics: ++ if performed for age and sex; + if performed only for age or only for sex; - if not performed at all
Matching or controlling for Underlying disease: ++ if performed for two or more criteria (e.g. comorbidity score, McCabe score, severity index, n. comorbidities, allergy, immunosuppression status, prior hospitalisation etc..); + if performed for only one criteria; - if not performed at all
Infection site: ++ if performed ; - if not performed
Hospital and unit/ward: ++ if same hospital and same unit/ward; + if same hospital but different unit/ward; - if not same hospital and/or not same unit/ward
Follow-up: ++ if ≥ 28 days; - if < 28 days

Risk difference
Statistically significant outcome results: ++ if results and 95%CI are statistically significant; - if results and 95%CI are not statistically significant
Case fatality and LOS refer to risk difference estimates in cases and controls **(no score)**

Scoring table

++	Matches completely/is completely fulfilled
+	Matches incompletely but sufficiently/is only partly but sufficiently fulfilled
-	Does not match or matches insufficiently/is insufficiently fulfilled
c.b.e.	Cannot be evaluated

VRE

Checklist for the evaluation of the most relevant studies for building antimicrobial resistance (AMR) outcome trees, healthcare-associated infection (HAI) attributable case fatality and attributable length-of-stay (LOS)

BSI
R vs S

Study			Study type	Sample size		Representativeness			Matching or controlling for confounders							Risk difference	
				N (cases;controls)	calculation *a priori*	Geographical	Demographical	Clinical	Exclusion/inclusion criteria	Demographics	Underlying disease	Infection site	Hospital and unit/ward	Follow-up	Statistically significat outcome results	Case fatality	LOS
Nr.	First author, year				□ ++; □ -; □ c.b.e	□ ++; +; -; □ c.b.e	□ ++; □ +; □ -; □ c.b.e	□ ++; -; □ c.b.e	□ ++; □ +; □ -; □ c.b.e	□ ++; □ +; □ -; □ c.b.e	□ ++; □ +; □ -; □ c.b.e	□ ++; □ -; □ c.b.e	□ ++; □ +; □ -; □ c.b.e	□ ++; -; □ c.b.e	□ ++; □ -; □ c.b.e	%; RR; OR; aHR; etc	(days)
1	De Silva, 2014	Score	-	30 cases<br>284 controls	-	-	++	++	++	-	-	-	++	c.b.e	++	OR 2.73; 95% CI: 1.09-7.78	no data
		Comments	Retrospective cohort study									all BSI	same hospital and controlled in univariate analyses fot ward	mortality in hospital			
2	Yoo, 2005	Score	-	19 cases<br>8 controls	-	+	++	-	++	++	+	-	++	c.b.e	-	Mort. rate cases=78.9%<br>Mort. rate controls=62.5%	Mean (SD)<br>Case=56.2 (28.2)<br>Controls=33.4(15.6)
		Comments	Retrospective cohort study					patients who received hematopoietic stem cell transplantation or cytotoxic chemotherapy		not matched but controlled in univariate analyses	not matched but controlled in univariate analyses for SAPS II score	all BSI		post-discharge mortality			
3	Lodise, 2002	Score	++	53 cases<br>53 controls	-	+	++	++	++	++	+	-	++	-	++	a)Mort. rate cases=52.8%<br>Mort. rate controls=26.4%<br>aOR=4.0; 95%CI(1.2–13.3)<br>a)Mort. rate cases=37.7%<br>Mort. rate controls=20.8%<br>aOR=52; 95%CI(1.4-2.0)	After diagnosis<br>Mean (SD)<br>Cases=22.7 (1.88)<br>Controls=15.9 (1.7)
		Comments	Retrospective matched							matched for age and controlled in univariate	matched for APACHE II score	all BSI		7 days		a) all-cause mortality<br>b) infection related mortality	
4	Peset, 2000	Score	+	17 cases<br>169 controls	-	++	++	++	++	++	-	-	+	c.b.e	-	Mort. rate cases=23.5%<br>Mort. rate controls=12.4%<br>OR=2.7; 95%CI(0.47–7.89)	Mean (SD)<br>Cases=74.75 (46.27)<br>Controls=47.26 (37.59)
		Comments	Prospective case-control							not matched but controlled in univariate analyses		all BSI		mortality in hospital			
5	Garbutt, 2000	Score	-	a)46 cases<br>23 controls<br>b) 76 cases<br>50 controls	-	+	++	++	++	-	-	-	+	c.b.e	-	a) Mort. rate cases=48%<br>Mort. rate controls=30%<br>OR=1.74; 95%CI(0.50–6.12)<br>b) Mort. rate cases=43%<br>Mort. rate controls=36%<br>OR=1.28; 95%CI(0.47–3.51)	Mean (SD)<br>Cases=39.7(25.5)<br>Controls=21(16.3)
		Comments	Retrospective cohort study	a) main cohort (2 positive samples)<br>b) full cohort (1 positive sample)<br>all patients of cohort a) are present also in cohort b)								all BSI		mortality in hospital		a) main cohort (2 positive samples)<br>b) full cohort (1 positive sample)<br>all patients of cohort a) are present also in cohort b)	
6	Bhavnani, 2000	Score	c.b.e	150 cases<br>150 controls	-	+	++	++	++	++	++	-	-	++	++	a) Mort. rate cases=52%<br>Mort. rate controls=27%<br>aOR=3.34;95%CI(1.61-6.91)<br>b) Mort. rate cases=32%<br>Mort. rate controls=17%	no data
		Comments	Multicentre case-control (both prospective and retrospective data)							not matched but controlled in univariate analyses	not matched but controlled in univariate analyses for main diseases, differences only in chronic renal failure	all BSI		30 days		a) all-cause mortality<br>b) infection related mortality	
7	Lucas, 1998	Score	c.b.e	93 cases<br>101 controls	-	+	++	++	++	++	-	-	+	c.b.e	++	Mort. rate cases=45%<br>Mort. rate controls=27%<br>aOR=2.07; 95%CI(0.96-4.42)	Mean<br>Cases=49.1 (48.3)<br>Controls=38.6 (31.2)
		Comments	Case-control study							not matched but controlled in univariate analyses		all BSI		mortality in hospital	only mortality rates comparison		
8	Stosor, 1998	Score	-	21 cases<br>32 controls	-	+	++	++	++	-	++	-	+	c.b.e	++	a) Mort. rate cases=76%<br>Mort. rate controls=41%<br>b)Mort. rate cases=38%<br>Mort. rate controls=9%<br>c)Mort. rate cases=24%<br>Mort. rate controls=19%<br>d)Mort. rate cases=14%<br>Mort. rate controls=13%	Mean<br>Cases=34.8 (23.3)<br>Controls=16.7 (16.1)
		Comments	Retrospective comparison								not matched but controlled in univariate analyses for severity of illness score	all BSI		mortality in hospital	only a) and b)	a) overall mortality<br>b) infection directly related mortality<br>c) infection indirectly related mortality<br>d) unrelated mortality	
9	Linden, 1996	Score	-	54 cases<br>48 controls	-	+	++	-	++	-	-	-	++	c.b.e	++	a) Mort. rate cases=57%<br>Mort. rate controls=35%<br>aRR=3.47(1.47-8.19)<br>b) Mort. rate cases=46%<br>Mort. rate controls=25%	After diagnosis<br>Median:<br>Cases=46<br>Controls=19
		Comments	Retrospective cohort study					the patients are either awaiting liver transplantation or have received a liver transplan				all BSI		mortality in hospital		a) all-cause mortality<br>b) infection related mortality	
10	Krcmery, 2001	Score	-	9 cases<br>92 controls	-	++	++	++	++	-	-	-	-	c.b.e	-	Mort. rate cases=22%<br>Mort. rate controls=41.3%	no data
		Comments	Retrospective review				all ages included					all BSI		not specified, but most probably mortality in hospital			
11	Vergis, 2000	Score	+	147 cases<br>251 controls	-	+	++	++	++	+	-	-	-	-	++	Mort. rate cases=33%<br>Mort. rate controls=11%<br>aOR=2.1(1.14-3.88)	median<br>cases=17<br>controls=3
		Comments	Prospective multicentre observational study							not matched but controlled in univariate analyses for gender, age is different		all BSI		14 days			
12	Shay, 1995	Score	-	46 cases<br>46 controls	-	+	++	++	++	-	-	-	+	c.b.e	-	Mort. rate cases=33%<br>Mort. rate controls=6.5%<br>aOR=3.3(0.7-15)	no data
		Comments	Retrospective case-control									all BSI		not specified, but most probably mortality in hospital			
13	Wells, 1995	Score	-	6 cases<br>37 controls	-	+	++	++	++	-	-	-	+	c.b.e	-	Mort. rate cases=17%<br>Mort. rate controls=27%	no data
		Comments	Retrospective case-control									all BSI		mortality in hospital			
14	Cheah, 2013	Score	++	116 cases<br>116 controls	-	+	++	++	++	++	-	-	-	c.b.e	++	Mort. rate cases=36%<br>Mort. rate controls=26%<br>aOR=1.21; 95%CI(0.52-2.79)	Median (IQR)<br>cases=35(22.5–50.5)<br>controls=25(16–45.5)
		Comments	Retrospective matched cohort study							not matched but controlled in univariate analyses		all BSI		mortality in hospital	only the comparison between the mortality rates		
15	DiazGranados, 2005(a)	Score	-	22 cases<br>61 controls	++	+	++	-	++	++	+	-	+	++	++	Mort. rate cases=63.6%<br>Mort. rate controls=41%<br>aHR=4.97; 95%CI(1.21-20.44)	Median<br>Cases=42 (24-75)<br>Controls=31 (2-72)
		Comments	Retrospective cohort study					patients with neutropenia		not matched but controlled in univariate analyses	not matched but controlled in univariate analyses for APACHE II score	all BSI		60 days	only HR		
16	Peel 2012	Score	-	80 resistant<br>80 sensitive<br>80 NI	-	+	++	++	++	-	-	-	++	c.b.e	++	Mort. rate resistant=37.5%<br>Mort. rate sensitive=23.8%<br>Mort. rate NI=2.5%	no data
		Comments	Case-case-control study									all BSI		not specified, but most probably mortality in hospital	in R and S higher than in NI		
17	Mainous, 1997	Score	-	10 cases<br>31 controls	-	+	++	+	++	-	-	NA	++	c.b.e	-	Mort. rate cases=40%<br>Mort. rate controls=39%	mean (SD)<br>cases=28 (18)<br>controls=12 (10)
		Comments	Retrospective cohort study					patients in ICU						mortality in hospital			
18	Salgado, 2003	Score	++	a)856 cases<br>1180 controls	++	NA	++	++	++	NA	NA	-	-	NA	++	a)Mort. rate cases=41.5(0-66.7)%<br>Mort. rate controls=47.8(17-100)%<br>b)Mort. rate cases=45.2%<br>Mort. rate controls=19.0%<br>RR=2.38; 95%CI(2.13-2.66)<br>c) Mort. rate cases=48.9%<br>Mort. rate controls=19%<br>RR=2.57; 95%CI(2.27-2.91)<br>d) Mort. rate cases=28.7%<br>Mort. rate controls=10.9%<br>RR=2.62; 95%CI(1.84-3.73)<br>e) Mort. rate cases=39.1%<br>Mort. rate controls=21.8%<br>RR=1.79; 95%CI(1.28-1-5)<br>f) Attributable mortality =17-46%	Excess of total stay of VRE vs VSE=10-46 days
		Comments	Review and Meta-analyses	a)pooled cases (14 studies) and controls (26 studies)		articles from different countries				diffent in different studies	diffent in different studies	all BSI			b), c), d) and e)	a)crude mortality rates of pooled cases (14 studies) and controls (26 studies)<br>b) pooled cases and controls from 13 studies<br>c) pooled cases and controls from 9 studies with higher sample size<br>d) pooled cases and controls from 5 studies with infection related mortality<br>e) studies of point d) excluded one that was producing heterogeneity<br>f) range of values from 4 studies which matched for severity of illness	
19	DiazGranados, 2005(b)	Score	++	683 cases<br>891 controls	-	NA	++	++	++	NA	++	-	-	NA	++	OR=2.52; 95%CI(1.9–3.4)	
		Comments	Review and Meta-analyses	pooled cases and controls from 9 studies		articles from different countries		1 study neutropenic patiens, 1 liver transplant patients but controlled with sensitivity analyses		diffent in different studies	studies matched or controlled for severity of illness	all BSI					
20	CDC, 1993	Score	-	71 cases<br>1810 controls	-	+	++	++	++	-	-	-	-	c.b.e	-	Mort. rate cases=36.6%<br>Mort. rate controls=16.4%	no data
		Comments	Surveillance study									all BSI		not specified, but most probably mortality in hospital			
21	Lautenbach, 1999	Score	-	72 cases<br>188 controls	-	+	++	++	++	-	-	-	+	c.b.e	c.b.e	Mort. rate cases=45.8%<br>Mort. rate controls=33.5%	median (range)<br>cases= 23 (19.18-27.82)<br>controls= 12 (9.0-15.0)
		Comments	Historical cohort study									all BSI		not specified, but most probably mortality in hospital	mortality rates were not compared and resistance was not significantly associated with mortality once severity of illness and age were taken into account		

	22	Stroud, 1996	Score	-	26 cases<br>119 controls	-	+	++	++	++	-	-	-	-	c.b.e	c.b.e	Mort. rate cases=69%<br>Mort. rate controls=76%	overall cases+controls mean(range)=81(9-583)<br>median=60
			Comments	Retrospective cohort study				patient>16 years					all BSI		not specified, but most probably mortality in hospital	no information	information extracted from available data in the article	
BSI<br>R vs NI	1	Song, 2003	Score	++	159 cases<br>159 controls	++	+	++	++	++	++	+	NA	++	c.b.e	++	Mort. rate cases=50.3%<br>Mort. rate controls=27.7%<br>aOR=3.04; 95%CI(1.66-5.53)	Median<br>Cases=53<br>Controls=28
			Comments	Population based matched cohort study								matched for APR-DRG complexity levels			mortality in hospital			
	2	Carmeli, 2002	Score	++	233 cases<br>647 controls	-	+	++	++	++	++	-	NA	+	c.b.e	++	RR= 12.3<br>Attributable mortality=25%	Multiplicative effect: 2.06
			Comments	Matched cohort study	total sample						not matched but controlled in univariate analyses				mortality in hospital		infection site specific results	
	3	Edmond, 1996	Score	++	27 cases<br>27 controls	-	+	++	++	++	++	++	NA	+	c.b.e	++	Mort. rate cases=67%<br>Mort. rate controls=30%<br>OR=2.3; 95%CI(1.2-4.1)	median<br>cases=31<br>controls=36
			Comments	Matched retrospective cohort											mortality in hospital			
	4	Edmond, 1995	Score	++	11 cases<br>22 controls	-	+	++	-	++	++	-	NA	++	c.b.e	-	Mort. rate cases=73%<br>Mort. rate controls=30%	Mean<br>Cases=74.75± 46.27<br>Controls=47.26±37.59
			Comments	Pairwise matched case-control study					Patients with tumor		not matched but controlled in univariate analyses	not matched but all patient with cance			mortality in hospital			
	5	Butler, 2010	Score	-	a)94 cases<br>182 cases<br>20150 controls	-	+	++	+	++	++	++	NA	+	c.b.e	++	Mort. rate resistant= 32%<br>Mort. rate sensitive=17%<br>Mort. rate controls=4%	Attributable LOS VRE=2.2 (1.7-2.7)<br>Attributable LOS VSE=1.1 (0.9-1.4)
			Comments	Retrospective cohort study					nor surgical units			matched for propensity score			mortality in hospital			
UTI<br>R vs S	1	Khair, 2013	Score	+	74 cases<br>180 controls	++	+	++	++	++	++	-	-	+	c.b.e	-	Mort. rate cases=14%<br>Mort. rate controls=7%	after bacteruria<br>mean (range)<br>cases= 6.8(0.1-150.8)<br>controls= 5(0.2-78.2)
			Comments	Prospective cohort study							not matched but controlled in univariate analyses		all UTI		mortality in hospital			
UTI<br>R vs NI	1	Carmeli, 2002	Score	++	233 cases<br>647 controls	-	+	++	++	++	++	-	NA	+	c.b.e	++	RR=4.28<br>Attributable to VRE=9%	Multiplicative effect=1.75<br>Attributable=5.4 days
			Comments	Matched cohort study	total sample						not matched but controlled in univariate analyses				mortality in hospital		infection site specific results	
SSTs<br>R vs NI	1	Carmeli, 2002	Score	++	233 cases<br>647 controls	-	+	++	++	++	++	-	NA	+	c.b.e	-	RR=2.00<br>Attributable to VRE=6%	Multiplicative effect=1.78<br>Attributable=6.2 days
			Comments	Matched cohort study	total sample						not matched but controlled in univariate analyses				mortality in hospital		infection site specific results	
Other<br>R vs NI	1	Carmeli, 2002	Score	++	647 controls	-	+	++	++	++	++	-	NA	+	c.b.e	++	RR=3.75<br>Attributable to VRE=3%	Multiplicative effect=1.42<br>Attributable=2.6 days
			Comments	Matched cohort study	total sample						not matched but controlled in univariate analyses				mortality in hospital		infection site specific results	
Mixed<br>R vs S	1	Tornieporth, 1996	Score	c.b.e	145 cases<br>145 controls	-	+	++	++	++	+	+	++	+	c.b.e	-	a)Mort. rate cases=37%<br>Mort. rate controls=19%<br>b)Mort. rate cases=58%<br>Mort. rate controls=43%	mean (range)<br>cases=64.5 (2-290)<br>controls=40.8 (2-248)<br>median<br>cases= 48<br>controls=26
			Comments	Case-control study							not matched but controlled in univariate analyses for gender, age is different	not matched but controlled in univariate analyses for main diseases, though there were some differences	not matched but controlle in univariate analyses by source of speciment		mortality in hospital		a) overall mortality<br>b) ICU admitted patients mortality	

Sample size
N refers to total number of cases and controls **(no score)**
Calculation *a priori*: ++ if performed (e.g. based on expected prevalence or on litterature data) or the study refers to large national surveys; - if not performed

Study type
Study type: ++ matched cohort or case-control studies; + prospective cohort or case-control studies; - restrospective database studies

Representativeness
Geographical: ++ if the study was conducted in an EU/EEA setting; + if the study was conducted in high-income country(ref. World bank); - if the study was not conducted in an EU/EEA or high-income country setting
Demographical: ++ if the study population represents all age groups; + if the study population represents selected age-specific groups (e.g. elderly, infants); - if the study population represents a selected sex category and age-specific groups
Clinical: ++ if the study population represents the overall hospital population; + if the study population partially represents the hospital population; - if the study population represents a disease-specific population (e.g. liver transplanted patients, cancer patients, etc..)
Exlusion/inclusion criteria: ++ if the same exclusion/inclusion criteria were adopted for both cases and controls; + if some (but not all) exclusion/inclusion criteria were adopted for both cases and controls; - if exclusion/inclusion criteria were different in cases and controls

Matching or controlling for confounders
Demographics: ++ if performed for age and sex; + if performed only for age or only for sex; - if not performed at all
Underlying disease: ++ if performed for two or more criteria (e.g. comorbidity score, McCabe score, severity index, n. comorbidities, allergy, immunosuppression status, prior hospitalisation etc..); + if performed for only one criteria; - if not performed at all
Infection site: ++ if performed ; - if not performed
Hospital and unit/ward: ++ if same hospital and same unit/ward; + if same hospital but different unit/ward; - if not same hospital and/or not same unit/ward
Follow-up: ++ if ≥ 28 days; - if < 28 days

Risk difference
Statistically significant outcome results: ++ if results and 95%CI are statistically significant; - if results and 95%CI are not statistically significant
Case fatality and LOS refer to risk difference estimates in cases and controls **(no score)**

Scoring table

++	Matches completely/is completely fulfilled
+	Matches incompletely but sufficiently/is only partly but sufficiently fulfilled
-	Does not match or matches insufficiently/is insufficiently fulfilled
c.b.e.	Cannot be evaluated

PRSP

Checklist for the evaluation of the most relevant studies for building antimicrobial resistance (AMR) outcome trees, healthcare-associated infection (HAI) attributable case fatality and attributable length-of-stay (LOS)

	Study			Study type	Sample size		Representativeness			Matching or controlling for confounders							Risk difference	
					N (cases;controls)	calculation *a priori*	Geographical	Demographical	Clinical	Exclusion/inclusion criteria	Demographics	Underlying disease	Infection site	Hospital and unit/ward	Follow-up	Statistically significat outcome results	Case fatality	LOS
	Nr.	First author, year				□ ++; □ -; □ c.b.e	□ ++; +; -; □ c.b.e	□ ++; □ +; □ -; □ c.b.e	□ ++; -; □ c.b.e	□ ++; □ +; □ -; □ c.b.e	□ ++; □ +; □ -; □ c.b.e	□ ++; □ +; □ -; □ c.b.e	□ ++; □ -; □ c.b.e	□ ++; □ +; □ -; □ c.b.e	□ ++; -; □ c.b.e	□ ++; □ -; □ c.b.e	%; RR; OR; aHR; etc	(days)
BSI<br>R vs S	1	Yu, 2003	Score	+	76 cases<br>598 controls	-	NA	++	++	++	-	-	-	-	-	-	Mort. rate case=23.7%<br>Mort. rate controls=15.1%	no data
			Comments	Prospective international observational study			global						all BSI		>14 days			
Resp.<br>Tract<br>R vs S	1	Watanabe, 2000	Score	-	24 cases<br>25 controls	-	+	++	+	++	++	++	-	+	-	-	a) Mort. rate case=0%<br>Mort. rate controls=8%<br>b) Mort. rate case=12.5%<br>Mort. rate controls=20%	no data
			Comments	Retrospective comparative study					patients with pneumonia		not matched but controlled in univariate analyses	not matched but controlled in univariate analyses	all resp tract inf, 1 control had BSI also		7 days after treatment		a) infection related mortality<br>b) mortality due to complications	
	1	Gouveia, 2011	Score	+	93 cases<br>548 total population	-	-	++	-	++	-	-	-	+	c.b.e	-	aHR=1.62; 95%CI (1.08-2.43)	no data
			Comments	Prospective observational study				all ages	patients with meningitis						mortality in hospital			
	2	Reechaipichitkul, 2006	Score	-	22 cases<br>42 controls	-	-	++	+	++	-	-	-	+	c.b.e	-	a)Mort. rate case=9.1%<br>Mort. rate controls=11.9%<br>b)Mort. rate case=9.1%<br>Mort. rate controls=9.5%	Mean(SD)<br>cases= 12 (9)<br>controls= (17.7)
			Comments	Cross-sectional					patients with CA pneumonia						mortality in hospital		a) all-cause mortality<br>b) pneumonia related mortality	
	3	Sun, 2006	Score	-	44 cases<br>168 controls	-	+	++	+	++	++	++	-	+	c.b.e	-	Mort. rate case=9%<br>Mort. rate controls=14%	median (range)<br>cases=6 (4–9.5)<br>6 (4–10.5)
			Comments	Retrospective cohort study	cases are non susceptible				patients with CA pneumonia		not matched but controlled in univariate analyses	not matched but controlled in univariate analyses for main diseases			mortality in hospital			
	4	Song, 2004	Score	-	69 cases<br>105 controls	-	NA	++	+	++	-	-	-	-	++	-	Mort. rate case=16%<br>Mort. rate controls=12.4%<br>OR=1.3; 95%CI(0.5–3.4)	no data
			Comments	Retrospective cohort study			Asia		patients with pneumonia						30 days			
	5	Einarsson, 1998	Score	-	36 cases<br>36 controls	-	++	++	+	++	++	+	-	+	c.b.e	-	Mort. rate case=11%<br>Mort. rate controls=5.5%	Mean<br>Cases= 26.8<br>Controls= 11.5
			Comments	Retrospective case-control study					patients with pneumonia			not matched but controlled in univariate analyses fot APACHE II score, differences in chronic diseases			not defined but most probably mortality in hospital			
	6	Sangthawan, 2003	Score	+	19 cases<br>27 controls	-	-	++	+	++	-	-	-	+	c.b.e	-	Mort. rate case=36.8%<br>Mort. rate controls=18.5%	no data
			Comments	Prospective study	cases were resistant and non susceptible			patients>15 years	patients with pneumonia						not defined but most probably mortality in hospital			
	7	Falco, 2004	Score	c.b.e	15 cases<br>181 controls	-	++	++	+	++	-	-	-	+	c.b.e	-	a)Mort. rate case=26.7%<br>Mort. rate controls=12.7%<br>b)Mort. rate case=13.3%<br>Mort. rate controls=9.9%	no data
			Comments	Retrospective and prospective study					patients with pneumonia						mortality in hospital		a) all-cause mortality<br>b) pneumonia related mortality	
	8	Pallares, 1995	Score	+	47 cases<br>440 controls	-	++	++	+	++	-	-	-	+	c.b.e	++	Mort. rate case=<br>Mort. rate controls=<br>aOR=1.0; 95%CI(0.5-1.9)	no data
			Comments	Prospective study					patients with pneumonia						mortality in hospital	only the comparison between the mortality rates		
	9	Ewig, 1999	Score	+	33 cases<br>52 controls	-	++	++	+	++	-	-	-	+	c.b.e	-	Mort. rate case=18%<br>Mort. rate controls=5.8%	no specific data
			Comments	Prospective study					patients with CA pneumonia						mortality in hospital			
	10	Aspa, 2004	Score	+	64 cases<br>409 controls	-	++	++	+	++	-	-	-	-	++	-	Mort. rate case=18.3%<br>Mort. rate controls=12.2%	no data
			Comments	Prospective multi-centre study				patients>16 years	patients with pneumonia						30 days			
	11	Faikin, 2000	Score	-	a) 37 cases<br>b) 20 cases<br>383 controls	-	+	++	+	++	-	-	-	-	++	-	a) OR= 1.3; 95%CI(0.5-3.7)<br>b) OR=2.3 ; 95%CI(0.7-7.4)	no data
			Comments	Population-based surveillance study	a) resistant<br>b) highly resistant, MIC>4				patients with CA pneumonia						30 days		a) resistant<br>b) highly resistant, MIC>4	
	12	Tjeyjeh, 2006	Score	+	10 studies	-	NA	++	+	++	NA	NA	-	-	c.b.e	++	a) Mort. rate non susceptible=19.4%<br>Mort. rate controls=15.7%<br>b)OR non-suscptible=1.3; 95%CI(1.08–1.59)<br>OR intermediate=1.34; 95% CI(1.13–1.60)<br>OR resistant=1.29; 95% CI(1.01–1.66)<br>c) aRR non susceptible=1.29; 95%CI(1.04–1.59)	no data
			Comments	Review and meta-analyses	some inlcuded also non susceptible				patients with CA pneumonia						different based on the inlcuded studies		a) pooled mortality rate<br>b) all cause mortality<br>c) combined adjusted (6 studies)	
	13	Yigla, 1995	Score	+	7 cases<br>15 controls	-	+	++	+	++	++	++	-	+	c.b.e	-	Mort. rate case=43%<br>Mort. rate controls=33%	no data
			Comments	Prospective study					patients with pneumonia		not matched but controlled in univariate analyses	not matched but controlled in univariate analyses for main diseases			mortality in hospital			
	14	Pallares, 2002	Score	+	77 cases<br>300 controls	-	++	++	++	++	-	-	-	+	++	c.b.e	Mort. rate case=43%<br>Mort. rate controls=20.3%	no data
			Comments	Prospective study	data extrapolated from the articles table 3				patients with nonmeningeal systemic pneumococcal infections						30 days	no information on the article because is focused on cephalosporin resistance, data extrapolated		
	15	Jehl, 2002	Score	-	49 cases<br>246 controls	-	++	++	+	++	-	-	-	-	++	-	a)Mort. rate case=8.3%<br>Mort. rate controls=3.5%<br>b)Mort. rate case=4.2%<br>Mort. rate controls=10.4%<br>c)Mort. rate case=12.5%<br>Mort. rate controls=13.9%	no data
			Comments	Prospective survey					patients with pneumonia						a) 3 days<br>b) 30 days<br>c) total		a) 3 days<br>b) 30 days<br>c) total	

Sample size
N refers to total number of cases and controls **(no score)**
Calculation *a priori*: ++ if performed (e.g. based on expected prevalence or on litterature data) or the study refers to large national surveys; - if not performed

Study type
Study type: ++ matched cohort or case-control studies; + prospective cohort or case-control studies; - restrospective database studies

Representativeness
Geographical: ++ if the study was conducted in an EU/EEA setting; + if the study was conducted in high-income country(ref. World bank); - if the study was not conducted in an EU/EEA or high-income country setting
Demographical: ++ if the study population represents all age groups; + if the study population represents selected age-specific groups (e.g. elderly, infants); - if the study population represents a selected sex category and age-specific groups
Clinical: ++ if the study population represents the overall hospital population; + if the study population partially represents the hospital population; - if the study population represents a disease-specific population (e.g. liver transplanted patients, cancer patients, etc..)

Matching or controlling for confounders
Exlusion/inclusion criteria: ++ if the same exclusion/inclusion criteria were adopted for both cases and controls; + if some (but not all) exclusion/inclusion criteria were adopted for both cases and controls; - if exclusion/inclusion criteria were different in cases and controls
Demographics: ++ if performed for age and sex; + if performed only for age or only for sex; - if not performed at all
Underlying disease: ++ if performed for two or more criteria (e.g. comorbidity score, McCabe score, severity index, n. comorbidities, allergy, immunosuppression status, prior hospitalisation etc..); + if performed for only one criteria; - if not performed at all
Infection site: ++ if performed ; - if not performed
Hospital and unit/ward: ++ if same hospital and same unit/ward; + if same hospital but different unit/ward; - if not same hospital and/or not same unit/ward
Follow-up: ++ if ≥ 28 days; - if < 28 days

Risk difference
Statistically significant outcome results: ++ if results and 95%CI are statistically significant; - if results and 95%CI are not statistically significant
Case fatality and LOS refer to risk difference estimates in cases and controls **(no score)**

Scoring table

++	Matches completely/is completely fulfilled
+	Matches incompletely but sufficiently/is only partlybut sufficiently fulfilled
-	Does not match or matches insufficiently/is insufficiently fulfilled
c.b.e.	Cannot be evaluated

Mixed
R vs S

Burden of antimicrobial resistance

Final disease outcome trees

Written by Alessandro Cassini and Diamantis Plachouras with contributions from Brecht Devleesschauwer, Daniel Lewandowski (for addressing comorbidities), Ana Hoxha, Carl Suetens, Liselotte Diaz Högberg, and Dominique Monnet

Contents

List of abbreviations 171

1. Disease outcome trees – baseline syndromic models 172
- 1.1. Blood-stream infections 172
- 1.2. Respiratory tract infections 173
- 1.3. Urinary tract infections 174
- 1.4. Surgical site infections 175
- 1.5. Other site infections 175

2. *Klebsiella pneumoniae* resistant to third-generation cephalosporin infections 177
- 2.1. Blood-stream infections 177
- 2.2. Respiratory tract infections 177
- 2.3. Urinary tract infections 177
- 2.4. Surgical site and other site infections 177

3. *Klebsiella pneumoniae* resistant to carbapenem infections 177
- 3.1. Blood-stream infections 177
- 3.2. Respiratory tract and surgical site infections 178
- 3.3. Urinary tract infections 178
- 3.4. Other site infections 178

4. *Klebsiella pneumoniae* resistant to colistin infections 178
- 4.1. All infection sites 178

5. *Escherichia coli* resistant to third-generation cephalosporin infections 179
- 5.1. Blood-stream infections 179
- 5.2. Urinary tract infections 179
- 5.3. Surgical site, respiratory tract and other site infections 179

6. *Escerichia coli* resistant to carbapenem infections 179
- 6.1. All infection sites 179

7. *Escerichia coli* resistant to colistin infections 180
- 7.1. All infection sites 180

8. Multidrug resistant *Acinetobacter* infections 180
- 8.1. Blood-stream infections 180
- 8.2. Respiratory tract infections 180
- 8.3. Urinary tract, surgical site and other site infections 180

9. Carbapenem resistant *Acinetobacter* infections 181
- 9.1. Blood-stream infections 181
- 9.2. Respiratory tract infections 181
- 9.3. Urinary tract, surgical site and other site infections 181

10. Colistin resistant *Acinetobacter* infections 181
- 10.1. Blood-stream infections 181
- 10.2. Urinary tract infections 181
- 10.3. Respiratory tract, surgical site and other site infections 181

11. Multidrug resistant *Pseudomonas aeruginosa* infections 182

11.1. Blood-stream infections ... 182
11.2. Respiratory tract infections ... 182
11.3. Urinary tract, surgical site and other site infections ... 182
12. Carbapenem resistant *Pseudomonas aeruginosa* infections ... 182
12.1. Blood-stream infections ... 182
12.2. Respiratory tract infections ... 183
12.3. Urinary tract, surgical site and other site infections ... 183
13. Colistin resistant *Pseudomonas aeruginosa* infections ... 183
13.1. All infection sites ... 183
14. Meticillin resistant *Staphylococcus aureus* infections ... 183
14.1. Blood-stream infections ... 183
14.2. Respiratory tract infections ... 184
14.3. Urinary tract, surgical site and other site infections ... 184
15. Vancomycin resistant *Enterococcus faecalis* and *faecium* infections 184
15.1. Blood-stream infections ... 184
15.2. Respiratory tract, urinary tract, surgical site and other site infections ... 184
16. Penicillin resistant *Streptococcus pneumoniae* infections ... 185
16.1. Blood-stream infections ... 185
16.2. Respiratory tract, urinary tract, surgical site and other site infections ... 185
17. Macrolide and penicillin resistant *Streptococcus pneumoniae* infections ... 185
17.1. All infection sites ... 185
References ... 186

List of abbreviations

3GCREC	third-generation cephalosporin-resistant *E. coli*
3GCRKP	third-generation cephalosporin-resistant *K. pneumoniae*
AMR	antimicrobial resistance
BSI	bloodstream infection
CAI	Community-associated infections
CI	confidence interval
ColRACI	colistin-resistant *Acinetobacter* spp.
ColREC	colistin-resistant *E. coli*
ColRKP	colistin-resistant *K. pneumoniae*
ColRPA	colistin-resistant *P. aeruginosa*
CRACI	carbapenem-resistant *Acinetobacter* spp.
CREC	carbapenem-resistant *E. coli*
CRKP	carbapenem-resistant *K. pneumoniae*
CRPA	carbapenem-resistant *P. aeruginosa*
CSF	cerebral spine fluid
DALY	disability-adjusted life years
EARS-Net	European Antimicrobial Resistance Surveillance Network
EEA	European Economic Area
EU	European Union
HAI	healthcare-associated infection
LOS	length of stay
MDR	multidrug-resistant
MDRACI	multidrug-resistant *Acinetobacter* spp
MDRPA	multidrug-resistant *P. aeruginosa*
MRSA	meticillin-resistant *Staphylococcus aureus*
MS	Member State
OECD	Organisation for Economic Co-operation and Development
OTH	other infection site including digestive tract infections, skin and soft tissue infections (SSTI), eye, ear, nose or mouth infections, bone and joint infections, cardiovascular infections, reproductive tract infections and other less frequent infections
PMRSP	penicillin- and macrolide-resistant S. pneumoniae
PPS	point prevalence survey of healthcare-associated infections and antimicrobial use in acute care hospitals
PRSP	penicillin-resistant *S. pneumoniae*;
RESP	respiratory infections (including pneumonia, and low respiratory tract infection)
S-BSI	secondary BSI
SP	specified pathogens
SPDAR	specified pathogens with defined antimicrobial resistance
SSI	surgical site infection
UTI	urinary tract infection
VRE	vancomycin-resistant enterococci

1. Disease outcome trees – baseline syndromic models

The systematic review of the literature on healthcare-associated infections was the main reference for the development of the baseline models (RKI, 2016). The baseline models were built starting from the models already published for the burden of HAIs (Cassini & Plachouras, 2016). Disability weights for recurrent health outcomes between the burden of HAIs and the burden of AMR studies remained unvaried, except when corrections accounting for comorbidity were addressed (see below). Disability weights were derived from the European disability weight study (Haagsma, 2015).

A summary of disease health outcome parameters (case fatality proportion [CFP] and length of stay [LOS]) are available, see Table 1.

The outcome tree for blood-stream infections (BSI) had a number of long-term sequelae that could happen at the same time. The issue of co-morbidities has been addressed in order to account for concurrent health outcomes and with the aim to estimate their related transition probabilities and disability weights.

We assumed that the health outcomes were independent. Therefore, considering that x health outcomes will result in 2^x possible combinations, we estimated 2^4=16 possible combinations. However, considering that physical impairment is always present as a consequence of complicated BSI infection (and discarding the ones without any transition), we considered 8 possible health outcomes, see Figure 1. The probability of each of these combinations is the product of the probability of observing each individual health state. Moreover, we also took the uncertainties around the original risk of developing the long-term sequelae through a Monte Carlo simulation approach, which implied simulating four vectors of random values for each probability and then performed calculations for each quadruplet of simulated values. One important assumption was internal comorbidity: the health outcomes are assumed to be due to a single exposure (infection with BSI) and not to multiple exposures (external comorbidity). The resulting estimated transition probabilities, median, 2.5% and 97.5% intervals, for long-term sequelae following complicated BSI infection are described in Figure 1.

As far as correcting for comorbidity of disability weights is concerned, we chose to follow the multiplicative approach (Haagsma, 2011). Here again we took uncertainties into account through Monte Carlo simulations, which resulted in a median, 2.5% and 97.5% intervals included in the related health outcomes, see Figure 1. The multiplicative approach is recommended for combining disability weights of simultaneously occurring health states. The approach allows for an increase of disability when health states are combined, while preventing the total disability level to become larger than 1, which would equal to "worse than death".

1.1. Blood-stream infections

The baseline attributable case fatality proportion (CFP) for blood-stream infections (BSI) was set to 7.1% (Oleachea, 2013; corresponding to mortality due to non-resistant bacterial infections as defined in the study) to 20.3% (Renaud, 2001).

The baseline attributable length of stay (LOS) was set to 5.87-11.54 days based on an extensive European study on Enterobacteriaceae and *Staphylococcus aureus* (Stewardson, 2016).

The risk of complications remained the same as for the Burden of HAIs as no specific information related to resistance patterns of the bacterial infections were available.

Figure 1. Baseline disease outcome tree for BSI

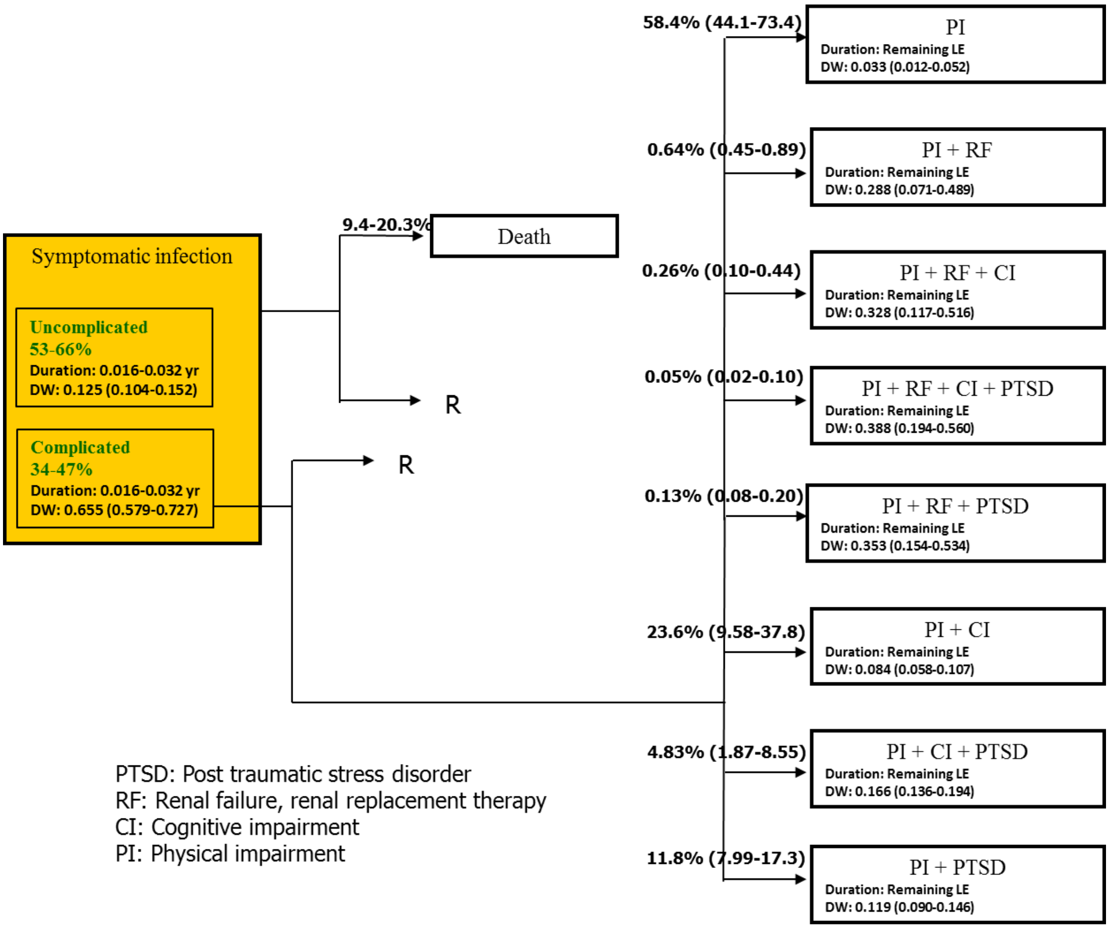

1.2. Respiratory tract infections

In order to estimate the CFP of respiratory infections for the baseline model, we analysed the data form the HAI-Net ICU from 2008 to 2012. After excluding all pneumonia infections developing secondary BSI and performing a propensity score matching (successful for 96.5% of pneumonia without secondary BSI), the attributable mortality was 3.6% (95% CI 2.7-4.5) and the median attributable LOS was 14 days.

The lower value of the attributable LOS remained the same as for the Burden of HAIs (7 days), as it was deemed that the propensity score matching was not ideal to estimate LOS; the higher value was set to 14 days.

The burden of HAI study considered that 39% of all patients develop severe sepsis/septic shock. Assuming that all patients with bacteraemia also have severe/septic shock, we calculated the number of patients developing secondary bacteraemia after pneumonia from the 2014 ICU-acquired infection surveillance data (ECDC, 2017). In this study 5,852 pneumonia episodes and 654 bacteraemia secondary to pneumonia were reported representing 11% of the episodes. Therefore, in order not to double-count bacteraemia we chose to subtract 11% from the 39% of risk of developing severe sepsis/septic shock. The risk of developing severe sepsis/septic shock is set to 28% and the risk of developing long-term sequelae remains the same as for the baseline BSI outcome tree described above.

Figure 2. Baseline disease outcome tree for respiratory infections

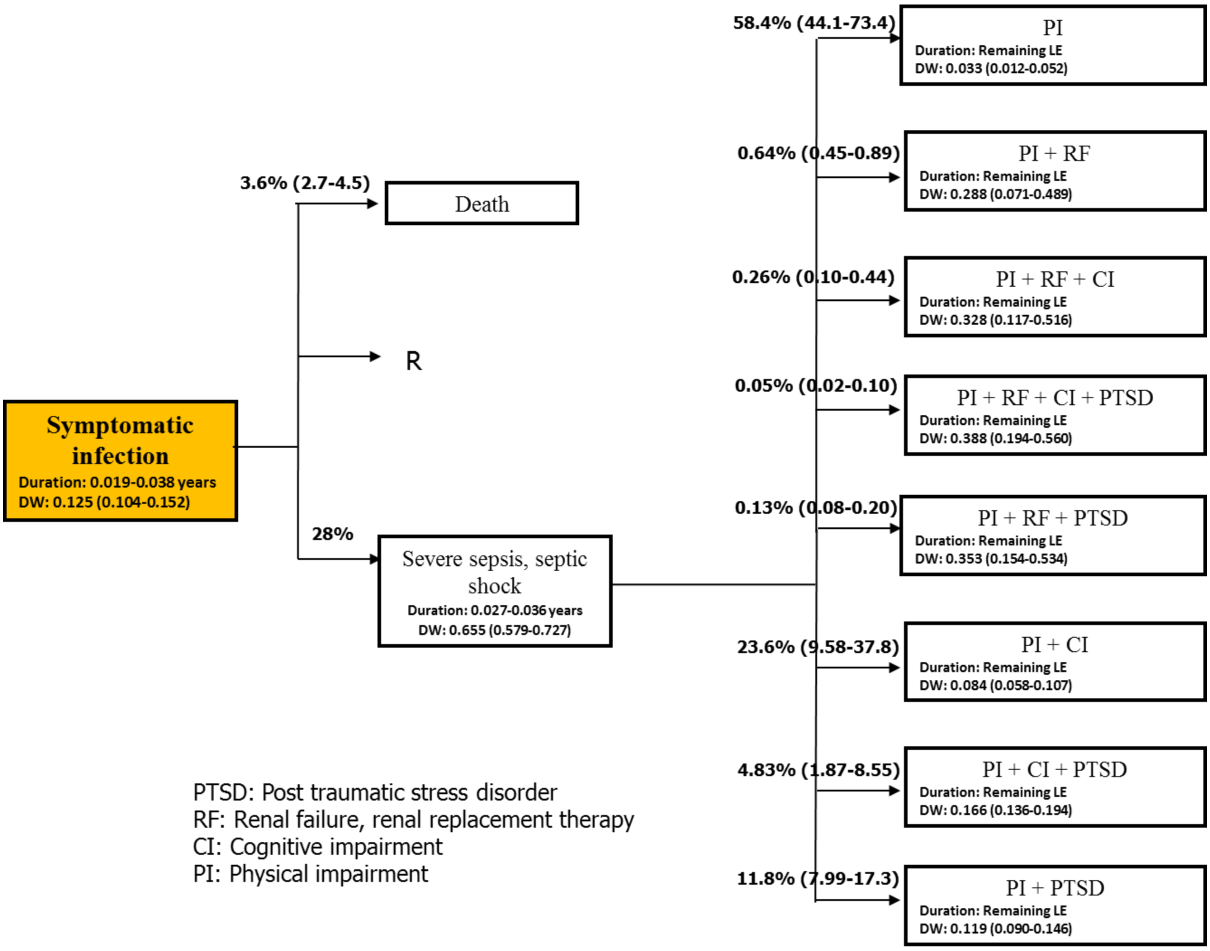

1.3. Urinary tract infections

It was considered that there is no risk of death due to urinary tract infections (UTIs) without development of secondary BSI (S-BSI). Therefore, the model does not include attributable CFP.

Limited evidence was available on the attributable LOS due to UTIs. Therefore, we chose to estimate the additional LOS caused by the infection from information available in the PPS 2011-2012 (ECDC, 2013). We estimated the median LN-INT, where LN is the length of stay of patients with an HAI, and INT is the length of stay before the onset of the HAI. All UTI infections were included except the resistant pathogens included in our study. Uncertainty intervals were derived by the inter-quartile range (IQR). The baseline attributable LOS was set to 7 days (4-11). Considering a high variability between cases, it was chosen to apply the 2.5% lower bound disability weight for “infectious disease, acute episode, moderate” and the 97.5% higher bound disability weight for “infectious disease, acute episode, severe”.

Figure 3. Baseline disease outcome tree for UTIs

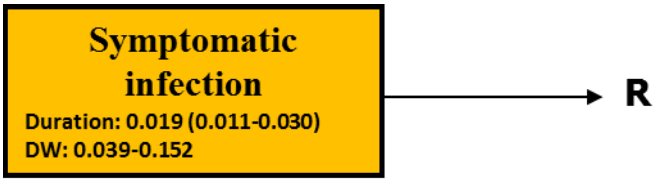

1.4. Surgical site infections

Attributable CFP remained the same as for the burden of HAIs study becaue no specific information related to bacterial infections susceptible to antibiotics were available. Moreover, death as an outcome of SSI without developing a secondary BSI was considered possible.

Attributable LOS was set to an average of 8.5 days (0-15.2 days) (Astagneau, 2001); this was lower than the attributable LOS chosen for the Burden of HAIs study because we did not exclude superficial SSI from the present burden of AMR study. Considering a high variability between cases, it was chosen to apply the 2.5% lower bound disability weight for “infectious disease, acute episode, moderate” and the 97.5% higher bound disability weight for “infectious disease, acute episode, severe”.

Figure 4. Baseline disease outcome tree for SSIs

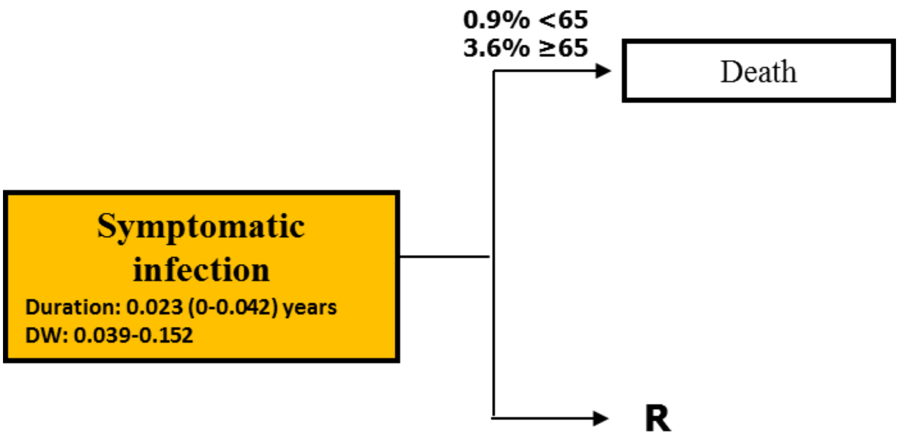

1.5. Other site infections

Other site infections include digestive tract infections, skin and soft tissue infections (SSTI), eye, ear, nose or mouth infections, bone and joint infections, cardiovascular infections, reproductive tract infections and other less frequent infections.

Deaths from these infections without concomitant BSI were considered sufficiently rare. In combination with the lack of reliable data, we chose not to consider CFP in this baseline model.

Given the lack of published evidence and the variability of OTH site infections, the additional LOS caused by the infection was derived from information available in the PPS 2011-2012 (ECDC, 2013). For the OTH infection sites, we estimated the median LN-INT, where LN is the length of stay of patients with an HAI, and INT is the length of stay before the onset of the HAI. All infections defined above as OTH were included except the resistant pathogens included in our study. Uncertainty intervals were derived by the inter-quartile range (IQR). The baseline attributable LOS was set to 6 days (3-11). Considering a high variability between cases, it was chosen to apply the 2.5% lower bound disability weight for “infectious disease, acute episode, moderate” and the 97.5% higher bound disability weight for “infectious disease, acute episode, severe”

Figure 5. Baseline disease outcome tree for OTH site infections

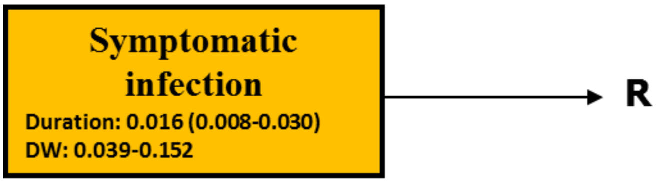

Table 1. Summary of disease health outcome parameters: case fatality proportion (CFP) and length of stay (LOS).

	Baseline model	*Klebsiella pneumoniae*			*Escherichia coli*			*Acinetobacter* spp.			*Pseudomonas aeruginosa*			*Staphylococcus aureus*	*Enterococcus faecalis* and *E. faecium*	*Streptococcus pneumoniae*	
		3GCRKP	CRKP	ColRKP	3GCREC	CREC	ColREC	MDRACI	CRACI	ColRACI	MDRPA	CRPA	ColRPA	MRSA	VRE	PRSP	PMRSP
**BSI**																	
Case fatality proportion (%)	7.1-20.3	14.4-19	20-51.3	32-88.8	17.1 (9.5-26)	20-51.3	32-88.8	7.1-32.9	7.1-34	7.1-34	7.1-35.2	7.1-38.7	7.1-38.7	17.9 (14.4-21.8)	22.9 (21.8-23.8)	15.7-20.3	15.7-20.3
Duration (days)	5.87-11.5	9.28 (9.20–9.35)	15-35	15-39.1	6-18.5	15-35	15-39.1	5.87-20.1	5.87-20.1	5.87-20.1	14.87-21.5	14.87-21.5	14.87-21.5	8.99-14.62	6.97-18.3	Baseline	Baseline
**RESP**																	
Case fatality proportion (%)	3.6 (2.7-4.5)	Baseline	Baseline	Baseline	Baseline	Baseline	Baseline	Baseline	Baseline	Baseline	2.7-9.8	2.7-10.5	2.7-10.5	Baseline	Baseline	Baseline	Baseline
Duration (days)	7-14	13.6-18.1	13.6-18.1	13.6-18.1	Baseline	13.6-18.1	13.6-18.1	Baseline	Baseline	Baseline	10-17	15-22	15-22	Baseline	Baseline	Baseline	Baseline
**UTI**																	
Case fatality proportion (%)	0	Baseline	Baseline	Baseline	Baseline	Baseline	Baseline	Baseline	Baseline	Baseline	Baseline	Baseline	Baseline	Baseline	Baseline	N/A	N/A
Duration (days)	7 (4-11)	7 (5-12)	7.5 (4-14)	7.5 (4-14)	7 (5-12)	7.5 (4-14)	7.5 (4-14)	8 (4-11)	8 (4-11)	8 (4-11)	8 (4-11)	8 (4-11)	8 (4-11)	Baseline	Baseline	N/A	N/A
**SSI**																	
Case fatality proportion (%)	0.9<65; 3.6>64	Baseline	Baseline	Baseline	Baseline	Baseline	Baseline	Baseline	Baseline	Baseline	Baseline	Baseline	Baseline	Baseline	Baseline	N/A	N/A
Duration (days)	8.5 (0-15.2)	Baseline	Baseline	Baseline	Baseline	Baseline	Baseline	Baseline	Baseline	Baseline	Baseline	Baseline	Baseline	Baseline	Baseline	N/A	N/A
**OTHER**																	
Case fatality proportion (%)	0	Baseline	Baseline	Baseline	Baseline	Baseline	Baseline	Baseline	Baseline	Baseline	Baseline	Baseline	Baseline	Baseline	Baseline	0	0
Duration (days)	6 (3-11)	12 (8-21)	12 (6-27)	12 (6-27)	12 (8-21)	12 (6-27)	12 (6-27)	14.5 (9-19)	14.5 (9-19)	14.5 (9-19)	14.5 (9-19)	14 (9-19)	14.5 (9-19)	12 (8-19)	Baseline	5-10	5-10

2. *Klebsiella pneumoniae* resistant to third-generation cephalosporin infections

2.1. Blood-stream infections

The review of the literature identified two studies comparing 3GCRKP infections to non-infected. A recent publication estimated an attributable CFP of 18% for MDRKP (Brady, 2017).

Attributable LOS to the 3GCRKP was set to 9.28 (9.20–9.35) (Stewardson, 2016) and the attributable CFP was set to 14.4-19% (Stewardson, 2016; Gallagher, 2014).

2.2. Respiratory tract infections

The review of the literature identified two studies comparing 3GCRKP infections to 3GCSKP. One study found a CFP risk difference of 8.9% and an added LOS of 9 days (Loh, 2006), whereas the second study found no added CFP and an attributable LOS of 6.6 days (Lee, 2011).

Given that the first study was done in a setting not representative of the European healthcare situation, we focused on the second study and have not added any CFP to the baseline model. We chose to add 6.6 days to the baseline model.

2.3. Urinary tract infections

The review of the literature identified one study focusing on solid organ transplant recipients. Moreover, this study did not exclude patients developing BSI and was excluded. Therefore, we chose to estimate the attributable LOS by applying the same methodology as the one chosen for the baseline infection model. All third-generation cephalosporin resistant Enterobacteriaceae from the PPS 2011-2012 were included and the attributable UTI LOS was set to 7 days (5-12).

2.4. Surgical site and other site infections

The review of the literature identified studies that did not differentiate other infection sites. Therefore, we kept the baseline models for SSIs.

The same approach for the baseline attributable LOS for OTH infection sites was used to estimate LOS. However, given the small number of observations for 3GCRKP, we chose to include all third-generation cephalosporin resistant Enterobacteriaceae from the PPS 2011-2012 in our estimation. We set the attributable LOS of OTH infections sites to 12 days (8-21).

3. *Klebsiella pneumoniae* resistant to carbapenem infections

3.1. Blood-stream infections

The review of the literature identified ten studies, three of which had non-infected patients as comparator. We excluded studies performed in specific populations, e.g. liver transplant patients (Mouloudi, 2014).

Of the two remaining studies, attributable CFP ranged from 27% to 50% and attributable LOS ranged from 3 to 35 days (Gallagher, 2014; Borer, 2009).

Seven studies compared susceptible infected patients to resistant. A met-analysis comparing CRKP to CSKP infections (Falagas, 2014) found an attributable CFP of 26-44% (this publication also reported two studies that showed no attributable mortality but these included mixed site of infection). A good-quality study (Mouloudi, 2010) found an attributable CFP compared to susceptible infections of 13%, similar to another study which found 14.7% (Hussein, 2013). The latter also found an attributable LOS of 5 days. Another retrospective cohort study found a higher attributable CFP, 31% (Ben-David, 2012), similar to a prospective observational study, 25.9% (Daikos, 2009).

When considering the baseline disease model for BSI and adding the attributable CFP, the latter ranges from 20% to 51.3%, which is similar to the studies using non-infected as comparator.

Therefore, we chose to apply a CFP of 20-51.3% to the CRKP BSI disease model.

Attributable LOS was set to 15 days (10 days from the baseline model to which we added 5 days [Hussein, 2013]) to 35 days (Gallagher, 2014).

3.2. Respiratory tract and surgical site infections

The literature review identified seven articles comparing CRKP infections to non-infected. We discarded those focusing on specific populations (e.g. transplant patients). Of the remaining studies, none provided specific mortality information based on site of infection. Therefore, the models baseline parameters were unvaried.

Following the review for 3GCRKP, we chose the following:

- Respiratory site infections: no added CFP. 6.6 days were added to the baseline model;
- SSI: no difference from baseline model;

3.3. Urinary tract infections

The review of the literature identified one study finding that CRKP UTIs have 6 added days of hospitalization compared to 3GCRKP.

We chose to estimate the attributable LOS by applying the same methodology as the one chosen for the baseline infection model. All carbapenem resistant Enterobacteriaceae from the PPS 2011-2012 were included and the attributable UTI LOS was set to 7.5 days (4-14).

3.4. Other site infections

The same approach for the baseline attributable LOS for OTH infection sites was used to estimate LOS. However, given the small number of observations for CRKP, we chose to include all carbapenem resistant Enterobacteriaceae in the PPS 2011-2012 in our estimation. We set the attributable LOS of OTH infections sites to 12 days (6-27).

4. *Klebsiella pneumoniae* resistant to colistin infections

4.1. All infection sites

The review of the literature identified four studies, all of which focused on mixed site of infection. The selection grid exercise clearly showed that two studies (matched case-control and prospective nested cohort) had a higher quality (Zarkotou, 2010; Rojas, 2016).

Attributable case fatality ranged from 12% (Rojas, 2016) to 37.5% (Zarkotou, 2010), and the attributable LOS ranged from no added days to 4.1 (+/- 8.5); however, it must be noted that controls were generally carbapenem resistant *K. pneumoniae* infections.

Rojas et al. show that increased attributable mortality is associated mostly with isolation in the blood. Therefore, the following decisions were made relatively to the disease models:

- Attributable CFP:
  - BSI: 12%-37.5% increase over CRKP
  - RESP: no increase from CRKP
  - SSI: no increase from CRKP
  - OTH: no increase from CRKP
- Attributable LOS
  - BSI: 0-4.1 days increase over CRKP
  - RESP: no increase from CRKP
  - SSI: no increase from CRKP
  - OTH: no increase from CRKP

5. *Escherichia coli* resistant to third-generation cephalosporin infections

5.1. Blood-stream infections

The review of the literature found one study comparing BSI resistant infections versus non-infected in 13 EU countries (de Kraker, 2011a). The univariate and multivariate analysis showed a significant impact of infections (with and without resistance) on the 30-day mortality. Death within 30 days after enrolment for resistant cases was 32% versus 6% in non-infected controls. LOS was 12 days (6-25) compared to 6 (3-16). Another EU publication (de Kraker, 2011b) estimated that 15,183 episodes of G3CREC BSIs were associated with 2,712 excess deaths, resulting in a CFP of 17.8%

The review also found 25 studies comparing BSI resistant versus susceptible infections. We excluded eight studies focusing on specific populations (e.g. haematological diseases) or where results for 3GCREC were not specifically addressed. Risk difference for CFP ranged from 2% (Van Aken, 2014) to 37.2%, although the latter was from a single-centre in 2003-2005 where the authors indicate that delayed treatment was the significant factor contributing to death (Melzer, 2007). One study with appropriate representativeness as well as a high number of cases (Martelius, 2016) found a CFP risk difference of 2.4%; another study with robust methodology undergone in the EU (Lambert, 2011) found 10% risk difference and 7 days LOS.

Of those providing ORS, three studies estimated a significant difference in mortality and three did not find a significant difference. When cumulating the information from the seventeen published studies considered above, we notice that 1,323 cases account for 276 deaths and that 10,991 controls account for 1,166 deaths. The risk difference was 10.3% (Wilson sample proportion 95% CI 8.71-11.9), which if added to the baseline values would account for a CFP of 17.4% to 30.6%.

However, we chose to combine the information from the more robust studies above and have added 2.4% and 10% to the baseline CFP of 7.1%, as well as considering the 26% from the first study. We had a similar approach for the LOS and considered 6 days (de Kraker, 2011a) compared to non-infected and 7 days (Lambert, 2011) to be added to the baseline LOS.

We applied a CFP of 17.1% (9.5-26) and a LOS of 6-18.5 days.

5.2. Urinary tract infections

The review of the literature found one study considering the difference in LOS between resistant infections and susceptible ones in patients with symptomatic UTIs. The study found 4.1 days LOS attributable to the resistant infection (Esteve-Palau, 2015) which if added to the baseline model would be 6.7 days.

Therefore, we chose to estimate the attributable LOS by applying the same methodology as the one chosen for the baseline infection model. All third-generation cephalosporin resistant Enterobacteriaceae from the PPS 2011-2012 were included and the attributable UTI LOS was set to 7 days (5-12).

5.3. Surgical site, respiratory tract and other site infections

The review of the literature did not find any specific information for other infections sites and we chose to apply the baseline models for SSI and RESP infections sites.

The same approach for the baseline attributable LOS for OTH infection sites was used to estimate LOS. However, given the small number of observations for 3CGREC, we chose to include all third-generation cephalosporin resistant Enterobacteriaceae in the PPS 2011-2012 in our estimation. We set the attributable LOS of OTH infections sites to 12 days (8-21).

6. *Escerichia coli* resistant to carbapenem infections

6.1. All infection sites

The review of the literature did not retrieve studies of sufficient quality or representativeness. Therefore, we decided to apply the same values as those for CRKP.

We chose to estimate the attributable UTI LOS by applying the same methodology as the one chosen for the baseline infection model. All carbapenem resistant Enterobacteriaceae from the PPS 2011-2012 were included and the attributable UTI LOS was set to 7.5 days (4-14).

The same approach for the baseline attributable LOS for OTH infection sites was used to estimate LOS. However, given the small number of observations for CREC, we chose to include all carbapenem resistant Enterobacteriaceae in the PPS 2011-2012 in our estimation. We set the attributable LOS of OTH infections sites to 12 days (6-27).

7. *Escerichia coli* resistant to colistin infections

7.1. All infection sites

The review of the literature did not retrieve studies of sufficient quality or representativeness. Therefore, we decided to apply the same values as those for ColRKP.

8. Multidrug resistant *Acinetobacter* infections

8.1. Blood-stream infections

The review of the literature identified two studies comparing MDRACI infections to non-infected. One study was excluded because it focused on ICU neonatal population (Al Jarousha, 2009). The other study found an attributable CFP of 24.4% (Gulen, 2015).

The review also found six studies comparing MDRACI BSI to susceptible BSI. Attributable CFP ranged from 17.8% (Smolyako, 2003) to 43.7% (Anunnatsiri, 2011) and LOS from 5.5 days (Fitzpatrick, 2015) to 20.1 days (Lee, 2007).

All four studies providing ORs found a significant impact of the resistant infection compared to the susceptible ones. When aggregating the information from the six studies, we found that 424 cases accounted for 162 deaths and 305 control accounted for 38 deaths. The risk difference was 25.8% (Wilson sample proportion 95% CI 24.5-26.2), which if added to the baseline values would account for a CFP of 32.9% to 46%.

Considering a methodologically robust study (Lambert, 2011) that found no CFP difference between susceptible and ceftazidime resistant infections, we chose to apply the baseline CFP (7.1%) and added the risk difference described above (25.8%) to this baseline CFP. Therefore, we set the MDRACI CFP to 7.1% to 32.9%. For the same reason, the lower bound value for attributable LOS of the baseline model was maintained (5.87 days) and the higher value was set to 20.1 days (Lee, 2007).

8.2. Respiratory tract infections

The review of the literature identified three studies comparing MDRACI infections to susceptible. We excluded one study because performed in a specific population (children) (Cai, 2012). One study found no significant difference (hazard ratio) between cases and controls (Inchai, 2015) and another had a small sample size (Park, 2006).

Therefore, we chose not to change the baseline parameters.

8.3. Urinary tract, surgical site and other site infections

The review of the literature identified studies that did not differentiate other infection sites (mixed infection sites). Therefore, we kept the baseline models for SSIs.

We chose to estimate the attributable UTI LOS by applying the same methodology as the one chosen for the baseline infection model. All resistant *Acinetobacter* and *Pseudomonas aeruginosa* from the PPS 2011-2012 were included and the attributable UTI LOS was set to 8 days (4-11).

The same approach for the baseline attributable LOS for OTH infection sites was used to estimate LOS. However, given the small number of observations, we chose to include all resistant

Acinetobacter and *Pseudomonas aeruginosa* from the PPS 2011-2012 in our estimation. We set the attributable LOS of OTH infections sites to 14.5 days (9-19).

9. Carbapenem resistant *Acinetobacter* infections

9.1. Blood-stream infections

The review of the literature identified two studies comparing CRACI infections to non-infected. Both were excluded because focused on specific populations (neonates in ICU and hematopoietic transplant patients).

The review also found eight studies comparing CRACI BSI to susceptible BSI. Excluding one study for the above-mentioned reasons (Kumar, 2014), attributable CFP OR was significant only in one study (Kim, 2012). All other studies estimating an OR (five studies) predominately showed no significant difference. When aggregating the information from the seven studies, we found that 413 cases accounted for 222 deaths and 577 controls accounted for 155 deaths. The risk difference was 26.9% (Wilson sample proportion 95% CI 25.5-27.9), which if added to the baseline values would account for a CFP of 34% to 47.2%.

Given the high uncertainty of the available evidence, we chose to maintain the baseline lower CFP (7.1%) and added the risk difference described above. Therefore, we set the CRACI CFP to 7.1% to 34%.

Two studies found an attributable LOS of 10 days (Jamulitrat, 2009; Tal-Jasper, 2016), hence we chose to maintain the same LOS as the one set for MDRACI.

9.2. Respiratory tract infections

The review of the literature identified one study comparing to non-infected (excluded because focusing on neonates in ICU) and three comparing to susceptible infections. The latter did not test for or find a significant attributable CFP or LOS, and the baseline parameters were kept.

9.3. Urinary tract, surgical site and other site infections

The review of the literature identified studies that did not differentiate other infection sites (mixed infection sites). Therefore, we kept the baseline models for SSIs.

We chose to estimate the attributable UTI LOS by applying the same methodology as the one chosen for the baseline infection model. All resistant *Acinetobacter* and *Pseudomonas aeruginosa* from the PPS 2011-2012 were included and the attributable UTI LOS was set to 8 days (4-11).

The same approach for the baseline attributable LOS for OTH infection sites was used to estimate LOS. However, given the small number of observations, we chose to include all resistant *Acinetobacter* and *Pseudomonas aeruginosa* from the PPS 2011-2012 in our estimation. We set the attributable LOS of OTH infections sites to 14.5 days (9-19).

10. Colistin resistant *Acinetobacter* infections

10.1. Blood-stream infections

The review of the literature identified one study comparing ColRACI infections to susceptible infections (Wang, 2015). No differences were reported and the CRACI model was maintained.

10.2. Urinary tract infections

The review of the literature identified one study comparing to susceptible infections (Taneja, 2011). No differences were reported and the CRACI model was maintained.

10.3. Respiratory tract, surgical site and other site infections

The review of the literature did not identify any other studies. Therefore, we kept the baseline models for respiratory tract infections and SSIs.

The same approach for the baseline attributable LOS for OTH infection sites was used to estimate LOS. However, given the small number of observations, we chose to include all resistant *Acinetobacter* and *Pseudomonas aeruginosa* from the PPS 2011-2012 in our estimation. We set the attributable LOS of OTH infections sites to 14.5 days (9-19).

11. Multidrug resistant *Pseudomonas aeruginosa* infections

11.1. Blood-stream infections

The review of the literature identified nine studies comparing MDRPA infections to susceptible infection. We discarded four studies that focused on specific populations (children, transplant patients, burned patients, haematological malignancies) and one without proper information on matching. Out of the remaining studies, the attributable CFP ranged from 0% (Morata, 2012; Joo, 2011) to 28.1% (Tam, 2010) and the attributable LOS ranged from 9 (Joo, 2011) days to 10 days (Tam, 2010; Tumbarello, 2011). Only two studies provided ORs, one indicating a significant difference in mortality and the other a non-significant difference.

When aggregating the information from the four studies, we found that 234 cases accounted for 71 deaths and that the 892 controls accounted for 245 deaths. The risk difference was 2.65% (Wilson sample proportion 95% CI 0-5.79). When added to the baseline scenario, the CFP ranged from 9.75% to 23%.

We chose to add 28.1% to the baseline CFP of 7.1% and kept 7.1% as the lower bound of the range. The MDRPA disease model was set to 7.1% to 35.2%. The attributable LOS was set 14.87 days to 21.5 days.

11.2. Respiratory tract infections

The review of the literature identified seven studies comparing to susceptible infections. We discarded one study that focused on COPD patients (Montero, 2009) and on ICU patients only without matching (Tumbarello, 2013); of the remaining studies, the attributable CFP ranged from 0% (Pena, 2013) to 13% (Micek, 2015) and the attributable LOS ranged from 2 (Micek, 2015) days to 3 days (Yang, 2009).

We also discarded those including secondary BSI (Micek, 2015; Yang, 2009) and the remaining studies found 0%, 4.8% (Yayan, 2015) and 5.3% (Cillóniz, 2016). We added the highest value to the highest in the baseline (4.5%); the disease model CFP was set to 2.7% to 9.8%. We set the attributable LOS to 10 to 17 days.

11.3. Urinary tract, surgical site and other site infections

The review of the literature did not identify any other studies. Therefore, we kept the baseline models for SSIs.

We chose to estimate the attributable UTI LOS by applying the same methodology as the one chosen for the baseline infection model. All resistant *Acinetobacter* and *Pseudomonas aeruginosa* from the PPS 2011-2012 were included and the attributable UTI LOS was set to 8 days (4-11).

The same approach for the baseline attributable LOS for OTH infection sites was used to estimate LOS. However, given the small number of observations, we chose to include all resistant *Acinetobacter* and *Pseudomonas aeruginosa* from the PPS 2011-2012 in our estimation. We set the attributable LOS of OTH infections sites to 14.5 days (9-19).

12. Carbapenem resistant *Pseudomonas aeruginosa* infections

12.1. Blood-stream infections

The review of the literature identified eight studies comparing CRPA infections to susceptible ones; we excluded one study because of a low number of cases (Hattemer, 2013) and another because of limited information on matching (Dantas, 2014). The attributable CFP ranged from 0% (Tuon, 2012;

Kim, 2014) to 18.4% (Kang, 2005) the attributable LOS ranged from 0 days (Tuon, 2012; Joo, 2011) to 10 days (Suarez, 2010).

One study found a significant difference between the mortality of resistant cases compared to susceptible, and 4 studies did not find a significant difference. When aggregating the information from the seven studies (all except the one without clear matching) and found that 410 cases accounted for 143 deaths and that 1,190 controls accounted for 338 deaths. The risk difference 6.48% (Wilson sample proportion 95% CI 4.51-8.58), which if added to the baseline would average a CFP of 13.6% to 26.8%.

Given the uncertainty and variability, we chose to maintain the baseline lower bound (7.1%) and added 18.4% to the baseline higher bound. The CRPA BSI CFP was set to 7.1%-38.7% and the attributable LOS was kept the same as for the MDRPA model.

12.2. Respiratory tract infections

The review of the literature identified one study comparing to susceptible that found 6% risk difference (Luyt, 2014) and 8 added days over susceptible infections. Therefore, we set the CFP to 2.7%-10.5% and the LOS to 15-22 days.

12.3. Urinary tract, surgical site and other site infections

The review of the literature did not identify any other studies. Therefore, we kept the baseline models for SSIs.

We chose to estimate the attributable UTI LOS by applying the same methodology as the one chosen for the baseline infection model. All resistant *Acinetobacter* and *Pseudomonas aeruginosa* from the PPS 2011-2012 were included and the attributable UTI LOS was set to 8 days (4-11).

The same approach for the baseline attributable LOS for OTH infection sites was used to estimate LOS. However, given the small number of observations, we chose to include all resistant *Acinetobacter* and *Pseudomonas aeruginosa* from the PPS 2011-2012 in our estimation. We set the attributable LOS of OTH infections sites to 14.5 days (9-19).

13. Colistin resistant *Pseudomonas aeruginosa* infections

13.1. All infection sites

The review of the literature did not retrieve studies eligible with the chosen selection criteria. Therefore, we decided to apply the same values as those for CRPA.

14. Meticillin resistant *Staphylococcus aureus* infections

14.1. Blood-stream infections

The review of the literature identified a large number of studies comparing attributable mortality and attributable LOS of MRSA to MSSA and/or non-infected patients. The WHO systematic review (WHO, 2014) found a significant impact of the resistant infection compared to susceptible ones. This meta-analysis of the WHO systematic review found an attributable mortality compared to MSSA of 10.8% (7.3-14.7) and an attributable LOS of 3.12 days (1.79-4.44). Added to the 7.1% CFP of the baseline model, the attributable CFP of MRSA would be 17.9% (14.4-21.8). When adding the attributable LOS to the baseline LOS (5.87-11.5 days), we estimated an attributable LOS compared to non-infected of 8.99-14.62 days. A recent publication estimated an attributable CFP of 19.5% (Brady, 2017).

The review of the literature identified twenty studies comparing MRSA and MSSA BSI attributable mortality. When aggregating the information from fourteen studies (after excluding those focusing only on specific populations, e.g. neonatal patients, patients with cardiac implants) we found that 1,353 cases accounted for 360 deaths and 2,954 controls accounted for 467 deaths. The resulting risk difference was 10.8% (Wilson sample proportion 95% CI 9.79-11.9), validating the result of the WHO review. Within EU countries, De Kraker et al. (de Kraker, 2011b) found that in 2007, 27,711 episodes

of MRSA BSIs were associated with 5,503 excess deaths, resulting in a 19.9% CFP. Moreover, more recent studies not included in the WHO systematic review (Stewardson, 2016; Yasmin, 2016; Atmaca, 2014) found similar attributable mortality compared to non-infected (20.4%, 20.3% and 18.2%). Therefore, we chose to apply the CFP of 17.9% (14.4-21.8).

Stewardson et al. also found an attributable LOS of 12.22 to 13.33 depending on the model used. These findings are in line with the chose attributable LOS for the MRSA model (8.99-14.62 days).

14.2. Respiratory tract infections

The review of the literature identified one study comparing MRSA to non-infected and five studies comparing to MSSA. However, these studies included S-BSI patients and were discarded. We chose to apply the baseline parameters to the MRSA model.

14.3. Urinary tract, surgical site and other site infections

The review of the literature did not identify any other studies. Therefore, we kept the baseline models for urinary tract infections, SSIs.

The same approach for the baseline attributable LOS for OTH infection sites was used to estimate LOS. The estimated attributable LOS of MRSA OTH infection sites was set to 12 days (8-19).

15. Vancomycin resistant *Enterococcus faecalis* and *faecium* infections

15.1. Blood-stream infections

The review of the literature identified five studies comparing VRE infection attributable CFP to non-infected. CFP ranged from 12.6% to 37%. Moreover, the review also identified two meta-analysis comparing VRE to VSE which found an attributable CFP of 17% (Salgado, 2003) and the other study did not provide a risk difference but also found a significant odds ratio (DiazGranados, 2005). A recent publication estimated an attributable CFP of 13.4% for vancomycin resistant E. faecalis and of 19.1% for vancomycin resistant E. faecium (Brady, 2017).

The review of the literature identified seventeen studies comparing VRE infection VSE. Attributable CFP ranged from 0% to 29%, after excluding studies focusing only in specific populations (e.g. patients with malignancy). When aggregating the information from thirteen studies, we found that 824 cases accounted for 297 deaths and 1208 controls accounted for 245 deaths. The resulting attributable CFP was 15.8% (Wilson sample proportion 95% CI 14.7-16.7), similar to the results of the meta-analysis mentioned above.

Therefore, we decided to add this result to the baseline conservative value of 7.1% and set the attributable CFP of the VRE model to 22.9% (Wilson sample proportion 95% CI 21.8-23.8).

Attributable LOS ranged from 1.1 days to 6.8 days which were added to the baseline LOS; the attributable LOS for the VRE BSI model was set to 6.97 to 18.3 days.

15.2. Respiratory tract, urinary tract, surgical site and other site infections

The review of the literature identified studies focusing on other infection sites but did not exclude death by S-BSI and were excluded.

The review of the literature did not identify any other studies. Therefore, we kept the baseline models for respiratory tract infections, UTIs, SSIs and OTH infection sites. For the attributable LOS of the OTH infection sites, we explored the possibility of adopting the same approach as for the baseline model but there was a limited number of observations. Therefore, we chose to adopt the baseline attributable LOS for VRE.

16. Penicillin resistant *Streptococcus pneumoniae* infections

16.1. Blood-stream infections

The review of the literature identified one study comparing PRSP BSI to susceptible BSI infections and found a mortality risk difference of 8.6% (Yu, 2003). No information on attributable LOS was available.

The review of the literature identified one study comparing PRSP respiratory to susceptible infections and found no difference in mortality (Watanabe, 2000). No information on attributable LOS was available. A recent publication estimated an attributable CFP of 16.7% (Brady, 2017).

Therefore, for BSIs we chose to add 8.6% to the lower baseline value and keep the higher value. The PRSP BSI attributable mortality was set to 15.7%-20.3% and the baseline attributable LOS was kept unvaried.

No changes on the baseline respiratory tract infection model was made.

16.2. Respiratory tract, urinary tract, surgical site and other site infections

The review of the literature did not retrieve studies eligible with the chosen selection criteria. Therefore, we decided to apply the same baseline values.

The only exception was the duration of OTH infection site (mainly otitis and sinusitis) to which we chose to apply 5-10 days (Mandell, 2015).

17. Macrolide and penicillin resistant *Streptococcus pneumoniae* infections

17.1. All infection sites

The review of the literature did not retrieve studies eligible with the chosen selection criteria. Therefore, we decided to apply the same values as those for PRSP.

References

Al Jarousha AM, El Jadba AH, Al Afifi AS, El Qouqa IA. Nosocomial multidrug-resistant *Acinetobacter baumannii* in the neonatal intensive care unit in Gaza City, Palestine. International journal of infectious diseases : IJID : official publication of the International Society for Infectious Diseases. Sep 2009;13(5):623-628.

Anunnatsiri S, Tonsawan P. Risk factors and clinical outcomes of multidrug-resistant *Acinetobacter baumannii* bacteremia at a university hospital in Thailand. The Southeast Asian journal of tropical medicine and public health. May 2011;42(3):693-703.

Astagneau P, Rioux C, Golliot F, Brucker G, Group INS. Morbidity and mortality associated with surgical site infections: results from the 1997-1999 INCISO surveillance. J Hosp Infect. 2001 Aug;48(4):267-74.

Atmaca O, Zarakolu P, Karahan C, Cakir B, Unal S. [Risk factors and antibiotic use in methicillin-resistant *Staphylococcus aureus* bacteremia in hospitalized patients at Hacettepe University Adult and Oncology Hospitals (2004-2011) and antimicrobial susceptibilities of the isolates: a nested case-control study]. Mikrobiyoloji bulteni. Oct 2014;48(4):523-537.

Ben-David D, Kordevani R, Keller N, et al. Outcome of carbapenem resistant *Klebsiella pneumoniae* bloodstream infections. Clinical Microbiology and Infection. 2012;18(1):54-60.

Borer A, Saidel-Odes L, Riesenberg K, et al. Attributable mortality rate for carbapenem-resistant *Klebsiella pneumoniae* bacteremia. Infection control and hospital epidemiology. 2009;30(10):972-976.

Brady M, Oza A, Cunney R, Burns K. Attributable mortality of hospital-acquired bloodstream infections in Ireland. J Hosp Infect. 2017 May;96(1):35-41.

Cai XF, Sun JM, Bao LS, Li WB. Risk factors and antibiotic resistance of pneumonia caused by multidrug resistant *Acinetobacter baumannii* in pediatric intensive care unit. World Journal of Emergency Medicine. 2012;3(3):202-207.

Cassini A, Plachouras D, Eckmanns T, Abu Sin M, Blank HP, Ducomble T, et al. Burden of Six Healthcare-Associated Infections on European Population Health: Estimating Incidence-Based Disability-Adjusted Life Years through a Population Prevalence-Based Modelling Study. PLoS Med. 2016;13(10):e1002150. DOI: 10.1371/journal.pone.0144988 PMID: 26672751

Cillóniz C, Gabarrús A, Ferrer M, et al. Community-Acquired Pneumonia Due to Multidrug- and Non–Multidrug-Resistant *Pseudomonas aeruginosa*. Chest. 2016;150(2):415-425.

Daikos GL, Petrikkos P, Psichogiou M, et al. Prospective observational study of the impact of VIM-1 metallo-beta-lactamase on the outcome of patients with *Klebsiella pneumoniae* bloodstream infections. Antimicrobial agents and chemotherapy. May 2009;53(5):1868-1873.

Dantas RC, Ferreira ML, Gontijo-Filho PP, Ribas RM. *Pseudomonas aeruginosa* bacteraemia: independent risk factors for mortality and impact of resistance on outcome. Journal of medical microbiology. Dec 2014;63(Pt 12):1679-1687.

a) de Kraker MEA, Wolkewitz M, Davey PG, et al. Burden of antimicrobial resistance in European hospitals: Excess mortality and length of hospital stay associated with bloodstream infections due to *Escherichia coli* resistant to third-generation cephalosporins. Journal of Antimicrobial Chemotherapy. 2011;66(2):398-407.

b) de Kraker ME, Davey PG, Grundmann H, group Bs. Mortality and hospital stay associated with resistant Staphylococcus aureus and Escherichia coli bacteremia: estimating the burden of antibiotic resistance in Europe. PLoS Med. Oct 2011;8(10):e1001104.

DiazGranados CA, Zimmer SM, Klein M, Jernigan JA. Comparison of mortality associated with vancomycin-resistant and vancomycin-susceptible enterococcal bloodstream infections: a meta-analysis. Clinical infectious diseases : an official publication of the Infectious Diseases Society of America. Aug 1 2005;41(3):327-333.

Esteve-Palau E, Solande G, Sanchez F, et al. Clinical and economic impact of urinary tract infections caused by ESBL-producing *Escherichia coli* requiring hospitalization: A matched cohort study. The Journal of infection. Dec 2015;71(6):667-674.

European Centre for Disease prevention and Control. Point prevalence survey of healthcare-associated infections and antimicrobial use in European acute care hospitals. Stockholm: ECDC; 2013. http://ecdc.europa.eu/en/publications/Publications/healthcare-associated-infections-antimicrobialuse-PPS.pdf.

European Centre for Disease Prevention and Control. Annual Epidemiological Report 2016 – Healthcare-associated infections acquired in intensive care units. [Internet]. Stockholm: ECDC; 2016. [cited 3 July 2017]. Available from: http://ecdc.europa.eu/en/healthtopics/HCAI_ICU/Pages/Annual-epidemiological-report-2016.aspx

European Centre for Disease Prevention and Control. Surveillance of healthcare-associated infections in intensive care units in Europe 2008–2012, including attributable mortality analyses. Stockholm: ECDC, 2017.

Falagas ME, Tansarli GS, Karageorgopoulos DE, Vardakas KZ. Deaths attributable to carbapenem-resistant Enterobacteriaceae infections. Emerging infectious diseases. Jul 2014;20(7):1170-1175.

Fitzpatrick MA, Ozer E, Bolon MK, Hauser AR. Influence of ACB complex genospecies on clinical outcomes in a U.S. hospital with high rates of multidrug resistance. The Journal of infection. Feb 2015;70(2):144-152.

Gallagher JC, Kuriakose S, Haynes K, Axelrod P. Case-case-control study of patients with carbapenem-resistant and third-generation-cephalosporin-resistant *Klebsiella pneumoniae* bloodstream infections. Antimicrobial agents and chemotherapy. Oct 2014;58(10):5732-5735.

Gulen TA, Guner R, Celikbilek N, Keske S, Tasyaran M. Clinical importance and cost of bacteremia caused by nosocomial multi drug resistant *Acinetobacter baumannii*. International journal of infectious diseases : IJID : official publication of the International Society for Infectious Diseases. Sep 2015;38:32-35.

Haagsma JA, van Beeck EF, Polinder S, Toet H, Panneman M, Bonsel GJ. The effect of comorbidity on health-related quality of life for injury patients in the first year following injury: comparison of three comorbidity adjustment approaches. Population Health Metrics. 2011;9:10. doi:10.1186/1478-7954-9-10.

Haagsma JA, Maertens de Noordhout C, Polinder S, Vos T, Havelaar AH, Cassini A, et al. Assessing disability weights based on the responses of 30,660 people from four European countries. Popul Health Metr. 2015; 13: 1±15. doi: 10.1186/s12963-015-0042-4 PMID: 26778920.

Hattemer A, Hauser A, Diaz M, et al. Bacterial and clinical characteristics of health care- and community-acquired bloodstream infections due to *Pseudomonas aeruginosa*. Antimicrobial agents and chemotherapy. Aug 2013;57(8):3969-3975.

Hussein K, Raz-Pasteur A, Finkelstein R, et al. Impact of carbapenem resistance on the outcome of patients' hospital-acquired bacteraemia caused by *Klebsiella pneumoniae*. Journal of Hospital Infection. 2013;83(4):307-313.

Inchai J, Pothirat C, Bumroongkit C, Limsukon A, Khositsakulchai W, Liwsrisakun C. Prognostic factors associated with mortality of drug-resistant *Acinetobacter baumannii* ventilator-associated pneumonia. Journal of Intensive Care. 2015;3(1).

Jamulitrat S, Arunpan P, Phainuphong P. Attributable mortality of imipenem-resistant nosocomial *Acinetobacter baumannii* bloodstream infection. Journal of the Medical Association of Thailand = Chotmaihet thangphaet. Mar 2009;92(3):413-419.

Joo EJ, Kang CI, Ha YE, et al. Risk factors for mortality in patients with *Pseudomonas aeruginosa* bacteremia: clinical impact of antimicrobial resistance on outcome. Microbial drug resistance (Larchmont, N.Y.). Jun 2011;17(2):305-312.

Kang CI, Kim SH, Park WB, et al. Risk factors for antimicrobial resistance and influence of resistance on mortality in patients with bloodstream infection caused by *Pseudomonas aeruginosa*. Microbial drug resistance (Larchmont, N.Y.). Spring 2005;11(1):68-74.

Kim YJ, Kim SI, Hong KW, Kim YR, Park YJ, Kang MW. Risk factors for mortality in patients with carbapenem-resistant *Acinetobacter baumannii* bacteremia: impact of appropriate antimicrobial therapy. Journal of Korean medical science. May 2012;27(5):471-475.

Kim YJ, Jun YH, Kim YR, et al. Risk factors for mortality in patients with *Pseudomonas aeruginosa* bacteremia; retrospective study of impact of combination antimicrobial therapy. BMC infectious diseases. Mar 24 2014;14:161.

Kumar A, Randhawa VS, Nirupam N, Rai Y, Saili A. Risk factors for carbapenem-resistant *Acinetobacter baumanii* blood stream infections in a neonatal intensive care unit, Delhi, India. Journal of infection in developing countries. Aug 13 2014;8(8):1049-1054.

Lambert ML, Suetens C, Savey A, et al. Clinical outcomes of health-care-associated infections and antimicrobial resistance in patients admitted to European intensive-care units: A cohort study. The Lancet Infectious Diseases. 2011;11(1):30-38.

Lee NY, Lee HC, Ko NY, et al. Clinical and economic impact of multidrug resistance in nosocomial *Acinetobacter baumannii* bacteremia. Infection control and hospital epidemiology. Jun 2007;28(6):713-719.

Lee JA, Kang CI, Joo EJ, et al. Epidemiology and clinical features of community-onset bacteremia caused by extended-spectrum beta-lactamase-producing *Klebsiella pneumoniae*. Microbial drug resistance (Larchmont, N.Y.). Jun 2011;17(2):267-273.

Loh LC, Nor Izran Hanim Bt Abdul S, Rosdara Masayuni Bt Mohd S, Raman S, Thayaparan T, Kumar S. Hospital Outcomes of Adult Respiratory Tract Infections with Extended-Spectrum Β-Lactamase (ESBL) Producing *Klebsiella Pneumoniae*. The Malaysian journal of medical sciences : MJMS. Jul 2007;14(2):36-40.

Luyt CE, Aubry A, Lu Q, et al. Imipenem, meropenem or doripenem to treat patients with *Pseudomonas aeruginosa* ventilator-associated pneumonia. American Journal of Respiratory and Critical Care Medicine. 2014;189.

Mandell, Douglas, and Bennett's Principles and Practice of Infectious Diseases, Eighth Edition (2015) Eds: John E. Bennett, Raphael Dolin, Martin J. Blaser. ISBN: 13-978-1-4557-4801-3, Elsevier Saunders

Martelius T, Jalava J, Karki T, Mottonen T, Ollgren J, Lyytikainen O. Nosocomial bloodstream infections caused by *Escherichia coli* and *Klebsiella pneumoniae* resistant to third-generation cephalosporins, Finland, 1999-2013: Trends, patient characteristics and mortality. Infectious diseases (London, England). 2016;48(3):229-234.

Melzer M, Petersen I. Mortality following bacteraemic infection caused by extended spectrum beta-lactamase (ESBL) producing *E. coli* compared to non-ESBL producing *E. coli*. The Journal of infection. Sep 2007;55(3):254-259.

Micek ST, Wunderink RG, Kollef MH, et al. An international multicenter retrospective study of *Pseudomonas aeruginosa* nosocomial pneumonia: impact of multidrug resistance. Critical care (London, England). May 06 2015;19:219.

Montero M, Dominguez M, Orozco-Levi M, Salvado M, Knobel H. Mortality of COPD patients infected with multi-resistant *Pseudomonas aeruginosa*: a case and control study. Infection. Feb 2009;37(1):16-19.

Morata L, Cobos-Trigueros N, Martinez JA, et al. Influence of multidrug resistance and appropriate empirical therapy on the 30-day mortality rate of *Pseudomonas aeruginosa* bacteremia. Antimicrobial agents and chemotherapy. Sep 2012;56(9):4833-4837.

Mouloudi E, Massa E, Papadopoulos S, et al. Bloodstream infections caused by carbapenemase-producing *Klebsiella pneumoniae* among intensive care unit patients after orthotopic liver

transplantation: Risk factors for infection and impact of resistance on outcomes. Transplantation Proceedings. 2014;46(9):3216-3218.

Mouloudi E, Protonotariou E, Zagorianou A, et al. Bloodstream infections caused by metallo-beta-lactamase/*Klebsiella pneumoniae* carbapenemase-producing *K. pneumoniae* among intensive care unit patients in Greece: risk factors for infection and impact of type of resistance on outcomes. Infection control and hospital epidemiology. Dec 2010;31(12):1250-1256.

Olaechea PM, Palomar M, Alvarez-Lerma F, Otal JJ, Insausti J, Lopez-Pueyo MJ, et al. Morbidity and mortality associated with primary and catheter-related bloodstream infections in critically ill patients. Rev Esp Quimioter. 2013 Mar;26(1):21-9. Renaud B, Brun-Buisson C, Group IC-BS. Outcomes of primary and catheter-related bacteremia. A cohort and case-control study in critically ill patients. Am J Respir Crit Care Med. 2001;163(7):1584-90.

Park II, Kim IK, Koo HC, et al. Clinical characteristics and prognosis of *Acinetobacter* nosocomial pneumonia between MDR and non-MDR. Tuberculosis and Respiratory Diseases. 2006;61(1):13-19.

Pena C, Gomez-Zorrilla S, Oriol I, et al. Impact of multidrug resistance on *Pseudomonas aeruginosa* ventilator-associated pneumonia outcome: predictors of early and crude mortality. European journal of clinical microbiology & infectious diseases : official publication of the European Society of Clinical Microbiology. Mar 2013;32(3):413-420.

Robert Koch Institute. Burden of healthcare associated infection (BHAI)—evidence-based and comorbidity-adjusted outcome trees for estimation of burden of disease. Berlin: RKI; 2016. http://www.rki.de/DE/Content/Institut/OrgEinheiten/Abt3/FG37/Research_Report_BHAI.pdf?blob=publicationFile.

Rojas LJ, Salim M, Cober E, et al. Colistin Resistance in Carbapenem-Resistant *Klebsiella pneumoniae*: Laboratory Detection and Impact on Mortality. Clinical infectious diseases : an official publication of the Infectious Diseases Society of America. Dec 10 2016.

Salgado CD, Farr BM. Outcomes associated with vancomycin-resistant enterococci: a meta-analysis. Infection control and hospital epidemiology. Sep 2003;24(9):690-698.

Smolyakov R, Borer A, Riesenberg K, et al. Nosocomial multi-drug resistant *Acinetobacter baumannii* bloodstream infection: risk factors and outcome with ampicillin-sulbactam treatment. The Journal of hospital infection. May 2003;54(1):32-38.

Stewardson AJ, Allignol A, Beyersmann J, et al. The health and economic burden of bloodstream infections caused by antimicrobial-susceptible and non-susceptible Enterobacteriaceae and *Staphylococcus aureus* in European hospitals, 2010 and 2011: A multicentre retrospective cohort study. Eurosurveillance. 2016;21(33).

Suarez C, Pena C, Gavalda L, et al. Influence of carbapenem resistance on mortality and the dynamics of mortality in *Pseudomonas aeruginosa* bloodstream infection. International journal of infectious diseases : IJID : official publication of the International Society for Infectious Diseases. Sep 2010;14 Suppl 3:e73-78.

Tal-Jasper R, Katz DE, Amrami N, et al. Clinical and epidemiological significance of carbapenem resistance in *Acinetobacter baumannii* infections. Antimicrobial agents and chemotherapy. 2016;60(5):3127-3131.

Tam VH, Rogers CA, Chang KT, Weston JS, Caeiro JP, Garey KW. Impact of multidrug-resistant *Pseudomonas aeruginosa* bacteremia on patient outcomes. Antimicrobial agents and chemotherapy. Sep 2010;54(9):3717-3722.

Taneja N, Singh G, Singh M, Sharma M. Emergence of tigecycline & colistin resistant *Acinetobacter baumanii* in patients with complicated urinary tract infections in north India. Indian J Med Res. Jun 2011;133:681-684.

Tumbarello M, Repetto E, Trecarichi EM, et al. Multidrug-resistant *Pseudomonas aeruginosa* bloodstream infections: risk factors and mortality. Epidemiology and infection. Nov 2011;139(11):1740-1749.

Tumbarello M, De Pascale G, Trecarichi EM, et al. Clinical outcomes of *Pseudomonas aeruginosa* pneumonia in intensive care unit patients. Intensive care medicine. Apr 2013;39(4):682-692.

Tuon FF, Gortz LW, Rocha JL. Risk factors for pan-resistant *Pseudomonas aeruginosa* bacteremia and the adequacy of antibiotic therapy. The Brazilian journal of infectious diseases : an official publication of the Brazilian Society of Infectious Diseases. Jul-Aug 2012;16(4):351-356.

Van Aken S, Lund N, Ahl J, Odenholt I, Tham J. Risk factors, outcome and impact of empirical antimicrobial treatment in extended-spectrum beta-lactamase-producing *Escherichia coli* bacteraemia. Scandinavian journal of infectious diseases. Nov 2014;46(11):753-762.

Wang YC, Lee YT, Yang YS, et al. Risk factors and outcome for colistin-resistant *Acinetobacter nosocomialis* bacteraemia in patients without previous colistin exposure. Clinical microbiology and infection : the official publication of the European Society of Clinical Microbiology and Infectious Diseases. Aug 2015;21(8):758-764.

Watanabe H, Sato S, Kawakami K, et al. A comparative clinical study of pneumonia by penicillin-resistant and -sensitive *Streptococcus pneumoniae* in a community hospital. Respirology. Mar 2000;5(1):59-64.

WHO. Antimicrobial resistance: global report on surveillance. France: WHO;2014.

Yang K, Zhuo H, Guglielmo BJ, Wiener-Kronish J. Multidrug-resistant *Pseudomonas aeruginosa* ventilator-associated pneumonia: the role of endotracheal aspirate surveillance cultures. The Annals of pharmacotherapy. Jan 2009;43(1):28-35.

Yayan J, Ghebremedhin B, Rasche K. Antibiotic resistance of *Pseudomonas aeruginosa* in pneumonia at a single university hospital center in Germany over a 10-Year Period. PloS one. 2015;10(10).

Yasmin M, El Hage H, Obeid R, El Haddad H, Zaarour M, Khalil A. Epidemiology of bloodstream infections caused by methicillin-resistant *Staphylococcus aureus* at a tertiary care hospital in New York. American journal of infection control. 2016;44(1):41-46.

Yu VL, Chiou CC, Feldman C, et al. An international prospective study of pneumococcal bacteremia: correlation with in vitro resistance, antibiotics administered, and clinical outcome. Clinical infectious diseases : an official publication of the Infectious Diseases Society of America. Jul 15 2003;37(2):230-237.

Zarkotou O, Pournaras S, Voulgari E, et al. Risk factors and outcomes associated with acquisition of colistin-resistant KPC-producing *Klebsiella pneumoniae*: a matched case-control study. J Clin Microbiol. Jun 2010;48(6):2271-2274.

Burden of antimicrobial resistance

Methodology protocol to estimate incidence

Written by Annalisa Quattrocchi and Alessandro Cassini with contributions from Carl Suetens, Liselotte Diaz Högberg, Mélanie Colomb-Cotinat, Diamantis Plachouras and Dominique Monnet

Contents

List of abbreviations 194

1. Introduction and background information 195

1.1. European Antimicrobial Resistance Surveillance Network (EARS-Net) 195

1.2. Point Prevalence Survey of healthcare-associated infections and antimicrobial use in acute care hospitals (PPS 2011-2012) 196

2. Step-by-step approach to estimate AMR infections 197

2.1. Selection of Specified Pathogens (SP) 197

2.2. Data modelling for estimating total SPDAR infections 198

2.2.1. Step 1: Country-coverage adjustment 198

2.2.2. Step 2: Estimation of number of other infection sites 200

2.2.3. Step 3: Adjustment for secondary BSI 200

3. Approach to estimate conversion factors for penicillin-resistant and combined penicillin- and macrolide-resistant *S. pneumoniae* 202

4. Limitations 204

References 206

Addendum 1 171

Addendum 2 172

Addendum 3 172

Addendum 4 173

List of abbreviations

3GCREC	third-generation cephalosporin-resistant *E. coli*
3GCRKP	third-generation cephalosporin-resistant *K. pneumoniae*
AMR	antimicrobial resistance
BSI	bloodstream infection
CAI	Community-associated infections
CI	confidence interval
ColRACI	colistin-resistant *Acinetobacter* spp.
ColREC	colistin-resistant *E. coli*
ColRKP	colistin-resistant *K. pneumoniae*
ColRPA	colistin-resistant *P. aeruginosa*
CRACI	carbapenem-resistant *Acinetobacter* spp.
CREC	carbapenem-resistant *E. coli*
CRKP	carbapenem-resistant *K. pneumoniae*
CRPA	carbapenem-resistant *P. aeruginosa*
CSF	cerebral spine fluid
DALY	disability-adjusted life years
EARS-Net	European Antimicrobial Resistance Surveillance Network
EEA	European Economic Area
EU	European Union
HAI	healthcare-associated infection
LOS	length of stay
MDR	multidrug-resistant
MDRACI	multidrug-resistant *Acinetobacter* spp
MDRPA	multidrug-resistant *P. aeruginosa*
MRSA	meticillin-resistant *Staphylococcus aureus*
MS	Member State
OECD	Organisation for Economic Co-operation and Development
OTH	other infection site including digestive tract infections, skin and soft tissue infections (SSTI), eye, ear, nose or mouth infections, bone and joint infections, cardiovascular infections, reproductive tract infections and other less frequent infections
PMRSP	penicillin- and macrolide-resistant S. pneumoniae
PPS	point prevalence survey of healthcare-associated infections and antimicrobial use in acute care hospitals
PRSP	penicillin-resistant *S. pneumoniae*;
RESP	respiratory infections (including pneumonia, and low respiratory tract infection)
S-BSI	secondary BSI
SP	specified pathogens
SPDAR	specified pathogens with defined antimicrobial resistance
SSI	surgical site infection
UTI	urinary tract infection
VRE	vancomycin-resistant enterococci

1. Introduction and background information

The aim of this study is to estimate the incidence of infections due to selected antimicrobial resistant (AMR) bacteria for the burden of AMR project in EU/EEA countries. Further objectives of the burden of AMR project are the estimation of the attributable case fatality, attributable length of stay (LOS), disability-adjusted life years (DALYs) and economic impact of these selected AMR infections in EU/EEA Member States.

The primary data source for the estimation of the incidence of AMR infections was the European Antimicrobial Resistance Surveillance Network (EARS-Net), for the year 2015 [3]. EARS-Net notifies the number of resistant invasive infections for a selection of pathogens. EARS-Net cases were adjusted through data from the point prevalence survey of healthcare-associated infections and antimicrobial use in acute care hospitals (PPS 2011-2012) [1] in order to estimate total country-specific number of infections, i.e. the number of non-BSI infections.

This project is conducted in collaboration with the Organisation for Economic Co-operation and Development (OECD) for the estimation of the economic burden of AMR.

a. European Antimicrobial Resistance Surveillance Network (EARS-Net)

EARS-Net is the main EU surveillance system for antimicrobial resistance, based on a network of representatives from the Member States collecting routine clinical antimicrobial susceptibility data from national AMR surveillance initiatives.

The network collects antimicrobial susceptibility data of the first blood and/or cerebrospinal fluid isolate of every patient with an invasive infection associated with one of the pathogens under surveillance. Both antimicrobial-susceptible and antimicrobial-non-susceptible isolates are reported. The objective of EARS-Net is to measure the percentage of non-susceptible or resistant bacteria by species. Assuming that all eligible invasive isolates are reported by the participating laboratories and with country-level knowledge of the denominators (country coverage), we estimated the incidence of cases. The panels of antimicrobial agent combinations under surveillance for each microorganism, as well as algorithms for isolate prioritisation and deduplication, are defined in the EARS-Net reporting protocol [4].

Cerebrospinal fluid isolates represented 0-3% of all isolates reported to EARS-Net (6% for *S. pneumoniae*); we assumed that the patients with meningitis would most likely also have a BSI and were included in our study as BSIs.

EARS-Net performs surveillance of antimicrobial susceptibility of eight bacterial pathogens commonly causing infections in humans, hereafter referred to as “specified pathogens” (SP) and listed in table 1.

Table 1. Specified pathogens (SP)

**Specified pathogen**
Acinetobacter spp.
Enterococcus faecalis and *E. faecium*
Escherichia coli
Klebsiella pneumoniae
Pseudomonas aeruginosa
Staphylococcus aureus
Streptococcus pneumoniae

A total of 30 countries (all EU Member States, Iceland and Norway) reported AMR data for 2015 to EARS-Net. For this study, an isolate was considered resistant to an antimicrobial group when tested and interpreted as resistant (R) in accordance with the clinical breakpoint criteria used by the local laboratory. Specific rules were applied to determine MRSA and penicillin susceptibility in *S.*

pneumoniae, in accordance with the EARS-Net methodology. The definition of resistance on antimicrobial group level is provided in the EARS-Net protocol [4].

b. Point Prevalence Survey of healthcare-associated infections and antimicrobial use in acute care hospitals (PPS 2011-2012)

The ECDC PPS was conducted in 2011–2012 in 29 EU/EEA Member States and Croatia, and included data from a total of 273,753 patients in 1,149 hospitals [1].

It represented the first EU/EEA-wide, ECDC-coordinated PPS of healthcare-associated infections (HAIs) and antimicrobial use in acute care hospitals, representing more than 510 million citizens according to 2011 Eurostat data.

PPS collects patient-based risk factors and case-based data on selected antimicrobial resistance markers depending on microorganism, if isolated, for each HAI.

Selected antimicrobial susceptibility testing data were available on the day of the survey for 85% of microorganisms reported in HAIs. The percentage of microorganisms with known antimicrobial susceptibility testing results varied between 58% and 92%.

Representativeness of the PPS data by country (based on compliance with the recommended sampling methodology of hospitals and sample size) was reported as being optimal or good in 76% of countries.

2. Step-by-step approach to estimate AMR infections

a. Selection of Specified Pathogens (SP)

The number of reported isolates including age and sex information (each representing one case) were extracted from EARS-Net and, based on resistance patterns, SPs with defined antimicrobial resistance (SPDAR) were selected. SPDAR are listed in Table 2.

Pathogens included in EARS-Net are selected in consultation with the EARS-Net disease-specific coordination committee, composed by officially nominated country representatives and external experts mainly representing collaborative institutions such as EUCAST and WHO. The European public health impact of the resistant infection, as well as availability of standardised laboratory methodology for species identification and susceptibility testing (in order to ensure reliable data) are amongst the criteria considered.

Table 2. Bacteria and antibiotic resistance categories included in the study

Bacteria	Antibiotic resistance [¥]	Acronym
Acinetobacter spp.	Colistin-resistant	ColRACI
	Carbapenem-resistant (excluding isolates also resistant to colistin)	CRACI
	Aminoglycoside- and fluoroquinolone-resistant[#] (excluding isolates also resistant to colistin and/or carbapenem)	MDRACI
Enterococcus faecalis and *E. faecium*	Vancomycin-resistant	VRE
Escherichia coli	Colistin-resistant	ColREC
	Carbapenem-resistant (excluding isolates also resistant to colistin)	CREC
	Third-generation cephalosporin-resistant* (excluding isolates also resistant to colistin and/or carbapenem)	3GCREC
Klebsiella pneumoniae	Colistin-resistant	ColRKP
	Carbapenem-resistant (excluding isolates also resistant to colistin)	CRKP
	Third-generation cephalosporin-resistant* (excluding isolates also resistant to colistin and/or carbapenem)	3GCRKP
Pseudomonas aeruginosa	Colistin-resistant	ColRPA
	Carbapenem-resistant (excluding isolates also resistant to colistin)	CRPA
	Resistance to three or more antibiotic groups[#] (excluding isolates also resistant to colistin and/or carbapenem)	MDRPA
Staphylococcus aureus	Meticillin-resistant	MRSA
Streptococcus pneumoniae	Penicillin-resistant (excluding isolates also resistant to macrolides)	PRSP
	Penicillin- and macrolide-resistant (excluding isolates only resistant to penicillin)	PMRSP

[¥] An isolate was considered resistant to an antimicrobial group when tested and interpreted as resistant (R) in accordance with the clinical breakpoint criteria used by the local laboratory

[#] Resistances used as a marker of multidrug resistance

[*] In 2015, most of the third-generation cephalosporin-resistant *E. coli (88.6%)* and *K. pneumoniae* (85.3%) isolates reported to EARS-Net produced an extended-spectrum β-lactamase (ESBL) (9).

For more information on the antibiotics included in each antibiotic group, please refer to the EARS-Net protocol (5).

For each pathogen/resistance combination, unknown age-groups and sex data were re-distributed by imputation according to known cases where possible. When insufficient data was available (more than 40% of cases for an antibiotic resistance-bacterium combination with unknown age and/or sex), the cumulative EU age-group and sex distribution of the same antibiotic resistance-bacterium combination was applied for imputation. Addendum 4 provides more details of unknowns and the imputation methodology applied for each antibiotic resistance-bacterium combination in each country.

b. Data modelling for estimating total SPDAR infections

In order to obtain all SPDAR infections a multi-step adjustment was performed. A summary description is shown in Figure 1.

Figure 1. Methodology for adjusting EARS-Net data in order to estimate the total number of SPDAR infections.

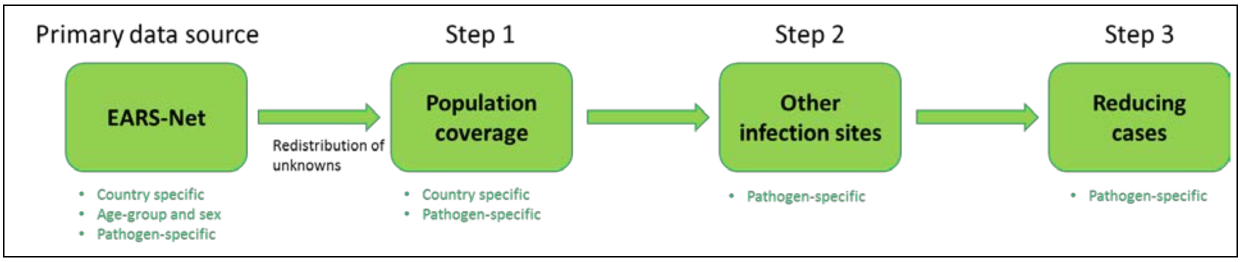

i.Step 1: Country-coverage adjustment

The primary data source was EARS-Net which is based on recruited laboratories in each Member State. EARS-Net is not a population-based surveillance network, the population coverage of the laboratories contributing data to EARS-Net varies between countries and the number of cases reported does not always reflect the total number of cases in a country. During the annual EARS-Net data call, a questionnaire on the estimated population coverage is distributed to the formally designated national Focal Points (NFP) for AMR and the Operational Contact Points (OCP) for AMR. The number of designated NFPs and OCPs per country varies, but represents those working closely with the national data collection and with good insight in geographical and demographical representativeness of EARS-Net.

A limitation related to this country coverage correction approach is that case ascertainment of patients with bloodstream infections (BSIs) is strongly linked to diagnostic practices and the frequency with which blood cultures are taken. Therefore, variations in blood culture frequency or propensity to test BSIs could increase uncertainty when comparing the reported number of cases between countries and potentially underestimate the total number of cases when the blood culture frequency is low. Other limitations of the EARS-Net database considered for our study was the representativeness of the hospitals and laboratories participating: for countries reporting data from only a small number of hospitals and laboratories located in one specific geographical area, the sample may not be representative for the whole country. In addition, for countries where not all laboratories/hospitals participate in EARS-Net, the distribution of healthcare level of the sample origin might affect the patient case-mix, with the potential to both over- and underestimate the total number of cases when applying a general population coverage estimate.

Methods aiming at improving the representativeness were explored, in particular the possibility of adjusting for the frequency of use of blood cultures. EARS-Net provides some information on the number of blood culture sets per 1,000 patient days as an indicator for case ascertainment of patients with bloodstream infections. However, this information is incomplete for many countries and the denominator (i.e. 1,000 patient days) was not always homogeneously estimated across countries. In

order to provide more information on blood culturing propensity, we provided the reported number of blood culture sets per 1,000 patient days for 2015 [3] and the 2015 EU LabCap Indicator 1.32 Blood culture test rate [12], see Addendum 4.

Therefore, the estimation of the country coverage for EARS-Net was mainly based on the pathogen-specific EARS-Net country population coverage, which was generally calculated based on the national ratio of admissions in reporting hospitals to total admissions in national hospitals. In an effort to partially take into account the uncertainties discussed above, designated contact points in each Member State were given the possibility of providing a range of estimated national population coverage. The estimates were expressed in percentages of population covered and the resulting multiplication factor was derived (100/population covered). Table 3 shows the estimated population country coverage per organism for each participating countries.

For each country, the total number of invasive infections due to each pathogen/resistance combination was then estimated by applying a factor based on this self-reported country coverage to the number of cases reported to EARS-Net.

Table 3. Estimated population country coverage per organism. All numbers are percentages.

Country	*S. pneumoniae*	*S. aureus*	*Enterococci*	*E. coli*	*K. pneumoniae*	*P. aeruginosa*	*Acinetobacter* spp
Austria	90	90	90	90	90	90	90
Belgium	87	24	24	24	23	24	8
Bulgaria	28-29-30	28-29-30	28-29-30	28-29-30	28-29-31	28-29-32	28-29-33
Cyprus	82.5-85-87.5	82.5-85-87.	82.5-85-87.5	82.5-85-87	82.5-85-87.5	82.5-85-87.5	82.5-85-87.5
Czech Republic	90	85	85	85	85	85	85
Germany	20-25-30	20-25-30	20-25-30	20-25-30	20-25-30	20-25-30	20-25-30
Denmark	100	100	100	100	100	100	100
Estonia	100	100	100	100	100	100	100
Greece	NA	57	54	60	53	52	60
Spain	27.8-31.05-34.3	27.8-31.05-	27.8-31.05-34	27.8-31.05	27.8-31.05-34.3	27.8-31.05-34.3	27.8-31.05-34.3
Finland	97-98-99	97-98-99	98-99-100	97-98-99	97-98-99	98-99-100	94-95-96
France	67.2	12.6-18-18	12.6-18-18	12.6-18-18	12.6-18-18	12.6-18-18	12.6-18-18
Croatia	85-90-95	85-90-95	85-90-95	85-90-95	85-90-95	85-90-95	85-90-95
Hungary	90	90	90	90	90	90	90
Ireland	97	97	97	97	97	97	97
Iceland	100	100	100	100	100	100	100
Italy	10-15-20	10-15-20	10-15-20	10-15-20	10-15-20	10-15-20	10-15-20
Lithuania	90	90	90	90	90	90	90
Luxembourg	100	100	100	100	100	100	100
Latvia	90	90	90	90	90	90	90
Malta	95	95	95	95	95	95	95
Netherlands	65	65	65	65	65	65	65
Norway	100	100	100	100	100	100	100
Poland	14	14	14	14	14	14	14
Portugal	95	96.1	96	95.2	96.5	95.3	91.2
Romania	12-15-17	12-15-17	12-15-17	12-15-17	12-15-17	12-15-17	12-15-17
Sweden	75-77.5-80	75-77.5-80	75-77.5-80	75-77.5-80	75-77.5-80	75-77.5-80	60
Slovenia	99.1	97.9	98.8	99.3	99.6	97.9	100
Slovakia	75	75	75	75	75	75	75
United Kingdom	21	26	12	12	12	12	12

ECDC is currently working with countries in standardising the sample frame and data collection methods, as well as evaluating the factors affecting the EARS-Net country coverage. These include (but not limited to): the population coverage, geographical representativeness and selection of

surveillance sites/laboratories, patient case-mix, sampling (propensity to test) and laboratory capacity and procedures (AST methods).

ii. Step 2: Estimation of number of other infection sites

A complete picture of the burden of disease caused by infections with antibiotic-resistant bacteria requires the estimation of the number of all other types of infection. The ECDC PPS 2011-2012 focused on 29 EU/EEA countries and Croatia, and included data from a total of 273,753 patients in 1,149 hospitals. Selected antimicrobial susceptibility testing data were available for 85% of microorganisms reported in HAIs. PPS data were used to estimate the number of infections in other infection sites, except for *S. pneumoniae*.

The EU/EEA total PPS infection site incidence for each SPDAR was calculated by applying the Rhame and Sudderth formula to the prevalence data [13]. We estimated the ratio from BSI to other infection sites from the incident EU/EEA cumulative number of infections in the PPS. Particularly, we computed the number of cases for the following infections sites (according to the EU/EEA case definitions [5]):

- Bloodstream infection (BSI);
- Respiratory infection (RESP);
- Urinary tract infection (UTI);
- Surgical site infection (SSI);
- Other infection sites (OTH: excluding BSI, UTI, RESP and SSI).

The OTH group included digestive tract infections, skin and soft tissue infections (SSTIs), eye, ear, nose or mouth infections, bone and joint infections, cardiovascular infections, reproductive tract infections and other less frequent infections, caused by the selected antibiotic-resistant bacteria.

When PPS did not collect information on given SPDAR, proxies were chosen. This was the case for the following SPs:

- PPS did not collect information on colistin resistance. Therefore, carbapenem resistance of the same pathogen was used instead.
- PPS did not have enough cases of CREC; therefore, conversion factors of 3GCREC were used instead.
- PPS did not collect information on MDR *Acinetobacter* spp. Therefore, carbapenem resistant *Acinetobacter* spp. was used instead.
- PPS did not collect information on MDR *P. aeruginosa*. Therefore, carbapenem resistant *P. aeruginosa* was used instead.
- PPS did not collect information on penicillin-resistant and combined penicillin- and macrolide-resistant *S. pneumoniae.* Therefore a review of the published literature was undertaken, described in paragraph 3.

The PPS estimated ratio between BSIs and other infection sites, including 95% confidence interval (CI), were applied to the country-specific age-group and sex tables resulting from step 1. The CI of the conversion factors for each infection site were estimated by combining the CIs of the estimated infection site incidence of each SPDAR with the CIs of the incidence of BSI. The proportion of the CIs around BSI and the other infection site incidence were added and applied to the conversion factor. This approach ensures that all uncertainties are considered, but also entail very wide CIs.

For more information on infection site conversion factors for each SPDAR, please refer to Addendum 1.

iii. Step 3: Adjustment for secondary BSI

Each infection leading to secondary BSI (S-BSI) retrieved through the PPS is expected to be reported as BSI in EARS-Net. Thus, the previous adjustment might lead to double counting of the infections, resulting in overestimation of the total number of AMR infections. Therefore, the percentage of S-BSIs from each of the other infection sites (reducing factors derived from the variable 'origin of BSI' in PPS data) was deducted from infections in other sites. This deduction was applied to the number of cases for each non-BSI infection site, similarly to the example for UTIs below:

$(UTI)_{step3}$ = $(UTI)_{step2}$ – ([BSI] * x)

With x being the percentage of S-BSIs.

For each country and for each SPDAR, the reducing factors were applied to age-group and sex adjusted cases obtained from step 2, in order to estimate the number of all infections for each SPDAR. When the initial number of cases is low, the resulting lower uncertainty bound after Step 3 can be negative. We assumed that in those cases the lower uncertainty bound is 0.

For more information on S-BSI reducing factors for each SPDAR, please refer to Addendum 2.

Approach to estimate conversion factors for penicillin-resistant and combined penicillin- and macrolide-resistant *S. pneumoniae*

The PPS 2011-2012 did not collect information on *S. pneumoniae*. Therefore, in order to estimate a BSI/other infection site conversion factor (step 2), a review of the published literature was undertaken. The search provided a number of studies focusing on penicillin-resistant *S. pneumoniae* infection sites in European countries. However, no studies were found specifically for penicillin/macrolide combined resistance. Conversion factors for the latter infections were assumed to be the same as for penicillin-resistant infections. No studies reported *S. pneumoniae* in SSIs or in UTIs. Table 4 summarises all the results of the literature search.

ECDC report "Time to react" [6] used 2.7 as conversion between RESP/BSI.

Vardhan et al. [7] report a number of penicillin-resistant *S. pneumoniae* infections by site in adults and in children. The latter did not experience any BSI and were not considered as a separate population. Isolates from chest were considered as "Respiratory (RESP)" infections and those from the following infection sites: eye, wound/burn, ear and sinus were all considered as "other (OTH)" infections' category Hence, the conversion factors derived from this study are RESP/BSI: 8.33 and OTH/BSI: 0.54.

A textbook [8] reported a respiratory infection to BSI conversion factor of 4.

A large study in France [9] reported percentages of penicillin-resistant *S. pneumoniae* for each infection site in adults and in children, although the age group of the latter were not defined. We added the cerebral spine fluid (CSF) infections to BSIs and the middle ear fluid/sinus infections as part of the "other" category. Conversion factors for RESP/BSI was 2.97 and for other/BSI was 0.33 in adults. In children these factors change to 3.86 and 7.18, respectively. Infections resistant to erythromycin had almost the same conversion factors. However, erythromycin-resistant infections were not specified if they were also resistant to penicillin. Therefore, we decided to retain only the information on penicillin-resistant *S. pneumoniae* in adults.

Hauser et al. [10] described the number of invasive and non-invasive penicillin-resistant *S. pneumoniae* infections in Switzerland. Assuming all invasive infections as BSI (and or CSF), we estimated a conversion factor OTH/BSI of 0.28.

A study undergone in Iceland [11] found that a conversion factor for RESP/BSI of 8.07.

Notably, no reducing factors (step 3) were applied for *S. pneumoniae*.

Table 4. Results of the literature search

	Time to React (2007) [6]	Vardham (2003) [7]	Janoff (2015) [8]	Demachy (2005) [9]	Hauser (2016) [10]	Hjalmarsdottir (2014) [11]
RESP/BSI	2.7	8.33	4	2.97	NA	8.07
OTH/BSI	NA	0.54	NA	0.33	0.28	NA

After expert opinion, it was considered that the publication from Demachy et al. [9] was the one with highest power and the following conversion factors were chosen:

Penicillin-resistant and combined penicillin- and macrolide-resistant *S. pneumoniae*			
	**Ratio**	**Lower CI**	**Upper CI**
**BSI**	1	1	1
**UTI**	0	0	0
**RESP**	2.97	2.7	8.33
**SSI**	0	0	0
**OTH**	0.33	0.28	0.54

Limitations

A number of limitations were identified and are listed here.
Limitations related to the main data source, 2015 EARS-Net data:

- EARS-Net data relies on blood culturing and diagnostic practices, which are heterogeneous across the EU/EEA. This variability mainly concerns the frequency of testing, where under-resources countries would test less and more severe/resistant infections. A potential effect could be the under-estimation of infection numbers and the over-representation of the resistance proportion.
- EARS-Net surveillance is based on sentinel laboratories, which are assumed to be a representative sample of the country. However, in some countries participating laboratories might be concentrated in specific regions and/or in selected reference hospitals focused on high-level tertiary care. In addition, for countries where not all laboratories/hospitals participate in EARS-Net, the distribution of healthcare level of the sample origin might affect the patient case-mix, with the potential to both over- and underestimate the total number of cases when applying a general population coverage estimate.
- In order to account for blood culturing propensity, stratification of countries according to the 2015 EU LabCap Indicator 1.32 (Blood culture test rate) could be explored, see Addendum 3 of this document. However, this information is incomplete for many countries and the denominator was not always homogeneously estimated across countries.
- Countries were able to allocate a range estimates for the country coverage, in an attempt to include the resulting uncertainties in the final estimations.
- EARS-Net collects antimicrobial susceptibility data of the first blood and/or cerebrospinal fluid isolate of every patient within the year (de-duplication of data). Therefore, re-hospitalised patients would not be included; however, it is important to note that data that are not de-duplicated are more likely to overestimate resistance rates and sequelae.

Limitations related to using the PPS 2011-2012 data for converting BSI to other infections sites and to account for S-BSI:

- In order to ensure an up-to-date epidemiological picture, we chose the latest available EARS-Net reported data, from 2015. The PPS with which we derive the BSI/non-BSI conversion factors and the reduction factors accounting for S-BSI was performed in 2011 and 2012 (depending on the country). The incidence of AMR infections might have changed between these years, although the extent to which this has an impact on the BSI/non-BSI ratio was deemed to be limited.
- A number of EARS-Net infections can be considered predominately HAIs (e.g. colistin-resistant infections, *Pseudomonas* and *Acinetobacter* infections) or community-associated infections (CAIs) (e.g. *Streptococcus pneumoniae*), whereas other infections may be HAI or CAI (e.g. MRSA, Third-generation cephalosporin-resistant *E. coli*). We chose to apply the same PPS BSI/non-BSI conversion factors and the reduction factors accounting for S-BSI, irrespective of the fact of the infection is HAI or CAI. At the present stage, no published literature on the evidence for different conversion factors in CAIs was retrieved, and it was not possible to differentiate between HAI and CAI conversion factors.
- The daily national prevalence of HAI in PPS is highly variable depending on the day of the measurement. Thus, the duration of different infection types may represent a potential limitation. Long-lasting infections have a higher probability of being registered in a cross-sectional study such as the PPS than infections lasting less time and/or those treated in a timely fashion. This might affect the ratio from BSI to other types of infections, potentially resulting in an under-estimation of non-BSI infections (i.e. in particular mild infections might be under-represented). We partially account for this limitation by applying and incidence approach to the estimation of the BSI/non-BSI conversion factors and the large uncertainty

intervals resulting from the Rhame and Sudderth formula estimation of the incidence.The estimation of non-BSIs might have been affected by the case-mix of patients, which could differ between hospitals.

- The PPS study does not collect information on penicillin-resistant and combined penicillin- and macrolide-resistant *S. pneumoniae.* Therefore, conversion factors were derived from the review of the published literature. Retrieved evidence was limited and may be a source of uncertainty.

References

1. European Centre for Disease Prevention and Control. Point prevalence survey of healthcare-associated infections and antimicrobial use in European acute care hospitals. Stockholm: ECDC; 2013. Available from: http://ecdc.europa.eu/en/publications/Publications/healthcare-associated-infections-antimicrobial-use-PPS.pdf
2. Cassini A, Plachouras D, Eckmanns T, Abu Sin M, Blank HP, Ducomble T, et al. Burden of Six Healthcare-Associated Infections on European Population Health: Estimating Incidence-Based Disability-Adjusted Life Years through a Population Prevalence-Based Modelling Study. PLoS Med. 2016 Oct;13(10):e1002150.
3. European Centre for Disease Prevention and Control. Antimicrobial resistance surveillance in Europe 2015. Annual Report of the European Antimicrobial Resistance Surveillance Network (EARS-Net). Stockholm: ECDC; 2017. Available from: http://ecdc.europa.eu/en/publications/Publications/antimicrobial-resistance-europe-2015.pdf
4. European Centre for Disease Prevention and Control. EARS-Net Reporting Protocol. July 2015. Stockholm: ECDC; 2015. Available from: http://ecdc.europa.eu/en/activities/surveillance/EARS-Net/Documents/2015-EARS-Net-reporting-protocol.pdf
5. Brown LD, Cai TT, DasGupta A. Interval Estimation for a Binomial Proportion. Statist. Sci. 16 (2001), no. 2, 101--133. doi:10.1214/ss/1009213286. http://projecteuclid.org/euclid.ss/1009213286.
6. The bacterial challenge: time to react. A call to narrow the gap between multidrug-resistant bacteria in the EU and the development of new antibacterial agents. ECCD/EMEA Joint Technical Report, 2009.
7. Vardhan MS, Allen KD. Epidemiology of penicillin-resistant pneumococci in a Merseyside Health District over a 14-year period. J Infect. 2003;46(1):23-9.
8. Janoff EN, Musher DM. Streptococcus pneumoniae: Clinical syndromes. Mandell, Douglas, and Bennett`s Principles and Practice of Infectious Diseases, Eighth Edition. 2015 ISBN: 978-1-4557-4801-3, Elsevier, Philadelphia. Page 2319
9. Demachy MC, Vernet-Garnier V, Cottin J, Cattier B, Chardon H, Chomarat M, et al. Antimicrobial resistance data on 16,756 Streptococcus pneumoniae isolates in 1999: A Pan-Regional Multicenter Surveillance Study in France. Microb Drug Resist. 2005 Winter;11(4):323-9.
10. Hauser C, Kronenberg A, Allemann A, Muhlemann K, Hilty M. Serotype/serogroup-specific antibiotic non-susceptibility of invasive and non-invasive Streptococcus pneumoniae, Switzerland, 2004 to 2014. Euro surveillance. 2016 May 26;21(21).
11. Hjalmarsdottir MA, Kristinsson KG. Epidemiology of penicillin-non-susceptible pneumococci in Iceland, 1995-2010. J Antimicrob Chemother. 2014 Apr;69(4):940-6.
12. European Centre for Disease Prevention and Control. European Centre for Disease Prevention and Control. EU Laboratory Capability Monitoring System (EULabCap) – Report on 2015 survey of EU/EEA country capabilities and capacities. Stockholm: ECDC; 2017. Available from: http://ecdc.europa.eu/en/publications/Publications/EULabCap_report-for-2015.pdf
13. Rhame FS, Sudderth WD. Incidence and prevalence as used in the analysis of the occurrence of nosocomial infections. Am J Epidemiol. 1981;113(1):1-11.
14.

Addendum 1

Infection site conversion factors for each SPDAR

**Colistin-/carbapenem-/multidrug-resistant *Acinetobacter* spp.***			
	**Ratio**	**Lower CI**	**Upper CI**
**BSI**	1.00	0.76	1.47
**UTI**	0.45	0.07	0.83
**RESP**	1.23	0.54	1.93
**SSI**	0.46	0.11	0.81
**OTH**	0.16	0.01	0.30

**Colistin-/carbapenem/third generation cephalosporin-resistant *E. coli****			
	**Ratio**	**Lower CI**	**Upper CI**
**BSI**	1.00	0.74	1.56
**UTI**	4.68	2.10	7.25
**RESP**	0.53	0.08	0.97
**SSI**	1.24	0.43	2.05
**OTH**	0.60	0.14	1.07

**Vancomycin-resistant *E. faecalis* and *E. faecium***			
	**Ratio**	**Lower CI**	**Upper CI**
**BSI**	1.00	0.59	3.25
**UTI**	0.75	0.00	1.87
**RESP**	0.00	0.00	0.00
**SSI**	1.88	0.00	4.13
**OTH**	1.63	0.00	3.64

Colistin-/carbapenem-resistant *K. pneumoniae**			
	**Ratio**	**Lower CI**	**Upper CI**
**BSI**	1.00	0.72	1.62
**UTI**	0.74	0.13	1.35
**RESP**	0.64	0.07	1.20
**SSI**	0.37	0.00	0.75
**OTH**	0.68	0.09	1.27

Third-generation cephalosporin- resistant *K. pneumoniae*			
	**Ratio**	**Lower CI**	**Upper CI**
**BSI**	1.00	0.78	1.34
**UTI**	1.52	0.79	2.29
**RESP**	0.94	0.44	1.47
**SSI**	0.43	0.16	0.72
**OTH**	0.50	0.21	0.81

Colistin-/carbapenem-/multidrug-resistant *P. aeruginosa**			
	**Ratio**	**Lower CI**	**Upper CI**
**BSI**	1.00	0.70	1.75
**UTI**	1.38	0.21	2.55
**RESP**	4.06	1.38	6.73
**SSI**	0.91	0.13	1.68
**OTH**	0.67	0.06	1.27

Meticillin-resistant *S. aureus*			
	**Ratio**	**Lower CI**	**Upper CI**
**BSI**	1.00	0.80	1.34
**UTI**	0.25	0.03	0.47
**RESP**	1.65	0.86	2.45
**SSI**	1.77	0.97	2.57
**OTH**	1.62	0.89	2.36

*When PPS did not provide information on given SPDAR, proxies were chosen, as detailed in the corresponding paragraph

Addendum 2

Secondary BSI reducing factors for each SPDAR*

Colistin-/carbapenem-/multidrug-resistant *Acinetobacter* spp.	
**BSI**	1
**UTI**	0.03
**RESP**	0.11
**SSI**	0.08
**OTH**	0.13

Colistin-/carbapenem-resistant *E. coli*	
**BSI**	1
**UTI**	0.25
**RESP**	0.00
**SSI**	0.00
**OTH**	0.00

Third-generation cephalosporin-resistant *E. coli*	
**BSI**	1
**UTI**	0.16
**RESP**	0.06
**SSI**	0.13
**OTH**	0.25

Vancomycin-resistant *E. faecalis* and *E. faecium*	
**BSI**	1
**UTI**	0.13
**RESP**	0.00
**SSI**	0.13
**OTH**	0.25

Colistin-/carbapenem-resistant *K. pneumoniae*	
**BSI**	1
**UTI**	0.10
**RESP**	0.19
**SSI**	0.06
**OTH**	0.18

Third-generation cephalosporin-resistant *K. pneumoniae*	
**BSI**	1
**UTI**	0.32
**RESP**	0.06
**SSI**	0.03
**OTH**	0.10

Colistin-/carbapenem-/multidrug-resistant *P. aeruginosa*	
**BSI**	1
**UTI**	0.00
**RESP**	0.08
**SSI**	0.16
**OTH**	0.12

Meticillin-resistant *S. aureus*	
**BSI**	1
**UTI**	0.00
**RESP**	0.11
**SSI**	0.09
**OTH**	0.11

*When PPS did not provide information on given SPDAR, proxies were chosen, as detailed in the corresponding paragraph

Addendum 3

Number of blood culture sets tested / 1 000 hospital inpatient days reported by EARS-Net participating hospitals from your country.

Country	Number of blood culture tests tested/1000 hospital inpatient days reported	Score 1.32
Austria	15.7	1
Belgium	NA	0
Bulgaria	8.2	1
Croatia	NA	0
Cyprus	41.4	2
Czech Republic	16.6	2
Denmark	NA	0
Estonia	17.8	2
Finland	NA	0
France	86.5	2
Germany	24.9	2
Greece	NA	0
Hungary	8.4	1
Iceland	43.1	2
Ireland	52.9	2
Italy	NA	0
Latvia	6.7	1
Lithuania	NA	0
Luxembourg	NA	0
Malta	22.7	2
Netherlands	NA	0
Norway	56.9	2
Poland	32.4	2
Portugal	64.6	2
Romania	NA	0
Slovakia	20.1	2
Slovenia	35.1	2
Spain	NA	0
Sweden	NA	0
United Kingdom	65.4	2

Score (will be calculated by the LabCap team):
0 = information not reported to EARS-Net
1 = low blood culture test utilisation rate/1000 patient days (first quartile)
2 = fair to high blood culture utilisation rate/1000 patient days (upper three quartiles)

Addendum 4

Imputation methodology applied for each antibiotic resistance-bacterium combination in each country for the redistribution of cases reported to EARS-Net with unknown age and/or sex cases

	3GCREC	CREC	ColREC	3GCRKP	CRKP	ColRKP	MDRACI	CRACI	ColRACI	MDRPA	CRPA	ColRPA	MRSA	VRE	PRSP	PMRSP
Austria	😐	☺	☺	☺	☺	☺	☺	☺	☺	☺	☺	☺	😐	☺	☺	☺
Belgium	☺	☺	☺	☺	☺	☺	☺	☺	☺	☺	☺	☺	😐	☺	☺	☺
Bulgaria	😐	☺	☺	😐	☺	☺	😐	😐	☺	☺	☺	☺	😐	☺	☺	☺
Cyprus	☺	☺	☺	😐	☺	☺	☺	😐	☹	☺	☺	☺	😐	☺	☺	☹
Czech Republic	☺	☺	☺	☺	☺	☺	☺	☺	☺	☺	☺	☺	☺	☺	☺	☺
Germany	😐	☺	☺	😐	☺	☺	☺	😐	☺	😐	😐	☺	😐	😐	☺	☺
Denmark	😐	☺	☺	☺	☺	☺	☺	☺	☺	☺	☺	☺	☺	☺	☹	☹
Estonia	☺	☺	☺	☺	☺	☺	☺	☺	☺	☺	☺	☺	☺	☺	☺	☺
Greece	☹	☹	☹	☹	☹	☹	☹	☹	☹	☹	☹	☹	☹	☹	NA	NA
Spain	☺	☺	☺	☺	☺	☺	☺	☺	☺	☺	☺	☺	😐	☺	☺	☺
Finland	☺	☺	☺	☺	☺	☺	☺	☺	☺	☺	☺	☺	☺	☺	☺	☺
France	☹	☹	☺	☹	😐	☺	☺	😐	☺	☹	😐	☺	☹	☹	☺	☺
Croatia	😐	☺	☺	😐	☺	☺	☺	😐	☺	😐	😐	☺	😐	😐	😐	😐
Hungary	☺	☺	☺	☺	☺	☺	☺	☺	☺	☺	☺	☺	☺	☺	☺	☺
Ireland	😐	☺	☺	☺	☺	☺	☺	☺	☺	☺	☺	☺	☺	☺	☺	☺
Iceland	☺	☺	☺	☺	☺	☺	☺	☺	☺	☺	☺	☺	☺	☺	☺	☺
Italy	☹	☹	☹	☹	☹	☹	☹	☹	☹	☹	☹	☹	☹	☹	☹	☹
Lithuania	☹	☺	☺	☹	☺	☺	☹	☹	☺	☹	☹	☺	☹	☹	☹	☹
Luxembourg	☺	☺	☺	☺	☺	☺	☺	☺	☺	☺	☺	☺	☺	☺	☺	☺
Latvia	☺	☺	☺	☺	☺	☺	☺	☺	☺	☺	☺	☺	☺	☺	☺	☺
Malta	☺	☺	☺	☺	☺	☺	☺	☺	☺	☺	☺	☺	☺	☺	☺	☺
Netherlands	☺	☺	☺	☺	☺	☺	☺	☺	☺	☺	☺	☺	☺	☺	☺	☺
Norway	☺	☺	☺	☺	☺	☺	☺	☺	☺	☺	☺	☺	☺	☺	☺	☺
Poland	☺	☺	☺	😐	☺	☺	☺	☺	☺	☺	☺	☺	☺	☺	☺	☺
Portugal	😐	☺	☺	😐	😐	☺	☺	😐	☺	☺	😐	☺	😐	😐	☺	☺
Romania	☺	☺	☺	☺	☺	☺	☺	☺	☺	☺	☺	☺	☺	☺	☺	☺
Sweden	😐	☺	☺	☺	☺	☺	☺	☺	☺	☺	☺	☺	☺	☺	😐	☺
Slovenia	☺	☺	☺	☺	☺	☺	☺	☺	☺	☺	☺	☺	☺	☺	☺	☺
Slovakia	☺	☺	☺	☺	☺	☺	☺	☺	☺	☺	☺	☺	☺	☺	☺	☺
United Kingdom	☺	☺	☺	☺	☺	☺	☺	☺	☺	☺	☺	☺	☺	☺	☺	☺

☺ No unknown cases reported

😐 Less than 40% of reported cases with unknown age and/or sex: redistribution of unknown cases based on imputation from the country-specific known cases for the same antibiotic resistance-bacterium combination

☹ More than 40% of reported data with unknown age and/or sex: redistribution of unknown cases based on imputation from EU/EEA cumulative known cases for the same antibiotic resistance-bacterium combination

Greece did not report data on *S. pneumoniae* isolates to EARS-Net in 2015

Burden of antimicrobial resistance

GATHER checklist and further analysis on MRSA and attribution as healthcare-associated infections

Written by Alessandro Cassini with contributions from Carl Suetens, Liselotte Diaz Högberg, Diamantis Plachouras, and Dominique Monnet

Contents

List of abbreviations 215

1. Estimation of proportion of infections due to antimicrobial resistant organisms that are healthcare-associated 216

2. Further analysis on MRSA trends between 2007 and 2015.................. 218

3. GATHER checklist.. 220

List of abbreviations

3GCREC	third-generation cephalosporin-resistant *E. coli*
3GCRKP	third-generation cephalosporin-resistant *K. pneumoniae*
AMR	antimicrobial resistance
BSI	bloodstream infection
CAI	Community-associated infections
CI	confidence interval
ColRACI	colistin-resistant *Acinetobacter* spp.
ColREC	colistin-resistant *E. coli*
ColRKP	colistin-resistant *K. pneumoniae*
ColRPA	colistin-resistant *P. aeruginosa*
CRACI	carbapenem-resistant *Acinetobacter* spp.
CREC	carbapenem-resistant *E. coli*
CRKP	carbapenem-resistant *K. pneumoniae*
CRPA	carbapenem-resistant *P. aeruginosa*
CSF	cerebral spine fluid
DALY	disability-adjusted life years
EARS-Net	European Antimicrobial Resistance Surveillance Network
EEA	European Economic Area
EU	European Union
HAI	healthcare-associated infection
LOS	length of stay
MDR	multidrug-resistant
MDRACI	multidrug-resistant *Acinetobacter* spp
MDRPA	multidrug-resistant *P. aeruginosa*
MRSA	meticillin-resistant *Staphylococcus aureus*
MS	Member State
OECD	Organisation for Economic Co-operation and Development
OTH	other infection site including digestive tract infections, skin and soft tissue infections (SSTI), eye, ear, nose or mouth infections, bone and joint infections, cardiovascular infections, reproductive tract infections and other less frequent infections
PMRSP	penicillin- and macrolide-resistant S. pneumoniae
PPS	point prevalence survey of healthcare-associated infections and antimicrobial use in acute care hospitals
PRSP	penicillin-resistant *S. pneumoniae*;
RESP	respiratory infections (including pneumonia, and low respiratory tract infection)
S-BSI	secondary BSI
SP	specified pathogens
SPDAR	specified pathogens with defined antimicrobial resistance
SSI	surgical site infection
UTI	urinary tract infection
VRE	vancomycin-resistant enterococci

Estimation of proportion of infections due to antimicrobial resistant organisms that are healthcare-associated

All *S. pneumoniae* (penicillin-resistant, combined macrolide- and penicillin-resistant) were assumed to be 100% community-associated infections (CAIs), while the following EARS-Net pathogens were assumed to be 100% HAIs:

- All infections resistant to carbapenems and/or colistin;
- *Acinetobacter* spp.;
- *Pseudomonas aeruginosa*;
- *Enterococcus faecalis* and *E. faecium*.

We estimated the proportion of HAIs and CAIs for the following infections due to antimicrobial resistant organisms:

- Third-generation cephalosporin-resistant *Klebsiella pneumoniae* and *Escherichia coli* (excluding those resistant to colistin and/or carbapenem);
- Meticillin-resistant *Staphylococcus aureus*.

Information from EARS-Net was initially used to split HAIs from CAIs. EARS-Net information on date of hospital admission is available for a minority of cases, varying for each SP and across countries. In 2015 the proportion of all EARS-Net cases with information on date of hospitalisation was around 23%. Information on the time of admission (hour: minute) is not available. Therefore, for each group of infections due to antimicrobial resistant organisms, the EU/EEA cumulative number of cases notified to EARS-Net with information on date of hospitalisation was used to estimate the proportion of HAIs and CAIs. HAI was defined as patient with positive isolate on day 3 or more, based on date of hospital admission. CAI was defined as a patient with a positive isolate before day 3 of admission.
However, as EARS-Net does not provide information on previous history of hospitalisation (e.g. readmission and patient transfer) the number is likely to underestimate the true proportion of HAIs. Therefore, for each specific pathogen/resistance combination, the resulting HAI/CAI proportion was further adjusted by applying the PPS percentage of HAIs present on hospital admission (assuming that a certain percentage of HAIs with recent discharge from a hospital [within 48 hours] was misclassified as CAI in EARS-Net).
Therefore, considering that $HAI_{EARS\text{-}Net} = HAI_{corrected} – x\% * HAI_{corrected}$ we used the following formula:

$$HAI_{corrected} = HAI_{EARS\text{-}Net} / (1 – x)$$

With x being the percentage of HAIs present on hospital admission from the PPS database.
The resulting HAI proportions were:

- Third-generation cephalosporin-resistant *Klebsiella pneumoniae* (excluding those resistant to colistin and/or carbapenem):
  - 68% (66-70%) were considered HAIs
- Third-generation cephalosporin-resistant *Escherichia coli* (excluding those resistant to colistin and/or carbapenem):
  - 43% (41-44%) were considered HAIs
- Meticillin-resistant *Staphylococcus aureus*:
  - 68% (66-70%) were considered HAIs

Our estimation of the proportion of infections with antibiotic-resistant bacteria that were considered HAIs or CAIs was inherently limited by our a priori classification of infections caused by several bacterial species or any isolate resistant to colistin or to carbapenem as 100% HAI, given the limited availability and quality of the data on the likely individual place of acquisition of the infections reported to EARS-Net. This over-simplification has introduced a misclassification bias towards overestimating the HAI fraction of total burden of disease.
Moreover, the data from EARS-Net do not distinguish between CAI and community onset infections. If not adjusted for, this might cause an underestimation of the true proportion of HAIs, as re-admissions with a HAI might be misclassified as CAI. We used the PPS percentage of HAIs present on hospital admission for each specific pathogen/resistance combination to adjust the number of CAIs reported from EARS-Net, hence limiting the impact of CAI misclassification.

Further analysis on MRSA trends between 2007 and 2015

As part of this study, the methodology developed to estimate the burden of AMR in 2015 was used with 2007 EARS-Net data in order to compare the epidemiological situation and trends across time.

From 2007 to 2015, the proportion of *Staphylococcus aureus* isolates that were resistant to meticillin in the EU/EEA decreased form 26.6% to 16.8%. the 2007 population-weighted EU/EEA mean percentage was determined by applying population-based weights to each country's data before calculating the arithmetic mean for all reporting countries. Country weightings were used to adjust for imbalances in reporting propensity and population coverage, as the total number of reported isolates by country in most cases does not reflect the population size. The weighting applied to each national data point represented the proportion of the country's population out of the total population of all countries included in the calculation. Annual population data were retrieved from the Eurostat online database However, in our study we found that between 2007 and 2015 the incidence of MRSA increased by 23% (6-41%). In order to partly explore the reasons behind this apparent discrepancy, we analysed the age-specific incidence in 2007 and 2015, see Figure 1.

Figure 1: age-group specific incidence rates of MRSA in 2007 and 2015. Median and 95% uncertainty intervals.

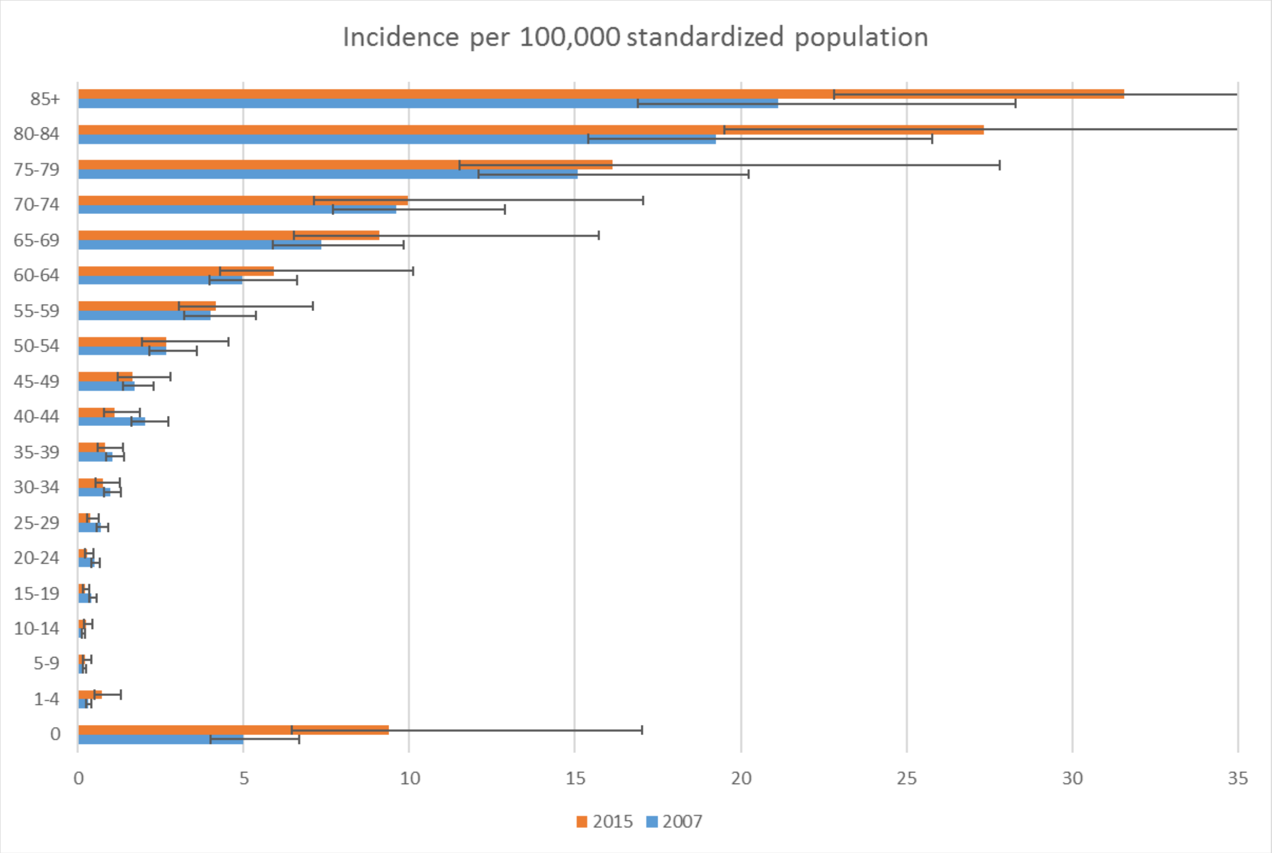

From Figure 1 it seems that the increase was experiences mainly in infants (and children under 4 years old) and in the elderly population (starting from those over 55 years old).

A number of reasons might explain the observed pattern:

- Increase of participating laboratories reporting EARS-Net and consequent country coverage;
- Increase in incidence of *S. aureus*

- Demographic increase in the elderly – ageing of the population which are more vulnerable and at higher chance of developing diseases, hence increasing the number of those at risk;
- Improvement of the neonatal services, increasing the survival of at-risk infants, hence increasing a vulnerable population at risk for infections;

The increase of MRSA incidence was also described in other studies:

1. Sarvikivi E, Ollgren J, Lyytikäinen O. Trends and outcome of healthcare-associated and community-onset bloodstream infections due to Staphylococcus aureus in Finland 2004-2015. European Scientific Conference on Applied Infectious Disease Epidemiology; 2017; Stockholm.
2. Swedres-Svarm 2016. Consumption of antibiotics and occurrence of resistance in Sweden. Solna/Uppsala ISSN1650-6332.

GATHER checklist

Checklist of information that should be included in new reports of global health estimates

Item #	Checklist item	Reported in manuscript and/or appendixes
**Objectives and funding**		
**1**	Define the indicator(s), populations (including age, sex, and geographic entities), and time period(s) for which estimates were made.	Information in the Methods section under "Study design and outcome measure" (indicator) and under "Study population and estimation of incidence" (population including age, sex, geography and time period)
**2**	List the funding sources for the work.	Statement available in the manuscript ("No specific funding was allocated for this study, which was conducted as part of routine work of ECDC and participating institutions.")
**Data Inputs**		
For all data inputs from multiple sources that are synthesized as part of the study:		
**3**	Describe how the data were identified and how the data were accessed.	Information available in the Methods section
**4**	Specify the inclusion and exclusion criteria. Identify all ad-hoc exclusions.	Information available in the Methods section
**5**	Provide information on all included data sources and their main characteristics. For each data source used, report reference information or contact name/institution, population represented, data collection method, year(s) of data collection, sex and age range, diagnostic criteria or measurement method, and sample size, as relevant.	Information available in the Methods section. Limitations related to data source and quality are available at the end of the Discussion section.
**6**	Identify and describe any categories of input data that have potentially important biases (e.g., based on characteristics listed in item 5).	Information available in the Methods section. Limitations related to data source and quality are available at the end of the Discussion section.
For data inputs that contribute to the analysis but were not synthesized as part of the study:		

**7**	Describe and give sources for any other data inputs.	Information available in the Methods section. Limitations related to data source and quality are available at the end of the Discussion section.
For all data inputs:		
**8**	Provide all data inputs in a file format from which data can be efficiently extracted (e.g., a spreadsheet rather than a PDF), including all relevant meta-data listed in item 5. For any data inputs that cannot be shared because of ethical or legal reasons, such as third-party ownership, provide a contact name or the name of the institution that retains the right to the data.	Information available in the Methods section and in Supplementary appendixes. Case-based data is not shared for ethical reasons but can be requested to country experts ("Burden of AMR Collaborative Group")
**Data analysis**		
**9**	Provide a conceptual overview of the data analysis method. A diagram may be helpful.	Information available in the Methods section and in Supplementary appendixes.
**10**	Provide a detailed description of all steps of the analysis, including mathematical formulae. This description should cover, as relevant, data cleaning, data pre-processing, data adjustments and weighting of data sources, and mathematical or statistical model(s).	Information available in the Methods section and in Supplementary appendixes.
**11**	Describe how candidate models were evaluated and how the final model(s) were selected.	Information available in the Methods section and in Supplementary appendixes.
**12**	Provide the results of an evaluation of model performance, if done, as well as the results of any relevant sensitivity analysis.	Information available in the Methods section and in Supplementary appendixes.
**13**	Describe methods for calculating uncertainty of the estimates. State which sources of uncertainty were, and were not, accounted for in the uncertainty analysis.	Information available in the Methods section and in Supplementary appendixes. Limitations related to data source and quality are available at the end of the Discussion section.
**14**	State how analytic or statistical source code used to generate estimates can be accessed.	Information available in the Methods section and in Supplementary appendixes.
**Results and Discussion**		
**15**	Provide published estimates in a file format from which data can be efficiently extracted.	Information available in the Results and Discussion sections, and in Supplementary appendixes.
**16**	Report a quantitative measure of the uncertainty of the estimates (e.g. uncertainty intervals).	Information available in the Results and Discussion sections, and in Supplementary appendixes.
**17**	Interpret results in light of existing evidence. If updating a previous set of estimates, describe the reasons for changes in estimates.	Information available in the Discussion section and in Supplementary appendixes.

**18**	Discuss limitations of the estimates. Include a discussion of any modelling assumptions or data limitations that affect interpretation of the estimates.	Information available in the Discussion section and in Supplementary appendixes.

Burden of antimicrobial resistance

Country specific results

Alessandro Cassini

Burden of antimicrobial resistance

Country specific results

Alessandro Cassini

17,706.86	20,562.69	23,408.62
2.5 % uncertainty	Median	97.5% uncertainty

3GCREC	third-generation cephalosporin-resistant *E. coli*
3GCRKP	third-generation cephalosporin-resistant *K. pneumoniae*
AMR	antimicrobial resistance
BSI	bloodstream infection
CAI	Community-associated infections
CI	confidence interval
ColRACI	colistin-resistant *Acinetobacter* spp.
ColREC	colistin-resistant *E. coli*
ColRKP	colistin-resistant *K. pneumoniae*
ColRPA	colistin-resistant *P. aeruginosa*
CRACI	carbapenem-resistant *Acinetobacter* spp.
CREC	carbapenem-resistant *E. coli*
CRKP	carbapenem-resistant *K. pneumoniae*
CRPA	carbapenem-resistant *P. aeruginosa*
CSF	cerebral spine fluid
DALY	disability-adjusted life years
EARS-Net	European Antimicrobial Resistance Surveillance Network
EEA	European Economic Area
EU	European Union
HAI	healthcare-associated infection
LOS	length of stay
MDR	multidrug-resistant
MDRACI	multidrug-resistant *Acinetobacter* spp
MDRPA	multidrug-resistant *P. aeruginosa*
MRSA	meticillin-resistant *Staphylococcus aureus*
MS	Member State
OECD	Organisation for Economic Co-operation and Development
OTH	other infection site including digestive tract infections, skin and soft tissue infections (SSTI), eye, ear, nose or mouth infections, bone and joint infections, cardiovascular infections, reproductive tract infections and other less frequent infections
PMRSP	penicillin- and macrolide-resistant S. pneumoniae
PPS	point prevalence survey of healthcare-associated infections and antimicrobial use in acute care hospitals
PRSP	penicillin-resistant *S. pneumoniae*;
RESP	respiratory infections (including pneumonia, and low respiratory tract infection)
S-BSI	secondary BSI
SP	specified pathogens
SPDAR	specified pathogens with defined antimicrobial resistance
SSI	surgical site infection
UTI	urinary tract infection
VRE	vancomycin-resistant enterococci

Infection	YLD			YLL			DALY			DALY per Case			YLD per 100,000		
Colistin resistant *Acinetobacter spp.*	457.22	524.78	596.79	2.235.67	2.788.66	3.394.41	2.720.88	3.314.19	3.976.79	7.65	9.07	10.58	0.09	0.10	0.12
Carbapenem resistant *Acinetobacter spp.* (excluding those resistant to colistin)	10,137.61	11,356.05	12,639.81	51,131.87	60,714.17	71,088.35	61,885.46	72,062.11	83,259.70	6.93	7.86	8.87	1.97	2.21	2.46
Aminoglycosides and fluoroquinolones resistant *Acinetobacter* spp. (excluding those resistant to colistin and/or carbapenem)	665.79	747.07	831.99	3,235.13	3,912.01	4,654.25	3,926.24	4,663.60	5,459.70	5.93	6.90	7.89	0.13	0.15	0.16
Vancomycin resistant Enterococci (*E. faecalis* and *E. faecium*)	2,297.49	2,699.67	3,215.58	21,742.25	25,509.67	30,074.34	24,033.96	28,223.26	33,244.92	6.18	6.70	7.33	0.45	0.53	0.63
Colistin resistant *Escherichia coli*	643.73	731.67	825.34	10,744.20	12,473.95	14,421.54	11,435.27	13,208.91	15,175.06	12.61	14.38	16.54	0.13	0.14	0.16
Carbapenem resistant *Escherichia coli* (excluding those resistant to colistin)	306.77	352.04	401.44	3,187.85	3,754.08	4,353.65	3,519.05	4,101.10	4,741.91	10.84	12.44	14.09	0.06	0.07	0.08
Third-generation cephalosporin resistant *Escherichia coli* (excluding those resistant to colistin and/or carbapenem)	24,348.48	27,655.43	31,161.25	143,743.01	163,372.77	185,130.96	168,565.98	191,127.06	214,981.67	4.70	5.14	5.64	4.74	5.38	6.06
Colistin resistant *Klebsiella pneumoniae*	2,041.86	2,364.62	2,716.36	35,076.83	41,708.08	48,711.50	36,976.93	44,035.45	51,345.52	15.68	17.93	20.20	0.40	0.46	0.53
Carbapenem resistant *Klebsiella pneumoniae* (excluding those resistant to colistin)	4,415.50	5,048.95	5,775.19	46,171.17	53,985.08	62,533.97	50,712.17	58,992.35	68,041.54	10.26	11.68	13.05	0.86	0.98	1.12
Third-generation cephalosporin resistant *Klebsiella pneumoniae* (excluding those resistant to colistin and/or carbapenem)	18,126.25	20,021.83	21,953.08	88,224.57	95,523.38	103,207.93	106,722.71	115,546.29	124,660.91	6.59	6.97	7.35	3.53	3.90	4.27
Colistin resistant *Pseudomonas aeruginosa*	414.44	496.03	585.54	1.930.83	2.510.29	3.157.62	2.373.82	3.007.09	3.706.76	8.69	10.67	12.84	0.08	0.10	0.11
Carbapenem resistant *Pseudomonas aeruginosa* (excluding those resistant to colistin)	20,316.09	23,189.72	26,234.73	96,716.06	116,604.32	139,420.87	118,131.39	139,831.71	164,327.31	8.64	10.02	11.29	3.95	4.51	5.10
Pseudomonas aeruginosa resistant to three or more antimicrobial groups (excluding those resistant to colistin and/or carbapenem)	2,449.52	2,815.97	3,249.53	10,669.81	13,319.44	16,346.41	13,381.34	16,141.82	19,304.91	6.68	7.92	9.16	0.48	0.55	0.63
Methicillin resistant *Staphylococcus aureus*	25.907.91	28.831.54	31.947.81	126.929.32	138.891.52	151.637.29	153.098.49	167.766.53	182.808.23	5.47	5.81	6.14	5.04	5.61	6.22
Penicillin and macrolides resistant *Streptococcus pneumoniae* (excluding those only resistant to penicillin)	1,097.16	1,261.59	1,509.49	2,957.32	3,341.28	3,900.33	4,077.11	4,601.09	5,374.64	8.44	9.05	9.79	0.21	0.25	0.29
Penicillin tesistant *Streptococcus pneumoniae* (excluding those resistant to macrolides)	1,651.03	1,856.81	2,104.56	5,581.19	6,057.78	6,617.21	7,276.05	7,918.61	8,658.90	8.78	9.20	9.67	0.32	0.36	0.41
Total	115,276.86	129,953.77	145,748.47	650,277.10	744,466.48	848,650.63	768,836.86	874,541.15	989,068.49				22.43	25.28	28.36

Infection	YLL per 100,000			DALY per 100,000			Cases per year			Incidence per 100,000			Deaths per year			Mortality per 100,000		
Colistin resistant *Acinetobacter spp.*	0.43	0.54	0.66	0.53	0.64	0.77	926.04	1.084.65	1.246.33	0.18	0.21	0.24	73.94	93.54	114.30	0.01	0.02	0.02
Carbapenem resistant *Acinetobacter spp.* (excluding those resistant to colistin)	9.95	11.81	13.83	12.04	14.02	16.20	24,063.67	27,342.65	30,793.69	4.68	5.32	5.99	1,947.02	2,362.70	2,809.51	0.38	0.46	0.55
Aminoglycosides and fluoroquinolones resistant *Acinetobacter* spp. (excluding those resistant to colistin and/or carbapenem)	0.63	0.76	0.91	0.76	0.91	1.06	1,775.87	2,012.55	2,251.84	0.35	0.39	0.44	140.54	172.08	205.62	0.03	0.03	0.04
Vancomycin resistant Enterococci (*E. faecalis* and *E. faecium*)	4.23	4.96	5.85	4.68	5.49	6.47	13,205.65	16,145.93	19,333.86	2.57	3.14	3.76	891.10	1,080.55	1,291.67	0.17	0.21	0.25
Colistin resistant *Escherichia coli*	2.09	2.43	2.81	2.22	2.57	2.95	6,107.04	7,156.05	8,241.41	1.19	1.39	1.60	518.08	621.46	751.38	0.10	0.12	0.15
Carbapenem resistant *Escherichia coli* (excluding those resistant to colistin)	0.62	0.73	0.85	0.68	0.80	0.92	2,268.97	2,618.97	2,960.52	0.44	0.51	0.58	118.70	141.38	165.33	0.02	0.03	0.03
Third-generation cephalosporin resistant *Escherichia coli* (excluding those resistant to colistin and/or carbapenem)	27.97	31.78	36.02	32.79	37.18	41.83	255,376.87	297,415.94	341,063.79	49.68	57.86	66.35	7,786.52	9,065.55	10,607.13	1.51	1.76	2.06
Colistin resistant *Klebsiella pneumoniae*	6.82	8.11	9.48	7.19	8.57	9.99	6,223.13	7,450.28	8,714.97	1.21	1.45	1.70	1,362.46	1,634.56	1,922.23	0.27	0.32	0.37
Carbapenem resistant *Klebsiella pneumoniae* (excluding those resistant to colistin)	8.98	10.50	12.17	9.87	11.48	13.24	13,473.30	15,946.79	18,477.96	2.62	3.10	3.59	1,794.85	2,118.08	2,472.64	0.35	0.41	0.48
Third-generation cephalosporin resistant *Klebsiella pneumoniae* (excluding those resistant to colistin and/or carbapenem)	17.16	18.58	20.08	20.76	22.48	24.25	61,458.60	68,588.12	76,067.65	11.96	13.34	14.80	3,370.17	3,686.52	4,031.20	0.66	0.72	0.78
Colistin resistant *Pseudomonas aeruginosa*	0.38	0.49	0.61	0.46	0.59	0.72	1.043.43	1.261.88	1.475.72	0.20	0.25	0.29	65.52	84.47	107.50	0.01	0.02	0.02
Carbapenem resistant *Pseudomonas aeruginosa* (excluding those resistant to colistin)	18.82	22.69	27.12	22.98	27.20	31.97	53,209.86	61,892.13	70,983.70	10.35	12.04	13.81	3,397.79	4,155.29	5,087.37	0.66	0.81	0.99
Pseudomonas aeruginosa resistant to three or more antimicrobial groups (excluding those resistant to colistin and/or carbapenem)	2.08	2.59	3.18	2.60	3.14	3.76	7,735.67	9,028.27	10,425.40	1.50	1.76	2.03	456.30	572.31	703.19	0.09	0.11	0.14
Methicillin resistant *Staphylococcus aureus*	24.69	27.02	29.50	29.79	32.64	35.57	131.756.82	148.727.41	166.361.33	25.63	28.94	32.37	6.307.61	7.049.41	7.863.35	1.23	1.37	1.53
Penicillin and macrolides resistant *Streptococcus pneumoniae* (excluding those only resistant to penicillin)	0.58	0.65	0.76	0.79	0.90	1.05	1,942.77	2,181.52	2,448.96	0.38	0.42	0.48	89.50	100.45	112.65	0.02	0.02	0.02
Penicillin tesistant *Streptococcus pneumoniae* (excluding those resistant to macrolides)	1.09	1.18	1.29	1.42	1.54	1.68	2,580.71	2,836.19	3,118.64	0.50	0.55	0.61	159.83	171.84	184.92	0.03	0.03	0.04
Total	126.51	144.84	165.11	149.58	170.14	192.43	583,148.39	671,689.34	763,965.77	113.45	130.68	148.63	28,479.94	33,110.20	38,429.98	5.54	6.44	7.48

Model	YLD			YLL			DALY			DALY per Case			YLD per 100,000		
EU + EEA - Acinetobacter spp. CRACI BSI - model 36	5,839.88	6,461.52	7,126.11	41,501.12	49,674.89	58,656.88	47,930.71	56,135.57	65,351.08	5.20	6.01	6.89	1.14	1.26	1.39
EU + EEA - Acinetobacter spp. CRACI OTH - model 40	0.93	1.33	1.76	0.00	0.00	0.00	0.93	1.33	1.76	3.09E-03	3.76E-03	4.42E-03	1.81E-04	2.59E-04	3.42E-04
EU + EEA - Acinetobacter spp. CRACI RESP - model 37	4,285.19	4,877.75	5,492.01	8,391.03	9,527.81	10,635.89	12,701.86	14,398.18	16,089.25	1.33	1.41	1.50	0.83	0.95	1.07
EU + EEA - Acinetobacter spp. CRACI SSI - model 39	5.49	7.48	9.79	1,239.73	1,511.48	1,795.58	1,245.84	1,519.07	1,807.47	0.40	0.43	0.47	1.07E-03	1.46E-03	1.90E-03
EU + EEA - Acinetobacter spp. CRACI UTI - model 38	6.12	7.97	10.14	0.00	0.00	0.00	6.12	7.97	10.14	1.68E-03	2.04E-03	2.43E-03	1.19E-03	1.55E-03	1.97E-03
EU + EEA - Acinetobacter spp. ColRACI BSI - model 41	261.93	297.84	334.48	1,810.82	2,289.23	2,815.52	2,103.40	2,587.00	3,141.13	5.73	6.99	8.33	0.05	0.06	0.07
EU + EEA - Acinetobacter spp. ColRACI OTH - model 45	0.04	0.05	0.07	0.00	0.00	0.00	0.04	0.05	0.07	2.95E-03	3.73E-03	4.57E-03	6.90E-06	1.00E-05	1.43E-05
EU + EEA - Acinetobacter spp. ColRACI RESP - model 42	194.82	226.28	261.43	377.46	440.19	506.99	569.61	667.29	763.04	1.51	1.64	1.78	0.04	0.04	0.05
EU + EEA - Acinetobacter spp. ColRACI SSI - model 44	0.20	0.29	0.40	47.39	59.24	71.90	47.61	59.54	72.15	0.40	0.43	0.47	3.94E-05	5.71E-05	7.78E-05
EU + EEA - Acinetobacter spp. ColRACI UTI - model 43	0.23	0.31	0.41	0.00	0.00	0.00	0.23	0.31	0.41	1.61E-03	2.04E-03	2.47E-03	4.44E-05	6.11E-05	7.98E-05
EU + EEA - Acinetobacter spp. MDRACI BSI - model 31	386.03	425.16	466.91	2,598.52	3,181.85	3,829.37	3,004.60	3,610.57	4,275.70	4.38	5.22	6.08	0.08	0.08	0.09
EU + EEA - Acinetobacter spp. MDRACI OTH - model 35	0.07	0.10	0.13	0.00	0.00	0.00	0.07	0.10	0.13	3.06E-03	3.75E-03	4.52E-03	1.29E-05	1.85E-05	2.46E-05
EU + EEA - Acinetobacter spp. MDRACI RESP - model 32	278.86	320.69	363.50	549.53	624.10	698.62	833.13	945.66	1,056.06	1.16	1.25	1.35	0.05	0.06	0.07
EU + EEA - Acinetobacter spp. MDRACI SSI - model 34	0.40	0.54	0.72	87.09	106.07	126.25	88.00	106.69	127.09	0.39	0.42	0.45	7.71E-05	1.06E-04	1.40E-04
EU + EEA - Acinetobacter spp. MDRACI UTI - model 33	0.44	0.58	0.74	0.00	0.00	0.00	0.44	0.58	0.74	1.67E-03	2.04E-03	2.46E-03	8.64E-05	1.13E-04	1.43E-04
EU + EEA - Enterococcus faecalis and E. faecium VRE BSI - model 66	2,280.69	2,676.63	3,184.67	19,760.50	22,990.83	26,952.06	22,036.80	25,679.74	30,092.01	5.76	6.24	6.83	0.44	0.52	0.62
EU + EEA - Enterococcus faecalis and E. faecium VRE OTH - model 70	5.39	7.11	9.39	0.00	0.00	0.00	5.39	7.11	9.39	1.34E-03	1.64E-03	2.02E-03	1.05E-03	1.38E-03	1.83E-03
EU + EEA - Enterococcus faecalis and E. faecium VRE RESP - model 67	0.00	0.00	0.00	0.00	0.00	0.00	0.00	0.00	0.00	NaN	NaN	NaN	0.00	0.00	0.00
EU + EEA - Enterococcus faecalis and E. faecium VRE SSI - model 69	8.46	12.06	16.39	1,981.76	2,518.85	3,122.27	1,988.83	2,532.54	3,138.39	0.41	0.45	0.49	1.65E-03	2.35E-03	3.19E-03
EU + EEA - Enterococcus faecalis and E. faecium VRE UTI - model 68	2.94	3.88	5.13	0.00	0.00	0.00	2.94	3.88	5.13	1.52E-03	1.86E-03	2.23E-03	5.73E-04	7.54E-04	9.99E-04
EU + EEA - Escherichia coli 3GCREC BSI - model 16	18,585.72	20,672.94	22,851.09	118,387.30	133,097.17	149,526.01	137,533.75	153,909.13	171,249.65	3.41	3.72	4.04	3.62	4.02	4.45
EU + EEA - Escherichia coli 3GCREC OTH - model 20	33.93	46.62	62.17	0.00	0.00	0.00	33.93	46.62	62.17	2.64E-03	3.36E-03	4.06E-03	6.60E-03	9.07E-03	0.01
EU + EEA - Escherichia coli 3GCREC RESP - model 17	5,389.03	6,492.06	7,669.89	10,555.71	12,559.28	14,823.00	15,864.13	19,005.76	22,379.93	0.91	1.01	1.16	1.05	1.26	1.49
EU + EEA - Escherichia coli 3GCREC SSI - model 19	69.64	93.85	126.82	14,800.00	17,716.31	20,781.95	14,864.02	17,815.60	20,838.64	0.37	0.40	0.43	0.01	0.02	0.02
EU + EEA - Escherichia coli 3GCREC UTI - model 18	270.15	349.95	451.28	0.00	0.00	0.00	270.15	349.95	451.28	1.59E-03	1.96E-03	2.39E-03	0.05	0.07	0.09
EU + EEA - Escherichia coli CREC BSI - model 21	228.03	256.99	288.23	2,890.06	3,397.54	3,936.56	3,142.82	3,649.62	4,213.92	9.13	10.51	11.91	0.04	0.05	0.06
EU + EEA - Escherichia coli CREC OTH - model 25	0.53	0.70	0.93	0.00	0.00	0.00	0.53	0.70	0.93	2.79E-03	3.50E-03	4.41E-03	1.03E-04	1.36E-04	1.81E-04
EU + EEA - Escherichia coli CREC RESP - model 22	75.19	90.40	107.18	144.14	175.58	208.40	218.81	265.94	313.80	1.29	1.48	1.71	0.01	0.02	0.02
EU + EEA - Escherichia coli CREC SSI - model 24	0.65	0.88	1.16	153.65	180.96	208.70	154.52	181.77	209.32	0.41	0.44	0.47	1.27E-04	1.71E-04	2.26E-04
EU + EEA - Escherichia coli CREC UTI - model 23	2.37	3.07	3.94	0.00	0.00	0.00	2.37	3.07	3.94	1.67E-03	2.10E-03	2.55E-03	4.61E-04	5.98E-04	7.66E-04
EU + EEA - Escherichia coli ColREC BSI - model 26	478.65	532.45	593.05	10,038.39	11,639.35	13,457.27	10,563.17	12,174.61	13,981.34	11.24	12.84	14.80	0.09	0.10	0.12
EU + EEA - Escherichia coli ColREC OTH - model 30	1.39	1.95	2.74	0.00	0.00	0.00	1.39	1.95	2.74	2.69E-03	3.51E-03	4.61E-03	2.71E-04	3.79E-04	5.33E-04
EU + EEA - Escherichia coli ColREC RESP - model 27	155.64	186.51	215.51	302.23	361.29	415.13	459.48	548.14	627.34	0.98	1.12	1.28	0.03	0.04	0.04
EU + EEA - Escherichia coli ColREC SSI - model 29	1.73	2.39	3.29	403.57	473.31	549.15	404.91	475.84	552.91	0.39	0.42	0.45	3.37E-04	4.65E-04	6.40E-04
EU + EEA - Escherichia coli ColREC UTI - model 28	6.32	8.37	10.74	0.00	0.00	0.00	6.32	8.37	10.74	1.63E-03	2.09E-03	2.61E-03	1.23E-03	1.63E-03	2.09E-03
EU + EEA - Klebsiella pneumoniae 3GCRKP BSI - model 1	11,361.18	12,394.50	13,452.19	72,431.36	77,722.21	83,340.90	84,112.69	90,092.50	96,381.25	4.83	5.08	5.34	2.21	2.41	2.62
EU + EEA - Klebsiella pneumoniae 3GCRKP OTH - model 5	18.84	23.83	29.87	0.00	0.00	0.00	18.84	23.83	29.87	2.77E-03	3.34E-03	4.02E-03	3.67E-03	4.64E-03	5.81E-03
EU + EEA - Klebsiella pneumoniae 3GCRKP RESP - model 2	6,701.57	7,546.87	8,400.76	13,095.24	14,639.94	16,235.61	19,843.79	22,211.32	24,548.46	1.33	1.43	1.54	1.30	1.47	1.63
EU + EEA - Klebsiella pneumoniae 3GCRKP SSI - model 4	11.56	15.14	19.96	2,697.97	3,161.23	3,631.42	2,714.28	3,177.15	3,651.01	0.42	0.45	0.47	2.25E-03	2.95E-03	3.88E-03
EU + EEA - Klebsiella pneumoniae 3GCRKP UTI - model 3	33.11	41.49	50.31	0.00	0.00	0.00	33.11	41.49	50.31	1.63E-03	1.96E-03	2.31E-03	6.44E-03	8.07E-03	9.79E-03
EU + EEA - Klebsiella pneumoniae CRKP BSI - model 6	3,449.19	3,872.75	4,365.28	43,729.02	50,988.85	58,934.05	47,292.27	54,820.25	63,050.45	8.60	9.84	11.00	0.67	0.75	0.85
EU + EEA - Klebsiella pneumoniae CRKP OTH - model 10	7.15	9.50	12.35	0.00	0.00	0.00	7.15	9.50	12.35	2.81E-03	3.51E-03	4.25E-03	1.39E-03	1.85E-03	2.40E-03
EU + EEA - Klebsiella pneumoniae CRKP RESP - model 7	950.96	1,155.68	1,383.47	1,848.94	2,240.89	2,665.68	2,811.71	3,397.02	4,031.03	1.26	1.39	1.56	0.19	0.22	0.27
EU + EEA - Klebsiella pneumoniae CRKP SSI - model 9	2.68	3.70	4.89	593.20	755.34	934.23	595.52	758.25	938.51	0.40	0.44	0.48	5.21E-04	7.20E-04	9.51E-04
EU + EEA - Klebsiella pneumoniae CRKP UTI - model 8	5.52	7.33	9.20	0.00	0.00	0.00	5.52	7.33	9.20	1.71E-03	2.09E-03	2.50E-03	1.07E-03	1.43E-03	1.79E-03
EU + EEA - Klebsiella pneumoniae ColRKP BSI - model 11	1,604.08	1,810.46	2,052.07	33,962.01	40,300.69	47,012.29	35,424.81	42,073.97	48,987.66	14.01	16.09	18.16	0.31	0.35	0.40
EU + EEA - Klebsiella pneumoniae ColRKP OTH - model 15	3.26	4.41	5.85	0.00	0.00	0.00	3.26	4.41	5.85	2.81E-03	3.52E-03	4.33E-03	6.34E-04	8.59E-04	1.14E-03
EU + EEA - Klebsiella pneumoniae ColRKP RESP - model 12	430.77	544.67	651.66	846.69	1,059.98	1,264.10	1,277.06	1,604.27	1,910.26	1.27	1.40	1.55	0.08	0.11	0.13
EU + EEA - Klebsiella pneumoniae ColRKP SSI - model 14	1.22	1.69	2.34	268.13	347.40	435.11	269.27	349.42	437.30	0.39	0.44	0.48	2.37E-04	3.30E-04	4.56E-04
EU + EEA - Klebsiella pneumoniae ColRKP UTI - model 13	2.54	3.38	4.44	0.00	0.00	0.00	2.54	3.38	4.44	1.69E-03	2.09E-03	2.54E-03	4.93E-04	6.57E-04	8.65E-04
EU + EEA - Pseudomonas aeruginosa CRPA BSI - model 51	5,844.74	6,500.84	7,287.13	45,552.59	55,274.57	66,218.66	52,137.77	61,723.63	73,112.51	6.15	7.19	8.14	1.14	1.26	1.42
EU + EEA - Pseudomonas aeruginosa CRPA OTH - model 55	12.46	16.13	20.62	0.00	0.00	0.00	12.46	16.13	20.62	3.03E-03	3.65E-03	4.31E-03	2.42E-03	3.14E-03	4.01E-03
EU + EEA - Pseudomonas aeruginosa CRPA RESP - model 52	14,431.29	16,637.38	18,881.62	49,071.82	58,743.30	70,091.18	63,859.43	75,470.76	88,038.73	2.09	2.39	2.67	2.81	3.24	3.67
EU + EEA - Pseudomonas aeruginosa CRPA SSI - model 54	9.44	12.66	16.74	2,091.65	2,586.45	3,111.02	2,103.57	2,598.48	3,126.83	0.40	0.44	0.47	1.84E-03	2.46E-03	3.26E-03
EU + EEA - Pseudomonas aeruginosa CRPA UTI - model 53	18.15	22.71	28.61	0.00	0.00	0.00	18.15	22.71	28.61	1.71E-03	2.05E-03	2.40E-03	3.53E-03	4.42E-03	5.57E-03
EU + EEA - Pseudomonas aeruginosa ColRPA BSI - model 56	119.66	137.94	161.31	914.78	1,185.34	1,504.30	1,041.49	1,323.02	1,635.93	6.18	7.68	9.37	0.02	0.03	0.03
EU + EEA - Pseudomonas aeruginosa ColRPA OTH - model 60	0.24	0.33	0.45	0.00	0.00	0.00	0.24	0.33	0.45	3.01E-03	3.76E-03	4.57E-03	4.74E-05	6.49E-05	8.81E-05
EU + EEA - Pseudomonas aeruginosa ColRPA RESP - model 57	294.01	357.03	422.80	972.53	1,268.63	1,583.68	1,287.83	1,626.68	1,999.75	2.08	2.52	2.95	0.06	0.07	0.08
EU + EEA - Pseudomonas aeruginosa ColRPA SSI - model 59	0.18	0.26	0.37	43.52	56.31	69.64	43.92	56.58	70.02	0.43	0.47	0.50	3.45E-05	5.05E-05	7.16E-05
EU + EEA - Pseudomonas aeruginosa ColRPA UTI - model 58	0.34	0.46	0.61	0.00	0.00	0.00	0.34	0.46	0.61	1.62E-03	2.05E-03	2.50E-03	6.61E-05	9.03E-05	1.19E-04
EU + EEA - Pseudomonas aeruginosa MDRPA BSI - model 46	694.79	787.00	893.39	4,931.01	6,157.65	7,532.02	5,725.34	6,949.49	8,341.16	4.65	5.59	6.54	0.14	0.15	0.17
EU + EEA - Pseudomonas aeruginosa MDRPA OTH - model 50	1.83	2.41	3.12	0.00	0.00	0.00	1.83	2.41	3.12	3.13E-03	3.74E-03	4.49E-03	3.56E-04	4.69E-04	6.07E-04
EU + EEA - Pseudomonas aeruginosa MDRPA RESP - model 47	1,749.03	2,021.36	2,346.18	5,457.29	6,806.58	8,385.19	7,368.79	8,829.57	10,524.04	1.65	1.91	2.17	0.34	0.39	0.46
EU + EEA - Pseudomonas aeruginosa MDRPA SSI - model 49	1.35	1.87	2.52	281.51	355.21	429.19	282.86	357.02	432.30	0.37	0.41	0.45	2.62E-04	3.63E-04	4.91E-04
EU + EEA - Pseudomonas aeruginosa MDRPA UTI - model 48	2.52	3.33	4.30	0.00	0.00	0.00	2.52	3.33	4.30	1.67E-03	2.04E-03	2.45E-03	4.91E-04	6.47E-04	8.37E-04
EU + EEA - Staphylococcus aureus MRSA BSI - model 61	12,672.50	13,889.12	15,270.49	86,406.78	93,284.62	100,497.18	99,222.78	107,206.04	115,174.81	4.03	4.26	4.49	2.47	2.70	2.97
EU + EEA - Staphylococcus aureus MRSA OTH - model 65	95.76	121.75	150.83	0.00	0.00	0.00	95.76	121.75	150.83	2.67E-03	3.26E-03	3.88E-03	0.02	0.02	0.03
EU + EEA - Staphylococcus aureus MRSA RESP - model 62	13,064.24	14,720.16	16,394.84	25,533.71	28,448.38	31,710.93	38,704.84	43,173.97	47,941.89	1.05	1.13	1.21	2.54	2.86	3.19
EU + EEA - Staphylococcus aureus MRSA SSI - model 64	66.51	88.63	116.22	14,988.83	17,158.52	19,429.18	15,066.20	17,252.90	19,525.28	0.39	0.41	0.44	0.01	0.02	0.02
EU + EEA - Staphylococcus aureus MRSA UTI - model 63	8.91	11.88	15.42	0.00	0.00	0.00	8.91	11.88	15.42	1.50E-03	1.88E-03	2.28E-03	1.73E-03	2.31E-03	3.00E-03
EU + EEA - Streptococcus pneumoniae PMRSP BSI - model 76	162.34	180.23	203.66	1,130.32	1,227.87	1,357.07	1,299.50	1,408.69	1,555.89	6.86	7.28	7.79	0.03	0.04	0.04
EU + EEA - Streptococcus pneumoniae PMRSP OTH - model 80	0.26	0.33	0.40	0.00	0.00	0.00	0.26	0.33	0.40	1.58E-03	1.96E-03	2.32E-03	5.13E-05	6.38E-05	7.75E-05
EU + EEA - Streptococcus pneumoniae PMRSP RESP - model 77	934.56	1,081.03	1,305.43	1,827.00	2,113.41	2,543.26	2,777.35	3,192.07	3,818.35	1.58	1.77	2.00	0.18	0.21	0.25
EU + EEA - Streptococcus pneumoniae PMRSP SSI - model 79	0.00	0.00	0.00	0.00	0.00	0.00	0.00	0.00	0.00	NaN	NaN	NaN	0.00	0.00	0.00
EU + EEA - Streptococcus pneumoniae PMRSP UTI - model 78	0.00	0.00	0.00	0.00	0.00	0.00	0.00	0.00	0.00	NaN	NaN	NaN	0.00	0.00	0.00
EU + EEA - Streptococcus pneumoniae PRSP BSI - model 71	460.51	497.71	533.82	3,235.66	3,388.30	3,535.87	3,722.93	3,884.84	4,039.47	7.01	7.28	7.58	0.09	0.10	0.10
EU + EEA - Streptococcus pneumoniae PRSP OTH - model 75	0.32	0.38	0.45	0.00	0.00	0.00	0.32	0.38	0.45	1.65E-03	1.96E-03	2.29E-03	6.23E-05	7.37E-05	8.75E-05
EU + EEA - Streptococcus pneumoniae PRSP RESP - model 72	1,190.20	1,358.72	1,570.29	2,345.53	2,669.49	3,081.34	3,552.80	4,033.38	4,618.99	1.76	1.92	2.09	0.23	0.26	0.31
EU + EEA - Streptococcus pneumoniae PRSP SSI - model 74	0.00	0.00	0.00	0.00	0.00	0.00	0.00	0.00	0.00	NaN	NaN	NaN	0.00	0.00	0.00
EU + EEA - Streptococcus pneumoniae PRSP UTI - model 73	0.00	0.00	0.00	0.00	0.00	0.00	0.00	0.00	0.00	NaN	NaN	NaN	0.00	0.00	0.00
**Total (Median & 95% UIs)**	115,276.86	129,953.77	145,748.47	650,277.10	744,466.48	848,650.63	768,836.86	874,541.15	989,068.49				22.43	25.28	28.36
**Total (average)**	**130,108.61**			**745,541.51**			**875,650.12**			-			**25.31**		

Model	YLL per 100,000			DALY per 100,000			Cases per year			Incidence per 100,000			Deaths per year			Mortality per 100,000		
EU + EEA - Acinetobacter spp. CRACI BSI - model 36	8.07	9.66	11.41	9.33	10.92	12.71	8,599.72	9,339.76	10,191.59	1.67	1.82	1.98	1,563.08	1,913.83	2,293.42	0.30	0.37	0.45
EU + EEA - Acinetobacter spp. CRACI OTH - model 40	0.00	0.00	0.00	1.81E-04	2.59E-04	3.42E-04	269.13	356.11	449.34	0.05	0.07	0.09	0.00	0.00	0.00	0.00	0.00	0.00
EU + EEA - Acinetobacter spp. CRACI RESP - model 37	1.63	1.85	2.07	2.47	2.80	3.13	8,972.57	10,216.67	11,500.88	1.75	1.99	2.24	318.09	367.52	417.51	0.06	0.07	0.08
EU + EEA - Acinetobacter spp. CRACI SSI - model 39	0.24	0.29	0.35	0.24	0.30	0.35	2,954.69	3,507.28	4,053.70	0.57	0.68	0.79	65.85	81.34	98.58	0.01	0.02	0.02
EU + EEA - Acinetobacter spp. CRACI UTI - model 38	0.00	0.00	0.00	1.19E-03	1.55E-03	1.97E-03	3,267.57	3,922.83	4,598.18	0.64	0.76	0.89	0.00	0.00	0.00	0.00	0.00	0.00
EU + EEA - Acinetobacter spp. ColRACI BSI - model 41	0.35	0.45	0.55	0.41	0.50	0.61	335.84	371.22	408.69	0.07	0.07	0.08	59.37	75.99	93.73	0.01	0.01	0.02
EU + EEA - Acinetobacter spp. ColRACI OTH - model 45	0.00	0.00	0.00	6.90E-06	1.00E-05	1.43E-05	9.94	13.80	18.56	1.93E-03	2.69E-03	3.61E-03	0.00	0.00	0.00	0.00	0.00	0.00
EU + EEA - Acinetobacter spp. ColRACI RESP - model 42	0.07	0.09	0.10	0.11	0.13	0.15	346.36	407.84	466.90	0.07	0.08	0.09	12.43	14.66	16.95	2.42E-03	2.85E-03	3.30E-03
EU + EEA - Acinetobacter spp. ColRACI SSI - model 44	9.22E-03	0.01	0.01	9.26E-03	0.01	0.01	110.86	137.30	164.28	0.02	0.03	0.03	2.13	2.89	3.61	4.15E-04	5.62E-04	7.03E-04
EU + EEA - Acinetobacter spp. ColRACI UTI - model 43	0.00	0.00	0.00	4.44E-05	6.11E-05	7.98E-05	123.04	154.49	187.90	0.02	0.03	0.04	0.00	0.00	0.00	0.00	0.00	0.00
EU + EEA - Acinetobacter spp. MDRACI BSI - model 31	0.51	0.62	0.75	0.58	0.70	0.83	637.88	692.68	746.73	0.12	0.13	0.15	111.53	138.44	167.17	0.02	0.03	0.03
EU + EEA - Acinetobacter spp. MDRACI OTH - model 35	0.00	0.00	0.00	1.29E-05	1.85E-05	2.46E-05	19.47	25.45	32.22	3.79E-03	4.95E-03	6.27E-03	0.00	0.00	0.00	0.00	0.00	0.00
EU + EEA - Acinetobacter spp. MDRACI RESP - model 32	0.11	0.12	0.14	0.16	0.18	0.21	665.28	754.20	838.47	0.13	0.15	0.16	23.79	27.11	30.56	4.63E-03	5.27E-03	5.95E-03
EU + EEA - Acinetobacter spp. MDRACI SSI - model 34	0.02	0.02	0.02	0.02	0.02	0.02	214.83	254.66	298.56	0.04	0.05	0.06	5.22	6.53	7.88	1.02E-03	1.27E-03	1.53E-03
EU + EEA - Acinetobacter spp. MDRACI UTI - model 33	0.00	0.00	0.00	8.64E-05	1.13E-04	1.43E-04	238.41	285.55	335.86	0.05	0.06	0.07	0.00	0.00	0.00	0.00	0.00	0.00
EU + EEA - Enterococcus faecalis and E. faecium VRE BSI - model 66	3.84	4.47	5.24	4.29	5.00	5.85	3,433.94	4,109.07	4,866.95	0.67	0.80	0.95	783.29	938.44	1,110.65	0.15	0.18	0.22
EU + EEA - Enterococcus faecalis and E. faecium VRE OTH - model 70	0.00	0.00	0.00	1.05E-03	1.38E-03	1.83E-03	3,564.88	4,350.09	5,195.14	0.69	0.85	1.01	0.00	0.00	0.00	0.00	0.00	0.00
EU + EEA - Enterococcus faecalis and E. faecium VRE RESP - model 67	0.00	0.00	0.00	0.00	0.00	0.00	0.00	0.00	0.00	0.00	0.00	0.00	0.00	0.00	0.00	0.00	0.00	0.00
EU + EEA - Enterococcus faecalis and E. faecium VRE SSI - model 69	0.39	0.49	0.61	0.39	0.49	0.61	4,558.85	5,607.00	6,762.02	0.89	1.09	1.32	107.81	142.11	181.02	0.02	0.03	0.04
EU + EEA - Enterococcus faecalis and E. faecium VRE UTI - model 68	0.00	0.00	0.00	5.73E-04	7.54E-04	9.99E-04	1,647.97	2,079.77	2,509.76	0.32	0.40	0.49	0.00	0.00	0.00	0.00	0.00	0.00
EU + EEA - Escherichia coli 3GCREC BSI - model 16	23.03	25.89	29.09	26.76	29.94	33.32	37,228.68	41,377.64	46,011.92	7.24	8.05	8.95	6,209.48	7,121.68	8,280.67	1.21	1.39	1.61
EU + EEA - Escherichia coli 3GCREC OTH - model 20	0.00	0.00	0.00	6.60E-03	9.07E-03	0.01	11,110.58	14,027.11	17,124.26	2.16	2.73	3.33	0.00	0.00	0.00	0.00	0.00	0.00
EU + EEA - Escherichia coli 3GCREC RESP - model 17	2.05	2.44	2.88	3.09	3.70	4.35	15,131.89	18,784.60	22,459.11	2.94	3.65	4.37	548.65	673.87	817.49	0.11	0.13	0.16
EU + EEA - Escherichia coli 3GCREC SSI - model 19	2.88	3.45	4.04	2.89	3.47	4.05	37,482.37	44,320.65	51,360.92	7.29	8.62	9.99	1,028.38	1,270.01	1,508.97	0.20	0.25	0.29
EU + EEA - Escherichia coli 3GCREC UTI - model 18	0.00	0.00	0.00	0.05	0.07	0.09	154,423.34	178,905.95	204,107.58	30.04	34.81	39.71	0.00	0.00	0.00	0.00	0.00	0.00
EU + EEA - Escherichia coli CREC BSI - model 21	0.56	0.66	0.77	0.61	0.71	0.82	316.85	348.29	378.97	0.06	0.07	0.07	104.82	124.53	145.32	0.02	0.02	0.03
EU + EEA - Escherichia coli CREC OTH - model 25	0.00	0.00	0.00	1.03E-04	1.36E-04	1.81E-04	168.22	200.37	234.66	0.03	0.04	0.05	0.00	0.00	0.00	0.00	0.00	0.00
EU + EEA - Escherichia coli CREC RESP - model 22	0.03	0.03	0.04	0.04	0.05	0.06	147.33	179.21	212.18	0.03	0.03	0.04	5.33	6.44	7.66	1.04E-03	1.25E-03	1.49E-03
EU + EEA - Escherichia coli CREC SSI - model 24	0.03	0.04	0.04	0.03	0.04	0.04	350.63	414.14	477.36	0.07	0.08	0.09	8.55	10.42	12.35	1.66E-03	2.03E-03	2.40E-03
EU + EEA - Escherichia coli CREC UTI - model 23	0.00	0.00	0.00	4.61E-04	5.98E-04	7.66E-04	1,285.93	1,476.96	1,657.35	0.25	0.29	0.32	0.00	0.00	0.00	0.00	0.00	0.00
EU + EEA - Escherichia coli ColREC BSI - model 26	1.95	2.26	2.62	2.06	2.37	2.72	851.97	949.39	1,054.03	0.17	0.18	0.21	476.71	570.53	689.68	0.09	0.11	0.13
EU + EEA - Escherichia coli ColREC OTH - model 30	0.00	0.00	0.00	2.71E-04	3.79E-04	5.33E-04	451.69	552.64	669.78	0.09	0.11	0.13	0.00	0.00	0.00	0.00	0.00	0.00
EU + EEA - Escherichia coli ColREC RESP - model 27	0.06	0.07	0.08	0.09	0.11	0.12	395.23	487.71	586.76	0.08	0.09	0.11	13.98	17.60	21.38	2.72E-03	3.42E-03	4.16E-03
EU + EEA - Escherichia coli ColREC SSI - model 29	0.08	0.09	0.11	0.08	0.09	0.11	967.84	1,138.67	1,328.46	0.19	0.22	0.26	27.39	33.34	40.33	5.33E-03	6.49E-03	7.85E-03
EU + EEA - Escherichia coli ColREC UTI - model 28	0.00	0.00	0.00	1.23E-03	1.63E-03	2.09E-03	3,440.31	4,027.64	4,602.38	0.67	0.78	0.90	0.00	0.00	0.00	0.00	0.00	0.00
EU + EEA - Klebsiella pneumoniae 3GCRKP BSI - model 1	14.09	15.12	16.21	16.36	17.53	18.75	16,574.99	17,739.39	19,006.58	3.22	3.45	3.70	2,735.80	2,956.27	3,205.88	0.53	0.58	0.62
EU + EEA - Klebsiella pneumoniae 3GCRKP OTH - model 5	0.00	0.00	0.00	3.67E-03	4.64E-03	5.81E-03	6,195.02	7,155.08	8,097.20	1.21	1.39	1.58	0.00	0.00	0.00	0.00	0.00	0.00
EU + EEA - Klebsiella pneumoniae 3GCRKP RESP - model 2	2.55	2.85	3.16	3.86	4.32	4.78	13,759.54	15,480.54	17,221.77	2.68	3.01	3.35	491.23	556.63	622.43	0.10	0.11	0.12
EU + EEA - Klebsiella pneumoniae 3GCRKP SSI - model 4	0.52	0.62	0.71	0.53	0.62	0.71	6,166.97	7,129.19	8,083.67	1.20	1.39	1.57	143.14	173.62	202.89	0.03	0.03	0.04
EU + EEA - Klebsiella pneumoniae 3GCRKP UTI - model 3	0.00	0.00	0.00	6.44E-03	8.07E-03	9.79E-03	18,762.08	21,083.92	23,658.43	3.65	4.10	4.60	0.00	0.00	0.00	0.00	0.00	0.00
EU + EEA - Klebsiella pneumoniae CRKP BSI - model 6	8.51	9.92	11.47	9.20	10.67	12.27	5,029.69	5,579.32	6,245.01	0.98	1.09	1.21	1,692.19	1,989.03	2,315.29	0.33	0.39	0.45
EU + EEA - Klebsiella pneumoniae CRKP OTH - model 10	0.00	0.00	0.00	1.39E-03	1.85E-03	2.40E-03	2,175.49	2,724.87	3,150.61	0.42	0.53	0.61	0.00	0.00	0.00	0.00	0.00	0.00
EU + EEA - Klebsiella pneumoniae CRKP RESP - model 7	0.36	0.44	0.52	0.55	0.66	0.78	1,991.68	2,435.94	2,911.14	0.39	0.47	0.57	71.00	87.62	105.41	0.01	0.02	0.02
EU + EEA - Klebsiella pneumoniae CRKP SSI - model 9	0.12	0.15	0.18	0.12	0.15	0.18	1,410.37	1,718.04	2,067.87	0.27	0.33	0.40	31.66	41.43	51.94	6.16E-03	8.06E-03	0.01
EU + EEA - Klebsiella pneumoniae CRKP UTI - model 8	0.00	0.00	0.00	1.07E-03	1.43E-03	1.79E-03	2,866.07	3,488.62	4,103.32	0.56	0.68	0.80	0.00	0.00	0.00	0.00	0.00	0.00
EU + EEA - Klebsiella pneumoniae ColRKP BSI - model 11	6.61	7.84	9.15	6.89	8.19	9.53	2,338.82	2,609.46	2,913.11	0.46	0.51	0.57	1,316.13	1,574.79	1,848.98	0.26	0.31	0.36
EU + EEA - Klebsiella pneumoniae ColRKP OTH - model 15	0.00	0.00	0.00	6.34E-04	8.59E-04	1.14E-03	1,008.28	1,264.71	1,518.48	0.20	0.25	0.30	0.00	0.00	0.00	0.00	0.00	0.00
EU + EEA - Klebsiella pneumoniae ColRKP RESP - model 12	0.16	0.21	0.25	0.25	0.31	0.37	902.44	1,144.59	1,372.14	0.18	0.22	0.27	32.43	41.14	49.39	6.31E-03	8.00E-03	9.61E-03
EU + EEA - Klebsiella pneumoniae ColRKP SSI - model 14	0.05	0.07	0.08	0.05	0.07	0.09	649.02	802.75	976.06	0.13	0.16	0.19	13.90	18.63	23.86	2.70E-03	3.63E-03	4.64E-03
EU + EEA - Klebsiella pneumoniae ColRKP UTI - model 13	0.00	0.00	0.00	4.93E-04	6.57E-04	8.65E-04	1,324.57	1,628.76	1,935.19	0.26	0.32	0.38	0.00	0.00	0.00	0.00	0.00	0.00
EU + EEA - Pseudomonas aeruginosa CRPA BSI - model 51	8.86	10.75	12.88	10.14	12.01	14.22	7,809.76	8,568.92	9,596.63	1.52	1.67	1.87	1,588.89	1,946.31	2,395.54	0.31	0.38	0.47
EU + EEA - Pseudomonas aeruginosa CRPA OTH - model 55	0.00	0.00	0.00	2.42E-03	3.14E-03	4.01E-03	3,593.33	4,439.65	5,262.68	0.70	0.86	1.02	0.00	0.00	0.00	0.00	0.00	0.00
EU + EEA - Pseudomonas aeruginosa CRPA RESP - model 52	9.55	11.43	13.64	12.42	14.68	17.13	27,589.38	31,781.98	36,069.10	5.37	6.18	7.02	1,712.04	2,081.77	2,534.71	0.33	0.41	0.49
EU + EEA - Pseudomonas aeruginosa CRPA SSI - model 54	0.41	0.50	0.61	0.41	0.51	0.61	4,923.99	5,957.66	7,003.61	0.96	1.16	1.36	96.86	127.22	157.12	0.02	0.02	0.03
EU + EEA - Pseudomonas aeruginosa CRPA UTI - model 53	0.00	0.00	0.00	3.53E-03	4.42E-03	5.57E-03	9,293.41	11,143.91	13,051.68	1.81	2.17	2.54	0.00	0.00	0.00	0.00	0.00	0.00
EU + EEA - Pseudomonas aeruginosa ColRPA BSI - model 56	0.18	0.23	0.29	0.20	0.26	0.32	151.40	172.99	196.20	0.03	0.03	0.04	30.45	39.29	50.31	5.92E-03	7.64E-03	9.79E-03
EU + EEA - Pseudomonas aeruginosa ColRPA OTH - model 60	0.00	0.00	0.00	4.74E-05	6.49E-05	8.81E-05	70.80	89.48	108.94	0.01	0.02	0.02	0.00	0.00	0.00	0.00	0.00	0.00
EU + EEA - Pseudomonas aeruginosa ColRPA RESP - model 57	0.19	0.25	0.31	0.25	0.32	0.39	545.94	649.51	752.39	0.11	0.13	0.15	33.15	42.57	53.87	6.45E-03	8.28E-03	0.01
EU + EEA - Pseudomonas aeruginosa ColRPA SSI - model 59	8.47E-03	0.01	0.01	8.54E-03	0.01	0.01	96.59	121.57	146.60	0.02	0.02	0.03	1.92	2.61	3.32	3.74E-04	5.08E-04	6.46E-04
EU + EEA - Pseudomonas aeruginosa ColRPA UTI - model 58	0.00	0.00	0.00	6.61E-05	9.03E-05	1.19E-04	178.70	228.34	271.59	0.03	0.04	0.05	0.00	0.00	0.00	0.00	0.00	0.00
EU + EEA - Pseudomonas aeruginosa MDRPA BSI - model 46	0.96	1.20	1.47	1.11	1.35	1.62	1,112.08	1,248.68	1,387.51	0.22	0.24	0.27	210.45	263.24	320.68	0.04	0.05	0.06
EU + EEA - Pseudomonas aeruginosa MDRPA OTH - model 50	0.00	0.00	0.00	3.56E-04	4.69E-04	6.07E-04	515.66	646.55	775.73	0.10	0.13	0.15	0.00	0.00	0.00	0.00	0.00	0.00
EU + EEA - Pseudomonas aeruginosa MDRPA RESP - model 47	1.06	1.32	1.63	1.43	1.72	2.05	4,041.45	4,625.92	5,300.98	0.79	0.90	1.03	229.81	288.20	356.95	0.04	0.06	0.07
EU + EEA - Pseudomonas aeruginosa MDRPA SSI - model 49	0.05	0.07	0.08	0.06	0.07	0.08	709.81	873.34	1,041.31	0.14	0.17	0.20	16.04	20.86	25.57	3.12E-03	4.06E-03	4.98E-03
EU + EEA - Pseudomonas aeruginosa MDRPA UTI - model 48	0.00	0.00	0.00	4.91E-04	6.47E-04	8.37E-04	1,356.67	1,633.79	1,919.87	0.26	0.32	0.37	0.00	0.00	0.00	0.00	0.00	0.00
EU + EEA - Staphylococcus aureus MRSA BSI - model 61	16.81	18.15	19.55	19.30	20.86	22.41	23,169.52	25,144.05	27,243.18	4.51	4.89	5.30	4,119.85	4,520.43	4,957.73	0.80	0.88	0.96
EU + EEA - Staphylococcus aureus MRSA OTH - model 65	0.00	0.00	0.00	0.02	0.02	0.03	33,149.06	37,431.19	41,837.57	6.45	7.28	8.14	0.00	0.00	0.00	0.00	0.00	0.00
EU + EEA - Staphylococcus aureus MRSA RESP - model 62	4.97	5.53	6.17	7.53	8.40	9.33	33,803.92	38,223.85	42,991.15	6.58	7.44	8.36	1,205.20	1,373.00	1,578.92	0.23	0.27	0.31
EU + EEA - Staphylococcus aureus MRSA SSI - model 64	2.92	3.34	3.78	2.93	3.36	3.80	36,485.09	41,583.67	46,717.57	7.10	8.09	9.09	982.57	1,155.98	1,326.69	0.19	0.22	0.26
EU + EEA - Staphylococcus aureus MRSA UTI - model 63	0.00	0.00	0.00	1.73E-03	2.31E-03	3.00E-03	5,149.23	6,344.64	7,571.87	1.00	1.23	1.47	0.00	0.00	0.00	0.00	0.00	0.00
EU + EEA - Streptococcus pneumoniae PMRSP BSI - model 76	0.22	0.24	0.26	0.25	0.27	0.30	179.96	193.60	207.34	0.04	0.04	0.04	32.31	34.85	37.55	6.29E-03	6.78E-03	7.31E-03
EU + EEA - Streptococcus pneumoniae PMRSP OTH - model 80	0.00	0.00	0.00	5.13E-05	6.38E-05	7.75E-05	156.38	168.01	181.63	0.03	0.03	0.04	0.00	0.00	0.00	0.00	0.00	0.00
EU + EEA - Streptococcus pneumoniae PMRSP RESP - model 77	0.36	0.41	0.49	0.54	0.62	0.74	1,606.44	1,819.90	2,059.99	0.31	0.35	0.40	57.19	65.60	75.10	0.01	0.01	0.01
EU + EEA - Streptococcus pneumoniae PMRSP SSI - model 79	0.00	0.00	0.00	0.00	0.00	0.00	0.00	0.00	0.00	0.00	0.00	0.00	0.00	0.00	0.00	0.00	0.00	0.00
EU + EEA - Streptococcus pneumoniae PMRSP UTI - model 78	0.00	0.00	0.00	0.00	0.00	0.00	0.00	0.00	0.00	0.00	0.00	0.00	0.00	0.00	0.00	0.00	0.00	0.00
EU + EEA - Streptococcus pneumoniae PRSP BSI - model 71	0.63	0.66	0.69	0.72	0.76	0.79	522.74	533.68	544.14	0.10	0.10	0.11	92.52	96.08	99.42	0.02	0.02	0.02
EU + EEA - Streptococcus pneumoniae PRSP OTH - model 75	0.00	0.00	0.00	6.23E-05	7.37E-05	8.75E-05	181.24	193.16	207.92	0.04	0.04	0.04	0.00	0.00	0.00	0.00	0.00	0.00
EU + EEA - Streptococcus pneumoniae PRSP RESP - model 72	0.46	0.52	0.60	0.69	0.78	0.90	1,876.72	2,109.35	2,366.58	0.37	0.41	0.46	67.31	75.76	85.50	0.01	0.01	0.02
EU + EEA - Streptococcus pneumoniae PRSP SSI - model 74	0.00	0.00	0.00	0.00	0.00	0.00	0.00	0.00	0.00	0.00	0.00	0.00	0.00	0.00	0.00	0.00	0.00	0.00
EU + EEA - Streptococcus pneumoniae PRSP UTI - model 73	0.00	0.00	0.00	0.00	0.00	0.00	0.00	0.00	0.00	0.00	0.00	0.00	0.00	0.00	0.00	0.00	0.00	0.00
**Total (Median & 95% UIs)**	126.51	144.84	165.11	149.58	170.14	192.43	583,148.39	671,689.34	763,965.77	113.45	130.68	148.63	28,479.94	33,110.20	38,429.98	5.54	6.44	7.48
**Total (average)**	**145.05**			**170.36**			**672,440.02**			**130.82**			**33,227.14**			**6.46**		

Infection	YLD			YLL			DALY			DALY per Case			YLD per 100,000			YLL per 100,000			DALY per 100,000			Cases per year			Incidence per 100,000			Deaths per year			Mortality per 100,000		
Colistin resistant *Acinetobacter spp.*	0.71	1.12	1.66	2.43	6.27	10.58	3.27	7.40	12.07	3.92	6.92	9.71	0.01	0.01	0.02	0.03	0.07	0.12	0.04	0.09	0.14	1.97	3.32	4.67	0.02	0.04	0.05	0.12	0.30	0.50	0.00	0.00	0.01
Carbapenem resistant *Acinetobacter spp.* (excluding those resistant to colistin)	15.10	18.36	21.99	68.77	98.14	135.79	85.60	116.46	156.19	12.88	17.03	21.72	0.18	0.21	0.26	0.80	1.14	1.58	1.00	1.36	1.82	16.53	19.96	23.54	0.19	0.23	0.27	1.25	1.71	2.25	0.01	0.02	0.03
Aminoglycosides and fluoroquinolones resistant *Acinetobacter* spp. (excluding those resistant to colistin and/or carbapenem)	0.00	0.00	0.00	0.00	0.00	0.00	0.00	0.00	0.00	0.00	0.00	0.00	0.00	0.00	0.00	0.00	0.00	0.00	0.00	0.00	0.00	0.00	0.00	0.00	0.00	0.00	0.00	0.00	0.00	0.00	0.00	0.00	0.00
Vancomycin resistant Enterococci (*E. faecalis* and *E. faecium*)	10.06	13.86	18.98	98.49	132.28	176.23	108.49	146.22	195.17	6.01	6.61	7.40	0.12	0.16	0.22	1.15	1.54	2.05	1.26	1.70	2.28	60.22	85.58	112.38	0.70	1.00	1.31	4.17	5.67	7.38	0.05	0.07	0.09
Colistin resistant *Escherichia coli*	0.15	0.25	0.39	1.94	3.80	6.32	2.11	4.05	6.64	2.24	3.68	5.02	0.00	0.00	0.00	0.02	0.04	0.07	0.02	0.05	0.08	5.15	8.76	12.32	0.06	0.10	0.14	0.39	0.76	1.26	0.00	0.01	0.01
Carbapenem resistant *Escherichia coli* (excluding those resistant to colistin)	0.54	0.90	1.39	5.23	10.06	16.49	5.87	10.98	17.76	6.67	9.99	13.31	0.01	0.01	0.02	0.06	0.12	0.19	0.07	0.13	0.21	5.11	8.65	12.30	0.06	0.10	0.14	0.25	0.48	0.78	0.00	0.01	0.01
Third-generation cephalosporin resistant *Escherichia coli* (excluding those resistant to colistin and/or carbapenem)	324.72	359.21	401.19	1,912.94	2,148.18	2,377.67	2,249.77	2,507.76	2,766.55	4.68	5.08	5.51	3.79	4.19	4.68	22.31	25.05	27.72	26.23	29.24	32.26	3,491.07	3,928.69	4,388.12	40.71	45.81	51.17	106.22	121.05	136.75	1.24	1.41	1.59
Colistin resistant *Klebsiella pneumoniae*	1.75	2.41	3.19	28.91	42.85	59.83	31.10	45.25	62.92	10.21	14.14	18.05	0.02	0.03	0.04	0.34	0.50	0.70	0.36	0.53	0.73	6.59	9.79	13.07	0.08	0.11	0.15	1.49	2.19	3.05	0.02	0.03	0.04
Carbapenem resistant *Klebsiella pneumoniae* (excluding those resistant to colistin)	4.88	6.53	8.60	48.50	69.73	93.21	54.39	76.17	101.29	7.66	10.37	12.82	0.06	0.08	0.10	0.57	0.81	1.09	0.63	0.89	1.18	16.77	22.82	29.00	0.20	0.27	0.34	2.28	3.08	3.98	0.03	0.04	0.05
Third-generation cephalosporin resistant *Klebsiella pneumoniae* (excluding those resistant to colistin and/or carbapenem)	86.79	95.99	106.24	425.99	460.42	494.72	514.85	556.26	598.94	6.26	6.59	6.93	1.01	1.12	1.24	4.97	5.37	5.77	6.00	6.49	6.98	312.24	347.04	382.12	3.64	4.05	4.46	17.32	18.87	20.46	0.20	0.22	0.24
Colistin resistant *Pseudomonas aeruginosa*	1.30	2.38	3.72	4.13	11.87	24.20	5.82	14.33	27.38	3.96	7.92	11.90	0.02	0.03	0.04	0.05	0.14	0.28	0.07	0.17	0.32	4.20	8.57	13.01	0.05	0.10	0.15	0.20	0.56	1.15	0.00	0.01	0.01
Carbapenem resistant *Pseudomonas aeruginosa* (excluding those resistant to colistin)	232.59	265.57	298.78	1,077.24	1,344.38	1,630.65	1,322.89	1,610.21	1,916.59	8.45	10.05	11.68	2.71	3.10	3.48	12.56	15.68	19.01	15.42	18.78	22.35	609.35	709.85	805.80	7.11	8.28	9.40	38.42	48.22	59.15	0.45	0.56	0.69
Pseudomonas aeruginosa resistant to three or more antimicrobial groups (excluding those resistant to colistin and/or carbapenem)	3.89	5.50	7.21	14.45	25.91	40.06	18.88	31.40	46.24	3.59	5.61	7.56	0.05	0.06	0.08	0.17	0.30	0.47	0.22	0.37	0.54	17.85	25.58	33.65	0.21	0.30	0.39	0.92	1.66	2.54	0.01	0.02	0.03
Methicillin resistant *Staphylococcus aureus*	208.98	230.38	253.15	1.049.75	1.135.65	1.215.94	1.262.98	1.365.34	1.463.25	4.74	4.97	5.19	2.44	2.69	2.95	12.24	13.24	14.18	14.73	15.92	17.06	1.273.64	1.397.96	1.525.10	14.85	16.30	17.78	62.18	67.63	72.91	0.73	0.79	0.85
Penicillin and macrolides resistant *Streptococcus pneumoniae* (excluding those only resistant to penicillin)	6.71	8.32	10.58	23.49	27.15	32.11	30.67	35.53	42.18	4.64	5.07	5.50	0.08	0.10	0.12	0.27	0.32	0.37	0.36	0.41	0.49	19.46	22.89	27.26	0.23	0.27	0.32	1.22	1.40	1.63	0.01	0.02	0.02
Penicillin tesistant *Streptococcus pneumoniae* (excluding those resistant to macrolides)	20.81	25.41	31.84	73.69	83.73	97.74	95.46	109.16	128.05	9.56	10.35	11.26	0.24	0.30	0.37	0.86	0.98	1.14	1.11	1.27	1.49	30.33	34.41	40.15	0.35	0.40	0.47	1.89	2.11	2.36	0.02	0.02	0.03
Total	918.98	1,036.20	1,168.92	4,835.97	5,600.42	6,411.54	5,792.14	6,636.51	7,541.23				10.72	12.08	13.63	56.39	65.30	74.76	67.54	77.38	87.93	5,870.48	6,633.87	7,422.48	68.45	77.35	86.55	238.30	275.70	316.17	2.78	3.21	3.69

Model	YLD			YLL			DALY			DALY per Case			YLD per 100,000			YLL per 100,000			DALY per 100,000			Cases per year			Incidence per 100,000			Deaths per year			Mortality per 100,000		
Austria - Acinetobacter spp. CoIRACI BSI - model 41	4.36E-01	6.38E-01	9.20E-01	1.78E+00	5.00E+00	8.67E+00	2.37E+00	5.64E+00	9.49E+00	2.17E+00	5.00E+00	7.62E+00	5.08E-03	7.44E-03	1.07E-02	2.07E-02	5.83E-02	1.01E-01	2.76E-02	6.57E-02	1.11E-01	9.04E-01	1.14E+00	1.44E+00	1.05E-02	1.33E-02	1.67E-02	8.43E-02	2.37E-01	4.12E-01	9.83E-04	2.76E-03	4.81E-03
Austria - Acinetobacter spp. CoIRACI OTH - model 45	1.81E-05	1.29E-04	4.28E-04	0.00	0.00	0.00	1.81E-05	1.29E-04	4.28E-04	1.61E-03	3.81E-03	6.48E-03	2.12E-07	1.50E-06	4.99E-06	0.00	0.00	0.00	2.12E-07	1.50E-06	4.99E-06	6.71E-03	0.04	0.09	7.82E-05	4.38E-04	1.04E-03	0.00	0.00	0.00	0.00	0.00	0.00
Austria - Acinetobacter spp. CoIRACI RESP - model 42	0.27	0.48	0.73	0.52	0.95	1.41	0.77	1.43	2.08	0.99	1.15	1.32	3.19E-03	5.64E-03	8.56E-03	6.10E-03	0.01	0.02	8.93E-03	0.02	0.02	0.70	1.25	1.72	8.15E-03	0.01	0.02	0.02	0.04	0.07	2.89E-04	5.23E-04	7.77E-04
Austria - Acinetobacter spp. CoIRACI SSI - model 44	1.45E-04	7.57E-04	2.22E-03	0.13	0.32	0.50	0.13	0.33	0.50	0.76	0.76	0.76	1.69E-06	8.82E-06	2.59E-05	1.53E-03	3.78E-03	5.88E-03	1.53E-03	3.79E-03	5.86E-03	0.17	0.43	0.66	2.02E-03	4.98E-03	7.75E-03	6.22E-03	0.02	0.02	7.26E-05	1.79E-04	2.79E-04
Austria - Acinetobacter spp. CoIRACI UTI - model 43	2.53E-04	8.54E-04	2.24E-03	0.00	0.00	0.00	2.53E-04	8.54E-04	2.24E-03	8.07E-04	2.03E-03	3.55E-03	2.95E-06	9.96E-06	2.62E-05	0.00	0.00	0.00	2.95E-06	9.96E-06	2.62E-05	0.19	0.47	0.76	2.18E-03	5.50E-03	8.90E-03	0.00	0.00	0.00	0.00	0.00	0.00
Austria - Acinetobacter spp. CRACI BSI - model 36	8.94	10.45	12.27	55.36	81.03	114.62	65.96	91.39	125.83	9.49	13.27	17.57	0.10	0.12	0.14	0.65	0.94	1.34	0.77	1.07	1.47	6.23	6.91	7.64	0.07	0.08	0.09	1.01	1.41	1.88	0.01	0.02	0.02
Austria - Acinetobacter spp. CRACI OTH - model 40	4.78E-04	8.93E-04	1.46E-03	0.00	0.00	0.00	4.78E-04	8.93E-04	1.46E-03	2.60E-03	3.71E-03	4.99E-03	5.58E-06	1.04E-05	1.71E-05	0.00	0.00	0.00	5.58E-06	1.04E-05	1.71E-05	0.14	0.24	0.35	1.67E-03	2.81E-03	4.08E-03	0.00	0.00	0.00	0.00	0.00	0.00
Austria - Acinetobacter spp. CRACI RESP - model 37	6.15	7.90	9.70	12.27	15.58	19.20	18.49	23.53	28.38	2.81	3.14	3.48	0.07	0.09	0.11	0.14	0.18	0.22	0.22	0.27	0.33	6.17	7.51	8.82	0.07	0.09	0.10	0.22	0.27	0.32	2.54E-03	3.16E-03	3.79E-03
Austria - Acinetobacter spp. CRACI SSI - model 39	3.09E-03	5.26E-03	8.24E-03	1.14	1.53	1.97	1.14	1.53	1.97	0.57	0.62	0.66	3.60E-05	6.13E-05	9.60E-05	0.01	0.02	0.02	0.01	0.02	0.02	1.86	2.49	3.19	0.02	0.03	0.04	0.02	0.03	0.04	2.72E-04	3.92E-04	5.21E-04
Austria - Acinetobacter spp. CRACI UTI - model 38	3.71E-03	5.69E-03	8.48E-03	0.00	0.00	0.00	3.71E-03	5.69E-03	8.48E-03	1.45E-03	2.05E-03	2.69E-03	4.33E-05	6.64E-05	9.88E-05	0.00	0.00	0.00	4.33E-05	6.64E-05	9.88E-05	2.13	2.81	3.54	0.02	0.03	0.04	0.00	0.00	0.00	0.00	0.00	0.00
Austria - Acinetobacter spp. MDRACI BSI - model 31	0.00	0.00	0.00	0.00	0.00	0.00	0.00	0.00	0.00	NaN	NaN	NaN	0.00	0.00	0.00	0.00	0.00	0.00	0.00	0.00	0.00	0.00	0.00	0.00	0.00	0.00	0.00	0.00	0.00	0.00	0.00	0.00	0.00
Austria - Acinetobacter spp. MDRACI OTH - model 35	0.00	0.00	0.00	0.00	0.00	0.00	0.00	0.00	0.00	NaN	NaN	NaN	0.00	0.00	0.00	0.00	0.00	0.00	0.00	0.00	0.00	0.00	0.00	0.00	0.00	0.00	0.00	0.00	0.00	0.00	0.00	0.00	0.00
Austria - Acinetobacter spp. MDRACI RESP - model 32	0.00	0.00	0.00	0.00	0.00	0.00	0.00	0.00	0.00	NaN	NaN	NaN	0.00	0.00	0.00	0.00	0.00	0.00	0.00	0.00	0.00	0.00	0.00	0.00	0.00	0.00	0.00	0.00	0.00	0.00	0.00	0.00	0.00
Austria - Acinetobacter spp. MDRACI SSI - model 34	0.00	0.00	0.00	0.00	0.00	0.00	0.00	0.00	0.00	NaN	NaN	NaN	0.00	0.00	0.00	0.00	0.00	0.00	0.00	0.00	0.00	0.00	0.00	0.00	0.00	0.00	0.00	0.00	0.00	0.00	0.00	0.00	0.00
Austria - Acinetobacter spp. MDRACI UTI - model 33	0.00	0.00	0.00	0.00	0.00	0.00	0.00	0.00	0.00	NaN	NaN	NaN	0.00	0.00	0.00	0.00	0.00	0.00	0.00	0.00	0.00	0.00	0.00	0.00	0.00	0.00	0.00	0.00	0.00	0.00	0.00	0.00	0.00
Austria - Enterococcus faecalis and E. faecium VRE BSI - model 66	9.99	13.74	18.79	88.34	117.34	156.30	98.27	131.10	175.04	5.56	6.11	6.83	0.12	0.16	0.22	1.03	1.37	1.82	1.15	1.53	2.04	16.10	21.52	27.84	0.19	0.25	0.32	3.68	4.92	6.37	0.04	0.06	0.07
Austria - Enterococcus faecalis and E. faecium VRE OTH - model 70	0.02	0.04	0.06	0.00	0.00	0.00	0.02	0.04	0.06	1.13E-03	1.64E-03	2.24E-03	2.66E-04	4.39E-04	6.83E-04	0.00	0.00	0.00	2.66E-04	4.39E-04	6.83E-04	16.34	23.13	30.44	0.19	0.27	0.35	0.00	0.00	0.00	0.00	0.00	0.00
Austria - Enterococcus faecalis and E. faecium VRE RESP - model 67	0.00	0.00	0.00	0.00	0.00	0.00	0.00	0.00	0.00	NaN	NaN	NaN	0.00	0.00	0.00	0.00	0.00	0.00	0.00	0.00	0.00	0.00	0.00	0.00	0.00	0.00	0.00	0.00	0.00	0.00	0.00	0.00	0.00
Austria - Enterococcus faecalis and E. faecium VRE SSI - model 69	0.04	0.06	0.10	10.15	14.94	19.93	10.18	15.06	20.05	0.45	0.51	0.56	4.09E-04	7.23E-04	1.22E-03	0.12	0.17	0.23	0.12	0.18	0.23	20.50	29.93	39.29	0.24	0.35	0.46	0.49	0.75	1.02	5.71E-03	8.77E-03	0.01
Austria - Enterococcus faecalis and E. faecium VRE UTI - model 68	0.01	0.02	0.03	0.00	0.00	0.00	0.01	0.02	0.03	1.30E-03	1.85E-03	2.54E-03	1.43E-04	2.36E-04	3.67E-04	0.00	0.00	0.00	1.43E-04	2.36E-04	3.67E-04	7.29	11.00	14.81	0.08	0.13	0.17	0.00	0.00	0.00	0.00	0.00	0.00
Austria - Escherichia coli 3GCREC BSI - model 16	249.81	270.32	296.49	1,570.39	1,747.64	1,915.50	1,831.16	2,018.12	2,201.85	3.37	3.65	3.95	2.91	3.15	3.46	18.31	20.38	22.33	21.35	23.53	25.67	510.59	552.00	593.32	5.95	6.44	6.92	84.52	95.31	106.78	0.99	1.11	1.25
Austria - Escherichia coli 3GCREC OTH - model 20	0.46	0.61	0.80	0.00	0.00	0.00	0.46	0.61	0.80	2.67E-03	3.33E-03	4.08E-03	5.34E-03	7.10E-03	9.32E-03	0.00	0.00	0.00	5.34E-03	7.10E-03	9.32E-03	146.82	182.73	217.04	1.71	2.13	2.53	0.00	0.00	0.00	0.00	0.00	0.00
Austria - Escherichia coli 3GCREC RESP - model 17	69.95	82.45	96.52	133.52	159.32	186.28	204.56	241.59	281.29	0.92	1.00	1.10	0.82	0.96	1.13	1.56	1.86	2.17	2.39	2.82	3.28	199.92	241.94	285.56	2.33	2.82	3.33	7.14	8.69	10.37	0.08	0.10	0.12
Austria - Escherichia coli 3GCREC SSI - model 19	0.87	1.23	1.66	209.04	241.21	275.89	209.96	242.84	276.89	0.39	0.42	0.44	0.01	0.01	0.02	2.44	2.81	3.22	2.45	2.83	3.23	508.32	583.54	655.96	5.93	6.80	7.65	14.56	17.04	19.60	0.17	0.20	0.23
Austria - Escherichia coli 3GCREC UTI - model 18	3.63	4.59	5.72	0.00	0.00	0.00	3.63	4.59	5.72	1.58E-03	1.95E-03	2.34E-03	0.04	0.05	0.07	0.00	0.00	0.00	0.04	0.05	0.07	2,125.42	2,368.48	2,636.24	24.78	27.62	30.74	0.00	0.00	0.00	0.00	0.00	0.00
Austria - Escherichia coli CoIREC BSI - model 26	0.12	0.18	0.26	1.76	3.45	5.78	1.90	3.62	5.98	1.82	3.21	4.50	1.42E-03	2.07E-03	3.02E-03	0.02	0.04	0.07	0.02	0.04	0.07	0.89	1.15	1.52	0.01	0.01	0.02	0.35	0.69	1.15	4.13E-03	8.08E-03	0.01
Austria - Escherichia coli CoIREC OTH - model 30	6.34E-04	2.12E-03	5.45E-03	0.00	0.00	0.00	6.34E-04	2.12E-03	5.45E-03	1.22E-03	3.31E-03	7.13E-03	7.39E-06	2.47E-05	6.35E-05	0.00	0.00	0.00	7.39E-06	2.47E-05	6.35E-05	0.32	0.68	1.03	3.76E-03	7.91E-03	0.01	0.00	0.00	0.00	0.00	0.00	0.00
Austria - Escherichia coli CoIREC RESP - model 27	0.02	0.06	0.10	0.04	0.11	0.17	0.07	0.17	0.26	0.24	0.28	0.32	2.88E-04	6.89E-04	1.13E-03	4.85E-04	1.23E-03	2.00E-03	7.94E-04	1.93E-03	3.08E-03	0.25	0.58	0.92	2.89E-03	6.81E-03	0.01	8.74E-03	0.02	0.03	1.02E-04	2.46E-04	3.99E-04
Austria - Escherichia coli CoIREC SSI - model 29	6.49E-04	2.60E-03	7.03E-03	0.13	0.25	0.37	0.13	0.25	0.37	0.18	0.18	0.19	7.57E-06	3.04E-05	8.20E-05	1.57E-03	2.89E-03	4.26E-03	1.57E-03	2.93E-03	4.30E-03	0.74	1.37	2.01	8.67E-03	0.02	0.02	0.03	0.05	0.07	3.12E-04	5.75E-04	8.44E-04
Austria - Escherichia coli CoIREC UTI - model 28	3.35E-03	9.42E-03	0.02	0.00	0.00	0.00	3.35E-03	9.42E-03	0.02	7.63E-04	1.98E-03	4.19E-03	3.90E-05	1.10E-04	2.68E-04	0.00	0.00	0.00	3.90E-05	1.10E-04	2.68E-04	2.95	4.98	6.84	0.03	0.06	0.08	0.00	0.00	0.00	0.00	0.00	0.00
Austria - Escherichia coli CREC BSI - model 21	0.45	0.66	0.96	4.48	8.58	14.24	5.02	9.25	15.11	4.93	8.07	11.22	5.24E-03	7.70E-03	0.01	0.05	0.10	0.17	0.06	0.11	0.18	0.89	1.15	1.53	0.01	0.01	0.02	0.21	0.41	0.68	2.48E-03	4.76E-03	7.89E-03
Austria - Escherichia coli CREC OTH - model 25	6.24E-04	2.10E-03	5.42E-03	0.00	0.00	0.00	6.24E-04	2.10E-03	5.42E-03	1.18E-03	3.19E-03	7.07E-03	7.28E-06	2.45E-05	6.32E-05	0.00	0.00	0.00	7.28E-06	2.45E-05	6.32E-05	0.30	0.66	1.04	3.51E-03	7.73E-03	0.01	0.00	0.00	0.00	0.00	0.00	0.00
Austria - Escherichia coli CREC RESP - model 22	0.09	0.23	0.39	0.17	0.45	0.75	0.26	0.68	1.11	0.98	1.15	1.31	1.05E-03	2.65E-03	4.51E-03	1.94E-03	5.21E-03	8.70E-03	3.07E-03	7.92E-03	0.01	0.22	0.59	0.94	2.62E-03	6.86E-03	0.01	7.91E-03	0.02	0.04	9.22E-05	2.47E-04	4.10E-04
Austria - Escherichia coli CREC SSI - model 24	6.02E-04	2.52E-03	6.99E-03	0.58	1.03	1.51	0.59	1.04	1.51	0.76	0.76	0.76	7.02E-06	2.94E-05	8.15E-05	6.79E-03	0.01	0.02	6.86E-03	0.01	0.02	0.77	1.36	1.99	8.93E-03	0.02	0.02	0.03	0.05	0.07	3.22E-04	5.72E-04	8.35E-04
Austria - Escherichia coli CREC UTI - model 23	3.25E-03	9.25E-03	0.02	0.00	0.00	0.00	3.25E-03	9.25E-03	0.02	7.47E-04	1.99E-03	3.93E-03	3.79E-05	1.08E-04	2.65E-04	0.00	0.00	0.00	3.79E-05	1.08E-04	2.65E-04	2.93	4.88	6.79	0.03	0.06	0.08	0.00	0.00	0.00	0.00	0.00	0.00
Austria - Klebsiella pneumoniae 3GCRKP BSI - model 1	54.91	59.72	65.42	350.51	374.81	398.33	406.56	434.38	462.35	4.57	4.79	5.00	0.64	0.70	0.76	4.09	4.37	4.64	4.74	5.06	5.39	86.05	90.74	95.47	1.00	1.06	1.11	14.09	15.14	16.23	0.16	0.18	0.19
Austria - Klebsiella pneumoniae 3GCRKP RESP - model 2	31.57	35.86	40.30	61.89	69.61	78.00	94.39	105.48	117.69	1.26	1.35	1.44	0.37	0.42	0.47	0.72	0.81	0.91	1.10	1.23	1.37	69.26	78.28	86.69	0.81	0.91	1.01	2.47	2.82	3.18	0.03	0.03	0.04
Austria - Klebsiella pneumoniae 3GCRKP UTI - model 3	0.16	0.21	0.26	0.00	0.00	0.00	0.16	0.21	0.26	1.59E-03	1.96E-03	2.39E-03	1.91E-03	2.44E-03	3.06E-03	0.00	0.00	0.00	1.91E-03	2.44E-03	3.06E-03	94.73	106.55	118.71	1.10	1.24	1.38	0.00	0.00	0.00	0.00	0.00	0.00
Austria - Klebsiella pneumoniae 3GCRKP SSI - model 4	0.06	0.08	0.10	13.59	16.01	18.40	13.65	16.08	18.48	0.43	0.45	0.48	6.57E-04	8.88E-04	1.18E-03	0.16	0.19	0.21	0.16	0.19	0.22	30.83	35.53	40.37	0.36	0.41	0.47	0.76	0.91	1.06	8.86E-03	0.01	0.01
Austria - Klebsiella pneumoniae 3GCRKP OTH - model 5	0.09	0.12	0.15	0.00	0.00	0.00	0.09	0.12	0.15	2.69E-03	3.33E-03	4.10E-03	1.06E-03	1.39E-03	1.79E-03	0.00	0.00	0.00	1.06E-03	1.39E-03	1.79E-03	31.38	35.94	40.88	0.37	0.42	0.48	0.00	0.00	0.00	0.00	0.00	0.00
Austria - Klebsiella pneumoniae CoIRKP BSI - model 11	1.44	1.86	2.36	27.91	41.07	57.22	29.79	42.90	59.51	8.57	12.35	16.12	0.02	0.02	0.03	0.33	0.48	0.67	0.35	0.50	0.69	2.94	3.48	4.15	0.03	0.04	0.05	1.43	2.09	2.92	0.02	0.02	0.03
Austria - Klebsiella pneumoniae CoIRKP OTH - model 15	2.48E-03	5.46E-03	0.01	0.00	0.00	0.00	2.48E-03	5.46E-03	0.01	1.83E-03	3.43E-03	5.58E-03	2.89E-05	6.36E-05	1.18E-04	0.00	0.00	0.00	2.89E-05	6.36E-05	1.18E-04	0.92	1.66	2.35	0.01	0.02	0.03	0.00	0.00	0.00	0.00	0.00	0.00
Austria - Klebsiella pneumoniae CoIRKP RESP - model 12	0.30	0.55	0.80	0.59	1.05	1.54	0.89	1.61	2.32	0.96	1.07	1.18	3.53E-03	6.37E-03	9.35E-03	6.89E-03	0.01	0.02	0.01	0.02	0.03	0.85	1.49	2.14	9.89E-03	0.02	0.02	0.03	0.05	0.08	3.52E-04	6.23E-04	9.23E-04
Austria - Klebsiella pneumoniae CoIRKP SSI - model 14	9.48E-04	2.18E-03	4.23E-03	0.41	0.73	1.07	0.41	0.73	1.08	0.67	0.71	0.74	1.11E-05	2.54E-05	4.93E-05	4.82E-03	8.51E-03	0.01	4.84E-03	8.53E-03	0.01	0.57	1.03	1.50	6.61E-03	0.01	0.02	0.02	0.04	0.05	2.38E-04	4.34E-04	6.31E-04
Austria - Klebsiella pneumoniae CoIRKP UTI - model 13	1.93E-03	4.35E-03	7.69E-03	0.00	0.00	0.00	1.93E-03	4.35E-03	7.69E-03	1.15E-03	2.07E-03	3.18E-03	2.26E-05	5.07E-05	8.97E-05	0.00	0.00	0.00	2.26E-05	5.07E-05	8.97E-05	1.31	2.13	2.92	0.02	0.02	0.03	0.00	0.00	0.00	0.00	0.00	0.00
Austria - Klebsiella pneumoniae CRKP BSI - model 6	4.01	5.03	6.40	46.33	66.06	87.86	51.41	71.01	93.82	6.34	8.76	10.94	0.05	0.06	0.07	0.54	0.77	1.02	0.60	0.83	1.09	7.03	8.16	9.41	0.08	0.10	0.11	2.17	2.91	3.74	0.03	0.03	0.04
Austria - Klebsiella pneumoniae CRKP OTH - model 10	6.91E-03	0.01	0.02	0.00	0.00	0.00	6.91E-03	0.01	0.02	2.16E-03	3.48E-03	5.18E-03	8.06E-05	1.54E-04	2.58E-04	0.00	0.00	0.00	8.06E-05	1.54E-04	2.58E-04	2.56	3.83	5.11	0.03	0.04	0.06	0.00	0.00	0.00	0.00	0.00	0.00
Austria - Klebsiella pneumoniae CRKP RESP - model 7	0.86	1.47	2.15	1.65	2.82	4.17	2.43	4.29	6.25	1.01	1.25	1.46	0.01	0.02	0.03	0.02	0.03	0.05	0.03	0.05	0.07	2.20	3.47	4.69	0.03	0.04	0.05	0.08	0.12	0.17	9.43E-04	1.45E-03	2.01E-03
Austria - Klebsiella pneumoniae CRKP SSI - model 9	2.47E-03	4.96E-03	8.81E-03	0.52	0.85	1.18	0.53	0.86	1.19	0.30	0.36	0.42	2.88E-05	5.78E-05	1.03E-04	6.12E-03	9.91E-03	0.01	6.15E-03	9.98E-03	0.01	1.50	2.39	3.33	0.02	0.03	0.04	0.03	0.05	0.07	3.65E-04	5.76E-04	7.90E-04
Austria - Klebsiella pneumoniae CRKP UTI - model 8	5.72E-03	0.01	0.02	0.00	0.00	0.00	5.72E-03	0.01	0.02	1.31E-03	2.07E-03	3.02E-03	6.67E-05	1.18E-04	1.92E-04	0.00	0.00	0.00	6.67E-05	1.18E-04	1.92E-04	3.48	4.97	6.46	0.04	0.06	0.08	0.00	0.00	0.00	0.00	0.00	0.00
Austria - Pseudomonas aeruginosa CoIRPA BSI - model 56	0.43	0.67	1.04	1.81	5.51	11.23	2.43	6.21	12.22	2.20	5.37	8.55	5.01E-03	7.80E-03	0.01	0.02	0.06	0.13	0.03	0.07	0.14	0.86	1.18	1.64	0.01	0.01	0.02	0.09	0.26	0.53	1.00E-03	3.05E-03	6.21E-03
Austria - Pseudomonas aeruginosa CoIRPA OTH - model 60	4.64E-04	2.04E-03	5.24E-03	0.00	0.00	0.00	4.64E-04	2.04E-03	5.24E-03	1.54E-03	3.74E-03	6.51E-03	5.41E-06	2.38E-05	6.11E-05	0.00	0.00	0.00	5.41E-06	2.38E-05	6.11E-05	0.16	0.63	1.01	1.84E-03	7.30E-03	0.01	0.00	0.00	0.00	0.00	0.00	0.00
Austria - Pseudomonas aeruginosa CoIRPA RESP - model 57	0.87	1.71	2.66	2.10	5.72	11.91	3.17	7.47	14.08	0.99	1.78	2.58	0.01	0.02	0.03	0.02	0.07	0.14	0.04	0.09	0.16	2.29	4.39	6.52	0.03	0.05	0.08	0.10	0.27	0.57	1.16E-03	3.16E-03	6.59E-03
Austria - Pseudomonas aeruginosa CoIRPA SSI - model 59	3.09E-04	1.55E-03	4.50E-03	0.21	0.64	1.06	0.21	0.64	1.06	0.76	0.76	0.76	3.60E-06	1.80E-05	5.25E-05	2.50E-03	7.47E-03	0.01	2.50E-03	7.49E-03	0.01	0.28	0.84	1.40	3.29E-03	9.83E-03	0.02	0.01	0.03	0.05	1.18E-04	3.54E-04	5.86E-04
Austria - Pseudomonas aeruginosa CoIRPA UTI - model 58	7.65E-04	2.80E-03	7.06E-03	0.00	0.00	0.00	7.65E-04	2.80E-03	7.06E-03	7.95E-04	1.94E-03	3.68E-03	8.92E-06	3.27E-05	8.23E-05	0.00	0.00	0.00	8.92E-06	3.27E-05	8.23E-05	0.61	1.54	2.45	7.10E-03	0.02	0.03	0.00	0.00	0.00	0.00	0.00	0.00
Austria - Pseudomonas aeruginosa CRPA BSI - model 51	67.52	74.89	82.82	516.93	639.79	776.19	587.21	715.93	856.42	5.98	7.21	8.48	0.79	0.87	0.97	6.03	7.46	9.05	6.85	8.35	9.99	91.22	99.05	107.93	1.06	1.15	1.26	18.34	22.81	27.72	0.21	0.27	0.32
Austria - Pseudomonas aeruginosa CRPA OTH - model 55	0.14	0.18	0.23	0.00	0.00	0.00	0.14	0.18	0.23	2.94E-03	3.66E-03	4.34E-03	1.58E-03	2.12E-03	2.74E-03	0.00	0.00	0.00	1.58E-03	2.12E-03	2.74E-03	41.03	50.08	59.00	0.48	0.58	0.69	0.00	0.00	0.00	0.00	0.00	0.00
Austria - Pseudomonas aeruginosa CRPA RESP - model 52	164.63	190.10	215.20	535.73	673.18	816.63	710.66	862.26	1,021.52	2.04	2.37	2.70	1.92	2.22	2.51	6.25	7.85	9.52	8.29	10.05	11.91	314.90	365.86	412.39	3.67	4.27	4.81	18.94	23.93	29.61	0.22	0.28	0.35
Austria - Pseudomonas aeruginosa CRPA SSI - model 54	0.10	0.14	0.20	24.59	31.41	37.82	24.68	31.59	38.08	0.42	0.46	0.50	1.20E-03	1.68E-03	2.30E-03	0.29	0.37	0.44	0.29	0.37	0.44	55.79	67.75	80.13	0.65	0.79	0.93	1.13	1.48	1.82	0.01	0.02	0.02
Austria - Pseudomonas aeruginosa CRPA UTI - model 53	0.20	0.26	0.32	0.00	0.00	0.00	0.20	0.26	0.32	1.67E-03	2.05E-03	2.45E-03	2.35E-03	2.99E-03	3.79E-03	0.00	0.00	0.00	2.35E-03	2.99E-03	3.79E-03	106.41	127.12	146.35	1.24	1.48	1.71	0.00	0.00	0.00	0.00	0.00	0.00
Austria - Pseudomonas aeruginosa MDRPA BSI - model 46	1.17	1.54	1.97	6.33	11.82	18.27	7.77	13.36	20.02	2.20	3.76	5.24	0.01	0.02	0.02	0.07	0.14	0.21	0.09	0.16	0.23	2.94	3.59	4.28	0.03	0.04	0.05	0.40	0.76	1.15	4.67E-03	8.83E-03	0.01
Austria - Pseudomonas aeruginosa MDRPA OTH - model 50	2.98E-03	6.43E-03	0.01	0.00	0.00	0.00	2.98E-03	6.43E-03	0.01	2.17E-03	3.65E-03	5.39E-03	3.48E-05	7.50E-05	1.28E-04	0.00	0.00	0.00	3.48E-05	7.50E-05	1.28E-04	1.03	1.78	2.52	0.01	0.02	0.03	0.00	0.00	0.00	0.00	0.00	0.00
Austria - Pseudomonas aeruginosa MDRPA RESP - model 47	2.71	3.93	5.21	7.33	12.68	19.82	10.31	16.61	24.22	0.89	1.27	1.66	0.03	0.05	0.06	0.09	0.15	0.23	0.12	0.19	0.28	9.57	13.16	17.03	0.11	0.15	0.20	0.47	0.81	1.26	5.48E-03	9.48E-03	0.01
Austria - Pseudomonas aeruginosa MDRPA SSI - model 49	2.25E-03	4.94E-03	9.70E-03	0.79	1.40	1.96	0.80	1.41	1.97	0.49	0.57	0.64	2.62E-05	5.76E-05	1.13E-04	9.24E-03	0.02	0.02	9.28E-03	0.02	0.02	1.46	2.48	3.51	0.02	0.03	0.04	0.05	0.09	0.13	6.13E-04	1.04E-03	1.47E-03
Austria - Pseudomonas aeruginosa MDRPA UTI - model 48	4.49E-03	9.08E-03	0.02	0.00	0.00	0.00	4.49E-03	9.08E-03	0.02	1.16E-03	1.99E-03	2.99E-03	5.24E-05	1.06E-04	1.81E-04	0.00	0.00	0.00	5.24E-05	1.06E-04	1.81E-04	2.85	4.57	6.30	0.03	0.05	0.07	0.00	0.00	0.00	0.00	0.00	0.00
Austria - Staphylococcus aureus MRSA BSI - model 61	103.17	111.91	121.27	702.63	748.06	788.94	807.95	859.70	906.63	3.45	3.60	3.74	1.20	1.30	1.41	8.19	8.72	9.20	9.42	10.02	10.57	226.21	238.86	251.18	2.64	2.79	2.93	40.20	42.97	45.70	0.47	0.50	0.53
Austria - Staphylococcus aureus MRSA OTH - model 65	0.90	1.15	1.43	0.00	0.00	0.00	0.90	1.15	1.43	2.61E-03	3.27E-03	4.01E-03	0.01	0.01	0.02	0.00	0.00	0.00	0.01	0.01	0.02	319.76	350.79	383.58	3.73	4.09	4.47	0.00	0.00	0.00	0.00	0.00	0.00
Austria - Staphylococcus aureus MRSA RESP - model 62	104.22	116.39	129.20	201.40	225.23	247.82	307.57	341.23	374.92	0.90	0.95	1.01	1.22	1.36	1.51	2.35	2.63	2.89	3.59	3.98	4.37	322.57	358.99	394.36	3.76	4.19	4.60	11.41	12.96	14.27	0.13	0.15	0.17
Austria - Staphylococcus aureus MRSA SSI - model 64	0.62	0.83	1.12	145.72	162.37	179.18	146.49	163.16	180.13	0.40	0.42	0.44	7.20E-03	9.65E-03	0.01	1.70	1.89	2.09	1.71	1.90	2.10	356.21	391.20	426.75	4.15	4.56	4.98	10.58	11.71	12.94	0.12	0.14	0.15
Austria - Staphylococcus aureus MRSA UTI - model 63	0.08	0.11	0.14	0.00	0.00	0.00	0.08	0.11	0.14	1.49E-03	1.87E-03	2.32E-03	9.38E-04	1.26E-03	1.65E-03	0.00	0.00	0.00	9.38E-04	1.26E-03	1.65E-03	48.89	58.12	69.23	0.57	0.68	0.81	0.00	0.00	0.00	0.00	0.00	0.00
Austria - Streptococcus pneumoniae PMRSP BSI - model 76	1.98	2.30	2.68	14.28	15.50	16.72	16.54	17.83	19.06	3.72	4.02	4.29	0.02	0.03	0.03	0.17	0.18	0.19	0.19	0.21	0.22	4.44	4.44	4.44	0.05	0.05	0.05	0.74	0.80	0.86	8.62E-03	9.31E-03	0.01
Austria - Streptococcus pneumoniae PMRSP OTH - model 80	1.98E-03	3.09E-03	4.55E-03	0.00	0.00	0.00	1.98E-03	3.09E-03	4.55E-03	1.30E-03	1.93E-03	2.75E-03	2.31E-05	3.61E-05	5.31E-05	0.00	0.00	0.00	2.31E-05	3.61E-05	5.31E-05	1.40	1.59	1.79	0.02	0.02	0.02	0.00	0.00	0.00	0.00	0.00	0.00
Austria - Streptococcus pneumoniae PMRSP RESP - model 77	4.72	6.02	7.90	9.21	11.65	15.40	14.13	17.70	23.12	0.92	1.05	1.20	0.06	0.07	0.09	0.11	0.14	0.18	0.16	0.21	0.27	13.62	16.86	21.03	0.16	0.20	0.25	0.48	0.61	0.77	5.59E-03	7.06E-03	8.99E-03
Austria - Streptococcus pneumoniae PMRSP SSI - model 79	0.00	0.00	0.00	0.00	0.00	0.00	0.00	0.00	0.00	NaN	NaN	NaN	0.00	0.00	0.00	0.00	0.00	0.00	0.00	0.00	0.00	0.00	0.00	0.00	0.00	0.00	0.00	0.00	0.00	0.00	0.00	0.00	0.00
Austria - Streptococcus pneumoniae PMRSP UTI - model 78	0.00	0.00	0.00	0.00	0.00	0.00	0.00	0.00	0.00	NaN	NaN	NaN	0.00	0.00	0.00	0.00	0.00	0.00	0.00	0.00	0.00	0.00	0.00	0.00	0.00	0.00	0.00	0.00	0.00	0.00	0.00	0.00	0.00
Austria - Streptococcus pneumoniae PRSP BSI - model 71	6.07	6.98	8.14	44.38	47.57	51.22	51.24	54.56	58.35	7.69	8.19	8.76	0.07	0.08	0.09	0.52	0.55	0.60	0.60	0.64	0.68	6.66	6.66	6.66	0.08	0.08	0.08	1.13	1.20	1.27	0.01	0.01	0.01
Austria - Streptococcus pneumoniae PRSP OTH - model 75	3.25E-03	4.64E-03	6.36E-03	0.00	0.00	0.00	3.25E-03	4.64E-03	6.36E-03	1.37E-03	1.94E-03	2.57E-03	3.79E-05	5.41E-05	7.42E-05	0.00	0.00	0.00	3.79E-05	5.41E-05	7.42E-05	2.16	2.39	2.62	0.03	0.03	0.03	0.00	0.00	0.00	0.00	0.00	0.00
Austria - Streptococcus pneumoniae PRSP RESP - model 72	14.74	18.42	23.69	29.31	36.16	46.52	44.22	54.59	69.69	1.87	2.15	2.50	0.17	0.21	0.28	0.34	0.42	0.54	0.52	0.64	0.81	21.51	25.36	30.87	0.25	0.30	0.36	0.76	0.91	1.10	8.87E-03	0.01	0.01
Austria - Streptococcus pneumoniae PRSP SSI - model 74	0.00	0.00	0.00	0.00	0.00	0.00	0.00	0.00	0.00	NaN	NaN	NaN	0.00	0.00	0.00	0.00	0.00	0.00	0.00	0.00	0.00	0.00	0.00	0.00	0.00	0.00	0.00	0.00	0.00	0.00	0.00	0.00	0.00
Austria - Streptococcus pneumoniae PRSP UTI - model 73	0.00	0.00	0.00	0.00	0.00	0.00	0.00	0.00	0.00	NaN	NaN	NaN	0.00	0.00	0.00	0.00	0.00	0.00	0.00	0.00	0.00	0.00	0.00	0.00	0.00	0.00	0.00	0.00	0.00	0.00	0.00	0.00	0.00
**Total (Median & 95% UIs)**	918.98	1,036.20	1,168.92	4,835.97	5,600.42	6,411.54	5,792.14	6,636.51	7,541.23		-		10.72	12.08	13.63	56.39	65.30	74.76	67.54	77.38	87.93	5,870.48	6,633.87	7,422.48	68.45	77.35	86.55	238.30	275.70	316.17	2.78	3.21	3.69
**Total (average)**		**1,037.52**			**5,608.35**			**6,645.87**			**-**			**12.10**			**65.39**			**77.49**			**6,635.04**			**77.37**			**276.25**			**3.22**	

Infection	YLD			YLL			DALY			DALY per Case			YLD per 100,000			YLL per 100,000			DALY per 100,000			Cases per year			Incidence per 100,000			Deaths per year			Mortality per 100,000		
Colistin resistant *Acinetobacter spp.*	6.56	10.09	14.98	21.70	55.30	95.93	29.52	65.25	108.57	3.13	5.40	7.70	0.06	0.09	0.13	0.19	0.49	0.85	0.26	0.58	0.97	22.56	37.09	53.19	0.20	0.33	0.47	1.30	3.30	5.73	0.01	0.03	0.05
Carbapenem resistant *Acinetobacter spp.* (excluding those resistant to colistin)	0.00	0.00	0.00	0.00	0.00	0.00	0.00	0.00	0.00	0.00	0.00	0.00	0.00	0.00	0.00	0.00	0.00	0.00	0.00	0.00	0.00	0.00	0.00	0.00	0.00	0.00	0.00	0.00	0.00	0.00	0.00	0.00	0.00
Aminoglycosides and fluoroquinolones resistant *Acinetobacter* spp. (excluding those resistant to colistin and/or carbapenem)	0.00	0.00	0.00	0.00	0.00	0.00	0.00	0.00	0.00	0.00	0.00	0.00	0.00	0.00	0.00	0.00	0.00	0.00	0.00	0.00	0.00	0.00	0.00	0.00	0.00	0.00	0.00	0.00	0.00	0.00	0.00	0.00	0.00
Vancomycin resistant Enterococci (*E. faecalis* and *E. faecium*)	3.94	6.56	11.08	37.60	63.05	97.72	41.94	69.48	107.23	5.98	6.44	6.92	0.04	0.06	0.10	0.33	0.56	0.87	0.37	0.62	0.95	21.59	42.45	65.68	0.19	0.38	0.58	1.64	2.72	4.20	0.01	0.02	0.04
Colistin resistant *Escherichia coli*	28.87	36.33	44.33	471.74	632.37	812.57	502.34	668.46	850.35	13.80	17.44	21.87	0.26	0.32	0.39	4.20	5.63	7.23	4.47	5.95	7.57	247.15	294.84	341.82	2.20	2.62	3.04	20.01	25.83	31.39	0.18	0.23	0.28
Carbapenem resistant *Escherichia coli* (excluding those resistant to colistin)	0.00	0.00	0.00	0.00	0.00	0.00	0.00	0.00	0.00	0.00	0.00	0.00	0.00	0.00	0.00	0.00	0.00	0.00	0.00	0.00	0.00	0.00	0.00	0.00	0.00	0.00	0.00	0.00	0.00	0.00	0.00	0.00	0.00
Third-generation cephalosporin resistant *Escherichia coli* (excluding those resistant to colistin and/or carbapenem)	667.29	738.57	823.18	3,923.37	4,386.78	4,862.52	4,606.63	5,127.84	5,662.92	4.79	5.22	5.68	5.94	6.57	7.33	34.91	39.04	43.27	40.99	45.63	50.39	6,950.79	7,814.04	8,677.86	61.85	69.54	77.22	210.62	240.27	272.39	1.87	2.14	2.42
Colistin resistant *Klebsiella pneumoniae*	12.64	16.59	20.99	201.79	287.25	389.79	215.75	304.34	410.60	8.95	11.81	15.35	0.11	0.15	0.19	1.80	2.56	3.47	1.92	2.71	3.65	56.36	76.88	98.03	0.50	0.68	0.87	12.50	17.10	22.99	0.11	0.15	0.20
Carbapenem resistant *Klebsiella pneumoniae* (excluding those resistant to colistin)	7.28	11.02	15.66	73.67	119.22	181.17	82.31	130.27	194.79	11.19	15.83	21.49	0.06	0.10	0.14	0.66	1.06	1.61	0.73	1.16	1.73	15.72	25.51	35.69	0.14	0.23	0.32	2.18	3.44	5.00	0.02	0.03	0.04
Third-generation cephalosporin resistant *Klebsiella pneumoniae* (excluding those resistant to colistin and/or carbapenem)	213.46	240.78	270.33	1,054.88	1,155.68	1,251.32	1,275.22	1,395.27	1,510.54	4.30	4.58	4.85	1.90	2.14	2.41	9.39	10.28	11.14	11.35	12.42	13.44	1,131.00	1,271.81	1,415.44	10.06	11.32	12.60	63.34	69.57	75.77	0.56	0.62	0.67
Colistin resistant *Pseudomonas aeruginosa*	4.84	8.98	14.09	15.71	45.13	90.40	21.61	54.19	102.68	4.01	8.02	11.91	0.04	0.08	0.13	0.14	0.40	0.80	0.19	0.48	0.91	15.87	31.86	48.66	0.14	0.28	0.43	0.75	2.14	4.29	0.01	0.02	0.04
Carbapenem resistant *Pseudomonas aeruginosa* (excluding those resistant to colistin)	67.10	91.05	113.89	281.54	459.08	681.85	362.04	550.01	785.94	5.35	7.72	10.55	0.60	0.81	1.01	2.51	4.09	6.07	3.22	4.89	6.99	243.67	322.39	399.25	2.17	2.87	3.55	14.37	21.81	31.15	0.13	0.19	0.28
Pseudomonas aeruginosa resistant to three or more antimicrobial groups (excluding those resistant to colistin and/or carbapenem)	25.08	36.65	50.63	85.20	170.08	278.06	115.56	207.89	325.19	4.22	7.14	10.04	0.22	0.33	0.45	0.76	1.51	2.47	1.03	1.85	2.89	86.39	127.62	170.63	0.77	1.14	1.52	4.32	7.99	12.54	0.04	0.07	0.11
Methicillin resistant *Staphylococcus aureus*	525.89	576.88	628.13	2.603.98	2.789.26	2.979.37	3.145.59	3.364.92	3.593.66	5.88	6.17	6.44	4.68	5.13	5.59	23.17	24.82	26.51	27.99	29.94	31.98	2.554.81	2.800.31	3.044.61	22.74	24.92	27.09	122.15	132.94	144.02	1.09	1.18	1.28
Penicillin and macrolides resistant *Streptococcus pneumoniae* (excluding those only resistant to penicillin)	11.68	14.19	17.48	41.15	46.72	53.80	53.72	60.80	70.38	6.28	6.69	7.15	0.10	0.13	0.16	0.37	0.42	0.48	0.48	0.54	0.63	25.74	29.53	34.99	0.23	0.26	0.31	1.61	1.82	2.08	0.01	0.02	0.02
Penicillin tesistant *Streptococcus pneumoniae* (excluding those resistant to macrolides)	13.13	16.56	21.84	47.15	55.07	65.72	61.69	71.64	86.56	12.20	13.21	14.27	0.12	0.15	0.19	0.42	0.49	0.58	0.55	0.64	0.77	15.04	17.58	21.82	0.13	0.16	0.19	0.93	1.09	1.30	0.01	0.01	0.01
Total	1,587.77	1,804.25	2,046.61	8,859.50	10,264.98	11,840.22	10,513.91	12,070.36	13,809.39				14.13	16.06	18.21	78.84	91.35	105.37	93.56	107.41	122.89	11,386.69	12,891.92	14,407.66	101.33	114.72	128.21	455.70	530.04	612.86	4.06	4.72	5.45

Model	YLD			YLL			DALY			DALY per Case			YLD per 100,000			YLL per 100,000			DALY per 100,000			Cases per year			Incidence per 100,000			Deaths per year			Mortality per 100,000		
Belgium - Acinetobacter spp. CoRACI BSI - model 41	4.04E+00	5.72E+00	8.21E+00	1.57E+01	4.40E+01	7.87E+01	2.09E+01	4.96E+01	8.50E+01	1.74E+00	3.87E+00	6.03E+00	3.60E-02	5.09E-02	7.31E-02	1.39E-01	3.92E-01	7.00E-01	1.86E-01	4.41E-01	7.57E-01	1.03E+01	1.27E+01	1.62E+01	9.12E-02	1.13E-01	1.44E-01	9.37E-01	2.63E+00	4.70E+00	8.34E-03	2.34E-02	4.18E-02
Belgium - Acinetobacter spp. CoRACI OTH - model 45	2.53E-04	1.59E-03	4.70E-03	0.00	0.00	0.00	2.53E-04	1.59E-03	4.70E-03	1.55E-03	3.70E-03	6.44E-03	2.26E-06	1.41E-05	4.18E-05	0.00	0.00	0.00	2.26E-06	1.41E-05	4.18E-05	0.08	0.45	0.97	7.33E-04	3.99E-03	8.65E-03	0.00	0.00	0.00	0.00	0.00	0.00
Belgium - Acinetobacter spp. CoRACI RESP - model 42	2.51	4.35	6.72	4.85	8.41	12.65	7.45	12.77	18.90	0.78	0.92	1.05	0.02	0.04	0.06	0.04	0.07	0.11	0.07	0.11	0.17	8.43	13.98	19.98	0.08	0.12	0.18	0.29	0.50	0.76	2.58E-03	4.47E-03	6.74E-03
Belgium - Acinetobacter spp. CoRACI SSI - model 44	1.79E-03	8.87E-03	0.03	1.17	2.84	4.59	1.18	2.85	4.60	0.60	0.61	0.61	1.59E-05	7.89E-05	2.38E-04	0.01	0.03	0.04	0.01	0.03	0.04	1.94	4.72	7.59	0.02	0.04	0.07	0.07	0.17	0.27	6.22E-04	1.51E-03	2.43E-03
Belgium - Acinetobacter spp. CoRACI UTI - model 43	2.62E-03	9.76E-03	0.02	0.00	0.00	0.00	2.62E-03	9.76E-03	0.02	8.17E-04	2.01E-03	3.63E-03	2.33E-05	8.68E-05	2.11E-04	0.00	0.00	0.00	2.33E-05	8.68E-05	2.11E-04	1.85	5.25	8.48	0.02	0.05	0.08	0.00	0.00	0.00	0.00	0.00	0.00
Belgium - Acinetobacter spp. CRACI BSI - model 36	0.00	0.00	0.00	0.00	0.00	0.00	0.00	0.00	0.00	NaN	NaN	NaN	0.00	0.00	0.00	0.00	0.00	0.00	0.00	0.00	0.00	0.00	0.00	0.00	0.00	0.00	0.00	0.00	0.00	0.00	0.00	0.00	0.00
Belgium - Acinetobacter spp. CRACI OTH - model 40	0.00	0.00	0.00	0.00	0.00	0.00	0.00	0.00	0.00	NaN	NaN	NaN	0.00	0.00	0.00	0.00	0.00	0.00	0.00	0.00	0.00	0.00	0.00	0.00	0.00	0.00	0.00	0.00	0.00	0.00	0.00	0.00	0.00
Belgium - Acinetobacter spp. CRACI RESP - model 37	0.00	0.00	0.00	0.00	0.00	0.00	0.00	0.00	0.00	NaN	NaN	NaN	0.00	0.00	0.00	0.00	0.00	0.00	0.00	0.00	0.00	0.00	0.00	0.00	0.00	0.00	0.00	0.00	0.00	0.00	0.00	0.00	0.00
Belgium - Acinetobacter spp. CRACI SSI - model 39	0.00	0.00	0.00	0.00	0.00	0.00	0.00	0.00	0.00	NaN	NaN	NaN	0.00	0.00	0.00	0.00	0.00	0.00	0.00	0.00	0.00	0.00	0.00	0.00	0.00	0.00	0.00	0.00	0.00	0.00	0.00	0.00	0.00
Belgium - Acinetobacter spp. CRACI UTI - model 38	0.00	0.00	0.00	0.00	0.00	0.00	0.00	0.00	0.00	NaN	NaN	NaN	0.00	0.00	0.00	0.00	0.00	0.00	0.00	0.00	0.00	0.00	0.00	0.00	0.00	0.00	0.00	0.00	0.00	0.00	0.00	0.00	0.00
Belgium - Acinetobacter spp. MDRACI BSI - model 31	0.00	0.00	0.00	0.00	0.00	0.00	0.00	0.00	0.00	NaN	NaN	NaN	0.00	0.00	0.00	0.00	0.00	0.00	0.00	0.00	0.00	0.00	0.00	0.00	0.00	0.00	0.00	0.00	0.00	0.00	0.00	0.00	0.00
Belgium - Acinetobacter spp. MDRACI OTH - model 35	0.00	0.00	0.00	0.00	0.00	0.00	0.00	0.00	0.00	NaN	NaN	NaN	0.00	0.00	0.00	0.00	0.00	0.00	0.00	0.00	0.00	0.00	0.00	0.00	0.00	0.00	0.00	0.00	0.00	0.00	0.00	0.00	0.00
Belgium - Acinetobacter spp. MDRACI RESP - model 32	0.00	0.00	0.00	0.00	0.00	0.00	0.00	0.00	0.00	NaN	NaN	NaN	0.00	0.00	0.00	0.00	0.00	0.00	0.00	0.00	0.00	0.00	0.00	0.00	0.00	0.00	0.00	0.00	0.00	0.00	0.00	0.00	0.00
Belgium - Acinetobacter spp. MDRACI SSI - model 34	0.00	0.00	0.00	0.00	0.00	0.00	0.00	0.00	0.00	NaN	NaN	NaN	0.00	0.00	0.00	0.00	0.00	0.00	0.00	0.00	0.00	0.00	0.00	0.00	0.00	0.00	0.00	0.00	0.00	0.00	0.00	0.00	0.00
Belgium - Acinetobacter spp. MDRACI UTI - model 33	0.00	0.00	0.00	0.00	0.00	0.00	0.00	0.00	0.00	NaN	NaN	NaN	0.00	0.00	0.00	0.00	0.00	0.00	0.00	0.00	0.00	0.00	0.00	0.00	0.00	0.00	0.00	0.00	0.00	0.00	0.00	0.00	0.00
Belgium - Enterococcus faecalis and E. faecium VRE BSI - model 66	3.92	6.51	10.96	34.65	55.65	86.03	38.97	62.03	95.47	5.65	5.95	6.25	0.03	0.06	0.10	0.31	0.50	0.77	0.35	0.55	0.85	6.61	10.46	16.05	0.06	0.09	0.14	1.50	2.39	3.66	0.01	0.02	0.03
Belgium - Enterococcus faecalis and E. faecium VRE OTH - model 70	6.75E-03	0.02	0.04	0.00	0.00	0.00	6.75E-03	0.02	0.04	7.64E-04	1.62E-03	2.77E-03	6.01E-05	1.65E-04	3.33E-04	0.00	0.00	0.00	6.01E-05	1.65E-04	3.33E-04	5.41	11.73	17.88	0.05	0.10	0.16	0.00	0.00	0.00	0.00	0.00	0.00
Belgium - Enterococcus faecalis and E. faecium VRE RESP - model 67	0.00	0.00	0.00	0.00	0.00	0.00	0.00	0.00	0.00	NaN	NaN	NaN	0.00	0.00	0.00	0.00	0.00	0.00	0.00	0.00	0.00	0.00	0.00	0.00	0.00	0.00	0.00	0.00	0.00	0.00	0.00	0.00	0.00
Belgium - Enterococcus faecalis and E. faecium VRE SSI - model 69	9.68E-03	0.03	0.07	2.95	7.40	11.69	2.96	7.43	11.69	0.34	0.49	0.66	8.61E-05	2.64E-04	6.08E-04	0.03	0.07	0.10	0.03	0.07	0.10	7.19	14.87	23.18	0.06	0.13	0.21	0.13	0.34	0.54	1.16E-03	3.00E-03	4.79E-03
Belgium - Enterococcus faecalis and E. faecium VRE UTI - model 68	3.75E-03	9.57E-03	0.02	0.00	0.00	0.00	3.75E-03	9.57E-03	0.02	9.30E-04	1.86E-03	3.05E-03	3.33E-05	8.52E-05	1.83E-04	0.00	0.00	0.00	3.33E-05	8.52E-05	1.83E-04	2.38	5.39	8.57	0.02	0.05	0.08	0.00	0.00	0.00	0.00	0.00	0.00
Belgium - Escherichia coli 3GCREC BSI - model 16	511.73	556.25	609.90	3,243.84	3,593.45	3,959.25	3,769.97	4,151.16	4,549.11	3.48	3.78	4.09	4.55	4.95	5.43	28.87	31.98	35.23	33.55	36.94	40.48	1,023.21	1,099.03	1,178.15	9.11	9.78	10.48	168.48	189.76	213.90	1.50	1.69	1.90
Belgium - Escherichia coli 3GCREC OTH - model 20	0.90	1.21	1.58	0.00	0.00	0.00	0.90	1.21	1.58	2.73E-03	3.36E-03	4.05E-03	8.02E-03	0.01	0.01	0.00	0.00	0.00	8.02E-03	0.01	0.01	292.26	364.25	434.13	2.60	3.24	3.86	0.00	0.00	0.00	0.00	0.00	0.00
Belgium - Escherichia coli 3GCREC RESP - model 17	145.49	169.43	196.89	280.27	328.77	376.84	426.55	499.16	572.45	0.93	1.03	1.15	1.29	1.51	1.75	2.49	2.93	3.35	3.80	4.44	5.09	396.79	483.98	567.54	3.53	4.31	5.05	14.15	17.39	20.63	0.13	0.15	0.18
Belgium - Escherichia coli 3GCREC SSI - model 19	1.79	2.47	3.33	399.27	464.56	526.43	401.84	467.11	528.29	0.38	0.40	0.43	0.02	0.02	0.03	3.55	4.13	4.68	3.58	4.16	4.70	1,001.36	1,162.17	1,302.46	8.91	10.34	11.59	27.98	33.12	37.86	0.25	0.29	0.34
Belgium - Escherichia coli 3GCREC UTI - model 18	7.38	9.20	11.49	0.00	0.00	0.00	7.38	9.20	11.49	1.60E-03	1.96E-03	2.35E-03	0.07	0.08	0.10	0.00	0.00	0.00	0.07	0.08	0.10	4,237.16	4,704.61	5,195.58	37.71	41.87	46.24	0.00	0.00	0.00	0.00	0.00	0.00
Belgium - Escherichia coli CoREC BSI - model 26	22.04	26.71	31.90	440.69	592.34	763.33	464.46	618.73	789.30	12.28	15.59	19.66	0.20	0.24	0.28	3.92	5.27	6.79	4.13	5.51	7.02	35.21	39.47	43.68	0.31	0.35	0.39	18.46	23.87	28.99	0.16	0.21	0.26
Belgium - Escherichia coli CoREC OTH - model 30	0.05	0.08	0.11	0.00	0.00	0.00	0.05	0.08	0.11	2.40E-03	3.49E-03	4.69E-03	4.45E-04	6.98E-04	1.01E-03	0.00	0.00	0.00	4.45E-04	6.98E-04	1.01E-03	17.92	22.73	27.46	0.16	0.20	0.24	0.00	0.00	0.00	0.00	0.00	0.00
Belgium - Escherichia coli CoREC RESP - model 27	6.48	9.09	11.70	12.80	17.69	22.56	19.21	26.86	33.70	1.08	1.36	1.67	0.06	0.08	0.10	0.11	0.16	0.20	0.17	0.24	0.30	15.23	19.77	24.10	0.14	0.18	0.21	0.55	0.71	0.88	4.86E-03	6.32E-03	7.85E-03
Belgium - Escherichia coli CoREC SSI - model 29	0.06	0.10	0.14	18.25	22.34	26.68	18.38	22.45	26.76	0.44	0.48	0.53	5.73E-04	8.80E-04	1.28E-03	0.16	0.20	0.24	0.16	0.20	0.24	38.19	46.32	54.95	0.34	0.41	0.49	1.00	1.25	1.52	8.91E-03	0.01	0.01
Belgium - Escherichia coli CoREC UTI - model 28	0.23	0.35	0.47	0.00	0.00	0.00	0.23	0.35	0.47	1.45E-03	2.07E-03	2.77E-03	2.08E-03	3.09E-03	4.21E-03	0.00	0.00	0.00	2.08E-03	3.09E-03	4.21E-03	140.60	166.55	191.64	1.25	1.48	1.71	0.00	0.00	0.00	0.00	0.00	0.00
Belgium - Escherichia coli CREC BSI - model 21	0.00	0.00	0.00	0.00	0.00	0.00	0.00	0.00	0.00	NaN	NaN	NaN	0.00	0.00	0.00	0.00	0.00	0.00	0.00	0.00	0.00	0.00	0.00	0.00	0.00	0.00	0.00	0.00	0.00	0.00	0.00	0.00	0.00
Belgium - Escherichia coli CREC OTH - model 25	0.00	0.00	0.00	0.00	0.00	0.00	0.00	0.00	0.00	NaN	NaN	NaN	0.00	0.00	0.00	0.00	0.00	0.00	0.00	0.00	0.00	0.00	0.00	0.00	0.00	0.00	0.00	0.00	0.00	0.00	0.00	0.00	0.00
Belgium - Escherichia coli CREC RESP - model 22	0.00	0.00	0.00	0.00	0.00	0.00	0.00	0.00	0.00	NaN	NaN	NaN	0.00	0.00	0.00	0.00	0.00	0.00	0.00	0.00	0.00	0.00	0.00	0.00	0.00	0.00	0.00	0.00	0.00	0.00	0.00	0.00	0.00
Belgium - Escherichia coli CREC SSI - model 24	0.00	0.00	0.00	0.00	0.00	0.00	0.00	0.00	0.00	NaN	NaN	NaN	0.00	0.00	0.00	0.00	0.00	0.00	0.00	0.00	0.00	0.00	0.00	0.00	0.00	0.00	0.00	0.00	0.00	0.00	0.00	0.00	0.00
Belgium - Escherichia coli CREC UTI - model 23	0.00	0.00	0.00	0.00	0.00	0.00	0.00	0.00	0.00	NaN	NaN	NaN	0.00	0.00	0.00	0.00	0.00	0.00	0.00	0.00	0.00	0.00	0.00	0.00	0.00	0.00	0.00	0.00	0.00	0.00	0.00	0.00	0.00
Belgium - Klebsiella pneumoniae 3GCRKP BSI - model 1	134.19	148.88	164.89	861.17	931.46	996.92	1,000.74	1,079.49	1,152.08	3.07	3.25	3.42	1.19	1.32	1.47	7.66	8.29	8.87	8.91	9.61	10.25	312.69	332.00	352.93	2.78	2.95	3.14	51.26	55.47	59.59	0.46	0.49	0.53
Belgium - Klebsiella pneumoniae 3GCRKP OTH - model 5	0.33	0.44	0.56	0.00	0.00	0.00	0.33	0.44	0.56	2.61E-03	3.33E-03	4.16E-03	2.92E-03	3.92E-03	5.03E-03	0.00	0.00	0.00	2.92E-03	3.92E-03	5.03E-03	112.84	132.04	152.33	1.00	1.18	1.36	0.00	0.00	0.00	0.00	0.00	0.00
Belgium - Klebsiella pneumoniae 3GCRKP RESP - model 2	78.16	90.42	103.52	150.73	173.06	195.20	230.41	263.15	297.40	0.85	0.92	0.99	0.70	0.80	0.92	1.34	1.54	1.74	2.05	2.34	2.65	250.40	287.19	321.97	2.23	2.56	2.87	8.89	10.33	11.77	0.08	0.09	0.10
Belgium - Klebsiella pneumoniae 3GCRKP UTI - model 3	0.58	0.76	0.97	0.00	0.00	0.00	0.58	0.76	0.97	1.52E-03	1.95E-03	2.39E-03	5.16E-03	6.76E-03	8.66E-03	0.00	0.00	0.00	5.16E-03	6.76E-03	8.66E-03	343.84	391.27	439.61	3.06	3.48	3.91	0.00	0.00	0.00	0.00	0.00	0.00
Belgium - Klebsiella pneumoniae 3GCRKP SSI - model 4	0.20	0.28	0.38	42.98	51.16	59.21	43.16	51.43	59.52	0.37	0.40	0.43	1.75E-03	2.48E-03	3.35E-03	0.38	0.46	0.53	0.38	0.46	0.53	111.22	129.32	148.58	0.99	1.15	1.32	3.19	3.77	4.41	0.03	0.03	0.04
Belgium - Klebsiella pneumoniae CoRKP BSI - model 11	10.32	12.83	15.70	195.81	277.63	376.44	207.36	290.94	392.20	7.93	10.57	13.83	0.09	0.11	0.14	1.74	2.47	3.35	1.85	2.59	3.49	23.61	27.58	31.88	0.21	0.25	0.28	12.11	16.47	22.11	0.11	0.15	0.20
Belgium - Klebsiella pneumoniae CoRKP OTH - model 15	0.02	0.04	0.07	0.00	0.00	0.00	0.02	0.04	0.07	2.11E-03	3.47E-03	5.05E-03	2.14E-04	3.92E-04	6.59E-04	0.00	0.00	0.00	2.14E-04	3.92E-04	6.59E-04	8.41	12.91	17.50	0.07	0.11	0.16	0.00	0.00	0.00	0.00	0.00	0.00
Belgium - Klebsiella pneumoniae CoRKP RESP - model 12	2.27	3.67	5.13	4.37	7.05	9.83	6.73	10.73	14.74	0.72	0.92	1.17	0.02	0.03	0.05	0.04	0.06	0.09	0.06	0.10	0.13	7.46	11.57	15.59	0.07	0.10	0.14	0.26	0.41	0.57	2.30E-03	3.69E-03	5.06E-03
Belgium - Klebsiella pneumoniae CoRKP SSI - model 14	8.60E-03	0.02	0.03	1.62	2.57	3.51	1.62	2.59	3.54	0.29	0.32	0.35	7.65E-05	1.51E-04	2.64E-04	0.01	0.02	0.03	0.01	0.02	0.03	5.23	8.09	11.07	0.05	0.07	0.10	0.13	0.22	0.31	1.17E-03	1.94E-03	2.79E-03
Belgium - Klebsiella pneumoniae CoRKP UTI - model 13	0.02	0.03	0.06	0.00	0.00	0.00	0.02	0.03	0.06	1.34E-03	2.06E-03	2.94E-03	1.78E-04	3.03E-04	4.94E-04	0.00	0.00	0.00	1.78E-04	3.03E-04	4.94E-04	11.65	16.73	22.00	0.10	0.15	0.20	0.00	0.00	0.00	0.00	0.00	0.00
Belgium - Klebsiella pneumoniae CRKP BSI - model 6	6.14	8.51	11.58	70.61	112.77	170.81	78.05	121.31	180.58	9.21	13.34	18.38	0.05	0.08	0.10	0.63	1.00	1.52	0.69	1.08	1.61	7.43	9.11	11.20	0.07	0.08	0.10	2.08	3.24	4.68	0.02	0.03	0.04
Belgium - Klebsiella pneumoniae CRKP OTH - model 10	5.32E-03	0.01	0.03	0.00	0.00	0.00	5.32E-03	0.01	0.03	1.68E-03	3.46E-03	6.03E-03	4.73E-05	1.27E-04	2.68E-04	0.00	0.00	0.00	4.73E-05	1.27E-04	2.68E-04	2.00	4.25	6.37	0.02	0.04	0.06	0.00	0.00	0.00	0.00	0.00	0.00
Belgium - Klebsiella pneumoniae CRKP RESP - model 7	1.13	2.48	4.02	2.27	4.82	7.86	3.44	7.30	11.65	1.46	1.88	2.41	0.01	0.02	0.04	0.02	0.04	0.07	0.03	0.06	0.10	1.91	3.88	5.85	0.02	0.03	0.05	0.07	0.14	0.22	6.17E-04	1.24E-03	1.94E-03
Belgium - Klebsiella pneumoniae CRKP SSI - model 9	1.74E-03	5.46E-03	0.01	0.80	1.63	2.49	0.81	1.63	2.51	0.51	0.60	0.69	1.55E-05	4.86E-05	1.03E-04	7.11E-03	0.01	0.02	7.18E-03	0.01	0.02	1.40	2.70	4.18	0.01	0.02	0.04	0.03	0.06	0.10	2.36E-04	5.44E-04	8.60E-04
Belgium - Klebsiella pneumoniae CRKP UTI - model 8	4.63E-03	0.01	0.02	0.00	0.00	0.00	4.63E-03	0.01	0.02	1.01E-03	1.96E-03	3.46E-03	4.12E-05	9.40E-05	1.94E-04	0.00	0.00	0.00	4.12E-05	9.40E-05	1.94E-04	2.97	5.57	8.08	0.03	0.05	0.07	0.00	0.00	0.00	0.00	0.00	0.00
Belgium - Pseudomonas aeruginosa CoRPA BSI - model 56	1.58	2.51	3.84	6.80	21.07	41.73	8.94	23.70	45.07	2.25	5.44	8.56	0.01	0.02	0.03	0.06	0.19	0.37	0.08	0.21	0.40	3.22	4.38	6.22	0.03	0.04	0.06	0.32	1.00	1.98	2.87E-03	8.90E-03	0.02
Belgium - Pseudomonas aeruginosa CoRPA OTH - model 60	1.65E-03	7.35E-03	0.02	0.00	0.00	0.00	1.65E-03	7.35E-03	0.02	1.52E-03	3.70E-03	6.59E-03	1.47E-05	6.54E-05	1.70E-04	0.00	0.00	0.00	1.47E-05	6.54E-05	1.70E-04	0.66	2.19	3.84	5.86E-03	0.02	0.03	0.00	0.00	0.00	0.00	0.00	0.00
Belgium - Pseudomonas aeruginosa CoRPA RESP - model 57	3.26	6.45	10.18	8.15	21.71	44.80	11.90	28.11	53.66	1.00	1.82	2.57	0.03	0.06	0.09	0.07	0.19	0.40	0.11	0.25	0.48	8.68	16.50	24.20	0.08	0.15	0.22	0.39	1.03	2.13	3.45E-03	9.18E-03	0.02
Belgium - Pseudomonas aeruginosa CoRPA SSI - model 59	1.10E-03	5.60E-03	0.02	0.76	2.35	3.86	0.76	2.36	3.90	0.76	0.76	0.76	9.75E-06	4.98E-05	1.48E-04	6.78E-03	0.02	0.03	6.79E-03	0.02	0.03	1.00	3.09	5.09	8.92E-03	0.03	0.05	0.04	0.11	0.18	3.21E-04	9.89E-04	1.63E-03
Belgium - Pseudomonas aeruginosa CoRPA UTI - model 58	3.12E-03	0.01	0.03	0.00	0.00	0.00	3.12E-03	0.01	0.03	8.06E-04	2.01E-03	3.60E-03	2.77E-05	9.25E-05	2.28E-04	0.00	0.00	0.00	2.77E-05	9.25E-05	2.28E-04	2.30	5.70	9.31	0.02	0.05	0.08	0.00	0.00	0.00	0.00	0.00	0.00
Belgium - Pseudomonas aeruginosa CRPA BSI - model 51	19.88	25.35	31.62	132.01	214.74	322.45	158.00	240.49	348.11	3.60	5.40	7.60	0.18	0.23	0.28	1.17	1.91	2.87	1.41	2.14	3.10	38.12	44.50	52.51	0.34	0.40	0.47	6.57	10.07	14.46	0.06	0.09	0.13
Belgium - Pseudomonas aeruginosa CRPA OTH - model 55	0.05	0.08	0.13	0.00	0.00	0.00	0.05	0.08	0.13	2.50E-03	3.60E-03	5.07E-03	4.24E-04	7.09E-04	1.17E-03	0.00	0.00	0.00	4.24E-04	7.09E-04	1.17E-03	14.67	22.46	30.28	0.13	0.20	0.27	0.00	0.00	0.00	0.00	0.00	0.00
Belgium - Pseudomonas aeruginosa CRPA RESP - model 52	47.07	65.44	81.84	139.07	227.85	337.33	193.35	292.77	415.34	1.27	1.78	2.36	0.42	0.58	0.73	1.24	2.03	3.00	1.72	2.61	3.70	128.37	166.71	203.31	1.14	1.48	1.81	7.12	10.71	15.33	0.06	0.10	0.14
Belgium - Pseudomonas aeruginosa CRPA SSI - model 54	0.03	0.06	0.12	10.46	16.49	22.07	10.57	16.56	22.18	0.49	0.54	0.58	3.04E-04	5.65E-04	1.03E-03	0.09	0.15	0.20	0.09	0.15	0.20	20.54	30.74	40.27	0.18	0.27	0.36	0.67	1.03	1.37	5.96E-03	9.13E-03	0.01
Belgium - Pseudomonas aeruginosa CRPA UTI - model 53	0.07	0.12	0.18	0.00	0.00	0.00	0.07	0.12	0.18	1.32E-03	2.04E-03	2.81E-03	6.30E-04	1.03E-03	1.59E-03	0.00	0.00	0.00	6.30E-04	1.03E-03	1.59E-03	41.97	57.98	72.88	0.37	0.52	0.65	0.00	0.00	0.00	0.00	0.00	0.00
Belgium - Pseudomonas aeruginosa MDRPA BSI - model 46	7.58	10.17	14.06	39.24	80.66	132.17	48.76	91.29	144.32	2.88	5.17	7.41	0.07	0.09	0.13	0.35	0.72	1.18	0.43	0.81	1.28	14.43	17.72	22.20	0.13	0.16	0.20	2.05	3.76	5.86	0.02	0.03	0.05
Belgium - Pseudomonas aeruginosa MDRPA OTH - model 50	0.01	0.03	0.06	0.00	0.00	0.00	0.01	0.03	0.06	2.15E-03	3.74E-03	5.56E-03	1.30E-04	3.02E-04	5.20E-04	0.00	0.00	0.00	1.30E-04	3.02E-04	5.20E-04	4.82	9.11	12.96	0.04	0.08	0.12	0.00	0.00	0.00	0.00	0.00	0.00
Belgium - Pseudomonas aeruginosa MDRPA RESP - model 47	17.45	26.37	36.38	44.24	86.64	141.92	65.03	113.72	176.71	1.12	1.73	2.38	0.16	0.23	0.32	0.39	0.77	1.26	0.58	1.01	1.57	45.85	65.85	86.50	0.41	0.59	0.77	2.17	4.04	6.41	0.02	0.04	0.06
Belgium - Pseudomonas aeruginosa MDRPA SSI - model 49	0.01	0.03	0.05	1.71	2.77	3.97	1.73	2.80	4.02	0.22	0.23	0.24	9.73E-05	2.29E-04	4.62E-04	0.02	0.02	0.04	0.02	0.02	0.04	7.46	12.20	17.32	0.07	0.11	0.15	0.11	0.19	0.28	9.39E-04	1.73E-03	2.46E-03
Belgium - Pseudomonas aeruginosa MDRPA UTI - model 48	0.02	0.05	0.08	0.00	0.00	0.00	0.02	0.05	0.08	1.19E-03	2.05E-03	3.11E-03	2.04E-04	4.05E-04	7.39E-04	0.00	0.00	0.00	2.04E-04	4.05E-04	7.39E-04	13.83	22.74	31.66	0.12	0.20	0.28	0.00	0.00	0.00	0.00	0.00	0.00
Belgium - Staphylococcus aureus MRSA BSI - model 61	258.69	280.12	302.16	1,782.31	1,883.47	1,986.72	2,052.89	2,162.82	2,279.44	4.33	4.54	4.71	2.30	2.49	2.69	15.86	16.76	17.68	18.27	19.25	20.28	453.21	477.54	501.95	4.03	4.25	4.47	80.06	85.68	91.38	0.71	0.76	0.81
Belgium - Staphylococcus aureus MRSA OTH - model 65	1.85	2.31	2.82	0.00	0.00	0.00	1.85	2.31	2.82	2.67E-03	3.28E-03	4.00E-03	0.02	0.02	0.03	0.00	0.00	0.00	0.02	0.02	0.03	642.57	702.29	765.50	5.72	6.25	6.81	0.00	0.00	0.00	0.00	0.00	0.00
Belgium - Staphylococcus aureus MRSA RESP - model 62	263.90	292.55	320.71	515.61	567.62	621.33	782.90	859.77	938.04	1.12	1.20	1.27	2.35	2.60	2.85	4.59	5.05	5.53	6.97	7.65	8.35	650.17	717.70	787.24	5.79	6.39	7.01	23.11	25.81	28.89	0.21	0.23	0.26
Belgium - Staphylococcus aureus MRSA SSI - model 64	1.29	1.69	2.17	306.06	338.17	371.32	307.80	339.81	373.08	0.42	0.43	0.45	0.01	0.02	0.02	2.72	3.01	3.30	2.74	3.02	3.32	715.48	786.21	854.27	6.37	7.00	7.60	18.98	21.45	23.76	0.17	0.19	0.21
Belgium - Staphylococcus aureus MRSA UTI - model 63	0.16	0.22	0.28	0.00	0.00	0.00	0.16	0.22	0.28	1.49E-03	1.87E-03	2.27E-03	1.45E-03	1.92E-03	2.49E-03	0.00	0.00	0.00	1.45E-03	1.92E-03	2.49E-03	93.38	116.58	135.64	0.83	1.04	1.21	0.00	0.00	0.00	0.00	0.00	0.00
Belgium - Streptococcus pneumoniae PMRSP BSI - model 76	3.42	3.91	4.46	24.80	26.54	28.33	28.69	30.45	32.28	4.99	5.30	5.61	0.03	0.03	0.04	0.22	0.24	0.25	0.26	0.27	0.29	5.75	5.75	5.75	0.05	0.05	0.05	0.97	1.04	1.10	8.66E-03	9.23E-03	9.80E-03
Belgium - Streptococcus pneumoniae PMRSP OTH - model 80	2.70E-03	3.97E-03	5.61E-03	0.00	0.00	0.00	2.70E-03	3.97E-03	5.61E-03	1.35E-03	1.94E-03	2.69E-03	2.40E-05	3.54E-05	4.99E-05	0.00	0.00	0.00	2.40E-05	3.54E-05	4.99E-05	1.84	2.05	2.30	0.02	0.02	0.02	0.00	0.00	0.00	0.00	0.00	0.00
Belgium - Streptococcus pneumoniae PMRSP RESP - model 77	8.26	10.27	13.01	16.35	20.17	25.47	25.02	30.35	38.10	1.29	1.40	1.53	0.07	0.09	0.12	0.15	0.18	0.23	0.22	0.27	0.34	18.15	21.73	26.95	0.16	0.19	0.24	0.64	0.79	0.98	5.70E-03	7.00E-03	8.76E-03
Belgium - Streptococcus pneumoniae PMRSP SSI - model 79	0.00	0.00	0.00	0.00	0.00	0.00	0.00	0.00	0.00	NaN	NaN	NaN	0.00	0.00	0.00	0.00	0.00	0.00	0.00	0.00	0.00	0.00	0.00	0.00	0.00	0.00	0.00	0.00	0.00	0.00	0.00	0.00	0.00
Belgium - Streptococcus pneumoniae PMRSP UTI - model 78	0.00	0.00	0.00	0.00	0.00	0.00	0.00	0.00	0.00	NaN	NaN	NaN	0.00	0.00	0.00	0.00	0.00	0.00	0.00	0.00	0.00	0.00	0.00	0.00	0.00	0.00	0.00	0.00	0.00	0.00	0.00	0.00	0.00
Belgium - Streptococcus pneumoniae PRSP BSI - model 71	3.84	4.58	5.42	28.78	31.52	34.07	33.42	36.05	38.80	9.69	10.45	11.25	0.03	0.04	0.05	0.26	0.28	0.30	0.30	0.32	0.35	3.45	3.45	3.45	0.03	0.03	0.03	0.57	0.62	0.67	5.03E-03	5.52E-03	5.96E-03
Belgium - Streptococcus pneumoniae PRSP OTH - model 75	1.36E-03	2.35E-03	3.62E-03	0.00	0.00	0.00	1.36E-03	2.35E-03	3.62E-03	1.14E-03	1.92E-03	2.86E-03	1.21E-05	2.09E-05	3.22E-05	0.00	0.00	0.00	1.21E-05	2.09E-05	3.22E-05	1.08	1.22	1.42	9.58E-03	0.01	0.01	0.00	0.00	0.00	0.00	0.00	0.00
Belgium - Streptococcus pneumoniae PRSP RESP - model 72	9.29	11.98	16.42	18.37	23.55	31.64	28.27	35.59	47.75	2.51	2.76	3.02	0.08	0.11	0.15	0.16	0.21	0.28	0.25	0.32	0.42	10.51	12.91	16.95	0.09	0.11	0.15	0.36	0.47	0.63	3.23E-03	4.14E-03	5.58E-03
Belgium - Streptococcus pneumoniae PRSP SSI - model 74	0.00	0.00	0.00	0.00	0.00	0.00	0.00	0.00	0.00	NaN	NaN	NaN	0.00	0.00	0.00	0.00	0.00	0.00	0.00	0.00	0.00	0.00	0.00	0.00	0.00	0.00	0.00	0.00	0.00	0.00	0.00	0.00	0.00
Belgium - Streptococcus pneumoniae PRSP UTI - model 73	0.00	0.00	0.00	0.00	0.00	0.00	0.00	0.00	0.00	NaN	NaN	NaN	0.00	0.00	0.00	0.00	0.00	0.00	0.00	0.00	0.00	0.00	0.00	0.00	0.00	0.00	0.00	0.00	0.00	0.00	0.00	0.00	0.00
**Total (Median & 95% UIs)**	1,587.77	1,804.25	2,046.61	8,859.50	10,264.98	11,840.22	10,513.91	12,070.36	13,809.39		-		14.13	16.06	18.21	78.84	91.35	105.37	93.56	107.41	122.89	11,386.69	12,891.92	14,407.66	101.33	114.72	128.21	455.70	530.04	612.86	4.06	4.72	5.45
**Total (average)**		**1,807.69**			**10,292.81**			**12,100.51**			-			**16.09**			**91.60**			**107.68**			**12,891.70**			**114.72**			**531.48**			**4.73**	

Infection	YLD			YLL			DALY			DALY per Case			YLD per 100,000			YLL per 100,000			DALY per 100,000			Cases per year			Incidence per 100,000			Deaths per year			Mortality per 100,000		
Colistin resistant *Acinetobacter spp.*	0.00	0.00	0.00	0.00	0.00	0.00	0.00	0.00	0.00	0.00	0.00	0.00	0.00	0.00	0.00	0.00	0.00	0.00	0.00	0.00	0.00	0.00	0.00	0.00	0.00	0.00	0.00	0.00	0.00	0.00	0.00	0.00	0.00
Carbapenem resistant *Acinetobacter spp.* (excluding those resistant to colistin)	512.91	575.63	641.66	2,535.38	3,088.33	3,686.61	3,071.28	3,666.11	4,295.22	9.23	10.75	12.38	7.12	7.99	8.91	35.20	42.88	51.19	42.64	50.90	59.64	887.30	1,001.34	1,111.02	12.32	13.90	15.43	72.34	86.95	102.94	1.00	1.21	1.43
Aminoglycosides and fluoroquinolones resistant *Acinetobacter* spp. (excluding those resistant to colistin and/or carbapenem)	30.79	37.31	45.21	138.29	196.45	257.65	170.77	233.97	297.95	6.39	8.33	10.13	0.43	0.52	0.63	1.92	2.73	3.58	2.37	3.25	4.14	69.12	83.32	97.71	0.96	1.16	1.36	5.10	7.15	9.15	0.07	0.10	0.13
Vancomycin resistant Enterococci (*E. faecalis* and *E. faecium*)	17.87	25.72	35.84	170.26	235.12	315.42	188.23	261.29	349.32	8.50	9.52	10.74	0.25	0.36	0.50	2.36	3.26	4.38	2.61	3.63	4.85	73.78	107.43	142.63	1.02	1.49	1.98	4.83	6.69	8.81	0.07	0.09	0.12
Colistin resistant *Escherichia coli*	0.00	0.00	0.00	0.00	0.00	0.00	0.00	0.00	0.00	0.00	0.00	0.00	0.00	0.00	0.00	0.00	0.00	0.00	0.00	0.00	0.00	0.00	0.00	0.00	0.00	0.00	0.00	0.00	0.00	0.00	0.00	0.00	0.00
Carbapenem resistant *Escherichia coli* (excluding those resistant to colistin)	0.00	0.00	0.00	0.00	0.00	0.00	0.00	0.00	0.00	0.00	0.00	0.00	0.00	0.00	0.00	0.00	0.00	0.00	0.00	0.00	0.00	0.00	0.00	0.00	0.00	0.00	0.00	0.00	0.00	0.00	0.00	0.00	0.00
Third-generation cephalosporin resistant *Escherichia coli* (excluding those resistant to colistin and/or carbapenem)	195.26	226.07	260.52	1,162.07	1,339.75	1,543.63	1,359.34	1,568.36	1,791.68	5.57	6.11	6.67	2.71	3.14	3.62	16.13	18.60	21.43	18.87	21.78	24.88	1,789.47	2,052.35	2,320.28	24.85	28.50	32.22	53.37	62.05	71.37	0.74	0.86	0.99
Colistin resistant *Klebsiella pneumoniae*	0.00	0.00	0.00	0.00	0.00	0.00	0.00	0.00	0.00	0.00	0.00	0.00	0.00	0.00	0.00	0.00	0.00	0.00	0.00	0.00	0.00	0.00	0.00	0.00	0.00	0.00	0.00	0.00	0.00	0.00	0.00	0.00	0.00
Carbapenem resistant *Klebsiella pneumoniae* (excluding those resistant to colistin)	8.17	13.15	19.85	82.56	142.89	229.17	91.63	156.21	245.40	15.63	23.98	33.43	0.11	0.18	0.28	1.15	1.98	3.18	1.27	2.17	3.41	12.28	20.58	29.18	0.17	0.29	0.41	1.68	2.73	4.10	0.02	0.04	0.06
Third-generation cephalosporin resistant *Klebsiella pneumoniae* (excluding those resistant to colistin and/or carbapenem)	380.33	436.18	500.65	1,893.69	2,090.75	2,326.96	2,285.91	2,525.80	2,806.04	10.29	11.01	11.79	5.28	6.06	6.95	26.29	29.03	32.31	31.74	35.07	38.96	832.20	935.35	1,039.62	11.55	12.99	14.43	45.83	49.97	54.71	0.64	0.69	0.76
Colistin resistant *Pseudomonas aeruginosa*	0.00	0.00	0.00	0.00	0.00	0.00	0.00	0.00	0.00	0.00	0.00	0.00	0.00	0.00	0.00	0.00	0.00	0.00	0.00	0.00	0.00	0.00	0.00	0.00	0.00	0.00	0.00	0.00	0.00	0.00	0.00	0.00	0.00
Carbapenem resistant *Pseudomonas aeruginosa* (excluding those resistant to colistin)	139.90	170.42	204.69	603.68	859.87	1,149.03	771.64	1,028.08	1,341.59	9.42	12.17	15.12	1.94	2.37	2.84	8.38	11.94	15.95	10.71	14.27	18.63	306.48	375.14	445.34	4.26	5.21	6.18	18.15	25.16	32.95	0.25	0.35	0.46
Pseudomonas aeruginosa resistant to three or more antimicrobial groups (excluding those resistant to colistin and/or carbapenem)	11.63	18.27	25.48	41.81	85.12	147.90	56.73	103.63	170.09	5.09	8.57	12.78	0.16	0.25	0.35	0.58	1.18	2.05	0.79	1.44	2.36	33.37	53.46	74.71	0.46	0.74	1.04	1.67	3.34	5.52	0.02	0.05	0.08
Methicillin resistant *Staphylococcus aureus*	172.48	191.41	214.14	831.65	896.90	968.85	1.008.13	1.088.32	1.176.85	8.96	9.38	9.80	2.39	2.66	2.97	11.55	12.45	13.45	14.00	15.11	16.34	554.12	602.77	654.78	7.69	8.37	9.09	24.99	26.95	28.98	0.35	0.37	0.40
Penicillin and macrolides resistant *Streptococcus pneumoniae* (excluding those only resistant to penicillin)	51.82	65.35	84.92	186.36	215.30	256.56	241.82	280.98	336.33	11.84	12.86	14.02	0.72	0.91	1.18	2.59	2.99	3.56	3.36	3.90	4.67	60.59	71.17	85.85	0.84	0.99	1.19	3.81	4.37	5.10	0.05	0.06	0.07
Penicillin tesistant *Streptococcus pneumoniae* (excluding those resistant to macrolides)	45.66	57.58	75.97	163.80	189.98	231.04	212.26	247.52	301.18	10.41	11.35	12.39	0.63	0.80	1.05	2.27	2.64	3.21	2.95	3.44	4.18	60.47	71.27	86.61	0.84	0.99	1.20	3.79	4.36	5.14	0.05	0.06	0.07
Total	1,566.81	1,817.08	2,108.93	7,809.54	9,340.44	11,112.82	9,457.75	11,160.29	13,111.65				21.75	25.23	29.28	108.43	129.69	154.30	131.32	154.96	182.05	4,679.17	5,374.19	6,087.71	64.97	74.62	84.53	235.55	279.72	328.78	3.27	3.88	4.57

Model	YLD			YLL			DALY			DALY per Case			YLD per 100,000			YLL per 100,000			DALY per 100,000			Cases per year			Incidence per 100,000			Deaths per year			Mortality per 100,000		
Bulgaria - Acinetobacter spp. ColRACI BSI - model 41	0.00	0.00	0.00	0.00	0.00	0.00	0.00	0.00	0.00	NaN	NaN	NaN	0.00	0.00	0.00	0.00	0.00	0.00	0.00	0.00	0.00	0.00	0.00	0.00	0.00	0.00	0.00	0.00	0.00	0.00	0.00	0.00	0.00
Bulgaria - Acinetobacter spp. ColRACI OTH - model 45	0.00	0.00	0.00	0.00	0.00	0.00	0.00	0.00	0.00	NaN	NaN	NaN	0.00	0.00	0.00	0.00	0.00	0.00	0.00	0.00	0.00	0.00	0.00	0.00	0.00	0.00	0.00	0.00	0.00	0.00	0.00	0.00	0.00
Bulgaria - Acinetobacter spp. ColRACI RESP - model 42	0.00	0.00	0.00	0.00	0.00	0.00	0.00	0.00	0.00	NaN	NaN	NaN	0.00	0.00	0.00	0.00	0.00	0.00	0.00	0.00	0.00	0.00	0.00	0.00	0.00	0.00	0.00	0.00	0.00	0.00	0.00	0.00	0.00
Bulgaria - Acinetobacter spp. ColRACI SSI - model 44	0.00	0.00	0.00	0.00	0.00	0.00	0.00	0.00	0.00	NaN	NaN	NaN	0.00	0.00	0.00	0.00	0.00	0.00	0.00	0.00	0.00	0.00	0.00	0.00	0.00	0.00	0.00	0.00	0.00	0.00	0.00	0.00	0.00
Bulgaria - Acinetobacter spp. ColRACI UTI - model 43	0.00	0.00	0.00	0.00	0.00	0.00	0.00	0.00	0.00	NaN	NaN	NaN	0.00	0.00	0.00	0.00	0.00	0.00	0.00	0.00	0.00	0.00	0.00	0.00	0.00	0.00	0.00	0.00	0.00	0.00	0.00	0.00	0.00
Bulgaria - Acinetobacter spp. CRACI BSI - model 36	296.17	328.53	361.68	2,062.37	2,547.40	3,078.02	2,376.63	2,877.90	3,412.30	6.95	8.34	9.82	4.11	4.56	5.02	28.64	35.37	42.74	33.00	39.96	47.38	322.21	345.59	369.93	4.47	4.80	5.14	58.70	71.35	85.34	0.82	0.99	1.18
Bulgaria - Acinetobacter spp. CRACI OTH - model 40	0.03	0.05	0.06	0.00	0.00	0.00	0.03	0.05	0.06	3.02E-03	3.75E-03	4.55E-03	4.54E-04	6.44E-04	8.94E-04	0.00	0.00	0.00	4.54E-04	6.44E-04	8.94E-04	9.22	12.38	15.71	0.13	0.17	0.22	0.00	0.00	0.00	0.00	0.00	0.00
Bulgaria - Acinetobacter spp. CRACI RESP - model 37	216.29	246.50	279.19	425.25	483.66	542.63	646.41	730.36	816.26	1.84	1.95	2.07	3.00	3.42	3.88	5.90	6.72	7.53	8.98	10.14	11.33	330.95	374.38	415.19	4.60	5.20	5.76	11.88	13.44	15.04	0.16	0.19	0.21
Bulgaria - Acinetobacter spp. CRACI SSI - model 39	0.20	0.27	0.36	47.77	57.27	65.96	47.99	57.52	66.22	0.43	0.46	0.48	2.73E-03	3.70E-03	4.94E-03	0.66	0.80	0.92	0.67	0.80	0.92	106.21	126.43	145.48	1.47	1.76	2.02	1.75	2.16	2.56	0.02	0.03	0.04
Bulgaria - Acinetobacter spp. CRACI UTI - model 38	0.22	0.29	0.37	0.00	0.00	0.00	0.22	0.29	0.37	1.67E-03	2.06E-03	2.45E-03	3.08E-03	4.03E-03	5.13E-03	0.00	0.00	0.00	3.08E-03	4.03E-03	5.13E-03	118.71	142.56	164.72	1.65	1.98	2.29	0.00	0.00	0.00	0.00	0.00	0.00
Bulgaria - Acinetobacter spp. MDRACI BSI - model 31	17.99	21.25	25.41	109.81	160.64	213.51	129.27	182.07	234.45	4.60	6.36	7.96	0.25	0.29	0.35	1.52	2.23	2.96	1.79	2.53	3.26	25.92	28.79	31.61	0.36	0.40	0.44	4.03	5.81	7.53	0.06	0.08	0.10
Bulgaria - Acinetobacter spp. MDRACI OTH - model 35	2.22E-03	3.88E-03	6.11E-03	0.00	0.00	0.00	2.22E-03	3.88E-03	6.11E-03	2.68E-03	3.72E-03	4.85E-03	3.08E-05	5.39E-05	8.48E-05	0.00	0.00	0.00	3.08E-05	5.39E-05	8.48E-05	0.68	1.05	1.49	9.42E-03	0.01	0.02	0.00	0.00	0.00	0.00	0.00	0.00
Bulgaria - Acinetobacter spp. MDRACI RESP - model 32	12.77	16.02	19.73	25.06	31.14	38.30	38.03	47.19	57.60	1.38	1.52	1.67	0.18	0.22	0.27	0.35	0.43	0.53	0.53	0.66	0.80	25.99	31.05	36.75	0.36	0.43	0.51	0.91	1.12	1.34	0.01	0.02	0.02
Bulgaria - Acinetobacter spp. MDRACI SSI - model 34	0.01	0.02	0.03	3.42	4.66	5.83	3.44	4.69	5.86	0.41	0.44	0.49	1.80E-04	3.04E-04	4.60E-04	0.05	0.06	0.08	0.05	0.07	0.08	7.77	10.53	13.09	0.11	0.15	0.18	0.15	0.22	0.28	2.14E-03	2.99E-03	3.84E-03
Bulgaria - Acinetobacter spp. MDRACI UTI - model 33	0.02	0.02	0.03	0.00	0.00	0.00	0.02	0.02	0.03	1.50E-03	2.05E-03	2.64E-03	2.19E-04	3.36E-04	4.77E-04	0.00	0.00	0.00	2.19E-04	3.36E-04	4.77E-04	8.76	11.90	14.77	0.12	0.17	0.21	0.00	0.00	0.00	0.00	0.00	0.00
Bulgaria - Enterococcus faecalis and E. faecium VRE BSI - model 66	17.78	25.57	35.60	160.46	220.73	296.27	178.34	246.76	329.95	8.15	9.14	10.32	0.25	0.35	0.49	2.23	3.06	4.11	2.48	3.43	4.58	19.92	27.02	35.39	0.28	0.38	0.49	4.52	6.18	8.10	0.06	0.09	0.11
Bulgaria - Enterococcus faecalis and E. faecium VRE OTH - model 70	0.03	0.05	0.08	0.00	0.00	0.00	0.03	0.05	0.08	1.12E-03	1.66E-03	2.32E-03	3.97E-04	6.62E-04	1.05E-03	0.00	0.00	0.00	3.97E-04	6.62E-04	1.05E-03	19.40	29.03	38.82	0.27	0.40	0.54	0.00	0.00	0.00	0.00	0.00	0.00
Bulgaria - Enterococcus faecalis and E. faecium VRE RESP - model 67	0.00	0.00	0.00	0.00	0.00	0.00	0.00	0.00	0.00	NaN	NaN	NaN	0.00	0.00	0.00	0.00	0.00	0.00	0.00	0.00	0.00	0.00	0.00	0.00	0.00	0.00	0.00	0.00	0.00	0.00	0.00	0.00	0.00
Bulgaria - Enterococcus faecalis and E. faecium VRE SSI - model 69	0.04	0.08	0.13	9.80	14.39	19.15	9.86	14.46	19.26	0.35	0.38	0.42	6.00E-04	1.09E-03	1.75E-03	0.14	0.20	0.27	0.14	0.20	0.27	25.26	37.58	49.67	0.35	0.52	0.69	0.32	0.51	0.71	4.41E-03	7.11E-03	9.79E-03
Bulgaria - Enterococcus faecalis and E. faecium VRE UTI - model 68	0.02	0.03	0.04	0.00	0.00	0.00	0.02	0.03	0.04	1.26E-03	1.86E-03	2.51E-03	2.10E-04	3.51E-04	5.36E-04	0.00	0.00	0.00	2.10E-04	3.51E-04	5.36E-04	9.20	13.81	18.75	0.13	0.19	0.26	0.00	0.00	0.00	0.00	0.00	0.00
Bulgaria - Escherichia coli 3GCREC BSI - model 16	150.42	170.30	193.11	967.05	1,102.57	1,261.94	1,119.06	1,275.49	1,444.65	4.03	4.44	4.87	2.09	2.36	2.68	13.43	15.31	17.52	15.54	17.71	20.06	262.47	286.77	311.54	3.64	3.98	4.33	43.17	49.67	56.79	0.60	0.69	0.79
Bulgaria - Escherichia coli 3GCREC OTH - model 20	0.23	0.32	0.43	0.00	0.00	0.00	0.23	0.32	0.43	2.63E-03	3.34E-03	4.17E-03	3.24E-03	4.43E-03	6.04E-03	0.00	0.00	0.00	3.24E-03	4.43E-03	6.04E-03	74.87	95.58	116.97	1.04	1.33	1.62	0.00	0.00	0.00	0.00	0.00	0.00
Bulgaria - Escherichia coli 3GCREC RESP - model 17	42.29	52.39	62.97	81.63	101.40	122.84	124.37	153.75	184.78	1.12	1.21	1.32	0.59	0.73	0.87	1.13	1.41	1.71	1.73	2.13	2.57	102.37	126.92	152.25	1.42	1.76	2.11	3.68	4.58	5.51	0.05	0.06	0.08
Bulgaria - Escherichia coli 3GCREC SSI - model 19	0.46	0.64	0.89	113.39	135.78	158.85	113.82	136.39	158.70	0.42	0.45	0.48	6.36E-03	8.94E-03	0.01	1.57	1.89	2.21	1.58	1.89	2.20	259.79	303.95	351.93	3.61	4.22	4.89	6.52	7.81	9.07	0.09	0.11	0.13
Bulgaria - Escherichia coli 3GCREC UTI - model 18	1.86	2.41	3.11	0.00	0.00	0.00	1.86	2.41	3.11	1.57E-03	1.95E-03	2.41E-03	0.03	0.03	0.04	0.00	0.00	0.00	0.03	0.03	0.04	1,089.98	1,239.12	1,387.60	15.13	17.20	19.27	0.00	0.00	0.00	0.00	0.00	0.00
Bulgaria - Escherichia coli ColREC BSI - model 26	0.00	0.00	0.00	0.00	0.00	0.00	0.00	0.00	0.00	NaN	NaN	NaN	0.00	0.00	0.00	0.00	0.00	0.00	0.00	0.00	0.00	0.00	0.00	0.00	0.00	0.00	0.00	0.00	0.00	0.00	0.00	0.00	0.00
Bulgaria - Escherichia coli ColREC OTH - model 30	0.00	0.00	0.00	0.00	0.00	0.00	0.00	0.00	0.00	NaN	NaN	NaN	0.00	0.00	0.00	0.00	0.00	0.00	0.00	0.00	0.00	0.00	0.00	0.00	0.00	0.00	0.00	0.00	0.00	0.00	0.00	0.00	0.00
Bulgaria - Escherichia coli ColREC RESP - model 27	0.00	0.00	0.00	0.00	0.00	0.00	0.00	0.00	0.00	NaN	NaN	NaN	0.00	0.00	0.00	0.00	0.00	0.00	0.00	0.00	0.00	0.00	0.00	0.00	0.00	0.00	0.00	0.00	0.00	0.00	0.00	0.00	0.00
Bulgaria - Escherichia coli ColREC SSI - model 29	0.00	0.00	0.00	0.00	0.00	0.00	0.00	0.00	0.00	NaN	NaN	NaN	0.00	0.00	0.00	0.00	0.00	0.00	0.00	0.00	0.00	0.00	0.00	0.00	0.00	0.00	0.00	0.00	0.00	0.00	0.00	0.00	0.00
Bulgaria - Escherichia coli ColREC UTI - model 28	0.00	0.00	0.00	0.00	0.00	0.00	0.00	0.00	0.00	NaN	NaN	NaN	0.00	0.00	0.00	0.00	0.00	0.00	0.00	0.00	0.00	0.00	0.00	0.00	0.00	0.00	0.00	0.00	0.00	0.00	0.00	0.00	0.00
Bulgaria - Escherichia coli CREC BSI - model 21	0.00	0.00	0.00	0.00	0.00	0.00	0.00	0.00	0.00	NaN	NaN	NaN	0.00	0.00	0.00	0.00	0.00	0.00	0.00	0.00	0.00	0.00	0.00	0.00	0.00	0.00	0.00	0.00	0.00	0.00	0.00	0.00	0.00
Bulgaria - Escherichia coli CREC OTH - model 25	0.00	0.00	0.00	0.00	0.00	0.00	0.00	0.00	0.00	NaN	NaN	NaN	0.00	0.00	0.00	0.00	0.00	0.00	0.00	0.00	0.00	0.00	0.00	0.00	0.00	0.00	0.00	0.00	0.00	0.00	0.00	0.00	0.00
Bulgaria - Escherichia coli CREC RESP - model 22	0.00	0.00	0.00	0.00	0.00	0.00	0.00	0.00	0.00	NaN	NaN	NaN	0.00	0.00	0.00	0.00	0.00	0.00	0.00	0.00	0.00	0.00	0.00	0.00	0.00	0.00	0.00	0.00	0.00	0.00	0.00	0.00	0.00
Bulgaria - Escherichia coli CREC SSI - model 24	0.00	0.00	0.00	0.00	0.00	0.00	0.00	0.00	0.00	NaN	NaN	NaN	0.00	0.00	0.00	0.00	0.00	0.00	0.00	0.00	0.00	0.00	0.00	0.00	0.00	0.00	0.00	0.00	0.00	0.00	0.00	0.00	0.00
Bulgaria - Escherichia coli CREC UTI - model 23	0.00	0.00	0.00	0.00	0.00	0.00	0.00	0.00	0.00	NaN	NaN	NaN	0.00	0.00	0.00	0.00	0.00	0.00	0.00	0.00	0.00	0.00	0.00	0.00	0.00	0.00	0.00	0.00	0.00	0.00	0.00	0.00	0.00
Bulgaria - Klebsiella pneumoniae 3GCRKP BSI - model 1	241.41	271.79	306.55	1,578.46	1,720.00	1,891.45	1,829.66	1,990.55	2,182.05	7.70	8.18	8.70	3.35	3.77	4.26	21.92	23.88	26.26	25.40	27.64	30.30	228.38	243.64	260.45	3.17	3.38	3.62	37.83	40.69	44.06	0.53	0.56	0.61
Bulgaria - Klebsiella pneumoniae 3GCRKP OTH - model 5	0.25	0.32	0.42	0.00	0.00	0.00	0.25	0.32	0.42	2.65E-03	3.36E-03	4.20E-03	3.45E-03	4.49E-03	5.85E-03	0.00	0.00	0.00	3.45E-03	4.49E-03	5.85E-03	82.49	97.28	110.82	1.15	1.35	1.54	0.00	0.00	0.00	0.00	0.00	0.00
Bulgaria - Klebsiella pneumoniae 3GCRKP RESP - model 2	138.09	163.30	192.69	272.89	319.76	375.94	413.03	483.19	562.95	2.08	2.30	2.52	1.92	2.27	2.68	3.79	4.44	5.22	5.73	6.71	7.82	185.11	210.90	236.70	2.57	2.93	3.29	6.60	7.59	8.64	0.09	0.11	0.12
Bulgaria - Klebsiella pneumoniae 3GCRKP SSI - model 4	0.15	0.20	0.28	42.34	50.99	59.56	42.53	51.19	59.91	0.50	0.53	0.57	2.04E-03	2.82E-03	3.92E-03	0.59	0.71	0.83	0.59	0.71	0.83	82.12	95.65	110.50	1.14	1.33	1.53	1.40	1.68	2.00	0.02	0.02	0.03
Bulgaria - Klebsiella pneumoniae 3GCRKP UTI - model 3	0.44	0.56	0.70	0.00	0.00	0.00	0.44	0.56	0.70	1.56E-03	1.96E-03	2.38E-03	6.06E-03	7.77E-03	9.75E-03	0.00	0.00	0.00	6.06E-03	7.77E-03	9.75E-03	254.10	287.89	321.15	3.53	4.00	4.46	0.00	0.00	0.00	0.00	0.00	0.00
Bulgaria - Klebsiella pneumoniae ColRKP BSI - model 11	0.00	0.00	0.00	0.00	0.00	0.00	0.00	0.00	0.00	NaN	NaN	NaN	0.00	0.00	0.00	0.00	0.00	0.00	0.00	0.00	0.00	0.00	0.00	0.00	0.00	0.00	0.00	0.00	0.00	0.00	0.00	0.00	0.00
Bulgaria - Klebsiella pneumoniae ColRKP OTH - model 15	0.00	0.00	0.00	0.00	0.00	0.00	0.00	0.00	0.00	NaN	NaN	NaN	0.00	0.00	0.00	0.00	0.00	0.00	0.00	0.00	0.00	0.00	0.00	0.00	0.00	0.00	0.00	0.00	0.00	0.00	0.00	0.00	0.00
Bulgaria - Klebsiella pneumoniae ColRKP RESP - model 12	0.00	0.00	0.00	0.00	0.00	0.00	0.00	0.00	0.00	NaN	NaN	NaN	0.00	0.00	0.00	0.00	0.00	0.00	0.00	0.00	0.00	0.00	0.00	0.00	0.00	0.00	0.00	0.00	0.00	0.00	0.00	0.00	0.00
Bulgaria - Klebsiella pneumoniae ColRKP SSI - model 14	0.00	0.00	0.00	0.00	0.00	0.00	0.00	0.00	0.00	NaN	NaN	NaN	0.00	0.00	0.00	0.00	0.00	0.00	0.00	0.00	0.00	0.00	0.00	0.00	0.00	0.00	0.00	0.00	0.00	0.00	0.00	0.00	0.00
Bulgaria - Klebsiella pneumoniae ColRKP UTI - model 13	0.00	0.00	0.00	0.00	0.00	0.00	0.00	0.00	0.00	NaN	NaN	NaN	0.00	0.00	0.00	0.00	0.00	0.00	0.00	0.00	0.00	0.00	0.00	0.00	0.00	0.00	0.00	0.00	0.00	0.00	0.00	0.00	0.00
Bulgaria - Klebsiella pneumoniae CRKP BSI - model 6	7.02	10.09	14.59	79.55	135.21	216.42	87.52	145.39	227.63	13.02	20.30	28.64	0.10	0.14	0.20	1.10	1.88	3.00	1.22	2.02	3.16	5.69	7.25	9.09	0.08	0.10	0.13	1.61	2.57	3.85	0.02	0.04	0.05
Bulgaria - Klebsiella pneumoniae CRKP OTH - model 10	4.38E-03	0.01	0.02	0.00	0.00	0.00	4.38E-03	0.01	0.02	1.46E-03	3.38E-03	6.04E-03	6.09E-05	1.59E-04	3.35E-04	0.00	0.00	0.00	6.09E-05	1.59E-04	3.35E-04	1.70	3.46	5.29	0.02	0.05	0.07	0.00	0.00	0.00	0.00	0.00	0.00
Bulgaria - Klebsiella pneumoniae CRKP RESP - model 7	1.15	3.04	5.22	2.24	6.01	10.16	3.34	9.14	15.12	1.84	2.90	4.01	0.02	0.04	0.07	0.03	0.08	0.14	0.05	0.13	0.21	1.42	3.12	4.81	0.02	0.04	0.07	0.05	0.11	0.17	6.95E-04	1.56E-03	2.43E-03
Bulgaria - Klebsiella pneumoniae CRKP SSI - model 9	1.32E-03	4.38E-03	9.51E-03	0.77	1.66	2.60	0.77	1.67	2.60	0.76	0.77	0.77	1.83E-05	6.09E-05	1.32E-04	0.01	0.02	0.04	0.01	0.02	0.04	1.00	2.18	3.39	0.01	0.03	0.05	0.02	0.05	0.08	2.57E-04	6.70E-04	1.11E-03
Bulgaria - Klebsiella pneumoniae CRKP UTI - model 8	3.61E-03	8.93E-03	0.02	0.00	0.00	0.00	3.61E-03	8.93E-03	0.02	9.79E-04	2.06E-03	3.37E-03	5.02E-05	1.24E-04	2.54E-04	0.00	0.00	0.00	5.02E-05	1.24E-04	2.54E-04	2.48	4.56	6.60	0.03	0.06	0.09	0.00	0.00	0.00	0.00	0.00	0.00
Bulgaria - Pseudomonas aeruginosa ColRPA BSI - model 56	0.00	0.00	0.00	0.00	0.00	0.00	0.00	0.00	0.00	NaN	NaN	NaN	0.00	0.00	0.00	0.00	0.00	0.00	0.00	0.00	0.00	0.00	0.00	0.00	0.00	0.00	0.00	0.00	0.00	0.00	0.00	0.00	0.00
Bulgaria - Pseudomonas aeruginosa ColRPA OTH - model 60	0.00	0.00	0.00	0.00	0.00	0.00	0.00	0.00	0.00	NaN	NaN	NaN	0.00	0.00	0.00	0.00	0.00	0.00	0.00	0.00	0.00	0.00	0.00	0.00	0.00	0.00	0.00	0.00	0.00	0.00	0.00	0.00	0.00
Bulgaria - Pseudomonas aeruginosa ColRPA RESP - model 57	0.00	0.00	0.00	0.00	0.00	0.00	0.00	0.00	0.00	NaN	NaN	NaN	0.00	0.00	0.00	0.00	0.00	0.00	0.00	0.00	0.00	0.00	0.00	0.00	0.00	0.00	0.00	0.00	0.00	0.00	0.00	0.00	0.00
Bulgaria - Pseudomonas aeruginosa ColRPA SSI - model 59	0.00	0.00	0.00	0.00	0.00	0.00	0.00	0.00	0.00	NaN	NaN	NaN	0.00	0.00	0.00	0.00	0.00	0.00	0.00	0.00	0.00	0.00	0.00	0.00	0.00	0.00	0.00	0.00	0.00	0.00	0.00	0.00	0.00
Bulgaria - Pseudomonas aeruginosa ColRPA UTI - model 58	0.00	0.00	0.00	0.00	0.00	0.00	0.00	0.00	0.00	NaN	NaN	NaN	0.00	0.00	0.00	0.00	0.00	0.00	0.00	0.00	0.00	0.00	0.00	0.00	0.00	0.00	0.00	0.00	0.00	0.00	0.00	0.00	0.00
Bulgaria - Pseudomonas aeruginosa CRPA BSI - model 51	41.00	47.77	56.65	296.50	414.31	553.49	342.36	461.21	608.19	6.75	8.86	11.15	0.57	0.66	0.79	4.12	5.75	7.69	4.75	6.40	8.44	46.06	52.04	59.18	0.64	0.72	0.82	8.75	12.00	15.84	0.12	0.17	0.22
Bulgaria - Pseudomonas aeruginosa CRPA OTH - model 55	0.07	0.10	0.14	0.00	0.00	0.00	0.07	0.10	0.14	2.74E-03	3.63E-03	4.63E-03	9.26E-04	1.33E-03	1.90E-03	0.00	0.00	0.00	9.26E-04	1.33E-03	1.90E-03	19.60	26.57	32.67	0.27	0.37	0.45	0.00	0.00	0.00	0.00	0.00	0.00
Bulgaria - Pseudomonas aeruginosa CRPA RESP - model 52	98.68	122.34	147.60	295.65	430.61	577.18	417.52	551.61	714.61	2.29	2.89	3.51	1.37	1.70	2.05	4.10	5.98	8.01	5.80	7.66	9.92	160.77	193.28	227.14	2.23	2.68	3.15	9.00	12.63	16.45	0.12	0.18	0.23
Bulgaria - Pseudomonas aeruginosa CRPA SSI - model 54	0.05	0.08	0.12	11.53	14.95	18.35	11.60	15.03	18.46	0.38	0.42	0.46	6.73E-04	1.06E-03	1.60E-03	0.16	0.21	0.25	0.16	0.21	0.26	27.45	36.15	44.32	0.38	0.50	0.62	0.40	0.53	0.66	5.50E-03	7.39E-03	9.22E-03
Bulgaria - Pseudomonas aeruginosa CRPA UTI - model 53	0.10	0.14	0.19	0.00	0.00	0.00	0.10	0.14	0.19	1.57E-03	2.05E-03	2.61E-03	1.41E-03	1.89E-03	2.59E-03	0.00	0.00	0.00	1.41E-03	1.89E-03	2.59E-03	52.61	67.11	82.02	0.73	0.93	1.14	0.00	0.00	0.00	0.00	0.00	0.00
Bulgaria - Pseudomonas aeruginosa MDRPA BSI - model 46	3.58	5.06	6.99	18.59	39.40	69.72	23.29	44.76	75.42	3.32	6.03	9.33	0.05	0.07	0.10	0.26	0.55	0.97	0.32	0.62	1.05	5.55	7.43	9.49	0.08	0.10	0.13	0.76	1.53	2.56	0.01	0.02	0.04
Bulgaria - Pseudomonas aeruginosa MDRPA OTH - model 50	5.46E-03	0.01	0.03	0.00	0.00	0.00	5.46E-03	0.01	0.03	1.97E-03	3.79E-03	5.70E-03	7.57E-05	1.94E-04	3.86E-04	0.00	0.00	0.00	7.57E-05	1.94E-04	3.86E-04	1.94	3.80	5.72	0.03	0.05	0.08	0.00	0.00	0.00	0.00	0.00	0.00
Bulgaria - Pseudomonas aeruginosa MDRPA RESP - model 47	8.03	13.16	18.40	22.09	43.38	74.58	32.29	56.50	90.98	1.39	2.08	2.91	0.11	0.18	0.26	0.31	0.60	1.04	0.45	0.78	1.26	18.27	27.46	37.62	0.25	0.38	0.52	0.86	1.70	2.78	0.01	0.02	0.04
Bulgaria - Pseudomonas aeruginosa MDRPA SSI - model 49	3.16E-03	0.01	0.02	1.13	2.33	3.61	1.14	2.34	3.63	0.38	0.46	0.54	4.39E-05	1.41E-04	3.12E-04	0.02	0.03	0.05	0.02	0.03	0.05	2.48	5.12	7.93	0.03	0.07	0.11	0.05	0.11	0.18	6.93E-04	1.58E-03	2.55E-03
Bulgaria - Pseudomonas aeruginosa MDRPA UTI - model 48	8.16E-03	0.02	0.04	0.00	0.00	0.00	8.16E-03	0.02	0.04	1.03E-03	2.03E-03	3.23E-03	1.13E-04	2.69E-04	4.91E-04	0.00	0.00	0.00	1.13E-04	2.69E-04	4.91E-04	5.12	9.66	13.93	0.07	0.13	0.19	0.00	0.00	0.00	0.00	0.00	0.00
Bulgaria - Staphylococcus aureus MRSA BSI - model 61	85.33	93.28	103.30	594.41	631.90	673.50	682.97	725.20	774.40	6.77	7.07	7.37	1.18	1.30	1.43	8.25	8.77	9.35	9.48	10.07	10.75	97.73	102.75	107.79	1.36	1.43	1.50	17.40	18.46	19.59	0.24	0.26	0.27
Bulgaria - Staphylococcus aureus MRSA OTH - model 65	0.40	0.49	0.59	0.00	0.00	0.00	0.40	0.49	0.59	2.67E-03	3.26E-03	3.83E-03	5.58E-03	6.86E-03	8.22E-03	0.00	0.00	0.00	5.58E-03	6.86E-03	8.22E-03	139.54	151.56	165.81	1.94	2.10	2.30	0.00	0.00	0.00	0.00	0.00	0.00
Bulgaria - Staphylococcus aureus MRSA RESP - model 62	86.43	97.23	109.73	170.00	190.34	213.55	257.17	287.56	319.69	1.76	1.86	1.97	1.20	1.35	1.52	2.36	2.64	2.97	3.57	3.99	4.44	141.49	154.51	168.75	1.96	2.15	2.34	5.00	5.56	6.14	0.07	0.08	0.09
Bulgaria - Staphylococcus aureus MRSA SSI - model 64	0.28	0.36	0.46	67.24	74.66	81.80	67.57	75.01	82.11	0.43	0.44	0.46	3.87E-03	5.00E-03	6.36E-03	0.93	1.04	1.14	0.94	1.04	1.14	153.93	168.67	183.01	2.14	2.34	2.54	2.59	2.93	3.25	0.04	0.04	0.05
Bulgaria - Staphylococcus aureus MRSA UTI - model 63	0.04	0.05	0.06	0.00	0.00	0.00	0.04	0.05	0.06	1.51E-03	1.88E-03	2.26E-03	5.04E-04	6.60E-04	8.35E-04	0.00	0.00	0.00	5.04E-04	6.60E-04	8.35E-04	21.42	25.28	29.42	0.30	0.35	0.41	0.00	0.00	0.00	0.00	0.00	0.00
Bulgaria - Streptococcus pneumoniae PMRSP BSI - model 76	15.23	17.88	21.06	113.51	122.52	132.97	130.55	140.48	151.33	9.49	10.18	10.96	0.21	0.25	0.29	1.58	1.70	1.85	1.81	1.95	2.10	13.62	13.79	13.96	0.19	0.19	0.19	2.30	2.48	2.67	0.03	0.03	0.04
Bulgaria - Streptococcus pneumoniae PMRSP OTH - model 80	6.34E-03	9.63E-03	0.01	0.00	0.00	0.00	6.34E-03	9.63E-03	0.01	1.29E-03	1.95E-03	2.75E-03	8.80E-05	1.34E-04	1.94E-04	0.00	0.00	0.00	8.80E-05	1.34E-04	1.94E-04	4.28	4.93	5.68	0.06	0.07	0.08	0.00	0.00	0.00	0.00	0.00	0.00
Bulgaria - Streptococcus pneumoniae PMRSP RESP - model 77	36.58	47.46	63.84	72.85	92.78	123.59	111.27	140.49	184.98	2.35	2.67	3.06	0.51	0.66	0.89	1.01	1.29	1.72	1.54	1.95	2.57	42.70	52.45	66.22	0.59	0.73	0.92	1.51	1.88	2.43	0.02	0.03	0.03
Bulgaria - Streptococcus pneumoniae PMRSP SSI - model 79	0.00	0.00	0.00	0.00	0.00	0.00	0.00	0.00	0.00	NaN	NaN	NaN	0.00	0.00	0.00	0.00	0.00	0.00	0.00	0.00	0.00	0.00	0.00	0.00	0.00	0.00	0.00	0.00	0.00	0.00	0.00	0.00	0.00
Bulgaria - Streptococcus pneumoniae PMRSP UTI - model 78	0.00	0.00	0.00	0.00	0.00	0.00	0.00	0.00	0.00	NaN	NaN	NaN	0.00	0.00	0.00	0.00	0.00	0.00	0.00	0.00	0.00	0.00	0.00	0.00	0.00	0.00	0.00	0.00	0.00	0.00	0.00	0.00	0.00
Bulgaria - Streptococcus pneumoniae PRSP BSI - model 71	13.30	15.79	18.50	99.91	108.04	117.54	115.10	123.82	133.23	8.36	8.97	9.64	0.18	0.22	0.26	1.39	1.50	1.63	1.60	1.72	1.85	13.60	13.80	13.98	0.19	0.19	0.19	2.32	2.48	2.68	0.03	0.03	0.04
Bulgaria - Streptococcus pneumoniae PRSP OTH - model 75	6.09E-03	9.48E-03	0.01	0.00	0.00	0.00	6.09E-03	9.48E-03	0.01	1.26E-03	1.93E-03	2.76E-03	8.45E-05	1.32E-04	2.01E-04	0.00	0.00	0.00	8.45E-05	1.32E-04	2.01E-04	4.30	4.92	5.74	0.06	0.07	0.08	0.00	0.00	0.00	0.00	0.00	0.00
Bulgaria - Streptococcus pneumoniae PRSP RESP - model 72	32.36	41.78	57.45	63.89	81.93	113.49	97.15	123.69	167.93	2.05	2.38	2.75	0.45	0.58	0.80	0.89	1.14	1.58	1.35	1.72	2.33	42.57	52.54	66.89	0.59	0.73	0.93	1.47	1.88	2.47	0.02	0.03	0.03
Bulgaria - Streptococcus pneumoniae PRSP SSI - model 74	0.00	0.00	0.00	0.00	0.00	0.00	0.00	0.00	0.00	NaN	NaN	NaN	0.00	0.00	0.00	0.00	0.00	0.00	0.00	0.00	0.00	0.00	0.00	0.00	0.00	0.00	0.00	0.00	0.00	0.00	0.00	0.00	0.00
Bulgaria - Streptococcus pneumoniae PRSP UTI - model 73	0.00	0.00	0.00	0.00	0.00	0.00	0.00	0.00	0.00	NaN	NaN	NaN	0.00	0.00	0.00	0.00	0.00	0.00	0.00	0.00	0.00	0.00	0.00	0.00	0.00	0.00	0.00	0.00	0.00	0.00	0.00	0.00	0.00
**Total (Median & 95% UIs)**	1,566.81	1,817.08	2,108.93	7,809.54	9,340.44	11,112.82	9,457.75	11,160.29	13,111.65		-		21.75	25.23	29.28	108.43	129.69	154.30	131.32	154.96	182.05	4,679.17	5,374.19	6,087.71	64.97	74.62	84.53	235.55	279.72	328.78	3.27	3.88	4.57
**Total (average)**		**1,823.84**			**9,377.19**			**11,201.03**			-			**25.32**			**130.20**			**155.52**			**5,376.59**			**74.65**			**280.14**			**3.89**	

Infection	YLD			YLL			DALY			DALY per Case			YLD per 100,000			YLL per 100,000			DALY per 100,000			Cases per year			Incidence per 100,000			Deaths per year			Mortality per 100,000		
Colistin resistant *Acinetobacter spp.*	3.33	4.65	6.32	13.95	25.01	37.95	17.86	29.74	43.46	8.08	12.43	16.91	0.39	0.55	0.75	1.65	2.95	4.48	2.11	3.51	5.13	5.42	7.04	8.86	0.64	0.83	1.05	0.39	0.60	0.87	0.05	0.07	0.10
Carbapenem resistant *Acinetobacter spp.* (excluding those resistant to colistin)	73.42	89.27	109.01	345.68	479.94	647.03	427.32	569.19	749.74	7.77	10.10	12.74	8.67	10.54	12.87	40.81	56.66	76.39	50.45	67.20	88.52	146.20	166.60	188.04	17.26	19.67	22.20	11.62	14.60	17.71	1.37	1.72	2.09
Aminoglycosides and fluoroquinolones resistant *Acinetobacter* spp. (excluding those resistant to colistin and/or carbapenem)	2.03	3.30	5.00	6.78	17.23	30.52	9.33	20.65	34.95	9.25	17.12	24.83	0.24	0.39	0.59	0.80	2.03	3.60	1.10	2.44	4.13	2.09	3.55	5.15	0.25	0.42	0.61	0.12	0.29	0.52	0.01	0.03	0.06
Vancomycin resistant Enterococci (*E. faecalis* and *E. faecium*)	6.01	8.67	12.43	59.58	83.73	116.31	65.92	92.40	127.49	6.15	7.27	8.84	0.71	1.02	1.47	7.03	9.89	13.73	7.78	10.91	15.05	33.32	48.67	65.24	3.93	5.75	7.70	2.40	3.29	4.40	0.28	0.39	0.52
Colistin resistant *Escherichia coli*	0.00	0.00	0.00	0.00	0.00	0.00	0.00	0.00	0.00	0.00	0.00	0.00	0.00	0.00	0.00	0.00	0.00	0.00	0.00	0.00	0.00	0.00	0.00	0.00	0.00	0.00	0.00	0.00	0.00	0.00	0.00	0.00	0.00
Carbapenem resistant *Escherichia coli* (excluding those resistant to colistin)	0.00	0.00	0.00	0.00	0.00	0.00	0.00	0.00	0.00	0.00	0.00	0.00	0.00	0.00	0.00	0.00	0.00	0.00	0.00	0.00	0.00	0.00	0.00	0.00	0.00	0.00	0.00	0.00	0.00	0.00	0.00	0.00	0.00
Third-generation cephalosporin resistant *Escherichia coli* (excluding those resistant to colistin and/or carbapenem)	22.45	25.99	29.83	133.84	154.30	177.53	157.42	180.19	206.54	4.20	4.66	5.15	2.65	3.07	3.52	15.80	18.22	20.96	18.59	21.27	24.38	268.66	309.49	350.24	31.72	36.54	41.35	8.23	9.51	10.94	0.97	1.12	1.29
Colistin resistant *Klebsiella pneumoniae*	3.59	5.57	7.96	59.80	100.37	155.07	63.49	106.03	162.41	29.51	45.40	61.31	0.42	0.66	0.94	7.06	11.85	18.31	7.50	12.52	19.17	4.29	7.02	9.88	0.51	0.83	1.17	0.91	1.55	2.36	0.11	0.18	0.28
Carbapenem resistant *Klebsiella pneumoniae* (excluding those resistant to colistin)	4.98	6.38	8.08	49.82	67.45	89.34	55.43	73.84	96.62	7.54	9.51	11.63	0.59	0.75	0.95	5.88	7.96	10.55	6.54	8.72	11.41	18.54	24.41	30.67	2.19	2.88	3.62	2.47	3.26	4.22	0.29	0.38	0.50
Third-generation cephalosporin resistant *Klebsiella pneumoniae* (excluding those resistant to colistin and/or carbapenem)	14.06	16.34	18.88	70.37	79.39	88.28	85.19	95.82	106.24	4.60	4.86	5.13	1.66	1.93	2.23	8.31	9.37	10.42	10.06	11.31	12.54	71.66	83.10	94.12	8.46	9.81	11.11	4.04	4.55	5.08	0.48	0.54	0.60
Colistin resistant *Pseudomonas aeruginosa*	1.38	2.55	4.06	4.66	12.84	25.82	6.43	15.39	29.17	4.00	7.93	11.94	0.16	0.30	0.48	0.55	1.52	3.05	0.76	1.82	3.44	4.42	9.16	14.05	0.52	1.08	1.66	0.22	0.61	1.23	0.03	0.07	0.14
Carbapenem resistant *Pseudomonas aeruginosa* (excluding those resistant to colistin)	34.97	43.35	52.71	150.63	220.51	300.92	189.46	263.42	348.20	10.26	14.17	18.06	4.13	5.12	6.22	17.78	26.03	35.53	22.37	31.10	41.11	66.08	82.09	98.44	7.80	9.69	11.62	3.86	5.51	7.37	0.46	0.65	0.87
Pseudomonas aeruginosa resistant to three or more antimicrobial groups (excluding those resistant to colistin and/or carbapenem)	0.00	0.00	0.00	0.00	0.00	0.00	0.00	0.00	0.00	0.00	0.00	0.00	0.00	0.00	0.00	0.00	0.00	0.00	0.00	0.00	0.00	0.00	0.00	0.00	0.00	0.00	0.00	0.00	0.00	0.00	0.00	0.00	0.00
Methicillin resistant *Staphylococcus aureus*	71.25	80.76	90.83	360.50	393.64	430.88	433.94	474.77	518.26	5.20	5.53	5.91	8.41	9.53	10.72	42.56	46.47	50.87	51.23	56.05	61.19	394.24	439.06	483.95	46.55	51.84	57.14	19.30	21.10	23.15	2.28	2.49	2.73
Penicillin and macrolides resistant *Streptococcus pneumoniae* (excluding those only resistant to penicillin)	6.76	8.46	11.17	24.03	27.75	33.00	31.47	36.16	43.85	8.99	9.75	10.45	0.80	1.00	1.32	2.84	3.28	3.90	3.72	4.27	5.18	10.47	12.08	15.17	1.24	1.43	1.79	0.65	0.74	0.88	0.08	0.09	0.10
Penicillin tesistant *Streptococcus pneumoniae* (excluding those resistant to macrolides)	0.00	0.00	0.00	0.00	0.00	0.00	0.00	0.00	0.00	0.00	0.00	0.00	0.00	0.00	0.00	0.00	0.00	0.00	0.00	0.00	0.00	0.00	0.00	0.00	0.00	0.00	0.00	0.00	0.00	0.00	0.00	0.00	0.00
Total	244.25	295.29	356.29	1,279.62	1,662.14	2,132.65	1,543.27	1,957.59	2,466.92				28.84	34.86	42.06	151.08	196.24	251.79	182.20	231.12	291.25	1,025.40	1,192.27	1,363.81	121.06	140.76	161.02	54.20	65.63	78.72	6.40	7.75	9.29

Model	YLD			YLL			DALY			DALY per Case			YLD per 100,000			YLL per 100,000			DALY per 100,000			Cases per year			Incidence per 100,000			Deaths per year			Mortality per 100,000		
Cyprus - Acinetobacter spp. ColRACI BSI - model 41	2.01E+00	2.66E+00	3.49E+00	1.10E+01	2.07E+01	3.19E+01	1.35E+01	2.34E+01	3.48E+01	5.67E+00	9.67E+00	1.38E+01	2.38E-01	3.14E-01	4.12E-01	1.30E+00	2.44E+00	3.76E+00	1.60E+00	2.76E+00	4.11E+00	2.12E+00	2.42E+00	2.81E+00	2.50E-01	2.86E-01	3.32E-01	3.04E-01	4.94E-01	7.32E-01	3.59E-02	5.83E-02	8.64E-02
Cyprus - Acinetobacter spp. ColRACI OTH - model 45	1.43E-04	3.06E-04	6.09E-04	0.00	0.00	0.00	1.43E-04	3.06E-04	6.09E-04	2.26E-03	3.77E-03	5.29E-03	1.69E-05	3.61E-05	7.19E-05	0.00	0.00	0.00	1.69E-05	3.61E-05	7.19E-05	0.04	0.08	0.14	4.90E-03	9.82E-03	0.02	0.00	0.00	0.00	0.00	0.00	0.00
Cyprus - Acinetobacter spp. ColRACI RESP - model 42	1.32	1.99	2.82	2.66	3.91	5.46	4.05	5.93	8.02	1.94	2.25	2.61	0.16	0.23	0.33	0.31	0.46	0.64	0.48	0.70	0.95	2.01	2.63	3.32	0.24	0.31	0.39	0.07	0.09	0.12	8.40E-03	0.01	0.01
Cyprus - Acinetobacter spp. ColRACI SSI - model 44	9.26E-04	1.82E-03	3.51E-03	0.28	0.45	0.61	0.29	0.45	0.62	0.47	0.50	0.53	1.09E-04	2.14E-04	4.15E-04	0.03	0.05	0.07	0.03	0.05	0.07	0.58	0.89	1.22	0.07	0.11	0.14	0.01	0.02	0.02	1.31E-03	1.87E-03	2.49E-03
Cyprus - Acinetobacter spp. ColRACI UTI - model 43	1.14E-03	2.04E-03	3.53E-03	0.00	0.00	0.00	1.14E-03	2.04E-03	3.53E-03	1.29E-03	2.04E-03	3.02E-03	1.34E-04	2.41E-04	4.17E-04	0.00	0.00	0.00	1.34E-04	2.41E-04	4.17E-04	0.67	1.02	1.37	0.08	0.12	0.16	0.00	0.00	0.00	0.00	0.00	0.00
Cyprus - Acinetobacter spp. CRACI BSI - model 36	42.81	50.77	61.57	278.00	394.26	542.08	328.54	444.49	597.81	5.75	7.78	10.13	5.05	5.99	7.27	32.82	46.55	64.00	38.79	52.48	70.58	53.54	57.45	62.16	6.32	6.78	7.34	9.30	11.88	14.56	1.10	1.40	1.72
Cyprus - Acinetobacter spp. CRACI OTH - model 40	5.21E-03	7.69E-03	0.01	0.00	0.00	0.00	5.21E-03	7.69E-03	0.01	2.95E-03	3.74E-03	4.65E-03	6.15E-04	9.08E-04	1.28E-03	0.00	0.00	0.00	6.15E-04	9.08E-04	1.28E-03	1.48	2.05	2.70	0.17	0.24	0.32	0.00	0.00	0.00	0.00	0.00	0.00
Cyprus - Acinetobacter spp. CRACI RESP - model 37	30.54	38.39	47.30	59.34	75.19	92.18	90.40	114.09	139.02	1.57	1.82	2.06	3.61	4.53	5.58	7.01	8.88	10.88	10.67	13.47	16.41	54.31	62.50	70.64	6.41	7.38	8.34	1.93	2.25	2.57	0.23	0.27	0.30
Cyprus - Acinetobacter spp. CRACI SSI - model 39	0.03	0.04	0.06	8.34	10.49	12.77	8.34	10.54	12.84	0.45	0.50	0.54	3.73E-03	5.29E-03	7.50E-03	0.98	1.24	1.51	0.99	1.24	1.52	17.55	21.08	24.88	2.07	2.49	2.94	0.39	0.48	0.58	0.05	0.06	0.07
Cyprus - Acinetobacter spp. CRACI UTI - model 38	0.04	0.05	0.06	0.00	0.00	0.00	0.04	0.05	0.06	1.60E-03	2.05E-03	2.50E-03	4.30E-03	5.63E-03	7.51E-03	0.00	0.00	0.00	4.30E-03	5.63E-03	7.51E-03	19.32	23.52	27.66	2.28	2.78	3.27	0.00	0.00	0.00	0.00	0.00	0.00
Cyprus - Acinetobacter spp. MDRACI BSI - model 31	1.24	1.88	2.80	5.09	14.21	25.91	6.84	16.17	28.34	5.98	13.37	20.64	0.15	0.22	0.33	0.60	1.68	3.06	0.81	1.91	3.35	0.95	1.21	1.58	0.11	0.14	0.19	0.09	0.24	0.44	0.01	0.03	0.05
Cyprus - Acinetobacter spp. MDRACI OTH - model 35	2.03E-05	1.34E-04	4.20E-04	0.00	0.00	0.00	2.03E-05	1.34E-04	4.20E-04	1.54E-03	3.73E-03	6.45E-03	2.39E-06	1.58E-05	4.96E-05	0.00	0.00	0.00	2.39E-06	1.58E-05	4.96E-05	7.96E-03	0.04	0.09	9.40E-04	4.57E-03	0.01	0.00	0.00	0.00	0.00	0.00	0.00
Cyprus - Acinetobacter spp. MDRACI RESP - model 32	0.79	1.41	2.19	1.60	2.78	4.23	2.40	4.24	6.23	2.74	3.20	3.64	0.09	0.17	0.26	0.19	0.33	0.50	0.28	0.50	0.74	0.78	1.34	1.93	0.09	0.16	0.23	0.03	0.05	0.07	3.21E-03	5.56E-03	8.48E-03
Cyprus - Acinetobacter spp. MDRACI SSI - model 34	1.84E-04	8.19E-04	2.46E-03	0.09	0.24	0.38	0.09	0.24	0.38	0.53	0.53	0.53	2.17E-05	9.66E-05	2.90E-04	0.01	0.03	0.05	0.01	0.03	0.05	0.17	0.45	0.72	0.02	0.05	0.09	1.55E-03	4.06E-03	6.50E-03	1.83E-04	4.79E-04	7.67E-04
Cyprus - Acinetobacter spp. MDRACI UTI - model 33	2.74E-04	9.37E-04	2.26E-03	0.00	0.00	0.00	2.74E-04	9.37E-04	2.26E-03	7.80E-04	1.98E-03	3.60E-03	3.23E-05	1.11E-04	2.67E-04	0.00	0.00	0.00	3.23E-05	1.11E-04	2.67E-04	0.18	0.51	0.82	0.02	0.06	0.10	0.00	0.00	0.00	0.00	0.00	0.00
Cyprus - Enterococcus faecalis and E. faecium VRE BSI - model 66	5.97	8.60	12.32	53.35	73.84	102.72	59.65	82.45	113.80	5.63	6.68	8.20	0.71	1.02	1.45	6.30	8.72	12.13	7.04	9.73	13.44	9.10	12.27	16.20	1.07	1.45	1.91	2.08	2.80	3.71	0.25	0.33	0.44
Cyprus - Enterococcus faecalis and E. faecium VRE OTH - model 70	0.01	0.02	0.04	0.00	0.00	0.00	0.01	0.02	0.04	1.07E-03	1.61E-03	2.28E-03	1.42E-03	2.46E-03	4.16E-03	0.00	0.00	0.00	1.42E-03	2.46E-03	4.16E-03	8.82	13.12	17.61	1.04	1.55	2.08	0.00	0.00	0.00	0.00	0.00	0.00
Cyprus - Enterococcus faecalis and E. faecium VRE RESP - model 67	0.00	0.00	0.00	0.00	0.00	0.00	0.00	0.00	0.00	NaN	NaN	NaN	0.00	0.00	0.00	0.00	0.00	0.00	0.00	0.00	0.00	0.00	0.00	0.00	0.00	0.00	0.00	0.00	0.00	0.00	0.00	0.00	0.00
Cyprus - Enterococcus faecalis and E. faecium VRE SSI - model 69	0.02	0.04	0.06	6.23	9.89	13.59	6.25	9.92	13.64	0.52	0.58	0.64	2.20E-03	4.14E-03	7.15E-03	0.74	1.17	1.60	0.74	1.17	1.61	11.30	16.98	22.94	1.33	2.00	2.71	0.31	0.49	0.68	0.04	0.06	0.08
Cyprus - Enterococcus faecalis and E. faecium VRE UTI - model 68	6.71E-03	0.01	0.02	0.00	0.00	0.00	6.71E-03	0.01	0.02	1.27E-03	1.86E-03	2.58E-03	7.92E-04	1.37E-03	2.16E-03	0.00	0.00	0.00	7.92E-04	1.37E-03	2.16E-03	4.10	6.29	8.48	0.48	0.74	1.00	0.00	0.00	0.00	0.00	0.00	0.00
Cyprus - Escherichia coli 3GCREC BSI - model 16	17.23	19.49	21.97	109.20	124.71	142.48	127.74	144.12	163.72	3.02	3.35	3.68	2.03	2.30	2.59	12.89	14.72	16.82	15.08	17.01	19.33	39.76	43.28	47.13	4.69	5.11	5.56	6.55	7.47	8.53	0.77	0.88	1.01
Cyprus - Escherichia coli 3GCREC OTH - model 20	0.03	0.05	0.06	0.00	0.00	0.00	0.03	0.05	0.06	2.69E-03	3.33E-03	4.17E-03	4.05E-03	5.72E-03	7.61E-03	0.00	0.00	0.00	4.05E-03	5.72E-03	7.61E-03	11.66	14.52	17.29	1.38	1.71	2.04	0.00	0.00	0.00	0.00	0.00	0.00
Cyprus - Escherichia coli 3GCREC RESP - model 17	4.84	5.99	7.20	9.43	11.55	14.05	14.06	17.52	21.23	0.81	0.91	1.04	0.57	0.71	0.85	1.11	1.36	1.66	1.66	2.07	2.51	15.17	19.22	22.99	1.79	2.27	2.71	0.55	0.69	0.83	0.07	0.08	0.10
Cyprus - Escherichia coli 3GCREC SSI - model 19	0.07	0.10	0.14	15.21	18.04	21.00	15.30	18.14	21.07	0.37	0.40	0.42	7.78E-03	0.01	0.02	1.80	2.13	2.48	1.81	2.14	2.49	39.07	45.75	52.45	4.61	5.40	6.19	1.12	1.34	1.58	0.13	0.16	0.19
Cyprus - Escherichia coli 3GCREC UTI - model 18	0.29	0.36	0.46	0.00	0.00	0.00	0.29	0.36	0.46	1.57E-03	1.96E-03	2.37E-03	0.03	0.04	0.05	0.00	0.00	0.00	0.03	0.04	0.05	163.01	186.72	210.38	19.25	22.04	24.84	0.00	0.00	0.00	0.00	0.00	0.00
Cyprus - Escherichia coli ColREC BSI - model 26	0.00	0.00	0.00	0.00	0.00	0.00	0.00	0.00	0.00	NaN	NaN	NaN	0.00	0.00	0.00	0.00	0.00	0.00	0.00	0.00	0.00	0.00	0.00	0.00	0.00	0.00	0.00	0.00	0.00	0.00	0.00	0.00	0.00
Cyprus - Escherichia coli ColREC OTH - model 30	0.00	0.00	0.00	0.00	0.00	0.00	0.00	0.00	0.00	NaN	NaN	NaN	0.00	0.00	0.00	0.00	0.00	0.00	0.00	0.00	0.00	0.00	0.00	0.00	0.00	0.00	0.00	0.00	0.00	0.00	0.00	0.00	0.00
Cyprus - Escherichia coli ColREC RESP - model 27	0.00	0.00	0.00	0.00	0.00	0.00	0.00	0.00	0.00	NaN	NaN	NaN	0.00	0.00	0.00	0.00	0.00	0.00	0.00	0.00	0.00	0.00	0.00	0.00	0.00	0.00	0.00	0.00	0.00	0.00	0.00	0.00	0.00
Cyprus - Escherichia coli ColREC SSI - model 29	0.00	0.00	0.00	0.00	0.00	0.00	0.00	0.00	0.00	NaN	NaN	NaN	0.00	0.00	0.00	0.00	0.00	0.00	0.00	0.00	0.00	0.00	0.00	0.00	0.00	0.00	0.00	0.00	0.00	0.00	0.00	0.00	0.00
Cyprus - Escherichia coli ColREC UTI - model 28	0.00	0.00	0.00	0.00	0.00	0.00	0.00	0.00	0.00	NaN	NaN	NaN	0.00	0.00	0.00	0.00	0.00	0.00	0.00	0.00	0.00	0.00	0.00	0.00	0.00	0.00	0.00	0.00	0.00	0.00	0.00	0.00	0.00
Cyprus - Escherichia coli CREC BSI - model 21	0.00	0.00	0.00	0.00	0.00	0.00	0.00	0.00	0.00	NaN	NaN	NaN	0.00	0.00	0.00	0.00	0.00	0.00	0.00	0.00	0.00	0.00	0.00	0.00	0.00	0.00	0.00	0.00	0.00	0.00	0.00	0.00	0.00
Cyprus - Escherichia coli CREC OTH - model 25	0.00	0.00	0.00	0.00	0.00	0.00	0.00	0.00	0.00	NaN	NaN	NaN	0.00	0.00	0.00	0.00	0.00	0.00	0.00	0.00	0.00	0.00	0.00	0.00	0.00	0.00	0.00	0.00	0.00	0.00	0.00	0.00	0.00
Cyprus - Escherichia coli CREC RESP - model 22	0.00	0.00	0.00	0.00	0.00	0.00	0.00	0.00	0.00	NaN	NaN	NaN	0.00	0.00	0.00	0.00	0.00	0.00	0.00	0.00	0.00	0.00	0.00	0.00	0.00	0.00	0.00	0.00	0.00	0.00	0.00	0.00	0.00
Cyprus - Escherichia coli CREC SSI - model 24	0.00	0.00	0.00	0.00	0.00	0.00	0.00	0.00	0.00	NaN	NaN	NaN	0.00	0.00	0.00	0.00	0.00	0.00	0.00	0.00	0.00	0.00	0.00	0.00	0.00	0.00	0.00	0.00	0.00	0.00	0.00	0.00	0.00
Cyprus - Escherichia coli CREC UTI - model 23	0.00	0.00	0.00	0.00	0.00	0.00	0.00	0.00	0.00	NaN	NaN	NaN	0.00	0.00	0.00	0.00	0.00	0.00	0.00	0.00	0.00	0.00	0.00	0.00	0.00	0.00	0.00	0.00	0.00	0.00	0.00	0.00	0.00
Cyprus - Klebsiella pneumoniae 3GCRKP BSI - model 1	8.84	10.11	11.55	57.15	63.30	69.41	66.60	73.47	80.19	3.23	3.39	3.57	1.04	1.19	1.36	6.75	7.47	8.19	7.86	8.67	9.47	19.94	21.64	23.31	2.35	2.56	2.75	3.26	3.61	3.97	0.39	0.43	0.47
Cyprus - Klebsiella pneumoniae 3GCRKP OTH - model 5	0.02	0.03	0.04	0.00	0.00	0.00	0.02	0.03	0.04	2.46E-03	3.36E-03	4.35E-03	2.38E-03	3.37E-03	4.72E-03	0.00	0.00	0.00	2.38E-03	3.37E-03	4.72E-03	6.91	8.58	10.01	0.82	1.01	1.18	0.00	0.00	0.00	0.00	0.00	0.00
Cyprus - Klebsiella pneumoniae 3GCRKP RESP - model 2	5.15	6.14	7.20	9.89	11.86	13.81	15.20	18.02	20.84	0.90	0.96	1.03	0.61	0.72	0.85	1.17	1.40	1.63	1.79	2.13	2.46	15.94	18.73	21.62	1.88	2.21	2.55	0.57	0.67	0.79	0.07	0.08	0.09
Cyprus - Klebsiella pneumoniae 3GCRKP SSI - model 4	0.01	0.02	0.03	3.33	4.23	5.07	3.34	4.25	5.10	0.47	0.50	0.52	1.41E-03	2.14E-03	3.09E-03	0.39	0.50	0.60	0.39	0.50	0.60	6.89	8.58	10.07	0.81	1.01	1.19	0.21	0.27	0.32	0.02	0.03	0.04
Cyprus - Klebsiella pneumoniae 3GCRKP UTI - model 3	0.04	0.05	0.07	0.00	0.00	0.00	0.04	0.05	0.07	1.43E-03	1.98E-03	2.48E-03	4.28E-03	5.89E-03	7.77E-03	0.00	0.00	0.00	4.28E-03	5.89E-03	7.77E-03	21.99	25.56	29.12	2.60	3.02	3.44	0.00	0.00	0.00	0.00	0.00	0.00
Cyprus - Klebsiella pneumoniae ColRKP BSI - model 11	3.05	4.32	5.94	58.60	97.45	150.50	61.77	101.85	155.91	26.31	41.30	56.27	0.36	0.51	0.70	6.92	11.51	17.77	7.29	12.02	18.41	1.96	2.50	3.11	0.23	0.29	0.37	0.89	1.51	2.29	0.11	0.18	0.27
Cyprus - Klebsiella pneumoniae ColRKP OTH - model 15	1.52E-03	3.92E-03	7.93E-03	0.00	0.00	0.00	1.52E-03	3.92E-03	7.93E-03	1.55E-03	3.41E-03	6.32E-03	1.79E-04	4.63E-04	9.36E-04	0.00	0.00	0.00	1.79E-04	4.63E-04	9.36E-04	0.60	1.17	1.75	0.07	0.14	0.21	0.00	0.00	0.00	0.00	0.00	0.00
Cyprus - Klebsiella pneumoniae ColRKP RESP - model 12	0.53	1.25	2.01	1.00	2.48	3.87	1.52	3.75	5.78	2.73	3.50	4.33	0.06	0.15	0.24	0.12	0.29	0.46	0.18	0.44	0.68	0.51	1.07	1.59	0.06	0.13	0.19	0.02	0.04	0.06	2.09E-03	4.56E-03	6.94E-03
Cyprus - Klebsiella pneumoniae ColRKP SSI - model 14	4.88E-04	1.47E-03	3.36E-03	0.19	0.43	0.70	0.20	0.43	0.70	0.47	0.59	0.70	5.76E-05	1.73E-04	3.97E-04	0.02	0.05	0.08	0.02	0.05	0.08	0.36	0.74	1.18	0.04	0.09	0.14	3.28E-03	6.67E-03	0.01	3.87E-04	7.88E-04	1.25E-03
Cyprus - Klebsiella pneumoniae ColRKP UTI - model 13	1.17E-03	3.02E-03	5.80E-03	0.00	0.00	0.00	1.17E-03	3.02E-03	5.80E-03	1.02E-03	2.02E-03	3.29E-03	1.38E-04	3.56E-04	6.85E-04	0.00	0.00	0.00	1.38E-04	3.56E-04	6.85E-04	0.86	1.55	2.25	0.10	0.18	0.27	0.00	0.00	0.00	0.00	0.00	0.00
Cyprus - Klebsiella pneumoniae CRKP BSI - model 6	4.01	4.92	6.01	47.22	63.54	83.98	51.82	68.44	89.25	6.17	7.94	9.83	0.47	0.58	0.71	5.57	7.50	9.91	6.12	8.08	10.54	7.52	8.69	10.05	0.89	1.03	1.19	2.34	3.06	3.95	0.28	0.36	0.47
Cyprus - Klebsiella pneumoniae CRKP OTH - model 10	8.48E-03	0.01	0.02	0.00	0.00	0.00	8.48E-03	0.01	0.02	2.24E-03	3.46E-03	5.04E-03	1.00E-03	1.64E-03	2.80E-03	0.00	0.00	0.00	1.00E-03	1.64E-03	2.80E-03	2.81	4.08	5.38	0.33	0.48	0.64	0.00	0.00	0.00	0.00	0.00	0.00
Cyprus - Klebsiella pneumoniae CRKP RESP - model 7	0.95	1.44	2.02	1.86	2.78	3.84	2.84	4.24	5.80	0.99	1.13	1.27	0.11	0.17	0.24	0.22	0.33	0.45	0.34	0.50	0.69	2.56	3.75	5.00	0.30	0.44	0.59	0.09	0.13	0.18	0.01	0.02	0.02
Cyprus - Klebsiella pneumoniae CRKP SSI - model 9	2.87E-03	5.36E-03	8.94E-03	0.74	1.13	1.52	0.75	1.13	1.52	0.37	0.44	0.53	3.38E-04	6.33E-04	1.06E-03	0.09	0.13	0.18	0.09	0.13	0.18	1.75	2.57	3.42	0.21	0.30	0.40	0.04	0.06	0.09	4.95E-03	7.41E-03	0.01
Cyprus - Klebsiella pneumoniae CRKP UTI - model 8	6.49E-03	0.01	0.02	0.00	0.00	0.00	6.49E-03	0.01	0.02	1.38E-03	2.07E-03	2.93E-03	7.66E-04	1.29E-03	2.04E-03	0.00	0.00	0.00	7.66E-04	1.29E-03	2.04E-03	3.90	5.32	6.83	0.46	0.63	0.81	0.00	0.00	0.00	0.00	0.00	0.00
Cyprus - Pseudomonas aeruginosa ColRPA BSI - model 56	0.46	0.71	1.11	1.98	5.98	12.01	2.55	6.69	12.82	2.23	5.36	8.59	0.05	0.08	0.13	0.23	0.71	1.42	0.30	0.79	1.51	0.89	1.25	1.79	0.11	0.15	0.21	0.09	0.28	0.57	0.01	0.03	0.07
Cyprus - Pseudomonas aeruginosa ColRPA OTH - model 60	5.60E-04	2.20E-03	5.34E-03	0.00	0.00	0.00	5.60E-04	2.20E-03	5.34E-03	1.52E-03	3.59E-03	6.40E-03	6.61E-05	2.59E-04	6.31E-04	0.00	0.00	0.00	6.61E-05	2.59E-04	6.31E-04	0.22	0.65	1.09	0.03	0.08	0.13	0.00	0.00	0.00	0.00	0.00	0.00
Cyprus - Pseudomonas aeruginosa ColRPA RESP - model 57	0.92	1.83	2.93	2.45	6.20	12.69	3.64	8.04	15.21	1.01	1.80	2.58	0.11	0.22	0.35	0.29	0.73	1.50	0.43	0.95	1.80	2.39	4.77	7.02	0.28	0.56	0.83	0.12	0.29	0.60	0.01	0.03	0.07
Cyprus - Pseudomonas aeruginosa ColRPA SSI - model 59	3.68E-04	1.68E-03	4.80E-03	0.23	0.66	1.12	0.23	0.66	1.13	0.76	0.76	0.76	4.34E-05	1.98E-04	5.66E-04	0.03	0.08	0.13	0.03	0.08	0.13	0.31	0.87	1.48	0.04	0.10	0.17	0.01	0.03	0.05	1.31E-03	3.71E-03	6.29E-03
Cyprus - Pseudomonas aeruginosa ColRPA UTI - model 58	8.50E-04	2.95E-03	7.62E-03	0.00	0.00	0.00	8.50E-04	2.95E-03	7.62E-03	8.36E-04	1.98E-03	3.72E-03	1.00E-04	3.48E-04	9.00E-04	0.00	0.00	0.00	1.00E-04	3.48E-04	9.00E-04	0.61	1.62	2.67	0.07	0.19	0.32	0.00	0.00	0.00	0.00	0.00	0.00
Cyprus - Pseudomonas aeruginosa CRPA BSI - model 51	10.11	12.16	14.52	71.38	104.40	144.41	82.53	116.20	157.03	7.20	10.30	13.34	1.19	1.44	1.71	8.43	12.33	17.05	9.74	13.72	18.54	9.90	11.39	13.06	1.17	1.35	1.54	1.82	2.60	3.49	0.22	0.31	0.41
Cyprus - Pseudomonas aeruginosa CRPA OTH - model 55	0.01	0.02	0.03	0.00	0.00	0.00	0.01	0.02	0.03	2.73E-03	3.65E-03	4.72E-03	1.64E-03	2.48E-03	3.59E-03	0.00	0.00	0.00	1.64E-03	2.48E-03	3.59E-03	4.27	5.84	7.25	0.50	0.69	0.86	0.00	0.00	0.00	0.00	0.00	0.00
Cyprus - Pseudomonas aeruginosa CRPA RESP - model 52	24.82	31.12	38.09	76.49	112.39	151.75	104.18	143.44	186.31	2.63	3.39	4.18	2.93	3.67	4.50	9.03	13.27	17.92	12.30	16.93	22.00	34.65	42.33	50.08	4.09	5.00	5.91	1.95	2.79	3.73	0.23	0.33	0.44
Cyprus - Pseudomonas aeruginosa CRPA SSI - model 54	0.01	0.02	0.03	2.76	3.72	4.76	2.73	3.73	4.78	0.43	0.48	0.53	1.19E-03	1.93E-03	2.96E-03	0.33	0.44	0.56	0.32	0.44	0.56	5.87	7.85	9.98	0.69	0.93	1.18	0.08	0.12	0.16	9.71E-03	0.01	0.02
Cyprus - Pseudomonas aeruginosa CRPA UTI - model 53	0.02	0.03	0.04	0.00	0.00	0.00	0.02	0.03	0.04	1.52E-03	2.03E-03	2.68E-03	2.35E-03	3.50E-03	5.02E-03	0.00	0.00	0.00	2.35E-03	3.50E-03	5.02E-03	11.39	14.67	18.07	1.34	1.73	2.13	0.00	0.00	0.00	0.00	0.00	0.00
Cyprus - Pseudomonas aeruginosa MDRPA BSI - model 46	0.00	0.00	0.00	0.00	0.00	0.00	0.00	0.00	0.00	NaN	NaN	NaN	0.00	0.00	0.00	0.00	0.00	0.00	0.00	0.00	0.00	0.00	0.00	0.00	0.00	0.00	0.00	0.00	0.00	0.00	0.00	0.00	0.00
Cyprus - Pseudomonas aeruginosa MDRPA OTH - model 50	0.00	0.00	0.00	0.00	0.00	0.00	0.00	0.00	0.00	NaN	NaN	NaN	0.00	0.00	0.00	0.00	0.00	0.00	0.00	0.00	0.00	0.00	0.00	0.00	0.00	0.00	0.00	0.00	0.00	0.00	0.00	0.00	0.00
Cyprus - Pseudomonas aeruginosa MDRPA RESP - model 47	0.00	0.00	0.00	0.00	0.00	0.00	0.00	0.00	0.00	NaN	NaN	NaN	0.00	0.00	0.00	0.00	0.00	0.00	0.00	0.00	0.00	0.00	0.00	0.00	0.00	0.00	0.00	0.00	0.00	0.00	0.00	0.00	0.00
Cyprus - Pseudomonas aeruginosa MDRPA SSI - model 49	0.00	0.00	0.00	0.00	0.00	0.00	0.00	0.00	0.00	NaN	NaN	NaN	0.00	0.00	0.00	0.00	0.00	0.00	0.00	0.00	0.00	0.00	0.00	0.00	0.00	0.00	0.00	0.00	0.00	0.00	0.00	0.00	0.00
Cyprus - Pseudomonas aeruginosa MDRPA UTI - model 48	0.00	0.00	0.00	0.00	0.00	0.00	0.00	0.00	0.00	NaN	NaN	NaN	0.00	0.00	0.00	0.00	0.00	0.00	0.00	0.00	0.00	0.00	0.00	0.00	0.00	0.00	0.00	0.00	0.00	0.00	0.00	0.00	0.00
Cyprus - Staphylococcus aureus MRSA BSI - model 61	35.28	39.16	43.56	244.10	262.58	283.78	280.97	301.98	324.37	3.80	4.04	4.29	4.17	4.62	5.14	28.82	31.00	33.50	33.17	35.65	38.30	70.45	74.73	79.59	8.32	8.82	9.40	12.50	13.43	14.53	1.48	1.59	1.72
Cyprus - Staphylococcus aureus MRSA OTH - model 65	0.28	0.36	0.46	0.00	0.00	0.00	0.28	0.36	0.46	2.56E-03	3.25E-03	4.09E-03	0.03	0.04	0.05	0.00	0.00	0.00	0.03	0.04	0.05	98.59	110.27	121.80	11.64	13.02	14.38	0.00	0.00	0.00	0.00	0.00	0.00
Cyprus - Staphylococcus aureus MRSA RESP - model 62	35.48	40.94	46.43	69.49	79.13	89.56	105.50	120.19	135.51	0.98	1.07	1.17	4.19	4.83	5.48	8.20	9.34	10.57	12.46	14.19	16.00	100.20	112.76	124.98	11.83	13.31	14.76	3.59	4.05	4.58	0.42	0.48	0.54
Cyprus - Staphylococcus aureus MRSA SSI - model 64	0.19	0.26	0.35	46.91	51.92	57.54	47.17	52.21	57.88	0.41	0.43	0.44	0.02	0.03	0.04	5.54	6.13	6.79	5.57	6.16	6.83	110.03	122.81	135.62	12.99	14.50	16.01	3.20	3.62	4.04	0.38	0.43	0.48
Cyprus - Staphylococcus aureus MRSA UTI - model 63	0.03	0.03	0.05	0.00	0.00	0.00	0.03	0.03	0.05	1.47E-03	1.85E-03	2.30E-03	2.98E-03	4.02E-03	5.47E-03	0.00	0.00	0.00	2.98E-03	4.02E-03	5.47E-03	14.98	18.50	21.97	1.77	2.18	2.59	0.00	0.00	0.00	0.00	0.00	0.00
Cyprus - Streptococcus pneumoniae PMRSP BSI - model 76	1.93	2.29	2.73	14.47	15.73	16.84	16.76	18.04	19.27	7.16	7.71	8.21	0.23	0.27	0.32	1.71	1.86	1.99	1.98	2.13	2.28	2.32	2.34	2.37	0.27	0.28	0.28	0.39	0.42	0.45	0.05	0.05	0.05
Cyprus - Streptococcus pneumoniae PMRSP OTH - model 80	1.05E-03	1.58E-03	2.49E-03	0.00	0.00	0.00	1.05E-03	1.58E-03	2.49E-03	1.28E-03	1.94E-03	2.84E-03	1.24E-04	1.87E-04	2.94E-04	0.00	0.00	0.00	1.24E-04	1.87E-04	2.94E-04	0.73	0.82	0.97	0.09	0.10	0.11	0.00	0.00	0.00	0.00	0.00	0.00
Cyprus - Streptococcus pneumoniae PMRSP RESP - model 77	4.83	6.16	8.45	9.56	12.01	16.16	14.72	18.12	24.58	1.83	2.03	2.24	0.57	0.73	1.00	1.13	1.42	1.91	1.74	2.14	2.90	7.42	8.92	11.83	0.88	1.05	1.40	0.26	0.32	0.43	0.03	0.04	0.05
Cyprus - Streptococcus pneumoniae PMRSP SSI - model 79	0.00	0.00	0.00	0.00	0.00	0.00	0.00	0.00	0.00	NaN	NaN	NaN	0.00	0.00	0.00	0.00	0.00	0.00	0.00	0.00	0.00	0.00	0.00	0.00	0.00	0.00	0.00	0.00	0.00	0.00	0.00	0.00	0.00
Cyprus - Streptococcus pneumoniae PMRSP UTI - model 78	0.00	0.00	0.00	0.00	0.00	0.00	0.00	0.00	0.00	NaN	NaN	NaN	0.00	0.00	0.00	0.00	0.00	0.00	0.00	0.00	0.00	0.00	0.00	0.00	0.00	0.00	0.00	0.00	0.00	0.00	0.00	0.00	0.00
Cyprus - Streptococcus pneumoniae PRSP BSI - model 71	0.00	0.00	0.00	0.00	0.00	0.00	0.00	0.00	0.00	NaN	NaN	NaN	0.00	0.00	0.00	0.00	0.00	0.00	0.00	0.00	0.00	0.00	0.00	0.00	0.00	0.00	0.00	0.00	0.00	0.00	0.00	0.00	0.00
Cyprus - Streptococcus pneumoniae PRSP OTH - model 75	0.00	0.00	0.00	0.00	0.00	0.00	0.00	0.00	0.00	NaN	NaN	NaN	0.00	0.00	0.00	0.00	0.00	0.00	0.00	0.00	0.00	0.00	0.00	0.00	0.00	0.00	0.00	0.00	0.00	0.00	0.00	0.00	0.00
Cyprus - Streptococcus pneumoniae PRSP RESP - model 72	0.00	0.00	0.00	0.00	0.00	0.00	0.00	0.00	0.00	NaN	NaN	NaN	0.00	0.00	0.00	0.00	0.00	0.00	0.00	0.00	0.00	0.00	0.00	0.00	0.00	0.00	0.00	0.00	0.00	0.00	0.00	0.00	0.00
Cyprus - Streptococcus pneumoniae PRSP SSI - model 74	0.00	0.00	0.00	0.00	0.00	0.00	0.00	0.00	0.00	NaN	NaN	NaN	0.00	0.00	0.00	0.00	0.00	0.00	0.00	0.00	0.00	0.00	0.00	0.00	0.00	0.00	0.00	0.00	0.00	0.00	0.00	0.00	0.00
Cyprus - Streptococcus pneumoniae PRSP UTI - model 73	0.00	0.00	0.00	0.00	0.00	0.00	0.00	0.00	0.00	NaN	NaN	NaN	0.00	0.00	0.00	0.00	0.00	0.00	0.00	0.00	0.00	0.00	0.00	0.00	0.00	0.00	0.00	0.00	0.00	0.00	0.00	0.00	0.00
**Total (Median & 95% UIs)**	244.25	295.29	356.29	1,279.62	1,662.14	2,132.65	1,543.27	1,957.59	2,466.92		-		28.84	34.86	42.06	151.08	196.24	251.79	182.20	231.12	291.25	1,025.40	1,192.27	1,363.81	121.06	140.76	161.02	54.20	65.63	78.72	6.40	7.75	9.29
**Total (average)**		**296.65**			**1,673.16**			**1,969.81**			-			**35.02**			**197.54**			**232.56**			**1,193.34**			**140.89**			**65.88**			**7.78**	

Infection	YLD			YLL			DALY			DALY per Case			YLD per 100,000		
Colistin resistant *Acinetobacter spp.*	4.05	5.19	6.51	17.84	27.69	39.42	22.37	32.85	45.18	4.87	6.84	9.02	0.04	0.05	0.06
Carbapenem resistant *Acinetobacter spp.* (excluding those resistant to colistin)	3.53	5.03	6.93	13.42	27.28	43.62	17.72	32.26	49.58	8.09	13.55	19.43	0.03	0.05	0.07
Aminoglycosides and fluoroquinolones resistant *Acinetobacter* spp. (excluding those resistant to colistin and/or carbapenem)	4.65	6.22	8.16	18.64	32.71	48.79	23.96	38.81	56.45	7.16	10.77	14.64	0.04	0.06	0.08
Vancomycin resistant Enterococci (*E. faecalis* and *E. faecium*)	27.15	33.63	41.24	261.10	316.08	379.10	289.58	349.43	418.52	6.08	6.73	7.42	0.26	0.32	0.39
Colistin resistant *Escherichia coli*	0.00	0.00	0.00	0.00	0.00	0.00	0.00	0.00	0.00	0.00	0.00	0.00	0.00	0.00	0.00
Carbapenem resistant *Escherichia coli* (excluding those resistant to colistin)	0.00	0.00	0.00	0.00	0.00	0.00	0.00	0.00	0.00	0.00	0.00	0.00	0.00	0.00	0.00
Third-generation cephalosporin resistant *Escherichia coli* (excluding those resistant to colistin and/or carbapenem)	293.04	330.59	368.99	1,763.72	1,986.27	2,228.42	2,069.13	2,316.46	2,584.17	4.18	4.55	4.93	2.78	3.14	3.50
Colistin resistant *Klebsiella pneumoniae*	12.10	14.28	16.81	202.11	252.90	304.61	215.97	266.96	321.08	12.54	15.35	17.93	0.11	0.14	0.16
Carbapenem resistant *Klebsiella pneumoniae* (excluding those resistant to colistin)	2.87	3.96	5.30	28.70	42.51	59.43	31.50	46.31	64.08	9.94	13.72	17.63	0.03	0.04	0.05
Third-generation cephalosporin resistant *Klebsiella pneumoniae* (excluding those resistant to colistin and/or carbapenem)	782.79	855.44	936.98	3,852.96	4,116.93	4,390.89	4,663.51	4,973.75	5,310.58	5.69	5.93	6.17	7.43	8.12	8.89
Colistin resistant *Pseudomonas aeruginosa*	1.75	2.71	3.94	5.78	13.21	23.71	7.94	15.97	26.95	2.16	4.03	6.21	0.02	0.03	0.04
Carbapenem resistant *Pseudomonas aeruginosa* (excluding those resistant to colistin)	112.98	130.32	149.29	528.00	659.08	815.23	647.22	789.31	958.99	6.58	7.92	9.28	1.07	1.24	1.42
Pseudomonas aeruginosa resistant to three or more antimicrobial groups (excluding those resistant to colistin and/or carbapenem)	103.63	119.60	138.27	450.44	573.88	704.24	558.63	692.44	833.33	5.95	7.20	8.41	0.98	1.13	1.31
Methicillin resistant *Staphylococcus aureus*	296.69	326.50	357.34	1.474.85	1.588.84	1.701.61	1.781.85	1.915.62	2.050.06	5.36	5.60	5.84	2.82	3.10	3.39
Penicillin and macrolides resistant *Streptococcus pneumoniae* (excluding those only resistant to penicillin)	0.00	0.00	0.00	0.00	0.00	0.00	0.00	0.00	0.00	0.00	0.00	0.00	0.00	0.00	0.00
Penicillin tesistant *Streptococcus pneumoniae* (excluding those resistant to macrolides)	0.00	0.00	0.00	0.00	0.00	0.00	0.00	0.00	0.00	0.00	0.00	0.00	0.00	0.00	0.00
Total	1,645.23	1,833.47	2,039.76	8,617.59	9,637.37	10,739.06	10,329.38	11,470.18	12,718.98				15.61	17.40	19.36

Infection	YLL per 100,000			DALY per 100,000			Cases per year			Incidence per 100,000			Deaths per year			Mortality per 100,000		
Colistin resistant *Acinetobacter spp.*	0.17	0.26	0.37	0.21	0.31	0.43	11.04	14.15	17.28	0.10	0.13	0.16	0.82	1.23	1.70	0.01	0.01	0.02
Carbapenem resistant *Acinetobacter spp.* (excluding those resistant to colistin)	0.13	0.26	0.41	0.17	0.31	0.47	5.02	7.03	9.14	0.05	0.07	0.09	0.31	0.61	0.95	0.00	0.01	0.01
Aminoglycosides and fluoroquinolones resistant *Acinetobacter* spp. (excluding those resistant to colistin and/or carbapenem)	0.18	0.31	0.46	0.23	0.37	0.54	8.03	10.59	13.24	0.08	0.10	0.13	0.53	0.89	1.32	0.01	0.01	0.01
Vancomycin resistant Enterococci (*E. faecalis* and *E. faecium*)	2.48	3.00	3.60	2.75	3.32	3.97	160.98	199.47	238.92	1.53	1.89	2.27	11.04	13.29	15.90	0.10	0.13	0.15
Colistin resistant *Escherichia coli*	0.00	0.00	0.00	0.00	0.00	0.00	0.00	0.00	0.00	0.00	0.00	0.00	0.00	0.00	0.00	0.00	0.00	0.00
Carbapenem resistant *Escherichia coli* (excluding those resistant to colistin)	0.00	0.00	0.00	0.00	0.00	0.00	0.00	0.00	0.00	0.00	0.00	0.00	0.00	0.00	0.00	0.00	0.00	0.00
Third-generation cephalosporin resistant *Escherichia coli* (excluding those resistant to colistin and/or carbapenem)	16.74	18.85	21.15	19.63	21.98	24.52	3,574.16	4,042.98	4,526.25	33.92	38.36	42.95	110.71	125.98	143.28	1.05	1.20	1.36
Colistin resistant *Klebsiella pneumoniae*	1.92	2.40	2.89	2.05	2.53	3.05	42.99	51.92	61.42	0.41	0.49	0.58	9.50	11.69	14.20	0.09	0.11	0.13
Carbapenem resistant *Klebsiella pneumoniae* (excluding those resistant to colistin)	0.27	0.40	0.56	0.30	0.44	0.61	7.00	10.38	13.80	0.07	0.10	0.13	0.98	1.39	1.94	0.01	0.01	0.02
Third-generation cephalosporin resistant *Klebsiella pneumoniae* (excluding those resistant to colistin and/or carbapenem)	36.56	39.07	41.67	44.25	47.20	50.39	3,152.21	3,462.27	3,769.55	29.91	32.85	35.77	175.90	188.90	203.09	1.67	1.79	1.93
Colistin resistant *Pseudomonas aeruginosa*	0.05	0.13	0.22	0.08	0.15	0.26	11.56	18.12	25.08	0.11	0.17	0.24	0.58	1.23	2.08	0.01	0.01	0.02
Carbapenem resistant *Pseudomonas aeruginosa* (excluding those resistant to colistin)	5.01	6.25	7.74	6.14	7.49	9.10	381.80	444.03	508.51	3.62	4.21	4.83	24.01	30.31	37.68	0.23	0.29	0.36
Pseudomonas aeruginosa resistant to three or more antimicrobial groups (excluding those resistant to colistin and/or carbapenem)	4.27	5.45	6.68	5.30	6.57	7.91	364.93	425.26	487.28	3.46	4.04	4.62	21.37	27.42	33.89	0.20	0.26	0.32
Methicillin resistant *Staphylococcus aureus*	14.00	15.08	16.15	16.91	18.18	19.45	1.604.67	1.751.85	1.899.12	15.23	16.62	18.02	77.13	83.14	89.47	0.73	0.79	0.85
Penicillin and macrolides resistant *Streptococcus pneumoniae* (excluding those only resistant to penicillin)	0.00	0.00	0.00	0.00	0.00	0.00	0.00	0.00	0.00	0.00	0.00	0.00	0.00	0.00	0.00	0.00	0.00	0.00
Penicillin tesistant *Streptococcus pneumoniae* (excluding those resistant to macrolides)	0.00	0.00	0.00	0.00	0.00	0.00	0.00	0.00	0.00	0.00	0.00	0.00	0.00	0.00	0.00	0.00	0.00	0.00
Total	81.77	91.45	101.91	98.02	108.84	120.69	9,324.39	10,438.06	11,569.58	88.48	99.05	109.79	432.87	486.09	545.49	4.11	4.61	5.18

Model	YLD			YLL			DALY			DALY per Case			YLD per 100,000		
Czech Republic - Acinetobacter spp. ColRACI BSI - model 41	2.37E+00	2.96E+00	3.65E+00	1.41E+01	2.27E+01	3.31E+01	1.70E+01	2.56E+01	3.60E+01	3.44E+00	5.21E+00	7.19E+00	2.25E-02	2.81E-02	3.46E-02
Czech Republic - Acinetobacter spp. ColRACI OTH - model 45	2.65E-04	6.07E-04	1.07E-03	0.00	0.00	0.00	2.65E-04	6.07E-04	1.07E-03	2.40E-03	3.80E-03	5.37E-03	2.52E-06	5.76E-06	1.02E-05
Czech Republic - Acinetobacter spp. ColRACI RESP - model 42	1.68	2.23	2.85	3.24	4.33	5.47	4.90	6.57	8.27	1.08	1.24	1.39	0.02	0.02	0.03
Czech Republic - Acinetobacter spp. ColRACI SSI - model 44	1.90E-03	3.74E-03	6.27E-03	0.45	0.68	0.89	0.46	0.69	0.90	0.34	0.38	0.43	1.81E-05	3.54E-05	5.95E-05
Czech Republic - Acinetobacter spp. ColRACI UTI - model 43	2.24E-03	3.98E-03	6.44E-03	0.00	0.00	0.00	2.24E-03	3.98E-03	6.44E-03	1.32E-03	2.02E-03	2.80E-03	2.12E-05	3.78E-05	6.11E-05
Czech Republic - Acinetobacter spp. CRACI BSI - model 36	2.13	2.87	3.89	10.27	22.39	36.69	12.96	25.20	39.96	5.43	10.41	15.71	0.02	0.03	0.04
Czech Republic - Acinetobacter spp. CRACI OTH - model 40	8.61E-05	2.88E-04	6.77E-04	0.00	0.00	0.00	8.61E-05	2.88E-04	6.77E-04	1.86E-03	3.68E-03	5.78E-03	8.17E-07	2.73E-06	6.43E-06
Czech Republic - Acinetobacter spp. CRACI RESP - model 37	1.40	2.16	3.03	2.81	4.27	6.05	4.42	6.44	8.74	2.00	2.45	2.99	0.01	0.02	0.03
Czech Republic - Acinetobacter spp. CRACI SSI - model 39	6.43E-04	1.84E-03	3.78E-03	0.35	0.61	0.87	0.35	0.61	0.87	0.66	0.69	0.73	6.10E-06	1.75E-05	3.59E-05
Czech Republic - Acinetobacter spp. CRACI UTI - model 38	8.90E-04	1.96E-03	3.71E-03	0.00	0.00	0.00	8.90E-04	1.96E-03	3.71E-03	1.05E-03	2.06E-03	3.22E-03	8.44E-06	1.86E-05	3.52E-05
Czech Republic - Acinetobacter spp. MDRACI BSI - model 31	2.75	3.57	4.53	14.45	26.82	41.08	17.72	30.29	45.20	5.01	8.29	11.77	0.03	0.03	0.04
Czech Republic - Acinetobacter spp. MDRACI OTH - model 35	1.67E-04	4.44E-04	9.27E-04	0.00	0.00	0.00	1.67E-04	4.44E-04	9.27E-04	2.16E-03	3.77E-03	5.54E-03	1.59E-06	4.21E-06	8.79E-06
Czech Republic - Acinetobacter spp. MDRACI RESP - model 32	1.89	2.65	3.62	3.77	5.24	6.84	5.83	7.86	10.38	1.71	1.99	2.33	0.02	0.03	0.03
Czech Republic - Acinetobacter spp. MDRACI SSI - model 34	1.21E-03	2.71E-03	5.36E-03	0.41	0.65	0.87	0.41	0.65	0.87	0.44	0.49	0.53	1.15E-05	2.57E-05	5.09E-05
Czech Republic - Acinetobacter spp. MDRACI UTI - model 33	1.62E-03	2.98E-03	5.19E-03	0.00	0.00	0.00	1.62E-03	2.98E-03	5.19E-03	1.19E-03	2.04E-03	3.07E-03	1.54E-05	2.83E-05	4.92E-05
Czech Republic - Enterococcus faecalis and E. faecium VRE BSI - model 66	26.95	33.35	40.85	238.36	285.71	341.14	266.63	318.82	380.18	5.69	6.28	6.93	0.26	0.32	0.39
Czech Republic - Enterococcus faecalis and E. faecium VRE OTH - model 70	0.06	0.09	0.12	0.00	0.00	0.00	0.06	0.09	0.12	1.27E-03	1.65E-03	2.05E-03	6.08E-04	8.36E-04	1.12E-03
Czech Republic - Enterococcus faecalis and E. faecium VRE RESP - model 67	0.00	0.00	0.00	0.00	0.00	0.00	0.00	0.00	0.00	NaN	NaN	NaN	0.00	0.00	0.00
Czech Republic - Enterococcus faecalis and E. faecium VRE SSI - model 69	0.10	0.15	0.21	22.74	30.36	37.96	22.86	30.47	38.15	0.39	0.44	0.49	9.75E-04	1.40E-03	1.97E-03
Czech Republic - Enterococcus faecalis and E. faecium VRE UTI - model 68	0.03	0.05	0.06	0.00	0.00	0.00	0.03	0.05	0.06	1.47E-03	1.87E-03	2.32E-03	3.21E-04	4.54E-04	6.09E-04
Czech Republic - Escherichia coli 3GCREC BSI - model 16	224.40	248.02	271.19	1,430.27	1,590.57	1,772.91	1,666.91	1,838.16	2,032.72	2.97	3.24	3.50	2.13	2.35	2.57
Czech Republic - Escherichia coli 3GCREC OTH - model 20	0.46	0.63	0.84	0.00	0.00	0.00	0.46	0.63	0.84	2.66E-03	3.34E-03	4.15E-03	4.32E-03	5.94E-03	8.01E-03
Czech Republic - Escherichia coli 3GCREC RESP - model 17	63.56	75.92	89.24	121.34	146.45	171.50	184.80	222.53	259.34	0.82	0.88	0.98	0.60	0.72	0.85
Czech Republic - Escherichia coli 3GCREC SSI - model 19	0.88	1.27	1.73	212.11	249.26	284.01	213.21	250.39	285.27	0.39	0.42	0.44	8.36E-03	0.01	0.02
Czech Republic - Escherichia coli 3GCREC UTI - model 18	3.76	4.75	6.00	0.00	0.00	0.00	3.76	4.75	6.00	1.58E-03	1.95E-03	2.39E-03	0.04	0.05	0.06
Czech Republic - Escherichia coli ColREC BSI - model 26	0.00	0.00	0.00	0.00	0.00	0.00	0.00	0.00	0.00	NaN	NaN	NaN	0.00	0.00	0.00
Czech Republic - Escherichia coli ColREC OTH - model 30	0.00	0.00	0.00	0.00	0.00	0.00	0.00	0.00	0.00	NaN	NaN	NaN	0.00	0.00	0.00
Czech Republic - Escherichia coli ColREC RESP - model 27	0.00	0.00	0.00	0.00	0.00	0.00	0.00	0.00	0.00	NaN	NaN	NaN	0.00	0.00	0.00
Czech Republic - Escherichia coli ColREC SSI - model 29	0.00	0.00	0.00	0.00	0.00	0.00	0.00	0.00	0.00	NaN	NaN	NaN	0.00	0.00	0.00
Czech Republic - Escherichia coli ColREC UTI - model 28	0.00	0.00	0.00	0.00	0.00	0.00	0.00	0.00	0.00	NaN	NaN	NaN	0.00	0.00	0.00
Czech Republic - Escherichia coli CREC BSI - model 21	0.00	0.00	0.00	0.00	0.00	0.00	0.00	0.00	0.00	NaN	NaN	NaN	0.00	0.00	0.00
Czech Republic - Escherichia coli CREC OTH - model 25	0.00	0.00	0.00	0.00	0.00	0.00	0.00	0.00	0.00	NaN	NaN	NaN	0.00	0.00	0.00
Czech Republic - Escherichia coli CREC RESP - model 22	0.00	0.00	0.00	0.00	0.00	0.00	0.00	0.00	0.00	NaN	NaN	NaN	0.00	0.00	0.00
Czech Republic - Escherichia coli CREC SSI - model 24	0.00	0.00	0.00	0.00	0.00	0.00	0.00	0.00	0.00	NaN	NaN	NaN	0.00	0.00	0.00
Czech Republic - Escherichia coli CREC UTI - model 23	0.00	0.00	0.00	0.00	0.00	0.00	0.00	0.00	0.00	NaN	NaN	NaN	0.00	0.00	0.00
Czech Republic - Klebsiella pneumoniae 3GCRKP BSI - model 1	491.60	532.04	579.51	3,162.45	3,342.49	3,526.28	3,679.25	3,875.67	4,090.94	4.12	4.28	4.42	4.66	5.05	5.50
Czech Republic - Klebsiella pneumoniae 3GCRKP RESP - model 2	288.02	319.38	352.44	556.42	616.46	682.55	846.75	936.11	1,032.79	1.15	1.20	1.27	2.73	3.03	3.34
Czech Republic - Klebsiella pneumoniae 3GCRKP UTI - model 3	1.66	2.08	2.53	0.00	0.00	0.00	1.66	2.08	2.53	1.59E-03	1.96E-03	2.34E-03	0.02	0.02	0.02
Czech Republic - Klebsiella pneumoniae 3GCRKP SSI - model 4	0.56	0.75	1.00	134.09	157.98	182.06	134.91	158.70	182.83	0.42	0.45	0.47	5.28E-03	7.10E-03	9.46E-03
Czech Republic - Klebsiella pneumoniae 3GCRKP OTH - model 5	0.95	1.20	1.49	0.00	0.00	0.00	0.95	1.20	1.49	2.72E-03	3.32E-03	3.99E-03	9.03E-03	0.01	0.01
Czech Republic - Klebsiella pneumoniae ColRKP BSI - model 11	9.61	11.04	12.66	195.63	244.55	294.14	206.96	255.38	306.54	11.17	13.75	16.05	0.09	0.10	0.12
Czech Republic - Klebsiella pneumoniae ColRKP OTH - model 15	0.02	0.03	0.04	0.00	0.00	0.00	0.02	0.03	0.04	2.58E-03	3.48E-03	4.65E-03	1.93E-04	2.83E-04	4.13E-04
Czech Republic - Klebsiella pneumoniae ColRKP RESP - model 12	2.44	3.17	4.06	4.77	6.13	7.70	7.25	9.29	11.68	1.00	1.19	1.42	0.02	0.03	0.04
Czech Republic - Klebsiella pneumoniae ColRKP SSI - model 14	7.39E-03	0.01	0.02	1.72	2.23	2.77	1.73	2.24	2.78	0.37	0.41	0.45	7.01E-05	1.11E-04	1.65E-04
Czech Republic - Klebsiella pneumoniae ColRKP UTI - model 13	0.02	0.02	0.03	0.00	0.00	0.00	0.02	0.02	0.03	1.57E-03	2.06E-03	2.64E-03	1.57E-04	2.16E-04	3.11E-04
Czech Republic - Klebsiella pneumoniae CRKP BSI - model 6	2.38	3.05	3.97	27.55	40.31	56.27	29.83	43.19	59.66	8.31	11.65	15.11	0.02	0.03	0.04
Czech Republic - Klebsiella pneumoniae CRKP OTH - model 10	2.60E-03	5.68E-03	0.01	0.00	0.00	0.00	2.60E-03	5.68E-03	0.01	1.98E-03	3.44E-03	5.49E-03	2.47E-05	5.39E-05	1.03E-04
Czech Republic - Klebsiella pneumoniae CRKP RESP - model 7	0.48	0.90	1.30	0.93	1.77	2.52	1.44	2.67	3.77	1.27	1.66	2.07	4.60E-03	8.51E-03	0.01
Czech Republic - Klebsiella pneumoniae CRKP SSI - model 9	7.62E-04	2.22E-03	4.33E-03	0.23	0.43	0.63	0.23	0.43	0.63	0.35	0.39	0.43	7.23E-06	2.11E-05	4.11E-05
Czech Republic - Klebsiella pneumoniae CRKP UTI - model 8	2.24E-03	4.60E-03	8.04E-03	0.00	0.00	0.00	2.24E-03	4.60E-03	8.04E-03	1.16E-03	2.07E-03	3.17E-03	2.13E-05	4.37E-05	7.63E-05
Czech Republic - Pseudomonas aeruginosa ColRPA BSI - model 56	0.54	0.75	1.05	2.52	6.01	10.81	3.23	6.82	11.71	1.32	2.73	4.32	5.08E-03	7.10E-03	9.95E-03
Czech Republic - Pseudomonas aeruginosa ColRPA OTH - model 60	1.79E-03	4.69E-03	9.17E-03	0.00	0.00	0.00	1.79E-03	4.69E-03	9.17E-03	1.90E-03	3.76E-03	5.77E-03	1.70E-05	4.45E-05	8.71E-05
Czech Republic - Pseudomonas aeruginosa ColRPA RESP - model 57	1.21	1.95	2.87	2.98	6.53	11.89	4.42	8.47	14.20	0.56	0.91	1.37	0.01	0.02	0.03
Czech Republic - Pseudomonas aeruginosa ColRPA SSI - model 59	1.10E-03	3.34E-03	7.75E-03	0.29	0.67	1.02	0.29	0.67	1.02	0.27	0.39	0.51	1.04E-05	3.17E-05	7.36E-05
Czech Republic - Pseudomonas aeruginosa ColRPA UTI - model 58	2.86E-03	6.61E-03	0.01	0.00	0.00	0.00	2.86E-03	6.61E-03	0.01	1.06E-03	2.06E-03	3.30E-03	2.72E-05	6.27E-05	1.18E-04
Czech Republic - Pseudomonas aeruginosa CRPA BSI - model 51	32.70	36.60	41.32	249.26	309.98	381.49	284.39	346.99	419.59	4.58	5.61	6.68	0.31	0.35	0.39
Czech Republic - Pseudomonas aeruginosa CRPA OTH - model 55	0.09	0.11	0.15	0.00	0.00	0.00	0.09	0.11	0.15	2.93E-03	3.67E-03	4.43E-03	8.17E-04	1.08E-03	1.39E-03
Czech Republic - Pseudomonas aeruginosa CRPA RESP - model 52	80.01	93.35	107.49	264.16	330.28	410.90	347.96	423.15	516.08	1.59	1.86	2.11	0.76	0.89	1.02
Czech Republic - Pseudomonas aeruginosa CRPA SSI - model 54	0.06	0.09	0.12	14.58	18.82	22.84	14.66	18.90	22.97	0.41	0.44	0.48	6.05E-04	8.56E-04	1.17E-03
Czech Republic - Pseudomonas aeruginosa CRPA UTI - model 53	0.12	0.16	0.21	0.00	0.00	0.00	0.12	0.16	0.21	1.64E-03	2.06E-03	2.46E-03	1.17E-03	1.54E-03	2.00E-03
Czech Republic - Pseudomonas aeruginosa MDRPA BSI - model 46	29.84	33.71	38.13	207.82	265.87	324.85	239.10	299.46	360.36	4.09	5.05	5.98	0.28	0.32	0.36
Czech Republic - Pseudomonas aeruginosa MDRPA OTH - model 50	0.08	0.11	0.15	0.00	0.00	0.00	0.08	0.11	0.15	2.96E-03	3.75E-03	4.59E-03	7.79E-04	1.06E-03	1.42E-03
Czech Republic - Pseudomonas aeruginosa MDRPA RESP - model 47	73.54	85.54	99.66	228.50	290.41	358.25	305.16	375.03	451.37	1.46	1.71	1.96	0.70	0.81	0.95
Czech Republic - Pseudomonas aeruginosa MDRPA SSI - model 49	0.06	0.09	0.12	14.12	17.59	21.14	14.18	17.69	21.25	0.40	0.44	0.47	5.75E-04	8.29E-04	1.16E-03
Czech Republic - Pseudomonas aeruginosa MDRPA UTI - model 48	0.11	0.15	0.20	0.00	0.00	0.00	0.11	0.15	0.20	1.61E-03	2.04E-03	2.50E-03	1.08E-03	1.46E-03	1.91E-03
Czech Republic - Staphylococcus aureus MRSA BSI - model 61	145.99	158.61	172.08	1,002.04	1,063.80	1,128.39	1,153.76	1,223.01	1,292.34	3.93	4.09	4.25	1.39	1.51	1.63
Czech Republic - Staphylococcus aureus MRSA OTH - model 65	1.16	1.44	1.77	0.00	0.00	0.00	1.16	1.44	1.77	2.70E-03	3.26E-03	3.93E-03	0.01	0.01	0.02
Czech Republic - Staphylococcus aureus MRSA RESP - model 62	148.64	165.27	181.98	288.21	320.67	350.04	441.46	485.61	531.52	1.02	1.08	1.14	1.41	1.57	1.73
Czech Republic - Staphylococcus aureus MRSA SSI - model 64	0.80	1.05	1.33	184.60	204.36	223.17	185.37	205.44	224.26	0.40	0.42	0.44	7.58E-03	9.94E-03	0.01
Czech Republic - Staphylococcus aureus MRSA UTI - model 63	0.10	0.13	0.18	0.00	0.00	0.00	0.10	0.13	0.18	1.50E-03	1.87E-03	2.28E-03	9.88E-04	1.28E-03	1.66E-03
Czech Republic - Streptococcus pneumoniae PMRSP BSI - model 76	0.00	0.00	0.00	0.00	0.00	0.00	0.00	0.00	0.00	NaN	NaN	NaN	0.00	0.00	0.00
Czech Republic - Streptococcus pneumoniae PMRSP OTH - model 80	0.00	0.00	0.00	0.00	0.00	0.00	0.00	0.00	0.00	NaN	NaN	NaN	0.00	0.00	0.00
Czech Republic - Streptococcus pneumoniae PMRSP RESP - model 77	0.00	0.00	0.00	0.00	0.00	0.00	0.00	0.00	0.00	NaN	NaN	NaN	0.00	0.00	0.00
Czech Republic - Streptococcus pneumoniae PMRSP SSI - model 79	0.00	0.00	0.00	0.00	0.00	0.00	0.00	0.00	0.00	NaN	NaN	NaN	0.00	0.00	0.00
Czech Republic - Streptococcus pneumoniae PMRSP UTI - model 78	0.00	0.00	0.00	0.00	0.00	0.00	0.00	0.00	0.00	NaN	NaN	NaN	0.00	0.00	0.00
Czech Republic - Streptococcus pneumoniae PRSP BSI - model 71	0.00	0.00	0.00	0.00	0.00	0.00	0.00	0.00	0.00	NaN	NaN	NaN	0.00	0.00	0.00
Czech Republic - Streptococcus pneumoniae PRSP OTH - model 75	0.00	0.00	0.00	0.00	0.00	0.00	0.00	0.00	0.00	NaN	NaN	NaN	0.00	0.00	0.00
Czech Republic - Streptococcus pneumoniae PRSP RESP - model 72	0.00	0.00	0.00	0.00	0.00	0.00	0.00	0.00	0.00	NaN	NaN	NaN	0.00	0.00	0.00
Czech Republic - Streptococcus pneumoniae PRSP SSI - model 74	0.00	0.00	0.00	0.00	0.00	0.00	0.00	0.00	0.00	NaN	NaN	NaN	0.00	0.00	0.00
Czech Republic - Streptococcus pneumoniae PRSP UTI - model 73	0.00	0.00	0.00	0.00	0.00	0.00	0.00	0.00	0.00	NaN	NaN	NaN	0.00	0.00	0.00
**Total (Median & 95% UIs)**	1,645.23	1,833.47	2,039.76	8,617.59	9,637.37	10,739.06	10,329.38	11,470.18	12,718.98		-		15.61	17.40	19.36
**Total (average)**		**1,835.95**			**9,647.06**			**11,483.01**			-			**17.42**	

Model	YLL per 100,000			DALY per 100,000			Cases per year			Incidence per 100,000			Deaths per year			Mortality per 100,000		
Czech Republic - Acinetobacter spp. ColRACI BSI - model 41	1.34E-01	2.15E-01	3.14E-01	1.61E-01	2.43E-01	3.42E-01	4.31E+00	4.89E+00	5.53E+00	4.09E-02	4.64E-02	5.25E-02	6.49E-01	9.98E-01	1.41E+00	6.16E-03	9.47E-03	1.34E-02
Czech Republic - Acinetobacter spp. ColRACI OTH - model 45	0.00	0.00	0.00	2.52E-06	5.76E-06	1.02E-05	0.08	0.16	0.26	7.70E-04	1.56E-03	2.44E-03	0.00	0.00	0.00	0.00	0.00	0.00
Czech Republic - Acinetobacter spp. ColRACI RESP - model 42	0.03	0.04	0.05	0.05	0.06	0.08	4.09	5.31	6.49	0.04	0.05	0.06	0.15	0.19	0.24	1.39E-03	1.80E-03	2.24E-03
Czech Republic - Acinetobacter spp. ColRACI SSI - model 44	4.31E-03	6.48E-03	8.48E-03	4.34E-03	6.51E-03	8.54E-03	1.23	1.79	2.33	0.01	0.02	0.02	0.03	0.04	0.05	2.46E-04	3.82E-04	5.14E-04
Czech Republic - Acinetobacter spp. ColRACI UTI - model 43	0.00	0.00	0.00	2.12E-05	3.78E-05	6.11E-05	1.33	2.00	2.67	0.01	0.02	0.03	0.00	0.00	0.00	0.00	0.00	0.00
Czech Republic - Acinetobacter spp. CRACI BSI - model 36	0.10	0.21	0.35	0.12	0.24	0.38	2.06	2.45	2.86	0.02	0.02	0.03	0.24	0.50	0.79	2.26E-03	4.73E-03	7.53E-03
Czech Republic - Acinetobacter spp. CRACI OTH - model 40	0.00	0.00	0.00	8.17E-07	2.73E-06	6.43E-06	0.03	0.08	0.15	2.61E-04	7.65E-04	1.44E-03	0.00	0.00	0.00	0.00	0.00	0.00
Czech Republic - Acinetobacter spp. CRACI RESP - model 37	0.03	0.04	0.06	0.04	0.06	0.08	1.88	2.63	3.43	0.02	0.02	0.03	0.07	0.09	0.12	6.31E-04	8.98E-04	1.18E-03
Czech Republic - Acinetobacter spp. CRACI SSI - model 39	3.28E-03	5.80E-03	8.27E-03	3.29E-03	5.81E-03	8.30E-03	0.49	0.89	1.26	4.69E-03	8.41E-03	0.01	9.22E-03	0.02	0.03	8.75E-05	1.90E-04	2.85E-04
Czech Republic - Acinetobacter spp. CRACI UTI - model 38	0.00	0.00	0.00	8.44E-06	1.86E-05	3.52E-05	0.55	0.99	1.44	5.26E-03	9.38E-03	0.01	0.00	0.00	0.00	0.00	0.00	0.00
Czech Republic - Acinetobacter spp. MDRACI BSI - model 31	0.14	0.25	0.39	0.17	0.29	0.43	3.16	3.66	4.21	0.03	0.03	0.04	0.41	0.72	1.10	3.87E-03	6.86E-03	0.01
Czech Republic - Acinetobacter spp. MDRACI OTH - model 35	0.00	0.00	0.00	1.59E-06	4.21E-06	8.79E-06	0.05	0.12	0.21	5.15E-04	1.14E-03	1.99E-03	0.00	0.00	0.00	0.00	0.00	0.00
Czech Republic - Acinetobacter spp. MDRACI RESP - model 32	0.04	0.05	0.06	0.06	0.07	0.10	3.03	3.96	4.98	0.03	0.04	0.05	0.11	0.14	0.18	1.00E-03	1.36E-03	1.73E-03
Czech Republic - Acinetobacter spp. MDRACI SSI - model 34	3.90E-03	6.13E-03	8.24E-03	3.92E-03	6.15E-03	8.26E-03	0.85	1.33	1.76	8.11E-03	0.01	0.02	0.01	0.02	0.03	1.32E-04	2.30E-04	3.26E-04
Czech Republic - Acinetobacter spp. MDRACI UTI - model 33	0.00	0.00	0.00	1.54E-05	2.83E-05	4.92E-05	0.92	1.52	2.08	8.75E-03	0.01	0.02	0.00	0.00	0.00	0.00	0.00	0.00
Czech Republic - Enterococcus faecalis and E. faecium VRE BSI - model 66	2.26	2.71	3.24	2.53	3.03	3.61	42.68	50.65	60.17	0.41	0.48	0.57	9.78	11.59	13.76	0.09	0.11	0.13
Czech Republic - Enterococcus faecalis and E. faecium VRE OTH - model 70	0.00	0.00	0.00	6.08E-04	8.36E-04	1.12E-03	42.73	53.80	64.11	0.41	0.51	0.61	0.00	0.00	0.00	0.00	0.00	0.00
Czech Republic - Enterococcus faecalis and E. faecium VRE RESP - model 67	0.00	0.00	0.00	0.00	0.00	0.00	0.00	0.00	0.00	0.00	0.00	0.00	0.00	0.00	0.00	0.00	0.00	0.00
Czech Republic - Enterococcus faecalis and E. faecium VRE SSI - model 69	0.22	0.29	0.36	0.22	0.29	0.36	55.32	69.35	83.10	0.52	0.66	0.79	1.26	1.70	2.14	0.01	0.02	0.02
Czech Republic - Enterococcus faecalis and E. faecium VRE UTI - model 68	0.00	0.00	0.00	3.21E-04	4.54E-04	6.09E-04	20.25	25.67	31.53	0.19	0.24	0.30	0.00	0.00	0.00	0.00	0.00	0.00
Czech Republic - Escherichia coli 3GCREC BSI - model 16	13.57	15.09	16.82	15.82	17.44	19.29	525.12	567.95	610.94	4.98	5.39	5.80	87.80	98.25	111.10	0.83	0.93	1.05
Czech Republic - Escherichia coli 3GCREC OTH - model 20	0.00	0.00	0.00	4.32E-03	5.94E-03	8.01E-03	151.07	188.26	229.16	1.43	1.79	2.17	0.00	0.00	0.00	0.00	0.00	0.00
Czech Republic - Escherichia coli 3GCREC RESP - model 17	1.15	1.39	1.63	1.75	2.11	2.46	204.27	250.99	296.25	1.94	2.38	2.81	7.27	9.05	10.74	0.07	0.09	0.10
Czech Republic - Escherichia coli 3GCREC SSI - model 19	2.01	2.37	2.70	2.02	2.38	2.71	514.63	600.53	681.87	4.88	5.70	6.47	15.64	18.68	21.44	0.15	0.18	0.20
Czech Republic - Escherichia coli 3GCREC UTI - model 18	0.00	0.00	0.00	0.04	0.05	0.06	2,179.07	2,435.25	2,708.02	20.68	23.11	25.70	0.00	0.00	0.00	0.00	0.00	0.00
Czech Republic - Escherichia coli ColREC BSI - model 26	0.00	0.00	0.00	0.00	0.00	0.00	0.00	0.00	0.00	0.00	0.00	0.00	0.00	0.00	0.00	0.00	0.00	0.00
Czech Republic - Escherichia coli ColREC OTH - model 30	0.00	0.00	0.00	0.00	0.00	0.00	0.00	0.00	0.00	0.00	0.00	0.00	0.00	0.00	0.00	0.00	0.00	0.00
Czech Republic - Escherichia coli ColREC RESP - model 27	0.00	0.00	0.00	0.00	0.00	0.00	0.00	0.00	0.00	0.00	0.00	0.00	0.00	0.00	0.00	0.00	0.00	0.00
Czech Republic - Escherichia coli ColREC SSI - model 29	0.00	0.00	0.00	0.00	0.00	0.00	0.00	0.00	0.00	0.00	0.00	0.00	0.00	0.00	0.00	0.00	0.00	0.00
Czech Republic - Escherichia coli ColREC UTI - model 28	0.00	0.00	0.00	0.00	0.00	0.00	0.00	0.00	0.00	0.00	0.00	0.00	0.00	0.00	0.00	0.00	0.00	0.00
Czech Republic - Escherichia coli CREC BSI - model 21	0.00	0.00	0.00	0.00	0.00	0.00	0.00	0.00	0.00	0.00	0.00	0.00	0.00	0.00	0.00	0.00	0.00	0.00
Czech Republic - Escherichia coli CREC OTH - model 25	0.00	0.00	0.00	0.00	0.00	0.00	0.00	0.00	0.00	0.00	0.00	0.00	0.00	0.00	0.00	0.00	0.00	0.00
Czech Republic - Escherichia coli CREC RESP - model 22	0.00	0.00	0.00	0.00	0.00	0.00	0.00	0.00	0.00	0.00	0.00	0.00	0.00	0.00	0.00	0.00	0.00	0.00
Czech Republic - Escherichia coli CREC SSI - model 24	0.00	0.00	0.00	0.00	0.00	0.00	0.00	0.00	0.00	0.00	0.00	0.00	0.00	0.00	0.00	0.00	0.00	0.00
Czech Republic - Escherichia coli CREC UTI - model 23	0.00	0.00	0.00	0.00	0.00	0.00	0.00	0.00	0.00	0.00	0.00	0.00	0.00	0.00	0.00	0.00	0.00	0.00
Czech Republic - Klebsiella pneumoniae 3GCRKP BSI - model 1	30.01	31.72	33.46	34.91	36.78	38.82	864.95	907.50	950.27	8.21	8.61	9.02	142.83	151.56	160.93	1.36	1.44	1.53
Czech Republic - Klebsiella pneumoniae 3GCRKP RESP - model 2	5.28	5.85	6.48	8.03	8.88	9.80	705.78	778.27	855.71	6.70	7.39	8.12	25.06	27.97	31.26	0.24	0.27	0.30
Czech Republic - Klebsiella pneumoniae 3GCRKP UTI - model 3	0.00	0.00	0.00	0.02	0.02	0.02	953.16	1,062.34	1,157.93	9.04	10.08	10.99	0.00	0.00	0.00	0.00	0.00	0.00
Czech Republic - Klebsiella pneumoniae 3GCRKP SSI - model 4	1.27	1.50	1.73	1.28	1.51	1.73	312.55	354.21	401.80	2.97	3.36	3.81	8.01	9.37	10.89	0.08	0.09	0.10
Czech Republic - Klebsiella pneumoniae 3GCRKP OTH - model 5	0.00	0.00	0.00	9.03E-03	0.01	0.01	315.76	359.95	403.84	3.00	3.42	3.83	0.00	0.00	0.00	0.00	0.00	0.00
Czech Republic - Klebsiella pneumoniae ColRKP BSI - model 11	1.86	2.32	2.79	1.96	2.42	2.91	17.00	18.68	20.68	0.16	0.18	0.20	9.18	11.26	13.66	0.09	0.11	0.13
Czech Republic - Klebsiella pneumoniae ColRKP OTH - model 15	0.00	0.00	0.00	1.93E-04	2.83E-04	4.13E-04	6.67	8.65	10.56	0.06	0.08	0.10	0.00	0.00	0.00	0.00	0.00	0.00
Czech Republic - Klebsiella pneumoniae ColRKP RESP - model 12	0.05	0.06	0.07	0.07	0.09	0.11	6.12	7.86	9.74	0.06	0.07	0.09	0.22	0.28	0.35	2.07E-03	2.68E-03	3.33E-03
Czech Republic - Klebsiella pneumoniae ColRKP SSI - model 14	0.02	0.02	0.03	0.02	0.02	0.03	4.15	5.51	6.84	0.04	0.05	0.06	0.11	0.15	0.19	1.02E-03	1.41E-03	1.82E-03
Czech Republic - Klebsiella pneumoniae ColRKP UTI - model 13	0.00	0.00	0.00	1.57E-04	2.16E-04	3.11E-04	9.06	11.22	13.60	0.09	0.11	0.13	0.00	0.00	0.00	0.00	0.00	0.00
Czech Republic - Klebsiella pneumoniae CRKP BSI - model 6	0.26	0.38	0.53	0.28	0.41	0.57	3.10	3.76	4.46	0.03	0.04	0.04	0.93	1.31	1.83	8.86E-03	0.01	0.02
Czech Republic - Klebsiella pneumoniae CRKP OTH - model 10	0.00	0.00	0.00	2.47E-05	5.39E-05	1.03E-04	0.99	1.70	2.45	9.40E-03	0.02	0.02	0.00	0.00	0.00	0.00	0.00	0.00
Czech Republic - Klebsiella pneumoniae CRKP RESP - model 7	8.78E-03	0.02	0.02	0.01	0.03	0.04	0.94	1.60	2.20	8.88E-03	0.02	0.02	0.03	0.06	0.08	3.16E-04	5.47E-04	7.66E-04
Czech Republic - Klebsiella pneumoniae CRKP SSI - model 9	2.18E-03	4.09E-03	5.96E-03	2.19E-03	4.12E-03	5.98E-03	0.59	1.10	1.59	5.59E-03	0.01	0.02	9.36E-03	0.02	0.03	8.89E-05	1.88E-04	2.84E-04
Czech Republic - Klebsiella pneumoniae CRKP UTI - model 8	0.00	0.00	0.00	2.13E-05	4.37E-05	7.63E-05	1.39	2.22	3.10	0.01	0.02	0.03	0.00	0.00	0.00	0.00	0.00	0.00
Czech Republic - Pseudomonas aeruginosa ColRPA BSI - model 56	0.02	0.06	0.10	0.03	0.06	0.11	1.95	2.51	3.17	0.02	0.02	0.03	0.25	0.56	0.94	2.38E-03	5.29E-03	8.91E-03
Czech Republic - Pseudomonas aeruginosa ColRPA OTH - model 60	0.00	0.00	0.00	1.70E-05	4.45E-05	8.71E-05	0.58	1.28	1.96	5.53E-03	0.01	0.02	0.00	0.00	0.00	0.00	0.00	0.00
Czech Republic - Pseudomonas aeruginosa ColRPA RESP - model 57	0.03	0.06	0.11	0.04	0.08	0.13	6.32	9.43	12.76	0.06	0.09	0.12	0.30	0.61	1.05	2.82E-03	5.78E-03	9.94E-03
Czech Republic - Pseudomonas aeruginosa ColRPA SSI - model 59	2.75E-03	6.37E-03	9.64E-03	2.77E-03	6.38E-03	9.67E-03	0.87	1.71	2.56	8.21E-03	0.02	0.02	0.03	0.06	0.09	2.96E-04	5.83E-04	8.73E-04
Czech Republic - Pseudomonas aeruginosa ColRPA UTI - model 58	0.00	0.00	0.00	2.72E-05	6.27E-05	1.18E-04	1.85	3.19	4.65	0.02	0.03	0.04	0.00	0.00	0.00	0.00	0.00	0.00
Czech Republic - Pseudomonas aeruginosa CRPA BSI - model 51	2.37	2.94	3.62	2.70	3.29	3.98	56.44	61.81	67.84	0.54	0.59	0.64	11.08	14.08	17.49	0.11	0.13	0.17
Czech Republic - Pseudomonas aeruginosa CRPA OTH - model 55	0.00	0.00	0.00	8.17E-04	1.08E-03	1.39E-03	25.67	31.44	36.94	0.24	0.30	0.35	0.00	0.00	0.00	0.00	0.00	0.00
Czech Republic - Pseudomonas aeruginosa CRPA RESP - model 52	2.51	3.13	3.90	3.30	4.02	4.90	199.11	228.53	260.23	1.89	2.17	2.47	12.07	15.11	18.83	0.11	0.14	0.18
Czech Republic - Pseudomonas aeruginosa CRPA SSI - model 54	0.14	0.18	0.22	0.14	0.18	0.22	34.47	42.70	50.39	0.33	0.41	0.48	0.86	1.11	1.35	8.15E-03	0.01	0.01
Czech Republic - Pseudomonas aeruginosa CRPA UTI - model 53	0.00	0.00	0.00	1.17E-03	1.54E-03	2.00E-03	66.10	79.54	93.11	0.63	0.75	0.88	0.00	0.00	0.00	0.00	0.00	0.00
Czech Republic - Pseudomonas aeruginosa MDRPA BSI - model 46	1.97	2.52	3.08	2.27	2.84	3.42	53.99	59.34	65.15	0.51	0.56	0.62	9.81	12.64	15.57	0.09	0.12	0.15
Czech Republic - Pseudomonas aeruginosa MDRPA OTH - model 50	0.00	0.00	0.00	7.79E-04	1.06E-03	1.42E-03	23.67	29.92	36.01	0.22	0.28	0.34	0.00	0.00	0.00	0.00	0.00	0.00
Czech Republic - Pseudomonas aeruginosa MDRPA RESP - model 47	2.17	2.76	3.40	2.90	3.56	4.28	191.32	219.46	249.54	1.82	2.08	2.37	10.73	13.71	17.02	0.10	0.13	0.16
Czech Republic - Pseudomonas aeruginosa MDRPA SSI - model 49	0.13	0.17	0.20	0.13	0.17	0.20	33.06	40.73	48.04	0.31	0.39	0.46	0.83	1.07	1.30	7.86E-03	0.01	0.01
Czech Republic - Pseudomonas aeruginosa MDRPA UTI - model 48	0.00	0.00	0.00	1.08E-03	1.46E-03	1.91E-03	62.90	75.82	88.53	0.60	0.72	0.84	0.00	0.00	0.00	0.00	0.00	0.00
Czech Republic - Staphylococcus aureus MRSA BSI - model 61	9.51	10.09	10.71	10.95	11.61	12.26	284.73	298.78	313.82	2.70	2.84	2.98	50.53	53.64	57.07	0.48	0.51	0.54
Czech Republic - Staphylococcus aureus MRSA OTH - model 65	0.00	0.00	0.00	0.01	0.01	0.02	402.11	439.72	480.15	3.82	4.17	4.56	0.00	0.00	0.00	0.00	0.00	0.00
Czech Republic - Staphylococcus aureus MRSA RESP - model 62	2.73	3.04	3.32	4.19	4.61	5.04	408.76	449.84	490.92	3.88	4.27	4.66	14.59	16.17	17.83	0.14	0.15	0.17
Czech Republic - Staphylococcus aureus MRSA SSI - model 64	1.75	1.94	2.12	1.76	1.95	2.13	447.70	490.50	529.18	4.25	4.65	5.02	12.01	13.33	14.58	0.11	0.13	0.14
Czech Republic - Staphylococcus aureus MRSA UTI - model 63	0.00	0.00	0.00	9.88E-04	1.28E-03	1.66E-03	61.36	73.01	85.05	0.58	0.69	0.81	0.00	0.00	0.00	0.00	0.00	0.00
Czech Republic - Streptococcus pneumoniae PMRSP BSI - model 76	0.00	0.00	0.00	0.00	0.00	0.00	0.00	0.00	0.00	0.00	0.00	0.00	0.00	0.00	0.00	0.00	0.00	0.00
Czech Republic - Streptococcus pneumoniae PMRSP OTH - model 80	0.00	0.00	0.00	0.00	0.00	0.00	0.00	0.00	0.00	0.00	0.00	0.00	0.00	0.00	0.00	0.00	0.00	0.00
Czech Republic - Streptococcus pneumoniae PMRSP RESP - model 77	0.00	0.00	0.00	0.00	0.00	0.00	0.00	0.00	0.00	0.00	0.00	0.00	0.00	0.00	0.00	0.00	0.00	0.00
Czech Republic - Streptococcus pneumoniae PMRSP SSI - model 79	0.00	0.00	0.00	0.00	0.00	0.00	0.00	0.00	0.00	0.00	0.00	0.00	0.00	0.00	0.00	0.00	0.00	0.00
Czech Republic - Streptococcus pneumoniae PMRSP UTI - model 78	0.00	0.00	0.00	0.00	0.00	0.00	0.00	0.00	0.00	0.00	0.00	0.00	0.00	0.00	0.00	0.00	0.00	0.00
Czech Republic - Streptococcus pneumoniae PRSP BSI - model 71	0.00	0.00	0.00	0.00	0.00	0.00	0.00	0.00	0.00	0.00	0.00	0.00	0.00	0.00	0.00	0.00	0.00	0.00
Czech Republic - Streptococcus pneumoniae PRSP OTH - model 75	0.00	0.00	0.00	0.00	0.00	0.00	0.00	0.00	0.00	0.00	0.00	0.00	0.00	0.00	0.00	0.00	0.00	0.00
Czech Republic - Streptococcus pneumoniae PRSP RESP - model 72	0.00	0.00	0.00	0.00	0.00	0.00	0.00	0.00	0.00	0.00	0.00	0.00	0.00	0.00	0.00	0.00	0.00	0.00
Czech Republic - Streptococcus pneumoniae PRSP SSI - model 74	0.00	0.00	0.00	0.00	0.00	0.00	0.00	0.00	0.00	0.00	0.00	0.00	0.00	0.00	0.00	0.00	0.00	0.00
Czech Republic - Streptococcus pneumoniae PRSP UTI - model 73	0.00	0.00	0.00	0.00	0.00	0.00	0.00	0.00	0.00	0.00	0.00	0.00	0.00	0.00	0.00	0.00	0.00	0.00
**Total (Median & 95% UIs)**	81.77	91.45	101.91	98.02	108.84	120.69	9,324.39	10,438.06	11,569.58	88.48	99.05	109.79	432.87	486.09	545.49	4.11	4.61	5.18
**Total (average)**		**91.54**			**108.96**			**10,443.14**			**99.10**			**486.73**			**4.62**	

Infection	YLD			YLL			DALY			DALY per Case			YLD per 100,000		
Colistin resistant *Acinetobacter spp.*	5.98	9.02	13.03	24.45	47.40	79.67	31.89	56.40	91.62	2.76	4.53	6.62	0.01	0.01	0.02
Carbapenem resistant *Acinetobacter spp.* (excluding those resistant to colistin)	67.99	81.36	96.34	329.26	434.42	548.22	402.81	516.36	641.25	4.58	5.60	6.69	0.08	0.10	0.12
Aminoglycosides and fluoroquinolones resistant *Acinetobacter* spp. (excluding those resistant to colistin and/or carbapenem)	9.36	14.21	21.17	38.02	75.01	127.45	49.32	89.10	144.73	4.36	7.05	10.26	0.01	0.02	0.03
Vancomycin resistant Enterococci (*E. faecalis* and *E. faecium*)	396.93	476.90	564.90	3,760.51	4,511.91	5,322.80	4,158.96	4,986.88	5,888.96	5.64	6.24	6.83	0.49	0.59	0.70
Colistin resistant *Escherichia coli*	59.32	72.26	87.65	949.88	1,227.95	1,515.11	1,016.50	1,301.06	1,612.52	9.25	11.37	13.80	0.07	0.09	0.11
Carbapenem resistant *Escherichia coli* (excluding those resistant to colistin)	5.31	7.84	11.10	52.60	81.67	121.86	58.63	89.79	133.41	5.48	7.24	9.47	0.01	0.01	0.01
Third-generation cephalosporin resistant *Escherichia coli* (excluding those resistant to colistin and/or carbapenem)	2,126.51	2,420.26	2,796.12	12,654.65	14,408.43	16,325.01	14,804.04	16,833.12	19,020.70	4.33	4.77	5.24	2.62	2.98	3.44
Colistin resistant *Klebsiella pneumoniae*	28.93	37.18	46.90	487.37	645.98	850.83	518.54	683.57	892.26	12.97	16.53	20.72	0.04	0.05	0.06
Carbapenem resistant *Klebsiella pneumoniae* (excluding those resistant to colistin)	5.64	9.35	15.12	55.45	97.36	170.61	62.00	107.12	185.22	9.20	13.83	19.49	0.01	0.01	0.02
Third-generation cephalosporin resistant *Klebsiella pneumoniae* (excluding those resistant to colistin and/or carbapenem)	458.79	519.21	583.26	2,252.82	2,478.12	2,742.34	2,731.20	2,997.72	3,313.56	4.77	5.09	5.45	0.57	0.64	0.72
Colistin resistant *Pseudomonas aeruginosa*	0.00	0.00	0.00	0.00	0.00	0.00	0.00	0.00	0.00	0.00	0.00	0.00	0.00	0.00	0.00
Carbapenem resistant *Pseudomonas aeruginosa* (excluding those resistant to colistin)	1,294.68	1,508.57	1,739.67	6,025.29	7,597.87	9,338.91	7,424.97	9,118.69	10,970.13	7.39	8.80	10.20	1.59	1.86	2.14
Pseudomonas aeruginosa resistant to three or more antimicrobial groups (excluding those resistant to colistin and/or carbapenem)	114.98	142.70	174.00	500.71	675.09	887.98	624.04	817.30	1,048.42	5.85	7.39	9.09	0.14	0.18	0.21
Methicillin resistant *Staphylococcus aureus*	2.052.50	2.328.34	2.611.74	10.241.34	11.426.94	12.628.84	12.322.15	13.757.79	15.186.19	4.89	5.16	5.47	2.53	2.87	3.22
Penicillin and macrolides resistant *Streptococcus pneumoniae* (excluding those only resistant to penicillin)	18.39	26.42	41.41	67.02	86.34	118.83	87.04	112.95	158.62	4.98	5.80	6.74	0.02	0.03	0.05
Penicillin tesistant *Streptococcus pneumoniae* (excluding those resistant to macrolides)	130.99	167.95	220.36	459.44	544.58	662.36	595.77	713.54	875.95	11.04	12.04	13.25	0.16	0.21	0.27
Total	6,776.30	7,821.59	9,022.76	37,898.82	44,339.05	51,440.82	44,887.87	52,181.40	60,163.53				8.35	9.63	11.11

Infection	YLL per 100,000			DALY per 100,000			Cases per year			Incidence per 100,000			Deaths per year			Mortality per 100,000		
Colistin resistant *Acinetobacter spp.*	0.03	0.06	0.10	0.04	0.07	0.11	26.13	37.62	50.23	0.03	0.05	0.06	1.90	3.30	5.04	0.00	0.00	0.01
Carbapenem resistant *Acinetobacter spp.* (excluding those resistant to colistin)	0.41	0.54	0.68	0.50	0.64	0.79	233.11	277.53	324.74	0.29	0.34	0.40	18.76	24.14	30.64	0.02	0.03	0.04
Aminoglycosides and fluoroquinolones resistant *Acinetobacter* spp. (excluding those resistant to colistin and/or carbapenem)	0.05	0.09	0.16	0.06	0.11	0.18	25.71	37.76	50.86	0.03	0.05	0.06	1.88	3.23	4.92	0.00	0.00	0.01
Vancomycin resistant Enterococci (*E. faecalis* and *E. faecium*)	4.63	5.56	6.56	5.12	6.14	7.25	2,483.18	3,088.59	3,700.08	3.06	3.80	4.56	167.77	205.46	245.73	0.21	0.25	0.30
Colistin resistant *Escherichia coli*	1.17	1.51	1.87	1.25	1.60	1.99	725.79	891.64	1,070.50	0.89	1.10	1.32	60.11	78.08	97.18	0.07	0.10	0.12
Carbapenem resistant *Escherichia coli* (excluding those resistant to colistin)	0.06	0.10	0.15	0.07	0.11	0.16	66.18	99.00	131.55	0.08	0.12	0.16	3.47	5.28	7.60	0.00	0.01	0.01
Third-generation cephalosporin resistant *Escherichia coli* (excluding those resistant to colistin and/or carbapenem)	15.59	17.74	20.11	18.23	20.73	23.43	24,000.61	28,391.89	32,720.22	29.56	34.97	40.30	734.98	867.92	1,011.37	0.91	1.07	1.25
Colistin resistant *Klebsiella pneumoniae*	0.60	0.80	1.05	0.64	0.84	1.10	95.27	125.61	155.89	0.12	0.15	0.19	21.10	27.49	35.00	0.03	0.03	0.04
Carbapenem resistant *Klebsiella pneumoniae* (excluding those resistant to colistin)	0.07	0.12	0.21	0.08	0.13	0.23	15.16	25.07	36.30	0.02	0.03	0.04	2.07	3.27	4.97	0.00	0.00	0.01
Third-generation cephalosporin resistant *Klebsiella pneumoniae* (excluding those resistant to colistin and/or carbapenem)	2.77	3.05	3.38	3.36	3.69	4.08	2,115.43	2,470.16	2,859.42	2.61	3.04	3.52	118.59	133.57	151.71	0.15	0.16	0.19
Colistin resistant *Pseudomonas aeruginosa*	0.00	0.00	0.00	0.00	0.00	0.00	0.00	0.00	0.00	0.00	0.00	0.00	0.00	0.00	0.00	0.00	0.00	0.00
Carbapenem resistant *Pseudomonas aeruginosa* (excluding those resistant to colistin)	7.42	9.36	11.50	9.14	11.23	13.51	3,872.69	4,628.24	5,387.34	4.77	5.70	6.63	241.23	310.73	389.36	0.30	0.38	0.48
Pseudomonas aeruginosa resistant to three or more antimicrobial groups (excluding those resistant to colistin and/or carbapenem)	0.62	0.83	1.09	0.77	1.01	1.29	396.25	490.57	592.89	0.49	0.60	0.73	22.60	31.22	40.14	0.03	0.04	0.05
Methicillin resistant *Staphylococcus aureus*	12.61	14.07	15.55	15.18	16.94	18.70	11.864.56	13.683.49	15.409.18	14.61	16.85	18.98	576.66	653.42	732.98	0.71	0.80	0.90
Penicillin and macrolides resistant *Streptococcus pneumoniae* (excluding those only resistant to penicillin)	0.08	0.11	0.15	0.11	0.14	0.20	50.10	64.99	86.60	0.06	0.08	0.11	3.22	3.95	4.90	0.00	0.00	0.01
Penicillin tesistant *Streptococcus pneumoniae* (excluding those resistant to macrolides)	0.57	0.67	0.82	0.73	0.88	1.08	163.60	196.65	232.91	0.20	0.24	0.29	10.29	11.81	13.54	0.01	0.01	0.02
Total	46.67	54.61	63.35	55.28	64.26	74.10	46,133.78	54,508.80	62,808.71	56.82	67.13	77.35	1,984.63	2,362.88	2,775.08	2.44	2.91	3.42

Model	YLD			YLL			DALY			DALY per Case			YLD per 100,000		
Germany - Acinetobacter spp. ColRACI BSI - model 41	3.53E+00	5.14E+00	7.28E+00	1.90E+01	3.88E+01	6.71E+01	2.39E+01	4.38E+01	7.35E+01	1.91E+00	3.47E+00	5.28E+00	4.35E-03	6.32E-03	8.96E-03
Germany - Acinetobacter spp. ColRACI OTH - model 45	6.36E-04	1.79E-03	3.46E-03	0.00	0.00	0.00	6.36E-04	1.79E-03	3.46E-03	2.24E-03	3.78E-03	5.59E-03	7.83E-07	2.21E-06	4.26E-06
Germany - Acinetobacter spp. ColRACI RESP - model 42	2.44	3.86	5.71	4.74	7.38	10.72	7.21	11.29	16.22	0.61	0.79	1.04	3.01E-03	4.75E-03	7.03E-03
Germany - Acinetobacter spp. ColRACI SSI - model 44	3.92E-03	9.77E-03	0.02	0.72	1.26	1.90	0.73	1.28	1.90	0.24	0.26	0.29	4.82E-06	1.20E-05	2.50E-05
Germany - Acinetobacter spp. ColRACI UTI - model 43	5.35E-03	0.01	0.02	0.00	0.00	0.00	5.35E-03	0.01	0.02	1.16E-03	2.05E-03	3.08E-03	6.59E-06	1.35E-05	2.39E-05
Germany - Acinetobacter spp. CRACI BSI - model 36	39.37	46.33	54.02	262.77	352.70	448.69	306.65	399.45	500.28	3.32	4.20	5.15	0.05	0.06	0.07
Germany - Acinetobacter spp. CRACI OTH - model 40	8.62E-03	0.01	0.02	0.00	0.00	0.00	8.62E-03	0.01	0.02	2.94E-03	3.73E-03	4.59E-03	1.06E-05	1.65E-05	2.38E-05
Germany - Acinetobacter spp. CRACI RESP - model 37	28.50	34.87	42.08	55.26	66.97	80.93	84.76	102.00	122.14	0.89	0.99	1.08	0.04	0.04	0.05
Germany - Acinetobacter spp. CRACI SSI - model 39	0.05	0.08	0.11	11.24	14.74	18.60	11.33	14.82	18.70	0.37	0.41	0.46	6.18E-05	9.24E-05	1.31E-04
Germany - Acinetobacter spp. CRACI UTI - model 38	0.06	0.08	0.11	0.00	0.00	0.00	0.06	0.08	0.11	1.60E-03	2.03E-03	2.52E-03	7.18E-05	9.98E-05	1.37E-04
Germany - Acinetobacter spp. MDRACI BSI - model 31	5.73	8.06	11.79	29.84	60.97	106.86	37.22	68.87	115.25	3.06	5.36	8.12	7.06E-03	9.93E-03	0.01
Germany - Acinetobacter spp. MDRACI OTH - model 35	6.28E-04	1.69E-03	3.53E-03	0.00	0.00	0.00	6.28E-04	1.69E-03	3.53E-03	2.11E-03	3.67E-03	5.48E-03	7.73E-07	2.08E-06	4.35E-06
Germany - Acinetobacter spp. MDRACI RESP - model 32	3.62	6.12	9.33	7.13	12.07	17.66	11.04	18.25	26.51	0.96	1.28	1.65	4.45E-03	7.54E-03	0.01
Germany - Acinetobacter spp. MDRACI SSI - model 34	4.21E-03	9.93E-03	0.02	1.04	1.97	2.93	1.06	1.98	2.94	0.33	0.41	0.48	5.19E-06	1.22E-05	2.47E-05
Germany - Acinetobacter spp. MDRACI UTI - model 33	5.24E-03	0.01	0.02	0.00	0.00	0.00	5.24E-03	0.01	0.02	1.22E-03	2.03E-03	3.04E-03	6.45E-06	1.33E-05	2.47E-05
Germany - Enterococcus faecalis and E. faecium VRE BSI - model 66	393.72	472.50	559.00	3,413.78	4,062.98	4,772.91	3,809.10	4,533.89	5,333.92	5.26	5.81	6.36	0.48	0.58	0.69
Germany - Enterococcus faecalis and E. faecium VRE OTH - model 70	1.01	1.36	1.79	0.00	0.00	0.00	1.01	1.36	1.79	1.31E-03	1.64E-03	2.01E-03	1.24E-03	1.67E-03	2.21E-03
Germany - Enterococcus faecalis and E. faecium VRE RESP - model 67	0.00	0.00	0.00	0.00	0.00	0.00	0.00	0.00	0.00	NaN	NaN	NaN	0.00	0.00	0.00
Germany - Enterococcus faecalis and E. faecium VRE SSI - model 69	1.65	2.31	3.13	346.73	448.93	549.89	348.30	450.90	552.28	0.38	0.42	0.46	2.03E-03	2.84E-03	3.85E-03
Germany - Enterococcus faecalis and E. faecium VRE UTI - model 68	0.55	0.74	0.97	0.00	0.00	0.00	0.55	0.74	0.97	1.51E-03	1.87E-03	2.24E-03	6.75E-04	9.13E-04	1.20E-03
Germany - Escherichia coli 3GCREC BSI - model 16	1,617.54	1,803.22	2,048.17	10,346.13	11,633.01	13,051.60	11,988.08	13,439.91	14,999.21	3.12	3.43	3.75	1.99	2.22	2.52
Germany - Escherichia coli 3GCREC OTH - model 20	3.15	4.48	6.00	0.00	0.00	0.00	3.15	4.48	6.00	2.63E-03	3.34E-03	4.19E-03	3.87E-03	5.52E-03	7.38E-03
Germany - Escherichia coli 3GCREC RESP - model 17	473.62	570.27	687.62	908.51	1,103.80	1,303.60	1,380.07	1,675.92	1,994.04	0.84	0.94	1.06	0.58	0.70	0.85
Germany - Escherichia coli 3GCREC SSI - model 19	6.56	8.99	12.47	1,400.01	1,671.62	1,969.81	1,407.08	1,679.51	1,979.59	0.37	0.40	0.43	8.07E-03	0.01	0.02
Germany - Escherichia coli 3GCREC UTI - model 18	25.66	33.30	41.86	0.00	0.00	0.00	25.66	33.30	41.86	1.54E-03	1.96E-03	2.37E-03	0.03	0.04	0.05
Germany - Escherichia coli ColREC BSI - model 26	43.99	52.27	62.34	876.10	1,132.84	1,397.41	927.44	1,185.82	1,470.96	8.11	10.06	12.28	0.05	0.06	0.08
Germany - Escherichia coli ColREC OTH - model 30	0.16	0.24	0.36	0.00	0.00	0.00	0.16	0.24	0.36	2.56E-03	3.49E-03	4.69E-03	1.95E-04	2.98E-04	4.40E-04
Germany - Escherichia coli ColREC RESP - model 27	14.24	18.40	23.04	27.09	35.30	44.31	41.38	53.82	66.04	0.75	0.88	1.03	0.02	0.02	0.03
Germany - Escherichia coli ColREC SSI - model 29	0.20	0.30	0.44	46.69	59.81	73.39	46.78	60.13	73.70	0.38	0.42	0.47	2.45E-04	3.70E-04	5.39E-04
Germany - Escherichia coli ColREC UTI - model 28	0.74	1.04	1.47	0.00	0.00	0.00	0.74	1.04	1.47	1.54E-03	2.10E-03	2.77E-03	9.14E-04	1.29E-03	1.81E-03
Germany - Escherichia coli CREC BSI - model 21	4.13	5.67	7.74	47.03	72.49	108.62	51.86	78.41	116.98	4.47	6.02	8.01	5.08E-03	6.98E-03	9.53E-03
Germany - Escherichia coli CREC OTH - model 25	0.01	0.03	0.05	0.00	0.00	0.00	0.01	0.03	0.05	2.00E-03	3.43E-03	5.71E-03	1.68E-05	3.27E-05	6.06E-05
Germany - Escherichia coli CREC RESP - model 22	1.09	2.00	3.06	2.15	3.81	5.86	3.26	5.83	8.78	0.70	0.86	1.07	1.35E-03	2.47E-03	3.77E-03
Germany - Escherichia coli CREC SSI - model 24	0.01	0.03	0.06	3.41	5.37	7.38	3.45	5.41	7.41	0.31	0.34	0.38	1.82E-05	3.98E-05	7.52E-05
Germany - Escherichia coli CREC UTI - model 23	0.06	0.11	0.20	0.00	0.00	0.00	0.06	0.11	0.20	1.21E-03	2.03E-03	3.22E-03	7.38E-05	1.36E-04	2.43E-04
Germany - Klebsiella pneumoniae 3GCRKP BSI - model 1	286.43	319.64	356.79	1,839.33	1,993.03	2,182.40	2,144.50	2,312.64	2,527.78	3.42	3.64	3.86	0.35	0.39	0.44
Germany - Klebsiella pneumoniae 3GCRKP OTH - model 5	0.63	0.86	1.15	0.00	0.00	0.00	0.63	0.86	1.15	2.63E-03	3.35E-03	4.19E-03	7.77E-04	1.06E-03	1.42E-03
Germany - Klebsiella pneumoniae 3GCRKP RESP - model 2	170.25	196.68	222.64	327.66	377.80	429.86	498.69	574.83	651.86	0.95	1.03	1.12	0.21	0.24	0.27
Germany - Klebsiella pneumoniae 3GCRKP SSI - model 4	0.38	0.54	0.78	85.83	107.29	130.08	86.27	107.90	130.86	0.40	0.42	0.45	4.62E-04	6.66E-04	9.59E-04
Germany - Klebsiella pneumoniae 3GCRKP UTI - model 3	1.11	1.48	1.90	0.00	0.00	0.00	1.11	1.48	1.90	1.53E-03	1.95E-03	2.41E-03	1.36E-03	1.83E-03	2.33E-03
Germany - Klebsiella pneumoniae ColRKP BSI - model 11	23.14	28.51	35.19	473.21	625.05	822.69	498.41	653.88	852.62	11.65	14.89	18.72	0.03	0.04	0.04
Germany - Klebsiella pneumoniae ColRKP OTH - model 15	0.05	0.07	0.11	0.00	0.00	0.00	0.05	0.07	0.11	2.46E-03	3.48E-03	4.71E-03	5.66E-05	9.13E-05	1.40E-04
Germany - Klebsiella pneumoniae ColRKP RESP - model 12	5.70	8.52	11.46	11.05	16.33	22.09	16.92	24.93	33.35	1.03	1.30	1.60	7.02E-03	0.01	0.01
Germany - Klebsiella pneumoniae ColRKP SSI - model 14	0.02	0.03	0.05	3.11	4.60	6.05	3.13	4.63	6.09	0.29	0.34	0.39	2.00E-05	3.49E-05	5.59E-05
Germany - Klebsiella pneumoniae ColRKP UTI - model 13	0.03	0.06	0.08	0.00	0.00	0.00	0.03	0.06	0.08	1.44E-03	2.07E-03	2.74E-03	4.29E-05	6.83E-05	1.03E-04
Germany - Klebsiella pneumoniae CRKP BSI - model 6	4.78	7.13	11.15	53.19	91.82	161.27	58.94	99.41	171.97	7.69	11.71	16.78	5.89E-03	8.78E-03	0.01
Germany - Klebsiella pneumoniae CRKP OTH - model 10	5.91E-03	0.01	0.03	0.00	0.00	0.00	5.91E-03	0.01	0.03	1.76E-03	3.41E-03	5.91E-03	7.28E-06	1.72E-05	3.43E-05
Germany - Klebsiella pneumoniae CRKP RESP - model 7	0.85	2.20	3.91	1.61	4.29	7.44	2.39	6.44	11.28	1.06	1.66	2.24	1.04E-03	2.71E-03	4.82E-03
Germany - Klebsiella pneumoniae CRKP SSI - model 9	2.41E-03	5.45E-03	0.01	0.65	1.24	1.91	0.66	1.24	1.91	0.45	0.45	0.46	2.97E-06	6.72E-06	1.48E-05
Germany - Klebsiella pneumoniae CRKP UTI - model 8	4.99E-03	0.01	0.02	0.00	0.00	0.00	4.99E-03	0.01	0.02	1.16E-03	2.06E-03	3.19E-03	6.15E-06	1.36E-05	2.57E-05
Germany - Pseudomonas aeruginosa ColRPA BSI - model 56	0.00	0.00	0.00	0.00	0.00	0.00	0.00	0.00	0.00	NaN	NaN	NaN	0.00	0.00	0.00
Germany - Pseudomonas aeruginosa ColRPA OTH - model 60	0.00	0.00	0.00	0.00	0.00	0.00	0.00	0.00	0.00	NaN	NaN	NaN	0.00	0.00	0.00
Germany - Pseudomonas aeruginosa ColRPA RESP - model 57	0.00	0.00	0.00	0.00	0.00	0.00	0.00	0.00	0.00	NaN	NaN	NaN	0.00	0.00	0.00
Germany - Pseudomonas aeruginosa ColRPA SSI - model 59	0.00	0.00	0.00	0.00	0.00	0.00	0.00	0.00	0.00	NaN	NaN	NaN	0.00	0.00	0.00
Germany - Pseudomonas aeruginosa ColRPA UTI - model 58	0.00	0.00	0.00	0.00	0.00	0.00	0.00	0.00	0.00	NaN	NaN	NaN	0.00	0.00	0.00
Germany - Pseudomonas aeruginosa CRPA BSI - model 51	368.73	419.12	479.26	2,817.61	3,566.26	4,370.71	3,224.51	3,993.12	4,817.67	5.21	6.29	7.37	0.45	0.52	0.59
Germany - Pseudomonas aeruginosa CRPA OTH - model 55	0.86	1.19	1.59	0.00	0.00	0.00	0.86	1.19	1.59	2.96E-03	3.64E-03	4.39E-03	1.06E-03	1.47E-03	1.96E-03
Germany - Pseudomonas aeruginosa CRPA RESP - model 52	923.15	1,085.62	1,255.28	3,060.11	3,844.58	4,738.51	4,049.98	4,934.79	5,917.72	1.78	2.08	2.37	1.14	1.34	1.55
Germany - Pseudomonas aeruginosa CRPA SSI - model 54	0.67	0.95	1.32	147.58	187.03	229.69	148.37	187.89	230.93	0.39	0.42	0.45	8.28E-04	1.17E-03	1.62E-03
Germany - Pseudomonas aeruginosa CRPA UTI - model 53	1.26	1.69	2.22	0.00	0.00	0.00	1.26	1.69	2.22	1.63E-03	2.05E-03	2.48E-03	1.55E-03	2.09E-03	2.73E-03
Germany - Pseudomonas aeruginosa MDRPA BSI - model 46	32.78	39.75	48.06	224.28	311.52	414.62	260.37	351.08	459.15	4.03	5.21	6.50	0.04	0.05	0.06
Germany - Pseudomonas aeruginosa MDRPA OTH - model 50	0.09	0.13	0.18	0.00	0.00	0.00	0.09	0.13	0.18	2.90E-03	3.75E-03	4.62E-03	1.06E-04	1.61E-04	2.27E-04
Germany - Pseudomonas aeruginosa MDRPA RESP - model 47	81.92	102.54	125.36	262.55	344.51	448.29	349.58	446.74	563.73	1.46	1.76	2.13	0.10	0.13	0.15
Germany - Pseudomonas aeruginosa MDRPA SSI - model 49	0.07	0.10	0.15	13.88	19.07	25.07	13.87	19.17	25.10	0.36	0.41	0.45	8.04E-05	1.23E-04	1.80E-04
Germany - Pseudomonas aeruginosa MDRPA UTI - model 48	0.13	0.18	0.25	0.00	0.00	0.00	0.13	0.18	0.25	1.58E-03	2.04E-03	2.56E-03	1.54E-04	2.23E-04	3.04E-04
Germany - Staphylococcus aureus MRSA BSI - model 61	1,004.52	1,121.82	1,234.78	6,835.22	7,509.22	8,150.49	7,865.50	8,633.22	9,353.04	3.56	3.74	3.95	1.24	1.38	1.52
Germany - Staphylococcus aureus MRSA OTH - model 65	8.81	11.19	14.12	0.00	0.00	0.00	8.81	11.19	14.12	2.59E-03	3.28E-03	3.97E-03	0.01	0.01	0.02
Germany - Staphylococcus aureus MRSA RESP - model 62	1,032.49	1,186.17	1,350.44	2,003.95	2,292.24	2,601.25	3,037.57	3,479.20	3,926.52	0.92	0.99	1.07	1.27	1.46	1.66
Germany - Staphylococcus aureus MRSA SSI - model 64	5.88	8.07	10.94	1,402.17	1,625.49	1,877.10	1,409.46	1,633.09	1,891.06	0.40	0.43	0.45	7.24E-03	9.94E-03	0.01
Germany - Staphylococcus aureus MRSA UTI - model 63	0.80	1.09	1.46	0.00	0.00	0.00	0.80	1.09	1.46	1.47E-03	1.87E-03	2.33E-03	9.88E-04	1.34E-03	1.79E-03
Germany - Streptococcus pneumoniae PMRSP BSI - model 76	5.58	7.22	9.21	41.90	48.61	56.96	48.41	55.90	64.97	4.09	4.59	5.12	6.87E-03	8.89E-03	0.01
Germany - Streptococcus pneumoniae PMRSP OTH - model 80	5.13E-03	8.54E-03	0.01	0.00	0.00	0.00	5.13E-03	8.54E-03	0.01	1.24E-03	1.95E-03	2.74E-03	6.32E-06	1.05E-05	1.63E-05
Germany - Streptococcus pneumoniae PMRSP RESP - model 77	12.81	19.19	32.18	25.13	37.73	61.87	38.63	57.03	93.64	0.88	1.20	1.62	0.02	0.02	0.04
Germany - Streptococcus pneumoniae PMRSP SSI - model 79	0.00	0.00	0.00	0.00	0.00	0.00	0.00	0.00	0.00	NaN	NaN	NaN	0.00	0.00	0.00
Germany - Streptococcus pneumoniae PMRSP UTI - model 78	0.00	0.00	0.00	0.00	0.00	0.00	0.00	0.00	0.00	NaN	NaN	NaN	0.00	0.00	0.00
Germany - Streptococcus pneumoniae PRSP BSI - model 71	38.19	44.54	52.25	275.05	303.45	335.15	317.13	348.01	383.05	8.89	9.54	10.28	0.05	0.05	0.06
Germany - Streptococcus pneumoniae PRSP OTH - model 75	0.02	0.03	0.03	0.00	0.00	0.00	0.02	0.03	0.03	1.46E-03	1.95E-03	2.51E-03	2.31E-05	3.17E-05	4.23E-05
Germany - Streptococcus pneumoniae PRSP RESP - model 72	92.78	123.39	168.07	184.39	241.13	327.21	278.62	365.51	492.87	2.14	2.50	2.97	0.11	0.15	0.21
Germany - Streptococcus pneumoniae PRSP SSI - model 74	0.00	0.00	0.00	0.00	0.00	0.00	0.00	0.00	0.00	NaN	NaN	NaN	0.00	0.00	0.00
Germany - Streptococcus pneumoniae PRSP UTI - model 73	0.00	0.00	0.00	0.00	0.00	0.00	0.00	0.00	0.00	NaN	NaN	NaN	0.00	0.00	0.00
**Total (Median & 95% UIs)**	6,776.30	7,821.59	9,022.76	37,898.82	44,339.05	51,440.82	44,887.87	52,181.40	60,163.53		-		8.35	9.63	11.11
**Total (average)**		**7,841.16**			**44,422.28**			**52,263.44**			-			**9.66**	

Model	YLL per 100,000			DALY per 100,000			Cases per year			Incidence per 100,000			Deaths per year			Mortality per 100,000		
Germany - Acinetobacter spp. ColRACI BSI - model 41	2.34E-02	4.77E-02	8.26E-02	2.95E-02	5.40E-02	9.05E-02	1.01E+01	1.29E+01	1.58E+01	1.25E-02	1.59E-02	1.95E-02	1.49E+00	2.67E+00	4.17E+00	1.83E-03	3.29E-03	5.13E-03
Germany - Acinetobacter spp. ColRACI OTH - model 45	0.00	0.00	0.00	7.83E-07	2.21E-06	4.26E-06	0.20	0.48	0.80	2.45E-04	5.92E-04	9.90E-04	0.00	0.00	0.00	0.00	0.00	0.00
Germany - Acinetobacter spp. ColRACI RESP - model 42	5.83E-03	9.09E-03	0.01	8.88E-03	0.01	0.02	9.85	13.99	18.65	0.01	0.02	0.02	0.34	0.50	0.68	4.23E-04	6.19E-04	8.35E-04
Germany - Acinetobacter spp. ColRACI SSI - model 44	8.89E-04	1.56E-03	2.34E-03	8.97E-04	1.57E-03	2.34E-03	2.79	4.82	7.16	3.43E-03	5.94E-03	8.82E-03	0.07	0.13	0.20	8.62E-05	1.59E-04	2.46E-04
Germany - Acinetobacter spp. ColRACI UTI - model 43	0.00	0.00	0.00	6.59E-06	1.35E-05	2.39E-05	3.16	5.46	7.79	3.89E-03	6.73E-03	9.59E-03	0.00	0.00	0.00	0.00	0.00	0.00
Germany - Acinetobacter spp. CRACI BSI - model 36	0.32	0.43	0.55	0.38	0.49	0.62	84.51	94.65	106.32	0.10	0.12	0.13	14.88	19.39	24.97	0.02	0.02	0.03
Germany - Acinetobacter spp. CRACI OTH - model 40	0.00	0.00	0.00	1.06E-05	1.65E-05	2.38E-05	2.48	3.60	4.80	3.05E-03	4.43E-03	5.91E-03	0.00	0.00	0.00	0.00	0.00	0.00
Germany - Acinetobacter spp. CRACI RESP - model 37	0.07	0.08	0.10	0.10	0.13	0.15	86.96	103.74	121.60	0.11	0.13	0.15	3.09	3.73	4.40	3.81E-03	4.59E-03	5.42E-03
Germany - Acinetobacter spp. CRACI SSI - model 39	0.01	0.02	0.02	0.01	0.02	0.02	28.01	35.65	43.81	0.03	0.04	0.05	0.78	1.02	1.28	9.65E-04	1.26E-03	1.57E-03
Germany - Acinetobacter spp. CRACI UTI - model 38	0.00	0.00	0.00	7.18E-05	9.98E-05	1.37E-04	31.16	39.90	48.21	0.04	0.05	0.06	0.00	0.00	0.00	0.00	0.00	0.00
Germany - Acinetobacter spp. MDRACI BSI - model 31	0.04	0.08	0.13	0.05	0.08	0.14	10.17	12.81	16.22	0.01	0.02	0.02	1.47	2.59	4.04	1.80E-03	3.19E-03	4.98E-03
Germany - Acinetobacter spp. MDRACI OTH - model 35	0.00	0.00	0.00	7.73E-07	2.08E-06	4.35E-06	0.20	0.47	0.81	2.46E-04	5.80E-04	9.95E-04	0.00	0.00	0.00	0.00	0.00	0.00
Germany - Acinetobacter spp. MDRACI RESP - model 32	8.78E-03	0.01	0.02	0.01	0.02	0.03	9.56	14.22	19.17	0.01	0.02	0.02	0.34	0.51	0.69	4.23E-04	6.27E-04	8.50E-04
Germany - Acinetobacter spp. MDRACI SSI - model 34	1.29E-03	2.43E-03	3.61E-03	1.30E-03	2.44E-03	3.63E-03	2.70	4.85	6.73	3.32E-03	5.97E-03	8.29E-03	0.07	0.13	0.19	8.24E-05	1.60E-04	2.37E-04
Germany - Acinetobacter spp. MDRACI UTI - model 33	0.00	0.00	0.00	6.45E-06	1.33E-05	2.47E-05	3.09	5.41	7.93	3.81E-03	6.66E-03	9.76E-03	0.00	0.00	0.00	0.00	0.00	0.00
Germany - Enterococcus faecalis and E. faecium VRE BSI - model 66	4.20	5.00	5.88	4.69	5.58	6.57	646.65	778.41	926.80	0.80	0.96	1.14	147.60	178.08	211.61	0.18	0.22	0.26
Germany - Enterococcus faecalis and E. faecium VRE OTH - model 70	0.00	0.00	0.00	1.24E-03	1.67E-03	2.21E-03	671.14	835.14	1,001.85	0.83	1.03	1.23	0.00	0.00	0.00	0.00	0.00	0.00
Germany - Enterococcus faecalis and E. faecium VRE RESP - model 67	0.00	0.00	0.00	0.00	0.00	0.00	0.00	0.00	0.00	0.00	0.00	0.00	0.00	0.00	0.00	0.00	0.00	0.00
Germany - Enterococcus faecalis and E. faecium VRE SSI - model 69	0.43	0.55	0.68	0.43	0.56	0.68	853.65	1,077.09	1,287.53	1.05	1.33	1.59	20.17	27.38	34.12	0.02	0.03	0.04
Germany - Enterococcus faecalis and E. faecium VRE UTI - model 68	0.00	0.00	0.00	6.75E-04	9.13E-04	1.20E-03	311.74	397.94	483.89	0.38	0.49	0.60	0.00	0.00	0.00	0.00	0.00	0.00
Germany - Escherichia coli 3GCREC BSI - model 16	12.74	14.33	16.07	14.76	16.55	18.47	3,516.29	3,923.04	4,408.07	4.33	4.83	5.43	584.41	677.28	781.21	0.72	0.83	0.96
Germany - Escherichia coli 3GCREC OTH - model 20	0.00	0.00	0.00	3.87E-03	5.52E-03	7.38E-03	1,036.87	1,341.48	1,653.45	1.28	1.65	2.04	0.00	0.00	0.00	0.00	0.00	0.00
Germany - Escherichia coli 3GCREC RESP - model 17	1.12	1.36	1.61	1.70	2.06	2.46	1,411.90	1,792.40	2,195.38	1.74	2.21	2.70	49.99	64.70	79.38	0.06	0.08	0.10
Germany - Escherichia coli 3GCREC SSI - model 19	1.72	2.06	2.43	1.73	2.07	2.44	3,473.15	4,227.64	4,960.32	4.28	5.21	6.11	100.58	125.94	150.78	0.12	0.16	0.19
Germany - Escherichia coli 3GCREC UTI - model 18	0.00	0.00	0.00	0.03	0.04	0.05	14,562.41	17,107.33	19,502.99	17.93	21.07	24.02	0.00	0.00	0.00	0.00	0.00	0.00
Germany - Escherichia coli ColREC BSI - model 26	1.08	1.40	1.72	1.14	1.46	1.81	101.38	118.09	135.36	0.12	0.15	0.17	54.93	71.33	88.84	0.07	0.09	0.11
Germany - Escherichia coli ColREC OTH - model 30	0.00	0.00	0.00	1.95E-04	2.98E-04	4.40E-04	53.65	69.04	86.84	0.07	0.09	0.11	0.00	0.00	0.00	0.00	0.00	0.00
Germany - Escherichia coli ColREC RESP - model 27	0.03	0.04	0.05	0.05	0.07	0.08	46.40	61.08	75.69	0.06	0.08	0.09	1.67	2.20	2.79	2.06E-03	2.71E-03	3.44E-03
Germany - Escherichia coli ColREC SSI - model 29	0.06	0.07	0.09	0.06	0.07	0.09	112.57	142.26	170.66	0.14	0.18	0.21	3.51	4.55	5.55	4.32E-03	5.60E-03	6.83E-03
Germany - Escherichia coli ColREC UTI - model 28	0.00	0.00	0.00	9.14E-04	1.29E-03	1.81E-03	411.80	501.17	601.95	0.51	0.62	0.74	0.00	0.00	0.00	0.00	0.00	0.00
Germany - Escherichia coli CREC BSI - model 21	0.06	0.09	0.13	0.06	0.10	0.14	10.08	13.04	16.51	0.01	0.02	0.02	3.07	4.62	6.66	3.78E-03	5.69E-03	8.20E-03
Germany - Escherichia coli CREC OTH - model 25	0.00	0.00	0.00	1.68E-05	3.27E-05	6.06E-05	4.71	7.76	10.97	5.80E-03	9.56E-03	0.01	0.00	0.00	0.00	0.00	0.00	0.00
Germany - Escherichia coli CREC RESP - model 22	2.65E-03	4.69E-03	7.21E-03	4.01E-03	7.18E-03	0.01	3.97	6.75	9.71	4.88E-03	8.32E-03	0.01	0.14	0.24	0.35	1.71E-04	2.98E-04	4.35E-04
Germany - Escherichia coli CREC SSI - model 24	4.20E-03	6.61E-03	9.09E-03	4.24E-03	6.66E-03	9.13E-03	9.97	15.66	21.46	0.01	0.02	0.03	0.26	0.42	0.59	3.22E-04	5.23E-04	7.30E-04
Germany - Escherichia coli CREC UTI - model 23	0.00	0.00	0.00	7.38E-05	1.36E-04	2.43E-04	37.45	55.79	72.90	0.05	0.07	0.09	0.00	0.00	0.00	0.00	0.00	0.00
Germany - Klebsiella pneumoniae 3GCRKP BSI - model 1	2.27	2.45	2.69	2.64	2.85	3.11	579.52	636.07	706.08	0.71	0.78	0.87	96.01	105.99	118.82	0.12	0.13	0.15
Germany - Klebsiella pneumoniae 3GCRKP OTH - model 5	0.00	0.00	0.00	7.77E-04	1.06E-03	1.42E-03	214.40	257.88	313.46	0.26	0.32	0.39	0.00	0.00	0.00	0.00	0.00	0.00
Germany - Klebsiella pneumoniae 3GCRKP RESP - model 2	0.40	0.47	0.53	0.61	0.71	0.80	472.94	559.07	657.16	0.58	0.69	0.81	16.78	20.09	23.65	0.02	0.02	0.03
Germany - Klebsiella pneumoniae 3GCRKP SSI - model 4	0.11	0.13	0.16	0.11	0.13	0.16	207.82	256.25	304.91	0.26	0.32	0.38	5.80	7.49	9.24	7.14E-03	9.22E-03	0.01
Germany - Klebsiella pneumoniae 3GCRKP UTI - model 3	0.00	0.00	0.00	1.36E-03	1.83E-03	2.33E-03	640.75	760.89	877.81	0.79	0.94	1.08	0.00	0.00	0.00	0.00	0.00	0.00
Germany - Klebsiella pneumoniae ColRKP BSI - model 11	0.58	0.77	1.01	0.61	0.81	1.05	37.20	43.92	51.17	0.05	0.05	0.06	20.42	26.50	33.69	0.03	0.03	0.04
Germany - Klebsiella pneumoniae ColRKP OTH - model 15	0.00	0.00	0.00	5.66E-05	9.13E-05	1.40E-04	14.95	21.41	27.60	0.02	0.03	0.03	0.00	0.00	0.00	0.00	0.00	0.00
Germany - Klebsiella pneumoniae ColRKP RESP - model 12	0.01	0.02	0.03	0.02	0.03	0.04	13.73	19.36	24.65	0.02	0.02	0.03	0.49	0.69	0.89	6.10E-04	8.54E-04	1.10E-03
Germany - Klebsiella pneumoniae ColRKP SSI - model 14	3.83E-03	5.67E-03	7.45E-03	3.86E-03	5.70E-03	7.50E-03	9.60	13.64	17.83	0.01	0.02	0.02	0.19	0.30	0.43	2.36E-04	3.74E-04	5.24E-04
Germany - Klebsiella pneumoniae ColRKP UTI - model 13	0.00	0.00	0.00	4.29E-05	6.83E-05	1.03E-04	19.80	27.28	34.64	0.02	0.03	0.04	0.00	0.00	0.00	0.00	0.00	0.00
Germany - Klebsiella pneumoniae CRKP BSI - model 6	0.07	0.11	0.20	0.07	0.12	0.21	6.69	8.69	11.54	8.24E-03	0.01	0.01	1.97	3.07	4.66	2.42E-03	3.78E-03	5.74E-03
Germany - Klebsiella pneumoniae CRKP OTH - model 10	0.00	0.00	0.00	7.28E-06	1.72E-05	3.43E-05	2.14	4.27	6.46	2.63E-03	5.26E-03	7.96E-03	0.00	0.00	0.00	0.00	0.00	0.00
Germany - Klebsiella pneumoniae CRKP RESP - model 7	1.99E-03	5.28E-03	9.16E-03	2.95E-03	7.94E-03	0.01	2.13	3.88	5.93	2.62E-03	4.78E-03	7.31E-03	0.07	0.14	0.22	8.89E-05	1.71E-04	2.68E-04
Germany - Klebsiella pneumoniae CRKP SSI - model 9	8.05E-04	1.53E-03	2.35E-03	8.08E-04	1.53E-03	2.36E-03	1.45	2.73	4.24	1.78E-03	3.37E-03	5.23E-03	0.03	0.06	0.09	4.00E-05	7.59E-05	1.15E-04
Germany - Klebsiella pneumoniae CRKP UTI - model 8	0.00	0.00	0.00	6.15E-06	1.36E-05	2.57E-05	2.76	5.50	8.13	3.40E-03	6.77E-03	0.01	0.00	0.00	0.00	0.00	0.00	0.00
Germany - Pseudomonas aeruginosa ColRPA BSI - model 56	0.00	0.00	0.00	0.00	0.00	0.00	0.00	0.00	0.00	0.00	0.00	0.00	0.00	0.00	0.00	0.00	0.00	0.00
Germany - Pseudomonas aeruginosa ColRPA OTH - model 60	0.00	0.00	0.00	0.00	0.00	0.00	0.00	0.00	0.00	0.00	0.00	0.00	0.00	0.00	0.00	0.00	0.00	0.00
Germany - Pseudomonas aeruginosa ColRPA RESP - model 57	0.00	0.00	0.00	0.00	0.00	0.00	0.00	0.00	0.00	0.00	0.00	0.00	0.00	0.00	0.00	0.00	0.00	0.00
Germany - Pseudomonas aeruginosa ColRPA SSI - model 59	0.00	0.00	0.00	0.00	0.00	0.00	0.00	0.00	0.00	0.00	0.00	0.00	0.00	0.00	0.00	0.00	0.00	0.00
Germany - Pseudomonas aeruginosa ColRPA UTI - model 58	0.00	0.00	0.00	0.00	0.00	0.00	0.00	0.00	0.00	0.00	0.00	0.00	0.00	0.00	0.00	0.00	0.00	0.00
Germany - Pseudomonas aeruginosa CRPA BSI - model 51	3.47	4.39	5.38	3.97	4.92	5.93	562.69	636.96	708.42	0.69	0.78	0.87	110.78	144.73	182.63	0.14	0.18	0.22
Germany - Pseudomonas aeruginosa CRPA OTH - model 55	0.00	0.00	0.00	1.06E-03	1.47E-03	1.96E-03	252.94	330.37	404.34	0.31	0.41	0.50	0.00	0.00	0.00	0.00	0.00	0.00
Germany - Pseudomonas aeruginosa CRPA RESP - model 52	3.77	4.73	5.84	4.99	6.08	7.29	2,011.89	2,380.26	2,741.98	2.48	2.93	3.38	122.38	155.41	193.43	0.15	0.19	0.24
Germany - Pseudomonas aeruginosa CRPA SSI - model 54	0.18	0.23	0.28	0.18	0.23	0.28	359.82	447.37	538.66	0.44	0.55	0.66	8.06	10.58	13.30	9.93E-03	0.01	0.02
Germany - Pseudomonas aeruginosa CRPA UTI - model 53	0.00	0.00	0.00	1.55E-03	2.09E-03	2.73E-03	685.35	833.27	993.94	0.84	1.03	1.22	0.00	0.00	0.00	0.00	0.00	0.00
Germany - Pseudomonas aeruginosa MDRPA BSI - model 46	0.28	0.38	0.51	0.32	0.43	0.57	58.04	67.82	78.52	0.07	0.08	0.10	10.34	14.30	18.32	0.01	0.02	0.02
Germany - Pseudomonas aeruginosa MDRPA OTH - model 50	0.00	0.00	0.00	1.06E-04	1.61E-04	2.27E-04	25.66	34.84	44.98	0.03	0.04	0.06	0.00	0.00	0.00	0.00	0.00	0.00
Germany - Pseudomonas aeruginosa MDRPA RESP - model 47	0.32	0.42	0.55	0.43	0.55	0.69	207.71	252.26	300.34	0.26	0.31	0.37	11.43	15.74	20.25	0.01	0.02	0.02
Germany - Pseudomonas aeruginosa MDRPA SSI - model 49	0.02	0.02	0.03	0.02	0.02	0.03	35.74	47.07	59.24	0.04	0.06	0.07	0.83	1.18	1.57	1.02E-03	1.46E-03	1.93E-03
Germany - Pseudomonas aeruginosa MDRPA UTI - model 48	0.00	0.00	0.00	1.54E-04	2.23E-04	3.04E-04	69.10	88.57	109.80	0.09	0.11	0.14	0.00	0.00	0.00	0.00	0.00	0.00
Germany - Staphylococcus aureus MRSA BSI - model 61	8.42	9.25	10.04	9.69	10.63	11.52	2,086.66	2,304.18	2,523.51	2.57	2.84	3.11	372.81	413.95	455.80	0.46	0.51	0.56
Germany - Staphylococcus aureus MRSA OTH - model 65	0.00	0.00	0.00	0.01	0.01	0.02	2,991.82	3,446.10	3,854.62	3.68	4.24	4.75	0.00	0.00	0.00	0.00	0.00	0.00
Germany - Staphylococcus aureus MRSA RESP - model 62	2.47	2.82	3.20	3.74	4.28	4.84	2,971.84	3,520.18	3,975.12	3.66	4.34	4.90	107.35	126.43	146.51	0.13	0.16	0.18
Germany - Staphylococcus aureus MRSA SSI - model 64	1.73	2.00	2.31	1.74	2.01	2.33	3,344.26	3,828.93	4,356.87	4.12	4.72	5.37	96.50	113.04	130.68	0.12	0.14	0.16
Germany - Staphylococcus aureus MRSA UTI - model 63	0.00	0.00	0.00	9.88E-04	1.34E-03	1.79E-03	469.97	584.09	699.07	0.58	0.72	0.86	0.00	0.00	0.00	0.00	0.00	0.00
Germany - Streptococcus pneumoniae PMRSP BSI - model 76	0.05	0.06	0.07	0.06	0.07	0.08	11.24	12.17	13.15	0.01	0.01	0.02	1.96	2.19	2.44	2.41E-03	2.70E-03	3.00E-03
Germany - Streptococcus pneumoniae PMRSP OTH - model 80	0.00	0.00	0.00	6.32E-06	1.05E-05	1.63E-05	3.58	4.44	5.53	4.41E-03	5.47E-03	6.81E-03	0.00	0.00	0.00	0.00	0.00	0.00
Germany - Streptococcus pneumoniae PMRSP RESP - model 77	0.03	0.05	0.08	0.05	0.07	0.12	35.28	48.38	67.92	0.04	0.06	0.08	1.26	1.75	2.47	1.56E-03	2.16E-03	3.04E-03
Germany - Streptococcus pneumoniae PMRSP SSI - model 79	0.00	0.00	0.00	0.00	0.00	0.00	0.00	0.00	0.00	0.00	0.00	0.00	0.00	0.00	0.00	0.00	0.00	0.00
Germany - Streptococcus pneumoniae PMRSP UTI - model 78	0.00	0.00	0.00	0.00	0.00	0.00	0.00	0.00	0.00	0.00	0.00	0.00	0.00	0.00	0.00	0.00	0.00	0.00
Germany - Streptococcus pneumoniae PRSP BSI - model 71	0.34	0.37	0.41	0.39	0.43	0.47	34.64	36.46	38.35	0.04	0.04	0.05	6.09	6.56	7.04	7.50E-03	8.08E-03	8.67E-03
Germany - Streptococcus pneumoniae PRSP OTH - model 75	0.00	0.00	0.00	2.31E-05	3.17E-05	4.23E-05	11.51	13.30	15.29	0.01	0.02	0.02	0.00	0.00	0.00	0.00	0.00	0.00
Germany - Streptococcus pneumoniae PRSP RESP - model 72	0.23	0.30	0.40	0.34	0.45	0.61	117.45	146.88	179.26	0.14	0.18	0.22	4.21	5.26	6.50	5.18E-03	6.47E-03	8.00E-03
Germany - Streptococcus pneumoniae PRSP SSI - model 74	0.00	0.00	0.00	0.00	0.00	0.00	0.00	0.00	0.00	0.00	0.00	0.00	0.00	0.00	0.00	0.00	0.00	0.00
Germany - Streptococcus pneumoniae PRSP UTI - model 73	0.00	0.00	0.00	0.00	0.00	0.00	0.00	0.00	0.00	0.00	0.00	0.00	0.00	0.00	0.00	0.00	0.00	0.00
**Total (Median & 95% UIs)**	46.67	54.61	63.35	55.28	64.26	74.10	46,133.78	54,508.80	62,808.71	56.82	67.13	77.35	1,984.63	2,362.88	2,775.08	2.44	2.91	3.42
**Total (average)**		**54.71**			**64.37**			**54,469.02**			**67.08**			**2,370.09**			**2.92**	

Infection	YLD			YLL			DALY			DALY per Case			YLD per 100,000			YLL per 100,000			DALY per 100,000			Cases per year			Incidence per 100,000			Deaths per year			Mortality per 100,000		
Colistin resistant *Acinetobacter spp.*	0.00	0.00	0.00	0.00	0.00	0.00	0.00	0.00	0.00	0.00	0.00	0.00	0.00	0.00	0.00	0.00	0.00	0.00	0.00	0.00	0.00	0.00	0.00	0.00	0.00	0.00	0.00	0.00	0.00	0.00	0.00	0.00	0.00	0.00	0.00	0.00
Carbapenem resistant *Acinetobacter spp.* (excluding those resistant to colistin)	3.31	4.35	5.63	13.23	23.34	34.17	17.20	27.66	39.51	5.97	9.10	12.07	0.06	0.08	0.10	0.23	0.41	0.60	0.30	0.49	0.70	6.75	8.95	11.30	0.12	0.16	0.20	0.46	0.78	1.13	0.01	0.01	0.02
Aminoglycosides and fluoroquinolones resistant *Acinetobacter* spp. (excluding those resistant to colistin and/or carbapenem)	0.00	0.00	0.00	0.00	0.00	0.00	0.00	0.00	0.00	0.00	0.00	0.00	0.00	0.00	0.00	0.00	0.00	0.00	0.00	0.00	0.00	0.00	0.00	0.00	0.00	0.00	0.00	0.00	0.00	0.00	0.00	0.00	0.00	0.00	0.00	0.00
Vancomycin resistant Enterococci (*E. faecalis* and *E. faecium*)	15.70	19.66	25.13	149.02	188.11	235.95	165.06	207.86	259.66	6.22	6.81	7.42	0.28	0.35	0.44	2.63	3.32	4.17	2.92	3.67	4.59	87.54	117.98	148.87	1.55	2.08	2.63	6.09	7.83	9.87	0.11	0.14	0.17
Colistin resistant *Escherichia coli*	0.00	0.00	0.00	0.00	0.00	0.00	0.00	0.00	0.00	0.00	0.00	0.00	0.00	0.00	0.00	0.00	0.00	0.00	0.00	0.00	0.00	0.00	0.00	0.00	0.00	0.00	0.00	0.00	0.00	0.00	0.00	0.00	0.00	0.00	0.00	0.00
Carbapenem resistant *Escherichia coli* (excluding those resistant to colistin)	1.07	1.84	2.88	10.44	20.32	31.43	11.82	22.18	33.87	14.32	22.30	29.66	0.02	0.03	0.05	0.18	0.36	0.56	0.21	0.39	0.60	4.59	7.90	11.07	0.08	0.14	0.20	0.21	0.41	0.64	0.00	0.01	0.01
Third-generation cephalosporin resistant *Escherichia coli* (excluding those resistant to colistin and/or carbapenem)	223.14	250.94	281.95	1,315.52	1,485.53	1,670.77	1,544.11	1,735.97	1,943.38	4.99	5.44	5.92	3.94	4.43	4.98	23.24	26.25	29.52	27.28	30.67	34.34	2,247.34	2,549.71	2,837.55	39.71	45.05	50.14	68.26	77.73	88.72	1.21	1.37	1.57
Colistin resistant *Klebsiella pneumoniae*	0.00	0.00	0.00	0.00	0.00	0.00	0.00	0.00	0.00	0.00	0.00	0.00	0.00	0.00	0.00	0.00	0.00	0.00	0.00	0.00	0.00	0.00	0.00	0.00	0.00	0.00	0.00	0.00	0.00	0.00	0.00	0.00	0.00	0.00	0.00	0.00
Carbapenem resistant *Klebsiella pneumoniae* (excluding those resistant to colistin)	0.00	0.00	0.00	0.00	0.00	0.00	0.00	0.00	0.00	0.00	0.00	0.00	0.00	0.00	0.00	0.00	0.00	0.00	0.00	0.00	0.00	0.00	0.00	0.00	0.00	0.00	0.00	0.00	0.00	0.00	0.00	0.00	0.00	0.00	0.00	0.00
Third-generation cephalosporin resistant *Klebsiella pneumoniae* (excluding those resistant to colistin and/or carbapenem)	53.53	60.28	67.46	265.30	288.86	313.87	321.35	349.03	378.63	4.90	5.16	5.44	0.95	1.07	1.19	4.69	5.10	5.55	5.68	6.17	6.69	252.26	280.89	309.77	4.46	4.96	5.47	14.01	15.31	16.66	0.25	0.27	0.29
Colistin resistant *Pseudomonas aeruginosa*	0.00	0.00	0.00	0.00	0.00	0.00	0.00	0.00	0.00	0.00	0.00	0.00	0.00	0.00	0.00	0.00	0.00	0.00	0.00	0.00	0.00	0.00	0.00	0.00	0.00	0.00	0.00	0.00	0.00	0.00	0.00	0.00	0.00	0.00	0.00	0.00
Carbapenem resistant *Pseudomonas aeruginosa* (excluding those resistant to colistin)	48.66	57.07	66.69	211.89	287.56	370.10	266.92	344.82	432.71	7.78	9.88	11.99	0.86	1.01	1.18	3.74	5.08	6.54	4.72	6.09	7.65	130.21	154.08	178.17	2.30	2.72	3.15	7.91	10.47	13.13	0.14	0.19	0.23
Pseudomonas aeruginosa resistant to three or more antimicrobial groups (excluding those resistant to colistin and/or carbapenem)	2.19	3.60	5.39	7.35	16.69	30.25	10.13	20.32	34.88	3.08	5.88	9.10	0.04	0.06	0.10	0.13	0.29	0.53	0.18	0.36	0.62	9.79	15.48	21.11	0.17	0.27	0.37	0.47	0.97	1.55	0.01	0.02	0.03
Methicillin resistant *Staphylococcus aureus*	30.36	34.85	39.33	151.30	166.02	181.60	182.77	201.01	220.10	5.39	5.74	6.11	0.54	0.62	0.69	2.67	2.93	3.21	3.23	3.55	3.89	161.57	180.10	198.31	2.85	3.18	3.50	7.72	8.47	9.25	0.14	0.15	0.16
Penicillin and macrolides resistant *Streptococcus pneumoniae* (excluding those only resistant to penicillin)	4.55	5.76	7.50	16.00	18.82	22.51	20.90	24.61	29.63	4.76	5.20	5.66	0.08	0.10	0.13	0.28	0.33	0.40	0.37	0.43	0.52	13.05	15.39	18.72	0.23	0.27	0.33	0.81	0.95	1.11	0.01	0.02	0.02
Penicillin tesistant *Streptococcus pneumoniae* (excluding those resistant to macrolides)	5.29	6.65	8.77	18.69	21.76	25.94	24.44	28.44	33.88	4.11	4.51	4.91	0.09	0.12	0.15	0.33	0.38	0.46	0.43	0.50	0.60	17.64	20.49	24.70	0.31	0.36	0.44	1.10	1.26	1.47	0.02	0.02	0.03
Total	387.79	445.00	510.74	2,158.73	2,517.01	2,916.60	2,564.70	2,961.91	3,406.25				6.85	7.86	9.02	38.14	44.47	51.53	45.31	52.33	60.18	2,930.74	3,350.95	3,759.56	51.78	59.21	66.43	107.06	124.17	143.53	1.89	2.19	2.54

Model	YLD			YLL			DALY			DALY per Case			YLD per 100,000			YLL per 100,000			DALY per 100,000			Cases per year			Incidence per 100,000			Deaths per year			Mortality per 100,000		
Denmark - Acinetobacter spp. ColRACI BSI - model 41	0.00	0.00	0.00	0.00	0.00	0.00	0.00	0.00	0.00	NaN	NaN	NaN	0.00	0.00	0.00	0.00	0.00	0.00	0.00	0.00	0.00	0.00	0.00	0.00	0.00	0.00	0.00	0.00	0.00	0.00	0.00	0.00	0.00
Denmark - Acinetobacter spp. ColRACI OTH - model 45	0.00	0.00	0.00	0.00	0.00	0.00	0.00	0.00	0.00	NaN	NaN	NaN	0.00	0.00	0.00	0.00	0.00	0.00	0.00	0.00	0.00	0.00	0.00	0.00	0.00	0.00	0.00	0.00	0.00	0.00	0.00	0.00	0.00
Denmark - Acinetobacter spp. ColRACI RESP - model 42	0.00	0.00	0.00	0.00	0.00	0.00	0.00	0.00	0.00	NaN	NaN	NaN	0.00	0.00	0.00	0.00	0.00	0.00	0.00	0.00	0.00	0.00	0.00	0.00	0.00	0.00	0.00	0.00	0.00	0.00	0.00	0.00	0.00
Denmark - Acinetobacter spp. ColRACI SSI - model 44	0.00	0.00	0.00	0.00	0.00	0.00	0.00	0.00	0.00	NaN	NaN	NaN	0.00	0.00	0.00	0.00	0.00	0.00	0.00	0.00	0.00	0.00	0.00	0.00	0.00	0.00	0.00	0.00	0.00	0.00	0.00	0.00	0.00
Denmark - Acinetobacter spp. ColRACI UTI - model 43	0.00	0.00	0.00	0.00	0.00	0.00	0.00	0.00	0.00	NaN	NaN	NaN	0.00	0.00	0.00	0.00	0.00	0.00	0.00	0.00	0.00	0.00	0.00	0.00	0.00	0.00	0.00	0.00	0.00	0.00	0.00	0.00	0.00
Denmark - Acinetobacter spp. CRACI BSI - model 36	1.98	2.48	3.16	10.24	19.16	28.73	12.82	21.61	31.64	4.12	6.98	9.65	0.04	0.04	0.06	0.18	0.34	0.51	0.23	0.38	0.56	2.68	3.10	3.61	0.05	0.05	0.06	0.36	0.63	0.94	6.32E-03	0.01	0.02
Denmark - Acinetobacter spp. CRACI OTH - model 40	1.67E-04	4.06E-04	7.91E-04	0.00	0.00	0.00	1.67E-04	4.06E-04	7.91E-04	2.19E-03	3.75E-03	5.47E-03	2.95E-06	7.17E-06	1.40E-05	0.00	0.00	0.00	2.95E-06	7.17E-06	1.40E-05	0.05	0.11	0.18	8.58E-04	1.99E-03	3.26E-03	0.00	0.00	0.00	0.00	0.00	0.00
Denmark - Acinetobacter spp. CRACI RESP - model 37	1.32	1.87	2.46	2.67	3.65	4.72	4.05	5.53	7.14	1.47	1.65	1.86	0.02	0.03	0.04	0.05	0.06	0.08	0.07	0.10	0.13	2.50	3.34	4.21	0.04	0.06	0.07	0.09	0.12	0.15	1.55E-03	2.12E-03	2.74E-03
Denmark - Acinetobacter spp. CRACI SSI - model 39	1.09E-03	2.33E-03	4.43E-03	0.32	0.53	0.72	0.32	0.53	0.73	0.38	0.46	0.55	1.93E-05	4.11E-05	7.84E-05	5.60E-03	9.30E-03	0.01	5.67E-03	9.33E-03	0.01	0.72	1.13	1.53	0.01	0.02	0.03	0.01	0.02	0.03	1.98E-04	3.57E-04	5.05E-04
Denmark - Acinetobacter spp. CRACI UTI - model 38	1.30E-03	2.56E-03	4.25E-03	0.00	0.00	0.00	1.30E-03	2.56E-03	4.25E-03	1.20E-03	2.05E-03	3.04E-03	2.30E-05	4.52E-05	7.51E-05	0.00	0.00	0.00	2.30E-05	4.52E-05	7.51E-05	0.80	1.27	1.77	0.01	0.02	0.03	0.00	0.00	0.00	0.00	0.00	0.00
Denmark - Acinetobacter spp. MDRACI BSI - model 31	0.00	0.00	0.00	0.00	0.00	0.00	0.00	0.00	0.00	NaN	NaN	NaN	0.00	0.00	0.00	0.00	0.00	0.00	0.00	0.00	0.00	0.00	0.00	0.00	0.00	0.00	0.00	0.00	0.00	0.00	0.00	0.00	0.00
Denmark - Acinetobacter spp. MDRACI OTH - model 35	0.00	0.00	0.00	0.00	0.00	0.00	0.00	0.00	0.00	NaN	NaN	NaN	0.00	0.00	0.00	0.00	0.00	0.00	0.00	0.00	0.00	0.00	0.00	0.00	0.00	0.00	0.00	0.00	0.00	0.00	0.00	0.00	0.00
Denmark - Acinetobacter spp. MDRACI RESP - model 32	0.00	0.00	0.00	0.00	0.00	0.00	0.00	0.00	0.00	NaN	NaN	NaN	0.00	0.00	0.00	0.00	0.00	0.00	0.00	0.00	0.00	0.00	0.00	0.00	0.00	0.00	0.00	0.00	0.00	0.00	0.00	0.00	0.00
Denmark - Acinetobacter spp. MDRACI SSI - model 34	0.00	0.00	0.00	0.00	0.00	0.00	0.00	0.00	0.00	NaN	NaN	NaN	0.00	0.00	0.00	0.00	0.00	0.00	0.00	0.00	0.00	0.00	0.00	0.00	0.00	0.00	0.00	0.00	0.00	0.00	0.00	0.00	0.00
Denmark - Acinetobacter spp. MDRACI UTI - model 33	0.00	0.00	0.00	0.00	0.00	0.00	0.00	0.00	0.00	NaN	NaN	NaN	0.00	0.00	0.00	0.00	0.00	0.00	0.00	0.00	0.00	0.00	0.00	0.00	0.00	0.00	0.00	0.00	0.00	0.00	0.00	0.00	0.00
Denmark - Enterococcus faecalis and E. faecium VRE BSI - model 66	15.60	19.49	24.88	135.43	167.54	208.32	151.34	187.11	231.67	5.78	6.31	6.85	0.28	0.34	0.44	2.39	2.96	3.68	2.67	3.31	4.09	23.93	29.60	36.88	0.42	0.52	0.65	5.43	6.78	8.45	0.10	0.12	0.15
Denmark - Enterococcus faecalis and E. faecium VRE OTH - model 70	0.03	0.05	0.08	0.00	0.00	0.00	0.03	0.05	0.08	1.20E-03	1.65E-03	2.24E-03	6.12E-04	9.13E-04	1.37E-03	0.00	0.00	0.00	6.12E-04	9.13E-04	1.37E-03	23.37	31.72	39.78	0.41	0.56	0.70	0.00	0.00	0.00	0.00	0.00	0.00
Denmark - Enterococcus faecalis and E. faecium VRE RESP - model 67	0.00	0.00	0.00	0.00	0.00	0.00	0.00	0.00	0.00	NaN	NaN	NaN	0.00	0.00	0.00	0.00	0.00	0.00	0.00	0.00	0.00	0.00	0.00	0.00	0.00	0.00	0.00	0.00	0.00	0.00	0.00	0.00	0.00
Denmark - Enterococcus faecalis and E. faecium VRE SSI - model 69	0.05	0.08	0.13	13.59	20.57	27.63	13.66	20.67	27.87	0.44	0.50	0.56	9.56E-04	1.49E-03	2.30E-03	0.24	0.36	0.49	0.24	0.37	0.49	29.31	41.35	52.32	0.52	0.73	0.92	0.66	1.05	1.42	0.01	0.02	0.03
Denmark - Enterococcus faecalis and E. faecium VRE UTI - model 68	0.02	0.03	0.04	0.00	0.00	0.00	0.02	0.03	0.04	1.39E-03	1.86E-03	2.46E-03	3.28E-04	4.98E-04	7.49E-04	0.00	0.00	0.00	3.28E-04	4.98E-04	7.49E-04	10.93	15.30	19.88	0.19	0.27	0.35	0.00	0.00	0.00	0.00	0.00	0.00
Denmark - Escherichia coli 3GCREC BSI - model 16	171.26	188.83	208.24	1,086.16	1,215.02	1,358.68	1,262.53	1,402.97	1,558.38	3.61	3.94	4.28	3.03	3.34	3.68	19.19	21.47	24.01	22.31	24.79	27.53	332.12	356.84	385.75	5.87	6.30	6.82	54.78	61.66	69.98	0.97	1.09	1.24
Denmark - Escherichia coli 3GCREC OTH - model 20	0.29	0.39	0.52	0.00	0.00	0.00	0.29	0.39	0.52	2.66E-03	3.37E-03	4.06E-03	5.11E-03	6.96E-03	9.22E-03	0.00	0.00	0.00	5.11E-03	6.96E-03	9.22E-03	95.99	118.04	139.32	1.70	2.09	2.46	0.00	0.00	0.00	0.00	0.00	0.00
Denmark - Escherichia coli 3GCREC RESP - model 17	48.61	57.91	68.41	93.93	112.12	130.93	142.76	170.29	198.67	0.98	1.07	1.19	0.86	1.02	1.21	1.66	1.98	2.31	2.52	3.01	3.51	131.17	158.34	186.44	2.32	2.80	3.29	4.67	5.68	6.79	0.08	0.10	0.12
Denmark - Escherichia coli 3GCREC SSI - model 19	0.59	0.81	1.06	135.43	158.39	181.16	136.13	159.32	182.09	0.39	0.42	0.45	0.01	0.01	0.02	2.39	2.80	3.20	2.41	2.81	3.22	330.05	377.96	428.11	5.83	6.68	7.56	8.82	10.39	11.96	0.16	0.18	0.21
Denmark - Escherichia coli 3GCREC UTI - model 18	2.39	3.00	3.72	0.00	0.00	0.00	2.39	3.00	3.72	1.59E-03	1.96E-03	2.34E-03	0.04	0.05	0.07	0.00	0.00	0.00	0.04	0.05	0.07	1,358.01	1,538.52	1,697.93	23.99	27.18	30.00	0.00	0.00	0.00	0.00	0.00	0.00
Denmark - Escherichia coli ColREC BSI - model 26	0.00	0.00	0.00	0.00	0.00	0.00	0.00	0.00	0.00	NaN	NaN	NaN	0.00	0.00	0.00	0.00	0.00	0.00	0.00	0.00	0.00	0.00	0.00	0.00	0.00	0.00	0.00	0.00	0.00	0.00	0.00	0.00	0.00
Denmark - Escherichia coli ColREC OTH - model 30	0.00	0.00	0.00	0.00	0.00	0.00	0.00	0.00	0.00	NaN	NaN	NaN	0.00	0.00	0.00	0.00	0.00	0.00	0.00	0.00	0.00	0.00	0.00	0.00	0.00	0.00	0.00	0.00	0.00	0.00	0.00	0.00	0.00
Denmark - Escherichia coli ColREC RESP - model 27	0.00	0.00	0.00	0.00	0.00	0.00	0.00	0.00	0.00	NaN	NaN	NaN	0.00	0.00	0.00	0.00	0.00	0.00	0.00	0.00	0.00	0.00	0.00	0.00	0.00	0.00	0.00	0.00	0.00	0.00	0.00	0.00	0.00
Denmark - Escherichia coli ColREC SSI - model 29	0.00	0.00	0.00	0.00	0.00	0.00	0.00	0.00	0.00	NaN	NaN	NaN	0.00	0.00	0.00	0.00	0.00	0.00	0.00	0.00	0.00	0.00	0.00	0.00	0.00	0.00	0.00	0.00	0.00	0.00	0.00	0.00	0.00
Denmark - Escherichia coli ColREC UTI - model 28	0.00	0.00	0.00	0.00	0.00	0.00	0.00	0.00	0.00	NaN	NaN	NaN	0.00	0.00	0.00	0.00	0.00	0.00	0.00	0.00	0.00	0.00	0.00	0.00	0.00	0.00	0.00	0.00	0.00	0.00	0.00	0.00	0.00
Denmark - Escherichia coli CREC BSI - model 21	0.88	1.35	2.04	9.77	18.84	29.08	10.96	20.20	30.71	11.60	19.19	26.17	0.02	0.02	0.04	0.17	0.33	0.51	0.19	0.36	0.54	0.81	1.05	1.37	0.01	0.02	0.02	0.20	0.38	0.59	3.52E-03	6.79E-03	0.01
Denmark - Escherichia coli CREC OTH - model 25	5.12E-04	1.89E-03	5.00E-03	0.00	0.00	0.00	5.12E-04	1.89E-03	5.00E-03	1.19E-03	3.31E-03	7.16E-03	9.04E-06	3.34E-05	8.83E-05	0.00	0.00	0.00	9.04E-06	3.34E-05	8.83E-05	0.26	0.59	0.92	4.54E-03	0.01	0.02	0.00	0.00	0.00	0.00	0.00	0.00
Denmark - Escherichia coli CREC RESP - model 22	0.18	0.48	0.82	0.36	0.93	1.55	0.55	1.42	2.33	2.28	2.66	3.03	3.17E-03	8.42E-03	0.01	6.42E-03	0.02	0.03	9.77E-03	0.03	0.04	0.21	0.54	0.85	3.79E-03	9.50E-03	0.01	7.41E-03	0.02	0.03	1.31E-04	3.34E-04	5.57E-04
Denmark - Escherichia coli CREC SSI - model 24	5.21E-04	2.43E-03	6.52E-03	0.30	0.55	0.81	0.30	0.55	0.81	0.44	0.44	0.45	9.21E-06	4.30E-05	1.15E-04	5.25E-03	9.78E-03	0.01	5.29E-03	9.80E-03	0.01	0.67	1.25	1.83	0.01	0.02	0.03	6.06E-03	0.01	0.02	1.07E-04	1.99E-04	2.92E-04
Denmark - Escherichia coli CREC UTI - model 23	3.15E-03	8.32E-03	0.02	0.00	0.00	0.00	3.15E-03	8.32E-03	0.02	7.79E-04	1.95E-03	3.97E-03	5.56E-05	1.47E-04	3.56E-04	0.00	0.00	0.00	5.56E-05	1.47E-04	3.56E-04	2.64	4.47	6.10	0.05	0.08	0.11	0.00	0.00	0.00	0.00	0.00	0.00
Denmark - Klebsiella pneumoniae 3GCRKP BSI - model 1	33.57	37.41	41.56	217.01	233.47	251.25	252.66	270.80	290.76	3.52	3.70	3.88	0.59	0.66	0.73	3.83	4.13	4.44	4.46	4.78	5.14	69.67	73.32	77.81	1.23	1.30	1.37	11.34	12.23	13.19	0.20	0.22	0.23
Denmark - Klebsiella pneumoniae 3GCRKP RESP - model 2	19.71	22.55	25.48	38.16	43.45	49.01	58.31	65.97	73.88	0.98	1.04	1.12	0.35	0.40	0.45	0.67	0.77	0.87	1.03	1.17	1.31	56.38	63.18	70.43	1.00	1.12	1.24	2.00	2.28	2.56	0.04	0.04	0.05
Denmark - Klebsiella pneumoniae 3GCRKP UTI - model 3	0.13	0.17	0.22	0.00	0.00	0.00	0.13	0.17	0.22	1.56E-03	1.96E-03	2.42E-03	2.32E-03	2.99E-03	3.83E-03	0.00	0.00	0.00	2.32E-03	2.99E-03	3.83E-03	76.23	86.53	96.06	1.35	1.53	1.70	0.00	0.00	0.00	0.00	0.00	0.00
Denmark - Klebsiella pneumoniae 3GCRKP SSI - model 4	0.04	0.06	0.08	10.13	11.94	13.61	10.17	12.00	13.65	0.40	0.42	0.44	7.73E-04	1.08E-03	1.43E-03	0.18	0.21	0.24	0.18	0.21	0.24	24.79	28.75	32.36	0.44	0.51	0.57	0.67	0.80	0.92	0.01	0.01	0.02
Denmark - Klebsiella pneumoniae 3GCRKP OTH - model 5	0.07	0.10	0.12	0.00	0.00	0.00	0.07	0.10	0.12	2.65E-03	3.36E-03	4.14E-03	1.29E-03	1.73E-03	2.18E-03	0.00	0.00	0.00	1.29E-03	1.73E-03	2.18E-03	25.19	29.11	33.12	0.45	0.51	0.59	0.00	0.00	0.00	0.00	0.00	0.00
Denmark - Klebsiella pneumoniae ColRKP BSI - model 11	0.00	0.00	0.00	0.00	0.00	0.00	0.00	0.00	0.00	NaN	NaN	NaN	0.00	0.00	0.00	0.00	0.00	0.00	0.00	0.00	0.00	0.00	0.00	0.00	0.00	0.00	0.00	0.00	0.00	0.00	0.00	0.00	0.00
Denmark - Klebsiella pneumoniae ColRKP OTH - model 15	0.00	0.00	0.00	0.00	0.00	0.00	0.00	0.00	0.00	NaN	NaN	NaN	0.00	0.00	0.00	0.00	0.00	0.00	0.00	0.00	0.00	0.00	0.00	0.00	0.00	0.00	0.00	0.00	0.00	0.00	0.00	0.00	0.00
Denmark - Klebsiella pneumoniae ColRKP RESP - model 12	0.00	0.00	0.00	0.00	0.00	0.00	0.00	0.00	0.00	NaN	NaN	NaN	0.00	0.00	0.00	0.00	0.00	0.00	0.00	0.00	0.00	0.00	0.00	0.00	0.00	0.00	0.00	0.00	0.00	0.00	0.00	0.00	0.00
Denmark - Klebsiella pneumoniae ColRKP SSI - model 14	0.00	0.00	0.00	0.00	0.00	0.00	0.00	0.00	0.00	NaN	NaN	NaN	0.00	0.00	0.00	0.00	0.00	0.00	0.00	0.00	0.00	0.00	0.00	0.00	0.00	0.00	0.00	0.00	0.00	0.00	0.00	0.00	0.00
Denmark - Klebsiella pneumoniae ColRKP UTI - model 13	0.00	0.00	0.00	0.00	0.00	0.00	0.00	0.00	0.00	NaN	NaN	NaN	0.00	0.00	0.00	0.00	0.00	0.00	0.00	0.00	0.00	0.00	0.00	0.00	0.00	0.00	0.00	0.00	0.00	0.00	0.00	0.00	0.00
Denmark - Klebsiella pneumoniae CRKP BSI - model 6	0.00	0.00	0.00	0.00	0.00	0.00	0.00	0.00	0.00	NaN	NaN	NaN	0.00	0.00	0.00	0.00	0.00	0.00	0.00	0.00	0.00	0.00	0.00	0.00	0.00	0.00	0.00	0.00	0.00	0.00	0.00	0.00	0.00
Denmark - Klebsiella pneumoniae CRKP OTH - model 10	0.00	0.00	0.00	0.00	0.00	0.00	0.00	0.00	0.00	NaN	NaN	NaN	0.00	0.00	0.00	0.00	0.00	0.00	0.00	0.00	0.00	0.00	0.00	0.00	0.00	0.00	0.00	0.00	0.00	0.00	0.00	0.00	0.00
Denmark - Klebsiella pneumoniae CRKP RESP - model 7	0.00	0.00	0.00	0.00	0.00	0.00	0.00	0.00	0.00	NaN	NaN	NaN	0.00	0.00	0.00	0.00	0.00	0.00	0.00	0.00	0.00	0.00	0.00	0.00	0.00	0.00	0.00	0.00	0.00	0.00	0.00	0.00	0.00
Denmark - Klebsiella pneumoniae CRKP SSI - model 9	0.00	0.00	0.00	0.00	0.00	0.00	0.00	0.00	0.00	NaN	NaN	NaN	0.00	0.00	0.00	0.00	0.00	0.00	0.00	0.00	0.00	0.00	0.00	0.00	0.00	0.00	0.00	0.00	0.00	0.00	0.00	0.00	0.00
Denmark - Klebsiella pneumoniae CRKP UTI - model 8	0.00	0.00	0.00	0.00	0.00	0.00	0.00	0.00	0.00	NaN	NaN	NaN	0.00	0.00	0.00	0.00	0.00	0.00	0.00	0.00	0.00	0.00	0.00	0.00	0.00	0.00	0.00	0.00	0.00	0.00	0.00	0.00	0.00
Denmark - Pseudomonas aeruginosa ColRPA BSI - model 56	0.00	0.00	0.00	0.00	0.00	0.00	0.00	0.00	0.00	NaN	NaN	NaN	0.00	0.00	0.00	0.00	0.00	0.00	0.00	0.00	0.00	0.00	0.00	0.00	0.00	0.00	0.00	0.00	0.00	0.00	0.00	0.00	0.00
Denmark - Pseudomonas aeruginosa ColRPA OTH - model 60	0.00	0.00	0.00	0.00	0.00	0.00	0.00	0.00	0.00	NaN	NaN	NaN	0.00	0.00	0.00	0.00	0.00	0.00	0.00	0.00	0.00	0.00	0.00	0.00	0.00	0.00	0.00	0.00	0.00	0.00	0.00	0.00	0.00
Denmark - Pseudomonas aeruginosa ColRPA RESP - model 57	0.00	0.00	0.00	0.00	0.00	0.00	0.00	0.00	0.00	NaN	NaN	NaN	0.00	0.00	0.00	0.00	0.00	0.00	0.00	0.00	0.00	0.00	0.00	0.00	0.00	0.00	0.00	0.00	0.00	0.00	0.00	0.00	0.00
Denmark - Pseudomonas aeruginosa ColRPA SSI - model 59	0.00	0.00	0.00	0.00	0.00	0.00	0.00	0.00	0.00	NaN	NaN	NaN	0.00	0.00	0.00	0.00	0.00	0.00	0.00	0.00	0.00	0.00	0.00	0.00	0.00	0.00	0.00	0.00	0.00	0.00	0.00	0.00	0.00
Denmark - Pseudomonas aeruginosa ColRPA UTI - model 58	0.00	0.00	0.00	0.00	0.00	0.00	0.00	0.00	0.00	NaN	NaN	NaN	0.00	0.00	0.00	0.00	0.00	0.00	0.00	0.00	0.00	0.00	0.00	0.00	0.00	0.00	0.00	0.00	0.00	0.00	0.00	0.00	0.00
Denmark - Pseudomonas aeruginosa CRPA BSI - model 51	13.91	16.01	18.53	99.30	136.31	176.27	114.80	152.39	192.86	5.45	7.09	8.69	0.25	0.28	0.33	1.75	2.41	3.11	2.03	2.69	3.41	19.33	21.51	23.79	0.34	0.38	0.42	3.73	4.91	6.18	0.07	0.09	0.11
Denmark - Pseudomonas aeruginosa CRPA OTH - model 55	0.03	0.04	0.05	0.00	0.00	0.00	0.03	0.04	0.05	2.90E-03	3.66E-03	4.54E-03	4.97E-04	6.99E-04	9.45E-04	0.00	0.00	0.00	4.97E-04	6.99E-04	9.45E-04	8.60	10.87	13.08	0.15	0.19	0.23	0.00	0.00	0.00	0.00	0.00	0.00
Denmark - Pseudomonas aeruginosa CRPA RESP - model 52	34.65	40.93	47.99	107.62	144.72	185.94	147.06	185.77	231.82	1.92	2.34	2.80	0.61	0.72	0.85	1.90	2.56	3.29	2.60	3.28	4.10	67.79	79.36	91.07	1.20	1.40	1.61	3.93	5.23	6.54	0.07	0.09	0.12
Denmark - Pseudomonas aeruginosa CRPA SSI - model 54	0.02	0.03	0.05	4.97	6.53	7.88	4.99	6.56	7.90	0.41	0.45	0.49	3.57E-04	5.49E-04	8.02E-04	0.09	0.12	0.14	0.09	0.12	0.14	11.83	14.72	17.72	0.21	0.26	0.31	0.26	0.33	0.41	4.54E-03	5.85E-03	7.19E-03
Denmark - Pseudomonas aeruginosa CRPA UTI - model 53	0.04	0.06	0.07	0.00	0.00	0.00	0.04	0.06	0.07	1.61E-03	2.03E-03	2.52E-03	7.26E-04	9.91E-04	1.30E-03	0.00	0.00	0.00	7.26E-04	9.91E-04	1.30E-03	22.66	27.61	32.50	0.40	0.49	0.57	0.00	0.00	0.00	0.00	0.00	0.00
Denmark - Pseudomonas aeruginosa MDRPA BSI - model 46	0.70	1.02	1.49	3.29	8.03	14.31	4.28	9.01	15.35	2.06	4.25	6.63	0.01	0.02	0.03	0.06	0.14	0.25	0.08	0.16	0.27	1.69	2.15	2.70	0.03	0.04	0.05	0.21	0.45	0.72	3.67E-03	7.94E-03	0.01
Denmark - Pseudomonas aeruginosa MDRPA OTH - model 50	1.53E-03	3.91E-03	7.60E-03	0.00	0.00	0.00	1.53E-03	3.91E-03	7.60E-03	1.85E-03	3.71E-03	5.82E-03	2.71E-05	6.90E-05	1.34E-04	0.00	0.00	0.00	2.71E-05	6.90E-05	1.34E-04	0.56	1.11	1.66	9.90E-03	0.02	0.03	0.00	0.00	0.00	0.00	0.00	0.00
Denmark - Pseudomonas aeruginosa MDRPA RESP - model 47	1.48	2.57	3.88	3.89	8.33	15.44	5.68	10.97	19.01	0.82	1.40	2.21	0.03	0.05	0.07	0.07	0.15	0.27	0.10	0.19	0.34	5.26	7.95	10.66	0.09	0.14	0.19	0.25	0.49	0.78	4.43E-03	8.64E-03	0.01
Denmark - Pseudomonas aeruginosa MDRPA SSI - model 49	9.94E-04	2.97E-03	6.88E-03	0.16	0.33	0.50	0.16	0.33	0.51	0.20	0.23	0.25	1.76E-05	5.24E-05	1.21E-04	2.86E-03	5.82E-03	8.80E-03	2.88E-03	5.88E-03	9.03E-03	0.70	1.47	2.19	0.01	0.03	0.04	0.01	0.03	0.05	2.49E-04	5.84E-04	9.11E-04
Denmark - Pseudomonas aeruginosa MDRPA UTI - model 48	2.24E-03	5.40E-03	0.01	0.00	0.00	0.00	2.24E-03	5.40E-03	0.01	1.02E-03	2.00E-03	3.33E-03	3.96E-05	9.55E-05	1.84E-04	0.00	0.00	0.00	3.96E-05	9.55E-05	1.84E-04	1.58	2.80	3.91	0.03	0.05	0.07	0.00	0.00	0.00	0.00	0.00	0.00
Denmark - Staphylococcus aureus MRSA BSI - model 61	14.98	16.88	18.80	105.75	113.77	122.63	121.48	130.71	140.94	4.01	4.25	4.51	0.26	0.30	0.33	1.87	2.01	2.17	2.15	2.31	2.49	29.04	30.73	32.52	0.51	0.54	0.57	5.15	5.53	5.93	0.09	0.10	0.10
Denmark - Staphylococcus aureus MRSA OTH - model 65	0.11	0.15	0.19	0.00	0.00	0.00	0.11	0.15	0.19	2.49E-03	3.24E-03	4.08E-03	1.96E-03	2.58E-03	3.32E-03	0.00	0.00	0.00	1.96E-03	2.58E-03	3.32E-03	40.40	45.20	49.82	0.71	0.80	0.88	0.00	0.00	0.00	0.00	0.00	0.00
Denmark - Staphylococcus aureus MRSA RESP - model 62	15.18	17.70	20.18	29.72	34.27	38.90	45.16	52.04	58.77	1.03	1.12	1.22	0.27	0.31	0.36	0.53	0.61	0.69	0.80	0.92	1.04	41.01	46.46	51.42	0.72	0.82	0.91	1.47	1.67	1.89	0.03	0.03	0.03
Denmark - Staphylococcus aureus MRSA SSI - model 64	0.08	0.11	0.14	15.84	17.98	20.07	16.02	18.10	20.19	0.35	0.36	0.37	1.36E-03	1.89E-03	2.56E-03	0.28	0.32	0.35	0.28	0.32	0.36	45.08	50.25	55.64	0.80	0.89	0.98	1.11	1.27	1.43	0.02	0.02	0.03
Denmark - Staphylococcus aureus MRSA UTI - model 63	0.01	0.01	0.02	0.00	0.00	0.00	0.01	0.01	0.02	1.46E-03	1.89E-03	2.35E-03	1.79E-04	2.49E-04	3.30E-04	0.00	0.00	0.00	1.79E-04	2.49E-04	3.30E-04	6.03	7.46	8.91	0.11	0.13	0.16	0.00	0.00	0.00	0.00	0.00	0.00
Denmark - Streptococcus pneumoniae PMRSP BSI - model 76	1.33	1.58	1.88	9.85	10.78	11.65	11.37	12.36	13.30	3.79	4.12	4.43	0.02	0.03	0.03	0.17	0.19	0.21	0.20	0.22	0.24	3.00	3.00	3.00	0.05	0.05	0.05	0.50	0.54	0.58	8.78E-03	9.54E-03	0.01
Denmark - Streptococcus pneumoniae PMRSP OTH - model 80	1.18E-03	2.08E-03	3.20E-03	0.00	0.00	0.00	1.18E-03	2.08E-03	3.20E-03	1.08E-03	1.95E-03	2.95E-03	2.08E-05	3.68E-05	5.66E-05	0.00	0.00	0.00	2.08E-05	3.68E-05	5.66E-05	0.93	1.07	1.24	0.02	0.02	0.02	0.00	0.00	0.00	0.00	0.00	0.00
Denmark - Streptococcus pneumoniae PMRSP RESP - model 77	3.21	4.17	5.61	6.15	8.03	10.86	9.53	12.25	16.32	0.96	1.08	1.22	0.06	0.07	0.10	0.11	0.14	0.19	0.17	0.22	0.29	9.11	11.32	14.48	0.16	0.20	0.26	0.32	0.41	0.53	5.60E-03	7.18E-03	9.35E-03
Denmark - Streptococcus pneumoniae PMRSP SSI - model 79	0.00	0.00	0.00	0.00	0.00	0.00	0.00	0.00	0.00	NaN	NaN	NaN	0.00	0.00	0.00	0.00	0.00	0.00	0.00	0.00	0.00	0.00	0.00	0.00	0.00	0.00	0.00	0.00	0.00	0.00	0.00	0.00	0.00
Denmark - Streptococcus pneumoniae PMRSP UTI - model 78	0.00	0.00	0.00	0.00	0.00	0.00	0.00	0.00	0.00	NaN	NaN	NaN	0.00	0.00	0.00	0.00	0.00	0.00	0.00	0.00	0.00	0.00	0.00	0.00	0.00	0.00	0.00	0.00	0.00	0.00	0.00	0.00	0.00
Denmark - Streptococcus pneumoniae PRSP BSI - model 71	1.57	1.84	2.18	11.38	12.41	13.42	13.19	14.26	15.29	3.30	3.57	3.82	0.03	0.03	0.04	0.20	0.22	0.24	0.23	0.25	0.27	4.00	4.00	4.00	0.07	0.07	0.07	0.67	0.72	0.77	0.01	0.01	0.01
Denmark - Streptococcus pneumoniae PRSP OTH - model 75	1.73E-03	2.82E-03	4.03E-03	0.00	0.00	0.00	1.73E-03	2.82E-03	4.03E-03	1.25E-03	1.97E-03	2.81E-03	3.06E-05	4.97E-05	7.12E-05	0.00	0.00	0.00	3.06E-05	4.97E-05	7.12E-05	1.26	1.42	1.63	0.02	0.03	0.03	0.00	0.00	0.00	0.00	0.00	0.00
Denmark - Streptococcus pneumoniae PRSP RESP - model 72	3.72	4.81	6.59	7.31	9.35	12.52	11.24	14.17	18.59	0.81	0.94	1.08	0.07	0.08	0.12	0.13	0.17	0.22	0.20	0.25	0.33	12.38	15.07	19.07	0.22	0.27	0.34	0.43	0.54	0.70	7.63E-03	9.57E-03	0.01
Denmark - Streptococcus pneumoniae PRSP SSI - model 74	0.00	0.00	0.00	0.00	0.00	0.00	0.00	0.00	0.00	NaN	NaN	NaN	0.00	0.00	0.00	0.00	0.00	0.00	0.00	0.00	0.00	0.00	0.00	0.00	0.00	0.00	0.00	0.00	0.00	0.00	0.00	0.00	0.00
Denmark - Streptococcus pneumoniae PRSP UTI - model 73	0.00	0.00	0.00	0.00	0.00	0.00	0.00	0.00	0.00	NaN	NaN	NaN	0.00	0.00	0.00	0.00	0.00	0.00	0.00	0.00	0.00	0.00	0.00	0.00	0.00	0.00	0.00	0.00	0.00	0.00	0.00	0.00	0.00
**Total (Median & 95% UIs)**	387.79	445.00	510.74	2,158.73	2,517.01	2,916.60	2,564.70	2,961.91	3,406.25		-		6.85	7.86	9.02	38.14	44.47	51.53	45.31	52.33	60.18	2,930.74	3,350.95	3,759.56	51.78	59.21	66.43	107.06	124.17	143.53	1.89	2.19	2.54
**Total (average)**		**445.70**			**2,524.26**			**2,969.96**			-			**7.87**			**44.60**			**52.48**			**3,347.53**			**59.15**			**124.51**			**2.20**	

Infection	YLD			YLL			DALY			DALY per Case			YLD per 100,000			YLL per 100,000			DALY per 100,000			Cases per year			Incidence per 100,000			Deaths per year			Mortality per 100,000		
Colistin resistant *Acinetobacter spp.*	1.14	1.88	2.76	3.99	9.98	17.40	5.42	11.91	19.83	6.27	11.55	16.87	0.09	0.14	0.21	0.30	0.76	1.32	0.41	0.91	1.51	1.79	3.00	4.25	0.14	0.23	0.32	0.10	0.25	0.44	0.01	0.02	0.03
Carbapenem resistant *Acinetobacter spp.* (excluding those resistant to colistin)	2.00	2.98	4.24	7.47	16.07	26.85	9.86	19.06	30.16	5.37	9.47	13.87	0.15	0.23	0.32	0.57	1.22	2.04	0.75	1.45	2.29	4.19	5.97	7.77	0.32	0.45	0.59	0.27	0.52	0.80	0.02	0.04	0.06
Aminoglycosides and fluoroquinolones resistant *Acinetobacter* spp. (excluding those resistant to colistin and/or carbapenem)	0.00	0.00	0.00	0.00	0.00	0.00	0.00	0.00	0.00	0.00	0.00	0.00	0.00	0.00	0.00	0.00	0.00	0.00	0.00	0.00	0.00	0.00	0.00	0.00	0.00	0.00	0.00	0.00	0.00	0.00	0.00	0.00	0.00
Vancomycin resistant Enterococci (*E. faecalis* and *E. faecium*)	0.00	0.00	0.00	0.00	0.00	0.00	0.00	0.00	0.00	0.00	0.00	0.00	0.00	0.00	0.00	0.00	0.00	0.00	0.00	0.00	0.00	0.00	0.00	0.00	0.00	0.00	0.00	0.00	0.00	0.00	0.00	0.00	0.00
Colistin resistant *Escherichia coli*	0.00	0.00	0.00	0.00	0.00	0.00	0.00	0.00	0.00	0.00	0.00	0.00	0.00	0.00	0.00	0.00	0.00	0.00	0.00	0.00	0.00	0.00	0.00	0.00	0.00	0.00	0.00	0.00	0.00	0.00	0.00	0.00	0.00
Carbapenem resistant *Escherichia coli* (excluding those resistant to colistin)	0.00	0.00	0.00	0.00	0.00	0.00	0.00	0.00	0.00	0.00	0.00	0.00	0.00	0.00	0.00	0.00	0.00	0.00	0.00	0.00	0.00	0.00	0.00	0.00	0.00	0.00	0.00	0.00	0.00	0.00	0.00	0.00	0.00
Third-generation cephalosporin resistant *Escherichia coli* (excluding those resistant to colistin and/or carbapenem)	14.64	17.18	20.14	85.81	101.17	118.85	101.37	118.48	137.73	4.06	4.51	5.01	1.11	1.31	1.53	6.53	7.69	9.04	7.71	9.01	10.48	180.71	208.68	237.75	13.74	15.87	18.08	5.52	6.40	7.40	0.42	0.49	0.56
Colistin resistant *Klebsiella pneumoniae*	0.00	0.00	0.00	0.00	0.00	0.00	0.00	0.00	0.00	0.00	0.00	0.00	0.00	0.00	0.00	0.00	0.00	0.00	0.00	0.00	0.00	0.00	0.00	0.00	0.00	0.00	0.00	0.00	0.00	0.00	0.00	0.00	0.00
Carbapenem resistant *Klebsiella pneumoniae* (excluding those resistant to colistin)	0.00	0.00	0.00	0.00	0.00	0.00	0.00	0.00	0.00	0.00	0.00	0.00	0.00	0.00	0.00	0.00	0.00	0.00	0.00	0.00	0.00	0.00	0.00	0.00	0.00	0.00	0.00	0.00	0.00	0.00	0.00	0.00	0.00
Third-generation cephalosporin resistant *Klebsiella pneumoniae* (excluding those resistant to colistin and/or carbapenem)	18.48	20.82	23.60	91.00	99.64	109.19	109.83	120.51	132.15	5.41	5.77	6.13	1.41	1.58	1.80	6.92	7.58	8.30	8.35	9.17	10.05	76.12	85.92	96.07	5.79	6.53	7.31	4.24	4.66	5.11	0.32	0.35	0.39
Colistin resistant *Pseudomonas aeruginosa*	0.00	0.00	0.00	0.00	0.00	0.00	0.00	0.00	0.00	0.00	0.00	0.00	0.00	0.00	0.00	0.00	0.00	0.00	0.00	0.00	0.00	0.00	0.00	0.00	0.00	0.00	0.00	0.00	0.00	0.00	0.00	0.00	0.00
Carbapenem resistant *Pseudomonas aeruginosa* (excluding those resistant to colistin)	4.00	6.09	8.61	14.32	29.74	51.50	19.31	36.08	59.11	5.91	10.40	15.31	0.30	0.46	0.65	1.09	2.26	3.92	1.47	2.74	4.50	9.63	15.37	21.16	0.73	1.17	1.61	0.48	1.00	1.69	0.04	0.08	0.13
Pseudomonas aeruginosa resistant to three or more antimicrobial groups (excluding those resistant to colistin and/or carbapenem)	0.00	0.00	0.00	0.00	0.00	0.00	0.00	0.00	0.00	0.00	0.00	0.00	0.00	0.00	0.00	0.00	0.00	0.00	0.00	0.00	0.00	0.00	0.00	0.00	0.00	0.00	0.00	0.00	0.00	0.00	0.00	0.00	0.00
Methicillin resistant *Staphylococcus aureus*	5.67	6.77	8.01	29.09	33.31	37.70	35.20	40.10	45.21	5.30	5.69	6.13	0.43	0.51	0.61	2.21	2.53	2.87	2.68	3.05	3.44	31.06	35.96	40.96	2.36	2.74	3.12	1.51	1.71	1.92	0.11	0.13	0.15
Penicillin and macrolides resistant *Streptococcus pneumoniae* (excluding those only resistant to penicillin)	2.86	4.11	6.53	11.05	13.81	18.35	14.37	17.91	24.47	10.11	11.52	12.94	0.22	0.31	0.50	0.84	1.05	1.40	1.09	1.36	1.86	4.04	5.04	7.21	0.31	0.38	0.55	0.25	0.31	0.42	0.02	0.02	0.03
Penicillin tesistant *Streptococcus pneumoniae* (excluding those resistant to macrolides)	0.35	0.50	0.78	1.25	1.56	2.08	1.66	2.06	2.79	1.17	1.33	1.49	0.03	0.04	0.06	0.10	0.12	0.16	0.13	0.16	0.21	4.04	4.97	7.15	0.31	0.38	0.54	0.25	0.31	0.41	0.02	0.02	0.03
Total	49.14	60.33	74.67	243.98	305.28	381.90	297.01	366.13	451.45				3.74	4.59	5.68	18.56	23.22	29.05	22.59	27.85	34.33	311.60	364.92	422.32	23.70	27.75	32.12	12.61	15.16	18.19	0.96	1.15	1.38

Model	YLD			YLL			DALY			DALY per Case			YLD per 100,000			YLL per 100,000			DALY per 100,000			Cases per year			Incidence per 100,000			Deaths per year			Mortality per 100,000		
Estonia - Acinetobacter spp. ColRACI BSI - model 41	7.17E-01	1.07E+00	1.54E+00	3.06	8.27	14.83	4.04E+00	9.37E+00	1.61E+01	4.07E+00	9.05E+00	1.41E+01	5.45E-02	8.14E-02	1.17E-01	0.23	0.63	1.13	3.07E-01	7.13E-01	1.23E+00	8.15E-01	1.04	1.31	6.20E-02	7.88E-02	9.93E-02	0.08	0.21	0.38	0.01	0.02	0.03
Estonia - Acinetobacter spp. ColRACI OTH - model 45	2.00E-05	1.19E-04	3.58E-04	0.00	0.00	0.00	2.00E-05	1.19E-04	3.58E-04	1.52E-03	3.67E-03	6.42E-03	1.52E-06	9.05E-06	2.73E-05	0.00	0.00	0.00	1.52E-06	9.05E-06	2.73E-05	7.27E-03	0.04	0.08	5.53E-04	2.75E-03	6.08E-03	0.00	0.00	0.00	0.00	0.00	0.00
Estonia - Acinetobacter spp. ColRACI RESP - model 42	0.42	0.81	1.22	0.88	1.58	2.36	1.32	2.40	3.50	1.84	2.14	2.45	0.03	0.06	0.09	0.07	0.12	0.18	0.10	0.18	0.27	0.66	1.13	1.59	0.05	0.09	0.12	0.02	0.04	0.06	1.70E-03	3.05E-03	4.55E-03
Estonia - Acinetobacter spp. ColRACI SSI - model 44	1.48E-04	7.21E-04	2.04E-03	0.05	0.13	0.21	0.05	0.14	0.21	0.36	0.36	0.36	1.13E-05	5.48E-05	1.55E-04	3.94E-03	0.01	0.02	3.94E-03	0.01	0.02	0.15	0.38	0.60	0.01	0.03	0.05	1.32E-03	3.42E-03	5.38E-03	1.00E-04	2.60E-04	4.09E-04
Estonia - Acinetobacter spp. ColRACI UTI - model 43	2.26E-04	7.50E-04	1.94E-03	0.00	0.00	0.00	2.26E-04	7.50E-04	1.94E-03	7.52E-04	1.97E-03	3.54E-03	1.72E-05	5.70E-05	1.48E-04	0.00	0.00	0.00	1.72E-05	5.70E-05	1.48E-04	0.16	0.42	0.67	0.01	0.03	0.05	0.00	0.00	0.00	0.00	0.00	0.00
Estonia - Acinetobacter spp. CRACI BSI - model 36	1.23	1.70	2.36	5.74	13.22	22.73	7.33	14.94	24.34	3.67	7.33	11.24	0.09	0.13	0.18	0.44	1.01	1.73	0.56	1.14	1.85	1.73	2.05	2.44	0.13	0.16	0.19	0.21	0.43	0.66	0.02	0.03	0.05
Estonia - Acinetobacter spp. CRACI OTH - model 40	8.97E-05	2.76E-04	5.94E-04	0.00	0.00	0.00	8.97E-05	2.76E-04	5.94E-04	1.94E-03	3.76E-03	5.71E-03	6.82E-06	2.10E-05	4.52E-05	0.00	0.00	0.00	6.82E-06	2.10E-05	4.52E-05	0.03	0.08	0.13	2.02E-03	5.83E-03	0.01	0.00	0.00	0.00	0.00	0.00	0.00
Estonia - Acinetobacter spp. CRACI RESP - model 37	0.77	1.28	1.88	1.56	2.54	3.67	2.36	3.81	5.37	1.32	1.72	2.17	0.06	0.10	0.14	0.12	0.19	0.28	0.18	0.29	0.41	1.55	2.22	2.91	0.12	0.17	0.22	0.05	0.08	0.11	4.12E-03	6.07E-03	8.32E-03
Estonia - Acinetobacter spp. CRACI SSI - model 39	5.27E-04	1.52E-03	3.26E-03	0.17	0.31	0.44	0.17	0.32	0.45	0.38	0.41	0.45	4.01E-05	1.16E-04	2.48E-04	0.01	0.02	0.03	0.01	0.02	0.03	0.41	0.76	1.08	0.03	0.06	0.08	8.34E-03	0.02	0.03	6.34E-04	1.30E-03	1.94E-03
Estonia - Acinetobacter spp. CRACI UTI - model 38	6.95E-04	1.67E-03	3.12E-03	0.00	0.00	0.00	6.95E-04	1.67E-03	3.12E-03	1.04E-03	1.99E-03	3.19E-03	5.28E-05	1.27E-04	2.38E-04	0.00	0.00	0.00	5.28E-05	1.27E-04	2.38E-04	0.46	0.85	1.21	0.04	0.06	0.09	0.00	0.00	0.00	0.00	0.00	0.00
Estonia - Acinetobacter spp. MDRACI BSI - model 31	0.00	0.00	0.00	0.00	0.00	0.00	0.00	0.00	0.00	NaN	NaN	NaN	0.00	0.00	0.00	0.00	0.00	0.00	0.00	0.00	0.00	0.00	0.00	0.00	0.00	0.00	0.00	0.00	0.00	0.00	0.00	0.00	0.00
Estonia - Acinetobacter spp. MDRACI OTH - model 35	0.00	0.00	0.00	0.00	0.00	0.00	0.00	0.00	0.00	NaN	NaN	NaN	0.00	0.00	0.00	0.00	0.00	0.00	0.00	0.00	0.00	0.00	0.00	0.00	0.00	0.00	0.00	0.00	0.00	0.00	0.00	0.00	0.00
Estonia - Acinetobacter spp. MDRACI RESP - model 32	0.00	0.00	0.00	0.00	0.00	0.00	0.00	0.00	0.00	NaN	NaN	NaN	0.00	0.00	0.00	0.00	0.00	0.00	0.00	0.00	0.00	0.00	0.00	0.00	0.00	0.00	0.00	0.00	0.00	0.00	0.00	0.00	0.00
Estonia - Acinetobacter spp. MDRACI SSI - model 34	0.00	0.00	0.00	0.00	0.00	0.00	0.00	0.00	0.00	NaN	NaN	NaN	0.00	0.00	0.00	0.00	0.00	0.00	0.00	0.00	0.00	0.00	0.00	0.00	0.00	0.00	0.00	0.00	0.00	0.00	0.00	0.00	0.00
Estonia - Acinetobacter spp. MDRACI UTI - model 33	0.00	0.00	0.00	0.00	0.00	0.00	0.00	0.00	0.00	NaN	NaN	NaN	0.00	0.00	0.00	0.00	0.00	0.00	0.00	0.00	0.00	0.00	0.00	0.00	0.00	0.00	0.00	0.00	0.00	0.00	0.00	0.00	0.00
Estonia - Enterococcus faecalis and E. faecium VRE BSI - model 66	0.00	0.00	0.00	0.00	0.00	0.00	0.00	0.00	0.00	NaN	NaN	NaN	0.00	0.00	0.00	0.00	0.00	0.00	0.00	0.00	0.00	0.00	0.00	0.00	0.00	0.00	0.00	0.00	0.00	0.00	0.00	0.00	0.00
Estonia - Enterococcus faecalis and E. faecium VRE OTH - model 70	0.00	0.00	0.00	0.00	0.00	0.00	0.00	0.00	0.00	NaN	NaN	NaN	0.00	0.00	0.00	0.00	0.00	0.00	0.00	0.00	0.00	0.00	0.00	0.00	0.00	0.00	0.00	0.00	0.00	0.00	0.00	0.00	0.00
Estonia - Enterococcus faecalis and E. faecium VRE RESP - model 67	0.00	0.00	0.00	0.00	0.00	0.00	0.00	0.00	0.00	NaN	NaN	NaN	0.00	0.00	0.00	0.00	0.00	0.00	0.00	0.00	0.00	0.00	0.00	0.00	0.00	0.00	0.00	0.00	0.00	0.00	0.00	0.00	0.00
Estonia - Enterococcus faecalis and E. faecium VRE SSI - model 69	0.00	0.00	0.00	0.00	0.00	0.00	0.00	0.00	0.00	NaN	NaN	NaN	0.00	0.00	0.00	0.00	0.00	0.00	0.00	0.00	0.00	0.00	0.00	0.00	0.00	0.00	0.00	0.00	0.00	0.00	0.00	0.00	0.00
Estonia - Enterococcus faecalis and E. faecium VRE UTI - model 68	0.00	0.00	0.00	0.00	0.00	0.00	0.00	0.00	0.00	NaN	NaN	NaN	0.00	0.00	0.00	0.00	0.00	0.00	0.00	0.00	0.00	0.00	0.00	0.00	0.00	0.00	0.00	0.00	0.00	0.00	0.00	0.00	0.00
Estonia - Escherichia coli 3GCREC BSI - model 16	11.37	12.86	14.76	70.35	82.05	96.00	82.55	95.06	109.66	2.91	3.24	3.60	0.87	0.98	1.12	5.35	6.24	7.30	6.28	7.23	8.34	26.95	29.36	32.09	2.05	2.23	2.44	4.41	5.05	5.80	0.34	0.38	0.44
Estonia - Escherichia coli 3GCREC OTH - model 20	0.02	0.03	0.04	0.00	0.00	0.00	0.02	0.03	0.04	2.52E-03	3.36E-03	4.21E-03	1.73E-03	2.48E-03	3.39E-03	0.00	0.00	0.00	1.73E-03	2.48E-03	3.39E-03	7.51	9.72	11.85	0.57	0.74	0.90	0.00	0.00	0.00	0.00	0.00	0.00
Estonia - Escherichia coli 3GCREC RESP - model 17	3.01	3.98	4.93	5.94	7.65	9.53	9.01	11.61	14.25	0.80	0.89	1.00	0.23	0.30	0.37	0.45	0.58	0.72	0.69	0.88	1.08	10.41	13.03	15.85	0.79	0.99	1.21	0.37	0.47	0.58	0.03	0.04	0.04
Estonia - Escherichia coli 3GCREC SSI - model 19	0.05	0.07	0.09	9.52	11.47	13.32	9.60	11.53	13.46	0.35	0.37	0.40	3.46E-03	4.99E-03	6.86E-03	0.72	0.87	1.01	0.73	0.88	1.02	26.38	31.01	36.00	2.01	2.36	2.74	0.73	0.88	1.03	0.06	0.07	0.08
Estonia - Escherichia coli 3GCREC UTI - model 18	0.19	0.25	0.32	0.00	0.00	0.00	0.19	0.25	0.32	1.53E-03	1.97E-03	2.43E-03	0.01	0.02	0.02	0.00	0.00	0.00	0.01	0.02	0.02	109.46	125.56	141.95	8.32	9.55	10.80	0.00	0.00	0.00	0.00	0.00	0.00
Estonia - Escherichia coli ColREC BSI - model 26	0.00	0.00	0.00	0.00	0.00	0.00	0.00	0.00	0.00	NaN	NaN	NaN	0.00	0.00	0.00	0.00	0.00	0.00	0.00	0.00	0.00	0.00	0.00	0.00	0.00	0.00	0.00	0.00	0.00	0.00	0.00	0.00	0.00
Estonia - Escherichia coli ColREC OTH - model 30	0.00	0.00	0.00	0.00	0.00	0.00	0.00	0.00	0.00	NaN	NaN	NaN	0.00	0.00	0.00	0.00	0.00	0.00	0.00	0.00	0.00	0.00	0.00	0.00	0.00	0.00	0.00	0.00	0.00	0.00	0.00	0.00	0.00
Estonia - Escherichia coli ColREC RESP - model 27	0.00	0.00	0.00	0.00	0.00	0.00	0.00	0.00	0.00	NaN	NaN	NaN	0.00	0.00	0.00	0.00	0.00	0.00	0.00	0.00	0.00	0.00	0.00	0.00	0.00	0.00	0.00	0.00	0.00	0.00	0.00	0.00	0.00
Estonia - Escherichia coli ColREC SSI - model 29	0.00	0.00	0.00	0.00	0.00	0.00	0.00	0.00	0.00	NaN	NaN	NaN	0.00	0.00	0.00	0.00	0.00	0.00	0.00	0.00	0.00	0.00	0.00	0.00	0.00	0.00	0.00	0.00	0.00	0.00	0.00	0.00	0.00
Estonia - Escherichia coli ColREC UTI - model 28	0.00	0.00	0.00	0.00	0.00	0.00	0.00	0.00	0.00	NaN	NaN	NaN	0.00	0.00	0.00	0.00	0.00	0.00	0.00	0.00	0.00	0.00	0.00	0.00	0.00	0.00	0.00	0.00	0.00	0.00	0.00	0.00	0.00
Estonia - Escherichia coli CREC BSI - model 21	0.00	0.00	0.00	0.00	0.00	0.00	0.00	0.00	0.00	NaN	NaN	NaN	0.00	0.00	0.00	0.00	0.00	0.00	0.00	0.00	0.00	0.00	0.00	0.00	0.00	0.00	0.00	0.00	0.00	0.00	0.00	0.00	0.00
Estonia - Escherichia coli CREC OTH - model 25	0.00	0.00	0.00	0.00	0.00	0.00	0.00	0.00	0.00	NaN	NaN	NaN	0.00	0.00	0.00	0.00	0.00	0.00	0.00	0.00	0.00	0.00	0.00	0.00	0.00	0.00	0.00	0.00	0.00	0.00	0.00	0.00	0.00
Estonia - Escherichia coli CREC RESP - model 22	0.00	0.00	0.00	0.00	0.00	0.00	0.00	0.00	0.00	NaN	NaN	NaN	0.00	0.00	0.00	0.00	0.00	0.00	0.00	0.00	0.00	0.00	0.00	0.00	0.00	0.00	0.00	0.00	0.00	0.00	0.00	0.00	0.00
Estonia - Escherichia coli CREC SSI - model 24	0.00	0.00	0.00	0.00	0.00	0.00	0.00	0.00	0.00	NaN	NaN	NaN	0.00	0.00	0.00	0.00	0.00	0.00	0.00	0.00	0.00	0.00	0.00	0.00	0.00	0.00	0.00	0.00	0.00	0.00	0.00	0.00	0.00
Estonia - Escherichia coli CREC UTI - model 23	0.00	0.00	0.00	0.00	0.00	0.00	0.00	0.00	0.00	NaN	NaN	NaN	0.00	0.00	0.00	0.00	0.00	0.00	0.00	0.00	0.00	0.00	0.00	0.00	0.00	0.00	0.00	0.00	0.00	0.00	0.00	0.00	0.00
Estonia - Klebsiella pneumoniae 3GCRKP BSI - model 1	11.67	12.89	14.53	75.12	81.03	87.86	87.04	94.00	101.88	3.95	4.19	4.42	0.89	0.98	1.10	5.71	6.16	6.68	6.62	7.15	7.75	21.08	22.48	23.94	1.60	1.71	1.82	3.46	3.75	4.05	0.26	0.28	0.31
Estonia - Klebsiella pneumoniae 3GCRKP OTH - model 5	0.02	0.03	0.04	0.00	0.00	0.00	0.02	0.03	0.04	2.56E-03	3.34E-03	4.22E-03	1.66E-03	2.24E-03	3.03E-03	0.00	0.00	0.00	1.66E-03	2.24E-03	3.03E-03	7.49	8.90	10.20	0.57	0.68	0.78	0.00	0.00	0.00	0.00	0.00	0.00
Estonia - Klebsiella pneumoniae 3GCRKP RESP - model 2	6.73	7.83	8.94	13.02	15.16	17.31	19.85	22.97	26.12	1.09	1.18	1.29	0.51	0.60	0.68	0.99	1.15	1.32	1.51	1.75	1.99	16.99	19.34	21.92	1.29	1.47	1.67	0.60	0.70	0.80	0.05	0.05	0.06
Estonia - Klebsiella pneumoniae 3GCRKP UTI - model 3	0.04	0.05	0.07	0.00	0.00	0.00	0.04	0.05	0.07	1.52E-03	1.96E-03	2.49E-03	2.91E-03	3.90E-03	5.12E-03	0.00	0.00	0.00	2.91E-03	3.90E-03	5.12E-03	23.17	26.37	29.72	1.76	2.01	2.26	0.00	0.00	0.00	0.00	0.00	0.00
Estonia - Klebsiella pneumoniae 3GCRKP SSI - model 4	0.01	0.02	0.03	2.87	3.44	4.02	2.88	3.46	4.04	0.37	0.39	0.41	9.92E-04	1.43E-03	2.00E-03	0.22	0.26	0.31	0.22	0.26	0.31	7.40	8.82	10.28	0.56	0.67	0.78	0.18	0.22	0.26	0.01	0.02	0.02
Estonia - Klebsiella pneumoniae ColRKP BSI - model 11	0.00	0.00	0.00	0.00	0.00	0.00	0.00	0.00	0.00	NaN	NaN	NaN	0.00	0.00	0.00	0.00	0.00	0.00	0.00	0.00	0.00	0.00	0.00	0.00	0.00	0.00	0.00	0.00	0.00	0.00	0.00	0.00	0.00
Estonia - Klebsiella pneumoniae ColRKP OTH - model 15	0.00	0.00	0.00	0.00	0.00	0.00	0.00	0.00	0.00	NaN	NaN	NaN	0.00	0.00	0.00	0.00	0.00	0.00	0.00	0.00	0.00	0.00	0.00	0.00	0.00	0.00	0.00	0.00	0.00	0.00	0.00	0.00	0.00
Estonia - Klebsiella pneumoniae ColRKP RESP - model 12	0.00	0.00	0.00	0.00	0.00	0.00	0.00	0.00	0.00	NaN	NaN	NaN	0.00	0.00	0.00	0.00	0.00	0.00	0.00	0.00	0.00	0.00	0.00	0.00	0.00	0.00	0.00	0.00	0.00	0.00	0.00	0.00	0.00
Estonia - Klebsiella pneumoniae ColRKP SSI - model 14	0.00	0.00	0.00	0.00	0.00	0.00	0.00	0.00	0.00	NaN	NaN	NaN	0.00	0.00	0.00	0.00	0.00	0.00	0.00	0.00	0.00	0.00	0.00	0.00	0.00	0.00	0.00	0.00	0.00	0.00	0.00	0.00	0.00
Estonia - Klebsiella pneumoniae ColRKP UTI - model 13	0.00	0.00	0.00	0.00	0.00	0.00	0.00	0.00	0.00	NaN	NaN	NaN	0.00	0.00	0.00	0.00	0.00	0.00	0.00	0.00	0.00	0.00	0.00	0.00	0.00	0.00	0.00	0.00	0.00	0.00	0.00	0.00	0.00
Estonia - Klebsiella pneumoniae CRKP BSI - model 6	0.00	0.00	0.00	0.00	0.00	0.00	0.00	0.00	0.00	NaN	NaN	NaN	0.00	0.00	0.00	0.00	0.00	0.00	0.00	0.00	0.00	0.00	0.00	0.00	0.00	0.00	0.00	0.00	0.00	0.00	0.00	0.00	0.00
Estonia - Klebsiella pneumoniae CRKP OTH - model 10	0.00	0.00	0.00	0.00	0.00	0.00	0.00	0.00	0.00	NaN	NaN	NaN	0.00	0.00	0.00	0.00	0.00	0.00	0.00	0.00	0.00	0.00	0.00	0.00	0.00	0.00	0.00	0.00	0.00	0.00	0.00	0.00	0.00
Estonia - Klebsiella pneumoniae CRKP RESP - model 7	0.00	0.00	0.00	0.00	0.00	0.00	0.00	0.00	0.00	NaN	NaN	NaN	0.00	0.00	0.00	0.00	0.00	0.00	0.00	0.00	0.00	0.00	0.00	0.00	0.00	0.00	0.00	0.00	0.00	0.00	0.00	0.00	0.00
Estonia - Klebsiella pneumoniae CRKP SSI - model 9	0.00	0.00	0.00	0.00	0.00	0.00	0.00	0.00	0.00	NaN	NaN	NaN	0.00	0.00	0.00	0.00	0.00	0.00	0.00	0.00	0.00	0.00	0.00	0.00	0.00	0.00	0.00	0.00	0.00	0.00	0.00	0.00	0.00
Estonia - Klebsiella pneumoniae CRKP UTI - model 8	0.00	0.00	0.00	0.00	0.00	0.00	0.00	0.00	0.00	NaN	NaN	NaN	0.00	0.00	0.00	0.00	0.00	0.00	0.00	0.00	0.00	0.00	0.00	0.00	0.00	0.00	0.00	0.00	0.00	0.00	0.00	0.00	0.00
Estonia - Pseudomonas aeruginosa ColRPA BSI - model 56	0.00	0.00	0.00	0.00	0.00	0.00	0.00	0.00	0.00	NaN	NaN	NaN	0.00	0.00	0.00	0.00	0.00	0.00	0.00	0.00	0.00	0.00	0.00	0.00	0.00	0.00	0.00	0.00	0.00	0.00	0.00	0.00	0.00
Estonia - Pseudomonas aeruginosa ColRPA OTH - model 60	0.00	0.00	0.00	0.00	0.00	0.00	0.00	0.00	0.00	NaN	NaN	NaN	0.00	0.00	0.00	0.00	0.00	0.00	0.00	0.00	0.00	0.00	0.00	0.00	0.00	0.00	0.00	0.00	0.00	0.00	0.00	0.00	0.00
Estonia - Pseudomonas aeruginosa ColRPA RESP - model 57	0.00	0.00	0.00	0.00	0.00	0.00	0.00	0.00	0.00	NaN	NaN	NaN	0.00	0.00	0.00	0.00	0.00	0.00	0.00	0.00	0.00	0.00	0.00	0.00	0.00	0.00	0.00	0.00	0.00	0.00	0.00	0.00	0.00
Estonia - Pseudomonas aeruginosa ColRPA SSI - model 59	0.00	0.00	0.00	0.00	0.00	0.00	0.00	0.00	0.00	NaN	NaN	NaN	0.00	0.00	0.00	0.00	0.00	0.00	0.00	0.00	0.00	0.00	0.00	0.00	0.00	0.00	0.00	0.00	0.00	0.00	0.00	0.00	0.00
Estonia - Pseudomonas aeruginosa ColRPA UTI - model 58	0.00	0.00	0.00	0.00	0.00	0.00	0.00	0.00	0.00	NaN	NaN	NaN	0.00	0.00	0.00	0.00	0.00	0.00	0.00	0.00	0.00	0.00	0.00	0.00	0.00	0.00	0.00	0.00	0.00	0.00	0.00	0.00	0.00
Estonia - Pseudomonas aeruginosa CRPA BSI - model 51	1.24	1.73	2.38	6.65	14.35	24.82	8.26	16.12	26.97	4.05	7.62	11.46	0.09	0.13	0.18	0.51	1.09	1.89	0.63	1.23	2.05	1.66	2.14	2.74	0.13	0.16	0.21	0.22	0.48	0.82	0.02	0.04	0.06
Estonia - Pseudomonas aeruginosa CRPA OTH - model 55	1.51E-03	3.76E-03	7.49E-03	0.00	0.00	0.00	1.51E-03	3.76E-03	7.49E-03	1.96E-03	3.60E-03	5.72E-03	1.14E-04	2.86E-04	5.70E-04	0.00	0.00	0.00	1.14E-04	2.86E-04	5.70E-04	0.54	1.09	1.61	0.04	0.08	0.12	0.00	0.00	0.00	0.00	0.00	0.00
Estonia - Pseudomonas aeruginosa CRPA RESP - model 52	2.76	4.34	6.20	7.46	14.98	26.09	10.83	19.54	31.53	1.60	2.50	3.54	0.21	0.33	0.47	0.57	1.14	1.98	0.82	1.49	2.40	5.18	7.90	10.69	0.39	0.60	0.81	0.25	0.50	0.85	0.02	0.04	0.06
Estonia - Pseudomonas aeruginosa CRPA SSI - model 54	1.03E-03	3.01E-03	6.21E-03	0.21	0.41	0.59	0.22	0.41	0.59	0.25	0.27	0.30	7.86E-05	2.29E-04	4.72E-04	0.02	0.03	0.04	0.02	0.03	0.05	0.80	1.51	2.16	0.06	0.11	0.16	7.20E-03	0.01	0.02	5.47E-04	1.03E-03	1.48E-03
Estonia - Pseudomonas aeruginosa CRPA UTI - model 53	2.21E-03	5.38E-03	0.01	0.00	0.00	0.00	2.21E-03	5.38E-03	0.01	1.05E-03	2.04E-03	3.20E-03	1.68E-04	4.09E-04	7.82E-04	0.00	0.00	0.00	1.68E-04	4.09E-04	7.82E-04	1.45	2.73	3.95	0.11	0.21	0.30	0.00	0.00	0.00	0.00	0.00	0.00
Estonia - Pseudomonas aeruginosa MDRPA BSI - model 46	0.00	0.00	0.00	0.00	0.00	0.00	0.00	0.00	0.00	NaN	NaN	NaN	0.00	0.00	0.00	0.00	0.00	0.00	0.00	0.00	0.00	0.00	0.00	0.00	0.00	0.00	0.00	0.00	0.00	0.00	0.00	0.00	0.00
Estonia - Pseudomonas aeruginosa MDRPA OTH - model 50	0.00	0.00	0.00	0.00	0.00	0.00	0.00	0.00	0.00	NaN	NaN	NaN	0.00	0.00	0.00	0.00	0.00	0.00	0.00	0.00	0.00	0.00	0.00	0.00	0.00	0.00	0.00	0.00	0.00	0.00	0.00	0.00	0.00
Estonia - Pseudomonas aeruginosa MDRPA RESP - model 47	0.00	0.00	0.00	0.00	0.00	0.00	0.00	0.00	0.00	NaN	NaN	NaN	0.00	0.00	0.00	0.00	0.00	0.00	0.00	0.00	0.00	0.00	0.00	0.00	0.00	0.00	0.00	0.00	0.00	0.00	0.00	0.00	0.00
Estonia - Pseudomonas aeruginosa MDRPA SSI - model 49	0.00	0.00	0.00	0.00	0.00	0.00	0.00	0.00	0.00	NaN	NaN	NaN	0.00	0.00	0.00	0.00	0.00	0.00	0.00	0.00	0.00	0.00	0.00	0.00	0.00	0.00	0.00	0.00	0.00	0.00	0.00	0.00	0.00
Estonia - Pseudomonas aeruginosa MDRPA UTI - model 48	0.00	0.00	0.00	0.00	0.00	0.00	0.00	0.00	0.00	NaN	NaN	NaN	0.00	0.00	0.00	0.00	0.00	0.00	0.00	0.00	0.00	0.00	0.00	0.00	0.00	0.00	0.00	0.00	0.00	0.00	0.00	0.00	0.00
Estonia - Staphylococcus aureus MRSA BSI - model 61	2.81	3.30	3.83	19.71	22.11	24.65	22.86	25.41	28.14	3.87	4.14	4.45	0.21	0.25	0.29	1.50	1.68	1.87	1.74	1.93	2.14	5.68	6.15	6.61	0.43	0.47	0.50	1.00	1.10	1.22	0.08	0.08	0.09
Estonia - Staphylococcus aureus MRSA OTH - model 65	0.02	0.03	0.04	0.00	0.00	0.00	0.02	0.03	0.04	2.33E-03	3.26E-03	4.32E-03	1.58E-03	2.24E-03	3.04E-03	0.00	0.00	0.00	1.58E-03	2.24E-03	3.04E-03	7.79	9.06	10.37	0.59	0.69	0.79	0.00	0.00	0.00	0.00	0.00	0.00
Estonia - Staphylococcus aureus MRSA RESP - model 62	2.83	3.42	4.10	5.51	6.67	7.88	8.42	10.11	11.82	1.00	1.09	1.19	0.21	0.26	0.31	0.42	0.51	0.60	0.64	0.77	0.90	7.88	9.20	10.65	0.60	0.70	0.81	0.28	0.33	0.39	0.02	0.03	0.03
Estonia - Staphylococcus aureus MRSA SSI - model 64	0.01	0.02	0.03	3.86	4.54	5.17	3.89	4.56	5.20	0.43	0.45	0.48	1.00E-03	1.62E-03	2.48E-03	0.29	0.34	0.39	0.30	0.35	0.40	8.63	10.03	11.43	0.66	0.76	0.87	0.23	0.27	0.31	0.02	0.02	0.02
Estonia - Staphylococcus aureus MRSA UTI - model 63	1.79E-03	2.77E-03	4.09E-03	0.00	0.00	0.00	1.79E-03	2.77E-03	4.09E-03	1.32E-03	1.87E-03	2.52E-03	1.37E-04	2.10E-04	3.11E-04	0.00	0.00	0.00	1.37E-04	2.10E-04	3.11E-04	1.09	1.52	1.89	0.08	0.12	0.14	0.00	0.00	0.00	0.00	0.00	0.00
Estonia - Streptococcus pneumoniae PMRSP BSI - model 76	0.86	1.16	1.55	7.01	7.98	8.90	8.07	9.13	10.20	8.07	9.13	10.20	0.07	0.09	0.12	0.53	0.61	0.68	0.61	0.69	0.78	1.00	1.00	1.00	0.08	0.08	0.08	0.16	0.18	0.20	0.01	0.01	0.02
Estonia - Streptococcus pneumoniae PMRSP OTH - model 80	2.61E-04	6.52E-04	1.33E-03	0.00	0.00	0.00	2.61E-04	6.52E-04	1.33E-03	7.62E-04	1.86E-03	3.53E-03	1.98E-05	4.96E-05	1.01E-04	0.00	0.00	0.00	1.98E-05	4.96E-05	1.01E-04	0.29	0.35	0.46	0.02	0.03	0.03	0.00	0.00	0.00	0.00	0.00	0.00
Estonia - Streptococcus pneumoniae PMRSP RESP - model 77	2.00	2.95	4.98	4.04	5.83	9.44	6.30	8.78	14.27	2.04	2.39	2.74	0.15	0.22	0.38	0.31	0.44	0.72	0.48	0.67	1.09	2.75	3.69	5.76	0.21	0.28	0.44	0.09	0.13	0.21	6.95E-03	0.01	0.02
Estonia - Streptococcus pneumoniae PMRSP SSI - model 79	0.00	0.00	0.00	0.00	0.00	0.00	0.00	0.00	0.00	NaN	NaN	NaN	0.00	0.00	0.00	0.00	0.00	0.00	0.00	0.00	0.00	0.00	0.00	0.00	0.00	0.00	0.00	0.00	0.00	0.00	0.00	0.00	0.00
Estonia - Streptococcus pneumoniae PMRSP UTI - model 78	0.00	0.00	0.00	0.00	0.00	0.00	0.00	0.00	0.00	NaN	NaN	NaN	0.00	0.00	0.00	0.00	0.00	0.00	0.00	0.00	0.00	0.00	0.00	0.00	0.00	0.00	0.00	0.00	0.00	0.00	0.00	0.00	0.00
Estonia - Streptococcus pneumoniae PRSP BSI - model 71	0.10	0.14	0.18	0.80	0.90	1.01	0.92	1.05	1.17	0.92	1.05	1.17	7.79E-03	0.01	0.01	0.06	0.07	0.08	0.07	0.08	0.09	1.00	1.00	1.00	0.08	0.08	0.08	0.16	0.18	0.20	0.01	0.01	0.02
Estonia - Streptococcus pneumoniae PRSP OTH - model 75	2.42E-04	6.86E-04	1.36E-03	0.00	0.00	0.00	2.42E-04	6.86E-04	1.36E-03	6.96E-04	1.91E-03	3.61E-03	1.84E-05	5.22E-05	1.03E-04	0.00	0.00	0.00	1.84E-05	5.22E-05	1.03E-04	0.29	0.36	0.46	0.02	0.03	0.03	0.00	0.00	0.00	0.00	0.00	0.00
Estonia - Streptococcus pneumoniae PRSP RESP - model 72	0.25	0.36	0.60	0.46	0.65	1.06	0.73	1.02	1.62	0.24	0.28	0.32	0.02	0.03	0.05	0.03	0.05	0.08	0.06	0.08	0.12	2.76	3.61	5.70	0.21	0.27	0.43	0.09	0.13	0.21	6.98E-03	9.90E-03	0.02
Estonia - Streptococcus pneumoniae PRSP SSI - model 74	0.00	0.00	0.00	0.00	0.00	0.00	0.00	0.00	0.00	NaN	NaN	NaN	0.00	0.00	0.00	0.00	0.00	0.00	0.00	0.00	0.00	0.00	0.00	0.00	0.00	0.00	0.00	0.00	0.00	0.00	0.00	0.00	0.00
Estonia - Streptococcus pneumoniae PRSP UTI - model 73	0.00	0.00	0.00	0.00	0.00	0.00	0.00	0.00	0.00	NaN	NaN	NaN	0.00	0.00	0.00	0.00	0.00	0.00	0.00	0.00	0.00	0.00	0.00	0.00	0.00	0.00	0.00	0.00	0.00	0.00	0.00	0.00	0.00
**Total (Median & 95% UIs)**	49.14	60.33	74.67	243.98	305.28	381.90	297.01	366.13	451.45		-		3.74	4.59	5.68	18.56	23.22	29.05	22.59	27.85	34.33	311.60	364.92	422.32	23.70	27.75	32.12	12.61	15.16	18.19	0.96	1.15	1.38
**Total (average)**		**60.74**			**307.34**			**368.08**			-			**4.62**			**23.37**			**27.99**			**356.56**			**27.12**			**15.23**			**1.16**	

Infection	YLD			YLL			DALY			DALY per Case			YLD per 100,000			YLL per 100,000			DALY per 100,000			Cases per year			Incidence per 100,000			Deaths per year			Mortality per 100,000		
Colistin resistant *Acinetobacter spp.*	171.38	191.96	214.09	840.12	1.025.21	1.225.95	1.018.48	1.215.89	1.431.18	7.74	9.19	10.60	1.58	1.77	1.97	7.74	9.44	11.29	9.38	11.20	13.18	345.62	389.63	436.06	3.18	3.59	4.02	27.27	34.02	40.72	0.25	0.31	0.38
Carbapenem resistant *Acinetobacter spp.* (excluding those resistant to colistin)	1,604.23	1,751.22	1,894.28	7,975.81	9,413.80	10,671.56	9,613.21	11,169.66	12,536.01	6.85	7.81	8.67	14.77	16.13	17.45	73.46	86.70	98.28	88.54	102.87	115.45	3,800.84	4,215.18	4,606.36	35.00	38.82	42.42	310.17	371.21	430.10	2.86	3.42	3.96
Aminoglycosides and fluoroquinolones resistant *Acinetobacter* spp. (excluding those resistant to colistin and/or carbapenem)	17.23	19.14	21.34	83.15	100.75	118.44	101.24	120.12	138.87	6.22	7.14	8.07	0.16	0.18	0.20	0.77	0.93	1.09	0.93	1.11	1.28	44.49	49.89	55.23	0.41	0.46	0.51	3.56	4.32	5.09	0.03	0.04	0.05
Vancomycin resistant Enterococci (*E. faecalis* and *E. faecium*)	95.72	110.78	128.76	899.48	1,043.80	1,204.88	1,002.45	1,155.16	1,331.12	6.27	6.77	7.29	0.88	1.02	1.19	8.28	9.61	11.10	9.23	10.64	12.26	545.96	655.43	770.85	5.03	6.04	7.10	37.02	43.70	51.61	0.34	0.40	0.48
Colistin resistant *Escherichia coli*	7.03	7.86	8.69	118.13	135.32	153.31	125.43	143.16	161.59	12.43	14.09	15.74	0.06	0.07	0.08	1.09	1.25	1.41	1.16	1.32	1.49	69.11	78.39	87.54	0.64	0.72	0.81	5.78	6.85	8.04	0.05	0.06	0.07
Carbapenem resistant *Escherichia coli* (excluding those resistant to colistin)	21.69	25.71	30.74	224.78	274.18	339.52	246.58	300.58	368.19	12.71	15.14	17.92	0.20	0.24	0.28	2.07	2.53	3.13	2.27	2.77	3.39	138.14	156.70	175.86	1.27	1.44	1.62	7.13	8.44	9.87	0.07	0.08	0.09
Third-generation cephalosporin resistant *Escherichia coli* (excluding those resistant to colistin and/or carbapenem)	231.58	257.13	284.27	1,367.01	1,529.18	1,705.64	1,606.96	1,787.34	1,983.88	4.65	5.04	5.45	2.13	2.37	2.62	12.59	14.08	15.71	14.80	16.46	18.27	2,499.83	2,829.91	3,136.03	23.02	26.06	28.88	75.84	86.78	98.63	0.70	0.80	0.91
Colistin resistant *Klebsiella pneumoniae*	347.43	391.45	439.44	5,942.01	6,936.69	7,971.62	6,318.30	7,324.17	8,377.90	15.80	17.97	20.24	3.20	3.61	4.05	54.72	63.89	73.42	58.19	67.45	77.16	1,050.15	1,211.57	1,371.30	9.67	11.16	12.63	233.50	271.40	314.40	2.15	2.50	2.90
Carbapenem resistant *Klebsiella pneumoniae* (excluding those resistant to colistin)	832.10	920.78	1,021.38	8,678.78	9,865.64	11,063.91	9,557.89	10,790.46	12,058.57	10.40	11.56	12.65	7.66	8.48	9.41	79.93	90.86	101.90	88.03	99.38	111.06	2,538.99	2,883.23	3,250.19	23.38	26.55	29.93	342.24	391.68	444.04	3.15	3.61	4.09
Third-generation cephalosporin resistant *Klebsiella pneumoniae* (excluding those resistant to colistin and/or carbapenem)	186.83	203.66	223.16	916.66	976.06	1,045.07	1,107.37	1,180.72	1,259.87	6.79	7.14	7.51	1.72	1.88	2.06	8.44	8.99	9.62	10.20	10.87	11.60	618.23	677.20	734.22	5.69	6.24	6.76	34.29	36.75	39.38	0.32	0.34	0.36
Colistin resistant *Pseudomonas aeruginosa*	18.51	20.87	23.48	143.11	184.99	229.97	164.37	206.03	250.13	6.38	7.79	9.29	0.17	0.19	0.22	1.32	1.70	2.12	1.51	1.90	2.30	78.58	93.58	108.24	0.72	0.86	1.00	5.05	6.59	8.09	0.05	0.06	0.07
Carbapenem resistant *Pseudomonas aeruginosa* (excluding those resistant to colistin)	1,292.83	1,438.21	1,592.97	6,091.59	7,270.50	8,566.85	7,437.71	8,707.90	10,095.84	8.47	9.76	11.05	11.91	13.25	14.67	56.10	66.96	78.90	68.50	80.20	92.98	2,880.38	3,241.79	3,581.90	26.53	29.86	32.99	220.15	267.87	318.31	2.03	2.47	2.93
Pseudomonas aeruginosa resistant to three or more antimicrobial groups (excluding those resistant to colistin and/or carbapenem)	30.37	34.28	38.58	133.01	162.69	195.31	165.47	197.08	231.52	6.27	7.34	8.44	0.28	0.32	0.36	1.22	1.50	1.80	1.52	1.82	2.13	102.04	118.70	134.82	0.94	1.09	1.24	6.02	7.57	9.30	0.06	0.07	0.09
Methicillin resistant *Staphylococcus aureus*	323.32	361.02	402.86	1.584.43	1.735.81	1.899.79	1.912.17	2.097.94	2.297.86	5.43	5.80	6.17	2.98	3.32	3.71	14.59	15.99	17.50	17.61	19.32	21.16	1.644.51	1.870.72	2.121.86	15.15	17.23	19.54	78.80	88.36	98.58	0.73	0.81	0.91
Penicillin and macrolides resistant *Streptococcus pneumoniae* (excluding those only resistant to penicillin)	0.00	0.00	0.00	0.00	0.00	0.00	0.00	0.00	0.00	0.00	0.00	0.00	0.00	0.00	0.00	0.00	0.00	0.00	0.00	0.00	0.00	0.00	0.00	0.00	0.00	0.00	0.00	0.00	0.00	0.00	0.00	0.00	0.00
Penicillin tesistant *Streptococcus pneumoniae* (excluding those resistant to macrolides)	0.00	0.00	0.00	0.00	0.00	0.00	0.00	0.00	0.00	0.00	0.00	0.00	0.00	0.00	0.00	0.00	0.00	0.00	0.00	0.00	0.00	0.00	0.00	0.00	0.00	0.00	0.00	0.00	0.00	0.00	0.00	0.00	0.00
Total	5,180.25	5,734.06	6,324.04	34,998.06	40,654.64	46,391.83	40,377.64	46,396.21	52,522.54				47.71	52.81	58.24	322.32	374.42	427.26	371.87	427.30	483.72	16,356.89	18,471.91	20,570.44	150.64	170.12	189.45	1,386.84	1,625.54	1,876.18	12.77	14.97	17.28

Model	YLD			YLL			DALY			DALY per Case			YLD per 100,000			YLL per 100,000			DALY per 100,000			Cases per year			Incidence per 100,000			Deaths per year			Mortality per 100,000		
Greece - Acinetobacter spp. ColRACI BSI - model 41	99.00	109.74	121.07	681.85	843.96	1,019.25	786.89	952.57	1,132.59	5.78	7.09	8.36	0.91	1.01	1.12	6.28	7.77	9.39	7.25	8.77	10.43	126.41	134.72	143.98	1.16	1.24	1.33	21.92	27.80	33.60	0.20	0.26	0.31
Greece - Acinetobacter spp. ColRACI OTH - model 45	0.01	0.02	0.02	0.00	0.00	0.00	0.01	0.02	0.02	2.99E-03	3.76E-03	4.53E-03	1.16E-04	1.64E-04	2.26E-04	0.00	0.00	0.00	1.16E-04	1.64E-04	2.26E-04	3.53	4.79	6.22	0.03	0.04	0.06	0.00	0.00	0.00	0.00	0.00	0.00
Greece - Acinetobacter spp. ColRACI RESP - model 42	72.21	81.99	92.71	140.57	160.15	181.68	213.71	241.99	273.33	1.56	1.66	1.77	0.67	0.76	0.85	1.29	1.47	1.67	1.97	2.23	2.52	129.14	146.03	163.69	1.19	1.34	1.51	4.57	5.25	5.94	0.04	0.05	0.05
Greece - Acinetobacter spp. ColRACI SSI - model 44	0.07	0.10	0.14	17.70	21.09	25.02	17.78	21.20	25.09	0.40	0.43	0.46	6.86E-04	9.66E-04	1.33E-03	0.16	0.19	0.23	0.16	0.20	0.23	40.97	49.13	58.25	0.38	0.45	0.54	0.77	0.97	1.18	7.13E-03	8.95E-03	0.01
Greece - Acinetobacter spp. ColRACI UTI - model 43	0.09	0.11	0.14	0.00	0.00	0.00	0.09	0.11	0.14	1.64E-03	2.03E-03	2.49E-03	7.83E-04	1.03E-03	1.32E-03	0.00	0.00	0.00	7.83E-04	1.03E-03	1.32E-03	45.57	54.96	63.93	0.42	0.51	0.59	0.00	0.00	0.00	0.00	0.00	0.00
Greece - Acinetobacter spp. CRACI BSI - model 36	929.90	1,001.35	1,075.31	6,459.08	7,731.36	8,821.84	7,419.96	8,736.11	9,867.90	5.12	5.98	6.74	8.56	9.22	9.90	59.49	71.20	81.25	68.34	80.46	90.88	1,385.37	1,460.75	1,545.98	12.76	13.45	14.24	248.51	302.00	353.07	2.29	2.78	3.25
Greece - Acinetobacter spp. CRACI OTH - model 40	0.14	0.19	0.25	0.00	0.00	0.00	0.14	0.19	0.25	3.10E-03	3.76E-03	4.47E-03	1.31E-03	1.78E-03	2.34E-03	0.00	0.00	0.00	1.31E-03	1.78E-03	2.34E-03	40.72	51.64	63.65	0.38	0.48	0.59	0.00	0.00	0.00	0.00	0.00	0.00
Greece - Acinetobacter spp. CRACI RESP - model 37	672.39	747.34	815.74	1,321.72	1,454.84	1,589.25	1,995.22	2,203.36	2,404.52	1.32	1.40	1.47	6.19	6.88	7.51	12.17	13.40	14.64	18.38	20.29	22.15	1,419.08	1,582.66	1,721.14	13.07	14.58	15.85	51.06	56.82	62.44	0.47	0.52	0.58
Greece - Acinetobacter spp. CRACI SSI - model 39	0.86	1.13	1.47	195.01	227.60	260.47	196.95	228.79	261.84	0.40	0.43	0.45	7.95E-03	0.01	0.01	1.80	2.10	2.40	1.81	2.11	2.41	453.31	531.78	603.55	4.17	4.90	5.56	10.60	12.39	14.60	0.10	0.11	0.13
Greece - Acinetobacter spp. CRACI UTI - model 38	0.94	1.20	1.50	0.00	0.00	0.00	0.94	1.20	1.50	1.70E-03	2.03E-03	2.41E-03	8.66E-03	0.01	0.01	0.00	0.00	0.00	8.66E-03	0.01	0.01	502.36	588.34	672.04	4.63	5.42	6.19	0.00	0.00	0.00	0.00	0.00	0.00
Greece - Acinetobacter spp. MDRACI BSI - model 31	9.98	10.94	12.08	66.58	81.86	97.18	77.45	93.01	108.53	4.55	5.36	6.19	0.09	0.10	0.11	0.61	0.75	0.89	0.71	0.86	1.00	16.24	17.27	18.36	0.15	0.16	0.17	2.83	3.48	4.14	0.03	0.03	0.04
Greece - Acinetobacter spp. MDRACI OTH - model 35	1.65E-03	2.31E-03	3.07E-03	0.00	0.00	0.00	1.65E-03	2.31E-03	3.07E-03	3.07E-03	3.73E-03	4.49E-03	1.52E-05	2.13E-05	2.83E-05	0.00	0.00	0.00	1.52E-05	2.13E-05	2.83E-05	0.47	0.61	0.77	4.33E-03	5.66E-03	7.09E-03	0.00	0.00	0.00	0.00	0.00	0.00
Greece - Acinetobacter spp. MDRACI RESP - model 32	7.23	8.17	9.22	14.16	15.88	17.67	21.35	24.07	26.71	1.22	1.29	1.37	0.07	0.08	0.08	0.13	0.15	0.16	0.20	0.22	0.25	16.60	18.70	20.77	0.15	0.17	0.19	0.60	0.67	0.76	5.48E-03	6.19E-03	6.98E-03
Greece - Acinetobacter spp. MDRACI SSI - model 34	0.01	0.01	0.02	2.41	3.01	3.60	2.42	3.03	3.61	0.44	0.48	0.51	9.35E-05	1.22E-04	1.64E-04	0.02	0.03	0.03	0.02	0.03	0.03	5.26	6.30	7.22	0.05	0.06	0.07	0.13	0.17	0.20	1.21E-03	1.53E-03	1.83E-03
Greece - Acinetobacter spp. MDRACI UTI - model 33	0.01	0.01	0.02	0.00	0.00	0.00	0.01	0.01	0.02	1.66E-03	2.03E-03	2.46E-03	9.83E-05	1.31E-04	1.67E-04	0.00	0.00	0.00	9.83E-05	1.31E-04	1.67E-04	5.91	7.00	8.11	0.05	0.06	0.07	0.00	0.00	0.00	0.00	0.00	0.00
Greece - Enterococcus faecalis and E. faecium VRE BSI - model 66	95.01	109.84	127.53	816.57	941.25	1,082.36	918.74	1,051.73	1,207.43	5.85	6.32	6.80	0.88	1.01	1.17	7.52	8.67	9.97	8.46	9.69	11.12	142.53	165.91	194.92	1.31	1.53	1.80	32.55	38.00	44.63	0.30	0.35	0.41
Greece - Enterococcus faecalis and E. faecium VRE OTH - model 70	0.22	0.29	0.38	0.00	0.00	0.00	0.22	0.29	0.38	1.32E-03	1.65E-03	2.00E-03	2.06E-03	2.68E-03	3.49E-03	0.00	0.00	0.00	2.06E-03	2.68E-03	3.49E-03	147.57	176.99	208.81	1.36	1.63	1.92	0.00	0.00	0.00	0.00	0.00	0.00
Greece - Enterococcus faecalis and E. faecium VRE RESP - model 67	0.00	0.00	0.00	0.00	0.00	0.00	0.00	0.00	0.00	NaN	NaN	NaN	0.00	0.00	0.00	0.00	0.00	0.00	0.00	0.00	0.00	0.00	0.00	0.00	0.00	0.00	0.00	0.00	0.00	0.00	0.00	0.00	0.00
Greece - Enterococcus faecalis and E. faecium VRE SSI - model 69	0.36	0.49	0.64	82.91	102.55	122.52	83.37	102.98	123.11	0.41	0.45	0.48	3.35E-03	4.50E-03	5.94E-03	0.76	0.94	1.13	0.77	0.95	1.13	187.70	228.11	266.63	1.73	2.10	2.46	4.47	5.70	6.98	0.04	0.05	0.06
Greece - Enterococcus faecalis and E. faecium VRE UTI - model 68	0.12	0.16	0.20	0.00	0.00	0.00	0.12	0.16	0.20	1.52E-03	1.87E-03	2.26E-03	1.08E-03	1.44E-03	1.88E-03	0.00	0.00	0.00	1.08E-03	1.44E-03	1.88E-03	68.15	84.42	100.49	0.63	0.78	0.93	0.00	0.00	0.00	0.00	0.00	0.00
Greece - Escherichia coli 3GCREC BSI - model 16	177.73	193.30	210.52	1,125.83	1,246.33	1,383.99	1,311.18	1,440.82	1,589.68	3.37	3.64	3.92	1.64	1.78	1.94	10.37	11.48	12.75	12.08	13.27	14.64	368.12	395.57	423.74	3.39	3.64	3.90	60.34	68.30	77.17	0.56	0.63	0.71
Greece - Escherichia coli 3GCREC OTH - model 20	0.32	0.43	0.56	0.00	0.00	0.00	0.32	0.43	0.56	2.67E-03	3.35E-03	4.03E-03	2.99E-03	3.99E-03	5.20E-03	0.00	0.00	0.00	2.99E-03	3.99E-03	5.20E-03	106.27	130.68	155.69	0.98	1.20	1.43	0.00	0.00	0.00	0.00	0.00	0.00
Greece - Escherichia coli 3GCREC RESP - model 17	50.22	59.17	67.84	96.71	114.76	130.90	147.62	173.73	197.76	0.90	0.99	1.10	0.46	0.54	0.62	0.89	1.06	1.21	1.36	1.60	1.82	142.18	174.68	202.45	1.31	1.61	1.86	5.09	6.29	7.38	0.05	0.06	0.07
Greece - Escherichia coli 3GCREC SSI - model 19	0.66	0.89	1.18	144.47	168.09	190.75	145.19	169.03	191.72	0.38	0.40	0.43	6.11E-03	8.20E-03	0.01	1.33	1.55	1.76	1.34	1.56	1.77	365.16	417.70	471.45	3.36	3.85	4.34	10.41	12.19	14.09	0.10	0.11	0.13
Greece - Escherichia coli 3GCREC UTI - model 18	2.64	3.33	4.17	0.00	0.00	0.00	2.64	3.33	4.17	1.57E-03	1.96E-03	2.36E-03	0.02	0.03	0.04	0.00	0.00	0.00	0.02	0.03	0.04	1,518.10	1,711.28	1,882.69	13.98	15.76	17.34	0.00	0.00	0.00	0.00	0.00	0.00
Greece - Escherichia coli ColREC BSI - model 26	5.27	5.77	6.27	110.41	126.38	143.20	115.96	132.13	149.10	11.05	12.58	14.06	0.05	0.05	0.06	1.02	1.16	1.32	1.07	1.22	1.37	9.70	10.53	11.33	0.09	0.10	0.10	5.32	6.30	7.40	0.05	0.06	0.07
Greece - Escherichia coli ColREC OTH - model 30	0.02	0.02	0.03	0.00	0.00	0.00	0.02	0.02	0.03	2.76E-03	3.51E-03	4.52E-03	1.46E-04	1.95E-04	2.68E-04	0.00	0.00	0.00	1.46E-04	1.95E-04	2.68E-04	5.11	6.05	6.99	0.05	0.06	0.06	0.00	0.00	0.00	0.00	0.00	0.00
Greece - Escherichia coli ColREC RESP - model 27	1.66	1.95	2.23	3.22	3.77	4.26	4.89	5.72	6.46	0.98	1.08	1.23	0.02	0.02	0.02	0.03	0.03	0.04	0.05	0.05	0.06	4.36	5.28	6.11	0.04	0.05	0.06	0.16	0.19	0.22	1.43E-03	1.74E-03	2.06E-03
Greece - Escherichia coli ColREC SSI - model 29	0.02	0.03	0.04	4.49	5.18	5.85	4.50	5.20	5.88	0.40	0.42	0.45	1.77E-04	2.42E-04	3.33E-04	0.04	0.05	0.05	0.04	0.05	0.05	10.63	12.39	13.93	0.10	0.11	0.13	0.30	0.37	0.42	2.78E-03	3.36E-03	3.84E-03
Greece - Escherichia coli ColREC UTI - model 28	0.07	0.09	0.12	0.00	0.00	0.00	0.07	0.09	0.12	1.64E-03	2.09E-03	2.61E-03	6.51E-04	8.45E-04	1.09E-03	0.00	0.00	0.00	6.51E-04	8.45E-04	1.09E-03	39.32	44.14	49.18	0.36	0.41	0.45	0.00	0.00	0.00	0.00	0.00	0.00
Greece - Escherichia coli CREC BSI - model 21	16.44	18.88	21.97	204.45	248.73	308.67	220.97	268.31	328.84	10.69	12.80	15.25	0.15	0.17	0.20	1.88	2.29	2.84	2.04	2.47	3.03	19.33	20.95	22.70	0.18	0.19	0.21	6.34	7.47	8.72	0.06	0.07	0.08
Greece - Escherichia coli CREC OTH - model 25	0.03	0.04	0.06	0.00	0.00	0.00	0.03	0.04	0.06	2.69E-03	3.50E-03	4.50E-03	2.86E-04	3.83E-04	5.19E-04	0.00	0.00	0.00	2.86E-04	3.83E-04	5.19E-04	10.04	11.99	13.84	0.09	0.11	0.13	0.00	0.00	0.00	0.00	0.00	0.00
Greece - Escherichia coli CREC RESP - model 22	5.03	6.54	8.39	9.85	12.85	16.10	14.93	19.40	24.23	1.54	1.82	2.12	0.05	0.06	0.08	0.09	0.12	0.15	0.14	0.18	0.22	8.75	10.69	12.56	0.08	0.10	0.12	0.31	0.38	0.46	2.89E-03	3.54E-03	4.20E-03
Greece - Escherichia coli CREC SSI - model 24	0.04	0.05	0.07	10.48	12.60	14.75	10.51	12.65	14.82	0.48	0.51	0.54	3.57E-04	4.84E-04	6.60E-04	0.10	0.12	0.14	0.10	0.12	0.14	21.29	24.63	27.93	0.20	0.23	0.26	0.48	0.59	0.69	4.46E-03	5.42E-03	6.37E-03
Greece - Escherichia coli CREC UTI - model 23	0.14	0.19	0.24	0.00	0.00	0.00	0.14	0.19	0.24	1.67E-03	2.10E-03	2.67E-03	1.32E-03	1.70E-03	2.24E-03	0.00	0.00	0.00	1.32E-03	1.70E-03	2.24E-03	78.74	88.43	98.83	0.73	0.81	0.91	0.00	0.00	0.00	0.00	0.00	0.00
Greece - Klebsiella pneumoniae 3GCRKP BSI - model 1	117.69	126.76	136.91	756.39	797.27	847.06	877.66	925.28	977.51	5.00	5.23	5.46	1.08	1.17	1.26	6.97	7.34	7.80	8.08	8.52	9.00	169.10	176.97	185.23	1.56	1.63	1.71	27.90	29.54	31.35	0.26	0.27	0.29
Greece - Klebsiella pneumoniae 3GCRKP OTH - model 5	0.19	0.23	0.28	0.00	0.00	0.00	0.19	0.23	0.28	2.79E-03	3.34E-03	3.88E-03	1.73E-03	2.16E-03	2.62E-03	0.00	0.00	0.00	1.73E-03	2.16E-03	2.62E-03	62.30	70.16	78.01	0.57	0.65	0.72	0.00	0.00	0.00	0.00	0.00	0.00
Greece - Klebsiella pneumoniae 3GCRKP RESP - model 2	68.50	76.11	85.28	133.59	148.29	163.88	202.38	224.13	247.28	1.37	1.47	1.58	0.63	0.70	0.79	1.23	1.37	1.51	1.86	2.06	2.28	137.86	152.63	167.52	1.27	1.41	1.54	4.94	5.51	6.08	0.05	0.05	0.06
Greece - Klebsiella pneumoniae 3GCRKP SSI - model 4	0.11	0.15	0.19	26.68	30.49	34.13	26.81	30.66	34.30	0.42	0.44	0.46	1.05E-03	1.36E-03	1.75E-03	0.25	0.28	0.31	0.25	0.28	0.32	61.82	69.53	77.08	0.57	0.64	0.71	1.46	1.71	1.95	0.01	0.02	0.02
Greece - Klebsiella pneumoniae 3GCRKP UTI - model 3	0.33	0.41	0.49	0.00	0.00	0.00	0.33	0.41	0.49	1.64E-03	1.97E-03	2.29E-03	3.04E-03	3.76E-03	4.55E-03	0.00	0.00	0.00	3.04E-03	3.76E-03	4.55E-03	187.16	207.92	226.38	1.72	1.91	2.08	0.00	0.00	0.00	0.00	0.00	0.00
Greece - Klebsiella pneumoniae ColRKP BSI - model 11	274.83	302.81	334.76	5,759.99	6,713.95	7,706.77	6,062.12	7,012.88	8,009.28	14.13	16.15	18.26	2.53	2.79	3.08	53.05	61.83	70.98	55.83	64.59	73.76	401.37	435.01	466.70	3.70	4.01	4.30	225.76	261.89	303.12	2.08	2.41	2.79
Greece - Klebsiella pneumoniae ColRKP OTH - model 15	0.54	0.71	0.95	0.00	0.00	0.00	0.54	0.71	0.95	2.85E-03	3.51E-03	4.36E-03	4.97E-03	6.57E-03	8.78E-03	0.00	0.00	0.00	4.97E-03	6.57E-03	8.78E-03	168.93	202.52	237.77	1.56	1.87	2.19	0.00	0.00	0.00	0.00	0.00	0.00
Greece - Klebsiella pneumoniae ColRKP RESP - model 12	71.44	87.11	102.65	139.02	168.60	199.55	211.98	255.56	301.37	1.28	1.40	1.52	0.66	0.80	0.95	1.28	1.55	1.84	1.95	2.35	2.78	152.74	183.14	212.38	1.41	1.69	1.96	5.50	6.59	7.72	0.05	0.06	0.07
Greece - Klebsiella pneumoniae ColRKP SSI - model 14	0.19	0.27	0.37	43.00	54.14	65.30	43.23	54.47	65.59	0.39	0.42	0.46	1.79E-03	2.51E-03	3.38E-03	0.40	0.50	0.60	0.40	0.50	0.60	105.03	128.18	152.62	0.97	1.18	1.41	2.24	2.92	3.56	0.02	0.03	0.03
Greece - Klebsiella pneumoniae ColRKP UTI - model 13	0.42	0.55	0.71	0.00	0.00	0.00	0.42	0.55	0.71	1.69E-03	2.10E-03	2.53E-03	3.89E-03	5.04E-03	6.53E-03	0.00	0.00	0.00	3.89E-03	5.04E-03	6.53E-03	222.08	262.71	301.82	2.05	2.42	2.78	0.00	0.00	0.00	0.00	0.00	0.00
Greece - Klebsiella pneumoniae CRKP BSI - model 6	654.97	711.60	778.72	8,240.54	9,334.49	10,446.92	8,935.48	10,049.83	11,199.14	8.72	9.73	10.67	6.03	6.55	7.17	75.89	85.97	96.21	82.29	92.56	103.14	967.90	1,033.56	1,107.85	8.91	9.52	10.20	323.16	368.66	416.64	2.98	3.40	3.84
Greece - Klebsiella pneumoniae CRKP OTH - model 10	1.30	1.69	2.18	0.00	0.00	0.00	1.30	1.69	2.18	2.85E-03	3.52E-03	4.18E-03	0.01	0.02	0.02	0.00	0.00	0.00	0.01	0.02	0.02	403.49	482.12	560.05	3.72	4.44	5.16	0.00	0.00	0.00	0.00	0.00	0.00
Greece - Klebsiella pneumoniae CRKP RESP - model 7	174.32	205.53	237.97	329.15	399.32	461.27	510.47	605.16	698.63	1.27	1.38	1.51	1.61	1.89	2.19	3.03	3.68	4.25	4.70	5.57	6.43	370.19	436.71	509.69	3.41	4.02	4.69	13.26	15.72	18.51	0.12	0.14	0.17
Greece - Klebsiella pneumoniae CRKP SSI - model 9	0.49	0.65	0.84	109.09	131.83	155.72	109.63	132.47	156.95	0.40	0.44	0.47	4.49E-03	5.97E-03	7.78E-03	1.00	1.21	1.43	1.01	1.22	1.45	256.31	304.98	357.62	2.36	2.81	3.29	5.82	7.31	8.88	0.05	0.07	0.08
Greece - Klebsiella pneumoniae CRKP UTI - model 8	1.02	1.31	1.67	0.00	0.00	0.00	1.02	1.31	1.67	1.69E-03	2.10E-03	2.52E-03	9.41E-03	0.01	0.02	0.00	0.00	0.00	9.41E-03	0.01	0.02	541.10	625.86	714.98	4.98	5.76	6.58	0.00	0.00	0.00	0.00	0.00	0.00
Greece - Pseudomonas aeruginosa ColRPA BSI - model 56	18.39	20.71	23.27	136.18	176.06	219.12	157.30	196.93	239.07	5.93	7.29	8.76	0.17	0.19	0.21	1.25	1.62	2.02	1.45	1.81	2.20	24.30	26.89	29.61	0.22	0.25	0.27	4.74	6.16	7.55	0.04	0.06	0.07
Greece - Pseudomonas aeruginosa ColRPA OTH - model 60	0.04	0.05	0.07	0.00	0.00	0.00	0.04	0.05	0.07	2.97E-03	3.75E-03	4.53E-03	3.44E-04	4.64E-04	6.08E-04	0.00	0.00	0.00	3.44E-04	4.64E-04	6.08E-04	10.73	13.59	16.29	0.10	0.13	0.15	0.00	0.00	0.00	0.00	0.00	0.00
Greece - Pseudomonas aeruginosa ColRPA RESP - model 57	0.00	0.00	0.00	0.00	0.00	0.00	0.00	0.00	0.00	NaN	NaN	NaN	0.00	0.00	0.00	0.00	0.00	0.00	0.00	0.00	0.00	0.00	0.00	0.00	0.00	0.00	0.00	0.00	0.00	0.00	0.00	0.00	0.00
Greece - Pseudomonas aeruginosa ColRPA SSI - model 59	0.03	0.04	0.05	6.93	8.93	10.85	6.97	8.97	10.89	0.44	0.49	0.52	2.54E-04	3.59E-04	5.00E-04	0.06	0.08	0.10	0.06	0.08	0.10	15.02	18.45	22.02	0.14	0.17	0.20	0.32	0.43	0.53	2.92E-03	3.93E-03	4.90E-03
Greece - Pseudomonas aeruginosa ColRPA UTI - model 58	0.05	0.07	0.09	0.00	0.00	0.00	0.05	0.07	0.09	1.65E-03	2.05E-03	2.52E-03	4.96E-04	6.49E-04	8.38E-04	0.00	0.00	0.00	4.96E-04	6.49E-04	8.38E-04	28.53	34.65	40.32	0.26	0.32	0.37	0.00	0.00	0.00	0.00	0.00	0.00
Greece - Pseudomonas aeruginosa CRPA BSI - model 51	371.40	404.83	442.37	2,867.61	3,444.75	4,066.36	3,269.62	3,846.17	4,490.63	6.01	7.01	8.00	3.42	3.73	4.07	26.41	31.73	37.45	30.11	35.42	41.36	509.25	549.94	591.81	4.69	5.06	5.45	102.50	125.36	149.42	0.94	1.15	1.38
Greece - Pseudomonas aeruginosa CRPA OTH - model 55	0.81	1.02	1.27	0.00	0.00	0.00	0.81	1.02	1.27	3.11E-03	3.66E-03	4.31E-03	7.43E-03	9.39E-03	0.01	0.00	0.00	0.00	7.43E-03	9.39E-03	0.01	236.42	279.18	322.82	2.18	2.57	2.97	0.00	0.00	0.00	0.00	0.00	0.00
Greece - Pseudomonas aeruginosa CRPA RESP - model 52	920.02	1,031.56	1,148.31	3,090.91	3,664.42	4,313.30	4,032.98	4,698.61	5,415.43	2.06	2.31	2.59	8.47	9.50	10.58	28.47	33.75	39.72	37.14	43.27	49.87	1,813.27	2,036.45	2,237.55	16.70	18.76	20.61	110.80	134.16	159.01	1.02	1.24	1.46
Greece - Pseudomonas aeruginosa CRPA SSI - model 54	0.61	0.80	1.03	133.07	161.34	187.19	134.31	162.10	188.50	0.40	0.43	0.46	5.63E-03	7.37E-03	9.45E-03	1.23	1.49	1.72	1.24	1.49	1.74	321.44	376.22	429.72	2.96	3.46	3.96	6.85	8.34	9.88	0.06	0.08	0.09
Greece - Pseudomonas aeruginosa CRPA UTI - model 53	0.00	0.00	0.00	0.00	0.00	0.00	0.00	0.00	0.00	NaN	NaN	NaN	0.00	0.00	0.00	0.00	0.00	0.00	0.00	0.00	0.00	0.00	0.00	0.00	0.00	0.00	0.00	0.00	0.00	0.00	0.00	0.00	0.00
Greece - Pseudomonas aeruginosa MDRPA BSI - model 46	8.74	9.63	10.69	62.01	75.70	90.39	71.67	85.39	100.52	4.34	5.16	6.00	0.08	0.09	0.10	0.57	0.70	0.83	0.66	0.79	0.93	15.13	16.49	18.08	0.14	0.15	0.17	2.79	3.49	4.26	0.03	0.03	0.04
Greece - Pseudomonas aeruginosa MDRPA OTH - model 50	0.02	0.03	0.04	0.00	0.00	0.00	0.02	0.03	0.04	3.01E-03	3.79E-03	4.53E-03	2.12E-04	2.89E-04	3.70E-04	0.00	0.00	0.00	2.12E-04	2.89E-04	3.70E-04	6.69	8.29	9.74	0.06	0.08	0.09	0.00	0.00	0.00	0.00	0.00	0.00
Greece - Pseudomonas aeruginosa MDRPA RESP - model 47	21.55	24.55	27.76	67.24	82.23	99.26	89.97	106.82	125.23	1.53	1.76	1.99	0.20	0.23	0.26	0.62	0.76	0.91	0.83	0.98	1.15	53.12	61.17	68.99	0.49	0.56	0.64	2.99	3.79	4.69	0.03	0.03	0.04
Greece - Pseudomonas aeruginosa MDRPA SSI - model 49	0.02	0.02	0.03	3.76	4.77	5.65	3.78	4.79	5.68	0.39	0.42	0.45	1.57E-04	2.23E-04	3.05E-04	0.03	0.04	0.05	0.03	0.04	0.05	9.18	11.43	13.45	0.08	0.11	0.12	0.23	0.29	0.36	2.15E-03	2.70E-03	3.28E-03
Greece - Pseudomonas aeruginosa MDRPA UTI - model 48	0.03	0.04	0.06	0.00	0.00	0.00	0.03	0.04	0.06	1.62E-03	2.05E-03	2.49E-03	3.04E-04	3.99E-04	5.17E-04	0.00	0.00	0.00	3.04E-04	3.99E-04	5.17E-04	17.92	21.30	24.57	0.17	0.20	0.23	0.00	0.00	0.00	0.00	0.00	0.00
Greece - Staphylococcus aureus MRSA BSI - model 61	158.15	173.82	189.69	1,080.13	1,165.94	1,258.36	1,240.69	1,340.29	1,444.79	4.01	4.26	4.51	1.46	1.60	1.75	9.95	10.74	11.59	11.43	12.34	13.31	288.28	314.70	346.01	2.66	2.90	3.19	51.44	56.51	61.92	0.47	0.52	0.57
Greece - Staphylococcus aureus MRSA OTH - model 65	1.21	1.53	1.92	0.00	0.00	0.00	1.21	1.53	1.92	2.65E-03	3.27E-03	3.93E-03	0.01	0.01	0.02	0.00	0.00	0.00	0.01	0.01	0.02	413.44	469.45	535.29	3.81	4.32	4.93	0.00	0.00	0.00	0.00	0.00	0.00
Greece - Staphylococcus aureus MRSA RESP - model 62	163.02	184.40	209.55	318.67	358.22	402.61	483.58	543.15	611.02	1.03	1.13	1.22	1.50	1.70	1.93	2.93	3.30	3.71	4.45	5.00	5.63	417.62	482.50	549.39	3.85	4.44	5.06	14.90	17.36	19.88	0.14	0.16	0.18
Greece - Staphylococcus aureus MRSA SSI - model 64	0.83	1.11	1.51	185.63	211.65	238.82	186.59	212.81	239.93	0.38	0.41	0.43	7.62E-03	0.01	0.01	1.71	1.95	2.20	1.72	1.96	2.21	460.24	522.99	592.26	4.24	4.82	5.45	12.45	14.50	16.78	0.11	0.13	0.15
Greece - Staphylococcus aureus MRSA UTI - model 63	0.11	0.15	0.20	0.00	0.00	0.00	0.11	0.15	0.20	1.52E-03	1.88E-03	2.28E-03	1.01E-03	1.39E-03	1.83E-03	0.00	0.00	0.00	1.01E-03	1.39E-03	1.83E-03	64.92	81.08	98.91	0.60	0.75	0.91	0.00	0.00	0.00	0.00	0.00	0.00
Greece - Streptococcus pneumoniae PMRSP BSI - model 76	0.00	0.00	0.00	0.00	0.00	0.00	0.00	0.00	0.00	NaN	NaN	NaN	0.00	0.00	0.00	0.00	0.00	0.00	0.00	0.00	0.00	0.00	0.00	0.00	0.00	0.00	0.00	0.00	0.00	0.00	0.00	0.00	0.00
Greece - Streptococcus pneumoniae PMRSP OTH - model 80	0.00	0.00	0.00	0.00	0.00	0.00	0.00	0.00	0.00	NaN	NaN	NaN	0.00	0.00	0.00	0.00	0.00	0.00	0.00	0.00	0.00	0.00	0.00	0.00	0.00	0.00	0.00	0.00	0.00	0.00	0.00	0.00	0.00
Greece - Streptococcus pneumoniae PMRSP RESP - model 77	0.00	0.00	0.00	0.00	0.00	0.00	0.00	0.00	0.00	NaN	NaN	NaN	0.00	0.00	0.00	0.00	0.00	0.00	0.00	0.00	0.00	0.00	0.00	0.00	0.00	0.00	0.00	0.00	0.00	0.00	0.00	0.00	0.00
Greece - Streptococcus pneumoniae PMRSP SSI - model 79	0.00	0.00	0.00	0.00	0.00	0.00	0.00	0.00	0.00	NaN	NaN	NaN	0.00	0.00	0.00	0.00	0.00	0.00	0.00	0.00	0.00	0.00	0.00	0.00	0.00	0.00	0.00	0.00	0.00	0.00	0.00	0.00	0.00
Greece - Streptococcus pneumoniae PMRSP UTI - model 78	0.00	0.00	0.00	0.00	0.00	0.00	0.00	0.00	0.00	NaN	NaN	NaN	0.00	0.00	0.00	0.00	0.00	0.00	0.00	0.00	0.00	0.00	0.00	0.00	0.00	0.00	0.00	0.00	0.00	0.00	0.00	0.00	0.00
Greece - Streptococcus pneumoniae PRSP BSI - model 71	0.00	0.00	0.00	0.00	0.00	0.00	0.00	0.00	0.00	NaN	NaN	NaN	0.00	0.00	0.00	0.00	0.00	0.00	0.00	0.00	0.00	0.00	0.00	0.00	0.00	0.00	0.00	0.00	0.00	0.00	0.00	0.00	0.00
Greece - Streptococcus pneumoniae PRSP OTH - model 75	0.00	0.00	0.00	0.00	0.00	0.00	0.00	0.00	0.00	NaN	NaN	NaN	0.00	0.00	0.00	0.00	0.00	0.00	0.00	0.00	0.00	0.00	0.00	0.00	0.00	0.00	0.00	0.00	0.00	0.00	0.00	0.00	0.00
Greece - Streptococcus pneumoniae PRSP RESP - model 72	0.00	0.00	0.00	0.00	0.00	0.00	0.00	0.00	0.00	NaN	NaN	NaN	0.00	0.00	0.00	0.00	0.00	0.00	0.00	0.00	0.00	0.00	0.00	0.00	0.00	0.00	0.00	0.00	0.00	0.00	0.00	0.00	0.00
Greece - Streptococcus pneumoniae PRSP SSI - model 74	0.00	0.00	0.00	0.00	0.00	0.00	0.00	0.00	0.00	NaN	NaN	NaN	0.00	0.00	0.00	0.00	0.00	0.00	0.00	0.00	0.00	0.00	0.00	0.00	0.00	0.00	0.00	0.00	0.00	0.00	0.00	0.00	0.00
Greece - Streptococcus pneumoniae PRSP UTI - model 73	0.00	0.00	0.00	0.00	0.00	0.00	0.00	0.00	0.00	NaN	NaN	NaN	0.00	0.00	0.00	0.00	0.00	0.00	0.00	0.00	0.00	0.00	0.00	0.00	0.00	0.00	0.00	0.00	0.00	0.00	0.00	0.00	0.00
**Total (Median & 95% UIs)**	10,820.16	5,624.30	6,202.95	75,011.91	39,810.68	45,372.58	86,025.35	45,443.62	51,389.92		-		99.65	51.80	57.13	690.84	366.65	417.87	792.27	418.53	473.29	34,703.01	18,332.40	20,420.24	319.61	168.84	188.07	2,992.14	1,597.74	1,842.58	27.56	14.71	16.97
**Total (average)**		**5,738.92**			**40,695.70**			**46,434.62**			**-**			**52.85**			**374.80**			**427.65**			**18,476.06**			**170.16**			**1,627.22**			**14.99**	

Infection	YLD			YLL			DALY			DALY per Case			YLD per 100,000			YLL per 100,000			DALY per 100,000			Cases per year			Incidence per 100,000			Deaths per year			Mortality per 100,000		
Colistin resistant *Acinetobacter spp.*	4.89	8.25	12.80	17.09	44.44	80.95	22.86	53.27	92.74	8.67	16.17	23.32	0.01	0.02	0.03	0.04	0.10	0.17	0.05	0.11	0.20	5.40	9.76	14.81	0.01	0.02	0.03	0.32	0.82	1.50	0.00	0.00	0.00
Carbapenem resistant *Acinetobacter spp.* (excluding those resistant to colistin)	180.13	207.71	240.65	867.44	1,119.60	1,370.43	1,055.15	1,329.96	1,595.48	6.67	7.96	9.25	0.39	0.45	0.52	1.87	2.41	2.95	2.27	2.86	3.43	424.90	493.97	566.39	0.91	1.06	1.22	33.70	43.11	53.21	0.07	0.09	0.11
Aminoglycosides and fluoroquinolones resistant *Acinetobacter* spp. (excluding those resistant to colistin and/or carbapenem)	12.47	15.65	19.18	56.99	82.20	111.23	70.93	98.11	129.24	3.25	4.27	5.47	0.03	0.03	0.04	0.12	0.18	0.24	0.15	0.21	0.28	55.03	69.34	84.84	0.12	0.15	0.18	4.31	6.02	7.98	0.01	0.01	0.02
Vancomycin resistant Enterococci (*E. faecalis* and *E. faecium*)	26.54	36.41	48.95	257.16	344.31	457.94	280.07	380.62	504.32	4.96	5.68	6.58	0.06	0.08	0.11	0.55	0.74	0.99	0.60	0.82	1.09	188.35	258.58	332.47	0.41	0.56	0.72	13.31	17.45	22.08	0.03	0.04	0.05
Colistin resistant *Escherichia coli*	24.19	29.96	37.31	384.40	509.86	652.52	411.08	540.47	685.05	10.03	12.47	15.26	0.05	0.06	0.08	0.83	1.10	1.40	0.89	1.16	1.47	275.83	335.66	397.33	0.59	0.72	0.86	22.89	29.27	35.95	0.05	0.06	0.08
Carbapenem resistant *Escherichia coli* (excluding those resistant to colistin)	2.09	3.69	6.19	20.41	39.34	65.95	22.43	42.75	71.96	8.80	13.60	18.10	0.00	0.01	0.01	0.04	0.08	0.14	0.05	0.09	0.15	13.73	25.68	38.53	0.03	0.06	0.08	0.68	1.31	2.21	0.00	0.00	0.00
Third-generation cephalosporin resistant *Escherichia coli* (excluding those resistant to colistin and/or carbapenem)	1,331.90	1,505.38	1,693.97	7,966.45	8,934.44	10,031.55	9,324.17	10,432.11	11,648.62	4.12	4.54	4.99	2.87	3.24	3.65	17.15	19.23	21.60	20.07	22.46	25.08	15,795.33	18,425.79	21,045.18	34.01	39.67	45.31	491.11	567.70	656.87	1.06	1.22	1.41
Colistin resistant *Klebsiella pneumoniae*	10.12	14.10	18.83	171.21	251.04	359.76	182.33	265.48	375.04	15.13	20.64	26.15	0.02	0.03	0.04	0.37	0.54	0.77	0.39	0.57	0.81	26.94	38.88	51.80	0.06	0.08	0.11	6.03	8.58	11.98	0.01	0.02	0.03
Carbapenem resistant *Klebsiella pneumoniae* (excluding those resistant to colistin)	92.03	110.55	131.68	969.84	1,185.49	1,446.26	1,068.99	1,296.15	1,573.54	11.65	13.75	16.08	0.20	0.24	0.28	2.09	2.55	3.11	2.30	2.79	3.39	240.62	292.52	347.45	0.52	0.63	0.75	32.54	39.32	46.68	0.07	0.08	0.10
Third-generation cephalosporin resistant *Klebsiella pneumoniae* (excluding those resistant to colistin and/or carbapenem)	877.00	970.47	1,067.91	4,294.17	4,644.18	5,036.10	5,138.10	5,612.47	6,086.28	6.28	6.65	7.05	1.89	2.09	2.30	9.24	10.00	10.84	11.06	12.08	13.10	3,114.62	3,488.68	3,873.67	6.71	7.51	8.34	172.69	188.71	205.75	0.37	0.41	0.44
Colistin resistant *Pseudomonas aeruginosa*	45.28	60.45	77.84	182.59	302.44	443.10	235.46	363.74	516.21	8.68	12.45	16.62	0.10	0.13	0.17	0.39	0.65	0.95	0.51	0.78	1.11	95.35	128.49	162.14	0.21	0.28	0.35	5.21	8.44	12.27	0.01	0.02	0.03
Carbapenem resistant *Pseudomonas aeruginosa* (excluding those resistant to colistin)	1,566.19	1,774.54	1,995.55	7,341.51	8,958.79	10,795.89	8,992.85	10,726.53	12,686.88	8.12	9.51	11.01	3.37	3.82	4.30	15.81	19.29	23.24	19.36	23.09	27.31	4,316.79	5,003.83	5,713.88	9.29	10.77	12.30	271.81	339.18	414.12	0.59	0.73	0.89
Pseudomonas aeruginosa resistant to three or more antimicrobial groups (excluding those resistant to colistin and/or carbapenem)	60.79	75.92	92.57	255.63	357.99	478.22	323.43	435.11	566.32	5.26	6.85	8.72	0.13	0.16	0.20	0.55	0.77	1.03	0.70	0.94	1.22	223.62	280.84	340.69	0.48	0.60	0.73	12.69	18.01	23.66	0.03	0.04	0.05
Methicillin resistant *Staphylococcus aureus*	1.555.41	1.721.24	1.899.14	7.674.28	8.368.15	9.031.65	9.267.34	10.089.67	10.873.83	4.98	5.27	5.57	3.35	3.71	4.09	16.52	18.02	19.44	19.95	21.72	23.41	8.777.87	9.836.53	10.908.83	18.90	21.18	23.49	423.59	469.48	516.90	0.91	1.01	1.11
Penicillin and macrolides resistant *Streptococcus pneumoniae* (excluding those only resistant to penicillin)	667.87	786.04	944.03	2,292.55	2,561.82	2,915.47	2,984.40	3,351.45	3,833.22	8.26	8.86	9.50	1.44	1.69	2.03	4.94	5.52	6.28	6.43	7.22	8.25	1,107.92	1,239.86	1,394.22	2.39	2.67	3.00	69.34	75.58	82.93	0.15	0.16	0.18
Penicillin tesistant *Streptococcus pneumoniae* (excluding those resistant to macrolides)	791.07	896.36	1,022.20	2,681.46	2,906.46	3,189.51	3,495.34	3,805.93	4,182.98	8.42	8.81	9.26	1.70	1.93	2.20	5.77	6.26	6.87	7.53	8.19	9.01	1,294.94	1,416.61	1,565.03	2.79	3.05	3.37	80.36	86.26	93.16	0.17	0.19	0.20
Total	7,247.98	8,216.72	9,308.80	35,433.20	40,610.56	46,466.54	42,874.93	48,823.81	55,421.71				15.60	17.69	20.04	76.28	87.43	100.04	92.30	105.11	119.32	35,957.22	41,345.01	46,837.28	77.41	89.01	100.83	1,640.58	1,899.25	2,187.24	3.53	4.09	4.71

Model	YLD			YLL			DALY			DALY per Case			YLD per 100,000			YLL per 100,000			DALY per 100,000			Cases per year			Incidence per 100,000			Deaths per year			Mortality per 100,000		
Spain - Acinetobacter spp. CRACI BSI - model 36	104.73	118.25	135.39	698.74	918.09	1,133.65	808.81	1,038.37	1,255.26	4.94	6.10	7.25	0.23	0.25	0.29	1.50	1.98	2.44	1.74	2.24	2.70	155.67	169.74	187.62	0.34	0.37	0.40	26.98	35.07	43.84	0.06	0.08	0.09
Spain - Acinetobacter spp. CRACI OTH - model 40	0.02	0.02	0.03	0.00	0.00	0.00	0.02	0.02	0.03	2.90E-03	3.76E-03	4.62E-03	3.28E-05	5.01E-05	7.23E-05	0.00	0.00	0.00	3.28E-05	5.01E-05	7.23E-05	4.49	6.25	8.26	9.67E-03	0.01	0.02	0.00	0.00	0.00	0.00	0.00	0.00
Spain - Acinetobacter spp. CRACI RESP - model 37	75.18	89.16	104.85	147.48	174.14	203.34	224.90	263.91	306.33	1.33	1.43	1.53	0.16	0.19	0.23	0.32	0.37	0.44	0.48	0.57	0.66	157.28	184.69	212.54	0.34	0.40	0.46	5.67	6.66	7.68	0.01	0.01	0.02
Spain - Acinetobacter spp. CRACI SSI - model 39	0.10	0.13	0.19	21.22	27.38	33.44	21.32	27.50	33.67	0.40	0.44	0.47	2.09E-04	2.86E-04	4.04E-04	0.05	0.06	0.07	0.05	0.06	0.07	50.74	62.97	74.30	0.11	0.14	0.16	1.05	1.38	1.69	2.27E-03	2.98E-03	3.63E-03
Spain - Acinetobacter spp. CRACI UTI - model 38	0.11	0.14	0.19	0.00	0.00	0.00	0.11	0.14	0.19	1.62E-03	2.03E-03	2.50E-03	2.32E-04	3.05E-04	4.06E-04	0.00	0.00	0.00	2.32E-04	3.05E-04	4.06E-04	56.73	70.32	83.66	0.12	0.15	0.18	0.00	0.00	0.00	0.00	0.00	0.00
Spain - Acinetobacter spp. ColRACI BSI - model 41	3.05	4.74	7.13	13.11	36.83	69.00	17.08	42.09	75.43	5.70	12.73	19.47	6.57E-03	0.01	0.02	0.03	0.08	0.15	0.04	0.09	0.16	2.45	3.32	4.52	5.28E-03	7.15E-03	9.72E-03	0.24	0.68	1.28	5.23E-04	1.47E-03	2.75E-03
Spain - Acinetobacter spp. ColRACI OTH - model 45	5.39E-05	3.88E-04	1.31E-03	0.00	0.00	0.00	5.39E-05	3.88E-04	1.31E-03	1.56E-03	3.75E-03	6.40E-03	1.16E-07	8.35E-07	2.82E-06	0.00	0.00	0.00	1.16E-07	8.35E-07	2.82E-06	0.02	0.12	0.27	4.19E-05	2.48E-04	5.89E-04	0.00	0.00	0.00	0.00	0.00	0.00
Spain - Acinetobacter spp. ColRACI RESP - model 42	1.84	3.50	5.66	3.76	6.98	10.91	5.57	10.54	16.26	2.49	2.95	3.36	3.96E-03	7.54E-03	0.01	8.10E-03	0.02	0.02	0.01	0.02	0.04	2.00	3.63	5.49	4.30E-03	7.82E-03	0.01	0.07	0.13	0.20	1.50E-04	2.78E-04	4.35E-04
Spain - Acinetobacter spp. ColRACI SSI - model 44	4.79E-04	2.41E-03	6.87E-03	0.22	0.63	1.04	0.22	0.63	1.04	0.49	0.49	0.49	1.03E-06	5.19E-06	1.48E-05	4.65E-04	1.36E-03	2.24E-03	4.66E-04	1.37E-03	2.24E-03	0.44	1.30	2.14	9.56E-04	2.80E-03	4.60E-03	4.00E-03	0.01	0.02	8.61E-06	2.52E-05	4.14E-05
Spain - Acinetobacter spp. ColRACI UTI - model 43	7.15E-04	2.53E-03	6.57E-03	0.00	0.00	0.00	7.15E-04	2.53E-03	6.57E-03	8.40E-04	1.99E-03	3.58E-03	1.54E-06	5.45E-06	1.41E-05	0.00	0.00	0.00	1.54E-06	5.45E-06	1.41E-05	0.49	1.39	2.40	1.04E-03	2.99E-03	5.16E-03	0.00	0.00	0.00	0.00	0.00	0.00
Spain - Acinetobacter spp. MDRACI BSI - model 31	7.28	8.92	10.75	44.68	66.03	90.78	53.65	75.15	100.70	2.27	3.14	4.15	0.02	0.02	0.02	0.10	0.14	0.20	0.12	0.16	0.22	20.80	23.75	27.49	0.04	0.05	0.06	3.37	4.80	6.44	7.26E-03	0.01	0.01
Spain - Acinetobacter spp. MDRACI OTH - model 35	1.63E-03	3.13E-03	5.44E-03	0.00	0.00	0.00	1.63E-03	3.13E-03	5.44E-03	2.52E-03	3.71E-03	5.05E-03	3.50E-06	6.74E-06	1.17E-05	0.00	0.00	0.00	3.50E-06	6.74E-06	1.17E-05	0.50	0.86	1.28	1.07E-03	1.85E-03	2.75E-03	0.00	0.00	0.00	0.00	0.00	0.00
Spain - Acinetobacter spp. MDRACI RESP - model 32	5.16	6.68	8.36	9.95	12.83	16.03	14.87	19.56	24.08	0.64	0.75	0.87	0.01	0.01	0.02	0.02	0.03	0.03	0.03	0.04	0.05	20.18	26.06	31.50	0.04	0.06	0.07	0.73	0.94	1.16	1.57E-03	2.02E-03	2.50E-03
Spain - Acinetobacter spp. MDRACI SSI - model 34	0.01	0.02	0.03	2.36	3.35	4.41	2.39	3.37	4.43	0.33	0.38	0.44	2.26E-05	4.01E-05	6.46E-05	5.09E-03	7.22E-03	9.50E-03	5.14E-03	7.26E-03	9.55E-03	6.47	8.74	11.55	0.01	0.02	0.02	0.20	0.28	0.38	4.41E-04	6.06E-04	8.11E-04
Spain - Acinetobacter spp. MDRACI UTI - model 33	0.01	0.02	0.03	0.00	0.00	0.00	0.01	0.02	0.03	1.41E-03	2.00E-03	2.78E-03	2.60E-05	4.29E-05	6.53E-05	0.00	0.00	0.00	2.60E-05	4.29E-05	6.53E-05	7.08	9.93	13.02	0.02	0.02	0.03	0.00	0.00	0.00	0.00	0.00	0.00
Spain - Enterococcus faecalis and E. faecium VRE BSI - model 66	26.31	36.05	48.39	230.67	306.83	409.06	253.39	342.76	454.97	4.58	5.25	6.11	0.06	0.08	0.10	0.50	0.66	0.88	0.55	0.74	0.98	50.47	65.67	82.67	0.11	0.14	0.18	11.55	15.02	18.93	0.02	0.03	0.04
Spain - Enterococcus faecalis and E. faecium VRE OTH - model 70	0.08	0.12	0.17	0.00	0.00	0.00	0.08	0.12	0.17	1.17E-03	1.65E-03	2.19E-03	1.62E-04	2.48E-04	3.70E-04	0.00	0.00	0.00	1.62E-04	2.48E-04	3.70E-04	48.86	70.25	90.82	0.11	0.15	0.20	0.00	0.00	0.00	0.00	0.00	0.00
Spain - Enterococcus faecalis and E. faecium VRE RESP - model 67	0.00	0.00	0.00	0.00	0.00	0.00	0.00	0.00	0.00	NaN	NaN	NaN	0.00	0.00	0.00	0.00	0.00	0.00	0.00	0.00	0.00	0.00	0.00	0.00	0.00	0.00	0.00	0.00	0.00	0.00	0.00	0.00	0.00
Spain - Enterococcus faecalis and E. faecium VRE SSI - model 69	0.12	0.19	0.29	26.49	37.48	48.88	26.57	37.68	49.08	0.38	0.42	0.47	2.49E-04	4.08E-04	6.32E-04	0.06	0.08	0.11	0.06	0.08	0.11	64.90	89.62	115.79	0.14	0.19	0.25	1.76	2.42	3.15	3.79E-03	5.22E-03	6.78E-03
Spain - Enterococcus faecalis and E. faecium VRE UTI - model 68	0.04	0.06	0.09	0.00	0.00	0.00	0.04	0.06	0.09	1.37E-03	1.86E-03	2.39E-03	8.59E-05	1.31E-04	1.96E-04	0.00	0.00	0.00	8.59E-05	1.31E-04	1.96E-04	24.12	33.04	43.20	0.05	0.07	0.09	0.00	0.00	0.00	0.00	0.00	0.00
Spain - Escherichia coli 3GCREC BSI - model 16	1,013.95	1,124.26	1,244.30	6,541.74	7,250.48	8,074.76	7,577.79	8,364.80	9,249.11	2.98	3.27	3.56	2.18	2.42	2.68	14.08	15.61	17.38	16.31	18.01	19.91	2,313.69	2,560.44	2,854.83	4.98	5.51	6.15	391.62	444.22	508.36	0.84	0.96	1.09
Spain - Escherichia coli 3GCREC OTH - model 20	2.02	2.86	3.96	0.00	0.00	0.00	2.02	2.86	3.96	2.63E-03	3.33E-03	4.22E-03	4.35E-03	6.15E-03	8.52E-03	0.00	0.00	0.00	4.35E-03	6.15E-03	8.52E-03	672.31	862.16	1,053.68	1.45	1.86	2.27	0.00	0.00	0.00	0.00	0.00	0.00
Spain - Escherichia coli 3GCREC RESP - model 17	295.06	350.88	410.29	562.44	674.84	782.49	860.21	1,028.44	1,188.74	0.79	0.89	1.02	0.64	0.76	0.88	1.21	1.45	1.68	1.85	2.21	2.56	904.51	1,145.42	1,394.68	1.95	2.47	3.00	32.39	41.33	50.26	0.07	0.09	0.11
Spain - Escherichia coli 3GCREC SSI - model 19	4.17	5.77	7.92	862.27	1,009.11	1,174.30	867.45	1,014.42	1,179.31	0.34	0.37	0.40	8.98E-03	0.01	0.02	1.86	2.17	2.53	1.87	2.18	2.54	2,289.23	2,737.97	3,214.56	4.93	5.89	6.92	67.10	82.14	98.24	0.14	0.18	0.21
Spain - Escherichia coli 3GCREC UTI - model 18	16.69	21.60	27.50	0.00	0.00	0.00	16.69	21.60	27.50	1.53E-03	1.94E-03	2.42E-03	0.04	0.05	0.06	0.00	0.00	0.00	0.04	0.05	0.06	9,615.59	11,119.80	12,527.42	20.70	23.94	26.97	0.00	0.00	0.00	0.00	0.00	0.00
Spain - Escherichia coli CREC BSI - model 21	1.73	2.71	4.42	19.18	36.35	61.01	20.82	38.81	65.37	7.12	11.68	15.95	3.73E-03	5.84E-03	9.51E-03	0.04	0.08	0.13	0.04	0.08	0.14	2.43	3.39	4.82	5.23E-03	7.31E-03	0.01	0.64	1.21	2.04	1.37E-03	2.60E-03	4.40E-03
Spain - Escherichia coli CREC OTH - model 25	1.60E-03	6.24E-03	0.02	0.00	0.00	0.00	1.60E-03	6.24E-03	0.02	1.12E-03	3.32E-03	7.52E-03	3.45E-06	1.34E-05	3.96E-05	0.00	0.00	0.00	3.45E-06	1.34E-05	3.96E-05	0.80	1.99	3.23	1.72E-03	4.28E-03	6.96E-03	0.00	0.00	0.00	0.00	0.00	0.00
Spain - Escherichia coli CREC RESP - model 22	0.35	0.94	1.66	0.68	1.86	3.21	1.05	2.77	4.77	1.41	1.64	1.86	7.44E-04	2.02E-03	3.58E-03	1.46E-03	4.01E-03	6.91E-03	2.26E-03	5.97E-03	0.01	0.65	1.69	2.88	1.39E-03	3.65E-03	6.20E-03	0.02	0.06	0.11	4.84E-05	1.33E-04	2.30E-04
Spain - Escherichia coli CREC SSI - model 24	1.77E-03	7.74E-03	0.02	0.55	1.13	1.72	0.56	1.13	1.74	0.27	0.27	0.28	3.82E-06	1.67E-05	5.33E-05	1.19E-03	2.42E-03	3.71E-03	1.20E-03	2.44E-03	3.74E-03	2.04	4.16	6.37	4.40E-03	8.96E-03	0.01	0.02	0.04	0.06	3.96E-05	8.07E-05	1.23E-04
Spain - Escherichia coli CREC UTI - model 23	0.01	0.03	0.07	0.00	0.00	0.00	0.01	0.03	0.07	8.12E-04	2.03E-03	4.08E-03	2.18E-05	6.05E-05	1.46E-04	0.00	0.00	0.00	2.18E-05	6.05E-05	1.46E-04	7.81	14.44	21.23	0.02	0.03	0.05	0.00	0.00	0.00	0.00	0.00	0.00
Spain - Escherichia coli ColREC BSI - model 26	18.21	21.78	26.40	358.21	475.92	610.19	378.93	498.24	631.85	8.86	11.13	13.72	0.04	0.05	0.06	0.77	1.02	1.31	0.82	1.07	1.36	39.11	44.56	50.86	0.08	0.10	0.11	21.10	26.97	33.11	0.05	0.06	0.07
Spain - Escherichia coli ColREC OTH - model 30	0.06	0.09	0.13	0.00	0.00	0.00	0.06	0.09	0.13	2.50E-03	3.52E-03	4.79E-03	1.29E-04	1.95E-04	2.87E-04	0.00	0.00	0.00	1.29E-04	1.95E-04	2.87E-04	20.25	25.99	31.80	0.04	0.06	0.07	0.00	0.00	0.00	0.00	0.00	0.00
Spain - Escherichia coli ColREC RESP - model 27	5.57	7.59	10.06	10.82	14.66	19.05	16.37	22.34	29.02	0.83	0.97	1.14	0.01	0.02	0.02	0.02	0.03	0.04	0.04	0.05	0.06	17.30	22.78	28.54	0.04	0.05	0.06	0.62	0.82	1.03	1.34E-03	1.77E-03	2.23E-03
Spain - Escherichia coli ColREC SSI - model 29	0.07	0.11	0.17	15.36	19.28	23.28	15.45	19.40	23.49	0.34	0.36	0.39	1.56E-04	2.41E-04	3.56E-04	0.03	0.04	0.05	0.03	0.04	0.05	42.71	53.37	64.79	0.09	0.11	0.14	1.17	1.48	1.81	2.52E-03	3.19E-03	3.89E-03
Spain - Escherichia coli ColREC UTI - model 28	0.28	0.39	0.55	0.00	0.00	0.00	0.28	0.39	0.55	1.53E-03	2.10E-03	2.76E-03	5.92E-04	8.43E-04	1.19E-03	0.00	0.00	0.00	5.92E-04	8.43E-04	1.19E-03	156.47	188.96	221.35	0.34	0.41	0.48	0.00	0.00	0.00	0.00	0.00	0.00
Spain - Klebsiella pneumoniae 3GCRKP BSI - model 1	551.27	600.45	652.67	3,532.69	3,776.05	4,060.83	4,047.27	4,375.52	4,700.07	4.61	4.85	5.10	1.19	1.29	1.41	7.61	8.13	8.74	8.71	9.42	10.12	848.11	904.21	965.04	1.83	1.95	2.08	140.07	151.14	162.77	0.30	0.33	0.35
Spain - Klebsiella pneumoniae 3GCRKP OTH - model 5	0.95	1.21	1.54	0.00	0.00	0.00	0.95	1.21	1.54	2.70E-03	3.34E-03	4.02E-03	2.06E-03	2.61E-03	3.32E-03	0.00	0.00	0.00	2.06E-03	2.61E-03	3.32E-03	314.92	363.72	417.72	0.68	0.78	0.90	0.00	0.00	0.00	0.00	0.00	0.00
Spain - Klebsiella pneumoniae 3GCRKP RESP - model 2	322.52	365.96	410.08	628.32	712.22	793.79	953.94	1,076.94	1,199.75	1.26	1.36	1.47	0.69	0.79	0.88	1.35	1.53	1.71	2.05	2.32	2.58	697.68	788.95	887.12	1.50	1.70	1.91	25.01	28.47	32.21	0.05	0.06	0.07
Spain - Klebsiella pneumoniae 3GCRKP SSI - model 4	0.58	0.76	1.01	133.16	155.91	181.48	134.25	156.70	182.32	0.41	0.44	0.47	1.24E-03	1.65E-03	2.18E-03	0.29	0.34	0.39	0.29	0.34	0.39	311.25	359.11	408.37	0.67	0.77	0.88	7.62	9.10	10.77	0.02	0.02	0.02
Spain - Klebsiella pneumoniae 3GCRKP UTI - model 3	1.68	2.09	2.60	0.00	0.00	0.00	1.68	2.09	2.60	1.61E-03	1.95E-03	2.36E-03	3.61E-03	4.50E-03	5.61E-03	0.00	0.00	0.00	3.61E-03	4.50E-03	5.61E-03	942.67	1,072.69	1,195.42	2.03	2.31	2.57	0.00	0.00	0.00	0.00	0.00	0.00
Spain - Klebsiella pneumoniae CRKP BSI - model 6	72.83	85.18	99.56	922.25	1,123.52	1,369.10	1,001.58	1,208.72	1,464.53	9.87	11.67	13.67	0.16	0.18	0.21	1.99	2.42	2.95	2.16	2.60	3.15	92.87	103.94	115.93	0.20	0.22	0.25	30.82	37.04	43.82	0.07	0.08	0.09
Spain - Klebsiella pneumoniae CRKP OTH - model 10	0.12	0.17	0.24	0.00	0.00	0.00	0.12	0.17	0.24	2.71E-03	3.50E-03	4.52E-03	2.62E-04	3.71E-04	5.21E-04	0.00	0.00	0.00	2.62E-04	3.71E-04	5.21E-04	37.73	49.32	61.48	0.08	0.11	0.13	0.00	0.00	0.00	0.00	0.00	0.00
Spain - Klebsiella pneumoniae CRKP RESP - model 7	18.94	25.00	31.61	37.36	49.00	61.14	56.92	74.09	92.35	1.40	1.66	1.94	0.04	0.05	0.07	0.08	0.11	0.13	0.12	0.16	0.20	34.52	44.60	54.92	0.07	0.10	0.12	1.23	1.61	1.98	2.65E-03	3.46E-03	4.26E-03
Spain - Klebsiella pneumoniae CRKP SSI - model 9	0.04	0.07	0.10	10.23	12.97	16.01	10.29	13.04	16.24	0.38	0.42	0.47	9.41E-05	1.42E-04	2.09E-04	0.02	0.03	0.03	0.02	0.03	0.03	24.43	31.12	38.56	0.05	0.07	0.08	0.49	0.67	0.87	1.06E-03	1.44E-03	1.88E-03
Spain - Klebsiella pneumoniae CRKP UTI - model 8	0.09	0.13	0.18	0.00	0.00	0.00	0.09	0.13	0.18	1.61E-03	2.07E-03	2.65E-03	2.01E-04	2.84E-04	3.87E-04	0.00	0.00	0.00	2.01E-04	2.84E-04	3.87E-04	51.06	63.53	76.56	0.11	0.14	0.16	0.00	0.00	0.00	0.00	0.00	0.00
Spain - Klebsiella pneumoniae ColRKP BSI - model 11	8.28	10.82	14.15	166.43	242.58	347.61	175.74	253.74	358.13	13.38	18.52	23.63	0.02	0.02	0.03	0.36	0.52	0.75	0.38	0.55	0.77	11.28	13.73	16.76	0.02	0.03	0.04	5.85	8.28	11.54	0.01	0.02	0.02
Spain - Klebsiella pneumoniae ColRKP OTH - model 15	0.01	0.02	0.04	0.00	0.00	0.00	0.01	0.02	0.04	2.00E-03	3.40E-03	5.30E-03	2.50E-05	4.76E-05	8.71E-05	0.00	0.00	0.00	2.50E-05	4.76E-05	8.71E-05	4.12	6.53	9.14	8.87E-03	0.01	0.02	0.00	0.00	0.00	0.00	0.00	0.00
Spain - Klebsiella pneumoniae ColRKP RESP - model 12	1.82	3.23	4.59	3.62	6.31	9.02	5.40	9.54	13.69	1.30	1.59	1.91	3.92E-03	6.95E-03	9.88E-03	7.79E-03	0.01	0.02	0.01	0.02	0.03	3.57	5.90	8.35	7.70E-03	0.01	0.02	0.13	0.21	0.30	2.73E-04	4.57E-04	6.45E-04
Spain - Klebsiella pneumoniae ColRKP SSI - model 14	3.98E-03	8.61E-03	0.02	1.16	2.15	3.14	1.17	2.16	3.15	0.44	0.52	0.60	8.57E-06	1.85E-05	3.47E-05	2.51E-03	4.63E-03	6.75E-03	2.52E-03	4.65E-03	6.79E-03	2.41	4.16	5.92	5.19E-03	8.96E-03	0.01	0.05	0.09	0.14	1.06E-04	2.02E-04	2.99E-04
Spain - Klebsiella pneumoniae ColRKP UTI - model 13	9.42E-03	0.02	0.03	0.00	0.00	0.00	9.42E-03	0.02	0.03	1.27E-03	2.08E-03	3.08E-03	2.03E-05	3.74E-05	6.21E-05	0.00	0.00	0.00	2.03E-05	3.74E-05	6.21E-05	5.55	8.56	11.63	0.01	0.02	0.03	0.00	0.00	0.00	0.00	0.00	0.00
Spain - Pseudomonas aeruginosa CRPA BSI - model 51	440.70	493.49	550.39	3,453.34	4,218.12	5,076.32	3,928.47	4,705.13	5,608.37	5.73	6.81	7.94	0.95	1.06	1.18	7.43	9.08	10.93	8.46	10.13	12.07	627.79	691.41	765.02	1.35	1.49	1.65	127.23	158.12	192.76	0.27	0.34	0.41
Spain - Pseudomonas aeruginosa CRPA OTH - model 55	0.99	1.29	1.64	0.00	0.00	0.00	0.99	1.29	1.64	3.02E-03	3.67E-03	4.30E-03	2.14E-03	2.78E-03	3.52E-03	0.00	0.00	0.00	2.14E-03	2.78E-03	3.52E-03	290.01	352.33	420.64	0.62	0.76	0.91	0.00	0.00	0.00	0.00	0.00	0.00
Spain - Pseudomonas aeruginosa CRPA RESP - model 52	1,122.30	1,276.91	1,439.81	3,720.03	4,526.13	5,461.71	4,893.01	5,802.74	6,815.36	1.98	2.25	2.58	2.42	2.75	3.10	8.01	9.74	11.76	10.53	12.49	14.67	2,244.41	2,576.26	2,914.98	4.83	5.55	6.28	136.03	169.91	207.70	0.29	0.37	0.45
Spain - Pseudomonas aeruginosa CRPA SSI - model 54	0.76	1.02	1.37	168.14	214.54	257.86	168.93	215.54	259.17	0.40	0.45	0.48	1.63E-03	2.20E-03	2.96E-03	0.36	0.46	0.56	0.36	0.46	0.56	393.94	483.38	566.32	0.85	1.04	1.22	8.55	11.15	13.66	0.02	0.02	0.03
Spain - Pseudomonas aeruginosa CRPA UTI - model 53	1.45	1.82	2.34	0.00	0.00	0.00	1.45	1.82	2.34	1.70E-03	2.03E-03	2.44E-03	3.12E-03	3.92E-03	5.04E-03	0.00	0.00	0.00	3.12E-03	3.92E-03	5.04E-03	760.64	900.46	1,046.92	1.64	1.94	2.25	0.00	0.00	0.00	0.00	0.00	0.00
Spain - Pseudomonas aeruginosa ColRPA BSI - model 56	13.17	16.87	21.68	87.23	143.32	211.16	102.15	160.53	232.60	6.09	9.03	12.29	0.03	0.04	0.05	0.19	0.31	0.45	0.22	0.35	0.50	14.48	17.67	21.33	0.03	0.04	0.05	2.51	3.96	5.78	5.40E-03	8.53E-03	0.01
Spain - Pseudomonas aeruginosa ColRPA OTH - model 60	0.02	0.03	0.05	0.00	0.00	0.00	0.02	0.03	0.05	2.51E-03	3.74E-03	5.03E-03	4.15E-05	7.32E-05	1.13E-04	0.00	0.00	0.00	4.15E-05	7.32E-05	1.13E-04	6.16	9.19	12.23	0.01	0.02	0.03	0.00	0.00	0.00	0.00	0.00	0.00
Spain - Pseudomonas aeruginosa ColRPA RESP - model 57	32.05	43.47	55.99	91.98	154.04	225.13	129.88	198.02	276.65	2.21	2.99	3.86	0.07	0.09	0.12	0.20	0.33	0.48	0.28	0.43	0.60	49.57	66.18	82.30	0.11	0.14	0.18	2.60	4.30	6.24	5.59E-03	9.27E-03	0.01
Spain - Pseudomonas aeruginosa ColRPA SSI - model 59	0.01	0.03	0.04	3.38	5.08	6.81	3.39	5.10	6.84	0.37	0.41	0.46	2.96E-05	5.54E-05	9.42E-05	7.28E-03	0.01	0.01	7.29E-03	0.01	0.01	8.50	12.36	16.43	0.02	0.03	0.04	0.11	0.18	0.25	2.34E-04	3.82E-04	5.31E-04
Spain - Pseudomonas aeruginosa ColRPA UTI - model 58	0.03	0.05	0.07	0.00	0.00	0.00	0.03	0.05	0.07	1.39E-03	2.06E-03	2.79E-03	6.10E-05	1.01E-04	1.54E-04	0.00	0.00	0.00	6.10E-05	1.01E-04	1.54E-04	16.63	23.08	29.85	0.04	0.05	0.06	0.00	0.00	0.00	0.00	0.00	0.00
Spain - Pseudomonas aeruginosa MDRPA BSI - model 46	17.81	21.32	25.45	115.14	165.88	220.47	136.05	187.23	244.05	3.62	4.83	6.22	0.04	0.05	0.05	0.25	0.36	0.47	0.29	0.40	0.53	33.45	38.82	44.68	0.07	0.08	0.10	5.84	8.29	10.79	0.01	0.02	0.02
Spain - Pseudomonas aeruginosa MDRPA OTH - model 50	0.05	0.07	0.11	0.00	0.00	0.00	0.05	0.07	0.11	2.68E-03	3.73E-03	4.73E-03	1.03E-04	1.60E-04	2.27E-04	0.00	0.00	0.00	1.03E-04	1.60E-04	2.27E-04	14.48	20.10	25.60	0.03	0.04	0.06	0.00	0.00	0.00	0.00	0.00	0.00
Spain - Pseudomonas aeruginosa MDRPA RESP - model 47	42.83	54.36	66.78	132.58	181.58	244.35	179.29	237.11	308.55	1.30	1.63	2.05	0.09	0.12	0.14	0.29	0.39	0.53	0.39	0.51	0.66	117.50	144.26	173.11	0.25	0.31	0.37	6.36	9.02	11.94	0.01	0.02	0.03
Spain - Pseudomonas aeruginosa MDRPA SSI - model 49	0.04	0.06	0.09	7.91	10.53	13.40	7.97	10.59	13.47	0.34	0.39	0.44	7.78E-05	1.23E-04	1.88E-04	0.02	0.02	0.03	0.02	0.02	0.03	20.03	26.99	34.15	0.04	0.06	0.07	0.49	0.71	0.93	1.05E-03	1.53E-03	2.01E-03
Spain - Pseudomonas aeruginosa MDRPA UTI - model 48	0.07	0.10	0.15	0.00	0.00	0.00	0.07	0.10	0.15	1.53E-03	2.03E-03	2.62E-03	1.51E-04	2.21E-04	3.13E-04	0.00	0.00	0.00	1.51E-04	2.21E-04	3.13E-04	38.17	50.67	63.15	0.08	0.11	0.14	0.00	0.00	0.00	0.00	0.00	0.00
Spain - Staphylococcus aureus MRSA BSI - model 61	761.03	832.78	912.99	5,184.36	5,571.43	5,953.14	5,975.92	6,406.69	6,823.75	3.65	3.85	4.05	1.64	1.79	1.97	11.16	11.99	12.82	12.87	13.79	14.69	1,552.57	1,664.13	1,789.30	3.34	3.58	3.85	274.63	298.69	324.48	0.59	0.64	0.70
Spain - Staphylococcus aureus MRSA OTH - model 65	6.28	8.01	9.93	0.00	0.00	0.00	6.28	8.01	9.93	2.64E-03	3.25E-03	3.95E-03	0.01	0.02	0.02	0.00	0.00	0.00	0.01	0.02	0.02	2,201.11	2,474.69	2,747.36	4.74	5.33	5.91	0.00	0.00	0.00	0.00	0.00	0.00
Spain - Staphylococcus aureus MRSA RESP - model 62	783.26	873.81	967.55	1,518.81	1,696.81	1,870.89	2,306.86	2,568.97	2,828.28	0.94	1.02	1.09	1.69	1.88	2.08	3.27	3.65	4.03	4.97	5.53	6.09	2,236.29	2,528.80	2,843.26	4.81	5.44	6.12	79.30	90.84	102.73	0.17	0.20	0.22
Spain - Staphylococcus aureus MRSA SSI - model 64	4.28	5.86	7.64	971.12	1,099.92	1,207.62	977.71	1,105.23	1,210.85	0.38	0.40	0.42	9.21E-03	0.01	0.02	2.09	2.37	2.60	2.10	2.38	2.61	2,453.75	2,753.38	3,033.92	5.28	5.93	6.53	69.66	79.95	89.69	0.15	0.17	0.19
Spain - Staphylococcus aureus MRSA UTI - model 63	0.56	0.77	1.02	0.00	0.00	0.00	0.56	0.77	1.02	1.47E-03	1.86E-03	2.24E-03	1.22E-03	1.67E-03	2.19E-03	0.00	0.00	0.00	1.22E-03	1.67E-03	2.19E-03	334.15	415.53	495.00	0.72	0.89	1.07	0.00	0.00	0.00	0.00	0.00	0.00
Spain - Streptococcus pneumoniae PMRSP BSI - model 76	190.38	212.46	237.61	1,358.55	1,442.88	1,531.27	1,569.76	1,656.73	1,749.74	6.66	7.02	7.37	0.41	0.46	0.51	2.92	3.11	3.30	3.38	3.57	3.77	230.91	236.08	240.72	0.50	0.51	0.52	40.67	42.48	44.46	0.09	0.09	0.10
Spain - Streptococcus pneumoniae PMRSP OTH - model 80	0.13	0.17	0.20	0.00	0.00	0.00	0.13	0.17	0.20	1.57E-03	1.97E-03	2.40E-03	2.84E-04	3.61E-04	4.41E-04	0.00	0.00	0.00	2.84E-04	3.61E-04	4.41E-04	78.74	85.20	94.46	0.17	0.18	0.20	0.00	0.00	0.00	0.00	0.00	0.00
Spain - Streptococcus pneumoniae PMRSP RESP - model 77	477.36	573.41	706.21	934.00	1,118.94	1,384.20	1,414.51	1,694.55	2,083.27	1.60	1.84	2.12	1.03	1.23	1.52	2.01	2.41	2.98	3.05	3.65	4.49	798.26	918.58	1,059.04	1.72	1.98	2.28	28.67	33.10	38.47	0.06	0.07	0.08
Spain - Streptococcus pneumoniae PMRSP SSI - model 79	0.00	0.00	0.00	0.00	0.00	0.00	0.00	0.00	0.00	NaN	NaN	NaN	0.00	0.00	0.00	0.00	0.00	0.00	0.00	0.00	0.00	0.00	0.00	0.00	0.00	0.00	0.00	0.00	0.00	0.00	0.00	0.00	0.00
Spain - Streptococcus pneumoniae PMRSP UTI - model 78	0.00	0.00	0.00	0.00	0.00	0.00	0.00	0.00	0.00	NaN	NaN	NaN	0.00	0.00	0.00	0.00	0.00	0.00	0.00	0.00	0.00	0.00	0.00	0.00	0.00	0.00	0.00	0.00	0.00	0.00	0.00	0.00	0.00
Spain - Streptococcus pneumoniae PRSP BSI - model 71	220.74	240.27	261.00	1,564.33	1,629.37	1,704.15	1,798.81	1,869.78	1,947.31	6.73	6.97	7.23	0.48	0.52	0.56	3.37	3.51	3.67	3.87	4.03	4.19	263.82	268.16	272.83	0.57	0.58	0.59	46.52	48.32	50.05	0.10	0.10	0.11
Spain - Streptococcus pneumoniae PRSP OTH - model 75	0.16	0.19	0.23	0.00	0.00	0.00	0.16	0.19	0.23	1.65E-03	1.95E-03	2.30E-03	3.34E-04	4.06E-04	4.88E-04	0.00	0.00	0.00	3.34E-04	4.06E-04	4.88E-04	90.37	96.92	103.12	0.19	0.21	0.22	0.00	0.00	0.00	0.00	0.00	0.00
Spain - Streptococcus pneumoniae PRSP RESP - model 72	570.17	655.91	760.97	1,117.13	1,277.10	1,485.36	1,696.37	1,935.96	2,235.43	1.69	1.84	2.03	1.23	1.41	1.64	2.41	2.75	3.20	3.65	4.17	4.81	940.74	1,051.53	1,189.08	2.03	2.26	2.56	33.84	37.94	43.11	0.07	0.08	0.09
Spain - Streptococcus pneumoniae PRSP SSI - model 74	0.00	0.00	0.00	0.00	0.00	0.00	0.00	0.00	0.00	NaN	NaN	NaN	0.00	0.00	0.00	0.00	0.00	0.00	0.00	0.00	0.00	0.00	0.00	0.00	0.00	0.00	0.00	0.00	0.00	0.00	0.00	0.00	0.00
Spain - Streptococcus pneumoniae PRSP UTI - model 73	0.00	0.00	0.00	0.00	0.00	0.00	0.00	0.00	0.00	NaN	NaN	NaN	0.00	0.00	0.00	0.00	0.00	0.00	0.00	0.00	0.00	0.00	0.00	0.00	0.00	0.00	0.00	0.00	0.00	0.00	0.00	0.00	0.00
**Total (Median & 95% UIs)**	7,247.98	8,216.72	9,308.80	35,433.20	40,610.56	46,466.54	42,874.93	48,823.81	55,421.71		-		15.60	17.69	20.04	76.28	87.43	100.04	92.30	105.11	119.32	35,957.22	41,345.01	46,837.28	77.41	89.01	100.83	1,640.58	1,899.25	2,187.24	3.53	4.09	4.71
**Total (average)**		**8,236.05**			**40,669.84**			**48,905.89**			-			**17.73**			**87.56**			**105.29**			**41,350.08**			**89.02**			**1,902.48**			**4.10**	

Infection	YLD			YLL			DALY			DALY per Case			YLD per 100,000			YLL per 100,000			DALY per 100,000			Cases per year			Incidence per 100,000			Deaths per year			Mortality per 100,000		
Colistin resistant *Acinetobacter spp.*	0.00	0.00	0.00	0.00	0.00	0.00	0.00	0.00	0.00	0.00	0.00	0.00	0.00	0.00	0.00	0.00	0.00	0.00	0.00	0.00	0.00	0.00	0.00	0.00	0.00	0.00	0.00	0.00	0.00	0.00	0.00	0.00	0.00
Carbapenem resistant *Acinetobacter spp.* (excluding those resistant to colistin)	1.50	2.42	3.62	5.09	12.61	22.70	6.99	15.05	25.73	7.80	14.15	21.12	0.03	0.04	0.07	0.09	0.23	0.41	0.13	0.28	0.47	1.85	3.13	4.50	0.03	0.06	0.08	0.10	0.26	0.46	0.00	0.00	0.01
Aminoglycosides and fluoroquinolones resistant *Acinetobacter* spp. (excluding those resistant to colistin and/or carbapenem)	0.00	0.00	0.00	0.00	0.00	0.00	0.00	0.00	0.00	0.00	0.00	0.00	0.00	0.00	0.00	0.00	0.00	0.00	0.00	0.00	0.00	0.00	0.00	0.00	0.00	0.00	0.00	0.00	0.00	0.00	0.00	0.00	0.00
Vancomycin resistant Enterococci (*E. faecalis* and *E. faecium*)	0.29	0.57	1.15	2.92	5.90	10.95	3.27	6.47	11.96	4.71	4.89	5.09	0.01	0.01	0.02	0.05	0.11	0.20	0.06	0.12	0.22	1.78	5.13	9.25	0.03	0.09	0.17	0.17	0.35	0.65	0.00	0.01	0.01
Colistin resistant *Escherichia coli*	0.00	0.00	0.00	0.00	0.00	0.00	0.00	0.00	0.00	0.00	0.00	0.00	0.00	0.00	0.00	0.00	0.00	0.00	0.00	0.00	0.00	0.00	0.00	0.00	0.00	0.00	0.00	0.00	0.00	0.00	0.00	0.00	0.00
Carbapenem resistant *Escherichia coli* (excluding those resistant to colistin)	0.00	0.00	0.00	0.00	0.00	0.00	0.00	0.00	0.00	0.00	0.00	0.00	0.00	0.00	0.00	0.00	0.00	0.00	0.00	0.00	0.00	0.00	0.00	0.00	0.00	0.00	0.00	0.00	0.00	0.00	0.00	0.00	0.00
Third-generation cephalosporin resistant *Escherichia coli* (excluding those resistant to colistin and/or carbapenem)	175.65	195.92	217.62	1,044.08	1,163.91	1,291.19	1,229.61	1,360.46	1,502.08	4.95	5.38	5.85	3.21	3.58	3.98	19.08	21.27	23.60	22.47	24.86	27.45	1,799.00	2,008.25	2,232.05	32.88	36.70	40.79	54.55	61.72	69.66	1.00	1.13	1.27
Colistin resistant *Klebsiella pneumoniae*	0.00	0.00	0.00	0.00	0.00	0.00	0.00	0.00	0.00	0.00	0.00	0.00	0.00	0.00	0.00	0.00	0.00	0.00	0.00	0.00	0.00	0.00	0.00	0.00	0.00	0.00	0.00	0.00	0.00	0.00	0.00	0.00	0.00
Carbapenem resistant *Klebsiella pneumoniae* (excluding those resistant to colistin)	0.00	0.00	0.00	0.00	0.00	0.00	0.00	0.00	0.00	0.00	0.00	0.00	0.00	0.00	0.00	0.00	0.00	0.00	0.00	0.00	0.00	0.00	0.00	0.00	0.00	0.00	0.00	0.00	0.00	0.00	0.00	0.00	0.00
Third-generation cephalosporin resistant *Klebsiella pneumoniae* (excluding those resistant to colistin and/or carbapenem)	18.50	20.97	24.05	92.19	101.66	111.35	111.37	122.59	134.21	6.29	6.70	7.15	0.34	0.38	0.44	1.68	1.86	2.04	2.04	2.24	2.45	66.96	75.72	84.51	1.22	1.38	1.54	3.76	4.14	4.53	0.07	0.08	0.08
Colistin resistant *Pseudomonas aeruginosa*	0.00	0.00	0.00	0.00	0.00	0.00	0.00	0.00	0.00	0.00	0.00	0.00	0.00	0.00	0.00	0.00	0.00	0.00	0.00	0.00	0.00	0.00	0.00	0.00	0.00	0.00	0.00	0.00	0.00	0.00	0.00	0.00	0.00
Carbapenem resistant *Pseudomonas aeruginosa* (excluding those resistant to colistin)	44.42	53.91	64.44	196.45	273.08	359.29	245.41	327.42	421.27	8.94	11.55	14.29	0.81	0.99	1.18	3.59	4.99	6.57	4.48	5.98	7.70	104.23	124.84	145.00	1.90	2.28	2.65	6.32	8.42	10.89	0.12	0.15	0.20
Pseudomonas aeruginosa resistant to three or more antimicrobial groups (excluding those resistant to colistin and/or carbapenem)	12.64	15.82	19.52	50.30	74.44	102.72	64.33	90.06	121.26	4.76	6.39	8.17	0.23	0.29	0.36	0.92	1.36	1.88	1.18	1.65	2.22	49.37	62.30	75.36	0.90	1.14	1.38	2.76	3.94	5.35	0.05	0.07	0.10
Methicillin resistant *Staphylococcus aureus*	59.07	66.86	75.46	285.86	312.22	340.65	347.33	378.93	413.22	7.73	8.23	8.78	1.08	1.22	1.38	5.22	5.71	6.23	6.35	6.93	7.55	214.76	239.50	263.46	3.92	4.38	4.81	9.87	10.84	11.87	0.18	0.20	0.22
Penicillin and macrolides resistant *Streptococcus pneumoniae* (excluding those only resistant to penicillin)	0.86	1.26	2.01	3.25	4.11	5.52	4.25	5.38	7.29	2.94	3.35	3.76	0.02	0.02	0.04	0.06	0.08	0.10	0.08	0.10	0.13	4.10	5.19	7.55	0.07	0.09	0.14	0.25	0.32	0.43	0.00	0.01	0.01
Penicillin tesistant *Streptococcus pneumoniae* (excluding those resistant to macrolides)	0.00	0.00	0.00	0.00	0.00	0.00	0.00	0.00	0.00	0.00	0.00	0.00	0.00	0.00	0.00	0.00	0.00	0.00	0.00	0.00	0.00	0.00	0.00	0.00	0.00	0.00	0.00	0.00	0.00	0.00	0.00	0.00	0.00
Total	312.93	357.73	407.87	1,680.14	1,947.92	2,244.37	2,012.55	2,306.37	2,637.03				5.72	6.54	7.45	30.71	35.60	41.02	36.78	42.15	48.19	2,242.06	2,524.07	2,821.68	40.98	46.13	51.57	77.79	89.98	103.85	1.42	1.64	1.90

Model	YLD			YLL			DALY			DALY per Case			YLD per 100,000			YLL per 100,000			DALY per 100,000			Cases per year			Incidence per 100,000			Deaths per year			Mortality per 100,000		
Finland - Acinetobacter spp. ColRACI BSI - model 41	0.00	0.00	0.00	0.00	0.00	0.00	0.00	0.00	0.00	NaN	NaN	NaN	0.00	0.00	0.00	0.00	0.00	0.00	0.00	0.00	0.00	0.00	0.00	0.00	0.00	0.00	0.00	0.00	0.00	0.00	0.00	0.00	0.00
Finland - Acinetobacter spp. ColRACI OTH - model 45	0.00	0.00	0.00	0.00	0.00	0.00	0.00	0.00	0.00	NaN	NaN	NaN	0.00	0.00	0.00	0.00	0.00	0.00	0.00	0.00	0.00	0.00	0.00	0.00	0.00	0.00	0.00	0.00	0.00	0.00	0.00	0.00	0.00
Finland - Acinetobacter spp. ColRACI RESP - model 42	0.00	0.00	0.00	0.00	0.00	0.00	0.00	0.00	0.00	NaN	NaN	NaN	0.00	0.00	0.00	0.00	0.00	0.00	0.00	0.00	0.00	0.00	0.00	0.00	0.00	0.00	0.00	0.00	0.00	0.00	0.00	0.00	0.00
Finland - Acinetobacter spp. ColRACI SSI - model 44	0.00	0.00	0.00	0.00	0.00	0.00	0.00	0.00	0.00	NaN	NaN	NaN	0.00	0.00	0.00	0.00	0.00	0.00	0.00	0.00	0.00	0.00	0.00	0.00	0.00	0.00	0.00	0.00	0.00	0.00	0.00	0.00	0.00
Finland - Acinetobacter spp. ColRACI UTI - model 43	0.00	0.00	0.00	0.00	0.00	0.00	0.00	0.00	0.00	NaN	NaN	NaN	0.00	0.00	0.00	0.00	0.00	0.00	0.00	0.00	0.00	0.00	0.00	0.00	0.00	0.00	0.00	0.00	0.00	0.00	0.00	0.00	0.00
Finland - Acinetobacter spp. CRACI BSI - model 36	0.93	1.39	1.97	3.82	10.36	19.30	5.13	11.74	20.87	5.06	11.03	17.62	0.02	0.03	0.04	0.07	0.19	0.35	0.09	0.21	0.38	0.85	1.07	1.37	0.02	0.02	0.03	0.08	0.21	0.39	1.43E-03	3.86E-03	7.15E-03
Finland - Acinetobacter spp. CRACI OTH - model 40	1.75E-05	1.27E-04	3.78E-04	0.00	0.00	0.00	1.75E-05	1.27E-04	3.78E-04	1.56E-03	3.67E-03	6.41E-03	3.20E-07	2.32E-06	6.91E-06	0.00	0.00	0.00	3.20E-07	2.32E-06	6.91E-06	5.90E-03	0.04	0.08	1.08E-04	6.95E-04	1.45E-03	0.00	0.00	0.00	0.00	0.00	0.00
Finland - Acinetobacter spp. CRACI RESP - model 37	0.58	1.03	1.64	1.20	2.07	3.12	1.79	3.13	4.57	2.30	2.67	3.04	0.01	0.02	0.03	0.02	0.04	0.06	0.03	0.06	0.08	0.68	1.17	1.70	0.01	0.02	0.03	0.02	0.04	0.06	4.48E-04	7.70E-04	1.16E-03
Finland - Acinetobacter spp. CRACI SSI - model 39	1.38E-04	7.22E-04	2.10E-03	0.07	0.18	0.28	0.07	0.18	0.28	0.44	0.44	0.45	2.52E-06	1.32E-05	3.85E-05	1.24E-03	3.23E-03	5.10E-03	1.24E-03	3.25E-03	5.14E-03	0.15	0.40	0.63	2.80E-03	7.32E-03	0.01	1.38E-03	3.61E-03	5.68E-03	2.52E-05	6.59E-05	1.04E-04
Finland - Acinetobacter spp. CRACI UTI - model 38	2.53E-04	8.48E-04	2.06E-03	0.00	0.00	0.00	2.53E-04	8.48E-04	2.06E-03	8.67E-04	2.03E-03	3.71E-03	4.62E-06	1.55E-05	3.76E-05	0.00	0.00	0.00	4.62E-06	1.55E-05	3.76E-05	0.17	0.45	0.73	3.14E-03	8.22E-03	0.01	0.00	0.00	0.00	0.00	0.00	0.00
Finland - Acinetobacter spp. MDRACI BSI - model 31	0.00	0.00	0.00	0.00	0.00	0.00	0.00	0.00	0.00	NaN	NaN	NaN	0.00	0.00	0.00	0.00	0.00	0.00	0.00	0.00	0.00	0.00	0.00	0.00	0.00	0.00	0.00	0.00	0.00	0.00	0.00	0.00	0.00
Finland - Acinetobacter spp. MDRACI OTH - model 35	0.00	0.00	0.00	0.00	0.00	0.00	0.00	0.00	0.00	NaN	NaN	NaN	0.00	0.00	0.00	0.00	0.00	0.00	0.00	0.00	0.00	0.00	0.00	0.00	0.00	0.00	0.00	0.00	0.00	0.00	0.00	0.00	0.00
Finland - Acinetobacter spp. MDRACI RESP - model 32	0.00	0.00	0.00	0.00	0.00	0.00	0.00	0.00	0.00	NaN	NaN	NaN	0.00	0.00	0.00	0.00	0.00	0.00	0.00	0.00	0.00	0.00	0.00	0.00	0.00	0.00	0.00	0.00	0.00	0.00	0.00	0.00	0.00
Finland - Acinetobacter spp. MDRACI SSI - model 34	0.00	0.00	0.00	0.00	0.00	0.00	0.00	0.00	0.00	NaN	NaN	NaN	0.00	0.00	0.00	0.00	0.00	0.00	0.00	0.00	0.00	0.00	0.00	0.00	0.00	0.00	0.00	0.00	0.00	0.00	0.00	0.00	0.00
Finland - Acinetobacter spp. MDRACI UTI - model 33	0.00	0.00	0.00	0.00	0.00	0.00	0.00	0.00	0.00	NaN	NaN	NaN	0.00	0.00	0.00	0.00	0.00	0.00	0.00	0.00	0.00	0.00	0.00	0.00	0.00	0.00	0.00	0.00	0.00	0.00	0.00	0.00	0.00
Finland - Enterococcus faecalis and E. faecium VRE BSI - model 66	0.29	0.56	1.13	2.61	4.83	9.03	2.96	5.39	10.02	4.11	4.28	4.48	5.29E-03	0.01	0.02	0.05	0.09	0.16	0.05	0.10	0.18	0.68	1.26	2.39	0.01	0.02	0.04	0.16	0.29	0.54	2.85E-03	5.27E-03	9.81E-03
Finland - Enterococcus faecalis and E. faecium VRE OTH - model 70	4.79E-04	2.05E-03	5.44E-03	0.00	0.00	0.00	4.79E-04	2.05E-03	5.44E-03	5.95E-04	1.55E-03	3.12E-03	8.75E-06	3.74E-05	9.95E-05	0.00	0.00	0.00	8.75E-06	3.74E-05	9.95E-05	0.41	1.43	2.43	7.50E-03	0.03	0.04	0.00	0.00	0.00	0.00	0.00	0.00
Finland - Enterococcus faecalis and E. faecium VRE RESP - model 67	0.00	0.00	0.00	0.00	0.00	0.00	0.00	0.00	0.00	NaN	NaN	NaN	0.00	0.00	0.00	0.00	0.00	0.00	0.00	0.00	0.00	0.00	0.00	0.00	0.00	0.00	0.00	0.00	0.00	0.00	0.00	0.00	0.00
Finland - Enterococcus faecalis and E. faecium VRE SSI - model 69	6.22E-04	3.24E-03	0.01	0.31	1.07	1.92	0.31	1.08	1.93	0.60	0.61	0.61	1.14E-05	5.93E-05	1.97E-04	5.63E-03	0.02	0.04	5.62E-03	0.02	0.04	0.51	1.78	3.19	9.33E-03	0.03	0.06	0.02	0.06	0.11	3.36E-04	1.17E-03	2.10E-03
Finland - Enterococcus faecalis and E. faecium VRE UTI - model 68	2.28E-04	1.14E-03	3.00E-03	0.00	0.00	0.00	2.28E-04	1.14E-03	3.00E-03	7.21E-04	1.83E-03	3.33E-03	4.17E-06	2.08E-05	5.48E-05	0.00	0.00	0.00	4.17E-06	2.08E-05	5.48E-05	0.17	0.65	1.24	3.13E-03	0.01	0.02	0.00	0.00	0.00	0.00	0.00	0.00
Finland - Escherichia coli 3GCREC BSI - model 16	134.39	147.48	161.22	864.63	954.75	1,052.46	1,009.37	1,102.61	1,208.01	3.59	3.90	4.22	2.46	2.70	2.95	15.80	17.45	19.23	18.45	20.15	22.08	263.78	282.48	302.12	4.82	5.16	5.52	43.69	48.81	54.58	0.80	0.89	1.00
Finland - Escherichia coli 3GCREC OTH - model 20	0.23	0.31	0.41	0.00	0.00	0.00	0.23	0.31	0.41	2.70E-03	3.33E-03	4.05E-03	4.12E-03	5.65E-03	7.48E-03	0.00	0.00	0.00	4.12E-03	5.65E-03	7.48E-03	74.85	93.22	111.17	1.37	1.70	2.03	0.00	0.00	0.00	0.00	0.00	0.00
Finland - Escherichia coli 3GCREC RESP - model 17	38.67	45.13	52.24	74.32	87.54	99.90	112.79	132.87	151.15	0.97	1.07	1.19	0.71	0.82	0.95	1.36	1.60	1.83	2.06	2.43	2.76	104.08	124.49	144.34	1.90	2.28	2.64	3.73	4.47	5.23	0.07	0.08	0.10
Finland - Escherichia coli 3GCREC SSI - model 19	0.47	0.64	0.83	105.14	121.62	138.83	105.33	122.33	139.58	0.38	0.41	0.43	8.61E-03	0.01	0.02	1.92	2.22	2.54	1.93	2.24	2.55	260.91	299.73	342.53	4.77	5.48	6.26	7.13	8.43	9.85	0.13	0.15	0.18
Finland - Escherichia coli 3GCREC UTI - model 18	1.89	2.35	2.93	0.00	0.00	0.00	1.89	2.35	2.93	1.61E-03	1.96E-03	2.31E-03	0.03	0.04	0.05	0.00	0.00	0.00	0.03	0.04	0.05	1,095.37	1,208.33	1,331.90	20.02	22.08	24.34	0.00	0.00	0.00	0.00	0.00	0.00
Finland - Escherichia coli ColREC BSI - model 26	0.00	0.00	0.00	0.00	0.00	0.00	0.00	0.00	0.00	NaN	NaN	NaN	0.00	0.00	0.00	0.00	0.00	0.00	0.00	0.00	0.00	0.00	0.00	0.00	0.00	0.00	0.00	0.00	0.00	0.00	0.00	0.00	0.00
Finland - Escherichia coli ColREC OTH - model 30	0.00	0.00	0.00	0.00	0.00	0.00	0.00	0.00	0.00	NaN	NaN	NaN	0.00	0.00	0.00	0.00	0.00	0.00	0.00	0.00	0.00	0.00	0.00	0.00	0.00	0.00	0.00	0.00	0.00	0.00	0.00	0.00	0.00
Finland - Escherichia coli ColREC RESP - model 27	0.00	0.00	0.00	0.00	0.00	0.00	0.00	0.00	0.00	NaN	NaN	NaN	0.00	0.00	0.00	0.00	0.00	0.00	0.00	0.00	0.00	0.00	0.00	0.00	0.00	0.00	0.00	0.00	0.00	0.00	0.00	0.00	0.00
Finland - Escherichia coli ColREC SSI - model 29	0.00	0.00	0.00	0.00	0.00	0.00	0.00	0.00	0.00	NaN	NaN	NaN	0.00	0.00	0.00	0.00	0.00	0.00	0.00	0.00	0.00	0.00	0.00	0.00	0.00	0.00	0.00	0.00	0.00	0.00	0.00	0.00	0.00
Finland - Escherichia coli ColREC UTI - model 28	0.00	0.00	0.00	0.00	0.00	0.00	0.00	0.00	0.00	NaN	NaN	NaN	0.00	0.00	0.00	0.00	0.00	0.00	0.00	0.00	0.00	0.00	0.00	0.00	0.00	0.00	0.00	0.00	0.00	0.00	0.00	0.00	0.00
Finland - Escherichia coli CREC BSI - model 21	0.00	0.00	0.00	0.00	0.00	0.00	0.00	0.00	0.00	NaN	NaN	NaN	0.00	0.00	0.00	0.00	0.00	0.00	0.00	0.00	0.00	0.00	0.00	0.00	0.00	0.00	0.00	0.00	0.00	0.00	0.00	0.00	0.00
Finland - Escherichia coli CREC OTH - model 25	0.00	0.00	0.00	0.00	0.00	0.00	0.00	0.00	0.00	NaN	NaN	NaN	0.00	0.00	0.00	0.00	0.00	0.00	0.00	0.00	0.00	0.00	0.00	0.00	0.00	0.00	0.00	0.00	0.00	0.00	0.00	0.00	0.00
Finland - Escherichia coli CREC RESP - model 22	0.00	0.00	0.00	0.00	0.00	0.00	0.00	0.00	0.00	NaN	NaN	NaN	0.00	0.00	0.00	0.00	0.00	0.00	0.00	0.00	0.00	0.00	0.00	0.00	0.00	0.00	0.00	0.00	0.00	0.00	0.00	0.00	0.00
Finland - Escherichia coli CREC SSI - model 24	0.00	0.00	0.00	0.00	0.00	0.00	0.00	0.00	0.00	NaN	NaN	NaN	0.00	0.00	0.00	0.00	0.00	0.00	0.00	0.00	0.00	0.00	0.00	0.00	0.00	0.00	0.00	0.00	0.00	0.00	0.00	0.00	0.00
Finland - Escherichia coli CREC UTI - model 23	0.00	0.00	0.00	0.00	0.00	0.00	0.00	0.00	0.00	NaN	NaN	NaN	0.00	0.00	0.00	0.00	0.00	0.00	0.00	0.00	0.00	0.00	0.00	0.00	0.00	0.00	0.00	0.00	0.00	0.00	0.00	0.00	0.00
Finland - Klebsiella pneumoniae 3GCRKP BSI - model 1	11.75	13.05	14.68	75.63	82.28	89.01	87.94	95.29	102.63	4.54	4.82	5.11	0.21	0.24	0.27	1.38	1.50	1.63	1.61	1.74	1.88	18.57	19.77	21.00	0.34	0.36	0.38	3.05	3.30	3.56	0.06	0.06	0.06
Finland - Klebsiella pneumoniae 3GCRKP OTH - model 5	0.02	0.03	0.04	0.00	0.00	0.00	0.02	0.03	0.04	2.56E-03	3.36E-03	4.28E-03	3.46E-04	4.80E-04	6.48E-04	0.00	0.00	0.00	3.46E-04	4.80E-04	6.48E-04	6.68	7.86	9.10	0.12	0.14	0.17	0.00	0.00	0.00	0.00	0.00	0.00
Finland - Klebsiella pneumoniae 3GCRKP RESP - model 2	6.69	7.84	9.26	13.17	15.29	17.59	19.97	23.12	26.72	1.24	1.35	1.49	0.12	0.14	0.17	0.24	0.28	0.32	0.36	0.42	0.49	14.95	17.10	19.41	0.27	0.31	0.35	0.53	0.61	0.71	9.71E-03	0.01	0.01
Finland - Klebsiella pneumoniae 3GCRKP SSI - model 4	0.01	0.02	0.02	3.39	4.08	4.75	3.41	4.10	4.76	0.50	0.52	0.55	1.96E-04	2.99E-04	4.13E-04	0.06	0.07	0.09	0.06	0.07	0.09	6.60	7.79	9.00	0.12	0.14	0.16	0.18	0.23	0.26	3.36E-03	4.12E-03	4.84E-03
Finland - Klebsiella pneumoniae 3GCRKP UTI - model 3	0.03	0.05	0.06	0.00	0.00	0.00	0.03	0.05	0.06	1.50E-03	1.97E-03	2.41E-03	6.20E-04	8.33E-04	1.08E-03	0.00	0.00	0.00	6.20E-04	8.33E-04	1.08E-03	20.16	23.20	26.01	0.37	0.42	0.48	0.00	0.00	0.00	0.00	0.00	0.00
Finland - Klebsiella pneumoniae ColRKP BSI - model 11	0.00	0.00	0.00	0.00	0.00	0.00	0.00	0.00	0.00	NaN	NaN	NaN	0.00	0.00	0.00	0.00	0.00	0.00	0.00	0.00	0.00	0.00	0.00	0.00	0.00	0.00	0.00	0.00	0.00	0.00	0.00	0.00	0.00
Finland - Klebsiella pneumoniae ColRKP OTH - model 15	0.00	0.00	0.00	0.00	0.00	0.00	0.00	0.00	0.00	NaN	NaN	NaN	0.00	0.00	0.00	0.00	0.00	0.00	0.00	0.00	0.00	0.00	0.00	0.00	0.00	0.00	0.00	0.00	0.00	0.00	0.00	0.00	0.00
Finland - Klebsiella pneumoniae ColRKP RESP - model 12	0.00	0.00	0.00	0.00	0.00	0.00	0.00	0.00	0.00	NaN	NaN	NaN	0.00	0.00	0.00	0.00	0.00	0.00	0.00	0.00	0.00	0.00	0.00	0.00	0.00	0.00	0.00	0.00	0.00	0.00	0.00	0.00	0.00
Finland - Klebsiella pneumoniae ColRKP SSI - model 14	0.00	0.00	0.00	0.00	0.00	0.00	0.00	0.00	0.00	NaN	NaN	NaN	0.00	0.00	0.00	0.00	0.00	0.00	0.00	0.00	0.00	0.00	0.00	0.00	0.00	0.00	0.00	0.00	0.00	0.00	0.00	0.00	0.00
Finland - Klebsiella pneumoniae ColRKP UTI - model 13	0.00	0.00	0.00	0.00	0.00	0.00	0.00	0.00	0.00	NaN	NaN	NaN	0.00	0.00	0.00	0.00	0.00	0.00	0.00	0.00	0.00	0.00	0.00	0.00	0.00	0.00	0.00	0.00	0.00	0.00	0.00	0.00	0.00
Finland - Klebsiella pneumoniae CRKP BSI - model 6	0.00	0.00	0.00	0.00	0.00	0.00	0.00	0.00	0.00	NaN	NaN	NaN	0.00	0.00	0.00	0.00	0.00	0.00	0.00	0.00	0.00	0.00	0.00	0.00	0.00	0.00	0.00	0.00	0.00	0.00	0.00	0.00	0.00
Finland - Klebsiella pneumoniae CRKP OTH - model 10	0.00	0.00	0.00	0.00	0.00	0.00	0.00	0.00	0.00	NaN	NaN	NaN	0.00	0.00	0.00	0.00	0.00	0.00	0.00	0.00	0.00	0.00	0.00	0.00	0.00	0.00	0.00	0.00	0.00	0.00	0.00	0.00	0.00
Finland - Klebsiella pneumoniae CRKP RESP - model 7	0.00	0.00	0.00	0.00	0.00	0.00	0.00	0.00	0.00	NaN	NaN	NaN	0.00	0.00	0.00	0.00	0.00	0.00	0.00	0.00	0.00	0.00	0.00	0.00	0.00	0.00	0.00	0.00	0.00	0.00	0.00	0.00	0.00
Finland - Klebsiella pneumoniae CRKP SSI - model 9	0.00	0.00	0.00	0.00	0.00	0.00	0.00	0.00	0.00	NaN	NaN	NaN	0.00	0.00	0.00	0.00	0.00	0.00	0.00	0.00	0.00	0.00	0.00	0.00	0.00	0.00	0.00	0.00	0.00	0.00	0.00	0.00	0.00
Finland - Klebsiella pneumoniae CRKP UTI - model 8	0.00	0.00	0.00	0.00	0.00	0.00	0.00	0.00	0.00	NaN	NaN	NaN	0.00	0.00	0.00	0.00	0.00	0.00	0.00	0.00	0.00	0.00	0.00	0.00	0.00	0.00	0.00	0.00	0.00	0.00	0.00	0.00	0.00
Finland - Pseudomonas aeruginosa ColRPA BSI - model 56	0.00	0.00	0.00	0.00	0.00	0.00	0.00	0.00	0.00	NaN	NaN	NaN	0.00	0.00	0.00	0.00	0.00	0.00	0.00	0.00	0.00	0.00	0.00	0.00	0.00	0.00	0.00	0.00	0.00	0.00	0.00	0.00	0.00
Finland - Pseudomonas aeruginosa ColRPA OTH - model 60	0.00	0.00	0.00	0.00	0.00	0.00	0.00	0.00	0.00	NaN	NaN	NaN	0.00	0.00	0.00	0.00	0.00	0.00	0.00	0.00	0.00	0.00	0.00	0.00	0.00	0.00	0.00	0.00	0.00	0.00	0.00	0.00	0.00
Finland - Pseudomonas aeruginosa ColRPA RESP - model 57	0.00	0.00	0.00	0.00	0.00	0.00	0.00	0.00	0.00	NaN	NaN	NaN	0.00	0.00	0.00	0.00	0.00	0.00	0.00	0.00	0.00	0.00	0.00	0.00	0.00	0.00	0.00	0.00	0.00	0.00	0.00	0.00	0.00
Finland - Pseudomonas aeruginosa ColRPA SSI - model 59	0.00	0.00	0.00	0.00	0.00	0.00	0.00	0.00	0.00	NaN	NaN	NaN	0.00	0.00	0.00	0.00	0.00	0.00	0.00	0.00	0.00	0.00	0.00	0.00	0.00	0.00	0.00	0.00	0.00	0.00	0.00	0.00	0.00
Finland - Pseudomonas aeruginosa ColRPA UTI - model 58	0.00	0.00	0.00	0.00	0.00	0.00	0.00	0.00	0.00	NaN	NaN	NaN	0.00	0.00	0.00	0.00	0.00	0.00	0.00	0.00	0.00	0.00	0.00	0.00	0.00	0.00	0.00	0.00	0.00	0.00	0.00	0.00	0.00
Finland - Pseudomonas aeruginosa CRPA BSI - model 51	12.92	15.06	17.77	92.86	128.72	167.47	107.08	143.80	184.60	6.30	8.31	10.42	0.24	0.28	0.32	1.70	2.35	3.06	1.96	2.63	3.37	15.54	17.33	19.44	0.28	0.32	0.36	2.93	3.95	5.09	0.05	0.07	0.09
Finland - Pseudomonas aeruginosa CRPA OTH - model 55	0.02	0.03	0.04	0.00	0.00	0.00	0.02	0.03	0.04	2.83E-03	3.63E-03	4.56E-03	4.09E-04	5.79E-04	7.88E-04	0.00	0.00	0.00	4.09E-04	5.79E-04	7.88E-04	6.83	8.81	10.71	0.12	0.16	0.20	0.00	0.00	0.00	0.00	0.00	0.00
Finland - Pseudomonas aeruginosa CRPA RESP - model 52	31.43	38.75	46.53	99.33	138.93	185.20	133.99	178.09	229.92	2.21	2.77	3.38	0.57	0.71	0.85	1.82	2.54	3.38	2.45	3.25	4.20	54.38	64.25	73.81	0.99	1.17	1.35	3.20	4.22	5.49	0.06	0.08	0.10
Finland - Pseudomonas aeruginosa CRPA SSI - model 54	0.02	0.03	0.04	4.26	5.43	6.62	4.28	5.46	6.66	0.43	0.46	0.49	3.05E-04	4.63E-04	6.53E-04	0.08	0.10	0.12	0.08	0.10	0.12	9.58	12.00	14.46	0.18	0.22	0.26	0.19	0.25	0.31	3.41E-03	4.54E-03	5.69E-03
Finland - Pseudomonas aeruginosa CRPA UTI - model 53	0.03	0.05	0.06	0.00	0.00	0.00	0.03	0.05	0.06	1.59E-03	2.04E-03	2.57E-03	5.85E-04	8.39E-04	1.09E-03	0.00	0.00	0.00	5.85E-04	8.39E-04	1.09E-03	17.90	22.45	26.58	0.33	0.41	0.49	0.00	0.00	0.00	0.00	0.00	0.00
Finland - Pseudomonas aeruginosa MDRPA BSI - model 46	3.68	4.45	5.37	23.36	34.63	48.28	27.56	39.10	53.05	3.23	4.49	5.82	0.07	0.08	0.10	0.43	0.63	0.88	0.50	0.71	0.97	7.50	8.69	9.96	0.14	0.16	0.18	1.28	1.81	2.49	0.02	0.03	0.05
Finland - Pseudomonas aeruginosa MDRPA OTH - model 50	0.01	0.02	0.02	0.00	0.00	0.00	0.01	0.02	0.02	2.60E-03	3.72E-03	4.95E-03	1.83E-04	3.00E-04	4.30E-04	0.00	0.00	0.00	1.83E-04	3.00E-04	4.30E-04	3.20	4.41	5.72	0.06	0.08	0.10	0.00	0.00	0.00	0.00	0.00	0.00
Finland - Pseudomonas aeruginosa MDRPA RESP - model 47	8.92	11.31	14.07	25.43	37.64	51.60	35.23	48.74	65.30	1.20	1.53	1.93	0.16	0.21	0.26	0.46	0.69	0.94	0.64	0.89	1.19	26.11	32.09	38.04	0.48	0.59	0.70	1.38	1.97	2.65	0.03	0.04	0.05
Finland - Pseudomonas aeruginosa MDRPA SSI - model 49	7.42E-03	0.01	0.02	1.51	2.17	2.84	1.52	2.18	2.85	0.33	0.36	0.40	1.36E-04	2.32E-04	3.77E-04	0.03	0.04	0.05	0.03	0.04	0.05	4.32	5.99	7.60	0.08	0.11	0.14	0.11	0.15	0.20	1.95E-03	2.82E-03	3.73E-03
Finland - Pseudomonas aeruginosa MDRPA UTI - model 48	0.01	0.02	0.03	0.00	0.00	0.00	0.01	0.02	0.03	1.42E-03	2.04E-03	2.68E-03	2.67E-04	4.13E-04	5.98E-04	0.00	0.00	0.00	2.67E-04	4.13E-04	5.98E-04	8.25	11.12	14.04	0.15	0.20	0.26	0.00	0.00	0.00	0.00	0.00	0.00
Finland - Staphylococcus aureus MRSA BSI - model 61	29.03	32.62	36.62	204.20	220.40	237.86	234.97	253.01	272.27	5.85	6.20	6.60	0.53	0.60	0.67	3.73	4.03	4.35	4.29	4.62	4.98	38.49	40.86	43.32	0.70	0.75	0.79	6.81	7.33	7.92	0.12	0.13	0.14
Finland - Staphylococcus aureus MRSA OTH - model 65	0.15	0.20	0.25	0.00	0.00	0.00	0.15	0.20	0.25	2.50E-03	3.31E-03	4.07E-03	2.71E-03	3.62E-03	4.63E-03	0.00	0.00	0.00	2.71E-03	3.62E-03	4.63E-03	53.48	60.08	66.46	0.98	1.10	1.21	0.00	0.00	0.00	0.00	0.00	0.00
Finland - Staphylococcus aureus MRSA RESP - model 62	29.78	33.88	38.36	58.44	66.14	74.75	88.82	99.88	112.48	1.51	1.63	1.77	0.54	0.62	0.70	1.07	1.21	1.37	1.62	1.83	2.06	54.61	61.34	67.86	1.00	1.12	1.24	1.94	2.20	2.48	0.04	0.04	0.05
Finland - Staphylococcus aureus MRSA SSI - model 64	0.10	0.14	0.20	23.22	25.69	28.04	23.38	25.82	28.20	0.37	0.38	0.40	1.87E-03	2.64E-03	3.60E-03	0.42	0.47	0.51	0.43	0.47	0.52	60.22	67.17	73.74	1.10	1.23	1.35	1.12	1.30	1.47	0.02	0.02	0.03
Finland - Staphylococcus aureus MRSA UTI - model 63	0.01	0.02	0.03	0.00	0.00	0.00	0.01	0.02	0.03	1.48E-03	1.87E-03	2.36E-03	2.50E-04	3.39E-04	4.71E-04	0.00	0.00	0.00	2.50E-04	3.39E-04	4.71E-04	7.96	10.04	12.08	0.15	0.18	0.22	0.00	0.00	0.00	0.00	0.00	0.00
Finland - Streptococcus pneumoniae PMRSP BSI - model 76	0.25	0.35	0.46	2.07	2.36	2.63	2.39	2.70	3.01	2.34	2.65	2.95	4.59E-03	6.37E-03	8.40E-03	0.04	0.04	0.05	0.04	0.05	0.05	1.01	1.02	1.03	0.02	0.02	0.02	0.16	0.18	0.21	2.94E-03	3.36E-03	3.75E-03
Finland - Streptococcus pneumoniae PMRSP OTH - model 80	2.60E-04	6.91E-04	1.38E-03	0.00	0.00	0.00	2.60E-04	6.91E-04	1.38E-03	7.08E-04	1.93E-03	3.64E-03	4.75E-06	1.26E-05	2.52E-05	0.00	0.00	0.00	4.75E-06	1.26E-05	2.52E-05	0.29	0.36	0.47	5.34E-03	6.59E-03	8.60E-03	0.00	0.00	0.00	0.00	0.00	0.00
Finland - Streptococcus pneumoniae PMRSP RESP - model 77	0.61	0.91	1.54	1.19	1.75	2.88	1.86	2.68	4.28	0.60	0.70	0.81	0.01	0.02	0.03	0.02	0.03	0.05	0.03	0.05	0.08	2.79	3.81	6.05	0.05	0.07	0.11	0.09	0.14	0.23	1.69E-03	2.50E-03	4.12E-03
Finland - Streptococcus pneumoniae PMRSP SSI - model 79	0.00	0.00	0.00	0.00	0.00	0.00	0.00	0.00	0.00	NaN	NaN	NaN	0.00	0.00	0.00	0.00	0.00	0.00	0.00	0.00	0.00	0.00	0.00	0.00	0.00	0.00	0.00	0.00	0.00	0.00	0.00	0.00	0.00
Finland - Streptococcus pneumoniae PMRSP UTI - model 78	0.00	0.00	0.00	0.00	0.00	0.00	0.00	0.00	0.00	NaN	NaN	NaN	0.00	0.00	0.00	0.00	0.00	0.00	0.00	0.00	0.00	0.00	0.00	0.00	0.00	0.00	0.00	0.00	0.00	0.00	0.00	0.00	0.00
Finland - Streptococcus pneumoniae PRSP BSI - model 71	0.00	0.00	0.00	0.00	0.00	0.00	0.00	0.00	0.00	NaN	NaN	NaN	0.00	0.00	0.00	0.00	0.00	0.00	0.00	0.00	0.00	0.00	0.00	0.00	0.00	0.00	0.00	0.00	0.00	0.00	0.00	0.00	0.00
Finland - Streptococcus pneumoniae PRSP OTH - model 75	0.00	0.00	0.00	0.00	0.00	0.00	0.00	0.00	0.00	NaN	NaN	NaN	0.00	0.00	0.00	0.00	0.00	0.00	0.00	0.00	0.00	0.00	0.00	0.00	0.00	0.00	0.00	0.00	0.00	0.00	0.00	0.00	0.00
Finland - Streptococcus pneumoniae PRSP RESP - model 72	0.00	0.00	0.00	0.00	0.00	0.00	0.00	0.00	0.00	NaN	NaN	NaN	0.00	0.00	0.00	0.00	0.00	0.00	0.00	0.00	0.00	0.00	0.00	0.00	0.00	0.00	0.00	0.00	0.00	0.00	0.00	0.00	0.00
Finland - Streptococcus pneumoniae PRSP SSI - model 74	0.00	0.00	0.00	0.00	0.00	0.00	0.00	0.00	0.00	NaN	NaN	NaN	0.00	0.00	0.00	0.00	0.00	0.00	0.00	0.00	0.00	0.00	0.00	0.00	0.00	0.00	0.00	0.00	0.00	0.00	0.00	0.00	0.00
Finland - Streptococcus pneumoniae PRSP UTI - model 73	0.00	0.00	0.00	0.00	0.00	0.00	0.00	0.00	0.00	NaN	NaN	NaN	0.00	0.00	0.00	0.00	0.00	0.00	0.00	0.00	0.00	0.00	0.00	0.00	0.00	0.00	0.00	0.00	0.00	0.00	0.00	0.00	0.00
**Total (Median & 95% UIs)**	312.93	357.73	407.87	1,680.14	1,947.92	2,244.37	2,012.55	2,306.37	2,637.03		-		5.72	6.54	7.45	30.71	35.60	41.02	36.78	42.15	48.19	2,242.06	2,524.07	2,821.68	40.98	46.13	51.57	77.79	89.98	103.85	1.42	1.64	1.90
**Total (average)**		**358.76**			**1,951.01**			**2,309.77**			-			**6.56**			**35.66**			**42.21**			**2,525.84**			**46.16**			**90.26**			**1.65**	

Infection	YLD			YLL			DALY			DALY per Case			YLD per 100,000			YLL per 100,000			DALY per 100,000			Cases per year			Incidence per 100,000			Deaths per year			Mortality per 100,000		
Colistin resistant *Acinetobacter spp.*	10.48	18.42	31.29	36.96	96.74	193.85	49.59	114.91	221.26	10.05	18.59	27.47	0.02	0.03	0.05	0.06	0.15	0.29	0.07	0.17	0.33	9.42	18.31	30.71	0.01	0.03	0.05	0.58	1.52	3.04	0.00	0.00	0.00
Carbapenem resistant *Acinetobacter spp.* (excluding those resistant to colistin)	164.25	192.97	229.19	807.57	1,029.49	1,282.49	977.46	1,221.64	1,499.06	6.74	8.14	9.67	0.25	0.29	0.34	1.21	1.55	1.93	1.47	1.84	2.25	376.59	444.76	519.41	0.57	0.67	0.78	30.98	38.20	46.44	0.05	0.06	0.07
Aminoglycosides and fluoroquinolones resistant *Acinetobacter* spp. (excluding those resistant to colistin and/or carbapenem)	73.97	93.78	119.59	351.48	494.04	674.26	432.93	587.94	783.36	6.04	7.95	9.97	0.11	0.14	0.18	0.53	0.74	1.01	0.65	0.88	1.18	175.69	221.89	273.51	0.26	0.33	0.41	13.65	18.78	24.72	0.02	0.03	0.04
Vancomycin resistant Enterococci (*E. faecalis* and *E. faecium*)	61.17	82.51	109.81	586.53	771.81	1,025.66	650.00	854.43	1,127.19	7.33	8.16	9.18	0.09	0.12	0.17	0.88	1.16	1.54	0.98	1.29	1.70	296.30	396.07	505.22	0.45	0.60	0.76	20.19	26.45	33.90	0.03	0.04	0.05
Colistin resistant *Escherichia coli*	23.84	31.91	42.69	395.89	553.44	768.54	421.65	585.86	806.42	11.74	15.75	20.25	0.04	0.05	0.06	0.60	0.83	1.16	0.63	0.88	1.21	211.32	290.90	378.43	0.32	0.44	0.57	17.76	25.20	34.73	0.03	0.04	0.05
Carbapenem resistant *Escherichia coli* (excluding those resistant to colistin)	12.87	16.63	22.26	132.28	176.12	236.27	144.47	192.63	257.65	8.53	10.69	13.00	0.02	0.03	0.03	0.20	0.26	0.36	0.22	0.29	0.39	105.14	144.24	190.08	0.16	0.22	0.29	5.44	7.63	10.52	0.01	0.01	0.02
Third-generation cephalosporin resistant *Escherichia coli* (excluding those resistant to colistin and/or carbapenem)	4,838.69	5,459.70	6,249.17	28,517.09	32,168.33	36,673.30	33,508.72	37,620.40	42,742.00	4.92	5.39	5.89	7.28	8.21	9.40	42.89	48.38	55.16	50.40	56.58	64.29	47,961.12	55,957.55	64,194.76	72.13	84.16	96.55	1,475.20	1,703.78	1,967.39	2.22	2.56	2.96
Colistin resistant *Klebsiella pneumoniae*	113.54	141.65	177.03	1,966.60	2,514.54	3,141.77	2,102.80	2,658.21	3,302.61	18.03	21.72	26.05	0.17	0.21	0.27	2.96	3.78	4.73	3.16	4.00	4.97	284.18	367.73	459.27	0.43	0.55	0.69	64.07	80.43	100.70	0.10	0.12	0.15
Carbapenem resistant *Klebsiella pneumoniae* (excluding those resistant to colistin)	50.72	67.22	87.26	524.71	715.83	939.36	581.97	784.23	1,022.05	10.61	13.29	15.99	0.08	0.10	0.13	0.79	1.08	1.41	0.88	1.18	1.54	128.08	183.99	250.28	0.19	0.28	0.38	17.37	24.45	33.72	0.03	0.04	0.05
Third-generation cephalosporin resistant *Klebsiella pneumoniae* (excluding those resistant to colistin and/or carbapenem)	4,138.74	4,573.06	5,076.61	20,093.84	21,885.65	23,873.94	24,286.06	26,475.68	28,928.40	6.33	6.66	7.03	6.22	6.88	7.64	30.22	32.92	35.91	36.53	39.82	43.51	14,478.03	16,487.78	18,464.21	21.78	24.80	27.77	797.93	884.58	977.33	1.20	1.33	1.47
Colistin resistant *Pseudomonas aeruginosa*	0.00	0.00	0.00	0.00	0.00	0.00	0.00	0.00	0.00	0.00	0.00	0.00	0.00	0.00	0.00	0.00	0.00	0.00	0.00	0.00	0.00	0.00	0.00	0.00	0.00	0.00	0.00	0.00	0.00	0.00	0.00	0.00	0.00
Carbapenem resistant *Pseudomonas aeruginosa* (excluding those resistant to colistin)	4,878.90	5,771.95	6,751.41	23,030.85	29,005.17	36,114.77	28,241.26	34,716.98	42,322.08	8.44	10.11	11.76	7.34	8.68	10.15	34.64	43.62	54.32	42.48	52.22	63.65	12,756.98	15,234.18	17,913.90	19.19	22.91	26.94	800.72	1,024.90	1,296.94	1.20	1.54	1.95
Pseudomonas aeruginosa resistant to three or more antimicrobial groups (excluding those resistant to colistin and/or carbapenem)	870.94	1,036.02	1,231.13	3,864.03	4,908.67	6,141.49	4,764.64	5,947.88	7,289.07	7.27	8.62	10.04	1.31	1.56	1.85	5.81	7.38	9.24	7.17	8.95	10.96	2,545.95	3,036.34	3,573.66	3.83	4.57	5.37	151.17	192.67	239.06	0.23	0.29	0.36
Methicillin resistant *Staphylococcus aureus*	5.335.81	6.003.83	6.661.74	26.424.83	28.900.05	31.481.46	31.795.87	34.915.84	38.104.46	5.30	5.63	5.99	8.03	9.03	10.02	39.74	43.47	47.35	47.82	52.51	57.31	28.174.64	31.998.99	36.149.02	42.38	48.13	54.37	1.347.31	1.513.19	1.687.73	2.03	2.28	2.54
Penicillin and macrolides resistant *Streptococcus pneumoniae* (excluding those only resistant to penicillin)	6.28	7.90	10.50	22.14	25.86	31.26	28.93	33.76	40.78	4.43	4.80	5.20	0.01	0.01	0.02	0.03	0.04	0.05	0.04	0.05	0.06	19.36	22.80	28.18	0.03	0.03	0.04	1.21	1.41	1.68	0.00	0.00	0.00
Penicillin tesistant *Streptococcus pneumoniae* (excluding those resistant to macrolides)	0.00	0.00	0.00	0.00	0.00	0.00	0.00	0.00	0.00	0.00	0.00	0.00	0.00	0.00	0.00	0.00	0.00	0.00	0.00	0.00	0.00	0.00	0.00	0.00	0.00	0.00	0.00	0.00	0.00	0.00	0.00	0.00	0.00
Total	20,580.20	23,497.54	26,799.68	106,754.80	123,245.73	142,578.41	127,986.35	146,710.40	168,446.37				30.95	35.34	40.31	160.56	185.36	214.44	192.49	220.66	253.35	107,522.80	124,805.54	142,930.63	161.72	187.71	214.97	4,743.59	5,543.20	6,457.89	7.13	8.34	9.71

Model	YLD			YLL			DALY			DALY per Case			YLD per 100,000			YLL per 100,000			DALY per 100,000			Cases per year			Incidence per 100,000			Deaths per year			Mortality per 100,000		
France - Acinetobacter spp. CoRACI BSI - model 41	6.55E+00	1.04E+01	1.73E+01	2.87E+01	7.98E+01	1.64E+02	3.75E+01	8.97E+01	1.78E+02	6.50E+00	1.46E+01	2.30E+01	9.86E-03	1.57E-02	2.61E-02	4.32E-02	1.20E-01	2.47E-01	5.64E-02	1.35E-01	2.67E-01	4.46E+00	6.18E+00	9.41E+00	6.71E-03	9.30E-03	1.42E-02	4.50E-01	1.25E+00	2.57E+00	6.77E-04	1.88E-03	3.87E-03
France - Acinetobacter spp. CoRACI OTH - model 45	9.50E-05	8.20E-04	2.82E-03	0.00	0.00	0.00	9.50E-05	8.20E-04	2.82E-03	1.62E-03	3.73E-03	6.49E-03	1.43E-07	1.23E-06	4.25E-06	0.00	0.00	0.00	1.43E-07	1.23E-06	4.25E-06	0.03	0.23	0.59	4.59E-05	3.47E-04	8.86E-04	0.00	0.00	0.00	0.00	0.00	0.00
France - Acinetobacter spp. CoRACI RESP - model 42	3.92	7.99	13.92	7.82	15.58	27.02	11.71	23.81	41.14	2.97	3.44	3.93	5.90E-03	0.01	0.02	0.01	0.02	0.04	0.02	0.04	0.06	3.44	6.88	11.47	5.17E-03	0.01	0.02	0.12	0.24	0.42	1.84E-04	3.67E-04	6.36E-04
France - Acinetobacter spp. CoRACI SSI - model 44	8.94E-04	4.36E-03	0.01	0.42	1.34	2.48	0.42	1.34	2.50	0.58	0.58	0.58	1.34E-06	6.56E-06	2.16E-05	6.25E-04	2.02E-03	3.73E-03	6.26E-04	2.02E-03	3.76E-03	0.72	2.33	4.32	1.09E-03	3.51E-03	6.49E-03	6.51E-03	0.02	0.04	9.79E-06	3.16E-05	5.85E-05
France - Acinetobacter spp. CoRACI UTI - model 43	1.10E-03	4.82E-03	0.01	0.00	0.00	0.00	1.10E-03	4.82E-03	0.01	8.05E-04	2.02E-03	3.67E-03	1.66E-06	7.25E-06	2.01E-05	0.00	0.00	0.00	1.66E-06	7.25E-06	2.01E-05	0.77	2.69	4.92	1.16E-03	4.04E-03	7.39E-03	0.00	0.00	0.00	0.00	0.00	0.00
France - Acinetobacter spp. CRACI BSI - model 36	95.49	110.36	127.69	657.26	845.48	1,059.92	757.91	954.76	1,177.04	5.03	6.27	7.61	0.14	0.17	0.19	0.99	1.27	1.59	1.14	1.44	1.77	136.55	152.40	168.64	0.21	0.23	0.25	25.13	31.08	37.98	0.04	0.05	0.06
France - Acinetobacter spp. CRACI OTH - model 40	0.01	0.02	0.03	0.00	0.00	0.00	0.01	0.02	0.03	2.93E-03	3.76E-03	4.54E-03	2.19E-05	3.35E-05	4.78E-05	0.00	0.00	0.00	2.19E-05	3.35E-05	4.78E-05	4.13	5.97	7.78	6.21E-03	8.97E-03	0.01	0.00	0.00	0.00	0.00	0.00	0.00
France - Acinetobacter spp. CRACI RESP - model 37	68.57	82.34	101.11	132.98	161.72	194.72	201.99	244.32	293.83	1.35	1.47	1.62	0.10	0.12	0.15	0.20	0.24	0.29	0.30	0.37	0.44	140.03	165.71	195.39	0.21	0.25	0.29	4.98	5.98	7.02	7.50E-03	8.99E-03	0.01
France - Acinetobacter spp. CRACI SSI - model 39	0.08	0.12	0.17	17.33	22.30	27.85	17.46	22.40	27.99	0.35	0.39	0.43	1.24E-04	1.84E-04	2.56E-04	0.03	0.03	0.04	0.03	0.03	0.04	45.52	57.17	69.43	0.07	0.09	0.10	0.87	1.15	1.44	1.31E-03	1.73E-03	2.16E-03
France - Acinetobacter spp. CRACI UTI - model 38	0.09	0.13	0.18	0.00	0.00	0.00	0.09	0.13	0.18	1.58E-03	2.04E-03	2.50E-03	1.40E-04	1.94E-04	2.64E-04	0.00	0.00	0.00	1.40E-04	1.94E-04	2.64E-04	50.37	63.51	78.18	0.08	0.10	0.12	0.00	0.00	0.00	0.00	0.00	0.00
France - Acinetobacter spp. MDRACI BSI - model 31	43.58	53.37	66.93	281.59	401.13	553.45	332.30	454.86	610.81	4.33	5.99	7.74	0.07	0.08	0.10	0.42	0.60	0.83	0.50	0.68	0.92	66.29	76.00	88.87	0.10	0.11	0.13	10.83	15.04	19.97	0.02	0.02	0.03
France - Acinetobacter spp. MDRACI OTH - model 35	5.78E-03	0.01	0.02	0.00	0.00	0.00	5.78E-03	0.01	0.02	2.61E-03	3.73E-03	4.94E-03	8.70E-06	1.65E-05	2.62E-05	0.00	0.00	0.00	8.70E-06	1.65E-05	2.62E-05	1.82	2.99	4.35	2.74E-03	4.49E-03	6.54E-03	0.00	0.00	0.00	0.00	0.00	0.00
France - Acinetobacter spp. MDRACI RESP - model 32	30.30	40.28	52.45	60.08	78.47	101.69	90.73	118.50	153.26	1.23	1.43	1.67	0.05	0.06	0.08	0.09	0.12	0.15	0.14	0.18	0.23	65.57	82.71	102.25	0.10	0.12	0.15	2.32	2.99	3.74	3.49E-03	4.50E-03	5.63E-03
France - Acinetobacter spp. MDRACI SSI - model 34	0.03	0.06	0.09	9.81	14.44	19.12	9.85	14.51	19.18	0.47	0.52	0.56	5.10E-05	8.73E-05	1.40E-04	0.01	0.02	0.03	0.01	0.02	0.03	19.68	28.26	36.47	0.03	0.04	0.05	0.50	0.75	1.01	7.50E-04	1.13E-03	1.52E-03
France - Acinetobacter spp. MDRACI UTI - model 33	0.04	0.06	0.10	0.00	0.00	0.00	0.04	0.06	0.10	1.50E-03	2.05E-03	2.66E-03	6.24E-05	9.67E-05	1.43E-04	0.00	0.00	0.00	6.24E-05	9.67E-05	1.43E-04	22.32	31.92	41.57	0.03	0.05	0.06	0.00	0.00	0.00	0.00	0.00	0.00
France - Enterococcus faecalis and E. faecium VRE BSI - model 66	60.81	81.95	108.99	538.41	703.92	934.63	601.57	785.96	1,035.42	6.89	7.65	8.62	0.09	0.12	0.16	0.81	1.06	1.41	0.90	1.18	1.56	79.85	102.42	129.46	0.12	0.15	0.19	18.08	23.42	29.74	0.03	0.04	0.04
France - Enterococcus faecalis and E. faecium VRE OTH - model 70	0.12	0.17	0.26	0.00	0.00	0.00	0.12	0.17	0.26	1.25E-03	1.65E-03	2.13E-03	1.73E-04	2.59E-04	3.84E-04	0.00	0.00	0.00	1.73E-04	2.59E-04	3.84E-04	77.65	106.17	135.53	0.12	0.16	0.20	0.00	0.00	0.00	0.00	0.00	0.00
France - Enterococcus faecalis and E. faecium VRE RESP - model 67	0.00	0.00	0.00	0.00	0.00	0.00	0.00	0.00	0.00	NaN	NaN	NaN	0.00	0.00	0.00	0.00	0.00	0.00	0.00	0.00	0.00	0.00	0.00	0.00	0.00	0.00	0.00	0.00	0.00	0.00	0.00	0.00	0.00
France - Enterococcus faecalis and E. faecium VRE SSI - model 69	0.19	0.29	0.43	48.12	67.89	91.02	48.25	68.20	91.37	0.44	0.50	0.55	2.79E-04	4.33E-04	6.49E-04	0.07	0.10	0.14	0.07	0.10	0.14	101.77	136.91	174.39	0.15	0.21	0.26	2.11	3.04	4.16	3.17E-03	4.57E-03	6.26E-03
France - Enterococcus faecalis and E. faecium VRE UTI - model 68	0.06	0.09	0.14	0.00	0.00	0.00	0.06	0.09	0.14	1.43E-03	1.88E-03	2.34E-03	9.52E-05	1.43E-04	2.05E-04	0.00	0.00	0.00	9.52E-05	1.43E-04	2.05E-04	37.03	50.58	65.84	0.06	0.08	0.10	0.00	0.00	0.00	0.00	0.00	0.00
France - Escherichia coli 3GCREC BSI - model 16	3,706.37	4,089.37	4,609.55	23,700.15	26,396.43	29,848.04	27,557.10	30,472.32	34,314.09	3.60	3.92	4.26	5.57	6.15	6.93	35.65	39.70	44.89	41.45	45.83	51.61	7,087.64	7,776.64	8,664.30	10.66	11.70	13.03	1,188.16	1,349.40	1,539.38	1.79	2.03	2.32
France - Escherichia coli 3GCREC OTH - model 20	6.45	8.80	11.54	0.00	0.00	0.00	6.45	8.80	11.54	2.69E-03	3.35E-03	4.05E-03	9.69E-03	0.01	0.02	0.00	0.00	0.00	9.69E-03	0.01	0.02	2,004.89	2,672.47	3,245.33	3.02	4.02	4.88	0.00	0.00	0.00	0.00	0.00	0.00
France - Escherichia coli 3GCREC RESP - model 17	1,061.00	1,278.32	1,520.76	2,073.30	2,479.77	2,905.21	3,135.91	3,762.93	4,391.38	0.95	1.07	1.20	1.60	1.92	2.29	3.12	3.73	4.37	4.72	5.66	6.60	2,841.65	3,520.04	4,214.31	4.27	5.29	6.34	102.04	126.41	152.35	0.15	0.19	0.23
France - Escherichia coli 3GCREC SSI - model 19	12.97	17.76	23.61	2,743.64	3,292.13	3,920.05	2,757.35	3,310.90	3,941.29	0.37	0.40	0.43	0.02	0.03	0.04	4.13	4.95	5.90	4.15	4.98	5.93	7,017.92	8,332.04	9,700.87	10.56	12.53	14.59	185.01	227.97	275.66	0.28	0.34	0.41
France - Escherichia coli 3GCREC UTI - model 18	51.91	65.45	83.71	0.00	0.00	0.00	51.91	65.45	83.71	1.60E-03	1.96E-03	2.34E-03	0.08	0.10	0.13	0.00	0.00	0.00	0.08	0.10	0.13	29,009.03	33,656.37	38,369.95	43.63	50.62	57.71	0.00	0.00	0.00	0.00	0.00	0.00
France - Escherichia coli CoREC BSI - model 26	18.42	23.29	30.01	370.75	516.09	716.86	390.64	539.87	742.24	10.32	14.07	18.20	0.03	0.04	0.05	0.56	0.78	1.08	0.59	0.81	1.12	32.19	38.35	46.97	0.05	0.06	0.07	16.48	23.24	32.00	0.02	0.03	0.05
France - Escherichia coli CoREC OTH - model 30	0.04	0.08	0.14	0.00	0.00	0.00	0.04	0.08	0.14	2.14E-03	3.44E-03	5.28E-03	6.35E-05	1.16E-04	2.04E-04	0.00	0.00	0.00	6.35E-05	1.16E-04	2.04E-04	15.29	22.86	30.83	0.02	0.03	0.05	0.00	0.00	0.00	0.00	0.00	0.00
France - Escherichia coli CoREC RESP - model 27	5.13	8.12	11.83	10.06	15.75	22.64	15.55	23.88	34.33	0.99	1.21	1.53	7.71E-03	0.01	0.02	0.02	0.02	0.03	0.02	0.04	0.05	12.58	19.63	28.01	0.02	0.03	0.04	0.46	0.71	1.03	6.91E-04	1.07E-03	1.54E-03
France - Escherichia coli CoREC SSI - model 29	0.05	0.10	0.17	15.08	21.60	29.04	15.21	21.70	29.17	0.43	0.47	0.51	7.71E-05	1.46E-04	2.49E-04	0.02	0.03	0.04	0.02	0.03	0.04	32.21	46.41	61.35	0.05	0.07	0.09	0.82	1.25	1.71	1.23E-03	1.88E-03	2.57E-03
France - Escherichia coli CoREC UTI - model 28	0.20	0.33	0.54	0.00	0.00	0.00	0.20	0.33	0.54	1.33E-03	2.05E-03	2.98E-03	3.04E-04	5.02E-04	8.13E-04	0.00	0.00	0.00	3.04E-04	5.02E-04	8.13E-04	119.06	163.65	211.26	0.18	0.25	0.32	0.00	0.00	0.00	0.00	0.00	0.00
France - Escherichia coli CREC BSI - model 21	9.86	12.10	15.65	120.21	158.82	212.36	129.53	170.82	227.29	7.07	9.00	11.01	0.01	0.02	0.02	0.18	0.24	0.32	0.19	0.26	0.34	15.61	19.26	23.87	0.02	0.03	0.04	4.88	6.77	9.28	7.34E-03	0.01	0.01
France - Escherichia coli CREC OTH - model 25	0.02	0.04	0.07	0.00	0.00	0.00	0.02	0.04	0.07	2.23E-03	3.45E-03	5.45E-03	3.11E-05	5.71E-05	1.07E-04	0.00	0.00	0.00	3.11E-05	5.71E-05	1.07E-04	7.28	11.19	15.61	0.01	0.02	0.02	0.00	0.00	0.00	0.00	0.00	0.00
France - Escherichia coli CREC RESP - model 22	2.86	4.27	6.18	5.55	8.29	11.85	8.27	12.56	17.87	1.10	1.29	1.53	4.30E-03	6.42E-03	9.30E-03	8.34E-03	0.01	0.02	0.01	0.02	0.03	6.28	9.81	14.07	9.45E-03	0.01	0.02	0.21	0.35	0.52	3.17E-04	5.28E-04	7.75E-04
France - Escherichia coli CREC SSI - model 24	0.03	0.05	0.09	6.52	9.01	12.06	6.55	9.06	12.15	0.36	0.40	0.45	3.86E-05	7.16E-05	1.32E-04	9.80E-03	0.01	0.02	9.85E-03	0.01	0.02	15.87	22.71	30.48	0.02	0.03	0.05	0.35	0.51	0.73	5.29E-04	7.72E-04	1.10E-03
France - Escherichia coli CREC UTI - model 23	0.10	0.17	0.27	0.00	0.00	0.00	0.10	0.17	0.27	1.36E-03	2.07E-03	3.10E-03	1.54E-04	2.51E-04	4.05E-04	0.00	0.00	0.00	1.54E-04	2.51E-04	4.05E-04	60.10	81.28	106.06	0.09	0.12	0.16	0.00	0.00	0.00	0.00	0.00	0.00
France - Klebsiella pneumoniae 3GCRKP BSI - model 1	2,597.66	2,823.82	3,097.94	16,503.69	17,790.67	19,214.48	19,138.66	20,631.37	22,296.37	4.63	4.85	5.08	3.91	4.25	4.66	24.82	26.76	28.90	28.79	31.03	33.53	3,907.42	4,253.05	4,600.98	5.88	6.40	6.92	649.39	710.15	774.22	0.98	1.07	1.16
France - Klebsiella pneumoniae 3GCRKP OTH - model 5	4.53	5.81	7.33	0.00	0.00	0.00	4.53	5.81	7.33	2.72E-03	3.35E-03	4.06E-03	6.81E-03	8.75E-03	0.01	0.00	0.00	0.00	6.81E-03	8.75E-03	0.01	1,488.72	1,727.53	1,975.97	2.24	2.60	2.97	0.00	0.00	0.00	0.00	0.00	0.00
France - Klebsiella pneumoniae 3GCRKP RESP - model 2	1,525.99	1,729.79	1,954.20	2,975.42	3,355.84	3,800.88	4,517.25	5,085.74	5,747.97	1.29	1.37	1.47	2.30	2.60	2.94	4.48	5.05	5.72	6.79	7.65	8.65	3,207.66	3,713.78	4,219.08	4.82	5.59	6.35	115.68	133.64	154.79	0.17	0.20	0.23
France - Klebsiella pneumoniae 3GCRKP SSI - model 4	2.66	3.61	4.82	614.73	739.14	858.57	617.74	742.73	864.41	0.40	0.43	0.47	4.00E-03	5.43E-03	7.25E-03	0.92	1.11	1.29	0.93	1.12	1.30	1,446.85	1,717.37	1,963.95	2.18	2.58	2.95	32.87	40.79	48.31	0.05	0.06	0.07
France - Klebsiella pneumoniae 3GCRKP UTI - model 3	7.89	10.02	12.32	0.00	0.00	0.00	7.89	10.02	12.32	1.64E-03	1.97E-03	2.34E-03	0.01	0.02	0.02	0.00	0.00	0.00	0.01	0.02	0.02	4,427.38	5,076.06	5,704.22	6.66	7.63	8.58	0.00	0.00	0.00	0.00	0.00	0.00
France - Klebsiella pneumoniae CoRKP BSI - model 11	90.17	109.18	131.80	1,910.17	2,433.40	3,030.80	2,023.15	2,544.94	3,146.17	16.17	19.56	23.56	0.14	0.16	0.20	2.87	3.66	4.56	3.04	3.83	4.73	112.50	130.23	150.01	0.17	0.20	0.23	62.14	77.65	96.86	0.09	0.12	0.15
France - Klebsiella pneumoniae CoRKP OTH - model 15	0.13	0.22	0.32	0.00	0.00	0.00	0.13	0.22	0.32	2.49E-03	3.51E-03	4.75E-03	2.03E-04	3.26E-04	4.87E-04	0.00	0.00	0.00	2.03E-04	3.26E-04	4.87E-04	44.21	62.03	81.49	0.07	0.09	0.12	0.00	0.00	0.00	0.00	0.00	0.00
France - Klebsiella pneumoniae CoRKP RESP - model 12	23.07	32.01	44.53	44.54	62.89	85.00	67.47	94.56	129.80	1.46	1.69	1.95	0.03	0.05	0.07	0.07	0.09	0.13	0.10	0.14	0.20	40.48	56.17	73.63	0.06	0.08	0.11	1.44	2.02	2.69	2.17E-03	3.04E-03	4.04E-03
France - Klebsiella pneumoniae CoRKP SSI - model 14	0.05	0.08	0.13	11.88	18.25	25.98	11.93	18.34	26.07	0.40	0.47	0.53	7.62E-05	1.25E-04	1.93E-04	0.02	0.03	0.04	0.02	0.03	0.04	27.50	39.15	53.11	0.04	0.06	0.08	0.48	0.76	1.16	7.28E-04	1.14E-03	1.74E-03
France - Klebsiella pneumoniae CoRKP UTI - model 13	0.11	0.16	0.24	0.00	0.00	0.00	0.11	0.16	0.24	1.51E-03	2.07E-03	2.69E-03	1.67E-04	2.45E-04	3.67E-04	0.00	0.00	0.00	1.67E-04	2.45E-04	3.67E-04	59.48	80.16	101.03	0.09	0.12	0.15	0.00	0.00	0.00	0.00	0.00	0.00
France - Klebsiella pneumoniae CRKP BSI - model 6	41.30	51.85	65.41	502.09	678.03	884.61	549.49	730.77	944.24	8.91	11.25	13.56	0.06	0.08	0.10	0.76	1.02	1.33	0.83	1.10	1.42	53.25	65.20	80.23	0.08	0.10	0.12	16.62	23.09	31.62	0.02	0.03	0.05
France - Klebsiella pneumoniae CRKP OTH - model 10	0.06	0.11	0.20	0.00	0.00	0.00	0.06	0.11	0.20	2.20E-03	3.48E-03	5.30E-03	8.73E-05	1.61E-04	2.94E-04	0.00	0.00	0.00	8.73E-05	1.61E-04	2.94E-04	19.42	31.03	44.43	0.03	0.05	0.07	0.00	0.00	0.00	0.00	0.00	0.00
France - Klebsiella pneumoniae CRKP RESP - model 7	9.29	15.14	21.44	18.16	29.44	41.72	27.89	44.87	64.38	1.33	1.61	1.93	0.01	0.02	0.03	0.03	0.04	0.06	0.04	0.07	0.10	16.40	28.04	40.71	0.02	0.04	0.06	0.59	1.00	1.48	8.93E-04	1.50E-03	2.23E-03
France - Klebsiella pneumoniae CRKP SSI - model 9	0.02	0.04	0.08	4.46	8.35	13.03	4.49	8.39	13.09	0.35	0.42	0.49	3.08E-05	6.30E-05	1.14E-04	6.71E-03	0.01	0.02	6.75E-03	0.01	0.02	11.88	19.73	28.83	0.02	0.03	0.04	0.16	0.36	0.62	2.36E-04	5.47E-04	9.30E-04
France - Klebsiella pneumoniae CRKP UTI - model 8	0.05	0.08	0.14	0.00	0.00	0.00	0.05	0.08	0.14	1.34E-03	2.02E-03	3.08E-03	6.82E-05	1.22E-04	2.10E-04	0.00	0.00	0.00	6.82E-05	1.22E-04	2.10E-04	27.13	39.99	56.07	0.04	0.06	0.08	0.00	0.00	0.00	0.00	0.00	0.00
France - Pseudomonas aeruginosa CoRPA BSI - model 56	0.00	0.00	0.00	0.00	0.00	0.00	0.00	0.00	0.00	NaN	NaN	NaN	0.00	0.00	0.00	0.00	0.00	0.00	0.00	0.00	0.00	0.00	0.00	0.00	0.00	0.00	0.00	0.00	0.00	0.00	0.00	0.00	0.00
France - Pseudomonas aeruginosa CoRPA OTH - model 60	0.00	0.00	0.00	0.00	0.00	0.00	0.00	0.00	0.00	NaN	NaN	NaN	0.00	0.00	0.00	0.00	0.00	0.00	0.00	0.00	0.00	0.00	0.00	0.00	0.00	0.00	0.00	0.00	0.00	0.00	0.00	0.00	0.00
France - Pseudomonas aeruginosa CoRPA RESP - model 57	0.00	0.00	0.00	0.00	0.00	0.00	0.00	0.00	0.00	NaN	NaN	NaN	0.00	0.00	0.00	0.00	0.00	0.00	0.00	0.00	0.00	0.00	0.00	0.00	0.00	0.00	0.00	0.00	0.00	0.00	0.00	0.00	0.00
France - Pseudomonas aeruginosa CoRPA SSI - model 59	0.00	0.00	0.00	0.00	0.00	0.00	0.00	0.00	0.00	NaN	NaN	NaN	0.00	0.00	0.00	0.00	0.00	0.00	0.00	0.00	0.00	0.00	0.00	0.00	0.00	0.00	0.00	0.00	0.00	0.00	0.00	0.00	0.00
France - Pseudomonas aeruginosa CoRPA UTI - model 58	0.00	0.00	0.00	0.00	0.00	0.00	0.00	0.00	0.00	NaN	NaN	NaN	0.00	0.00	0.00	0.00	0.00	0.00	0.00	0.00	0.00	0.00	0.00	0.00	0.00	0.00	0.00	0.00	0.00	0.00	0.00	0.00	0.00
France - Pseudomonas aeruginosa CRPA BSI - model 51	1,392.96	1,608.46	1,846.87	10,780.43	13,671.34	17,161.57	12,301.64	15,267.37	18,814.63	5.98	7.28	8.54	2.10	2.42	2.78	16.21	20.56	25.81	18.50	22.96	28.30	1,845.46	2,108.01	2,391.14	2.78	3.17	3.60	377.49	479.83	610.14	0.57	0.72	0.92
France - Pseudomonas aeruginosa CRPA OTH - model 55	2.89	3.94	5.26	0.00	0.00	0.00	2.89	3.94	5.26	2.96E-03	3.66E-03	4.34E-03	4.35E-03	5.93E-03	7.92E-03	0.00	0.00	0.00	4.35E-03	5.93E-03	7.92E-03	852.65	1,078.40	1,335.76	1.28	1.62	2.01	0.00	0.00	0.00	0.00	0.00	0.00
France - Pseudomonas aeruginosa CRPA RESP - model 52	3,476.77	4,150.82	4,887.65	11,775.71	14,727.94	18,208.62	15,455.20	18,831.06	22,746.91	2.08	2.41	2.75	5.23	6.24	7.35	17.71	22.15	27.39	23.25	28.32	34.21	6,669.68	7,828.58	9,123.13	10.03	11.77	13.72	401.68	516.51	650.88	0.60	0.78	0.98
France - Pseudomonas aeruginosa CRPA SSI - model 54	2.20	3.11	4.34	474.72	605.89	744.58	477.45	609.00	747.99	0.37	0.41	0.46	3.31E-03	4.68E-03	6.52E-03	0.71	0.91	1.12	0.72	0.92	1.12	1,168.17	1,468.75	1,785.32	1.76	2.21	2.69	21.55	28.57	35.91	0.03	0.04	0.05
France - Pseudomonas aeruginosa CRPA UTI - model 53	4.08	5.61	7.30	0.00	0.00	0.00	4.08	5.61	7.30	1.66E-03	2.05E-03	2.47E-03	6.14E-03	8.44E-03	0.01	0.00	0.00	0.00	6.14E-03	8.44E-03	0.01	2,221.02	2,750.44	3,278.55	3.34	4.14	4.93	0.00	0.00	0.00	0.00	0.00	0.00
France - Pseudomonas aeruginosa MDRPA BSI - model 46	251.80	290.44	333.91	1,821.28	2,271.31	2,846.41	2,087.20	2,562.57	3,157.94	5.11	6.12	7.22	0.38	0.44	0.50	2.74	3.42	4.28	3.14	3.85	4.75	370.43	420.14	471.95	0.56	0.63	0.71	69.07	88.39	109.15	0.10	0.13	0.16
France - Pseudomonas aeruginosa MDRPA OTH - model 50	0.59	0.80	1.08	0.00	0.00	0.00	0.59	0.80	1.08	3.03E-03	3.73E-03	4.49E-03	8.91E-04	1.21E-03	1.62E-03	0.00	0.00	0.00	8.91E-04	1.21E-03	1.62E-03	172.84	215.88	264.35	0.26	0.32	0.40	0.00	0.00	0.00	0.00	0.00	0.00
France - Pseudomonas aeruginosa MDRPA RESP - model 47	617.27	743.03	893.84	1,952.53	2,520.72	3,152.09	2,585.43	3,266.22	3,985.09	1.79	2.09	2.37	0.93	1.12	1.34	2.94	3.79	4.74	3.89	4.91	5.99	1,326.84	1,561.74	1,826.84	2.00	2.35	2.75	77.47	98.21	122.44	0.12	0.15	0.18
France - Pseudomonas aeruginosa MDRPA SSI - model 49	0.43	0.63	0.85	90.21	116.64	142.99	90.56	117.17	143.51	0.37	0.40	0.45	6.44E-04	9.45E-04	1.27E-03	0.14	0.18	0.22	0.14	0.18	0.22	232.92	291.32	352.76	0.35	0.44	0.53	4.63	6.06	7.47	6.96E-03	9.12E-03	0.01
France - Pseudomonas aeruginosa MDRPA UTI - model 48	0.85	1.11	1.45	0.00	0.00	0.00	0.85	1.11	1.45	1.68E-03	2.05E-03	2.45E-03	1.28E-03	1.67E-03	2.19E-03	0.00	0.00	0.00	1.28E-03	1.67E-03	2.19E-03	442.93	547.26	657.76	0.67	0.82	0.99	0.00	0.00	0.00	0.00	0.00	0.00
France - Staphylococcus aureus MRSA BSI - model 61	2,612.97	2,895.78	3,173.65	18,026.27	19,438.54	20,846.50	20,661.21	22,340.22	23,968.00	3.91	4.13	4.38	3.93	4.36	4.77	27.11	29.24	31.35	31.08	33.60	36.05	4,943.56	5,398.80	5,873.52	7.44	8.12	8.83	880.88	970.19	1,064.74	1.32	1.46	1.60
France - Staphylococcus aureus MRSA OTH - model 65	20.50	26.06	33.25	0.00	0.00	0.00	20.50	26.06	33.25	2.60E-03	3.23E-03	3.93E-03	0.03	0.04	0.05	0.00	0.00	0.00	0.03	0.04	0.05	7,039.04	8,083.11	9,176.02	10.59	12.16	13.80	0.00	0.00	0.00	0.00	0.00	0.00
France - Staphylococcus aureus MRSA RESP - model 62	2,686.04	3,060.54	3,426.78	5,297.17	5,921.51	6,623.23	7,984.01	8,988.88	10,065.35	1.01	1.09	1.18	4.04	4.60	5.15	7.97	8.91	9.96	12.01	13.52	15.14	7,179.17	8,224.06	9,361.24	10.80	12.37	14.08	254.94	296.39	337.42	0.38	0.45	0.51
France - Staphylococcus aureus MRSA SSI - model 64	14.47	18.92	24.66	3,101.39	3,540.00	4,011.72	3,128.32	3,558.16	4,034.47	0.37	0.40	0.42	0.02	0.03	0.04	4.66	5.32	6.03	4.71	5.35	6.07	7,905.63	8,925.26	10,082.37	11.89	13.42	15.16	211.48	246.60	285.57	0.32	0.37	0.43
France - Staphylococcus aureus MRSA UTI - model 63	1.84	2.52	3.39	0.00	0.00	0.00	1.84	2.52	3.39	1.49E-03	1.86E-03	2.26E-03	2.77E-03	3.79E-03	5.10E-03	0.00	0.00	0.00	2.77E-03	3.79E-03	5.10E-03	1,107.24	1,367.76	1,655.88	1.67	2.06	2.49	0.00	0.00	0.00	0.00	0.00	0.00
France - Streptococcus pneumoniae PMRSP BSI - model 76	1.83	2.19	2.59	13.60	14.78	16.02	15.75	16.99	18.20	3.52	3.80	4.07	2.75E-03	3.29E-03	3.89E-03	0.02	0.02	0.02	0.02	0.03	0.03	4.47	4.47	4.47	6.72E-03	6.72E-03	6.72E-03	0.74	0.81	0.87	1.12E-03	1.21E-03	1.31E-03
France - Streptococcus pneumoniae PMRSP OTH - model 80	1.77E-03	3.08E-03	4.74E-03	0.00	0.00	0.00	1.77E-03	3.08E-03	4.74E-03	1.15E-03	1.95E-03	2.88E-03	2.67E-06	4.63E-06	7.13E-06	0.00	0.00	0.00	2.67E-06	4.63E-06	7.13E-06	1.39	1.58	1.83	2.09E-03	2.38E-03	2.75E-03	0.00	0.00	0.00	0.00	0.00	0.00
France - Streptococcus pneumoniae PMRSP RESP - model 77	4.45	5.71	7.91	8.54	11.08	15.24	13.18	16.76	22.57	0.91	1.00	1.12	6.69E-03	8.59E-03	0.01	0.01	0.02	0.02	0.02	0.03	0.03	13.50	16.75	21.87	0.02	0.03	0.03	0.47	0.61	0.81	7.05E-04	9.13E-04	1.22E-03
France - Streptococcus pneumoniae PMRSP SSI - model 79	0.00	0.00	0.00	0.00	0.00	0.00	0.00	0.00	0.00	NaN	NaN	NaN	0.00	0.00	0.00	0.00	0.00	0.00	0.00	0.00	0.00	0.00	0.00	0.00	0.00	0.00	0.00	0.00	0.00	0.00	0.00	0.00	0.00
France - Streptococcus pneumoniae PMRSP UTI - model 78	0.00	0.00	0.00	0.00	0.00	0.00	0.00	0.00	0.00	NaN	NaN	NaN	0.00	0.00	0.00	0.00	0.00	0.00	0.00	0.00	0.00	0.00	0.00	0.00	0.00	0.00	0.00	0.00	0.00	0.00	0.00	0.00	0.00
France - Streptococcus pneumoniae PRSP BSI - model 71	0.00	0.00	0.00	0.00	0.00	0.00	0.00	0.00	0.00	NaN	NaN	NaN	0.00	0.00	0.00	0.00	0.00	0.00	0.00	0.00	0.00	0.00	0.00	0.00	0.00	0.00	0.00	0.00	0.00	0.00	0.00	0.00	0.00
France - Streptococcus pneumoniae PRSP OTH - model 75	0.00	0.00	0.00	0.00	0.00	0.00	0.00	0.00	0.00	NaN	NaN	NaN	0.00	0.00	0.00	0.00	0.00	0.00	0.00	0.00	0.00	0.00	0.00	0.00	0.00	0.00	0.00	0.00	0.00	0.00	0.00	0.00	0.00
France - Streptococcus pneumoniae PRSP RESP - model 72	0.00	0.00	0.00	0.00	0.00	0.00	0.00	0.00	0.00	NaN	NaN	NaN	0.00	0.00	0.00	0.00	0.00	0.00	0.00	0.00	0.00	0.00	0.00	0.00	0.00	0.00	0.00	0.00	0.00	0.00	0.00	0.00	0.00
France - Streptococcus pneumoniae PRSP SSI - model 74	0.00	0.00	0.00	0.00	0.00	0.00	0.00	0.00	0.00	NaN	NaN	NaN	0.00	0.00	0.00	0.00	0.00	0.00	0.00	0.00	0.00	0.00	0.00	0.00	0.00	0.00	0.00	0.00	0.00	0.00	0.00	0.00	0.00
France - Streptococcus pneumoniae PRSP UTI - model 73	0.00	0.00	0.00	0.00	0.00	0.00	0.00	0.00	0.00	NaN	NaN	NaN	0.00	0.00	0.00	0.00	0.00	0.00	0.00	0.00	0.00	0.00	0.00	0.00	0.00	0.00	0.00	0.00	0.00	0.00	0.00	0.00	0.00
**Total (Median & 95% UIs)**	20,580.20	23,497.54	26,799.68	106,754.80	123,245.73	142,578.41	127,986.35	146,710.40	168,446.37		-		30.95	35.34	40.31	160.56	185.36	214.44	192.49	220.66	253.35	107,522.80	124,805.54	142,930.63	161.72	187.71	214.97	4,743.59	5,543.20	6,457.89	7.13	8.34	9.71
**Total (average)**		**23,539.73**			**123,505.38**			**147,045.12**			-			**35.40**			**185.76**			**221.16**			**125,011.54**			**188.02**			**5,551.56**			**8.35**	

Infection	YLD			YLL			DALY			DALY per Case			YLD per 100,000		
Colistin resistant *Acinetobacter spp.*	0.00	0.00	0.00	0.00	0.00	0.00	0.00	0.00	0.00	0.00	0.00	0.00	0.00	0.00	0.00
Carbapenem resistant *Acinetobacter spp.* (excluding those resistant to colistin)	233.98	261.48	288.46	1,151.96	1,401.48	1,635.75	1,401.00	1,663.77	1,911.05	7.08	8.17	9.25	5.54	6.19	6.83
Aminoglycosides and fluoroquinolones resistant *Acinetobacter* spp. (excluding those resistant to colistin and/or carbapenem)	2.27	3.00	3.85	9.09	15.87	22.73	11.64	18.85	26.29	3.88	5.66	7.38	0.05	0.07	0.09
Vancomycin resistant Enterococci (*E. faecalis* and *E. faecium*)	20.42	25.56	32.03	191.53	237.84	293.31	211.88	263.28	324.35	6.34	7.00	7.71	0.48	0.60	0.76
Colistin resistant *Escherichia coli*	0.00	0.00	0.00	0.00	0.00	0.00	0.00	0.00	0.00	0.00	0.00	0.00	0.00	0.00	0.00
Carbapenem resistant *Escherichia coli* (excluding those resistant to colistin)	0.00	0.00	0.00	0.00	0.00	0.00	0.00	0.00	0.00	0.00	0.00	0.00	0.00	0.00	0.00
Third-generation cephalosporin resistant *Escherichia coli* (excluding those resistant to colistin and/or carbapenem)	105.10	118.23	133.11	619.33	697.55	785.36	728.10	816.70	917.22	5.39	5.93	6.51	2.49	2.80	3.15
Colistin resistant *Klebsiella pneumoniae*	0.69	1.22	1.98	10.72	21.83	36.83	11.49	23.03	38.67	12.49	20.73	29.14	0.02	0.03	0.05
Carbapenem resistant *Klebsiella pneumoniae* (excluding those resistant to colistin)	7.21	9.06	11.06	75.66	97.20	122.35	83.90	106.36	132.65	10.25	12.51	14.87	0.17	0.21	0.26
Third-generation cephalosporin resistant *Klebsiella pneumoniae* (excluding those resistant to colistin and/or carbapenem)	245.25	271.61	299.42	1,202.87	1,299.04	1,397.17	1,454.22	1,571.00	1,688.08	8.21	8.66	9.13	5.80	6.43	7.09
Colistin resistant *Pseudomonas aeruginosa*	0.00	0.00	0.00	0.00	0.00	0.00	0.00	0.00	0.00	0.00	0.00	0.00	0.00	0.00	0.00
Carbapenem resistant *Pseudomonas aeruginosa* (excluding those resistant to colistin)	310.30	353.02	395.85	1,447.00	1,783.90	2,153.34	1,773.64	2,137.48	2,539.59	9.39	10.98	12.68	7.34	8.35	9.37
Pseudomonas aeruginosa resistant to three or more antimicrobial groups (excluding those resistant to colistin and/or carbapenem)	14.68	20.88	27.55	57.57	97.63	149.89	74.57	118.31	175.34	6.80	10.07	13.43	0.35	0.49	0.65
Methicillin resistant *Staphylococcus aureus*	149.11	165.46	182.16	734.02	792.88	855.17	886.47	957.83	1.032.02	5.88	6.15	6.45	3.53	3.92	4.31
Penicillin and macrolides resistant *Streptococcus pneumoniae* (excluding those only resistant to penicillin)	5.78	8.58	13.68	22.87	28.84	39.61	29.65	37.25	52.15	19.20	21.83	24.53	0.14	0.20	0.32
Penicillin tesistant *Streptococcus pneumoniae* (excluding those resistant to macrolides)	0.00	0.00	0.00	0.00	0.00	0.00	0.00	0.00	0.00	0.00	0.00	0.00	0.00	0.00	0.00
Total	1,094.78	1,238.10	1,389.15	5,522.62	6,474.07	7,491.50	6,666.56	7,713.86	8,837.41				25.91	29.30	32.88

Infection	YLL per 100,000			DALY per 100,000			Cases per year			Incidence per 100,000			Deaths per year			Mortality per 100,000		
Colistin resistant *Acinetobacter spp.*	0.00	0.00	0.00	0.00	0.00	0.00	0.00	0.00	0.00	0.00	0.00	0.00	0.00	0.00	0.00	0.00	0.00	0.00
Carbapenem resistant *Acinetobacter spp.* (excluding those resistant to colistin)	27.26	33.17	38.71	33.16	39.38	45.23	534.10	600.12	668.28	12.64	14.20	15.82	42.80	52.48	62.06	1.01	1.24	1.47
Aminoglycosides and fluoroquinolones resistant *Acinetobacter* spp. (excluding those resistant to colistin and/or carbapenem)	0.22	0.38	0.54	0.28	0.45	0.62	7.35	10.05	12.84	0.17	0.24	0.30	0.51	0.87	1.23	0.01	0.02	0.03
Vancomycin resistant Enterococci (*E. faecalis* and *E. faecium*)	4.53	5.63	6.94	5.01	6.23	7.68	113.41	145.38	179.36	2.68	3.44	4.24	7.65	9.50	11.78	0.18	0.22	0.28
Colistin resistant *Escherichia coli*	0.00	0.00	0.00	0.00	0.00	0.00	0.00	0.00	0.00	0.00	0.00	0.00	0.00	0.00	0.00	0.00	0.00	0.00
Carbapenem resistant *Escherichia coli* (excluding those resistant to colistin)	0.00	0.00	0.00	0.00	0.00	0.00	0.00	0.00	0.00	0.00	0.00	0.00	0.00	0.00	0.00	0.00	0.00	0.00
Third-generation cephalosporin resistant *Escherichia coli* (excluding those resistant to colistin and/or carbapenem)	14.66	16.51	18.59	17.23	19.33	21.71	969.54	1,103.25	1,240.19	22.95	26.11	29.35	29.26	33.69	38.32	0.69	0.80	0.91
Colistin resistant *Klebsiella pneumoniae*	0.25	0.52	0.87	0.27	0.55	0.92	1.47	3.28	5.29	0.03	0.08	0.13	0.36	0.73	1.23	0.01	0.02	0.03
Carbapenem resistant *Klebsiella pneumoniae* (excluding those resistant to colistin)	1.79	2.30	2.90	1.99	2.52	3.14	20.32	26.52	32.77	0.48	0.63	0.78	2.77	3.56	4.48	0.07	0.08	0.11
Third-generation cephalosporin resistant *Klebsiella pneumoniae* (excluding those resistant to colistin and/or carbapenem)	28.47	30.74	33.07	34.42	37.18	39.95	676.61	740.55	804.46	16.01	17.53	19.04	36.95	39.80	42.53	0.87	0.94	1.01
Colistin resistant *Pseudomonas aeruginosa*	0.00	0.00	0.00	0.00	0.00	0.00	0.00	0.00	0.00	0.00	0.00	0.00	0.00	0.00	0.00	0.00	0.00	0.00
Carbapenem resistant *Pseudomonas aeruginosa* (excluding those resistant to colistin)	34.25	42.22	50.96	41.98	50.59	60.10	750.78	860.21	969.10	17.77	20.36	22.94	46.87	57.94	70.40	1.11	1.37	1.67
Pseudomonas aeruginosa resistant to three or more antimicrobial groups (excluding those resistant to colistin and/or carbapenem)	1.36	2.31	3.55	1.76	2.80	4.15	36.94	51.93	67.43	0.87	1.23	1.60	1.98	3.25	4.84	0.05	0.08	0.11
Methicillin resistant *Staphylococcus aureus*	17.37	18.76	20.24	20.98	22.67	24.42	723.30	800.68	878.41	17.12	18.95	20.79	34.40	37.58	41.10	0.81	0.89	0.97
Penicillin and macrolides resistant *Streptococcus pneumoniae* (excluding those only resistant to penicillin)	0.54	0.68	0.94	0.70	0.88	1.23	4.31	5.50	8.42	0.10	0.13	0.20	0.27	0.34	0.47	0.01	0.01	0.01
Penicillin tesistant *Streptococcus pneumoniae* (excluding those resistant to macrolides)	0.00	0.00	0.00	0.00	0.00	0.00	0.00	0.00	0.00	0.00	0.00	0.00	0.00	0.00	0.00	0.00	0.00	0.00
Total	130.70	153.22	177.30	157.78	182.56	209.15	3,838.12	4,347.46	4,866.55	90.84	102.89	115.18	203.82	239.74	278.43	4.82	5.67	6.59

Model	YLD			YLL			DALY			DALY per Case			YLD per 100,000		
Croatia - Acinetobacter spp. ColRACI BSI - model 41	0.00	0.00	0.00	0.00	0.00	0.00	0.00	0.00	0.00	NaN	NaN	NaN	0.00	0.00	0.00
Croatia - Acinetobacter spp. ColRACI OTH - model 45	0.00	0.00	0.00	0.00	0.00	0.00	0.00	0.00	0.00	NaN	NaN	NaN	0.00	0.00	0.00
Croatia - Acinetobacter spp. ColRACI RESP - model 42	0.00	0.00	0.00	0.00	0.00	0.00	0.00	0.00	0.00	NaN	NaN	NaN	0.00	0.00	0.00
Croatia - Acinetobacter spp. ColRACI SSI - model 44	0.00	0.00	0.00	0.00	0.00	0.00	0.00	0.00	0.00	NaN	NaN	NaN	0.00	0.00	0.00
Croatia - Acinetobacter spp. ColRACI UTI - model 43	0.00	0.00	0.00	0.00	0.00	0.00	0.00	0.00	0.00	NaN	NaN	NaN	0.00	0.00	0.00
Croatia - Acinetobacter spp. CRACI BSI - model 36	135.00	148.94	162.47	931.65	1,150.62	1,354.40	1,079.92	1,300.59	1,505.01	5.29	6.27	7.22	3.20	3.52	3.85
Croatia - Acinetobacter spp. CRACI OTH - model 40	0.02	0.03	0.04	0.00	0.00	0.00	0.02	0.03	0.04	3.04E-03	3.72E-03	4.43E-03	4.69E-04	6.57E-04	8.92E-04
Croatia - Acinetobacter spp. CRACI RESP - model 37	98.70	112.18	125.52	193.46	218.39	243.56	293.95	330.35	367.76	1.39	1.47	1.56	2.34	2.65	2.97
Croatia - Acinetobacter spp. CRACI SSI - model 39	0.12	0.16	0.22	26.85	32.47	37.79	26.97	32.63	38.03	0.40	0.43	0.46	2.84E-03	3.80E-03	5.11E-03
Croatia - Acinetobacter spp. CRACI UTI - model 38	0.13	0.17	0.22	0.00	0.00	0.00	0.13	0.17	0.22	1.66E-03	2.03E-03	2.43E-03	3.14E-03	4.07E-03	5.15E-03
Croatia - Acinetobacter spp. MDRACI BSI - model 31	1.36	1.70	2.13	6.99	12.85	18.62	8.62	14.52	20.56	2.61	4.22	5.74	0.03	0.04	0.05
Croatia - Acinetobacter spp. MDRACI OTH - model 35	1.70E-04	4.56E-04	8.67E-04	0.00	0.00	0.00	1.70E-04	4.56E-04	8.67E-04	2.13E-03	3.74E-03	5.48E-03	4.01E-06	1.08E-05	2.05E-05
Croatia - Acinetobacter spp. MDRACI RESP - model 32	0.91	1.30	1.70	1.76	2.47	3.32	2.68	3.78	4.93	0.89	1.00	1.14	0.02	0.03	0.04
Croatia - Acinetobacter spp. MDRACI SSI - model 34	1.09E-03	2.70E-03	4.89E-03	0.34	0.55	0.79	0.34	0.55	0.79	0.37	0.43	0.50	2.59E-05	6.39E-05	1.16E-04
Croatia - Acinetobacter spp. MDRACI UTI - model 33	1.45E-03	2.81E-03	4.90E-03	0.00	0.00	0.00	1.45E-03	2.81E-03	4.90E-03	1.23E-03	2.02E-03	3.05E-03	3.44E-05	6.65E-05	1.16E-04
Croatia - Enterococcus faecalis and E. faecium VRE BSI - model 66	20.28	25.35	31.74	176.28	217.61	267.67	196.48	242.84	298.54	5.98	6.59	7.26	0.48	0.60	0.75
Croatia - Enterococcus faecalis and E. faecium VRE OTH - model 70	0.04	0.06	0.09	0.00	0.00	0.00	0.04	0.06	0.09	1.25E-03	1.66E-03	2.08E-03	1.05E-03	1.52E-03	2.13E-03
Croatia - Enterococcus faecalis and E. faecium VRE RESP - model 67	0.00	0.00	0.00	0.00	0.00	0.00	0.00	0.00	0.00	NaN	NaN	NaN	0.00	0.00	0.00
Croatia - Enterococcus faecalis and E. faecium VRE SSI - model 69	0.07	0.11	0.15	15.25	20.23	25.64	15.33	20.34	25.67	0.36	0.40	0.45	1.71E-03	2.53E-03	3.56E-03
Croatia - Enterococcus faecalis and E. faecium VRE UTI - model 68	0.02	0.03	0.05	0.00	0.00	0.00	0.02	0.03	0.05	1.44E-03	1.88E-03	2.38E-03	5.80E-04	8.24E-04	1.16E-03
Croatia - Escherichia coli 3GCREC BSI - model 16	80.44	88.98	98.54	515.78	576.40	645.15	599.60	666.21	742.36	3.94	4.33	4.71	1.90	2.11	2.33
Croatia - Escherichia coli 3GCREC OTH - model 20	0.12	0.17	0.22	0.00	0.00	0.00	0.12	0.17	0.22	2.64E-03	3.33E-03	4.04E-03	2.93E-03	4.03E-03	5.21E-03
Croatia - Escherichia coli 3GCREC RESP - model 17	23.26	27.44	32.25	44.93	53.22	62.64	68.35	80.74	94.88	1.05	1.18	1.34	0.55	0.65	0.76
Croatia - Escherichia coli 3GCREC SSI - model 19	0.26	0.35	0.47	58.62	67.94	77.57	59.00	68.28	78.14	0.39	0.42	0.44	6.24E-03	8.24E-03	0.01
Croatia - Escherichia coli 3GCREC UTI - model 18	1.02	1.30	1.62	0.00	0.00	0.00	1.02	1.30	1.62	1.59E-03	1.96E-03	2.33E-03	0.02	0.03	0.04
Croatia - Escherichia coli ColREC BSI - model 26	0.00	0.00	0.00	0.00	0.00	0.00	0.00	0.00	0.00	NaN	NaN	NaN	0.00	0.00	0.00
Croatia - Escherichia coli ColREC OTH - model 30	0.00	0.00	0.00	0.00	0.00	0.00	0.00	0.00	0.00	NaN	NaN	NaN	0.00	0.00	0.00
Croatia - Escherichia coli ColREC RESP - model 27	0.00	0.00	0.00	0.00	0.00	0.00	0.00	0.00	0.00	NaN	NaN	NaN	0.00	0.00	0.00
Croatia - Escherichia coli ColREC SSI - model 29	0.00	0.00	0.00	0.00	0.00	0.00	0.00	0.00	0.00	NaN	NaN	NaN	0.00	0.00	0.00
Croatia - Escherichia coli ColREC UTI - model 28	0.00	0.00	0.00	0.00	0.00	0.00	0.00	0.00	0.00	NaN	NaN	NaN	0.00	0.00	0.00
Croatia - Escherichia coli CREC BSI - model 21	0.00	0.00	0.00	0.00	0.00	0.00	0.00	0.00	0.00	NaN	NaN	NaN	0.00	0.00	0.00
Croatia - Escherichia coli CREC OTH - model 25	0.00	0.00	0.00	0.00	0.00	0.00	0.00	0.00	0.00	NaN	NaN	NaN	0.00	0.00	0.00
Croatia - Escherichia coli CREC RESP - model 22	0.00	0.00	0.00	0.00	0.00	0.00	0.00	0.00	0.00	NaN	NaN	NaN	0.00	0.00	0.00
Croatia - Escherichia coli CREC SSI - model 24	0.00	0.00	0.00	0.00	0.00	0.00	0.00	0.00	0.00	NaN	NaN	NaN	0.00	0.00	0.00
Croatia - Escherichia coli CREC UTI - model 23	0.00	0.00	0.00	0.00	0.00	0.00	0.00	0.00	0.00	NaN	NaN	NaN	0.00	0.00	0.00
Croatia - Klebsiella pneumoniae 3GCRKP BSI - model 1	154.24	169.15	184.43	995.63	1,066.34	1,137.54	1,156.21	1,235.59	1,315.52	6.11	6.41	6.71	3.65	4.00	4.36
Croatia - Klebsiella pneumoniae 3GCRKP RESP - model 2	90.31	101.59	113.94	177.06	198.58	221.37	267.05	300.42	333.29	1.67	1.80	1.94	2.14	2.40	2.70
Croatia - Klebsiella pneumoniae 3GCRKP UTI - model 3	0.37	0.44	0.53	0.00	0.00	0.00	0.37	0.44	0.53	1.65E-03	1.96E-03	2.28E-03	8.68E-03	0.01	0.01
Croatia - Klebsiella pneumoniae 3GCRKP OTH - model 5	0.21	0.26	0.31	0.00	0.00	0.00	0.21	0.26	0.31	2.81E-03	3.35E-03	3.91E-03	4.91E-03	6.07E-03	7.42E-03
Croatia - Klebsiella pneumoniae 3GCRKP SSI - model 4	0.13	0.16	0.21	30.18	34.12	38.26	30.38	34.29	38.43	0.43	0.45	0.47	2.98E-03	3.86E-03	4.93E-03
Croatia - Klebsiella pneumoniae ColRKP BSI - model 11	0.62	0.94	1.44	10.56	21.18	35.67	11.26	22.10	36.98	10.82	18.81	26.98	0.01	0.02	0.03
Croatia - Klebsiella pneumoniae ColRKP OTH - model 15	3.92E-04	1.63E-03	4.87E-03	0.00	0.00	0.00	3.92E-04	1.63E-03	4.87E-03	1.24E-03	3.24E-03	7.10E-03	9.27E-06	3.86E-05	1.15E-04
Croatia - Klebsiella pneumoniae ColRKP RESP - model 12	0.07	0.28	0.53	0.14	0.55	1.00	0.21	0.84	1.51	1.40	1.64	1.88	1.68E-03	6.62E-03	0.01
Croatia - Klebsiella pneumoniae ColRKP SSI - model 14	1.12E-04	6.33E-04	1.98E-03	0.03	0.09	0.17	0.03	0.09	0.17	0.27	0.27	0.27	2.65E-06	1.50E-05	4.70E-05
Croatia - Klebsiella pneumoniae ColRKP UTI - model 13	3.23E-04	1.29E-03	3.27E-03	0.00	0.00	0.00	3.23E-04	1.29E-03	3.27E-03	7.62E-04	1.95E-03	4.02E-03	7.64E-06	3.04E-05	7.75E-05
Croatia - Klebsiella pneumoniae CRKP BSI - model 6	5.80	6.99	8.27	71.97	91.79	115.12	78.79	98.86	122.70	8.48	10.49	12.57	0.14	0.17	0.20
Croatia - Klebsiella pneumoniae CRKP OTH - model 10	8.88E-03	0.02	0.02	0.00	0.00	0.00	8.88E-03	0.02	0.02	2.32E-03	3.51E-03	5.05E-03	2.10E-04	3.65E-04	5.88E-04
Croatia - Klebsiella pneumoniae CRKP RESP - model 7	1.39	2.03	2.73	2.78	3.96	5.24	4.17	6.01	7.92	1.32	1.49	1.70	0.03	0.05	0.06
Croatia - Klebsiella pneumoniae CRKP SSI - model 9	3.27E-03	5.76E-03	9.40E-03	0.92	1.45	1.99	0.93	1.46	1.99	0.44	0.52	0.59	7.74E-05	1.36E-04	2.22E-04
Croatia - Klebsiella pneumoniae CRKP UTI - model 8	7.37E-03	0.01	0.02	0.00	0.00	0.00	7.37E-03	0.01	0.02	1.39E-03	2.10E-03	2.90E-03	1.75E-04	2.80E-04	4.33E-04
Croatia - Pseudomonas aeruginosa ColRPA BSI - model 56	0.00	0.00	0.00	0.00	0.00	0.00	0.00	0.00	0.00	NaN	NaN	NaN	0.00	0.00	0.00
Croatia - Pseudomonas aeruginosa ColRPA OTH - model 60	0.00	0.00	0.00	0.00	0.00	0.00	0.00	0.00	0.00	NaN	NaN	NaN	0.00	0.00	0.00
Croatia - Pseudomonas aeruginosa ColRPA RESP - model 57	0.00	0.00	0.00	0.00	0.00	0.00	0.00	0.00	0.00	NaN	NaN	NaN	0.00	0.00	0.00
Croatia - Pseudomonas aeruginosa ColRPA SSI - model 59	0.00	0.00	0.00	0.00	0.00	0.00	0.00	0.00	0.00	NaN	NaN	NaN	0.00	0.00	0.00
Croatia - Pseudomonas aeruginosa ColRPA UTI - model 58	0.00	0.00	0.00	0.00	0.00	0.00	0.00	0.00	0.00	NaN	NaN	NaN	0.00	0.00	0.00
Croatia - Pseudomonas aeruginosa CRPA BSI - model 51	88.11	99.18	110.23	685.69	849.43	1,020.77	782.55	949.29	1,130.63	6.67	7.93	9.24	2.09	2.35	2.61
Croatia - Pseudomonas aeruginosa CRPA OTH - model 55	0.17	0.22	0.28	0.00	0.00	0.00	0.17	0.22	0.28	3.02E-03	3.66E-03	4.25E-03	3.98E-03	5.26E-03	6.61E-03
Croatia - Pseudomonas aeruginosa CRPA RESP - model 52	221.64	253.12	284.71	731.07	898.91	1,091.06	960.27	1,151.90	1,366.58	2.31	2.61	2.97	5.25	5.99	6.74
Croatia - Pseudomonas aeruginosa CRPA SSI - model 54	0.13	0.18	0.23	30.24	35.56	41.51	30.40	35.75	41.70	0.40	0.43	0.47	3.10E-03	4.16E-03	5.48E-03
Croatia - Pseudomonas aeruginosa CRPA UTI - model 53	0.25	0.32	0.40	0.00	0.00	0.00	0.25	0.32	0.40	1.70E-03	2.06E-03	2.44E-03	5.94E-03	7.46E-03	9.47E-03
Croatia - Pseudomonas aeruginosa MDRPA BSI - model 46	4.37	5.83	7.65	26.74	46.15	71.19	31.70	51.93	77.67	4.72	7.29	9.90	0.10	0.14	0.18
Croatia - Pseudomonas aeruginosa MDRPA OTH - model 50	6.80E-03	0.01	0.02	0.00	0.00	0.00	6.80E-03	0.01	0.02	2.44E-03	3.72E-03	5.24E-03	1.61E-04	3.25E-04	5.19E-04
Croatia - Pseudomonas aeruginosa MDRPA RESP - model 47	10.29	15.01	19.83	29.82	49.83	76.40	41.83	64.68	95.31	1.77	2.44	3.16	0.24	0.36	0.47
Croatia - Pseudomonas aeruginosa MDRPA SSI - model 49	4.76E-03	0.01	0.02	1.01	1.65	2.29	1.02	1.67	2.32	0.31	0.33	0.36	1.13E-04	2.46E-04	4.50E-04
Croatia - Pseudomonas aeruginosa MDRPA UTI - model 48	0.01	0.02	0.03	0.00	0.00	0.00	0.01	0.02	0.03	1.26E-03	2.01E-03	2.89E-03	2.43E-04	4.36E-04	7.12E-04
Croatia - Staphylococcus aureus MRSA BSI - model 61	73.49	80.16	87.51	505.16	538.81	574.76	581.32	618.43	658.39	4.35	4.54	4.74	1.74	1.90	2.07
Croatia - Staphylococcus aureus MRSA OTH - model 65	0.52	0.66	0.81	0.00	0.00	0.00	0.52	0.66	0.81	2.62E-03	3.25E-03	3.98E-03	0.01	0.02	0.02
Croatia - Staphylococcus aureus MRSA RESP - model 62	74.69	84.10	93.15	146.90	163.01	180.21	222.30	247.16	272.06	1.13	1.20	1.28	1.77	1.99	2.20
Croatia - Staphylococcus aureus MRSA SSI - model 64	0.36	0.48	0.61	81.96	91.06	100.19	82.28	91.53	100.67	0.39	0.41	0.42	8.62E-03	0.01	0.01
Croatia - Staphylococcus aureus MRSA UTI - model 63	0.05	0.06	0.08	0.00	0.00	0.00	0.05	0.06	0.08	1.53E-03	1.88E-03	2.29E-03	1.14E-03	1.48E-03	1.92E-03
Croatia - Streptococcus pneumoniae PMRSP BSI - model 76	1.76	2.43	3.23	14.59	16.69	18.97	16.90	19.12	21.69	15.35	17.30	19.30	0.04	0.06	0.08
Croatia - Streptococcus pneumoniae PMRSP OTH - model 80	2.86E-04	7.36E-04	1.53E-03	0.00	0.00	0.00	2.86E-04	7.36E-04	1.53E-03	7.76E-04	1.85E-03	3.51E-03	6.77E-06	1.74E-05	3.61E-05
Croatia - Streptococcus pneumoniae PMRSP RESP - model 77	4.02	6.15	10.45	8.27	12.15	20.63	12.75	18.13	30.47	3.85	4.53	5.23	0.10	0.15	0.25
Croatia - Streptococcus pneumoniae PMRSP SSI - model 79	0.00	0.00	0.00	0.00	0.00	0.00	0.00	0.00	0.00	NaN	NaN	NaN	0.00	0.00	0.00
Croatia - Streptococcus pneumoniae PMRSP UTI - model 78	0.00	0.00	0.00	0.00	0.00	0.00	0.00	0.00	0.00	NaN	NaN	NaN	0.00	0.00	0.00
Croatia - Streptococcus pneumoniae PRSP BSI - model 71	0.00	0.00	0.00	0.00	0.00	0.00	0.00	0.00	0.00	NaN	NaN	NaN	0.00	0.00	0.00
Croatia - Streptococcus pneumoniae PRSP OTH - model 75	0.00	0.00	0.00	0.00	0.00	0.00	0.00	0.00	0.00	NaN	NaN	NaN	0.00	0.00	0.00
Croatia - Streptococcus pneumoniae PRSP RESP - model 72	0.00	0.00	0.00	0.00	0.00	0.00	0.00	0.00	0.00	NaN	NaN	NaN	0.00	0.00	0.00
Croatia - Streptococcus pneumoniae PRSP SSI - model 74	0.00	0.00	0.00	0.00	0.00	0.00	0.00	0.00	0.00	NaN	NaN	NaN	0.00	0.00	0.00
Croatia - Streptococcus pneumoniae PRSP UTI - model 73	0.00	0.00	0.00	0.00	0.00	0.00	0.00	0.00	0.00	NaN	NaN	NaN	0.00	0.00	0.00
**Total (Median & 95% UIs)**	1,094.78	1,238.10	1,389.15	5,522.62	6,474.07	7,491.50	6,666.56	7,713.86	8,837.41		-		25.91	29.30	32.88
**Total (average)**		1,239.78			6,484.89			7,724.67			-			29.34	

Model	YLL per 100,000			DALY per 100,000			Cases per year			Incidence per 100,000			Deaths per year			Mortality per 100,000		
Croatia - Acinetobacter spp. ColRACI BSI - model 41	0.00	0.00	0.00	0.00	0.00	0.00	0.00	0.00	0.00	0.00	0.00	0.00	0.00	0.00	0.00	0.00	0.00	0.00
Croatia - Acinetobacter spp. ColRACI OTH - model 45	0.00	0.00	0.00	0.00	0.00	0.00	0.00	0.00	0.00	0.00	0.00	0.00	0.00	0.00	0.00	0.00	0.00	0.00
Croatia - Acinetobacter spp. ColRACI RESP - model 42	0.00	0.00	0.00	0.00	0.00	0.00	0.00	0.00	0.00	0.00	0.00	0.00	0.00	0.00	0.00	0.00	0.00	0.00
Croatia - Acinetobacter spp. ColRACI SSI - model 44	0.00	0.00	0.00	0.00	0.00	0.00	0.00	0.00	0.00	0.00	0.00	0.00	0.00	0.00	0.00	0.00	0.00	0.00
Croatia - Acinetobacter spp. ColRACI UTI - model 43	0.00	0.00	0.00	0.00	0.00	0.00	0.00	0.00	0.00	0.00	0.00	0.00	0.00	0.00	0.00	0.00	0.00	0.00
Croatia - Acinetobacter spp. CRACI BSI - model 36	22.05	27.23	32.05	25.56	30.78	35.62	193.06	206.99	221.77	4.57	4.90	5.25	34.35	42.75	50.97	0.81	1.01	1.21
Croatia - Acinetobacter spp. CRACI OTH - model 40	0.00	0.00	0.00	4.69E-04	6.57E-04	8.92E-04	5.74	7.49	9.30	0.14	0.18	0.22	0.00	0.00	0.00	0.00	0.00	0.00
Croatia - Acinetobacter spp. CRACI RESP - model 37	4.58	5.17	5.76	6.96	7.82	8.70	199.58	224.45	250.98	4.72	5.31	5.94	7.11	8.08	9.14	0.17	0.19	0.22
Croatia - Acinetobacter spp. CRACI SSI - model 39	0.64	0.77	0.89	0.64	0.77	0.90	64.65	75.96	88.35	1.53	1.80	2.09	1.34	1.65	1.94	0.03	0.04	0.05
Croatia - Acinetobacter spp. CRACI UTI - model 38	0.00	0.00	0.00	3.14E-03	4.07E-03	5.15E-03	71.07	85.22	97.87	1.68	2.02	2.32	0.00	0.00	0.00	0.00	0.00	0.00
Croatia - Acinetobacter spp. MDRACI BSI - model 31	0.17	0.30	0.44	0.20	0.34	0.49	2.88	3.46	4.07	0.07	0.08	0.10	0.39	0.70	1.00	9.20E-03	0.02	0.02
Croatia - Acinetobacter spp. MDRACI OTH - model 35	0.00	0.00	0.00	4.01E-06	1.08E-05	2.05E-05	0.06	0.12	0.21	1.33E-03	2.94E-03	4.87E-03	0.00	0.00	0.00	0.00	0.00	0.00
Croatia - Acinetobacter spp. MDRACI RESP - model 32	0.04	0.06	0.08	0.06	0.09	0.12	2.75	3.78	4.80	0.07	0.09	0.11	0.10	0.14	0.18	2.31E-03	3.20E-03	4.20E-03
Croatia - Acinetobacter spp. MDRACI SSI - model 34	7.99E-03	0.01	0.02	8.02E-03	0.01	0.02	0.79	1.28	1.79	0.02	0.03	0.04	0.02	0.03	0.05	4.69E-04	8.20E-04	1.17E-03
Croatia - Acinetobacter spp. MDRACI UTI - model 33	0.00	0.00	0.00	3.44E-05	6.65E-05	1.16E-04	0.86	1.41	1.97	0.02	0.03	0.05	0.00	0.00	0.00	0.00	0.00	0.00
Croatia - Enterococcus faecalis and E. faecium VRE BSI - model 66	4.17	5.15	6.33	4.65	5.75	7.07	30.01	36.85	45.38	0.71	0.87	1.07	6.86	8.42	10.39	0.16	0.20	0.25
Croatia - Enterococcus faecalis and E. faecium VRE OTH - model 70	0.00	0.00	0.00	1.05E-03	1.52E-03	2.13E-03	30.16	39.33	48.33	0.71	0.93	1.14	0.00	0.00	0.00	0.00	0.00	0.00
Croatia - Enterococcus faecalis and E. faecium VRE RESP - model 67	0.00	0.00	0.00	0.00	0.00	0.00	0.00	0.00	0.00	0.00	0.00	0.00	0.00	0.00	0.00	0.00	0.00	0.00
Croatia - Enterococcus faecalis and E. faecium VRE SSI - model 69	0.36	0.48	0.61	0.36	0.48	0.61	38.89	50.55	62.28	0.92	1.20	1.47	0.79	1.09	1.38	0.02	0.03	0.03
Croatia - Enterococcus faecalis and E. faecium VRE UTI - model 68	0.00	0.00	0.00	5.80E-04	8.24E-04	1.16E-03	14.35	18.64	23.37	0.34	0.44	0.55	0.00	0.00	0.00	0.00	0.00	0.00
Croatia - Escherichia coli 3GCREC BSI - model 16	12.21	13.64	15.27	14.19	15.77	17.57	142.20	153.97	166.33	3.37	3.64	3.94	23.47	26.73	30.10	0.56	0.63	0.71
Croatia - Escherichia coli 3GCREC OTH - model 20	0.00	0.00	0.00	2.93E-03	4.03E-03	5.21E-03	41.80	51.13	61.82	0.99	1.21	1.46	0.00	0.00	0.00	0.00	0.00	0.00
Croatia - Escherichia coli 3GCREC RESP - model 17	1.06	1.26	1.48	1.62	1.91	2.25	56.51	68.60	81.26	1.34	1.62	1.92	2.01	2.47	2.96	0.05	0.06	0.07
Croatia - Escherichia coli 3GCREC SSI - model 19	1.39	1.61	1.84	1.40	1.62	1.85	141.64	163.06	186.32	3.35	3.86	4.41	3.78	4.48	5.26	0.09	0.11	0.12
Croatia - Escherichia coli 3GCREC UTI - model 18	0.00	0.00	0.00	0.02	0.03	0.04	587.39	666.49	744.46	13.90	15.77	17.62	0.00	0.00	0.00	0.00	0.00	0.00
Croatia - Escherichia coli ColREC BSI - model 26	0.00	0.00	0.00	0.00	0.00	0.00	0.00	0.00	0.00	0.00	0.00	0.00	0.00	0.00	0.00	0.00	0.00	0.00
Croatia - Escherichia coli ColREC OTH - model 30	0.00	0.00	0.00	0.00	0.00	0.00	0.00	0.00	0.00	0.00	0.00	0.00	0.00	0.00	0.00	0.00	0.00	0.00
Croatia - Escherichia coli ColREC RESP - model 27	0.00	0.00	0.00	0.00	0.00	0.00	0.00	0.00	0.00	0.00	0.00	0.00	0.00	0.00	0.00	0.00	0.00	0.00
Croatia - Escherichia coli ColREC SSI - model 29	0.00	0.00	0.00	0.00	0.00	0.00	0.00	0.00	0.00	0.00	0.00	0.00	0.00	0.00	0.00	0.00	0.00	0.00
Croatia - Escherichia coli ColREC UTI - model 28	0.00	0.00	0.00	0.00	0.00	0.00	0.00	0.00	0.00	0.00	0.00	0.00	0.00	0.00	0.00	0.00	0.00	0.00
Croatia - Escherichia coli CREC BSI - model 21	0.00	0.00	0.00	0.00	0.00	0.00	0.00	0.00	0.00	0.00	0.00	0.00	0.00	0.00	0.00	0.00	0.00	0.00
Croatia - Escherichia coli CREC OTH - model 25	0.00	0.00	0.00	0.00	0.00	0.00	0.00	0.00	0.00	0.00	0.00	0.00	0.00	0.00	0.00	0.00	0.00	0.00
Croatia - Escherichia coli CREC RESP - model 22	0.00	0.00	0.00	0.00	0.00	0.00	0.00	0.00	0.00	0.00	0.00	0.00	0.00	0.00	0.00	0.00	0.00	0.00
Croatia - Escherichia coli CREC SSI - model 24	0.00	0.00	0.00	0.00	0.00	0.00	0.00	0.00	0.00	0.00	0.00	0.00	0.00	0.00	0.00	0.00	0.00	0.00
Croatia - Escherichia coli CREC UTI - model 23	0.00	0.00	0.00	0.00	0.00	0.00	0.00	0.00	0.00	0.00	0.00	0.00	0.00	0.00	0.00	0.00	0.00	0.00
Croatia - Klebsiella pneumoniae 3GCRKP BSI - model 1	23.56	25.24	26.92	27.36	29.24	31.13	182.60	193.03	202.18	4.32	4.57	4.78	30.22	32.27	34.16	0.72	0.76	0.81
Croatia - Klebsiella pneumoniae 3GCRKP RESP - model 2	4.19	4.70	5.24	6.32	7.11	7.89	150.27	166.40	182.19	3.56	3.94	4.31	5.39	5.99	6.61	0.13	0.14	0.16
Croatia - Klebsiella pneumoniae 3GCRKP UTI - model 3	0.00	0.00	0.00	8.68E-03	0.01	0.01	207.26	227.91	248.92	4.91	5.39	5.89	0.00	0.00	0.00	0.00	0.00	0.00
Croatia - Klebsiella pneumoniae 3GCRKP OTH - model 5	0.00	0.00	0.00	4.91E-03	6.07E-03	7.42E-03	68.56	77.00	86.01	1.62	1.82	2.04	0.00	0.00	0.00	0.00	0.00	0.00
Croatia - Klebsiella pneumoniae 3GCRKP SSI - model 4	0.71	0.81	0.91	0.72	0.81	0.91	67.91	76.21	85.17	1.61	1.80	2.02	1.34	1.55	1.76	0.03	0.04	0.04
Croatia - Klebsiella pneumoniae ColRKP BSI - model 11	0.25	0.50	0.84	0.27	0.52	0.88	0.84	1.17	1.62	0.02	0.03	0.04	0.35	0.71	1.19	8.32E-03	0.02	0.03
Croatia - Klebsiella pneumoniae ColRKP OTH - model 15	0.00	0.00	0.00	9.27E-06	3.86E-05	1.15E-04	0.15	0.55	0.97	3.53E-03	0.01	0.02	0.00	0.00	0.00	0.00	0.00	0.00
Croatia - Klebsiella pneumoniae ColRKP RESP - model 12	3.27E-03	0.01	0.02	4.92E-03	0.02	0.04	0.13	0.51	0.90	3.08E-03	0.01	0.02	4.60E-03	0.02	0.03	1.09E-04	4.37E-04	7.84E-04
Croatia - Klebsiella pneumoniae ColRKP SSI - model 14	5.93E-04	2.23E-03	3.99E-03	5.96E-04	2.24E-03	4.06E-03	0.09	0.35	0.62	2.19E-03	8.25E-03	0.01	8.32E-04	3.14E-03	5.60E-03	1.97E-05	7.42E-05	1.33E-04
Croatia - Klebsiella pneumoniae ColRKP UTI - model 13	0.00	0.00	0.00	7.64E-06	3.04E-05	7.75E-05	0.26	0.71	1.18	6.16E-03	0.02	0.03	0.00	0.00	0.00	0.00	0.00	0.00
Croatia - Klebsiella pneumoniae CRKP BSI - model 6	1.70	2.17	2.72	1.86	2.34	2.90	8.21	9.48	10.78	0.19	0.22	0.26	2.63	3.35	4.20	0.06	0.08	0.10
Croatia - Klebsiella pneumoniae CRKP OTH - model 10	0.00	0.00	0.00	2.10E-04	3.65E-04	5.88E-04	3.18	4.45	5.76	0.08	0.11	0.14	0.00	0.00	0.00	0.00	0.00	0.00
Croatia - Klebsiella pneumoniae CRKP RESP - model 7	0.07	0.09	0.12	0.10	0.14	0.19	2.78	4.04	5.21	0.07	0.10	0.12	0.10	0.14	0.19	2.37E-03	3.42E-03	4.55E-03
Croatia - Klebsiella pneumoniae CRKP SSI - model 9	0.02	0.03	0.05	0.02	0.03	0.05	1.96	2.82	3.67	0.05	0.07	0.09	0.04	0.06	0.09	9.07E-04	1.49E-03	2.10E-03
Croatia - Klebsiella pneumoniae CRKP UTI - model 8	0.00	0.00	0.00	1.75E-04	2.80E-04	4.33E-04	4.19	5.73	7.34	0.10	0.14	0.17	0.00	0.00	0.00	0.00	0.00	0.00
Croatia - Pseudomonas aeruginosa ColRPA BSI - model 56	0.00	0.00	0.00	0.00	0.00	0.00	0.00	0.00	0.00	0.00	0.00	0.00	0.00	0.00	0.00	0.00	0.00	0.00
Croatia - Pseudomonas aeruginosa ColRPA OTH - model 60	0.00	0.00	0.00	0.00	0.00	0.00	0.00	0.00	0.00	0.00	0.00	0.00	0.00	0.00	0.00	0.00	0.00	0.00
Croatia - Pseudomonas aeruginosa ColRPA RESP - model 57	0.00	0.00	0.00	0.00	0.00	0.00	0.00	0.00	0.00	0.00	0.00	0.00	0.00	0.00	0.00	0.00	0.00	0.00
Croatia - Pseudomonas aeruginosa ColRPA SSI - model 59	0.00	0.00	0.00	0.00	0.00	0.00	0.00	0.00	0.00	0.00	0.00	0.00	0.00	0.00	0.00	0.00	0.00	0.00
Croatia - Pseudomonas aeruginosa ColRPA UTI - model 58	0.00	0.00	0.00	0.00	0.00	0.00	0.00	0.00	0.00	0.00	0.00	0.00	0.00	0.00	0.00	0.00	0.00	0.00
Croatia - Pseudomonas aeruginosa CRPA BSI - model 51	16.23	20.10	24.16	18.52	22.47	26.76	108.78	119.36	129.71	2.57	2.82	3.07	22.19	27.52	33.22	0.53	0.65	0.79
Croatia - Pseudomonas aeruginosa CRPA OTH - model 55	0.00	0.00	0.00	3.98E-03	5.26E-03	6.61E-03	50.28	60.87	71.16	1.19	1.44	1.68	0.00	0.00	0.00	0.00	0.00	0.00
Croatia - Pseudomonas aeruginosa CRPA RESP - model 52	17.30	21.27	25.82	22.73	27.26	32.34	389.82	443.27	492.83	9.23	10.49	11.66	23.38	28.83	35.28	0.55	0.68	0.84
Croatia - Pseudomonas aeruginosa CRPA SSI - model 54	0.72	0.84	0.98	0.72	0.85	0.99	69.41	82.63	96.84	1.64	1.96	2.29	1.29	1.59	1.89	0.03	0.04	0.04
Croatia - Pseudomonas aeruginosa CRPA UTI - model 53	0.00	0.00	0.00	5.94E-03	7.46E-03	9.47E-03	132.49	154.08	178.56	3.14	3.65	4.23	0.00	0.00	0.00	0.00	0.00	0.00
Croatia - Pseudomonas aeruginosa MDRPA BSI - model 46	0.63	1.09	1.68	0.75	1.23	1.84	5.86	7.19	8.77	0.14	0.17	0.21	0.91	1.52	2.26	0.02	0.04	0.05
Croatia - Pseudomonas aeruginosa MDRPA OTH - model 50	0.00	0.00	0.00	1.61E-04	3.25E-04	5.19E-04	2.30	3.71	5.10	0.05	0.09	0.12	0.00	0.00	0.00	0.00	0.00	0.00
Croatia - Pseudomonas aeruginosa MDRPA RESP - model 47	0.71	1.18	1.81	0.99	1.53	2.26	19.45	26.75	34.09	0.46	0.63	0.81	1.03	1.66	2.49	0.02	0.04	0.06
Croatia - Pseudomonas aeruginosa MDRPA SSI - model 49	0.02	0.04	0.05	0.02	0.04	0.05	3.09	5.02	6.91	0.07	0.12	0.16	0.04	0.07	0.09	9.76E-04	1.60E-03	2.24E-03
Croatia - Pseudomonas aeruginosa MDRPA UTI - model 48	0.00	0.00	0.00	2.43E-04	4.36E-04	7.12E-04	6.23	9.27	12.56	0.15	0.22	0.30	0.00	0.00	0.00	0.00	0.00	0.00
Croatia - Staphylococcus aureus MRSA BSI - model 61	11.96	12.75	13.60	13.76	14.64	15.58	128.36	136.26	144.89	3.04	3.22	3.43	22.74	24.48	26.39	0.54	0.58	0.62
Croatia - Staphylococcus aureus MRSA OTH - model 65	0.00	0.00	0.00	0.01	0.02	0.02	180.10	201.90	222.27	4.26	4.78	5.26	0.00	0.00	0.00	0.00	0.00	0.00
Croatia - Staphylococcus aureus MRSA RESP - model 62	3.48	3.86	4.27	5.26	5.85	6.44	185.36	205.12	226.26	4.39	4.85	5.35	6.60	7.38	8.32	0.16	0.17	0.20
Croatia - Staphylococcus aureus MRSA SSI - model 64	1.94	2.16	2.37	1.95	2.17	2.38	201.68	223.91	245.61	4.77	5.30	5.81	5.06	5.71	6.39	0.12	0.14	0.15
Croatia - Staphylococcus aureus MRSA UTI - model 63	0.00	0.00	0.00	1.14E-03	1.48E-03	1.92E-03	27.79	33.48	39.39	0.66	0.79	0.93	0.00	0.00	0.00	0.00	0.00	0.00
Croatia - Streptococcus pneumoniae PMRSP BSI - model 76	0.35	0.40	0.45	0.40	0.45	0.51	1.07	1.11	1.16	0.03	0.03	0.03	0.17	0.20	0.23	4.13E-03	4.72E-03	5.37E-03
Croatia - Streptococcus pneumoniae PMRSP OTH - model 80	0.00	0.00	0.00	6.77E-06	1.74E-05	3.61E-05	0.31	0.39	0.54	7.25E-03	9.33E-03	0.01	0.00	0.00	0.00	0.00	0.00	0.00
Croatia - Streptococcus pneumoniae PMRSP RESP - model 77	0.20	0.29	0.49	0.30	0.43	0.72	2.94	3.99	6.72	0.07	0.09	0.16	0.10	0.15	0.25	2.34E-03	3.44E-03	5.84E-03
Croatia - Streptococcus pneumoniae PMRSP SSI - model 79	0.00	0.00	0.00	0.00	0.00	0.00	0.00	0.00	0.00	0.00	0.00	0.00	0.00	0.00	0.00	0.00	0.00	0.00
Croatia - Streptococcus pneumoniae PMRSP UTI - model 78	0.00	0.00	0.00	0.00	0.00	0.00	0.00	0.00	0.00	0.00	0.00	0.00	0.00	0.00	0.00	0.00	0.00	0.00
Croatia - Streptococcus pneumoniae PRSP BSI - model 71	0.00	0.00	0.00	0.00	0.00	0.00	0.00	0.00	0.00	0.00	0.00	0.00	0.00	0.00	0.00	0.00	0.00	0.00
Croatia - Streptococcus pneumoniae PRSP OTH - model 75	0.00	0.00	0.00	0.00	0.00	0.00	0.00	0.00	0.00	0.00	0.00	0.00	0.00	0.00	0.00	0.00	0.00	0.00
Croatia - Streptococcus pneumoniae PRSP RESP - model 72	0.00	0.00	0.00	0.00	0.00	0.00	0.00	0.00	0.00	0.00	0.00	0.00	0.00	0.00	0.00	0.00	0.00	0.00
Croatia - Streptococcus pneumoniae PRSP SSI - model 74	0.00	0.00	0.00	0.00	0.00	0.00	0.00	0.00	0.00	0.00	0.00	0.00	0.00	0.00	0.00	0.00	0.00	0.00
Croatia - Streptococcus pneumoniae PRSP UTI - model 73	0.00	0.00	0.00	0.00	0.00	0.00	0.00	0.00	0.00	0.00	0.00	0.00	0.00	0.00	0.00	0.00	0.00	0.00
**Total (Median & 95% UIs)**	130.70	153.22	177.30	157.78	182.56	209.15	3,838.12	4,347.46	4,866.55	90.84	102.89	115.18	203.82	239.74	278.43	4.82	5.67	6.59
**Total (average)**		153.48			182.82			4,347.31			102.89			239.96			5.68	

Infection	YLD			YLL			DALY			DALY per Case			YLD per 100,000			YLL per 100,000			DALY per 100,000			Cases per year			Incidence per 100,000			Deaths per year			Mortality per 100,000		
Colistin resistant *Acinetobacter spp.*	0.00	0.00	0.00	0.00	0.00	0.00	0.00	0.00	0.00	0.00	0.00	0.00	0.00	0.00	0.00	0.00	0.00	0.00	0.00	0.00	0.00	0.00	0.00	0.00	0.00	0.00	0.00	0.00	0.00	0.00	0.00	0.00	0.00
Carbapenem resistant *Acinetobacter spp.* (excluding those resistant to colistin)	310.09	341.22	375.20	1,509.77	1,826.14	2,136.89	1,835.02	2,165.80	2,491.25	6.52	7.45	8.39	3.15	3.46	3.81	15.32	18.53	21.68	18.62	21.98	25.28	774.15	860.35	946.80	7.85	8.73	9.61	62.51	74.96	88.22	0.63	0.76	0.90
Aminoglycosides and fluoroquinolones resistant *Acinetobacter* spp. (excluding those resistant to colistin and/or carbapenem)	45.30	51.52	58.85	212.77	270.68	328.86	261.18	322.64	384.73	6.14	7.36	8.61	0.46	0.52	0.60	2.16	2.75	3.34	2.65	3.27	3.90	114.05	129.61	146.50	1.16	1.32	1.49	8.79	11.13	13.61	0.09	0.11	0.14
Vancomycin resistant Enterococci (*E. faecalis* and *E. faecium*)	28.01	34.24	42.38	265.80	329.24	394.15	292.85	363.53	437.22	5.53	6.06	6.67	0.28	0.35	0.43	2.70	3.34	4.00	2.97	3.69	4.44	179.10	228.37	279.45	1.82	2.32	2.84	12.61	15.66	18.73	0.13	0.16	0.19
Colistin resistant *Escherichia coli*	0.00	0.00	0.00	0.00	0.00	0.00	0.00	0.00	0.00	0.00	0.00	0.00	0.00	0.00	0.00	0.00	0.00	0.00	0.00	0.00	0.00	0.00	0.00	0.00	0.00	0.00	0.00	0.00	0.00	0.00	0.00	0.00	0.00
Carbapenem resistant *Escherichia coli* (excluding those resistant to colistin)	1.92	3.27	4.92	18.16	35.22	57.32	20.55	38.56	62.15	22.76	35.49	47.57	0.02	0.03	0.05	0.18	0.36	0.58	0.21	0.39	0.63	5.17	8.65	12.28	0.05	0.09	0.12	0.23	0.45	0.73	0.00	0.00	0.01
Third-generation cephalosporin resistant *Escherichia coli* (excluding those resistant to colistin and/or carbapenem)	259.27	291.12	324.12	1,541.46	1,724.47	1,921.94	1,814.62	2,015.39	2,247.60	5.28	5.71	6.16	2.63	2.95	3.29	15.64	17.50	19.50	18.41	20.45	22.81	2,516.61	2,820.71	3,123.08	25.53	28.62	31.69	76.09	85.76	96.81	0.77	0.87	0.98
Colistin resistant *Klebsiella pneumoniae*	0.00	0.00	0.00	0.00	0.00	0.00	0.00	0.00	0.00	0.00	0.00	0.00	0.00	0.00	0.00	0.00	0.00	0.00	0.00	0.00	0.00	0.00	0.00	0.00	0.00	0.00	0.00	0.00	0.00	0.00	0.00	0.00	0.00
Carbapenem resistant *Klebsiella pneumoniae* (excluding those resistant to colistin)	1.01	1.75	2.79	9.98	18.79	31.34	11.19	20.56	33.56	12.83	19.57	26.60	0.01	0.02	0.03	0.10	0.19	0.32	0.11	0.21	0.34	1.57	3.28	5.09	0.02	0.03	0.05	0.23	0.43	0.71	0.00	0.00	0.01
Third-generation cephalosporin resistant *Klebsiella pneumoniae* (excluding those resistant to colistin and/or carbapenem)	298.48	323.97	350.13	1,464.50	1,556.13	1,649.60	1,772.85	1,881.26	1,988.84	6.58	6.84	7.13	3.03	3.29	3.55	14.86	15.79	16.74	17.99	19.09	20.18	1,039.64	1,131.76	1,230.49	10.55	11.48	12.49	57.32	61.42	65.84	0.58	0.62	0.67
Colistin resistant *Pseudomonas aeruginosa*	2.12	3.91	6.08	6.38	18.58	38.34	9.19	22.45	43.51	5.52	11.82	18.64	0.02	0.04	0.06	0.06	0.19	0.39	0.09	0.23	0.44	4.29	8.51	12.88	0.04	0.09	0.13	0.18	0.54	1.11	0.00	0.01	0.01
Carbapenem resistant *Pseudomonas aeruginosa* (excluding those resistant to colistin)	866.53	964.16	1,065.39	4,096.11	4,857.62	5,707.05	4,991.98	5,822.40	6,740.55	9.56	10.94	12.49	8.79	9.78	10.81	41.56	49.29	57.91	50.65	59.08	68.39	2,096.64	2,356.96	2,630.92	21.27	23.92	26.69	132.30	159.57	191.71	1.34	1.62	1.95
Pseudomonas aeruginosa resistant to three or more antimicrobial groups (excluding those resistant to colistin and/or carbapenem)	59.77	71.86	85.02	252.41	340.71	437.65	318.01	412.44	519.44	7.01	8.80	10.67	0.61	0.73	0.86	2.56	3.46	4.44	3.23	4.18	5.27	167.51	206.26	243.86	1.70	2.09	2.47	9.59	13.06	16.98	0.10	0.13	0.17
Methicillin resistant *Staphylococcus aureus*	415.07	458.12	503.10	2.081.78	2.239.54	2.404.91	2.506.29	2.698.09	2.892.11	5.29	5.53	5.77	4.21	4.65	5.10	21.12	22.72	24.40	25.43	27.38	29.34	2.271.74	2.493.50	2.706.84	23.05	25.30	27.47	109.44	118.74	127.88	1.11	1.20	1.30
Penicillin and macrolides resistant *Streptococcus pneumoniae* (excluding those only resistant to penicillin)	3.78	4.93	6.79	13.45	16.31	19.96	17.67	21.24	26.28	5.50	6.07	6.67	0.04	0.05	0.07	0.14	0.17	0.20	0.18	0.22	0.27	9.34	11.37	14.53	0.09	0.12	0.15	0.58	0.70	0.85	0.01	0.01	0.01
Penicillin tesistant *Streptococcus pneumoniae* (excluding those resistant to macrolides)	4.82	6.59	10.02	18.10	21.93	28.47	23.53	28.56	37.89	7.19	8.26	9.39	0.05	0.07	0.10	0.18	0.22	0.29	0.24	0.29	0.38	9.36	11.29	14.86	0.09	0.11	0.15	0.58	0.70	0.86	0.01	0.01	0.01
Total	2,296.17	2,556.67	2,834.78	11,490.67	13,255.37	15,156.49	13,874.92	15,812.92	17,905.14				23.30	25.94	28.76	116.59	134.50	153.79	140.78	160.45	181.68	9,189.16	10,270.61	11,367.59	93.24	104.21	115.34	470.46	543.11	624.03	4.77	5.51	6.33

Model	YLD			YLL			DALY			DALY per Case			YLD per 100,000			YLL per 100,000			DALY per 100,000			Cases per year			Incidence per 100,000			Deaths per year			Mortality per 100,000		
Hungary - Acinetobacter spp. CoIRACI BSI - model 41	0.00	0.00	0.00	0.00	0.00	0.00	0.00	0.00	0.00	NaN	NaN	NaN	0.00	0.00	0.00	0.00	0.00	0.00	0.00	0.00	0.00	0.00	0.00	0.00	0.00	0.00	0.00	0.00	0.00	0.00	0.00	0.00	0.00
Hungary - Acinetobacter spp. CoIRACI OTH - model 45	0.00	0.00	0.00	0.00	0.00	0.00	0.00	0.00	0.00	NaN	NaN	NaN	0.00	0.00	0.00	0.00	0.00	0.00	0.00	0.00	0.00	0.00	0.00	0.00	0.00	0.00	0.00	0.00	0.00	0.00	0.00	0.00	0.00
Hungary - Acinetobacter spp. CoIRACI RESP - model 42	0.00	0.00	0.00	0.00	0.00	0.00	0.00	0.00	0.00	NaN	NaN	NaN	0.00	0.00	0.00	0.00	0.00	0.00	0.00	0.00	0.00	0.00	0.00	0.00	0.00	0.00	0.00	0.00	0.00	0.00	0.00	0.00	0.00
Hungary - Acinetobacter spp. CoIRACI SSI - model 44	0.00	0.00	0.00	0.00	0.00	0.00	0.00	0.00	0.00	NaN	NaN	NaN	0.00	0.00	0.00	0.00	0.00	0.00	0.00	0.00	0.00	0.00	0.00	0.00	0.00	0.00	0.00	0.00	0.00	0.00	0.00	0.00	0.00
Hungary - Acinetobacter spp. CoIRACI UTI - model 43	0.00	0.00	0.00	0.00	0.00	0.00	0.00	0.00	0.00	NaN	NaN	NaN	0.00	0.00	0.00	0.00	0.00	0.00	0.00	0.00	0.00	0.00	0.00	0.00	0.00	0.00	0.00	0.00	0.00	0.00	0.00	0.00	0.00
Hungary - Acinetobacter spp. CRACI BSI - model 36	179.24	194.50	212.61	1,216.21	1,495.21	1,766.69	1,409.08	1,688.58	1,962.07	4.84	5.68	6.51	1.82	1.97	2.16	12.34	15.17	17.93	14.30	17.13	19.91	280.60	297.37	315.56	2.85	3.02	3.20	50.10	60.84	72.40	0.51	0.62	0.73
Hungary - Acinetobacter spp. CRACI OTH - model 40	0.03	0.04	0.05	0.00	0.00	0.00	0.03	0.04	0.05	3.07E-03	3.72E-03	4.44E-03	2.94E-04	3.97E-04	5.23E-04	0.00	0.00	0.00	2.94E-04	3.97E-04	5.23E-04	8.14	10.53	13.13	0.08	0.11	0.13	0.00	0.00	0.00	0.00	0.00	0.00
Hungary - Acinetobacter spp. CRACI RESP - model 37	130.45	146.21	161.92	254.59	284.68	317.03	386.54	430.47	475.39	1.27	1.34	1.41	1.32	1.48	1.64	2.58	2.89	3.22	3.92	4.37	4.82	289.66	321.91	354.29	2.94	3.27	3.59	10.32	11.58	12.85	0.10	0.12	0.13
Hungary - Acinetobacter spp. CRACI SSI - model 39	0.17	0.23	0.30	38.96	46.26	53.17	39.17	46.46	53.44	0.40	0.43	0.46	1.75E-03	2.32E-03	3.05E-03	0.40	0.47	0.54	0.40	0.47	0.54	92.73	108.85	123.85	0.94	1.10	1.26	2.10	2.54	2.98	0.02	0.03	0.03
Hungary - Acinetobacter spp. CRACI UTI - model 38	0.19	0.25	0.31	0.00	0.00	0.00	0.19	0.25	0.31	1.70E-03	2.04E-03	2.40E-03	1.97E-03	2.52E-03	3.14E-03	0.00	0.00	0.00	1.97E-03	2.52E-03	3.14E-03	103.02	121.68	139.98	1.05	1.23	1.42	0.00	0.00	0.00	0.00	0.00	0.00
Hungary - Acinetobacter spp. MDRACI BSI - model 31	26.30	29.49	33.35	170.11	220.39	270.91	199.06	250.32	301.91	4.47	5.56	6.67	0.27	0.30	0.34	1.73	2.24	2.75	2.02	2.54	3.06	41.83	44.89	48.59	0.42	0.46	0.49	6.96	9.00	11.15	0.07	0.09	0.11
Hungary - Acinetobacter spp. MDRACI OTH - model 35	3.95E-03	5.85E-03	8.35E-03	0.00	0.00	0.00	3.95E-03	5.85E-03	8.35E-03	2.86E-03	3.73E-03	4.66E-03	4.00E-05	5.94E-05	8.47E-05	0.00	0.00	0.00	4.00E-05	5.94E-05	8.47E-05	1.13	1.57	2.08	0.01	0.02	0.02	0.00	0.00	0.00	0.00	0.00	0.00
Hungary - Acinetobacter spp. MDRACI RESP - model 32	18.95	21.95	25.39	36.74	42.83	48.95	56.14	64.78	73.70	1.24	1.33	1.43	0.19	0.22	0.26	0.37	0.43	0.50	0.57	0.66	0.75	42.63	48.53	54.85	0.43	0.49	0.56	1.52	1.74	1.99	0.02	0.02	0.02
Hungary - Acinetobacter spp. MDRACI SSI - model 34	0.02	0.03	0.05	5.92	7.46	9.00	5.95	7.50	9.06	0.42	0.46	0.50	2.41E-04	3.51E-04	5.02E-04	0.06	0.08	0.09	0.06	0.08	0.09	13.64	16.33	19.20	0.14	0.17	0.19	0.31	0.39	0.48	3.12E-03	3.98E-03	4.84E-03
Hungary - Acinetobacter spp. MDRACI UTI - model 33	0.03	0.04	0.05	0.00	0.00	0.00	0.03	0.04	0.05	1.56E-03	2.03E-03	2.53E-03	2.68E-04	3.75E-04	4.98E-04	0.00	0.00	0.00	2.68E-04	3.75E-04	4.98E-04	14.82	18.29	21.78	0.15	0.19	0.22	0.00	0.00	0.00	0.00	0.00	0.00
Hungary - Enterococcus faecalis and E. faecium VRE BSI - model 66	27.79	33.92	41.91	236.18	289.83	344.97	262.97	323.77	387.61	5.07	5.56	6.12	0.28	0.34	0.43	2.40	2.94	3.50	2.67	3.29	3.93	47.46	58.32	69.35	0.48	0.59	0.70	10.89	13.34	15.81	0.11	0.14	0.16
Hungary - Enterococcus faecalis and E. faecium VRE OTH - model 70	0.07	0.10	0.14	0.00	0.00	0.00	0.07	0.10	0.14	1.27E-03	1.64E-03	2.11E-03	7.11E-04	1.03E-03	1.44E-03	0.00	0.00	0.00	7.11E-04	1.03E-03	1.44E-03	48.05	61.57	76.05	0.49	0.62	0.77	0.00	0.00	0.00	0.00	0.00	0.00
Hungary - Enterococcus faecalis and E. faecium VRE RESP - model 67	0.00	0.00	0.00	0.00	0.00	0.00	0.00	0.00	0.00	NaN	NaN	NaN	0.00	0.00	0.00	0.00	0.00	0.00	0.00	0.00	0.00	0.00	0.00	0.00	0.00	0.00	0.00	0.00	0.00	0.00	0.00	0.00	0.00
Hungary - Enterococcus faecalis and E. faecium VRE SSI - model 69	0.11	0.17	0.24	29.62	39.41	49.18	29.77	39.60	49.39	0.46	0.50	0.54	1.14E-03	1.71E-03	2.48E-03	0.30	0.40	0.50	0.30	0.40	0.50	61.32	79.29	97.84	0.62	0.80	0.99	1.72	2.32	2.92	0.02	0.02	0.03
Hungary - Enterococcus faecalis and E. faecium VRE UTI - model 68	0.04	0.05	0.08	0.00	0.00	0.00	0.04	0.05	0.08	1.44E-03	1.87E-03	2.35E-03	3.89E-04	5.50E-04	7.64E-04	0.00	0.00	0.00	3.89E-04	5.50E-04	7.64E-04	22.27	29.18	36.21	0.23	0.30	0.37	0.00	0.00	0.00	0.00	0.00	0.00
Hungary - Escherichia coli 3GCREC BSI - model 16	199.25	219.31	239.95	1,279.11	1,416.24	1,566.13	1,491.13	1,635.06	1,807.91	3.83	4.14	4.47	2.02	2.23	2.43	12.98	14.37	15.89	15.13	16.59	18.34	369.33	395.73	424.08	3.75	4.02	4.30	61.32	68.32	76.54	0.62	0.69	0.78
Hungary - Escherichia coli 3GCREC OTH - model 20	0.33	0.43	0.57	0.00	0.00	0.00	0.33	0.43	0.57	2.73E-03	3.36E-03	4.07E-03	3.34E-03	4.40E-03	5.81E-03	0.00	0.00	0.00	3.34E-03	4.40E-03	5.81E-03	106.22	130.28	155.77	1.08	1.32	1.58	0.00	0.00	0.00	0.00	0.00	0.00
Hungary - Escherichia coli 3GCREC RESP - model 17	56.36	67.16	78.27	108.25	130.30	151.07	165.49	197.66	229.25	1.05	1.13	1.22	0.57	0.68	0.79	1.10	1.32	1.53	1.68	2.01	2.33	144.23	175.53	202.42	1.46	1.78	2.05	5.19	6.29	7.34	0.05	0.06	0.07
Hungary - Escherichia coli 3GCREC SSI - model 19	0.65	0.89	1.16	154.09	177.93	204.74	154.98	178.92	205.71	0.41	0.43	0.45	6.55E-03	8.98E-03	0.01	1.56	1.81	2.08	1.57	1.82	2.09	367.79	416.09	469.57	3.73	4.22	4.76	9.58	11.15	12.93	0.10	0.11	0.13
Hungary - Escherichia coli 3GCREC UTI - model 18	2.69	3.33	4.17	0.00	0.00	0.00	2.69	3.33	4.17	1.61E-03	1.97E-03	2.36E-03	0.03	0.03	0.04	0.00	0.00	0.00	0.03	0.03	0.04	1,529.03	1,703.08	1,871.24	15.51	17.28	18.99	0.00	0.00	0.00	0.00	0.00	0.00
Hungary - Escherichia coli CoIREC BSI - model 26	0.00	0.00	0.00	0.00	0.00	0.00	0.00	0.00	0.00	NaN	NaN	NaN	0.00	0.00	0.00	0.00	0.00	0.00	0.00	0.00	0.00	0.00	0.00	0.00	0.00	0.00	0.00	0.00	0.00	0.00	0.00	0.00	0.00
Hungary - Escherichia coli CoIREC OTH - model 30	0.00	0.00	0.00	0.00	0.00	0.00	0.00	0.00	0.00	NaN	NaN	NaN	0.00	0.00	0.00	0.00	0.00	0.00	0.00	0.00	0.00	0.00	0.00	0.00	0.00	0.00	0.00	0.00	0.00	0.00	0.00	0.00	0.00
Hungary - Escherichia coli CoIREC RESP - model 27	0.00	0.00	0.00	0.00	0.00	0.00	0.00	0.00	0.00	NaN	NaN	NaN	0.00	0.00	0.00	0.00	0.00	0.00	0.00	0.00	0.00	0.00	0.00	0.00	0.00	0.00	0.00	0.00	0.00	0.00	0.00	0.00	0.00
Hungary - Escherichia coli CoIREC SSI - model 29	0.00	0.00	0.00	0.00	0.00	0.00	0.00	0.00	0.00	NaN	NaN	NaN	0.00	0.00	0.00	0.00	0.00	0.00	0.00	0.00	0.00	0.00	0.00	0.00	0.00	0.00	0.00	0.00	0.00	0.00	0.00	0.00	0.00
Hungary - Escherichia coli CoIREC UTI - model 28	0.00	0.00	0.00	0.00	0.00	0.00	0.00	0.00	0.00	NaN	NaN	NaN	0.00	0.00	0.00	0.00	0.00	0.00	0.00	0.00	0.00	0.00	0.00	0.00	0.00	0.00	0.00	0.00	0.00	0.00	0.00	0.00	0.00
Hungary - Escherichia coli CREC BSI - model 21	1.59	2.41	3.50	16.97	32.62	53.16	19.02	35.11	56.63	18.40	30.52	42.01	0.02	0.02	0.04	0.17	0.33	0.54	0.19	0.36	0.57	0.89	1.15	1.50	9.05E-03	0.01	0.02	0.22	0.41	0.68	2.19E-03	4.20E-03	6.85E-03
Hungary - Escherichia coli CREC OTH - model 25	5.84E-04	2.07E-03	5.47E-03	0.00	0.00	0.00	5.84E-04	2.07E-03	5.47E-03	1.19E-03	3.31E-03	7.23E-03	5.93E-06	2.10E-05	5.55E-05	0.00	0.00	0.00	5.93E-06	2.10E-05	5.55E-05	0.29	0.66	1.04	2.94E-03	6.73E-03	0.01	0.00	0.00	0.00	0.00	0.00	0.00
Hungary - Escherichia coli CREC RESP - model 22	0.33	0.84	1.39	0.66	1.63	2.73	1.00	2.47	4.06	3.65	4.25	4.84	3.33E-03	8.53E-03	0.01	6.74E-03	0.02	0.03	0.01	0.03	0.04	0.25	0.59	0.93	2.51E-03	5.94E-03	9.41E-03	8.44E-03	0.02	0.03	8.56E-05	2.09E-04	3.52E-04
Hungary - Escherichia coli CREC SSI - model 24	6.11E-04	2.61E-03	7.23E-03	0.52	0.97	1.43	0.52	0.97	1.44	0.71	0.71	0.71	6.20E-06	2.65E-05	7.33E-05	5.29E-03	9.82E-03	0.01	5.31E-03	9.85E-03	0.01	0.74	1.37	2.02	7.47E-03	0.01	0.02	6.62E-03	0.01	0.02	6.72E-05	1.25E-04	1.84E-04
Hungary - Escherichia coli CREC UTI - model 23	3.42E-03	9.52E-03	0.02	0.00	0.00	0.00	3.42E-03	9.52E-03	0.02	7.95E-04	1.98E-03	3.88E-03	3.47E-05	9.66E-05	2.12E-04	0.00	0.00	0.00	3.47E-05	9.66E-05	2.12E-04	3.01	4.89	6.79	0.03	0.05	0.07	0.00	0.00	0.00	0.00	0.00	0.00
Hungary - Klebsiella pneumoniae 3GCRKP BSI - model 1	187.36	201.52	215.91	1,203.38	1,267.34	1,332.20	1,399.79	1,469.49	1,538.92	4.80	4.97	5.16	1.90	2.04	2.19	12.21	12.86	13.52	14.20	14.91	15.61	282.85	295.35	309.55	2.87	3.00	3.14	46.65	49.34	52.34	0.47	0.50	0.53
Hungary - Klebsiella pneumoniae 3GCRKP OTH - model 5	0.32	0.39	0.48	0.00	0.00	0.00	0.32	0.39	0.48	2.76E-03	3.36E-03	3.99E-03	3.21E-03	3.98E-03	4.92E-03	0.00	0.00	0.00	3.21E-03	3.98E-03	4.92E-03	104.40	117.49	130.77	1.06	1.19	1.33	0.00	0.00	0.00	0.00	0.00	0.00
Hungary - Klebsiella pneumoniae 3GCRKP RESP - model 2	110.07	121.13	132.59	214.36	234.96	256.28	325.23	356.62	387.22	1.33	1.40	1.47	1.12	1.23	1.35	2.18	2.38	2.60	3.30	3.62	3.93	231.83	254.73	279.15	2.35	2.58	2.83	8.20	9.18	10.18	0.08	0.09	0.10
Hungary - Klebsiella pneumoniae 3GCRKP SSI - model 4	0.19	0.25	0.32	46.76	53.84	61.13	46.96	54.08	61.39	0.44	0.47	0.49	1.90E-03	2.52E-03	3.20E-03	0.47	0.55	0.62	0.48	0.55	0.62	102.62	116.04	129.69	1.04	1.18	1.32	2.48	2.91	3.32	0.03	0.03	0.03
Hungary - Klebsiella pneumoniae 3GCRKP UTI - model 3	0.55	0.68	0.83	0.00	0.00	0.00	0.55	0.68	0.83	1.64E-03	1.96E-03	2.33E-03	5.60E-03	6.93E-03	8.47E-03	0.00	0.00	0.00	5.60E-03	6.93E-03	8.47E-03	317.94	348.14	381.34	3.23	3.53	3.87	0.00	0.00	0.00	0.00	0.00	0.00
Hungary - Klebsiella pneumoniae CoIRKP BSI - model 11	0.00	0.00	0.00	0.00	0.00	0.00	0.00	0.00	0.00	NaN	NaN	NaN	0.00	0.00	0.00	0.00	0.00	0.00	0.00	0.00	0.00	0.00	0.00	0.00	0.00	0.00	0.00	0.00	0.00	0.00	0.00	0.00	0.00
Hungary - Klebsiella pneumoniae CoIRKP OTH - model 15	0.00	0.00	0.00	0.00	0.00	0.00	0.00	0.00	0.00	NaN	NaN	NaN	0.00	0.00	0.00	0.00	0.00	0.00	0.00	0.00	0.00	0.00	0.00	0.00	0.00	0.00	0.00	0.00	0.00	0.00	0.00	0.00	0.00
Hungary - Klebsiella pneumoniae CoIRKP RESP - model 12	0.00	0.00	0.00	0.00	0.00	0.00	0.00	0.00	0.00	NaN	NaN	NaN	0.00	0.00	0.00	0.00	0.00	0.00	0.00	0.00	0.00	0.00	0.00	0.00	0.00	0.00	0.00	0.00	0.00	0.00	0.00	0.00	0.00
Hungary - Klebsiella pneumoniae CoIRKP SSI - model 14	0.00	0.00	0.00	0.00	0.00	0.00	0.00	0.00	0.00	NaN	NaN	NaN	0.00	0.00	0.00	0.00	0.00	0.00	0.00	0.00	0.00	0.00	0.00	0.00	0.00	0.00	0.00	0.00	0.00	0.00	0.00	0.00	0.00
Hungary - Klebsiella pneumoniae CoIRKP UTI - model 13	0.00	0.00	0.00	0.00	0.00	0.00	0.00	0.00	0.00	NaN	NaN	NaN	0.00	0.00	0.00	0.00	0.00	0.00	0.00	0.00	0.00	0.00	0.00	0.00	0.00	0.00	0.00	0.00	0.00	0.00	0.00	0.00	0.00
Hungary - Klebsiella pneumoniae CRKP BSI - model 6	0.86	1.34	2.05	9.67	17.85	29.69	10.74	19.20	31.19	10.37	16.75	23.45	8.78E-03	0.01	0.02	0.10	0.18	0.30	0.11	0.19	0.32	0.87	1.16	1.54	8.87E-03	0.01	0.02	0.22	0.40	0.67	2.22E-03	4.10E-03	6.82E-03
Hungary - Klebsiella pneumoniae CRKP OTH - model 10	3.89E-04	1.69E-03	4.99E-03	0.00	0.00	0.00	3.89E-04	1.69E-03	4.99E-03	1.18E-03	3.25E-03	7.18E-03	3.95E-06	1.71E-05	5.06E-05	0.00	0.00	0.00	3.95E-06	1.71E-05	5.06E-05	0.18	0.56	0.93	1.79E-03	5.65E-03	9.47E-03	0.00	0.00	0.00	0.00	0.00	0.00
Hungary - Klebsiella pneumoniae CRKP RESP - model 7	0.14	0.40	0.73	0.27	0.81	1.42	0.41	1.23	2.12	2.06	2.42	2.74	1.42E-03	4.10E-03	7.43E-03	2.77E-03	8.17E-03	0.01	4.20E-03	0.01	0.02	0.17	0.51	0.85	1.76E-03	5.18E-03	8.58E-03	6.17E-03	0.02	0.03	6.26E-05	1.85E-04	3.25E-04
Hungary - Klebsiella pneumoniae CRKP SSI - model 9	1.08E-04	6.27E-04	1.99E-03	0.03	0.13	0.24	0.04	0.13	0.24	0.40	0.40	0.40	1.10E-06	6.37E-06	2.02E-05	3.50E-04	1.35E-03	2.41E-03	3.59E-04	1.36E-03	2.44E-03	0.09	0.33	0.60	8.81E-04	3.40E-03	6.07E-03	7.81E-04	3.01E-03	5.38E-03	7.93E-06	3.06E-05	5.46E-05
Hungary - Klebsiella pneumoniae CRKP UTI - model 8	3.78E-04	1.36E-03	3.57E-03	0.00	0.00	0.00	3.78E-04	1.36E-03	3.57E-03	7.28E-04	2.02E-03	3.94E-03	3.83E-06	1.38E-05	3.62E-05	0.00	0.00	0.00	3.83E-06	1.38E-05	3.62E-05	0.26	0.72	1.17	2.62E-03	7.31E-03	0.01	0.00	0.00	0.00	0.00	0.00	0.00
Hungary - Pseudomonas aeruginosa CoIRPA BSI - model 56	0.68	1.07	1.64	2.91	8.90	18.03	3.86	9.99	19.41	3.59	8.57	14.08	6.89E-03	0.01	0.02	0.03	0.09	0.18	0.04	0.10	0.20	0.85	1.17	1.63	8.61E-03	0.01	0.02	0.08	0.26	0.52	8.53E-04	2.60E-03	5.28E-03
Hungary - Pseudomonas aeruginosa CoIRPA OTH - model 60	5.38E-04	2.11E-03	5.09E-03	0.00	0.00	0.00	5.38E-04	2.11E-03	5.09E-03	1.55E-03	3.74E-03	6.49E-03	5.46E-06	2.14E-05	5.17E-05	0.00	0.00	0.00	5.46E-06	2.14E-05	5.17E-05	0.19	0.62	1.02	1.96E-03	6.26E-03	0.01	0.00	0.00	0.00	0.00	0.00	0.00
Hungary - Pseudomonas aeruginosa CoIRPA RESP - model 57	1.44	2.83	4.42	3.38	9.43	19.90	5.25	12.20	23.66	1.61	2.93	4.23	0.01	0.03	0.04	0.03	0.10	0.20	0.05	0.12	0.24	2.38	4.38	6.47	0.02	0.04	0.07	0.10	0.27	0.57	9.91E-04	2.76E-03	5.82E-03
Hungary - Pseudomonas aeruginosa CoIRPA SSI - model 59	2.74E-04	1.48E-03	4.49E-03	0.08	0.25	0.42	0.08	0.25	0.42	0.31	0.31	0.32	2.78E-06	1.50E-05	4.56E-05	7.96E-04	2.56E-03	4.25E-03	8.20E-04	2.57E-03	4.28E-03	0.25	0.81	1.34	2.55E-03	8.18E-03	0.01	2.26E-03	7.26E-03	0.01	2.30E-05	7.36E-05	1.22E-04
Hungary - Pseudomonas aeruginosa CoIRPA UTI - model 58	7.38E-04	2.70E-03	6.56E-03	0.00	0.00	0.00	7.38E-04	2.70E-03	6.56E-03	8.08E-04	1.92E-03	3.62E-03	7.49E-06	2.74E-05	6.65E-05	0.00	0.00	0.00	7.49E-06	2.74E-05	6.65E-05	0.62	1.53	2.42	6.28E-03	0.02	0.02	0.00	0.00	0.00	0.00	0.00	0.00
Hungary - Pseudomonas aeruginosa CRPA BSI - model 51	247.78	270.21	297.43	1,940.23	2,296.16	2,700.44	2,179.75	2,562.87	2,989.59	6.82	7.86	9.07	2.51	2.74	3.02	19.69	23.30	27.40	22.12	26.00	30.33	304.44	327.17	353.52	3.09	3.32	3.59	62.22	74.91	89.20	0.63	0.76	0.91
Hungary - Pseudomonas aeruginosa CRPA OTH - model 55	0.48	0.61	0.75	0.00	0.00	0.00	0.48	0.61	0.75	3.08E-03	3.65E-03	4.31E-03	4.83E-03	6.16E-03	7.61E-03	0.00	0.00	0.00	4.83E-03	6.16E-03	7.61E-03	140.40	166.15	191.96	1.42	1.69	1.95	0.00	0.00	0.00	0.00	0.00	0.00
Hungary - Pseudomonas aeruginosa CRPA RESP - model 52	617.22	692.01	765.53	2,068.71	2,458.18	2,886.10	2,723.50	3,154.34	3,628.11	2.31	2.61	2.92	6.26	7.02	7.77	20.99	24.94	29.28	27.63	32.01	36.81	1,091.39	1,217.63	1,345.72	11.07	12.35	13.65	66.24	79.95	96.96	0.67	0.81	0.98
Hungary - Pseudomonas aeruginosa CRPA SSI - model 54	0.37	0.48	0.61	87.17	103.28	120.51	87.56	103.72	121.04	0.43	0.46	0.49	3.72E-03	4.84E-03	6.22E-03	0.88	1.05	1.22	0.89	1.05	1.23	192.45	224.57	260.70	1.95	2.28	2.65	3.85	4.71	5.55	0.04	0.05	0.06
Hungary - Pseudomonas aeruginosa CRPA UTI - model 53	0.69	0.86	1.06	0.00	0.00	0.00	0.69	0.86	1.06	1.71E-03	2.05E-03	2.41E-03	6.99E-03	8.74E-03	0.01	0.00	0.00	0.00	6.99E-03	8.74E-03	0.01	367.97	421.44	479.03	3.73	4.28	4.86	0.00	0.00	0.00	0.00	0.00	0.00
Hungary - Pseudomonas aeruginosa MDRPA BSI - model 46	17.30	20.17	23.31	114.92	158.27	204.25	134.05	178.60	225.63	4.85	6.21	7.67	0.18	0.20	0.24	1.17	1.61	2.07	1.36	1.81	2.29	25.43	28.68	32.27	0.26	0.29	0.33	4.39	6.03	7.87	0.04	0.06	0.08
Hungary - Pseudomonas aeruginosa MDRPA OTH - model 50	0.04	0.05	0.07	0.00	0.00	0.00	0.04	0.05	0.07	2.79E-03	3.73E-03	4.70E-03	3.69E-04	5.35E-04	7.55E-04	0.00	0.00	0.00	3.69E-04	5.35E-04	7.55E-04	10.77	14.47	17.59	0.11	0.15	0.18	0.00	0.00	0.00	0.00	0.00	0.00
Hungary - Pseudomonas aeruginosa MDRPA RESP - model 47	42.36	51.53	61.47	131.65	173.78	222.50	178.02	225.02	282.68	1.78	2.14	2.50	0.43	0.52	0.62	1.34	1.76	2.26	1.81	2.28	2.87	87.95	106.33	125.15	0.89	1.08	1.27	4.93	6.62	8.60	0.05	0.07	0.09
Hungary - Pseudomonas aeruginosa MDRPA SSI - model 49	0.03	0.04	0.06	5.84	8.66	10.90	5.85	8.69	10.96	0.38	0.44	0.50	2.64E-04	4.20E-04	6.42E-04	0.06	0.09	0.11	0.06	0.09	0.11	14.59	19.78	24.12	0.15	0.20	0.24	0.27	0.40	0.51	2.72E-03	4.06E-03	5.17E-03
Hungary - Pseudomonas aeruginosa MDRPA UTI - model 48	0.05	0.07	0.11	0.00	0.00	0.00	0.05	0.07	0.11	1.51E-03	2.04E-03	2.63E-03	5.26E-04	7.53E-04	1.07E-03	0.00	0.00	0.00	5.26E-04	7.53E-04	1.07E-03	28.76	37.00	44.73	0.29	0.38	0.45	0.00	0.00	0.00	0.00	0.00	0.00
Hungary - Staphylococcus aureus MRSA BSI - model 61	204.14	222.46	241.24	1,404.30	1,491.28	1,581.59	1,613.63	1,714.08	1,812.11	3.87	4.03	4.19	2.07	2.26	2.45	14.25	15.13	16.05	16.37	17.39	18.39	404.63	425.32	445.11	4.11	4.32	4.52	71.35	76.37	80.97	0.72	0.77	0.82
Hungary - Staphylococcus aureus MRSA OTH - model 65	1.66	2.05	2.51	0.00	0.00	0.00	1.66	2.05	2.51	2.70E-03	3.28E-03	3.92E-03	0.02	0.02	0.03	0.00	0.00	0.00	0.02	0.02	0.03	564.42	628.05	682.77	5.73	6.37	6.93	0.00	0.00	0.00	0.00	0.00	0.00
Hungary - Staphylococcus aureus MRSA RESP - model 62	207.98	231.91	257.16	407.01	448.65	494.39	618.90	680.82	746.66	1.01	1.07	1.12	2.11	2.35	2.61	4.13	4.55	5.02	6.28	6.91	7.58	580.41	638.86	699.08	5.89	6.48	7.09	20.71	23.01	25.61	0.21	0.23	0.26
Hungary - Staphylococcus aureus MRSA SSI - model 64	1.13	1.50	1.93	270.47	299.60	328.93	271.94	300.94	330.59	0.41	0.43	0.45	0.01	0.02	0.02	2.74	3.04	3.34	2.76	3.05	3.35	635.27	697.43	757.98	6.45	7.08	7.69	17.38	19.36	21.30	0.18	0.20	0.22
Hungary - Staphylococcus aureus MRSA UTI - model 63	0.15	0.19	0.25	0.00	0.00	0.00	0.15	0.19	0.25	1.49E-03	1.88E-03	2.28E-03	1.50E-03	1.96E-03	2.55E-03	0.00	0.00	0.00	1.50E-03	1.96E-03	2.55E-03	87.01	103.83	121.90	0.88	1.05	1.24	0.00	0.00	0.00	0.00	0.00	0.00
Hungary - Streptococcus pneumoniae PMRSP BSI - model 76	1.10	1.37	1.67	8.36	9.30	10.24	9.67	10.66	11.66	4.36	4.80	5.25	0.01	0.01	0.02	0.08	0.09	0.10	0.10	0.11	0.12	2.22	2.22	2.22	0.02	0.02	0.02	0.36	0.40	0.44	3.64E-03	4.05E-03	4.46E-03
Hungary - Streptococcus pneumoniae PMRSP OTH - model 80	7.57E-04	1.51E-03	2.57E-03	0.00	0.00	0.00	7.57E-04	1.51E-03	2.57E-03	9.89E-04	1.94E-03	3.17E-03	7.68E-06	1.54E-05	2.61E-05	0.00	0.00	0.00	7.68E-06	1.54E-05	2.61E-05	0.67	0.79	0.95	6.83E-03	8.04E-03	9.60E-03	0.00	0.00	0.00	0.00	0.00	0.00
Hungary - Streptococcus pneumoniae PMRSP RESP - model 77	2.67	3.56	5.13	5.10	7.02	9.72	7.99	10.58	14.62	1.14	1.27	1.42	0.03	0.04	0.05	0.05	0.07	0.10	0.08	0.11	0.15	6.44	8.35	11.36	0.07	0.08	0.12	0.22	0.30	0.41	2.23E-03	3.05E-03	4.20E-03
Hungary - Streptococcus pneumoniae PMRSP SSI - model 79	0.00	0.00	0.00	0.00	0.00	0.00	0.00	0.00	0.00	NaN	NaN	NaN	0.00	0.00	0.00	0.00	0.00	0.00	0.00	0.00	0.00	0.00	0.00	0.00	0.00	0.00	0.00	0.00	0.00	0.00	0.00	0.00	0.00
Hungary - Streptococcus pneumoniae PMRSP UTI - model 78	0.00	0.00	0.00	0.00	0.00	0.00	0.00	0.00	0.00	NaN	NaN	NaN	0.00	0.00	0.00	0.00	0.00	0.00	0.00	0.00	0.00	0.00	0.00	0.00	0.00	0.00	0.00	0.00	0.00	0.00	0.00	0.00	0.00
Hungary - Streptococcus pneumoniae PRSP BSI - model 71	1.42	1.83	2.33	11.28	12.69	14.03	13.01	14.53	16.06	5.86	6.55	7.23	0.01	0.02	0.02	0.11	0.13	0.14	0.13	0.15	0.16	2.22	2.22	2.22	0.02	0.02	0.02	0.36	0.40	0.44	3.64E-03	4.06E-03	4.46E-03
Hungary - Streptococcus pneumoniae PRSP OTH - model 75	8.23E-04	1.51E-03	2.66E-03	0.00	0.00	0.00	8.23E-04	1.51E-03	2.66E-03	1.05E-03	1.94E-03	3.18E-03	8.35E-06	1.53E-05	2.70E-05	0.00	0.00	0.00	8.35E-06	1.53E-05	2.70E-05	0.67	0.79	0.95	6.84E-03	8.02E-03	9.62E-03	0.00	0.00	0.00	0.00	0.00	0.00
Hungary - Streptococcus pneumoniae PRSP RESP - model 72	3.40	4.77	7.70	6.82	9.24	14.44	10.52	14.03	21.82	1.33	1.71	2.15	0.03	0.05	0.08	0.07	0.09	0.15	0.11	0.14	0.22	6.46	8.28	11.69	0.07	0.08	0.12	0.22	0.30	0.42	2.25E-03	3.01E-03	4.23E-03
Hungary - Streptococcus pneumoniae PRSP SSI - model 74	0.00	0.00	0.00	0.00	0.00	0.00	0.00	0.00	0.00	NaN	NaN	NaN	0.00	0.00	0.00	0.00	0.00	0.00	0.00	0.00	0.00	0.00	0.00	0.00	0.00	0.00	0.00	0.00	0.00	0.00	0.00	0.00	0.00
Hungary - Streptococcus pneumoniae PRSP UTI - model 73	0.00	0.00	0.00	0.00	0.00	0.00	0.00	0.00	0.00	NaN	NaN	NaN	0.00	0.00	0.00	0.00	0.00	0.00	0.00	0.00	0.00	0.00	0.00	0.00	0.00	0.00	0.00	0.00	0.00	0.00	0.00	0.00	0.00
**Total (Median & 95% UIs)**	2,296.17	2,556.67	2,834.78	11,490.67	13,255.37	15,156.49	13,874.92	15,812.92	17,905.14		-		23.30	25.94	28.76	116.59	134.50	153.79	140.78	160.45	181.68	9,189.16	10,270.61	11,367.59	93.24	104.21	115.34	470.46	543.11	624.03	4.77	5.51	6.33
**Total (average)**		**2,558.99**			**13,292.76**			**15,851.75**			-			**25.96**			**134.88**			**160.84**			**10,270.46**			**104.21**			**544.36**			**5.52**	

Infection	YLD			YLL			DALY			DALY per Case			YLD per 100,000		
Colistin resistant *Acinetobacter spp.*	0.00	0.00	0.00	0.00	0.00	0.00	0.00	0.00	0.00	0.00	0.00	0.00	0.00	0.00	0.00
Carbapenem resistant *Acinetobacter spp.* (excluding those resistant to colistin)	7.28	8.99	11.04	32.08	48.54	65.89	40.37	57.46	76.02	8.07	10.94	13.94	0.16	0.19	0.24
Aminoglycosides and fluoroquinolones resistant *Acinetobacter* spp. (excluding those resistant to colistin and/or carbapenem)	0.00	0.00	0.00	0.00	0.00	0.00	0.00	0.00	0.00	0.00	0.00	0.00	0.00	0.00	0.00
Vancomycin resistant Enterococci (*E. faecalis* and *E. faecium*)	151.85	178.31	208.15	1,430.17	1,670.34	1,955.75	1,592.95	1,848.81	2,163.73	6.62	7.12	7.66	3.28	3.85	4.50
Colistin resistant *Escherichia coli*	0.00	0.00	0.00	0.00	0.00	0.00	0.00	0.00	0.00	0.00	0.00	0.00	0.00	0.00	0.00
Carbapenem resistant *Escherichia coli* (excluding those resistant to colistin)	1.77	3.00	4.66	17.06	33.14	52.73	19.13	36.13	56.70	22.78	35.63	47.28	0.04	0.06	0.10
Third-generation cephalosporin resistant *Escherichia coli* (excluding those resistant to colistin and/or carbapenem)	191.54	213.70	236.50	1,146.66	1,268.18	1,402.65	1,344.06	1,482.28	1,628.82	4.70	5.13	5.58	4.14	4.62	5.11
Colistin resistant *Klebsiella pneumoniae*	0.00	0.00	0.00	0.00	0.00	0.00	0.00	0.00	0.00	0.00	0.00	0.00	0.00	0.00	0.00
Carbapenem resistant *Klebsiella pneumoniae* (excluding those resistant to colistin)	0.97	1.50	2.20	9.50	15.86	24.45	10.65	17.38	26.45	6.05	8.81	12.08	0.02	0.03	0.05
Third-generation cephalosporin resistant *Klebsiella pneumoniae* (excluding those resistant to colistin and/or carbapenem)	49.97	55.54	61.50	246.35	267.45	287.67	298.61	323.39	346.23	5.74	6.05	6.35	1.08	1.20	1.33
Colistin resistant *Pseudomonas aeruginosa*	0.00	0.00	0.00	0.00	0.00	0.00	0.00	0.00	0.00	0.00	0.00	0.00	0.00	0.00	0.00
Carbapenem resistant *Pseudomonas aeruginosa* (excluding those resistant to colistin)	48.17	57.39	66.96	216.34	292.03	373.06	269.29	348.84	435.17	8.45	10.79	13.06	1.04	1.24	1.45
Pseudomonas aeruginosa resistant to three or more antimicrobial groups (excluding those resistant to colistin and/or carbapenem)	1.99	3.59	5.65	6.11	16.51	33.32	8.56	20.03	38.14	5.58	11.23	17.18	0.04	0.08	0.12
Methicillin resistant *Staphylococcus aureus*	208.93	227.69	246.67	1.028.67	1.097.22	1.163.36	1.242.77	1.324.80	1.403.29	5.50	5.75	6.01	4.51	4.92	5.33
Penicillin and macrolides resistant *Streptococcus pneumoniae* (excluding those only resistant to penicillin)	0.65	0.94	1.51	2.40	3.00	4.06	3.15	3.93	5.39	2.15	2.44	2.73	0.01	0.02	0.03
Penicillin tesistant *Streptococcus pneumoniae* (excluding those resistant to macrolides)	0.00	0.00	0.00	0.00	0.00	0.00	0.00	0.00	0.00	0.00	0.00	0.00	0.00	0.00	0.00
Total	663.13	750.65	844.83	4,135.35	4,712.28	5,362.94	4,829.53	5,463.04	6,179.93				14.33	16.22	18.25

Infection	YLL per 100,000			DALY per 100,000			Cases per year			Incidence per 100,000			Deaths per year			Mortality per 100,000		
Colistin resistant *Acinetobacter spp.*	0.00	0.00	0.00	0.00	0.00	0.00	0.00	0.00	0.00	0.00	0.00	0.00	0.00	0.00	0.00	0.00	0.00	0.00
Carbapenem resistant *Acinetobacter spp.* (excluding those resistant to colistin)	0.69	1.05	1.42	0.87	1.24	1.64	12.36	15.43	18.37	0.27	0.33	0.40	0.93	1.35	1.78	0.02	0.03	0.04
Aminoglycosides and fluoroquinolones resistant *Acinetobacter* spp. (excluding those resistant to colistin and/or carbapenem)	0.00	0.00	0.00	0.00	0.00	0.00	0.00	0.00	0.00	0.00	0.00	0.00	0.00	0.00	0.00	0.00	0.00	0.00
Vancomycin resistant Enterococci (*E. faecalis* and *E. faecium*)	30.90	36.08	42.25	34.41	39.94	46.74	836.27	1,001.78	1,173.54	18.07	21.64	25.35	56.45	66.35	77.73	1.22	1.43	1.68
Colistin resistant *Escherichia coli*	0.00	0.00	0.00	0.00	0.00	0.00	0.00	0.00	0.00	0.00	0.00	0.00	0.00	0.00	0.00	0.00	0.00	0.00
Carbapenem resistant *Escherichia coli* (excluding those resistant to colistin)	0.37	0.72	1.14	0.41	0.78	1.22	4.75	8.01	11.44	0.10	0.17	0.25	0.22	0.42	0.67	0.00	0.01	0.01
Third-generation cephalosporin resistant *Escherichia coli* (excluding those resistant to colistin and/or carbapenem)	24.77	27.40	30.30	29.04	32.02	35.19	2,051.92	2,302.37	2,567.52	44.33	49.74	55.47	62.50	71.03	80.27	1.35	1.53	1.73
Colistin resistant *Klebsiella pneumoniae*	0.00	0.00	0.00	0.00	0.00	0.00	0.00	0.00	0.00	0.00	0.00	0.00	0.00	0.00	0.00	0.00	0.00	0.00
Carbapenem resistant *Klebsiella pneumoniae* (excluding those resistant to colistin)	0.21	0.34	0.53	0.23	0.38	0.57	3.70	6.02	8.50	0.08	0.13	0.18	0.51	0.81	1.20	0.01	0.02	0.03
Third-generation cephalosporin resistant *Klebsiella pneumoniae* (excluding those resistant to colistin and/or carbapenem)	5.32	5.78	6.21	6.45	6.99	7.48	199.90	221.41	242.30	4.32	4.78	5.23	11.13	12.09	13.01	0.24	0.26	0.28
Colistin resistant *Pseudomonas aeruginosa*	0.00	0.00	0.00	0.00	0.00	0.00	0.00	0.00	0.00	0.00	0.00	0.00	0.00	0.00	0.00	0.00	0.00	0.00
Carbapenem resistant *Pseudomonas aeruginosa* (excluding those resistant to colistin)	4.67	6.31	8.06	5.82	7.54	9.40	122.04	142.70	163.37	2.64	3.08	3.53	7.66	9.76	12.16	0.17	0.21	0.26
Pseudomonas aeruginosa resistant to three or more antimicrobial groups (excluding those resistant to colistin and/or carbapenem)	0.13	0.36	0.72	0.18	0.43	0.82	4.01	7.98	11.93	0.09	0.17	0.26	0.18	0.48	0.96	0.00	0.01	0.02
Methicillin resistant *Staphylococcus aureus*	22.22	23.70	25.13	26.85	28.62	30.32	1.082.96	1.182.35	1.281.80	23.40	25.54	27.69	51.87	56.25	60.48	1.12	1.22	1.31
Penicillin and macrolides resistant *Streptococcus pneumoniae* (excluding those only resistant to penicillin)	0.05	0.06	0.09	0.07	0.08	0.12	4.18	5.18	7.69	0.09	0.11	0.17	0.26	0.32	0.44	0.01	0.01	0.01
Penicillin tesistant *Streptococcus pneumoniae* (excluding those resistant to macrolides)	0.00	0.00	0.00	0.00	0.00	0.00	0.00	0.00	0.00	0.00	0.00	0.00	0.00	0.00	0.00	0.00	0.00	0.00
Total	89.34	101.80	115.86	104.33	118.02	133.51	4,322.08	4,893.24	5,486.45	93.37	105.71	118.52	191.69	218.87	248.69	4.14	4.73	5.37

Model	YLD			YLL			DALY			DALY per Case			YLD per 100,000		
Ireland - Acinetobacter spp. CRACI BSI - model 36	4.32	5.12	6.25	25.59	40.04	55.32	30.74	45.09	60.80	5.84	8.44	11.16	0.09	0.11	0.13
Ireland - Acinetobacter spp. CRACI OTH - model 40	3.65E-04	7.12E-04	1.18E-03	0.00	0.00	0.00	3.65E-04	7.12E-04	1.18E-03	2.50E-03	3.78E-03	5.23E-03	7.89E-06	1.54E-05	2.56E-05
Ireland - Acinetobacter spp. CRACI RESP - model 37	2.96	3.85	4.78	5.79	7.48	9.25	8.92	11.34	13.90	1.74	1.96	2.19	0.06	0.08	0.10
Ireland - Acinetobacter spp. CRACI SSI - model 39	2.18E-03	4.09E-03	6.72E-03	0.70	1.02	1.31	0.71	1.02	1.32	0.48	0.53	0.58	4.71E-05	8.84E-05	1.45E-04
Ireland - Acinetobacter spp. CRACI UTI - model 38	2.62E-03	4.37E-03	6.55E-03	0.00	0.00	0.00	2.62E-03	4.37E-03	6.55E-03	1.37E-03	2.04E-03	2.78E-03	5.66E-05	9.43E-05	1.41E-04
Ireland - Acinetobacter spp. ColRACI BSI - model 41	0.00	0.00	0.00	0.00	0.00	0.00	0.00	0.00	0.00	NaN	NaN	NaN	0.00	0.00	0.00
Ireland - Acinetobacter spp. ColRACI OTH - model 45	0.00	0.00	0.00	0.00	0.00	0.00	0.00	0.00	0.00	NaN	NaN	NaN	0.00	0.00	0.00
Ireland - Acinetobacter spp. ColRACI RESP - model 42	0.00	0.00	0.00	0.00	0.00	0.00	0.00	0.00	0.00	NaN	NaN	NaN	0.00	0.00	0.00
Ireland - Acinetobacter spp. ColRACI SSI - model 44	0.00	0.00	0.00	0.00	0.00	0.00	0.00	0.00	0.00	NaN	NaN	NaN	0.00	0.00	0.00
Ireland - Acinetobacter spp. ColRACI UTI - model 43	0.00	0.00	0.00	0.00	0.00	0.00	0.00	0.00	0.00	NaN	NaN	NaN	0.00	0.00	0.00
Ireland - Acinetobacter spp. MDRACI BSI - model 31	0.00	0.00	0.00	0.00	0.00	0.00	0.00	0.00	0.00	NaN	NaN	NaN	0.00	0.00	0.00
Ireland - Acinetobacter spp. MDRACI OTH - model 35	0.00	0.00	0.00	0.00	0.00	0.00	0.00	0.00	0.00	NaN	NaN	NaN	0.00	0.00	0.00
Ireland - Acinetobacter spp. MDRACI RESP - model 32	0.00	0.00	0.00	0.00	0.00	0.00	0.00	0.00	0.00	NaN	NaN	NaN	0.00	0.00	0.00
Ireland - Acinetobacter spp. MDRACI SSI - model 34	0.00	0.00	0.00	0.00	0.00	0.00	0.00	0.00	0.00	NaN	NaN	NaN	0.00	0.00	0.00
Ireland - Acinetobacter spp. MDRACI UTI - model 33	0.00	0.00	0.00	0.00	0.00	0.00	0.00	0.00	0.00	NaN	NaN	NaN	0.00	0.00	0.00
Ireland - Enterococcus faecalis and E. faecium VRE BSI - model 66	150.80	176.89	206.22	1,307.89	1,514.03	1,764.99	1,469.26	1,691.07	1,971.15	6.20	6.67	7.17	3.26	3.82	4.45
Ireland - Enterococcus faecalis and E. faecium VRE OTH - model 70	0.33	0.44	0.57	0.00	0.00	0.00	0.33	0.44	0.57	1.33E-03	1.64E-03	2.02E-03	7.24E-03	9.56E-03	0.01
Ireland - Enterococcus faecalis and E. faecium VRE RESP - model 67	0.00	0.00	0.00	0.00	0.00	0.00	0.00	0.00	0.00	NaN	NaN	NaN	0.00	0.00	0.00
Ireland - Enterococcus faecalis and E. faecium VRE SSI - model 69	0.54	0.74	1.06	122.28	156.32	190.77	123.17	157.05	191.70	0.42	0.45	0.49	0.01	0.02	0.02
Ireland - Enterococcus faecalis and E. faecium VRE UTI - model 68	0.18	0.24	0.30	0.00	0.00	0.00	0.18	0.24	0.30	1.56E-03	1.88E-03	2.21E-03	3.99E-03	5.24E-03	6.56E-03
Ireland - Escherichia coli 3GCREC BSI - model 16	146.80	161.05	175.35	946.19	1,036.63	1,138.59	1,098.28	1,198.11	1,304.48	3.40	3.71	4.01	3.17	3.48	3.79
Ireland - Escherichia coli 3GCREC OTH - model 20	0.26	0.35	0.48	0.00	0.00	0.00	0.26	0.35	0.48	2.69E-03	3.32E-03	4.09E-03	5.69E-03	7.64E-03	0.01
Ireland - Escherichia coli 3GCREC RESP - model 17	41.85	48.84	56.30	81.33	94.64	108.77	123.87	143.45	164.31	0.92	1.01	1.14	0.90	1.06	1.22
Ireland - Escherichia coli 3GCREC SSI - model 19	0.54	0.73	0.98	119.14	136.91	155.29	119.57	137.64	156.16	0.38	0.40	0.43	0.01	0.02	0.02
Ireland - Escherichia coli 3GCREC UTI - model 18	2.09	2.73	3.39	0.00	0.00	0.00	2.09	2.73	3.39	1.60E-03	1.97E-03	2.36E-03	0.05	0.06	0.07
Ireland - Escherichia coli CREC BSI - model 21	1.47	2.21	3.32	15.99	30.68	48.93	17.73	32.87	51.62	18.41	30.63	41.66	0.03	0.05	0.07
Ireland - Escherichia coli CREC OTH - model 25	5.58E-04	1.87E-03	5.49E-03	0.00	0.00	0.00	5.58E-04	1.87E-03	5.49E-03	1.15E-03	3.17E-03	7.32E-03	1.20E-05	4.03E-05	1.19E-04
Ireland - Escherichia coli CREC RESP - model 22	0.30	0.78	1.31	0.59	1.54	2.48	0.91	2.33	3.73	3.65	4.28	4.90	6.50E-03	0.02	0.03
Ireland - Escherichia coli CREC SSI - model 24	5.81E-04	2.52E-03	6.48E-03	0.49	0.91	1.33	0.49	0.92	1.33	0.71	0.71	0.71	1.26E-05	5.44E-05	1.40E-04
Ireland - Escherichia coli CREC UTI - model 23	3.17E-03	8.94E-03	0.02	0.00	0.00	0.00	3.17E-03	8.94E-03	0.02	8.35E-04	2.01E-03	4.05E-03	6.84E-05	1.93E-04	4.49E-04
Ireland - Escherichia coli ColREC BSI - model 26	0.00	0.00	0.00	0.00	0.00	0.00	0.00	0.00	0.00	NaN	NaN	NaN	0.00	0.00	0.00
Ireland - Escherichia coli ColREC OTH - model 30	0.00	0.00	0.00	0.00	0.00	0.00	0.00	0.00	0.00	NaN	NaN	NaN	0.00	0.00	0.00
Ireland - Escherichia coli ColREC RESP - model 27	0.00	0.00	0.00	0.00	0.00	0.00	0.00	0.00	0.00	NaN	NaN	NaN	0.00	0.00	0.00
Ireland - Escherichia coli ColREC SSI - model 29	0.00	0.00	0.00	0.00	0.00	0.00	0.00	0.00	0.00	NaN	NaN	NaN	0.00	0.00	0.00
Ireland - Escherichia coli ColREC UTI - model 28	0.00	0.00	0.00	0.00	0.00	0.00	0.00	0.00	0.00	NaN	NaN	NaN	0.00	0.00	0.00
Ireland - Klebsiella pneumoniae 3GCRKP BSI - model 1	31.45	34.52	37.90	201.45	216.53	230.63	234.86	251.32	266.22	4.14	4.34	4.53	0.68	0.75	0.82
Ireland - Klebsiella pneumoniae 3GCRKP OTH - model 5	0.06	0.08	0.10	0.00	0.00	0.00	0.06	0.08	0.10	2.73E-03	3.32E-03	4.03E-03	1.30E-03	1.65E-03	2.06E-03
Ireland - Klebsiella pneumoniae 3GCRKP RESP - model 2	18.32	20.76	23.27	35.61	40.10	44.63	54.27	60.99	67.27	1.14	1.23	1.31	0.40	0.45	0.50
Ireland - Klebsiella pneumoniae 3GCRKP SSI - model 4	0.04	0.05	0.06	9.28	10.83	12.41	9.32	10.87	12.47	0.45	0.48	0.50	7.81E-04	1.06E-03	1.39E-03
Ireland - Klebsiella pneumoniae 3GCRKP UTI - model 3	0.10	0.13	0.17	0.00	0.00	0.00	0.10	0.13	0.17	1.54E-03	1.95E-03	2.36E-03	2.25E-03	2.86E-03	3.59E-03
Ireland - Klebsiella pneumoniae CRKP BSI - model 6	0.82	1.16	1.63	9.13	15.02	23.08	10.12	16.20	24.56	5.01	7.43	10.28	0.02	0.03	0.04
Ireland - Klebsiella pneumoniae CRKP OTH - model 10	1.24E-03	3.20E-03	7.06E-03	0.00	0.00	0.00	1.24E-03	3.20E-03	7.06E-03	1.60E-03	3.33E-03	6.08E-03	2.67E-05	6.91E-05	1.52E-04
Ireland - Klebsiella pneumoniae CRKP RESP - model 7	0.14	0.33	0.55	0.28	0.65	1.08	0.44	0.98	1.58	0.76	1.07	1.47	3.12E-03	7.19E-03	0.01
Ireland - Klebsiella pneumoniae CRKP SSI - model 9	4.08E-04	1.25E-03	2.72E-03	0.09	0.19	0.29	0.09	0.19	0.30	0.29	0.30	0.32	8.82E-06	2.71E-05	5.88E-05
Ireland - Klebsiella pneumoniae CRKP UTI - model 8	1.13E-03	2.58E-03	5.27E-03	0.00	0.00	0.00	1.13E-03	2.58E-03	5.27E-03	1.02E-03	2.03E-03	3.50E-03	2.45E-05	5.57E-05	1.14E-04
Ireland - Klebsiella pneumoniae ColRKP BSI - model 11	0.00	0.00	0.00	0.00	0.00	0.00	0.00	0.00	0.00	NaN	NaN	NaN	0.00	0.00	0.00
Ireland - Klebsiella pneumoniae ColRKP OTH - model 15	0.00	0.00	0.00	0.00	0.00	0.00	0.00	0.00	0.00	NaN	NaN	NaN	0.00	0.00	0.00
Ireland - Klebsiella pneumoniae ColRKP RESP - model 12	0.00	0.00	0.00	0.00	0.00	0.00	0.00	0.00	0.00	NaN	NaN	NaN	0.00	0.00	0.00
Ireland - Klebsiella pneumoniae ColRKP SSI - model 14	0.00	0.00	0.00	0.00	0.00	0.00	0.00	0.00	0.00	NaN	NaN	NaN	0.00	0.00	0.00
Ireland - Klebsiella pneumoniae ColRKP UTI - model 13	0.00	0.00	0.00	0.00	0.00	0.00	0.00	0.00	0.00	NaN	NaN	NaN	0.00	0.00	0.00
Ireland - Pseudomonas aeruginosa CRPA BSI - model 51	13.77	16.10	18.67	101.29	137.88	176.47	116.60	154.08	193.42	5.94	7.74	9.48	0.30	0.35	0.40
Ireland - Pseudomonas aeruginosa CRPA OTH - model 55	0.03	0.04	0.05	0.00	0.00	0.00	0.03	0.04	0.05	2.89E-03	3.66E-03	4.43E-03	5.82E-04	7.94E-04	1.02E-03
Ireland - Pseudomonas aeruginosa CRPA RESP - model 52	34.32	41.17	48.14	109.99	147.60	188.62	147.51	188.09	233.62	2.07	2.56	3.06	0.74	0.89	1.04
Ireland - Pseudomonas aeruginosa CRPA SSI - model 54	0.02	0.03	0.04	5.06	6.55	7.97	5.10	6.58	8.03	0.44	0.48	0.52	4.30E-04	6.20E-04	8.68E-04
Ireland - Pseudomonas aeruginosa CRPA UTI - model 53	0.04	0.05	0.07	0.00	0.00	0.00	0.04	0.05	0.07	1.63E-03	2.05E-03	2.51E-03	8.39E-04	1.12E-03	1.46E-03
Ireland - Pseudomonas aeruginosa ColRPA BSI - model 56	0.00	0.00	0.00	0.00	0.00	0.00	0.00	0.00	0.00	NaN	NaN	NaN	0.00	0.00	0.00
Ireland - Pseudomonas aeruginosa ColRPA OTH - model 60	0.00	0.00	0.00	0.00	0.00	0.00	0.00	0.00	0.00	NaN	NaN	NaN	0.00	0.00	0.00
Ireland - Pseudomonas aeruginosa ColRPA RESP - model 57	0.00	0.00	0.00	0.00	0.00	0.00	0.00	0.00	0.00	NaN	NaN	NaN	0.00	0.00	0.00
Ireland - Pseudomonas aeruginosa ColRPA SSI - model 59	0.00	0.00	0.00	0.00	0.00	0.00	0.00	0.00	0.00	NaN	NaN	NaN	0.00	0.00	0.00
Ireland - Pseudomonas aeruginosa ColRPA UTI - model 58	0.00	0.00	0.00	0.00	0.00	0.00	0.00	0.00	0.00	NaN	NaN	NaN	0.00	0.00	0.00
Ireland - Pseudomonas aeruginosa MDRPA BSI - model 46	0.67	1.01	1.60	2.81	8.00	15.77	3.66	9.00	17.23	3.64	8.11	12.85	0.01	0.02	0.03
Ireland - Pseudomonas aeruginosa MDRPA OTH - model 50	4.92E-04	1.92E-03	4.59E-03	0.00	0.00	0.00	4.92E-04	1.92E-03	4.59E-03	1.59E-03	3.80E-03	6.46E-03	1.06E-05	4.14E-05	9.91E-05
Ireland - Pseudomonas aeruginosa MDRPA RESP - model 47	1.32	2.57	4.03	3.23	8.27	17.16	4.81	10.78	20.50	1.63	2.80	4.00	0.03	0.06	0.09
Ireland - Pseudomonas aeruginosa MDRPA SSI - model 49	2.69E-04	1.42E-03	4.17E-03	0.08	0.24	0.39	0.08	0.25	0.40	0.31	0.31	0.32	5.81E-06	3.07E-05	9.00E-05
Ireland - Pseudomonas aeruginosa MDRPA UTI - model 48	7.77E-04	2.63E-03	6.53E-03	0.00	0.00	0.00	7.77E-04	2.63E-03	6.53E-03	8.43E-04	1.96E-03	3.76E-03	1.68E-05	5.67E-05	1.41E-04
Ireland - Staphylococcus aureus MRSA BSI - model 61	102.28	110.40	119.11	702.34	740.38	777.90	809.44	850.68	892.20	4.06	4.23	4.40	2.21	2.38	2.57
Ireland - Staphylococcus aureus MRSA OTH - model 65	0.79	0.97	1.17	0.00	0.00	0.00	0.79	0.97	1.17	2.70E-03	3.27E-03	3.91E-03	0.02	0.02	0.03
Ireland - Staphylococcus aureus MRSA RESP - model 62	105.26	115.53	125.36	205.03	224.12	241.46	310.66	339.67	365.08	1.05	1.12	1.19	2.27	2.50	2.71
Ireland - Staphylococcus aureus MRSA SSI - model 64	0.53	0.70	0.91	121.30	132.73	144.00	121.81	133.39	144.72	0.39	0.40	0.42	0.01	0.02	0.02
Ireland - Staphylococcus aureus MRSA UTI - model 63	0.07	0.09	0.12	0.00	0.00	0.00	0.07	0.09	0.12	1.52E-03	1.88E-03	2.25E-03	1.52E-03	1.98E-03	2.54E-03
Ireland - Streptococcus pneumoniae PMRSP BSI - model 76	0.19	0.26	0.34	1.52	1.72	1.93	1.76	1.98	2.21	1.71	1.93	2.15	4.13E-03	5.54E-03	7.37E-03
Ireland - Streptococcus pneumoniae PMRSP OTH - model 80	2.60E-04	6.79E-04	1.40E-03	0.00	0.00	0.00	2.60E-04	6.79E-04	1.40E-03	7.28E-04	1.86E-03	3.65E-03	5.61E-06	1.47E-05	3.02E-05
Ireland - Streptococcus pneumoniae PMRSP RESP - model 77	0.46	0.68	1.17	0.89	1.28	2.12	1.39	1.95	3.18	0.44	0.51	0.58	9.90E-03	0.01	0.03
Ireland - Streptococcus pneumoniae PMRSP SSI - model 79	0.00	0.00	0.00	0.00	0.00	0.00	0.00	0.00	0.00	NaN	NaN	NaN	0.00	0.00	0.00
Ireland - Streptococcus pneumoniae PMRSP UTI - model 78	0.00	0.00	0.00	0.00	0.00	0.00	0.00	0.00	0.00	NaN	NaN	NaN	0.00	0.00	0.00
Ireland - Streptococcus pneumoniae PRSP BSI - model 71	0.00	0.00	0.00	0.00	0.00	0.00	0.00	0.00	0.00	NaN	NaN	NaN	0.00	0.00	0.00
Ireland - Streptococcus pneumoniae PRSP OTH - model 75	0.00	0.00	0.00	0.00	0.00	0.00	0.00	0.00	0.00	NaN	NaN	NaN	0.00	0.00	0.00
Ireland - Streptococcus pneumoniae PRSP RESP - model 72	0.00	0.00	0.00	0.00	0.00	0.00	0.00	0.00	0.00	NaN	NaN	NaN	0.00	0.00	0.00
Ireland - Streptococcus pneumoniae PRSP SSI - model 74	0.00	0.00	0.00	0.00	0.00	0.00	0.00	0.00	0.00	NaN	NaN	NaN	0.00	0.00	0.00
Ireland - Streptococcus pneumoniae PRSP UTI - model 73	0.00	0.00	0.00	0.00	0.00	0.00	0.00	0.00	0.00	NaN	NaN	NaN	0.00	0.00	0.00
**Total (Median & 95% UIs)**	663.13	750.65	844.83	4,135.35	4,712.28	5,362.94	4,829.53	5,463.04	6,179.93		-		14.33	16.22	18.25
**Total (average)**		**751.71**			**4,722.69**			**5,474.41**			-			**16.24**	

Model	YLL per 100,000			DALY per 100,000			Cases per year			Incidence per 100,000			Deaths per year			Mortality per 100,000		
Ireland - Acinetobacter spp. CRACI BSI - model 36	0.55	0.87	1.20	0.66	0.97	1.31	4.76	5.34	5.97	0.10	0.12	0.13	0.74	1.10	1.48	0.02	0.02	0.03
Ireland - Acinetobacter spp. CRACI OTH - model 40	0.00	0.00	0.00	7.89E-06	1.54E-05	2.56E-05	0.11	0.19	0.28	2.46E-03	4.19E-03	6.11E-03	0.00	0.00	0.00	0.00	0.00	0.00
Ireland - Acinetobacter spp. CRACI RESP - model 37	0.13	0.16	0.20	0.19	0.25	0.30	4.60	5.79	6.85	0.10	0.13	0.15	0.16	0.21	0.25	3.52E-03	4.50E-03	5.41E-03
Ireland - Acinetobacter spp. CRACI SSI - model 39	0.02	0.02	0.03	0.02	0.02	0.03	1.33	1.92	2.45	0.03	0.04	0.05	0.02	0.04	0.05	5.21E-04	8.16E-04	1.10E-03
Ireland - Acinetobacter spp. CRACI UTI - model 38	0.00	0.00	0.00	5.66E-05	9.43E-05	1.41E-04	1.55	2.18	2.82	0.03	0.05	0.06	0.00	0.00	0.00	0.00	0.00	0.00
Ireland - Acinetobacter spp. ColRACI BSI - model 41	0.00	0.00	0.00	0.00	0.00	0.00	0.00	0.00	0.00	0.00	0.00	0.00	0.00	0.00	0.00	0.00	0.00	0.00
Ireland - Acinetobacter spp. ColRACI OTH - model 45	0.00	0.00	0.00	0.00	0.00	0.00	0.00	0.00	0.00	0.00	0.00	0.00	0.00	0.00	0.00	0.00	0.00	0.00
Ireland - Acinetobacter spp. ColRACI RESP - model 42	0.00	0.00	0.00	0.00	0.00	0.00	0.00	0.00	0.00	0.00	0.00	0.00	0.00	0.00	0.00	0.00	0.00	0.00
Ireland - Acinetobacter spp. ColRACI SSI - model 44	0.00	0.00	0.00	0.00	0.00	0.00	0.00	0.00	0.00	0.00	0.00	0.00	0.00	0.00	0.00	0.00	0.00	0.00
Ireland - Acinetobacter spp. ColRACI UTI - model 43	0.00	0.00	0.00	0.00	0.00	0.00	0.00	0.00	0.00	0.00	0.00	0.00	0.00	0.00	0.00	0.00	0.00	0.00
Ireland - Acinetobacter spp. MDRACI BSI - model 31	0.00	0.00	0.00	0.00	0.00	0.00	0.00	0.00	0.00	0.00	0.00	0.00	0.00	0.00	0.00	0.00	0.00	0.00
Ireland - Acinetobacter spp. MDRACI OTH - model 35	0.00	0.00	0.00	0.00	0.00	0.00	0.00	0.00	0.00	0.00	0.00	0.00	0.00	0.00	0.00	0.00	0.00	0.00
Ireland - Acinetobacter spp. MDRACI RESP - model 32	0.00	0.00	0.00	0.00	0.00	0.00	0.00	0.00	0.00	0.00	0.00	0.00	0.00	0.00	0.00	0.00	0.00	0.00
Ireland - Acinetobacter spp. MDRACI SSI - model 34	0.00	0.00	0.00	0.00	0.00	0.00	0.00	0.00	0.00	0.00	0.00	0.00	0.00	0.00	0.00	0.00	0.00	0.00
Ireland - Acinetobacter spp. MDRACI UTI - model 33	0.00	0.00	0.00	0.00	0.00	0.00	0.00	0.00	0.00	0.00	0.00	0.00	0.00	0.00	0.00	0.00	0.00	0.00
Ireland - Enterococcus faecalis and E. faecium VRE BSI - model 66	28.25	32.71	38.13	31.74	36.53	42.58	218.20	253.95	296.47	4.71	5.49	6.40	50.05	58.04	67.61	1.08	1.25	1.46
Ireland - Enterococcus faecalis and E. faecium VRE OTH - model 70	0.00	0.00	0.00	7.24E-03	9.56E-03	0.01	226.85	271.50	315.57	4.90	5.87	6.82	0.00	0.00	0.00	0.00	0.00	0.00
Ireland - Enterococcus faecalis and E. faecium VRE RESP - model 67	0.00	0.00	0.00	0.00	0.00	0.00	0.00	0.00	0.00	0.00	0.00	0.00	0.00	0.00	0.00	0.00	0.00	0.00
Ireland - Enterococcus faecalis and E. faecium VRE SSI - model 69	2.64	3.38	4.12	2.66	3.39	4.14	284.64	347.61	410.07	6.15	7.51	8.86	6.40	8.30	10.12	0.14	0.18	0.22
Ireland - Enterococcus faecalis and E. faecium VRE UTI - model 68	0.00	0.00	0.00	3.99E-03	5.24E-03	6.56E-03	106.59	128.72	151.43	2.30	2.78	3.27	0.00	0.00	0.00	0.00	0.00	0.00
Ireland - Escherichia coli 3GCREC BSI - model 16	20.44	22.39	24.60	23.73	25.88	28.18	301.40	322.67	347.53	6.51	6.97	7.51	50.02	55.94	62.79	1.08	1.21	1.36
Ireland - Escherichia coli 3GCREC OTH - model 20	0.00	0.00	0.00	5.69E-03	7.64E-03	0.01	87.76	106.33	127.30	1.90	2.30	2.75	0.00	0.00	0.00	0.00	0.00	0.00
Ireland - Escherichia coli 3GCREC RESP - model 17	1.76	2.04	2.35	2.68	3.10	3.55	117.42	141.56	166.21	2.54	3.06	3.59	4.16	5.09	6.02	0.09	0.11	0.13
Ireland - Escherichia coli 3GCREC SSI - model 19	2.57	2.96	3.35	2.58	2.97	3.37	296.25	342.05	385.08	6.40	7.39	8.32	8.32	10.00	11.46	0.18	0.22	0.25
Ireland - Escherichia coli 3GCREC UTI - model 18	0.00	0.00	0.00	0.05	0.06	0.07	1,249.08	1,389.76	1,541.40	26.98	30.02	33.30	0.00	0.00	0.00	0.00	0.00	0.00
Ireland - Escherichia coli CREC BSI - model 21	0.35	0.66	1.06	0.38	0.71	1.12	0.82	1.08	1.42	0.02	0.02	0.03	0.20	0.39	0.62	4.39E-03	8.43E-03	0.01
Ireland - Escherichia coli CREC OTH - model 25	0.00	0.00	0.00	1.20E-05	4.03E-05	1.19E-04	0.28	0.62	0.97	6.10E-03	0.01	0.02	0.00	0.00	0.00	0.00	0.00	0.00
Ireland - Escherichia coli CREC RESP - model 22	0.01	0.03	0.05	0.02	0.05	0.08	0.21	0.55	0.86	4.55E-03	0.01	0.02	7.48E-03	0.02	0.03	1.62E-04	4.24E-04	6.80E-04
Ireland - Escherichia coli CREC SSI - model 24	0.01	0.02	0.03	0.01	0.02	0.03	0.69	1.29	1.87	0.01	0.03	0.04	6.20E-03	0.01	0.02	1.34E-04	2.50E-04	3.64E-04
Ireland - Escherichia coli CREC UTI - model 23	0.00	0.00	0.00	6.84E-05	1.93E-04	4.49E-04	2.75	4.48	6.32	0.06	0.10	0.14	0.00	0.00	0.00	0.00	0.00	0.00
Ireland - Escherichia coli ColREC BSI - model 26	0.00	0.00	0.00	0.00	0.00	0.00	0.00	0.00	0.00	0.00	0.00	0.00	0.00	0.00	0.00	0.00	0.00	0.00
Ireland - Escherichia coli ColREC OTH - model 30	0.00	0.00	0.00	0.00	0.00	0.00	0.00	0.00	0.00	0.00	0.00	0.00	0.00	0.00	0.00	0.00	0.00	0.00
Ireland - Escherichia coli ColREC RESP - model 27	0.00	0.00	0.00	0.00	0.00	0.00	0.00	0.00	0.00	0.00	0.00	0.00	0.00	0.00	0.00	0.00	0.00	0.00
Ireland - Escherichia coli ColREC SSI - model 29	0.00	0.00	0.00	0.00	0.00	0.00	0.00	0.00	0.00	0.00	0.00	0.00	0.00	0.00	0.00	0.00	0.00	0.00
Ireland - Escherichia coli ColREC UTI - model 28	0.00	0.00	0.00	0.00	0.00	0.00	0.00	0.00	0.00	0.00	0.00	0.00	0.00	0.00	0.00	0.00	0.00	0.00
Ireland - Klebsiella pneumoniae 3GCRKP BSI - model 1	4.35	4.68	4.98	5.07	5.43	5.75	54.78	57.86	60.91	1.18	1.25	1.32	8.99	9.66	10.30	0.19	0.21	0.22
Ireland - Klebsiella pneumoniae 3GCRKP OTH - model 5	0.00	0.00	0.00	1.30E-03	1.65E-03	2.06E-03	19.97	22.96	25.81	0.43	0.50	0.56	0.00	0.00	0.00	0.00	0.00	0.00
Ireland - Klebsiella pneumoniae 3GCRKP RESP - model 2	0.77	0.87	0.96	1.17	1.32	1.45	44.49	49.77	54.90	0.96	1.08	1.19	1.60	1.79	1.98	0.03	0.04	0.04
Ireland - Klebsiella pneumoniae 3GCRKP SSI - model 4	0.20	0.23	0.27	0.20	0.23	0.27	19.86	22.73	25.66	0.43	0.49	0.55	0.54	0.64	0.73	0.01	0.01	0.02
Ireland - Klebsiella pneumoniae 3GCRKP UTI - model 3	0.00	0.00	0.00	2.25E-03	2.86E-03	3.59E-03	60.81	68.09	75.02	1.31	1.47	1.62	0.00	0.00	0.00	0.00	0.00	0.00
Ireland - Klebsiella pneumoniae CRKP BSI - model 6	0.20	0.32	0.50	0.22	0.35	0.53	1.76	2.16	2.65	0.04	0.05	0.06	0.49	0.77	1.13	0.01	0.02	0.02
Ireland - Klebsiella pneumoniae CRKP OTH - model 10	0.00	0.00	0.00	2.67E-05	6.91E-05	1.52E-04	0.47	1.00	1.53	0.01	0.02	0.03	0.00	0.00	0.00	0.00	0.00	0.00
Ireland - Klebsiella pneumoniae CRKP RESP - model 7	6.15E-03	0.01	0.02	9.53E-03	0.02	0.03	0.45	0.91	1.40	9.69E-03	0.02	0.03	0.02	0.03	0.05	3.39E-04	7.08E-04	1.10E-03
Ireland - Klebsiella pneumoniae CRKP SSI - model 9	1.85E-03	4.15E-03	6.36E-03	1.86E-03	4.17E-03	6.41E-03	0.29	0.64	0.97	6.25E-03	0.01	0.02	5.52E-03	0.01	0.02	1.19E-04	3.01E-04	4.96E-04
Ireland - Klebsiella pneumoniae CRKP UTI - model 8	0.00	0.00	0.00	2.45E-05	5.57E-05	1.14E-04	0.73	1.31	1.95	0.02	0.03	0.04	0.00	0.00	0.00	0.00	0.00	0.00
Ireland - Klebsiella pneumoniae ColRKP BSI - model 11	0.00	0.00	0.00	0.00	0.00	0.00	0.00	0.00	0.00	0.00	0.00	0.00	0.00	0.00	0.00	0.00	0.00	0.00
Ireland - Klebsiella pneumoniae ColRKP OTH - model 15	0.00	0.00	0.00	0.00	0.00	0.00	0.00	0.00	0.00	0.00	0.00	0.00	0.00	0.00	0.00	0.00	0.00	0.00
Ireland - Klebsiella pneumoniae ColRKP RESP - model 12	0.00	0.00	0.00	0.00	0.00	0.00	0.00	0.00	0.00	0.00	0.00	0.00	0.00	0.00	0.00	0.00	0.00	0.00
Ireland - Klebsiella pneumoniae ColRKP SSI - model 14	0.00	0.00	0.00	0.00	0.00	0.00	0.00	0.00	0.00	0.00	0.00	0.00	0.00	0.00	0.00	0.00	0.00	0.00
Ireland - Klebsiella pneumoniae ColRKP UTI - model 13	0.00	0.00	0.00	0.00	0.00	0.00	0.00	0.00	0.00	0.00	0.00	0.00	0.00	0.00	0.00	0.00	0.00	0.00
Ireland - Pseudomonas aeruginosa CRPA BSI - model 51	2.19	2.98	3.81	2.52	3.33	4.18	18.14	19.88	21.99	0.39	0.43	0.47	3.52	4.57	5.68	0.08	0.10	0.12
Ireland - Pseudomonas aeruginosa CRPA OTH - model 55	0.00	0.00	0.00	5.82E-04	7.94E-04	1.02E-03	8.16	10.06	12.04	0.18	0.22	0.26	0.00	0.00	0.00	0.00	0.00	0.00
Ireland - Pseudomonas aeruginosa CRPA RESP - model 52	2.38	3.19	4.07	3.19	4.06	5.05	63.64	73.66	83.12	1.37	1.59	1.80	3.89	4.86	6.07	0.08	0.11	0.13
Ireland - Pseudomonas aeruginosa CRPA SSI - model 54	0.11	0.14	0.17	0.11	0.14	0.17	10.98	13.64	16.28	0.24	0.29	0.35	0.25	0.33	0.41	5.36E-03	7.07E-03	8.78E-03
Ireland - Pseudomonas aeruginosa CRPA UTI - model 53	0.00	0.00	0.00	8.39E-04	1.12E-03	1.46E-03	21.13	25.46	29.94	0.46	0.55	0.65	0.00	0.00	0.00	0.00	0.00	0.00
Ireland - Pseudomonas aeruginosa ColRPA BSI - model 56	0.00	0.00	0.00	0.00	0.00	0.00	0.00	0.00	0.00	0.00	0.00	0.00	0.00	0.00	0.00	0.00	0.00	0.00
Ireland - Pseudomonas aeruginosa ColRPA OTH - model 60	0.00	0.00	0.00	0.00	0.00	0.00	0.00	0.00	0.00	0.00	0.00	0.00	0.00	0.00	0.00	0.00	0.00	0.00
Ireland - Pseudomonas aeruginosa ColRPA RESP - model 57	0.00	0.00	0.00	0.00	0.00	0.00	0.00	0.00	0.00	0.00	0.00	0.00	0.00	0.00	0.00	0.00	0.00	0.00
Ireland - Pseudomonas aeruginosa ColRPA SSI - model 59	0.00	0.00	0.00	0.00	0.00	0.00	0.00	0.00	0.00	0.00	0.00	0.00	0.00	0.00	0.00	0.00	0.00	0.00
Ireland - Pseudomonas aeruginosa ColRPA UTI - model 58	0.00	0.00	0.00	0.00	0.00	0.00	0.00	0.00	0.00	0.00	0.00	0.00	0.00	0.00	0.00	0.00	0.00	0.00
Ireland - Pseudomonas aeruginosa MDRPA BSI - model 46	0.06	0.17	0.34	0.08	0.19	0.37	0.79	1.12	1.55	0.02	0.02	0.03	0.08	0.23	0.45	1.75E-03	4.98E-03	9.82E-03
Ireland - Pseudomonas aeruginosa MDRPA OTH - model 50	0.00	0.00	0.00	1.06E-05	4.14E-05	9.91E-05	0.17	0.55	0.95	3.57E-03	0.01	0.02	0.00	0.00	0.00	0.00	0.00	0.00
Ireland - Pseudomonas aeruginosa MDRPA RESP - model 47	0.07	0.18	0.37	0.10	0.23	0.44	2.23	4.13	5.92	0.05	0.09	0.13	0.09	0.24	0.49	2.01E-03	5.15E-03	0.01
Ireland - Pseudomonas aeruginosa MDRPA SSI - model 49	1.72E-03	5.27E-03	8.53E-03	1.73E-03	5.30E-03	8.57E-03	0.26	0.78	1.26	5.52E-03	0.02	0.03	2.30E-03	7.03E-03	0.01	4.97E-05	1.52E-04	2.46E-04
Ireland - Pseudomonas aeruginosa MDRPA UTI - model 48	0.00	0.00	0.00	1.68E-05	5.67E-05	1.41E-04	0.57	1.41	2.24	0.01	0.03	0.05	0.00	0.00	0.00	0.00	0.00	0.00
Ireland - Staphylococcus aureus MRSA BSI - model 61	15.17	15.99	16.81	17.49	18.38	19.27	191.93	201.27	211.66	4.15	4.35	4.57	33.89	36.13	38.38	0.73	0.78	0.83
Ireland - Staphylococcus aureus MRSA OTH - model 65	0.00	0.00	0.00	0.02	0.02	0.03	271.19	297.01	323.58	5.86	6.42	6.99	0.00	0.00	0.00	0.00	0.00	0.00
Ireland - Staphylococcus aureus MRSA RESP - model 62	4.43	4.84	5.22	6.71	7.34	7.89	275.10	304.53	331.14	5.94	6.58	7.15	9.79	10.98	12.03	0.21	0.24	0.26
Ireland - Staphylococcus aureus MRSA SSI - model 64	2.62	2.87	3.11	2.63	2.88	3.13	303.65	330.11	358.52	6.56	7.13	7.75	8.19	9.15	10.07	0.18	0.20	0.22
Ireland - Staphylococcus aureus MRSA UTI - model 63	0.00	0.00	0.00	1.52E-03	1.98E-03	2.54E-03	41.09	49.43	56.90	0.89	1.07	1.23	0.00	0.00	0.00	0.00	0.00	0.00
Ireland - Streptococcus pneumoniae PMRSP BSI - model 76	0.03	0.04	0.04	0.04	0.04	0.05	1.03	1.03	1.03	0.02	0.02	0.02	0.16	0.18	0.21	3.52E-03	3.99E-03	4.48E-03
Ireland - Streptococcus pneumoniae PMRSP OTH - model 80	0.00	0.00	0.00	5.61E-06	1.47E-05	3.02E-05	0.30	0.36	0.47	6.46E-03	7.83E-03	0.01	0.00	0.00	0.00	0.00	0.00	0.00
Ireland - Streptococcus pneumoniae PMRSP RESP - model 77	0.02	0.03	0.05	0.03	0.04	0.07	2.85	3.79	6.18	0.06	0.08	0.13	0.10	0.14	0.23	2.06E-03	2.97E-03	4.92E-03
Ireland - Streptococcus pneumoniae PMRSP SSI - model 79	0.00	0.00	0.00	0.00	0.00	0.00	0.00	0.00	0.00	0.00	0.00	0.00	0.00	0.00	0.00	0.00	0.00	0.00
Ireland - Streptococcus pneumoniae PMRSP UTI - model 78	0.00	0.00	0.00	0.00	0.00	0.00	0.00	0.00	0.00	0.00	0.00	0.00	0.00	0.00	0.00	0.00	0.00	0.00
Ireland - Streptococcus pneumoniae PRSP BSI - model 71	0.00	0.00	0.00	0.00	0.00	0.00	0.00	0.00	0.00	0.00	0.00	0.00	0.00	0.00	0.00	0.00	0.00	0.00
Ireland - Streptococcus pneumoniae PRSP OTH - model 75	0.00	0.00	0.00	0.00	0.00	0.00	0.00	0.00	0.00	0.00	0.00	0.00	0.00	0.00	0.00	0.00	0.00	0.00
Ireland - Streptococcus pneumoniae PRSP RESP - model 72	0.00	0.00	0.00	0.00	0.00	0.00	0.00	0.00	0.00	0.00	0.00	0.00	0.00	0.00	0.00	0.00	0.00	0.00
Ireland - Streptococcus pneumoniae PRSP SSI - model 74	0.00	0.00	0.00	0.00	0.00	0.00	0.00	0.00	0.00	0.00	0.00	0.00	0.00	0.00	0.00	0.00	0.00	0.00
Ireland - Streptococcus pneumoniae PRSP UTI - model 73	0.00	0.00	0.00	0.00	0.00	0.00	0.00	0.00	0.00	0.00	0.00	0.00	0.00	0.00	0.00	0.00	0.00	0.00
**Total (Median & 95% UIs)**	89.34	101.80	115.86	104.33	118.02	133.51	4,322.08	4,893.24	5,486.45	93.37	105.71	118.52	191.69	218.87	248.69	4.14	4.73	5.37
**Total (average)**		**102.03**			**118.26**			**4,898.90**			**105.83**			**219.18**			**4.73**	

Infection	YLD			YLL			DALY			DALY per Case			YLD per 100,000			YLL per 100,000			DALY per 100,000			Cases per year			Incidence per 100,000			Deaths per year			Mortality per 100,000		
Colistin resistant *Acinetobacter spp.*	0.00	0.00	0.00	0.00	0.00	0.00	0.00	0.00	0.00	0.00	0.00	0.00	0.00	0.00	0.00	0.00	0.00	0.00	0.00	0.00	0.00	0.00	0.00	0.00	0.00	0.00	0.00	0.00	0.00	0.00	0.00	0.00	0.00
Carbapenem resistant *Acinetobacter spp.* (excluding those resistant to colistin)	0.00	0.00	0.00	0.00	0.00	0.00	0.00	0.00	0.00	0.00	0.00	0.00	0.00	0.00	0.00	0.00	0.00	0.00	0.00	0.00	0.00	0.00	0.00	0.00	0.00	0.00	0.00	0.00	0.00	0.00	0.00	0.00	0.00
Aminoglycosides and fluoroquinolones resistant *Acinetobacter* spp. (excluding those resistant to colistin and/or carbapenem)	0.00	0.00	0.00	0.00	0.00	0.00	0.00	0.00	0.00	0.00	0.00	0.00	0.00	0.00	0.00	0.00	0.00	0.00	0.00	0.00	0.00	0.00	0.00	0.00	0.00	0.00	0.00	0.00	0.00	0.00	0.00	0.00	0.00
Vancomycin resistant Enterococci (*E. faecalis* and *E. faecium*)	0.00	0.00	0.00	0.00	0.00	0.00	0.00	0.00	0.00	0.00	0.00	0.00	0.00	0.00	0.00	0.00	0.00	0.00	0.00	0.00	0.00	0.00	0.00	0.00	0.00	0.00	0.00	0.00	0.00	0.00	0.00	0.00	0.00
Colistin resistant *Escherichia coli*	0.00	0.00	0.00	0.00	0.00	0.00	0.00	0.00	0.00	0.00	0.00	0.00	0.00	0.00	0.00	0.00	0.00	0.00	0.00	0.00	0.00	0.00	0.00	0.00	0.00	0.00	0.00	0.00	0.00	0.00	0.00	0.00	0.00
Carbapenem resistant *Escherichia coli* (excluding those resistant to colistin)	0.00	0.00	0.00	0.00	0.00	0.00	0.00	0.00	0.00	0.00	0.00	0.00	0.00	0.00	0.00	0.00	0.00	0.00	0.00	0.00	0.00	0.00	0.00	0.00	0.00	0.00	0.00	0.00	0.00	0.00	0.00	0.00	0.00
Third-generation cephalosporin resistant *Escherichia coli* (excluding those resistant to colistin and/or carbapenem)	1.03	1.51	2.12	5.84	8.51	11.89	7.06	10.03	13.85	2.68	3.59	4.70	0.31	0.46	0.65	1.78	2.59	3.61	2.14	3.05	4.21	16.67	22.36	28.15	5.07	6.79	8.55	0.50	0.69	0.89	0.15	0.21	0.27
Colistin resistant *Klebsiella pneumoniae*	0.00	0.00	0.00	0.00	0.00	0.00	0.00	0.00	0.00	0.00	0.00	0.00	0.00	0.00	0.00	0.00	0.00	0.00	0.00	0.00	0.00	0.00	0.00	0.00	0.00	0.00	0.00	0.00	0.00	0.00	0.00	0.00	0.00
Carbapenem resistant *Klebsiella pneumoniae* (excluding those resistant to colistin)	0.00	0.00	0.00	0.00	0.00	0.00	0.00	0.00	0.00	0.00	0.00	0.00	0.00	0.00	0.00	0.00	0.00	0.00	0.00	0.00	0.00	0.00	0.00	0.00	0.00	0.00	0.00	0.00	0.00	0.00	0.00	0.00	0.00
Third-generation cephalosporin resistant *Klebsiella pneumoniae* (excluding those resistant to colistin and/or carbapenem)	0.00	0.00	0.00	0.00	0.00	0.00	0.00	0.00	0.00	0.00	0.00	0.00	0.00	0.00	0.00	0.00	0.00	0.00	0.00	0.00	0.00	0.00	0.00	0.00	0.00	0.00	0.00	0.00	0.00	0.00	0.00	0.00	0.00
Colistin resistant *Pseudomonas aeruginosa*	0.00	0.00	0.00	0.00	0.00	0.00	0.00	0.00	0.00	0.00	0.00	0.00	0.00	0.00	0.00	0.00	0.00	0.00	0.00	0.00	0.00	0.00	0.00	0.00	0.00	0.00	0.00	0.00	0.00	0.00	0.00	0.00	0.00
Carbapenem resistant *Pseudomonas aeruginosa* (excluding those resistant to colistin)	0.00	0.00	0.00	0.00	0.00	0.00	0.00	0.00	0.00	0.00	0.00	0.00	0.00	0.00	0.00	0.00	0.00	0.00	0.00	0.00	0.00	0.00	0.00	0.00	0.00	0.00	0.00	0.00	0.00	0.00	0.00	0.00	0.00
Pseudomonas aeruginosa resistant to three or more antimicrobial groups (excluding those resistant to colistin and/or carbapenem)	0.00	0.00	0.00	0.00	0.00	0.00	0.00	0.00	0.00	0.00	0.00	0.00	0.00	0.00	0.00	0.00	0.00	0.00	0.00	0.00	0.00	0.00	0.00	0.00	0.00	0.00	0.00	0.00	0.00	0.00	0.00	0.00	0.00
Methicillin resistant *Staphylococcus aureus*	0.00	0.00	0.00	0.00	0.00	0.00	0.00	0.00	0.00	0.00	0.00	0.00	0.00	0.00	0.00	0.00	0.00	0.00	0.00	0.00	0.00	0.00	0.00	0.00	0.00	0.00	0.00	0.00	0.00	0.00	0.00	0.00	0.00
Penicillin and macrolides resistant *Streptococcus pneumoniae* (excluding those only resistant to penicillin)	1.09	1.57	2.52	4.16	5.21	7.06	5.43	6.77	9.38	3.86	4.38	4.90	0.33	0.48	0.77	1.27	1.58	2.14	1.65	2.06	2.85	4.04	4.97	7.23	1.23	1.51	2.20	0.25	0.31	0.42	0.08	0.09	0.13
Penicillin tesistant *Streptococcus pneumoniae* (excluding those resistant to macrolides)	0.00	0.00	0.00	0.00	0.00	0.00	0.00	0.00	0.00	0.00	0.00	0.00	0.00	0.00	0.00	0.00	0.00	0.00	0.00	0.00	0.00	0.00	0.00	0.00	0.00	0.00	0.00	0.00	0.00	0.00	0.00	0.00	0.00
Total	2.12	3.08	4.65	10.01	13.72	18.95	12.49	16.80	23.23				0.64	0.94	1.41	3.04	4.17	5.76	3.80	5.11	7.06	20.72	27.33	35.38	6.30	8.30	10.75	0.75	1.00	1.31	0.23	0.30	0.40

Model	YLD			YLL			DALY			DALY per Case			YLD per 100,000			YLL per 100,000			DALY per 100,000			Cases per year			Incidence per 100,000			Deaths per year			Mortality per 100,000		
Iceland - Acinetobacter spp. ColRACI BSI - model 41	0.00	0.00	0.00	0.00	0.00	0.00	0.00	0.00	0.00	NaN	NaN	NaN	0.00	0.00	0.00	0.00	0.00	0.00	0.00	0.00	0.00	0.00	0.00	0.00	0.00	0.00	0.00	0.00	0.00	0.00	0.00	0.00	0.00
Iceland - Acinetobacter spp. ColRACI OTH - model 45	0.00	0.00	0.00	0.00	0.00	0.00	0.00	0.00	0.00	NaN	NaN	NaN	0.00	0.00	0.00	0.00	0.00	0.00	0.00	0.00	0.00	0.00	0.00	0.00	0.00	0.00	0.00	0.00	0.00	0.00	0.00	0.00	0.00
Iceland - Acinetobacter spp. ColRACI RESP - model 42	0.00	0.00	0.00	0.00	0.00	0.00	0.00	0.00	0.00	NaN	NaN	NaN	0.00	0.00	0.00	0.00	0.00	0.00	0.00	0.00	0.00	0.00	0.00	0.00	0.00	0.00	0.00	0.00	0.00	0.00	0.00	0.00	0.00
Iceland - Acinetobacter spp. ColRACI SSI - model 44	0.00	0.00	0.00	0.00	0.00	0.00	0.00	0.00	0.00	NaN	NaN	NaN	0.00	0.00	0.00	0.00	0.00	0.00	0.00	0.00	0.00	0.00	0.00	0.00	0.00	0.00	0.00	0.00	0.00	0.00	0.00	0.00	0.00
Iceland - Acinetobacter spp. ColRACI UTI - model 43	0.00	0.00	0.00	0.00	0.00	0.00	0.00	0.00	0.00	NaN	NaN	NaN	0.00	0.00	0.00	0.00	0.00	0.00	0.00	0.00	0.00	0.00	0.00	0.00	0.00	0.00	0.00	0.00	0.00	0.00	0.00	0.00	0.00
Iceland - Acinetobacter spp. CRACI BSI - model 36	0.00	0.00	0.00	0.00	0.00	0.00	0.00	0.00	0.00	NaN	NaN	NaN	0.00	0.00	0.00	0.00	0.00	0.00	0.00	0.00	0.00	0.00	0.00	0.00	0.00	0.00	0.00	0.00	0.00	0.00	0.00	0.00	0.00
Iceland - Acinetobacter spp. CRACI OTH - model 40	0.00	0.00	0.00	0.00	0.00	0.00	0.00	0.00	0.00	NaN	NaN	NaN	0.00	0.00	0.00	0.00	0.00	0.00	0.00	0.00	0.00	0.00	0.00	0.00	0.00	0.00	0.00	0.00	0.00	0.00	0.00	0.00	0.00
Iceland - Acinetobacter spp. CRACI RESP - model 37	0.00	0.00	0.00	0.00	0.00	0.00	0.00	0.00	0.00	NaN	NaN	NaN	0.00	0.00	0.00	0.00	0.00	0.00	0.00	0.00	0.00	0.00	0.00	0.00	0.00	0.00	0.00	0.00	0.00	0.00	0.00	0.00	0.00
Iceland - Acinetobacter spp. CRACI SSI - model 39	0.00	0.00	0.00	0.00	0.00	0.00	0.00	0.00	0.00	NaN	NaN	NaN	0.00	0.00	0.00	0.00	0.00	0.00	0.00	0.00	0.00	0.00	0.00	0.00	0.00	0.00	0.00	0.00	0.00	0.00	0.00	0.00	0.00
Iceland - Acinetobacter spp. CRACI UTI - model 38	0.00	0.00	0.00	0.00	0.00	0.00	0.00	0.00	0.00	NaN	NaN	NaN	0.00	0.00	0.00	0.00	0.00	0.00	0.00	0.00	0.00	0.00	0.00	0.00	0.00	0.00	0.00	0.00	0.00	0.00	0.00	0.00	0.00
Iceland - Acinetobacter spp. MDRACI BSI - model 31	0.00	0.00	0.00	0.00	0.00	0.00	0.00	0.00	0.00	NaN	NaN	NaN	0.00	0.00	0.00	0.00	0.00	0.00	0.00	0.00	0.00	0.00	0.00	0.00	0.00	0.00	0.00	0.00	0.00	0.00	0.00	0.00	0.00
Iceland - Acinetobacter spp. MDRACI OTH - model 35	0.00	0.00	0.00	0.00	0.00	0.00	0.00	0.00	0.00	NaN	NaN	NaN	0.00	0.00	0.00	0.00	0.00	0.00	0.00	0.00	0.00	0.00	0.00	0.00	0.00	0.00	0.00	0.00	0.00	0.00	0.00	0.00	0.00
Iceland - Acinetobacter spp. MDRACI RESP - model 32	0.00	0.00	0.00	0.00	0.00	0.00	0.00	0.00	0.00	NaN	NaN	NaN	0.00	0.00	0.00	0.00	0.00	0.00	0.00	0.00	0.00	0.00	0.00	0.00	0.00	0.00	0.00	0.00	0.00	0.00	0.00	0.00	0.00
Iceland - Acinetobacter spp. MDRACI SSI - model 34	0.00	0.00	0.00	0.00	0.00	0.00	0.00	0.00	0.00	NaN	NaN	NaN	0.00	0.00	0.00	0.00	0.00	0.00	0.00	0.00	0.00	0.00	0.00	0.00	0.00	0.00	0.00	0.00	0.00	0.00	0.00	0.00	0.00
Iceland - Acinetobacter spp. MDRACI UTI - model 33	0.00	0.00	0.00	0.00	0.00	0.00	0.00	0.00	0.00	NaN	NaN	NaN	0.00	0.00	0.00	0.00	0.00	0.00	0.00	0.00	0.00	0.00	0.00	0.00	0.00	0.00	0.00	0.00	0.00	0.00	0.00	0.00	0.00
Iceland - Enterococcus faecalis and E. faecium VRE BSI - model 66	0.00	0.00	0.00	0.00	0.00	0.00	0.00	0.00	0.00	NaN	NaN	NaN	0.00	0.00	0.00	0.00	0.00	0.00	0.00	0.00	0.00	0.00	0.00	0.00	0.00	0.00	0.00	0.00	0.00	0.00	0.00	0.00	0.00
Iceland - Enterococcus faecalis and E. faecium VRE OTH - model 70	0.00	0.00	0.00	0.00	0.00	0.00	0.00	0.00	0.00	NaN	NaN	NaN	0.00	0.00	0.00	0.00	0.00	0.00	0.00	0.00	0.00	0.00	0.00	0.00	0.00	0.00	0.00	0.00	0.00	0.00	0.00	0.00	0.00
Iceland - Enterococcus faecalis and E. faecium VRE RESP - model 67	0.00	0.00	0.00	0.00	0.00	0.00	0.00	0.00	0.00	NaN	NaN	NaN	0.00	0.00	0.00	0.00	0.00	0.00	0.00	0.00	0.00	0.00	0.00	0.00	0.00	0.00	0.00	0.00	0.00	0.00	0.00	0.00	0.00
Iceland - Enterococcus faecalis and E. faecium VRE SSI - model 69	0.00	0.00	0.00	0.00	0.00	0.00	0.00	0.00	0.00	NaN	NaN	NaN	0.00	0.00	0.00	0.00	0.00	0.00	0.00	0.00	0.00	0.00	0.00	0.00	0.00	0.00	0.00	0.00	0.00	0.00	0.00	0.00	0.00
Iceland - Enterococcus faecalis and E. faecium VRE UTI - model 68	0.00	0.00	0.00	0.00	0.00	0.00	0.00	0.00	0.00	NaN	NaN	NaN	0.00	0.00	0.00	0.00	0.00	0.00	0.00	0.00	0.00	0.00	0.00	0.00	0.00	0.00	0.00	0.00	0.00	0.00	0.00	0.00	0.00
Iceland - Escherichia coli 3GCREC BSI - model 16	0.83	1.12	1.53	5.00	7.14	9.96	6.01	8.27	11.34	2.01	2.65	3.47	0.25	0.34	0.46	1.52	2.17	3.03	1.82	2.51	3.45	2.70	3.14	3.69	0.82	0.95	1.12	0.41	0.55	0.70	0.12	0.17	0.21
Iceland - Escherichia coli 3GCREC OTH - model 20	1.59E-03	3.40E-03	6.09E-03	0.00	0.00	0.00	1.59E-03	3.40E-03	6.09E-03	1.94E-03	3.30E-03	4.89E-03	4.83E-04	1.03E-03	1.85E-03	0.00	0.00	0.00	4.83E-04	1.03E-03	1.85E-03	0.61	1.05	1.52	0.18	0.32	0.46	0.00	0.00	0.00	0.00	0.00	0.00
Iceland - Escherichia coli 3GCREC RESP - model 17	0.17	0.35	0.54	0.35	0.67	1.01	0.54	1.02	1.54	0.47	0.72	1.00	0.05	0.11	0.16	0.11	0.20	0.31	0.16	0.31	0.47	0.89	1.39	1.92	0.27	0.42	0.58	0.03	0.05	0.07	9.39E-03	0.02	0.02
Iceland - Escherichia coli 3GCREC SSI - model 19	3.12E-03	6.91E-03	0.01	0.50	0.71	0.93	0.50	0.71	0.93	0.20	0.21	0.23	9.48E-04	2.10E-03	3.71E-03	0.15	0.21	0.28	0.15	0.22	0.28	2.34	3.34	4.33	0.71	1.01	1.32	0.06	0.09	0.12	0.02	0.03	0.04
Iceland - Escherichia coli 3GCREC UTI - model 18	0.01	0.03	0.04	0.00	0.00	0.00	0.01	0.03	0.04	1.16E-03	1.94E-03	2.87E-03	4.37E-03	7.95E-03	0.01	0.00	0.00	0.00	4.37E-03	7.95E-03	0.01	10.14	13.45	16.68	3.08	4.09	5.07	0.00	0.00	0.00	0.00	0.00	0.00
Iceland - Escherichia coli ColREC BSI - model 26	0.00	0.00	0.00	0.00	0.00	0.00	0.00	0.00	0.00	NaN	NaN	NaN	0.00	0.00	0.00	0.00	0.00	0.00	0.00	0.00	0.00	0.00	0.00	0.00	0.00	0.00	0.00	0.00	0.00	0.00	0.00	0.00	0.00
Iceland - Escherichia coli ColREC OTH - model 30	0.00	0.00	0.00	0.00	0.00	0.00	0.00	0.00	0.00	NaN	NaN	NaN	0.00	0.00	0.00	0.00	0.00	0.00	0.00	0.00	0.00	0.00	0.00	0.00	0.00	0.00	0.00	0.00	0.00	0.00	0.00	0.00	0.00
Iceland - Escherichia coli ColREC RESP - model 27	0.00	0.00	0.00	0.00	0.00	0.00	0.00	0.00	0.00	NaN	NaN	NaN	0.00	0.00	0.00	0.00	0.00	0.00	0.00	0.00	0.00	0.00	0.00	0.00	0.00	0.00	0.00	0.00	0.00	0.00	0.00	0.00	0.00
Iceland - Escherichia coli ColREC SSI - model 29	0.00	0.00	0.00	0.00	0.00	0.00	0.00	0.00	0.00	NaN	NaN	NaN	0.00	0.00	0.00	0.00	0.00	0.00	0.00	0.00	0.00	0.00	0.00	0.00	0.00	0.00	0.00	0.00	0.00	0.00	0.00	0.00	0.00
Iceland - Escherichia coli ColREC UTI - model 28	0.00	0.00	0.00	0.00	0.00	0.00	0.00	0.00	0.00	NaN	NaN	NaN	0.00	0.00	0.00	0.00	0.00	0.00	0.00	0.00	0.00	0.00	0.00	0.00	0.00	0.00	0.00	0.00	0.00	0.00	0.00	0.00	0.00
Iceland - Escherichia coli CREC BSI - model 21	0.00	0.00	0.00	0.00	0.00	0.00	0.00	0.00	0.00	NaN	NaN	NaN	0.00	0.00	0.00	0.00	0.00	0.00	0.00	0.00	0.00	0.00	0.00	0.00	0.00	0.00	0.00	0.00	0.00	0.00	0.00	0.00	0.00
Iceland - Escherichia coli CREC OTH - model 25	0.00	0.00	0.00	0.00	0.00	0.00	0.00	0.00	0.00	NaN	NaN	NaN	0.00	0.00	0.00	0.00	0.00	0.00	0.00	0.00	0.00	0.00	0.00	0.00	0.00	0.00	0.00	0.00	0.00	0.00	0.00	0.00	0.00
Iceland - Escherichia coli CREC RESP - model 22	0.00	0.00	0.00	0.00	0.00	0.00	0.00	0.00	0.00	NaN	NaN	NaN	0.00	0.00	0.00	0.00	0.00	0.00	0.00	0.00	0.00	0.00	0.00	0.00	0.00	0.00	0.00	0.00	0.00	0.00	0.00	0.00	0.00
Iceland - Escherichia coli CREC SSI - model 24	0.00	0.00	0.00	0.00	0.00	0.00	0.00	0.00	0.00	NaN	NaN	NaN	0.00	0.00	0.00	0.00	0.00	0.00	0.00	0.00	0.00	0.00	0.00	0.00	0.00	0.00	0.00	0.00	0.00	0.00	0.00	0.00	0.00
Iceland - Escherichia coli CREC UTI - model 23	0.00	0.00	0.00	0.00	0.00	0.00	0.00	0.00	0.00	NaN	NaN	NaN	0.00	0.00	0.00	0.00	0.00	0.00	0.00	0.00	0.00	0.00	0.00	0.00	0.00	0.00	0.00	0.00	0.00	0.00	0.00	0.00	0.00
Iceland - Klebsiella pneumoniae 3GCRKP BSI - model 1	0.00	0.00	0.00	0.00	0.00	0.00	0.00	0.00	0.00	NaN	NaN	NaN	0.00	0.00	0.00	0.00	0.00	0.00	0.00	0.00	0.00	0.00	0.00	0.00	0.00	0.00	0.00	0.00	0.00	0.00	0.00	0.00	0.00
Iceland - Klebsiella pneumoniae 3GCRKP OTH - model 5	0.00	0.00	0.00	0.00	0.00	0.00	0.00	0.00	0.00	NaN	NaN	NaN	0.00	0.00	0.00	0.00	0.00	0.00	0.00	0.00	0.00	0.00	0.00	0.00	0.00	0.00	0.00	0.00	0.00	0.00	0.00	0.00	0.00
Iceland - Klebsiella pneumoniae 3GCRKP RESP - model 2	0.00	0.00	0.00	0.00	0.00	0.00	0.00	0.00	0.00	NaN	NaN	NaN	0.00	0.00	0.00	0.00	0.00	0.00	0.00	0.00	0.00	0.00	0.00	0.00	0.00	0.00	0.00	0.00	0.00	0.00	0.00	0.00	0.00
Iceland - Klebsiella pneumoniae 3GCRKP SSI - model 4	0.00	0.00	0.00	0.00	0.00	0.00	0.00	0.00	0.00	NaN	NaN	NaN	0.00	0.00	0.00	0.00	0.00	0.00	0.00	0.00	0.00	0.00	0.00	0.00	0.00	0.00	0.00	0.00	0.00	0.00	0.00	0.00	0.00
Iceland - Klebsiella pneumoniae 3GCRKP UTI - model 3	0.00	0.00	0.00	0.00	0.00	0.00	0.00	0.00	0.00	NaN	NaN	NaN	0.00	0.00	0.00	0.00	0.00	0.00	0.00	0.00	0.00	0.00	0.00	0.00	0.00	0.00	0.00	0.00	0.00	0.00	0.00	0.00	0.00
Iceland - Klebsiella pneumoniae ColRKP BSI - model 11	0.00	0.00	0.00	0.00	0.00	0.00	0.00	0.00	0.00	NaN	NaN	NaN	0.00	0.00	0.00	0.00	0.00	0.00	0.00	0.00	0.00	0.00	0.00	0.00	0.00	0.00	0.00	0.00	0.00	0.00	0.00	0.00	0.00
Iceland - Klebsiella pneumoniae ColRKP OTH - model 15	0.00	0.00	0.00	0.00	0.00	0.00	0.00	0.00	0.00	NaN	NaN	NaN	0.00	0.00	0.00	0.00	0.00	0.00	0.00	0.00	0.00	0.00	0.00	0.00	0.00	0.00	0.00	0.00	0.00	0.00	0.00	0.00	0.00
Iceland - Klebsiella pneumoniae ColRKP RESP - model 12	0.00	0.00	0.00	0.00	0.00	0.00	0.00	0.00	0.00	NaN	NaN	NaN	0.00	0.00	0.00	0.00	0.00	0.00	0.00	0.00	0.00	0.00	0.00	0.00	0.00	0.00	0.00	0.00	0.00	0.00	0.00	0.00	0.00
Iceland - Klebsiella pneumoniae ColRKP SSI - model 14	0.00	0.00	0.00	0.00	0.00	0.00	0.00	0.00	0.00	NaN	NaN	NaN	0.00	0.00	0.00	0.00	0.00	0.00	0.00	0.00	0.00	0.00	0.00	0.00	0.00	0.00	0.00	0.00	0.00	0.00	0.00	0.00	0.00
Iceland - Klebsiella pneumoniae ColRKP UTI - model 13	0.00	0.00	0.00	0.00	0.00	0.00	0.00	0.00	0.00	NaN	NaN	NaN	0.00	0.00	0.00	0.00	0.00	0.00	0.00	0.00	0.00	0.00	0.00	0.00	0.00	0.00	0.00	0.00	0.00	0.00	0.00	0.00	0.00
Iceland - Klebsiella pneumoniae CRKP BSI - model 6	0.00	0.00	0.00	0.00	0.00	0.00	0.00	0.00	0.00	NaN	NaN	NaN	0.00	0.00	0.00	0.00	0.00	0.00	0.00	0.00	0.00	0.00	0.00	0.00	0.00	0.00	0.00	0.00	0.00	0.00	0.00	0.00	0.00
Iceland - Klebsiella pneumoniae CRKP OTH - model 10	0.00	0.00	0.00	0.00	0.00	0.00	0.00	0.00	0.00	NaN	NaN	NaN	0.00	0.00	0.00	0.00	0.00	0.00	0.00	0.00	0.00	0.00	0.00	0.00	0.00	0.00	0.00	0.00	0.00	0.00	0.00	0.00	0.00
Iceland - Klebsiella pneumoniae CRKP RESP - model 7	0.00	0.00	0.00	0.00	0.00	0.00	0.00	0.00	0.00	NaN	NaN	NaN	0.00	0.00	0.00	0.00	0.00	0.00	0.00	0.00	0.00	0.00	0.00	0.00	0.00	0.00	0.00	0.00	0.00	0.00	0.00	0.00	0.00
Iceland - Klebsiella pneumoniae CRKP SSI - model 9	0.00	0.00	0.00	0.00	0.00	0.00	0.00	0.00	0.00	NaN	NaN	NaN	0.00	0.00	0.00	0.00	0.00	0.00	0.00	0.00	0.00	0.00	0.00	0.00	0.00	0.00	0.00	0.00	0.00	0.00	0.00	0.00	0.00
Iceland - Klebsiella pneumoniae CRKP UTI - model 8	0.00	0.00	0.00	0.00	0.00	0.00	0.00	0.00	0.00	NaN	NaN	NaN	0.00	0.00	0.00	0.00	0.00	0.00	0.00	0.00	0.00	0.00	0.00	0.00	0.00	0.00	0.00	0.00	0.00	0.00	0.00	0.00	0.00
Iceland - Pseudomonas aeruginosa ColRPA BSI - model 56	0.00	0.00	0.00	0.00	0.00	0.00	0.00	0.00	0.00	NaN	NaN	NaN	0.00	0.00	0.00	0.00	0.00	0.00	0.00	0.00	0.00	0.00	0.00	0.00	0.00	0.00	0.00	0.00	0.00	0.00	0.00	0.00	0.00
Iceland - Pseudomonas aeruginosa ColRPA OTH - model 60	0.00	0.00	0.00	0.00	0.00	0.00	0.00	0.00	0.00	NaN	NaN	NaN	0.00	0.00	0.00	0.00	0.00	0.00	0.00	0.00	0.00	0.00	0.00	0.00	0.00	0.00	0.00	0.00	0.00	0.00	0.00	0.00	0.00
Iceland - Pseudomonas aeruginosa ColRPA RESP - model 57	0.00	0.00	0.00	0.00	0.00	0.00	0.00	0.00	0.00	NaN	NaN	NaN	0.00	0.00	0.00	0.00	0.00	0.00	0.00	0.00	0.00	0.00	0.00	0.00	0.00	0.00	0.00	0.00	0.00	0.00	0.00	0.00	0.00
Iceland - Pseudomonas aeruginosa ColRPA SSI - model 59	0.00	0.00	0.00	0.00	0.00	0.00	0.00	0.00	0.00	NaN	NaN	NaN	0.00	0.00	0.00	0.00	0.00	0.00	0.00	0.00	0.00	0.00	0.00	0.00	0.00	0.00	0.00	0.00	0.00	0.00	0.00	0.00	0.00
Iceland - Pseudomonas aeruginosa ColRPA UTI - model 58	0.00	0.00	0.00	0.00	0.00	0.00	0.00	0.00	0.00	NaN	NaN	NaN	0.00	0.00	0.00	0.00	0.00	0.00	0.00	0.00	0.00	0.00	0.00	0.00	0.00	0.00	0.00	0.00	0.00	0.00	0.00	0.00	0.00
Iceland - Pseudomonas aeruginosa CRPA BSI - model 51	0.00	0.00	0.00	0.00	0.00	0.00	0.00	0.00	0.00	NaN	NaN	NaN	0.00	0.00	0.00	0.00	0.00	0.00	0.00	0.00	0.00	0.00	0.00	0.00	0.00	0.00	0.00	0.00	0.00	0.00	0.00	0.00	0.00
Iceland - Pseudomonas aeruginosa CRPA OTH - model 55	0.00	0.00	0.00	0.00	0.00	0.00	0.00	0.00	0.00	NaN	NaN	NaN	0.00	0.00	0.00	0.00	0.00	0.00	0.00	0.00	0.00	0.00	0.00	0.00	0.00	0.00	0.00	0.00	0.00	0.00	0.00	0.00	0.00
Iceland - Pseudomonas aeruginosa CRPA RESP - model 52	0.00	0.00	0.00	0.00	0.00	0.00	0.00	0.00	0.00	NaN	NaN	NaN	0.00	0.00	0.00	0.00	0.00	0.00	0.00	0.00	0.00	0.00	0.00	0.00	0.00	0.00	0.00	0.00	0.00	0.00	0.00	0.00	0.00
Iceland - Pseudomonas aeruginosa CRPA SSI - model 54	0.00	0.00	0.00	0.00	0.00	0.00	0.00	0.00	0.00	NaN	NaN	NaN	0.00	0.00	0.00	0.00	0.00	0.00	0.00	0.00	0.00	0.00	0.00	0.00	0.00	0.00	0.00	0.00	0.00	0.00	0.00	0.00	0.00
Iceland - Pseudomonas aeruginosa CRPA UTI - model 53	0.00	0.00	0.00	0.00	0.00	0.00	0.00	0.00	0.00	NaN	NaN	NaN	0.00	0.00	0.00	0.00	0.00	0.00	0.00	0.00	0.00	0.00	0.00	0.00	0.00	0.00	0.00	0.00	0.00	0.00	0.00	0.00	0.00
Iceland - Pseudomonas aeruginosa MDRPA BSI - model 46	0.00	0.00	0.00	0.00	0.00	0.00	0.00	0.00	0.00	NaN	NaN	NaN	0.00	0.00	0.00	0.00	0.00	0.00	0.00	0.00	0.00	0.00	0.00	0.00	0.00	0.00	0.00	0.00	0.00	0.00	0.00	0.00	0.00
Iceland - Pseudomonas aeruginosa MDRPA OTH - model 50	0.00	0.00	0.00	0.00	0.00	0.00	0.00	0.00	0.00	NaN	NaN	NaN	0.00	0.00	0.00	0.00	0.00	0.00	0.00	0.00	0.00	0.00	0.00	0.00	0.00	0.00	0.00	0.00	0.00	0.00	0.00	0.00	0.00
Iceland - Pseudomonas aeruginosa MDRPA RESP - model 47	0.00	0.00	0.00	0.00	0.00	0.00	0.00	0.00	0.00	NaN	NaN	NaN	0.00	0.00	0.00	0.00	0.00	0.00	0.00	0.00	0.00	0.00	0.00	0.00	0.00	0.00	0.00	0.00	0.00	0.00	0.00	0.00	0.00
Iceland - Pseudomonas aeruginosa MDRPA SSI - model 49	0.00	0.00	0.00	0.00	0.00	0.00	0.00	0.00	0.00	NaN	NaN	NaN	0.00	0.00	0.00	0.00	0.00	0.00	0.00	0.00	0.00	0.00	0.00	0.00	0.00	0.00	0.00	0.00	0.00	0.00	0.00	0.00	0.00
Iceland - Pseudomonas aeruginosa MDRPA UTI - model 48	0.00	0.00	0.00	0.00	0.00	0.00	0.00	0.00	0.00	NaN	NaN	NaN	0.00	0.00	0.00	0.00	0.00	0.00	0.00	0.00	0.00	0.00	0.00	0.00	0.00	0.00	0.00	0.00	0.00	0.00	0.00	0.00	0.00
Iceland - Staphylococcus aureus MRSA BSI - model 61	0.00	0.00	0.00	0.00	0.00	0.00	0.00	0.00	0.00	NaN	NaN	NaN	0.00	0.00	0.00	0.00	0.00	0.00	0.00	0.00	0.00	0.00	0.00	0.00	0.00	0.00	0.00	0.00	0.00	0.00	0.00	0.00	0.00
Iceland - Staphylococcus aureus MRSA OTH - model 65	0.00	0.00	0.00	0.00	0.00	0.00	0.00	0.00	0.00	NaN	NaN	NaN	0.00	0.00	0.00	0.00	0.00	0.00	0.00	0.00	0.00	0.00	0.00	0.00	0.00	0.00	0.00	0.00	0.00	0.00	0.00	0.00	0.00
Iceland - Staphylococcus aureus MRSA RESP - model 62	0.00	0.00	0.00	0.00	0.00	0.00	0.00	0.00	0.00	NaN	NaN	NaN	0.00	0.00	0.00	0.00	0.00	0.00	0.00	0.00	0.00	0.00	0.00	0.00	0.00	0.00	0.00	0.00	0.00	0.00	0.00	0.00	0.00
Iceland - Staphylococcus aureus MRSA SSI - model 64	0.00	0.00	0.00	0.00	0.00	0.00	0.00	0.00	0.00	NaN	NaN	NaN	0.00	0.00	0.00	0.00	0.00	0.00	0.00	0.00	0.00	0.00	0.00	0.00	0.00	0.00	0.00	0.00	0.00	0.00	0.00	0.00	0.00
Iceland - Staphylococcus aureus MRSA UTI - model 63	0.00	0.00	0.00	0.00	0.00	0.00	0.00	0.00	0.00	NaN	NaN	NaN	0.00	0.00	0.00	0.00	0.00	0.00	0.00	0.00	0.00	0.00	0.00	0.00	0.00	0.00	0.00	0.00	0.00	0.00	0.00	0.00	0.00
Iceland - Streptococcus pneumoniae PMRSP BSI - model 76	0.33	0.44	0.58	2.65	3.03	3.39	3.07	3.46	3.86	3.07	3.46	3.86	0.10	0.13	0.18	0.81	0.92	1.03	0.93	1.05	1.17	1.00	1.00	1.00	0.30	0.30	0.30	0.16	0.18	0.20	0.05	0.05	0.06
Iceland - Streptococcus pneumoniae PMRSP OTH - model 80	2.32E-04	6.78E-04	1.32E-03	0.00	0.00	0.00	2.32E-04	6.78E-04	1.32E-03	7.50E-04	1.93E-03	3.57E-03	7.04E-05	2.06E-04	4.01E-04	0.00	0.00	0.00	7.04E-05	2.06E-04	4.01E-04	0.29	0.35	0.45	0.09	0.11	0.14	0.00	0.00	0.00	0.00	0.00	0.00
Iceland - Streptococcus pneumoniae PMRSP RESP - model 77	0.77	1.13	1.94	1.51	2.18	3.67	2.37	3.31	5.52	0.79	0.92	1.04	0.23	0.34	0.59	0.46	0.66	1.12	0.72	1.00	1.68	2.76	3.62	5.77	0.84	1.10	1.75	0.09	0.13	0.22	0.03	0.04	0.07
Iceland - Streptococcus pneumoniae PMRSP SSI - model 79	0.00	0.00	0.00	0.00	0.00	0.00	0.00	0.00	0.00	NaN	NaN	NaN	0.00	0.00	0.00	0.00	0.00	0.00	0.00	0.00	0.00	0.00	0.00	0.00	0.00	0.00	0.00	0.00	0.00	0.00	0.00	0.00	0.00
Iceland - Streptococcus pneumoniae PMRSP UTI - model 78	0.00	0.00	0.00	0.00	0.00	0.00	0.00	0.00	0.00	NaN	NaN	NaN	0.00	0.00	0.00	0.00	0.00	0.00	0.00	0.00	0.00	0.00	0.00	0.00	0.00	0.00	0.00	0.00	0.00	0.00	0.00	0.00	0.00
Iceland - Streptococcus pneumoniae PRSP BSI - model 71	0.00	0.00	0.00	0.00	0.00	0.00	0.00	0.00	0.00	NaN	NaN	NaN	0.00	0.00	0.00	0.00	0.00	0.00	0.00	0.00	0.00	0.00	0.00	0.00	0.00	0.00	0.00	0.00	0.00	0.00	0.00	0.00	0.00
Iceland - Streptococcus pneumoniae PRSP OTH - model 75	0.00	0.00	0.00	0.00	0.00	0.00	0.00	0.00	0.00	NaN	NaN	NaN	0.00	0.00	0.00	0.00	0.00	0.00	0.00	0.00	0.00	0.00	0.00	0.00	0.00	0.00	0.00	0.00	0.00	0.00	0.00	0.00	0.00
Iceland - Streptococcus pneumoniae PRSP RESP - model 72	0.00	0.00	0.00	0.00	0.00	0.00	0.00	0.00	0.00	NaN	NaN	NaN	0.00	0.00	0.00	0.00	0.00	0.00	0.00	0.00	0.00	0.00	0.00	0.00	0.00	0.00	0.00	0.00	0.00	0.00	0.00	0.00	0.00
Iceland - Streptococcus pneumoniae PRSP SSI - model 74	0.00	0.00	0.00	0.00	0.00	0.00	0.00	0.00	0.00	NaN	NaN	NaN	0.00	0.00	0.00	0.00	0.00	0.00	0.00	0.00	0.00	0.00	0.00	0.00	0.00	0.00	0.00	0.00	0.00	0.00	0.00	0.00	0.00
Iceland - Streptococcus pneumoniae PRSP UTI - model 73	0.00	0.00	0.00	0.00	0.00	0.00	0.00	0.00	0.00	NaN	NaN	NaN	0.00	0.00	0.00	0.00	0.00	0.00	0.00	0.00	0.00	0.00	0.00	0.00	0.00	0.00	0.00	0.00	0.00	0.00	0.00	0.00	0.00
**Total (Median & 95% UIs)**	2.12	3.08	4.65	10.01	13.72	18.95	12.49	16.80	23.23		-		0.64	0.94	1.41	3.04	4.17	5.76	3.80	5.11	7.06	20.72	27.33	35.38	6.30	8.30	10.75	0.75	1.00	1.31	0.23	0.30	0.40
**Total (average)**		**3.17**			**13.96**			**17.13**			-			**0.96**			**4.24**			**5.20**			**27.54**			**8.37**			**1.01**			**0.31**	

Infection	YLD			YLL			DALY			DALY per Case			YLD per 100,000		
Colistin resistant *Acinetobacter spp.*	122.94	153.39	189.59	579.02	817.56	1.085.03	713.50	971.89	1.265.67	6.38	8.21	10.10	0.20	0.25	0.31
Carbapenem resistant *Acinetobacter spp.* (excluding those resistant to colistin)	3,848.26	4,414.09	4,991.46	19,315.09	23,532.41	27,711.84	23,329.24	27,969.97	32,449.30	6.40	7.36	8.35	6.33	7.26	8.21
Aminoglycosides and fluoroquinolones resistant *Acinetobacter* spp. (excluding those resistant to colistin and/or carbapenem)	60.17	70.38	81.64	294.27	364.90	442.53	357.31	435.39	520.51	3.89	4.59	5.39	0.10	0.12	0.13
Vancomycin resistant Enterococci (*E. faecalis* and *E. faecium*)	516.65	651.96	811.84	4,907.99	6,186.34	7,659.62	5,384.80	6,831.01	8,439.87	5.57	6.25	7.05	0.85	1.07	1.34
Colistin resistant *Escherichia coli*	173.12	205.49	241.27	2,877.51	3,477.76	4,143.86	3,054.49	3,683.32	4,369.91	11.40	13.37	15.57	0.28	0.34	0.40
Carbapenem resistant *Escherichia coli* (excluding those resistant to colistin)	75.83	98.70	128.94	783.87	1,042.96	1,425.08	861.66	1,140.06	1,528.26	11.27	14.35	17.81	0.12	0.16	0.21
Third-generation cephalosporin resistant *Escherichia coli* (excluding those resistant to colistin and/or carbapenem)	6,972.23	8,131.30	9,355.87	41,429.12	48,116.17	56,147.84	48,647.39	56,308.85	65,145.23	4.44	4.93	5.43	11.47	13.37	15.39
Colistin resistant *Klebsiella pneumoniae*	1,264.92	1,490.77	1,748.46	22,073.91	26,233.98	31,088.92	23,400.34	27,727.18	32,761.23	15.57	17.88	20.25	2.08	2.45	2.88
Carbapenem resistant *Klebsiella pneumoniae* (excluding those resistant to colistin)	2,757.85	3,233.62	3,721.76	28,944.34	34,149.32	39,976.25	31,788.08	37,393.79	43,543.67	9.81	11.04	12.33	4.54	5.32	6.12
Third-generation cephalosporin resistant *Klebsiella pneumoniae* (excluding those resistant to colistin and/or carbapenem)	3,227.41	3,695.03	4,208.36	15,693.72	17,584.74	19,849.57	18,991.66	21,303.20	23,997.88	6.53	7.08	7.70	5.31	6.08	6.92
Colistin resistant *Pseudomonas aeruginosa*	178.93	228.62	289.42	826.99	1.140.02	1.606.30	1.020.14	1.369.14	1.884.08	8.29	10.66	13.48	0.29	0.38	0.48
Carbapenem resistant *Pseudomonas aeruginosa* (excluding those resistant to colistin)	4,496.72	5,270.27	6,072.92	21,551.99	26,500.58	32,143.76	26,328.34	31,786.16	38,022.58	8.60	9.97	11.48	7.40	8.67	9.99
Pseudomonas aeruginosa resistant to three or more antimicrobial groups (excluding those resistant to colistin and/or carbapenem)	442.58	539.51	642.46	1,934.83	2,532.87	3,198.92	2,407.10	3,074.30	3,819.15	5.51	6.70	7.97	0.73	0.89	1.06
Methicillin resistant *Staphylococcus aureus*	7.658.44	8.772.65	10.095.06	37.715.16	42.387.01	47.572.19	45.565.05	51.188.16	57.428.68	5.47	5.91	6.42	12.60	14.43	16.60
Penicillin and macrolides resistant *Streptococcus pneumoniae* (excluding those only resistant to penicillin)	77.06	113.96	188.11	278.31	368.33	522.29	361.46	482.88	700.69	7.32	8.51	9.89	0.13	0.19	0.31
Penicillin tesistant *Streptococcus pneumoniae* (excluding those resistant to macrolides)	183.02	227.42	291.49	614.77	730.14	879.80	798.61	957.63	1,163.32	9.59	10.38	11.37	0.30	0.37	0.48
Total	32,056.11	37,297.16	43,058.64	199,820.89	235,165.11	275,453.79	233,009.16	272,622.93	317,040.02				52.73	61.35	70.83

Infection	YLL per 100,000			DALY per 100,000			Cases per year			Incidence per 100,000			Deaths per year			Mortality per 100,000		
Colistin resistant *Acinetobacter spp.*	0.95	1.34	1.78	1.17	1.60	2.08	281.73	357.97	436.99	0.46	0.59	0.72	22.37	30.77	40.75	0.04	0.05	0.07
Carbapenem resistant *Acinetobacter spp.* (excluding those resistant to colistin)	31.77	38.71	45.58	38.37	46.01	53.37	9,770.46	11,410.09	13,106.23	16.07	18.77	21.56	789.46	976.44	1,175.05	1.30	1.61	1.93
Aminoglycosides and fluoroquinolones resistant *Acinetobacter* spp. (excluding those resistant to colistin and/or carbapenem)	0.48	0.60	0.73	0.59	0.72	0.86	226.34	288.83	359.24	0.37	0.48	0.59	17.87	24.48	32.23	0.03	0.04	0.05
Vancomycin resistant Enterococci (*E. faecalis* and *E. faecium*)	8.07	10.18	12.60	8.86	11.24	13.88	3,186.35	4,190.98	5,248.14	5.24	6.89	8.63	217.79	281.27	356.24	0.36	0.46	0.59
Colistin resistant *Escherichia coli*	4.73	5.72	6.82	5.02	6.06	7.19	1,740.76	2,149.90	2,634.70	2.86	3.54	4.33	144.84	185.53	236.66	0.24	0.31	0.39
Carbapenem resistant *Escherichia coli* (excluding those resistant to colistin)	1.29	1.72	2.34	1.42	1.88	2.51	522.53	638.20	760.92	0.86	1.05	1.25	27.36	33.62	42.13	0.05	0.06	0.07
Third-generation cephalosporin resistant *Escherichia coli* (excluding those resistant to colistin and/or carbapenem)	68.14	79.14	92.36	80.02	92.62	107.15	75,454.48	92,178.96	109,552.34	124.11	151.62	180.20	2,332.84	2,825.53	3,409.85	3.84	4.65	5.61
Colistin resistant *Klebsiella pneumoniae*	36.31	43.15	51.14	38.49	45.61	53.89	3,917.41	4,754.92	5,639.23	6.44	7.82	9.28	862.15	1,039.24	1,229.36	1.42	1.71	2.02
Carbapenem resistant *Klebsiella pneumoniae* (excluding those resistant to colistin)	47.61	56.17	65.76	52.29	61.51	71.62	8,941.81	10,840.47	12,717.78	14.71	17.83	20.92	1,181.96	1,425.11	1,689.62	1.94	2.34	2.78
Third-generation cephalosporin resistant *Klebsiella pneumoniae* (excluding those resistant to colistin and/or carbapenem)	25.81	28.92	32.65	31.24	35.04	39.47	10,941.77	12,572.01	14,384.70	18.00	20.68	23.66	590.78	669.57	750.71	0.97	1.10	1.23
Colistin resistant *Pseudomonas aeruginosa*	1.36	1.88	2.64	1.68	2.25	3.10	448.84	581.04	723.80	0.74	0.96	1.19	27.98	38.81	52.78	0.05	0.06	0.09
Carbapenem resistant *Pseudomonas aeruginosa* (excluding those resistant to colistin)	35.45	43.59	52.87	43.31	52.28	62.54	11,998.76	14,207.39	16,643.73	19.74	23.37	27.38	769.46	950.83	1,186.92	1.27	1.56	1.95
Pseudomonas aeruginosa resistant to three or more antimicrobial groups (excluding those resistant to colistin and/or carbapenem)	3.18	4.17	5.26	3.96	5.06	6.28	1,650.03	2,036.26	2,453.92	2.71	3.35	4.04	97.07	128.19	164.14	0.16	0.21	0.27
Methicillin resistant *Staphylococcus aureus*	62.04	69.72	78.25	74.95	84.20	94.46	38.324.20	44.877.93	51.912.59	63.04	73.82	85.39	1.844.27	2.123.09	2.414.77	3.03	3.49	3.97
Penicillin and macrolides resistant *Streptococcus pneumoniae* (excluding those only resistant to penicillin)	0.46	0.61	0.86	0.59	0.79	1.15	141.16	190.32	264.79	0.23	0.31	0.44	8.98	11.42	14.85	0.01	0.02	0.02
Penicillin tesistant *Streptococcus pneumoniae* (excluding those resistant to macrolides)	1.01	1.20	1.45	1.31	1.58	1.91	262.68	308.30	368.16	0.43	0.51	0.61	16.08	18.34	21.17	0.03	0.03	0.03
Total	328.68	386.81	453.08	383.27	448.43	521.49	167,809.32	201,583.57	237,207.25	276.02	331.58	390.17	8,951.28	10,762.23	12,817.23	14.72	17.70	21.08

Model	YLD			YLL			DALY			DALY per Case			YLD per 100,000		
Italy - Acinetobacter spp. CRACI BSI - model 36	2,197.61	2,484.65	2,786.44	15,592.39	19,160.62	22,711.43	17,964.48	21,666.00	25,268.12	4.80	5.60	6.45	3.61	4.09	4.58
Italy - Acinetobacter spp. CRACI OTH - model 40	0.42	0.58	0.80	0.00	0.00	0.00	0.42	0.58	0.80	3.10E-03	3.76E-03	4.45E-03	6.86E-04	9.57E-04	1.32E-03
Italy - Acinetobacter spp. CRACI RESP - model 37	1,645.35	1,922.34	2,195.60	3,217.07	3,742.16	4,237.00	4,854.77	5,667.12	6,409.21	1.21	1.32	1.43	2.71	3.16	3.61
Italy - Acinetobacter spp. CRACI SSI - model 39	2.33	3.15	4.28	505.63	629.63	763.41	507.02	632.92	766.84	0.39	0.43	0.47	3.83E-03	5.19E-03	7.03E-03
Italy - Acinetobacter spp. CRACI UTI - model 38	2.55	3.36	4.34	0.00	0.00	0.00	2.55	3.36	4.34	1.68E-03	2.05E-03	2.44E-03	4.19E-03	5.52E-03	7.13E-03
Italy - Acinetobacter spp. ColRACI BSI - model 41	71.07	86.31	105.05	463.43	668.05	899.77	545.32	755.04	996.52	4.72	6.32	7.94	0.12	0.14	0.17
Italy - Acinetobacter spp. ColRACI OTH - model 45	0.01	0.02	0.03	0.00	0.00	0.00	0.01	0.02	0.03	2.65E-03	3.76E-03	4.75E-03	1.81E-05	2.93E-05	4.43E-05
Italy - Acinetobacter spp. ColRACI RESP - model 42	51.73	66.86	84.21	101.44	129.98	160.02	153.88	197.10	243.58	1.27	1.46	1.69	0.09	0.11	0.14
Italy - Acinetobacter spp. ColRACI SSI - model 44	0.06	0.10	0.15	14.15	19.54	25.23	14.23	19.63	25.39	0.39	0.42	0.46	9.73E-05	1.61E-04	2.45E-04
Italy - Acinetobacter spp. ColRACI UTI - model 43	0.07	0.11	0.15	0.00	0.00	0.00	0.07	0.11	0.15	1.52E-03	2.03E-03	2.60E-03	1.14E-04	1.73E-04	2.46E-04
Italy - Acinetobacter spp. MDRACI BSI - model 31	34.64	39.69	45.40	234.99	293.11	357.00	272.48	332.93	399.65	2.83	3.40	4.04	0.06	0.07	0.07
Italy - Acinetobacter spp. MDRACI OTH - model 35	8.91E-03	0.01	0.02	0.00	0.00	0.00	8.91E-03	0.01	0.02	2.77E-03	3.73E-03	4.85E-03	1.46E-05	2.38E-05	3.79E-05
Italy - Acinetobacter spp. MDRACI RESP - model 32	25.42	30.51	35.97	49.10	58.61	68.79	74.51	89.10	103.89	0.73	0.83	0.94	0.04	0.05	0.06
Italy - Acinetobacter spp. MDRACI SSI - model 34	0.05	0.08	0.12	10.18	13.18	16.74	10.25	13.27	16.82	0.32	0.35	0.39	8.27E-05	1.30E-04	2.02E-04
Italy - Acinetobacter spp. MDRACI UTI - model 33	0.06	0.08	0.12	0.00	0.00	0.00	0.06	0.08	0.12	1.55E-03	2.02E-03	2.60E-03	9.24E-05	1.38E-04	2.05E-04
Italy - Enterococcus faecalis and E. faecium VRE BSI - model 66	512.54	646.05	803.31	4,434.34	5,532.04	6,795.47	4,907.43	6,169.80	7,567.52	5.17	5.80	6.54	0.84	1.06	1.32
Italy - Enterococcus faecalis and E. faecium VRE OTH - model 70	1.33	1.84	2.59	0.00	0.00	0.00	1.33	1.84	2.59	1.30E-03	1.64E-03	2.06E-03	2.19E-03	3.02E-03	4.26E-03
Italy - Enterococcus faecalis and E. faecium VRE RESP - model 67	0.00	0.00	0.00	0.00	0.00	0.00	0.00	0.00	0.00	NaN	NaN	NaN	0.00	0.00	0.00
Italy - Enterococcus faecalis and E. faecium VRE SSI - model 69	2.07	3.06	4.51	473.65	654.30	864.14	475.33	658.36	868.32	0.40	0.45	0.50	3.40E-03	5.03E-03	7.42E-03
Italy - Enterococcus faecalis and E. faecium VRE UTI - model 68	0.71	1.01	1.43	0.00	0.00	0.00	0.71	1.01	1.43	1.49E-03	1.87E-03	2.30E-03	1.17E-03	1.67E-03	2.35E-03
Italy - Escherichia coli 3GCREC BSI - model 16	5,280.83	6,033.34	6,855.80	33,916.01	38,834.30	45,030.80	39,416.51	44,932.84	51,542.39	3.21	3.55	3.88	8.69	9.92	11.28
Italy - Escherichia coli 3GCREC OTH - model 20	10.58	14.76	20.40	0.00	0.00	0.00	10.58	14.76	20.40	2.73E-03	3.35E-03	4.16E-03	0.02	0.02	0.03
Italy - Escherichia coli 3GCREC RESP - model 17	1,579.93	1,946.66	2,297.82	3,039.18	3,761.74	4,474.03	4,639.63	5,705.94	6,754.94	0.86	0.97	1.11	2.60	3.20	3.78
Italy - Escherichia coli 3GCREC SSI - model 19	20.77	29.23	40.60	4,473.94	5,520.13	6,643.01	4,500.55	5,547.99	6,686.25	0.37	0.40	0.44	0.03	0.05	0.07
Italy - Escherichia coli 3GCREC UTI - model 18	80.12	107.32	141.25	0.00	0.00	0.00	80.12	107.32	141.25	1.54E-03	1.95E-03	2.39E-03	0.13	0.18	0.23
Italy - Escherichia coli CREC BSI - model 21	57.36	71.48	91.45	713.91	947.25	1,299.83	772.80	1,017.47	1,366.95	9.50	12.17	15.16	0.09	0.12	0.15
Italy - Escherichia coli CREC OTH - model 25	0.12	0.17	0.25	0.00	0.00	0.00	0.12	0.17	0.25	2.63E-03	3.49E-03	4.64E-03	1.97E-04	2.87E-04	4.13E-04
Italy - Escherichia coli CREC RESP - model 22	17.66	26.07	35.91	34.39	50.70	70.14	52.47	76.47	105.15	1.36	1.73	2.16	0.03	0.04	0.06
Italy - Escherichia coli CREC SSI - model 24	0.15	0.22	0.30	35.56	45.01	55.11	35.74	45.19	54.88	0.40	0.44	0.49	2.41E-04	3.61E-04	4.97E-04
Italy - Escherichia coli CREC UTI - model 23	0.54	0.75	1.02	0.00	0.00	0.00	0.54	0.75	1.02	1.60E-03	2.09E-03	2.64E-03	8.89E-04	1.24E-03	1.68E-03
Italy - Escherichia coli ColREC BSI - model 26	127.90	148.61	170.69	2,685.27	3,238.68	3,856.43	2,817.47	3,387.72	4,013.44	10.16	11.92	13.88	0.21	0.24	0.28
Italy - Escherichia coli ColREC OTH - model 30	0.41	0.58	0.87	0.00	0.00	0.00	0.41	0.58	0.87	2.62E-03	3.50E-03	4.75E-03	6.67E-04	9.56E-04	1.43E-03
Italy - Escherichia coli ColREC RESP - model 27	42.52	53.11	65.16	82.65	102.03	121.82	124.77	154.89	185.63	0.88	1.04	1.24	0.07	0.09	0.11
Italy - Escherichia coli ColREC SSI - model 29	0.50	0.72	1.07	109.58	137.05	165.61	110.05	137.65	166.49	0.36	0.40	0.45	8.25E-04	1.19E-03	1.76E-03
Italy - Escherichia coli ColREC UTI - model 28	1.79	2.47	3.48	0.00	0.00	0.00	1.79	2.47	3.48	1.58E-03	2.07E-03	2.66E-03	2.95E-03	4.07E-03	5.72E-03
Italy - Klebsiella pneumoniae 3GCRKP BSI - model 1	2,017.76	2,265.71	2,548.33	12,844.56	14,249.69	15,951.80	14,921.13	16,538.30	18,471.74	4.81	5.17	5.58	3.32	3.73	4.19
Italy - Klebsiella pneumoniae 3GCRKP OTH - model 5	3.43	4.42	5.72	0.00	0.00	0.00	3.43	4.42	5.72	2.75E-03	3.36E-03	4.03E-03	5.64E-03	7.27E-03	9.40E-03
Italy - Klebsiella pneumoniae 3GCRKP RESP - model 2	1,198.12	1,414.46	1,641.01	2,358.44	2,747.63	3,202.52	3,567.82	4,162.37	4,812.36	1.31	1.46	1.64	1.97	2.33	2.70
Italy - Klebsiella pneumoniae 3GCRKP SSI - model 4	2.12	2.83	3.75	490.73	587.43	695.25	493.29	590.49	698.51	0.41	0.45	0.48	3.48E-03	4.65E-03	6.17E-03
Italy - Klebsiella pneumoniae 3GCRKP UTI - model 3	5.99	7.62	9.56	0.00	0.00	0.00	5.99	7.62	9.56	1.63E-03	1.96E-03	2.33E-03	9.85E-03	0.01	0.02
Italy - Klebsiella pneumoniae CRKP BSI - model 6	2,136.76	2,463.07	2,803.93	27,362.27	32,172.48	37,565.39	29,592.92	34,647.55	40,226.68	8.22	9.27	10.37	3.51	4.05	4.61
Italy - Klebsiella pneumoniae CRKP OTH - model 10	4.80	6.46	8.56	0.00	0.00	0.00	4.80	6.46	8.56	2.82E-03	3.50E-03	4.31E-03	7.89E-03	0.01	0.01
Italy - Klebsiella pneumoniae CRKP RESP - model 7	610.89	756.72	899.22	1,187.56	1,464.67	1,760.91	1,791.00	2,220.40	2,648.78	1.19	1.32	1.46	1.00	1.24	1.48
Italy - Klebsiella pneumoniae CRKP SSI - model 9	1.77	2.49	3.53	394.51	512.17	649.94	395.74	514.50	653.13	0.40	0.44	0.48	2.91E-03	4.10E-03	5.81E-03
Italy - Klebsiella pneumoniae CRKP UTI - model 8	3.63	4.88	6.52	0.00	0.00	0.00	3.63	4.88	6.52	1.67E-03	2.07E-03	2.53E-03	5.96E-03	8.03E-03	0.01
Italy - Klebsiella pneumoniae ColRKP BSI - model 11	991.41	1,138.24	1,317.89	21,368.55	25,338.32	29,977.40	22,417.38	26,478.49	31,229.21	13.91	16.05	18.21	1.63	1.87	2.17
Italy - Klebsiella pneumoniae ColRKP OTH - model 15	2.05	2.85	3.74	0.00	0.00	0.00	2.05	2.85	3.74	2.78E-03	3.52E-03	4.35E-03	3.37E-03	4.68E-03	6.16E-03
Italy - Klebsiella pneumoniae ColRKP RESP - model 12	269.06	346.42	422.49	530.85	670.22	821.16	804.10	1,017.02	1,233.17	1.25	1.38	1.55	0.44	0.57	0.69
Italy - Klebsiella pneumoniae ColRKP SSI - model 14	0.78	1.11	1.48	174.51	225.45	290.36	175.18	226.67	292.26	0.40	0.44	0.48	1.28E-03	1.82E-03	2.44E-03
Italy - Klebsiella pneumoniae ColRKP UTI - model 13	1.63	2.15	2.85	0.00	0.00	0.00	1.63	2.15	2.85	1.69E-03	2.10E-03	2.56E-03	2.68E-03	3.54E-03	4.69E-03
Italy - Pseudomonas aeruginosa CRPA BSI - model 51	1,284.24	1,467.02	1,669.36	10,260.64	12,409.92	15,179.89	11,637.31	13,870.39	16,740.78	6.11	7.15	8.28	2.11	2.41	2.75
Italy - Pseudomonas aeruginosa CRPA OTH - model 55	2.83	3.73	4.78	0.00	0.00	0.00	2.83	3.73	4.78	3.06E-03	3.65E-03	4.34E-03	4.66E-03	6.14E-03	7.87E-03
Italy - Pseudomonas aeruginosa CRPA RESP - model 52	3,203.50	3,791.26	4,388.13	10,804.56	13,474.68	16,212.37	14,195.10	17,287.90	20,513.42	2.08	2.37	2.71	5.27	6.24	7.22
Italy - Pseudomonas aeruginosa CRPA SSI - model 54	2.18	2.97	4.03	486.79	615.97	751.50	489.14	618.84	756.98	0.41	0.45	0.49	3.58E-03	4.88E-03	6.63E-03
Italy - Pseudomonas aeruginosa CRPA UTI - model 53	3.96	5.30	6.61	0.00	0.00	0.00	3.96	5.30	6.61	1.72E-03	2.05E-03	2.40E-03	6.51E-03	8.71E-03	0.01
Italy - Pseudomonas aeruginosa ColRPA BSI - model 56	51.67	63.06	78.79	386.47	536.22	754.12	441.84	599.94	828.01	5.83	7.67	9.69	0.08	0.10	0.13
Italy - Pseudomonas aeruginosa ColRPA OTH - model 60	0.11	0.15	0.22	0.00	0.00	0.00	0.11	0.15	0.22	2.83E-03	3.71E-03	4.67E-03	1.76E-04	2.54E-04	3.65E-04
Italy - Pseudomonas aeruginosa ColRPA RESP - model 57	126.92	165.07	209.92	421.91	577.01	817.53	559.35	741.92	1,020.73	2.02	2.52	3.27	0.21	0.27	0.35
Italy - Pseudomonas aeruginosa ColRPA SSI - model 59	0.07	0.12	0.18	18.61	26.78	34.66	18.69	26.90	34.81	0.43	0.47	0.51	1.23E-04	1.98E-04	3.04E-04
Italy - Pseudomonas aeruginosa ColRPA UTI - model 58	0.15	0.21	0.30	0.00	0.00	0.00	0.15	0.21	0.30	1.55E-03	2.04E-03	2.61E-03	2.47E-04	3.53E-04	4.98E-04
Italy - Pseudomonas aeruginosa MDRPA BSI - model 46	124.83	149.52	175.81	886.48	1,158.77	1,465.22	1,024.93	1,310.93	1,628.30	3.84	4.72	5.66	0.21	0.25	0.29
Italy - Pseudomonas aeruginosa MDRPA OTH - model 50	0.38	0.55	0.73	0.00	0.00	0.00	0.38	0.55	0.73	3.03E-03	3.75E-03	4.49E-03	6.30E-04	9.04E-04	1.21E-03
Italy - Pseudomonas aeruginosa MDRPA RESP - model 47	316.54	388.27	464.35	991.37	1,301.30	1,643.63	1,323.89	1,688.87	2,098.57	1.33	1.61	1.91	0.52	0.64	0.76
Italy - Pseudomonas aeruginosa MDRPA SSI - model 49	0.28	0.42	0.59	56.98	72.80	90.07	57.35	73.20	90.57	0.33	0.37	0.40	4.55E-04	6.94E-04	9.77E-04
Italy - Pseudomonas aeruginosa MDRPA UTI - model 48	0.55	0.75	0.98	0.00	0.00	0.00	0.55	0.75	0.98	1.64E-03	2.05E-03	2.50E-03	9.10E-04	1.23E-03	1.61E-03
Italy - Staphylococcus aureus MRSA BSI - model 61	3,754.16	4,226.31	4,746.59	25,666.29	28,364.97	31,346.41	29,534.31	32,604.82	35,956.78	4.03	4.33	4.69	6.18	6.95	7.81
Italy - Staphylococcus aureus MRSA OTH - model 65	27.96	36.53	47.18	0.00	0.00	0.00	27.96	36.53	47.18	2.63E-03	3.24E-03	3.92E-03	0.05	0.06	0.08
Italy - Staphylococcus aureus MRSA RESP - model 62	3,853.80	4,479.30	5,261.58	7,545.08	8,694.19	10,007.97	11,467.00	13,189.14	15,179.54	1.04	1.15	1.27	6.34	7.37	8.65
Italy - Staphylococcus aureus MRSA SSI - model 64	19.82	26.88	34.57	4,503.78	5,327.85	6,217.80	4,533.08	5,354.04	6,240.04	0.40	0.43	0.46	0.03	0.04	0.06
Italy - Staphylococcus aureus MRSA UTI - model 63	2.70	3.64	5.14	0.00	0.00	0.00	2.70	3.64	5.14	1.50E-03	1.88E-03	2.30E-03	4.44E-03	5.99E-03	8.46E-03
Italy - Streptococcus pneumoniae PMRSP BSI - model 76	23.37	30.00	39.26	169.83	204.63	248.09	195.79	234.81	285.56	5.96	6.74	7.66	0.04	0.05	0.06
Italy - Streptococcus pneumoniae PMRSP OTH - model 80	0.02	0.02	0.04	0.00	0.00	0.00	0.02	0.02	0.04	1.25E-03	1.95E-03	2.81E-03	2.54E-05	4.03E-05	6.47E-05
Italy - Streptococcus pneumoniae PMRSP RESP - model 77	53.67	83.93	148.80	108.49	163.70	274.20	165.65	248.04	415.09	1.36	1.76	2.23	0.09	0.14	0.24
Italy - Streptococcus pneumoniae PMRSP SSI - model 79	0.00	0.00	0.00	0.00	0.00	0.00	0.00	0.00	0.00	NaN	NaN	NaN	0.00	0.00	0.00
Italy - Streptococcus pneumoniae PMRSP UTI - model 78	0.00	0.00	0.00	0.00	0.00	0.00	0.00	0.00	0.00	NaN	NaN	NaN	0.00	0.00	0.00
Italy - Streptococcus pneumoniae PRSP BSI - model 71	51.58	58.29	66.73	357.24	396.75	443.31	409.58	455.29	507.94	7.70	8.22	8.82	0.08	0.10	0.11
Italy - Streptococcus pneumoniae PRSP OTH - model 75	0.03	0.04	0.05	0.00	0.00	0.00	0.03	0.04	0.05	1.57E-03	1.96E-03	2.38E-03	5.21E-05	6.64E-05	8.64E-05
Italy - Streptococcus pneumoniae PRSP RESP - model 72	131.40	169.09	224.70	257.54	333.40	436.49	388.99	502.30	655.33	1.89	2.16	2.55	0.22	0.28	0.37
Italy - Streptococcus pneumoniae PRSP SSI - model 74	0.00	0.00	0.00	0.00	0.00	0.00	0.00	0.00	0.00	NaN	NaN	NaN	0.00	0.00	0.00
Italy - Streptococcus pneumoniae PRSP UTI - model 73	0.00	0.00	0.00	0.00	0.00	0.00	0.00	0.00	0.00	NaN	NaN	NaN	0.00	0.00	0.00
**Total (Median & 95% UIs)**	32,056.11	37,297.16	43,058.64	199,820.89	235,165.11	275,453.79	233,009.16	272,622.93	317,040.02		-		52.73	61.35	70.83
**Total (average)**		**37,384.89**			**235,742.11**			**273,127.00**			-			**61.49**	

Model	YLL per 100,000			DALY per 100,000			Cases per year			Incidence per 100,000			Deaths per year			Mortality per 100,000		
Italy - Acinetobacter spp. CRACI BSI - model 36	25.65	31.52	37.36	29.55	35.64	41.56	3,431.55	3,843.46	4,301.01	5.64	6.32	7.07	631.02	786.05	951.80	1.04	1.29	1.57
Italy - Acinetobacter spp. CRACI OTH - model 40	0.00	0.00	0.00	6.86E-04	9.57E-04	1.32E-03	116.22	156.41	196.85	0.19	0.26	0.32	0.00	0.00	0.00	0.00	0.00	0.00
Italy - Acinetobacter spp. CRACI RESP - model 37	5.29	6.16	6.97	7.99	9.32	10.54	3,648.05	4,281.89	4,900.69	6.00	7.04	8.06	130.32	154.00	178.04	0.21	0.25	0.29
Italy - Acinetobacter spp. CRACI SSI - model 39	0.83	1.04	1.26	0.83	1.04	1.26	1,225.22	1,479.02	1,742.89	2.02	2.43	2.87	28.12	36.39	45.21	0.05	0.06	0.07
Italy - Acinetobacter spp. CRACI UTI - model 38	0.00	0.00	0.00	4.19E-03	5.52E-03	7.13E-03	1,349.42	1,649.31	1,964.80	2.22	2.71	3.23	0.00	0.00	0.00	0.00	0.00	0.00
Italy - Acinetobacter spp. ColRACI BSI - model 41	0.76	1.10	1.48	0.90	1.24	1.64	101.64	120.35	139.86	0.17	0.20	0.23	17.88	24.87	33.35	0.03	0.04	0.05
Italy - Acinetobacter spp. ColRACI OTH - model 45	0.00	0.00	0.00	1.81E-05	2.93E-05	4.43E-05	3.13	4.82	6.81	5.15E-03	7.93E-03	0.01	0.00	0.00	0.00	0.00	0.00	0.00
Italy - Acinetobacter spp. ColRACI RESP - model 42	0.17	0.21	0.26	0.25	0.32	0.40	104.68	134.58	163.49	0.17	0.22	0.27	3.78	4.84	5.94	6.22E-03	7.96E-03	9.77E-03
Italy - Acinetobacter spp. ColRACI SSI - model 44	0.02	0.03	0.04	0.02	0.03	0.04	33.85	46.29	59.52	0.06	0.08	0.10	0.72	1.06	1.46	1.18E-03	1.75E-03	2.39E-03
Italy - Acinetobacter spp. ColRACI UTI - model 43	0.00	0.00	0.00	1.14E-04	1.73E-04	2.46E-04	38.43	51.93	67.30	0.06	0.09	0.11	0.00	0.00	0.00	0.00	0.00	0.00
Italy - Acinetobacter spp. MDRACI BSI - model 31	0.39	0.48	0.59	0.45	0.55	0.66	81.79	97.49	116.87	0.13	0.16	0.19	14.06	19.41	25.80	0.02	0.03	0.04
Italy - Acinetobacter spp. MDRACI OTH - model 35	0.00	0.00	0.00	1.46E-05	2.38E-05	3.79E-05	2.53	3.91	5.53	4.16E-03	6.42E-03	9.09E-03	0.00	0.00	0.00	0.00	0.00	0.00
Italy - Acinetobacter spp. MDRACI RESP - model 32	0.08	0.10	0.11	0.12	0.15	0.17	84.61	108.07	134.12	0.14	0.18	0.22	3.00	3.89	4.87	4.93E-03	6.40E-03	8.02E-03
Italy - Acinetobacter spp. MDRACI SSI - model 34	0.02	0.02	0.03	0.02	0.02	0.03	27.51	37.71	48.35	0.05	0.06	0.08	0.82	1.17	1.56	1.35E-03	1.92E-03	2.56E-03
Italy - Acinetobacter spp. MDRACI UTI - model 33	0.00	0.00	0.00	9.24E-05	1.38E-04	2.05E-04	29.91	41.65	54.38	0.05	0.07	0.09	0.00	0.00	0.00	0.00	0.00	0.00
Italy - Enterococcus faecalis and E. faecium VRE BSI - model 66	7.29	9.10	11.18	8.07	10.15	12.45	832.03	1,058.52	1,327.58	1.37	1.74	2.18	190.70	242.17	303.81	0.31	0.40	0.50
Italy - Enterococcus faecalis and E. faecium VRE OTH - model 70	0.00	0.00	0.00	2.19E-03	3.02E-03	4.26E-03	867.22	1,132.41	1,380.43	1.43	1.86	2.27	0.00	0.00	0.00	0.00	0.00	0.00
Italy - Enterococcus faecalis and E. faecium VRE RESP - model 67	0.00	0.00	0.00	0.00	0.00	0.00	0.00	0.00	0.00	0.00	0.00	0.00	0.00	0.00	0.00	0.00	0.00	0.00
Italy - Enterococcus faecalis and E. faecium VRE SSI - model 69	0.78	1.08	1.42	0.78	1.08	1.43	1,085.97	1,456.15	1,846.49	1.79	2.40	3.04	27.09	39.09	52.44	0.04	0.06	0.09
Italy - Enterococcus faecalis and E. faecium VRE UTI - model 68	0.00	0.00	0.00	1.17E-03	1.67E-03	2.35E-03	401.13	543.89	693.63	0.66	0.89	1.14	0.00	0.00	0.00	0.00	0.00	0.00
Italy - Escherichia coli 3GCREC BSI - model 16	55.79	63.88	74.07	64.83	73.91	84.78	10,940.86	12,713.67	14,501.06	18.00	20.91	23.85	1,835.80	2,206.40	2,635.90	3.02	3.63	4.34
Italy - Escherichia coli 3GCREC OTH - model 20	0.00	0.00	0.00	0.02	0.02	0.03	3,321.97	4,409.72	5,460.09	5.46	7.25	8.98	0.00	0.00	0.00	0.00	0.00	0.00
Italy - Escherichia coli 3GCREC RESP - model 17	5.00	6.19	7.36	7.63	9.39	11.11	4,574.24	5,874.01	7,315.00	7.52	9.66	12.03	164.93	210.02	266.57	0.27	0.35	0.44
Italy - Escherichia coli 3GCREC SSI - model 19	7.36	9.08	10.93	7.40	9.13	11.00	11,238.00	13,789.50	16,615.15	18.48	22.68	27.33	332.12	409.11	507.38	0.55	0.67	0.83
Italy - Escherichia coli 3GCREC UTI - model 18	0.00	0.00	0.00	0.13	0.18	0.23	45,379.41	55,392.06	65,661.04	74.64	91.11	108.00	0.00	0.00	0.00	0.00	0.00	0.00
Italy - Escherichia coli CREC BSI - model 21	1.17	1.56	2.14	1.27	1.67	2.25	72.13	83.90	97.56	0.12	0.14	0.16	24.33	29.67	37.26	0.04	0.05	0.06
Italy - Escherichia coli CREC OTH - model 25	0.00	0.00	0.00	1.97E-04	2.87E-04	4.13E-04	39.61	50.03	62.05	0.07	0.08	0.10	0.00	0.00	0.00	0.00	0.00	0.00
Italy - Escherichia coli CREC RESP - model 22	0.06	0.08	0.12	0.09	0.13	0.17	33.90	44.13	55.11	0.06	0.07	0.09	1.21	1.58	1.97	1.99E-03	2.61E-03	3.24E-03
Italy - Escherichia coli CREC SSI - model 24	0.06	0.07	0.09	0.06	0.07	0.09	81.21	101.86	120.65	0.13	0.17	0.20	1.82	2.36	2.90	2.99E-03	3.89E-03	4.76E-03
Italy - Escherichia coli CREC UTI - model 23	0.00	0.00	0.00	8.89E-04	1.24E-03	1.68E-03	295.68	358.27	425.54	0.49	0.59	0.70	0.00	0.00	0.00	0.00	0.00	0.00
Italy - Escherichia coli ColREC BSI - model 26	4.42	5.33	6.34	4.63	5.57	6.60	239.79	283.74	336.88	0.39	0.47	0.55	133.21	170.04	217.04	0.22	0.28	0.36
Italy - Escherichia coli ColREC OTH - model 30	0.00	0.00	0.00	6.67E-04	9.56E-04	1.43E-03	128.27	168.00	214.02	0.21	0.28	0.35	0.00	0.00	0.00	0.00	0.00	0.00
Italy - Escherichia coli ColREC RESP - model 27	0.14	0.17	0.20	0.21	0.25	0.31	113.49	149.04	186.48	0.19	0.25	0.31	4.07	5.33	6.79	6.70E-03	8.77E-03	0.01
Italy - Escherichia coli ColREC SSI - model 29	0.18	0.23	0.27	0.18	0.23	0.27	271.72	344.32	418.12	0.45	0.57	0.69	7.55	10.15	12.83	0.01	0.02	0.02
Italy - Escherichia coli ColREC UTI - model 28	0.00	0.00	0.00	2.95E-03	4.07E-03	5.72E-03	987.49	1,204.79	1,479.19	1.62	1.98	2.43	0.00	0.00	0.00	0.00	0.00	0.00
Italy - Klebsiella pneumoniae 3GCRKP BSI - model 1	21.13	23.44	26.24	24.54	27.20	30.38	2,883.16	3,197.63	3,536.19	4.74	5.26	5.82	476.53	533.96	591.81	0.78	0.88	0.97
Italy - Klebsiella pneumoniae 3GCRKP OTH - model 5	0.00	0.00	0.00	5.64E-03	7.27E-03	9.40E-03	1,123.28	1,325.62	1,545.42	1.85	2.18	2.54	0.00	0.00	0.00	0.00	0.00	0.00
Italy - Klebsiella pneumoniae 3GCRKP RESP - model 2	3.88	4.52	5.27	5.87	6.85	7.92	2,452.03	2,853.01	3,281.20	4.03	4.69	5.40	87.42	102.67	118.57	0.14	0.17	0.20
Italy - Klebsiella pneumoniae 3GCRKP SSI - model 4	0.81	0.97	1.14	0.81	0.97	1.15	1,125.84	1,322.81	1,581.85	1.85	2.18	2.60	26.83	32.94	40.34	0.04	0.05	0.07
Italy - Klebsiella pneumoniae 3GCRKP UTI - model 3	0.00	0.00	0.00	9.85E-03	0.01	0.02	3,357.46	3,872.93	4,440.03	5.52	6.37	7.30	0.00	0.00	0.00	0.00	0.00	0.00
Italy - Klebsiella pneumoniae CRKP BSI - model 6	45.01	52.92	61.79	48.68	56.99	66.17	3,237.93	3,748.65	4,208.80	5.33	6.17	6.92	1,110.93	1,335.34	1,579.12	1.83	2.20	2.60
Italy - Klebsiella pneumoniae CRKP OTH - model 10	0.00	0.00	0.00	7.89E-03	0.01	0.01	1,468.12	1,863.37	2,227.63	2.41	3.06	3.66	0.00	0.00	0.00	0.00	0.00	0.00
Italy - Klebsiella pneumoniae CRKP RESP - model 7	1.95	2.41	2.90	2.95	3.65	4.36	1,371.24	1,684.07	2,011.76	2.26	2.77	3.31	49.00	60.45	72.71	0.08	0.10	0.12
Italy - Klebsiella pneumoniae CRKP SSI - model 9	0.65	0.84	1.07	0.65	0.85	1.07	939.57	1,170.50	1,427.97	1.55	1.93	2.35	22.03	29.32	37.79	0.04	0.05	0.06
Italy - Klebsiella pneumoniae CRKP UTI - model 8	0.00	0.00	0.00	5.96E-03	8.03E-03	0.01	1,924.96	2,373.88	2,841.61	3.17	3.90	4.67	0.00	0.00	0.00	0.00	0.00	0.00
Italy - Klebsiella pneumoniae ColRKP BSI - model 11	35.15	41.68	49.31	36.87	43.55	51.37	1,461.83	1,652.59	1,872.91	2.40	2.72	3.08	832.65	1,000.39	1,181.06	1.37	1.65	1.94
Italy - Klebsiella pneumoniae ColRKP OTH - model 15	0.00	0.00	0.00	3.37E-03	4.68E-03	6.16E-03	643.51	809.76	989.66	1.06	1.33	1.63	0.00	0.00	0.00	0.00	0.00	0.00
Italy - Klebsiella pneumoniae ColRKP RESP - model 12	0.87	1.10	1.35	1.32	1.67	2.03	571.23	736.52	899.87	0.94	1.21	1.48	20.51	26.64	32.39	0.03	0.04	0.05
Italy - Klebsiella pneumoniae ColRKP SSI - model 14	0.29	0.37	0.48	0.29	0.37	0.48	400.84	516.04	639.40	0.66	0.85	1.05	9.00	12.21	15.91	0.01	0.02	0.03
Italy - Klebsiella pneumoniae ColRKP UTI - model 13	0.00	0.00	0.00	2.68E-03	3.54E-03	4.69E-03	840.01	1,040.01	1,237.39	1.38	1.71	2.04	0.00	0.00	0.00	0.00	0.00	0.00
Italy - Pseudomonas aeruginosa CRPA BSI - model 51	16.88	20.41	24.97	19.14	22.81	27.54	1,696.83	1,947.55	2,209.51	2.79	3.20	3.63	358.12	441.32	550.71	0.59	0.73	0.91
Italy - Pseudomonas aeruginosa CRPA OTH - model 55	0.00	0.00	0.00	4.66E-03	6.14E-03	7.87E-03	832.04	1,024.92	1,219.63	1.37	1.69	2.01	0.00	0.00	0.00	0.00	0.00	0.00
Italy - Pseudomonas aeruginosa CRPA RESP - model 52	17.77	22.16	26.67	23.35	28.44	33.74	6,223.54	7,282.21	8,471.98	10.24	11.98	13.94	388.05	478.41	597.99	0.64	0.79	0.98
Italy - Pseudomonas aeruginosa CRPA SSI - model 54	0.80	1.01	1.24	0.80	1.02	1.25	1,123.48	1,386.63	1,655.40	1.85	2.28	2.72	23.29	31.10	38.22	0.04	0.05	0.06
Italy - Pseudomonas aeruginosa CRPA UTI - model 53	0.00	0.00	0.00	6.51E-03	8.71E-03	0.01	2,122.87	2,566.08	3,087.21	3.49	4.22	5.08	0.00	0.00	0.00	0.00	0.00	0.00
Italy - Pseudomonas aeruginosa ColRPA BSI - model 56	0.64	0.88	1.24	0.73	0.99	1.36	65.69	78.88	95.60	0.11	0.13	0.16	13.03	18.13	24.67	0.02	0.03	0.04
Italy - Pseudomonas aeruginosa ColRPA OTH - model 60	0.00	0.00	0.00	1.76E-04	2.54E-04	3.65E-04	31.05	41.80	55.07	0.05	0.07	0.09	0.00	0.00	0.00	0.00	0.00	0.00
Italy - Pseudomonas aeruginosa ColRPA RESP - model 57	0.69	0.95	1.34	0.92	1.22	1.68	232.08	298.01	366.83	0.38	0.49	0.60	14.13	19.42	26.38	0.02	0.03	0.04
Italy - Pseudomonas aeruginosa ColRPA SSI - model 59	0.03	0.04	0.06	0.03	0.04	0.06	40.64	56.99	71.92	0.07	0.09	0.12	0.82	1.26	1.74	1.35E-03	2.07E-03	2.85E-03
Italy - Pseudomonas aeruginosa ColRPA UTI - model 58	0.00	0.00	0.00	2.47E-04	3.53E-04	4.98E-04	79.37	105.36	134.38	0.13	0.17	0.22	0.00	0.00	0.00	0.00	0.00	0.00
Italy - Pseudomonas aeruginosa MDRPA BSI - model 46	1.46	1.91	2.41	1.69	2.16	2.68	234.50	277.04	320.33	0.39	0.46	0.53	43.98	58.21	74.70	0.07	0.10	0.12
Italy - Pseudomonas aeruginosa MDRPA OTH - model 50	0.00	0.00	0.00	6.30E-04	9.04E-04	1.21E-03	110.41	146.85	181.71	0.18	0.24	0.30	0.00	0.00	0.00	0.00	0.00	0.00
Italy - Pseudomonas aeruginosa MDRPA RESP - model 47	1.63	2.14	2.70	2.18	2.78	3.45	854.91	1,044.22	1,256.83	1.41	1.72	2.07	49.40	64.86	82.91	0.08	0.11	0.14
Italy - Pseudomonas aeruginosa MDRPA SSI - model 49	0.09	0.12	0.15	0.09	0.12	0.15	155.17	198.69	248.53	0.26	0.33	0.41	3.68	5.12	6.53	6.06E-03	8.42E-03	0.01
Italy - Pseudomonas aeruginosa MDRPA UTI - model 48	0.00	0.00	0.00	9.10E-04	1.23E-03	1.61E-03	295.04	369.46	446.53	0.49	0.61	0.73	0.00	0.00	0.00	0.00	0.00	0.00
Italy - Staphylococcus aureus MRSA BSI - model 61	42.22	46.66	51.56	48.58	53.63	59.14	6,717.11	7,520.15	8,359.43	11.05	12.37	13.75	1,199.90	1,352.58	1,506.38	1.97	2.22	2.48
Italy - Staphylococcus aureus MRSA OTH - model 65	0.00	0.00	0.00	0.05	0.06	0.08	9,617.43	11,343.18	13,231.13	15.82	18.66	21.76	0.00	0.00	0.00	0.00	0.00	0.00
Italy - Staphylococcus aureus MRSA RESP - model 62	12.41	14.30	16.46	18.86	21.69	24.97	9,687.17	11,487.03	13,302.64	15.93	18.89	21.88	349.21	414.08	482.85	0.57	0.68	0.79
Italy - Staphylococcus aureus MRSA SSI - model 64	7.41	8.76	10.23	7.46	8.81	10.26	10,751.95	12,569.28	14,579.36	17.69	20.67	23.98	295.16	356.43	425.53	0.49	0.59	0.70
Italy - Staphylococcus aureus MRSA UTI - model 63	0.00	0.00	0.00	4.44E-03	5.99E-03	8.46E-03	1,550.54	1,958.29	2,440.03	2.55	3.22	4.01	0.00	0.00	0.00	0.00	0.00	0.00
Italy - Streptococcus pneumoniae PMRSP BSI - model 76	0.28	0.34	0.41	0.32	0.39	0.47	30.67	34.86	39.35	0.05	0.06	0.06	5.43	6.27	7.28	8.93E-03	0.01	0.01
Italy - Streptococcus pneumoniae PMRSP OTH - model 80	0.00	0.00	0.00	2.54E-05	4.03E-05	6.47E-05	9.92	12.81	16.88	0.02	0.02	0.03	0.00	0.00	0.00	0.00	0.00	0.00
Italy - Streptococcus pneumoniae PMRSP RESP - model 77	0.18	0.27	0.45	0.27	0.41	0.68	100.57	142.65	208.55	0.17	0.23	0.34	3.55	5.15	7.57	5.84E-03	8.47E-03	0.01
Italy - Streptococcus pneumoniae PMRSP SSI - model 79	0.00	0.00	0.00	0.00	0.00	0.00	0.00	0.00	0.00	0.00	0.00	0.00	0.00	0.00	0.00	0.00	0.00	0.00
Italy - Streptococcus pneumoniae PMRSP UTI - model 78	0.00	0.00	0.00	0.00	0.00	0.00	0.00	0.00	0.00	0.00	0.00	0.00	0.00	0.00	0.00	0.00	0.00	0.00
Italy - Streptococcus pneumoniae PRSP BSI - model 71	0.59	0.65	0.73	0.67	0.75	0.84	51.59	55.41	59.67	0.08	0.09	0.10	9.21	9.97	10.82	0.02	0.02	0.02
Italy - Streptococcus pneumoniae PRSP OTH - model 75	0.00	0.00	0.00	5.21E-05	6.64E-05	8.64E-05	18.16	20.71	24.04	0.03	0.03	0.04	0.00	0.00	0.00	0.00	0.00	0.00
Italy - Streptococcus pneumoniae PRSP RESP - model 72	0.42	0.55	0.72	0.64	0.83	1.08	192.94	232.19	284.46	0.32	0.38	0.47	6.88	8.37	10.35	0.01	0.01	0.02
Italy - Streptococcus pneumoniae PRSP SSI - model 74	0.00	0.00	0.00	0.00	0.00	0.00	0.00	0.00	0.00	0.00	0.00	0.00	0.00	0.00	0.00	0.00	0.00	0.00
Italy - Streptococcus pneumoniae PRSP UTI - model 73	0.00	0.00	0.00	0.00	0.00	0.00	0.00	0.00	0.00	0.00	0.00	0.00	0.00	0.00	0.00	0.00	0.00	0.00
**Total (Median & 95% UIs)**	328.68	386.81	453.08	383.27	448.43	521.49	167,809.32	201,583.57	237,207.25	276.02	331.58	390.17	8,951.28	10,762.23	12,817.23	14.72	17.70	21.08
**Total (average)**		**387.76**			**449.25**			**201,845.48**			**332.01**			**10,785.46**			**17.74**	

Infection	YLD			YLL			DALY			DALY per Case			YLD per 100,000			YLL per 100,000			DALY per 100,000			Cases per year			Incidence per 100,000			Deaths per year			Mortality per 100,000		
Colistin resistant *Acinetobacter spp.*	0.00	0.00	0.00	0.00	0.00	0.00	0.00	0.00	0.00	0.00	0.00	0.00	0.00	0.00	0.00	0.00	0.00	0.00	0.00	0.00	0.00	0.00	0.00	0.00	0.00	0.00	0.00	0.00	0.00	0.00	0.00	0.00	0.00	0.00	0.00	0.00
Carbapenem resistant *Acinetobacter spp.* (excluding those resistant to colistin)	75.41	83.58	92.50	372.67	449.84	527.43	450.90	533.16	617.38	6.97	8.02	9.06	2.58	2.86	3.17	12.76	15.40	18.05	15.44	18.25	21.13	176.55	196.51	217.08	6.04	6.73	7.43	14.17	17.27	20.14	0.49	0.59	0.69
Aminoglycosides and fluoroquinolones resistant *Acinetobacter* spp. (excluding those resistant to colistin and/or carbapenem)	6.35	7.46	8.65	29.41	39.04	48.56	36.27	46.49	56.61	3.75	4.64	5.50	0.22	0.26	0.30	1.01	1.34	1.66	1.24	1.59	1.94	25.51	29.95	34.40	0.87	1.03	1.18	1.97	2.58	3.19	0.07	0.09	0.11
Vancomycin resistant Enterococci (*E. faecalis* and *E. faecium*)	6.50	8.01	9.90	62.67	75.69	91.56	69.22	83.77	101.30	4.13	4.71	5.34	0.22	0.27	0.34	2.15	2.59	3.13	2.37	2.87	3.47	53.76	68.52	84.16	1.84	2.35	2.88	3.74	4.67	5.71	0.13	0.16	0.20
Colistin resistant *Escherichia coli*	0.00	0.00	0.00	0.00	0.00	0.00	0.00	0.00	0.00	0.00	0.00	0.00	0.00	0.00	0.00	0.00	0.00	0.00	0.00	0.00	0.00	0.00	0.00	0.00	0.00	0.00	0.00	0.00	0.00	0.00	0.00	0.00	0.00
Carbapenem resistant *Escherichia coli* (excluding those resistant to colistin)	0.00	0.00	0.00	0.00	0.00	0.00	0.00	0.00	0.00	0.00	0.00	0.00	0.00	0.00	0.00	0.00	0.00	0.00	0.00	0.00	0.00	0.00	0.00	0.00	0.00	0.00	0.00	0.00	0.00	0.00	0.00	0.00	0.00
Third-generation cephalosporin resistant *Escherichia coli* (excluding those resistant to colistin and/or carbapenem)	70.49	78.95	88.64	410.82	469.36	522.30	490.04	547.94	606.75	5.23	5.66	6.12	2.41	2.70	3.03	14.06	16.07	17.88	16.77	18.76	20.77	687.20	772.65	858.82	23.52	26.45	29.40	20.74	23.66	26.60	0.71	0.81	0.91
Colistin resistant *Klebsiella pneumoniae*	0.00	0.00	0.00	0.00	0.00	0.00	0.00	0.00	0.00	0.00	0.00	0.00	0.00	0.00	0.00	0.00	0.00	0.00	0.00	0.00	0.00	0.00	0.00	0.00	0.00	0.00	0.00	0.00	0.00	0.00	0.00	0.00	0.00
Carbapenem resistant *Klebsiella pneumoniae* (excluding those resistant to colistin)	0.00	0.00	0.00	0.00	0.00	0.00	0.00	0.00	0.00	0.00	0.00	0.00	0.00	0.00	0.00	0.00	0.00	0.00	0.00	0.00	0.00	0.00	0.00	0.00	0.00	0.00	0.00	0.00	0.00	0.00	0.00	0.00	0.00
Third-generation cephalosporin resistant *Klebsiella pneumoniae* (excluding those resistant to colistin and/or carbapenem)	78.63	86.62	95.73	385.44	415.69	447.15	466.56	502.27	539.45	4.97	5.21	5.46	2.69	2.97	3.28	13.19	14.23	15.31	15.97	17.19	18.47	359.04	399.21	438.45	12.29	13.67	15.01	20.12	21.80	23.62	0.69	0.75	0.81
Colistin resistant *Pseudomonas aeruginosa*	0.00	0.00	0.00	0.00	0.00	0.00	0.00	0.00	0.00	0.00	0.00	0.00	0.00	0.00	0.00	0.00	0.00	0.00	0.00	0.00	0.00	0.00	0.00	0.00	0.00	0.00	0.00	0.00	0.00	0.00	0.00	0.00	0.00
Carbapenem resistant *Pseudomonas aeruginosa* (excluding those resistant to colistin)	18.27	21.83	26.01	79.91	109.92	143.95	99.55	131.85	167.91	4.84	6.28	7.68	0.63	0.75	0.89	2.74	3.76	4.93	3.41	4.51	5.75	78.18	94.11	110.63	2.68	3.22	3.79	4.70	6.45	8.38	0.16	0.22	0.29
Pseudomonas aeruginosa resistant to three or more antimicrobial groups (excluding those resistant to colistin and/or carbapenem)	5.48	7.45	9.86	21.67	35.12	53.40	27.94	42.47	61.99	4.96	7.43	10.15	0.19	0.26	0.34	0.74	1.20	1.83	0.96	1.45	2.12	19.59	25.71	31.80	0.67	0.88	1.09	1.08	1.63	2.30	0.04	0.06	0.08
Methicillin resistant *Staphylococcus aureus*	38.13	42.24	46.77	187.18	202.33	217.27	226.38	244.80	262.53	5.62	5.90	6.18	1.31	1.45	1.60	6.41	6.93	7.44	7.75	8.38	8.99	194.32	213.15	231.73	6.65	7.30	7.93	9.20	9.99	10.79	0.32	0.34	0.37
Penicillin and macrolides resistant *Streptococcus pneumoniae* (excluding those only resistant to penicillin)	1.04	1.38	1.93	3.72	4.46	5.61	4.91	5.83	7.46	3.04	3.36	3.69	0.04	0.05	0.07	0.13	0.15	0.19	0.17	0.20	0.26	4.67	5.62	7.40	0.16	0.19	0.25	0.29	0.35	0.44	0.01	0.01	0.01
Penicillin tesistant *Streptococcus pneumoniae* (excluding those resistant to macrolides)	8.88	10.95	14.00	31.22	35.83	42.22	40.73	46.77	55.54	6.21	6.68	7.17	0.30	0.37	0.48	1.07	1.23	1.45	1.39	1.60	1.90	19.62	22.78	27.62	0.67	0.78	0.95	1.22	1.40	1.66	0.04	0.05	0.06
Total	309.17	348.47	394.00	1,584.69	1,837.29	2,099.45	1,912.49	2,185.36	2,476.93				10.58	11.93	13.49	54.25	62.89	71.87	65.47	74.81	84.79	1,618.44	1,828.20	2,042.07	55.40	62.58	69.90	77.23	89.81	102.83	2.64	3.07	3.52

Model	YLD			YLL			DALY			DALY per Case			YLD per 100,000			YLL per 100,000			DALY per 100,000			Cases per year			Incidence per 100,000			Deaths per year			Mortality per 100,000		
Lithuania - Acinetobacter spp. CRACI BSI - model 36	43.53	47.63	52.27	300.84	368.71	436.88	346.39	416.10	486.59	5.19	6.12	7.05	1.49	1.63	1.79	10.30	12.62	14.96	11.86	14.24	16.66	63.88	67.89	72.07	2.19	2.32	2.47	11.32	14.03	16.49	0.39	0.48	0.56
Lithuania - Acinetobacter spp. CRACI OTH - model 40	6.43E-03	8.95E-03	0.01	0.00	0.00	0.00	6.43E-03	8.95E-03	0.01	3.02E-03	3.75E-03	4.49E-03	2.20E-04	3.06E-04	4.19E-04	0.00	0.00	0.00	2.20E-04	3.06E-04	4.19E-04	1.81	2.42	3.05	0.06	0.08	0.10	0.00	0.00	0.00	0.00	0.00	0.00
Lithuania - Acinetobacter spp. CRACI RESP - model 37	31.79	35.83	40.08	62.58	69.88	77.33	95.15	105.70	117.45	1.35	1.43	1.53	1.09	1.23	1.37	2.14	2.39	2.65	3.26	3.62	4.02	66.26	73.68	81.23	2.27	2.52	2.78	2.39	2.66	2.96	0.08	0.09	0.10
Lithuania - Acinetobacter spp. CRACI SSI - model 39	0.04	0.05	0.07	9.26	11.25	13.21	9.30	11.30	13.26	0.43	0.45	0.48	1.40E-03	1.80E-03	2.45E-03	0.32	0.38	0.45	0.32	0.39	0.45	21.18	24.87	28.61	0.73	0.85	0.98	0.46	0.58	0.69	0.02	0.02	0.02
Lithuania - Acinetobacter spp. CRACI UTI - model 38	0.04	0.06	0.07	0.00	0.00	0.00	0.04	0.06	0.07	1.64E-03	2.05E-03	2.46E-03	1.51E-03	1.94E-03	2.45E-03	0.00	0.00	0.00	1.51E-03	1.94E-03	2.45E-03	23.42	27.65	32.12	0.80	0.95	1.10	0.00	0.00	0.00	0.00	0.00	0.00
Lithuania - Acinetobacter spp. CoRACI BSI - model 41	0.00	0.00	0.00	0.00	0.00	0.00	0.00	0.00	0.00	NaN	NaN	NaN	0.00	0.00	0.00	0.00	0.00	0.00	0.00	0.00	0.00	0.00	0.00	0.00	0.00	0.00	0.00	0.00	0.00	0.00	0.00	0.00	0.00
Lithuania - Acinetobacter spp. CoRACI OTH - model 45	0.00	0.00	0.00	0.00	0.00	0.00	0.00	0.00	0.00	NaN	NaN	NaN	0.00	0.00	0.00	0.00	0.00	0.00	0.00	0.00	0.00	0.00	0.00	0.00	0.00	0.00	0.00	0.00	0.00	0.00	0.00	0.00	0.00
Lithuania - Acinetobacter spp. CoRACI RESP - model 42	0.00	0.00	0.00	0.00	0.00	0.00	0.00	0.00	0.00	NaN	NaN	NaN	0.00	0.00	0.00	0.00	0.00	0.00	0.00	0.00	0.00	0.00	0.00	0.00	0.00	0.00	0.00	0.00	0.00	0.00	0.00	0.00	0.00
Lithuania - Acinetobacter spp. CoRACI SSI - model 44	0.00	0.00	0.00	0.00	0.00	0.00	0.00	0.00	0.00	NaN	NaN	NaN	0.00	0.00	0.00	0.00	0.00	0.00	0.00	0.00	0.00	0.00	0.00	0.00	0.00	0.00	0.00	0.00	0.00	0.00	0.00	0.00	0.00
Lithuania - Acinetobacter spp. CoRACI UTI - model 43	0.00	0.00	0.00	0.00	0.00	0.00	0.00	0.00	0.00	NaN	NaN	NaN	0.00	0.00	0.00	0.00	0.00	0.00	0.00	0.00	0.00	0.00	0.00	0.00	0.00	0.00	0.00	0.00	0.00	0.00	0.00	0.00	0.00
Lithuania - Acinetobacter spp. MDRACI BSI - model 31	3.68	4.25	4.84	23.24	31.58	39.77	27.45	35.83	44.14	2.67	3.45	4.19	0.13	0.15	0.17	0.80	1.08	1.36	0.94	1.23	1.51	9.53	10.38	11.29	0.33	0.36	0.39	1.55	2.06	2.58	0.05	0.07	0.09
Lithuania - Acinetobacter spp. MDRACI OTH - model 35	8.35E-04	1.35E-03	2.04E-03	0.00	0.00	0.00	8.35E-04	1.35E-03	2.04E-03	2.74E-03	3.74E-03	4.83E-03	2.86E-05	4.62E-05	6.99E-05	0.00	0.00	0.00	2.86E-05	4.62E-05	6.99E-05	0.24	0.37	0.52	8.26E-03	0.01	0.02	0.00	0.00	0.00	0.00	0.00	0.00
Lithuania - Acinetobacter spp. MDRACI RESP - model 32	2.66	3.19	3.77	5.15	6.13	7.17	7.80	9.31	10.83	0.75	0.83	0.92	0.09	0.11	0.13	0.18	0.21	0.25	0.27	0.32	0.37	9.57	11.23	12.92	0.33	0.38	0.44	0.34	0.41	0.47	0.01	0.01	0.02
Lithuania - Acinetobacter spp. MDRACI SSI - model 34	5.02E-03	7.96E-03	0.01	1.01	1.33	1.62	1.02	1.34	1.63	0.32	0.35	0.38	1.72E-04	2.73E-04	4.04E-04	0.03	0.05	0.06	0.03	0.05	0.06	2.96	3.79	4.55	0.10	0.13	0.16	0.08	0.11	0.14	2.85E-03	3.89E-03	4.76E-03
Lithuania - Acinetobacter spp. MDRACI UTI - model 33	5.54E-03	8.44E-03	0.01	0.00	0.00	0.00	5.54E-03	8.44E-03	0.01	1.51E-03	2.05E-03	2.67E-03	1.90E-04	2.89E-04	4.13E-04	0.00	0.00	0.00	1.90E-04	2.89E-04	4.13E-04	3.21	4.19	5.12	0.11	0.14	0.18	0.00	0.00	0.00	0.00	0.00	0.00
Lithuania - Enterococcus faecalis and E. faecium VRE BSI - model 66	6.43	7.92	9.76	55.91	67.02	80.90	62.39	75.01	90.51	3.80	4.34	4.93	0.22	0.27	0.33	1.91	2.29	2.77	2.14	2.57	3.10	14.14	17.35	21.12	0.48	0.59	0.72	3.23	3.97	4.83	0.11	0.14	0.17
Lithuania - Enterococcus faecalis and E. faecium VRE OTH - model 70	0.02	0.03	0.04	0.00	0.00	0.00	0.02	0.03	0.04	1.27E-03	1.63E-03	2.11E-03	7.20E-04	1.03E-03	1.43E-03	0.00	0.00	0.00	7.20E-04	1.03E-03	1.43E-03	14.37	18.50	22.59	0.49	0.63	0.77	0.00	0.00	0.00	0.00	0.00	0.00
Lithuania - Enterococcus faecalis and E. faecium VRE RESP - model 67	0.00	0.00	0.00	0.00	0.00	0.00	0.00	0.00	0.00	NaN	NaN	NaN	0.00	0.00	0.00	0.00	0.00	0.00	0.00	0.00	0.00	0.00	0.00	0.00	0.00	0.00	0.00	0.00	0.00	0.00	0.00	0.00	0.00
Lithuania - Enterococcus faecalis and E. faecium VRE SSI - model 69	0.03	0.05	0.08	6.75	8.67	10.66	6.80	8.72	10.72	0.33	0.37	0.40	1.15E-03	1.73E-03	2.57E-03	0.23	0.30	0.36	0.23	0.30	0.37	18.56	23.85	29.49	0.64	0.82	1.01	0.51	0.70	0.88	0.02	0.02	0.03
Lithuania - Enterococcus faecalis and E. faecium VRE UTI - model 68	0.01	0.02	0.02	0.00	0.00	0.00	0.01	0.02	0.02	1.45E-03	1.86E-03	2.40E-03	4.04E-04	5.61E-04	7.95E-04	0.00	0.00	0.00	4.04E-04	5.61E-04	7.95E-04	6.69	8.82	10.95	0.23	0.30	0.37	0.00	0.00	0.00	0.00	0.00	0.00
Lithuania - Escherichia coli 3GCREC BSI - model 16	54.09	59.53	65.80	338.61	385.53	425.87	401.40	444.65	487.61	3.79	4.11	4.44	1.85	2.04	2.25	11.59	13.20	14.58	13.74	15.22	16.69	100.74	108.44	116.73	3.45	3.71	4.00	16.64	18.79	20.93	0.57	0.64	0.72
Lithuania - Escherichia coli 3GCREC OTH - model 20	0.09	0.12	0.15	0.00	0.00	0.00	0.09	0.12	0.15	2.72E-03	3.33E-03	4.01E-03	3.06E-03	4.10E-03	5.24E-03	0.00	0.00	0.00	3.06E-03	4.10E-03	5.24E-03	28.96	35.94	42.35	0.99	1.23	1.45	0.00	0.00	0.00	0.00	0.00	0.00
Lithuania - Escherichia coli 3GCREC RESP - model 17	15.39	18.14	21.22	30.04	35.12	41.18	45.44	53.26	62.26	1.03	1.13	1.23	0.53	0.62	0.73	1.03	1.20	1.41	1.56	1.82	2.13	40.17	47.64	55.61	1.38	1.63	1.90	1.41	1.71	2.04	0.05	0.06	0.07
Lithuania - Escherichia coli 3GCREC SSI - model 19	0.18	0.24	0.32	42.17	48.71	55.26	42.37	49.00	55.59	0.41	0.43	0.45	6.21E-03	8.26E-03	0.01	1.44	1.67	1.89	1.45	1.68	1.90	99.96	114.60	129.65	3.42	3.92	4.44	2.68	3.16	3.63	0.09	0.11	0.12
Lithuania - Escherichia coli 3GCREC UTI - model 18	0.74	0.91	1.14	0.00	0.00	0.00	0.74	0.91	1.14	1.59E-03	1.96E-03	2.37E-03	0.03	0.03	0.04	0.00	0.00	0.00	0.03	0.03	0.04	417.36	466.02	514.48	14.29	15.95	17.61	0.00	0.00	0.00	0.00	0.00	0.00
Lithuania - Escherichia coli CREC BSI - model 21	0.00	0.00	0.00	0.00	0.00	0.00	0.00	0.00	0.00	NaN	NaN	NaN	0.00	0.00	0.00	0.00	0.00	0.00	0.00	0.00	0.00	0.00	0.00	0.00	0.00	0.00	0.00	0.00	0.00	0.00	0.00	0.00	0.00
Lithuania - Escherichia coli CREC OTH - model 25	0.00	0.00	0.00	0.00	0.00	0.00	0.00	0.00	0.00	NaN	NaN	NaN	0.00	0.00	0.00	0.00	0.00	0.00	0.00	0.00	0.00	0.00	0.00	0.00	0.00	0.00	0.00	0.00	0.00	0.00	0.00	0.00	0.00
Lithuania - Escherichia coli CREC RESP - model 22	0.00	0.00	0.00	0.00	0.00	0.00	0.00	0.00	0.00	NaN	NaN	NaN	0.00	0.00	0.00	0.00	0.00	0.00	0.00	0.00	0.00	0.00	0.00	0.00	0.00	0.00	0.00	0.00	0.00	0.00	0.00	0.00	0.00
Lithuania - Escherichia coli CREC SSI - model 24	0.00	0.00	0.00	0.00	0.00	0.00	0.00	0.00	0.00	NaN	NaN	NaN	0.00	0.00	0.00	0.00	0.00	0.00	0.00	0.00	0.00	0.00	0.00	0.00	0.00	0.00	0.00	0.00	0.00	0.00	0.00	0.00	0.00
Lithuania - Escherichia coli CREC UTI - model 23	0.00	0.00	0.00	0.00	0.00	0.00	0.00	0.00	0.00	NaN	NaN	NaN	0.00	0.00	0.00	0.00	0.00	0.00	0.00	0.00	0.00	0.00	0.00	0.00	0.00	0.00	0.00	0.00	0.00	0.00	0.00	0.00	0.00
Lithuania - Escherichia coli CoREC BSI - model 26	0.00	0.00	0.00	0.00	0.00	0.00	0.00	0.00	0.00	NaN	NaN	NaN	0.00	0.00	0.00	0.00	0.00	0.00	0.00	0.00	0.00	0.00	0.00	0.00	0.00	0.00	0.00	0.00	0.00	0.00	0.00	0.00	0.00
Lithuania - Escherichia coli CoREC OTH - model 30	0.00	0.00	0.00	0.00	0.00	0.00	0.00	0.00	0.00	NaN	NaN	NaN	0.00	0.00	0.00	0.00	0.00	0.00	0.00	0.00	0.00	0.00	0.00	0.00	0.00	0.00	0.00	0.00	0.00	0.00	0.00	0.00	0.00
Lithuania - Escherichia coli CoREC RESP - model 27	0.00	0.00	0.00	0.00	0.00	0.00	0.00	0.00	0.00	NaN	NaN	NaN	0.00	0.00	0.00	0.00	0.00	0.00	0.00	0.00	0.00	0.00	0.00	0.00	0.00	0.00	0.00	0.00	0.00	0.00	0.00	0.00	0.00
Lithuania - Escherichia coli CoREC SSI - model 29	0.00	0.00	0.00	0.00	0.00	0.00	0.00	0.00	0.00	NaN	NaN	NaN	0.00	0.00	0.00	0.00	0.00	0.00	0.00	0.00	0.00	0.00	0.00	0.00	0.00	0.00	0.00	0.00	0.00	0.00	0.00	0.00	0.00
Lithuania - Escherichia coli CoREC UTI - model 28	0.00	0.00	0.00	0.00	0.00	0.00	0.00	0.00	0.00	NaN	NaN	NaN	0.00	0.00	0.00	0.00	0.00	0.00	0.00	0.00	0.00	0.00	0.00	0.00	0.00	0.00	0.00	0.00	0.00	0.00	0.00	0.00	0.00
Lithuania - Klebsiella pneumoniae 3GCRKP BSI - model 1	49.51	53.84	58.50	315.84	336.81	358.98	367.34	390.54	414.61	3.60	3.75	3.90	1.69	1.84	2.00	10.81	11.53	12.29	12.57	13.37	14.19	98.69	104.40	109.89	3.38	3.57	3.76	16.30	17.43	18.70	0.56	0.60	0.64
Lithuania - Klebsiella pneumoniae 3GCRKP OTH - model 5	0.11	0.14	0.18	0.00	0.00	0.00	0.11	0.14	0.18	2.70E-03	3.34E-03	4.13E-03	3.68E-03	4.71E-03	6.05E-03	0.00	0.00	0.00	3.68E-03	4.71E-03	6.05E-03	35.91	41.49	46.89	1.23	1.42	1.61	0.00	0.00	0.00	0.00	0.00	0.00
Lithuania - Klebsiella pneumoniae 3GCRKP RESP - model 2	28.76	32.32	36.64	55.64	62.51	69.66	84.87	94.91	105.71	0.99	1.06	1.13	0.98	1.11	1.25	1.90	2.14	2.38	2.91	3.25	3.62	80.42	90.02	100.14	2.75	3.08	3.43	2.87	3.24	3.63	0.10	0.11	0.12
Lithuania - Klebsiella pneumoniae 3GCRKP SSI - model 4	0.06	0.09	0.12	13.96	16.36	18.52	14.05	16.45	18.65	0.38	0.40	0.42	2.17E-03	2.93E-03	3.94E-03	0.48	0.56	0.63	0.48	0.56	0.64	35.60	40.91	46.12	1.22	1.40	1.58	0.95	1.13	1.29	0.03	0.04	0.04
Lithuania - Klebsiella pneumoniae 3GCRKP UTI - model 3	0.18	0.24	0.30	0.00	0.00	0.00	0.18	0.24	0.30	1.59E-03	1.96E-03	2.36E-03	6.32E-03	8.19E-03	0.01	0.00	0.00	0.00	6.32E-03	8.19E-03	0.01	108.42	122.39	135.41	3.71	4.19	4.64	0.00	0.00	0.00	0.00	0.00	0.00
Lithuania - Klebsiella pneumoniae CRKP BSI - model 6	0.00	0.00	0.00	0.00	0.00	0.00	0.00	0.00	0.00	NaN	NaN	NaN	0.00	0.00	0.00	0.00	0.00	0.00	0.00	0.00	0.00	0.00	0.00	0.00	0.00	0.00	0.00	0.00	0.00	0.00	0.00	0.00	0.00
Lithuania - Klebsiella pneumoniae CRKP OTH - model 10	0.00	0.00	0.00	0.00	0.00	0.00	0.00	0.00	0.00	NaN	NaN	NaN	0.00	0.00	0.00	0.00	0.00	0.00	0.00	0.00	0.00	0.00	0.00	0.00	0.00	0.00	0.00	0.00	0.00	0.00	0.00	0.00	0.00
Lithuania - Klebsiella pneumoniae CRKP RESP - model 7	0.00	0.00	0.00	0.00	0.00	0.00	0.00	0.00	0.00	NaN	NaN	NaN	0.00	0.00	0.00	0.00	0.00	0.00	0.00	0.00	0.00	0.00	0.00	0.00	0.00	0.00	0.00	0.00	0.00	0.00	0.00	0.00	0.00
Lithuania - Klebsiella pneumoniae CRKP SSI - model 9	0.00	0.00	0.00	0.00	0.00	0.00	0.00	0.00	0.00	NaN	NaN	NaN	0.00	0.00	0.00	0.00	0.00	0.00	0.00	0.00	0.00	0.00	0.00	0.00	0.00	0.00	0.00	0.00	0.00	0.00	0.00	0.00	0.00
Lithuania - Klebsiella pneumoniae CRKP UTI - model 8	0.00	0.00	0.00	0.00	0.00	0.00	0.00	0.00	0.00	NaN	NaN	NaN	0.00	0.00	0.00	0.00	0.00	0.00	0.00	0.00	0.00	0.00	0.00	0.00	0.00	0.00	0.00	0.00	0.00	0.00	0.00	0.00	0.00
Lithuania - Klebsiella pneumoniae CoRKP BSI - model 11	0.00	0.00	0.00	0.00	0.00	0.00	0.00	0.00	0.00	NaN	NaN	NaN	0.00	0.00	0.00	0.00	0.00	0.00	0.00	0.00	0.00	0.00	0.00	0.00	0.00	0.00	0.00	0.00	0.00	0.00	0.00	0.00	0.00
Lithuania - Klebsiella pneumoniae CoRKP OTH - model 15	0.00	0.00	0.00	0.00	0.00	0.00	0.00	0.00	0.00	NaN	NaN	NaN	0.00	0.00	0.00	0.00	0.00	0.00	0.00	0.00	0.00	0.00	0.00	0.00	0.00	0.00	0.00	0.00	0.00	0.00	0.00	0.00	0.00
Lithuania - Klebsiella pneumoniae CoRKP RESP - model 12	0.00	0.00	0.00	0.00	0.00	0.00	0.00	0.00	0.00	NaN	NaN	NaN	0.00	0.00	0.00	0.00	0.00	0.00	0.00	0.00	0.00	0.00	0.00	0.00	0.00	0.00	0.00	0.00	0.00	0.00	0.00	0.00	0.00
Lithuania - Klebsiella pneumoniae CoRKP SSI - model 14	0.00	0.00	0.00	0.00	0.00	0.00	0.00	0.00	0.00	NaN	NaN	NaN	0.00	0.00	0.00	0.00	0.00	0.00	0.00	0.00	0.00	0.00	0.00	0.00	0.00	0.00	0.00	0.00	0.00	0.00	0.00	0.00	0.00
Lithuania - Klebsiella pneumoniae CoRKP UTI - model 13	0.00	0.00	0.00	0.00	0.00	0.00	0.00	0.00	0.00	NaN	NaN	NaN	0.00	0.00	0.00	0.00	0.00	0.00	0.00	0.00	0.00	0.00	0.00	0.00	0.00	0.00	0.00	0.00	0.00	0.00	0.00	0.00	0.00
Lithuania - Pseudomonas aeruginosa CRPA BSI - model 51	5.24	6.12	7.11	36.82	51.40	67.53	42.52	57.49	74.21	3.28	4.40	5.50	0.18	0.21	0.24	1.26	1.76	2.31	1.46	1.97	2.54	11.82	13.09	14.69	0.40	0.45	0.50	2.17	2.99	3.88	0.07	0.10	0.13
Lithuania - Pseudomonas aeruginosa CRPA OTH - model 55	0.02	0.02	0.03	0.00	0.00	0.00	0.02	0.02	0.03	2.78E-03	3.66E-03	4.63E-03	5.83E-04	8.20E-04	1.17E-03	0.00	0.00	0.00	5.83E-04	8.20E-04	1.17E-03	5.06	6.62	8.29	0.17	0.23	0.28	0.00	0.00	0.00	0.00	0.00	0.00
Lithuania - Pseudomonas aeruginosa CRPA RESP - model 52	12.97	15.63	18.79	40.35	54.84	71.84	54.25	70.61	89.02	1.17	1.46	1.74	0.44	0.54	0.64	1.38	1.88	2.46	1.86	2.42	3.05	40.80	48.35	56.02	1.40	1.66	1.92	2.34	3.20	4.17	0.08	0.11	0.14
Lithuania - Pseudomonas aeruginosa CRPA SSI - model 54	0.01	0.02	0.03	2.74	3.68	4.59	2.74	3.70	4.60	0.38	0.41	0.44	4.21E-04	6.58E-04	9.73E-04	0.09	0.13	0.16	0.09	0.13	0.16	6.81	9.01	11.18	0.23	0.31	0.38	0.19	0.26	0.33	6.38E-03	8.87E-03	0.01
Lithuania - Pseudomonas aeruginosa CRPA UTI - model 53	0.02	0.03	0.05	0.00	0.00	0.00	0.02	0.03	0.05	1.58E-03	2.04E-03	2.56E-03	8.39E-04	1.17E-03	1.61E-03	0.00	0.00	0.00	8.39E-04	1.17E-03	1.61E-03	13.69	17.04	20.45	0.47	0.58	0.70	0.00	0.00	0.00	0.00	0.00	0.00
Lithuania - Pseudomonas aeruginosa CoRPA BSI - model 56	0.00	0.00	0.00	0.00	0.00	0.00	0.00	0.00	0.00	NaN	NaN	NaN	0.00	0.00	0.00	0.00	0.00	0.00	0.00	0.00	0.00	0.00	0.00	0.00	0.00	0.00	0.00	0.00	0.00	0.00	0.00	0.00	0.00
Lithuania - Pseudomonas aeruginosa CoRPA OTH - model 60	0.00	0.00	0.00	0.00	0.00	0.00	0.00	0.00	0.00	NaN	NaN	NaN	0.00	0.00	0.00	0.00	0.00	0.00	0.00	0.00	0.00	0.00	0.00	0.00	0.00	0.00	0.00	0.00	0.00	0.00	0.00	0.00	0.00
Lithuania - Pseudomonas aeruginosa CoRPA RESP - model 57	0.00	0.00	0.00	0.00	0.00	0.00	0.00	0.00	0.00	NaN	NaN	NaN	0.00	0.00	0.00	0.00	0.00	0.00	0.00	0.00	0.00	0.00	0.00	0.00	0.00	0.00	0.00	0.00	0.00	0.00	0.00	0.00	0.00
Lithuania - Pseudomonas aeruginosa CoRPA SSI - model 59	0.00	0.00	0.00	0.00	0.00	0.00	0.00	0.00	0.00	NaN	NaN	NaN	0.00	0.00	0.00	0.00	0.00	0.00	0.00	0.00	0.00	0.00	0.00	0.00	0.00	0.00	0.00	0.00	0.00	0.00	0.00	0.00	0.00
Lithuania - Pseudomonas aeruginosa CoRPA UTI - model 58	0.00	0.00	0.00	0.00	0.00	0.00	0.00	0.00	0.00	NaN	NaN	NaN	0.00	0.00	0.00	0.00	0.00	0.00	0.00	0.00	0.00	0.00	0.00	0.00	0.00	0.00	0.00	0.00	0.00	0.00	0.00	0.00	0.00
Lithuania - Pseudomonas aeruginosa MDRPA BSI - model 46	1.65	2.09	2.72	9.63	16.49	25.03	11.67	18.59	27.23	3.28	5.25	7.25	0.06	0.07	0.09	0.33	0.56	0.86	0.40	0.64	0.93	3.04	3.57	4.13	0.10	0.12	0.14	0.49	0.75	1.09	0.02	0.03	0.04
Lithuania - Pseudomonas aeruginosa MDRPA OTH - model 50	3.86E-03	6.73E-03	0.01	0.00	0.00	0.00	3.86E-03	6.73E-03	0.01	2.51E-03	3.73E-03	5.15E-03	1.32E-04	2.30E-04	3.67E-04	0.00	0.00	0.00	1.32E-04	2.30E-04	3.67E-04	1.23	1.81	2.41	0.04	0.06	0.08	0.00	0.00	0.00	0.00	0.00	0.00
Lithuania - Pseudomonas aeruginosa MDRPA RESP - model 47	3.82	5.34	7.11	11.33	17.57	26.97	15.55	22.80	33.32	1.28	1.74	2.42	0.13	0.18	0.24	0.39	0.60	0.92	0.53	0.78	1.14	10.34	13.26	16.11	0.35	0.45	0.55	0.54	0.82	1.13	0.02	0.03	0.04
Lithuania - Pseudomonas aeruginosa MDRPA SSI - model 49	2.80E-03	5.12E-03	9.02E-03	0.71	1.06	1.40	0.71	1.07	1.41	0.40	0.43	0.47	9.59E-05	1.75E-04	3.09E-04	0.02	0.04	0.05	0.02	0.04	0.05	1.65	2.46	3.23	0.06	0.08	0.11	0.04	0.07	0.09	1.49E-03	2.27E-03	2.94E-03
Lithuania - Pseudomonas aeruginosa MDRPA UTI - model 48	5.73E-03	9.30E-03	0.01	0.00	0.00	0.00	5.73E-03	9.30E-03	0.01	1.34E-03	2.05E-03	2.80E-03	1.96E-04	3.18E-04	4.92E-04	0.00	0.00	0.00	1.96E-04	3.18E-04	4.92E-04	3.32	4.61	5.92	0.11	0.16	0.20	0.00	0.00	0.00	0.00	0.00	0.00
Lithuania - Staphylococcus aureus MRSA BSI - model 61	18.79	20.56	22.50	129.08	138.00	146.45	148.61	158.74	168.26	4.16	4.36	4.55	0.64	0.70	0.77	4.42	4.72	5.01	5.09	5.43	5.76	34.46	36.38	38.49	1.18	1.25	1.32	6.11	6.54	6.99	0.21	0.22	0.24
Lithuania - Staphylococcus aureus MRSA OTH - model 65	0.14	0.18	0.21	0.00	0.00	0.00	0.14	0.18	0.21	2.66E-03	3.26E-03	3.93E-03	4.77E-03	6.01E-03	7.34E-03	0.00	0.00	0.00	4.77E-03	6.01E-03	7.34E-03	48.70	53.81	58.13	1.67	1.84	1.99	0.00	0.00	0.00	0.00	0.00	0.00
Lithuania - Staphylococcus aureus MRSA RESP - model 62	19.09	21.36	23.87	37.40	41.56	45.94	56.82	62.96	69.06	1.08	1.15	1.23	0.65	0.73	0.82	1.28	1.42	1.57	1.95	2.16	2.36	49.49	54.47	59.77	1.69	1.86	2.05	1.76	1.96	2.16	0.06	0.07	0.07
Lithuania - Staphylococcus aureus MRSA SSI - model 64	0.10	0.13	0.17	20.69	22.77	24.87	20.79	22.91	24.97	0.37	0.38	0.40	3.28E-03	4.34E-03	5.70E-03	0.71	0.78	0.85	0.71	0.78	0.85	54.40	59.59	64.93	1.86	2.04	2.22	1.33	1.49	1.64	0.05	0.05	0.06
Lithuania - Staphylococcus aureus MRSA UTI - model 63	0.01	0.02	0.02	0.00	0.00	0.00	0.01	0.02	0.02	1.51E-03	1.87E-03	2.29E-03	4.23E-04	5.65E-04	7.46E-04	0.00	0.00	0.00	4.23E-04	5.65E-04	7.46E-04	7.27	8.89	10.41	0.25	0.30	0.36	0.00	0.00	0.00	0.00	0.00	0.00
Lithuania - Streptococcus pneumoniae PMRSP BSI - model 76	0.31	0.38	0.47	2.30	2.56	2.82	2.68	2.95	3.22	2.41	2.65	2.90	0.01	0.01	0.02	0.08	0.09	0.10	0.09	0.10	0.11	1.11	1.11	1.11	0.04	0.04	0.04	0.18	0.20	0.22	6.13E-03	6.84E-03	7.52E-03
Lithuania - Streptococcus pneumoniae PMRSP OTH - model 80	3.94E-04	7.35E-04	1.22E-03	0.00	0.00	0.00	3.94E-04	7.35E-04	1.22E-03	1.03E-03	1.91E-03	3.06E-03	1.35E-05	2.52E-05	4.16E-05	0.00	0.00	0.00	1.35E-05	2.52E-05	4.16E-05	0.33	0.39	0.46	0.01	0.01	0.02	0.00	0.00	0.00	0.00	0.00	0.00
Lithuania - Streptococcus pneumoniae PMRSP RESP - model 77	0.73	0.99	1.46	1.42	1.90	2.79	2.23	2.89	4.24	0.63	0.70	0.78	0.02	0.03	0.05	0.05	0.07	0.10	0.08	0.10	0.15	3.23	4.12	5.83	0.11	0.14	0.20	0.11	0.15	0.22	3.79E-03	5.08E-03	7.44E-03
Lithuania - Streptococcus pneumoniae PMRSP SSI - model 79	0.00	0.00	0.00	0.00	0.00	0.00	0.00	0.00	0.00	NaN	NaN	NaN	0.00	0.00	0.00	0.00	0.00	0.00	0.00	0.00	0.00	0.00	0.00	0.00	0.00	0.00	0.00	0.00	0.00	0.00	0.00	0.00	0.00
Lithuania - Streptococcus pneumoniae PMRSP UTI - model 78	0.00	0.00	0.00	0.00	0.00	0.00	0.00	0.00	0.00	NaN	NaN	NaN	0.00	0.00	0.00	0.00	0.00	0.00	0.00	0.00	0.00	0.00	0.00	0.00	0.00	0.00	0.00	0.00	0.00	0.00	0.00	0.00	0.00
Lithuania - Streptococcus pneumoniae PRSP BSI - model 71	2.59	3.01	3.48	18.92	20.44	22.05	21.88	23.47	25.07	4.93	5.29	5.65	0.09	0.10	0.12	0.65	0.70	0.75	0.75	0.80	0.86	4.44	4.44	4.44	0.15	0.15	0.15	0.74	0.80	0.86	0.03	0.03	0.03
Lithuania - Streptococcus pneumoniae PRSP OTH - model 75	1.90E-03	3.06E-03	4.48E-03	0.00	0.00	0.00	1.90E-03	3.06E-03	4.48E-03	1.23E-03	1.92E-03	2.81E-03	6.50E-05	1.05E-04	1.53E-04	0.00	0.00	0.00	6.50E-05	1.05E-04	1.53E-04	1.40	1.58	1.81	0.05	0.05	0.06	0.00	0.00	0.00	0.00	0.00	0.00
Lithuania - Streptococcus pneumoniae PRSP RESP - model 72	6.29	7.94	10.52	12.29	15.39	20.17	18.85	23.29	30.47	1.28	1.40	1.52	0.22	0.27	0.36	0.42	0.53	0.69	0.65	0.80	1.04	13.78	16.76	21.37	0.47	0.57	0.73	0.48	0.60	0.80	0.02	0.02	0.03
Lithuania - Streptococcus pneumoniae PRSP SSI - model 74	0.00	0.00	0.00	0.00	0.00	0.00	0.00	0.00	0.00	NaN	NaN	NaN	0.00	0.00	0.00	0.00	0.00	0.00	0.00	0.00	0.00	0.00	0.00	0.00	0.00	0.00	0.00	0.00	0.00	0.00	0.00	0.00	0.00
Lithuania - Streptococcus pneumoniae PRSP UTI - model 73	0.00	0.00	0.00	0.00	0.00	0.00	0.00	0.00	0.00	NaN	NaN	NaN	0.00	0.00	0.00	0.00	0.00	0.00	0.00	0.00	0.00	0.00	0.00	0.00	0.00	0.00	0.00	0.00	0.00	0.00	0.00	0.00	0.00
**Total (Median & 95% UIs)**	309.17	348.47	394.00	1,584.69	1,837.29	2,099.45	1,912.49	2,185.36	2,476.93		-		10.58	11.93	13.49	54.25	62.89	71.87	65.47	74.81	84.79	1,618.44	1,828.20	2,042.07	55.40	62.58	69.90	77.23	89.81	102.83	2.64	3.07	3.52
**Total (average)**		**349.35**			**1,838.94**			**2,188.28**			-			**11.96**			**62.95**			**74.91**			**1,828.61**			**62.60**			**89.85**			**3.08**	

Infection	YLD			YLL			DALY			DALY per Case			YLD per 100,000			YLL per 100,000			DALY per 100,000			Cases per year			Incidence per 100,000			Deaths per year			Mortality per 100,000		
Colistin resistant *Acinetobacter spp.*	0.00	0.00	0.00	0.00	0.00	0.00	0.00	0.00	0.00	0.00	0.00	0.00	0.00	0.00	0.00	0.00	0.00	0.00	0.00	0.00	0.00	0.00	0.00	0.00	0.00	0.00	0.00	0.00	0.00	0.00	0.00	0.00	0.00	0.00	0.00	0.00
Carbapenem resistant *Acinetobacter spp.* (excluding those resistant to colistin)	0.00	0.00	0.00	0.00	0.00	0.00	0.00	0.00	0.00	0.00	0.00	0.00	0.00	0.00	0.00	0.00	0.00	0.00	0.00	0.00	0.00	0.00	0.00	0.00	0.00	0.00	0.00	0.00	0.00	0.00	0.00	0.00	0.00	0.00	0.00	0.00
Aminoglycosides and fluoroquinolones resistant *Acinetobacter* spp. (excluding those resistant to colistin and/or carbapenem)	0.00	0.00	0.00	0.00	0.00	0.00	0.00	0.00	0.00	0.00	0.00	0.00	0.00	0.00	0.00	0.00	0.00	0.00	0.00	0.00	0.00	0.00	0.00	0.00	0.00	0.00	0.00	0.00	0.00	0.00	0.00	0.00	0.00	0.00	0.00	0.00
Vancomycin resistant Enterococci (*E. faecalis* and *E. faecium*)	0.10	0.18	0.37	0.86	1.75	3.16	0.96	1.93	3.49	1.42	1.48	1.53	0.02	0.03	0.07	0.15	0.31	0.56	0.17	0.34	0.62	1.76	5.06	9.04	0.31	0.90	1.61	0.17	0.35	0.63	0.03	0.06	0.11
Colistin resistant *Escherichia coli*	0.00	0.00	0.00	0.00	0.00	0.00	0.00	0.00	0.00	0.00	0.00	0.00	0.00	0.00	0.00	0.00	0.00	0.00	0.00	0.00	0.00	0.00	0.00	0.00	0.00	0.00	0.00	0.00	0.00	0.00	0.00	0.00	0.00	0.00	0.00	0.00
Carbapenem resistant *Escherichia coli* (excluding those resistant to colistin)	0.00	0.00	0.00	0.00	0.00	0.00	0.00	0.00	0.00	0.00	0.00	0.00	0.00	0.00	0.00	0.00	0.00	0.00	0.00	0.00	0.00	0.00	0.00	0.00	0.00	0.00	0.00	0.00	0.00	0.00	0.00	0.00	0.00	0.00	0.00	0.00
Third-generation cephalosporin resistant *Escherichia coli* (excluding those resistant to colistin and/or carbapenem)	24.27	28.38	32.43	145.46	171.00	196.61	171.28	199.40	227.14	4.27	4.80	5.37	4.31	5.04	5.76	25.84	30.37	34.92	30.42	35.42	40.35	280.78	328.68	378.56	49.88	58.38	67.25	8.63	10.27	12.00	1.53	1.82	2.13
Colistin resistant *Klebsiella pneumoniae*	0.00	0.00	0.00	0.00	0.00	0.00	0.00	0.00	0.00	0.00	0.00	0.00	0.00	0.00	0.00	0.00	0.00	0.00	0.00	0.00	0.00	0.00	0.00	0.00	0.00	0.00	0.00	0.00	0.00	0.00	0.00	0.00	0.00	0.00	0.00	0.00
Carbapenem resistant *Klebsiella pneumoniae* (excluding those resistant to colistin)	0.00	0.00	0.00	0.00	0.00	0.00	0.00	0.00	0.00	0.00	0.00	0.00	0.00	0.00	0.00	0.00	0.00	0.00	0.00	0.00	0.00	0.00	0.00	0.00	0.00	0.00	0.00	0.00	0.00	0.00	0.00	0.00	0.00	0.00	0.00	0.00
Third-generation cephalosporin resistant *Klebsiella pneumoniae* (excluding those resistant to colistin and/or carbapenem)	11.66	13.63	15.80	58.94	65.99	73.61	71.00	79.58	88.76	4.68	5.02	5.37	2.07	2.42	2.81	10.47	11.72	13.08	12.61	14.14	15.77	56.99	66.20	75.41	10.12	11.76	13.40	3.25	3.63	4.04	0.58	0.64	0.72
Colistin resistant *Pseudomonas aeruginosa*	0.00	0.00	0.00	0.00	0.00	0.00	0.00	0.00	0.00	0.00	0.00	0.00	0.00	0.00	0.00	0.00	0.00	0.00	0.00	0.00	0.00	0.00	0.00	0.00	0.00	0.00	0.00	0.00	0.00	0.00	0.00	0.00	0.00	0.00	0.00	0.00
Carbapenem resistant *Pseudomonas aeruginosa* (excluding those resistant to colistin)	2.57	3.90	5.45	9.14	19.75	33.02	12.34	23.59	37.93	4.15	7.04	9.98	0.46	0.69	0.97	1.62	3.51	5.87	2.19	4.19	6.74	9.69	15.35	21.17	1.72	2.73	3.76	0.49	1.05	1.73	0.09	0.19	0.31
Pseudomonas aeruginosa resistant to three or more antimicrobial groups (excluding those resistant to colistin and/or carbapenem)	0.00	0.00	0.00	0.00	0.00	0.00	0.00	0.00	0.00	0.00	0.00	0.00	0.00	0.00	0.00	0.00	0.00	0.00	0.00	0.00	0.00	0.00	0.00	0.00	0.00	0.00	0.00	0.00	0.00	0.00	0.00	0.00	0.00	0.00	0.00	0.00
Methicillin resistant *Staphylococcus aureus*	13.93	16.27	18.91	69.96	78.31	87.15	84.19	94.55	104.79	6.25	6.74	7.31	2.47	2.89	3.36	12.43	13.91	15.48	14.95	16.79	18.61	64.39	71.96	79.73	11.44	12.78	14.16	3.11	3.41	3.74	0.55	0.61	0.66
Penicillin and macrolides resistant *Streptococcus pneumoniae* (excluding those only resistant to penicillin)	0.00	0.00	0.00	0.00	0.00	0.00	0.00	0.00	0.00	0.00	0.00	0.00	0.00	0.00	0.00	0.00	0.00	0.00	0.00	0.00	0.00	0.00	0.00	0.00	0.00	0.00	0.00	0.00	0.00	0.00	0.00	0.00	0.00	0.00	0.00	0.00
Penicillin tesistant *Streptococcus pneumoniae* (excluding those resistant to macrolides)	0.00	0.00	0.00	0.00	0.00	0.00	0.00	0.00	0.00	0.00	0.00	0.00	0.00	0.00	0.00	0.00	0.00	0.00	0.00	0.00	0.00	0.00	0.00	0.00	0.00	0.00	0.00	0.00	0.00	0.00	0.00	0.00	0.00	0.00	0.00	0.00
Total	52.52	62.36	72.97	284.36	336.80	393.55	339.76	399.05	462.11				9.33	11.08	12.96	50.51	59.83	69.91	60.35	70.88	82.09	413.62	487.25	563.92	73.47	86.55	100.17	15.64	18.71	22.13	2.78	3.32	3.93

Model	YLD			YLL			DALY			DALY per Case			YLD per 100,000			YLL per 100,000			DALY per 100,000			Cases per year			Incidence per 100,000			Deaths per year			Mortality per 100,000		
Luxembourg - Acinetobacter spp. CRACI BSI - model 36	0.00	0.00	0.00	0.00	0.00	0.00	0.00	0.00	0.00	NaN	NaN	NaN	0.00	0.00	0.00	0.00	0.00	0.00	0.00	0.00	0.00	0.00	0.00	0.00	0.00	0.00	0.00	0.00	0.00	0.00	0.00	0.00	0.00	0.00	0.00	0.00
Luxembourg - Acinetobacter spp. CRACI OTH - model 40	0.00	0.00	0.00	0.00	0.00	0.00	0.00	0.00	0.00	NaN	NaN	NaN	0.00	0.00	0.00	0.00	0.00	0.00	0.00	0.00	0.00	0.00	0.00	0.00	0.00	0.00	0.00	0.00	0.00	0.00	0.00	0.00	0.00	0.00	0.00	0.00
Luxembourg - Acinetobacter spp. CRACI RESP - model 37	0.00	0.00	0.00	0.00	0.00	0.00	0.00	0.00	0.00	NaN	NaN	NaN	0.00	0.00	0.00	0.00	0.00	0.00	0.00	0.00	0.00	0.00	0.00	0.00	0.00	0.00	0.00	0.00	0.00	0.00	0.00	0.00	0.00	0.00	0.00	0.00
Luxembourg - Acinetobacter spp. CRACI SSI - model 39	0.00	0.00	0.00	0.00	0.00	0.00	0.00	0.00	0.00	NaN	NaN	NaN	0.00	0.00	0.00	0.00	0.00	0.00	0.00	0.00	0.00	0.00	0.00	0.00	0.00	0.00	0.00	0.00	0.00	0.00	0.00	0.00	0.00	0.00	0.00	0.00
Luxembourg - Acinetobacter spp. CRACI UTI - model 38	0.00	0.00	0.00	0.00	0.00	0.00	0.00	0.00	0.00	NaN	NaN	NaN	0.00	0.00	0.00	0.00	0.00	0.00	0.00	0.00	0.00	0.00	0.00	0.00	0.00	0.00	0.00	0.00	0.00	0.00	0.00	0.00	0.00	0.00	0.00	0.00
Luxembourg - Acinetobacter spp. ColRACI BSI - model 41	0.00	0.00	0.00	0.00	0.00	0.00	0.00	0.00	0.00	NaN	NaN	NaN	0.00	0.00	0.00	0.00	0.00	0.00	0.00	0.00	0.00	0.00	0.00	0.00	0.00	0.00	0.00	0.00	0.00	0.00	0.00	0.00	0.00	0.00	0.00	0.00
Luxembourg - Acinetobacter spp. ColRACI OTH - model 45	0.00	0.00	0.00	0.00	0.00	0.00	0.00	0.00	0.00	NaN	NaN	NaN	0.00	0.00	0.00	0.00	0.00	0.00	0.00	0.00	0.00	0.00	0.00	0.00	0.00	0.00	0.00	0.00	0.00	0.00	0.00	0.00	0.00	0.00	0.00	0.00
Luxembourg - Acinetobacter spp. ColRACI RESP - model 42	0.00	0.00	0.00	0.00	0.00	0.00	0.00	0.00	0.00	NaN	NaN	NaN	0.00	0.00	0.00	0.00	0.00	0.00	0.00	0.00	0.00	0.00	0.00	0.00	0.00	0.00	0.00	0.00	0.00	0.00	0.00	0.00	0.00	0.00	0.00	0.00
Luxembourg - Acinetobacter spp. ColRACI SSI - model 44	0.00	0.00	0.00	0.00	0.00	0.00	0.00	0.00	0.00	NaN	NaN	NaN	0.00	0.00	0.00	0.00	0.00	0.00	0.00	0.00	0.00	0.00	0.00	0.00	0.00	0.00	0.00	0.00	0.00	0.00	0.00	0.00	0.00	0.00	0.00	0.00
Luxembourg - Acinetobacter spp. ColRACI UTI - model 43	0.00	0.00	0.00	0.00	0.00	0.00	0.00	0.00	0.00	NaN	NaN	NaN	0.00	0.00	0.00	0.00	0.00	0.00	0.00	0.00	0.00	0.00	0.00	0.00	0.00	0.00	0.00	0.00	0.00	0.00	0.00	0.00	0.00	0.00	0.00	0.00
Luxembourg - Acinetobacter spp. MDRACI BSI - model 31	0.00	0.00	0.00	0.00	0.00	0.00	0.00	0.00	0.00	NaN	NaN	NaN	0.00	0.00	0.00	0.00	0.00	0.00	0.00	0.00	0.00	0.00	0.00	0.00	0.00	0.00	0.00	0.00	0.00	0.00	0.00	0.00	0.00	0.00	0.00	0.00
Luxembourg - Acinetobacter spp. MDRACI OTH - model 35	0.00	0.00	0.00	0.00	0.00	0.00	0.00	0.00	0.00	NaN	NaN	NaN	0.00	0.00	0.00	0.00	0.00	0.00	0.00	0.00	0.00	0.00	0.00	0.00	0.00	0.00	0.00	0.00	0.00	0.00	0.00	0.00	0.00	0.00	0.00	0.00
Luxembourg - Acinetobacter spp. MDRACI RESP - model 32	0.00	0.00	0.00	0.00	0.00	0.00	0.00	0.00	0.00	NaN	NaN	NaN	0.00	0.00	0.00	0.00	0.00	0.00	0.00	0.00	0.00	0.00	0.00	0.00	0.00	0.00	0.00	0.00	0.00	0.00	0.00	0.00	0.00	0.00	0.00	0.00
Luxembourg - Acinetobacter spp. MDRACI SSI - model 34	0.00	0.00	0.00	0.00	0.00	0.00	0.00	0.00	0.00	NaN	NaN	NaN	0.00	0.00	0.00	0.00	0.00	0.00	0.00	0.00	0.00	0.00	0.00	0.00	0.00	0.00	0.00	0.00	0.00	0.00	0.00	0.00	0.00	0.00	0.00	0.00
Luxembourg - Acinetobacter spp. MDRACI UTI - model 33	0.00	0.00	0.00	0.00	0.00	0.00	0.00	0.00	0.00	NaN	NaN	NaN	0.00	0.00	0.00	0.00	0.00	0.00	0.00	0.00	0.00	0.00	0.00	0.00	0.00	0.00	0.00	0.00	0.00	0.00	0.00	0.00	0.00	0.00	0.00	0.00
Luxembourg - Enterococcus faecalis and E. faecium VRE BSI - model 66	0.09	0.18	0.35	0.77	1.43	2.60	0.86	1.61	2.92	1.24	1.29	1.34	0.02	0.03	0.06	0.14	0.25	0.46	0.15	0.29	0.52	0.67	1.24	2.28	0.12	0.22	0.40	0.15	0.28	0.52	0.03	0.05	0.09
Luxembourg - Enterococcus faecalis and E. faecium VRE OTH - model 70	4.22E-04	2.03E-03	5.95E-03	0.00	0.00	0.00	4.22E-04	2.03E-03	5.95E-03	5.50E-04	1.55E-03	3.20E-03	7.49E-05	3.61E-04	1.06E-03	0.00	0.00	0.00	7.49E-05	3.61E-04	1.06E-03	0.38	1.39	2.43	0.07	0.25	0.43	0.00	0.00	0.00	0.00	0.00	0.00
Luxembourg - Enterococcus faecalis and E. faecium VRE RESP - model 67	0.00	0.00	0.00	0.00	0.00	0.00	0.00	0.00	0.00	NaN	NaN	NaN	0.00	0.00	0.00	0.00	0.00	0.00	0.00	0.00	0.00	0.00	0.00	0.00	0.00	0.00	0.00	0.00	0.00	0.00	0.00	0.00	0.00	0.00	0.00	0.00
Luxembourg - Enterococcus faecalis and E. faecium VRE SSI - model 69	6.37E-04	3.24E-03	0.01	0.10	0.32	0.56	0.10	0.33	0.56	0.18	0.18	0.19	1.13E-04	5.75E-04	1.90E-03	0.02	0.06	0.10	0.02	0.06	0.10	0.53	1.78	3.09	0.09	0.32	0.55	0.02	0.06	0.11	3.41E-03	0.01	0.02
Luxembourg - Enterococcus faecalis and E. faecium VRE UTI - model 68	2.16E-04	1.04E-03	3.03E-03	0.00	0.00	0.00	2.16E-04	1.04E-03	3.03E-03	7.07E-04	1.75E-03	3.44E-03	3.84E-05	1.84E-04	5.38E-04	0.00	0.00	0.00	3.84E-05	1.84E-04	5.38E-04	0.17	0.65	1.25	0.03	0.11	0.22	0.00	0.00	0.00	0.00	0.00	0.00
Luxembourg - Escherichia coli 3GCREC BSI - model 16	18.77	21.26	23.79	117.96	136.90	155.85	138.11	158.22	177.91	3.06	3.42	3.78	3.33	3.78	4.23	20.95	24.32	27.68	24.53	28.11	31.60	42.19	46.11	50.40	7.49	8.19	8.95	6.81	7.99	9.23	1.21	1.42	1.64
Luxembourg - Escherichia coli 3GCREC OTH - model 20	0.04	0.05	0.07	0.00	0.00	0.00	0.04	0.05	0.07	2.48E-03	3.31E-03	4.25E-03	6.29E-03	8.93E-03	0.01	0.00	0.00	0.00	6.29E-03	8.93E-03	0.01	11.51	15.23	19.03	2.04	2.70	3.38	0.00	0.00	0.00	0.00	0.00	0.00
Luxembourg - Escherichia coli 3GCREC RESP - model 17	5.11	6.57	7.90	9.96	12.70	15.48	15.16	19.25	23.25	0.81	0.94	1.09	0.91	1.17	1.40	1.77	2.26	2.75	2.69	3.42	4.13	15.70	20.41	25.46	2.79	3.63	4.52	0.56	0.74	0.92	0.10	0.13	0.16
Luxembourg - Escherichia coli 3GCREC SSI - model 19	0.07	0.10	0.15	17.54	21.40	25.28	17.68	21.50	25.39	0.40	0.44	0.49	0.01	0.02	0.03	3.12	3.80	4.49	3.14	3.82	4.51	40.93	48.69	57.25	7.27	8.65	10.17	1.26	1.54	1.85	0.22	0.27	0.33
Luxembourg - Escherichia coli 3GCREC UTI - model 18	0.29	0.39	0.52	0.00	0.00	0.00	0.29	0.39	0.52	1.50E-03	1.95E-03	2.53E-03	0.05	0.07	0.09	0.00	0.00	0.00	0.05	0.07	0.09	170.45	198.24	226.43	30.28	35.21	40.22	0.00	0.00	0.00	0.00	0.00	0.00
Luxembourg - Escherichia coli CREC BSI - model 21	0.00	0.00	0.00	0.00	0.00	0.00	0.00	0.00	0.00	NaN	NaN	NaN	0.00	0.00	0.00	0.00	0.00	0.00	0.00	0.00	0.00	0.00	0.00	0.00	0.00	0.00	0.00	0.00	0.00	0.00	0.00	0.00	0.00	0.00	0.00	0.00
Luxembourg - Escherichia coli CREC OTH - model 25	0.00	0.00	0.00	0.00	0.00	0.00	0.00	0.00	0.00	NaN	NaN	NaN	0.00	0.00	0.00	0.00	0.00	0.00	0.00	0.00	0.00	0.00	0.00	0.00	0.00	0.00	0.00	0.00	0.00	0.00	0.00	0.00	0.00	0.00	0.00	0.00
Luxembourg - Escherichia coli CREC RESP - model 22	0.00	0.00	0.00	0.00	0.00	0.00	0.00	0.00	0.00	NaN	NaN	NaN	0.00	0.00	0.00	0.00	0.00	0.00	0.00	0.00	0.00	0.00	0.00	0.00	0.00	0.00	0.00	0.00	0.00	0.00	0.00	0.00	0.00	0.00	0.00	0.00
Luxembourg - Escherichia coli CREC SSI - model 24	0.00	0.00	0.00	0.00	0.00	0.00	0.00	0.00	0.00	NaN	NaN	NaN	0.00	0.00	0.00	0.00	0.00	0.00	0.00	0.00	0.00	0.00	0.00	0.00	0.00	0.00	0.00	0.00	0.00	0.00	0.00	0.00	0.00	0.00	0.00	0.00
Luxembourg - Escherichia coli CREC UTI - model 23	0.00	0.00	0.00	0.00	0.00	0.00	0.00	0.00	0.00	NaN	NaN	NaN	0.00	0.00	0.00	0.00	0.00	0.00	0.00	0.00	0.00	0.00	0.00	0.00	0.00	0.00	0.00	0.00	0.00	0.00	0.00	0.00	0.00	0.00	0.00	0.00
Luxembourg - Escherichia coli ColREC BSI - model 26	0.00	0.00	0.00	0.00	0.00	0.00	0.00	0.00	0.00	NaN	NaN	NaN	0.00	0.00	0.00	0.00	0.00	0.00	0.00	0.00	0.00	0.00	0.00	0.00	0.00	0.00	0.00	0.00	0.00	0.00	0.00	0.00	0.00	0.00	0.00	0.00
Luxembourg - Escherichia coli ColREC OTH - model 30	0.00	0.00	0.00	0.00	0.00	0.00	0.00	0.00	0.00	NaN	NaN	NaN	0.00	0.00	0.00	0.00	0.00	0.00	0.00	0.00	0.00	0.00	0.00	0.00	0.00	0.00	0.00	0.00	0.00	0.00	0.00	0.00	0.00	0.00	0.00	0.00
Luxembourg - Escherichia coli ColREC RESP - model 27	0.00	0.00	0.00	0.00	0.00	0.00	0.00	0.00	0.00	NaN	NaN	NaN	0.00	0.00	0.00	0.00	0.00	0.00	0.00	0.00	0.00	0.00	0.00	0.00	0.00	0.00	0.00	0.00	0.00	0.00	0.00	0.00	0.00	0.00	0.00	0.00
Luxembourg - Escherichia coli ColREC SSI - model 29	0.00	0.00	0.00	0.00	0.00	0.00	0.00	0.00	0.00	NaN	NaN	NaN	0.00	0.00	0.00	0.00	0.00	0.00	0.00	0.00	0.00	0.00	0.00	0.00	0.00	0.00	0.00	0.00	0.00	0.00	0.00	0.00	0.00	0.00	0.00	0.00
Luxembourg - Escherichia coli ColREC UTI - model 28	0.00	0.00	0.00	0.00	0.00	0.00	0.00	0.00	0.00	NaN	NaN	NaN	0.00	0.00	0.00	0.00	0.00	0.00	0.00	0.00	0.00	0.00	0.00	0.00	0.00	0.00	0.00	0.00	0.00	0.00	0.00	0.00	0.00	0.00	0.00	0.00
Luxembourg - Klebsiella pneumoniae 3GCRKP BSI - model 1	7.34	8.44	9.62	48.39	53.04	58.15	56.12	61.44	67.25	3.33	3.55	3.77	1.30	1.50	1.71	8.60	9.42	10.33	9.97	10.91	11.95	16.09	17.30	18.68	2.86	3.07	3.32	2.65	2.89	3.16	0.47	0.51	0.56
Luxembourg - Klebsiella pneumoniae 3GCRKP OTH - model 5	0.02	0.02	0.03	0.00	0.00	0.00	0.02	0.02	0.03	2.43E-03	3.34E-03	4.30E-03	2.82E-03	4.03E-03	5.62E-03	0.00	0.00	0.00	2.82E-03	4.03E-03	5.62E-03	5.61	6.85	8.14	1.00	1.22	1.45	0.00	0.00	0.00	0.00	0.00	0.00
Luxembourg - Klebsiella pneumoniae 3GCRKP RESP - model 2	4.26	5.11	6.08	8.14	9.82	11.65	12.44	14.94	17.60	0.92	1.00	1.09	0.76	0.91	1.08	1.45	1.74	2.07	2.21	2.65	3.13	12.58	14.95	17.09	2.24	2.66	3.04	0.45	0.54	0.63	0.08	0.10	0.11
Luxembourg - Klebsiella pneumoniae 3GCRKP SSI - model 4	8.95E-03	0.01	0.02	2.40	3.13	3.80	2.41	3.14	3.82	0.42	0.46	0.50	1.59E-03	2.52E-03	3.88E-03	0.43	0.56	0.68	0.43	0.56	0.68	5.38	6.83	8.15	0.96	1.21	1.45	0.15	0.20	0.25	0.03	0.04	0.04
Luxembourg - Klebsiella pneumoniae 3GCRKP UTI - model 3	0.03	0.04	0.05	0.00	0.00	0.00	0.03	0.04	0.05	1.42E-03	1.96E-03	2.58E-03	5.09E-03	7.04E-03	9.49E-03	0.00	0.00	0.00	5.09E-03	7.04E-03	9.49E-03	17.33	20.27	23.36	3.08	3.60	4.15	0.00	0.00	0.00	0.00	0.00	0.00
Luxembourg - Klebsiella pneumoniae CRKP BSI - model 6	0.00	0.00	0.00	0.00	0.00	0.00	0.00	0.00	0.00	NaN	NaN	NaN	0.00	0.00	0.00	0.00	0.00	0.00	0.00	0.00	0.00	0.00	0.00	0.00	0.00	0.00	0.00	0.00	0.00	0.00	0.00	0.00	0.00	0.00	0.00	0.00
Luxembourg - Klebsiella pneumoniae CRKP OTH - model 10	0.00	0.00	0.00	0.00	0.00	0.00	0.00	0.00	0.00	NaN	NaN	NaN	0.00	0.00	0.00	0.00	0.00	0.00	0.00	0.00	0.00	0.00	0.00	0.00	0.00	0.00	0.00	0.00	0.00	0.00	0.00	0.00	0.00	0.00	0.00	0.00
Luxembourg - Klebsiella pneumoniae CRKP RESP - model 7	0.00	0.00	0.00	0.00	0.00	0.00	0.00	0.00	0.00	NaN	NaN	NaN	0.00	0.00	0.00	0.00	0.00	0.00	0.00	0.00	0.00	0.00	0.00	0.00	0.00	0.00	0.00	0.00	0.00	0.00	0.00	0.00	0.00	0.00	0.00	0.00
Luxembourg - Klebsiella pneumoniae CRKP SSI - model 9	0.00	0.00	0.00	0.00	0.00	0.00	0.00	0.00	0.00	NaN	NaN	NaN	0.00	0.00	0.00	0.00	0.00	0.00	0.00	0.00	0.00	0.00	0.00	0.00	0.00	0.00	0.00	0.00	0.00	0.00	0.00	0.00	0.00	0.00	0.00	0.00
Luxembourg - Klebsiella pneumoniae CRKP UTI - model 8	0.00	0.00	0.00	0.00	0.00	0.00	0.00	0.00	0.00	NaN	NaN	NaN	0.00	0.00	0.00	0.00	0.00	0.00	0.00	0.00	0.00	0.00	0.00	0.00	0.00	0.00	0.00	0.00	0.00	0.00	0.00	0.00	0.00	0.00	0.00	0.00
Luxembourg - Klebsiella pneumoniae ColRKP BSI - model 11	0.00	0.00	0.00	0.00	0.00	0.00	0.00	0.00	0.00	NaN	NaN	NaN	0.00	0.00	0.00	0.00	0.00	0.00	0.00	0.00	0.00	0.00	0.00	0.00	0.00	0.00	0.00	0.00	0.00	0.00	0.00	0.00	0.00	0.00	0.00	0.00
Luxembourg - Klebsiella pneumoniae ColRKP OTH - model 15	0.00	0.00	0.00	0.00	0.00	0.00	0.00	0.00	0.00	NaN	NaN	NaN	0.00	0.00	0.00	0.00	0.00	0.00	0.00	0.00	0.00	0.00	0.00	0.00	0.00	0.00	0.00	0.00	0.00	0.00	0.00	0.00	0.00	0.00	0.00	0.00
Luxembourg - Klebsiella pneumoniae ColRKP RESP - model 12	0.00	0.00	0.00	0.00	0.00	0.00	0.00	0.00	0.00	NaN	NaN	NaN	0.00	0.00	0.00	0.00	0.00	0.00	0.00	0.00	0.00	0.00	0.00	0.00	0.00	0.00	0.00	0.00	0.00	0.00	0.00	0.00	0.00	0.00	0.00	0.00
Luxembourg - Klebsiella pneumoniae ColRKP SSI - model 14	0.00	0.00	0.00	0.00	0.00	0.00	0.00	0.00	0.00	NaN	NaN	NaN	0.00	0.00	0.00	0.00	0.00	0.00	0.00	0.00	0.00	0.00	0.00	0.00	0.00	0.00	0.00	0.00	0.00	0.00	0.00	0.00	0.00	0.00	0.00	0.00
Luxembourg - Klebsiella pneumoniae ColRKP UTI - model 13	0.00	0.00	0.00	0.00	0.00	0.00	0.00	0.00	0.00	NaN	NaN	NaN	0.00	0.00	0.00	0.00	0.00	0.00	0.00	0.00	0.00	0.00	0.00	0.00	0.00	0.00	0.00	0.00	0.00	0.00	0.00	0.00	0.00	0.00	0.00	0.00
Luxembourg - Pseudomonas aeruginosa CRPA BSI - model 51	0.79	1.09	1.52	4.18	8.91	15.08	5.18	9.93	16.34	2.48	4.74	7.07	0.14	0.19	0.27	0.74	1.58	2.68	0.92	1.76	2.90	1.71	2.14	2.73	0.30	0.38	0.48	0.22	0.47	0.79	0.04	0.08	0.14
Luxembourg - Pseudomonas aeruginosa CRPA OTH - model 55	1.51E-03	3.80E-03	7.31E-03	0.00	0.00	0.00	1.51E-03	3.80E-03	7.31E-03	1.90E-03	3.69E-03	5.65E-03	2.69E-04	6.76E-04	1.30E-03	0.00	0.00	0.00	2.69E-04	6.76E-04	1.30E-03	0.53	1.08	1.61	0.09	0.19	0.29	0.00	0.00	0.00	0.00	0.00	0.00
Luxembourg - Pseudomonas aeruginosa CRPA RESP - model 52	1.77	2.79	3.91	4.44	9.85	16.51	6.63	12.65	20.12	1.02	1.62	2.19	0.31	0.50	0.69	0.79	1.75	2.93	1.18	2.25	3.57	5.17	7.95	10.81	0.92	1.41	1.92	0.24	0.52	0.86	0.04	0.09	0.15
Luxembourg - Pseudomonas aeruginosa CRPA SSI - model 54	9.93E-04	2.89E-03	6.36E-03	0.52	1.00	1.44	0.52	1.00	1.44	0.64	0.68	0.72	1.76E-04	5.13E-04	1.13E-03	0.09	0.18	0.26	0.09	0.18	0.26	0.71	1.47	2.11	0.13	0.26	0.38	0.03	0.05	0.08	4.57E-03	9.42E-03	0.01
Luxembourg - Pseudomonas aeruginosa CRPA UTI - model 53	2.35E-03	5.39E-03	0.01	0.00	0.00	0.00	2.35E-03	5.39E-03	0.01	1.09E-03	2.05E-03	3.23E-03	4.18E-04	9.57E-04	1.83E-03	0.00	0.00	0.00	4.18E-04	9.57E-04	1.83E-03	1.57	2.70	3.91	0.28	0.48	0.70	0.00	0.00	0.00	0.00	0.00	0.00
Luxembourg - Pseudomonas aeruginosa ColRPA BSI - model 56	0.00	0.00	0.00	0.00	0.00	0.00	0.00	0.00	0.00	NaN	NaN	NaN	0.00	0.00	0.00	0.00	0.00	0.00	0.00	0.00	0.00	0.00	0.00	0.00	0.00	0.00	0.00	0.00	0.00	0.00	0.00	0.00	0.00	0.00	0.00	0.00
Luxembourg - Pseudomonas aeruginosa ColRPA OTH - model 60	0.00	0.00	0.00	0.00	0.00	0.00	0.00	0.00	0.00	NaN	NaN	NaN	0.00	0.00	0.00	0.00	0.00	0.00	0.00	0.00	0.00	0.00	0.00	0.00	0.00	0.00	0.00	0.00	0.00	0.00	0.00	0.00	0.00	0.00	0.00	0.00
Luxembourg - Pseudomonas aeruginosa ColRPA RESP - model 57	0.00	0.00	0.00	0.00	0.00	0.00	0.00	0.00	0.00	NaN	NaN	NaN	0.00	0.00	0.00	0.00	0.00	0.00	0.00	0.00	0.00	0.00	0.00	0.00	0.00	0.00	0.00	0.00	0.00	0.00	0.00	0.00	0.00	0.00	0.00	0.00
Luxembourg - Pseudomonas aeruginosa ColRPA SSI - model 59	0.00	0.00	0.00	0.00	0.00	0.00	0.00	0.00	0.00	NaN	NaN	NaN	0.00	0.00	0.00	0.00	0.00	0.00	0.00	0.00	0.00	0.00	0.00	0.00	0.00	0.00	0.00	0.00	0.00	0.00	0.00	0.00	0.00	0.00	0.00	0.00
Luxembourg - Pseudomonas aeruginosa ColRPA UTI - model 58	0.00	0.00	0.00	0.00	0.00	0.00	0.00	0.00	0.00	NaN	NaN	NaN	0.00	0.00	0.00	0.00	0.00	0.00	0.00	0.00	0.00	0.00	0.00	0.00	0.00	0.00	0.00	0.00	0.00	0.00	0.00	0.00	0.00	0.00	0.00	0.00
Luxembourg - Pseudomonas aeruginosa MDRPA BSI - model 46	0.00	0.00	0.00	0.00	0.00	0.00	0.00	0.00	0.00	NaN	NaN	NaN	0.00	0.00	0.00	0.00	0.00	0.00	0.00	0.00	0.00	0.00	0.00	0.00	0.00	0.00	0.00	0.00	0.00	0.00	0.00	0.00	0.00	0.00	0.00	0.00
Luxembourg - Pseudomonas aeruginosa MDRPA OTH - model 50	0.00	0.00	0.00	0.00	0.00	0.00	0.00	0.00	0.00	NaN	NaN	NaN	0.00	0.00	0.00	0.00	0.00	0.00	0.00	0.00	0.00	0.00	0.00	0.00	0.00	0.00	0.00	0.00	0.00	0.00	0.00	0.00	0.00	0.00	0.00	0.00
Luxembourg - Pseudomonas aeruginosa MDRPA RESP - model 47	0.00	0.00	0.00	0.00	0.00	0.00	0.00	0.00	0.00	NaN	NaN	NaN	0.00	0.00	0.00	0.00	0.00	0.00	0.00	0.00	0.00	0.00	0.00	0.00	0.00	0.00	0.00	0.00	0.00	0.00	0.00	0.00	0.00	0.00	0.00	0.00
Luxembourg - Pseudomonas aeruginosa MDRPA SSI - model 49	0.00	0.00	0.00	0.00	0.00	0.00	0.00	0.00	0.00	NaN	NaN	NaN	0.00	0.00	0.00	0.00	0.00	0.00	0.00	0.00	0.00	0.00	0.00	0.00	0.00	0.00	0.00	0.00	0.00	0.00	0.00	0.00	0.00	0.00	0.00	0.00
Luxembourg - Pseudomonas aeruginosa MDRPA UTI - model 48	0.00	0.00	0.00	0.00	0.00	0.00	0.00	0.00	0.00	NaN	NaN	NaN	0.00	0.00	0.00	0.00	0.00	0.00	0.00	0.00	0.00	0.00	0.00	0.00	0.00	0.00	0.00	0.00	0.00	0.00	0.00	0.00	0.00	0.00	0.00	0.00
Luxembourg - Staphylococcus aureus MRSA BSI - model 61	6.95	7.90	9.12	48.69	53.44	58.70	55.96	61.37	66.94	4.65	4.98	5.37	1.23	1.40	1.62	8.65	9.49	10.43	9.94	10.90	11.89	11.57	12.28	13.07	2.05	2.18	2.32	2.05	2.20	2.38	0.36	0.39	0.42
Luxembourg - Staphylococcus aureus MRSA OTH - model 65	0.04	0.06	0.08	0.00	0.00	0.00	0.04	0.06	0.08	2.59E-03	3.26E-03	4.10E-03	7.97E-03	0.01	0.01	0.00	0.00	0.00	7.97E-03	0.01	0.01	16.11	18.12	20.07	2.86	3.22	3.57	0.00	0.00	0.00	0.00	0.00	0.00
Luxembourg - Staphylococcus aureus MRSA RESP - model 62	6.91	8.26	9.65	13.60	16.14	18.71	20.48	24.34	27.97	1.18	1.32	1.48	1.23	1.47	1.71	2.42	2.87	3.32	3.64	4.32	4.97	16.29	18.44	20.58	2.89	3.28	3.66	0.58	0.67	0.75	0.10	0.12	0.13
Luxembourg - Staphylococcus aureus MRSA SSI - model 64	0.03	0.04	0.06	7.67	8.73	9.73	7.70	8.77	9.79	0.42	0.44	0.45	5.36E-03	7.63E-03	0.01	1.36	1.55	1.73	1.37	1.56	1.74	18.01	20.11	22.39	3.20	3.57	3.98	0.47	0.54	0.61	0.08	0.10	0.11
Luxembourg - Staphylococcus aureus MRSA UTI - model 63	3.90E-03	5.64E-03	7.77E-03	0.00	0.00	0.00	3.90E-03	5.64E-03	7.77E-03	1.42E-03	1.87E-03	2.34E-03	6.93E-04	1.00E-03	1.38E-03	0.00	0.00	0.00	6.93E-04	1.00E-03	1.38E-03	2.41	3.01	3.61	0.43	0.54	0.64	0.00	0.00	0.00	0.00	0.00	0.00
Luxembourg - Streptococcus pneumoniae PMRSP BSI - model 76	0.00	0.00	0.00	0.00	0.00	0.00	0.00	0.00	0.00	NaN	NaN	NaN	0.00	0.00	0.00	0.00	0.00	0.00	0.00	0.00	0.00	0.00	0.00	0.00	0.00	0.00	0.00	0.00	0.00	0.00	0.00	0.00	0.00	0.00	0.00	0.00
Luxembourg - Streptococcus pneumoniae PMRSP OTH - model 80	0.00	0.00	0.00	0.00	0.00	0.00	0.00	0.00	0.00	NaN	NaN	NaN	0.00	0.00	0.00	0.00	0.00	0.00	0.00	0.00	0.00	0.00	0.00	0.00	0.00	0.00	0.00	0.00	0.00	0.00	0.00	0.00	0.00	0.00	0.00	0.00
Luxembourg - Streptococcus pneumoniae PMRSP RESP - model 77	0.00	0.00	0.00	0.00	0.00	0.00	0.00	0.00	0.00	NaN	NaN	NaN	0.00	0.00	0.00	0.00	0.00	0.00	0.00	0.00	0.00	0.00	0.00	0.00	0.00	0.00	0.00	0.00	0.00	0.00	0.00	0.00	0.00	0.00	0.00	0.00
Luxembourg - Streptococcus pneumoniae PMRSP SSI - model 79	0.00	0.00	0.00	0.00	0.00	0.00	0.00	0.00	0.00	NaN	NaN	NaN	0.00	0.00	0.00	0.00	0.00	0.00	0.00	0.00	0.00	0.00	0.00	0.00	0.00	0.00	0.00	0.00	0.00	0.00	0.00	0.00	0.00	0.00	0.00	0.00
Luxembourg - Streptococcus pneumoniae PMRSP UTI - model 78	0.00	0.00	0.00	0.00	0.00	0.00	0.00	0.00	0.00	NaN	NaN	NaN	0.00	0.00	0.00	0.00	0.00	0.00	0.00	0.00	0.00	0.00	0.00	0.00	0.00	0.00	0.00	0.00	0.00	0.00	0.00	0.00	0.00	0.00	0.00	0.00
Luxembourg - Streptococcus pneumoniae PRSP BSI - model 71	0.00	0.00	0.00	0.00	0.00	0.00	0.00	0.00	0.00	NaN	NaN	NaN	0.00	0.00	0.00	0.00	0.00	0.00	0.00	0.00	0.00	0.00	0.00	0.00	0.00	0.00	0.00	0.00	0.00	0.00	0.00	0.00	0.00	0.00	0.00	0.00
Luxembourg - Streptococcus pneumoniae PRSP OTH - model 75	0.00	0.00	0.00	0.00	0.00	0.00	0.00	0.00	0.00	NaN	NaN	NaN	0.00	0.00	0.00	0.00	0.00	0.00	0.00	0.00	0.00	0.00	0.00	0.00	0.00	0.00	0.00	0.00	0.00	0.00	0.00	0.00	0.00	0.00	0.00	0.00
Luxembourg - Streptococcus pneumoniae PRSP RESP - model 72	0.00	0.00	0.00	0.00	0.00	0.00	0.00	0.00	0.00	NaN	NaN	NaN	0.00	0.00	0.00	0.00	0.00	0.00	0.00	0.00	0.00	0.00	0.00	0.00	0.00	0.00	0.00	0.00	0.00	0.00	0.00	0.00	0.00	0.00	0.00	0.00
Luxembourg - Streptococcus pneumoniae PRSP SSI - model 74	0.00	0.00	0.00	0.00	0.00	0.00	0.00	0.00	0.00	NaN	NaN	NaN	0.00	0.00	0.00	0.00	0.00	0.00	0.00	0.00	0.00	0.00	0.00	0.00	0.00	0.00	0.00	0.00	0.00	0.00	0.00	0.00	0.00	0.00	0.00	0.00
Luxembourg - Streptococcus pneumoniae PRSP UTI - model 73	0.00	0.00	0.00	0.00	0.00	0.00	0.00	0.00	0.00	NaN	NaN	NaN	0.00	0.00	0.00	0.00	0.00	0.00	0.00	0.00	0.00	0.00	0.00	0.00	0.00	0.00	0.00	0.00	0.00	0.00	0.00	0.00	0.00	0.00	0.00	0.00
**Total (Median & 95% UIs)**	52.52	62.36	72.97	284.36	336.80	393.55	339.76	399.05	462.11		-		9.33	11.08	12.96	50.51	59.83	69.91	60.35	70.88	82.09	413.62	487.25	563.92	73.47	86.55	100.17	15.64	18.71	22.13	2.78	3.32	3.93
**Total (average)**	**62.49**			**337.01**			**399.50**			-			**11.10**			**59.86**			**70.97**			**487.79**			**86.65**			**18.75**			**3.33**		

Infection	YLD			YLL			DALY			DALY per Case			YLD per 100,000			YLL per 100,000			DALY per 100,000			Cases per year			Incidence per 100,000			Deaths per year			Mortality per 100,000		
Colistin resistant *Acinetobacter spp.*	0.00	0.00	0.00	0.00	0.00	0.00	0.00	0.00	0.00	0.00	0.00	0.00	0.00	0.00	0.00	0.00	0.00	0.00	0.00	0.00	0.00	0.00	0.00	0.00	0.00	0.00	0.00	0.00	0.00	0.00	0.00	0.00	0.00
Carbapenem resistant *Acinetobacter spp.* (excluding those resistant to colistin)	58.01	66.05	74.24	285.01	355.62	427.28	345.10	422.13	498.05	7.49	8.85	10.26	2.92	3.33	3.74	14.35	17.91	21.51	17.38	21.25	25.08	123.23	139.69	156.01	6.20	7.03	7.86	9.92	12.23	14.60	0.50	0.62	0.73
Aminoglycosides and fluoroquinolones resistant *Acinetobacter* spp. (excluding those resistant to colistin and/or carbapenem)	5.05	6.69	9.16	19.59	35.37	52.44	25.40	42.06	60.59	6.07	9.29	12.39	0.25	0.34	0.46	0.99	1.78	2.64	1.28	2.12	3.05	9.90	13.30	16.92	0.50	0.67	0.85	0.66	1.13	1.66	0.03	0.06	0.08
Vancomycin resistant Enterococci (*E. faecalis* and *E. faecium*)	6.24	8.89	12.58	58.92	82.29	110.99	65.00	91.33	123.53	7.86	8.81	10.48	0.31	0.45	0.63	2.97	4.14	5.59	3.27	4.60	6.22	27.15	39.82	52.98	1.37	2.00	2.67	1.87	2.54	3.33	0.09	0.13	0.17
Colistin resistant *Escherichia coli*	0.00	0.00	0.00	0.00	0.00	0.00	0.00	0.00	0.00	0.00	0.00	0.00	0.00	0.00	0.00	0.00	0.00	0.00	0.00	0.00	0.00	0.00	0.00	0.00	0.00	0.00	0.00	0.00	0.00	0.00	0.00	0.00	0.00
Carbapenem resistant *Escherichia coli* (excluding those resistant to colistin)	0.00	0.00	0.00	0.00	0.00	0.00	0.00	0.00	0.00	0.00	0.00	0.00	0.00	0.00	0.00	0.00	0.00	0.00	0.00	0.00	0.00	0.00	0.00	0.00	0.00	0.00	0.00	0.00	0.00	0.00	0.00	0.00	0.00
Third-generation cephalosporin resistant *Escherichia coli* (excluding those resistant to colistin and/or carbapenem)	28.28	32.61	37.44	165.87	192.33	220.28	196.34	225.24	255.89	5.45	6.02	6.62	1.42	1.64	1.89	8.35	9.68	11.09	9.89	11.34	12.88	262.12	299.25	337.90	13.20	15.07	17.01	7.75	9.00	10.21	0.39	0.45	0.51
Colistin resistant *Klebsiella pneumoniae*	0.00	0.00	0.00	0.00	0.00	0.00	0.00	0.00	0.00	0.00	0.00	0.00	0.00	0.00	0.00	0.00	0.00	0.00	0.00	0.00	0.00	0.00	0.00	0.00	0.00	0.00	0.00	0.00	0.00	0.00	0.00	0.00	0.00
Carbapenem resistant *Klebsiella pneumoniae* (excluding those resistant to colistin)	0.00	0.00	0.00	0.00	0.00	0.00	0.00	0.00	0.00	0.00	0.00	0.00	0.00	0.00	0.00	0.00	0.00	0.00	0.00	0.00	0.00	0.00	0.00	0.00	0.00	0.00	0.00	0.00	0.00	0.00	0.00	0.00	0.00
Third-generation cephalosporin resistant *Klebsiella pneumoniae* (excluding those resistant to colistin and/or carbapenem)	68.84	77.90	87.91	340.07	372.39	404.82	411.59	450.77	490.57	7.46	7.84	8.25	3.47	3.92	4.43	17.12	18.75	20.38	20.72	22.70	24.70	210.28	233.48	257.02	10.59	11.76	12.94	11.58	12.57	13.56	0.58	0.63	0.68
Colistin resistant *Pseudomonas aeruginosa*	0.00	0.00	0.00	0.00	0.00	0.00	0.00	0.00	0.00	0.00	0.00	0.00	0.00	0.00	0.00	0.00	0.00	0.00	0.00	0.00	0.00	0.00	0.00	0.00	0.00	0.00	0.00	0.00	0.00	0.00	0.00	0.00	0.00
Carbapenem resistant *Pseudomonas aeruginosa* (excluding those resistant to colistin)	9.49	14.74	20.33	35.23	74.70	126.63	48.03	89.11	145.52	13.06	23.18	33.64	0.48	0.74	1.02	1.77	3.76	6.38	2.42	4.49	7.33	10.67	17.06	23.30	0.54	0.86	1.17	0.53	1.13	1.92	0.03	0.06	0.10
Pseudomonas aeruginosa resistant to three or more antimicrobial groups (excluding those resistant to colistin and/or carbapenem)	0.00	0.00	0.00	0.00	0.00	0.00	0.00	0.00	0.00	0.00	0.00	0.00	0.00	0.00	0.00	0.00	0.00	0.00	0.00	0.00	0.00	0.00	0.00	0.00	0.00	0.00	0.00	0.00	0.00	0.00	0.00	0.00	0.00
Methicillin resistant *Staphylococcus aureus*	26.05	30.21	34.81	127.72	142.21	157.97	154.89	172.48	191.33	9.01	9.58	10.12	1.31	1.52	1.75	6.43	7.16	7.95	7.80	8.68	9.63	82.43	93.32	103.87	4.15	4.70	5.23	3.78	4.21	4.66	0.19	0.21	0.23
Penicillin and macrolides resistant *Streptococcus pneumoniae* (excluding those only resistant to penicillin)	0.00	0.00	0.00	0.00	0.00	0.00	0.00	0.00	0.00	0.00	0.00	0.00	0.00	0.00	0.00	0.00	0.00	0.00	0.00	0.00	0.00	0.00	0.00	0.00	0.00	0.00	0.00	0.00	0.00	0.00	0.00	0.00	0.00
Penicillin tesistant *Streptococcus pneumoniae* (excluding those resistant to macrolides)	4.17	5.42	7.61	14.86	17.83	22.18	19.30	23.22	29.42	6.01	6.69	7.33	0.21	0.27	0.38	0.75	0.90	1.12	0.97	1.17	1.48	9.35	11.31	15.04	0.47	0.57	0.76	0.58	0.70	0.87	0.03	0.04	0.04
Total	206.13	242.50	284.08	1,047.26	1,272.73	1,522.59	1,265.66	1,516.34	1,794.90				10.38	12.21	14.30	52.73	64.08	76.66	63.73	76.35	90.37	735.13	847.23	963.04	37.01	42.66	48.49	36.67	43.50	50.81	1.85	2.19	2.56

Model	YLD			YLL			DALY			DALY per Case			YLD per 100,000			YLL per 100,000			DALY per 100,000			Cases per year			Incidence per 100,000			Deaths per year			Mortality per 100,000		
Latvia - Acinetobacter spp. CRACI BSI - model 36	33.86	37.81	42.23	230.60	292.67	355.33	266.03	330.81	394.55	5.58	6.80	8.06	1.70	1.90	2.13	11.61	14.74	17.89	13.39	16.66	19.87	45.36	48.49	51.79	2.28	2.44	2.61	7.99	9.99	12.02	0.40	0.50	0.61
Latvia - Acinetobacter spp. CRACI OTH - model 40	4.45E-03	6.35E-03	8.83E-03	0.00	0.00	0.00	4.45E-03	6.35E-03	8.83E-03	2.97E-03	3.74E-03	4.53E-03	2.24E-04	3.20E-04	4.45E-04	0.00	0.00	0.00	2.24E-04	3.20E-04	4.45E-04	1.28	1.70	2.20	0.06	0.09	0.11	0.00	0.00	0.00	0.00	0.00	0.00
Latvia - Acinetobacter spp. CRACI RESP - model 37	24.09	28.16	31.90	47.77	54.88	62.33	72.37	83.15	93.77	1.49	1.59	1.70	1.21	1.42	1.61	2.41	2.76	3.14	3.64	4.19	4.72	45.88	52.18	58.06	2.31	2.63	2.92	1.63	1.87	2.13	0.08	0.09	0.11
Latvia - Acinetobacter spp. CRACI SSI - model 39	0.03	0.04	0.05	6.64	8.07	9.63	6.67	8.11	9.67	0.42	0.46	0.49	1.37E-03	1.88E-03	2.54E-03	0.33	0.41	0.48	0.34	0.41	0.49	14.68	17.62	20.71	0.74	0.89	1.04	0.30	0.37	0.45	0.02	0.02	0.02
Latvia - Acinetobacter spp. CRACI UTI - model 38	0.03	0.04	0.05	0.00	0.00	0.00	0.03	0.04	0.05	1.64E-03	2.05E-03	2.51E-03	1.50E-03	2.03E-03	2.65E-03	0.00	0.00	0.00	1.50E-03	2.03E-03	2.65E-03	16.03	19.69	23.26	0.81	0.99	1.17	0.00	0.00	0.00	0.00	0.00	0.00
Latvia - Acinetobacter spp. ColRACI BSI - model 41	0.00	0.00	0.00	0.00	0.00	0.00	0.00	0.00	0.00	NaN	NaN	NaN	0.00	0.00	0.00	0.00	0.00	0.00	0.00	0.00	0.00	0.00	0.00	0.00	0.00	0.00	0.00	0.00	0.00	0.00	0.00	0.00	0.00
Latvia - Acinetobacter spp. ColRACI OTH - model 45	0.00	0.00	0.00	0.00	0.00	0.00	0.00	0.00	0.00	NaN	NaN	NaN	0.00	0.00	0.00	0.00	0.00	0.00	0.00	0.00	0.00	0.00	0.00	0.00	0.00	0.00	0.00	0.00	0.00	0.00	0.00	0.00	0.00
Latvia - Acinetobacter spp. ColRACI RESP - model 42	0.00	0.00	0.00	0.00	0.00	0.00	0.00	0.00	0.00	NaN	NaN	NaN	0.00	0.00	0.00	0.00	0.00	0.00	0.00	0.00	0.00	0.00	0.00	0.00	0.00	0.00	0.00	0.00	0.00	0.00	0.00	0.00	0.00
Latvia - Acinetobacter spp. ColRACI SSI - model 44	0.00	0.00	0.00	0.00	0.00	0.00	0.00	0.00	0.00	NaN	NaN	NaN	0.00	0.00	0.00	0.00	0.00	0.00	0.00	0.00	0.00	0.00	0.00	0.00	0.00	0.00	0.00	0.00	0.00	0.00	0.00	0.00	0.00
Latvia - Acinetobacter spp. ColRACI UTI - model 43	0.00	0.00	0.00	0.00	0.00	0.00	0.00	0.00	0.00	NaN	NaN	NaN	0.00	0.00	0.00	0.00	0.00	0.00	0.00	0.00	0.00	0.00	0.00	0.00	0.00	0.00	0.00	0.00	0.00	0.00	0.00	0.00	0.00
Latvia - Acinetobacter spp. MDRACI BSI - model 31	3.01	3.84	5.05	15.29	29.12	44.03	18.92	32.92	48.50	4.19	7.18	10.03	0.15	0.19	0.25	0.77	1.47	2.22	0.95	1.66	2.44	3.98	4.62	5.41	0.20	0.23	0.27	0.51	0.93	1.40	0.03	0.05	0.07
Latvia - Acinetobacter spp. MDRACI OTH - model 35	2.25E-04	5.76E-04	1.19E-03	0.00	0.00	0.00	2.25E-04	5.76E-04	1.19E-03	2.12E-03	3.70E-03	5.53E-03	1.13E-05	2.90E-05	5.99E-05	0.00	0.00	0.00	1.13E-05	2.90E-05	5.99E-05	0.07	0.16	0.27	3.56E-03	8.06E-03	0.01	0.00	0.00	0.00	0.00	0.00	0.00
Latvia - Acinetobacter spp. MDRACI RESP - model 32	2.04	2.84	4.09	3.88	5.58	7.50	6.05	8.47	11.18	1.52	1.70	1.90	0.10	0.14	0.21	0.20	0.28	0.38	0.30	0.43	0.56	3.66	4.98	6.36	0.18	0.25	0.32	0.13	0.18	0.23	6.51E-03	8.97E-03	0.01
Latvia - Acinetobacter spp. MDRACI SSI - model 34	1.49E-03	3.42E-03	6.85E-03	0.42	0.67	0.90	0.42	0.67	0.91	0.36	0.40	0.45	7.53E-05	1.72E-04	3.45E-04	0.02	0.03	0.05	0.02	0.03	0.05	1.04	1.68	2.32	0.05	0.08	0.12	0.02	0.03	0.04	7.81E-04	1.33E-03	1.85E-03
Latvia - Acinetobacter spp. MDRACI UTI - model 33	1.84E-03	3.72E-03	6.37E-03	0.00	0.00	0.00	1.84E-03	3.72E-03	6.37E-03	1.14E-03	2.04E-03	3.03E-03	9.26E-05	1.87E-04	3.20E-04	0.00	0.00	0.00	9.26E-05	1.87E-04	3.20E-04	1.14	1.86	2.56	0.06	0.09	0.13	0.00	0.00	0.00	0.00	0.00	0.00
Latvia - Enterococcus faecalis and E. faecium VRE BSI - model 66	6.20	8.83	12.49	54.87	75.89	102.44	60.91	84.88	114.92	7.48	8.34	9.92	0.31	0.44	0.63	2.76	3.82	5.16	3.07	4.27	5.79	7.48	10.11	13.22	0.38	0.51	0.67	1.72	2.31	3.01	0.09	0.12	0.15
Latvia - Enterococcus faecalis and E. faecium VRE OTH - model 70	0.01	0.02	0.03	0.00	0.00	0.00	0.01	0.02	0.03	1.08E-03	1.66E-03	2.36E-03	5.25E-04	8.87E-04	1.43E-03	0.00	0.00	0.00	5.25E-04	8.87E-04	1.43E-03	7.27	10.79	14.17	0.37	0.54	0.71	0.00	0.00	0.00	0.00	0.00	0.00
Latvia - Enterococcus faecalis and E. faecium VRE RESP - model 67	0.00	0.00	0.00	0.00	0.00	0.00	0.00	0.00	0.00	NaN	NaN	NaN	0.00	0.00	0.00	0.00	0.00	0.00	0.00	0.00	0.00	0.00	0.00	0.00	0.00	0.00	0.00	0.00	0.00	0.00	0.00	0.00	0.00
Latvia - Enterococcus faecalis and E. faecium VRE SSI - model 69	0.01	0.03	0.05	4.05	6.39	8.54	4.07	6.42	8.57	0.38	0.46	0.55	7.45E-04	1.47E-03	2.55E-03	0.20	0.32	0.43	0.20	0.32	0.43	9.00	13.85	18.62	0.45	0.70	0.94	0.14	0.23	0.31	7.21E-03	0.01	0.02
Latvia - Enterococcus faecalis and E. faecium VRE UTI - model 68	5.65E-03	9.42E-03	0.02	0.00	0.00	0.00	5.65E-03	9.42E-03	0.02	1.27E-03	1.88E-03	2.55E-03	2.84E-04	4.74E-04	7.64E-04	0.00	0.00	0.00	2.84E-04	4.74E-04	7.64E-04	3.40	5.08	6.96	0.17	0.26	0.35	0.00	0.00	0.00	0.00	0.00	0.00
Latvia - Escherichia coli 3GCREC BSI - model 16	21.85	24.55	27.61	138.48	159.17	181.82	162.51	183.98	207.65	3.96	4.40	4.84	1.10	1.24	1.39	6.97	8.01	9.15	8.18	9.26	10.46	38.55	41.83	45.34	1.94	2.11	2.28	6.32	7.24	8.15	0.32	0.36	0.41
Latvia - Escherichia coli 3GCREC OTH - model 20	0.03	0.05	0.06	0.00	0.00	0.00	0.03	0.05	0.06	2.62E-03	3.33E-03	4.14E-03	1.63E-03	2.33E-03	3.09E-03	0.00	0.00	0.00	1.63E-03	2.33E-03	3.09E-03	11.02	13.93	16.77	0.55	0.70	0.84	0.00	0.00	0.00	0.00	0.00	0.00
Latvia - Escherichia coli 3GCREC RESP - model 17	6.06	7.57	9.20	11.71	14.72	17.66	17.77	22.33	26.78	1.09	1.20	1.32	0.31	0.38	0.46	0.59	0.74	0.89	0.89	1.12	1.35	15.15	18.62	22.08	0.76	0.94	1.11	0.54	0.67	0.80	0.03	0.03	0.04
Latvia - Escherichia coli 3GCREC SSI - model 19	0.07	0.09	0.13	15.68	18.43	20.79	15.76	18.53	20.95	0.39	0.42	0.45	3.38E-03	4.70E-03	6.43E-03	0.79	0.93	1.05	0.79	0.93	1.05	37.87	44.19	50.05	1.91	2.22	2.52	0.90	1.09	1.26	0.05	0.06	0.06
Latvia - Escherichia coli 3GCREC UTI - model 18	0.27	0.35	0.44	0.00	0.00	0.00	0.27	0.35	0.44	1.57E-03	1.96E-03	2.40E-03	0.01	0.02	0.02	0.00	0.00	0.00	0.01	0.02	0.02	159.53	180.69	203.66	8.03	9.10	10.25	0.00	0.00	0.00	0.00	0.00	0.00
Latvia - Escherichia coli CREC BSI - model 21	0.00	0.00	0.00	0.00	0.00	0.00	0.00	0.00	0.00	NaN	NaN	NaN	0.00	0.00	0.00	0.00	0.00	0.00	0.00	0.00	0.00	0.00	0.00	0.00	0.00	0.00	0.00	0.00	0.00	0.00	0.00	0.00	0.00
Latvia - Escherichia coli CREC OTH - model 25	0.00	0.00	0.00	0.00	0.00	0.00	0.00	0.00	0.00	NaN	NaN	NaN	0.00	0.00	0.00	0.00	0.00	0.00	0.00	0.00	0.00	0.00	0.00	0.00	0.00	0.00	0.00	0.00	0.00	0.00	0.00	0.00	0.00
Latvia - Escherichia coli CREC RESP - model 22	0.00	0.00	0.00	0.00	0.00	0.00	0.00	0.00	0.00	NaN	NaN	NaN	0.00	0.00	0.00	0.00	0.00	0.00	0.00	0.00	0.00	0.00	0.00	0.00	0.00	0.00	0.00	0.00	0.00	0.00	0.00	0.00	0.00
Latvia - Escherichia coli CREC SSI - model 24	0.00	0.00	0.00	0.00	0.00	0.00	0.00	0.00	0.00	NaN	NaN	NaN	0.00	0.00	0.00	0.00	0.00	0.00	0.00	0.00	0.00	0.00	0.00	0.00	0.00	0.00	0.00	0.00	0.00	0.00	0.00	0.00	0.00
Latvia - Escherichia coli CREC UTI - model 23	0.00	0.00	0.00	0.00	0.00	0.00	0.00	0.00	0.00	NaN	NaN	NaN	0.00	0.00	0.00	0.00	0.00	0.00	0.00	0.00	0.00	0.00	0.00	0.00	0.00	0.00	0.00	0.00	0.00	0.00	0.00	0.00	0.00
Latvia - Escherichia coli ColREC BSI - model 26	0.00	0.00	0.00	0.00	0.00	0.00	0.00	0.00	0.00	NaN	NaN	NaN	0.00	0.00	0.00	0.00	0.00	0.00	0.00	0.00	0.00	0.00	0.00	0.00	0.00	0.00	0.00	0.00	0.00	0.00	0.00	0.00	0.00
Latvia - Escherichia coli ColREC OTH - model 30	0.00	0.00	0.00	0.00	0.00	0.00	0.00	0.00	0.00	NaN	NaN	NaN	0.00	0.00	0.00	0.00	0.00	0.00	0.00	0.00	0.00	0.00	0.00	0.00	0.00	0.00	0.00	0.00	0.00	0.00	0.00	0.00	0.00
Latvia - Escherichia coli ColREC RESP - model 27	0.00	0.00	0.00	0.00	0.00	0.00	0.00	0.00	0.00	NaN	NaN	NaN	0.00	0.00	0.00	0.00	0.00	0.00	0.00	0.00	0.00	0.00	0.00	0.00	0.00	0.00	0.00	0.00	0.00	0.00	0.00	0.00	0.00
Latvia - Escherichia coli ColREC SSI - model 29	0.00	0.00	0.00	0.00	0.00	0.00	0.00	0.00	0.00	NaN	NaN	NaN	0.00	0.00	0.00	0.00	0.00	0.00	0.00	0.00	0.00	0.00	0.00	0.00	0.00	0.00	0.00	0.00	0.00	0.00	0.00	0.00	0.00
Latvia - Escherichia coli ColREC UTI - model 28	0.00	0.00	0.00	0.00	0.00	0.00	0.00	0.00	0.00	NaN	NaN	NaN	0.00	0.00	0.00	0.00	0.00	0.00	0.00	0.00	0.00	0.00	0.00	0.00	0.00	0.00	0.00	0.00	0.00	0.00	0.00	0.00	0.00
Latvia - Klebsiella pneumoniae 3GCRKP BSI - model 1	43.67	48.57	53.78	283.23	305.86	328.45	329.13	354.76	380.53	5.54	5.79	6.07	2.20	2.45	2.71	14.26	15.40	16.54	16.57	17.86	19.16	57.96	61.10	64.38	2.92	3.08	3.24	9.54	10.22	10.91	0.48	0.51	0.55
Latvia - Klebsiella pneumoniae 3GCRKP OTH - model 5	0.06	0.08	0.10	0.00	0.00	0.00	0.06	0.08	0.10	2.71E-03	3.36E-03	4.06E-03	3.21E-03	4.10E-03	5.16E-03	0.00	0.00	0.00	3.21E-03	4.10E-03	5.16E-03	21.11	24.26	27.28	1.06	1.22	1.37	0.00	0.00	0.00	0.00	0.00	0.00
Latvia - Klebsiella pneumoniae 3GCRKP RESP - model 2	24.95	29.06	33.78	48.30	56.61	65.03	73.71	85.81	98.37	1.53	1.63	1.73	1.26	1.46	1.70	2.43	2.85	3.27	3.71	4.32	4.95	46.45	52.68	58.77	2.34	2.65	2.96	1.65	1.89	2.14	0.08	0.10	0.11
Latvia - Klebsiella pneumoniae 3GCRKP SSI - model 4	0.04	0.05	0.07	8.54	9.93	11.33	8.59	9.98	11.38	0.40	0.41	0.44	1.86E-03	2.57E-03	3.49E-03	0.43	0.50	0.57	0.43	0.50	0.57	20.71	24.07	27.13	1.04	1.21	1.37	0.39	0.46	0.52	0.02	0.02	0.03
Latvia - Klebsiella pneumoniae 3GCRKP UTI - model 3	0.11	0.14	0.17	0.00	0.00	0.00	0.11	0.14	0.17	1.55E-03	1.95E-03	2.38E-03	5.49E-03	6.99E-03	8.79E-03	0.00	0.00	0.00	5.49E-03	6.99E-03	8.79E-03	64.04	71.37	79.47	3.22	3.59	4.00	0.00	0.00	0.00	0.00	0.00	0.00
Latvia - Klebsiella pneumoniae CRKP BSI - model 6	0.00	0.00	0.00	0.00	0.00	0.00	0.00	0.00	0.00	NaN	NaN	NaN	0.00	0.00	0.00	0.00	0.00	0.00	0.00	0.00	0.00	0.00	0.00	0.00	0.00	0.00	0.00	0.00	0.00	0.00	0.00	0.00	0.00
Latvia - Klebsiella pneumoniae CRKP OTH - model 10	0.00	0.00	0.00	0.00	0.00	0.00	0.00	0.00	0.00	NaN	NaN	NaN	0.00	0.00	0.00	0.00	0.00	0.00	0.00	0.00	0.00	0.00	0.00	0.00	0.00	0.00	0.00	0.00	0.00	0.00	0.00	0.00	0.00
Latvia - Klebsiella pneumoniae CRKP RESP - model 7	0.00	0.00	0.00	0.00	0.00	0.00	0.00	0.00	0.00	NaN	NaN	NaN	0.00	0.00	0.00	0.00	0.00	0.00	0.00	0.00	0.00	0.00	0.00	0.00	0.00	0.00	0.00	0.00	0.00	0.00	0.00	0.00	0.00
Latvia - Klebsiella pneumoniae CRKP SSI - model 9	0.00	0.00	0.00	0.00	0.00	0.00	0.00	0.00	0.00	NaN	NaN	NaN	0.00	0.00	0.00	0.00	0.00	0.00	0.00	0.00	0.00	0.00	0.00	0.00	0.00	0.00	0.00	0.00	0.00	0.00	0.00	0.00	0.00
Latvia - Klebsiella pneumoniae CRKP UTI - model 8	0.00	0.00	0.00	0.00	0.00	0.00	0.00	0.00	0.00	NaN	NaN	NaN	0.00	0.00	0.00	0.00	0.00	0.00	0.00	0.00	0.00	0.00	0.00	0.00	0.00	0.00	0.00	0.00	0.00	0.00	0.00	0.00	0.00
Latvia - Klebsiella pneumoniae ColRKP BSI - model 11	0.00	0.00	0.00	0.00	0.00	0.00	0.00	0.00	0.00	NaN	NaN	NaN	0.00	0.00	0.00	0.00	0.00	0.00	0.00	0.00	0.00	0.00	0.00	0.00	0.00	0.00	0.00	0.00	0.00	0.00	0.00	0.00	0.00
Latvia - Klebsiella pneumoniae ColRKP OTH - model 15	0.00	0.00	0.00	0.00	0.00	0.00	0.00	0.00	0.00	NaN	NaN	NaN	0.00	0.00	0.00	0.00	0.00	0.00	0.00	0.00	0.00	0.00	0.00	0.00	0.00	0.00	0.00	0.00	0.00	0.00	0.00	0.00	0.00
Latvia - Klebsiella pneumoniae ColRKP RESP - model 12	0.00	0.00	0.00	0.00	0.00	0.00	0.00	0.00	0.00	NaN	NaN	NaN	0.00	0.00	0.00	0.00	0.00	0.00	0.00	0.00	0.00	0.00	0.00	0.00	0.00	0.00	0.00	0.00	0.00	0.00	0.00	0.00	0.00
Latvia - Klebsiella pneumoniae ColRKP SSI - model 14	0.00	0.00	0.00	0.00	0.00	0.00	0.00	0.00	0.00	NaN	NaN	NaN	0.00	0.00	0.00	0.00	0.00	0.00	0.00	0.00	0.00	0.00	0.00	0.00	0.00	0.00	0.00	0.00	0.00	0.00	0.00	0.00	0.00
Latvia - Klebsiella pneumoniae ColRKP UTI - model 13	0.00	0.00	0.00	0.00	0.00	0.00	0.00	0.00	0.00	NaN	NaN	NaN	0.00	0.00	0.00	0.00	0.00	0.00	0.00	0.00	0.00	0.00	0.00	0.00	0.00	0.00	0.00	0.00	0.00	0.00	0.00	0.00	0.00
Latvia - Pseudomonas aeruginosa CRPA BSI - model 51	2.98	4.09	5.50	16.64	36.30	60.77	21.31	40.15	65.69	8.88	16.98	25.36	0.15	0.21	0.28	0.84	1.83	3.06	1.07	2.02	3.31	1.88	2.38	2.99	0.09	0.12	0.15	0.26	0.55	0.92	0.01	0.03	0.05
Latvia - Pseudomonas aeruginosa CRPA OTH - model 55	1.77E-03	4.31E-03	8.35E-03	0.00	0.00	0.00	1.77E-03	4.31E-03	8.35E-03	1.98E-03	3.72E-03	5.78E-03	8.90E-05	2.17E-04	4.20E-04	0.00	0.00	0.00	8.90E-05	2.17E-04	4.20E-04	0.56	1.21	1.79	0.03	0.06	0.09	0.00	0.00	0.00	0.00	0.00	0.00
Latvia - Pseudomonas aeruginosa CRPA RESP - model 52	6.51	10.64	14.80	18.08	37.44	64.44	26.20	47.99	78.38	3.61	5.59	7.63	0.33	0.54	0.75	0.91	1.89	3.24	1.32	2.42	3.95	5.67	8.81	11.79	0.29	0.44	0.59	0.27	0.57	0.98	0.01	0.03	0.05
Latvia - Pseudomonas aeruginosa CRPA SSI - model 54	1.08E-03	3.12E-03	7.37E-03	0.51	0.96	1.42	0.52	0.97	1.43	0.56	0.60	0.64	5.45E-05	1.57E-04	3.71E-04	0.03	0.05	0.07	0.03	0.05	0.07	0.83	1.61	2.36	0.04	0.08	0.12	7.49E-03	0.01	0.02	3.77E-04	7.30E-04	1.07E-03
Latvia - Pseudomonas aeruginosa CRPA UTI - model 53	2.54E-03	5.98E-03	0.01	0.00	0.00	0.00	2.54E-03	5.98E-03	0.01	1.00E-03	2.03E-03	3.32E-03	1.28E-04	3.01E-04	5.84E-04	0.00	0.00	0.00	1.28E-04	3.01E-04	5.84E-04	1.73	3.06	4.37	0.09	0.15	0.22	0.00	0.00	0.00	0.00	0.00	0.00
Latvia - Pseudomonas aeruginosa ColRPA BSI - model 56	0.00	0.00	0.00	0.00	0.00	0.00	0.00	0.00	0.00	NaN	NaN	NaN	0.00	0.00	0.00	0.00	0.00	0.00	0.00	0.00	0.00	0.00	0.00	0.00	0.00	0.00	0.00	0.00	0.00	0.00	0.00	0.00	0.00
Latvia - Pseudomonas aeruginosa ColRPA OTH - model 60	0.00	0.00	0.00	0.00	0.00	0.00	0.00	0.00	0.00	NaN	NaN	NaN	0.00	0.00	0.00	0.00	0.00	0.00	0.00	0.00	0.00	0.00	0.00	0.00	0.00	0.00	0.00	0.00	0.00	0.00	0.00	0.00	0.00
Latvia - Pseudomonas aeruginosa ColRPA RESP - model 57	0.00	0.00	0.00	0.00	0.00	0.00	0.00	0.00	0.00	NaN	NaN	NaN	0.00	0.00	0.00	0.00	0.00	0.00	0.00	0.00	0.00	0.00	0.00	0.00	0.00	0.00	0.00	0.00	0.00	0.00	0.00	0.00	0.00
Latvia - Pseudomonas aeruginosa ColRPA SSI - model 59	0.00	0.00	0.00	0.00	0.00	0.00	0.00	0.00	0.00	NaN	NaN	NaN	0.00	0.00	0.00	0.00	0.00	0.00	0.00	0.00	0.00	0.00	0.00	0.00	0.00	0.00	0.00	0.00	0.00	0.00	0.00	0.00	0.00
Latvia - Pseudomonas aeruginosa ColRPA UTI - model 58	0.00	0.00	0.00	0.00	0.00	0.00	0.00	0.00	0.00	NaN	NaN	NaN	0.00	0.00	0.00	0.00	0.00	0.00	0.00	0.00	0.00	0.00	0.00	0.00	0.00	0.00	0.00	0.00	0.00	0.00	0.00	0.00	0.00
Latvia - Pseudomonas aeruginosa MDRPA BSI - model 46	0.00	0.00	0.00	0.00	0.00	0.00	0.00	0.00	0.00	NaN	NaN	NaN	0.00	0.00	0.00	0.00	0.00	0.00	0.00	0.00	0.00	0.00	0.00	0.00	0.00	0.00	0.00	0.00	0.00	0.00	0.00	0.00	0.00
Latvia - Pseudomonas aeruginosa MDRPA OTH - model 50	0.00	0.00	0.00	0.00	0.00	0.00	0.00	0.00	0.00	NaN	NaN	NaN	0.00	0.00	0.00	0.00	0.00	0.00	0.00	0.00	0.00	0.00	0.00	0.00	0.00	0.00	0.00	0.00	0.00	0.00	0.00	0.00	0.00
Latvia - Pseudomonas aeruginosa MDRPA RESP - model 47	0.00	0.00	0.00	0.00	0.00	0.00	0.00	0.00	0.00	NaN	NaN	NaN	0.00	0.00	0.00	0.00	0.00	0.00	0.00	0.00	0.00	0.00	0.00	0.00	0.00	0.00	0.00	0.00	0.00	0.00	0.00	0.00	0.00
Latvia - Pseudomonas aeruginosa MDRPA SSI - model 49	0.00	0.00	0.00	0.00	0.00	0.00	0.00	0.00	0.00	NaN	NaN	NaN	0.00	0.00	0.00	0.00	0.00	0.00	0.00	0.00	0.00	0.00	0.00	0.00	0.00	0.00	0.00	0.00	0.00	0.00	0.00	0.00	0.00
Latvia - Pseudomonas aeruginosa MDRPA UTI - model 48	0.00	0.00	0.00	0.00	0.00	0.00	0.00	0.00	0.00	NaN	NaN	NaN	0.00	0.00	0.00	0.00	0.00	0.00	0.00	0.00	0.00	0.00	0.00	0.00	0.00	0.00	0.00	0.00	0.00	0.00	0.00	0.00	0.00
Latvia - Staphylococcus aureus MRSA BSI - model 61	12.98	14.76	16.87	91.11	99.80	109.44	104.71	114.65	125.32	6.79	7.20	7.58	0.65	0.74	0.85	4.59	5.02	5.51	5.27	5.77	6.31	14.86	15.91	16.97	0.75	0.80	0.85	2.62	2.86	3.13	0.13	0.14	0.16
Latvia - Staphylococcus aureus MRSA OTH - model 65	0.06	0.08	0.10	0.00	0.00	0.00	0.06	0.08	0.10	2.46E-03	3.27E-03	4.24E-03	2.78E-03	3.87E-03	5.07E-03	0.00	0.00	0.00	2.78E-03	3.87E-03	5.07E-03	20.68	23.46	26.27	1.04	1.18	1.32	0.00	0.00	0.00	0.00	0.00	0.00
Latvia - Staphylococcus aureus MRSA RESP - model 62	12.97	15.32	17.75	25.65	30.00	34.81	39.12	45.29	52.10	1.76	1.90	2.04	0.65	0.77	0.89	1.29	1.51	1.75	1.97	2.28	2.62	20.80	23.96	26.99	1.05	1.21	1.36	0.74	0.86	0.99	0.04	0.04	0.05
Latvia - Staphylococcus aureus MRSA SSI - model 64	0.04	0.05	0.08	10.96	12.41	13.73	11.00	12.45	13.79	0.46	0.48	0.50	1.88E-03	2.74E-03	4.02E-03	0.55	0.62	0.69	0.55	0.63	0.69	23.06	26.07	28.85	1.16	1.31	1.45	0.42	0.49	0.55	0.02	0.02	0.03
Latvia - Staphylococcus aureus MRSA UTI - model 63	4.95E-03	7.21E-03	0.01	0.00	0.00	0.00	4.95E-03	7.21E-03	0.01	1.35E-03	1.87E-03	2.41E-03	2.49E-04	3.63E-04	5.17E-04	0.00	0.00	0.00	2.49E-04	3.63E-04	5.17E-04	3.04	3.92	4.77	0.15	0.20	0.24	0.00	0.00	0.00	0.00	0.00	0.00
Latvia - Streptococcus pneumoniae PMRSP BSI - model 76	0.00	0.00	0.00	0.00	0.00	0.00	0.00	0.00	0.00	NaN	NaN	NaN	0.00	0.00	0.00	0.00	0.00	0.00	0.00	0.00	0.00	0.00	0.00	0.00	0.00	0.00	0.00	0.00	0.00	0.00	0.00	0.00	0.00
Latvia - Streptococcus pneumoniae PMRSP OTH - model 80	0.00	0.00	0.00	0.00	0.00	0.00	0.00	0.00	0.00	NaN	NaN	NaN	0.00	0.00	0.00	0.00	0.00	0.00	0.00	0.00	0.00	0.00	0.00	0.00	0.00	0.00	0.00	0.00	0.00	0.00	0.00	0.00	0.00
Latvia - Streptococcus pneumoniae PMRSP RESP - model 77	0.00	0.00	0.00	0.00	0.00	0.00	0.00	0.00	0.00	NaN	NaN	NaN	0.00	0.00	0.00	0.00	0.00	0.00	0.00	0.00	0.00	0.00	0.00	0.00	0.00	0.00	0.00	0.00	0.00	0.00	0.00	0.00	0.00
Latvia - Streptococcus pneumoniae PMRSP SSI - model 79	0.00	0.00	0.00	0.00	0.00	0.00	0.00	0.00	0.00	NaN	NaN	NaN	0.00	0.00	0.00	0.00	0.00	0.00	0.00	0.00	0.00	0.00	0.00	0.00	0.00	0.00	0.00	0.00	0.00	0.00	0.00	0.00	0.00
Latvia - Streptococcus pneumoniae PMRSP UTI - model 78	0.00	0.00	0.00	0.00	0.00	0.00	0.00	0.00	0.00	NaN	NaN	NaN	0.00	0.00	0.00	0.00	0.00	0.00	0.00	0.00	0.00	0.00	0.00	0.00	0.00	0.00	0.00	0.00	0.00	0.00	0.00	0.00	0.00
Latvia - Streptococcus pneumoniae PRSP BSI - model 71	1.20	1.50	1.86	9.19	10.25	11.25	10.62	11.76	12.83	4.78	5.30	5.78	0.06	0.08	0.09	0.46	0.52	0.57	0.53	0.59	0.65	2.22	2.22	2.22	0.11	0.11	0.11	0.36	0.40	0.44	0.02	0.02	0.02
Latvia - Streptococcus pneumoniae PRSP OTH - model 75	7.67E-04	1.53E-03	2.57E-03	0.00	0.00	0.00	7.67E-04	1.53E-03	2.57E-03	9.84E-04	1.93E-03	3.17E-03	3.86E-05	7.71E-05	1.30E-04	0.00	0.00	0.00	3.86E-05	7.71E-05	1.30E-04	0.67	0.79	0.95	0.03	0.04	0.05	0.00	0.00	0.00	0.00	0.00	0.00
Latvia - Streptococcus pneumoniae PRSP RESP - model 72	2.97	3.92	5.75	5.67	7.59	10.93	8.68	11.47	16.59	1.23	1.39	1.54	0.15	0.20	0.29	0.29	0.38	0.55	0.44	0.58	0.84	6.46	8.30	11.87	0.33	0.42	0.60	0.22	0.30	0.43	0.01	0.01	0.02
Latvia - Streptococcus pneumoniae PRSP SSI - model 74	0.00	0.00	0.00	0.00	0.00	0.00	0.00	0.00	0.00	NaN	NaN	NaN	0.00	0.00	0.00	0.00	0.00	0.00	0.00	0.00	0.00	0.00	0.00	0.00	0.00	0.00	0.00	0.00	0.00	0.00	0.00	0.00	0.00
Latvia - Streptococcus pneumoniae PRSP UTI - model 73	0.00	0.00	0.00	0.00	0.00	0.00	0.00	0.00	0.00		-		0.00	0.00	0.00	0.00	0.00	0.00	0.00	0.00	0.00	0.00	0.00	0.00	0.00	0.00	0.00	0.00	0.00	0.00	0.00	0.00	0.00
**Total (Median & 95% UIs)**	206.13	242.50	284.08	1,047.26	1,272.73	1,522.59	1,265.66	1,516.34	1,794.90				10.38	12.21	14.30	52.73	64.08	76.66	63.73	76.35	90.37	735.13	847.23	963.04	37.01	42.66	48.49	36.67	43.50	50.81	1.85	2.19	2.56
**Total (average)**		**243.29**			**1,276.47**			**1,519.76**			**-**			**12.25**			**64.27**			**76.52**			**847.97**			**42.70**			**43.59**			**2.19**	

Infection	YLD			YLL			DALY			DALY per Case			YLD per 100,000			YLL per 100,000			DALY per 100,000			Cases per year			Incidence per 100,000			Deaths per year			Mortality per 100,000		
Colistin resistant *Acinetobacter spp.*	0.52	0.84	1.26	1.84	4.58	7.96	2.55	5.44	9.09	3.19	5.39	7.68	0.12	0.19	0.29	0.43	1.07	1.85	0.59	1.27	2.12	1.84	3.11	4.46	0.43	0.73	1.04	0.11	0.27	0.48	0.03	0.06	0.11
Carbapenem resistant *Acinetobacter spp.* (excluding those resistant to colistin)	3.25	4.82	6.82	12.40	25.42	42.71	16.46	30.06	48.64	8.28	14.14	21.08	0.76	1.12	1.59	2.89	5.92	9.95	3.83	7.00	11.33	4.45	6.31	8.27	1.04	1.47	1.93	0.28	0.55	0.84	0.07	0.13	0.20
Aminoglycosides and fluoroquinolones resistant *Acinetobacter* spp. (excluding those resistant to colistin and/or carbapenem)	0.00	0.00	0.00	0.00	0.00	0.00	0.00	0.00	0.00	0.00	0.00	0.00	0.00	0.00	0.00	0.00	0.00	0.00	0.00	0.00	0.00	0.00	0.00	0.00	0.00	0.00	0.00	0.00	0.00	0.00	0.00	0.00	0.00
Vancomycin resistant Enterococci (*E. faecalis* and *E. faecium*)	0.00	0.00	0.00	0.00	0.00	0.00	0.00	0.00	0.00	0.00	0.00	0.00	0.00	0.00	0.00	0.00	0.00	0.00	0.00	0.00	0.00	0.00	0.00	0.00	0.00	0.00	0.00	0.00	0.00	0.00	0.00	0.00	0.00
Colistin resistant *Escherichia coli*	0.00	0.00	0.00	0.00	0.00	0.00	0.00	0.00	0.00	0.00	0.00	0.00	0.00	0.00	0.00	0.00	0.00	0.00	0.00	0.00	0.00	0.00	0.00	0.00	0.00	0.00	0.00	0.00	0.00	0.00	0.00	0.00	0.00
Carbapenem resistant *Escherichia coli* (excluding those resistant to colistin)	0.00	0.00	0.00	0.00	0.00	0.00	0.00	0.00	0.00	0.00	0.00	0.00	0.00	0.00	0.00	0.00	0.00	0.00	0.00	0.00	0.00	0.00	0.00	0.00	0.00	0.00	0.00	0.00	0.00	0.00	0.00	0.00	0.00
Third-generation cephalosporin resistant *Escherichia coli* (excluding those resistant to colistin and/or carbapenem)	16.19	18.69	21.33	96.74	110.43	124.80	113.56	129.03	145.33	4.00	4.50	5.05	3.77	4.35	4.97	22.53	25.72	29.07	26.45	30.05	33.85	195.78	228.91	261.33	45.60	53.32	60.87	5.98	7.09	8.22	1.39	1.65	1.91
Colistin resistant *Klebsiella pneumoniae*	0.91	1.34	1.89	14.39	23.73	34.30	15.45	25.03	35.88	8.15	12.35	16.20	0.21	0.31	0.44	3.35	5.53	7.99	3.60	5.83	8.36	3.72	6.22	8.71	0.87	1.45	2.03	0.85	1.40	2.04	0.20	0.33	0.47
Carbapenem resistant *Klebsiella pneumoniae* (excluding those resistant to colistin)	2.85	4.02	5.46	28.96	43.26	62.87	32.00	47.38	67.80	11.23	15.78	20.51	0.66	0.94	1.27	6.74	10.07	14.64	7.45	11.04	15.79	6.27	9.22	12.27	1.46	2.15	2.86	0.84	1.24	1.74	0.20	0.29	0.41
Third-generation cephalosporin resistant *Klebsiella pneumoniae* (excluding those resistant to colistin and/or carbapenem)	10.20	12.39	15.09	52.13	60.31	69.47	63.02	72.77	83.59	6.70	7.37	8.11	2.38	2.89	3.51	12.14	14.05	16.18	14.68	16.95	19.47	33.85	41.02	48.27	7.88	9.56	11.24	1.94	2.23	2.57	0.45	0.52	0.60
Colistin resistant *Pseudomonas aeruginosa*	0.00	0.00	0.00	0.00	0.00	0.00	0.00	0.00	0.00	0.00	0.00	0.00	0.00	0.00	0.00	0.00	0.00	0.00	0.00	0.00	0.00	0.00	0.00	0.00	0.00	0.00	0.00	0.00	0.00	0.00	0.00	0.00	0.00
Carbapenem resistant *Pseudomonas aeruginosa* (excluding those resistant to colistin)	8.39	11.05	14.28	33.34	56.32	80.57	43.40	67.23	93.77	6.29	9.26	12.12	1.95	2.57	3.33	7.77	13.12	18.77	10.11	15.66	21.84	23.92	32.47	41.10	5.57	7.56	9.57	1.32	2.20	3.15	0.31	0.51	0.73
Pseudomonas aeruginosa resistant to three or more antimicrobial groups (excluding those resistant to colistin and/or carbapenem)	0.32	0.58	0.91	0.91	2.58	5.17	1.31	3.17	5.95	0.97	1.85	2.67	0.07	0.14	0.21	0.21	0.60	1.20	0.31	0.74	1.39	4.04	8.09	12.23	0.94	1.88	2.85	0.18	0.52	1.03	0.04	0.12	0.24
Methicillin resistant *Staphylococcus aureus*	35.82	40.83	46.19	182.20	201.22	221.67	219.50	242.19	265.91	4.28	4.54	4.81	8.34	9.51	10.76	42.44	46.87	51.63	51.12	56.41	61.93	242.85	272.20	300.89	56.56	63.40	70.08	11.87	13.10	14.29	2.76	3.05	3.33
Penicillin and macrolides resistant *Streptococcus pneumoniae* (excluding those only resistant to penicillin)	0.00	0.00	0.00	0.00	0.00	0.00	0.00	0.00	0.00	0.00	0.00	0.00	0.00	0.00	0.00	0.00	0.00	0.00	0.00	0.00	0.00	0.00	0.00	0.00	0.00	0.00	0.00	0.00	0.00	0.00	0.00	0.00	0.00
Penicillin tesistant *Streptococcus pneumoniae* (excluding those resistant to macrolides)	0.00	0.00	0.00	0.00	0.00	0.00	0.00	0.00	0.00	0.00	0.00	0.00	0.00	0.00	0.00	0.00	0.00	0.00	0.00	0.00	0.00	0.00	0.00	0.00	0.00	0.00	0.00	0.00	0.00	0.00	0.00	0.00	0.00
Total	78.45	94.56	113.23	422.92	527.84	649.52	507.25	622.31	755.97				18.27	22.02	26.37	98.50	122.94	151.28	118.15	144.94	176.08	516.72	607.56	697.54	120.35	141.51	162.47	23.38	28.61	34.36	5.45	6.66	8.00

Model	YLD			YLL			DALY			DALY per Case			YLD per 100,000			YLL per 100,000			DALY per 100,000			Cases per year			Incidence per 100,000			Deaths per year			Mortality per 100,000		
Malta - Acinetobacter spp. CRACI BSI - model 36	1.94	2.73	3.80	9.65	20.79	36.30	12.30	23.30	39.36	5.63	10.88	17.16	0.45	0.64	0.88	2.25	4.84	8.45	2.86	5.43	9.17	1.84	2.18	2.58	0.43	0.51	0.60	0.22	0.45	0.70	0.05	0.10	0.16
Malta - Acinetobacter spp. CRACI OTH - model 40	8.50E-05	2.67E-04	6.02E-04	0.00	0.00	0.00	8.50E-05	2.67E-04	6.02E-04	1.93E-03	3.77E-03	5.91E-03	1.98E-05	6.22E-05	1.40E-04	0.00	0.00	0.00	1.98E-05	6.22E-05	1.40E-04	0.03	0.08	0.13	6.45E-03	0.02	0.03	0.00	0.00	0.00	0.00	0.00	0.00
Malta - Acinetobacter spp. CRACI RESP - model 37	1.30	2.09	3.01	2.48	4.11	5.64	3.88	6.24	8.51	2.01	2.60	3.22	0.30	0.49	0.70	0.58	0.96	1.31	0.90	1.45	1.98	1.68	2.38	3.08	0.39	0.55	0.72	0.06	0.08	0.11	0.01	0.02	0.03
Malta - Acinetobacter spp. CRACI SSI - model 39	5.91E-04	1.58E-03	3.43E-03	0.28	0.52	0.76	0.28	0.52	0.76	0.63	0.66	0.68	1.38E-04	3.67E-04	7.99E-04	0.06	0.12	0.18	0.06	0.12	0.18	0.42	0.79	1.15	0.10	0.18	0.27	8.53E-03	0.02	0.03	1.99E-03	4.11E-03	6.27E-03
Malta - Acinetobacter spp. CRACI UTI - model 38	7.19E-04	1.75E-03	3.36E-03	0.00	0.00	0.00	7.19E-04	1.75E-03	3.36E-03	1.05E-03	2.04E-03	3.14E-03	1.67E-04	4.08E-04	7.84E-04	0.00	0.00	0.00	1.67E-04	4.08E-04	7.84E-04	0.48	0.89	1.32	0.11	0.21	0.31	0.00	0.00	0.00	0.00	0.00	0.00
Malta - Acinetobacter spp. ColRACI BSI - model 41	0.32	0.47	0.68	1.36	3.64	6.52	1.85	4.14	7.12	1.81	3.86	6.01	0.08	0.11	0.16	0.32	0.85	1.52	0.43	0.96	1.66	0.86	1.07	1.36	0.20	0.25	0.32	0.08	0.22	0.39	0.02	0.05	0.09
Malta - Acinetobacter spp. ColRACI OTH - model 45	1.89E-05	1.23E-04	4.08E-04	0.00	0.00	0.00	1.89E-05	1.23E-04	4.08E-04	1.56E-03	3.65E-03	6.39E-03	4.41E-06	2.86E-05	9.49E-05	0.00	0.00	0.00	4.41E-06	2.86E-05	9.49E-05	6.41E-03	0.04	0.08	1.49E-03	8.61E-03	0.02	0.00	0.00	0.00	0.00	0.00	0.00
Malta - Acinetobacter spp. ColRACI RESP - model 42	0.20	0.36	0.57	0.39	0.70	1.06	0.60	1.07	1.59	0.78	0.91	1.05	0.05	0.08	0.13	0.09	0.16	0.25	0.14	0.25	0.37	0.66	1.18	1.66	0.15	0.27	0.39	0.02	0.04	0.06	5.38E-03	9.72E-03	0.01
Malta - Acinetobacter spp. ColRACI SSI - model 44	1.49E-04	7.27E-04	2.21E-03	0.10	0.24	0.38	0.10	0.24	0.38	0.60	0.61	0.61	3.47E-05	1.69E-04	5.15E-04	0.02	0.06	0.09	0.02	0.06	0.09	0.16	0.40	0.63	0.04	0.09	0.15	5.68E-03	0.01	0.02	1.32E-03	3.31E-03	5.28E-03
Malta - Acinetobacter spp. ColRACI UTI - model 43	2.33E-04	7.96E-04	2.02E-03	0.00	0.00	0.00	2.33E-04	7.96E-04	2.02E-03	7.70E-04	1.98E-03	3.64E-03	5.43E-05	1.85E-04	4.70E-04	0.00	0.00	0.00	5.43E-05	1.85E-04	4.70E-04	0.16	0.44	0.73	0.04	0.10	0.17	0.00	0.00	0.00	0.00	0.00	0.00
Malta - Acinetobacter spp. MDRACI BSI - model 31	0.00	0.00	0.00	0.00	0.00	0.00	0.00	0.00	0.00	NaN	NaN	NaN	0.00	0.00	0.00	0.00	0.00	0.00	0.00	0.00	0.00	0.00	0.00	0.00	0.00	0.00	0.00	0.00	0.00	0.00	0.00	0.00	0.00
Malta - Acinetobacter spp. MDRACI OTH - model 35	0.00	0.00	0.00	0.00	0.00	0.00	0.00	0.00	0.00	NaN	NaN	NaN	0.00	0.00	0.00	0.00	0.00	0.00	0.00	0.00	0.00	0.00	0.00	0.00	0.00	0.00	0.00	0.00	0.00	0.00	0.00	0.00	0.00
Malta - Acinetobacter spp. MDRACI RESP - model 32	0.00	0.00	0.00	0.00	0.00	0.00	0.00	0.00	0.00	NaN	NaN	NaN	0.00	0.00	0.00	0.00	0.00	0.00	0.00	0.00	0.00	0.00	0.00	0.00	0.00	0.00	0.00	0.00	0.00	0.00	0.00	0.00	0.00
Malta - Acinetobacter spp. MDRACI SSI - model 34	0.00	0.00	0.00	0.00	0.00	0.00	0.00	0.00	0.00	NaN	NaN	NaN	0.00	0.00	0.00	0.00	0.00	0.00	0.00	0.00	0.00	0.00	0.00	0.00	0.00	0.00	0.00	0.00	0.00	0.00	0.00	0.00	0.00
Malta - Acinetobacter spp. MDRACI UTI - model 33	0.00	0.00	0.00	0.00	0.00	0.00	0.00	0.00	0.00	NaN	NaN	NaN	0.00	0.00	0.00	0.00	0.00	0.00	0.00	0.00	0.00	0.00	0.00	0.00	0.00	0.00	0.00	0.00	0.00	0.00	0.00	0.00	0.00
Malta - Enterococcus faecalis and E. faecium VRE BSI - model 66	0.00	0.00	0.00	0.00	0.00	0.00	0.00	0.00	0.00	NaN	NaN	NaN	0.00	0.00	0.00	0.00	0.00	0.00	0.00	0.00	0.00	0.00	0.00	0.00	0.00	0.00	0.00	0.00	0.00	0.00	0.00	0.00	0.00
Malta - Enterococcus faecalis and E. faecium VRE OTH - model 70	0.00	0.00	0.00	0.00	0.00	0.00	0.00	0.00	0.00	NaN	NaN	NaN	0.00	0.00	0.00	0.00	0.00	0.00	0.00	0.00	0.00	0.00	0.00	0.00	0.00	0.00	0.00	0.00	0.00	0.00	0.00	0.00	0.00
Malta - Enterococcus faecalis and E. faecium VRE RESP - model 67	0.00	0.00	0.00	0.00	0.00	0.00	0.00	0.00	0.00	NaN	NaN	NaN	0.00	0.00	0.00	0.00	0.00	0.00	0.00	0.00	0.00	0.00	0.00	0.00	0.00	0.00	0.00	0.00	0.00	0.00	0.00	0.00	0.00
Malta - Enterococcus faecalis and E. faecium VRE SSI - model 69	0.00	0.00	0.00	0.00	0.00	0.00	0.00	0.00	0.00	NaN	NaN	NaN	0.00	0.00	0.00	0.00	0.00	0.00	0.00	0.00	0.00	0.00	0.00	0.00	0.00	0.00	0.00	0.00	0.00	0.00	0.00	0.00	0.00
Malta - Enterococcus faecalis and E. faecium VRE UTI - model 68	0.00	0.00	0.00	0.00	0.00	0.00	0.00	0.00	0.00	NaN	NaN	NaN	0.00	0.00	0.00	0.00	0.00	0.00	0.00	0.00	0.00	0.00	0.00	0.00	0.00	0.00	0.00	0.00	0.00	0.00	0.00	0.00	0.00
Malta - Escherichia coli 3GCREC BSI - model 16	12.48	14.03	15.68	79.85	90.15	101.16	92.88	104.08	116.16	2.93	3.25	3.62	2.91	3.27	3.65	18.60	21.00	23.56	21.63	24.24	27.06	29.33	32.02	34.88	6.83	7.46	8.12	4.76	5.55	6.37	1.11	1.29	1.48
Malta - Escherichia coli 3GCREC OTH - model 20	0.02	0.04	0.05	0.00	0.00	0.00	0.02	0.04	0.05	2.51E-03	3.34E-03	4.24E-03	5.63E-03	8.21E-03	0.01	0.00	0.00	0.00	5.63E-03	8.21E-03	0.01	8.11	10.57	12.92	1.89	2.46	3.01	0.00	0.00	0.00	0.00	0.00	0.00
Malta - Escherichia coli 3GCREC RESP - model 17	3.43	4.29	5.14	6.67	8.26	9.93	10.16	12.56	14.97	0.74	0.89	1.04	0.80	1.00	1.20	1.55	1.92	2.31	2.37	2.93	3.49	11.01	14.15	17.10	2.56	3.30	3.98	0.40	0.51	0.63	0.09	0.12	0.15
Malta - Escherichia coli 3GCREC SSI - model 19	0.05	0.07	0.10	10.22	12.02	13.71	10.29	12.09	13.79	0.33	0.36	0.39	0.01	0.02	0.02	2.38	2.80	3.19	2.40	2.82	3.21	27.92	33.80	39.43	6.50	7.87	9.18	0.82	1.03	1.22	0.19	0.24	0.28
Malta - Escherichia coli 3GCREC UTI - model 18	0.21	0.27	0.36	0.00	0.00	0.00	0.21	0.27	0.36	1.49E-03	1.96E-03	2.51E-03	0.05	0.06	0.08	0.00	0.00	0.00	0.05	0.06	0.08	119.41	138.38	156.99	27.81	32.23	36.56	0.00	0.00	0.00	0.00	0.00	0.00
Malta - Escherichia coli CREC BSI - model 21	0.00	0.00	0.00	0.00	0.00	0.00	0.00	0.00	0.00	NaN	NaN	NaN	0.00	0.00	0.00	0.00	0.00	0.00	0.00	0.00	0.00	0.00	0.00	0.00	0.00	0.00	0.00	0.00	0.00	0.00	0.00	0.00	0.00
Malta - Escherichia coli CREC OTH - model 25	0.00	0.00	0.00	0.00	0.00	0.00	0.00	0.00	0.00	NaN	NaN	NaN	0.00	0.00	0.00	0.00	0.00	0.00	0.00	0.00	0.00	0.00	0.00	0.00	0.00	0.00	0.00	0.00	0.00	0.00	0.00	0.00	0.00
Malta - Escherichia coli CREC RESP - model 22	0.00	0.00	0.00	0.00	0.00	0.00	0.00	0.00	0.00	NaN	NaN	NaN	0.00	0.00	0.00	0.00	0.00	0.00	0.00	0.00	0.00	0.00	0.00	0.00	0.00	0.00	0.00	0.00	0.00	0.00	0.00	0.00	0.00
Malta - Escherichia coli CREC SSI - model 24	0.00	0.00	0.00	0.00	0.00	0.00	0.00	0.00	0.00	NaN	NaN	NaN	0.00	0.00	0.00	0.00	0.00	0.00	0.00	0.00	0.00	0.00	0.00	0.00	0.00	0.00	0.00	0.00	0.00	0.00	0.00	0.00	0.00
Malta - Escherichia coli CREC UTI - model 23	0.00	0.00	0.00	0.00	0.00	0.00	0.00	0.00	0.00	NaN	NaN	NaN	0.00	0.00	0.00	0.00	0.00	0.00	0.00	0.00	0.00	0.00	0.00	0.00	0.00	0.00	0.00	0.00	0.00	0.00	0.00	0.00	0.00
Malta - Escherichia coli ColREC BSI - model 26	0.00	0.00	0.00	0.00	0.00	0.00	0.00	0.00	0.00	NaN	NaN	NaN	0.00	0.00	0.00	0.00	0.00	0.00	0.00	0.00	0.00	0.00	0.00	0.00	0.00	0.00	0.00	0.00	0.00	0.00	0.00	0.00	0.00
Malta - Escherichia coli ColREC OTH - model 30	0.00	0.00	0.00	0.00	0.00	0.00	0.00	0.00	0.00	NaN	NaN	NaN	0.00	0.00	0.00	0.00	0.00	0.00	0.00	0.00	0.00	0.00	0.00	0.00	0.00	0.00	0.00	0.00	0.00	0.00	0.00	0.00	0.00
Malta - Escherichia coli ColREC RESP - model 27	0.00	0.00	0.00	0.00	0.00	0.00	0.00	0.00	0.00	NaN	NaN	NaN	0.00	0.00	0.00	0.00	0.00	0.00	0.00	0.00	0.00	0.00	0.00	0.00	0.00	0.00	0.00	0.00	0.00	0.00	0.00	0.00	0.00
Malta - Escherichia coli ColREC SSI - model 29	0.00	0.00	0.00	0.00	0.00	0.00	0.00	0.00	0.00	NaN	NaN	NaN	0.00	0.00	0.00	0.00	0.00	0.00	0.00	0.00	0.00	0.00	0.00	0.00	0.00	0.00	0.00	0.00	0.00	0.00	0.00	0.00	0.00
Malta - Escherichia coli ColREC UTI - model 28	0.00	0.00	0.00	0.00	0.00	0.00	0.00	0.00	0.00	NaN	NaN	NaN	0.00	0.00	0.00	0.00	0.00	0.00	0.00	0.00	0.00	0.00	0.00	0.00	0.00	0.00	0.00	0.00	0.00	0.00	0.00	0.00	0.00
Malta - Klebsiella pneumoniae 3GCRKP BSI - model 1	6.50	7.74	9.26	43.33	48.75	55.12	50.50	56.58	63.62	4.86	5.29	5.77	1.51	1.80	2.16	10.09	11.35	12.84	11.76	13.18	14.82	9.79	10.71	11.79	2.28	2.50	2.75	1.59	1.79	2.01	0.37	0.42	0.47
Malta - Klebsiella pneumoniae 3GCRKP OTH - model 5	9.02E-03	0.01	0.02	0.00	0.00	0.00	9.02E-03	0.01	0.02	2.30E-03	3.31E-03	4.67E-03	2.10E-03	3.23E-03	4.90E-03	0.00	0.00	0.00	2.10E-03	3.23E-03	4.90E-03	3.26	4.23	5.32	0.76	0.99	1.24	0.00	0.00	0.00	0.00	0.00	0.00
Malta - Klebsiella pneumoniae 3GCRKP RESP - model 2	3.67	4.60	5.76	6.97	9.09	11.18	10.66	13.68	16.74	1.30	1.48	1.69	0.85	1.07	1.34	1.62	2.12	2.61	2.48	3.19	3.90	7.40	9.23	11.10	1.72	2.15	2.58	0.26	0.33	0.41	0.06	0.08	0.10
Malta - Klebsiella pneumoniae 3GCRKP SSI - model 4	5.01E-03	8.71E-03	0.02	1.83	2.47	3.16	1.83	2.47	3.18	0.54	0.59	0.64	1.17E-03	2.03E-03	3.50E-03	0.43	0.57	0.74	0.43	0.58	0.74	3.25	4.20	5.22	0.76	0.98	1.22	0.09	0.12	0.15	0.02	0.03	0.04
Malta - Klebsiella pneumoniae 3GCRKP UTI - model 3	0.02	0.02	0.04	0.00	0.00	0.00	0.02	0.02	0.04	1.34E-03	1.95E-03	2.68E-03	3.71E-03	5.66E-03	8.38E-03	0.00	0.00	0.00	3.71E-03	5.66E-03	8.38E-03	10.15	12.65	14.85	2.36	2.95	3.46	0.00	0.00	0.00	0.00	0.00	0.00
Malta - Klebsiella pneumoniae CRKP BSI - model 6	2.38	3.11	4.07	27.73	41.03	59.48	30.31	44.23	63.09	9.36	13.34	17.50	0.55	0.72	0.95	6.46	9.56	13.85	7.06	10.30	14.70	2.79	3.31	3.91	0.65	0.77	0.91	0.80	1.17	1.64	0.19	0.27	0.38
Malta - Klebsiella pneumoniae CRKP OTH - model 10	2.38E-03	5.12E-03	9.82E-03	0.00	0.00	0.00	2.38E-03	5.12E-03	9.82E-03	1.94E-03	3.41E-03	5.81E-03	5.54E-04	1.19E-03	2.29E-03	0.00	0.00	0.00	5.54E-04	1.19E-03	2.29E-03	0.90	1.54	2.18	0.21	0.36	0.51	0.00	0.00	0.00	0.00	0.00	0.00
Malta - Klebsiella pneumoniae CRKP RESP - model 7	0.47	0.90	1.37	0.97	1.74	2.66	1.43	2.65	3.96	1.47	1.92	2.39	0.11	0.21	0.32	0.23	0.41	0.62	0.33	0.62	0.92	0.78	1.38	2.01	0.18	0.32	0.47	0.03	0.05	0.07	6.54E-03	0.01	0.02
Malta - Klebsiella pneumoniae CRKP SSI - model 9	7.68E-04	1.98E-03	4.06E-03	0.26	0.49	0.73	0.26	0.49	0.73	0.40	0.51	0.61	1.79E-04	4.62E-04	9.45E-04	0.06	0.11	0.17	0.06	0.11	0.17	0.52	0.97	1.40	0.12	0.22	0.33	8.09E-03	0.02	0.03	1.88E-03	4.00E-03	6.22E-03
Malta - Klebsiella pneumoniae CRKP UTI - model 8	1.93E-03	4.07E-03	7.12E-03	0.00	0.00	0.00	1.93E-03	4.07E-03	7.12E-03	1.12E-03	2.03E-03	3.27E-03	4.48E-04	9.47E-04	1.66E-03	0.00	0.00	0.00	4.48E-04	9.47E-04	1.66E-03	1.28	2.01	2.78	0.30	0.47	0.65	0.00	0.00	0.00	0.00	0.00	0.00
Malta - Klebsiella pneumoniae ColRKP BSI - model 11	0.77	1.03	1.40	13.94	22.74	32.79	14.85	23.75	33.90	6.86	10.80	14.38	0.18	0.24	0.33	3.25	5.30	7.64	3.46	5.53	7.89	1.78	2.23	2.74	0.41	0.52	0.64	0.82	1.34	1.95	0.19	0.31	0.45
Malta - Klebsiella pneumoniae ColRKP OTH - model 15	1.14E-03	3.28E-03	7.34E-03	0.00	0.00	0.00	1.14E-03	3.28E-03	7.34E-03	1.59E-03	3.44E-03	6.37E-03	2.66E-04	7.65E-04	1.71E-03	0.00	0.00	0.00	2.66E-04	7.65E-04	1.71E-03	0.47	1.02	1.53	0.11	0.24	0.36	0.00	0.00	0.00	0.00	0.00	0.00
Malta - Klebsiella pneumoniae ColRKP RESP - model 12	0.14	0.30	0.47	0.26	0.58	0.89	0.41	0.88	1.35	0.77	0.93	1.11	0.03	0.07	0.11	0.06	0.14	0.21	0.10	0.21	0.31	0.45	0.95	1.43	0.10	0.22	0.33	0.02	0.03	0.05	3.66E-03	7.98E-03	0.01
Malta - Klebsiella pneumoniae ColRKP SSI - model 14	4.46E-04	1.26E-03	2.95E-03	0.19	0.40	0.62	0.19	0.40	0.62	0.52	0.61	0.70	1.04E-04	2.94E-04	6.87E-04	0.04	0.09	0.14	0.04	0.09	0.15	0.32	0.65	1.01	0.07	0.15	0.24	0.01	0.02	0.04	2.68E-03	5.44E-03	8.47E-03
Malta - Klebsiella pneumoniae ColRKP UTI - model 13	1.11E-03	2.72E-03	5.55E-03	0.00	0.00	0.00	1.11E-03	2.72E-03	5.55E-03	9.91E-04	2.06E-03	3.60E-03	2.58E-04	6.33E-04	1.29E-03	0.00	0.00	0.00	2.58E-04	6.33E-04	1.29E-03	0.70	1.36	2.01	0.16	0.32	0.47	0.00	0.00	0.00	0.00	0.00	0.00
Malta - Pseudomonas aeruginosa CRPA BSI - model 51	2.45	3.09	3.89	16.18	26.67	38.33	19.26	29.65	41.84	4.34	6.58	8.80	0.57	0.72	0.91	3.77	6.21	8.93	4.49	6.91	9.75	3.77	4.52	5.32	0.88	1.05	1.24	0.63	1.04	1.47	0.15	0.24	0.34
Malta - Pseudomonas aeruginosa CRPA OTH - model 55	4.50E-03	8.31E-03	0.01	0.00	0.00	0.00	4.50E-03	8.31E-03	0.01	2.36E-03	3.62E-03	5.13E-03	1.05E-03	1.94E-03	3.09E-03	0.00	0.00	0.00	1.05E-03	1.94E-03	3.09E-03	1.48	2.30	3.08	0.35	0.54	0.72	0.00	0.00	0.00	0.00	0.00	0.00
Malta - Pseudomonas aeruginosa CRPA RESP - model 52	5.93	7.94	10.35	16.19	28.06	40.06	23.17	35.96	49.71	1.52	2.16	2.71	1.38	1.85	2.41	3.77	6.54	9.33	5.40	8.38	11.58	12.76	16.80	20.99	2.97	3.91	4.89	0.65	1.09	1.58	0.15	0.25	0.37
Malta - Pseudomonas aeruginosa CRPA SSI - model 54	3.31E-03	6.43E-03	0.01	0.96	1.59	2.18	0.96	1.60	2.19	0.42	0.52	0.61	7.72E-04	1.50E-03	2.64E-03	0.22	0.37	0.51	0.22	0.37	0.51	2.01	3.09	4.20	0.47	0.72	0.98	0.04	0.07	0.10	9.69E-03	0.02	0.02
Malta - Pseudomonas aeruginosa CRPA UTI - model 53	6.49E-03	0.01	0.02	0.00	0.00	0.00	6.49E-03	0.01	0.02	1.28E-03	2.03E-03	2.90E-03	1.51E-03	2.69E-03	4.42E-03	0.00	0.00	0.00	1.51E-03	2.69E-03	4.42E-03	3.89	5.75	7.51	0.91	1.34	1.75	0.00	0.00	0.00	0.00	0.00	0.00
Malta - Pseudomonas aeruginosa ColRPA BSI - model 56	0.00	0.00	0.00	0.00	0.00	0.00	0.00	0.00	0.00	NaN	NaN	NaN	0.00	0.00	0.00	0.00	0.00	0.00	0.00	0.00	0.00	0.00	0.00	0.00	0.00	0.00	0.00	0.00	0.00	0.00	0.00	0.00	0.00
Malta - Pseudomonas aeruginosa ColRPA OTH - model 60	0.00	0.00	0.00	0.00	0.00	0.00	0.00	0.00	0.00	NaN	NaN	NaN	0.00	0.00	0.00	0.00	0.00	0.00	0.00	0.00	0.00	0.00	0.00	0.00	0.00	0.00	0.00	0.00	0.00	0.00	0.00	0.00	0.00
Malta - Pseudomonas aeruginosa ColRPA RESP - model 57	0.00	0.00	0.00	0.00	0.00	0.00	0.00	0.00	0.00	NaN	NaN	NaN	0.00	0.00	0.00	0.00	0.00	0.00	0.00	0.00	0.00	0.00	0.00	0.00	0.00	0.00	0.00	0.00	0.00	0.00	0.00	0.00	0.00
Malta - Pseudomonas aeruginosa ColRPA SSI - model 59	0.00	0.00	0.00	0.00	0.00	0.00	0.00	0.00	0.00	NaN	NaN	NaN	0.00	0.00	0.00	0.00	0.00	0.00	0.00	0.00	0.00	0.00	0.00	0.00	0.00	0.00	0.00	0.00	0.00	0.00	0.00	0.00	0.00
Malta - Pseudomonas aeruginosa ColRPA UTI - model 58	0.00	0.00	0.00	0.00	0.00	0.00	0.00	0.00	0.00	NaN	NaN	NaN	0.00	0.00	0.00	0.00	0.00	0.00	0.00	0.00	0.00	0.00	0.00	0.00	0.00	0.00	0.00	0.00	0.00	0.00	0.00	0.00	0.00
Malta - Pseudomonas aeruginosa MDRPA BSI - model 46	0.11	0.16	0.26	0.40	1.19	2.39	0.54	1.36	2.61	0.54	1.24	1.88	0.02	0.04	0.06	0.09	0.28	0.56	0.12	0.32	0.61	0.80	1.11	1.56	0.19	0.26	0.36	0.08	0.24	0.48	0.02	0.06	0.11
Malta - Pseudomonas aeruginosa MDRPA OTH - model 50	5.09E-04	2.06E-03	4.93E-03	0.00	0.00	0.00	5.09E-04	2.06E-03	4.93E-03	1.52E-03	3.72E-03	6.38E-03	1.19E-04	4.79E-04	1.15E-03	0.00	0.00	0.00	1.19E-04	4.79E-04	1.15E-03	0.17	0.58	1.00	0.04	0.14	0.23	0.00	0.00	0.00	0.00	0.00	0.00
Malta - Pseudomonas aeruginosa MDRPA RESP - model 47	0.21	0.41	0.64	0.47	1.25	2.55	0.73	1.65	3.09	0.24	0.42	0.59	0.05	0.10	0.15	0.11	0.29	0.59	0.17	0.38	0.72	2.21	4.12	6.08	0.51	0.96	1.42	0.09	0.25	0.51	0.02	0.06	0.12
Malta - Pseudomonas aeruginosa MDRPA SSI - model 49	2.83E-04	1.47E-03	4.52E-03	0.05	0.14	0.23	0.05	0.15	0.24	0.18	0.18	0.19	6.59E-05	3.43E-04	1.05E-03	0.01	0.03	0.05	0.01	0.03	0.06	0.27	0.80	1.29	0.06	0.19	0.30	9.59E-03	0.03	0.05	2.23E-03	6.70E-03	0.01
Malta - Pseudomonas aeruginosa MDRPA UTI - model 48	6.95E-04	2.63E-03	6.57E-03	0.00	0.00	0.00	6.95E-04	2.63E-03	6.57E-03	8.09E-04	1.86E-03	3.63E-03	1.62E-04	6.12E-04	1.53E-03	0.00	0.00	0.00	1.62E-04	6.12E-04	1.53E-03	0.60	1.47	2.30	0.14	0.34	0.54	0.00	0.00	0.00	0.00	0.00	0.00
Malta - Staphylococcus aureus MRSA BSI - model 61	17.56	19.70	21.94	121.53	131.59	142.71	140.30	151.35	163.36	3.10	3.27	3.46	4.09	4.59	5.11	28.31	30.65	33.24	32.68	35.25	38.05	43.28	46.27	48.97	10.08	10.78	11.41	7.69	8.32	8.92	1.79	1.94	2.08
Malta - Staphylococcus aureus MRSA OTH - model 65	0.17	0.22	0.28	0.00	0.00	0.00	0.17	0.22	0.28	2.55E-03	3.29E-03	4.03E-03	0.04	0.05	0.07	0.00	0.00	0.00	0.04	0.05	0.07	61.33	68.39	75.77	14.29	15.93	17.65	0.00	0.00	0.00	0.00	0.00	0.00
Malta - Staphylococcus aureus MRSA RESP - model 62	17.96	20.73	23.72	34.39	39.90	45.50	52.60	60.70	68.60	0.80	0.87	0.93	4.18	4.83	5.52	8.01	9.29	10.60	12.25	14.14	15.98	61.76	69.96	77.90	14.39	16.29	18.14	2.19	2.52	2.84	0.51	0.59	0.66
Malta - Staphylococcus aureus MRSA SSI - model 64	0.11	0.16	0.22	26.28	29.73	33.46	26.41	29.90	33.63	0.37	0.39	0.41	0.03	0.04	0.05	6.12	6.92	7.79	6.15	6.96	7.83	67.49	76.20	84.58	15.72	17.75	19.70	1.99	2.26	2.54	0.46	0.53	0.59
Malta - Staphylococcus aureus MRSA UTI - model 63	0.01	0.02	0.03	0.00	0.00	0.00	0.01	0.02	0.03	1.42E-03	1.87E-03	2.37E-03	3.46E-03	4.92E-03	6.73E-03	0.00	0.00	0.00	3.46E-03	4.92E-03	6.73E-03	8.98	11.38	13.67	2.09	2.65	3.18	0.00	0.00	0.00	0.00	0.00	0.00
Malta - Streptococcus pneumoniae PMRSP BSI - model 76	0.00	0.00	0.00	0.00	0.00	0.00	0.00	0.00	0.00	NaN	NaN	NaN	0.00	0.00	0.00	0.00	0.00	0.00	0.00	0.00	0.00	0.00	0.00	0.00	0.00	0.00	0.00	0.00	0.00	0.00	0.00	0.00	0.00
Malta - Streptococcus pneumoniae PMRSP OTH - model 80	0.00	0.00	0.00	0.00	0.00	0.00	0.00	0.00	0.00	NaN	NaN	NaN	0.00	0.00	0.00	0.00	0.00	0.00	0.00	0.00	0.00	0.00	0.00	0.00	0.00	0.00	0.00	0.00	0.00	0.00	0.00	0.00	0.00
Malta - Streptococcus pneumoniae PMRSP RESP - model 77	0.00	0.00	0.00	0.00	0.00	0.00	0.00	0.00	0.00	NaN	NaN	NaN	0.00	0.00	0.00	0.00	0.00	0.00	0.00	0.00	0.00	0.00	0.00	0.00	0.00	0.00	0.00	0.00	0.00	0.00	0.00	0.00	0.00
Malta - Streptococcus pneumoniae PMRSP SSI - model 79	0.00	0.00	0.00	0.00	0.00	0.00	0.00	0.00	0.00	NaN	NaN	NaN	0.00	0.00	0.00	0.00	0.00	0.00	0.00	0.00	0.00	0.00	0.00	0.00	0.00	0.00	0.00	0.00	0.00	0.00	0.00	0.00	0.00
Malta - Streptococcus pneumoniae PMRSP UTI - model 78	0.00	0.00	0.00	0.00	0.00	0.00	0.00	0.00	0.00	NaN	NaN	NaN	0.00	0.00	0.00	0.00	0.00	0.00	0.00	0.00	0.00	0.00	0.00	0.00	0.00	0.00	0.00	0.00	0.00	0.00	0.00	0.00	0.00
Malta - Streptococcus pneumoniae PRSP BSI - model 71	0.00	0.00	0.00	0.00	0.00	0.00	0.00	0.00	0.00	NaN	NaN	NaN	0.00	0.00	0.00	0.00	0.00	0.00	0.00	0.00	0.00	0.00	0.00	0.00	0.00	0.00	0.00	0.00	0.00	0.00	0.00	0.00	0.00
Malta - Streptococcus pneumoniae PRSP OTH - model 75	0.00	0.00	0.00	0.00	0.00	0.00	0.00	0.00	0.00	NaN	NaN	NaN	0.00	0.00	0.00	0.00	0.00	0.00	0.00	0.00	0.00	0.00	0.00	0.00	0.00	0.00	0.00	0.00	0.00	0.00	0.00	0.00	0.00
Malta - Streptococcus pneumoniae PRSP RESP - model 72	0.00	0.00	0.00	0.00	0.00	0.00	0.00	0.00	0.00	NaN	NaN	NaN	0.00	0.00	0.00	0.00	0.00	0.00	0.00	0.00	0.00	0.00	0.00	0.00	0.00	0.00	0.00	0.00	0.00	0.00	0.00	0.00	0.00
Malta - Streptococcus pneumoniae PRSP SSI - model 74	0.00	0.00	0.00	0.00	0.00	0.00	0.00	0.00	0.00	NaN	NaN	NaN	0.00	0.00	0.00	0.00	0.00	0.00	0.00	0.00	0.00	0.00	0.00	0.00	0.00	0.00	0.00	0.00	0.00	0.00	0.00	0.00	0.00
Malta - Streptococcus pneumoniae PRSP UTI - model 73	0.00	0.00	0.00	0.00	0.00	0.00	0.00	0.00	0.00	NaN	NaN	NaN	0.00	0.00	0.00	0.00	0.00	0.00	0.00	0.00	0.00	0.00	0.00	0.00	0.00	0.00	0.00	0.00	0.00	0.00	0.00	0.00	0.00
**Total (Median & 95% UIs)**	78.45	94.56	113.23	422.92	527.84	649.52	507.25	622.31	755.97		-		18.27	22.02	26.37	98.50	122.94	151.28	118.15	144.94	176.08	516.72	607.56	697.54	120.35	141.51	162.47	23.38	28.61	34.36	5.45	6.66	8.00
**Total (average)**		**94.90**			**529.36**			**624.26**			-			**22.10**			**123.30**			**145.40**			**606.94**			**141.36**			**28.66**			**6.68**	

Infection	YLD			YLL			DALY			DALY per Case			YLD per 100,000		
Colistin resistant *Acinetobacter spp.*	4.54	6.94	9.95	16.94	37.07	62.49	21.15	43.95	71.30	7.91	14.09	21.30	0.03	0.04	0.06
Carbapenem resistant *Acinetobacter spp.* (excluding those resistant to colistin)	3.64	5.41	7.63	13.82	28.87	47.17	18.00	34.36	53.64	4.32	7.30	10.41	0.02	0.03	0.05
Aminoglycosides and fluoroquinolones resistant *Acinetobacter* spp. (excluding those resistant to colistin and/or carbapenem)	2.91	4.12	5.55	11.47	21.57	33.75	14.96	25.72	38.83	5.34	8.34	11.55	0.02	0.02	0.03
Vancomycin resistant Enterococci (*E. faecalis* and *E. faecium*)	6.42	9.28	13.53	63.49	90.27	128.82	70.54	99.43	140.82	5.57	6.15	6.88	0.04	0.05	0.08
Colistin resistant *Escherichia coli*	21.50	25.06	29.13	355.22	435.40	520.82	377.41	460.06	549.30	11.24	13.37	15.49	0.13	0.15	0.17
Carbapenem resistant *Escherichia coli* (excluding those resistant to colistin)	1.04	1.74	2.72	9.63	18.11	29.27	10.95	19.82	31.58	8.69	13.26	18.08	0.01	0.01	0.02
Third-generation cephalosporin resistant *Escherichia coli* (excluding those resistant to colistin and/or carbapenem)	312.03	352.48	393.50	1,871.90	2,098.90	2,344.43	2,189.15	2,449.98	2,727.98	5.16	5.59	6.04	1.85	2.09	2.33
Colistin resistant *Klebsiella pneumoniae*	5.43	7.04	8.95	89.86	123.98	164.06	96.14	131.03	172.34	11.05	14.58	18.10	0.03	0.04	0.05
Carbapenem resistant *Klebsiella pneumoniae* (excluding those resistant to colistin)	0.00	0.00	0.00	0.00	0.00	0.00	0.00	0.00	0.00	0.00	0.00	0.00	0.00	0.00	0.00
Third-generation cephalosporin resistant *Klebsiella pneumoniae* (excluding those resistant to colistin and/or carbapenem)	90.71	99.78	110.00	446.24	480.59	514.85	539.78	580.67	622.29	5.16	5.43	5.72	0.54	0.59	0.65
Colistin resistant *Pseudomonas aeruginosa*	2.20	3.49	5.18	7.83	17.39	30.41	10.66	20.91	34.82	2.28	4.11	6.28	0.01	0.02	0.03
Carbapenem resistant *Pseudomonas aeruginosa* (excluding those resistant to colistin)	61.84	73.93	88.15	272.29	377.60	497.80	339.93	451.31	576.06	6.66	8.51	10.37	0.37	0.44	0.52
Pseudomonas aeruginosa resistant to three or more antimicrobial groups (excluding those resistant to colistin and/or carbapenem)	12.76	16.48	20.68	49.77	77.89	111.25	64.86	94.28	130.30	4.30	5.95	7.71	0.08	0.10	0.12
Methicillin resistant *Staphylococcus aureus*	59.55	67.22	76.61	293.66	321.54	353.47	355.48	389.18	426.28	7.60	8.05	8.50	0.35	0.40	0.45
Penicillin and macrolides resistant *Streptococcus pneumoniae* (excluding those only resistant to penicillin)	12.20	15.85	21.35	44.50	52.12	64.30	58.20	67.86	83.97	6.24	7.05	7.96	0.07	0.09	0.13
Penicillin tesistant *Streptococcus pneumoniae* (excluding those resistant to macrolides)	5.47	6.92	9.00	19.40	22.47	26.88	25.34	29.41	35.35	3.74	4.04	4.35	0.03	0.04	0.05
Total	602.23	695.73	801.92	3,566.04	4,203.77	4,929.77	4,192.56	4,897.96	5,694.86				3.56	4.12	4.74

Infection	YLL per 100,000			DALY per 100,000			Cases per year			Incidence per 100,000			Deaths per year			Mortality per 100,000		
Colistin resistant *Acinetobacter spp.*	0.10	0.22	0.37	0.13	0.26	0.42	6.52	9.21	12.05	0.04	0.05	0.07	0.42	0.79	1.24	0.00	0.00	0.01
Carbapenem resistant *Acinetobacter spp.* (excluding those resistant to colistin)	0.08	0.17	0.28	0.11	0.20	0.32	9.59	13.85	18.37	0.06	0.08	0.11	0.60	1.19	1.86	0.00	0.01	0.01
Aminoglycosides and fluoroquinolones resistant *Acinetobacter* spp. (excluding those resistant to colistin and/or carbapenem)	0.07	0.13	0.20	0.09	0.15	0.23	6.43	9.24	11.94	0.04	0.05	0.07	0.42	0.78	1.21	0.00	0.00	0.01
Vancomycin resistant Enterococci (*E. faecalis* and *E. faecium*)	0.38	0.53	0.76	0.42	0.59	0.83	38.97	62.51	90.07	0.23	0.37	0.53	2.89	4.21	6.27	0.02	0.02	0.04
Colistin resistant *Escherichia coli*	2.10	2.58	3.08	2.23	2.72	3.25	229.42	265.04	302.69	1.36	1.57	1.79	19.13	23.35	27.92	0.11	0.14	0.17
Carbapenem resistant *Escherichia coli* (excluding those resistant to colistin)	0.06	0.11	0.17	0.06	0.12	0.19	7.06	12.00	17.28	0.04	0.07	0.10	0.32	0.60	0.98	0.00	0.00	0.01
Third-generation cephalosporin resistant *Escherichia coli* (excluding those resistant to colistin and/or carbapenem)	11.08	12.42	13.87	12.95	14.50	16.14	3,115.24	3,503.37	3,899.09	18.43	20.73	23.07	93.11	107.10	121.02	0.55	0.63	0.72
Colistin resistant *Klebsiella pneumoniae*	0.53	0.73	0.97	0.57	0.78	1.02	19.78	27.09	34.34	0.12	0.16	0.20	4.45	6.03	7.86	0.03	0.04	0.05
Carbapenem resistant *Klebsiella pneumoniae* (excluding those resistant to colistin)	0.00	0.00	0.00	0.00	0.00	0.00	0.00	0.00	0.00	0.00	0.00	0.00	0.00	0.00	0.00	0.00	0.00	0.00
Third-generation cephalosporin resistant *Klebsiella pneumoniae* (excluding those resistant to colistin and/or carbapenem)	2.64	2.84	3.05	3.19	3.44	3.68	399.02	444.33	490.19	2.36	2.63	2.90	22.35	24.39	26.34	0.13	0.14	0.16
Colistin resistant *Pseudomonas aeruginosa*	0.05	0.10	0.18	0.06	0.12	0.21	14.91	23.67	32.29	0.09	0.14	0.19	0.78	1.62	2.65	0.00	0.01	0.02
Carbapenem resistant *Pseudomonas aeruginosa* (excluding those resistant to colistin)	1.61	2.23	2.95	2.01	2.67	3.41	194.09	236.36	279.81	1.15	1.40	1.66	11.70	16.30	21.52	0.07	0.10	0.13
Pseudomonas aeruginosa resistant to three or more antimicrobial groups (excluding those resistant to colistin and/or carbapenem)	0.29	0.46	0.66	0.38	0.56	0.77	55.66	71.05	86.25	0.33	0.42	0.51	3.10	4.59	6.21	0.02	0.03	0.04
Methicillin resistant *Staphylococcus aureus*	1.74	1.90	2.09	2.10	2.30	2.52	225.17	249.03	273.31	1.33	1.47	1.62	10.56	11.53	12.56	0.06	0.07	0.07
Penicillin and macrolides resistant *Streptococcus pneumoniae* (excluding those only resistant to penicillin)	0.26	0.31	0.38	0.34	0.40	0.50	26.52	31.32	39.41	0.16	0.19	0.23	1.67	1.93	2.35	0.01	0.01	0.01
Penicillin tesistant *Streptococcus pneumoniae* (excluding those resistant to macrolides)	0.11	0.13	0.16	0.15	0.17	0.21	19.94	23.55	29.06	0.12	0.14	0.17	1.25	1.46	1.73	0.01	0.01	0.01
Total	21.10	24.87	29.17	24.81	28.98	33.70	4,368.31	4,981.61	5,616.15	25.85	29.48	33.23	172.75	205.87	241.72	1.02	1.22	1.43

Model	YLD			YLL			DALY			DALY per Case			YLD per 100,000		
Netherlands - Acinetobacter spp. CRACI BSI - model 36	2.25	3.10	4.32	10.58	23.76	39.90	13.50	26.92	43.29	2.90	5.64	8.47	0.01	0.02	0.03
Netherlands - Acinetobacter spp. CRACI OTH - model 40	1.82E-04	5.98E-04	1.35E-03	0.00	0.00	0.00	1.82E-04	5.98E-04	1.35E-03	1.89E-03	3.72E-03	5.92E-03	1.07E-06	3.54E-06	8.01E-06
Netherlands - Acinetobacter spp. CRACI RESP - model 37	1.38	2.30	3.29	2.92	4.53	6.42	4.17	6.86	9.48	1.12	1.32	1.54	8.19E-03	0.01	0.02
Netherlands - Acinetobacter spp. CRACI SSI - model 39	1.29E-03	3.52E-03	7.29E-03	0.32	0.58	0.85	0.32	0.59	0.86	0.30	0.34	0.38	7.61E-06	2.08E-05	4.31E-05
Netherlands - Acinetobacter spp. CRACI UTI - model 38	1.52E-03	3.84E-03	7.69E-03	0.00	0.00	0.00	1.52E-03	3.84E-03	7.69E-03	1.01E-03	2.05E-03	3.27E-03	8.99E-06	2.27E-05	4.55E-05
Netherlands - Acinetobacter spp. ColRACI BSI - model 41	2.75	3.95	5.62	13.00	30.57	53.11	15.27	34.44	57.77	5.57	10.96	17.33	0.02	0.02	0.03
Netherlands - Acinetobacter spp. ColRACI OTH - model 45	1.15E-04	3.73E-04	8.12E-04	0.00	0.00	0.00	1.15E-04	3.73E-04	8.12E-04	1.91E-03	3.69E-03	5.88E-03	6.79E-07	2.21E-06	4.81E-06
Netherlands - Acinetobacter spp. ColRACI RESP - model 42	1.78	2.98	4.31	3.60	5.85	8.43	5.53	8.85	12.57	1.90	2.58	3.30	0.01	0.02	0.03
Netherlands - Acinetobacter spp. ColRACI SSI - model 44	8.49E-04	2.40E-03	5.17E-03	0.34	0.65	0.95	0.35	0.65	0.96	0.45	0.55	0.66	5.02E-06	1.42E-05	3.06E-05
Netherlands - Acinetobacter spp. ColRACI UTI - model 43	9.90E-04	2.55E-03	4.74E-03	0.00	0.00	0.00	9.90E-04	2.55E-03	4.74E-03	1.01E-03	2.03E-03	3.21E-03	5.86E-06	1.51E-05	2.81E-05
Netherlands - Acinetobacter spp. MDRACI BSI - model 31	1.77	2.34	3.14	8.84	17.48	28.20	11.07	19.83	31.00	3.57	6.27	9.17	0.01	0.01	0.02
Netherlands - Acinetobacter spp. MDRACI OTH - model 35	1.00E-04	3.63E-04	8.30E-04	0.00	0.00	0.00	1.00E-04	3.63E-04	8.30E-04	1.87E-03	3.72E-03	5.97E-03	5.94E-07	2.15E-06	4.91E-06
Netherlands - Acinetobacter spp. MDRACI RESP - model 32	1.14	1.77	2.40	2.27	3.46	4.63	3.53	5.25	6.90	1.33	1.52	1.74	6.75E-03	0.01	0.01
Netherlands - Acinetobacter spp. MDRACI SSI - model 34	8.41E-04	2.35E-03	5.14E-03	0.36	0.63	0.92	0.36	0.64	0.93	0.43	0.54	0.64	4.98E-06	1.39E-05	3.04E-05
Netherlands - Acinetobacter spp. MDRACI UTI - model 33	1.02E-03	2.53E-03	4.86E-03	0.00	0.00	0.00	1.02E-03	2.53E-03	4.86E-03	1.03E-03	2.00E-03	3.22E-03	6.01E-06	1.50E-05	2.87E-05
Netherlands - Enterococcus faecalis and E. faecium VRE BSI - model 66	6.38	9.19	13.37	56.26	78.03	111.05	63.26	87.09	122.94	5.06	5.58	6.26	0.04	0.05	0.08
Netherlands - Enterococcus faecalis and E. faecium VRE OTH - model 70	0.01	0.03	0.05	0.00	0.00	0.00	0.01	0.03	0.05	9.86E-04	1.59E-03	2.52E-03	8.12E-05	1.56E-04	2.89E-04
Netherlands - Enterococcus faecalis and E. faecium VRE RESP - model 67	0.00	0.00	0.00	0.00	0.00	0.00	0.00	0.00	0.00	NaN	NaN	NaN	0.00	0.00	0.00
Netherlands - Enterococcus faecalis and E. faecium VRE SSI - model 69	0.02	0.04	0.09	7.24	12.25	17.77	7.26	12.30	17.81	0.51	0.57	0.62	1.24E-04	2.63E-04	5.25E-04
Netherlands - Enterococcus faecalis and E. faecium VRE UTI - model 68	7.44E-03	0.01	0.03	0.00	0.00	0.00	7.44E-03	0.01	0.03	1.11E-03	1.87E-03	2.80E-03	4.40E-05	8.62E-05	1.61E-04
Netherlands - Escherichia coli 3GCREC BSI - model 16	239.51	265.65	290.64	1,548.14	1,716.16	1,897.92	1,792.74	1,980.31	2,179.07	3.73	4.04	4.36	1.42	1.57	1.72
Netherlands - Escherichia coli 3GCREC OTH - model 20	0.40	0.54	0.71	0.00	0.00	0.00	0.40	0.54	0.71	2.70E-03	3.35E-03	4.15E-03	2.39E-03	3.17E-03	4.23E-03
Netherlands - Escherichia coli 3GCREC RESP - model 17	68.08	81.05	95.53	131.88	157.36	185.24	199.88	238.45	280.44	1.01	1.10	1.20	0.40	0.48	0.57
Netherlands - Escherichia coli 3GCREC SSI - model 19	0.81	1.10	1.44	191.88	225.38	261.27	192.91	226.54	262.58	0.41	0.44	0.46	4.77E-03	6.53E-03	8.51E-03
Netherlands - Escherichia coli 3GCREC UTI - model 18	3.22	4.14	5.17	0.00	0.00	0.00	3.22	4.14	5.17	1.56E-03	1.95E-03	2.38E-03	0.02	0.02	0.03
Netherlands - Escherichia coli CREC BSI - model 21	0.87	1.27	1.92	9.02	16.71	27.04	10.17	17.94	28.59	6.99	11.35	15.93	5.12E-03	7.49E-03	0.01
Netherlands - Escherichia coli CREC OTH - model 25	9.66E-04	2.87E-03	8.01E-03	0.00	0.00	0.00	9.66E-04	2.87E-03	8.01E-03	1.23E-03	3.18E-03	7.40E-03	5.71E-06	1.70E-05	4.74E-05
Netherlands - Escherichia coli CREC RESP - model 22	0.17	0.45	0.75	0.33	0.88	1.46	0.50	1.34	2.18	1.42	1.63	1.87	1.02E-03	2.68E-03	4.46E-03
Netherlands - Escherichia coli CREC SSI - model 24	8.29E-04	3.55E-03	9.83E-03	0.28	0.52	0.77	0.28	0.52	0.77	0.27	0.27	0.28	4.91E-06	2.10E-05	5.81E-05
Netherlands - Escherichia coli CREC UTI - model 23	4.50E-03	0.01	0.03	0.00	0.00	0.00	4.50E-03	0.01	0.03	7.61E-04	1.97E-03	4.04E-03	2.66E-05	7.59E-05	1.70E-04
Netherlands - Escherichia coli ColREC BSI - model 26	16.20	18.32	20.86	330.51	405.15	484.68	347.40	423.05	505.12	9.90	11.90	13.87	0.10	0.11	0.12
Netherlands - Escherichia coli ColREC OTH - model 30	0.05	0.07	0.10	0.00	0.00	0.00	0.05	0.07	0.10	2.56E-03	3.50E-03	4.75E-03	2.91E-04	4.20E-04	5.99E-04
Netherlands - Escherichia coli ColREC RESP - model 27	4.97	6.27	7.63	9.74	12.03	14.78	14.69	18.31	22.23	0.92	1.03	1.14	0.03	0.04	0.05
Netherlands - Escherichia coli ColREC SSI - model 29	0.06	0.09	0.12	14.97	18.23	21.36	15.04	18.32	21.45	0.41	0.44	0.46	3.62E-04	5.27E-04	7.36E-04
Netherlands - Escherichia coli ColREC UTI - model 28	0.22	0.31	0.40	0.00	0.00	0.00	0.22	0.31	0.40	1.57E-03	2.08E-03	2.68E-03	1.33E-03	1.83E-03	2.40E-03
Netherlands - Klebsiella pneumoniae 3GCRKP BSI - model 1	57.21	61.91	67.60	364.49	387.78	410.84	424.04	449.83	476.15	3.70	3.87	4.06	0.34	0.37	0.40
Netherlands - Klebsiella pneumoniae 3GCRKP OTH - model 5	0.11	0.15	0.20	0.00	0.00	0.00	0.11	0.15	0.20	2.65E-03	3.34E-03	4.11E-03	6.79E-04	9.08E-04	1.16E-03
Netherlands - Klebsiella pneumoniae 3GCRKP RESP - model 2	33.10	37.35	41.73	63.96	72.02	80.22	97.53	109.53	121.68	1.02	1.09	1.17	0.20	0.22	0.25
Netherlands - Klebsiella pneumoniae 3GCRKP SSI - model 4	0.07	0.10	0.13	17.79	20.79	23.80	17.88	20.89	23.93	0.43	0.46	0.48	4.29E-04	5.72E-04	7.71E-04
Netherlands - Klebsiella pneumoniae 3GCRKP UTI - model 3	0.21	0.27	0.33	0.00	0.00	0.00	0.21	0.27	0.33	1.60E-03	1.96E-03	2.37E-03	1.23E-03	1.58E-03	1.98E-03
Netherlands - Klebsiella pneumoniae CRKP BSI - model 6	0.00	0.00	0.00	0.00	0.00	0.00	0.00	0.00	0.00	NaN	NaN	NaN	0.00	0.00	0.00
Netherlands - Klebsiella pneumoniae CRKP OTH - model 10	0.00	0.00	0.00	0.00	0.00	0.00	0.00	0.00	0.00	NaN	NaN	NaN	0.00	0.00	0.00
Netherlands - Klebsiella pneumoniae CRKP RESP - model 7	0.00	0.00	0.00	0.00	0.00	0.00	0.00	0.00	0.00	NaN	NaN	NaN	0.00	0.00	0.00
Netherlands - Klebsiella pneumoniae CRKP SSI - model 9	0.00	0.00	0.00	0.00	0.00	0.00	0.00	0.00	0.00	NaN	NaN	NaN	0.00	0.00	0.00
Netherlands - Klebsiella pneumoniae CRKP UTI - model 8	0.00	0.00	0.00	0.00	0.00	0.00	0.00	0.00	0.00	NaN	NaN	NaN	0.00	0.00	0.00
Netherlands - Klebsiella pneumoniae ColRKP BSI - model 11	4.44	5.42	6.68	87.17	119.52	157.87	92.41	124.95	163.89	9.66	12.96	16.25	0.03	0.03	0.04
Netherlands - Klebsiella pneumoniae ColRKP OTH - model 15	7.64E-03	0.02	0.03	0.00	0.00	0.00	7.64E-03	0.02	0.03	2.16E-03	3.46E-03	5.34E-03	4.52E-05	9.13E-05	1.62E-04
Netherlands - Klebsiella pneumoniae ColRKP RESP - model 12	0.97	1.58	2.21	1.88	3.03	4.18	2.89	4.63	6.38	0.99	1.12	1.27	5.75E-03	9.33E-03	0.01
Netherlands - Klebsiella pneumoniae ColRKP SSI - model 14	3.14E-03	5.95E-03	0.01	0.81	1.43	2.02	0.82	1.43	2.02	0.40	0.49	0.57	1.86E-05	3.52E-05	6.04E-05
Netherlands - Klebsiella pneumoniae ColRKP UTI - model 13	6.91E-03	0.01	0.02	0.00	0.00	0.00	6.91E-03	0.01	0.02	1.36E-03	2.08E-03	3.11E-03	4.09E-05	7.18E-05	1.17E-04
Netherlands - Pseudomonas aeruginosa CRPA BSI - model 51	17.93	20.90	24.39	129.14	179.10	235.34	149.27	199.83	256.82	4.63	6.05	7.46	0.11	0.12	0.14
Netherlands - Pseudomonas aeruginosa CRPA OTH - model 55	0.04	0.06	0.09	0.00	0.00	0.00	0.04	0.06	0.09	2.78E-03	3.68E-03	4.69E-03	2.46E-04	3.61E-04	5.12E-04
Netherlands - Pseudomonas aeruginosa CRPA RESP - model 52	43.78	52.83	63.47	135.74	188.11	249.15	183.12	240.89	305.66	1.60	1.99	2.41	0.26	0.31	0.38
Netherlands - Pseudomonas aeruginosa CRPA SSI - model 54	0.03	0.05	0.07	7.41	10.39	13.32	7.44	10.45	13.38	0.42	0.46	0.50	1.79E-04	2.78E-04	4.31E-04
Netherlands - Pseudomonas aeruginosa CRPA UTI - model 53	0.06	0.09	0.12	0.00	0.00	0.00	0.06	0.09	0.12	1.52E-03	2.04E-03	2.65E-03	3.53E-04	5.10E-04	7.17E-04
Netherlands - Pseudomonas aeruginosa ColRPA BSI - model 56	0.68	0.98	1.38	3.62	8.12	14.17	4.57	9.11	15.35	1.44	2.81	4.38	4.03E-03	5.81E-03	8.16E-03
Netherlands - Pseudomonas aeruginosa ColRPA OTH - model 60	2.48E-03	6.11E-03	0.01	0.00	0.00	0.00	2.48E-03	6.11E-03	0.01	1.91E-03	3.77E-03	5.70E-03	1.47E-05	3.62E-05	7.17E-05
Netherlands - Pseudomonas aeruginosa ColRPA RESP - model 57	1.51	2.49	3.76	3.80	8.37	14.88	5.67	10.88	18.08	0.56	0.91	1.38	8.93E-03	0.01	0.02
Netherlands - Pseudomonas aeruginosa ColRPA SSI - model 59	1.49E-03	4.47E-03	9.64E-03	0.41	0.89	1.36	0.41	0.90	1.37	0.28	0.39	0.51	8.83E-06	2.65E-05	5.70E-05
Netherlands - Pseudomonas aeruginosa ColRPA UTI - model 58	3.51E-03	8.35E-03	0.02	0.00	0.00	0.00	3.51E-03	8.35E-03	0.02	1.02E-03	2.00E-03	3.30E-03	2.08E-05	4.94E-05	9.18E-05
Netherlands - Pseudomonas aeruginosa MDRPA BSI - model 46	3.78	4.63	5.64	22.34	35.83	51.65	26.65	40.38	56.18	2.83	4.09	5.41	0.02	0.03	0.03
Netherlands - Pseudomonas aeruginosa MDRPA OTH - model 50	0.01	0.02	0.03	0.00	0.00	0.00	0.01	0.02	0.03	2.66E-03	3.72E-03	4.96E-03	6.82E-05	1.10E-04	1.66E-04
Netherlands - Pseudomonas aeruginosa MDRPA RESP - model 47	8.95	11.79	14.96	25.23	38.93	55.54	35.97	50.71	69.98	1.05	1.39	1.77	0.05	0.07	0.09
Netherlands - Pseudomonas aeruginosa MDRPA SSI - model 49	8.42E-03	0.01	0.02	2.19	3.13	4.06	2.20	3.15	4.08	0.41	0.47	0.52	4.98E-05	8.46E-05	1.32E-04
Netherlands - Pseudomonas aeruginosa MDRPA UTI - model 48	0.02	0.03	0.04	0.00	0.00	0.00	0.02	0.03	0.04	1.44E-03	2.05E-03	2.75E-03	9.70E-05	1.54E-04	2.31E-04
Netherlands - Staphylococcus aureus MRSA BSI - model 61	29.46	32.72	36.75	205.36	221.80	239.28	236.78	254.86	273.38	5.67	5.99	6.28	0.17	0.19	0.22
Netherlands - Staphylococcus aureus MRSA OTH - model 65	0.16	0.20	0.26	0.00	0.00	0.00	0.16	0.20	0.26	2.55E-03	3.25E-03	4.04E-03	9.27E-04	1.21E-03	1.54E-03
Netherlands - Staphylococcus aureus MRSA RESP - model 62	29.82	34.13	39.38	58.72	66.55	77.49	88.82	100.76	115.76	1.47	1.58	1.72	0.18	0.20	0.23
Netherlands - Staphylococcus aureus MRSA SSI - model 64	0.10	0.15	0.20	29.59	33.19	36.70	29.71	33.33	36.85	0.46	0.48	0.50	6.19E-04	8.89E-04	1.18E-03
Netherlands - Staphylococcus aureus MRSA UTI - model 63	0.01	0.02	0.03	0.00	0.00	0.00	0.01	0.02	0.03	1.47E-03	1.87E-03	2.33E-03	8.32E-05	1.13E-04	1.56E-04
Netherlands - Streptococcus pneumoniae PMRSP BSI - model 76	3.60	4.42	5.36	27.15	29.92	32.72	31.49	34.38	37.59	5.11	5.58	6.10	0.02	0.03	0.03
Netherlands - Streptococcus pneumoniae PMRSP OTH - model 80	2.37E-03	4.22E-03	6.79E-03	0.00	0.00	0.00	2.37E-03	4.22E-03	6.79E-03	1.12E-03	1.93E-03	2.92E-03	1.40E-05	2.50E-05	4.02E-05
Netherlands - Streptococcus pneumoniae PMRSP RESP - model 77	8.59	11.42	15.99	17.35	22.20	31.58	26.71	33.48	46.37	1.13	1.47	1.85	0.05	0.07	0.09
Netherlands - Streptococcus pneumoniae PMRSP SSI - model 79	0.00	0.00	0.00	0.00	0.00	0.00	0.00	0.00	0.00	NaN	NaN	NaN	0.00	0.00	0.00
Netherlands - Streptococcus pneumoniae PMRSP UTI - model 78	0.00	0.00	0.00	0.00	0.00	0.00	0.00	0.00	0.00	NaN	NaN	NaN	0.00	0.00	0.00
Netherlands - Streptococcus pneumoniae PRSP BSI - model 71	1.61	1.90	2.24	11.85	12.84	13.94	13.69	14.75	15.85	2.96	3.19	3.43	9.50E-03	0.01	0.01
Netherlands - Streptococcus pneumoniae PRSP OTH - model 75	1.82E-03	3.23E-03	5.01E-03	0.00	0.00	0.00	1.82E-03	3.23E-03	5.01E-03	1.12E-03	1.97E-03	2.97E-03	1.07E-05	1.91E-05	2.97E-05
Netherlands - Streptococcus pneumoniae PRSP RESP - model 72	3.86	5.02	6.76	7.55	9.62	12.95	11.64	14.65	19.50	0.77	0.85	0.92	0.02	0.03	0.04
Netherlands - Streptococcus pneumoniae PRSP SSI - model 74	0.00	0.00	0.00	0.00	0.00	0.00	0.00	0.00	0.00	NaN	NaN	NaN	0.00	0.00	0.00
Netherlands - Streptococcus pneumoniae PRSP UTI - model 73	0.00	0.00	0.00	0.00	0.00	0.00	0.00	0.00	0.00	NaN	NaN	NaN	0.00	0.00	0.00
**Total (Median & 95% UIs)**	602.23	695.73	801.92	3,566.04	4,203.77	4,929.77	4,192.56	4,897.96	5,694.86		-		3.56	4.12	4.74
**Total (average)**		**698.40**			**4,215.35**			**4,913.75**			-			**4.13**	

Model	YLL per 100,000			DALY per 100,000			Cases per year			Incidence per 100,000			Deaths per year			Mortality per 100,000		
Netherlands - Acinetobacter spp. CRACI BSI - model 36	0.06	0.14	0.24	0.08	0.16	0.26	4.00	4.80	5.76	0.02	0.03	0.03	0.46	0.98	1.56	2.71E-03	5.78E-03	9.26E-03
Netherlands - Acinetobacter spp. CRACI OTH - model 40	0.00	0.00	0.00	1.07E-06	3.54E-06	8.01E-06	0.06	0.16	0.31	3.32E-04	9.59E-04	1.82E-03	0.00	0.00	0.00	0.00	0.00	0.00
Netherlands - Acinetobacter spp. CRACI RESP - model 37	0.02	0.03	0.04	0.02	0.04	0.06	3.58	5.20	6.81	0.02	0.03	0.04	0.13	0.19	0.25	7.67E-04	1.10E-03	1.50E-03
Netherlands - Acinetobacter spp. CRACI SSI - model 39	1.89E-03	3.44E-03	5.01E-03	1.90E-03	3.46E-03	5.09E-03	0.93	1.74	2.59	5.52E-03	0.01	0.02	0.02	0.03	0.05	9.51E-05	1.86E-04	2.73E-04
Netherlands - Acinetobacter spp. CRACI UTI - model 38	0.00	0.00	0.00	8.99E-06	2.27E-05	4.55E-05	1.02	1.95	2.90	6.03E-03	0.01	0.02	0.00	0.00	0.00	0.00	0.00	0.00
Netherlands - Acinetobacter spp. ColRACI BSI - model 41	0.08	0.18	0.31	0.09	0.20	0.34	2.69	3.18	3.77	0.02	0.02	0.02	0.32	0.64	1.04	1.89E-03	3.79E-03	6.13E-03
Netherlands - Acinetobacter spp. ColRACI OTH - model 45	0.00	0.00	0.00	6.79E-07	2.21E-06	4.81E-06	0.04	0.11	0.19	2.19E-04	6.35E-04	1.15E-03	0.00	0.00	0.00	0.00	0.00	0.00
Netherlands - Acinetobacter spp. ColRACI RESP - model 42	0.02	0.03	0.05	0.03	0.05	0.07	2.44	3.45	4.49	0.01	0.02	0.03	0.09	0.12	0.17	5.10E-04	7.25E-04	9.91E-04
Netherlands - Acinetobacter spp. ColRACI SSI - model 44	2.04E-03	3.84E-03	5.65E-03	2.05E-03	3.85E-03	5.66E-03	0.65	1.18	1.70	3.84E-03	6.98E-03	0.01	0.01	0.03	0.04	8.12E-05	1.58E-04	2.34E-04
Netherlands - Acinetobacter spp. ColRACI UTI - model 43	0.00	0.00	0.00	5.86E-06	1.51E-05	2.81E-05	0.71	1.29	1.91	4.21E-03	7.65E-03	0.01	0.00	0.00	0.00	0.00	0.00	0.00
Netherlands - Acinetobacter spp. MDRACI BSI - model 31	0.05	0.10	0.17	0.07	0.12	0.18	2.69	3.18	3.77	0.02	0.02	0.02	0.32	0.63	1.00	1.90E-03	3.75E-03	5.93E-03
Netherlands - Acinetobacter spp. MDRACI OTH - model 35	0.00	0.00	0.00	5.94E-07	2.15E-06	4.91E-06	0.03	0.10	0.19	2.00E-04	6.11E-04	1.11E-03	0.00	0.00	0.00	0.00	0.00	0.00
Netherlands - Acinetobacter spp. MDRACI RESP - model 32	0.01	0.02	0.03	0.02	0.03	0.04	2.39	3.46	4.43	0.01	0.02	0.03	0.08	0.12	0.16	4.92E-04	7.36E-04	9.71E-04
Netherlands - Acinetobacter spp. MDRACI SSI - model 34	2.10E-03	3.75E-03	5.46E-03	2.12E-03	3.76E-03	5.48E-03	0.69	1.18	1.66	4.07E-03	7.01E-03	9.83E-03	0.01	0.03	0.04	8.15E-05	1.56E-04	2.34E-04
Netherlands - Acinetobacter spp. MDRACI UTI - model 33	0.00	0.00	0.00	6.01E-06	1.50E-05	2.87E-05	0.63	1.31	1.89	3.74E-03	7.75E-03	0.01	0.00	0.00	0.00	0.00	0.00	0.00
Netherlands - Enterococcus faecalis and E. faecium VRE BSI - model 66	0.33	0.46	0.66	0.37	0.52	0.73	11.15	15.64	23.20	0.07	0.09	0.14	2.54	3.57	5.33	0.02	0.02	0.03
Netherlands - Enterococcus faecalis and E. faecium VRE OTH - model 70	0.00	0.00	0.00	8.12E-05	1.56E-04	2.89E-04	9.94	17.00	24.11	0.06	0.10	0.14	0.00	0.00	0.00	0.00	0.00	0.00
Netherlands - Enterococcus faecalis and E. faecium VRE RESP - model 67	0.00	0.00	0.00	0.00	0.00	0.00	0.00	0.00	0.00	0.00	0.00	0.00	0.00	0.00	0.00	0.00	0.00	0.00
Netherlands - Enterococcus faecalis and E. faecium VRE SSI - model 69	0.04	0.07	0.11	0.04	0.07	0.11	13.05	21.82	31.12	0.08	0.13	0.18	0.35	0.63	0.94	2.10E-03	3.75E-03	5.57E-03
Netherlands - Enterococcus faecalis and E. faecium VRE UTI - model 68	0.00	0.00	0.00	4.40E-05	8.62E-05	1.61E-04	4.83	8.05	11.65	0.03	0.05	0.07	0.00	0.00	0.00	0.00	0.00	0.00
Netherlands - Escherichia coli 3GCREC BSI - model 16	9.16	10.15	11.23	10.61	11.72	12.89	457.06	490.76	530.03	2.70	2.90	3.14	74.61	84.85	95.09	0.44	0.50	0.56
Netherlands - Escherichia coli 3GCREC OTH - model 20	0.00	0.00	0.00	2.39E-03	3.17E-03	4.23E-03	131.16	162.13	192.32	0.78	0.96	1.14	0.00	0.00	0.00	0.00	0.00	0.00
Netherlands - Escherichia coli 3GCREC RESP - model 17	0.78	0.93	1.10	1.18	1.41	1.66	180.17	216.71	254.57	1.07	1.28	1.51	6.38	7.80	9.30	0.04	0.05	0.06
Netherlands - Escherichia coli 3GCREC SSI - model 19	1.14	1.33	1.55	1.14	1.34	1.55	449.46	518.75	589.03	2.66	3.07	3.49	12.12	14.44	16.63	0.07	0.09	0.10
Netherlands - Escherichia coli 3GCREC UTI - model 18	0.00	0.00	0.00	0.02	0.02	0.03	1,897.39	2,115.01	2,333.14	11.23	12.51	13.80	0.00	0.00	0.00	0.00	0.00	0.00
Netherlands - Escherichia coli CREC BSI - model 21	0.05	0.10	0.16	0.06	0.11	0.17	1.23	1.59	2.11	7.29E-03	9.41E-03	0.01	0.30	0.56	0.90	1.78E-03	3.29E-03	5.34E-03
Netherlands - Escherichia coli CREC OTH - model 25	0.00	0.00	0.00	5.71E-06	1.70E-05	4.74E-05	0.43	0.92	1.45	2.57E-03	5.45E-03	8.59E-03	0.00	0.00	0.00	0.00	0.00	0.00
Netherlands - Escherichia coli CREC RESP - model 22	1.95E-03	5.22E-03	8.63E-03	2.98E-03	7.94E-03	0.01	0.31	0.82	1.30	1.84E-03	4.88E-03	7.71E-03	0.01	0.03	0.05	6.48E-05	1.74E-04	2.87E-04
Netherlands - Escherichia coli CREC SSI - model 24	1.63E-03	3.06E-03	4.53E-03	1.64E-03	3.09E-03	4.55E-03	1.02	1.91	2.82	6.04E-03	0.01	0.02	9.19E-03	0.02	0.03	5.44E-05	1.02E-04	1.50E-04
Netherlands - Escherichia coli CREC UTI - model 23	0.00	0.00	0.00	2.66E-05	7.59E-05	1.70E-04	4.06	6.75	9.59	0.02	0.04	0.06	0.00	0.00	0.00	0.00	0.00	0.00
Netherlands - Escherichia coli ColREC BSI - model 26	1.96	2.40	2.87	2.06	2.50	2.99	32.74	35.63	38.83	0.19	0.21	0.23	17.62	21.50	25.72	0.10	0.13	0.15
Netherlands - Escherichia coli ColREC OTH - model 30	0.00	0.00	0.00	2.91E-04	4.20E-04	5.99E-04	16.61	20.40	23.91	0.10	0.12	0.14	0.00	0.00	0.00	0.00	0.00	0.00
Netherlands - Escherichia coli ColREC RESP - model 27	0.06	0.07	0.09	0.09	0.11	0.13	14.54	17.83	21.55	0.09	0.11	0.13	0.51	0.64	0.78	3.05E-03	3.79E-03	4.63E-03
Netherlands - Escherichia coli ColREC SSI - model 29	0.09	0.11	0.13	0.09	0.11	0.13	35.45	42.00	48.64	0.21	0.25	0.29	0.99	1.20	1.42	5.86E-03	7.12E-03	8.42E-03
Netherlands - Escherichia coli ColREC UTI - model 28	0.00	0.00	0.00	1.33E-03	1.83E-03	2.40E-03	130.08	149.18	169.75	0.77	0.88	1.00	0.00	0.00	0.00	0.00	0.00	0.00
Netherlands - Klebsiella pneumoniae 3GCRKP BSI - model 1	2.16	2.29	2.43	2.51	2.66	2.82	109.61	116.14	122.60	0.65	0.69	0.73	18.08	19.42	20.71	0.11	0.11	0.12
Netherlands - Klebsiella pneumoniae 3GCRKP OTH - model 5	0.00	0.00	0.00	6.79E-04	9.08E-04	1.16E-03	39.29	45.83	52.76	0.23	0.27	0.31	0.00	0.00	0.00	0.00	0.00	0.00
Netherlands - Klebsiella pneumoniae 3GCRKP RESP - model 2	0.38	0.43	0.47	0.58	0.65	0.72	89.13	100.41	111.56	0.53	0.59	0.66	3.14	3.62	4.06	0.02	0.02	0.02
Netherlands - Klebsiella pneumoniae 3GCRKP SSI - model 4	0.11	0.12	0.14	0.11	0.12	0.14	39.36	45.60	52.01	0.23	0.27	0.31	1.13	1.36	1.57	6.68E-03	8.03E-03	9.29E-03
Netherlands - Klebsiella pneumoniae 3GCRKP UTI - model 3	0.00	0.00	0.00	1.23E-03	1.58E-03	1.98E-03	121.63	136.36	151.25	0.72	0.81	0.89	0.00	0.00	0.00	0.00	0.00	0.00
Netherlands - Klebsiella pneumoniae CRKP BSI - model 6	0.00	0.00	0.00	0.00	0.00	0.00	0.00	0.00	0.00	0.00	0.00	0.00	0.00	0.00	0.00	0.00	0.00	0.00
Netherlands - Klebsiella pneumoniae CRKP OTH - model 10	0.00	0.00	0.00	0.00	0.00	0.00	0.00	0.00	0.00	0.00	0.00	0.00	0.00	0.00	0.00	0.00	0.00	0.00
Netherlands - Klebsiella pneumoniae CRKP RESP - model 7	0.00	0.00	0.00	0.00	0.00	0.00	0.00	0.00	0.00	0.00	0.00	0.00	0.00	0.00	0.00	0.00	0.00	0.00
Netherlands - Klebsiella pneumoniae CRKP SSI - model 9	0.00	0.00	0.00	0.00	0.00	0.00	0.00	0.00	0.00	0.00	0.00	0.00	0.00	0.00	0.00	0.00	0.00	0.00
Netherlands - Klebsiella pneumoniae CRKP UTI - model 8	0.00	0.00	0.00	0.00	0.00	0.00	0.00	0.00	0.00	0.00	0.00	0.00	0.00	0.00	0.00	0.00	0.00	0.00
Netherlands - Klebsiella pneumoniae ColRKP BSI - model 11	0.52	0.71	0.93	0.55	0.74	0.97	8.35	9.67	11.15	0.05	0.06	0.07	4.31	5.80	7.55	0.03	0.03	0.04
Netherlands - Klebsiella pneumoniae ColRKP OTH - model 15	0.00	0.00	0.00	4.52E-05	9.13E-05	1.62E-04	2.89	4.58	6.03	0.02	0.03	0.04	0.00	0.00	0.00	0.00	0.00	0.00
Netherlands - Klebsiella pneumoniae ColRKP RESP - model 12	0.01	0.02	0.02	0.02	0.03	0.04	2.64	4.09	5.49	0.02	0.02	0.03	0.09	0.15	0.20	5.61E-04	8.68E-04	1.19E-03
Netherlands - Klebsiella pneumoniae ColRKP SSI - model 14	4.80E-03	8.44E-03	0.01	4.85E-03	8.48E-03	0.01	1.91	2.86	3.89	0.01	0.02	0.02	0.05	0.08	0.11	2.75E-04	4.59E-04	6.45E-04
Netherlands - Klebsiella pneumoniae ColRKP UTI - model 13	0.00	0.00	0.00	4.09E-05	7.18E-05	1.17E-04	4.00	5.89	7.78	0.02	0.03	0.05	0.00	0.00	0.00	0.00	0.00	0.00
Netherlands - Pseudomonas aeruginosa CRPA BSI - model 51	0.76	1.06	1.39	0.88	1.18	1.52	29.24	33.03	37.15	0.17	0.20	0.22	5.49	7.69	10.04	0.03	0.05	0.06
Netherlands - Pseudomonas aeruginosa CRPA OTH - model 55	0.00	0.00	0.00	2.46E-04	3.61E-04	5.12E-04	12.76	16.70	20.69	0.08	0.10	0.12	0.00	0.00	0.00	0.00	0.00	0.00
Netherlands - Pseudomonas aeruginosa CRPA RESP - model 52	0.80	1.11	1.47	1.08	1.43	1.81	101.61	121.58	142.81	0.60	0.72	0.85	5.79	8.01	10.70	0.03	0.05	0.06
Netherlands - Pseudomonas aeruginosa CRPA SSI - model 54	0.04	0.06	0.08	0.04	0.06	0.08	17.17	22.60	27.84	0.10	0.13	0.16	0.42	0.60	0.78	2.49E-03	3.55E-03	4.60E-03
Netherlands - Pseudomonas aeruginosa CRPA UTI - model 53	0.00	0.00	0.00	3.53E-04	5.10E-04	7.17E-04	33.31	42.45	51.32	0.20	0.25	0.30	0.00	0.00	0.00	0.00	0.00	0.00
Netherlands - Pseudomonas aeruginosa ColRPA BSI - model 56	0.02	0.05	0.08	0.03	0.05	0.09	2.54	3.30	4.15	0.02	0.02	0.02	0.36	0.76	1.24	2.14E-03	4.49E-03	7.35E-03
Netherlands - Pseudomonas aeruginosa ColRPA OTH - model 60	0.00	0.00	0.00	1.47E-05	3.62E-05	7.17E-05	0.87	1.69	2.52	5.14E-03	0.01	0.01	0.00	0.00	0.00	0.00	0.00	0.00
Netherlands - Pseudomonas aeruginosa ColRPA RESP - model 57	0.02	0.05	0.09	0.03	0.06	0.11	8.00	12.12	16.13	0.05	0.07	0.10	0.38	0.78	1.28	2.22E-03	4.63E-03	7.58E-03
Netherlands - Pseudomonas aeruginosa ColRPA SSI - model 59	2.41E-03	5.29E-03	8.03E-03	2.43E-03	5.33E-03	8.08E-03	1.17	2.28	3.43	6.95E-03	0.01	0.02	0.04	0.08	0.12	2.50E-04	4.86E-04	7.31E-04
Netherlands - Pseudomonas aeruginosa ColRPA UTI - model 58	0.00	0.00	0.00	2.08E-05	4.94E-05	9.18E-05	2.34	4.28	6.06	0.01	0.03	0.04	0.00	0.00	0.00	0.00	0.00	0.00
Netherlands - Pseudomonas aeruginosa MDRPA BSI - model 46	0.13	0.21	0.31	0.16	0.24	0.33	8.55	9.91	11.32	0.05	0.06	0.07	1.39	2.11	2.80	8.25E-03	0.01	0.02
Netherlands - Pseudomonas aeruginosa MDRPA OTH - model 50	0.00	0.00	0.00	6.82E-05	1.10E-04	1.66E-04	3.63	5.06	6.51	0.02	0.03	0.04	0.00	0.00	0.00	0.00	0.00	0.00
Netherlands - Pseudomonas aeruginosa MDRPA RESP - model 47	0.15	0.23	0.33	0.21	0.30	0.41	29.18	36.52	43.69	0.17	0.22	0.26	1.56	2.26	3.14	9.23E-03	0.01	0.02
Netherlands - Pseudomonas aeruginosa MDRPA SSI - model 49	0.01	0.02	0.02	0.01	0.02	0.02	4.84	6.76	8.64	0.03	0.04	0.05	0.15	0.21	0.28	8.78E-04	1.26E-03	1.64E-03
Netherlands - Pseudomonas aeruginosa MDRPA UTI - model 48	0.00	0.00	0.00	9.70E-05	1.54E-04	2.31E-04	9.45	12.80	16.09	0.06	0.08	0.10	0.00	0.00	0.00	0.00	0.00	0.00
Netherlands - Staphylococcus aureus MRSA BSI - model 61	1.22	1.31	1.42	1.40	1.51	1.62	40.15	42.49	45.04	0.24	0.25	0.27	7.11	7.63	8.19	0.04	0.05	0.05
Netherlands - Staphylococcus aureus MRSA OTH - model 65	0.00	0.00	0.00	9.27E-04	1.21E-03	1.54E-03	56.04	62.60	68.83	0.33	0.37	0.41	0.00	0.00	0.00	0.00	0.00	0.00
Netherlands - Staphylococcus aureus MRSA RESP - model 62	0.35	0.39	0.46	0.53	0.60	0.68	57.53	63.79	70.64	0.34	0.38	0.42	2.04	2.30	2.58	0.01	0.01	0.02
Netherlands - Staphylococcus aureus MRSA SSI - model 64	0.18	0.20	0.22	0.18	0.20	0.22	63.05	69.82	76.47	0.37	0.41	0.45	1.41	1.60	1.79	8.36E-03	9.49E-03	0.01
Netherlands - Staphylococcus aureus MRSA UTI - model 63	0.00	0.00	0.00	8.32E-05	1.13E-04	1.56E-04	8.41	10.32	12.32	0.05	0.06	0.07	0.00	0.00	0.00	0.00	0.00	0.00
Netherlands - Streptococcus pneumoniae PMRSP BSI - model 76	0.16	0.18	0.19	0.19	0.20	0.22	6.16	6.16	6.16	0.04	0.04	0.04	1.01	1.11	1.20	6.00E-03	6.56E-03	7.09E-03
Netherlands - Streptococcus pneumoniae PMRSP OTH - model 80	0.00	0.00	0.00	1.40E-05	2.50E-05	4.02E-05	1.91	2.18	2.58	0.01	0.01	0.02	0.00	0.00	0.00	0.00	0.00	0.00
Netherlands - Streptococcus pneumoniae PMRSP RESP - model 77	0.10	0.13	0.19	0.16	0.20	0.27	18.45	22.98	30.66	0.11	0.14	0.18	0.65	0.83	1.15	3.85E-03	4.89E-03	6.81E-03
Netherlands - Streptococcus pneumoniae PMRSP SSI - model 79	0.00	0.00	0.00	0.00	0.00	0.00	0.00	0.00	0.00	0.00	0.00	0.00	0.00	0.00	0.00	0.00	0.00	0.00
Netherlands - Streptococcus pneumoniae PMRSP UTI - model 78	0.00	0.00	0.00	0.00	0.00	0.00	0.00	0.00	0.00	0.00	0.00	0.00	0.00	0.00	0.00	0.00	0.00	0.00
Netherlands - Streptococcus pneumoniae PRSP BSI - model 71	0.07	0.08	0.08	0.08	0.09	0.09	4.62	4.62	4.62	0.03	0.03	0.03	0.77	0.83	0.90	4.54E-03	4.92E-03	5.33E-03
Netherlands - Streptococcus pneumoniae PRSP OTH - model 75	0.00	0.00	0.00	1.07E-05	1.91E-05	2.97E-05	1.44	1.64	1.91	8.50E-03	9.72E-03	0.01	0.00	0.00	0.00	0.00	0.00	0.00
Netherlands - Streptococcus pneumoniae PRSP RESP - model 72	0.04	0.06	0.08	0.07	0.09	0.12	13.89	17.29	22.53	0.08	0.10	0.13	0.49	0.63	0.83	2.89E-03	3.70E-03	4.91E-03
Netherlands - Streptococcus pneumoniae PRSP SSI - model 74	0.00	0.00	0.00	0.00	0.00	0.00	0.00	0.00	0.00	0.00	0.00	0.00	0.00	0.00	0.00	0.00	0.00	0.00
Netherlands - Streptococcus pneumoniae PRSP UTI - model 73	0.00	0.00	0.00	0.00	0.00	0.00	0.00	0.00	0.00	0.00	0.00	0.00	0.00	0.00	0.00	0.00	0.00	0.00
**Total (Median & 95% UIs)**	21.10	24.87	29.17	24.81	28.98	33.70	4,368.31	4,981.61	5,616.15	25.85	29.48	33.23	172.75	205.87	241.72	1.02	1.22	1.43
**Total (average)**		**24.94**			**29.07**			**4,982.94**			**29.48**			**206.31**			**1.22**	

Infection	YLD			YLL			DALY			DALY per Case			YLD per 100,000		
Colistin resistant *Acinetobacter spp.*	0.00	0.00	0.00	0.00	0.00	0.00	0.00	0.00	0.00	0.00	0.00	0.00	0.00	0.00	0.00
Carbapenem resistant *Acinetobacter spp.* (excluding those resistant to colistin)	3.33	4.40	5.77	14.13	23.99	35.88	17.99	28.32	40.67	6.33	9.43	12.63	0.06	0.09	0.11
Aminoglycosides and fluoroquinolones resistant *Acinetobacter* spp. (excluding those resistant to colistin and/or carbapenem)	0.00	0.00	0.00	0.00	0.00	0.00	0.00	0.00	0.00	0.00	0.00	0.00	0.00	0.00	0.00
Vancomycin resistant Enterococci (*E. faecalis* and *E. faecium*)	0.00	0.00	0.00	0.00	0.00	0.00	0.00	0.00	0.00	0.00	0.00	0.00	0.00	0.00	0.00
Colistin resistant *Escherichia coli*	0.00	0.00	0.00	0.00	0.00	0.00	0.00	0.00	0.00	0.00	0.00	0.00	0.00	0.00	0.00
Carbapenem resistant *Escherichia coli* (excluding those resistant to colistin)	0.22	0.38	0.59	2.09	3.99	6.52	2.38	4.39	7.01	3.00	4.48	5.87	0.00	0.01	0.01
Third-generation cephalosporin resistant *Escherichia coli* (excluding those resistant to colistin and/or carbapenem)	136.48	153.20	170.25	810.20	907.38	1,015.70	949.98	1,061.23	1,184.78	5.26	5.72	6.20	2.64	2.97	3.30
Colistin resistant *Klebsiella pneumoniae*	0.00	0.00	0.00	0.00	0.00	0.00	0.00	0.00	0.00	0.00	0.00	0.00	0.00	0.00	0.00
Carbapenem resistant *Klebsiella pneumoniae* (excluding those resistant to colistin)	0.92	1.59	2.56	8.81	17.21	28.31	10.03	18.79	30.31	12.70	19.73	26.64	0.02	0.03	0.05
Third-generation cephalosporin resistant *Klebsiella pneumoniae* (excluding those resistant to colistin and/or carbapenem)	27.03	30.31	33.80	134.00	146.11	158.66	161.96	176.54	190.84	5.19	5.52	5.85	0.52	0.59	0.65
Colistin resistant *Pseudomonas aeruginosa*	0.31	0.56	0.89	0.92	2.55	5.17	1.30	3.11	5.94	0.97	1.92	2.86	0.01	0.01	0.02
Carbapenem resistant *Pseudomonas aeruginosa* (excluding those resistant to colistin)	24.24	29.33	34.98	106.60	148.64	194.84	132.80	177.90	226.17	6.14	7.92	9.86	0.47	0.57	0.68
Pseudomonas aeruginosa resistant to three or more antimicrobial groups (excluding those resistant to colistin and/or carbapenem)	0.00	0.00	0.00	0.00	0.00	0.00	0.00	0.00	0.00	0.00	0.00	0.00	0.00	0.00	0.00
Methicillin resistant *Staphylococcus aureus*	21.44	24.60	28.22	106.47	117.73	130.18	128.90	142.36	156.87	6.66	7.20	7.77	0.41	0.48	0.55
Penicillin and macrolides resistant *Streptococcus pneumoniae* (excluding those only resistant to penicillin)	0.00	0.00	0.00	0.00	0.00	0.00	0.00	0.00	0.00	0.00	0.00	0.00	0.00	0.00	0.00
Penicillin tesistant *Streptococcus pneumoniae* (excluding those resistant to macrolides)	19.17	23.04	28.39	67.53	76.26	88.07	88.12	99.35	115.47	8.34	8.98	9.72	0.37	0.45	0.55
Total	233.15	267.41	305.44	1,250.75	1,443.85	1,663.33	1,493.47	1,711.97	1,958.06				4.51	5.18	5.91

Infection	YLL per 100,000			DALY per 100,000			Cases per year			Incidence per 100,000			Deaths per year			Mortality per 100,000		
Colistin resistant *Acinetobacter spp.*	0.00	0.00	0.00	0.00	0.00	0.00	0.00	0.00	0.00	0.00	0.00	0.00	0.00	0.00	0.00	0.00	0.00	0.00
Carbapenem resistant *Acinetobacter spp.* (excluding those resistant to colistin)	0.27	0.46	0.69	0.35	0.55	0.79	6.84	8.97	11.27	0.13	0.17	0.22	0.48	0.79	1.14	0.01	0.02	0.02
Aminoglycosides and fluoroquinolones resistant *Acinetobacter* spp. (excluding those resistant to colistin and/or carbapenem)	0.00	0.00	0.00	0.00	0.00	0.00	0.00	0.00	0.00	0.00	0.00	0.00	0.00	0.00	0.00	0.00	0.00	0.00
Vancomycin resistant Enterococci (*E. faecalis* and *E. faecium*)	0.00	0.00	0.00	0.00	0.00	0.00	0.00	0.00	0.00	0.00	0.00	0.00	0.00	0.00	0.00	0.00	0.00	0.00
Colistin resistant *Escherichia coli*	0.00	0.00	0.00	0.00	0.00	0.00	0.00	0.00	0.00	0.00	0.00	0.00	0.00	0.00	0.00	0.00	0.00	0.00
Carbapenem resistant *Escherichia coli* (excluding those resistant to colistin)	0.04	0.08	0.13	0.05	0.08	0.14	4.58	7.82	11.19	0.09	0.15	0.22	0.23	0.43	0.70	0.00	0.01	0.01
Third-generation cephalosporin resistant *Escherichia coli* (excluding those resistant to colistin and/or carbapenem)	15.68	17.56	19.66	18.39	20.54	22.93	1,320.91	1,483.51	1,646.22	25.57	28.71	31.86	39.90	45.27	50.80	0.77	0.88	0.98
Colistin resistant *Klebsiella pneumoniae*	0.00	0.00	0.00	0.00	0.00	0.00	0.00	0.00	0.00	0.00	0.00	0.00	0.00	0.00	0.00	0.00	0.00	0.00
Carbapenem resistant *Klebsiella pneumoniae* (excluding those resistant to colistin)	0.17	0.33	0.55	0.19	0.36	0.59	1.40	2.95	4.60	0.03	0.06	0.09	0.20	0.39	0.64	0.00	0.01	0.01
Third-generation cephalosporin resistant *Klebsiella pneumoniae* (excluding those resistant to colistin and/or carbapenem)	2.59	2.83	3.07	3.13	3.42	3.69	117.75	132.48	147.54	2.28	2.56	2.86	6.63	7.27	7.95	0.13	0.14	0.15
Colistin resistant *Pseudomonas aeruginosa*	0.02	0.05	0.10	0.03	0.06	0.11	3.86	7.66	11.66	0.07	0.15	0.23	0.18	0.51	1.04	0.00	0.01	0.02
Carbapenem resistant *Pseudomonas aeruginosa* (excluding those resistant to colistin)	2.06	2.88	3.77	2.57	3.44	4.38	82.20	100.15	117.47	1.59	1.94	2.27	4.91	6.82	8.98	0.10	0.13	0.17
Pseudomonas aeruginosa resistant to three or more antimicrobial groups (excluding those resistant to colistin and/or carbapenem)	0.00	0.00	0.00	0.00	0.00	0.00	0.00	0.00	0.00	0.00	0.00	0.00	0.00	0.00	0.00	0.00	0.00	0.00
Methicillin resistant *Staphylococcus aureus*	2.06	2.28	2.52	2.49	2.76	3.04	91.68	101.97	112.82	1.77	1.97	2.18	4.38	4.82	5.28	0.08	0.09	0.10
Penicillin and macrolides resistant *Streptococcus pneumoniae* (excluding those only resistant to penicillin)	0.00	0.00	0.00	0.00	0.00	0.00	0.00	0.00	0.00	0.00	0.00	0.00	0.00	0.00	0.00	0.00	0.00	0.00
Penicillin tesistant *Streptococcus pneumoniae* (excluding those resistant to macrolides)	1.31	1.48	1.70	1.71	1.92	2.24	31.59	36.05	42.19	0.61	0.70	0.82	1.98	2.22	2.53	0.04	0.04	0.05
Total	24.21	27.95	32.19	28.91	33.14	37.90	1,660.81	1,881.57	2,104.97	32.15	36.42	40.74	58.87	68.53	79.07	1.14	1.33	1.53

Model	YLD			YLL			DALY			DALY per Case			YLD per 100,000		
Norway - Acinetobacter spp. CRACI BSI - model 36	2.00	2.53	3.24	11.04	19.63	30.02	13.52	22.11	32.43	4.35	7.14	9.98	0.04	0.05	0.06
Norway - Acinetobacter spp. CRACI OTH - model 40	1.61E-04	4.08E-04	7.75E-04	0.00	0.00	0.00	1.61E-04	4.08E-04	7.75E-04	2.26E-03	3.73E-03	5.40E-03	3.12E-06	7.90E-06	1.50E-05
Norway - Acinetobacter spp. CRACI RESP - model 37	1.32	1.87	2.52	2.62	3.66	4.91	4.00	5.51	7.29	1.40	1.66	1.97	0.03	0.04	0.05
Norway - Acinetobacter spp. CRACI SSI - model 39	1.00E-03	2.32E-03	4.39E-03	0.47	0.70	0.95	0.47	0.70	0.95	0.58	0.62	0.66	1.94E-05	4.50E-05	8.50E-05
Norway - Acinetobacter spp. CRACI UTI - model 38	1.23E-03	2.49E-03	4.14E-03	0.00	0.00	0.00	1.23E-03	2.49E-03	4.14E-03	1.18E-03	2.01E-03	3.05E-03	2.38E-05	4.82E-05	8.02E-05
Norway - Acinetobacter spp. CoRACI BSI - model 41	0.00	0.00	0.00	0.00	0.00	0.00	0.00	0.00	0.00	NaN	NaN	NaN	0.00	0.00	0.00
Norway - Acinetobacter spp. CoRACI OTH - model 45	0.00	0.00	0.00	0.00	0.00	0.00	0.00	0.00	0.00	NaN	NaN	NaN	0.00	0.00	0.00
Norway - Acinetobacter spp. CoRACI RESP - model 42	0.00	0.00	0.00	0.00	0.00	0.00	0.00	0.00	0.00	NaN	NaN	NaN	0.00	0.00	0.00
Norway - Acinetobacter spp. CoRACI SSI - model 44	0.00	0.00	0.00	0.00	0.00	0.00	0.00	0.00	0.00	NaN	NaN	NaN	0.00	0.00	0.00
Norway - Acinetobacter spp. CoRACI UTI - model 43	0.00	0.00	0.00	0.00	0.00	0.00	0.00	0.00	0.00	NaN	NaN	NaN	0.00	0.00	0.00
Norway - Acinetobacter spp. MDRACI BSI - model 31	0.00	0.00	0.00	0.00	0.00	0.00	0.00	0.00	0.00	NaN	NaN	NaN	0.00	0.00	0.00
Norway - Acinetobacter spp. MDRACI OTH - model 35	0.00	0.00	0.00	0.00	0.00	0.00	0.00	0.00	0.00	NaN	NaN	NaN	0.00	0.00	0.00
Norway - Acinetobacter spp. MDRACI RESP - model 32	0.00	0.00	0.00	0.00	0.00	0.00	0.00	0.00	0.00	NaN	NaN	NaN	0.00	0.00	0.00
Norway - Acinetobacter spp. MDRACI SSI - model 34	0.00	0.00	0.00	0.00	0.00	0.00	0.00	0.00	0.00	NaN	NaN	NaN	0.00	0.00	0.00
Norway - Acinetobacter spp. MDRACI UTI - model 33	0.00	0.00	0.00	0.00	0.00	0.00	0.00	0.00	0.00	NaN	NaN	NaN	0.00	0.00	0.00
Norway - Enterococcus faecalis and E. faecium VRE BSI - model 66	0.00	0.00	0.00	0.00	0.00	0.00	0.00	0.00	0.00	NaN	NaN	NaN	0.00	0.00	0.00
Norway - Enterococcus faecalis and E. faecium VRE OTH - model 70	0.00	0.00	0.00	0.00	0.00	0.00	0.00	0.00	0.00	NaN	NaN	NaN	0.00	0.00	0.00
Norway - Enterococcus faecalis and E. faecium VRE RESP - model 67	0.00	0.00	0.00	0.00	0.00	0.00	0.00	0.00	0.00	NaN	NaN	NaN	0.00	0.00	0.00
Norway - Enterococcus faecalis and E. faecium VRE SSI - model 69	0.00	0.00	0.00	0.00	0.00	0.00	0.00	0.00	0.00	NaN	NaN	NaN	0.00	0.00	0.00
Norway - Enterococcus faecalis and E. faecium VRE UTI - model 68	0.00	0.00	0.00	0.00	0.00	0.00	0.00	0.00	0.00	NaN	NaN	NaN	0.00	0.00	0.00
Norway - Escherichia coli 3GCREC BSI - model 16	105.02	115.38	125.91	672.15	743.98	828.01	780.66	860.08	953.00	3.82	4.14	4.49	2.03	2.23	2.44
Norway - Escherichia coli 3GCREC OTH - model 20	0.17	0.23	0.29	0.00	0.00	0.00	0.17	0.23	0.29	2.72E-03	3.35E-03	4.06E-03	3.35E-03	4.47E-03	5.69E-03
Norway - Escherichia coli 3GCREC RESP - model 17	29.56	35.38	41.30	57.00	68.61	79.13	86.61	103.86	120.05	1.03	1.13	1.24	0.57	0.68	0.80
Norway - Escherichia coli 3GCREC SSI - model 19	0.35	0.47	0.61	81.05	94.79	108.56	81.15	95.32	109.29	0.41	0.43	0.46	6.75E-03	9.12E-03	0.01
Norway - Escherichia coli 3GCREC UTI - model 18	1.38	1.74	2.14	0.00	0.00	0.00	1.38	1.74	2.14	1.59E-03	1.95E-03	2.34E-03	0.03	0.03	0.04
Norway - Escherichia coli CREC BSI - model 21	0.18	0.28	0.40	1.80	3.40	5.62	2.04	3.68	5.92	2.22	3.62	4.94	3.57E-03	5.35E-03	7.80E-03
Norway - Escherichia coli CREC OTH - model 25	6.15E-04	1.87E-03	4.91E-03	0.00	0.00	0.00	6.15E-04	1.87E-03	4.91E-03	1.23E-03	3.29E-03	7.28E-03	1.19E-05	3.61E-05	9.51E-05
Norway - Escherichia coli CREC RESP - model 22	0.04	0.09	0.16	0.07	0.18	0.30	0.11	0.27	0.45	0.44	0.52	0.58	7.06E-04	1.83E-03	3.11E-03
Norway - Escherichia coli CREC SSI - model 24	5.82E-04	2.34E-03	6.38E-03	0.23	0.42	0.61	0.23	0.42	0.61	0.34	0.34	0.34	1.13E-05	4.54E-05	1.24E-04
Norway - Escherichia coli CREC UTI - model 23	2.83E-03	8.57E-03	0.02	0.00	0.00	0.00	2.83E-03	8.57E-03	0.02	7.49E-04	1.99E-03	4.04E-03	5.49E-05	1.66E-04	3.83E-04
Norway - Escherichia coli CoREC BSI - model 26	0.00	0.00	0.00	0.00	0.00	0.00	0.00	0.00	0.00	NaN	NaN	NaN	0.00	0.00	0.00
Norway - Escherichia coli CoREC OTH - model 30	0.00	0.00	0.00	0.00	0.00	0.00	0.00	0.00	0.00	NaN	NaN	NaN	0.00	0.00	0.00
Norway - Escherichia coli CoREC RESP - model 27	0.00	0.00	0.00	0.00	0.00	0.00	0.00	0.00	0.00	NaN	NaN	NaN	0.00	0.00	0.00
Norway - Escherichia coli CoREC SSI - model 29	0.00	0.00	0.00	0.00	0.00	0.00	0.00	0.00	0.00	NaN	NaN	NaN	0.00	0.00	0.00
Norway - Escherichia coli CoREC UTI - model 28	0.00	0.00	0.00	0.00	0.00	0.00	0.00	0.00	0.00	NaN	NaN	NaN	0.00	0.00	0.00
Norway - Klebsiella pneumoniae 3GCRKP BSI - model 1	17.04	18.80	20.85	109.61	118.10	127.04	127.53	136.97	146.63	3.73	3.95	4.16	0.33	0.36	0.40
Norway - Klebsiella pneumoniae 3GCRKP OTH - model 5	0.03	0.05	0.06	0.00	0.00	0.00	0.03	0.05	0.06	2.56E-03	3.34E-03	4.19E-03	6.57E-04	8.80E-04	1.17E-03
Norway - Klebsiella pneumoniae 3GCRKP RESP - model 2	9.88	11.35	12.75	19.26	21.96	24.54	29.18	33.37	36.92	1.03	1.11	1.21	0.19	0.22	0.25
Norway - Klebsiella pneumoniae 3GCRKP SSI - model 4	0.02	0.03	0.04	5.13	6.04	7.09	5.16	6.07	7.12	0.43	0.45	0.47	3.89E-04	5.52E-04	7.73E-04
Norway - Klebsiella pneumoniae 3GCRKP UTI - model 3	0.06	0.08	0.10	0.00	0.00	0.00	0.06	0.08	0.10	1.53E-03	1.98E-03	2.43E-03	1.20E-03	1.55E-03	1.96E-03
Norway - Klebsiella pneumoniae CRKP BSI - model 6	0.82	1.22	1.89	8.56	16.38	26.83	9.66	17.59	28.19	10.24	16.92	23.45	0.02	0.02	0.04
Norway - Klebsiella pneumoniae CRKP OTH - model 10	3.95E-04	1.54E-03	4.34E-03	0.00	0.00	0.00	3.95E-04	1.54E-03	4.34E-03	1.27E-03	3.38E-03	7.39E-03	7.65E-06	2.98E-05	8.40E-05
Norway - Klebsiella pneumoniae CRKP RESP - model 7	0.10	0.36	0.66	0.22	0.71	1.26	0.34	1.07	1.90	2.06	2.41	2.77	2.02E-03	7.05E-03	0.01
Norway - Klebsiella pneumoniae CRKP SSI - model 9	9.89E-05	5.57E-04	1.70E-03	0.03	0.12	0.22	0.04	0.12	0.22	0.40	0.40	0.40	1.91E-06	1.08E-05	3.29E-05
Norway - Klebsiella pneumoniae CRKP UTI - model 8	3.87E-04	1.21E-03	3.29E-03	0.00	0.00	0.00	3.87E-04	1.21E-03	3.29E-03	7.85E-04	2.04E-03	4.04E-03	7.48E-06	2.34E-05	6.36E-05
Norway - Klebsiella pneumoniae CoRKP BSI - model 11	0.00	0.00	0.00	0.00	0.00	0.00	0.00	0.00	0.00	NaN	NaN	NaN	0.00	0.00	0.00
Norway - Klebsiella pneumoniae CoRKP OTH - model 15	0.00	0.00	0.00	0.00	0.00	0.00	0.00	0.00	0.00	NaN	NaN	NaN	0.00	0.00	0.00
Norway - Klebsiella pneumoniae CoRKP RESP - model 12	0.00	0.00	0.00	0.00	0.00	0.00	0.00	0.00	0.00	NaN	NaN	NaN	0.00	0.00	0.00
Norway - Klebsiella pneumoniae CoRKP SSI - model 14	0.00	0.00	0.00	0.00	0.00	0.00	0.00	0.00	0.00	NaN	NaN	NaN	0.00	0.00	0.00
Norway - Klebsiella pneumoniae CoRKP UTI - model 13	0.00	0.00	0.00	0.00	0.00	0.00	0.00	0.00	0.00	NaN	NaN	NaN	0.00	0.00	0.00
Norway - Pseudomonas aeruginosa CRPA BSI - model 51	6.94	8.20	9.60	49.78	69.97	92.79	57.51	78.08	101.52	4.29	5.64	7.12	0.13	0.16	0.19
Norway - Pseudomonas aeruginosa CRPA OTH - model 55	0.02	0.03	0.04	0.00	0.00	0.00	0.02	0.03	0.04	2.81E-03	3.65E-03	4.63E-03	3.39E-04	5.04E-04	6.98E-04
Norway - Pseudomonas aeruginosa CRPA RESP - model 52	17.25	21.04	25.26	54.07	74.84	97.22	72.48	95.91	119.72	1.49	1.86	2.29	0.33	0.41	0.49
Norway - Pseudomonas aeruginosa CRPA SSI - model 54	0.01	0.02	0.03	2.75	3.83	4.83	2.77	3.85	4.84	0.35	0.40	0.45	2.45E-04	3.90E-04	5.93E-04
Norway - Pseudomonas aeruginosa CRPA UTI - model 53	0.03	0.04	0.05	0.00	0.00	0.00	0.03	0.04	0.05	1.56E-03	2.06E-03	2.59E-03	4.85E-04	7.11E-04	9.67E-04
Norway - Pseudomonas aeruginosa CoRPA BSI - model 56	0.10	0.15	0.24	0.40	1.21	2.37	0.54	1.37	2.57	0.54	1.31	2.05	1.93E-03	2.95E-03	4.61E-03
Norway - Pseudomonas aeruginosa CoRPA OTH - model 60	4.13E-04	1.88E-03	4.73E-03	0.00	0.00	0.00	4.13E-04	1.88E-03	4.73E-03	1.49E-03	3.70E-03	6.50E-03	8.00E-06	3.64E-05	9.16E-05
Norway - Pseudomonas aeruginosa CoRPA RESP - model 57	0.21	0.40	0.63	0.47	1.21	2.58	0.72	1.60	3.14	0.25	0.43	0.62	3.97E-03	7.79E-03	0.01
Norway - Pseudomonas aeruginosa CoRPA SSI - model 59	2.51E-04	1.39E-03	4.07E-03	0.04	0.13	0.22	0.05	0.14	0.22	0.18	0.18	0.19	4.86E-06	2.69E-05	7.88E-05
Norway - Pseudomonas aeruginosa CoRPA UTI - model 58	7.43E-04	2.60E-03	6.30E-03	0.00	0.00	0.00	7.43E-04	2.60E-03	6.30E-03	8.10E-04	1.97E-03	3.70E-03	1.44E-05	5.03E-05	1.22E-04
Norway - Pseudomonas aeruginosa MDRPA BSI - model 46	0.00	0.00	0.00	0.00	0.00	0.00	0.00	0.00	0.00	NaN	NaN	NaN	0.00	0.00	0.00
Norway - Pseudomonas aeruginosa MDRPA OTH - model 50	0.00	0.00	0.00	0.00	0.00	0.00	0.00	0.00	0.00	NaN	NaN	NaN	0.00	0.00	0.00
Norway - Pseudomonas aeruginosa MDRPA RESP - model 47	0.00	0.00	0.00	0.00	0.00	0.00	0.00	0.00	0.00	NaN	NaN	NaN	0.00	0.00	0.00
Norway - Pseudomonas aeruginosa MDRPA SSI - model 49	0.00	0.00	0.00	0.00	0.00	0.00	0.00	0.00	0.00	NaN	NaN	NaN	0.00	0.00	0.00
Norway - Pseudomonas aeruginosa MDRPA UTI - model 48	0.00	0.00	0.00	0.00	0.00	0.00	0.00	0.00	0.00	NaN	NaN	NaN	0.00	0.00	0.00
Norway - Staphylococcus aureus MRSA BSI - model 61	10.57	11.99	13.46	74.36	80.93	88.43	85.65	92.95	100.81	4.97	5.35	5.74	0.20	0.23	0.26
Norway - Staphylococcus aureus MRSA OTH - model 65	0.06	0.08	0.10	0.00	0.00	0.00	0.06	0.08	0.10	2.55E-03	3.23E-03	4.03E-03	1.23E-03	1.61E-03	2.03E-03
Norway - Staphylococcus aureus MRSA RESP - model 62	10.75	12.46	14.57	20.98	24.34	27.91	31.99	36.80	42.05	1.27	1.41	1.56	0.21	0.24	0.28
Norway - Staphylococcus aureus MRSA SSI - model 64	0.04	0.06	0.08	11.13	12.45	13.84	11.19	12.51	13.90	0.41	0.44	0.46	8.24E-04	1.19E-03	1.57E-03
Norway - Staphylococcus aureus MRSA UTI - model 63	5.66E-03	7.97E-03	0.01	0.00	0.00	0.00	5.66E-03	7.97E-03	0.01	1.44E-03	1.89E-03	2.38E-03	1.10E-04	1.54E-04	2.11E-04
Norway - Streptococcus pneumoniae PMRSP BSI - model 76	0.00	0.00	0.00	0.00	0.00	0.00	0.00	0.00	0.00	NaN	NaN	NaN	0.00	0.00	0.00
Norway - Streptococcus pneumoniae PMRSP OTH - model 80	0.00	0.00	0.00	0.00	0.00	0.00	0.00	0.00	0.00	NaN	NaN	NaN	0.00	0.00	0.00
Norway - Streptococcus pneumoniae PMRSP RESP - model 77	0.00	0.00	0.00	0.00	0.00	0.00	0.00	0.00	0.00	NaN	NaN	NaN	0.00	0.00	0.00
Norway - Streptococcus pneumoniae PMRSP SSI - model 79	0.00	0.00	0.00	0.00	0.00	0.00	0.00	0.00	0.00	NaN	NaN	NaN	0.00	0.00	0.00
Norway - Streptococcus pneumoniae PMRSP UTI - model 78	0.00	0.00	0.00	0.00	0.00	0.00	0.00	0.00	0.00	NaN	NaN	NaN	0.00	0.00	0.00
Norway - Streptococcus pneumoniae PRSP BSI - model 71	5.61	6.39	7.32	40.60	43.50	46.47	46.91	49.85	53.05	6.70	7.12	7.58	0.11	0.12	0.14
Norway - Streptococcus pneumoniae PRSP OTH - model 75	3.32E-03	4.87E-03	6.64E-03	0.00	0.00	0.00	3.32E-03	4.87E-03	6.64E-03	1.37E-03	1.95E-03	2.61E-03	6.43E-05	9.42E-05	1.29E-04
Norway - Streptococcus pneumoniae PRSP RESP - model 72	13.56	16.64	21.06	26.93	32.76	41.60	41.21	49.50	62.42	1.63	1.86	2.14	0.26	0.32	0.41
Norway - Streptococcus pneumoniae PRSP SSI - model 74	0.00	0.00	0.00	0.00	0.00	0.00	0.00	0.00	0.00	NaN	NaN	NaN	0.00	0.00	0.00
Norway - Streptococcus pneumoniae PRSP UTI - model 73	0.00	0.00	0.00	0.00	0.00	0.00	0.00	0.00	0.00	NaN	NaN	NaN	0.00	0.00	0.00
**Total (Median & 95% UIs)**	233.15	267.41	305.44	1,250.75	1,443.85	1,663.33	1,493.47	1,711.97	1,958.06		-		4.51	5.18	5.91
**Total (average)**	**268.11**			**1,448.21**			**1,716.32**			-			**5.19**		

Model	YLL per 100,000			DALY per 100,000			Cases per year			Incidence per 100,000			Deaths per year			Mortality per 100,000		
Norway - Acinetobacter spp. CRACI BSI - model 36	0.21	0.38	0.58	0.26	0.43	0.63	2.71	3.12	3.57	0.05	0.06	0.07	0.37	0.64	0.95	7.15E-03	0.01	0.02
Norway - Acinetobacter spp. CRACI OTH - model 40	0.00	0.00	0.00	3.12E-06	7.90E-06	1.50E-05	0.05	0.11	0.18	9.92E-04	2.20E-03	3.57E-03	0.00	0.00	0.00	0.00	0.00	0.00
Norway - Acinetobacter spp. CRACI RESP - model 37	0.05	0.07	0.10	0.08	0.11	0.14	2.52	3.35	4.20	0.05	0.06	0.08	0.09	0.12	0.15	1.71E-03	2.33E-03	3.00E-03
Norway - Acinetobacter spp. CRACI SSI - model 39	9.10E-03	0.01	0.02	9.12E-03	0.01	0.02	0.76	1.13	1.58	0.01	0.02	0.03	0.02	0.03	0.04	3.84E-04	5.92E-04	8.31E-04
Norway - Acinetobacter spp. CRACI UTI - model 38	0.00	0.00	0.00	2.38E-05	4.82E-05	8.02E-05	0.80	1.25	1.74	0.02	0.02	0.03	0.00	0.00	0.00	0.00	0.00	0.00
Norway - Acinetobacter spp. CoRACI BSI - model 41	0.00	0.00	0.00	0.00	0.00	0.00	0.00	0.00	0.00	0.00	0.00	0.00	0.00	0.00	0.00	0.00	0.00	0.00
Norway - Acinetobacter spp. CoRACI OTH - model 45	0.00	0.00	0.00	0.00	0.00	0.00	0.00	0.00	0.00	0.00	0.00	0.00	0.00	0.00	0.00	0.00	0.00	0.00
Norway - Acinetobacter spp. CoRACI RESP - model 42	0.00	0.00	0.00	0.00	0.00	0.00	0.00	0.00	0.00	0.00	0.00	0.00	0.00	0.00	0.00	0.00	0.00	0.00
Norway - Acinetobacter spp. CoRACI SSI - model 44	0.00	0.00	0.00	0.00	0.00	0.00	0.00	0.00	0.00	0.00	0.00	0.00	0.00	0.00	0.00	0.00	0.00	0.00
Norway - Acinetobacter spp. CoRACI UTI - model 43	0.00	0.00	0.00	0.00	0.00	0.00	0.00	0.00	0.00	0.00	0.00	0.00	0.00	0.00	0.00	0.00	0.00	0.00
Norway - Acinetobacter spp. MDRACI BSI - model 31	0.00	0.00	0.00	0.00	0.00	0.00	0.00	0.00	0.00	0.00	0.00	0.00	0.00	0.00	0.00	0.00	0.00	0.00
Norway - Acinetobacter spp. MDRACI OTH - model 35	0.00	0.00	0.00	0.00	0.00	0.00	0.00	0.00	0.00	0.00	0.00	0.00	0.00	0.00	0.00	0.00	0.00	0.00
Norway - Acinetobacter spp. MDRACI RESP - model 32	0.00	0.00	0.00	0.00	0.00	0.00	0.00	0.00	0.00	0.00	0.00	0.00	0.00	0.00	0.00	0.00	0.00	0.00
Norway - Acinetobacter spp. MDRACI SSI - model 34	0.00	0.00	0.00	0.00	0.00	0.00	0.00	0.00	0.00	0.00	0.00	0.00	0.00	0.00	0.00	0.00	0.00	0.00
Norway - Acinetobacter spp. MDRACI UTI - model 33	0.00	0.00	0.00	0.00	0.00	0.00	0.00	0.00	0.00	0.00	0.00	0.00	0.00	0.00	0.00	0.00	0.00	0.00
Norway - Enterococcus faecalis and E. faecium VRE BSI - model 66	0.00	0.00	0.00	0.00	0.00	0.00	0.00	0.00	0.00	0.00	0.00	0.00	0.00	0.00	0.00	0.00	0.00	0.00
Norway - Enterococcus faecalis and E. faecium VRE OTH - model 70	0.00	0.00	0.00	0.00	0.00	0.00	0.00	0.00	0.00	0.00	0.00	0.00	0.00	0.00	0.00	0.00	0.00	0.00
Norway - Enterococcus faecalis and E. faecium VRE RESP - model 67	0.00	0.00	0.00	0.00	0.00	0.00	0.00	0.00	0.00	0.00	0.00	0.00	0.00	0.00	0.00	0.00	0.00	0.00
Norway - Enterococcus faecalis and E. faecium VRE SSI - model 69	0.00	0.00	0.00	0.00	0.00	0.00	0.00	0.00	0.00	0.00	0.00	0.00	0.00	0.00	0.00	0.00	0.00	0.00
Norway - Enterococcus faecalis and E. faecium VRE UTI - model 68	0.00	0.00	0.00	0.00	0.00	0.00	0.00	0.00	0.00	0.00	0.00	0.00	0.00	0.00	0.00	0.00	0.00	0.00
Norway - Escherichia coli 3GCREC BSI - model 16	13.01	14.40	16.03	15.11	16.65	18.45	193.50	207.59	221.90	3.75	4.02	4.29	32.02	35.88	39.92	0.62	0.69	0.77
Norway - Escherichia coli 3GCREC OTH - model 20	0.00	0.00	0.00	3.35E-03	4.47E-03	5.69E-03	56.77	68.72	82.33	1.10	1.33	1.59	0.00	0.00	0.00	0.00	0.00	0.00
Norway - Escherichia coli 3GCREC RESP - model 17	1.10	1.33	1.53	1.68	2.01	2.32	75.88	92.15	106.70	1.47	1.78	2.07	2.73	3.31	3.86	0.05	0.06	0.07
Norway - Escherichia coli 3GCREC SSI - model 19	1.57	1.83	2.10	1.57	1.84	2.12	190.68	220.04	247.06	3.69	4.26	4.78	5.15	6.09	7.03	0.10	0.12	0.14
Norway - Escherichia coli 3GCREC UTI - model 18	0.00	0.00	0.00	0.03	0.03	0.04	804.08	895.02	988.24	15.56	17.32	19.13	0.00	0.00	0.00	0.00	0.00	0.00
Norway - Escherichia coli CREC BSI - model 21	0.03	0.07	0.11	0.04	0.07	0.11	0.80	1.03	1.35	0.02	0.02	0.03	0.19	0.37	0.60	3.75E-03	7.09E-03	0.01
Norway - Escherichia coli CREC OTH - model 25	0.00	0.00	0.00	1.19E-05	3.61E-05	9.51E-05	0.29	0.61	0.93	5.52E-03	0.01	0.02	0.00	0.00	0.00	0.00	0.00	0.00
Norway - Escherichia coli CREC RESP - model 22	1.37E-03	3.43E-03	5.81E-03	2.04E-03	5.31E-03	8.76E-03	0.21	0.53	0.86	4.16E-03	0.01	0.02	7.63E-03	0.02	0.03	1.48E-04	3.69E-04	6.25E-04
Norway - Escherichia coli CREC SSI - model 24	4.36E-03	8.09E-03	0.01	4.38E-03	8.14E-03	0.01	0.67	1.25	1.82	0.01	0.02	0.04	0.02	0.04	0.07	4.67E-04	8.68E-04	1.27E-03
Norway - Escherichia coli CREC UTI - model 23	0.00	0.00	0.00	5.49E-05	1.66E-04	3.83E-04	2.61	4.40	6.22	0.05	0.09	0.12	0.00	0.00	0.00	0.00	0.00	0.00
Norway - Escherichia coli CoREC BSI - model 26	0.00	0.00	0.00	0.00	0.00	0.00	0.00	0.00	0.00	0.00	0.00	0.00	0.00	0.00	0.00	0.00	0.00	0.00
Norway - Escherichia coli CoREC OTH - model 30	0.00	0.00	0.00	0.00	0.00	0.00	0.00	0.00	0.00	0.00	0.00	0.00	0.00	0.00	0.00	0.00	0.00	0.00
Norway - Escherichia coli CoREC RESP - model 27	0.00	0.00	0.00	0.00	0.00	0.00	0.00	0.00	0.00	0.00	0.00	0.00	0.00	0.00	0.00	0.00	0.00	0.00
Norway - Escherichia coli CoREC SSI - model 29	0.00	0.00	0.00	0.00	0.00	0.00	0.00	0.00	0.00	0.00	0.00	0.00	0.00	0.00	0.00	0.00	0.00	0.00
Norway - Escherichia coli CoREC UTI - model 28	0.00	0.00	0.00	0.00	0.00	0.00	0.00	0.00	0.00	0.00	0.00	0.00	0.00	0.00	0.00	0.00	0.00	0.00
Norway - Klebsiella pneumoniae 3GCRKP BSI - model 1	2.12	2.29	2.46	2.47	2.65	2.84	32.68	34.70	36.88	0.63	0.67	0.71	5.36	5.79	6.25	0.10	0.11	0.12
Norway - Klebsiella pneumoniae 3GCRKP OTH - model 5	0.00	0.00	0.00	6.57E-04	8.80E-04	1.17E-03	11.69	13.67	15.58	0.23	0.26	0.30	0.00	0.00	0.00	0.00	0.00	0.00
Norway - Klebsiella pneumoniae 3GCRKP RESP - model 2	0.37	0.43	0.47	0.56	0.65	0.71	26.07	29.93	33.52	0.50	0.58	0.65	0.93	1.08	1.22	0.02	0.02	0.02
Norway - Klebsiella pneumoniae 3GCRKP SSI - model 4	0.10	0.12	0.14	0.10	0.12	0.14	11.60	13.56	15.76	0.22	0.26	0.30	0.33	0.40	0.48	6.41E-03	7.78E-03	9.21E-03
Norway - Klebsiella pneumoniae 3GCRKP UTI - model 3	0.00	0.00	0.00	1.20E-03	1.55E-03	1.96E-03	35.71	40.63	45.80	0.69	0.79	0.89	0.00	0.00	0.00	0.00	0.00	0.00
Norway - Klebsiella pneumoniae CRKP BSI - model 6	0.17	0.32	0.52	0.19	0.34	0.55	0.79	1.05	1.43	0.02	0.02	0.03	0.19	0.37	0.61	3.73E-03	7.17E-03	0.01
Norway - Klebsiella pneumoniae CRKP OTH - model 10	0.00	0.00	0.00	7.65E-06	2.98E-05	8.40E-05	0.16	0.50	0.84	3.14E-03	9.70E-03	0.02	0.00	0.00	0.00	0.00	0.00	0.00
Norway - Klebsiella pneumoniae CRKP RESP - model 7	4.17E-03	0.01	0.02	6.51E-03	0.02	0.04	0.13	0.45	0.76	2.61E-03	8.66E-03	0.01	4.87E-03	0.02	0.03	9.43E-05	3.11E-04	5.54E-04
Norway - Klebsiella pneumoniae CRKP SSI - model 9	6.71E-04	2.39E-03	4.17E-03	6.79E-04	2.41E-03	4.21E-03	0.09	0.31	0.54	1.69E-03	6.03E-03	0.01	7.84E-04	2.80E-03	4.87E-03	1.52E-05	5.42E-05	9.43E-05
Norway - Klebsiella pneumoniae CRKP UTI - model 8	0.00	0.00	0.00	7.48E-06	2.34E-05	6.36E-05	0.23	0.64	1.04	4.50E-03	0.01	0.02	0.00	0.00	0.00	0.00	0.00	0.00
Norway - Klebsiella pneumoniae CoRKP BSI - model 11	0.00	0.00	0.00	0.00	0.00	0.00	0.00	0.00	0.00	0.00	0.00	0.00	0.00	0.00	0.00	0.00	0.00	0.00
Norway - Klebsiella pneumoniae CoRKP OTH - model 15	0.00	0.00	0.00	0.00	0.00	0.00	0.00	0.00	0.00	0.00	0.00	0.00	0.00	0.00	0.00	0.00	0.00	0.00
Norway - Klebsiella pneumoniae CoRKP RESP - model 12	0.00	0.00	0.00	0.00	0.00	0.00	0.00	0.00	0.00	0.00	0.00	0.00	0.00	0.00	0.00	0.00	0.00	0.00
Norway - Klebsiella pneumoniae CoRKP SSI - model 14	0.00	0.00	0.00	0.00	0.00	0.00	0.00	0.00	0.00	0.00	0.00	0.00	0.00	0.00	0.00	0.00	0.00	0.00
Norway - Klebsiella pneumoniae CoRKP UTI - model 13	0.00	0.00	0.00	0.00	0.00	0.00	0.00	0.00	0.00	0.00	0.00	0.00	0.00	0.00	0.00	0.00	0.00	0.00
Norway - Pseudomonas aeruginosa CRPA BSI - model 51	0.96	1.35	1.80	1.11	1.51	1.96	12.45	13.91	15.64	0.24	0.27	0.30	2.22	3.20	4.26	0.04	0.06	0.08
Norway - Pseudomonas aeruginosa CRPA OTH - model 55	0.00	0.00	0.00	3.39E-04	5.04E-04	6.98E-04	5.33	7.09	8.79	0.10	0.14	0.17	0.00	0.00	0.00	0.00	0.00	0.00
Norway - Pseudomonas aeruginosa CRPA RESP - model 52	1.05	1.45	1.88	1.40	1.86	2.32	43.33	51.45	59.73	0.84	1.00	1.16	2.51	3.38	4.40	0.05	0.07	0.09
Norway - Pseudomonas aeruginosa CRPA SSI - model 54	0.05	0.07	0.09	0.05	0.07	0.09	7.30	9.63	11.75	0.14	0.19	0.23	0.17	0.25	0.32	3.38E-03	4.75E-03	6.10E-03
Norway - Pseudomonas aeruginosa CRPA UTI - model 53	0.00	0.00	0.00	4.85E-04	7.11E-04	9.67E-04	13.78	18.07	21.56	0.27	0.35	0.42	0.00	0.00	0.00	0.00	0.00	0.00
Norway - Pseudomonas aeruginosa CoRPA BSI - model 56	7.80E-03	0.02	0.05	0.01	0.03	0.05	0.77	1.05	1.46	0.01	0.02	0.03	0.08	0.24	0.47	1.56E-03	4.70E-03	9.16E-03
Norway - Pseudomonas aeruginosa CoRPA OTH - model 60	0.00	0.00	0.00	8.00E-06	3.64E-05	9.16E-05	0.17	0.55	0.91	3.31E-03	0.01	0.02	0.00	0.00	0.00	0.00	0.00	0.00
Norway - Pseudomonas aeruginosa CoRPA RESP - model 57	9.13E-03	0.02	0.05	0.01	0.03	0.06	2.10	3.94	5.90	0.04	0.08	0.11	0.09	0.24	0.52	1.83E-03	4.69E-03	0.01
Norway - Pseudomonas aeruginosa CoRPA SSI - model 59	8.63E-04	2.59E-03	4.17E-03	8.80E-04	2.62E-03	4.24E-03	0.25	0.74	1.19	4.75E-03	0.01	0.02	8.84E-03	0.03	0.04	1.71E-04	5.15E-04	8.29E-04
Norway - Pseudomonas aeruginosa CoRPA UTI - model 58	0.00	0.00	0.00	1.44E-05	5.03E-05	1.22E-04	0.58	1.38	2.20	0.01	0.03	0.04	0.00	0.00	0.00	0.00	0.00	0.00
Norway - Pseudomonas aeruginosa MDRPA BSI - model 46	0.00	0.00	0.00	0.00	0.00	0.00	0.00	0.00	0.00	0.00	0.00	0.00	0.00	0.00	0.00	0.00	0.00	0.00
Norway - Pseudomonas aeruginosa MDRPA OTH - model 50	0.00	0.00	0.00	0.00	0.00	0.00	0.00	0.00	0.00	0.00	0.00	0.00	0.00	0.00	0.00	0.00	0.00	0.00
Norway - Pseudomonas aeruginosa MDRPA RESP - model 47	0.00	0.00	0.00	0.00	0.00	0.00	0.00	0.00	0.00	0.00	0.00	0.00	0.00	0.00	0.00	0.00	0.00	0.00
Norway - Pseudomonas aeruginosa MDRPA SSI - model 49	0.00	0.00	0.00	0.00	0.00	0.00	0.00	0.00	0.00	0.00	0.00	0.00	0.00	0.00	0.00	0.00	0.00	0.00
Norway - Pseudomonas aeruginosa MDRPA UTI - model 48	0.00	0.00	0.00	0.00	0.00	0.00	0.00	0.00	0.00	0.00	0.00	0.00	0.00	0.00	0.00	0.00	0.00	0.00
Norway - Staphylococcus aureus MRSA BSI - model 61	1.44	1.57	1.71	1.66	1.80	1.95	16.34	17.38	18.58	0.32	0.34	0.36	2.89	3.12	3.37	0.06	0.06	0.07
Norway - Staphylococcus aureus MRSA OTH - model 65	0.00	0.00	0.00	1.23E-03	1.61E-03	2.03E-03	23.16	25.66	28.38	0.45	0.50	0.55	0.00	0.00	0.00	0.00	0.00	0.00
Norway - Staphylococcus aureus MRSA RESP - model 62	0.41	0.47	0.54	0.62	0.71	0.81	23.30	26.15	29.15	0.45	0.51	0.56	0.82	0.94	1.06	0.02	0.02	0.02
Norway - Staphylococcus aureus MRSA SSI - model 64	0.22	0.24	0.27	0.22	0.24	0.27	25.50	28.52	31.56	0.49	0.55	0.61	0.67	0.76	0.85	0.01	0.01	0.02
Norway - Staphylococcus aureus MRSA UTI - model 63	0.00	0.00	0.00	1.10E-04	1.54E-04	2.11E-04	3.38	4.26	5.14	0.07	0.08	0.10	0.00	0.00	0.00	0.00	0.00	0.00
Norway - Streptococcus pneumoniae PMRSP BSI - model 76	0.00	0.00	0.00	0.00	0.00	0.00	0.00	0.00	0.00	0.00	0.00	0.00	0.00	0.00	0.00	0.00	0.00	0.00
Norway - Streptococcus pneumoniae PMRSP OTH - model 80	0.00	0.00	0.00	0.00	0.00	0.00	0.00	0.00	0.00	0.00	0.00	0.00	0.00	0.00	0.00	0.00	0.00	0.00
Norway - Streptococcus pneumoniae PMRSP RESP - model 77	0.00	0.00	0.00	0.00	0.00	0.00	0.00	0.00	0.00	0.00	0.00	0.00	0.00	0.00	0.00	0.00	0.00	0.00
Norway - Streptococcus pneumoniae PMRSP SSI - model 79	0.00	0.00	0.00	0.00	0.00	0.00	0.00	0.00	0.00	0.00	0.00	0.00	0.00	0.00	0.00	0.00	0.00	0.00
Norway - Streptococcus pneumoniae PMRSP UTI - model 78	0.00	0.00	0.00	0.00	0.00	0.00	0.00	0.00	0.00	0.00	0.00	0.00	0.00	0.00	0.00	0.00	0.00	0.00
Norway - Streptococcus pneumoniae PRSP BSI - model 71	0.79	0.84	0.90	0.91	0.96	1.03	7.00	7.00	7.00	0.14	0.14	0.14	1.18	1.26	1.34	0.02	0.02	0.03
Norway - Streptococcus pneumoniae PRSP OTH - model 75	0.00	0.00	0.00	6.43E-05	9.42E-05	1.29E-04	2.26	2.48	2.77	0.04	0.05	0.05	0.00	0.00	0.00	0.00	0.00	0.00
Norway - Streptococcus pneumoniae PRSP RESP - model 72	0.52	0.63	0.81	0.80	0.96	1.21	22.32	26.56	32.42	0.43	0.51	0.63	0.80	0.95	1.20	0.02	0.02	0.02
Norway - Streptococcus pneumoniae PRSP SSI - model 74	0.00	0.00	0.00	0.00	0.00	0.00	0.00	0.00	0.00	0.00	0.00	0.00	0.00	0.00	0.00	0.00	0.00	0.00
Norway - Streptococcus pneumoniae PRSP UTI - model 73	0.00	0.00	0.00	0.00	0.00	0.00	0.00	0.00	0.00	0.00	0.00	0.00	0.00	0.00	0.00	0.00	0.00	0.00
**Total (Median & 95% UIs)**	24.21	27.95	32.19	28.91	33.14	37.90	1,660.81	1,881.57	2,104.97	32.15	36.42	40.74	58.87	68.53	79.07	1.14	1.33	1.53
**Total (average)**	**28.03**			**33.22**			**1,881.69**			**36.42**			**68.65**			**1.33**		

Infection	YLD			YLL			DALY			DALY per Case			YLD per 100,000		
Colistin resistant *Acinetobacter spp.*	6.37	10.32	15.22	22.29	53.89	96.33	30.36	64.18	109.72	4.87	8.84	12.95	0.02	0.03	0.04
Carbapenem resistant *Acinetobacter spp.* (excluding those resistant to colistin)	1,249.15	1,375.21	1,524.03	6,105.86	7,386.49	8,681.89	7,377.78	8,765.98	10,121.98	6.54	7.56	8.60	3.29	3.62	4.01
Aminoglycosides and fluoroquinolones resistant *Acinetobacter* spp. (excluding those resistant to colistin and/or carbapenem)	243.64	277.60	316.14	1,154.54	1,449.11	1,768.35	1,407.18	1,727.10	2,064.00	5.49	6.61	7.74	0.64	0.73	0.83
Vancomycin resistant Enterococci (*E. faecalis* and *E. faecium*)	318.92	378.50	452.85	2,975.53	3,532.04	4,152.77	3,302.09	3,910.32	4,578.31	7.67	8.23	8.83	0.84	1.00	1.19
Colistin resistant *Escherichia coli*	0.00	0.00	0.00	0.00	0.00	0.00	0.00	0.00	0.00	0.00	0.00	0.00	0.00	0.00	0.00
Carbapenem resistant *Escherichia coli* (excluding those resistant to colistin)	19.16	27.66	38.94	183.46	296.03	439.81	206.36	323.94	474.28	15.80	23.14	30.84	0.05	0.07	0.10
Third-generation cephalosporin resistant *Escherichia coli* (excluding those resistant to colistin and/or carbapenem)	1,020.18	1,131.03	1,267.57	6,013.89	6,701.32	7,449.35	7,076.91	7,829.74	8,657.23	5.63	6.10	6.59	2.68	2.98	3.34
Colistin resistant *Klebsiella pneumoniae*	62.73	77.23	93.14	1,054.53	1,364.11	1,718.54	1,126.60	1,442.02	1,805.72	13.57	16.94	20.68	0.17	0.20	0.25
Carbapenem resistant *Klebsiella pneumoniae* (excluding those resistant to colistin)	9.15	12.73	17.19	89.23	133.49	186.09	100.69	146.21	201.50	5.34	7.26	9.34	0.02	0.03	0.05
Third-generation cephalosporin resistant *Klebsiella pneumoniae* (excluding those resistant to colistin and/or carbapenem)	3,595.65	3,935.98	4,276.89	17,656.84	18,834.92	20,144.00	21,300.64	22,775.65	24,302.88	7.61	7.97	8.33	9.46	10.36	11.25
Colistin resistant *Pseudomonas aeruginosa*	0.00	0.00	0.00	0.00	0.00	0.00	0.00	0.00	0.00	0.00	0.00	0.00	0.00	0.00	0.00
Carbapenem resistant *Pseudomonas aeruginosa* (excluding those resistant to colistin)	1,921.88	2,149.52	2,381.24	8,889.39	10,849.65	12,844.07	10,885.31	13,015.71	15,107.61	9.52	11.08	12.69	5.06	5.66	6.27
Pseudomonas aeruginosa resistant to three or more antimicrobial groups (excluding those resistant to colistin and/or carbapenem)	180.81	227.78	280.27	749.80	1,077.34	1,448.41	958.12	1,304.37	1,711.32	5.73	7.51	9.38	0.48	0.60	0.74
Methicillin resistant *Staphylococcus aureus*	1.408.49	1.528.69	1.658.16	6.849.43	7.328.95	7.783.41	8.319.53	8.859.44	9.399.38	6.77	7.05	7.34	3.71	4.02	4.36
Penicillin and macrolides resistant *Streptococcus pneumoniae* (excluding those only resistant to penicillin)	114.15	150.09	217.18	422.23	499.54	636.12	549.64	648.74	836.13	8.63	9.69	11.00	0.30	0.39	0.57
Penicillin tesistant *Streptococcus pneumoniae* (excluding those resistant to macrolides)	12.02	17.02	27.10	45.71	56.55	76.79	59.44	73.59	101.75	5.87	6.68	7.47	0.03	0.04	0.07
Total	10,162.29	11,299.37	12,565.91	52,212.72	59,563.43	67,425.94	62,700.66	70,886.98	79,471.80				26.74	29.73	33.06

Infection	YLL per 100,000			DALY per 100,000			Cases per year			Incidence per 100,000			Deaths per year			Mortality per 100,000		
Colistin resistant *Acinetobacter spp.*	0.06	0.14	0.25	0.08	0.17	0.29	12.66	21.36	30.57	0.03	0.06	0.08	0.74	1.79	3.20	0.00	0.00	0.01
Carbapenem resistant *Acinetobacter spp.* (excluding those resistant to colistin)	16.07	19.44	22.84	19.41	23.06	26.63	3,066.24	3,427.95	3,775.21	8.07	9.02	9.93	242.41	300.01	352.94	0.64	0.79	0.93
Aminoglycosides and fluoroquinolones resistant *Acinetobacter* spp. (excluding those resistant to colistin and/or carbapenem)	3.04	3.81	4.65	3.70	4.54	5.43	677.34	770.60	865.34	1.78	2.03	2.28	53.58	65.83	80.31	0.14	0.17	0.21
Vancomycin resistant Enterococci (*E. faecalis* and *E. faecium*)	7.83	9.29	10.93	8.69	10.29	12.05	1,505.25	1,830.82	2,159.62	3.96	4.82	5.68	99.69	119.17	141.37	0.26	0.31	0.37
Colistin resistant *Escherichia coli*	0.00	0.00	0.00	0.00	0.00	0.00	0.00	0.00	0.00	0.00	0.00	0.00	0.00	0.00	0.00	0.00	0.00	0.00
Carbapenem resistant *Escherichia coli* (excluding those resistant to colistin)	0.48	0.78	1.16	0.54	0.85	1.25	77.19	112.03	146.25	0.20	0.29	0.38	3.63	5.81	8.18	0.01	0.02	0.02
Third-generation cephalosporin resistant *Escherichia coli* (excluding those resistant to colistin and/or carbapenem)	15.82	17.63	19.60	18.62	20.60	22.78	9,170.41	10,248.90	11,319.62	24.13	26.97	29.78	276.85	311.77	350.77	0.73	0.82	0.92
Colistin resistant *Klebsiella pneumoniae*	2.77	3.59	4.52	2.96	3.79	4.75	201.56	251.67	301.72	0.53	0.66	0.79	44.38	55.98	69.34	0.12	0.15	0.18
Carbapenem resistant *Klebsiella pneumoniae* (excluding those resistant to colistin)	0.23	0.35	0.49	0.26	0.38	0.53	43.29	62.97	84.57	0.11	0.17	0.22	5.65	8.46	11.70	0.01	0.02	0.03
Third-generation cephalosporin resistant *Klebsiella pneumoniae* (excluding those resistant to colistin and/or carbapenem)	46.46	49.56	53.00	56.05	59.93	63.95	10,713.03	11,678.08	12,652.19	28.19	30.73	33.29	589.55	631.34	675.45	1.55	1.66	1.78
Colistin resistant *Pseudomonas aeruginosa*	0.00	0.00	0.00	0.00	0.00	0.00	0.00	0.00	0.00	0.00	0.00	0.00	0.00	0.00	0.00	0.00	0.00	0.00
Carbapenem resistant *Pseudomonas aeruginosa* (excluding those resistant to colistin)	23.39	28.55	33.80	28.64	34.25	39.75	4,558.16	5,177.74	5,796.59	11.99	13.62	15.25	283.41	350.71	418.84	0.75	0.92	1.10
Pseudomonas aeruginosa resistant to three or more antimicrobial groups (excluding those resistant to colistin and/or carbapenem)	1.97	2.83	3.81	2.52	3.43	4.50	617.91	769.63	921.48	1.63	2.03	2.42	35.26	49.12	64.55	0.09	0.13	0.17
Methicillin resistant *Staphylococcus aureus*	18.02	19.28	20.48	21.89	23.31	24.73	5.965.19	6.463.03	6.964.73	15.70	17.01	18.33	281.73	302.30	323.75	0.74	0.80	0.85
Penicillin and macrolides resistant *Streptococcus pneumoniae* (excluding those only resistant to penicillin)	1.11	1.31	1.67	1.45	1.71	2.20	188.86	219.13	267.10	0.50	0.58	0.70	11.82	13.50	15.81	0.03	0.04	0.04
Penicillin tesistant *Streptococcus pneumoniae* (excluding those resistant to macrolides)	0.12	0.15	0.20	0.16	0.19	0.27	29.09	35.44	52.56	0.08	0.09	0.14	1.79	2.21	3.00	0.00	0.01	0.01
Total	137.38	156.72	177.41	164.98	186.52	209.11	36,826.17	41,069.36	45,337.54	96.90	108.06	119.29	1,930.50	2,218.00	2,519.22	5.08	5.84	6.63

Model	YLD			YLL			DALY			DALY per Case			YLD per 100,000		
Poland - Acinetobacter spp. CRACI BSI - model 36	722.44	783.46	861.09	4,923.96	6,049.56	7,191.49	5,668.90	6,839.15	7,978.79	4.84	5.76	6.68	1.90	2.06	2.27
Poland - Acinetobacter spp. CRACI OTH - model 40	0.11	0.16	0.21	0.00	0.00	0.00	0.11	0.16	0.21	3.04E-03	3.75E-03	4.48E-03	2.92E-04	4.13E-04	5.58E-04
Poland - Acinetobacter spp. CRACI RESP - model 37	525.16	589.70	660.25	1,025.22	1,148.25	1,267.02	1,550.60	1,736.15	1,917.41	1.28	1.35	1.45	1.38	1.55	1.74
Poland - Acinetobacter spp. CRACI SSI - model 39	0.68	0.92	1.23	156.67	188.68	223.37	157.42	189.54	224.32	0.41	0.44	0.47	1.78E-03	2.42E-03	3.22E-03
Poland - Acinetobacter spp. CRACI UTI - model 38	0.76	0.98	1.25	0.00	0.00	0.00	0.76	0.98	1.25	1.68E-03	2.04E-03	2.46E-03	1.99E-03	2.58E-03	3.29E-03
Poland - Acinetobacter spp. CoRACI BSI - model 41	3.91	5.79	8.57	17.02	44.32	81.86	22.42	50.03	89.22	3.20	6.92	10.79	0.01	0.02	0.02
Poland - Acinetobacter spp. CoRACI OTH - model 45	1.38E-04	8.16E-04	2.75E-03	0.00	0.00	0.00	1.38E-04	8.16E-04	2.75E-03	1.58E-03	3.60E-03	6.29E-03	3.63E-07	2.15E-06	7.22E-06
Poland - Acinetobacter spp. CoRACI RESP - model 42	2.46	4.52	6.62	4.99	8.85	13.31	7.65	13.42	19.33	1.40	1.64	1.88	6.48E-03	0.01	0.02
Poland - Acinetobacter spp. CoRACI SSI - model 44	1.06E-03	5.07E-03	0.01	0.28	0.72	1.16	0.29	0.73	1.16	0.27	0.27	0.28	2.80E-06	1.33E-05	3.86E-05
Poland - Acinetobacter spp. CoRACI UTI - model 43	1.50E-03	5.69E-03	0.01	0.00	0.00	0.00	1.50E-03	5.69E-03	0.01	8.02E-04	2.02E-03	3.57E-03	3.94E-06	1.50E-05	3.56E-05
Poland - Acinetobacter spp. MDRACI BSI - model 31	141.19	158.62	179.56	926.18	1,181.17	1,461.57	1,074.83	1,340.36	1,622.54	4.03	5.02	6.01	0.37	0.42	0.47
Poland - Acinetobacter spp. MDRACI OTH - model 35	0.02	0.04	0.05	0.00	0.00	0.00	0.02	0.04	0.05	2.85E-03	3.72E-03	4.57E-03	6.20E-05	9.22E-05	1.31E-04
Poland - Acinetobacter spp. MDRACI RESP - model 32	102.12	118.51	135.96	199.04	231.36	263.92	302.51	349.69	398.08	1.12	1.21	1.31	0.27	0.31	0.36
Poland - Acinetobacter spp. MDRACI SSI - model 34	0.14	0.21	0.28	29.32	36.59	42.86	29.65	36.79	43.04	0.34	0.38	0.41	3.80E-04	5.43E-04	7.37E-04
Poland - Acinetobacter spp. MDRACI UTI - model 33	0.16	0.22	0.29	0.00	0.00	0.00	0.16	0.22	0.29	1.63E-03	2.05E-03	2.52E-03	4.33E-04	5.88E-04	7.73E-04
Poland - Enterococcus faecalis and E. faecium VRE BSI - model 66	316.99	375.90	449.43	2,741.77	3,235.36	3,795.41	3,066.80	3,610.97	4,217.56	7.24	7.76	8.32	0.83	0.99	1.18
Poland - Enterococcus faecalis and E. faecium VRE OTH - model 70	0.62	0.82	1.05	0.00	0.00	0.00	0.62	0.82	1.05	1.35E-03	1.65E-03	2.02E-03	1.64E-03	2.15E-03	2.76E-03
Poland - Enterococcus faecalis and E. faecium VRE RESP - model 67	0.00	0.00	0.00	0.00	0.00	0.00	0.00	0.00	0.00	NaN	NaN	NaN	0.00	0.00	0.00
Poland - Enterococcus faecalis and E. faecium VRE SSI - model 69	0.98	1.35	1.80	233.75	296.68	357.37	234.33	298.10	359.12	0.43	0.47	0.51	2.57E-03	3.55E-03	4.74E-03
Poland - Enterococcus faecalis and E. faecium VRE UTI - model 68	0.33	0.44	0.57	0.00	0.00	0.00	0.33	0.44	0.57	1.53E-03	1.86E-03	2.28E-03	8.67E-04	1.15E-03	1.50E-03
Poland - Escherichia coli 3GCREC BSI - model 16	784.96	853.96	942.04	5,022.45	5,534.58	6,113.11	5,844.02	6,385.93	7,001.25	4.10	4.44	4.78	2.07	2.25	2.48
Poland - Escherichia coli 3GCREC OTH - model 20	1.18	1.58	2.05	0.00	0.00	0.00	1.18	1.58	2.05	2.71E-03	3.31E-03	4.01E-03	3.11E-03	4.16E-03	5.40E-03
Poland - Escherichia coli 3GCREC RESP - model 17	221.99	260.16	304.19	425.64	506.42	588.27	653.48	766.91	887.96	1.11	1.21	1.34	0.58	0.68	0.80
Poland - Escherichia coli 3GCREC SSI - model 19	2.43	3.24	4.23	565.81	660.32	747.97	568.61	663.24	750.92	0.41	0.43	0.46	6.39E-03	8.54E-03	0.01
Poland - Escherichia coli 3GCREC UTI - model 18	9.63	12.08	15.05	0.00	0.00	0.00	9.63	12.08	15.05	1.60E-03	1.96E-03	2.39E-03	0.03	0.03	0.04
Poland - Escherichia coli CREC BSI - model 21	15.42	20.39	27.46	171.24	273.75	407.37	190.17	294.32	431.15	13.24	19.86	26.87	0.04	0.05	0.07
Poland - Escherichia coli CREC OTH - model 25	0.01	0.03	0.06	0.00	0.00	0.00	0.01	0.03	0.06	1.72E-03	3.34E-03	6.13E-03	3.25E-05	7.53E-05	1.58E-04
Poland - Escherichia coli CREC RESP - model 22	3.66	7.08	11.10	7.16	14.05	21.29	11.11	21.20	31.62	2.17	2.81	3.42	9.62E-03	0.02	0.03
Poland - Escherichia coli CREC SSI - model 24	0.01	0.04	0.07	5.06	8.22	11.15	5.00	8.27	11.21	0.39	0.47	0.54	3.48E-05	9.47E-05	1.89E-04
Poland - Escherichia coli CREC UTI - model 23	0.06	0.13	0.24	0.00	0.00	0.00	0.06	0.13	0.24	1.07E-03	2.05E-03	3.48E-03	1.63E-04	3.34E-04	6.34E-04
Poland - Escherichia coli CoREC BSI - model 26	0.00	0.00	0.00	0.00	0.00	0.00	0.00	0.00	0.00	NaN	NaN	NaN	0.00	0.00	0.00
Poland - Escherichia coli CoREC OTH - model 30	0.00	0.00	0.00	0.00	0.00	0.00	0.00	0.00	0.00	NaN	NaN	NaN	0.00	0.00	0.00
Poland - Escherichia coli CoREC RESP - model 27	0.00	0.00	0.00	0.00	0.00	0.00	0.00	0.00	0.00	NaN	NaN	NaN	0.00	0.00	0.00
Poland - Escherichia coli CoREC SSI - model 29	0.00	0.00	0.00	0.00	0.00	0.00	0.00	0.00	0.00	NaN	NaN	NaN	0.00	0.00	0.00
Poland - Escherichia coli CoREC UTI - model 28	0.00	0.00	0.00	0.00	0.00	0.00	0.00	0.00	0.00	NaN	NaN	NaN	0.00	0.00	0.00
Poland - Klebsiella pneumoniae 3GCRKP BSI - model 1	2,259.71	2,444.68	2,635.00	14,574.84	15,409.07	16,339.72	16,868.12	17,850.32	18,873.23	5.61	5.85	6.08	5.95	6.43	6.93
Poland - Klebsiella pneumoniae 3GCRKP OTH - model 5	3.36	4.06	5.06	0.00	0.00	0.00	3.36	4.06	5.06	2.82E-03	3.36E-03	4.02E-03	8.83E-03	0.01	0.01
Poland - Klebsiella pneumoniae 3GCRKP RESP - model 2	1,324.86	1,477.69	1,625.12	2,594.16	2,873.09	3,181.65	3,933.26	4,358.67	4,789.44	1.56	1.65	1.75	3.49	3.89	4.28
Poland - Klebsiella pneumoniae 3GCRKP SSI - model 4	2.01	2.55	3.29	487.85	552.75	622.62	490.19	555.58	626.72	0.44	0.47	0.49	5.28E-03	6.70E-03	8.65E-03
Poland - Klebsiella pneumoniae 3GCRKP UTI - model 3	5.72	7.01	8.42	0.00	0.00	0.00	5.72	7.01	8.42	1.66E-03	1.96E-03	2.31E-03	0.02	0.02	0.02
Poland - Klebsiella pneumoniae CRKP BSI - model 6	7.59	9.80	12.76	85.24	125.79	174.57	95.25	135.53	185.62	4.35	6.05	7.86	0.02	0.03	0.03
Poland - Klebsiella pneumoniae CRKP OTH - model 10	0.02	0.04	0.07	0.00	0.00	0.00	0.02	0.04	0.07	1.94E-03	3.49E-03	5.60E-03	4.19E-05	9.48E-05	1.83E-04
Poland - Klebsiella pneumoniae CRKP RESP - model 7	1.52	2.85	4.29	2.76	5.45	8.19	4.19	8.34	12.42	0.69	0.87	1.08	4.01E-03	7.49E-03	0.01
Poland - Klebsiella pneumoniae CRKP SSI - model 9	5.27E-03	0.01	0.03	1.22	2.26	3.33	1.22	2.27	3.34	0.30	0.34	0.39	1.39E-05	3.50E-05	7.17E-05
Poland - Klebsiella pneumoniae CRKP UTI - model 8	0.01	0.03	0.05	0.00	0.00	0.00	0.01	0.03	0.05	1.16E-03	2.06E-03	3.23E-03	3.74E-05	7.27E-05	1.30E-04
Poland - Klebsiella pneumoniae CoRKP BSI - model 11	50.36	59.87	70.14	1,023.20	1,320.64	1,662.11	1,082.62	1,381.09	1,726.62	12.09	15.22	18.70	0.13	0.16	0.18
Poland - Klebsiella pneumoniae CoRKP OTH - model 15	0.09	0.15	0.22	0.00	0.00	0.00	0.09	0.15	0.22	2.45E-03	3.52E-03	4.85E-03	2.47E-04	3.84E-04	5.88E-04
Poland - Klebsiella pneumoniae CoRKP RESP - model 12	12.16	17.05	22.52	23.91	33.16	43.57	36.36	50.31	65.77	1.12	1.32	1.55	0.03	0.04	0.06
Poland - Klebsiella pneumoniae CoRKP SSI - model 14	0.03	0.06	0.09	7.41	10.31	12.87	7.46	10.36	12.94	0.36	0.39	0.42	9.02E-05	1.48E-04	2.28E-04
Poland - Klebsiella pneumoniae CoRKP UTI - model 13	0.08	0.11	0.16	0.00	0.00	0.00	0.08	0.11	0.16	1.50E-03	2.08E-03	2.71E-03	2.01E-04	2.98E-04	4.24E-04
Poland - Pseudomonas aeruginosa CRPA BSI - model 51	544.16	602.08	660.45	4,208.30	5,158.37	6,132.59	4,784.01	5,765.36	6,752.00	6.82	8.01	9.24	1.43	1.58	1.74
Poland - Pseudomonas aeruginosa CRPA OTH - model 55	1.02	1.34	1.70	0.00	0.00	0.00	1.02	1.34	1.70	3.06E-03	3.68E-03	4.33E-03	2.68E-03	3.52E-03	4.48E-03
Poland - Pseudomonas aeruginosa CRPA RESP - model 52	1,374.41	1,543.16	1,715.30	4,500.35	5,474.13	6,459.58	5,917.22	7,028.92	8,098.19	2.29	2.62	2.97	3.62	4.06	4.51
Poland - Pseudomonas aeruginosa CRPA SSI - model 54	0.78	1.04	1.37	180.73	217.15	251.89	181.55	218.20	253.29	0.41	0.44	0.47	2.05E-03	2.74E-03	3.60E-03
Poland - Pseudomonas aeruginosa CRPA UTI - model 53	1.51	1.90	2.43	0.00	0.00	0.00	1.51	1.90	2.43	1.69E-03	2.05E-03	2.42E-03	3.97E-03	5.01E-03	6.39E-03
Poland - Pseudomonas aeruginosa CoRPA BSI - model 56	0.00	0.00	0.00	0.00	0.00	0.00	0.00	0.00	0.00	NaN	NaN	NaN	0.00	0.00	0.00
Poland - Pseudomonas aeruginosa CoRPA OTH - model 60	0.00	0.00	0.00	0.00	0.00	0.00	0.00	0.00	0.00	NaN	NaN	NaN	0.00	0.00	0.00
Poland - Pseudomonas aeruginosa CoRPA RESP - model 57	0.00	0.00	0.00	0.00	0.00	0.00	0.00	0.00	0.00	NaN	NaN	NaN	0.00	0.00	0.00
Poland - Pseudomonas aeruginosa CoRPA SSI - model 59	0.00	0.00	0.00	0.00	0.00	0.00	0.00	0.00	0.00	NaN	NaN	NaN	0.00	0.00	0.00
Poland - Pseudomonas aeruginosa CoRPA UTI - model 58	0.00	0.00	0.00	0.00	0.00	0.00	0.00	0.00	0.00	NaN	NaN	NaN	0.00	0.00	0.00
Poland - Pseudomonas aeruginosa MDRPA BSI - model 46	51.87	63.81	76.75	342.42	498.99	669.47	406.96	562.20	745.41	3.93	5.26	6.66	0.14	0.17	0.20
Poland - Pseudomonas aeruginosa MDRPA OTH - model 50	0.13	0.20	0.29	0.00	0.00	0.00	0.13	0.20	0.29	2.79E-03	3.75E-03	4.84E-03	3.43E-04	5.39E-04	7.60E-04
Poland - Pseudomonas aeruginosa MDRPA RESP - model 47	128.52	163.34	202.60	385.13	546.13	737.67	528.50	709.29	923.78	1.43	1.80	2.21	0.34	0.43	0.53
Poland - Pseudomonas aeruginosa MDRPA SSI - model 49	0.10	0.15	0.23	22.25	32.23	41.26	22.35	32.40	41.45	0.38	0.44	0.51	2.51E-04	4.05E-04	6.16E-04
Poland - Pseudomonas aeruginosa MDRPA UTI - model 48	0.19	0.28	0.39	0.00	0.00	0.00	0.19	0.28	0.39	1.49E-03	2.04E-03	2.63E-03	4.91E-04	7.33E-04	1.03E-03
Poland - Staphylococcus aureus MRSA BSI - model 61	689.10	741.99	801.34	4,742.39	5,013.47	5,271.13	5,486.50	5,756.50	6,042.00	5.02	5.22	5.42	1.81	1.95	2.11
Poland - Staphylococcus aureus MRSA OTH - model 65	4.29	5.30	6.39	0.00	0.00	0.00	4.29	5.30	6.39	2.73E-03	3.25E-03	3.89E-03	0.01	0.01	0.02
Poland - Staphylococcus aureus MRSA RESP - model 62	711.71	777.05	844.88	1,381.52	1,515.67	1,641.62	2,099.15	2,293.44	2,475.21	1.31	1.38	1.46	1.87	2.04	2.22
Poland - Staphylococcus aureus MRSA SSI - model 64	3.01	3.86	4.92	725.53	799.81	870.66	729.21	803.70	875.16	0.43	0.44	0.46	7.92E-03	0.01	0.01
Poland - Staphylococcus aureus MRSA UTI - model 63	0.39	0.50	0.63	0.00	0.00	0.00	0.39	0.50	0.63	1.56E-03	1.87E-03	2.24E-03	1.02E-03	1.32E-03	1.65E-03
Poland - Streptococcus pneumoniae PMRSP BSI - model 76	33.57	41.97	52.52	260.58	287.73	316.52	299.45	329.84	361.66	6.99	7.70	8.44	0.09	0.11	0.14
Poland - Streptococcus pneumoniae PMRSP OTH - model 80	0.02	0.03	0.04	0.00	0.00	0.00	0.02	0.03	0.04	1.20E-03	1.91E-03	2.87E-03	4.81E-05	7.66E-05	1.15E-04
Poland - Streptococcus pneumoniae PMRSP RESP - model 77	80.56	108.09	164.61	161.65	211.81	319.60	250.17	318.87	474.42	1.64	1.99	2.56	0.21	0.28	0.43
Poland - Streptococcus pneumoniae PMRSP SSI - model 79	0.00	0.00	0.00	0.00	0.00	0.00	0.00	0.00	0.00	NaN	NaN	NaN	0.00	0.00	0.00
Poland - Streptococcus pneumoniae PMRSP UTI - model 78	0.00	0.00	0.00	0.00	0.00	0.00	0.00	0.00	0.00	NaN	NaN	NaN	0.00	0.00	0.00
Poland - Streptococcus pneumoniae PRSP BSI - model 71	3.55	4.79	6.30	28.89	32.79	36.77	33.31	37.73	41.91	4.66	5.28	5.87	9.35E-03	0.01	0.02
Poland - Streptococcus pneumoniae PRSP OTH - model 75	1.96E-03	4.74E-03	9.48E-03	0.00	0.00	0.00	1.96E-03	4.74E-03	9.48E-03	7.72E-04	1.88E-03	3.67E-03	5.16E-06	1.25E-05	2.49E-05
Poland - Streptococcus pneumoniae PRSP RESP - model 72	8.46	12.23	20.78	16.81	23.76	40.03	26.13	35.85	59.83	1.20	1.39	1.60	0.02	0.03	0.05
Poland - Streptococcus pneumoniae PRSP SSI - model 74	0.00	0.00	0.00	0.00	0.00	0.00	0.00	0.00	0.00	NaN	NaN	NaN	0.00	0.00	0.00
Poland - Streptococcus pneumoniae PRSP UTI - model 73	0.00	0.00	0.00	0.00	0.00	0.00	0.00	0.00	0.00	NaN	NaN	NaN	0.00	0.00	0.00
**Total (Median & 95% UIs)**	10,162.29	11,299.37	12,565.91	52,212.72	59,563.43	67,425.94	62,700.66	70,886.98	79,471.80		-		26.74	29.73	33.06
**Total (average)**		**11,313.14**			**59,604.27**			**70,917.41**			-			**29.77**	

Model	YLL per 100,000			DALY per 100,000			Cases per year			Incidence per 100,000			Deaths per year			Mortality per 100,000		
Poland - Acinetobacter spp. CRACI BSI - model 36	12.96	15.92	18.92	14.92	18.00	20.99	1,115.93	1,184.86	1,253.98	2.94	3.12	3.30	193.35	243.62	289.81	0.51	0.64	0.76
Poland - Acinetobacter spp. CRACI OTH - model 40	0.00	0.00	0.00	2.92E-04	4.13E-04	5.58E-04	31.87	41.75	52.12	0.08	0.11	0.14	0.00	0.00	0.00	0.00	0.00	0.00
Poland - Acinetobacter spp. CRACI RESP - model 37	2.70	3.02	3.33	4.08	4.57	5.05	1,141.72	1,285.27	1,406.69	3.00	3.38	3.70	40.78	46.27	51.19	0.11	0.12	0.13
Poland - Acinetobacter spp. CRACI SSI - model 39	0.41	0.50	0.59	0.41	0.50	0.59	371.99	434.17	498.40	0.98	1.14	1.31	8.28	10.11	11.94	0.02	0.03	0.03
Poland - Acinetobacter spp. CRACI UTI - model 38	0.00	0.00	0.00	1.99E-03	2.58E-03	3.29E-03	404.73	481.89	564.02	1.06	1.27	1.48	0.00	0.00	0.00	0.00	0.00	0.00
Poland - Acinetobacter spp. CoRACI BSI - model 41	0.04	0.12	0.22	0.06	0.13	0.23	5.80	7.33	9.32	0.02	0.02	0.02	0.57	1.48	2.72	1.49E-03	3.88E-03	7.16E-03
Poland - Acinetobacter spp. CoRACI OTH - model 45	0.00	0.00	0.00	3.63E-07	2.15E-06	7.22E-06	0.05	0.25	0.57	1.19E-04	6.50E-04	1.49E-03	0.00	0.00	0.00	0.00	0.00	0.00
Poland - Acinetobacter spp. CoRACI RESP - model 42	0.01	0.02	0.04	0.02	0.04	0.05	4.66	8.11	11.39	0.01	0.02	0.03	0.17	0.29	0.44	4.37E-04	7.74E-04	1.17E-03
Poland - Acinetobacter spp. CoRACI SSI - model 44	7.32E-04	1.91E-03	3.06E-03	7.54E-04	1.92E-03	3.06E-03	1.03	2.68	4.30	2.70E-03	7.04E-03	0.01	9.25E-03	0.02	0.04	2.43E-05	6.34E-05	1.02E-04
Poland - Acinetobacter spp. CoRACI UTI - model 43	0.00	0.00	0.00	3.94E-06	1.50E-05	3.56E-05	1.13	2.99	4.99	2.97E-03	7.88E-03	0.01	0.00	0.00	0.00	0.00	0.00	0.00
Poland - Acinetobacter spp. MDRACI BSI - model 31	2.44	3.11	3.85	2.83	3.53	4.27	249.51	266.34	286.64	0.66	0.70	0.75	42.62	53.09	65.67	0.11	0.14	0.17
Poland - Acinetobacter spp. MDRACI OTH - model 35	0.00	0.00	0.00	6.20E-05	9.22E-05	1.31E-04	6.99	9.45	12.12	0.02	0.02	0.03	0.00	0.00	0.00	0.00	0.00	0.00
Poland - Acinetobacter spp. MDRACI RESP - model 32	0.52	0.61	0.69	0.80	0.92	1.05	253.56	288.84	324.44	0.67	0.76	0.85	9.06	10.41	11.85	0.02	0.03	0.03
Poland - Acinetobacter spp. MDRACI SSI - model 34	0.08	0.10	0.11	0.08	0.10	0.11	78.68	97.16	113.66	0.21	0.26	0.30	1.89	2.33	2.79	4.98E-03	6.13E-03	7.33E-03
Poland - Acinetobacter spp. MDRACI UTI - model 33	0.00	0.00	0.00	4.33E-04	5.88E-04	7.73E-04	88.58	108.81	128.48	0.23	0.29	0.34	0.00	0.00	0.00	0.00	0.00	0.00
Poland - Enterococcus faecalis and E. faecium VRE BSI - model 66	7.21	8.51	9.99	8.07	9.50	11.10	392.04	462.87	546.76	1.03	1.22	1.44	89.57	105.81	125.14	0.24	0.28	0.33
Poland - Enterococcus faecalis and E. faecium VRE OTH - model 70	0.00	0.00	0.00	1.64E-03	2.15E-03	2.76E-03	403.94	497.09	581.34	1.06	1.31	1.53	0.00	0.00	0.00	0.00	0.00	0.00
Poland - Enterococcus faecalis and E. faecium VRE RESP - model 67	0.00	0.00	0.00	0.00	0.00	0.00	0.00	0.00	0.00	0.00	0.00	0.00	0.00	0.00	0.00	0.00	0.00	0.00
Poland - Enterococcus faecalis and E. faecium VRE SSI - model 69	0.62	0.78	0.94	0.62	0.78	0.94	519.76	636.52	752.66	1.37	1.67	1.98	10.12	13.37	16.24	0.03	0.04	0.04
Poland - Enterococcus faecalis and E. faecium VRE UTI - model 68	0.00	0.00	0.00	8.67E-04	1.15E-03	1.50E-03	189.52	234.34	278.86	0.50	0.62	0.73	0.00	0.00	0.00	0.00	0.00	0.00
Poland - Escherichia coli 3GCREC BSI - model 16	13.22	14.56	16.08	15.38	16.80	18.42	1,344.17	1,440.47	1,540.66	3.54	3.79	4.05	223.77	248.74	277.88	0.59	0.65	0.73
Poland - Escherichia coli 3GCREC OTH - model 20	0.00	0.00	0.00	3.11E-03	4.16E-03	5.40E-03	393.33	475.18	562.16	1.03	1.25	1.48	0.00	0.00	0.00	0.00	0.00	0.00
Poland - Escherichia coli 3GCREC RESP - model 17	1.12	1.33	1.55	1.72	2.02	2.34	526.54	631.62	730.30	1.39	1.66	1.92	19.00	22.63	26.65	0.05	0.06	0.07
Poland - Escherichia coli 3GCREC SSI - model 19	1.49	1.74	1.97	1.50	1.75	1.98	1,341.54	1,528.26	1,709.28	3.53	4.02	4.50	34.08	40.39	46.24	0.09	0.11	0.12
Poland - Escherichia coli 3GCREC UTI - model 18	0.00	0.00	0.00	0.03	0.03	0.04	5,564.83	6,173.37	6,777.22	14.64	16.24	17.83	0.00	0.00	0.00	0.00	0.00	0.00
Poland - Escherichia coli CREC BSI - model 21	0.45	0.72	1.07	0.50	0.77	1.13	12.36	14.93	18.37	0.03	0.04	0.05	3.38	5.39	7.58	8.89E-03	0.01	0.02
Poland - Escherichia coli CREC OTH - model 25	0.00	0.00	0.00	3.25E-05	7.53E-05	1.58E-04	5.10	8.80	12.37	0.01	0.02	0.03	0.00	0.00	0.00	0.00	0.00	0.00
Poland - Escherichia coli CREC RESP - model 22	0.02	0.04	0.06	0.03	0.06	0.08	4.14	7.52	10.86	0.01	0.02	0.03	0.15	0.27	0.39	3.87E-04	7.10E-04	1.04E-03
Poland - Escherichia coli CREC SSI - model 24	0.01	0.02	0.03	0.01	0.02	0.03	11.50	17.64	23.37	0.03	0.05	0.06	0.10	0.16	0.21	2.72E-04	4.18E-04	5.53E-04
Poland - Escherichia coli CREC UTI - model 23	0.00	0.00	0.00	1.63E-04	3.34E-04	6.34E-04	44.10	63.15	81.28	0.12	0.17	0.21	0.00	0.00	0.00	0.00	0.00	0.00
Poland - Escherichia coli CoREC BSI - model 26	0.00	0.00	0.00	0.00	0.00	0.00	0.00	0.00	0.00	0.00	0.00	0.00	0.00	0.00	0.00	0.00	0.00	0.00
Poland - Escherichia coli CoREC OTH - model 30	0.00	0.00	0.00	0.00	0.00	0.00	0.00	0.00	0.00	0.00	0.00	0.00	0.00	0.00	0.00	0.00	0.00	0.00
Poland - Escherichia coli CoREC RESP - model 27	0.00	0.00	0.00	0.00	0.00	0.00	0.00	0.00	0.00	0.00	0.00	0.00	0.00	0.00	0.00	0.00	0.00	0.00
Poland - Escherichia coli CoREC SSI - model 29	0.00	0.00	0.00	0.00	0.00	0.00	0.00	0.00	0.00	0.00	0.00	0.00	0.00	0.00	0.00	0.00	0.00	0.00
Poland - Escherichia coli CoREC UTI - model 28	0.00	0.00	0.00	0.00	0.00	0.00	0.00	0.00	0.00	0.00	0.00	0.00	0.00	0.00	0.00	0.00	0.00	0.00
Poland - Klebsiella pneumoniae 3GCRKP BSI - model 1	38.35	40.54	42.99	44.38	46.97	49.66	2,918.33	3,053.53	3,191.64	7.68	8.03	8.40	481.13	510.34	540.16	1.27	1.34	1.42
Poland - Klebsiella pneumoniae 3GCRKP OTH - model 5	0.00	0.00	0.00	8.83E-03	0.01	0.01	1,073.52	1,211.48	1,349.04	2.82	3.19	3.55	0.00	0.00	0.00	0.00	0.00	0.00
Poland - Klebsiella pneumoniae 3GCRKP RESP - model 2	6.83	7.56	8.37	10.35	11.47	12.60	2,390.80	2,642.22	2,876.89	6.29	6.95	7.57	85.48	94.92	105.29	0.22	0.25	0.28
Poland - Klebsiella pneumoniae 3GCRKP SSI - model 4	1.28	1.45	1.64	1.29	1.46	1.65	1,074.84	1,192.83	1,339.51	2.83	3.14	3.52	22.95	26.08	30.00	0.06	0.07	0.08
Poland - Klebsiella pneumoniae 3GCRKP UTI - model 3	0.00	0.00	0.00	0.02	0.02	0.02	3,255.54	3,578.03	3,895.10	8.57	9.41	10.25	0.00	0.00	0.00	0.00	0.00	0.00
Poland - Klebsiella pneumoniae CRKP BSI - model 6	0.22	0.33	0.46	0.25	0.36	0.49	18.79	22.53	26.95	0.05	0.06	0.07	5.37	7.93	10.93	0.01	0.02	0.03
Poland - Klebsiella pneumoniae CRKP OTH - model 10	0.00	0.00	0.00	4.19E-05	9.48E-05	1.83E-04	6.52	10.64	15.47	0.02	0.03	0.04	0.00	0.00	0.00	0.00	0.00	0.00
Poland - Klebsiella pneumoniae CRKP RESP - model 7	7.27E-03	0.01	0.02	0.01	0.02	0.03	5.64	9.60	13.66	0.01	0.03	0.04	0.19	0.35	0.50	5.02E-04	9.15E-04	1.30E-03
Poland - Klebsiella pneumoniae CRKP SSI - model 9	3.22E-03	5.95E-03	8.76E-03	3.21E-03	5.96E-03	8.79E-03	3.68	6.58	9.52	9.68E-03	0.02	0.03	0.09	0.18	0.27	2.37E-04	4.67E-04	7.02E-04
Poland - Klebsiella pneumoniae CRKP UTI - model 8	0.00	0.00	0.00	3.74E-05	7.27E-05	1.30E-04	8.66	13.62	18.97	0.02	0.04	0.05	0.00	0.00	0.00	0.00	0.00	0.00
Poland - Klebsiella pneumoniae CoRKP BSI - model 11	2.69	3.47	4.37	2.85	3.63	4.54	80.98	90.38	100.61	0.21	0.24	0.26	42.96	54.01	66.84	0.11	0.14	0.18
Poland - Klebsiella pneumoniae CoRKP OTH - model 15	0.00	0.00	0.00	2.47E-04	3.84E-04	5.88E-04	30.64	41.66	53.14	0.08	0.11	0.14	0.00	0.00	0.00	0.00	0.00	0.00
Poland - Klebsiella pneumoniae CoRKP RESP - model 12	0.06	0.09	0.11	0.10	0.13	0.17	28.40	38.01	47.33	0.07	0.10	0.12	1.01	1.37	1.73	2.67E-03	3.59E-03	4.54E-03
Poland - Klebsiella pneumoniae CoRKP SSI - model 14	0.02	0.03	0.03	0.02	0.03	0.03	19.08	26.70	33.31	0.05	0.07	0.09	0.41	0.60	0.77	1.08E-03	1.59E-03	2.04E-03
Poland - Klebsiella pneumoniae CoRKP UTI - model 13	0.00	0.00	0.00	2.01E-04	2.98E-04	4.24E-04	42.46	54.92	67.33	0.11	0.14	0.18	0.00	0.00	0.00	0.00	0.00	0.00
Poland - Pseudomonas aeruginosa CRPA BSI - model 51	11.07	13.57	16.14	12.59	15.17	17.77	661.62	721.26	781.08	1.74	1.90	2.06	132.90	165.73	198.88	0.35	0.44	0.52
Poland - Pseudomonas aeruginosa CRPA OTH - model 55	0.00	0.00	0.00	2.68E-03	3.52E-03	4.48E-03	302.44	366.56	416.25	0.80	0.96	1.10	0.00	0.00	0.00	0.00	0.00	0.00
Poland - Pseudomonas aeruginosa CRPA RESP - model 52	11.84	14.40	17.00	15.57	18.49	21.31	2,388.97	2,671.59	2,967.88	6.29	7.03	7.81	143.12	175.69	208.94	0.38	0.46	0.55
Poland - Pseudomonas aeruginosa CRPA SSI - model 54	0.48	0.57	0.66	0.48	0.57	0.67	413.37	494.92	566.37	1.09	1.30	1.49	7.40	9.28	11.02	0.02	0.02	0.03
Poland - Pseudomonas aeruginosa CRPA UTI - model 53	0.00	0.00	0.00	3.97E-03	5.01E-03	6.39E-03	791.75	923.41	1,065.01	2.08	2.43	2.80	0.00	0.00	0.00	0.00	0.00	0.00
Poland - Pseudomonas aeruginosa CoRPA BSI - model 56	0.00	0.00	0.00	0.00	0.00	0.00	0.00	0.00	0.00	0.00	0.00	0.00	0.00	0.00	0.00	0.00	0.00	0.00
Poland - Pseudomonas aeruginosa CoRPA OTH - model 60	0.00	0.00	0.00	0.00	0.00	0.00	0.00	0.00	0.00	0.00	0.00	0.00	0.00	0.00	0.00	0.00	0.00	0.00
Poland - Pseudomonas aeruginosa CoRPA RESP - model 57	0.00	0.00	0.00	0.00	0.00	0.00	0.00	0.00	0.00	0.00	0.00	0.00	0.00	0.00	0.00	0.00	0.00	0.00
Poland - Pseudomonas aeruginosa CoRPA SSI - model 59	0.00	0.00	0.00	0.00	0.00	0.00	0.00	0.00	0.00	0.00	0.00	0.00	0.00	0.00	0.00	0.00	0.00	0.00
Poland - Pseudomonas aeruginosa CoRPA UTI - model 58	0.00	0.00	0.00	0.00	0.00	0.00	0.00	0.00	0.00	0.00	0.00	0.00	0.00	0.00	0.00	0.00	0.00	0.00
Poland - Pseudomonas aeruginosa MDRPA BSI - model 46	0.90	1.31	1.76	1.07	1.48	1.96	94.52	107.08	121.57	0.25	0.28	0.32	15.99	22.53	29.77	0.04	0.06	0.08
Poland - Pseudomonas aeruginosa MDRPA OTH - model 50	0.00	0.00	0.00	3.43E-04	5.39E-04	7.60E-04	39.63	54.48	69.47	0.10	0.14	0.18	0.00	0.00	0.00	0.00	0.00	0.00
Poland - Pseudomonas aeruginosa MDRPA RESP - model 47	1.01	1.44	1.94	1.39	1.87	2.43	326.12	397.37	469.75	0.86	1.05	1.24	17.96	24.81	32.53	0.05	0.07	0.09
Poland - Pseudomonas aeruginosa MDRPA SSI - model 49	0.06	0.08	0.11	0.06	0.09	0.11	54.87	73.29	92.06	0.14	0.19	0.24	1.31	1.79	2.25	3.46E-03	4.70E-03	5.92E-03
Poland - Pseudomonas aeruginosa MDRPA UTI - model 48	0.00	0.00	0.00	4.91E-04	7.33E-04	1.03E-03	102.78	137.40	168.63	0.27	0.36	0.44	0.00	0.00	0.00	0.00	0.00	0.00
Poland - Staphylococcus aureus MRSA BSI - model 61	12.48	13.19	13.87	14.44	15.15	15.90	1,055.06	1,102.88	1,150.65	2.78	2.90	3.03	187.34	197.76	209.20	0.49	0.52	0.55
Poland - Staphylococcus aureus MRSA OTH - model 65	0.00	0.00	0.00	0.01	0.01	0.02	1,500.25	1,626.76	1,751.19	3.95	4.28	4.61	0.00	0.00	0.00	0.00	0.00	0.00
Poland - Staphylococcus aureus MRSA RESP - model 62	3.64	3.99	4.32	5.52	6.03	6.51	1,521.26	1,660.27	1,805.96	4.00	4.37	4.75	54.14	59.76	65.40	0.14	0.16	0.17
Poland - Staphylococcus aureus MRSA SSI - model 64	1.91	2.10	2.29	1.92	2.11	2.30	1,663.73	1,804.40	1,948.39	4.38	4.75	5.13	40.26	44.78	49.15	0.11	0.12	0.13
Poland - Staphylococcus aureus MRSA UTI - model 63	0.00	0.00	0.00	1.02E-03	1.32E-03	1.65E-03	224.89	268.72	308.53	0.59	0.71	0.81	0.00	0.00	0.00	0.00	0.00	0.00
Poland - Streptococcus pneumoniae PMRSP BSI - model 76	0.69	0.76	0.83	0.79	0.87	0.95	42.86	42.86	42.86	0.11	0.11	0.11	7.16	7.72	8.31	0.02	0.02	0.02
Poland - Streptococcus pneumoniae PMRSP OTH - model 80	0.00	0.00	0.00	4.81E-05	7.66E-05	1.15E-04	13.36	15.21	17.45	0.04	0.04	0.05	0.00	0.00	0.00	0.00	0.00	0.00
Poland - Streptococcus pneumoniae PMRSP RESP - model 77	0.43	0.56	0.84	0.66	0.84	1.25	132.65	161.06	206.78	0.35	0.42	0.54	4.66	5.78	7.50	0.01	0.02	0.02
Poland - Streptococcus pneumoniae PMRSP SSI - model 79	0.00	0.00	0.00	0.00	0.00	0.00	0.00	0.00	0.00	0.00	0.00	0.00	0.00	0.00	0.00	0.00	0.00	0.00
Poland - Streptococcus pneumoniae PMRSP UTI - model 78	0.00	0.00	0.00	0.00	0.00	0.00	0.00	0.00	0.00	0.00	0.00	0.00	0.00	0.00	0.00	0.00	0.00	0.00
Poland - Streptococcus pneumoniae PRSP BSI - model 71	0.08	0.09	0.10	0.09	0.10	0.11	7.14	7.14	7.14	0.02	0.02	0.02	1.13	1.28	1.44	2.98E-03	3.38E-03	3.79E-03
Poland - Streptococcus pneumoniae PRSP OTH - model 75	0.00	0.00	0.00	5.16E-06	1.25E-05	2.49E-05	2.07	2.49	3.26	5.43E-03	6.55E-03	8.58E-03	0.00	0.00	0.00	0.00	0.00	0.00
Poland - Streptococcus pneumoniae PRSP RESP - model 72	0.04	0.06	0.11	0.07	0.09	0.16	19.89	25.81	42.16	0.05	0.07	0.11	0.66	0.93	1.56	1.74E-03	2.45E-03	4.09E-03
Poland - Streptococcus pneumoniae PRSP SSI - model 74	0.00	0.00	0.00	0.00	0.00	0.00	0.00	0.00	0.00	0.00	0.00	0.00	0.00	0.00	0.00	0.00	0.00	0.00
Poland - Streptococcus pneumoniae PRSP UTI - model 73	0.00	0.00	0.00	0.00	0.00	0.00	0.00	0.00	0.00	0.00	0.00	0.00	0.00	0.00	0.00	0.00	0.00	0.00
**Total (Median & 95% UIs)**	137.38	156.72	177.41	164.98	186.52	209.11	36,826.17	41,069.36	45,337.54	96.90	108.06	119.29	1,930.50	2,218.00	2,519.22	5.08	5.84	6.63
**Total (average)**		**156.83**			**186.60**			**41,076.10**			**108.08**			**2,219.88**			**5.84**	

Infection	YLD			YLL			DALY			DALY per Case		
Colistin resistant *Acinetobacter spp.*	5.22	6.69	8.29	22.05	35.25	50.91	27.95	42.03	58.13	6.65	9.32	12.25
Carbapenem resistant *Acinetobacter spp.* (excluding those resistant to colistin)	190.43	211.50	232.95	944.07	1,131.38	1,317.73	1,142.70	1,342.32	1,537.58	5.95	6.87	7.77
Aminoglycosides and fluoroquinolones resistant *Acinetobacter* spp. (excluding those resistant to colistin and/or carbapenem)	2.12	3.32	4.98	7.13	17.37	30.31	9.93	20.66	34.65	10.12	18.43	26.84
Vancomycin resistant Enterococci (*E. faecalis* and *E. faecium*)	82.57	99.22	116.83	787.19	931.73	1,097.86	868.78	1,031.11	1,214.94	5.91	6.58	7.25
Colistin resistant *Escherichia coli*	0.00	0.00	0.00	0.00	0.00	0.00	0.00	0.00	0.00	0.00	0.00	0.00
Carbapenem resistant *Escherichia coli* (excluding those resistant to colistin)	5.47	7.27	9.59	54.03	77.47	104.57	60.05	84.71	113.37	12.21	16.35	21.13
Third-generation cephalosporin resistant *Escherichia coli* (excluding those resistant to colistin and/or carbapenem)	464.10	519.27	576.82	2,800.09	3,107.39	3,477.27	3,285.82	3,624.68	4,039.62	3.94	4.28	4.65
Colistin resistant *Klebsiella pneumoniae*	0.99	1.49	2.10	15.38	25.93	38.67	16.30	27.37	40.79	8.81	13.43	18.33
Carbapenem resistant *Klebsiella pneumoniae* (excluding those resistant to colistin)	54.23	62.18	70.59	568.66	667.21	772.66	625.72	729.18	837.80	9.48	10.71	11.93
Third-generation cephalosporin resistant *Klebsiella pneumoniae* (excluding those resistant to colistin and/or carbapenem)	663.34	725.09	788.31	3,261.20	3,478.45	3,690.54	3,939.60	4,203.28	4,464.47	5.27	5.52	5.80
Colistin resistant *Pseudomonas aeruginosa*	8.02	11.16	14.92	30.43	56.11	88.64	40.33	67.23	102.56	7.67	12.30	17.35
Carbapenem resistant *Pseudomonas aeruginosa* (excluding those resistant to colistin)	543.51	610.89	678.87	2,511.59	3,085.29	3,663.15	3,095.19	3,697.41	4,305.79	7.29	8.61	9.85
Pseudomonas aeruginosa resistant to three or more antimicrobial groups (excluding those resistant to colistin and/or carbapenem)	0.00	0.00	0.00	0.00	0.00	0.00	0.00	0.00	0.00	0.00	0.00	0.00
Methicillin resistant *Staphylococcus aureus*	1.520.11	1.663.80	1.814.19	7.634.02	8.157.09	8.707.45	9.176.47	9.817.87	10.473.09	4.51	4.73	4.97
Penicillin and macrolides resistant *Streptococcus pneumoniae* (excluding those only resistant to penicillin)	39.35	45.03	52.62	135.08	147.61	163.07	176.15	192.67	213.38	6.82	7.21	7.64
Penicillin tesistant *Streptococcus pneumoniae* (excluding those resistant to macrolides)	42.50	49.26	57.91	146.84	162.27	180.79	191.12	211.39	236.58	8.53	9.06	9.62
Total	3,621.96	4,016.17	4,428.98	18,917.76	21,080.58	23,383.62	22,656.12	25,091.92	27,672.77			

Infection	YLD per 100,000			YLL per 100,000			DALY per 100,000			Cases per year			Incidence per 100,000			Deaths per year			Mortality per 100,000		
Colistin resistant *Acinetobacter spp.*	0.05	0.06	0.08	0.21	0.34	0.49	0.27	0.41	0.56	10.33	13.15	15.95	0.10	0.13	0.15	0.72	1.13	1.55	0.01	0.01	0.01
Carbapenem resistant *Acinetobacter spp.* (excluding those resistant to colistin)	1.84	2.04	2.25	9.10	10.91	12.70	11.01	12.94	14.82	518.02	578.28	635.88	4.99	5.57	6.13	41.93	50.68	59.29	0.40	0.49	0.57
Aminoglycosides and fluoroquinolones resistant *Acinetobacter* spp. (excluding those resistant to colistin and/or carbapenem)	0.02	0.03	0.05	0.07	0.17	0.29	0.10	0.20	0.33	1.99	3.27	4.70	0.02	0.03	0.05	0.11	0.27	0.47	0.00	0.00	0.00
Vancomycin resistant Enterococci (*E. faecalis* and *E. faecium*)	0.80	0.96	1.13	7.59	8.98	10.58	8.37	9.94	11.71	495.79	605.57	716.40	4.78	5.84	6.91	33.83	40.35	47.71	0.33	0.39	0.46
Colistin resistant *Escherichia coli*	0.00	0.00	0.00	0.00	0.00	0.00	0.00	0.00	0.00	0.00	0.00	0.00	0.00	0.00	0.00	0.00	0.00	0.00	0.00	0.00	0.00
Carbapenem resistant *Escherichia coli* (excluding those resistant to colistin)	0.05	0.07	0.09	0.52	0.75	1.01	0.58	0.82	1.09	32.04	41.31	50.76	0.31	0.40	0.49	1.56	2.19	2.90	0.02	0.02	0.03
Third-generation cephalosporin resistant *Escherichia coli* (excluding those resistant to colistin and/or carbapenem)	4.47	5.01	5.56	26.99	29.95	33.52	31.67	34.94	38.94	5,890.48	6,754.39	7,599.84	56.78	65.10	73.25	182.89	210.48	242.31	1.76	2.03	2.34
Colistin resistant *Klebsiella pneumoniae*	0.01	0.01	0.02	0.15	0.25	0.37	0.16	0.26	0.39	3.75	6.12	8.53	0.04	0.06	0.08	0.82	1.36	2.00	0.01	0.01	0.02
Carbapenem resistant *Klebsiella pneumoniae* (excluding those resistant to colistin)	0.52	0.60	0.68	5.48	6.43	7.45	6.03	7.03	8.08	183.24	210.45	238.84	1.77	2.03	2.30	24.70	28.60	32.82	0.24	0.28	0.32
Third-generation cephalosporin resistant *Klebsiella pneumoniae* (excluding those resistant to colistin and/or carbapenem)	6.39	6.99	7.60	31.43	33.53	35.57	37.97	40.51	43.03	2,842.12	3,142.56	3,436.49	27.39	30.29	33.12	158.99	171.66	184.92	1.53	1.65	1.78
Colistin resistant *Pseudomonas aeruginosa*	0.08	0.11	0.14	0.29	0.54	0.85	0.39	0.65	0.99	16.95	24.17	31.43	0.16	0.23	0.30	0.90	1.62	2.51	0.01	0.02	0.02
Carbapenem resistant *Pseudomonas aeruginosa* (excluding those resistant to colistin)	5.24	5.89	6.54	24.21	29.74	35.31	29.83	35.64	41.50	1,670.15	1,901.37	2,131.29	16.10	18.33	20.54	104.37	129.06	155.27	1.01	1.24	1.50
Pseudomonas aeruginosa resistant to three or more antimicrobial groups (excluding those resistant to colistin and/or carbapenem)	0.00	0.00	0.00	0.00	0.00	0.00	0.00	0.00	0.00	0.00	0.00	0.00	0.00	0.00	0.00	0.00	0.00	0.00	0.00	0.00	0.00
Methicillin resistant *Staphylococcus aureus*	14.65	16.04	17.49	73.58	78.62	83.93	88.45	94.63	100.95	9.588.43	10.577.74	11.558.01	92.42	101.96	111.40	468.54	510.92	554.22	4.52	4.92	5.34
Penicillin and macrolides resistant *Streptococcus pneumoniae* (excluding those only resistant to penicillin)	0.38	0.43	0.51	1.30	1.42	1.57	1.70	1.86	2.06	79.57	86.99	96.83	0.77	0.84	0.93	4.95	5.35	5.81	0.05	0.05	0.06
Penicillin tesistant *Streptococcus pneumoniae* (excluding those resistant to macrolides)	0.41	0.47	0.56	1.42	1.56	1.74	1.84	2.04	2.28	68.67	75.97	87.05	0.66	0.73	0.84	4.27	4.67	5.15	0.04	0.05	0.05
Total	34.91	38.71	42.69	182.34	203.19	225.39	218.38	241.85	266.73	21,401.52	24,021.33	26,611.99	206.28	231.53	256.51	1,028.59	1,158.34	1,296.95	9.91	11.16	12.50

Model	YLD			YLL			DALY			DALY per Case		
Portugal - Acinetobacter spp. CRACI BSI - model 36	110.10	120.81	131.97	762.51	926.12	1,087.97	879.90	1,046.50	1,209.40	4.40	5.23	6.01
Portugal - Acinetobacter spp. CRACI OTH - model 40	0.02	0.03	0.03	0.00	0.00	0.00	0.02	0.03	0.03	2.97E-03	3.75E-03	4.47E-03
Portugal - Acinetobacter spp. CRACI RESP - model 37	80.07	90.34	100.53	156.25	175.27	195.00	237.25	265.48	293.00	1.15	1.23	1.31
Portugal - Acinetobacter spp. CRACI SSI - model 39	0.11	0.15	0.21	25.31	29.98	34.76	25.41	30.15	34.94	0.39	0.41	0.44
Portugal - Acinetobacter spp. CRACI UTI - model 38	0.13	0.17	0.21	0.00	0.00	0.00	0.13	0.17	0.21	1.67E-03	2.05E-03	2.49E-03
Portugal - Acinetobacter spp. ColRACI BSI - model 41	3.09	3.82	4.69	17.41	29.00	42.98	21.06	32.89	46.64	4.77	7.22	9.91
Portugal - Acinetobacter spp. ColRACI OTH - model 45	2.64E-04	5.60E-04	1.02E-03	0.00	0.00	0.00	2.64E-04	5.60E-04	1.02E-03	2.27E-03	3.70E-03	5.11E-03
Portugal - Acinetobacter spp. ColRACI RESP - model 42	2.13	2.85	3.59	4.21	5.63	7.10	6.47	8.51	10.66	1.54	1.72	1.93
Portugal - Acinetobacter spp. ColRACI SSI - model 44	1.77E-03	3.47E-03	6.07E-03	0.42	0.62	0.82	0.42	0.62	0.83	0.35	0.37	0.40
Portugal - Acinetobacter spp. ColRACI UTI - model 43	2.06E-03	3.74E-03	6.03E-03	0.00	0.00	0.00	2.06E-03	3.74E-03	6.03E-03	1.28E-03	2.08E-03	2.97E-03
Portugal - Acinetobacter spp. MDRACI BSI - model 31	1.27	1.89	2.82	5.38	14.33	25.78	7.28	16.16	28.04	6.54	14.38	22.29
Portugal - Acinetobacter spp. MDRACI OTH - model 35	1.86E-05	1.25E-04	3.97E-04	0.00	0.00	0.00	1.86E-05	1.25E-04	3.97E-04	1.58E-03	3.69E-03	6.51E-03
Portugal - Acinetobacter spp. MDRACI RESP - model 32	0.85	1.43	2.16	1.65	2.80	4.15	2.55	4.26	6.23	3.00	3.46	3.96
Portugal - Acinetobacter spp. MDRACI SSI - model 34	1.62E-04	7.95E-04	2.20E-03	0.10	0.24	0.38	0.10	0.24	0.38	0.58	0.58	0.58
Portugal - Acinetobacter spp. MDRACI UTI - model 33	2.41E-04	8.46E-04	2.13E-03	0.00	0.00	0.00	2.41E-04	8.46E-04	2.13E-03	7.90E-04	1.96E-03	3.72E-03
Portugal - Enterococcus faecalis and E. faecium VRE BSI - model 66	81.95	98.36	115.67	715.88	842.46	990.18	796.75	940.95	1,106.45	5.53	6.14	6.78
Portugal - Enterococcus faecalis and E. faecium VRE OTH - model 70	0.20	0.27	0.35	0.00	0.00	0.00	0.20	0.27	0.35	1.31E-03	1.65E-03	2.01E-03
Portugal - Enterococcus faecalis and E. faecium VRE RESP - model 67	0.00	0.00	0.00	0.00	0.00	0.00	0.00	0.00	0.00	NaN	NaN	NaN
Portugal - Enterococcus faecalis and E. faecium VRE SSI - model 69	0.32	0.45	0.62	71.32	89.27	107.68	71.72	89.75	107.95	0.38	0.43	0.47
Portugal - Enterococcus faecalis and E. faecium VRE UTI - model 68	0.11	0.15	0.19	0.00	0.00	0.00	0.11	0.15	0.19	1.55E-03	1.87E-03	2.30E-03
Portugal - Escherichia coli 3GCREC BSI - model 16	354.87	389.14	425.50	2,278.69	2,502.81	2,783.29	2,653.81	2,890.12	3,196.92	2.83	3.06	3.30
Portugal - Escherichia coli 3GCREC OTH - model 20	0.76	1.04	1.41	0.00	0.00	0.00	0.76	1.04	1.41	2.59E-03	3.34E-03	4.13E-03
Portugal - Escherichia coli 3GCREC RESP - model 17	100.75	119.00	136.93	193.58	228.36	263.50	296.81	347.19	398.03	0.76	0.83	0.94
Portugal - Escherichia coli 3GCREC SSI - model 19	1.53	2.12	2.87	327.82	376.22	430.48	328.25	378.35	433.14	0.36	0.38	0.41
Portugal - Escherichia coli 3GCREC UTI - model 18	6.19	7.97	10.12	0.00	0.00	0.00	6.19	7.97	10.12	1.53E-03	1.97E-03	2.41E-03
Portugal - Escherichia coli CREC BSI - model 21	4.28	5.35	6.89	49.65	71.04	96.00	54.49	76.37	102.16	10.32	13.94	18.06
Portugal - Escherichia coli CREC OTH - model 25	5.81E-03	0.01	0.02	0.00	0.00	0.00	5.81E-03	0.01	0.02	2.08E-03	3.43E-03	5.42E-03
Portugal - Escherichia coli CREC RESP - model 22	1.15	1.84	2.59	2.31	3.63	5.02	3.44	5.46	7.54	1.49	1.97	2.59
Portugal - Escherichia coli CREC SSI - model 24	7.16E-03	0.01	0.02	2.07	2.80	3.55	2.09	2.82	3.57	0.39	0.43	0.47
Portugal - Escherichia coli CREC UTI - model 23	0.03	0.05	0.08	0.00	0.00	0.00	0.03	0.05	0.08	1.29E-03	2.07E-03	3.07E-03
Portugal - Escherichia coli ColREC BSI - model 26	0.00	0.00	0.00	0.00	0.00	0.00	0.00	0.00	0.00	NaN	NaN	NaN
Portugal - Escherichia coli ColREC OTH - model 30	0.00	0.00	0.00	0.00	0.00	0.00	0.00	0.00	0.00	NaN	NaN	NaN
Portugal - Escherichia coli ColREC RESP - model 27	0.00	0.00	0.00	0.00	0.00	0.00	0.00	0.00	0.00	NaN	NaN	NaN
Portugal - Escherichia coli ColREC SSI - model 29	0.00	0.00	0.00	0.00	0.00	0.00	0.00	0.00	0.00	NaN	NaN	NaN
Portugal - Escherichia coli ColREC UTI - model 28	0.00	0.00	0.00	0.00	0.00	0.00	0.00	0.00	0.00	NaN	NaN	NaN
Portugal - Klebsiella pneumoniae 3GCRKP BSI - model 1	416.62	449.31	484.87	2,672.70	2,819.52	2,963.21	3,100.74	3,268.83	3,438.33	3.82	3.98	4.15
Portugal - Klebsiella pneumoniae 3GCRKP OTH - model 5	0.84	1.09	1.34	0.00	0.00	0.00	0.84	1.09	1.34	2.71E-03	3.35E-03	4.01E-03
Portugal - Klebsiella pneumoniae 3GCRKP RESP - model 2	243.87	272.12	298.88	471.83	525.44	574.72	719.27	797.32	869.10	1.05	1.12	1.20
Portugal - Klebsiella pneumoniae 3GCRKP SSI - model 4	0.51	0.68	0.90	116.67	133.50	152.61	117.25	134.15	153.38	0.39	0.42	0.44
Portugal - Klebsiella pneumoniae 3GCRKP UTI - model 3	1.50	1.90	2.32	0.00	0.00	0.00	1.50	1.90	2.32	1.63E-03	1.97E-03	2.34E-03
Portugal - Klebsiella pneumoniae CRKP BSI - model 6	43.01	48.08	53.45	540.00	631.55	729.72	586.27	679.39	778.04	7.95	9.03	10.08
Portugal - Klebsiella pneumoniae CRKP OTH - model 10	0.09	0.12	0.17	0.00	0.00	0.00	0.09	0.12	0.17	2.79E-03	3.52E-03	4.35E-03
Portugal - Klebsiella pneumoniae CRKP RESP - model 7	11.02	13.83	16.78	21.49	26.71	32.40	32.09	40.56	48.87	1.16	1.28	1.40
Portugal - Klebsiella pneumoniae CRKP SSI - model 9	0.03	0.05	0.06	7.16	8.96	10.54	7.20	9.00	10.59	0.37	0.40	0.44
Portugal - Klebsiella pneumoniae CRKP UTI - model 8	0.07	0.10	0.12	0.00	0.00	0.00	0.07	0.10	0.12	1.69E-03	2.10E-03	2.58E-03
Portugal - Klebsiella pneumoniae ColRKP BSI - model 11	0.84	1.15	1.56	14.99	25.07	37.33	15.75	26.18	38.93	7.68	12.03	16.62
Portugal - Klebsiella pneumoniae ColRKP OTH - model 15	1.25E-03	3.24E-03	7.05E-03	0.00	0.00	0.00	1.25E-03	3.24E-03	7.05E-03	1.66E-03	3.29E-03	6.12E-03
Portugal - Klebsiella pneumoniae ColRKP RESP - model 12	0.15	0.33	0.52	0.29	0.63	0.99	0.45	0.96	1.51	0.85	1.05	1.30
Portugal - Klebsiella pneumoniae ColRKP SSI - model 14	4.79E-04	1.33E-03	3.01E-03	0.10	0.23	0.35	0.10	0.23	0.35	0.28	0.35	0.41
Portugal - Klebsiella pneumoniae ColRKP UTI - model 13	1.12E-03	2.66E-03	5.20E-03	0.00	0.00	0.00	1.12E-03	2.66E-03	5.20E-03	1.03E-03	2.04E-03	3.42E-03
Portugal - Pseudomonas aeruginosa CRPA BSI - model 51	154.36	170.82	188.74	1,183.70	1,448.04	1,726.48	1,348.19	1,618.79	1,906.91	5.12	6.14	7.07
Portugal - Pseudomonas aeruginosa CRPA OTH - model 55	0.38	0.49	0.61	0.00	0.00	0.00	0.38	0.49	0.61	3.02E-03	3.63E-03	4.34E-03
Portugal - Pseudomonas aeruginosa CRPA RESP - model 52	387.95	438.51	488.15	1,265.74	1,560.71	1,846.81	1,683.57	2,000.53	2,307.14	1.77	2.04	2.32
Portugal - Pseudomonas aeruginosa CRPA SSI - model 54	0.29	0.39	0.51	62.15	76.55	89.86	62.52	76.91	90.26	0.40	0.42	0.45
Portugal - Pseudomonas aeruginosa CRPA UTI - model 53	0.54	0.69	0.86	0.00	0.00	0.00	0.54	0.69	0.86	1.68E-03	2.06E-03	2.43E-03
Portugal - Pseudomonas aeruginosa ColRPA BSI - model 56	2.43	3.17	4.15	14.04	26.93	42.14	17.07	30.06	45.98	5.17	8.88	12.82
Portugal - Pseudomonas aeruginosa ColRPA OTH - model 60	3.02E-03	6.28E-03	0.01	0.00	0.00	0.00	3.02E-03	6.28E-03	0.01	2.14E-03	3.71E-03	5.36E-03
Portugal - Pseudomonas aeruginosa ColRPA RESP - model 57	5.57	7.97	10.73	15.78	28.11	45.00	22.64	36.08	55.06	2.07	2.95	4.01
Portugal - Pseudomonas aeruginosa ColRPA SSI - model 59	2.05E-03	4.71E-03	9.17E-03	0.62	1.07	1.49	0.62	1.08	1.50	0.42	0.47	0.51
Portugal - Pseudomonas aeruginosa ColRPA UTI - model 58	4.41E-03	8.60E-03	0.01	0.00	0.00	0.00	4.41E-03	8.60E-03	0.01	1.19E-03	2.00E-03	3.03E-03
Portugal - Pseudomonas aeruginosa MDRPA BSI - model 46	0.00	0.00	0.00	0.00	0.00	0.00	0.00	0.00	0.00	NaN	NaN	NaN
Portugal - Pseudomonas aeruginosa MDRPA OTH - model 50	0.00	0.00	0.00	0.00	0.00	0.00	0.00	0.00	0.00	NaN	NaN	NaN
Portugal - Pseudomonas aeruginosa MDRPA RESP - model 47	0.00	0.00	0.00	0.00	0.00	0.00	0.00	0.00	0.00	NaN	NaN	NaN
Portugal - Pseudomonas aeruginosa MDRPA SSI - model 49	0.00	0.00	0.00	0.00	0.00	0.00	0.00	0.00	0.00	NaN	NaN	NaN
Portugal - Pseudomonas aeruginosa MDRPA UTI - model 48	0.00	0.00	0.00	0.00	0.00	0.00	0.00	0.00	0.00	NaN	NaN	NaN
Portugal - Staphylococcus aureus MRSA BSI - model 61	748.90	805.09	869.86	5,119.85	5,390.26	5,689.33	5,888.91	6,193.00	6,522.76	3.29	3.43	3.59
Portugal - Staphylococcus aureus MRSA OTH - model 65	6.92	8.67	10.74	0.00	0.00	0.00	6.92	8.67	10.74	2.66E-03	3.26E-03	4.00E-03
Portugal - Staphylococcus aureus MRSA RESP - model 62	758.95	842.92	924.04	1,477.93	1,620.37	1,760.88	2,235.24	2,462.49	2,674.89	0.85	0.91	0.97
Portugal - Staphylococcus aureus MRSA SSI - model 64	4.71	6.30	8.47	1,036.24	1,146.46	1,257.24	1,044.78	1,152.90	1,263.63	0.37	0.39	0.41
Portugal - Staphylococcus aureus MRSA UTI - model 63	0.61	0.82	1.08	0.00	0.00	0.00	0.61	0.82	1.08	1.49E-03	1.87E-03	2.29E-03
Portugal - Streptococcus pneumoniae PMRSP BSI - model 76	11.13	12.31	13.51	79.76	83.68	87.56	91.96	95.95	99.96	5.47	5.70	5.94
Portugal - Streptococcus pneumoniae PMRSP OTH - model 80	9.29E-03	0.01	0.01	0.00	0.00	0.00	9.29E-03	0.01	0.01	1.56E-03	1.97E-03	2.38E-03
Portugal - Streptococcus pneumoniae PMRSP RESP - model 77	28.21	32.71	39.10	55.32	63.93	75.51	84.18	96.71	113.41	1.35	1.51	1.70
Portugal - Streptococcus pneumoniae PMRSP SSI - model 79	0.00	0.00	0.00	0.00	0.00	0.00	0.00	0.00	0.00	NaN	NaN	NaN
Portugal - Streptococcus pneumoniae PMRSP UTI - model 78	0.00	0.00	0.00	0.00	0.00	0.00	0.00	0.00	0.00	NaN	NaN	NaN
Portugal - Streptococcus pneumoniae PRSP BSI - model 71	12.09	13.54	15.06	86.86	92.05	96.63	100.54	105.52	110.56	6.83	7.17	7.51
Portugal - Streptococcus pneumoniae PRSP OTH - model 75	7.84E-03	0.01	0.01	0.00	0.00	0.00	7.84E-03	0.01	0.01	1.47E-03	1.95E-03	2.49E-03
Portugal - Streptococcus pneumoniae PRSP RESP - model 72	30.40	35.72	42.83	59.98	70.23	84.16	90.57	105.85	126.01	1.70	1.89	2.11
Portugal - Streptococcus pneumoniae PRSP SSI - model 74	0.00	0.00	0.00	0.00	0.00	0.00	0.00	0.00	0.00	NaN	NaN	NaN
Portugal - Streptococcus pneumoniae PRSP UTI - model 73	0.00	0.00	0.00	0.00	0.00	0.00	0.00	0.00	0.00	NaN	NaN	NaN
**Total (Median & 95% UIs)**	3,621.96	4,016.17	4,428.98	18,917.76	21,080.58	23,383.62	22,656.12	25,091.92	27,672.77		-	
**Total (average)**		**4,019.65**			**21,094.33**			**25,113.98**			-	

Model	YLD per 100,000			YLL per 100,000			DALY per 100,000			Cases per year			Incidence per 100,000			Deaths per year			Mortality per 100,000		
Portugal - Acinetobacter spp. CRACI BSI - model 36	1.06	1.16	1.27	7.35	8.93	10.49	8.48	10.09	11.66	188.74	200.47	212.44	1.82	1.93	2.05	33.52	41.06	48.46	0.32	0.40	0.47
Portugal - Acinetobacter spp. CRACI OTH - model 40	1.80E-04	2.54E-04	3.30E-04	0.00	0.00	0.00	1.80E-04	2.54E-04	3.30E-04	5.33	7.07	8.74	0.05	0.07	0.08	0.00	0.00	0.00	0.00	0.00	0.00
Portugal - Acinetobacter spp. CRACI RESP - model 37	0.77	0.87	0.97	1.51	1.69	1.88	2.29	2.56	2.82	192.97	216.37	236.85	1.86	2.09	2.28	6.90	7.77	8.65	0.07	0.07	0.08
Portugal - Acinetobacter spp. CRACI SSI - model 39	1.09E-03	1.49E-03	1.99E-03	0.24	0.29	0.34	0.24	0.29	0.34	61.72	73.18	83.64	0.59	0.71	0.81	1.50	1.85	2.19	0.01	0.02	0.02
Portugal - Acinetobacter spp. CRACI UTI - model 38	1.22E-03	1.60E-03	2.03E-03	0.00	0.00	0.00	1.22E-03	1.60E-03	2.03E-03	69.25	81.18	94.21	0.67	0.78	0.91	0.00	0.00	0.00	0.00	0.00	0.00
Portugal - Acinetobacter spp. ColRACI BSI - model 41	0.03	0.04	0.05	0.17	0.28	0.41	0.20	0.32	0.45	4.04	4.55	5.14	0.04	0.04	0.05	0.57	0.92	1.30	5.52E-03	8.89E-03	0.01
Portugal - Acinetobacter spp. ColRACI OTH - model 45	2.55E-06	5.40E-06	9.86E-06	0.00	0.00	0.00	2.55E-06	5.40E-06	9.86E-06	0.09	0.16	0.24	8.37E-04	1.50E-03	2.28E-03	0.00	0.00	0.00	0.00	0.00	0.00
Portugal - Acinetobacter spp. ColRACI RESP - model 42	0.02	0.03	0.03	0.04	0.05	0.07	0.06	0.08	0.10	3.82	4.93	5.93	0.04	0.05	0.06	0.13	0.18	0.22	1.30E-03	1.72E-03	2.10E-03
Portugal - Acinetobacter spp. ColRACI SSI - model 44	1.71E-05	3.35E-05	5.85E-05	4.03E-03	5.96E-03	7.95E-03	4.05E-03	6.00E-03	8.00E-03	1.13	1.67	2.22	0.01	0.02	0.02	0.02	0.03	0.04	1.53E-04	2.53E-04	3.45E-04
Portugal - Acinetobacter spp. ColRACI UTI - model 43	1.99E-05	3.60E-05	5.82E-05	0.00	0.00	0.00	1.99E-05	3.60E-05	5.82E-05	1.25	1.84	2.42	0.01	0.02	0.02	0.00	0.00	0.00	0.00	0.00	0.00
Portugal - Acinetobacter spp. MDRACI BSI - model 31	0.01	0.02	0.03	0.05	0.14	0.25	0.07	0.16	0.27	0.90	1.13	1.45	8.70E-03	0.01	0.01	0.08	0.22	0.40	8.12E-04	2.16E-03	3.89E-03
Portugal - Acinetobacter spp. MDRACI OTH - model 35	1.79E-07	1.21E-06	3.83E-06	0.00	0.00	0.00	1.79E-07	1.21E-06	3.83E-06	6.18E-03	0.04	0.09	5.96E-05	3.72E-04	8.42E-04	0.00	0.00	0.00	0.00	0.00	0.00
Portugal - Acinetobacter spp. MDRACI RESP - model 32	8.15E-03	0.01	0.02	0.02	0.03	0.04	0.02	0.04	0.06	0.73	1.23	1.76	7.03E-03	0.01	0.02	0.03	0.04	0.06	2.49E-04	4.22E-04	6.26E-04
Portugal - Acinetobacter spp. MDRACI SSI - model 34	1.56E-06	7.66E-06	2.12E-05	9.59E-04	2.32E-03	3.66E-03	9.59E-04	2.33E-03	3.67E-03	0.17	0.42	0.66	1.67E-03	4.04E-03	6.37E-03	1.56E-03	3.77E-03	5.95E-03	1.50E-05	3.63E-05	5.74E-05
Portugal - Acinetobacter spp. MDRACI UTI - model 33	2.32E-06	8.15E-06	2.05E-05	0.00	0.00	0.00	2.32E-06	8.15E-06	2.05E-05	0.18	0.46	0.75	1.73E-03	4.40E-03	7.19E-03	0.00	0.00	0.00	0.00	0.00	0.00
Portugal - Enterococcus faecalis and E. faecium VRE BSI - model 66	0.79	0.95	1.11	6.90	8.12	9.54	7.68	9.07	10.66	130.06	153.32	180.76	1.25	1.48	1.74	29.74	35.04	41.22	0.29	0.34	0.40
Portugal - Enterococcus faecalis and E. faecium VRE OTH - model 70	1.92E-03	2.59E-03	3.38E-03	0.00	0.00	0.00	1.92E-03	2.59E-03	3.38E-03	132.68	164.16	192.21	1.28	1.58	1.85	0.00	0.00	0.00	0.00	0.00	0.00
Portugal - Enterococcus faecalis and E. faecium VRE RESP - model 67	0.00	0.00	0.00	0.00	0.00	0.00	0.00	0.00	0.00	0.00	0.00	0.00	0.00	0.00	0.00	0.00	0.00	0.00	0.00	0.00	0.00
Portugal - Enterococcus faecalis and E. faecium VRE SSI - model 69	3.12E-03	4.30E-03	5.96E-03	0.69	0.86	1.04	0.69	0.87	1.04	171.78	210.10	249.76	1.66	2.03	2.41	4.10	5.31	6.50	0.04	0.05	0.06
Portugal - Enterococcus faecalis and E. faecium VRE UTI - model 68	1.02E-03	1.41E-03	1.86E-03	0.00	0.00	0.00	1.02E-03	1.41E-03	1.86E-03	61.27	77.99	93.67	0.59	0.75	0.90	0.00	0.00	0.00	0.00	0.00	0.00
Portugal - Escherichia coli 3GCREC BSI - model 16	3.42	3.75	4.10	21.96	24.12	26.83	25.58	27.86	30.81	871.37	948.27	1,028.08	8.40	9.14	9.91	144.75	164.50	188.06	1.40	1.59	1.81
Portugal - Escherichia coli 3GCREC OTH - model 20	7.31E-03	0.01	0.01	0.00	0.00	0.00	7.31E-03	0.01	0.01	249.80	313.85	378.16	2.41	3.03	3.64	0.00	0.00	0.00	0.00	0.00	0.00
Portugal - Escherichia coli 3GCREC RESP - model 17	0.97	1.15	1.32	1.87	2.20	2.54	2.86	3.35	3.84	340.93	417.38	492.96	3.29	4.02	4.75	12.22	14.97	17.93	0.12	0.14	0.17
Portugal - Escherichia coli 3GCREC SSI - model 19	0.01	0.02	0.03	3.16	3.63	4.15	3.16	3.65	4.17	850.50	995.02	1,144.37	8.20	9.59	11.03	25.92	31.02	36.32	0.25	0.30	0.35
Portugal - Escherichia coli 3GCREC UTI - model 18	0.06	0.08	0.10	0.00	0.00	0.00	0.06	0.08	0.10	3,577.88	4,079.86	4,556.27	34.49	39.32	43.92	0.00	0.00	0.00	0.00	0.00	0.00
Portugal - Escherichia coli CREC BSI - model 21	0.04	0.05	0.07	0.48	0.68	0.93	0.53	0.74	0.98	4.71	5.50	6.31	0.05	0.05	0.06	1.41	1.96	2.59	0.01	0.02	0.02
Portugal - Escherichia coli CREC OTH - model 25	5.60E-05	1.06E-04	1.75E-04	0.00	0.00	0.00	5.60E-05	1.06E-04	1.75E-04	2.23	3.18	4.09	0.02	0.03	0.04	0.00	0.00	0.00	0.00	0.00	0.00
Portugal - Escherichia coli CREC RESP - model 22	0.01	0.02	0.02	0.02	0.03	0.05	0.03	0.05	0.07	1.80	2.78	3.71	0.02	0.03	0.04	0.06	0.10	0.14	6.24E-04	9.56E-04	1.31E-03
Portugal - Escherichia coli CREC SSI - model 24	6.90E-05	1.31E-04	2.28E-04	0.02	0.03	0.03	0.02	0.03	0.03	4.82	6.55	8.26	0.05	0.06	0.08	0.08	0.13	0.17	8.19E-04	1.24E-03	1.68E-03
Portugal - Escherichia coli CREC UTI - model 23	2.74E-04	4.61E-04	7.32E-04	0.00	0.00	0.00	2.74E-04	4.61E-04	7.32E-04	18.48	23.30	28.39	0.18	0.22	0.27	0.00	0.00	0.00	0.00	0.00	0.00
Portugal - Escherichia coli ColREC BSI - model 26	0.00	0.00	0.00	0.00	0.00	0.00	0.00	0.00	0.00	0.00	0.00	0.00	0.00	0.00	0.00	0.00	0.00	0.00	0.00	0.00	0.00
Portugal - Escherichia coli ColREC OTH - model 30	0.00	0.00	0.00	0.00	0.00	0.00	0.00	0.00	0.00	0.00	0.00	0.00	0.00	0.00	0.00	0.00	0.00	0.00	0.00	0.00	0.00
Portugal - Escherichia coli ColREC RESP - model 27	0.00	0.00	0.00	0.00	0.00	0.00	0.00	0.00	0.00	0.00	0.00	0.00	0.00	0.00	0.00	0.00	0.00	0.00	0.00	0.00	0.00
Portugal - Escherichia coli ColREC SSI - model 29	0.00	0.00	0.00	0.00	0.00	0.00	0.00	0.00	0.00	0.00	0.00	0.00	0.00	0.00	0.00	0.00	0.00	0.00	0.00	0.00	0.00
Portugal - Escherichia coli ColREC UTI - model 28	0.00	0.00	0.00	0.00	0.00	0.00	0.00	0.00	0.00	0.00	0.00	0.00	0.00	0.00	0.00	0.00	0.00	0.00	0.00	0.00	0.00
Portugal - Klebsiella pneumoniae 3GCRKP BSI - model 1	4.02	4.33	4.67	25.76	27.18	28.56	29.89	31.51	33.14	779.06	821.97	864.55	7.51	7.92	8.33	128.99	137.28	146.39	1.24	1.32	1.41
Portugal - Klebsiella pneumoniae 3GCRKP OTH - model 5	8.12E-03	0.01	0.01	0.00	0.00	0.00	8.12E-03	0.01	0.01	283.61	325.03	366.27	2.73	3.13	3.53	0.00	0.00	0.00	0.00	0.00	0.00
Portugal - Klebsiella pneumoniae 3GCRKP RESP - model 2	2.35	2.62	2.88	4.55	5.06	5.54	6.93	7.69	8.38	632.41	709.56	784.21	6.10	6.84	7.56	22.54	25.53	28.38	0.22	0.25	0.27
Portugal - Klebsiella pneumoniae 3GCRKP SSI - model 4	4.92E-03	6.58E-03	8.65E-03	1.12	1.29	1.47	1.13	1.29	1.48	281.45	320.80	361.22	2.71	3.09	3.48	7.46	8.85	10.15	0.07	0.09	0.10
Portugal - Klebsiella pneumoniae 3GCRKP UTI - model 3	0.01	0.02	0.02	0.00	0.00	0.00	0.01	0.02	0.02	865.60	965.21	1,060.24	8.34	9.30	10.22	0.00	0.00	0.00	0.00	0.00	0.00
Portugal - Klebsiella pneumoniae CRKP BSI - model 6	0.41	0.46	0.52	5.20	6.09	7.03	5.65	6.55	7.50	70.16	75.53	81.03	0.68	0.73	0.78	23.34	26.92	30.84	0.22	0.26	0.30
Portugal - Klebsiella pneumoniae CRKP OTH - model 10	8.84E-04	1.19E-03	1.61E-03	0.00	0.00	0.00	8.84E-04	1.19E-03	1.61E-03	29.78	35.39	41.22	0.29	0.34	0.40	0.00	0.00	0.00	0.00	0.00	0.00
Portugal - Klebsiella pneumoniae CRKP RESP - model 7	0.11	0.13	0.16	0.21	0.26	0.31	0.31	0.39	0.47	26.28	31.86	37.83	0.25	0.31	0.36	0.95	1.15	1.36	9.16E-03	0.01	0.01
Portugal - Klebsiella pneumoniae CRKP SSI - model 9	3.32E-04	4.56E-04	6.03E-04	0.07	0.09	0.10	0.07	0.09	0.10	18.30	22.29	26.18	0.18	0.21	0.25	0.41	0.52	0.63	3.99E-03	5.04E-03	6.08E-03
Portugal - Klebsiella pneumoniae CRKP UTI - model 8	6.92E-04	9.19E-04	1.19E-03	0.00	0.00	0.00	6.92E-04	9.19E-04	1.19E-03	38.72	45.37	52.59	0.37	0.44	0.51	0.00	0.00	0.00	0.00	0.00	0.00
Portugal - Klebsiella pneumoniae ColRKP BSI - model 11	8.12E-03	0.01	0.02	0.14	0.24	0.36	0.15	0.25	0.38	1.76	2.19	2.69	0.02	0.02	0.03	0.79	1.31	1.93	7.66E-03	0.01	0.02
Portugal - Klebsiella pneumoniae ColRKP OTH - model 15	1.21E-05	3.13E-05	6.79E-05	0.00	0.00	0.00	1.21E-05	3.13E-05	6.79E-05	0.49	1.02	1.51	4.69E-03	9.83E-03	0.01	0.00	0.00	0.00	0.00	0.00	0.00
Portugal - Klebsiella pneumoniae ColRKP RESP - model 12	1.45E-03	3.19E-03	5.00E-03	2.75E-03	6.10E-03	9.57E-03	4.30E-03	9.28E-03	0.01	0.44	0.91	1.41	4.26E-03	8.76E-03	0.01	0.02	0.03	0.05	1.54E-04	3.13E-04	4.91E-04
Portugal - Klebsiella pneumoniae ColRKP SSI - model 14	4.61E-06	1.28E-05	2.90E-05	9.89E-04	2.21E-03	3.35E-03	9.94E-04	2.22E-03	3.37E-03	0.32	0.66	0.99	3.07E-03	6.37E-03	9.57E-03	6.06E-03	0.01	0.02	5.84E-05	1.44E-04	2.27E-04
Portugal - Klebsiella pneumoniae ColRKP UTI - model 13	1.08E-05	2.56E-05	5.01E-05	0.00	0.00	0.00	1.08E-05	2.56E-05	5.01E-05	0.73	1.33	1.92	7.08E-03	0.01	0.02	0.00	0.00	0.00	0.00	0.00	0.00
Portugal - Pseudomonas aeruginosa CRPA BSI - model 51	1.49	1.65	1.82	11.41	13.96	16.64	12.99	15.60	18.38	243.47	264.50	287.52	2.35	2.55	2.77	48.29	60.14	72.73	0.47	0.58	0.70
Portugal - Pseudomonas aeruginosa CRPA OTH - model 55	3.62E-03	4.68E-03	5.89E-03	0.00	0.00	0.00	3.62E-03	4.68E-03	5.89E-03	110.09	134.29	155.62	1.06	1.29	1.50	0.00	0.00	0.00	0.00	0.00	0.00
Portugal - Pseudomonas aeruginosa CRPA RESP - model 52	3.74	4.23	4.71	12.20	15.04	17.80	16.23	19.28	22.24	877.59	980.11	1,090.13	8.46	9.45	10.51	52.52	64.45	77.18	0.51	0.62	0.74
Portugal - Pseudomonas aeruginosa CRPA SSI - model 54	2.78E-03	3.74E-03	4.88E-03	0.60	0.74	0.87	0.60	0.74	0.87	151.30	182.26	211.08	1.46	1.76	2.03	3.56	4.47	5.35	0.03	0.04	0.05
Portugal - Pseudomonas aeruginosa CRPA UTI - model 53	5.19E-03	6.70E-03	8.31E-03	0.00	0.00	0.00	5.19E-03	6.70E-03	8.31E-03	287.70	340.21	386.93	2.77	3.28	3.73	0.00	0.00	0.00	0.00	0.00	0.00
Portugal - Pseudomonas aeruginosa ColRPA BSI - model 56	0.02	0.03	0.04	0.14	0.26	0.41	0.16	0.29	0.44	2.77	3.40	4.17	0.03	0.03	0.04	0.41	0.77	1.19	3.99E-03	7.42E-03	0.01
Portugal - Pseudomonas aeruginosa ColRPA OTH - model 60	2.91E-05	6.06E-05	1.05E-04	0.00	0.00	0.00	2.91E-05	6.06E-05	1.05E-04	1.01	1.70	2.42	9.74E-03	0.02	0.02	0.00	0.00	0.00	0.00	0.00	0.00
Portugal - Pseudomonas aeruginosa ColRPA RESP - model 57	0.05	0.08	0.10	0.15	0.27	0.43	0.22	0.35	0.53	9.15	12.41	15.88	0.09	0.12	0.15	0.47	0.81	1.26	4.48E-03	7.80E-03	0.01
Portugal - Pseudomonas aeruginosa ColRPA SSI - model 59	1.97E-05	4.54E-05	8.84E-05	5.94E-03	0.01	0.01	5.97E-03	0.01	0.01	1.33	2.31	3.12	0.01	0.02	0.03	0.02	0.04	0.06	1.88E-04	3.96E-04	5.85E-04
Portugal - Pseudomonas aeruginosa ColRPA UTI - model 58	4.25E-05	8.29E-05	1.44E-04	0.00	0.00	0.00	4.25E-05	8.29E-05	1.44E-04	2.69	4.34	5.84	0.03	0.04	0.06	0.00	0.00	0.00	0.00	0.00	0.00
Portugal - Pseudomonas aeruginosa MDRPA BSI - model 46	0.00	0.00	0.00	0.00	0.00	0.00	0.00	0.00	0.00	0.00	0.00	0.00	0.00	0.00	0.00	0.00	0.00	0.00	0.00	0.00	0.00
Portugal - Pseudomonas aeruginosa MDRPA OTH - model 50	0.00	0.00	0.00	0.00	0.00	0.00	0.00	0.00	0.00	0.00	0.00	0.00	0.00	0.00	0.00	0.00	0.00	0.00	0.00	0.00	0.00
Portugal - Pseudomonas aeruginosa MDRPA RESP - model 47	0.00	0.00	0.00	0.00	0.00	0.00	0.00	0.00	0.00	0.00	0.00	0.00	0.00	0.00	0.00	0.00	0.00	0.00	0.00	0.00	0.00
Portugal - Pseudomonas aeruginosa MDRPA SSI - model 49	0.00	0.00	0.00	0.00	0.00	0.00	0.00	0.00	0.00	0.00	0.00	0.00	0.00	0.00	0.00	0.00	0.00	0.00	0.00	0.00	0.00
Portugal - Pseudomonas aeruginosa MDRPA UTI - model 48	0.00	0.00	0.00	0.00	0.00	0.00	0.00	0.00	0.00	0.00	0.00	0.00	0.00	0.00	0.00	0.00	0.00	0.00	0.00	0.00	0.00
Portugal - Staphylococcus aureus MRSA BSI - model 61	7.22	7.76	8.38	49.35	51.96	54.84	56.76	59.69	62.87	1,715.34	1,803.08	1,899.90	16.53	17.38	18.31	302.39	323.97	346.83	2.91	3.12	3.34
Portugal - Staphylococcus aureus MRSA OTH - model 65	0.07	0.08	0.10	0.00	0.00	0.00	0.07	0.08	0.10	2,389.05	2,660.89	2,916.67	23.03	25.65	28.11	0.00	0.00	0.00	0.00	0.00	0.00
Portugal - Staphylococcus aureus MRSA RESP - model 62	7.32	8.12	8.91	14.25	15.62	16.97	21.54	23.74	25.78	2,442.56	2,710.84	2,987.62	23.54	26.13	28.80	86.99	97.51	108.39	0.84	0.94	1.04
Portugal - Staphylococcus aureus MRSA SSI - model 64	0.05	0.06	0.08	9.99	11.05	12.12	10.07	11.11	12.18	2,685.84	2,961.90	3,235.76	25.89	28.55	31.19	79.16	89.44	98.99	0.76	0.86	0.95
Portugal - Staphylococcus aureus MRSA UTI - model 63	5.89E-03	7.91E-03	0.01	0.00	0.00	0.00	5.89E-03	7.91E-03	0.01	355.62	441.03	518.05	3.43	4.25	4.99	0.00	0.00	0.00	0.00	0.00	0.00
Portugal - Streptococcus pneumoniae PMRSP BSI - model 76	0.11	0.12	0.13	0.77	0.81	0.84	0.89	0.92	0.96	16.82	16.82	16.82	0.16	0.16	0.16	2.91	3.03	3.15	0.03	0.03	0.03
Portugal - Streptococcus pneumoniae PMRSP OTH - model 80	8.95E-05	1.14E-04	1.41E-04	0.00	0.00	0.00	8.95E-05	1.14E-04	1.41E-04	5.59	6.02	6.45	0.05	0.06	0.06	0.00	0.00	0.00	0.00	0.00	0.00
Portugal - Streptococcus pneumoniae PMRSP RESP - model 77	0.27	0.32	0.38	0.53	0.62	0.73	0.81	0.93	1.09	57.17	64.15	73.56	0.55	0.62	0.71	2.04	2.31	2.66	0.02	0.02	0.03
Portugal - Streptococcus pneumoniae PMRSP SSI - model 79	0.00	0.00	0.00	0.00	0.00	0.00	0.00	0.00	0.00	0.00	0.00	0.00	0.00	0.00	0.00	0.00	0.00	0.00	0.00	0.00	0.00
Portugal - Streptococcus pneumoniae PMRSP UTI - model 78	0.00	0.00	0.00	0.00	0.00	0.00	0.00	0.00	0.00	0.00	0.00	0.00	0.00	0.00	0.00	0.00	0.00	0.00	0.00	0.00	0.00
Portugal - Streptococcus pneumoniae PRSP BSI - model 71	0.12	0.13	0.15	0.84	0.89	0.93	0.97	1.02	1.07	14.72	14.72	14.72	0.14	0.14	0.14	2.53	2.65	2.77	0.02	0.03	0.03
Portugal - Streptococcus pneumoniae PRSP OTH - model 75	7.56E-05	9.90E-05	1.28E-04	0.00	0.00	0.00	7.56E-05	9.90E-05	1.28E-04	4.85	5.27	5.74	0.05	0.05	0.06	0.00	0.00	0.00	0.00	0.00	0.00
Portugal - Streptococcus pneumoniae PRSP RESP - model 72	0.29	0.34	0.41	0.58	0.68	0.81	0.87	1.02	1.21	49.10	55.99	66.59	0.47	0.54	0.64	1.74	2.02	2.38	0.02	0.02	0.02
Portugal - Streptococcus pneumoniae PRSP SSI - model 74	0.00	0.00	0.00	0.00	0.00	0.00	0.00	0.00	0.00	0.00	0.00	0.00	0.00	0.00	0.00	0.00	0.00	0.00	0.00	0.00	0.00
Portugal - Streptococcus pneumoniae PRSP UTI - model 73	0.00	0.00	0.00	0.00	0.00	0.00	0.00	0.00	0.00	0.00	0.00	0.00	0.00	0.00	0.00	0.00	0.00	0.00	0.00	0.00	0.00
**Total (Median & 95% UIs)**	34.91	38.71	42.69	182.34	203.19	225.39	218.38	241.85	266.73	21,401.52	24,021.33	26,611.99	206.28	231.53	256.51	1,028.59	1,158.34	1,296.95	9.91	11.16	12.50
**Total (average)**		**38.74**			**203.32**			**242.07**			**24,027.46**			**231.59**			**1,160.11**			**11.18**	

Infection	YLD			YLL			DALY			DALY per Case			YLD per 100,000			YLL per 100,000			DALY per 100,000			Cases per year			Incidence per 100,000			Deaths per year			Mortality per 100,000		
Colistin resistant *Acinetobacter spp.*	46.17	61.57	81.10	202.22	325.58	484.55	253.90	388.09	557.86	7.74	10.98	14.61	0.23	0.31	0.41	1.02	1.64	2.44	1.28	1.95	2.81	79.63	104.74	133.45	0.40	0.53	0.67	5.73	8.91	12.74	0.03	0.04	0.06
Carbapenem resistant *Acinetobacter spp.* (excluding those resistant to colistin)	1,370.64	1,542.53	1,720.42	6,919.58	8,270.35	9,617.31	8,313.24	9,811.36	11,311.03	8.03	9.15	10.39	6.90	7.76	8.66	34.82	41.62	48.40	41.84	49.38	56.92	2,783.42	3,182.06	3,584.39	14.01	16.01	18.04	227.74	275.43	323.61	1.15	1.39	1.63
Aminoglycosides and fluoroquinolones resistant *Acinetobacter* spp. (excluding those resistant to colistin and/or carbapenem)	44.70	58.81	76.75	199.36	307.87	443.98	250.04	367.41	513.78	7.59	10.41	13.57	0.22	0.30	0.39	1.00	1.55	2.23	1.26	1.85	2.59	78.99	105.05	133.13	0.40	0.53	0.67	5.94	8.81	12.30	0.03	0.04	0.06
Vancomycin resistant Enterococci (*E. faecalis* and *E. faecium*)	136.43	181.82	235.76	1,306.09	1,705.85	2,195.25	1,434.63	1,885.40	2,427.19	7.45	8.51	9.96	0.69	0.92	1.19	6.57	8.58	11.05	7.22	9.49	12.21	634.24	856.75	1,119.39	3.19	4.31	5.63	41.42	55.84	74.23	0.21	0.28	0.37
Colistin resistant *Escherichia coli*	0.00	0.00	0.00	0.00	0.00	0.00	0.00	0.00	0.00	0.00	0.00	0.00	0.00	0.00	0.00	0.00	0.00	0.00	0.00	0.00	0.00	0.00	0.00	0.00	0.00	0.00	0.00	0.00	0.00	0.00	0.00	0.00	0.00
Carbapenem resistant *Escherichia coli* (excluding those resistant to colistin)	38.95	51.67	67.18	408.97	549.02	719.22	451.64	601.73	778.45	10.09	12.45	15.56	0.20	0.26	0.34	2.06	2.76	3.62	2.27	3.03	3.92	292.85	383.26	481.57	1.47	1.93	2.42	15.39	20.49	27.45	0.08	0.10	0.14
Third-generation cephalosporin resistant *Escherichia coli* (excluding those resistant to colistin and/or carbapenem)	505.64	582.74	666.36	2,968.40	3,418.86	3,903.35	3,498.48	4,003.58	4,574.33	6.16	6.67	7.24	2.54	2.93	3.35	14.94	17.21	19.64	17.61	20.15	23.02	4,161.60	4,815.42	5,536.68	20.94	24.23	27.86	123.33	143.85	168.98	0.62	0.72	0.85
Colistin resistant *Klebsiella pneumoniae*	98.28	121.73	147.99	1,658.12	2,154.32	2,677.87	1,772.17	2,277.25	2,813.84	16.16	19.40	22.67	0.49	0.61	0.74	8.34	10.84	13.48	8.92	11.46	14.16	279.36	355.98	438.42	1.41	1.79	2.21	60.89	77.76	97.27	0.31	0.39	0.49
Carbapenem resistant *Klebsiella pneumoniae* (excluding those resistant to colistin)	337.16	409.91	503.20	3,311.48	4,123.68	5,119.40	3,665.10	4,535.67	5,584.90	12.65	14.69	16.98	1.70	2.06	2.53	16.67	20.75	25.76	18.44	22.83	28.11	889.66	1,085.93	1,282.98	4.48	5.46	6.46	106.16	128.33	155.13	0.53	0.65	0.78
Third-generation cephalosporin resistant *Klebsiella pneumoniae* (excluding those resistant to colistin and/or carbapenem)	1,078.51	1,262.50	1,472.81	5,288.99	6,006.92	6,944.57	6,408.99	7,265.10	8,371.60	8.43	9.24	10.15	5.43	6.35	7.41	26.62	30.23	34.95	32.25	36.56	42.13	2,868.54	3,242.05	3,634.86	14.44	16.32	18.29	156.87	173.23	191.19	0.79	0.87	0.96
Colistin resistant *Pseudomonas aeruginosa*	11.39	24.94	42.74	38.97	118.77	264.23	54.58	142.81	297.38	5.66	12.22	18.69	0.06	0.13	0.22	0.20	0.60	1.33	0.27	0.72	1.50	22.65	54.89	91.98	0.11	0.28	0.46	1.12	3.43	7.62	0.01	0.02	0.04
Carbapenem resistant *Pseudomonas aeruginosa* (excluding those resistant to colistin)	1,140.79	1,344.67	1,566.95	5,279.39	6,784.31	8,502.33	6,510.58	8,130.10	9,993.43	8.98	11.00	12.99	5.74	6.77	7.89	26.57	34.14	42.79	32.76	40.92	50.29	2,754.54	3,279.14	3,837.11	13.86	16.50	19.31	172.87	219.25	275.97	0.87	1.10	1.39
Pseudomonas aeruginosa resistant to three or more antimicrobial groups (excluding those resistant to colistin and/or carbapenem)	25.67	47.87	80.03	91.18	215.62	443.65	120.92	262.76	510.57	5.82	10.77	18.31	0.13	0.24	0.40	0.46	1.09	2.23	0.61	1.32	2.57	60.48	108.19	161.62	0.30	0.54	0.81	3.16	6.61	11.81	0.02	0.03	0.06
Methicillin resistant *Staphylococcus aureus*	1.558.93	1.747.58	1.945.11	7.510.08	8.277.00	9.075.98	9.124.16	10.026.32	10.981.09	6.98	7.35	7.70	7.85	8.79	9.79	37.79	41.65	45.68	45.92	50.46	55.26	6.343.63	7.106.78	7.884.42	31.92	35.77	39.68	291.56	323.98	357.35	1.47	1.63	1.80
Penicillin and macrolides resistant *Streptococcus pneumoniae* (excluding those only resistant to penicillin)	33.51	48.59	78.20	122.90	160.06	227.32	159.00	208.75	299.70	8.71	9.72	10.81	0.17	0.24	0.39	0.62	0.81	1.14	0.80	1.05	1.51	51.06	70.92	105.63	0.26	0.36	0.53	3.32	4.30	5.96	0.02	0.02	0.03
Penicillin tesistant *Streptococcus pneumoniae* (excluding those resistant to macrolides)	167.44	209.84	261.49	575.93	680.17	799.33	751.91	890.51	1,059.24	8.31	8.99	9.69	0.84	1.06	1.32	2.90	3.42	4.02	3.78	4.48	5.33	267.23	325.96	396.66	1.34	1.64	2.00	16.93	19.72	22.90	0.09	0.10	0.12
Total	6,594.20	7,696.78	8,946.11	35,881.65	43,098.38	51,418.34	42,769.33	50,796.86	60,074.39				33.19	38.73	45.02	180.58	216.89	258.77	215.24	255.64	302.33	21,567.89	25,077.12	28,822.30	108.54	126.20	145.05	1,232.43	1,469.94	1,744.51	6.20	7.40	8.78

Model	YLD			YLL			DALY			DALY per Case			YLD per 100,000			YLL per 100,000			DALY per 100,000			Cases per year			Incidence per 100,000			Deaths per year			Mortality per 100,000		
Romania - Acinetobacter spp. CRACI BSI - model 36	782.82	875.49	966.03	5,627.61	6,782.35	7,936.10	6,440.41	7,657.52	8,879.08	6.05	7.03	8.12	3.94	4.41	4.86	28.32	34.13	39.94	32.41	38.54	44.68	995.14	1,086.96	1,179.94	5.01	5.47	5.94	183.78	224.02	264.10	0.92	1.13	1.33
Romania - Acinetobacter spp. CRACI OTH - model 40	0.11	0.15	0.20	0.00	0.00	0.00	0.11	0.15	0.20	3.08E-03	3.74E-03	4.41E-03	5.69E-04	7.77E-04	1.03E-03	0.00	0.00	0.00	5.69E-04	7.77E-04	1.03E-03	32.07	41.36	52.45	0.16	0.21	0.26	0.00	0.00	0.00	0.00	0.00	0.00
Romania - Acinetobacter spp. CRACI RESP - model 37	586.34	665.09	751.88	1,142.33	1,301.29	1,461.13	1,721.65	1,965.11	2,209.11	1.54	1.65	1.77	2.95	3.35	3.78	5.75	6.55	7.35	8.66	9.89	11.12	1,039.45	1,189.83	1,344.91	5.23	5.99	6.77	37.29	42.84	49.11	0.19	0.22	0.25
Romania - Acinetobacter spp. CRACI SSI - model 39	0.65	0.87	1.12	149.64	186.71	220.08	150.35	187.64	221.45	0.43	0.46	0.49	3.28E-03	4.36E-03	5.65E-03	0.75	0.94	1.11	0.76	0.94	1.11	337.01	406.28	476.09	1.70	2.04	2.40	6.68	8.57	10.40	0.03	0.04	0.05
Romania - Acinetobacter spp. CRACI UTI - model 38	0.72	0.93	1.19	0.00	0.00	0.00	0.72	0.93	1.19	1.70E-03	2.04E-03	2.44E-03	3.63E-03	4.69E-03	5.97E-03	0.00	0.00	0.00	3.63E-03	4.69E-03	5.97E-03	379.75	457.63	531.01	1.91	2.30	2.67	0.00	0.00	0.00	0.00	0.00	0.00
Romania - Acinetobacter spp. ColRACI BSI - model 41	27.15	35.05	45.39	161.02	268.10	409.16	192.96	304.00	447.64	5.62	8.54	11.84	0.14	0.18	0.23	0.81	1.35	2.06	0.97	1.53	2.25	29.70	36.01	42.95	0.15	0.18	0.22	4.57	7.32	10.65	0.02	0.04	0.05
Romania - Acinetobacter spp. ColRACI OTH - model 45	2.44E-03	4.98E-03	8.73E-03	0.00	0.00	0.00	2.44E-03	4.98E-03	8.73E-03	2.49E-03	3.73E-03	5.09E-03	1.23E-05	2.51E-05	4.39E-05	0.00	0.00	0.00	1.23E-05	2.51E-05	4.39E-05	0.74	1.36	2.14	3.74E-03	6.85E-03	0.01	0.00	0.00	0.00	0.00	0.00	0.00
Romania - Acinetobacter spp. ColRACI RESP - model 42	18.99	26.46	35.60	37.35	51.76	67.71	57.06	78.31	102.47	1.74	2.01	2.29	0.10	0.13	0.18	0.19	0.26	0.34	0.29	0.39	0.52	29.28	39.03	50.10	0.15	0.20	0.25	1.04	1.40	1.82	5.24E-03	7.05E-03	9.15E-03
Romania - Acinetobacter spp. ColRACI SSI - model 44	0.02	0.03	0.05	3.84	5.72	7.67	3.86	5.75	7.69	0.38	0.43	0.47	7.71E-05	1.43E-04	2.37E-04	0.02	0.03	0.04	0.02	0.03	0.04	9.31	13.48	18.09	0.05	0.07	0.09	0.12	0.20	0.27	6.15E-04	9.83E-04	1.37E-03
Romania - Acinetobacter spp. ColRACI UTI - model 43	0.02	0.03	0.05	0.00	0.00	0.00	0.02	0.03	0.05	1.38E-03	2.05E-03	2.82E-03	9.08E-05	1.52E-04	2.38E-04	0.00	0.00	0.00	9.08E-05	1.52E-04	2.38E-04	10.60	14.87	20.17	0.05	0.07	0.10	0.00	0.00	0.00	0.00	0.00	0.00
Romania - Acinetobacter spp. MDRACI BSI - model 31	26.27	33.48	42.28	158.59	251.99	370.95	190.01	286.13	405.47	5.50	8.02	10.83	0.13	0.17	0.21	0.80	1.27	1.87	0.96	1.44	2.04	30.01	35.89	42.96	0.15	0.18	0.22	4.73	7.14	10.10	0.02	0.04	0.05
Romania - Acinetobacter spp. MDRACI OTH - model 35	2.40E-03	5.08E-03	8.72E-03	0.00	0.00	0.00	2.40E-03	5.08E-03	8.72E-03	2.39E-03	3.75E-03	5.22E-03	1.21E-05	2.56E-05	4.39E-05	0.00	0.00	0.00	1.21E-05	2.56E-05	4.39E-05	0.75	1.36	2.07	3.79E-03	6.84E-03	0.01	0.00	0.00	0.00	0.00	0.00	0.00
Romania - Acinetobacter spp. MDRACI RESP - model 32	18.39	25.28	34.36	36.48	49.48	64.31	55.73	74.82	99.49	1.64	1.91	2.22	0.09	0.13	0.17	0.18	0.25	0.32	0.28	0.38	0.50	29.25	39.24	49.61	0.15	0.20	0.25	1.05	1.41	1.82	5.27E-03	7.09E-03	9.17E-03
Romania - Acinetobacter spp. MDRACI SSI - model 34	0.01	0.03	0.05	4.29	6.40	8.72	4.29	6.42	8.77	0.44	0.48	0.51	7.05E-05	1.41E-04	2.37E-04	0.02	0.03	0.04	0.02	0.03	0.04	9.11	13.46	18.14	0.05	0.07	0.09	0.16	0.26	0.38	8.12E-04	1.33E-03	1.90E-03
Romania - Acinetobacter spp. MDRACI UTI - model 33	0.02	0.03	0.05	0.00	0.00	0.00	0.02	0.03	0.05	1.35E-03	2.03E-03	2.80E-03	8.75E-05	1.52E-04	2.44E-04	0.00	0.00	0.00	8.75E-05	1.52E-04	2.44E-04	9.86	15.11	20.34	0.05	0.08	0.10	0.00	0.00	0.00	0.00	0.00	0.00
Romania - Enterococcus faecalis and E. faecium VRE BSI - model 66	135.65	180.62	233.87	1,201.62	1,552.04	1,984.01	1,329.38	1,730.49	2,214.45	7.01	7.99	9.37	0.68	0.91	1.18	6.05	7.81	9.98	6.69	8.71	11.14	163.46	215.05	284.67	0.82	1.08	1.43	37.30	49.29	64.97	0.19	0.25	0.33
Romania - Enterococcus faecalis and E. faecium VRE OTH - model 70	0.25	0.37	0.56	0.00	0.00	0.00	0.25	0.37	0.56	1.21E-03	1.63E-03	2.18E-03	1.24E-03	1.88E-03	2.81E-03	0.00	0.00	0.00	1.24E-03	1.88E-03	2.81E-03	171.44	231.46	300.30	0.86	1.16	1.51	0.00	0.00	0.00	0.00	0.00	0.00
Romania - Enterococcus faecalis and E. faecium VRE RESP - model 67	0.00	0.00	0.00	0.00	0.00	0.00	0.00	0.00	0.00	NaN	NaN	NaN	0.00	0.00	0.00	0.00	0.00	0.00	0.00	0.00	0.00	0.00	0.00	0.00	0.00	0.00	0.00	0.00	0.00	0.00	0.00	0.00	0.00
Romania - Enterococcus faecalis and E. faecium VRE SSI - model 69	0.40	0.62	1.02	104.46	153.80	211.24	104.87	154.32	211.88	0.44	0.52	0.58	2.00E-03	3.12E-03	5.15E-03	0.53	0.77	1.06	0.53	0.78	1.07	220.37	299.39	387.69	1.11	1.51	1.95	4.11	6.54	9.27	0.02	0.03	0.05
Romania - Enterococcus faecalis and E. faecium VRE UTI - model 68	0.13	0.20	0.31	0.00	0.00	0.00	0.13	0.20	0.31	1.38E-03	1.87E-03	2.43E-03	6.61E-04	1.03E-03	1.56E-03	0.00	0.00	0.00	6.61E-04	1.03E-03	1.56E-03	78.97	110.85	146.74	0.40	0.56	0.74	0.00	0.00	0.00	0.00	0.00	0.00
Romania - Escherichia coli 3GCREC BSI - model 16	385.57	436.20	491.72	2,480.37	2,825.89	3,199.99	2,890.24	3,263.57	3,698.56	4.51	4.89	5.31	1.94	2.20	2.47	12.48	14.22	16.10	14.55	16.42	18.61	599.67	667.38	743.73	3.02	3.36	3.74	100.24	115.33	134.59	0.50	0.58	0.68
Romania - Escherichia coli 3GCREC OTH - model 20	0.56	0.76	1.02	0.00	0.00	0.00	0.56	0.76	1.02	2.65E-03	3.34E-03	4.14E-03	2.81E-03	3.83E-03	5.15E-03	0.00	0.00	0.00	2.81E-03	3.83E-03	5.15E-03	178.80	228.69	274.41	0.90	1.15	1.38	0.00	0.00	0.00	0.00	0.00	0.00
Romania - Escherichia coli 3GCREC RESP - model 17	113.97	138.57	164.25	223.99	268.99	317.71	337.85	408.13	479.96	1.22	1.33	1.45	0.57	0.70	0.83	1.13	1.35	1.60	1.70	2.05	2.42	248.74	306.29	364.04	1.25	1.54	1.83	8.92	11.00	13.28	0.04	0.06	0.07
Romania - Escherichia coli 3GCREC SSI - model 19	1.14	1.54	2.04	264.04	323.97	385.66	265.44	325.45	387.45	0.42	0.45	0.48	5.74E-03	7.73E-03	0.01	1.33	1.63	1.94	1.34	1.64	1.95	611.77	720.09	836.45	3.08	3.62	4.21	14.16	17.53	21.12	0.07	0.09	0.11
Romania - Escherichia coli 3GCREC UTI - model 18	4.40	5.68	7.34	0.00	0.00	0.00	4.40	5.68	7.34	1.59E-03	1.98E-03	2.39E-03	0.02	0.03	0.04	0.00	0.00	0.00	0.02	0.03	0.04	2,522.63	2,892.96	3,318.05	12.70	14.56	16.70	0.00	0.00	0.00	0.00	0.00	0.00
Romania - Escherichia coli CREC BSI - model 21	29.68	37.51	47.23	369.87	492.58	645.57	403.12	531.01	685.77	8.41	10.46	13.20	0.15	0.19	0.24	1.86	2.48	3.25	2.03	2.67	3.45	42.44	50.73	61.35	0.21	0.26	0.31	13.71	18.04	24.21	0.07	0.09	0.12
Romania - Escherichia coli CREC OTH - model 25	0.06	0.10	0.17	0.00	0.00	0.00	0.06	0.10	0.17	2.26E-03	3.48E-03	5.10E-03	3.00E-04	5.24E-04	8.63E-04	0.00	0.00	0.00	3.00E-04	5.24E-04	8.63E-04	21.43	29.81	39.21	0.11	0.15	0.20	0.00	0.00	0.00	0.00	0.00	0.00
Romania - Escherichia coli CREC RESP - model 22	8.87	13.48	18.90	17.25	26.28	35.64	26.42	39.89	53.89	1.22	1.49	1.83	0.04	0.07	0.10	0.09	0.13	0.18	0.13	0.20	0.27	18.01	26.60	35.53	0.09	0.13	0.18	0.64	0.95	1.27	3.21E-03	4.78E-03	6.41E-03
Romania - Escherichia coli CREC SSI - model 24	0.07	0.13	0.21	21.84	30.17	38.01	21.77	30.28	37.94	0.46	0.49	0.53	3.65E-04	6.41E-04	1.08E-03	0.11	0.15	0.19	0.11	0.15	0.19	44.86	61.12	78.13	0.23	0.31	0.39	1.04	1.50	1.97	5.24E-03	7.54E-03	9.90E-03
Romania - Escherichia coli CREC UTI - model 23	0.27	0.44	0.67	0.00	0.00	0.00	0.27	0.44	0.67	1.34E-03	2.06E-03	2.94E-03	1.36E-03	2.23E-03	3.37E-03	0.00	0.00	0.00	1.36E-03	2.23E-03	3.37E-03	166.12	215.01	267.36	0.84	1.08	1.35	0.00	0.00	0.00	0.00	0.00	0.00
Romania - Escherichia coli ColREC BSI - model 26	0.00	0.00	0.00	0.00	0.00	0.00	0.00	0.00	0.00	NaN	NaN	NaN	0.00	0.00	0.00	0.00	0.00	0.00	0.00	0.00	0.00	0.00	0.00	0.00	0.00	0.00	0.00	0.00	0.00	0.00	0.00	0.00	0.00
Romania - Escherichia coli ColREC OTH - model 30	0.00	0.00	0.00	0.00	0.00	0.00	0.00	0.00	0.00	NaN	NaN	NaN	0.00	0.00	0.00	0.00	0.00	0.00	0.00	0.00	0.00	0.00	0.00	0.00	0.00	0.00	0.00	0.00	0.00	0.00	0.00	0.00	0.00
Romania - Escherichia coli ColREC RESP - model 27	0.00	0.00	0.00	0.00	0.00	0.00	0.00	0.00	0.00	NaN	NaN	NaN	0.00	0.00	0.00	0.00	0.00	0.00	0.00	0.00	0.00	0.00	0.00	0.00	0.00	0.00	0.00	0.00	0.00	0.00	0.00	0.00	0.00
Romania - Escherichia coli ColREC SSI - model 29	0.00	0.00	0.00	0.00	0.00	0.00	0.00	0.00	0.00	NaN	NaN	NaN	0.00	0.00	0.00	0.00	0.00	0.00	0.00	0.00	0.00	0.00	0.00	0.00	0.00	0.00	0.00	0.00	0.00	0.00	0.00	0.00	0.00
Romania - Escherichia coli ColREC UTI - model 28	0.00	0.00	0.00	0.00	0.00	0.00	0.00	0.00	0.00	NaN	NaN	NaN	0.00	0.00	0.00	0.00	0.00	0.00	0.00	0.00	0.00	0.00	0.00	0.00	0.00	0.00	0.00	0.00	0.00	0.00	0.00	0.00	0.00
Romania - Klebsiella pneumoniae 3GCRKP BSI - model 1	682.93	782.03	900.54	4,380.52	4,912.66	5,632.98	5,097.63	5,689.37	6,488.20	6.26	6.82	7.48	3.44	3.94	4.53	22.05	24.72	28.35	25.65	28.63	32.65	778.89	837.10	901.80	3.92	4.21	4.54	128.25	139.91	152.65	0.65	0.70	0.77
Romania - Klebsiella pneumoniae 3GCRKP OTH - model 5	0.89	1.12	1.43	0.00	0.00	0.00	0.89	1.12	1.43	2.74E-03	3.31E-03	3.99E-03	4.47E-03	5.64E-03	7.22E-03	0.00	0.00	0.00	4.47E-03	5.64E-03	7.22E-03	289.97	339.76	387.52	1.46	1.71	1.95	0.00	0.00	0.00	0.00	0.00	0.00
Romania - Klebsiella pneumoniae 3GCRKP RESP - model 2	392.62	476.69	567.44	770.29	930.34	1,118.57	1,170.13	1,408.01	1,685.62	1.70	1.92	2.14	1.98	2.40	2.86	3.88	4.68	5.63	5.89	7.09	8.48	638.88	732.10	826.93	3.22	3.68	4.16	22.81	26.39	30.25	0.11	0.13	0.15
Romania - Klebsiella pneumoniae 3GCRKP SSI - model 4	0.54	0.71	0.96	138.17	163.92	193.02	138.81	164.65	193.90	0.46	0.49	0.52	2.73E-03	3.59E-03	4.83E-03	0.70	0.82	0.97	0.70	0.83	0.98	289.89	334.31	388.48	1.46	1.68	1.96	5.81	6.94	8.29	0.03	0.03	0.04
Romania - Klebsiella pneumoniae 3GCRKP UTI - model 3	1.53	1.95	2.43	0.00	0.00	0.00	1.53	1.95	2.43	1.64E-03	1.95E-03	2.31E-03	7.72E-03	9.81E-03	0.01	0.00	0.00	0.00	7.72E-03	9.81E-03	0.01	870.90	998.78	1,130.14	4.38	5.03	5.69	0.00	0.00	0.00	0.00	0.00	0.00
Romania - Klebsiella pneumoniae CRKP BSI - model 6	248.38	289.33	342.00	3,098.58	3,834.75	4,745.70	3,357.42	4,126.72	5,051.82	10.54	12.22	14.13	1.25	1.46	1.72	15.59	19.30	23.88	16.90	20.77	25.42	296.32	338.50	378.55	1.49	1.70	1.91	99.53	119.84	144.64	0.50	0.60	0.73
Romania - Klebsiella pneumoniae CRKP OTH - model 10	0.50	0.68	0.93	0.00	0.00	0.00	0.50	0.68	0.93	2.78E-03	3.51E-03	4.47E-03	2.49E-03	3.43E-03	4.69E-03	0.00	0.00	0.00	2.49E-03	3.43E-03	4.69E-03	154.32	195.76	238.60	0.78	0.99	1.20	0.00	0.00	0.00	0.00	0.00	0.00
Romania - Klebsiella pneumoniae CRKP RESP - model 7	87.72	119.11	159.21	169.32	232.73	303.49	263.01	351.23	460.93	1.69	2.00	2.34	0.44	0.60	0.80	0.85	1.17	1.53	1.32	1.77	2.32	140.50	175.97	212.79	0.71	0.89	1.07	4.96	6.35	7.83	0.02	0.03	0.04
Romania - Klebsiella pneumoniae CRKP SSI - model 9	0.18	0.26	0.37	43.58	56.20	70.21	43.78	56.51	70.54	0.41	0.46	0.50	9.26E-04	1.32E-03	1.87E-03	0.22	0.28	0.35	0.22	0.28	0.35	97.16	123.77	154.26	0.49	0.62	0.78	1.66	2.14	2.67	8.37E-03	0.01	0.01
Romania - Klebsiella pneumoniae CRKP UTI - model 8	0.38	0.52	0.69	0.00	0.00	0.00	0.38	0.52	0.69	1.64E-03	2.09E-03	2.58E-03	1.92E-03	2.64E-03	3.47E-03	0.00	0.00	0.00	1.92E-03	2.64E-03	3.47E-03	201.37	251.93	298.77	1.01	1.27	1.50	0.00	0.00	0.00	0.00	0.00	0.00
Romania - Klebsiella pneumoniae ColRKP BSI - model 11	78.49	93.39	111.11	1,607.40	2,082.62	2,584.28	1,701.54	2,177.16	2,683.81	14.44	17.45	20.43	0.40	0.47	0.56	8.09	10.48	13.01	8.56	10.96	13.51	107.27	124.56	144.31	0.54	0.63	0.73	58.90	74.95	93.61	0.30	0.38	0.47
Romania - Klebsiella pneumoniae ColRKP OTH - model 15	0.14	0.21	0.31	0.00	0.00	0.00	0.14	0.21	0.31	2.57E-03	3.48E-03	4.73E-03	6.99E-04	1.06E-03	1.54E-03	0.00	0.00	0.00	6.99E-04	1.06E-03	1.54E-03	45.47	60.72	77.12	0.23	0.31	0.39	0.00	0.00	0.00	0.00	0.00	0.00
Romania - Klebsiella pneumoniae ColRKP RESP - model 12	19.49	27.88	36.23	38.76	54.46	70.83	58.31	82.39	106.62	1.31	1.50	1.72	0.10	0.14	0.18	0.20	0.27	0.36	0.29	0.41	0.54	40.08	54.87	70.48	0.20	0.28	0.35	1.43	1.98	2.56	7.20E-03	9.94E-03	0.01
Romania - Klebsiella pneumoniae ColRKP SSI - model 14	0.05	0.08	0.12	11.95	17.25	22.77	12.08	17.34	22.87	0.40	0.45	0.51	2.54E-04	4.04E-04	5.95E-04	0.06	0.09	0.11	0.06	0.09	0.12	27.72	38.33	49.04	0.14	0.19	0.25	0.56	0.83	1.11	2.81E-03	4.19E-03	5.57E-03
Romania - Klebsiella pneumoniae ColRKP UTI - model 13	0.11	0.16	0.23	0.00	0.00	0.00	0.11	0.16	0.23	1.59E-03	2.09E-03	2.71E-03	5.42E-04	8.11E-04	1.15E-03	0.00	0.00	0.00	5.42E-04	8.11E-04	1.15E-03	58.82	77.49	97.46	0.30	0.39	0.49	0.00	0.00	0.00	0.00	0.00	0.00
Romania - Pseudomonas aeruginosa CRPA BSI - model 51	322.69	372.54	433.26	2,462.03	3,200.97	4,035.37	2,809.51	3,573.56	4,454.02	6.32	7.91	9.41	1.62	1.87	2.18	12.39	16.11	20.31	14.14	17.98	22.42	401.86	450.10	512.22	2.02	2.27	2.58	80.62	102.69	128.85	0.41	0.52	0.65
Romania - Pseudomonas aeruginosa CRPA OTH - model 55	0.64	0.85	1.12	0.00	0.00	0.00	0.64	0.85	1.12	2.95E-03	3.64E-03	4.38E-03	3.24E-03	4.29E-03	5.66E-03	0.00	0.00	0.00	3.24E-03	4.29E-03	5.66E-03	186.12	234.60	286.13	0.94	1.18	1.44	0.00	0.00	0.00	0.00	0.00	0.00
Romania - Pseudomonas aeruginosa CRPA RESP - model 52	816.10	969.39	1,130.03	2,701.84	3,434.68	4,284.22	3,583.50	4,405.12	5,353.01	2.23	2.62	3.07	4.11	4.88	5.69	13.60	17.29	21.56	18.03	22.17	26.94	1,428.45	1,684.73	1,951.17	7.19	8.48	9.82	87.48	110.13	138.93	0.44	0.55	0.70
Romania - Pseudomonas aeruginosa CRPA SSI - model 54	0.47	0.67	0.94	115.52	148.66	182.74	116.04	149.36	183.68	0.43	0.47	0.50	2.38E-03	3.37E-03	4.71E-03	0.58	0.75	0.92	0.58	0.75	0.92	254.02	318.53	380.40	1.28	1.60	1.91	4.78	6.44	8.18	0.02	0.03	0.04
Romania - Pseudomonas aeruginosa CRPA UTI - model 53	0.89	1.21	1.60	0.00	0.00	0.00	0.89	1.21	1.60	1.67E-03	2.05E-03	2.45E-03	4.50E-03	6.10E-03	8.06E-03	0.00	0.00	0.00	4.50E-03	6.10E-03	8.06E-03	484.10	591.19	707.19	2.44	2.98	3.56	0.00	0.00	0.00	0.00	0.00	0.00
Romania - Pseudomonas aeruginosa ColRPA BSI - model 56	3.72	6.74	11.78	18.34	57.21	123.17	23.38	63.48	130.79	3.72	8.99	14.14	0.02	0.03	0.06	0.09	0.29	0.62	0.12	0.32	0.66	4.59	7.38	11.56	0.02	0.04	0.06	0.53	1.65	3.55	2.66E-03	8.30E-03	0.02
Romania - Pseudomonas aeruginosa ColRPA OTH - model 60	2.76E-03	0.01	0.04	0.00	0.00	0.00	2.76E-03	0.01	0.04	1.55E-03	3.67E-03	6.46E-03	1.39E-05	6.51E-05	1.86E-04	0.00	0.00	0.00	1.39E-05	6.51E-05	1.86E-04	0.94	3.84	7.09	4.72E-03	0.02	0.04	0.00	0.00	0.00	0.00	0.00	0.00
Romania - Pseudomonas aeruginosa ColRPA RESP - model 57	7.66	18.16	30.85	20.25	59.94	138.13	30.81	77.67	163.54	1.63	2.91	4.22	0.04	0.09	0.16	0.10	0.30	0.70	0.16	0.39	0.82	12.49	28.68	46.33	0.06	0.14	0.23	0.58	1.73	3.98	2.94E-03	8.70E-03	0.02
Romania - Pseudomonas aeruginosa ColRPA SSI - model 59	1.25E-03	9.29E-03	0.03	0.37	1.61	2.93	0.38	1.63	2.96	0.31	0.31	0.32	6.28E-06	4.68E-05	1.42E-04	1.87E-03	8.12E-03	0.01	1.89E-03	8.19E-03	0.01	1.19	5.17	9.38	6.00E-03	0.03	0.05	0.01	0.05	0.08	5.40E-05	2.34E-04	4.25E-04
Romania - Pseudomonas aeruginosa ColRPA UTI - model 58	4.84E-03	0.02	0.05	0.00	0.00	0.00	4.84E-03	0.02	0.05	7.68E-04	1.96E-03	3.77E-03	2.44E-05	8.99E-05	2.38E-04	0.00	0.00	0.00	2.44E-05	8.99E-05	2.38E-04	3.44	9.82	17.62	0.02	0.05	0.09	0.00	0.00	0.00	0.00	0.00	0.00
Romania - Pseudomonas aeruginosa MDRPA BSI - model 46	8.00	13.28	21.84	40.23	99.38	205.60	50.24	113.03	222.33	3.87	7.69	13.35	0.04	0.07	0.11	0.20	0.50	1.03	0.25	0.57	1.12	10.23	14.76	20.76	0.05	0.07	0.10	1.43	2.98	5.31	7.21E-03	0.02	0.03
Romania - Pseudomonas aeruginosa MDRPA OTH - model 50	0.01	0.03	0.06	0.00	0.00	0.00	0.01	0.03	0.06	1.95E-03	3.66E-03	5.72E-03	5.43E-05	1.35E-04	2.78E-04	0.00	0.00	0.00	5.43E-05	1.35E-04	2.78E-04	3.35	7.65	12.40	0.02	0.04	0.06	0.00	0.00	0.00	0.00	0.00	0.00
Romania - Pseudomonas aeruginosa MDRPA RESP - model 47	17.64	34.51	58.01	48.91	111.77	230.73	68.62	145.18	280.77	1.58	2.64	4.46	0.09	0.17	0.29	0.25	0.56	1.16	0.35	0.73	1.41	32.67	56.09	81.56	0.16	0.28	0.41	1.64	3.40	6.10	8.24E-03	0.02	0.03
Romania - Pseudomonas aeruginosa MDRPA SSI - model 49	6.76E-03	0.02	0.05	2.03	4.47	7.32	2.04	4.49	7.34	0.38	0.43	0.49	3.40E-05	1.06E-04	2.34E-04	0.01	0.02	0.04	0.01	0.02	0.04	4.59	10.36	16.56	0.02	0.05	0.08	0.09	0.23	0.39	4.47E-04	1.18E-03	1.98E-03
Romania - Pseudomonas aeruginosa MDRPA UTI - model 48	0.01	0.04	0.08	0.00	0.00	0.00	0.01	0.04	0.08	1.00E-03	2.02E-03	3.21E-03	6.93E-05	1.91E-04	3.87E-04	0.00	0.00	0.00	6.93E-05	1.91E-04	3.87E-04	9.64	19.34	30.34	0.05	0.10	0.15	0.00	0.00	0.00	0.00	0.00	0.00
Romania - Staphylococcus aureus MRSA BSI - model 61	763.03	840.97	934.05	5,229.19	5,686.49	6,190.15	6,031.22	6,529.83	7,095.30	5.21	5.47	5.73	3.84	4.23	4.70	26.32	28.62	31.15	30.35	32.86	35.71	1,104.27	1,194.12	1,292.45	5.56	6.01	6.50	196.18	214.22	233.62	0.99	1.08	1.18
Romania - Staphylococcus aureus MRSA OTH - model 65	4.70	5.86	7.24	0.00	0.00	0.00	4.70	5.86	7.24	2.72E-03	3.27E-03	3.89E-03	0.02	0.03	0.04	0.00	0.00	0.00	0.02	0.03	0.04	1,591.83	1,795.71	1,991.49	8.01	9.04	10.02	0.00	0.00	0.00	0.00	0.00	0.00
Romania - Staphylococcus aureus MRSA RESP - model 62	787.56	895.97	997.71	1,543.24	1,750.48	1,945.50	2,343.14	2,645.46	2,933.09	1.36	1.44	1.53	3.96	4.51	5.02	7.77	8.81	9.79	11.79	13.31	14.76	1,624.64	1,827.16	2,029.68	8.18	9.20	10.21	57.58	65.96	73.81	0.29	0.33	0.37
Romania - Staphylococcus aureus MRSA SSI - model 64	3.21	4.21	5.38	737.65	840.03	940.32	744.67	844.61	944.73	0.40	0.43	0.44	0.02	0.02	0.03	3.71	4.23	4.73	3.75	4.25	4.75	1,773.64	1,985.25	2,211.86	8.93	9.99	11.13	37.81	43.79	49.91	0.19	0.22	0.25
Romania - Staphylococcus aureus MRSA UTI - model 63	0.43	0.56	0.73	0.00	0.00	0.00	0.43	0.56	0.73	1.53E-03	1.87E-03	2.24E-03	2.18E-03	2.84E-03	3.66E-03	0.00	0.00	0.00	2.18E-03	2.84E-03	3.66E-03	249.24	304.54	358.94	1.25	1.53	1.81	0.00	0.00	0.00	0.00	0.00	0.00
Romania - Streptococcus pneumoniae PMRSP BSI - model 76	10.29	13.24	17.04	77.42	90.50	105.40	89.97	103.68	119.99	6.98	7.69	8.43	0.05	0.07	0.09	0.39	0.46	0.53	0.45	0.52	0.60	12.08	13.51	15.06	0.06	0.07	0.08	2.09	2.43	2.81	0.01	0.01	0.01
Romania - Streptococcus pneumoniae PMRSP OTH - model 80	4.51E-03	9.17E-03	0.02	0.00	0.00	0.00	4.51E-03	9.17E-03	0.02	1.00E-03	1.91E-03	3.07E-03	2.27E-05	4.61E-05	7.99E-05	0.00	0.00	0.00	2.27E-05	4.61E-05	7.99E-05	3.67	4.88	6.57	0.02	0.02	0.03	0.00	0.00	0.00	0.00	0.00	0.00
Romania - Streptococcus pneumoniae PMRSP RESP - model 77	23.21	35.35	61.15	45.48	69.56	121.92	69.02	105.06	179.70	1.72	2.03	2.38	0.12	0.18	0.31	0.23	0.35	0.61	0.35	0.53	0.90	35.30	52.52	84.00	0.18	0.26	0.42	1.22	1.87	3.15	6.16E-03	9.42E-03	0.02
Romania - Streptococcus pneumoniae PMRSP SSI - model 79	0.00	0.00	0.00	0.00	0.00	0.00	0.00	0.00	0.00	NaN	NaN	NaN	0.00	0.00	0.00	0.00	0.00	0.00	0.00	0.00	0.00	0.00	0.00	0.00	0.00	0.00	0.00	0.00	0.00	0.00	0.00	0.00	0.00
Romania - Streptococcus pneumoniae PMRSP UTI - model 78	0.00	0.00	0.00	0.00	0.00	0.00	0.00	0.00	0.00	NaN	NaN	NaN	0.00	0.00	0.00	0.00	0.00	0.00	0.00	0.00	0.00	0.00	0.00	0.00	0.00	0.00	0.00	0.00	0.00	0.00	0.00	0.00	0.00
Romania - Streptococcus pneumoniae PRSP BSI - model 71	49.14	55.49	62.68	345.32	377.31	406.56	396.95	432.89	467.15	6.67	7.12	7.54	0.25	0.28	0.32	1.74	1.90	2.05	2.00	2.18	2.35	57.28	60.72	64.28	0.29	0.31	0.32	10.08	10.95	11.79	0.05	0.06	0.06
Romania - Streptococcus pneumoniae PRSP OTH - model 75	0.03	0.04	0.06	0.00	0.00	0.00	0.03	0.04	0.06	1.43E-03	1.94E-03	2.53E-03	1.54E-04	2.17E-04	3.00E-04	0.00	0.00	0.00	1.54E-04	2.17E-04	3.00E-04	19.22	22.31	25.93	0.10	0.11	0.13	0.00	0.00	0.00	0.00	0.00	0.00
Romania - Streptococcus pneumoniae PRSP RESP - model 72	118.26	154.31	198.75	230.60	302.85	392.77	354.93	457.58	592.03	1.64	1.87	2.15	0.60	0.78	1.00	1.16	1.52	1.98	1.79	2.30	2.98	190.72	242.93	306.45	0.96	1.22	1.54	6.85	8.76	11.11	0.03	0.04	0.06
Romania - Streptococcus pneumoniae PRSP SSI - model 74	0.00	0.00	0.00	0.00	0.00	0.00	0.00	0.00	0.00	NaN	NaN	NaN	0.00	0.00	0.00	0.00	0.00	0.00	0.00	0.00	0.00	0.00	0.00	0.00	0.00	0.00	0.00	0.00	0.00	0.00	0.00	0.00	0.00
Romania - Streptococcus pneumoniae PRSP UTI - model 73	0.00	0.00	0.00	0.00	0.00	0.00	0.00	0.00	0.00	NaN	NaN	NaN	0.00	0.00	0.00	0.00	0.00	0.00	0.00	0.00	0.00	0.00	0.00	0.00	0.00	0.00	0.00	0.00	0.00	0.00	0.00	0.00	0.00
**Total (Median & 95% UIs)**	6,594.20	7,696.78	8,946.11	35,881.65	43,098.38	51,418.34	42,769.33	50,796.86	60,074.39	-			33.19	38.73	45.02	180.58	216.89	258.77	215.24	255.64	302.33	21,567.89	25,077.12	28,822.30	108.54	126.20	145.05	1,232.43	1,469.94	1,744.51	6.20	7.40	8.78
**Total (average)**	**7,715.68**			**43,254.56**			**50,970.24**			-			**38.83**			**217.68**			**256.51**			**25,118.19**			**126.41**			**1,475.82**			**7.43**		

Infection	YLD			YLL			DALY			DALY per Case			YLD per 100,000		
Colistin resistant *Acinetobacter spp.*	0.00	0.00	0.00	0.00	0.00	0.00	0.00	0.00	0.00	0.00	0.00	0.00	0.00	0.00	0.00
Carbapenem resistant *Acinetobacter spp.* (excluding those resistant to colistin)	1.53	2.38	3.54	4.96	12.71	22.73	6.75	15.21	25.72	4.74	8.85	12.98	0.02	0.02	0.04
Aminoglycosides and fluoroquinolones resistant *Acinetobacter* spp. (excluding those resistant to colistin and/or carbapenem)	0.00	0.00	0.00	0.00	0.00	0.00	0.00	0.00	0.00	0.00	0.00	0.00	0.00	0.00	0.00
Vancomycin resistant Enterococci (*E. faecalis* and *E. faecium*)	0.45	0.93	1.88	4.63	9.63	17.89	5.15	10.56	19.54	5.94	6.15	6.39	0.00	0.01	0.02
Colistin resistant *Escherichia coli*	0.00	0.00	0.00	0.00	0.00	0.00	0.00	0.00	0.00	0.00	0.00	0.00	0.00	0.00	0.00
Carbapenem resistant *Escherichia coli* (excluding those resistant to colistin)	4.20	6.05	8.35	41.92	65.69	95.89	46.76	71.62	103.20	9.90	14.12	18.57	0.04	0.06	0.09
Third-generation cephalosporin resistant *Escherichia coli* (excluding those resistant to colistin and/or carbapenem)	337.75	373.96	413.70	1,988.39	2,218.23	2,442.21	2,338.10	2,591.23	2,844.66	5.31	5.74	6.21	3.47	3.84	4.24
Colistin resistant *Klebsiella pneumoniae*	0.00	0.00	0.00	0.00	0.00	0.00	0.00	0.00	0.00	0.00	0.00	0.00	0.00	0.00	0.00
Carbapenem resistant *Klebsiella pneumoniae* (excluding those resistant to colistin)	0.00	0.00	0.00	0.00	0.00	0.00	0.00	0.00	0.00	0.00	0.00	0.00	0.00	0.00	0.00
Third-generation cephalosporin resistant *Klebsiella pneumoniae* (excluding those resistant to colistin and/or carbapenem)	36.89	41.85	47.03	182.64	200.47	220.27	220.96	242.45	265.91	5.65	5.99	6.37	0.38	0.43	0.48
Colistin resistant *Pseudomonas aeruginosa*	0.00	0.00	0.00	0.00	0.00	0.00	0.00	0.00	0.00	0.00	0.00	0.00	0.00	0.00	0.00
Carbapenem resistant *Pseudomonas aeruginosa* (excluding those resistant to colistin)	83.39	98.23	116.58	378.10	495.50	640.08	469.00	592.88	744.72	8.09	10.17	12.35	0.86	1.01	1.20
Pseudomonas aeruginosa resistant to three or more antimicrobial groups (excluding those resistant to colistin and/or carbapenem)	1.30	2.00	2.91	4.26	9.20	15.63	5.87	11.27	18.37	1.51	2.56	3.63	0.01	0.02	0.03
Methicillin resistant *Staphylococcus aureus*	51.06	57.55	64.82	246.01	269.14	293.48	299.14	326.63	356.61	8.21	8.71	9.30	0.52	0.59	0.67
Penicillin and macrolides resistant *Streptococcus pneumoniae* (excluding those only resistant to penicillin)	52.20	60.87	71.32	180.80	198.23	219.28	234.78	259.09	287.58	5.21	5.50	5.81	0.54	0.62	0.73
Penicillin tesistant *Streptococcus pneumoniae* (excluding those resistant to macrolides)	52.08	61.01	72.93	180.33	199.29	223.88	234.36	260.28	293.17	6.66	7.05	7.44	0.53	0.63	0.75
Total	620.84	704.82	803.06	3,212.05	3,678.09	4,191.35	3,860.87	4,381.22	4,959.49				6.37	7.23	8.24

Infection	YLL per 100,000			DALY per 100,000			Cases per year			Incidence per 100,000			Deaths per year			Mortality per 100,000		
Colistin resistant *Acinetobacter spp.*	0.00	0.00	0.00	0.00	0.00	0.00	0.00	0.00	0.00	0.00	0.00	0.00	0.00	0.00	0.00	0.00	0.00	0.00
Carbapenem resistant *Acinetobacter spp.* (excluding those resistant to colistin)	0.05	0.13	0.23	0.07	0.16	0.26	3.03	5.01	7.10	0.03	0.05	0.07	0.17	0.42	0.76	0.00	0.00	0.01
Aminoglycosides and fluoroquinolones resistant *Acinetobacter* spp. (excluding those resistant to colistin and/or carbapenem)	0.00	0.00	0.00	0.00	0.00	0.00	0.00	0.00	0.00	0.00	0.00	0.00	0.00	0.00	0.00	0.00	0.00	0.00
Vancomycin resistant Enterococci (*E. faecalis* and *E. faecium*)	0.05	0.10	0.18	0.05	0.11	0.20	2.26	6.62	11.89	0.02	0.07	0.12	0.22	0.46	0.85	0.00	0.00	0.01
Colistin resistant *Escherichia coli*	0.00	0.00	0.00	0.00	0.00	0.00	0.00	0.00	0.00	0.00	0.00	0.00	0.00	0.00	0.00	0.00	0.00	0.00
Carbapenem resistant *Escherichia coli* (excluding those resistant to colistin)	0.43	0.67	0.98	0.48	0.73	1.06	27.41	40.53	55.12	0.28	0.42	0.57	1.34	2.19	3.33	0.01	0.02	0.03
Third-generation cephalosporin resistant *Escherichia coli* (excluding those resistant to colistin and/or carbapenem)	20.40	22.76	25.06	23.99	26.58	29.18	3,194.27	3,601.38	4,002.14	32.77	36.95	41.06	96.83	110.14	123.19	0.99	1.13	1.26
Colistin resistant *Klebsiella pneumoniae*	0.00	0.00	0.00	0.00	0.00	0.00	0.00	0.00	0.00	0.00	0.00	0.00	0.00	0.00	0.00	0.00	0.00	0.00
Carbapenem resistant *Klebsiella pneumoniae* (excluding those resistant to colistin)	0.00	0.00	0.00	0.00	0.00	0.00	0.00	0.00	0.00	0.00	0.00	0.00	0.00	0.00	0.00	0.00	0.00	0.00
Third-generation cephalosporin resistant *Klebsiella pneumoniae* (excluding those resistant to colistin and/or carbapenem)	1.87	2.06	2.26	2.27	2.49	2.73	147.45	167.21	186.99	1.51	1.72	1.92	8.22	9.09	9.95	0.08	0.09	0.10
Colistin resistant *Pseudomonas aeruginosa*	0.00	0.00	0.00	0.00	0.00	0.00	0.00	0.00	0.00	0.00	0.00	0.00	0.00	0.00	0.00	0.00	0.00	0.00
Carbapenem resistant *Pseudomonas aeruginosa* (excluding those resistant to colistin)	3.88	5.08	6.57	4.81	6.08	7.64	221.42	261.51	301.75	2.27	2.68	3.10	13.94	17.73	22.28	0.14	0.18	0.23
Pseudomonas aeruginosa resistant to three or more antimicrobial groups (excluding those resistant to colistin and/or carbapenem)	0.04	0.09	0.16	0.06	0.12	0.19	12.43	20.01	28.01	0.13	0.21	0.29	0.61	1.29	2.16	0.01	0.01	0.02
Methicillin resistant *Staphylococcus aureus*	2.52	2.76	3.01	3.07	3.35	3.66	175.91	194.27	212.84	1.80	1.99	2.18	8.09	8.88	9.68	0.08	0.09	0.10
Penicillin and macrolides resistant *Streptococcus pneumoniae* (excluding those only resistant to penicillin)	1.85	2.03	2.25	2.41	2.66	2.95	137.64	153.31	174.06	1.41	1.57	1.79	8.59	9.42	10.45	0.09	0.10	0.11
Penicillin tesistant *Streptococcus pneumoniae* (excluding those resistant to macrolides)	1.85	2.04	2.30	2.40	2.67	3.01	107.27	120.70	139.88	1.10	1.24	1.44	6.69	7.38	8.30	0.07	0.08	0.09
Total	32.95	37.73	43.00	39.61	44.95	50.88	4,029.07	4,570.54	5,119.78	41.34	46.89	52.52	144.70	166.99	190.94	1.48	1.71	1.96

Model	YLD			YLL			DALY			DALY per Case			YLD per 100,000		
Sweden - Acinetobacter spp. CRACI BSI - model 36	0.94	1.36	1.97	3.75	10.56	19.52	4.90	12.01	21.05	3.06	6.93	10.83	9.61E-03	0.01	0.02
Sweden - Acinetobacter spp. CRACI OTH - model 40	3.19E-05	2.11E-04	6.50E-04	0.00	0.00	0.00	3.19E-05	2.11E-04	6.50E-04	1.49E-03	3.72E-03	6.44E-03	3.28E-07	2.17E-06	6.66E-06
Sweden - Acinetobacter spp. CRACI RESP - model 37	0.59	1.01	1.56	1.15	1.98	2.94	1.78	3.03	4.39	1.40	1.64	1.86	6.08E-03	0.01	0.02
Sweden - Acinetobacter spp. CRACI SSI - model 39	2.31E-04	1.19E-03	3.55E-03	0.07	0.17	0.27	0.07	0.17	0.28	0.27	0.27	0.28	2.37E-06	1.22E-05	3.64E-05
Sweden - Acinetobacter spp. CRACI UTI - model 38	4.44E-04	1.30E-03	3.27E-03	0.00	0.00	0.00	4.44E-04	1.30E-03	3.27E-03	8.03E-04	1.97E-03	3.67E-03	4.56E-06	1.33E-05	3.35E-05
Sweden - Acinetobacter spp. ColRACI BSI - model 41	0.00	0.00	0.00	0.00	0.00	0.00	0.00	0.00	0.00	NaN	NaN	NaN	0.00	0.00	0.00
Sweden - Acinetobacter spp. ColRACI OTH - model 45	0.00	0.00	0.00	0.00	0.00	0.00	0.00	0.00	0.00	NaN	NaN	NaN	0.00	0.00	0.00
Sweden - Acinetobacter spp. ColRACI RESP - model 42	0.00	0.00	0.00	0.00	0.00	0.00	0.00	0.00	0.00	NaN	NaN	NaN	0.00	0.00	0.00
Sweden - Acinetobacter spp. ColRACI SSI - model 44	0.00	0.00	0.00	0.00	0.00	0.00	0.00	0.00	0.00	NaN	NaN	NaN	0.00	0.00	0.00
Sweden - Acinetobacter spp. ColRACI UTI - model 43	0.00	0.00	0.00	0.00	0.00	0.00	0.00	0.00	0.00	NaN	NaN	NaN	0.00	0.00	0.00
Sweden - Acinetobacter spp. MDRACI BSI - model 31	0.00	0.00	0.00	0.00	0.00	0.00	0.00	0.00	0.00	NaN	NaN	NaN	0.00	0.00	0.00
Sweden - Acinetobacter spp. MDRACI OTH - model 35	0.00	0.00	0.00	0.00	0.00	0.00	0.00	0.00	0.00	NaN	NaN	NaN	0.00	0.00	0.00
Sweden - Acinetobacter spp. MDRACI RESP - model 32	0.00	0.00	0.00	0.00	0.00	0.00	0.00	0.00	0.00	NaN	NaN	NaN	0.00	0.00	0.00
Sweden - Acinetobacter spp. MDRACI SSI - model 34	0.00	0.00	0.00	0.00	0.00	0.00	0.00	0.00	0.00	NaN	NaN	NaN	0.00	0.00	0.00
Sweden - Acinetobacter spp. MDRACI UTI - model 33	0.00	0.00	0.00	0.00	0.00	0.00	0.00	0.00	0.00	NaN	NaN	NaN	0.00	0.00	0.00
Sweden - Enterococcus faecalis and E. faecium VRE BSI - model 66	0.45	0.92	1.86	4.11	7.86	14.79	4.62	8.78	16.42	5.18	5.39	5.62	4.63E-03	9.44E-03	0.02
Sweden - Enterococcus faecalis and E. faecium VRE OTH - model 70	5.94E-04	2.64E-03	7.29E-03	0.00	0.00	0.00	5.94E-04	2.64E-03	7.29E-03	5.68E-04	1.58E-03	3.19E-03	6.09E-06	2.71E-05	7.48E-05
Sweden - Enterococcus faecalis and E. faecium VRE RESP - model 67	0.00	0.00	0.00	0.00	0.00	0.00	0.00	0.00	0.00	NaN	NaN	NaN	0.00	0.00	0.00
Sweden - Enterococcus faecalis and E. faecium VRE SSI - model 69	7.15E-04	4.23E-03	0.01	0.52	1.78	3.10	0.52	1.78	3.11	0.76	0.76	0.76	7.33E-06	4.34E-05	1.33E-04
Sweden - Enterococcus faecalis and E. faecium VRE UTI - model 68	2.70E-04	1.43E-03	3.96E-03	0.00	0.00	0.00	2.70E-04	1.43E-03	3.96E-03	7.40E-04	1.81E-03	3.44E-03	2.77E-06	1.47E-05	4.06E-05
Sweden - Escherichia coli 3GCREC BSI - model 16	259.25	281.34	306.24	1,653.32	1,824.52	1,991.22	1,922.67	2,104.89	2,287.71	3.87	4.17	4.49	2.66	2.89	3.14
Sweden - Escherichia coli 3GCREC OTH - model 20	0.42	0.55	0.73	0.00	0.00	0.00	0.42	0.55	0.73	2.70E-03	3.32E-03	4.03E-03	4.30E-03	5.65E-03	7.49E-03
Sweden - Escherichia coli 3GCREC RESP - model 17	73.86	86.68	99.94	144.93	168.41	193.24	219.77	255.12	291.59	1.03	1.14	1.25	0.76	0.89	1.03
Sweden - Escherichia coli 3GCREC SSI - model 19	0.84	1.14	1.49	190.15	225.29	257.75	191.86	226.42	259.33	0.40	0.42	0.45	8.61E-03	0.01	0.02
Sweden - Escherichia coli 3GCREC UTI - model 18	3.37	4.25	5.30	0.00	0.00	0.00	3.37	4.25	5.30	1.61E-03	1.96E-03	2.34E-03	0.03	0.04	0.05
Sweden - Escherichia coli CREC BSI - model 21	3.28	4.44	5.93	37.37	58.18	85.17	41.29	62.51	90.13	7.89	11.74	15.65	0.03	0.05	0.06
Sweden - Escherichia coli CREC OTH - model 25	3.88E-03	0.01	0.02	0.00	0.00	0.00	3.88E-03	0.01	0.02	1.52E-03	3.39E-03	6.55E-03	3.98E-05	1.04E-04	2.44E-04
Sweden - Escherichia coli CREC RESP - model 22	0.89	1.54	2.27	1.70	3.00	4.45	2.59	4.54	6.67	1.35	1.67	2.18	9.18E-03	0.02	0.02
Sweden - Escherichia coli CREC SSI - model 24	4.59E-03	0.01	0.03	2.85	4.50	6.27	2.85	4.51	6.28	0.67	0.70	0.73	4.71E-05	1.28E-04	2.95E-04
Sweden - Escherichia coli CREC UTI - model 23	0.02	0.05	0.09	0.00	0.00	0.00	0.02	0.05	0.09	9.42E-04	2.05E-03	3.64E-03	2.01E-04	4.71E-04	9.48E-04
Sweden - Escherichia coli ColREC BSI - model 26	0.00	0.00	0.00	0.00	0.00	0.00	0.00	0.00	0.00	NaN	NaN	NaN	0.00	0.00	0.00
Sweden - Escherichia coli ColREC OTH - model 30	0.00	0.00	0.00	0.00	0.00	0.00	0.00	0.00	0.00	NaN	NaN	NaN	0.00	0.00	0.00
Sweden - Escherichia coli ColREC RESP - model 27	0.00	0.00	0.00	0.00	0.00	0.00	0.00	0.00	0.00	NaN	NaN	NaN	0.00	0.00	0.00
Sweden - Escherichia coli ColREC SSI - model 29	0.00	0.00	0.00	0.00	0.00	0.00	0.00	0.00	0.00	NaN	NaN	NaN	0.00	0.00	0.00
Sweden - Escherichia coli ColREC UTI - model 28	0.00	0.00	0.00	0.00	0.00	0.00	0.00	0.00	0.00	NaN	NaN	NaN	0.00	0.00	0.00
Sweden - Klebsiella pneumoniae 3GCRKP BSI - model 1	23.33	26.00	28.81	150.39	162.86	177.19	174.88	188.96	204.89	4.12	4.33	4.57	0.24	0.27	0.30
Sweden - Klebsiella pneumoniae 3GCRKP OTH - model 5	0.04	0.06	0.08	0.00	0.00	0.00	0.04	0.06	0.08	2.59E-03	3.34E-03	4.14E-03	4.42E-04	5.93E-04	7.73E-04
Sweden - Klebsiella pneumoniae 3GCRKP RESP - model 2	13.41	15.66	17.97	26.03	30.24	34.48	39.71	45.92	52.17	1.13	1.22	1.32	0.14	0.16	0.18
Sweden - Klebsiella pneumoniae 3GCRKP SSI - model 4	0.03	0.04	0.05	6.21	7.37	8.60	6.26	7.41	8.64	0.40	0.43	0.46	2.67E-04	3.74E-04	5.00E-04
Sweden - Klebsiella pneumoniae 3GCRKP UTI - model 3	0.08	0.10	0.13	0.00	0.00	0.00	0.08	0.10	0.13	1.56E-03	1.98E-03	2.41E-03	7.89E-04	1.03E-03	1.32E-03
Sweden - Klebsiella pneumoniae CRKP BSI - model 6	0.00	0.00	0.00	0.00	0.00	0.00	0.00	0.00	0.00	NaN	NaN	NaN	0.00	0.00	0.00
Sweden - Klebsiella pneumoniae CRKP OTH - model 10	0.00	0.00	0.00	0.00	0.00	0.00	0.00	0.00	0.00	NaN	NaN	NaN	0.00	0.00	0.00
Sweden - Klebsiella pneumoniae CRKP RESP - model 7	0.00	0.00	0.00	0.00	0.00	0.00	0.00	0.00	0.00	NaN	NaN	NaN	0.00	0.00	0.00
Sweden - Klebsiella pneumoniae CRKP SSI - model 9	0.00	0.00	0.00	0.00	0.00	0.00	0.00	0.00	0.00	NaN	NaN	NaN	0.00	0.00	0.00
Sweden - Klebsiella pneumoniae CRKP UTI - model 8	0.00	0.00	0.00	0.00	0.00	0.00	0.00	0.00	0.00	NaN	NaN	NaN	0.00	0.00	0.00
Sweden - Klebsiella pneumoniae ColRKP BSI - model 11	0.00	0.00	0.00	0.00	0.00	0.00	0.00	0.00	0.00	NaN	NaN	NaN	0.00	0.00	0.00
Sweden - Klebsiella pneumoniae ColRKP OTH - model 15	0.00	0.00	0.00	0.00	0.00	0.00	0.00	0.00	0.00	NaN	NaN	NaN	0.00	0.00	0.00
Sweden - Klebsiella pneumoniae ColRKP RESP - model 12	0.00	0.00	0.00	0.00	0.00	0.00	0.00	0.00	0.00	NaN	NaN	NaN	0.00	0.00	0.00
Sweden - Klebsiella pneumoniae ColRKP SSI - model 14	0.00	0.00	0.00	0.00	0.00	0.00	0.00	0.00	0.00	NaN	NaN	NaN	0.00	0.00	0.00
Sweden - Klebsiella pneumoniae ColRKP UTI - model 13	0.00	0.00	0.00	0.00	0.00	0.00	0.00	0.00	0.00	NaN	NaN	NaN	0.00	0.00	0.00
Sweden - Pseudomonas aeruginosa CRPA BSI - model 51	24.07	27.53	32.12	180.09	235.51	307.48	207.35	262.48	335.04	5.68	7.30	8.97	0.25	0.28	0.33
Sweden - Pseudomonas aeruginosa CRPA OTH - model 55	0.05	0.07	0.09	0.00	0.00	0.00	0.05	0.07	0.09	2.88E-03	3.66E-03	4.47E-03	5.08E-04	6.94E-04	9.05E-04
Sweden - Pseudomonas aeruginosa CRPA RESP - model 52	59.16	70.48	84.18	188.82	248.17	317.90	252.30	318.35	394.69	1.97	2.38	2.86	0.61	0.72	0.86
Sweden - Pseudomonas aeruginosa CRPA SSI - model 54	0.04	0.05	0.07	9.19	11.82	14.69	9.23	11.88	14.77	0.44	0.48	0.52	3.76E-04	5.39E-04	7.58E-04
Sweden - Pseudomonas aeruginosa CRPA UTI - model 53	0.07	0.10	0.12	0.00	0.00	0.00	0.07	0.10	0.12	1.66E-03	2.06E-03	2.50E-03	7.51E-04	9.97E-04	1.27E-03
Sweden - Pseudomonas aeruginosa ColRPA BSI - model 56	0.00	0.00	0.00	0.00	0.00	0.00	0.00	0.00	0.00	NaN	NaN	NaN	0.00	0.00	0.00
Sweden - Pseudomonas aeruginosa ColRPA OTH - model 60	0.00	0.00	0.00	0.00	0.00	0.00	0.00	0.00	0.00	NaN	NaN	NaN	0.00	0.00	0.00
Sweden - Pseudomonas aeruginosa ColRPA RESP - model 57	0.00	0.00	0.00	0.00	0.00	0.00	0.00	0.00	0.00	NaN	NaN	NaN	0.00	0.00	0.00
Sweden - Pseudomonas aeruginosa ColRPA SSI - model 59	0.00	0.00	0.00	0.00	0.00	0.00	0.00	0.00	0.00	NaN	NaN	NaN	0.00	0.00	0.00
Sweden - Pseudomonas aeruginosa ColRPA UTI - model 58	0.00	0.00	0.00	0.00	0.00	0.00	0.00	0.00	0.00	NaN	NaN	NaN	0.00	0.00	0.00
Sweden - Pseudomonas aeruginosa MDRPA BSI - model 46	0.40	0.56	0.80	1.86	4.16	6.99	2.38	4.74	7.75	0.91	1.71	2.50	4.06E-03	5.74E-03	8.22E-03
Sweden - Pseudomonas aeruginosa MDRPA OTH - model 50	2.18E-03	5.16E-03	9.54E-03	0.00	0.00	0.00	2.18E-03	5.16E-03	9.54E-03	1.95E-03	3.75E-03	5.74E-03	2.23E-05	5.29E-05	9.79E-05
Sweden - Pseudomonas aeruginosa MDRPA RESP - model 47	0.90	1.42	2.08	2.16	4.55	7.88	3.24	6.02	9.83	0.38	0.59	0.81	9.23E-03	0.01	0.02
Sweden - Pseudomonas aeruginosa MDRPA SSI - model 49	1.28E-03	3.80E-03	8.62E-03	0.24	0.49	0.76	0.25	0.50	0.77	0.21	0.26	0.31	1.31E-05	3.90E-05	8.84E-05
Sweden - Pseudomonas aeruginosa MDRPA UTI - model 48	3.04E-03	7.00E-03	0.01	0.00	0.00	0.00	3.04E-03	7.00E-03	0.01	1.02E-03	2.01E-03	3.19E-03	3.12E-05	7.18E-05	1.36E-04
Sweden - Staphylococcus aureus MRSA BSI - model 61	25.25	28.08	31.19	175.67	189.54	204.74	202.14	217.53	235.00	6.22	6.56	6.97	0.26	0.29	0.32
Sweden - Staphylococcus aureus MRSA OTH - model 65	0.12	0.16	0.20	0.00	0.00	0.00	0.12	0.16	0.20	2.62E-03	3.28E-03	3.91E-03	1.27E-03	1.64E-03	2.01E-03
Sweden - Staphylococcus aureus MRSA RESP - model 62	25.59	29.19	33.26	50.33	57.27	64.24	76.75	86.49	96.76	1.59	1.73	1.89	0.26	0.30	0.34
Sweden - Staphylococcus aureus MRSA SSI - model 64	0.09	0.12	0.15	20.01	22.33	24.50	20.11	22.43	24.64	0.40	0.41	0.43	9.06E-04	1.19E-03	1.55E-03
Sweden - Staphylococcus aureus MRSA UTI - model 63	0.01	0.02	0.02	0.00	0.00	0.00	0.01	0.02	0.02	1.48E-03	1.87E-03	2.28E-03	1.17E-04	1.56E-04	2.04E-04
Sweden - Streptococcus pneumoniae PMRSP BSI - model 76	15.11	16.61	18.36	107.25	112.51	118.21	123.50	129.25	135.00	4.16	4.35	4.55	0.16	0.17	0.19
Sweden - Streptococcus pneumoniae PMRSP OTH - model 80	0.02	0.02	0.03	0.00	0.00	0.00	0.02	0.02	0.03	1.51E-03	1.95E-03	2.50E-03	1.62E-04	2.12E-04	2.75E-04
Sweden - Streptococcus pneumoniae PMRSP RESP - model 77	37.07	44.23	52.94	73.55	85.72	101.07	111.27	129.82	152.56	1.04	1.15	1.26	0.38	0.45	0.54
Sweden - Streptococcus pneumoniae PMRSP SSI - model 79	0.00	0.00	0.00	0.00	0.00	0.00	0.00	0.00	0.00	NaN	NaN	NaN	0.00	0.00	0.00
Sweden - Streptococcus pneumoniae PMRSP UTI - model 78	0.00	0.00	0.00	0.00	0.00	0.00	0.00	0.00	0.00	NaN	NaN	NaN	0.00	0.00	0.00
Sweden - Streptococcus pneumoniae PRSP BSI - model 71	14.93	16.66	18.50	107.03	113.04	119.34	123.09	129.64	136.00	5.30	5.58	5.85	0.15	0.17	0.19
Sweden - Streptococcus pneumoniae PRSP OTH - model 75	0.01	0.02	0.02	0.00	0.00	0.00	0.01	0.02	0.02	1.47E-03	1.94E-03	2.60E-03	1.21E-04	1.65E-04	2.22E-04
Sweden - Streptococcus pneumoniae PRSP RESP - model 72	37.14	44.33	54.40	73.30	86.26	104.54	111.26	130.62	157.15	1.36	1.47	1.59	0.38	0.45	0.56
Sweden - Streptococcus pneumoniae PRSP SSI - model 74	0.00	0.00	0.00	0.00	0.00	0.00	0.00	0.00	0.00	NaN	NaN	NaN	0.00	0.00	0.00
Sweden - Streptococcus pneumoniae PRSP UTI - model 73	0.00	0.00	0.00	0.00	0.00	0.00	0.00	0.00	0.00	NaN	NaN	NaN	0.00	0.00	0.00
**Total (Median & 95% UIs)**	620.84	704.82	803.06	3,212.05	3,678.09	4,191.35	3,860.87	4,381.22	4,959.49	-			6.37	7.23	8.24
**Total (average)**	**706.43**			**3,683.83**			**4,390.26**			-			**7.25**		

Model	YLL per 100,000			DALY per 100,000			Cases per year			Incidence per 100,000			Deaths per year			Mortality per 100,000		
Sweden - Acinetobacter spp. CRACI BSI - model 36	0.04	0.11	0.20	0.05	0.12	0.22	1.36	1.73	2.19	0.01	0.02	0.02	0.12	0.35	0.65	1.28E-03	3.61E-03	6.66E-03
Sweden - Acinetobacter spp. CRACI OTH - model 40	0.00	0.00	0.00	3.28E-07	2.17E-06	6.66E-06	9.83E-03	0.06	0.13	1.01E-04	6.42E-04	1.31E-03	0.00	0.00	0.00	0.00	0.00	0.00
Sweden - Acinetobacter spp. CRACI RESP - model 37	0.01	0.02	0.03	0.02	0.03	0.05	1.09	1.88	2.63	0.01	0.02	0.03	0.04	0.07	0.10	3.91E-04	6.78E-04	1.01E-03
Sweden - Acinetobacter spp. CRACI SSI - model 39	7.12E-04	1.75E-03	2.81E-03	7.20E-04	1.76E-03	2.82E-03	0.26	0.63	1.01	2.63E-03	6.46E-03	0.01	2.31E-03	5.67E-03	9.09E-03	2.37E-05	5.82E-05	9.33E-05
Sweden - Acinetobacter spp. CRACI UTI - model 38	0.00	0.00	0.00	4.56E-06	1.33E-05	3.35E-05	0.31	0.71	1.14	3.13E-03	7.26E-03	0.01	0.00	0.00	0.00	0.00	0.00	0.00
Sweden - Acinetobacter spp. ColRACI BSI - model 41	0.00	0.00	0.00	0.00	0.00	0.00	0.00	0.00	0.00	0.00	0.00	0.00	0.00	0.00	0.00	0.00	0.00	0.00
Sweden - Acinetobacter spp. ColRACI OTH - model 45	0.00	0.00	0.00	0.00	0.00	0.00	0.00	0.00	0.00	0.00	0.00	0.00	0.00	0.00	0.00	0.00	0.00	0.00
Sweden - Acinetobacter spp. ColRACI RESP - model 42	0.00	0.00	0.00	0.00	0.00	0.00	0.00	0.00	0.00	0.00	0.00	0.00	0.00	0.00	0.00	0.00	0.00	0.00
Sweden - Acinetobacter spp. ColRACI SSI - model 44	0.00	0.00	0.00	0.00	0.00	0.00	0.00	0.00	0.00	0.00	0.00	0.00	0.00	0.00	0.00	0.00	0.00	0.00
Sweden - Acinetobacter spp. ColRACI UTI - model 43	0.00	0.00	0.00	0.00	0.00	0.00	0.00	0.00	0.00	0.00	0.00	0.00	0.00	0.00	0.00	0.00	0.00	0.00
Sweden - Acinetobacter spp. MDRACI BSI - model 31	0.00	0.00	0.00	0.00	0.00	0.00	0.00	0.00	0.00	0.00	0.00	0.00	0.00	0.00	0.00	0.00	0.00	0.00
Sweden - Acinetobacter spp. MDRACI OTH - model 35	0.00	0.00	0.00	0.00	0.00	0.00	0.00	0.00	0.00	0.00	0.00	0.00	0.00	0.00	0.00	0.00	0.00	0.00
Sweden - Acinetobacter spp. MDRACI RESP - model 32	0.00	0.00	0.00	0.00	0.00	0.00	0.00	0.00	0.00	0.00	0.00	0.00	0.00	0.00	0.00	0.00	0.00	0.00
Sweden - Acinetobacter spp. MDRACI SSI - model 34	0.00	0.00	0.00	0.00	0.00	0.00	0.00	0.00	0.00	0.00	0.00	0.00	0.00	0.00	0.00	0.00	0.00	0.00
Sweden - Acinetobacter spp. MDRACI UTI - model 33	0.00	0.00	0.00	0.00	0.00	0.00	0.00	0.00	0.00	0.00	0.00	0.00	0.00	0.00	0.00	0.00	0.00	0.00
Sweden - Enterococcus faecalis and E. faecium VRE BSI - model 66	0.04	0.08	0.15	0.05	0.09	0.17	0.85	1.64	3.04	8.71E-03	0.02	0.03	0.19	0.37	0.70	2.00E-03	3.82E-03	7.18E-03
Sweden - Enterococcus faecalis and E. faecium VRE OTH - model 70	0.00	0.00	0.00	6.09E-06	2.71E-05	7.48E-05	0.51	1.80	3.17	5.24E-03	0.02	0.03	0.00	0.00	0.00	0.00	0.00	0.00
Sweden - Enterococcus faecalis and E. faecium VRE RESP - model 67	0.00	0.00	0.00	0.00	0.00	0.00	0.00	0.00	0.00	0.00	0.00	0.00	0.00	0.00	0.00	0.00	0.00	0.00
Sweden - Enterococcus faecalis and E. faecium VRE SSI - model 69	5.35E-03	0.02	0.03	5.38E-03	0.02	0.03	0.69	2.34	4.09	7.05E-03	0.02	0.04	0.02	0.08	0.15	2.54E-04	8.63E-04	1.51E-03
Sweden - Enterococcus faecalis and E. faecium VRE UTI - model 68	0.00	0.00	0.00	2.77E-06	1.47E-05	4.06E-05	0.21	0.84	1.59	2.14E-03	8.64E-03	0.02	0.00	0.00	0.00	0.00	0.00	0.00
Sweden - Escherichia coli 3GCREC BSI - model 16	16.96	18.72	20.43	19.73	21.59	23.47	466.67	502.70	541.24	4.79	5.16	5.55	77.83	87.34	96.75	0.80	0.90	0.99
Sweden - Escherichia coli 3GCREC OTH - model 20	0.00	0.00	0.00	4.30E-03	5.65E-03	7.49E-03	137.52	166.34	199.09	1.41	1.71	2.04	0.00	0.00	0.00	0.00	0.00	0.00
Sweden - Escherichia coli 3GCREC RESP - model 17	1.49	1.73	1.98	2.25	2.62	2.99	186.64	224.16	259.78	1.91	2.30	2.67	6.71	8.07	9.40	0.07	0.08	0.10
Sweden - Escherichia coli 3GCREC SSI - model 19	1.95	2.31	2.64	1.97	2.32	2.66	459.47	533.23	601.06	4.71	5.47	6.17	12.29	14.72	17.03	0.13	0.15	0.17
Sweden - Escherichia coli 3GCREC UTI - model 18	0.00	0.00	0.00	0.03	0.04	0.05	1,943.97	2,174.94	2,400.97	19.94	22.31	24.63	0.00	0.00	0.00	0.00	0.00	0.00
Sweden - Escherichia coli CREC BSI - model 21	0.38	0.60	0.87	0.42	0.64	0.92	4.36	5.42	6.87	0.04	0.06	0.07	1.18	1.91	2.92	0.01	0.02	0.03
Sweden - Escherichia coli CREC OTH - model 25	0.00	0.00	0.00	3.98E-05	1.04E-04	2.44E-04	1.76	3.07	4.59	0.02	0.03	0.05	0.00	0.00	0.00	0.00	0.00	0.00
Sweden - Escherichia coli CREC RESP - model 22	0.02	0.03	0.05	0.03	0.05	0.07	1.46	2.71	4.06	0.02	0.03	0.04	0.05	0.10	0.15	5.27E-04	9.88E-04	1.53E-03
Sweden - Escherichia coli CREC SSI - model 24	0.03	0.05	0.06	0.03	0.05	0.06	4.10	6.38	8.88	0.04	0.07	0.09	0.11	0.19	0.27	1.13E-03	1.92E-03	2.75E-03
Sweden - Escherichia coli CREC UTI - model 23	0.00	0.00	0.00	2.01E-04	4.71E-04	9.48E-04	15.72	22.96	30.72	0.16	0.24	0.32	0.00	0.00	0.00	0.00	0.00	0.00
Sweden - Escherichia coli ColREC BSI - model 26	0.00	0.00	0.00	0.00	0.00	0.00	0.00	0.00	0.00	0.00	0.00	0.00	0.00	0.00	0.00	0.00	0.00	0.00
Sweden - Escherichia coli ColREC OTH - model 30	0.00	0.00	0.00	0.00	0.00	0.00	0.00	0.00	0.00	0.00	0.00	0.00	0.00	0.00	0.00	0.00	0.00	0.00
Sweden - Escherichia coli ColREC RESP - model 27	0.00	0.00	0.00	0.00	0.00	0.00	0.00	0.00	0.00	0.00	0.00	0.00	0.00	0.00	0.00	0.00	0.00	0.00
Sweden - Escherichia coli ColREC SSI - model 29	0.00	0.00	0.00	0.00	0.00	0.00	0.00	0.00	0.00	0.00	0.00	0.00	0.00	0.00	0.00	0.00	0.00	0.00
Sweden - Escherichia coli ColREC UTI - model 28	0.00	0.00	0.00	0.00	0.00	0.00	0.00	0.00	0.00	0.00	0.00	0.00	0.00	0.00	0.00	0.00	0.00	0.00
Sweden - Klebsiella pneumoniae 3GCRKP BSI - model 1	1.54	1.67	1.82	1.79	1.94	2.10	40.66	43.58	46.66	0.42	0.45	0.48	6.71	7.29	7.89	0.07	0.07	0.08
Sweden - Klebsiella pneumoniae 3GCRKP OTH - model 5	0.00	0.00	0.00	4.42E-04	5.93E-04	7.73E-04	14.70	17.35	19.96	0.15	0.18	0.20	0.00	0.00	0.00	0.00	0.00	0.00
Sweden - Klebsiella pneumoniae 3GCRKP RESP - model 2	0.27	0.31	0.35	0.41	0.47	0.54	32.58	37.65	42.39	0.33	0.39	0.43	1.15	1.36	1.55	0.01	0.01	0.02
Sweden - Klebsiella pneumoniae 3GCRKP SSI - model 4	0.06	0.08	0.09	0.06	0.08	0.09	14.71	17.13	19.95	0.15	0.18	0.20	0.36	0.44	0.52	3.70E-03	4.47E-03	5.29E-03
Sweden - Klebsiella pneumoniae 3GCRKP UTI - model 3	0.00	0.00	0.00	7.89E-04	1.03E-03	1.32E-03	44.79	51.50	58.03	0.46	0.53	0.60	0.00	0.00	0.00	0.00	0.00	0.00
Sweden - Klebsiella pneumoniae CRKP BSI - model 6	0.00	0.00	0.00	0.00	0.00	0.00	0.00	0.00	0.00	0.00	0.00	0.00	0.00	0.00	0.00	0.00	0.00	0.00
Sweden - Klebsiella pneumoniae CRKP OTH - model 10	0.00	0.00	0.00	0.00	0.00	0.00	0.00	0.00	0.00	0.00	0.00	0.00	0.00	0.00	0.00	0.00	0.00	0.00
Sweden - Klebsiella pneumoniae CRKP RESP - model 7	0.00	0.00	0.00	0.00	0.00	0.00	0.00	0.00	0.00	0.00	0.00	0.00	0.00	0.00	0.00	0.00	0.00	0.00
Sweden - Klebsiella pneumoniae CRKP SSI - model 9	0.00	0.00	0.00	0.00	0.00	0.00	0.00	0.00	0.00	0.00	0.00	0.00	0.00	0.00	0.00	0.00	0.00	0.00
Sweden - Klebsiella pneumoniae CRKP UTI - model 8	0.00	0.00	0.00	0.00	0.00	0.00	0.00	0.00	0.00	0.00	0.00	0.00	0.00	0.00	0.00	0.00	0.00	0.00
Sweden - Klebsiella pneumoniae ColRKP BSI - model 11	0.00	0.00	0.00	0.00	0.00	0.00	0.00	0.00	0.00	0.00	0.00	0.00	0.00	0.00	0.00	0.00	0.00	0.00
Sweden - Klebsiella pneumoniae ColRKP OTH - model 15	0.00	0.00	0.00	0.00	0.00	0.00	0.00	0.00	0.00	0.00	0.00	0.00	0.00	0.00	0.00	0.00	0.00	0.00
Sweden - Klebsiella pneumoniae ColRKP RESP - model 12	0.00	0.00	0.00	0.00	0.00	0.00	0.00	0.00	0.00	0.00	0.00	0.00	0.00	0.00	0.00	0.00	0.00	0.00
Sweden - Klebsiella pneumoniae ColRKP SSI - model 14	0.00	0.00	0.00	0.00	0.00	0.00	0.00	0.00	0.00	0.00	0.00	0.00	0.00	0.00	0.00	0.00	0.00	0.00
Sweden - Klebsiella pneumoniae ColRKP UTI - model 13	0.00	0.00	0.00	0.00	0.00	0.00	0.00	0.00	0.00	0.00	0.00	0.00	0.00	0.00	0.00	0.00	0.00	0.00
Sweden - Pseudomonas aeruginosa CRPA BSI - model 51	1.85	2.42	3.15	2.13	2.69	3.44	32.62	36.28	39.88	0.33	0.37	0.41	6.53	8.31	10.40	0.07	0.09	0.11
Sweden - Pseudomonas aeruginosa CRPA OTH - model 55	0.00	0.00	0.00	5.08E-04	6.94E-04	9.05E-04	14.85	18.48	22.12	0.15	0.19	0.23	0.00	0.00	0.00	0.00	0.00	0.00
Sweden - Pseudomonas aeruginosa CRPA RESP - model 52	1.94	2.55	3.26	2.59	3.27	4.05	115.44	134.59	154.66	1.18	1.38	1.59	6.94	8.80	11.11	0.07	0.09	0.11
Sweden - Pseudomonas aeruginosa CRPA SSI - model 54	0.09	0.12	0.15	0.09	0.12	0.15	19.93	25.04	29.97	0.20	0.26	0.31	0.47	0.61	0.76	4.81E-03	6.29E-03	7.82E-03
Sweden - Pseudomonas aeruginosa CRPA UTI - model 53	0.00	0.00	0.00	7.51E-04	9.97E-04	1.27E-03	38.57	47.12	55.11	0.40	0.48	0.57	0.00	0.00	0.00	0.00	0.00	0.00
Sweden - Pseudomonas aeruginosa ColRPA BSI - model 56	0.00	0.00	0.00	0.00	0.00	0.00	0.00	0.00	0.00	0.00	0.00	0.00	0.00	0.00	0.00	0.00	0.00	0.00
Sweden - Pseudomonas aeruginosa ColRPA OTH - model 60	0.00	0.00	0.00	0.00	0.00	0.00	0.00	0.00	0.00	0.00	0.00	0.00	0.00	0.00	0.00	0.00	0.00	0.00
Sweden - Pseudomonas aeruginosa ColRPA RESP - model 57	0.00	0.00	0.00	0.00	0.00	0.00	0.00	0.00	0.00	0.00	0.00	0.00	0.00	0.00	0.00	0.00	0.00	0.00
Sweden - Pseudomonas aeruginosa ColRPA SSI - model 59	0.00	0.00	0.00	0.00	0.00	0.00	0.00	0.00	0.00	0.00	0.00	0.00	0.00	0.00	0.00	0.00	0.00	0.00
Sweden - Pseudomonas aeruginosa ColRPA UTI - model 58	0.00	0.00	0.00	0.00	0.00	0.00	0.00	0.00	0.00	0.00	0.00	0.00	0.00	0.00	0.00	0.00	0.00	0.00
Sweden - Pseudomonas aeruginosa MDRPA BSI - model 46	0.02	0.04	0.07	0.02	0.05	0.08	2.14	2.77	3.65	0.02	0.03	0.04	0.27	0.58	0.96	2.75E-03	5.96E-03	9.86E-03
Sweden - Pseudomonas aeruginosa MDRPA OTH - model 50	0.00	0.00	0.00	2.23E-05	5.29E-05	9.79E-05	0.69	1.43	2.11	7.04E-03	0.01	0.02	0.00	0.00	0.00	0.00	0.00	0.00
Sweden - Pseudomonas aeruginosa MDRPA RESP - model 47	0.02	0.05	0.08	0.03	0.06	0.10	6.68	10.27	14.14	0.07	0.11	0.15	0.31	0.64	1.10	3.20E-03	6.56E-03	0.01
Sweden - Pseudomonas aeruginosa MDRPA SSI - model 49	2.46E-03	5.04E-03	7.82E-03	2.55E-03	5.09E-03	7.88E-03	0.94	1.90	2.95	9.65E-03	0.02	0.03	0.03	0.07	0.11	3.47E-04	7.03E-04	1.09E-03
Sweden - Pseudomonas aeruginosa MDRPA UTI - model 48	0.00	0.00	0.00	3.12E-05	7.18E-05	1.36E-04	1.98	3.63	5.16	0.02	0.04	0.05	0.00	0.00	0.00	0.00	0.00	0.00
Sweden - Staphylococcus aureus MRSA BSI - model 61	1.80	1.94	2.10	2.07	2.23	2.41	31.26	33.12	35.14	0.32	0.34	0.36	5.50	5.95	6.40	0.06	0.06	0.07
Sweden - Staphylococcus aureus MRSA OTH - model 65	0.00	0.00	0.00	1.27E-03	1.64E-03	2.01E-03	43.93	48.79	53.15	0.45	0.50	0.55	0.00	0.00	0.00	0.00	0.00	0.00
Sweden - Staphylococcus aureus MRSA RESP - model 62	0.52	0.59	0.66	0.79	0.89	0.99	44.66	49.81	54.96	0.46	0.51	0.56	1.59	1.79	2.00	0.02	0.02	0.02
Sweden - Staphylococcus aureus MRSA SSI - model 64	0.21	0.23	0.25	0.21	0.23	0.25	49.26	54.39	60.03	0.51	0.56	0.62	1.00	1.13	1.28	0.01	0.01	0.01
Sweden - Staphylococcus aureus MRSA UTI - model 63	0.00	0.00	0.00	1.17E-04	1.56E-04	2.04E-04	6.79	8.16	9.56	0.07	0.08	0.10	0.00	0.00	0.00	0.00	0.00	0.00
Sweden - Streptococcus pneumoniae PMRSP BSI - model 76	1.10	1.15	1.21	1.27	1.33	1.38	29.46	29.68	29.93	0.30	0.30	0.31	5.09	5.35	5.61	0.05	0.05	0.06
Sweden - Streptococcus pneumoniae PMRSP OTH - model 80	0.00	0.00	0.00	1.62E-04	2.12E-04	2.75E-04	9.71	10.61	11.67	0.10	0.11	0.12	0.00	0.00	0.00	0.00	0.00	0.00
Sweden - Streptococcus pneumoniae PMRSP RESP - model 77	0.75	0.88	1.04	1.14	1.33	1.57	98.47	113.01	132.47	1.01	1.16	1.36	3.50	4.07	4.84	0.04	0.04	0.05
Sweden - Streptococcus pneumoniae PMRSP SSI - model 79	0.00	0.00	0.00	0.00	0.00	0.00	0.00	0.00	0.00	0.00	0.00	0.00	0.00	0.00	0.00	0.00	0.00	0.00
Sweden - Streptococcus pneumoniae PMRSP UTI - model 78	0.00	0.00	0.00	0.00	0.00	0.00	0.00	0.00	0.00	0.00	0.00	0.00	0.00	0.00	0.00	0.00	0.00	0.00
Sweden - Streptococcus pneumoniae PRSP BSI - model 71	1.10	1.16	1.22	1.26	1.33	1.40	23.03	23.23	23.42	0.24	0.24	0.24	3.96	4.18	4.40	0.04	0.04	0.05
Sweden - Streptococcus pneumoniae PRSP OTH - model 75	0.00	0.00	0.00	1.21E-04	1.65E-04	2.22E-04	7.58	8.31	9.19	0.08	0.09	0.09	0.00	0.00	0.00	0.00	0.00	0.00
Sweden - Streptococcus pneumoniae PRSP RESP - model 72	0.75	0.88	1.07	1.14	1.34	1.61	76.65	89.16	107.27	0.79	0.91	1.10	2.72	3.20	3.90	0.03	0.03	0.04
Sweden - Streptococcus pneumoniae PRSP SSI - model 74	0.00	0.00	0.00	0.00	0.00	0.00	0.00	0.00	0.00	0.00	0.00	0.00	0.00	0.00	0.00	0.00	0.00	0.00
Sweden - Streptococcus pneumoniae PRSP UTI - model 73	0.00	0.00	0.00	0.00	0.00	0.00	0.00	0.00	0.00	0.00	0.00	0.00	0.00	0.00	0.00	0.00	0.00	0.00
**Total (Median & 95% UIs)**	32.95	37.73	43.00	39.61	44.95	50.88	4,029.07	4,570.54	5,119.78	41.34	46.89	52.52	144.70	166.99	190.94	1.48	1.71	1.96
**Total (average)**	**37.79**			**45.04**			**4,572.83**			**46.91**			**167.11**			**1.71**		

Infection	YLD			YLL			DALY			DALY per Case			YLD per 100,000			YLL per 100,000			DALY per 100,000			Cases per year			Incidence per 100,000			Deaths per year			Mortality per 100,000		
Colistin resistant *Acinetobacter spp.*	0.00	0.00	0.00	0.00	0.00	0.00	0.00	0.00	0.00	0.00	0.00	0.00	0.00	0.00	0.00	0.00	0.00	0.00	0.00	0.00	0.00	0.00	0.00	0.00	0.00	0.00	0.00	0.00	0.00	0.00	0.00	0.00	0.00
Carbapenem resistant *Acinetobacter spp.* (excluding those resistant to colistin)	9.67	11.53	13.64	43.42	61.20	79.75	54.13	72.69	92.33	4.63	5.98	7.38	0.47	0.56	0.66	2.10	2.97	3.87	2.62	3.52	4.48	30.91	35.93	41.25	1.50	1.74	2.00	2.38	3.14	3.97	0.12	0.15	0.19
Aminoglycosides and fluoroquinolones resistant *Acinetobacter* spp. (excluding those resistant to colistin and/or carbapenem)	1.00	1.62	2.37	3.51	8.53	15.00	4.69	10.18	17.15	3.10	5.25	7.50	0.05	0.08	0.12	0.17	0.41	0.73	0.23	0.49	0.83	3.53	5.95	8.51	0.17	0.29	0.41	0.21	0.51	0.90	0.01	0.02	0.04
Vancomycin resistant Enterococci (*E. faecalis* and *E. faecium*)	3.37	4.98	7.39	32.16	45.53	65.61	35.59	50.51	72.72	5.58	6.36	7.04	0.16	0.24	0.36	1.56	2.21	3.18	1.73	2.45	3.53	20.53	31.29	42.92	1.00	1.52	2.08	1.41	1.99	2.73	0.07	0.10	0.13
Colistin resistant *Escherichia coli*	0.00	0.00	0.00	0.00	0.00	0.00	0.00	0.00	0.00	0.00	0.00	0.00	0.00	0.00	0.00	0.00	0.00	0.00	0.00	0.00	0.00	0.00	0.00	0.00	0.00	0.00	0.00	0.00	0.00	0.00	0.00	0.00	0.00
Carbapenem resistant *Escherichia coli* (excluding those resistant to colistin)	0.00	0.00	0.00	0.00	0.00	0.00	0.00	0.00	0.00	0.00	0.00	0.00	0.00	0.00	0.00	0.00	0.00	0.00	0.00	0.00	0.00	0.00	0.00	0.00	0.00	0.00	0.00	0.00	0.00	0.00	0.00	0.00	0.00
Third-generation cephalosporin resistant *Escherichia coli* (excluding those resistant to colistin and/or carbapenem)	108.93	122.75	137.24	646.94	724.41	807.05	761.47	847.79	939.78	4.54	4.95	5.41	5.28	5.95	6.65	31.36	35.12	39.12	36.91	41.10	45.56	1,195.81	1,363.27	1,529.28	57.97	66.09	74.13	36.37	41.97	47.92	1.76	2.03	2.32
Colistin resistant *Klebsiella pneumoniae*	0.00	0.00	0.00	0.00	0.00	0.00	0.00	0.00	0.00	0.00	0.00	0.00	0.00	0.00	0.00	0.00	0.00	0.00	0.00	0.00	0.00	0.00	0.00	0.00	0.00	0.00	0.00	0.00	0.00	0.00	0.00	0.00	0.00
Carbapenem resistant *Klebsiella pneumoniae* (excluding those resistant to colistin)	1.22	1.82	2.53	11.81	19.36	28.88	13.36	21.20	31.27	5.00	7.47	10.21	0.06	0.09	0.12	0.57	0.94	1.40	0.65	1.03	1.52	5.33	8.86	12.51	0.26	0.43	0.61	0.71	1.19	1.81	0.03	0.06	0.09
Third-generation cephalosporin resistant *Klebsiella pneumoniae* (excluding those resistant to colistin and/or carbapenem)	50.32	55.97	62.42	248.23	268.93	289.58	300.40	324.77	349.55	6.34	6.68	7.07	2.44	2.71	3.03	12.03	13.04	14.04	14.56	15.74	16.94	180.10	199.74	219.38	8.73	9.68	10.63	9.97	10.84	11.74	0.48	0.53	0.57
Colistin resistant *Pseudomonas aeruginosa*	0.00	0.00	0.00	0.00	0.00	0.00	0.00	0.00	0.00	0.00	0.00	0.00	0.00	0.00	0.00	0.00	0.00	0.00	0.00	0.00	0.00	0.00	0.00	0.00	0.00	0.00	0.00	0.00	0.00	0.00	0.00	0.00	0.00
Carbapenem resistant *Pseudomonas aeruginosa* (excluding those resistant to colistin)	37.65	44.96	53.03	167.44	225.97	295.16	207.90	271.33	345.37	5.50	6.98	8.56	1.83	2.18	2.57	8.12	10.95	14.31	10.08	13.15	16.74	144.46	172.98	201.83	7.00	8.39	9.78	8.93	11.81	15.16	0.43	0.57	0.73
Pseudomonas aeruginosa resistant to three or more antimicrobial groups (excluding those resistant to colistin and/or carbapenem)	7.35	10.70	14.72	25.14	50.89	81.68	33.94	61.56	94.61	6.63	11.42	16.37	0.36	0.52	0.71	1.22	2.47	3.96	1.65	2.98	4.59	16.85	23.67	30.62	0.82	1.15	1.48	0.83	1.50	2.28	0.04	0.07	0.11
Methicillin resistant *Staphylococcus aureus*	46.97	53.57	61.64	235.33	260.09	286.23	285.41	314.16	344.31	5.28	5.60	5.92	2.28	2.60	2.99	11.41	12.61	13.88	13.84	15.23	16.69	259.18	287.92	317.52	12.56	13.96	15.39	12.42	13.62	14.92	0.60	0.66	0.72
Penicillin and macrolides resistant *Streptococcus pneumoniae* (excluding those only resistant to penicillin)	31.32	36.08	42.62	107.43	118.55	132.42	140.46	154.77	173.48	5.65	6.05	6.47	1.52	1.75	2.07	5.21	5.75	6.42	6.81	7.50	8.41	75.48	83.32	94.47	3.66	4.04	4.58	4.69	5.11	5.65	0.23	0.25	0.27
Penicillin tesistant *Streptococcus pneumoniae* (excluding those resistant to macrolides)	54.24	66.43	86.71	194.55	220.47	259.65	252.54	287.27	340.11	12.86	13.94	14.99	2.63	3.22	4.20	9.43	10.69	12.59	12.24	13.93	16.49	59.68	67.34	78.70	2.89	3.26	3.81	3.73	4.14	4.68	0.18	0.20	0.23
Total	352.04	410.41	484.30	1,715.97	2,003.93	2,341.00	2,089.87	2,416.21	2,800.68				17.07	19.89	23.48	83.18	97.14	113.48	101.31	117.13	135.77	1,991.86	2,280.27	2,576.98	96.56	110.54	124.92	81.64	95.83	111.76	3.96	4.65	5.42

Model	YLD			YLL			DALY			DALY per Case			YLD per 100,000			YLL per 100,000			DALY per 100,000			Cases per year			Incidence per 100,000			Deaths per year			Mortality per 100,000		
Slovenia - Acinetobacter spp. CRACI BSI - model 36	5.65	6.58	7.66	34.45	50.14	66.49	40.92	56.67	73.29	3.33	4.56	5.82	0.27	0.32	0.37	1.67	2.43	3.22	1.98	2.75	3.55	11.50	12.47	13.50	0.56	0.60	0.65	1.89	2.55	3.28	0.09	0.12	0.16
Slovenia - Acinetobacter spp. CRACI OTH - model 40	1.03E-03	1.68E-03	2.49E-03	0.00	0.00	0.00	1.03E-03	1.68E-03	2.49E-03	2.77E-03	3.73E-03	4.84E-03	4.99E-05	8.16E-05	1.21E-04	0.00	0.00	0.00	4.99E-05	8.16E-05	1.21E-04	0.31	0.46	0.61	0.02	0.02	0.03	0.00	0.00	0.00	0.00	0.00	0.00
Slovenia - Acinetobacter spp. CRACI RESP - model 37	4.01	4.92	5.94	7.78	9.54	11.41	12.00	14.47	17.17	0.99	1.08	1.17	0.19	0.24	0.29	0.38	0.46	0.55	0.58	0.70	0.83	11.46	13.41	15.44	0.56	0.65	0.75	0.41	0.48	0.56	0.02	0.02	0.03
Slovenia - Acinetobacter spp. CRACI SSI - model 39	6.18E-03	9.70E-03	0.01	1.19	1.53	1.85	1.20	1.54	1.86	0.31	0.34	0.38	3.00E-04	4.70E-04	6.79E-04	0.06	0.07	0.09	0.06	0.07	0.09	3.61	4.54	5.49	0.17	0.22	0.27	0.08	0.10	0.12	3.92E-03	4.96E-03	5.99E-03
Slovenia - Acinetobacter spp. CRACI UTI - model 38	7.24E-03	0.01	0.01	0.00	0.00	0.00	7.24E-03	0.01	0.01	1.55E-03	2.02E-03	2.60E-03	3.51E-04	4.96E-04	6.86E-04	0.00	0.00	0.00	3.51E-04	4.96E-04	6.86E-04	4.03	5.05	6.20	0.20	0.24	0.30	0.00	0.00	0.00	0.00	0.00	0.00
Slovenia - Acinetobacter spp. ColRACI BSI - model 41	0.00	0.00	0.00	0.00	0.00	0.00	0.00	0.00	0.00	NaN	NaN	NaN	0.00	0.00	0.00	0.00	0.00	0.00	0.00	0.00	0.00	0.00	0.00	0.00	0.00	0.00	0.00	0.00	0.00	0.00	0.00	0.00	0.00
Slovenia - Acinetobacter spp. ColRACI OTH - model 45	0.00	0.00	0.00	0.00	0.00	0.00	0.00	0.00	0.00	NaN	NaN	NaN	0.00	0.00	0.00	0.00	0.00	0.00	0.00	0.00	0.00	0.00	0.00	0.00	0.00	0.00	0.00	0.00	0.00	0.00	0.00	0.00	0.00
Slovenia - Acinetobacter spp. ColRACI RESP - model 42	0.00	0.00	0.00	0.00	0.00	0.00	0.00	0.00	0.00	NaN	NaN	NaN	0.00	0.00	0.00	0.00	0.00	0.00	0.00	0.00	0.00	0.00	0.00	0.00	0.00	0.00	0.00	0.00	0.00	0.00	0.00	0.00	0.00
Slovenia - Acinetobacter spp. ColRACI SSI - model 44	0.00	0.00	0.00	0.00	0.00	0.00	0.00	0.00	0.00	NaN	NaN	NaN	0.00	0.00	0.00	0.00	0.00	0.00	0.00	0.00	0.00	0.00	0.00	0.00	0.00	0.00	0.00	0.00	0.00	0.00	0.00	0.00	0.00
Slovenia - Acinetobacter spp. ColRACI UTI - model 43	0.00	0.00	0.00	0.00	0.00	0.00	0.00	0.00	0.00	NaN	NaN	NaN	0.00	0.00	0.00	0.00	0.00	0.00	0.00	0.00	0.00	0.00	0.00	0.00	0.00	0.00	0.00	0.00	0.00	0.00	0.00	0.00	0.00
Slovenia - Acinetobacter spp. MDRACI BSI - model 31	0.61	0.91	1.32	2.58	6.74	12.28	3.35	7.66	13.42	1.72	3.73	5.84	0.03	0.04	0.06	0.13	0.33	0.60	0.16	0.37	0.65	1.62	2.03	2.57	0.08	0.10	0.12	0.15	0.40	0.73	7.47E-03	0.02	0.04
Slovenia - Acinetobacter spp. MDRACI OTH - model 35	4.22E-05	2.44E-04	7.77E-04	0.00	0.00	0.00	4.22E-05	2.44E-04	7.77E-04	1.59E-03	3.75E-03	6.37E-03	2.05E-06	1.18E-05	3.77E-05	0.00	0.00	0.00	2.05E-06	1.18E-05	3.77E-05	0.01	0.07	0.16	6.72E-04	3.53E-03	7.58E-03	0.00	0.00	0.00	0.00	0.00	0.00
Slovenia - Acinetobacter spp. MDRACI RESP - model 32	0.39	0.70	1.05	0.75	1.34	2.00	1.16	2.05	2.99	0.78	0.92	1.04	0.02	0.03	0.05	0.04	0.06	0.10	0.06	0.10	0.14	1.26	2.25	3.19	0.06	0.11	0.15	0.04	0.08	0.12	2.17E-03	3.87E-03	5.77E-03
Slovenia - Acinetobacter spp. MDRACI SSI - model 34	3.04E-04	1.37E-03	4.32E-03	0.18	0.46	0.72	0.18	0.46	0.73	0.60	0.61	0.61	1.47E-05	6.64E-05	2.10E-04	8.75E-03	0.02	0.04	8.72E-03	0.02	0.04	0.30	0.76	1.21	0.01	0.04	0.06	0.01	0.03	0.04	5.22E-04	1.32E-03	2.11E-03
Slovenia - Acinetobacter spp. MDRACI UTI - model 33	4.72E-04	1.56E-03	3.73E-03	0.00	0.00	0.00	4.72E-04	1.56E-03	3.73E-03	8.03E-04	2.03E-03	3.55E-03	2.29E-05	7.56E-05	1.81E-04	0.00	0.00	0.00	2.29E-05	7.56E-05	1.81E-04	0.33	0.83	1.39	0.02	0.04	0.07	0.00	0.00	0.00	0.00	0.00	0.00
Slovenia - Enterococcus faecalis and E. faecium VRE BSI - model 66	3.35	4.94	7.32	29.96	42.09	61.01	33.36	47.02	68.06	5.28	6.04	6.70	0.16	0.24	0.35	1.45	2.04	2.96	1.62	2.28	3.30	5.59	7.83	10.73	0.27	0.38	0.52	1.29	1.79	2.46	0.06	0.09	0.12
Slovenia - Enterococcus faecalis and E. faecium VRE OTH - model 70	7.16E-03	0.01	0.02	0.00	0.00	0.00	7.16E-03	0.01	0.02	1.04E-03	1.62E-03	2.43E-03	3.47E-04	6.62E-04	1.08E-03	0.00	0.00	0.00	3.47E-04	6.62E-04	1.08E-03	5.41	8.45	11.42	0.26	0.41	0.55	0.00	0.00	0.00	0.00	0.00	0.00
Slovenia - Enterococcus faecalis and E. faecium VRE RESP - model 67	0.00	0.00	0.00	0.00	0.00	0.00	0.00	0.00	0.00	NaN	NaN	NaN	0.00	0.00	0.00	0.00	0.00	0.00	0.00	0.00	0.00	0.00	0.00	0.00	0.00	0.00	0.00	0.00	0.00	0.00	0.00	0.00	0.00
Slovenia - Enterococcus faecalis and E. faecium VRE SSI - model 69	0.01	0.02	0.04	2.20	3.44	4.60	2.22	3.46	4.62	0.29	0.32	0.34	5.58E-04	1.09E-03	1.92E-03	0.11	0.17	0.22	0.11	0.17	0.22	7.03	10.99	15.15	0.34	0.53	0.73	0.12	0.20	0.27	5.71E-03	9.55E-03	0.01
Slovenia - Enterococcus faecalis and E. faecium VRE UTI - model 68	3.93E-03	7.43E-03	0.01	0.00	0.00	0.00	3.93E-03	7.43E-03	0.01	1.22E-03	1.84E-03	2.67E-03	1.91E-04	3.60E-04	6.01E-04	0.00	0.00	0.00	1.91E-04	3.60E-04	6.01E-04	2.50	4.02	5.61	0.12	0.19	0.27	0.00	0.00	0.00	0.00	0.00	0.00
Slovenia - Escherichia coli 3GCREC BSI - model 16	83.72	92.35	101.46	534.28	593.64	656.89	623.39	686.52	754.10	3.29	3.59	3.90	4.06	4.48	4.92	25.90	28.78	31.84	30.22	33.28	36.56	177.66	191.34	206.63	8.61	9.28	10.02	29.09	33.18	37.67	1.41	1.61	1.83
Slovenia - Escherichia coli 3GCREC OTH - model 20	0.15	0.21	0.28	0.00	0.00	0.00	0.15	0.21	0.28	2.64E-03	3.35E-03	4.13E-03	7.40E-03	0.01	0.01	0.00	0.00	0.00	7.40E-03	0.01	0.01	50.85	63.06	75.83	2.47	3.06	3.68	0.00	0.00	0.00	0.00	0.00	0.00
Slovenia - Escherichia coli 3GCREC RESP - model 17	23.48	28.14	32.93	46.34	54.14	62.94	69.96	82.40	95.72	0.88	0.98	1.10	1.14	1.36	1.60	2.25	2.62	3.05	3.39	3.99	4.64	69.37	84.27	99.15	3.36	4.09	4.81	2.46	3.04	3.60	0.12	0.15	0.17
Slovenia - Escherichia coli 3GCREC SSI - model 19	0.31	0.43	0.58	66.32	76.63	87.22	66.70	77.04	87.68	0.36	0.38	0.40	0.01	0.02	0.03	3.21	3.71	4.23	3.23	3.73	4.25	175.03	201.96	228.95	8.48	9.79	11.10	4.83	5.76	6.66	0.23	0.28	0.32
Slovenia - Escherichia coli 3GCREC UTI - model 18	1.26	1.62	2.00	0.00	0.00	0.00	1.26	1.62	2.00	1.60E-03	1.96E-03	2.38E-03	0.06	0.08	0.10	0.00	0.00	0.00	0.06	0.08	0.10	722.89	822.64	918.72	35.04	39.88	44.54	0.00	0.00	0.00	0.00	0.00	0.00
Slovenia - Escherichia coli CREC BSI - model 21	0.00	0.00	0.00	0.00	0.00	0.00	0.00	0.00	0.00	NaN	NaN	NaN	0.00	0.00	0.00	0.00	0.00	0.00	0.00	0.00	0.00	0.00	0.00	0.00	0.00	0.00	0.00	0.00	0.00	0.00	0.00	0.00	0.00
Slovenia - Escherichia coli CREC OTH - model 25	0.00	0.00	0.00	0.00	0.00	0.00	0.00	0.00	0.00	NaN	NaN	NaN	0.00	0.00	0.00	0.00	0.00	0.00	0.00	0.00	0.00	0.00	0.00	0.00	0.00	0.00	0.00	0.00	0.00	0.00	0.00	0.00	0.00
Slovenia - Escherichia coli CREC RESP - model 22	0.00	0.00	0.00	0.00	0.00	0.00	0.00	0.00	0.00	NaN	NaN	NaN	0.00	0.00	0.00	0.00	0.00	0.00	0.00	0.00	0.00	0.00	0.00	0.00	0.00	0.00	0.00	0.00	0.00	0.00	0.00	0.00	0.00
Slovenia - Escherichia coli CREC SSI - model 24	0.00	0.00	0.00	0.00	0.00	0.00	0.00	0.00	0.00	NaN	NaN	NaN	0.00	0.00	0.00	0.00	0.00	0.00	0.00	0.00	0.00	0.00	0.00	0.00	0.00	0.00	0.00	0.00	0.00	0.00	0.00	0.00	0.00
Slovenia - Escherichia coli CREC UTI - model 23	0.00	0.00	0.00	0.00	0.00	0.00	0.00	0.00	0.00	NaN	NaN	NaN	0.00	0.00	0.00	0.00	0.00	0.00	0.00	0.00	0.00	0.00	0.00	0.00	0.00	0.00	0.00	0.00	0.00	0.00	0.00	0.00	0.00
Slovenia - Escherichia coli ColREC BSI - model 26	0.00	0.00	0.00	0.00	0.00	0.00	0.00	0.00	0.00	NaN	NaN	NaN	0.00	0.00	0.00	0.00	0.00	0.00	0.00	0.00	0.00	0.00	0.00	0.00	0.00	0.00	0.00	0.00	0.00	0.00	0.00	0.00	0.00
Slovenia - Escherichia coli ColREC OTH - model 30	0.00	0.00	0.00	0.00	0.00	0.00	0.00	0.00	0.00	NaN	NaN	NaN	0.00	0.00	0.00	0.00	0.00	0.00	0.00	0.00	0.00	0.00	0.00	0.00	0.00	0.00	0.00	0.00	0.00	0.00	0.00	0.00	0.00
Slovenia - Escherichia coli ColREC RESP - model 27	0.00	0.00	0.00	0.00	0.00	0.00	0.00	0.00	0.00	NaN	NaN	NaN	0.00	0.00	0.00	0.00	0.00	0.00	0.00	0.00	0.00	0.00	0.00	0.00	0.00	0.00	0.00	0.00	0.00	0.00	0.00	0.00	0.00
Slovenia - Escherichia coli ColREC SSI - model 29	0.00	0.00	0.00	0.00	0.00	0.00	0.00	0.00	0.00	NaN	NaN	NaN	0.00	0.00	0.00	0.00	0.00	0.00	0.00	0.00	0.00	0.00	0.00	0.00	0.00	0.00	0.00	0.00	0.00	0.00	0.00	0.00	0.00
Slovenia - Escherichia coli ColREC UTI - model 28	0.00	0.00	0.00	0.00	0.00	0.00	0.00	0.00	0.00	NaN	NaN	NaN	0.00	0.00	0.00	0.00	0.00	0.00	0.00	0.00	0.00	0.00	0.00	0.00	0.00	0.00	0.00	0.00	0.00	0.00	0.00	0.00	0.00
Slovenia - Klebsiella pneumoniae 3GCRKP BSI - model 1	31.68	34.75	38.56	204.63	218.98	233.29	237.97	253.67	269.65	4.64	4.86	5.11	1.54	1.68	1.87	9.92	10.62	11.31	11.54	12.30	13.07	49.46	52.11	55.08	2.40	2.53	2.67	8.11	8.70	9.31	0.39	0.42	0.45
Slovenia - Klebsiella pneumoniae 3GCRKP OTH - model 5	0.05	0.07	0.09	0.00	0.00	0.00	0.05	0.07	0.09	2.67E-03	3.34E-03	4.04E-03	2.57E-03	3.35E-03	4.31E-03	0.00	0.00	0.00	2.57E-03	3.35E-03	4.31E-03	18.03	20.77	23.50	0.87	1.01	1.14	0.00	0.00	0.00	0.00	0.00	0.00
Slovenia - Klebsiella pneumoniae 3GCRKP RESP - model 2	18.46	20.98	23.56	35.82	40.78	45.76	54.51	61.69	69.08	1.27	1.37	1.48	0.89	1.02	1.14	1.74	1.98	2.22	2.64	2.99	3.35	40.11	44.94	49.62	1.94	2.18	2.41	1.43	1.61	1.83	0.07	0.08	0.09
Slovenia - Klebsiella pneumoniae 3GCRKP SSI - model 4	0.03	0.04	0.06	7.78	9.17	10.53	7.77	9.22	10.58	0.43	0.45	0.47	1.52E-03	2.10E-03	2.86E-03	0.38	0.44	0.51	0.38	0.45	0.51	17.79	20.55	23.31	0.86	1.00	1.13	0.43	0.52	0.61	0.02	0.03	0.03
Slovenia - Klebsiella pneumoniae 3GCRKP UTI - model 3	0.10	0.12	0.15	0.00	0.00	0.00	0.10	0.12	0.15	1.58E-03	1.95E-03	2.36E-03	4.62E-03	5.78E-03	7.20E-03	0.00	0.00	0.00	4.62E-03	5.78E-03	7.20E-03	54.71	61.37	67.87	2.65	2.97	3.29	0.00	0.00	0.00	0.00	0.00	0.00
Slovenia - Klebsiella pneumoniae CRKP BSI - model 6	1.03	1.40	1.88	11.32	18.29	27.23	12.67	19.70	29.00	4.08	6.27	8.63	0.05	0.07	0.09	0.55	0.89	1.32	0.61	0.96	1.41	2.53	3.15	3.95	0.12	0.15	0.19	0.68	1.12	1.69	0.03	0.05	0.08
Slovenia - Klebsiella pneumoniae CRKP OTH - model 10	1.79E-03	4.86E-03	0.01	0.00	0.00	0.00	1.79E-03	4.86E-03	0.01	1.60E-03	3.47E-03	6.57E-03	8.68E-05	2.36E-04	5.64E-04	0.00	0.00	0.00	8.68E-05	2.36E-04	5.64E-04	0.71	1.48	2.27	0.03	0.07	0.11	0.00	0.00	0.00	0.00	0.00	0.00
Slovenia - Klebsiella pneumoniae CRKP RESP - model 7	0.19	0.41	0.63	0.37	0.78	1.19	0.56	1.19	1.79	0.62	0.88	1.25	9.03E-03	0.02	0.03	0.02	0.04	0.06	0.03	0.06	0.09	0.66	1.36	2.02	0.03	0.07	0.10	0.02	0.05	0.07	1.12E-03	2.34E-03	3.58E-03
Slovenia - Klebsiella pneumoniae CRKP SSI - model 9	6.47E-04	1.83E-03	4.57E-03	0.13	0.30	0.46	0.13	0.30	0.46	0.29	0.32	0.33	3.14E-05	8.85E-05	2.21E-04	6.23E-03	0.01	0.02	6.31E-03	0.01	0.02	0.46	0.94	1.46	0.02	0.05	0.07	9.04E-03	0.03	0.04	4.38E-04	1.23E-03	2.07E-03
Slovenia - Klebsiella pneumoniae CRKP UTI - model 8	1.53E-03	3.81E-03	7.99E-03	0.00	0.00	0.00	1.53E-03	3.81E-03	7.99E-03	9.54E-04	2.09E-03	3.56E-03	7.40E-05	1.84E-04	3.87E-04	0.00	0.00	0.00	7.40E-05	1.84E-04	3.87E-04	0.98	1.92	2.80	0.05	0.09	0.14	0.00	0.00	0.00	0.00	0.00	0.00
Slovenia - Klebsiella pneumoniae ColRKP BSI - model 11	0.00	0.00	0.00	0.00	0.00	0.00	0.00	0.00	0.00	NaN	NaN	NaN	0.00	0.00	0.00	0.00	0.00	0.00	0.00	0.00	0.00	0.00	0.00	0.00	0.00	0.00	0.00	0.00	0.00	0.00	0.00	0.00	0.00
Slovenia - Klebsiella pneumoniae ColRKP OTH - model 15	0.00	0.00	0.00	0.00	0.00	0.00	0.00	0.00	0.00	NaN	NaN	NaN	0.00	0.00	0.00	0.00	0.00	0.00	0.00	0.00	0.00	0.00	0.00	0.00	0.00	0.00	0.00	0.00	0.00	0.00	0.00	0.00	0.00
Slovenia - Klebsiella pneumoniae ColRKP RESP - model 12	0.00	0.00	0.00	0.00	0.00	0.00	0.00	0.00	0.00	NaN	NaN	NaN	0.00	0.00	0.00	0.00	0.00	0.00	0.00	0.00	0.00	0.00	0.00	0.00	0.00	0.00	0.00	0.00	0.00	0.00	0.00	0.00	0.00
Slovenia - Klebsiella pneumoniae ColRKP SSI - model 14	0.00	0.00	0.00	0.00	0.00	0.00	0.00	0.00	0.00	NaN	NaN	NaN	0.00	0.00	0.00	0.00	0.00	0.00	0.00	0.00	0.00	0.00	0.00	0.00	0.00	0.00	0.00	0.00	0.00	0.00	0.00	0.00	0.00
Slovenia - Klebsiella pneumoniae ColRKP UTI - model 13	0.00	0.00	0.00	0.00	0.00	0.00	0.00	0.00	0.00	NaN	NaN	NaN	0.00	0.00	0.00	0.00	0.00	0.00	0.00	0.00	0.00	0.00	0.00	0.00	0.00	0.00	0.00	0.00	0.00	0.00	0.00	0.00	0.00
Slovenia - Pseudomonas aeruginosa CRPA BSI - model 51	10.90	12.58	14.72	76.48	106.51	137.25	88.43	119.20	150.95	3.79	4.93	6.14	0.53	0.61	0.71	3.71	5.16	6.65	4.29	5.78	7.32	21.75	24.10	26.66	1.05	1.17	1.29	4.11	5.50	7.02	0.20	0.27	0.34
Slovenia - Pseudomonas aeruginosa CRPA OTH - model 55	0.03	0.04	0.06	0.00	0.00	0.00	0.03	0.04	0.06	2.82E-03	3.67E-03	4.58E-03	1.50E-03	2.17E-03	2.97E-03	0.00	0.00	0.00	1.50E-03	2.17E-03	2.97E-03	9.21	12.27	14.94	0.45	0.60	0.72	0.00	0.00	0.00	0.00	0.00	0.00
Slovenia - Pseudomonas aeruginosa CRPA RESP - model 52	26.65	32.24	38.10	86.00	112.70	149.55	114.41	145.21	185.86	1.34	1.64	1.97	1.29	1.56	1.85	4.17	5.46	7.25	5.55	7.04	9.01	75.46	88.97	103.26	3.66	4.31	5.01	4.48	5.85	7.58	0.22	0.28	0.37
Slovenia - Pseudomonas aeruginosa CRPA SSI - model 54	0.02	0.04	0.05	4.95	6.77	8.36	4.98	6.80	8.41	0.37	0.41	0.44	1.12E-03	1.71E-03	2.47E-03	0.24	0.33	0.41	0.24	0.33	0.41	12.94	16.60	20.17	0.63	0.80	0.98	0.34	0.46	0.57	0.02	0.02	0.03
Slovenia - Pseudomonas aeruginosa CRPA UTI - model 53	0.05	0.06	0.09	0.00	0.00	0.00	0.05	0.06	0.09	1.52E-03	2.04E-03	2.54E-03	2.21E-03	3.06E-03	4.22E-03	0.00	0.00	0.00	2.21E-03	3.06E-03	4.22E-03	25.11	31.03	36.81	1.22	1.50	1.78	0.00	0.00	0.00	0.00	0.00	0.00
Slovenia - Pseudomonas aeruginosa ColRPA BSI - model 56	0.00	0.00	0.00	0.00	0.00	0.00	0.00	0.00	0.00	NaN	NaN	NaN	0.00	0.00	0.00	0.00	0.00	0.00	0.00	0.00	0.00	0.00	0.00	0.00	0.00	0.00	0.00	0.00	0.00	0.00	0.00	0.00	0.00
Slovenia - Pseudomonas aeruginosa ColRPA OTH - model 60	0.00	0.00	0.00	0.00	0.00	0.00	0.00	0.00	0.00	NaN	NaN	NaN	0.00	0.00	0.00	0.00	0.00	0.00	0.00	0.00	0.00	0.00	0.00	0.00	0.00	0.00	0.00	0.00	0.00	0.00	0.00	0.00	0.00
Slovenia - Pseudomonas aeruginosa ColRPA RESP - model 57	0.00	0.00	0.00	0.00	0.00	0.00	0.00	0.00	0.00	NaN	NaN	NaN	0.00	0.00	0.00	0.00	0.00	0.00	0.00	0.00	0.00	0.00	0.00	0.00	0.00	0.00	0.00	0.00	0.00	0.00	0.00	0.00	0.00
Slovenia - Pseudomonas aeruginosa ColRPA SSI - model 59	0.00	0.00	0.00	0.00	0.00	0.00	0.00	0.00	0.00	NaN	NaN	NaN	0.00	0.00	0.00	0.00	0.00	0.00	0.00	0.00	0.00	0.00	0.00	0.00	0.00	0.00	0.00	0.00	0.00	0.00	0.00	0.00	0.00
Slovenia - Pseudomonas aeruginosa ColRPA UTI - model 58	0.00	0.00	0.00	0.00	0.00	0.00	0.00	0.00	0.00	NaN	NaN	NaN	0.00	0.00	0.00	0.00	0.00	0.00	0.00	0.00	0.00	0.00	0.00	0.00	0.00	0.00	0.00	0.00	0.00	0.00	0.00	0.00	0.00
Slovenia - Pseudomonas aeruginosa MDRPA BSI - model 46	2.23	2.99	4.15	11.53	24.36	38.85	14.53	27.34	42.44	4.56	8.28	12.03	0.11	0.14	0.20	0.56	1.18	1.88	0.70	1.33	2.06	2.69	3.29	3.95	0.13	0.16	0.19	0.38	0.70	1.06	0.02	0.03	0.05
Slovenia - Pseudomonas aeruginosa MDRPA OTH - model 50	3.03E-03	6.02E-03	0.01	0.00	0.00	0.00	3.03E-03	6.02E-03	0.01	2.13E-03	3.74E-03	5.39E-03	1.47E-04	2.92E-04	5.32E-04	0.00	0.00	0.00	1.47E-04	2.92E-04	5.32E-04	1.03	1.65	2.40	0.05	0.08	0.12	0.00	0.00	0.00	0.00	0.00	0.00
Slovenia - Pseudomonas aeruginosa MDRPA RESP - model 47	5.12	7.69	10.54	13.14	25.73	41.70	18.93	33.40	51.00	1.77	2.78	3.91	0.25	0.37	0.51	0.64	1.25	2.02	0.92	1.62	2.47	8.95	12.23	15.44	0.43	0.59	0.75	0.43	0.76	1.15	0.02	0.04	0.06
Slovenia - Pseudomonas aeruginosa MDRPA SSI - model 49	2.02E-03	4.71E-03	8.66E-03	0.47	0.80	1.14	0.48	0.81	1.14	0.30	0.36	0.42	9.81E-05	2.28E-04	4.20E-04	0.02	0.04	0.06	0.02	0.04	0.06	1.40	2.26	3.13	0.07	0.11	0.15	0.02	0.04	0.06	1.06E-03	2.02E-03	2.88E-03
Slovenia - Pseudomonas aeruginosa MDRPA UTI - model 48	4.48E-03	8.45E-03	0.01	0.00	0.00	0.00	4.48E-03	8.45E-03	0.01	1.22E-03	2.02E-03	2.95E-03	2.17E-04	4.09E-04	6.74E-04	0.00	0.00	0.00	2.17E-04	4.09E-04	6.74E-04	2.79	4.24	5.70	0.14	0.21	0.28	0.00	0.00	0.00	0.00	0.00	0.00
Slovenia - Staphylococcus aureus MRSA BSI - model 61	23.27	25.96	29.29	160.15	174.10	188.36	185.00	200.28	215.48	3.87	4.09	4.31	1.13	1.26	1.42	7.76	8.44	9.13	8.97	9.71	10.45	46.35	49.03	51.95	2.25	2.38	2.52	8.16	8.80	9.50	0.40	0.43	0.46
Slovenia - Staphylococcus aureus MRSA OTH - model 65	0.18	0.24	0.31	0.00	0.00	0.00	0.18	0.24	0.31	2.56E-03	3.27E-03	4.09E-03	8.83E-03	0.01	0.01	0.00	0.00	0.00	8.83E-03	0.01	0.01	65.06	72.24	79.95	3.15	3.50	3.88	0.00	0.00	0.00	0.00	0.00	0.00
Slovenia - Staphylococcus aureus MRSA RESP - model 62	23.39	27.18	31.79	45.39	52.54	60.70	70.23	80.01	91.16	1.01	1.08	1.16	1.13	1.32	1.54	2.20	2.55	2.94	3.40	3.88	4.42	65.57	73.99	82.37	3.18	3.59	3.99	2.34	2.66	3.02	0.11	0.13	0.15
Slovenia - Staphylococcus aureus MRSA SSI - model 64	0.12	0.17	0.23	29.79	33.44	37.16	29.98	33.61	37.33	0.40	0.42	0.44	5.97E-03	8.29E-03	0.01	1.44	1.62	1.80	1.45	1.63	1.81	72.50	80.70	88.77	3.51	3.91	4.30	1.92	2.16	2.40	0.09	0.10	0.12
Slovenia - Staphylococcus aureus MRSA UTI - model 63	0.02	0.02	0.03	0.00	0.00	0.00	0.02	0.02	0.03	1.44E-03	1.87E-03	2.34E-03	7.77E-04	1.09E-03	1.46E-03	0.00	0.00	0.00	7.77E-04	1.09E-03	1.46E-03	9.70	11.95	14.48	0.47	0.58	0.70	0.00	0.00	0.00	0.00	0.00	0.00
Slovenia - Streptococcus pneumoniae PMRSP BSI - model 76	8.92	9.90	11.01	63.82	67.43	71.03	73.52	77.36	80.97	4.55	4.79	5.01	0.43	0.48	0.53	3.09	3.27	3.44	3.56	3.75	3.93	16.16	16.16	16.16	0.78	0.78	0.78	2.77	2.91	3.05	0.13	0.14	0.15
Slovenia - Streptococcus pneumoniae PMRSP OTH - model 80	8.40E-03	0.01	0.01	0.00	0.00	0.00	8.40E-03	0.01	0.01	1.48E-03	1.96E-03	2.51E-03	4.07E-04	5.40E-04	7.00E-04	0.00	0.00	0.00	4.07E-04	5.40E-04	7.00E-04	5.32	5.71	6.21	0.26	0.28	0.30	0.00	0.00	0.00	0.00	0.00	0.00
Slovenia - Streptococcus pneumoniae PMRSP RESP - model 77	22.39	26.17	31.59	43.61	51.12	61.40	66.93	77.40	92.50	1.10	1.26	1.46	1.09	1.27	1.53	2.11	2.48	2.98	3.24	3.75	4.48	54.00	61.45	72.10	2.62	2.98	3.50	1.92	2.20	2.61	0.09	0.11	0.13
Slovenia - Streptococcus pneumoniae PMRSP SSI - model 79	0.00	0.00	0.00	0.00	0.00	0.00	0.00	0.00	0.00	NaN	NaN	NaN	0.00	0.00	0.00	0.00	0.00	0.00	0.00	0.00	0.00	0.00	0.00	0.00	0.00	0.00	0.00	0.00	0.00	0.00	0.00	0.00	0.00
Slovenia - Streptococcus pneumoniae PMRSP UTI - model 78	0.00	0.00	0.00	0.00	0.00	0.00	0.00	0.00	0.00	NaN	NaN	NaN	0.00	0.00	0.00	0.00	0.00	0.00	0.00	0.00	0.00	0.00	0.00	0.00	0.00	0.00	0.00	0.00	0.00	0.00	0.00	0.00	0.00
Slovenia - Streptococcus pneumoniae PRSP BSI - model 71	15.86	18.42	21.66	117.64	126.63	135.12	135.32	145.12	153.94	10.31	11.05	11.72	0.77	0.89	1.05	5.70	6.14	6.55	6.56	7.03	7.46	13.13	13.13	13.13	0.64	0.64	0.64	2.23	2.36	2.50	0.11	0.11	0.12
Slovenia - Streptococcus pneumoniae PRSP OTH - model 75	6.52E-03	9.09E-03	0.01	0.00	0.00	0.00	6.52E-03	9.09E-03	0.01	1.43E-03	1.95E-03	2.63E-03	3.16E-04	4.40E-04	6.13E-04	0.00	0.00	0.00	3.16E-04	4.40E-04	6.13E-04	4.24	4.66	5.20	0.21	0.23	0.25	0.00	0.00	0.00	0.00	0.00	0.00
Slovenia - Streptococcus pneumoniae PRSP RESP - model 72	38.37	48.00	65.03	76.90	93.83	124.52	117.22	142.14	186.16	2.55	2.89	3.27	1.86	2.33	3.15	3.73	4.55	6.04	5.68	6.89	9.02	42.31	49.55	60.37	2.05	2.40	2.93	1.50	1.78	2.18	0.07	0.09	0.11
Slovenia - Streptococcus pneumoniae PRSP SSI - model 74	0.00	0.00	0.00	0.00	0.00	0.00	0.00	0.00	0.00	NaN	NaN	NaN	0.00	0.00	0.00	0.00	0.00	0.00	0.00	0.00	0.00	0.00	0.00	0.00	0.00	0.00	0.00	0.00	0.00	0.00	0.00	0.00	0.00
Slovenia - Streptococcus pneumoniae PRSP UTI - model 73	0.00	0.00	0.00	0.00	0.00	0.00	0.00	0.00	0.00	NaN	NaN	NaN	0.00	0.00	0.00	0.00	0.00	0.00	0.00	0.00	0.00	0.00	0.00	0.00	0.00	0.00	0.00	0.00	0.00	0.00	0.00	0.00	0.00
**Total (Median & 95% UIs)**	352.04	410.41	484.30	1,715.97	2,003.93	2,341.00	2,089.87	2,416.21	2,800.68		-		17.07	19.89	23.48	83.18	97.14	113.48	101.31	117.13	135.77	1,991.86	2,280.27	2,576.98	96.56	110.54	124.92	81.64	95.83	111.76	3.96	4.65	5.42
**Total (average)**		**412.61**			**2,012.87**			**2,425.48**			-			**20.00**			**97.58**			**117.58**			**2,281.21**			**110.58**			**96.03**			**4.66**	

Infection	YLD			YLL			DALY			DALY per Case			YLD per 100,000			YLL per 100,000			DALY per 100,000			Cases per year			Incidence per 100,000			Deaths per year			Mortality per 100,000		
Colistin resistant *Acinetobacter spp.*	0.85	1.35	1.98	2.90	7.58	13.12	3.90	8.89	14.89	3.89	6.92	9.71	0.02	0.02	0.04	0.05	0.14	0.24	0.07	0.16	0.27	2.40	4.01	5.71	0.04	0.07	0.11	0.14	0.36	0.62	0.00	0.01	0.01
Carbapenem resistant *Acinetobacter spp.* (excluding those resistant to colistin)	57.83	65.36	73.85	285.30	350.86	426.52	344.43	416.32	493.22	6.51	7.71	8.99	1.07	1.21	1.36	5.26	6.47	7.87	6.35	7.68	9.10	141.74	159.95	177.82	2.61	2.95	3.28	11.21	13.98	16.90	0.21	0.26	0.31
Aminoglycosides and fluoroquinolones resistant *Acinetobacter* spp. (excluding those resistant to colistin and/or carbapenem)	36.94	42.21	48.16	175.01	222.83	272.77	215.14	265.17	318.45	5.76	6.85	8.01	0.68	0.78	0.89	3.23	4.11	5.03	3.97	4.89	5.87	99.44	115.63	131.77	1.83	2.13	2.43	7.74	9.97	12.37	0.14	0.18	0.23
Vancomycin resistant Enterococci (*E. faecalis* and *E. faecium*)	25.69	31.92	40.44	239.26	298.10	364.97	266.01	329.94	405.28	7.62	8.44	9.32	0.47	0.59	0.75	4.41	5.50	6.73	4.91	6.09	7.48	117.98	150.61	186.83	2.18	2.78	3.45	7.82	9.81	11.95	0.14	0.18	0.22
Colistin resistant *Escherichia coli*	0.56	0.81	1.15	8.02	13.25	19.02	8.65	14.00	20.11	3.47	5.22	6.92	0.01	0.01	0.02	0.15	0.24	0.35	0.16	0.26	0.37	14.56	20.93	27.36	0.27	0.39	0.50	1.13	1.86	2.65	0.02	0.03	0.05
Carbapenem resistant *Escherichia coli* (excluding those resistant to colistin)	0.00	0.00	0.00	0.00	0.00	0.00	0.00	0.00	0.00	0.00	0.00	0.00	0.00	0.00	0.00	0.00	0.00	0.00	0.00	0.00	0.00	0.00	0.00	0.00	0.00	0.00	0.00	0.00	0.00	0.00	0.00	0.00	0.00
Third-generation cephalosporin resistant *Escherichia coli* (excluding those resistant to colistin and/or carbapenem)	240.92	269.18	300.73	1,417.98	1,588.66	1,787.51	1,664.54	1,858.08	2,080.03	5.15	5.58	6.03	4.44	4.97	5.55	26.16	29.30	32.97	30.70	34.27	38.37	2,357.48	2,646.16	2,931.03	43.49	48.81	54.06	70.77	80.68	91.27	1.31	1.49	1.68
Colistin resistant *Klebsiella pneumoniae*	10.28	13.37	17.11	172.10	237.26	322.49	183.69	250.14	337.71	18.32	23.79	30.12	0.19	0.25	0.32	3.17	4.38	5.95	3.39	4.61	6.23	24.29	31.47	38.82	0.45	0.58	0.72	5.37	6.99	8.97	0.10	0.13	0.17
Carbapenem resistant *Klebsiella pneumoniae* (excluding those resistant to colistin)	6.15	8.18	10.78	61.41	89.86	121.50	67.99	97.94	131.17	14.19	19.19	24.33	0.11	0.15	0.20	1.13	1.66	2.24	1.25	1.81	2.42	11.37	15.72	20.10	0.21	0.29	0.37	1.53	2.13	2.75	0.03	0.04	0.05
Third-generation cephalosporin resistant *Klebsiella pneumoniae* (excluding those resistant to colistin and/or carbapenem)	453.56	493.49	537.08	2,219.25	2,365.01	2,516.71	2,687.87	2,861.99	3,042.12	7.04	7.37	7.70	8.37	9.10	9.91	40.94	43.62	46.42	49.58	52.79	56.11	1,459.90	1,590.42	1,721.88	26.93	29.34	31.76	80.69	86.08	91.99	1.49	1.59	1.70
Colistin resistant *Pseudomonas aeruginosa*	17.76	24.68	32.56	72.72	123.14	186.71	95.64	148.29	216.67	10.36	15.90	21.60	0.33	0.46	0.60	1.34	2.27	3.44	1.76	2.74	4.00	30.33	40.99	52.21	0.56	0.76	0.96	1.68	2.70	3.99	0.03	0.05	0.07
Carbapenem resistant *Pseudomonas aeruginosa* (excluding those resistant to colistin)	514.13	580.85	653.43	2,410.45	2,937.25	3,509.98	2,955.68	3,519.22	4,128.58	9.47	11.17	12.83	9.48	10.71	12.05	44.46	54.18	64.74	54.52	64.91	76.15	1,228.54	1,389.04	1,547.65	22.66	25.62	28.55	76.77	93.83	112.68	1.42	1.73	2.08
Pseudomonas aeruginosa resistant to three or more antimicrobial groups (excluding those resistant to colistin and/or carbapenem)	23.20	28.43	34.34	92.75	134.14	182.35	119.42	162.69	213.04	4.71	6.35	8.05	0.43	0.52	0.63	1.71	2.47	3.36	2.20	3.00	3.93	89.40	112.79	136.71	1.65	2.08	2.52	4.93	7.20	9.81	0.09	0.13	0.18
Methicillin resistant *Staphylococcus aureus*	282.69	308.35	338.95	1.381.88	1.476.71	1.580.26	1.668.68	1.786.09	1.910.92	6.75	7.02	7.30	5.21	5.69	6.25	25.49	27.24	29.15	30.78	32.95	35.25	1.208.82	1.310.41	1.417.76	22.30	24.17	26.15	56.47	60.81	65.38	1.04	1.12	1.21
Penicillin and macrolides resistant *Streptococcus pneumoniae* (excluding those only resistant to penicillin)	9.19	11.11	13.76	31.61	36.10	41.84	41.36	47.24	54.70	4.14	4.48	4.82	0.17	0.20	0.25	0.58	0.67	0.77	0.76	0.87	1.01	29.87	34.34	40.37	0.55	0.63	0.74	1.86	2.11	2.41	0.03	0.04	0.04
Penicillin tesistant *Streptococcus pneumoniae* (excluding those resistant to macrolides)	0.00	0.00	0.00	0.00	0.00	0.00	0.00	0.00	0.00	0.00	0.00	0.00	0.00	0.00	0.00	0.00	0.00	0.00	0.00	0.00	0.00	0.00	0.00	0.00	0.00	0.00	0.00	0.00	0.00	0.00	0.00	0.00	0.00
Total	1,679.75	1,879.28	2,104.32	8,570.64	9,880.76	11,345.76	10,323.01	11,765.99	13,366.90				30.98	34.66	38.82	158.09	182.26	209.28	190.41	217.03	246.56	6,816.10	7,622.46	8,436.00	125.73	140.60	155.61	328.12	378.50	433.74	6.05	6.98	8.00

Model	YLD			YLL			DALY			DALY per Case			YLD per 100,000			YLL per 100,000			DALY per 100,000			Cases per year			Incidence per 100,000			Deaths per year			Mortality per 100,000		
Slovakia - Acinetobacter spp. CRACI BSI - model 36	33.46	37.23	41.72	230.55	287.49	354.31	264.61	324.84	389.13	4.81	5.89	7.04	0.62	0.69	0.77	4.25	5.30	6.54	4.88	5.99	7.18	51.85	55.33	58.80	0.96	1.02	1.08	8.96	11.37	13.93	0.17	0.21	0.26
Slovakia - Acinetobacter spp. CRACI OTH - model 40	4.84E-03	7.25E-03	9.94E-03	0.00	0.00	0.00	4.84E-03	7.25E-03	9.94E-03	2.95E-03	3.74E-03	4.61E-03	8.92E-05	1.34E-04	1.83E-04	0.00	0.00	0.00	8.92E-05	1.34E-04	1.83E-04	1.39	1.94	2.45	0.03	0.04	0.05	0.00	0.00	0.00	0.00	0.00	0.00
Slovakia - Acinetobacter spp. CRACI RESP - model 37	24.29	28.04	32.00	47.69	54.60	61.80	72.69	82.61	93.55	1.30	1.38	1.48	0.45	0.52	0.59	0.88	1.01	1.14	1.34	1.52	1.73	53.37	59.90	66.65	0.98	1.10	1.23	1.88	2.15	2.43	0.03	0.04	0.04
Slovakia - Acinetobacter spp. CRACI SSI - model 39	0.03	0.04	0.06	7.06	8.78	10.42	7.10	8.82	10.47	0.40	0.44	0.47	5.60E-04	7.90E-04	1.09E-03	0.13	0.16	0.19	0.13	0.16	0.19	16.78	20.26	23.52	0.31	0.37	0.43	0.37	0.46	0.54	6.75E-03	8.43E-03	0.01
Slovakia - Acinetobacter spp. CRACI UTI - model 38	0.03	0.05	0.06	0.00	0.00	0.00	0.03	0.05	0.06	1.61E-03	2.03E-03	2.48E-03	6.30E-04	8.38E-04	1.09E-03	0.00	0.00	0.00	6.30E-04	8.38E-04	1.09E-03	18.35	22.53	26.40	0.34	0.42	0.49	0.00	0.00	0.00	0.00	0.00	0.00
Slovakia - Acinetobacter spp. ColRACI BSI - model 41	0.53	0.77	1.11	2.12	6.05	10.84	2.80	6.77	11.76	2.16	5.00	7.62	9.72E-03	0.01	0.02	0.04	0.11	0.20	0.05	0.12	0.22	1.09	1.37	1.76	0.02	0.03	0.03	0.10	0.29	0.51	1.86E-03	5.30E-03	9.44E-03
Slovakia - Acinetobacter spp. ColRACI OTH - model 45	2.69E-05	1.66E-04	4.74E-04	0.00	0.00	0.00	2.69E-05	1.66E-04	4.74E-04	1.55E-03	3.78E-03	6.38E-03	4.96E-07	3.07E-06	8.74E-06	0.00	0.00	0.00	4.96E-07	3.07E-06	8.74E-06	9.39E-03	0.05	0.10	1.73E-04	8.91E-04	1.89E-03	0.00	0.00	0.00	0.00	0.00	0.00
Slovakia - Acinetobacter spp. ColRACI RESP - model 42	0.32	0.58	0.87	0.61	1.14	1.67	0.94	1.73	2.51	0.97	1.15	1.31	5.97E-03	0.01	0.02	0.01	0.02	0.03	0.02	0.03	0.05	0.87	1.52	2.13	0.02	0.03	0.04	0.03	0.05	0.08	5.38E-04	1.00E-03	1.46E-03
Slovakia - Acinetobacter spp. ColRACI SSI - model 44	2.01E-04	9.68E-04	2.73E-03	0.16	0.38	0.61	0.16	0.39	0.61	0.76	0.76	0.76	3.71E-06	1.79E-05	5.03E-05	2.92E-03	7.09E-03	0.01	2.91E-03	7.10E-03	0.01	0.21	0.51	0.80	3.84E-03	9.33E-03	0.01	7.49E-03	0.02	0.03	1.38E-04	3.36E-04	5.33E-04
Slovakia - Acinetobacter spp. ColRACI UTI - model 43	2.98E-04	1.00E-03	2.54E-03	0.00	0.00	0.00	2.98E-04	1.00E-03	2.54E-03	8.36E-04	1.90E-03	3.65E-03	5.50E-06	1.85E-05	4.68E-05	0.00	0.00	0.00	5.50E-06	1.85E-05	4.68E-05	0.22	0.56	0.92	4.00E-03	0.01	0.02	0.00	0.00	0.00	0.00	0.00	0.00
Slovakia - Acinetobacter spp. MDRACI BSI - model 31	21.56	24.10	27.17	139.04	180.06	223.41	163.55	204.23	248.60	4.11	5.09	6.13	0.40	0.44	0.50	2.56	3.32	4.12	3.02	3.77	4.59	37.08	40.08	43.06	0.68	0.74	0.79	6.11	8.00	10.06	0.11	0.15	0.19
Slovakia - Acinetobacter spp. MDRACI OTH - model 35	3.34E-03	5.25E-03	7.71E-03	0.00	0.00	0.00	3.34E-03	5.25E-03	7.71E-03	2.87E-03	3.73E-03	4.68E-03	6.16E-05	9.68E-05	1.42E-04	0.00	0.00	0.00	6.16E-05	9.68E-05	1.42E-04	0.97	1.41	1.89	0.02	0.03	0.03	0.00	0.00	0.00	0.00	0.00	0.00
Slovakia - Acinetobacter spp. MDRACI RESP - model 32	15.34	18.04	20.89	30.01	35.05	39.98	45.58	53.14	60.39	1.15	1.23	1.32	0.28	0.33	0.39	0.55	0.65	0.74	0.84	0.98	1.11	36.93	43.17	49.34	0.68	0.80	0.91	1.31	1.55	1.80	0.02	0.03	0.03
Slovakia - Acinetobacter spp. MDRACI SSI - model 34	0.02	0.03	0.05	5.97	7.72	9.38	5.99	7.76	9.41	0.49	0.53	0.56	3.71E-04	5.68E-04	8.39E-04	0.11	0.14	0.17	0.11	0.14	0.17	11.75	14.64	17.51	0.22	0.27	0.32	0.32	0.42	0.51	5.92E-03	7.71E-03	9.41E-03
Slovakia - Acinetobacter spp. MDRACI UTI - model 33	0.02	0.03	0.05	0.00	0.00	0.00	0.02	0.03	0.05	1.53E-03	2.01E-03	2.57E-03	4.21E-04	6.08E-04	8.34E-04	0.00	0.00	0.00	4.21E-04	6.08E-04	8.34E-04	12.71	16.32	19.96	0.23	0.30	0.37	0.00	0.00	0.00	0.00	0.00	0.00
Slovakia - Enterococcus faecalis and E. faecium VRE BSI - model 66	25.54	31.70	40.14	220.77	273.43	333.88	247.38	305.04	373.92	7.19	7.96	8.80	0.47	0.58	0.74	4.07	5.04	6.16	4.56	5.63	6.90	31.05	38.21	46.81	0.57	0.70	0.86	7.10	8.76	10.59	0.13	0.16	0.20
Slovakia - Enterococcus faecalis and E. faecium VRE OTH - model 70	0.05	0.07	0.09	0.00	0.00	0.00	0.05	0.07	0.09	1.23E-03	1.65E-03	2.07E-03	8.53E-04	1.24E-03	1.72E-03	0.00	0.00	0.00	8.53E-04	1.24E-03	1.72E-03	32.08	41.05	50.56	0.59	0.76	0.93	0.00	0.00	0.00	0.00	0.00	0.00
Slovakia - Enterococcus faecalis and E. faecium VRE RESP - model 67	0.00	0.00	0.00	0.00	0.00	0.00	0.00	0.00	0.00	NaN	NaN	NaN	0.00	0.00	0.00	0.00	0.00	0.00	0.00	0.00	0.00	0.00	0.00	0.00	0.00	0.00	0.00	0.00	0.00	0.00	0.00	0.00	0.00
Slovakia - Enterococcus faecalis and E. faecium VRE SSI - model 69	0.07	0.11	0.16	18.49	24.67	31.09	18.56	24.79	31.23	0.43	0.48	0.52	1.37E-03	2.02E-03	2.96E-03	0.34	0.46	0.57	0.34	0.46	0.58	40.18	52.03	65.32	0.74	0.96	1.20	0.73	1.05	1.36	0.01	0.02	0.03
Slovakia - Enterococcus faecalis and E. faecium VRE UTI - model 68	0.02	0.04	0.05	0.00	0.00	0.00	0.02	0.04	0.05	1.43E-03	1.88E-03	2.37E-03	4.47E-04	6.65E-04	9.02E-04	0.00	0.00	0.00	4.47E-04	6.65E-04	9.02E-04	14.67	19.32	24.14	0.27	0.36	0.45	0.00	0.00	0.00	0.00	0.00	0.00
Slovakia - Escherichia coli 3GCREC BSI - model 16	185.55	203.20	222.34	1,177.24	1,307.68	1,461.78	1,369.57	1,510.44	1,677.25	3.74	4.05	4.38	3.42	3.75	4.10	21.71	24.12	26.96	25.26	27.86	30.94	347.38	372.94	399.63	6.41	6.88	7.37	56.92	64.35	72.37	1.05	1.19	1.33
Slovakia - Escherichia coli 3GCREC OTH - model 20	0.30	0.41	0.52	0.00	0.00	0.00	0.30	0.41	0.52	2.71E-03	3.33E-03	4.07E-03	5.59E-03	7.51E-03	9.61E-03	0.00	0.00	0.00	5.59E-03	7.51E-03	9.61E-03	99.50	122.35	143.82	1.84	2.26	2.65	0.00	0.00	0.00	0.00	0.00	0.00
Slovakia - Escherichia coli 3GCREC RESP - model 17	52.00	61.63	72.93	100.84	118.85	140.02	152.72	181.09	212.31	1.02	1.11	1.21	0.96	1.14	1.35	1.86	2.19	2.58	2.82	3.34	3.92	135.58	163.31	191.93	2.50	3.01	3.54	4.89	5.87	6.98	0.09	0.11	0.13
Slovakia - Escherichia coli 3GCREC SSI - model 19	0.62	0.83	1.07	139.90	162.13	185.71	139.50	163.02	186.07	0.39	0.42	0.44	0.01	0.02	0.02	2.58	2.99	3.43	2.57	3.01	3.43	334.20	391.49	441.53	6.16	7.22	8.14	8.96	10.46	11.92	0.17	0.19	0.22
Slovakia - Escherichia coli 3GCREC UTI - model 18	2.44	3.12	3.87	0.00	0.00	0.00	2.44	3.12	3.87	1.58E-03	1.96E-03	2.35E-03	0.05	0.06	0.07	0.00	0.00	0.00	0.05	0.06	0.07	1,440.82	1,596.07	1,754.11	26.58	29.44	32.36	0.00	0.00	0.00	0.00	0.00	0.00
Slovakia - Escherichia coli CREC BSI - model 21	0.00	0.00	0.00	0.00	0.00	0.00	0.00	0.00	0.00	NaN	NaN	NaN	0.00	0.00	0.00	0.00	0.00	0.00	0.00	0.00	0.00	0.00	0.00	0.00	0.00	0.00	0.00	0.00	0.00	0.00	0.00	0.00	0.00
Slovakia - Escherichia coli CREC OTH - model 25	0.00	0.00	0.00	0.00	0.00	0.00	0.00	0.00	0.00	NaN	NaN	NaN	0.00	0.00	0.00	0.00	0.00	0.00	0.00	0.00	0.00	0.00	0.00	0.00	0.00	0.00	0.00	0.00	0.00	0.00	0.00	0.00	0.00
Slovakia - Escherichia coli CREC RESP - model 22	0.00	0.00	0.00	0.00	0.00	0.00	0.00	0.00	0.00	NaN	NaN	NaN	0.00	0.00	0.00	0.00	0.00	0.00	0.00	0.00	0.00	0.00	0.00	0.00	0.00	0.00	0.00	0.00	0.00	0.00	0.00	0.00	0.00
Slovakia - Escherichia coli CREC SSI - model 24	0.00	0.00	0.00	0.00	0.00	0.00	0.00	0.00	0.00	NaN	NaN	NaN	0.00	0.00	0.00	0.00	0.00	0.00	0.00	0.00	0.00	0.00	0.00	0.00	0.00	0.00	0.00	0.00	0.00	0.00	0.00	0.00	0.00
Slovakia - Escherichia coli CREC UTI - model 23	0.00	0.00	0.00	0.00	0.00	0.00	0.00	0.00	0.00	NaN	NaN	NaN	0.00	0.00	0.00	0.00	0.00	0.00	0.00	0.00	0.00	0.00	0.00	0.00	0.00	0.00	0.00	0.00	0.00	0.00	0.00	0.00	0.00
Slovakia - Escherichia coli ColREC BSI - model 26	0.44	0.58	0.78	7.30	12.03	17.33	7.80	12.55	18.06	2.90	4.56	6.14	8.11E-03	0.01	0.01	0.13	0.22	0.32	0.14	0.23	0.33	2.30	2.76	3.37	0.04	0.05	0.06	1.02	1.69	2.42	0.02	0.03	0.04
Slovakia - Escherichia coli ColREC OTH - model 30	2.19E-03	5.38E-03	0.01	0.00	0.00	0.00	2.19E-03	5.38E-03	0.01	1.56E-03	3.41E-03	5.94E-03	4.03E-05	9.92E-05	1.90E-04	0.00	0.00	0.00	4.03E-05	9.92E-05	1.90E-04	0.93	1.61	2.27	0.02	0.03	0.04	0.00	0.00	0.00	0.00	0.00	0.00
Slovakia - Escherichia coli ColREC RESP - model 27	0.11	0.20	0.30	0.19	0.36	0.54	0.30	0.56	0.84	0.33	0.39	0.48	1.98E-03	3.60E-03	5.60E-03	3.58E-03	6.68E-03	9.99E-03	5.59E-03	0.01	0.02	0.82	1.42	2.07	0.02	0.03	0.04	0.03	0.05	0.07	5.32E-04	9.35E-04	1.37E-03
Slovakia - Escherichia coli ColREC SSI - model 29	2.81E-03	6.75E-03	0.01	0.53	0.86	1.15	0.54	0.87	1.16	0.23	0.26	0.29	5.18E-05	1.25E-04	2.44E-04	9.82E-03	0.02	0.02	9.90E-03	0.02	0.02	2.15	3.34	4.44	0.04	0.06	0.08	0.08	0.12	0.16	1.43E-03	2.22E-03	2.95E-03
Slovakia - Escherichia coli ColREC UTI - model 28	0.01	0.02	0.04	0.00	0.00	0.00	0.01	0.02	0.04	1.00E-03	2.02E-03	3.42E-03	2.09E-04	4.37E-04	8.14E-04	0.00	0.00	0.00	2.09E-04	4.37E-04	8.14E-04	8.36	11.79	15.21	0.15	0.22	0.28	0.00	0.00	0.00	0.00	0.00	0.00
Slovakia - Klebsiella pneumoniae 3GCRKP BSI - model 1	285.97	307.51	331.22	1,831.08	1,932.76	2,045.36	2,128.91	2,243.76	2,366.47	5.19	5.39	5.60	5.27	5.67	6.11	33.78	35.65	37.73	39.27	41.39	43.65	397.96	416.06	434.35	7.34	7.67	8.01	65.86	69.37	73.38	1.21	1.28	1.35
Slovakia - Klebsiella pneumoniae 3GCRKP OTH - model 5	0.45	0.55	0.67	0.00	0.00	0.00	0.45	0.55	0.67	2.79E-03	3.35E-03	3.95E-03	8.30E-03	0.01	0.01	0.00	0.00	0.00	8.30E-03	0.01	0.01	147.22	164.85	182.06	2.72	3.04	3.36	0.00	0.00	0.00	0.00	0.00	0.00
Slovakia - Klebsiella pneumoniae 3GCRKP RESP - model 2	166.10	184.13	203.60	322.96	357.99	387.87	492.20	542.08	590.01	1.42	1.52	1.61	3.06	3.40	3.76	5.96	6.60	7.15	9.08	10.00	10.88	324.69	357.04	392.59	5.99	6.59	7.24	11.54	12.87	14.24	0.21	0.24	0.26
Slovakia - Klebsiella pneumoniae 3GCRKP SSI - model 4	0.27	0.35	0.44	65.21	74.26	83.47	65.53	74.64	83.81	0.43	0.46	0.48	5.00E-03	6.41E-03	8.07E-03	1.20	1.37	1.54	1.21	1.38	1.55	145.76	163.42	181.73	2.69	3.01	3.35	3.30	3.84	4.37	0.06	0.07	0.08
Slovakia - Klebsiella pneumoniae 3GCRKP UTI - model 3	0.78	0.96	1.16	0.00	0.00	0.00	0.78	0.96	1.16	1.64E-03	1.96E-03	2.30E-03	0.01	0.02	0.02	0.00	0.00	0.00	0.01	0.02	0.02	444.27	489.04	531.15	8.19	9.02	9.80	0.00	0.00	0.00	0.00	0.00	0.00
Slovakia - Klebsiella pneumoniae CRKP BSI - model 6	5.07	6.31	8.07	58.70	85.39	115.04	64.22	91.59	122.12	11.96	16.37	20.77	0.09	0.12	0.15	1.08	1.58	2.12	1.18	1.69	2.25	4.83	5.62	6.53	0.09	0.10	0.12	1.47	2.02	2.59	0.03	0.04	0.05
Slovakia - Klebsiella pneumoniae CRKP OTH - model 10	4.57E-03	8.88E-03	0.01	0.00	0.00	0.00	4.57E-03	8.88E-03	0.01	2.10E-03	3.47E-03	5.22E-03	8.43E-05	1.64E-04	2.76E-04	0.00	0.00	0.00	8.43E-05	1.64E-04	2.76E-04	1.75	2.63	3.55	0.03	0.05	0.07	0.00	0.00	0.00	0.00	0.00	0.00
Slovakia - Klebsiella pneumoniae CRKP RESP - model 7	1.07	1.85	2.68	2.19	3.59	5.24	3.25	5.46	7.79	1.80	2.28	2.93	0.02	0.03	0.05	0.04	0.07	0.10	0.06	0.10	0.14	1.52	2.40	3.18	0.03	0.04	0.06	0.05	0.09	0.12	9.91E-04	1.58E-03	2.19E-03
Slovakia - Klebsiella pneumoniae CRKP SSI - model 9	1.69E-03	3.40E-03	6.22E-03	0.51	0.87	1.23	0.51	0.88	1.23	0.43	0.53	0.62	3.12E-05	6.27E-05	1.15E-04	9.48E-03	0.02	0.02	9.48E-03	0.02	0.02	1.03	1.66	2.29	0.02	0.03	0.04	0.01	0.03	0.04	2.66E-04	4.83E-04	7.10E-04
Slovakia - Klebsiella pneumoniae CRKP UTI - model 8	3.66E-03	7.08E-03	0.01	0.00	0.00	0.00	3.66E-03	7.08E-03	0.01	1.28E-03	2.09E-03	3.09E-03	6.74E-05	1.31E-04	2.16E-04	0.00	0.00	0.00	6.74E-05	1.31E-04	2.16E-04	2.23	3.42	4.55	0.04	0.06	0.08	0.00	0.00	0.00	0.00	0.00	0.00
Slovakia - Klebsiella pneumoniae ColRKP BSI - model 11	8.41	10.31	12.81	167.34	229.76	312.19	177.10	239.59	323.28	16.37	21.44	27.36	0.16	0.19	0.24	3.09	4.24	5.76	3.27	4.42	5.96	9.90	11.22	12.75	0.18	0.21	0.24	5.21	6.75	8.66	0.10	0.12	0.16
Slovakia - Klebsiella pneumoniae ColRKP OTH - model 15	0.01	0.02	0.03	0.00	0.00	0.00	0.01	0.02	0.03	2.36E-03	3.51E-03	4.94E-03	2.06E-04	3.37E-04	5.25E-04	0.00	0.00	0.00	2.06E-04	3.37E-04	5.25E-04	3.84	5.29	6.74	0.07	0.10	0.12	0.00	0.00	0.00	0.00	0.00	0.00
Slovakia - Klebsiella pneumoniae ColRKP RESP - model 12	1.84	3.02	4.24	3.69	5.89	8.18	5.49	8.90	12.25	1.52	1.86	2.21	0.03	0.06	0.08	0.07	0.11	0.15	0.10	0.16	0.23	3.30	4.80	6.18	0.06	0.09	0.11	0.12	0.17	0.22	2.18E-03	3.18E-03	4.14E-03
Slovakia - Klebsiella pneumoniae ColRKP SSI - model 14	3.83E-03	7.00E-03	0.01	1.08	1.60	2.12	1.08	1.61	2.13	0.43	0.48	0.54	7.06E-05	1.29E-04	2.10E-04	0.02	0.03	0.04	0.02	0.03	0.04	2.30	3.33	4.46	0.04	0.06	0.08	0.04	0.06	0.09	7.25E-04	1.18E-03	1.57E-03
Slovakia - Klebsiella pneumoniae ColRKP UTI - model 13	8.93E-03	0.01	0.02	0.00	0.00	0.00	8.93E-03	0.01	0.02	1.43E-03	2.07E-03	2.87E-03	1.65E-04	2.58E-04	3.96E-04	0.00	0.00	0.00	1.65E-04	2.58E-04	3.96E-04	4.96	6.83	8.69	0.09	0.13	0.16	0.00	0.00	0.00	0.00	0.00	0.00
Slovakia - Pseudomonas aeruginosa CRPA BSI - model 51	146.40	162.67	179.73	1,132.88	1,400.71	1,675.67	1,290.00	1,561.93	1,842.31	6.74	8.07	9.37	2.70	3.00	3.32	20.90	25.84	30.91	23.79	28.81	33.98	178.36	193.05	208.12	3.29	3.56	3.84	36.19	44.40	53.62	0.67	0.82	0.99
Slovakia - Pseudomonas aeruginosa CRPA OTH - model 55	0.28	0.36	0.46	0.00	0.00	0.00	0.28	0.36	0.46	3.06E-03	3.67E-03	4.29E-03	5.13E-03	6.55E-03	8.41E-03	0.00	0.00	0.00	5.13E-03	6.55E-03	8.41E-03	82.98	97.51	112.77	1.53	1.80	2.08	0.00	0.00	0.00	0.00	0.00	0.00
Slovakia - Pseudomonas aeruginosa CRPA RESP - model 52	366.84	417.03	472.25	1,229.65	1,479.27	1,767.99	1,616.84	1,898.85	2,218.57	2.32	2.66	2.98	6.77	7.69	8.71	22.68	27.29	32.61	29.82	35.03	40.92	637.03	716.86	791.25	11.75	13.22	14.60	38.55	46.97	56.15	0.71	0.87	1.04
Slovakia - Pseudomonas aeruginosa CRPA SSI - model 54	0.21	0.28	0.36	47.92	57.28	66.32	48.16	57.57	66.62	0.40	0.43	0.47	3.85E-03	5.22E-03	6.68E-03	0.88	1.06	1.22	0.89	1.06	1.23	112.49	132.34	152.79	2.07	2.44	2.82	2.03	2.46	2.90	0.04	0.05	0.05
Slovakia - Pseudomonas aeruginosa CRPA UTI - model 53	0.40	0.51	0.63	0.00	0.00	0.00	0.40	0.51	0.63	1.69E-03	2.05E-03	2.39E-03	7.46E-03	9.38E-03	0.01	0.00	0.00	0.00	7.46E-03	9.38E-03	0.01	217.68	249.28	282.71	4.02	4.60	5.21	0.00	0.00	0.00	0.00	0.00	0.00
Slovakia - Pseudomonas aeruginosa ColRPA BSI - model 56	5.33	6.86	8.95	32.14	59.52	89.58	40.43	66.64	98.29	7.23	11.67	16.03	0.10	0.13	0.17	0.59	1.10	1.65	0.75	1.23	1.81	4.74	5.65	6.81	0.09	0.10	0.13	0.77	1.30	1.94	0.01	0.02	0.04
Slovakia - Pseudomonas aeruginosa ColRPA OTH - model 60	5.97E-03	0.01	0.02	0.00	0.00	0.00	5.97E-03	0.01	0.02	2.42E-03	3.78E-03	5.21E-03	1.10E-04	1.97E-04	3.09E-04	0.00	0.00	0.00	1.10E-04	1.97E-04	3.09E-04	1.91	2.85	3.91	0.04	0.05	0.07	0.00	0.00	0.00	0.00	0.00	0.00
Slovakia - Pseudomonas aeruginosa ColRPA RESP - model 57	12.41	17.78	23.56	39.59	62.01	94.93	54.20	80.01	116.13	2.78	3.81	5.08	0.23	0.33	0.43	0.73	1.14	1.75	1.00	1.48	2.14	16.04	21.23	26.51	0.30	0.39	0.49	0.89	1.37	2.00	0.02	0.03	0.04
Slovakia - Pseudomonas aeruginosa ColRPA SSI - model 59	4.10E-03	8.05E-03	0.01	0.99	1.61	2.21	1.00	1.61	2.22	0.35	0.41	0.48	7.56E-05	1.48E-04	2.68E-04	0.02	0.03	0.04	0.02	0.03	0.04	2.54	3.91	5.30	0.05	0.07	0.10	0.02	0.04	0.05	4.21E-04	6.50E-04	8.79E-04
Slovakia - Pseudomonas aeruginosa ColRPA UTI - model 58	8.33E-03	0.01	0.02	0.00	0.00	0.00	8.33E-03	0.01	0.02	1.29E-03	2.01E-03	2.86E-03	1.54E-04	2.70E-04	4.17E-04	0.00	0.00	0.00	1.54E-04	2.70E-04	4.17E-04	5.10	7.34	9.68	0.09	0.14	0.18	0.00	0.00	0.00	0.00	0.00	0.00
Slovakia - Pseudomonas aeruginosa MDRPA BSI - model 46	6.78	7.98	9.52	42.83	61.82	84.76	51.15	70.00	92.48	3.23	4.47	5.77	0.13	0.15	0.18	0.79	1.14	1.56	0.94	1.29	1.71	13.71	15.60	18.00	0.25	0.29	0.33	2.25	3.29	4.51	0.04	0.06	0.08
Slovakia - Pseudomonas aeruginosa MDRPA OTH - model 50	0.02	0.03	0.04	0.00	0.00	0.00	0.02	0.03	0.04	2.67E-03	3.74E-03	4.92E-03	3.39E-04	5.46E-04	8.06E-04	0.00	0.00	0.00	3.39E-04	5.46E-04	8.06E-04	5.66	7.98	10.14	0.10	0.15	0.19	0.00	0.00	0.00	0.00	0.00	0.00
Slovakia - Pseudomonas aeruginosa MDRPA RESP - model 47	16.36	20.35	24.69	47.25	68.71	93.06	65.54	88.98	115.88	1.17	1.54	1.89	0.30	0.38	0.46	0.87	1.27	1.72	1.21	1.64	2.14	46.98	58.20	69.78	0.87	1.07	1.29	2.49	3.62	4.92	0.05	0.07	0.09
Slovakia - Pseudomonas aeruginosa MDRPA SSI - model 49	0.01	0.02	0.04	2.66	3.61	4.54	2.69	3.64	4.58	0.30	0.34	0.38	2.47E-04	4.20E-04	6.83E-04	0.05	0.07	0.08	0.05	0.07	0.08	7.76	10.79	13.59	0.14	0.20	0.25	0.19	0.28	0.38	3.51E-03	5.20E-03	6.99E-03
Slovakia - Pseudomonas aeruginosa MDRPA UTI - model 48	0.03	0.04	0.06	0.00	0.00	0.00	0.03	0.04	0.06	1.42E-03	2.03E-03	2.63E-03	5.02E-04	7.53E-04	1.08E-03	0.00	0.00	0.00	5.02E-04	7.53E-04	1.08E-03	15.28	20.22	25.21	0.28	0.37	0.46	0.00	0.00	0.00	0.00	0.00	0.00
Slovakia - Staphylococcus aureus MRSA BSI - model 61	139.59	150.72	163.40	961.80	1,014.32	1,074.11	1,103.29	1,165.82	1,230.91	5.02	5.21	5.40	2.57	2.78	3.01	17.74	18.71	19.81	20.35	21.50	22.70	213.84	223.76	233.75	3.94	4.13	4.31	37.84	40.26	42.60	0.70	0.74	0.79
Slovakia - Staphylococcus aureus MRSA OTH - model 65	0.87	1.08	1.32	0.00	0.00	0.00	0.87	1.08	1.32	2.71E-03	3.28E-03	3.89E-03	0.02	0.02	0.02	0.00	0.00	0.00	0.02	0.02	0.02	304.25	329.77	356.86	5.61	6.08	6.58	0.00	0.00	0.00	0.00	0.00	0.00
Slovakia - Staphylococcus aureus MRSA RESP - model 62	141.58	155.66	173.10	276.91	304.42	333.30	420.54	460.39	504.71	1.31	1.37	1.45	2.61	2.87	3.19	5.11	5.62	6.15	7.76	8.49	9.31	305.72	334.70	366.08	5.64	6.17	6.75	10.97	12.05	13.38	0.20	0.22	0.25
Slovakia - Staphylococcus aureus MRSA SSI - model 64	0.58	0.78	0.99	143.17	157.97	172.86	143.90	158.69	173.85	0.41	0.43	0.45	0.01	0.01	0.02	2.64	2.91	3.19	2.65	2.93	3.21	338.63	367.23	397.67	6.25	6.77	7.34	7.66	8.51	9.40	0.14	0.16	0.17
Slovakia - Staphylococcus aureus MRSA UTI - model 63	0.08	0.10	0.13	0.00	0.00	0.00	0.08	0.10	0.13	1.52E-03	1.87E-03	2.23E-03	1.46E-03	1.88E-03	2.40E-03	0.00	0.00	0.00	1.46E-03	1.88E-03	2.40E-03	46.38	54.94	63.39	0.86	1.01	1.17	0.00	0.00	0.00	0.00	0.00	0.00
Slovakia - Streptococcus pneumoniae PMRSP BSI - model 76	2.63	3.04	3.51	19.00	20.56	21.94	22.01	23.58	25.02	3.31	3.55	3.76	0.05	0.06	0.06	0.35	0.38	0.40	0.41	0.44	0.46	6.65	6.65	6.65	0.12	0.12	0.12	1.12	1.20	1.27	0.02	0.02	0.02
Slovakia - Streptococcus pneumoniae PMRSP OTH - model 80	3.11E-03	4.69E-03	6.44E-03	0.00	0.00	0.00	3.11E-03	4.69E-03	6.44E-03	1.32E-03	1.99E-03	2.65E-03	5.74E-05	8.65E-05	1.19E-04	0.00	0.00	0.00	5.74E-05	8.65E-05	1.19E-04	2.13	2.36	2.66	0.04	0.04	0.05	0.00	0.00	0.00	0.00	0.00	0.00
Slovakia - Streptococcus pneumoniae PMRSP RESP - model 77	6.55	8.07	10.24	12.61	15.54	19.89	19.34	23.66	29.67	0.83	0.93	1.05	0.12	0.15	0.19	0.23	0.29	0.37	0.36	0.44	0.55	21.10	25.32	31.07	0.39	0.47	0.57	0.74	0.91	1.14	0.01	0.02	0.02
Slovakia - Streptococcus pneumoniae PMRSP SSI - model 79	0.00	0.00	0.00	0.00	0.00	0.00	0.00	0.00	0.00	NaN	NaN	NaN	0.00	0.00	0.00	0.00	0.00	0.00	0.00	0.00	0.00	0.00	0.00	0.00	0.00	0.00	0.00	0.00	0.00	0.00	0.00	0.00	0.00
Slovakia - Streptococcus pneumoniae PMRSP UTI - model 78	0.00	0.00	0.00	0.00	0.00	0.00	0.00	0.00	0.00	NaN	NaN	NaN	0.00	0.00	0.00	0.00	0.00	0.00	0.00	0.00	0.00	0.00	0.00	0.00	0.00	0.00	0.00	0.00	0.00	0.00	0.00	0.00	0.00
Slovakia - Streptococcus pneumoniae PRSP BSI - model 71	0.00	0.00	0.00	0.00	0.00	0.00	0.00	0.00	0.00	NaN	NaN	NaN	0.00	0.00	0.00	0.00	0.00	0.00	0.00	0.00	0.00	0.00	0.00	0.00	0.00	0.00	0.00	0.00	0.00	0.00	0.00	0.00	0.00
Slovakia - Streptococcus pneumoniae PRSP OTH - model 75	0.00	0.00	0.00	0.00	0.00	0.00	0.00	0.00	0.00	NaN	NaN	NaN	0.00	0.00	0.00	0.00	0.00	0.00	0.00	0.00	0.00	0.00	0.00	0.00	0.00	0.00	0.00	0.00	0.00	0.00	0.00	0.00	0.00
Slovakia - Streptococcus pneumoniae PRSP RESP - model 72	0.00	0.00	0.00	0.00	0.00	0.00	0.00	0.00	0.00	NaN	NaN	NaN	0.00	0.00	0.00	0.00	0.00	0.00	0.00	0.00	0.00	0.00	0.00	0.00	0.00	0.00	0.00	0.00	0.00	0.00	0.00	0.00	0.00
Slovakia - Streptococcus pneumoniae PRSP SSI - model 74	0.00	0.00	0.00	0.00	0.00	0.00	0.00	0.00	0.00	NaN	NaN	NaN	0.00	0.00	0.00	0.00	0.00	0.00	0.00	0.00	0.00	0.00	0.00	0.00	0.00	0.00	0.00	0.00	0.00	0.00	0.00	0.00	0.00
Slovakia - Streptococcus pneumoniae PRSP UTI - model 73	0.00	0.00	0.00	0.00	0.00	0.00	0.00	0.00	0.00	NaN	NaN	NaN	0.00	0.00	0.00	0.00	0.00	0.00	0.00	0.00	0.00	0.00	0.00	0.00	0.00	0.00	0.00	0.00	0.00	0.00	0.00	0.00	0.00
**Total (Median & 95% UIs)**	1,679.75	1,879.28	2,104.32	8,570.64	9,880.76	11,345.76	10,323.01	11,765.99	13,366.90		-		30.98	34.66	38.82	158.09	182.26	209.28	190.41	217.03	246.56	6,816.10	7,622.46	8,436.00	125.73	140.60	155.61	328.12	378.50	433.74	6.05	6.98	8.00
**Total (average)**		**1,882.89**			**9,897.54**			**11,780.42**			-			**34.73**			**182.57**			**217.30**			**7,626.77**			**140.68**			**379.06**			**6.99**	

Infection	YLD			YLL			DALY			DALY per Case			YLD per 100,000			YLL per 100,000			DALY per 100,000			Cases per year			Incidence per 100,000			Deaths per year			Mortality per 100,000		
Colistin resistant *Acinetobacter spp.*	19.04	28.90	41.82	70.54	156.88	258.77	93.38	186.86	295.61	5.98	10.88	16.32	0.03	0.04	0.06	0.11	0.24	0.40	0.14	0.29	0.46	34.96	49.76	65.70	0.05	0.08	0.10	2.11	4.35	6.59	0.00	0.01	0.01
Carbapenem resistant *Acinetobacter spp.* (excluding those resistant to colistin)	6.35	10.10	15.13	21.52	54.19	95.48	29.24	64.49	107.55	4.10	7.58	11.01	0.01	0.02	0.02	0.03	0.08	0.15	0.05	0.10	0.17	15.00	24.96	35.52	0.02	0.04	0.05	0.85	2.12	3.74	0.00	0.00	0.01
Aminoglycosides and fluoroquinolones resistant *Acinetobacter* spp. (excluding those resistant to colistin and/or carbapenem)	18.48	26.39	36.74	69.15	134.44	224.24	90.98	160.92	255.53	5.90	9.62	14.09	0.03	0.04	0.06	0.11	0.21	0.35	0.14	0.25	0.39	34.80	49.97	65.13	0.05	0.08	0.10	2.25	4.17	6.40	0.00	0.01	0.01
Vancomycin resistant Enterococci (*E. faecalis* and *E. faecium*)	223.00	272.88	327.55	2,181.32	2,613.75	3,103.97	2,393.74	2,888.29	3,431.78	5.02	5.60	6.29	0.34	0.42	0.50	3.36	4.03	4.78	3.69	4.45	5.29	1,541.91	1,969.67	2,408.11	2.38	3.04	3.71	107.38	134.13	164.46	0.17	0.21	0.25
Colistin resistant *Escherichia coli*	278.44	321.33	366.84	4,576.77	5,508.83	6,525.32	4,874.78	5,835.05	6,892.37	13.60	16.07	18.80	0.43	0.50	0.57	7.05	8.49	10.06	7.51	8.99	10.62	2,468.48	2,803.92	3,157.72	3.80	4.32	4.87	207.45	245.43	291.66	0.32	0.38	0.45
Carbapenem resistant *Escherichia coli* (excluding those resistant to colistin)	77.07	92.63	112.20	768.49	977.48	1,226.39	857.85	1,069.66	1,326.78	7.46	9.24	11.34	0.12	0.14	0.17	1.18	1.51	1.89	1.32	1.65	2.05	761.92	917.10	1,060.09	1.17	1.41	1.63	39.92	50.21	61.69	0.06	0.08	0.10
Third-generation cephalosporin resistant *Escherichia coli* (excluding those resistant to colistin and/or carbapenem)	3,107.73	3,438.00	3,786.16	18,279.14	20,233.00	22,173.81	21,495.01	23,673.98	25,797.07	4.98	5.38	5.84	4.79	5.30	5.84	28.18	31.19	34.18	33.13	36.49	39.76	31,137.62	35,135.47	39,243.87	48.00	54.16	60.49	936.33	1,075.77	1,222.56	1.44	1.66	1.88
Colistin resistant *Klebsiella pneumoniae*	20.84	27.32	34.60	335.29	472.32	628.25	361.66	500.37	661.36	9.31	12.19	15.50	0.03	0.04	0.05	0.52	0.73	0.97	0.56	0.77	1.02	91.41	123.03	154.98	0.14	0.19	0.24	20.26	27.31	35.65	0.03	0.04	0.05
Carbapenem resistant *Klebsiella pneumoniae* (excluding those resistant to colistin)	24.88	33.79	44.16	247.11	363.23	493.63	275.44	397.15	533.81	9.37	12.40	15.93	0.04	0.05	0.07	0.38	0.56	0.76	0.42	0.61	0.82	71.32	97.70	125.44	0.11	0.15	0.19	9.57	13.32	17.61	0.01	0.02	0.03
Third-generation cephalosporin resistant *Klebsiella pneumoniae* (excluding those resistant to colistin and/or carbapenem)	705.48	787.24	880.97	3,493.17	3,782.09	4,106.88	4,227.18	4,572.67	4,952.15	5.98	6.37	6.80	1.09	1.21	1.36	5.38	5.83	6.33	6.52	7.05	7.63	2,681.67	2,958.82	3,242.30	4.13	4.56	5.00	148.66	160.85	173.49	0.23	0.25	0.27
Colistin resistant *Pseudomonas aeruginosa*	29.89	47.53	68.68	107.22	237.26	419.45	145.83	284.56	476.33	5.48	9.97	14.99	0.05	0.07	0.11	0.17	0.37	0.65	0.22	0.44	0.73	80.29	128.49	176.56	0.12	0.20	0.27	3.90	8.63	14.33	0.01	0.01	0.02
Carbapenem resistant *Pseudomonas aeruginosa* (excluding those resistant to colistin)	217.33	262.00	309.32	939.47	1,312.61	1,729.84	1,181.32	1,575.70	2,025.68	6.90	9.02	11.25	0.33	0.40	0.48	1.45	2.02	2.67	1.82	2.43	3.12	645.00	772.36	899.85	0.99	1.19	1.39	39.19	52.49	66.53	0.06	0.08	0.10
Pseudomonas aeruginosa resistant to three or more antimicrobial groups (excluding those resistant to colistin and/or carbapenem)	288.93	351.96	418.08	1,227.69	1,681.62	2,202.87	1,550.46	2,038.81	2,601.35	7.27	9.33	11.65	0.45	0.54	0.64	1.89	2.59	3.40	2.39	3.14	4.01	801.65	960.29	1,133.33	1.24	1.48	1.75	46.65	61.75	79.42	0.07	0.10	0.12
Methicillin resistant *Staphylococcus aureus*	1.323.95	1.434.76	1.557.09	6.437.59	6.890.04	7.312.38	7.801.14	8.328.94	8.806.84	5.92	6.21	6.50	2.04	2.21	2.40	9.92	10.62	11.27	12.02	12.84	13.58	6.314.63	6.906.89	7.490.37	9.73	10.65	11.55	301.84	326.63	352.69	0.47	0.50	0.54
Penicillin and macrolides resistant *Streptococcus pneumoniae* (excluding those only resistant to penicillin)	33.26	45.39	68.10	126.75	152.83	197.79	164.84	198.28	260.92	11.74	13.33	15.05	0.05	0.07	0.10	0.20	0.24	0.30	0.25	0.31	0.40	40.03	48.45	62.85	0.06	0.07	0.10	2.51	3.00	3.66	0.00	0.00	0.01
Penicillin tesistant *Streptococcus pneumoniae* (excluding those resistant to macrolides)	4.03	5.82	9.40	15.35	18.98	25.22	19.95	24.80	34.15	2.96	3.36	3.78	0.01	0.01	0.01	0.02	0.03	0.04	0.03	0.04	0.05	19.30	23.81	35.36	0.03	0.04	0.05	1.20	1.48	1.97	0.00	0.00	0.00
Total	6,378.72	7,186.04	8,076.84	38,896.57	44,589.55	50,724.29	45,562.81	51,800.54	58,459.30				9.83	11.08	12.45	59.96	68.73	78.19	70.23	79.85	90.11	46,740.01	52,970.70	59,357.18	72.05	81.65	91.49	1,870.04	2,171.64	2,502.47	2.88	3.35	3.86

Model	YLD			YLL			DALY			DALY per Case			YLD per 100,000			YLL per 100,000			DALY per 100,000			Cases per year			Incidence per 100,000			Deaths per year			Mortality per 100,000		
United Kingdom - Acinetobacter spp. CRACI BSI - model 36	3.89	5.79	8.40	16.23	44.90	81.75	21.41	50.88	87.78	2.68	5.95	9.18	5.99E-03	8.92E-03	0.01	0.03	0.07	0.13	0.03	0.08	0.14	6.76	8.62	10.94	0.01	0.01	0.02	0.64	1.76	3.20	9.85E-04	2.71E-03	4.94E-03
United Kingdom - Acinetobacter spp. CRACI OTH - model 40	1.53E-04	1.02E-03	3.02E-03	0.00	0.00	0.00	1.53E-04	1.02E-03	3.02E-03	1.56E-03	3.71E-03	6.56E-03	2.36E-07	1.57E-06	4.65E-06	0.00	0.00	0.00	2.36E-07	1.57E-06	4.65E-06	0.06	0.31	0.66	8.67E-05	4.77E-04	1.01E-03	0.00	0.00	0.00	0.00	0.00	0.00
United Kingdom - Acinetobacter spp. CRACI RESP - model 37	2.46	4.30	6.70	5.01	8.57	12.58	7.55	12.87	18.61	1.19	1.39	1.58	3.79E-03	6.63E-03	0.01	7.71E-03	0.01	0.02	0.01	0.02	0.03	5.61	9.30	13.15	8.65E-03	0.01	0.02	0.20	0.34	0.49	3.02E-04	5.18E-04	7.61E-04
United Kingdom - Acinetobacter spp. CRACI SSI - model 39	1.15E-03	5.86E-03	0.02	0.29	0.72	1.14	0.29	0.73	1.15	0.23	0.23	0.23	1.77E-06	9.04E-06	2.63E-05	4.40E-04	1.12E-03	1.76E-03	4.40E-04	1.13E-03	1.77E-03	1.24	3.16	4.98	1.91E-03	4.88E-03	7.67E-03	0.01	0.03	0.04	1.72E-05	4.39E-05	6.91E-05
United Kingdom - Acinetobacter spp. CRACI UTI - model 38	1.87E-03	6.70E-03	0.02	0.00	0.00	0.00	1.87E-03	6.70E-03	0.02	7.92E-04	2.00E-03	3.59E-03	2.88E-06	1.03E-05	2.44E-05	0.00	0.00	0.00	2.88E-06	1.03E-05	2.44E-05	1.33	3.58	5.80	2.05E-03	5.51E-03	8.94E-03	0.00	0.00	0.00	0.00	0.00	0.00
United Kingdom - Acinetobacter spp. ColRACI BSI - model 41	11.54	16.37	23.22	53.64	129.43	218.96	68.72	146.69	238.23	4.10	8.44	13.25	0.02	0.03	0.04	0.08	0.20	0.34	0.11	0.23	0.37	14.33	17.14	20.59	0.02	0.03	0.03	1.58	3.54	5.46	2.44E-03	5.45E-03	8.42E-03
United Kingdom - Acinetobacter spp. ColRACI OTH - model 45	7.97E-04	2.12E-03	4.67E-03	0.00	0.00	0.00	7.97E-04	2.12E-03	4.67E-03	1.89E-03	3.62E-03	5.87E-03	1.23E-06	3.27E-06	7.20E-06	0.00	0.00	0.00	1.23E-06	3.27E-06	7.20E-06	0.24	0.61	1.08	3.64E-04	9.38E-04	1.66E-03	0.00	0.00	0.00	0.00	0.00	0.00
United Kingdom - Acinetobacter spp. ColRACI RESP - model 42	7.49	12.50	18.54	15.32	24.55	35.65	23.07	37.25	53.15	1.47	1.98	2.54	0.01	0.02	0.03	0.02	0.04	0.05	0.04	0.06	0.08	13.06	18.65	24.84	0.02	0.03	0.04	0.46	0.67	0.92	7.07E-04	1.03E-03	1.42E-03
United Kingdom - Acinetobacter spp. ColRACI SSI - model 44	4.55E-03	0.01	0.03	1.57	2.90	4.16	1.59	2.91	4.19	0.40	0.46	0.52	7.02E-06	1.95E-05	4.11E-05	2.43E-03	4.47E-03	6.42E-03	2.44E-03	4.49E-03	6.46E-03	3.50	6.34	9.09	5.39E-03	9.77E-03	0.01	0.07	0.14	0.21	1.12E-04	2.18E-04	3.27E-04
United Kingdom - Acinetobacter spp. ColRACI UTI - model 43	6.21E-03	0.01	0.03	0.00	0.00	0.00	6.21E-03	0.01	0.03	1.08E-03	2.02E-03	3.22E-03	9.57E-06	2.15E-05	3.90E-05	0.00	0.00	0.00	9.57E-06	2.15E-05	3.90E-05	3.83	7.03	10.10	5.91E-03	0.01	0.02	0.00	0.00	0.00	0.00	0.00	0.00
United Kingdom - Acinetobacter spp. MDRACI BSI - model 31	11.21	15.04	20.56	53.53	109.11	188.65	67.76	124.15	204.94	4.00	7.33	11.39	0.02	0.02	0.03	0.08	0.17	0.29	0.10	0.19	0.32	14.37	17.23	20.44	0.02	0.03	0.03	1.72	3.35	5.28	2.66E-03	5.16E-03	8.14E-03
United Kingdom - Acinetobacter spp. MDRACI OTH - model 35	6.28E-04	2.13E-03	4.87E-03	0.00	0.00	0.00	6.28E-04	2.13E-03	4.87E-03	1.91E-03	3.68E-03	5.81E-03	9.68E-07	3.28E-06	7.51E-06	0.00	0.00	0.00	9.68E-07	3.28E-06	7.51E-06	0.19	0.60	1.11	3.00E-04	9.23E-04	1.70E-03	0.00	0.00	0.00	0.00	0.00	0.00
United Kingdom - Acinetobacter spp. MDRACI RESP - model 32	7.26	11.32	16.13	13.95	22.35	31.31	21.55	33.76	46.27	1.43	1.81	2.21	0.01	0.02	0.02	0.02	0.03	0.05	0.03	0.05	0.07	12.91	18.80	24.60	0.02	0.03	0.04	0.45	0.68	0.91	6.96E-04	1.05E-03	1.41E-03
United Kingdom - Acinetobacter spp. MDRACI SSI - model 34	4.22E-03	0.01	0.03	1.67	2.98	4.28	1.67	2.99	4.29	0.47	0.48	0.48	6.51E-06	1.92E-05	4.22E-05	2.58E-03	4.60E-03	6.59E-03	2.58E-03	4.61E-03	6.61E-03	3.58	6.29	9.03	5.52E-03	9.70E-03	0.01	0.07	0.14	0.21	1.12E-04	2.18E-04	3.23E-04
United Kingdom - Acinetobacter spp. MDRACI UTI - model 33	5.87E-03	0.01	0.03	0.00	0.00	0.00	5.87E-03	0.01	0.03	9.72E-04	2.02E-03	3.31E-03	9.05E-06	2.10E-05	4.14E-05	0.00	0.00	0.00	9.05E-06	2.10E-05	4.14E-05	3.74	7.04	9.96	5.76E-03	0.01	0.02	0.00	0.00	0.00	0.00	0.00	0.00
United Kingdom - Enterococcus faecalis and E. faecium VRE BSI - model 66	221.07	270.10	323.59	1,947.73	2,307.00	2,720.95	2,158.72	2,578.78	3,045.32	4.61	5.14	5.79	0.34	0.42	0.50	3.00	3.56	4.19	3.33	3.97	4.69	406.32	499.11	607.60	0.63	0.77	0.94	92.52	114.08	138.97	0.14	0.18	0.21
United Kingdom - Enterococcus faecalis and E. faecium VRE OTH - model 70	0.61	0.87	1.19	0.00	0.00	0.00	0.61	0.87	1.19	1.23E-03	1.66E-03	2.09E-03	9.40E-04	1.35E-03	1.84E-03	0.00	0.00	0.00	9.40E-04	1.35E-03	1.84E-03	415.17	535.17	648.50	0.64	0.82	1.00	0.00	0.00	0.00	0.00	0.00	0.00
United Kingdom - Enterococcus faecalis and E. faecium VRE RESP - model 67	0.00	0.00	0.00	0.00	0.00	0.00	0.00	0.00	0.00	NaN	NaN	NaN	0.00	0.00	0.00	0.00	0.00	0.00	0.00	0.00	0.00	0.00	0.00	0.00	0.00	0.00	0.00	0.00	0.00	0.00	0.00	0.00	0.00
United Kingdom - Enterococcus faecalis and E. faecium VRE SSI - model 69	1.00	1.44	2.11	233.60	306.75	383.02	234.08	308.17	384.61	0.41	0.45	0.49	1.54E-03	2.22E-03	3.26E-03	0.36	0.47	0.59	0.36	0.48	0.59	527.26	680.78	835.27	0.81	1.05	1.29	14.86	20.05	25.49	0.02	0.03	0.04
United Kingdom - Enterococcus faecalis and E. faecium VRE UTI - model 68	0.32	0.47	0.66	0.00	0.00	0.00	0.32	0.47	0.66	1.43E-03	1.87E-03	2.37E-03	5.00E-04	7.25E-04	1.02E-03	0.00	0.00	0.00	5.00E-04	7.25E-04	1.02E-03	193.16	254.61	316.74	0.30	0.39	0.49	0.00	0.00	0.00	0.00	0.00	0.00
United Kingdom - Escherichia coli 3GCREC BSI - model 16	2,381.04	2,593.66	2,813.95	15,223.00	16,679.91	18,165.74	17,703.96	19,273.28	20,830.59	3.65	3.91	4.22	3.67	4.00	4.34	23.47	25.71	28.00	27.29	29.71	32.11	4,611.23	4,915.53	5,291.89	7.11	7.58	8.16	751.04	850.67	959.88	1.16	1.31	1.48
United Kingdom - Escherichia coli 3GCREC OTH - model 20	4.14	5.43	7.21	0.00	0.00	0.00	4.14	5.43	7.21	2.72E-03	3.35E-03	4.12E-03	6.39E-03	8.37E-03	0.01	0.00	0.00	0.00	6.39E-03	8.37E-03	0.01	1,334.81	1,626.73	1,926.19	2.06	2.51	2.97	0.00	0.00	0.00	0.00	0.00	0.00
United Kingdom - Escherichia coli 3GCREC RESP - model 17	681.75	786.46	898.04	1,304.85	1,527.52	1,744.29	1,995.63	2,316.94	2,632.72	0.96	1.07	1.20	1.05	1.21	1.38	2.01	2.35	2.69	3.08	3.57	4.06	1,797.87	2,167.99	2,557.98	2.77	3.34	3.94	63.64	77.70	92.51	0.10	0.12	0.14
United Kingdom - Escherichia coli 3GCREC SSI - model 19	8.14	11.07	14.97	1,751.29	2,025.56	2,263.78	1,758.63	2,036.95	2,274.56	0.37	0.39	0.42	0.01	0.02	0.02	2.70	3.12	3.49	2.71	3.14	3.51	4,491.76	5,213.80	5,915.71	6.92	8.04	9.12	121.65	147.40	170.17	0.19	0.23	0.26
United Kingdom - Escherichia coli 3GCREC UTI - model 18	32.65	41.38	51.99	0.00	0.00	0.00	32.65	41.38	51.99	1.59E-03	1.96E-03	2.37E-03	0.05	0.06	0.08	0.00	0.00	0.00	0.05	0.06	0.08	18,901.95	21,211.41	23,552.11	29.14	32.70	36.30	0.00	0.00	0.00	0.00	0.00	0.00
United Kingdom - Escherichia coli CREC BSI - model 21	58.47	67.77	80.07	686.38	875.15	1,103.36	756.19	942.48	1,173.48	6.21	7.73	9.52	0.09	0.10	0.12	1.06	1.35	1.70	1.17	1.45	1.81	109.10	122.58	135.89	0.17	0.19	0.21	34.81	43.58	53.69	0.05	0.07	0.08
United Kingdom - Escherichia coli CREC OTH - model 25	0.16	0.24	0.36	0.00	0.00	0.00	0.16	0.24	0.36	2.47E-03	3.46E-03	4.86E-03	2.48E-04	3.74E-04	5.56E-04	0.00	0.00	0.00	2.48E-04	3.74E-04	5.56E-04	54.57	70.86	85.28	0.08	0.11	0.13	0.00	0.00	0.00	0.00	0.00	0.00
United Kingdom - Escherichia coli CREC RESP - model 22	17.51	23.25	29.81	33.86	44.86	56.98	52.04	68.13	84.94	0.88	1.11	1.37	0.03	0.04	0.05	0.05	0.07	0.09	0.08	0.11	0.13	46.93	62.15	76.05	0.07	0.10	0.12	1.67	2.24	2.78	2.57E-03	3.45E-03	4.29E-03
United Kingdom - Escherichia coli CREC SSI - model 24	0.19	0.31	0.45	48.25	57.46	66.05	48.71	57.75	66.50	0.37	0.40	0.44	2.95E-04	4.75E-04	6.93E-04	0.07	0.09	0.10	0.08	0.09	0.10	116.93	145.27	168.54	0.18	0.22	0.26	3.44	4.39	5.22	5.30E-03	6.77E-03	8.05E-03
United Kingdom - Escherichia coli CREC UTI - model 23	0.74	1.06	1.50	0.00	0.00	0.00	0.74	1.06	1.50	1.50E-03	2.06E-03	2.80E-03	1.15E-03	1.64E-03	2.31E-03	0.00	0.00	0.00	1.15E-03	1.64E-03	2.31E-03	434.40	516.25	594.34	0.67	0.80	0.92	0.00	0.00	0.00	0.00	0.00	0.00
United Kingdom - Escherichia coli ColREC BSI - model 26	209.65	235.20	263.21	4,287.44	5,165.38	6,128.03	4,516.46	5,404.93	6,393.85	12.13	14.39	16.87	0.32	0.36	0.41	6.61	7.96	9.45	6.96	8.33	9.86	349.09	375.46	407.69	0.54	0.58	0.63	191.02	225.74	268.68	0.29	0.35	0.41
United Kingdom - Escherichia coli ColREC OTH - model 30	0.55	0.75	1.03	0.00	0.00	0.00	0.55	0.75	1.03	2.69E-03	3.50E-03	4.55E-03	8.45E-04	1.16E-03	1.59E-03	0.00	0.00	0.00	8.45E-04	1.16E-03	1.59E-03	181.37	216.19	249.69	0.28	0.33	0.38	0.00	0.00	0.00	0.00	0.00	0.00
United Kingdom - Escherichia coli ColREC RESP - model 27	65.09	81.14	97.10	126.09	157.18	187.97	191.33	238.79	282.86	1.06	1.25	1.47	0.10	0.13	0.15	0.19	0.24	0.29	0.29	0.37	0.44	154.49	190.07	221.55	0.24	0.29	0.34	5.52	6.81	8.05	8.51E-03	0.01	0.01
United Kingdom - Escherichia coli ColREC SSI - model 29	0.68	0.94	1.27	163.24	186.27	209.32	163.98	187.29	210.39	0.40	0.42	0.45	1.04E-03	1.44E-03	1.95E-03	0.25	0.29	0.32	0.25	0.29	0.32	382.24	442.82	504.72	0.59	0.68	0.78	10.91	12.89	14.93	0.02	0.02	0.02
United Kingdom - Escherichia coli ColREC UTI - model 28	2.47	3.30	4.24	0.00	0.00	0.00	2.47	3.30	4.24	1.62E-03	2.09E-03	2.61E-03	3.81E-03	5.08E-03	6.54E-03	0.00	0.00	0.00	3.81E-03	5.08E-03	6.54E-03	1,401.29	1,579.39	1,774.07	2.16	2.43	2.73	0.00	0.00	0.00	0.00	0.00	0.00
United Kingdom - Klebsiella pneumoniae 3GCRKP BSI - model 1	446.07	488.34	542.26	2,875.21	3,076.91	3,302.18	3,343.90	3,568.97	3,817.01	4.37	4.62	4.91	0.69	0.75	0.84	4.43	4.74	5.09	5.15	5.50	5.88	734.24	772.40	809.50	1.13	1.19	1.25	120.65	128.84	137.32	0.19	0.20	0.21
United Kingdom - Klebsiella pneumoniae 3GCRKP OTH - model 5	0.79	1.02	1.29	0.00	0.00	0.00	0.79	1.02	1.29	2.68E-03	3.37E-03	4.05E-03	1.22E-03	1.57E-03	1.98E-03	0.00	0.00	0.00	1.22E-03	1.57E-03	1.98E-03	270.04	306.57	344.77	0.42	0.47	0.53	0.00	0.00	0.00	0.00	0.00	0.00
United Kingdom - Klebsiella pneumoniae 3GCRKP RESP - model 2	256.73	295.46	334.41	505.17	573.97	653.46	767.78	869.05	979.66	1.20	1.30	1.42	0.40	0.46	0.52	0.78	0.88	1.01	1.18	1.34	1.51	596.96	667.01	739.34	0.92	1.03	1.14	21.22	23.96	26.79	0.03	0.04	0.04
United Kingdom - Klebsiella pneumoniae 3GCRKP SSI - model 4	0.48	0.64	0.85	112.80	131.21	151.25	113.30	131.86	152.02	0.41	0.43	0.46	7.37E-04	9.92E-04	1.32E-03	0.17	0.20	0.23	0.17	0.20	0.23	264.90	302.97	344.10	0.41	0.47	0.53	6.79	8.05	9.38	0.01	0.01	0.01
United Kingdom - Klebsiella pneumoniae 3GCRKP UTI - model 3	1.41	1.77	2.17	0.00	0.00	0.00	1.41	1.77	2.17	1.57E-03	1.96E-03	2.33E-03	2.18E-03	2.73E-03	3.34E-03	0.00	0.00	0.00	2.18E-03	2.73E-03	3.34E-03	815.53	909.88	1,004.59	1.26	1.40	1.55	0.00	0.00	0.00	0.00	0.00	0.00
United Kingdom - Klebsiella pneumoniae CRKP BSI - model 6	20.41	26.07	32.90	235.81	343.78	465.88	259.66	369.89	495.12	7.81	10.45	13.49	0.03	0.04	0.05	0.36	0.53	0.72	0.40	0.57	0.76	30.24	35.18	40.66	0.05	0.05	0.06	9.09	12.55	16.54	0.01	0.02	0.03
United Kingdom - Klebsiella pneumoniae CRKP OTH - model 10	0.03	0.06	0.10	0.00	0.00	0.00	0.03	0.06	0.10	2.00E-03	3.52E-03	5.25E-03	4.46E-05	8.61E-05	1.48E-04	0.00	0.00	0.00	4.46E-05	8.61E-05	1.48E-04	10.34	16.25	22.24	0.02	0.03	0.03	0.00	0.00	0.00	0.00	0.00	0.00
United Kingdom - Klebsiella pneumoniae CRKP RESP - model 7	4.41	7.60	11.06	8.49	14.77	21.09	12.90	22.46	31.84	1.17	1.49	1.90	6.80E-03	0.01	0.02	0.01	0.02	0.03	0.02	0.03	0.05	9.76	14.86	20.03	0.02	0.02	0.03	0.35	0.54	0.74	5.37E-04	8.28E-04	1.13E-03
United Kingdom - Klebsiella pneumoniae CRKP SSI - model 9	9.93E-03	0.02	0.04	2.81	4.67	6.66	2.83	4.70	6.69	0.38	0.45	0.54	1.53E-05	3.27E-05	5.96E-05	4.33E-03	7.20E-03	0.01	4.36E-03	7.25E-03	0.01	6.48	10.33	14.18	9.98E-03	0.02	0.02	0.13	0.23	0.34	2.02E-04	3.58E-04	5.19E-04
United Kingdom - Klebsiella pneumoniae CRKP UTI - model 8	0.02	0.04	0.07	0.00	0.00	0.00	0.02	0.04	0.07	1.25E-03	2.06E-03	3.03E-03	3.51E-05	6.57E-05	1.07E-04	0.00	0.00	0.00	3.51E-05	6.57E-05	1.07E-04	14.50	21.09	28.32	0.02	0.03	0.04	0.00	0.00	0.00	0.00	0.00	0.00
United Kingdom - Klebsiella pneumoniae ColRKP BSI - model 11	16.87	21.06	25.95	325.07	456.45	606.44	347.46	478.24	631.07	8.22	10.90	13.98	0.03	0.03	0.04	0.50	0.70	0.93	0.54	0.74	0.97	38.09	43.99	50.32	0.06	0.07	0.08	19.63	26.32	34.29	0.03	0.04	0.05
United Kingdom - Klebsiella pneumoniae ColRKP OTH - model 15	0.04	0.07	0.11	0.00	0.00	0.00	0.04	0.07	0.11	2.20E-03	3.53E-03	5.10E-03	6.02E-05	1.12E-04	1.77E-04	0.00	0.00	0.00	6.02E-05	1.12E-04	1.77E-04	14.06	20.70	27.22	0.02	0.03	0.04	0.00	0.00	0.00	0.00	0.00	0.00
United Kingdom - Klebsiella pneumoniae ColRKP RESP - model 12	3.88	6.10	8.40	7.46	11.64	16.06	11.36	17.75	24.27	0.78	0.96	1.15	5.98E-03	9.40E-03	0.01	0.01	0.02	0.02	0.02	0.03	0.04	12.01	18.46	25.10	0.02	0.03	0.04	0.43	0.67	0.91	6.68E-04	1.03E-03	1.40E-03
United Kingdom - Klebsiella pneumoniae ColRKP SSI - model 14	0.01	0.03	0.04	2.75	4.23	5.76	2.77	4.26	5.82	0.30	0.33	0.36	2.04E-05	4.13E-05	6.78E-05	4.24E-03	6.53E-03	8.88E-03	4.27E-03	6.56E-03	8.97E-03	8.39	12.95	17.41	0.01	0.02	0.03	0.20	0.32	0.46	3.12E-04	5.00E-04	7.03E-04
United Kingdom - Klebsiella pneumoniae ColRKP UTI - model 13	0.03	0.06	0.09	0.00	0.00	0.00	0.03	0.06	0.09	1.34E-03	2.08E-03	2.95E-03	5.00E-05	8.59E-05	1.33E-04	0.00	0.00	0.00	5.00E-05	8.59E-05	1.33E-04	18.86	26.92	34.93	0.03	0.04	0.05	0.00	0.00	0.00	0.00	0.00	0.00
United Kingdom - Pseudomonas aeruginosa CRPA BSI - model 51	62.77	73.23	84.56	436.44	618.73	808.47	506.06	690.95	887.10	4.82	6.44	8.13	0.10	0.11	0.13	0.67	0.95	1.25	0.78	1.07	1.37	96.45	107.50	118.52	0.15	0.17	0.18	17.89	24.65	31.38	0.03	0.04	0.05
United Kingdom - Pseudomonas aeruginosa CRPA OTH - model 55	0.14	0.20	0.27	0.00	0.00	0.00	0.14	0.20	0.27	2.78E-03	3.66E-03	4.57E-03	2.16E-04	3.10E-04	4.17E-04	0.00	0.00	0.00	2.16E-04	3.10E-04	4.17E-04	42.19	54.63	67.30	0.07	0.08	0.10	0.00	0.00	0.00	0.00	0.00	0.00
United Kingdom - Pseudomonas aeruginosa CRPA RESP - model 52	154.11	188.13	223.90	478.43	662.06	882.33	650.13	852.28	1,098.77	1.69	2.14	2.63	0.24	0.29	0.35	0.74	1.02	1.36	1.00	1.31	1.69	338.59	399.06	458.23	0.52	0.62	0.71	20.03	26.18	33.10	0.03	0.04	0.05
United Kingdom - Pseudomonas aeruginosa CRPA SSI - model 54	0.10	0.16	0.22	24.60	31.82	39.04	24.80	31.99	39.15	0.39	0.43	0.48	1.61E-04	2.43E-04	3.36E-04	0.04	0.05	0.06	0.04	0.05	0.06	57.52	74.17	89.88	0.09	0.11	0.14	1.27	1.66	2.04	1.96E-03	2.56E-03	3.14E-03
United Kingdom - Pseudomonas aeruginosa CRPA UTI - model 53	0.20	0.28	0.38	0.00	0.00	0.00	0.20	0.28	0.38	1.57E-03	2.04E-03	2.61E-03	3.08E-04	4.30E-04	5.88E-04	0.00	0.00	0.00	3.08E-04	4.30E-04	5.88E-04	110.25	136.99	165.92	0.17	0.21	0.26	0.00	0.00	0.00	0.00	0.00	0.00
United Kingdom - Pseudomonas aeruginosa ColRPA BSI - model 56	9.42	13.32	18.18	47.90	112.09	198.61	61.41	125.10	214.39	3.60	7.14	11.04	0.01	0.02	0.03	0.07	0.17	0.31	0.09	0.19	0.33	14.01	17.85	22.38	0.02	0.03	0.03	1.75	4.07	6.75	2.70E-03	6.27E-03	0.01
United Kingdom - Pseudomonas aeruginosa ColRPA OTH - model 60	0.01	0.03	0.06	0.00	0.00	0.00	0.01	0.03	0.06	1.92E-03	3.67E-03	5.63E-03	2.01E-05	4.95E-05	9.85E-05	0.00	0.00	0.00	2.01E-05	4.95E-05	9.85E-05	4.62	9.01	13.41	7.12E-03	0.01	0.02	0.00	0.00	0.00	0.00	0.00	0.00
United Kingdom - Pseudomonas aeruginosa ColRPA RESP - model 57	20.43	34.11	50.29	56.33	119.29	212.09	81.39	153.48	253.00	1.45	2.34	3.40	0.03	0.05	0.08	0.09	0.18	0.33	0.13	0.24	0.39	42.10	66.68	89.73	0.06	0.10	0.14	2.02	4.28	7.16	3.11E-03	6.60E-03	0.01
United Kingdom - Pseudomonas aeruginosa ColRPA SSI - model 59	8.61E-03	0.02	0.06	2.99	5.88	8.75	3.00	5.91	8.79	0.42	0.48	0.55	1.33E-05	3.71E-05	8.48E-05	4.61E-03	9.06E-03	0.01	4.62E-03	9.11E-03	0.01	6.19	12.33	18.19	9.54E-03	0.02	0.03	0.12	0.27	0.42	1.90E-04	4.23E-04	6.52E-04
United Kingdom - Pseudomonas aeruginosa ColRPA UTI - model 58	0.02	0.05	0.09	0.00	0.00	0.00	0.02	0.05	0.09	1.03E-03	2.05E-03	3.33E-03	3.08E-05	7.10E-05	1.35E-04	0.00	0.00	0.00	3.08E-05	7.10E-05	1.35E-04	13.37	22.62	32.84	0.02	0.03	0.05	0.00	0.00	0.00	0.00	0.00	0.00
United Kingdom - Pseudomonas aeruginosa MDRPA BSI - model 46	85.11	99.75	116.93	567.61	785.59	1,040.09	656.84	885.71	1,150.85	4.98	6.58	8.37	0.13	0.15	0.18	0.87	1.21	1.60	1.01	1.37	1.77	120.70	134.27	151.16	0.19	0.21	0.23	21.29	28.42	36.36	0.03	0.04	0.06
United Kingdom - Pseudomonas aeruginosa MDRPA OTH - model 50	0.18	0.25	0.35	0.00	0.00	0.00	0.18	0.25	0.35	2.86E-03	3.71E-03	4.67E-03	2.70E-04	3.86E-04	5.40E-04	0.00	0.00	0.00	2.70E-04	3.86E-04	5.40E-04	52.21	67.61	84.62	0.08	0.10	0.13	0.00	0.00	0.00	0.00	0.00	0.00
United Kingdom - Pseudomonas aeruginosa MDRPA RESP - model 47	203.26	251.40	300.02	623.48	848.09	1,103.05	856.45	1,104.36	1,389.69	1.81	2.23	2.72	0.31	0.39	0.46	0.96	1.31	1.70	1.32	1.70	2.14	415.39	492.47	571.94	0.64	0.76	0.88	23.50	30.84	39.92	0.04	0.05	0.06
United Kingdom - Pseudomonas aeruginosa MDRPA SSI - model 49	0.13	0.20	0.29	36.59	47.94	59.73	36.75	48.14	59.97	0.48	0.52	0.56	2.02E-04	3.02E-04	4.48E-04	0.06	0.07	0.09	0.06	0.07	0.09	72.15	92.42	113.32	0.11	0.14	0.17	1.85	2.49	3.14	2.86E-03	3.85E-03	4.84E-03
United Kingdom - Pseudomonas aeruginosa MDRPA UTI - model 48	0.25	0.36	0.49	0.00	0.00	0.00	0.25	0.36	0.49	1.56E-03	2.05E-03	2.55E-03	3.86E-04	5.48E-04	7.50E-04	0.00	0.00	0.00	3.86E-04	5.48E-04	7.50E-04	141.19	173.53	212.29	0.22	0.27	0.33	0.00	0.00	0.00	0.00	0.00	0.00
United Kingdom - Staphylococcus aureus MRSA BSI - model 61	653.93	696.60	750.64	4,432.30	4,693.43	4,922.23	5,118.27	5,391.77	5,631.70	4.39	4.59	4.78	1.01	1.07	1.16	6.83	7.23	7.59	7.89	8.31	8.68	1,124.60	1,175.46	1,233.31	1.73	1.81	1.90	198.69	211.27	225.04	0.31	0.33	0.35
United Kingdom - Staphylococcus aureus MRSA OTH - model 65	4.56	5.61	6.97	0.00	0.00	0.00	4.56	5.61	6.97	2.69E-03	3.26E-03	3.92E-03	7.03E-03	8.65E-03	0.01	0.00	0.00	0.00	7.03E-03	8.65E-03	0.01	1,587.30	1,741.44	1,886.68	2.45	2.68	2.91	0.00	0.00	0.00	0.00	0.00	0.00
United Kingdom - Staphylococcus aureus MRSA RESP - model 62	661.93	727.88	793.45	1,291.49	1,414.97	1,542.79	1,960.37	2,145.45	2,316.33	1.13	1.21	1.29	1.02	1.12	1.22	1.99	2.18	2.38	3.02	3.31	3.57	1,597.83	1,771.48	1,933.18	2.46	2.73	2.98	56.68	63.73	70.59	0.09	0.10	0.11
United Kingdom - Staphylococcus aureus MRSA SSI - model 64	3.10	4.13	5.35	713.80	781.63	847.36	717.51	785.57	851.16	0.39	0.41	0.42	4.78E-03	6.37E-03	8.24E-03	1.10	1.20	1.31	1.11	1.21	1.31	1,766.84	1,930.02	2,101.45	2.72	2.97	3.24	46.47	51.62	57.06	0.07	0.08	0.09
United Kingdom - Staphylococcus aureus MRSA UTI - model 63	0.42	0.53	0.68	0.00	0.00	0.00	0.42	0.53	0.68	1.52E-03	1.86E-03	2.27E-03	6.50E-04	8.21E-04	1.05E-03	0.00	0.00	0.00	6.50E-04	8.21E-04	1.05E-03	238.05	288.50	335.75	0.37	0.44	0.52	0.00	0.00	0.00	0.00	0.00	0.00
United Kingdom - Streptococcus pneumoniae PMRSP BSI - model 76	9.82	12.79	16.31	78.91	87.84	97.10	90.96	100.47	110.73	9.55	10.55	11.63	0.02	0.02	0.03	0.12	0.14	0.15	0.14	0.15	0.17	9.52	9.52	9.52	0.01	0.01	0.01	1.55	1.71	1.88	2.39E-03	2.64E-03	2.90E-03
United Kingdom - Streptococcus pneumoniae PMRSP OTH - model 80	3.35E-03	6.61E-03	0.01	0.00	0.00	0.00	3.35E-03	6.61E-03	0.01	1.00E-03	1.95E-03	3.16E-03	5.16E-06	1.02E-05	1.68E-05	0.00	0.00	0.00	5.16E-06	1.02E-05	1.68E-05	2.88	3.36	4.04	4.44E-03	5.18E-03	6.23E-03	0.00	0.00	0.00	0.00	0.00	0.00
United Kingdom - Streptococcus pneumoniae PMRSP RESP - model 77	23.44	32.60	51.78	47.84	65.00	100.69	73.88	97.81	150.18	2.18	2.78	3.41	0.04	0.05	0.08	0.07	0.10	0.16	0.11	0.15	0.23	27.63	35.58	49.29	0.04	0.05	0.08	0.96	1.28	1.77	1.48E-03	1.98E-03	2.73E-03
United Kingdom - Streptococcus pneumoniae PMRSP SSI - model 79	0.00	0.00	0.00	0.00	0.00	0.00	0.00	0.00	0.00	NaN	NaN	NaN	0.00	0.00	0.00	0.00	0.00	0.00	0.00	0.00	0.00	0.00	0.00	0.00	0.00	0.00	0.00	0.00	0.00	0.00	0.00	0.00	0.00
United Kingdom - Streptococcus pneumoniae PMRSP UTI - model 78	0.00	0.00	0.00	0.00	0.00	0.00	0.00	0.00	0.00	NaN	NaN	NaN	0.00	0.00	0.00	0.00	0.00	0.00	0.00	0.00	0.00	0.00	0.00	0.00	0.00	0.00	0.00	0.00	0.00	0.00	0.00	0.00	0.00
United Kingdom - Streptococcus pneumoniae PRSP BSI - model 71	1.21	1.64	2.17	9.67	10.99	12.33	11.21	12.66	14.17	2.36	2.66	2.98	1.87E-03	2.53E-03	3.35E-03	0.01	0.02	0.02	0.02	0.02	0.02	4.76	4.76	4.76	7.34E-03	7.34E-03	7.34E-03	0.75	0.86	0.96	1.16E-03	1.32E-03	1.48E-03
United Kingdom - Streptococcus pneumoniae PRSP OTH - model 75	1.26E-03	3.10E-03	6.48E-03	0.00	0.00	0.00	1.26E-03	3.10E-03	6.48E-03	7.70E-04	1.87E-03	3.68E-03	1.94E-06	4.78E-06	1.00E-05	0.00	0.00	0.00	1.94E-06	4.78E-06	1.00E-05	1.38	1.66	2.16	2.12E-03	2.56E-03	3.33E-03	0.00	0.00	0.00	0.00	0.00	0.00
United Kingdom - Streptococcus pneumoniae PRSP RESP - model 72	2.81	4.17	7.22	5.68	7.99	12.90	8.74	12.14	19.97	0.60	0.70	0.80	4.34E-03	6.42E-03	0.01	8.76E-03	0.01	0.02	0.01	0.02	0.03	13.16	17.40	28.44	0.02	0.03	0.04	0.44	0.62	1.01	6.83E-04	9.60E-04	1.55E-03
United Kingdom - Streptococcus pneumoniae PRSP SSI - model 74	0.00	0.00	0.00	0.00	0.00	0.00	0.00	0.00	0.00	NaN	NaN	NaN	0.00	0.00	0.00	0.00	0.00	0.00	0.00	0.00	0.00	0.00	0.00	0.00	0.00	0.00	0.00	0.00	0.00	0.00	0.00	0.00	0.00
United Kingdom - Streptococcus pneumoniae PRSP UTI - model 73	0.00	0.00	0.00	0.00	0.00	0.00	0.00	0.00	0.00	NaN	NaN	NaN	0.00	0.00	0.00	0.00	0.00	0.00	0.00	0.00	0.00	0.00	0.00	0.00	0.00	0.00	0.00	0.00	0.00	0.00	0.00	0.00	0.00
**Total (Median & 95% UIs)**	6,378.72	7,186.04	8,076.84	38,896.57	44,589.55	50,724.29	45,562.81	51,800.54	58,459.30		-		9.83	11.08	12.45	59.96	68.73	78.19	70.23	79.85	90.11	46,740.01	52,970.70	59,357.18	72.05	81.65	91.49	1,870.04	2,171.64	2,502.47	2.88	3.35	3.86
**Total (average)**		**7,205.86**			**44,695.95**			**51,901.81**			-			**11.11**			**68.90**			**80.00**			**52,992.69**			**81.68**			**2,176.86**			**3.36**	
